# Programmable remodelling of carbon–nitrogen connectivity in amines

**DOI:** 10.1038/s41586-026-11009-1

**Published:** 2026-08-17

**Authors:** James D. Robinson, François Richard, Kohl A. Ratkovich, Joseph M. Phelps, Matthew J. Gaunt

**Affiliations:** Yusuf Hamied Department of Chemistry, University of Cambridge, Cambridge, UK

**Keywords:** Synthetic chemistry methodology, Synthetic chemistry methodology

## Abstract

Tertiary amines are ubiquitous motifs in biologically active molecules, in which they play central parts in molecular recognition and function^1,2^. Among these, tertiary benzylamines are particularly prominent in discovery chemistry as readily assembled starting points for lead generation from abundant building blocks^3,4^, with favourable physicochemical and binding properties. However, despite their accessibility, the core C–N connectivity of these frameworks remains effectively locked^5,6^, confining diversification to benzylamine derivatives and preventing direct access to structurally distinct aryl–alkylamine architectures. Overcoming this limitation requires direct and selective reconfiguration of C–N connectivity within fully elaborated amine scaffolds. Here we show that tertiary benzylamines can be transformed from static scaffolds into programmable platforms for molecular diversification through catalytic remodelling of their C–N bonds^7–9^. N-alkylation with a bifunctional electrophile generates a quaternary ammonium intermediate that encodes palladium-catalysed disassembly and reconfiguration of C–N connectivity. This concept is first exemplified through one-carbon homologation of tertiary benzylamines and, more significantly, establishes a general strategy for programmable insertion of modular units—ranging from single carbon atoms to complex molecular fragments, including hydrocarbon chains, heterocycles and aryl groups—directly across the C–N bond on fully elaborated amine scaffolds. This ability opens a route to programmable diversification of amine frameworks, enabling systematic exploration of amine connectivity and molecular architecture.

## Abstract (web summary)

Tertiary benzylamines can be transformed from static scaffolds into programmable platforms for molecular diversification through catalytic remodelling of their C–N bonds.

## Main

Tertiary benzylamines represent a central molecular feature in bioactive molecules, linking an aromatic ring to a basic nitrogen centre through a single carbon atom. Their prevalence reflects both synthetic accessibility and their ability to engage in key molecular recognition and electrostatic interactions across diverse biological targets^10,11^. Yet this apparent versatility masks a fundamental limitation. Once formed, the carbon–nitrogen (C–N) bond of tertiary benzylamines remains resistant to reconfiguration: existing transformations may decorate the periphery^12^ or cleave the amine entirely^5,6^, but do not alter the connectivity of the C–N bond itself^13^. As a result, the underlying topology of these scaffolds remains fixed, constraining their structural evolution. Tertiary benzylamines thus represent a paradox in modern synthesis—readily accessible and broadly useful structures that resist modification at the level most relevant to function.

**Fig. 1:**
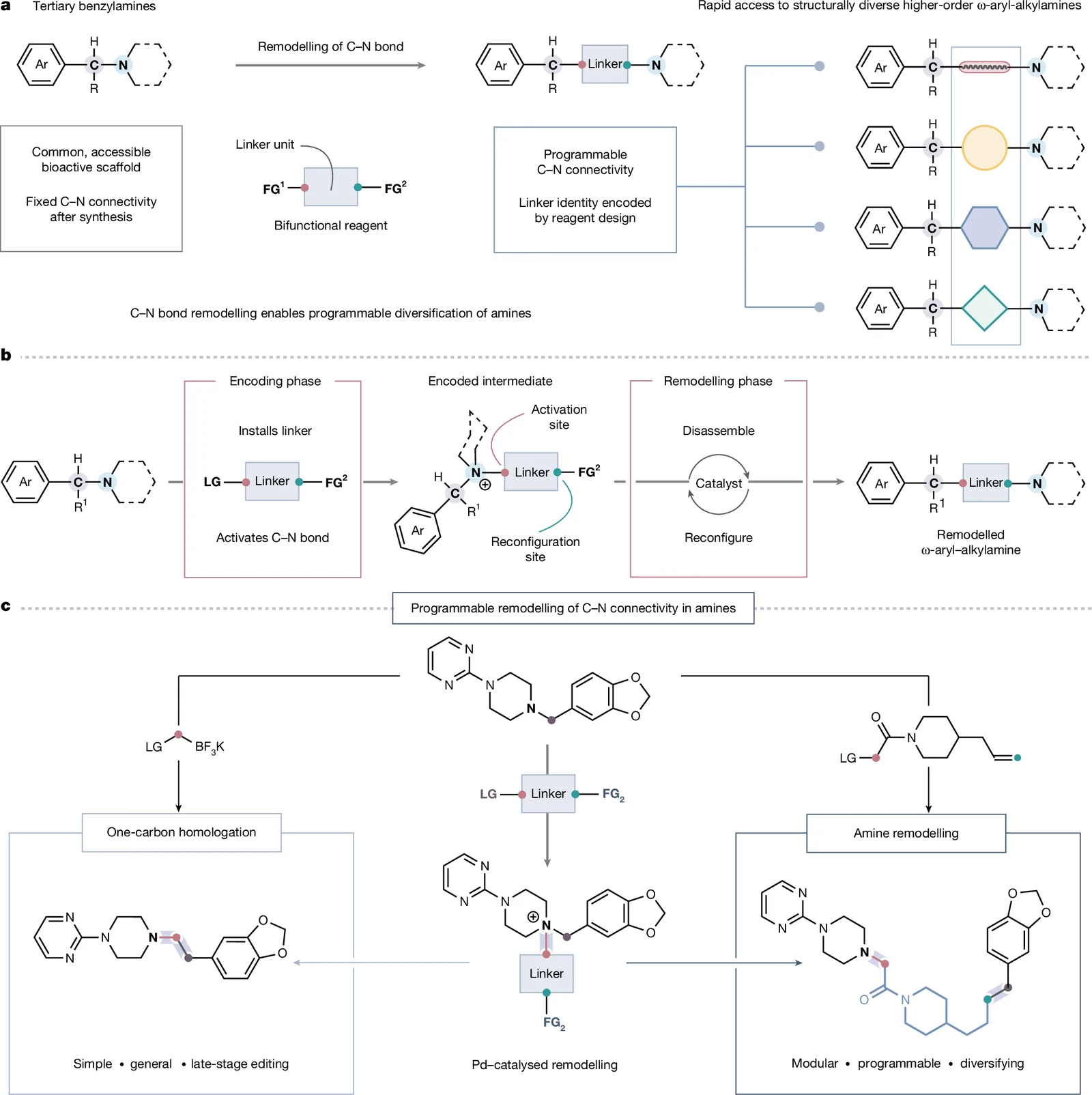
Development of a C–N bond remodelling strategy for tertiary benzylamines. **a**, Concept. Programmable C–N bond remodelling converts tertiary benzylamines into topologically distinct ω-aryl–alkylamines. Bifunctional reagents encode linker identity, enabling catalytic reconfiguration of amine connectivity and access to structurally diverse higher-order amines from a common scaffold. **b**, Design principle. N-alkylation with a bifunctional electrophile generates an encoded ammonium intermediate that activates the C–N bond while installing a linker motif. Under catalytic conditions, controlled disassembly and reconfiguration of this intermediate regenerates an amine framework with modified connectivity. **c**, Realization. The encoded ammonium intermediate defines a platform for a new Pd-catalysed C–N bond remodelling process, delivering two distinct outcomes. One-carbon homologation provides access to β-aryl–ethylamines, whereas programmable remodelling enables insertion of multi-atom linkers to generate structurally diverse aryl–alkylamines from a single tertiary amine precursor. 1C, one carbon; Ar, aryl; FG_1_, functional group that serves as a nucleofuge; FG_2_, a metal-engaging functional group; LG, leaving group.

Beyond tertiary benzylamines, higher-order aryl–alkylamines represent a broader class of architectures in which the identity, length and composition of the linker between the amine and arene again greatly influence molecular behaviour. These structural variations are also central to modulating biological activity, particularly in systems in which subtle changes in spacing and polarity can alter target engagement and pharmacological properties, as exemplified by β-aryl–ethylamine motifs found in numerous bioactive compounds and their extended aryl–alkylamine analogues^14–18^. However, access to such architectures typically relies on homologated aryl–alkyl building blocks, whose availability declines sharply beyond the benzylic position. This disparity reflects a fundamental bias in synthetic methodology. Although direct one-carbon functionalization of arenes provides ready access to a diverse array of benzyl-derived feedstocks, comparatively few general strategies enable analogous access to higher homologues^19–21^. Consequently, homologated ω-aryl–alkyl building blocks typically require multistep synthesis and, in the case of phenethyl derivatives, often exhibit less favourable synthetic properties, limiting the efficiency and modularity of analogue diversification. This disconnect points to a simple but powerful idea. If the core element of the abundant tertiary benzylamine scaffold could be structurally reconfigured—its C–N linkage remodelled to alter the aryl–amine spacing—it could serve as a platform to access a diverse array of these higher-order aryl–alkylamines. In this light, the constraint that limits tertiary benzylamines also defines an opportunity: a chance to transform one of the most accessible amine classes into a platform for molecular innovation (Fig. 1a).

## Reaction design

To unlock the programmable potential in tertiary benzylamines, we predicted a catalytic transformation that remodels their C–N bonds from within by inserting discrete molecular units between the aryl and amine fragments, thereby directly diversifying lead amine scaffolds into structurally and functionally distinct architectures. This strategy redefines the C–N bond not as a static linkage, but as an editable axis of design, enabling controlled variation of amine topology through a programmable transformation. Our design centres on a bifunctional alkylating reagent that encodes the elements required for C–N bond activation, linker installation and amine reconfiguration within a single intermediate (Fig. 1b). N-alkylation of a tertiary benzylamine generates a quaternary ammonium species that renders the C–N bond susceptible to activation^6,13^, enabling disassembly of the amine framework. This intermediate contains both the structural elements of the linker and a metal-engaging group, allowing grafting of the encoded unit into the amine framework under catalytic conditions. The resulting process orchestrates disassembly and reconfiguration, enabling capture of the fragments and installation of the linker to generate a new amine topology.

Conceptually, this design draws inspiration from the classical Stevens rearrangement, a transformation that formally effects carbon migration across a quaternary ammonium centre^22^. Although this reaction demonstrates that carbon–nitrogen connectivity can, in principle, be reorganized through a quaternary ammonium intermediate, its reliance on substrate-controlled reactivity has confined its utility to a narrow and often unpredictable reaction space. Notably, the simple benzylammonium salts most directly relevant to the present study do not undergo productive 1,2-Stevens rearrangement, but instead preferentially react through the competing 2,3-Sommelet–Hauser pathway^23^. Rather than attempting to overcome these intrinsic limitations, we instead abstract its underlying conceptual logic—the reorganization of C–N connectivity—and reimplement it within a catalytic framework based on programmable bond activation and fragment recombination. Accordingly, we implemented this strategy through a Pd-catalysed process for the controlled reconfiguration of tertiary benzylamines. This catalytic manifold is first realized through one-carbon homologation^24–29^, providing the simplest demonstration of the underlying design principle (Fig. 1c). Building on this foundation, extension of the activation mode enables progressively more complex molecular units to be encoded within the bifunctional reagent and inserted across the C–N bond, establishing a programmable platform for the direct diversification of tertiary benzylamines into structurally distinct aryl–alkylamine architectures.

## Development of the one-carbon homologation platform

**Fig. 2:**
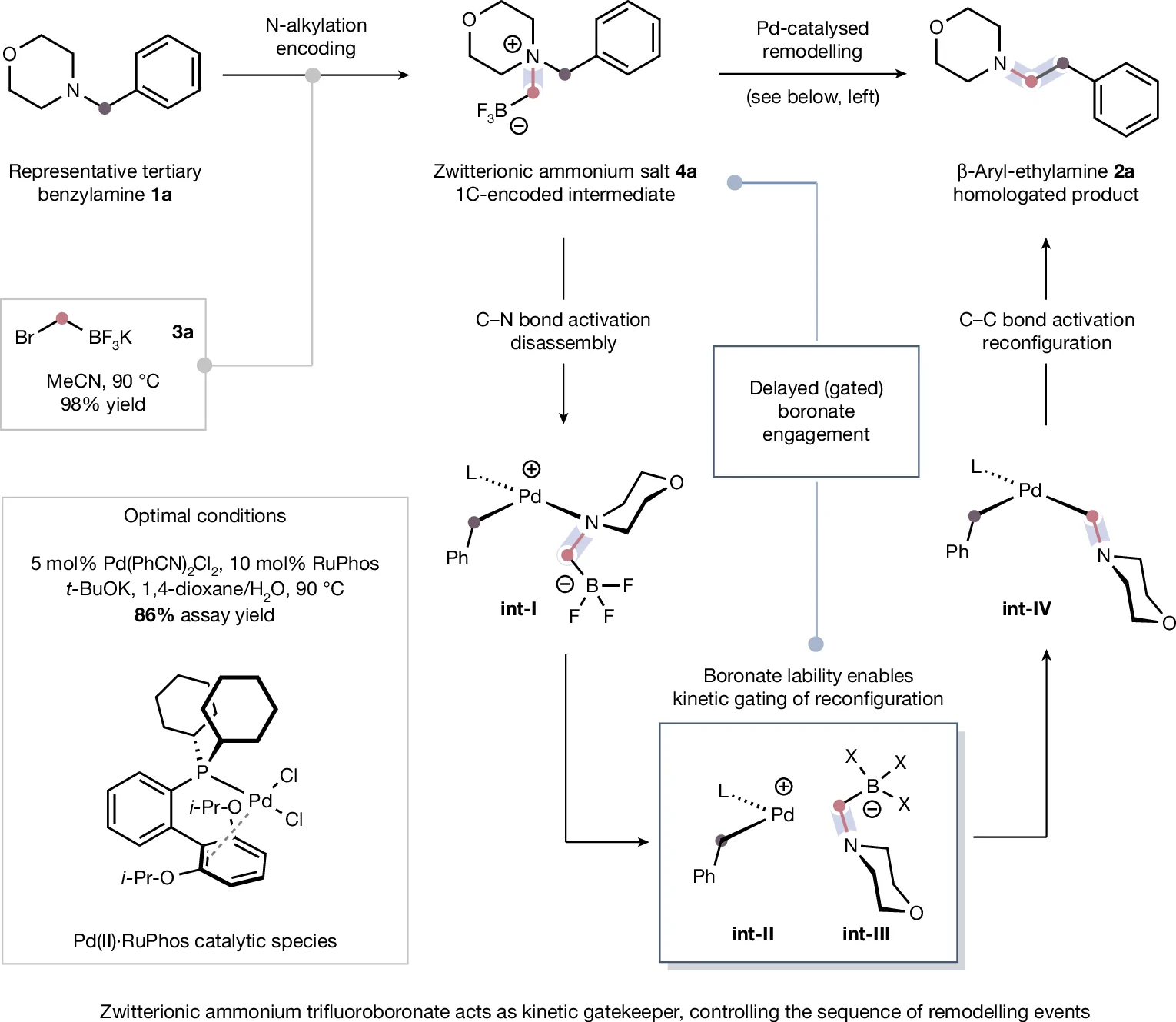
Reaction development and proposed mechanism for Pd-catalysed C–N bond remodelling. Optimization of reaction parameters identified the catalyst, ligand and base required for productive remodelling. A proposed mechanism for the process begins with N-alkylation to generate a quaternary ammonium intermediate that encodes the molecular unit for insertion. Subsequent oxidative addition, transmetalation and reductive elimination^34^ reconfigure the amine framework by forming a new C(*sp*^3^)–C(*sp*^3^) bond. Mechanistic studies support formation of a zwitterionic ammonium trifluoroborate intermediate that functions as a kinetic gatekeeper^33^, controlling the sequence of catalytic events leading to productive remodelling. RuPhos, a commercial phosphine ligand^31^.

To evaluate the feasibility of this remodelling concept, we began with a simple tertiary benzylamine substrate **1a**, for which encoding of a single-carbon linker would enable one-carbon homologation to the corresponding β-aryl–ethylamine **2a** (Fig. 2). Following our design logic, N-alkylation of amine **1a** with bromomethyl–BF_3_K (**3a**), a commercial ambiphilic reagent, furnished the corresponding quaternary ammonium salt **4a**, installing the additional carbon unit within a zwitterionic precursor to the catalytic C–N bond remodelling step. A complementary protocol using boronic ester derivatives, followed by conversion to the corresponding organotrifluoroborate^30^, also provides access to this intermediate under milder conditions (see Supplementary Information section 2.1.1 and Supplementary Tables 1 and 2).

With ammonium salt **4a** in hand, we next sought to implement a catalytic process that can remodel the C–N bond of the tertiary benzylamine scaffold to the β-aryl–ethylamine framework. After extensive evaluation of key reaction parameters (Supplementary Information section 2.1.2 and Supplementary Tables 3–7), a phosphine-ligated palladium system using RuPhos^31^ in combination with *t-*BuOK was identified that effects clean conversion of **4a** to the homologated product **2a** in high yield. A complementary N-heterocyclic carbene-based system (Pd·PEPPSI-IPent^32^/K_3_PO_4_), operating under milder basic conditions, proved effective for more sensitive substrates, establishing two broadly applicable catalytic regimes for the one-carbon homologation to the β-aryl–ethylamine analogue.

Although the transformation proceeds through steps consistent with palladium-mediated C–N bond activation^6,13,26^ of the ammonium species followed by fragment capture, its defining feature lies in the properties of intermediate **4a**. The quaternary ammonium trifluoroborate is solvolytically stable under the reaction conditions, consistent with the known behaviour of related zwitterionic organotrifluoroborates^33^. In the present system, however, this stability plays a distinct mechanistic part: it preserves the organoboron fragment (**int-I**) before C–N bond activation and amine dissociation (to **int-II** and **int-III**) from the palladium centre, after which rapid hydrolysis of the trifluoroboronate regulates the subsequent reconfiguration event (through **int-IV**). In this way, the zwitterionic species functions as a kinetic gatekeeper, enabling controlled remodelling of the amine framework (Supplementary Information section 2.3).

β-Aryl–alkylamines are accessible through several synthetic approaches^19–21^, including palladium-catalysed cross-coupling of pre-functionalized amine derivatives^34^. These methods, however, rely on the use of highly functionalized substrates, in which requisite reactivity is pre-installed and proceeds through a canonical bond-forming pathway without altering the intrinsic connectivity of the amine framework. By contrast, the present strategy engages the native tertiary benzylamine scaffold directly through a functionalized ammonium intermediate, enabling controlled disassembly and reconfiguration of C–N connectivity under catalytic conditions. The transformation, therefore, constitutes a remodelling of the underlying scaffold rather than a functionalization of a pre-assembled precursor.

The present catalytic manifold realizes the conceptual carbon transposition embodied by the classical 1,2-Stevens rearrangement through an entirely distinct catalytic mechanism^23^. Whereas classical Stevens rearrangements rely on substrate-controlled ylide reactivity and remain confined to a narrow and often unpredictable reaction space, the present system achieves catalyst-controlled reorganization of C–N connectivity in simple tertiary benzylamines. In doing so, it establishes the first general catalytic Stevens-type manifold for one-carbon homologation of tertiary benzylamines and provides a practical solution to this longstanding synthetic challenge.

## Scope evaluation

A stringent test of this one-carbon homologation concept lies in the direct late-stage editing of complex pharmaceutical molecules, in which synthetic constraints are most pronounced: an objective that typically requires de novo analogue construction. Subtle changes in the spacing between an amine and an aryl group are known to influence molecular conformation, recognition and physicochemical properties in bioactive compounds^35–38^, yet these variations are rarely accessible through direct modification of fully elaborated structures. Application of this C–N remodelling strategy to donepezil provides a validation of one-carbon homologation on tertiary benzylamine-containing drugs under the standard conditions, allowing controlled extension of aryl–amine connectivity (donepezil through the one-carbon-encoded ammonium intermediate to **5**; Fig. 4a).

**Fig. 3:**
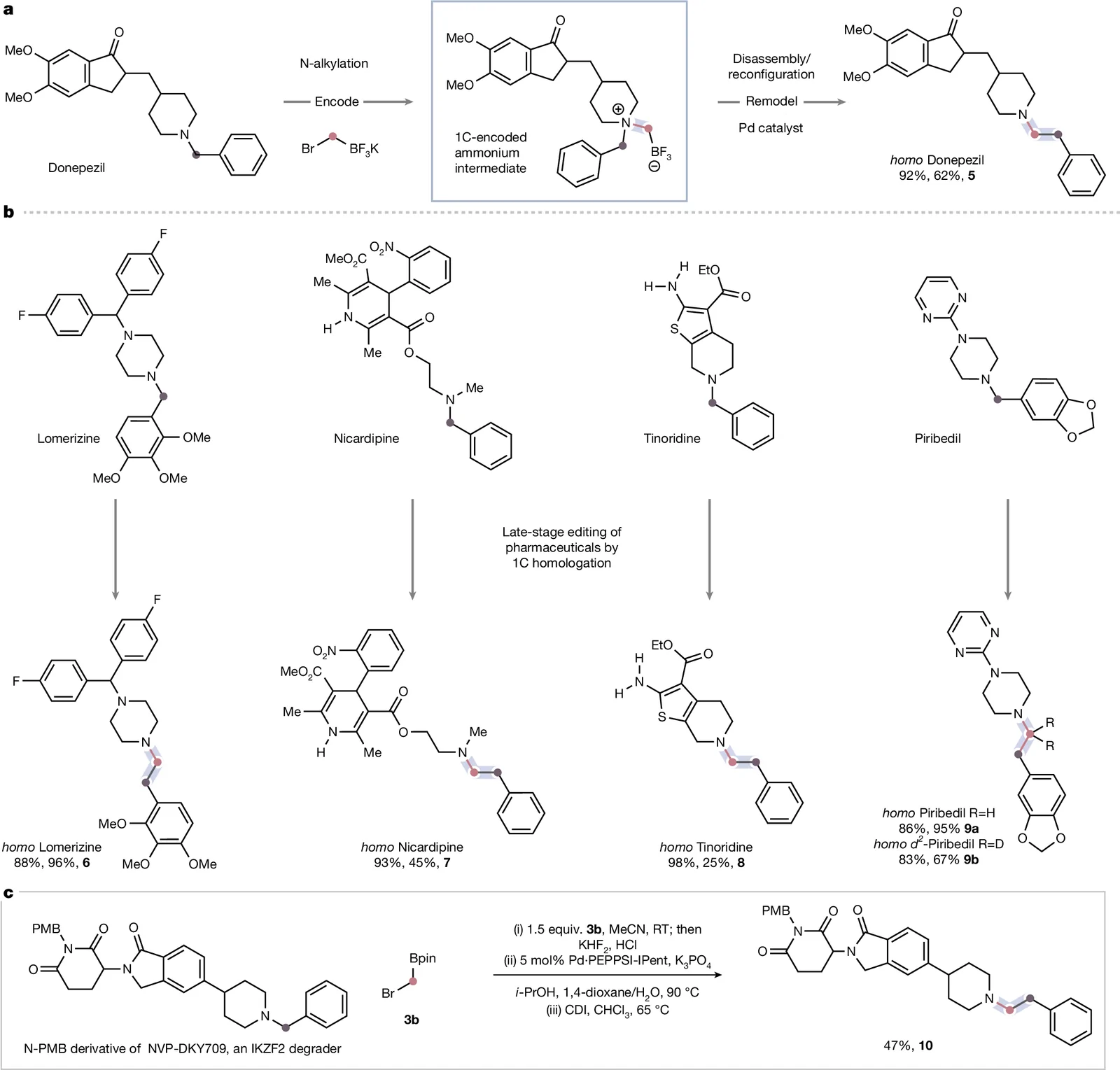
One-carbon late-stage editing of pharmaceuticals. **a**, One-carbon homologation of donepezil through N-alkylation to an encoded ammonium intermediate followed by Pd-catalysed remodelling. **b**, Late-stage editing across representative pharmaceutical scaffolds, enabling one-carbon insertion at tertiary benzylamine sites. **c**, Application to a complex IKZF2 degrader derivative, demonstrating compatibility with relevant functional features. Pd·PEPPSI-IPent, a commercial N-heterocyclic carbene-ligated palladium catalyst^32^.

One-carbon homologation of further representative pharmaceutical agents—including lomerizine, nicardipine, tinoridine, piribedil (Fig. 3b) and a derivative of the molecular glue degrader candidate NVP-DKY709 (ref. ^39^) (Fig. 3c)—proceeded with retention of heteroaromatic complexity and sensitive functionality across electronically diverse, densely functionalized and coordinating scaffolds (**5**–**10**). Where required, complementary catalytic conditions were used to accommodate more sensitive substrates (Supplementary Information sections 2.3.5.2 and 2.3.5.3). The versatility of this approach is further demonstrated using a deuterated ambiphilic methylene reagent, which furnished the corresponding CD_2_-extended analogue of piribedil (**9b**) with equal efficiency, highlighting the potential for rapid access to isotopically labelled derivatives. Collectively, these results demonstrate that programmable C–N bond remodelling enables direct late-stage structural editing of fully elaborated amine scaffolds, providing a practical platform for single-carbon chain extension of complex molecules in discovery settings.

**Fig. 4:**
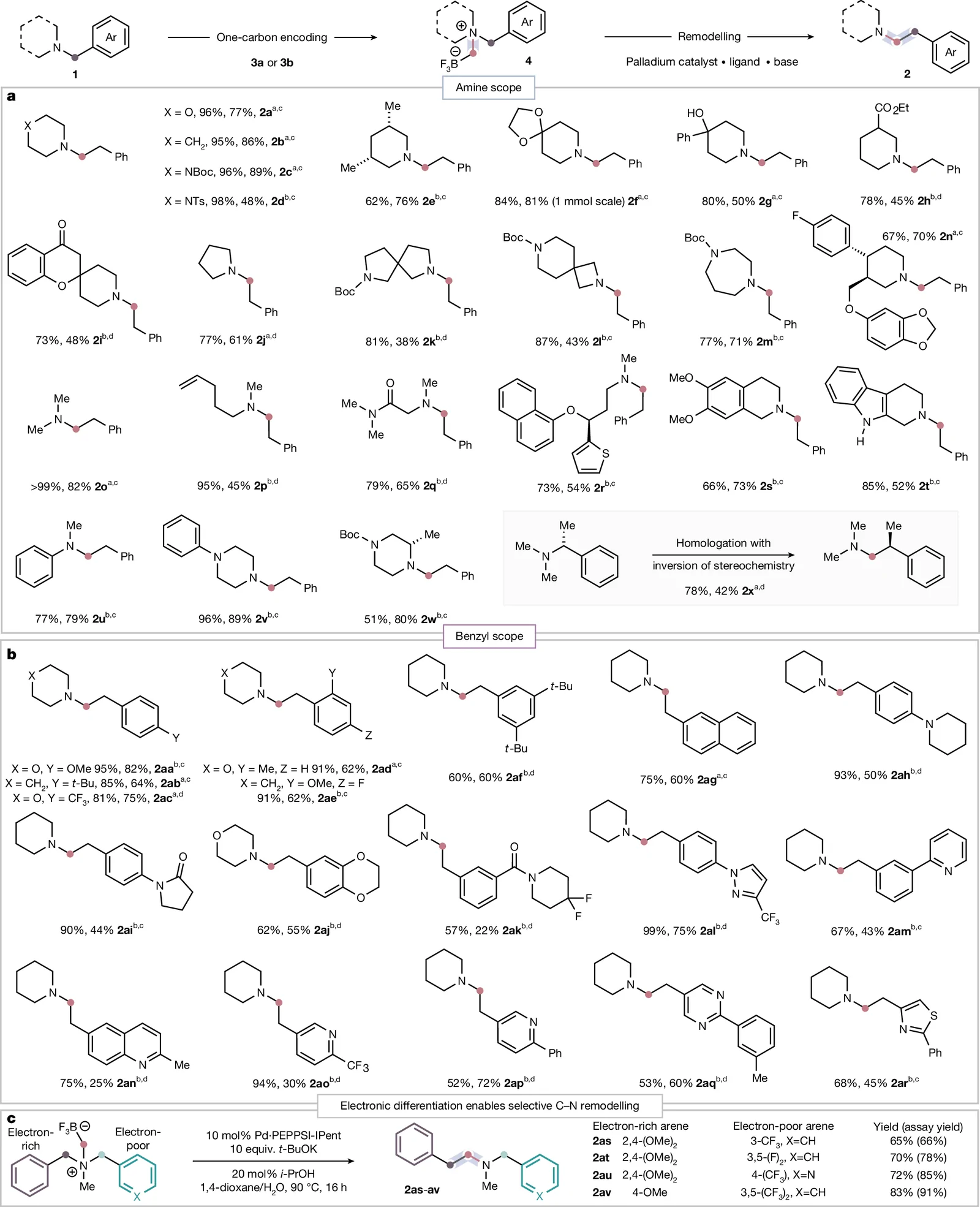
A broadly applicable one-carbon homologation of tertiary benzylamines. **a**, Scope of one-carbon homologation in amine component. **b**, Scope of one-carbon homologation in benzyl component. ^a^Alkylation with potassium (bromomethyl)trifluoroborate (**3a**). ^b^Alkylation with (bromomethyl)boronic acid pinacol ester (**3b**). ^c^Reaction with Pd(PhCN)_2_Cl_2_, RuPhos^31^ and *t*-BuOK. ^d^Reaction with Pd·PEPPSI-IPent^32^ and K_3_PO_4_. **c**, Selectivity between electronically distinct bis-benzylmethylamines: reaction favours homologation at the electron-rich benzyl unit. Boc, tert-butoxycarbonyl.

Beyond late-stage editing of pharmaceutical agents, the utility of this transformation lies in its broad applicability across structurally diverse tertiary benzylamines, the very motifs that commonly serve as entry points in discovery chemistry. To define this generality, we evaluated its performance across a representative set of amine architectures (Fig. 4). Although the RuPhos-ligated palladium system^31^ provided consistent performance across most substrates, the complementary PEPPSI-based catalyst system^32^, operating under milder basic conditions, proved important for enabling reactivity in more sensitive cases (Supplementary Information sections 2.3.5.2 and 2.3.5.3).

A wide range of tertiary benzylamines proved compatible, encompassing both cyclic and acyclic tertiary amines (Fig. 4a). Notably, morpholine, piperidine, pyrrolidine, azetidine and azepane derivatives (**2a**–**n**)—common motifs in medicinal chemistry—undergo efficient one-carbon homologation under the standard conditions. A dioxolane-protected piperidine underwent homologation to **2f** and serves as a convenient surrogate for primary amines^40^. Acyclic tertiary amines are likewise accommodated, including simple dialkylamines and substrates bearing various common functional groups (to form **2o**–**r**). Notably, substrates bearing α-carbonyl functionality, which introduce additional potentially labile C–N bonds, undergo selective remodelling at the benzylic position (**2q**), with no detectable activation of alternative sites. This chemoselectivity extends to more complex systems: *N*-benzyltetrahydroisoquinoline derivatives, which contain both exocyclic and endocyclic benzylic C–N bonds, undergo exclusive homologation at the exocyclic benzylic position, with no detectable activation of the endocyclic C–N bond (**2s**, **2t**). A substrate bearing both *N-*aryl and *N-*benzyl substituents at the quaternary centre undergoes selective homologation at the *N-*benzyl position (**2u**), despite the well-established propensity of aniline-derived ammonium salts to participate in cross-coupling reactions. A polyamine similarly engages the reaction selectively, through N-alkylation at the more reactive benzylamine nitrogen, demonstrating predictable site discrimination in multifunctional systems (**2v**). Sterically encumbered substrates, including α-substituted tertiary amines, also participate efficiently (**2w**), and enantiopure substrates undergo homologation (to **2x**) with complete conservation of optical purity, consistent with stereospecific inversion during C–N bond activation^41^.

Successful variation of the benzylic fragment further underscores the generality of this approach (Fig. 4b). Electronically and sterically diverse aryl groups, including ortho-substituted systems, undergo efficient homologation under the standard conditions (**2aa**–**ak**). The reaction is also compatible with a range of heteroaromatic motifs—including pyridines, pyrazoles, pyrimidines, thiazoles and quinolines—enabling direct access to β-heteroaryl–ethylamines (**2ai**–**ar**). Although heteroaromatic substrates can present competing sites for initial alkylation, this behaviour proved predictable and could be anticipated using a simple ^11^B NMR assay to assess alkylation selectivity (Supplementary Information section 2.2.2), enabling identification of substrates that engage selectively at the tertiary amine before remodelling. The functional and structural tolerance observed across these examples enables predictable chain extension directly on complex amine frameworks, offering a powerful and operationally simple strategy for lead optimization in medicinal chemistry.

An important extension of this strategy is the ability to selectively address individual benzylic C–N bonds within multiply benzylated amines. Whereas electronically similar benzyl substituents exhibit little intrinsic differentiation (Supplementary Information section 2.2.3), introducing a pronounced electronic bias between an electron-rich and an electron-deficient benzylic substituent enables highly chemoselective remodelling at the electron-rich benzylic position across a range of substrate classes, providing desired products **2as**–**av** in generally high assay yields (Fig. 4c). Rather than representing an isolated selectivity effect, these studies establish predictable electronic differentiation as a general design principle for programmable C–N remodelling, enabling individual benzylic C–N bonds to be selectively addressed within complex amine frameworks.

## Development of the amine remodelling platform

The one-carbon homologation process represents the minimal expression of a broader remodelling model, in which connectivity changes can be encoded and executed through catalytic bond activation. Incorporation of more complex architectures within the ambiphilic alkylating reagent translates this principle into direct insertion of discrete molecular units across the C–N bond, transforming tertiary benzylamines into programmable frameworks for controlled reconfiguration of amine connectivity. Realization of this concept required the development of ambiphilic reagents that can engage the catalytic manifold beyond single-carbon insertion. Initial attempts to extend the transformation using longer-chain bromoalkyl boronate derivatives—effectively demanding C(*sp*^3^)–C(*sp*^3^) bond formation under conditions susceptible to β-hydride elimination—demonstrated that although the corresponding three-carbon homologue could be obtained in modest yield, inefficient transmetalation and competing β-hydride elimination limited the efficiency of this approach (Supplementary Sections 2.4.1 and 2.4.2), necessitating a more sophisticated reactivity-matched design.

**Fig. 5:**
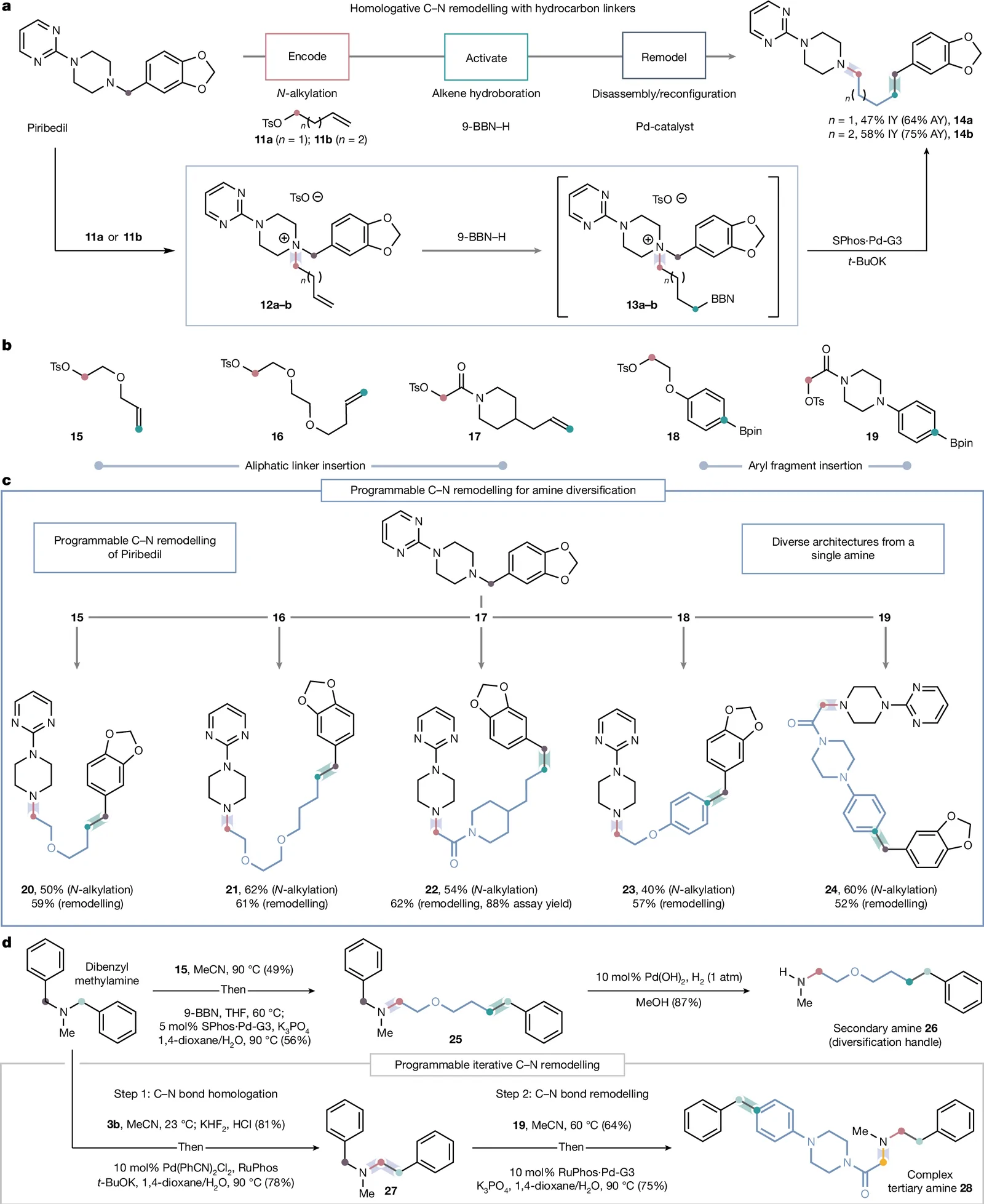
Programmable C–N remodelling of tertiary benzylamines. **a**, A unifying solution for the multi-carbon homologation of tertiary benzylamines. **b**, Bifunctional linker motifs. **c**, Programmable C–N remodelling of tertiary benzylamines to architecturally diverse ω-aryl alkylamines. Reaction of piribedil with **15,** **16** or **17**: N-alkylation with **15**, **16** or **17** in MeCN, 90 °C; 9-BBN, THF from **15** and **16** or (9-BBN)_2_, 1,4-dioxane from **17**; then 10 mol% SPhos·Pd-G3 (ref. ^31^), *t*-BuOK, 1,4-dioxane/H_2_O, 90 °C. Reaction of piribedil with **18** or **19**: N-alkylation with **18** or **19**, MeCN, 90 °C; 10 mol% RuPhos·Pd-G3, *t*-BuOK, 1,4-dioxane/H_2_O, 90 °C. **d**, Sequential remodelling of benzylic C–N bonds. 9-BBN, 9-borabicyclononane; SPhos·Pd-G3, a commercial palladium precatalyst; Ts, *para*-toluenesulfonyl.

A unified solution emerged through the use of alkenyl tosylates (**11a**, **11b**) as latent ambiphilic reagents (Fig. 5a), readily derived from abundant alkenol feedstocks: a tosylate leaving group to suppress degradation during N-alkylation and an embedded alkene to enable in situ hydroboration to generate a highly reactive organoborane intermediate, thereby accelerating transmetalation and promoting efficient catalytic turnover^42^. In this way, the bifunctional reagent is tuned to the catalytic cycle, remaining stable during alkylation yet becoming highly reactive for subsequent bond reconfiguration. This design proved effective in practice. Alkylation of piribedil with the butenyl and pentenyl tosylate reagents proceeded cleanly to the corresponding quaternary ammonium intermediates (**12a**, **12b**), which on hydroboration with 9-BBN (to **13a**,** 13b**) and Pd-catalysed remodelling using an SPhos ligand^31^ delivered the corresponding higher homologues (**14a**, **14b**) in good yield. Carbon chain length is thus encoded directly within the reagent, enabling programmable—multi-carbon—homologation without iterative bond construction. To the best of our knowledge, these transformations on amines have not been previously described^43–45^.

This chain extending ability is further expanded through incorporation of linkers of increasing structural complexity (**15**–**19**; Fig. 5b). Oxygenated linkers derived from ethylene glycol-based precursors (**15**, **16**) are readily introduced, enabling direct installation of polar and conformationally flexible motifs into tertiary benzylamines (**20**, **21**). Heterocyclic scaffolds can be embedded within the bifunctional reagent (**17**) and transferred intact during the remodelling sequence, allowing direct insertion of saturated ring systems between the aryl and amine fragments (to **22**). This represents a powerful mode of structural editing, enabling remodelling of amine connectivity through installation of cyclic motifs.

A distinct expansion in accessible chemical space arises from incorporation of aryl–boronic esters within the bifunctional reagent (**18**, **19**), enabling formation of new C(*sp*^2^)–C(*sp*^3^) bonds across the C–N linkage and insertion of rigid aryl motifs between the amine and aryl fragments (to **23**). This ability enables precise incorporation of rigid, π-rich motifs within the amine framework, introducing structural and electronic features that are often important in molecular design. Incorporation of both a saturated heterocycle and an aryl boronic ester within a single bifunctional reagent (**19**) enables formation of a highly elaborated ω-aryl alkylamine (**24**), demonstrating that multiple, structurally distinct elements can be integrated within a single remodelling event.

Beyond expanding the diversity accessible through individual remodelling reactions, the C–N activation strategy enables sequential editing of multiply benzylated amine frameworks (Fig. 5d). Following remodelling of one benzylic C–N bond in dibenzyl methylamine with bifunctional linker **15** (to **25**), the remaining benzylic C–N bond can be exploited independently. Thus, selective debenzylation after linker insertion provides direct access to a structurally differentiated secondary amine **26**, illustrating how one benzylic substituent can serve as a programmable handle, while the second remains available for subsequent diversification. More significantly, the residual benzylic C–N bond can itself undergo a subsequent remodelling event. For example, sequential one-carbon homologation of dibenzyl methylamine with **1b** (to **27**) followed by complex linker insertion (with **19**) and remodelling furnishes a highly elaborated tertiary amine **28** wherein two distinct C–N remodelling operations are leveraged from a readily accessible dibenzylamine precursor. Rather than representing isolated synthetic transformations, these studies illustrate programmable iterative C–N remodelling as a practical strategy for the stepwise evolution of tertiary benzylamines into architecturally and functionally distinct amine scaffolds.

## Outlook

This C–N bond remodelling concept enables modular insertion of diverse structural elements—including single carbon, aliphatic chains, polar linkers, heterocycles and aryl groups—directly across the benzylamine linkage. The breadth of linker topology aligns closely with the demands of emerging modalities—from PROTACs to molecular glues to bifunctional chemical probes—positioning this activation mode as a powerful platform for molecular design^46–50^. Importantly, transformations that reconfigure amine connectivity within complex molecular architectures are not accessible through existing synthetic strategies. More fundamentally, the ability to encode both activation and reconstruction within a single reagent shows a general principle: amine connectivity can be programmed through reagent design and catalytic control. In this way, C–N bonds are transformed from static structural features into dynamic loci for reconfiguration, providing a foundation for rapid diversification and exploration of amine-containing chemical space in discovery chemistry.

## Online content

Any methods, additional references, Nature Portfolio reporting summaries, source data, extended data, supplementary information, acknowledgements, peer review information; details of author contributions and competing interests; and statements of data and code availability are available at https://doi.org/10.1038/s41586-026-11009-1.

## Supplementary information

Supplementary Information This file contains the following sections: (1) Materials and methods; (2) Reaction optimization and procedures; (3) Substrate scope; (4) NMR spectra; and (5) References.

Peer Review File

## Data Availability

All data are available in the manuscript or Supplementary Information.
