# Supplementary material for: Programmable remodelling of carbon–nitrogen connectivity in amines
Source: Nature. 2026 Aug 17;658(8134):94–102. doi: 10.1038/s41586-026-11009-1 (PMC13626948; doi:10.1038/s41586-026-11009-1)
Supplement: Supplementary file 1 [file 41586_2026_11009_MOESM1_ESM.pdf]

nature portfolio

https://doi.org/10.1038/s41586-026-11009-1

Supplementary information

Programmable remodelling of carbon–nitrogen connectivity in amines

In the format provided by the authors and unedited

Supplementary Information for

Programmable remodeling of carbon-nitrogen connectivity in amines

James D. Robinson[‡], François Richard[‡], Kohl A. Ratkovich[‡], Joseph M. Phelps and Matthew J. Gaunt*

Yusuf Hamied Department of Chemistry, University of Cambridge, Lensfield Road, Cambridge, United Kingdom. CB2 1EW.

Correspondence to: mjg32@cam.ac.uk

Table of Contents

1. Materials and Methods....................S1
2. Reaction Optimisation and Procedures....................S3
    2.1. Single-Carbon Homologation Optimisation....................S3
    2.2. NMR-Based Assessment of Alkylation Selectivity....................S10
    2.3. Mechanistic Studies....................S13
    2.4. Carbon-Nitrogen Bond Remodeling Optimisation....................S16
    2.5. General Procedures....................S25
3. Substrate Scope....................S31
    3.1. Starting Materials....................S31
    3.2. Betaine Intermediates....................S61
    3.3. Phenethylamine Products....................S96
    3.4. Carbon-Nitrogen Bond Remodeling Intermediates and Products....................S128
4. NMR Spectra.................... S154
5. References....................S393

1. Materials and Methods

All reactions were run under an inert atmosphere (N_2) unless otherwise stated, with oven-dried glassware, using standard techniques. Yields refer to isolated, chromatographically and spectroscopically (^{1}H NMR) homogeneous materials, unless otherwise indicated. Diastereomeric ratios were determined using crude ^{1}H NMR analysis unless stated otherwise. All synthesised compounds that had been previously reported in literature gave rise to spectra in agreement with the literature reference cited. Anhydrous solvents were obtained from Acros (Extra Dry, AcroSeal) or from solvent stills where they were dried and distilled using standard methods (Perrin, D. D.; Armarego, W. L. F. *Purification of Laboratory Chemicals*; Pergamon Press: Oxford, 1997). Amines and aldehydes were distilled prior to use and stored at 5 °C under N_2. All other commercial reagents were used as supplied unless otherwise stated. Petroleum ether refers to the fraction of hydrocarbons in petroleum with a boiling point between 40 ˚C and 60 ˚C.

Analytical thin-layer chromatography (TLC) was performed on Merck Kieselgel 60 F254 0.20 mm precoated, glass backed silica gel plates. Visualisation of the developed chromatogram was performed by UV absorbance (λ_{max} = 254 nm) and/or by aqueous $KMnO_4$ or ninhydrin stain. Flash column chromatography was performed using silica gel (Merck Geduran Si 60 [40–63 μm]) with the indicated solvent system, or using a Teledyne ISCO Combi*Flash* NextGen 100 using silica columns unless specified otherwise. Reversed-phase flash column chromatography was performed using RediSep Gold C18Aq reversed-phase columns (20–40 μm spherical media). Strong cation exchange (SCX) chromatography was performed using HyperSep SCX cartridges (1000 mg bed weight) from Thermo Scientific. Centrifugation was performed using an Eppendorf MiniSpin Microcentrifuge with F-45-12-11 Rotor for 12 × 1.5/2.0 mL Tubes.

Nuclear magnetic resonance (NMR) spectra were recorded at 400 MHz, 500 MHz, 600 MHz or 700 MHz on Bruker AM-400/500/600/700 instruments at 298 K. Samples were run in deuterated solvents. Chemical shifts (δ) for ^{1}H NMR spectra are reported in parts per million (ppm) relative to tetramethylsilane referenced to residual protic solvent ($CDCl_3$ = 7.26, CD_2Cl_2 = 5.32, DMSO-*d*6 = 2.50, CD_3OD = 3.31, acetone-*d*6 = 2.05, acetonitrile-*d*3 = 1.94 ppm) Coupling constants (*J*) are reported in Hertz (Hz). Abbreviations for splitting patterns are as follows: s, singlet; d, doublet; t, triplet; q, quartet; p, pentet; hept, heptet; oct, octet; m, multiplet; br., broad. Where coincident or

additional coupling constants were observed, the apparent (app.) multiplicity of the resonance is reported. Chemical shifts (δ) for ^{13}C NMR spectra were recorded with complete proton decoupling and are reported in ppm from tetramethylsilane referenced to the solvent resonance ($CDCl_3$ = 77.16 ppm, CD_2Cl_2 = 53.84 ppm, DMSO-*d*6 = 39.52, CD_3OD = 49.00 ppm, acetone-*d*6 = 29.84, acetonitrile-*d*3 = 118.26 ppm). ^{13}C chemical shifts are reported to one decimal place unless an additional decimal place is required to differentiate two close-lying resonances. Chemical shifts (δ) for ^{19}F spectra are reported in ppm. Spectra were analysed using MNova software. All the carbons attached to boron were not found due to the quadrupolar relaxation. ^{1}H NMR yields were determined by analysis of crude reaction mixtures with reference to 1,1,2,2-tetrachloroethane or 1,3-dinitrobenzene as an internal standard.

Infrared (IR) spectra were recorded using a Thermo Fisher Scientific Nicolet Summit PRO FTIR Spectrometer or an Agilent Cary 630 FTIR Spectrometer as solids or films, either through direct application or deposited as solutions. Absorption maxima (ν_{max}) are given in wavenumbers in units of cm^{-1}.

High-resolution mass spectrometry (HRMS) experiments were carried out using a Micromass Q-TOF or Waters XEVO GII-S Q-TOF mass spectrometer (measured by service staff at the Yusuf Hamied Department of Chemistry) or a Shimadzu LCMS-9030 Q-TOF mass spectrometer (measured in the Gaunt group by R. Kang, B. Kraus, M. Pychtin or A. Lelke) with the specified ionisation method (ESI, electrospray ionisation; ASAP, atmospheric solids analysis probe).

Compound names are those generated by ChemDraw Professional 16.0 software (PerkinElmer), following the IUPAC nomenclature.

2. Reaction Optimisation and Procedures

2.1. Single-Carbon Homologation Optimisation and Procedures

2.1.1. Tertiary Benzylamine Alkylation Optimisation Studies

Experimental procedure: An oven-dried round-bottom flask or 4 mL PTFE/Silicone-lined septa screw cap clear glass vial was charged with a stirrer bar. The flask was sealed and evacuated-refilled with nitrogen (3 cycles). Anhydrous acetonitrile (0.40 – 0.80 mL), 1-benzylmorpholine or 1-benzylpiperidine (**1a** or **1b**) (45 μL, 0.20 mmol, 1.0 equiv), and then (bromomethyl)boronic acid pinacol ester or potassium (bromomethyl)trifluoroborate (**3a** or **3b**) (1.0 – 1.5 equiv) were then added. The reaction mixture was stirred at room temperature or at 50 ˚C for 4 hours or 24 hours. The solvent was removed *in vacuo* and conversion was measured using ^{1}H NMR spectroscopy using 1,1,2,2-tetrachloroethane (21 μL, 0.20 mmol, 1.0 equiv) as an internal standard.

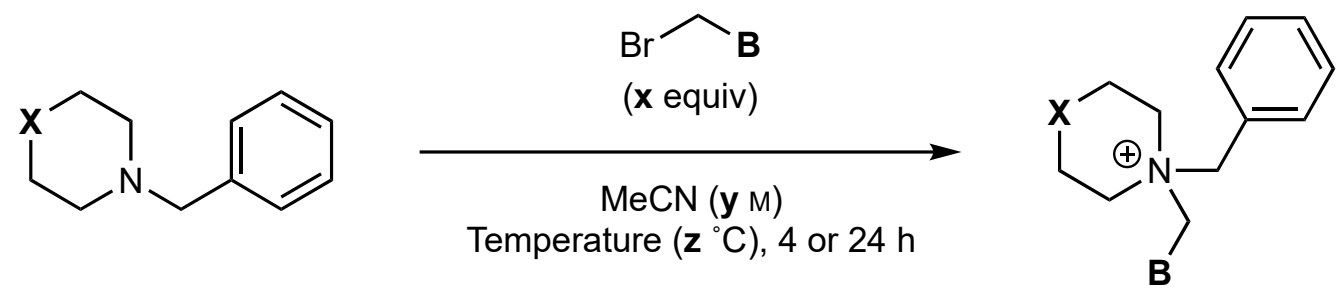

1a or **1b**

Entry	X	B	x (equiv)	y (M)	z (˚C)	Time (hours)	Yield (%)
1	O	BF_3	1	0.50	90	4	26
2	O	BF_3	1.5	0.50	90	24	98
3	CH_2	BF_3	1.5	0.50	90	24	94
3	CH_2	Bpin	1	0.50	room temperature	4	57
4	CH_2	BPin	1	0.50	50	4	57
5	CH_2	Bpin	1.2	0.50	50	4	53
6	CH_2	Bpin	1.5	0.50	50	4	48
7[a]	CH_2	Bpin	1.5	0.50	50	4	63
8[a]	CH_2	Bpin	1.5	0.50	room temperature	24	>99
9	O	Bpin	1.5	0.50	room temperature	24	>99

Table S1 | Optimisation of the *N*-alkylation between 1-benzylpiperidine and (bromomethyl)boronic acid pinacol ester – assay yields were generated via ^{1}H NMR spectroscopy using 1,1,2,2-tetrachloroethane as an internal standard. [a]The reaction was performed in a round-bottom flask.

An oven-dried round-bottom flask was charged with a stirrer bar and 1-benzyl-4-phenylpiperazine (**1v**) (252 mg, 1.0 mmol, 1.0 equiv) (if necessary). The flask was sealed and evacuated-refilled with nitrogen (3 cycles). Anhydrous solvent (2.0 mL), 1-benzylmorpholine or 1-benzylpiperidine (**1a** or **1b**) (1.0 equiv) (if necessary), and (bromomethyl)boronic acid pinacol ester (331 mg, 1.5 mmol, 1.5 equiv) were then added. The reaction mixture was stirred at room temperature until full consumption of amine was observed via TLC (1–72 hours). Aqueous potassium hydrogenfluoride (3 M, 0.50–0.75 mL, 1.5–2.3 equiv) and hydrogen chloride (4 M in 1,4-dioxane, 0.25–0.50 mL, 1.0–2.0 equiv) were then added. The reaction mixture was then diluted with anhydrous acetonitrile and HPLC grade water to give final concentration 4:1 MeCN/H_2O (0.10 M). The reaction mixture was stirred at room temperature for 8 hours. The solvent was removed *in vacuo* and conversion was measured using ^{1}H NMR spectroscopy using 1,1,2,2-tetrachloroethane (106 µL, 1.0 mmol, 1.0 equiv) as an internal standard.

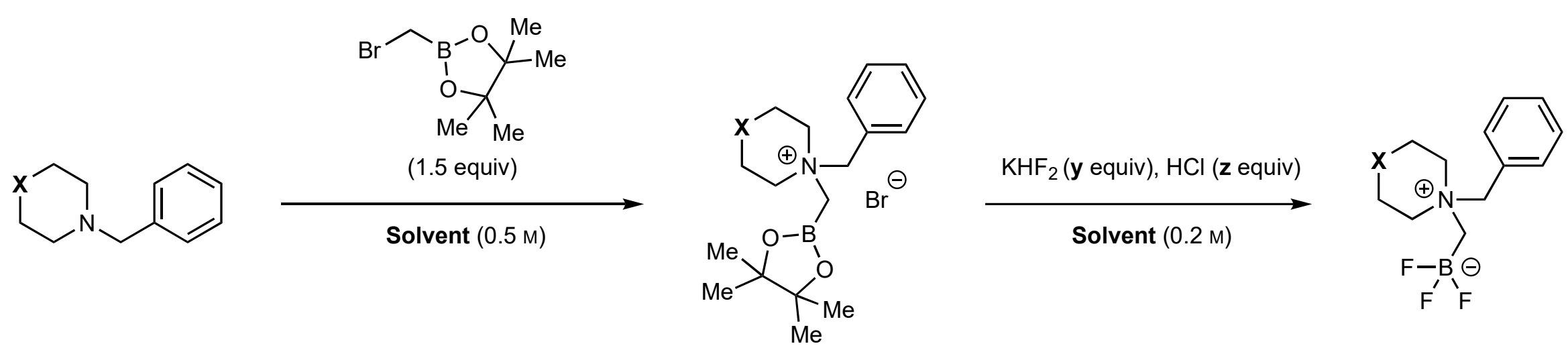

Entry	X	y (equiv)	z (equiv)	Solvent	Yield 4 (%)
1	CH_2	1.5	1.0	1,4-dioxane	68
2	CH_2	2.3	1.0	1,4-dioxane	98
3	CH_2	2.3	1.0	1,4-dioxane	99
4	NPh	2.3	1.0	acetonitrile	73
5	NPh	2.3	2.0	acetonitrile	99
5	O	2.3	2.0	acetonitrile	96

Table S2 | Optimisation of a one-pot procedure for alkylation of benzylamines with (bromomethyl)boronic acid pinacol ester – assay yields were generated via quantitative ^{1}H NMR spectroscopy using 1,1,2,2-tetrachloroethane as an internal standard.

2.1.2. Betaine Coupling Optimisation Studies

Initial one-carbon coupling optimisation

Experimental procedure: An oven-dried 4 mL PTFE/Silicone-lined septa screw cap clear glass vial equipped with a stirrer bar was charged with betaine (**4a**) (26 mg, 0.10 mmol, 1.0 equiv),

catalyst (2–10 mol%), ligand (4–20 mol%) (if necessary), and base (2.0–15 equiv) before being sealed and evacuated-refilled with nitrogen (3 cycles). A degassed solvent mixture (0.3–2.0 mL) was added. The reaction mixture was heated at 90 ˚C overnight (16 hours) with vigorous stirring (1000 rpm). Upon completion the reaction mixture was filtered through Celite©. The solvent was removed *in vacuo* and conversion was measured using ^{1}H NMR spectroscopy using 1,1,2,2-tetrachloroethane (11 μL, 0.10 mmol, 1.0 equiv) as an internal standard.

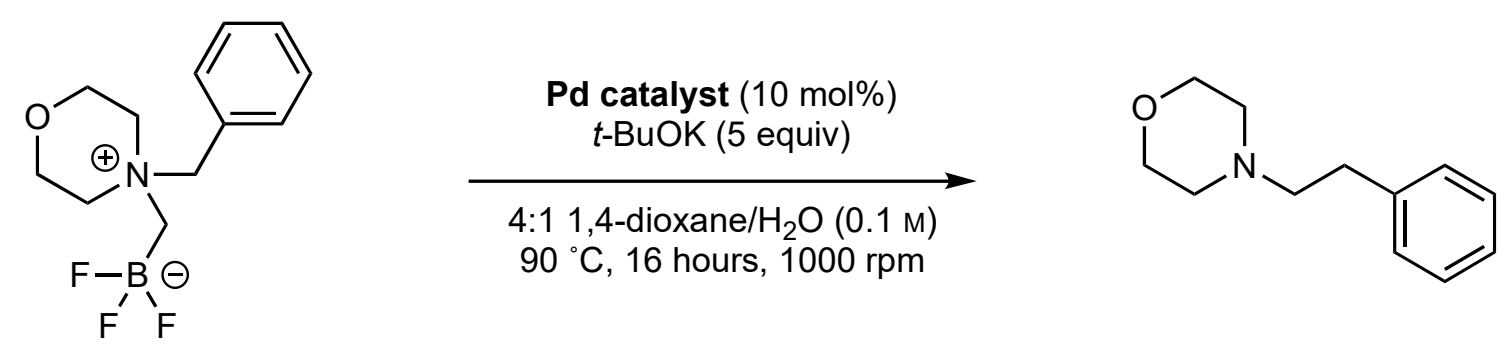

Entry	Pd Catalyst	Yield 2a (%)
1	PPh_3 Pd G2	0
2	P(*t*-Bu)$_3$ Pd G3	3
3	cataCXium A Pd G3	0
4	BrettPhos Pd G3	36
5	AdBrettPhos Pd G3	19
6	XPhos Pd G3	67
7	*t*-BuXPhos Pd G3	23
8	MorDalPhos Pd G3	0
9	XantPhos Pd G2	6
10	SPhos Pd G3	50
11	DavePhos Pd G3	36
12	CPhos Pd G3	64
13	RuPhos Pd G3	70
14	Pd-PEPPSI™-IPent	63
15	Pd-PEPPSI™-IPr	17

Table S3 | Catalyst screen for the one-carbon homologation of benzylic amines – assay yields were generated via quantitative ^{1}H NMR spectroscopy using 1,1,2,2-tetrachloroethane as an internal standard.

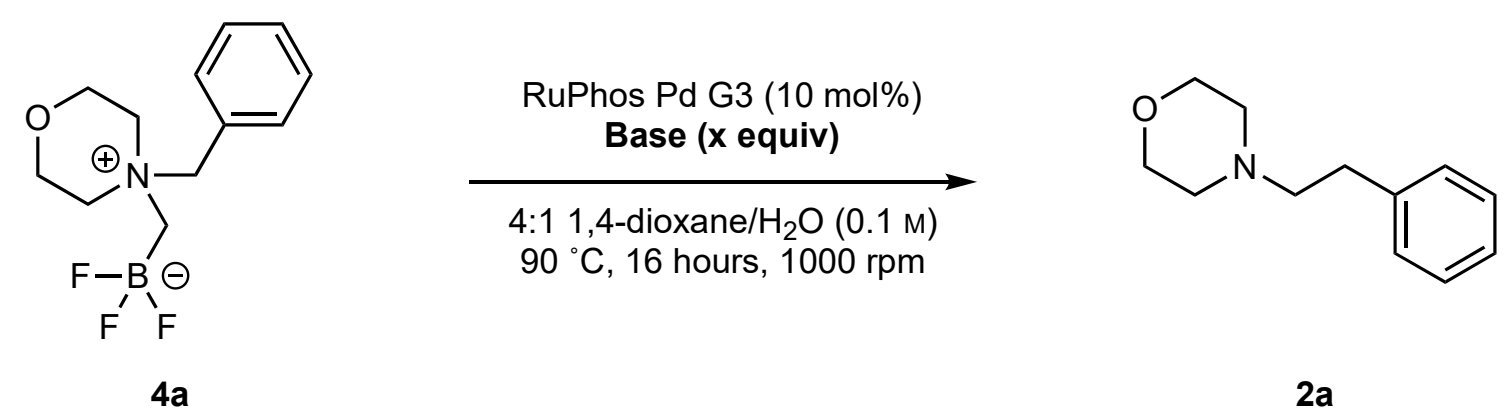

Entry	Base	x (equiv)	Yield 2a (%)
1	none	0	0
2	*t*-BuOK	5.0	70
3	*t*-BuONa	5.0	56
4	*t*-BuOLi	5.0	55
5	KOH	5.0	67
6	Na_3PO_4	5.0	47
7	K_3PO_4	5.0	54
8[a]	K_3PO_4	10.0	75
9	Na_2CO_3	5.0	34
10	K_2CO_3	5.0	30
11	Cs_2CO_3	5.0	46
12	CsF	5.0	0
13	KOAc	5.0	0
14	NEt_3	5.0	0
15	MTBD	5.0	0
16	*t*-BuOK	2.0	29
17	*t*-BuOK	3.0	40
18	*t*-BuOK	5.0	70
19	*t*-BuOK	7.0	72
20	*t*-BuOK	10	71
21	*t*-BuOK	15	68

Table S4 | Base screen for the one carbon homologation of benzylic amines – assay yields were generated via quantitative ^{1}H NMR spectroscopy using 1,1,2,2-tetrachloroethane as an internal standard. [a] Pd-PEPPSI-IPent was used instead of RuPhos Pd G3.

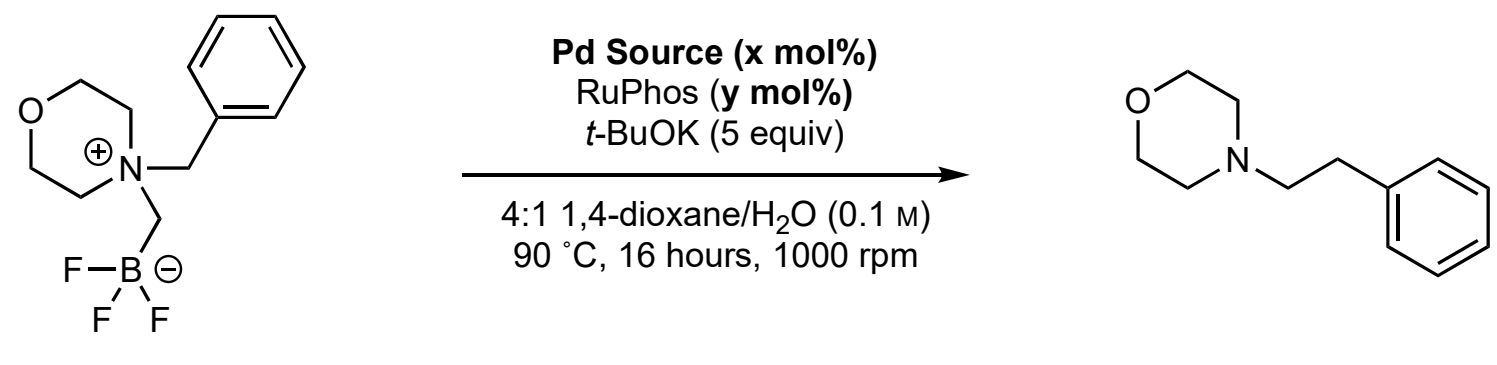

Entry	Pd Source	x (mol%)	Ligand	y (mol%)	Yield 2a (%)
1	RuPhos Pd G3	10	none	0	70
2	$Pd_2(dba)_3$	5	RuPhos	20	64
3	$Pd(PPh_3)_4$	10	RuPhos	20	18
4	$PdCl_2$	10	RuPhos	20	0
5	$PdBr_2$	10	RuPhos	20	6
6	PdI_2	10	RuPhos	20	0
7	$Pd(MeCN)_4(BF_4)_2$	10	RuPhos	20	68
8	$Pd(PhCN)_2Cl_2$	10	RuPhos	20	85
9	$Pd(OAc)_2$	10	RuPhos	20	54
10	$Pd(acac)_2$	10	RuPhos	20	74
11	$Pd(dppf)Cl_2$	10	RuPhos	20	57
12	$Pd(bpy)Cl_2$	10	RuPhos	20	0
13	$Pd(PhCN)_2Cl_2$	10	RuPhos	10	64
14	$Pd(PhCN)_2Cl_2$	10	RuPhos	15	76
15	$Pd(PhCN)_2Cl_2$	10	RuPhos	20	85
16	$Pd(PhCN)_2Cl_2$	8	RuPhos	15	78
17	$Pd(PhCN)_2Cl_2$	5	RuPhos	10	83
18	$Pd(PhCN)_2Cl_2$	2	RuPhos	4	69

Table S5 | Palladium source screen for the one carbon homologation of benzylic amines – assay yields were generated via quantitative ^{1}H NMR spectroscopy using 1,1,2,2-tetrachloroethane as an internal standard.

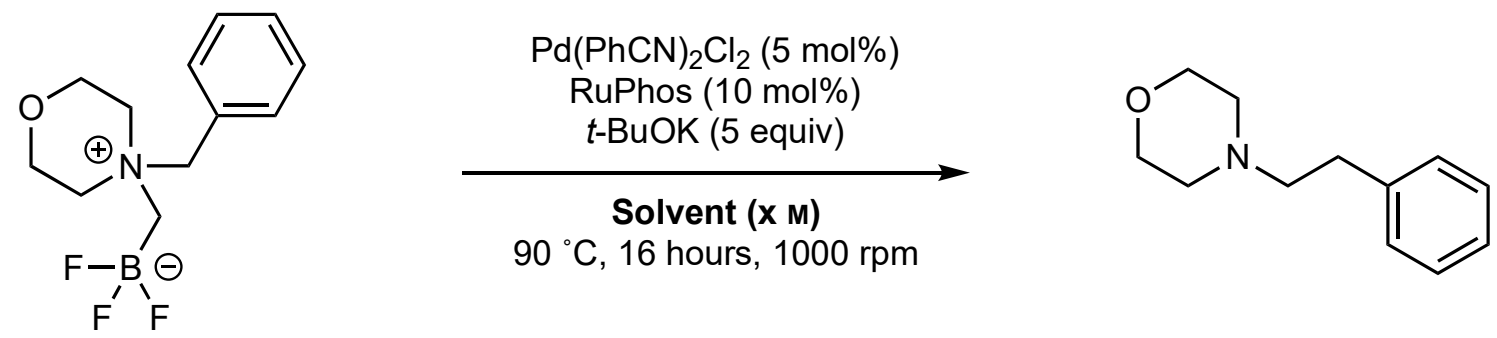

4a **2a**

Entry	Solvent Identities	Solvent Ratio	x (M)	Yield 2a (%)
1	1,4-dioxane/H_2O	4:1	0.10	83
2	*t*-AmOH/H_2O	4:1	0.10	34
3	1,2-dichloroethane/H_2O	4:1	0.10	0
4	toluene/H_2O	4:1	0.10	27
5	THF/H_2O	4:1	0.10	50
6	1,4-dioxane/H_2O	4:1	0.30	85
7	1,4-dioxane/H_2O	4:1	0.20	86
8	1,4-dioxane/H_2O	4:1	0.10	83
9	1,4-dioxane/H_2O	4:1	0.05	76
10	1,4-dioxane	N/A	0.10	0
11	1,4-dioxane/H_2O	19:1	0.10	4
12	1,4-dioxane/H_2O	9:1	0.10	51
13	1,4-dioxane/H_2O	4:1	0.10	84
14	1,4-dioxane/H_2O	3:2	0.10	56
15	1,4-dioxane/H_2O	1:1	0.10	33

Table S6 | Solvent screen for the one carbon homologation of benzylic amines – assay yields were generated via quantitative ^{1}H NMR spectroscopy using 1,1,2,2-tetrachloroethane as an internal standard.

Additive screening for palladium catalyst activation

Experimental procedure: An oven-dried 4 mL PTFE/Silicone-lined septa screw cap clear glass vial equipped with a stirrer bar was charged with betaine (**4b**) (26 mg, 0.10 mmol, 1.0 equiv), Pd-PEPPSI^{TM}-IPent (4 mg, 0.005 mmol, 5 mol%), base (0.50 mmol, 5.0 equiv) and additive (0.02–0.04 mmol, 20–40 mol%) before being sealed and evacuated-refilled with nitrogen (3 cycles). A degassed 4:1 mixture of 1,4-dioxane and HPLC grade water was added (0.5 mL). The reaction mixture was heated at 90 ˚C overnight (16 hours) with vigorous stirring (1000 rpm). Upon completion the reaction mixture was filtered through Celite© and concentrated *in vacuo*. Yield was determined using ^{1}H NMR spectroscopy using 1,1,2,2-tetrachloroethane (11 μL, 0.10 mmol, 1.0 equiv) as an internal standard.

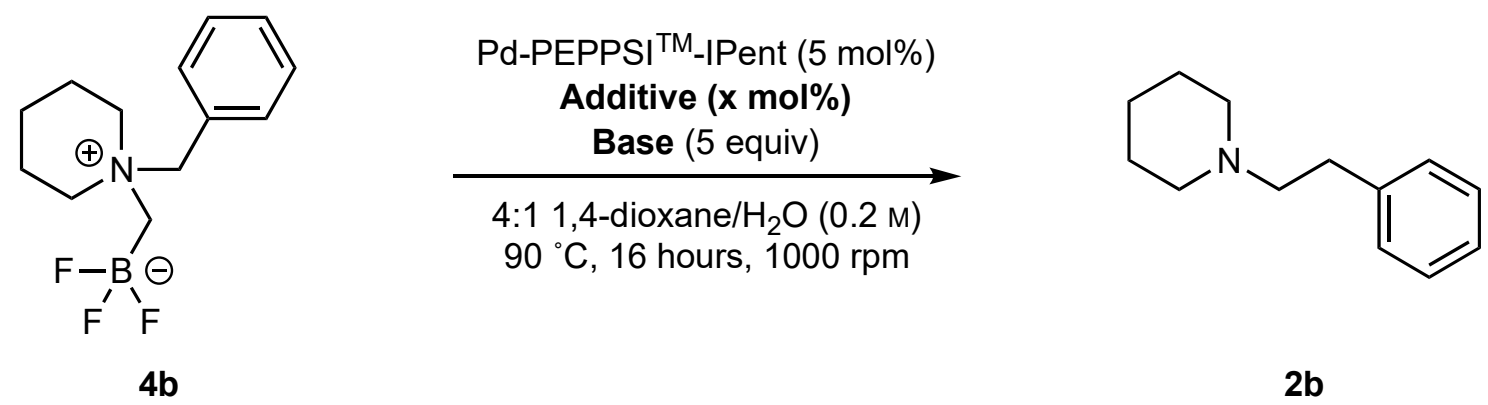

Entry	Additive	x mol%	Base	Conv. (%)	Yield 2b (%)
1	Pinacol	20	*t*-BuOK	15	0
2	KF	20	*t*-BuOK	0	0
3	KBr	20	*t*-BuOK	6	0
4	$BrCH_2BF_3K$	20	*t*-BuOK	94	64
5	$BrCH_2BF_3K$	40	*t*-BuOK	100	63
6	BHT	40	*t*-BuOK	100	68
7	*i*-PrOH	40	*t*-BuOK	100	69
8	BHT	40	K_3PO_4	100	54
9	*i*-PrOH	40	K_3PO_4	100	57

Table S7 | Additive screening for Pd activation – assay yields were generated via quantitative ^1H NMR spectroscopy using 1,1,2,2-tetrachloroethane as an internal standard.

i-PrOH (Entry 7 and 9) was selected as an additive for the methodology as reproducibility issues have been noted when using BHT.

2.2. NMR-Based Assessment of Alkylation Selectivity

2.2.1. ^{11}B NMR Reference

References of (bromomethyl)boronic acid pinacol ester and ammonium salt **4b** were measured in CD_3CN (Spectra 1 and 2).

Experimental procedure: To a solution of pyridine (8.7 µL, 0.10 mmol, 1.0 equiv) in MeCN (0.5 mL) was added (bromomethyl)boronic acid pinacol ester (33 mg, 0.15 mmol, 1.5 equiv). The reaction was stirred at 900 rpm for 30 minutes, after which 0.3 mL of the reaction were taken into an NMR tube and diluted with 0.3 mL of CD_3CN (Spectrum 3).

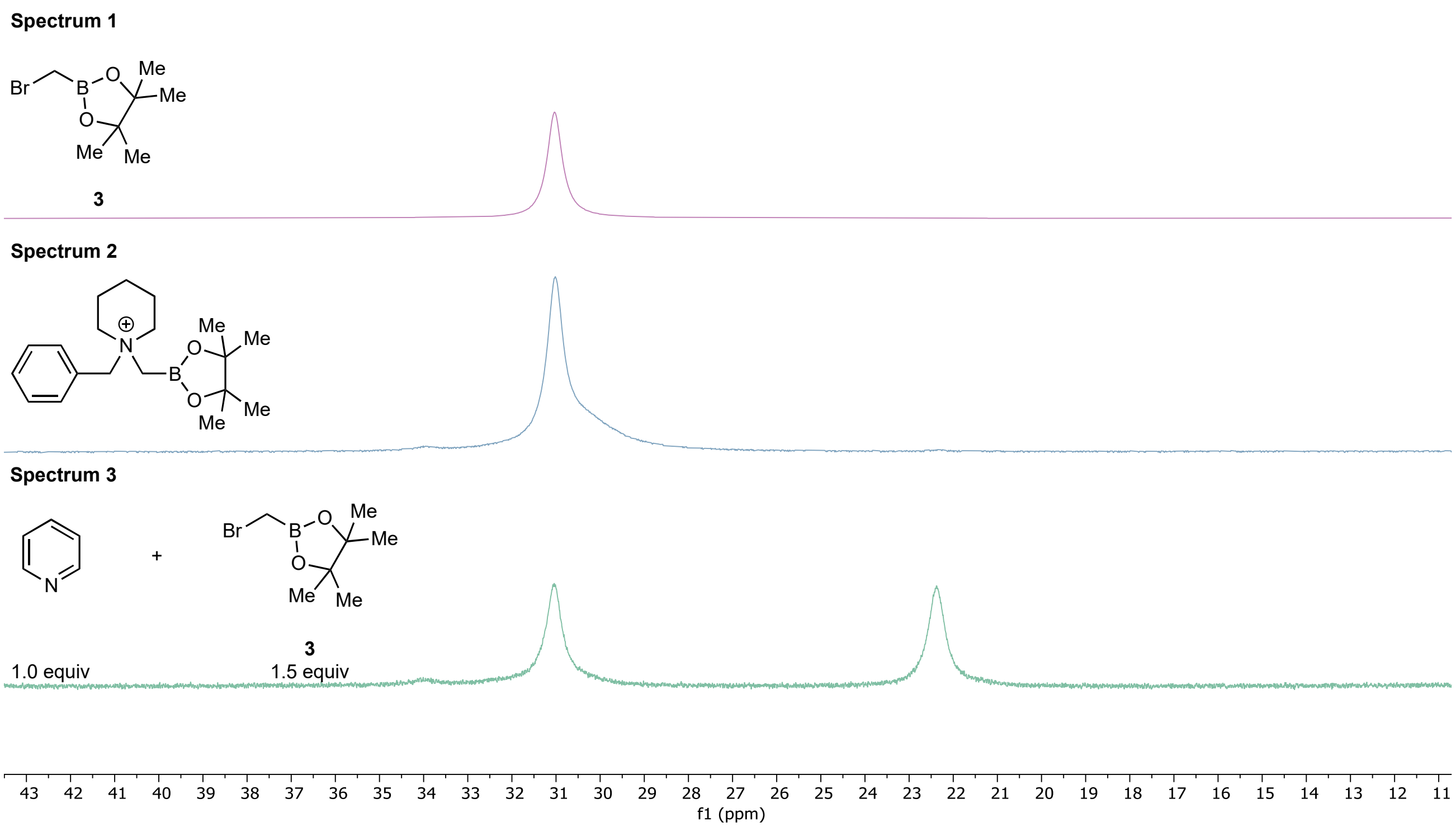

Figure S1 | ^{11}B NMR reference spectra for (bromomethyl)boronic acid pinacol ester, ammonium salt and pyridine/(bromomethyl)boronic acid pinacol ester mixture.

2.2.2. ^{11}B NMR Selectivity Assessment Procedure

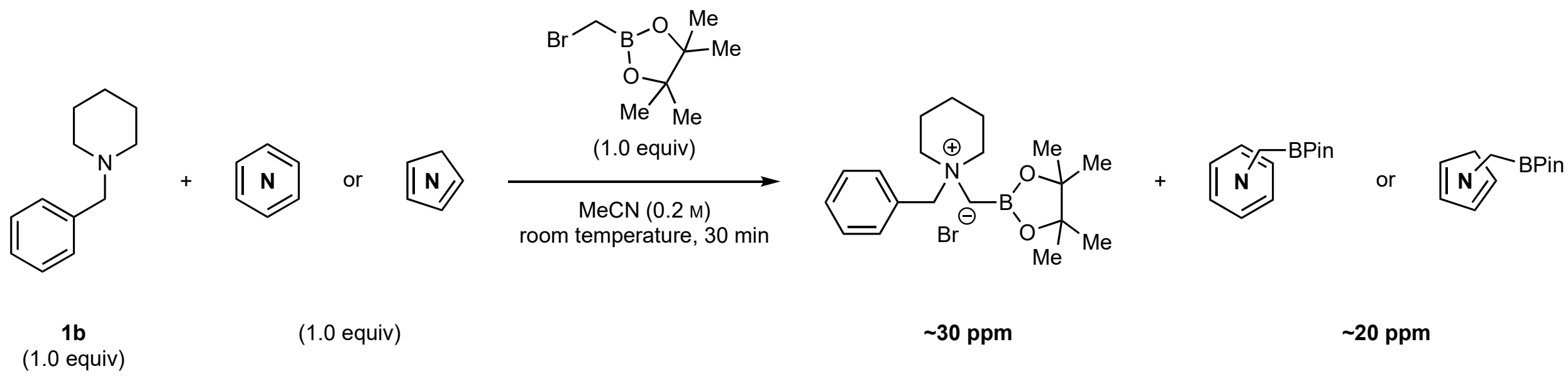

Experimental procedure: To a solution of 1-benzylpiperidine **1b** (18 mg, 0.10 mmol, 1.0 equiv) in MeCN (0.50 mL) was added the desired heterocycle (0.10 mmol, 1.0 equiv). The reaction was stirred at 900 rpm for 5 minutes, followed by the addition of (bromomethyl)boronic acid pinacol ester (22 mg, 0.10 mmol, 1.0 equiv). The resulting mixture was left to stir 30 minutes at 900 rpm, after which 0.30 mL of the reaction were taken into an NMR tube and diluted with 0.30 mL of CD_3CN.

Precipitation into a white/beige solid was often observed upon sole formation of the desired product (^{11}B NMR ≈ 31 ppm); while a dark to bright red precipitate was observed when alkylation of the heterocycle was exclusive (^{11}B NMR ≈ 22 ppm).

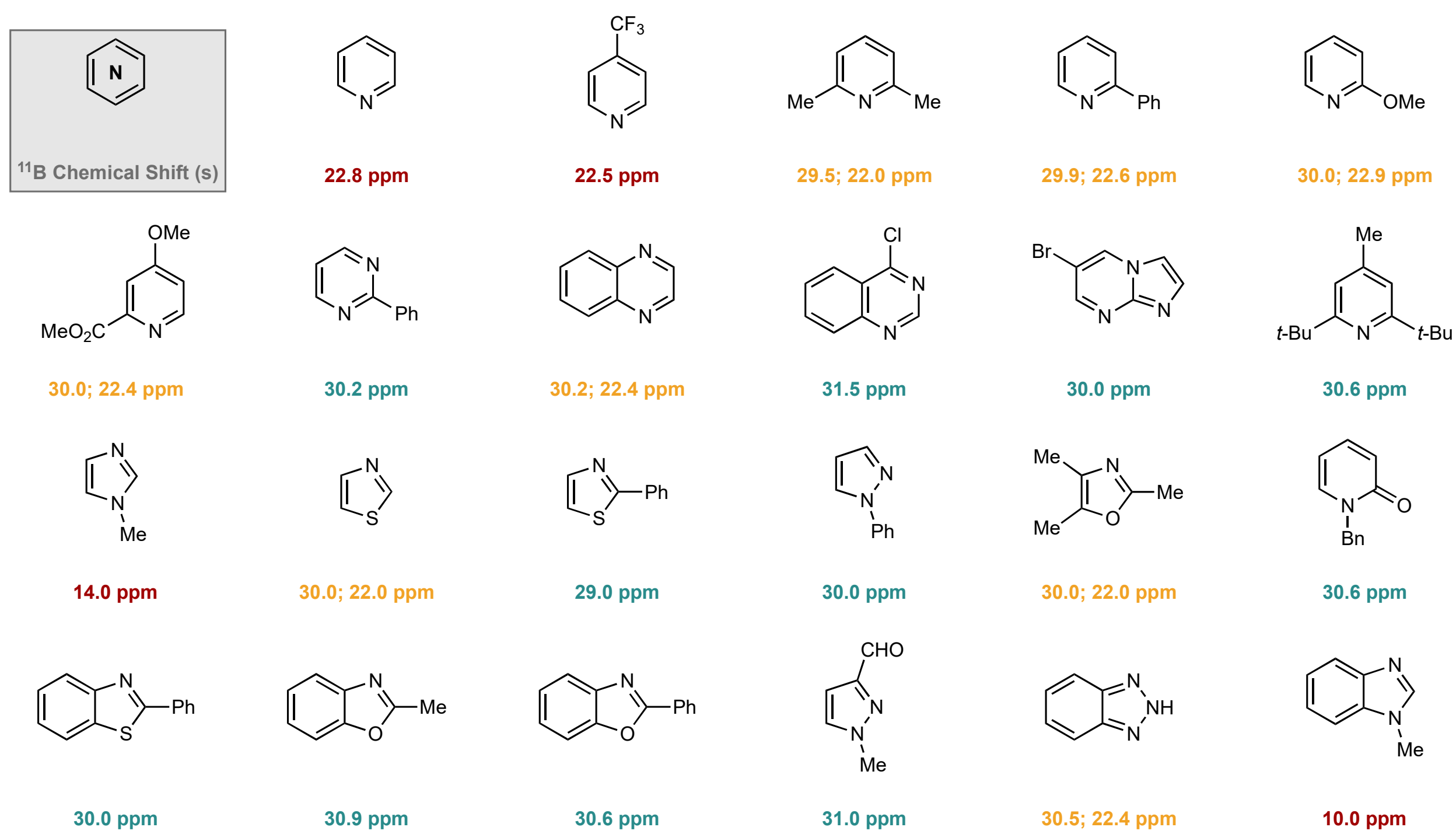

Figure S2 | ^{11}B NMR chemical shift observed for each heterocycle assessed.

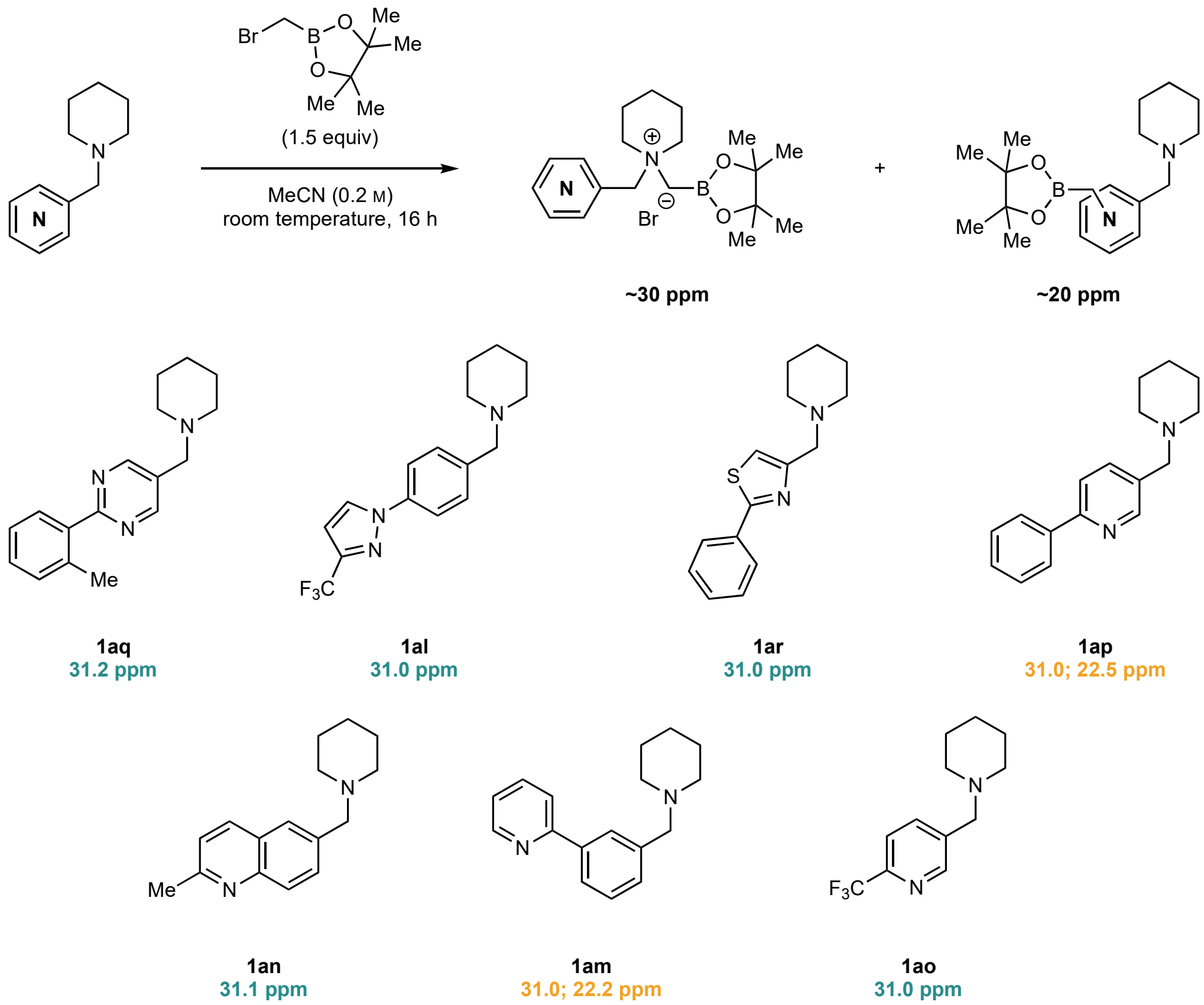

Figure S3 | ^{11}B NMR chemical shift observed for selected scope of heterobenzylic substrates.

2.3. Mechanistic Studies

2.3.1. Hydrolysis of the Betaine Trifluoroborate

The betaine trifluoroborate intermediate **4b** was subjected to the standard reaction conditions in the absence of palladium catalyst. After 16 hours, no hydrolysis of the trifluoroborate was observed by ^{1}H NMR, ^{11}B NMR or LCMS, while the starting trifluoroborate was recovered in >95% assay yield. Therefore, this result suggests that hydrolysis of the trifluoroborate occurs solely after oxidative addition of the palladium catalyst.

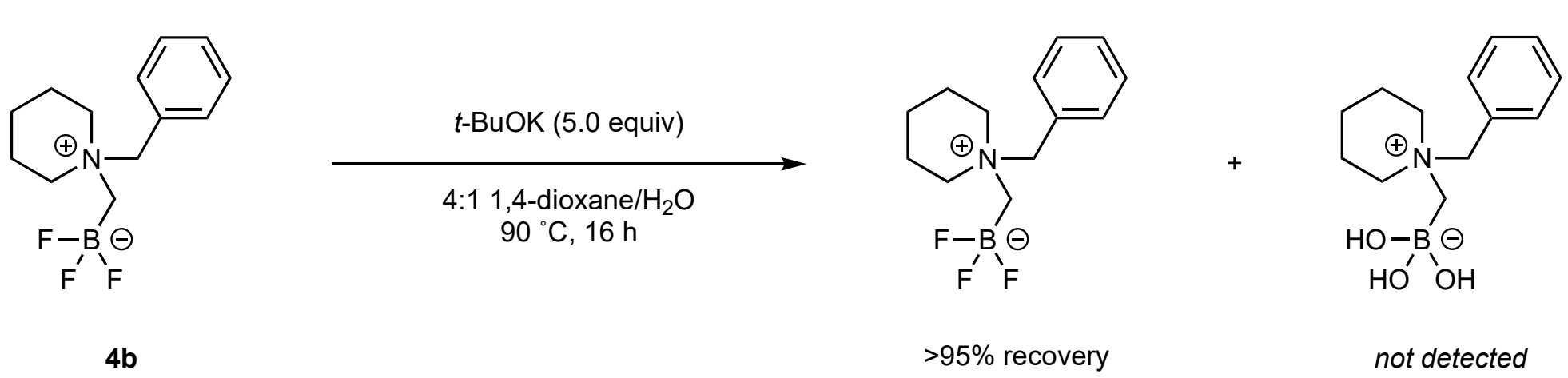

Figure S4 | Hydrolytic stability of the starting trifluoroborate in standard reaction conditions.

2.3.2. Oxidative Addition

Enantiopure *N,N*-dimethyl-1-phenethanamine **1x** was used in the two-step homologative process (**Figure S5a**). The (*R*)-enantiopure homologated amine was obtained with >99% ee (by comparison to an authentic sample prepared by methylation of commercially available (R)-(+)-β-methylphenethylamine). As stereochemical information from oxidative addition is known to be retained in the transmetalation and reductive elimination step, this result supports that oxidative addition into the benzylic ammonium betaine C−N bond occurs via a S_N2-type mechanism, resulting in inversion of configuration at the benzylic stereocenter. All-alkyl quaternary ammonium was subjected to homologation conditions (**Figure S5b**). The starting ammonium was left unconsumed independently of the condition used, supporting that benzylic activation is necessarily for oxidative addition.

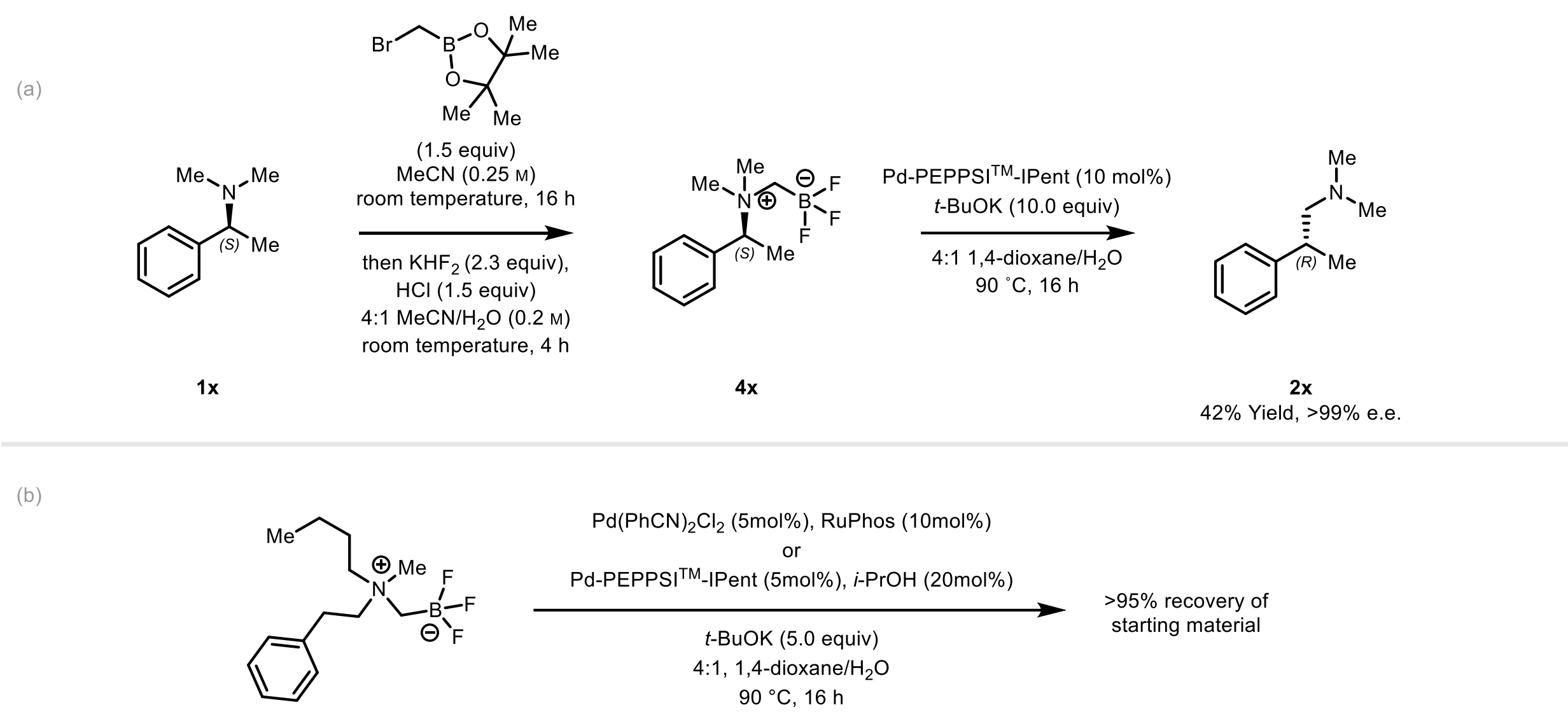

Figure S5 | (a) Homologation procedure on an enantiopure tertiary benzylic amine; (b) Homologation procedure attempt on an all-alkyl quaternary ammonium.

2.3.3. Crossover Experiment

The nature of the transmetalation process was then examined through performing a crossover experiment. When the morpholine- (**4aa**) and *N*-Boc piperazine-derived (**4c**) betaines, each containing distinct benzyl groups, were subjected to our optimised reaction conditions, a mixture of four products was observed (**Figure S6a**). This divergence in reactivity indicated the release of

the amine component from the palladium complex upon oxidative addition and, thus, an intermolecular transmetalation process. Similarly, when a betaine containing two electronically distinct benzyl groups, was exposed to our optimised reaction conditions, a mixture of four homologated amine products was observed (**Figure S6b**). This observation provides further evidence for the intermolecular nature of the transmetalation step. In addition, the lack of selectivity associated with this oxidative addition process of two-electronically distinct benzyl groups indicates oxidative addition is not the rate-limiting step in this catalytic cycle.

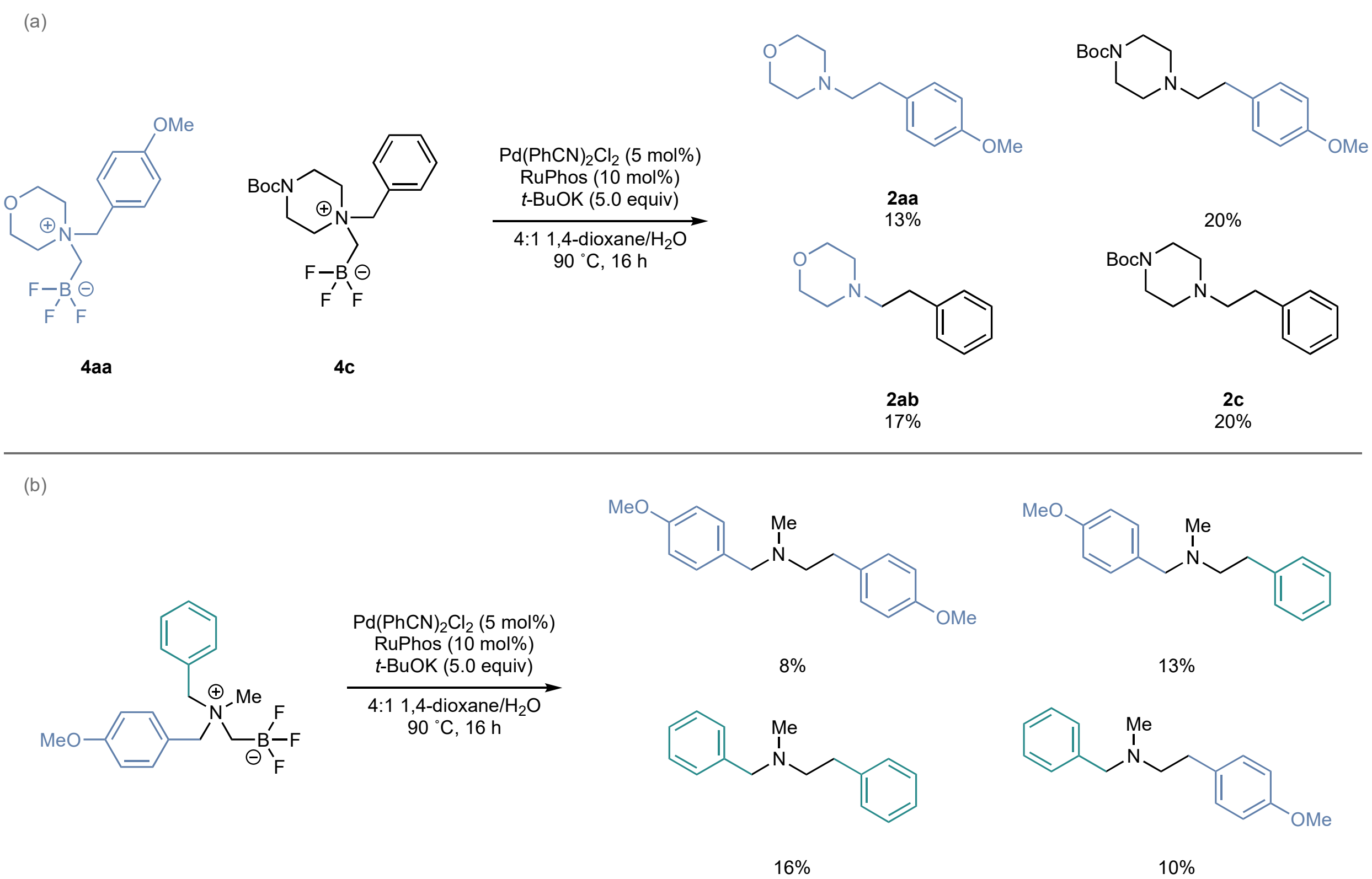

Figure S6 | (a) Crossover experiment; (b) Intramolecular competition experiment. Assay yields were generated via quantitative ^{1}H NMR spectroscopy using 1,1,2,2-tetrachloroethane as an internal standard.

Based on this evidence and previous mechanistic studies on the Suzuki coupling of benzylic ammonium salts, a plausible mechanism is depicted in Figure **S7**. The active Pd$^{(0)}$ catalyst, generated by oxidation of the phosphine ligand or dehydrogenation of isopropanol, undergoes S_N2-type oxidative addition with the starting betaine, generating an amine-bound, palladium species. Ligand exchange then occurs, thus forming a Pd-OH intermediate and an

aminomethyltrifluoroborate intermediate. The latter can then hydrolyse to the corresponding boronic acid, which then undergoes transmetalation to the Pd-OH complex, followed by reductive elimination to give the desired, homologated amine product.

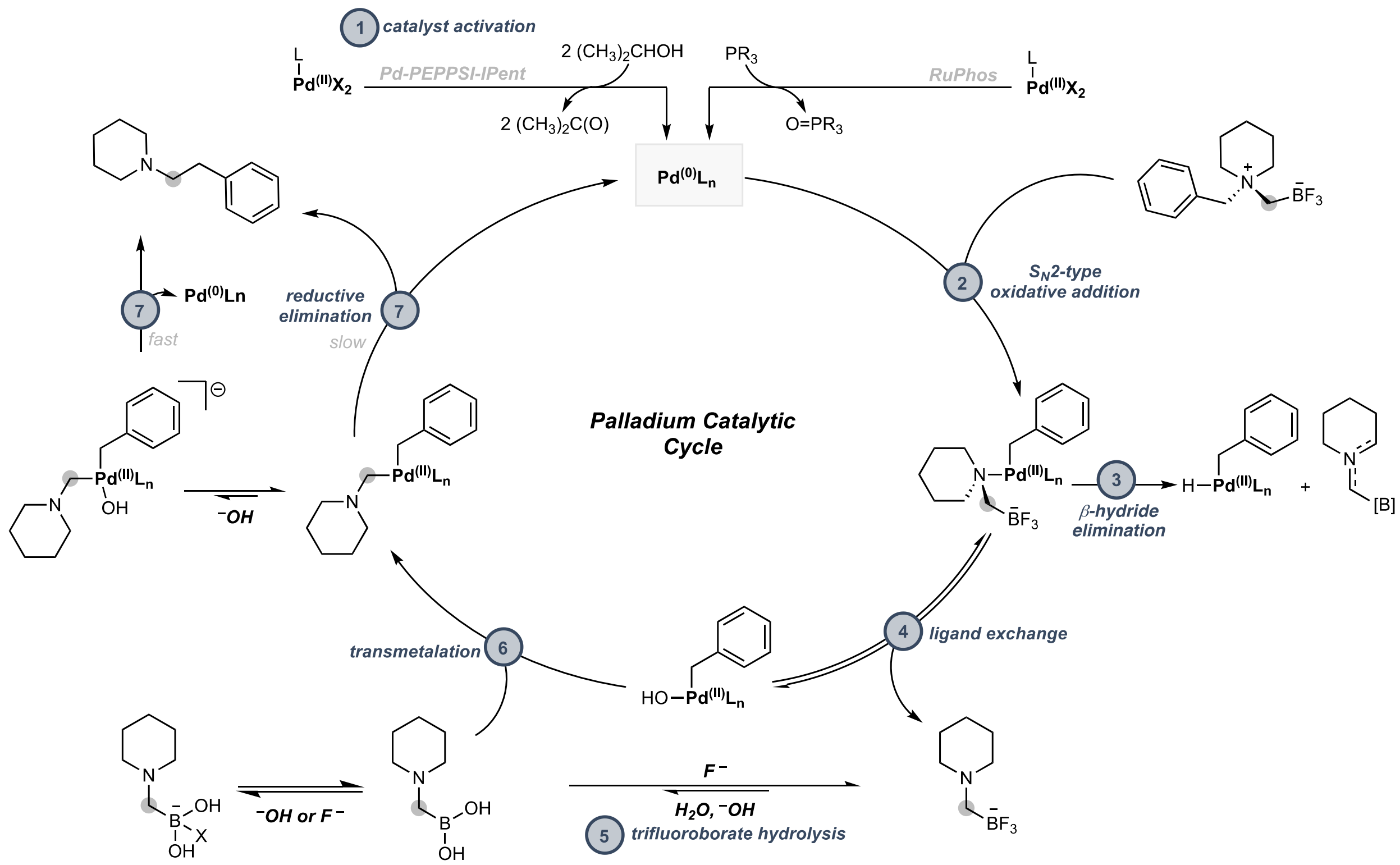

Figure S7 | Proposed mechanism for the palladium-catalysed homologation of benzylic tertiary amines and reaction map for side products formation.

2.4. Carbon–Nitrogen Bond Remodeling Optimisation

2.4.1. Bifunctional Linker Alkylation Optimisation Studies

Alkylation with a two-carbon linker

Experimental procedure: An oven-dried round-bottom flask (or microwave vial) was charged with a stirrer bar and two-carbon linker (0.30 mmol, 1.5 equiv). The flask was sealed and evacuated-refilled with nitrogen (3 cycles). 1-benzylpiperidine (35 mg, 0.20 mmol, 1.0 equiv) and anhydrous acetonitrile (0.80 mL) were then added. The reaction mixture was stirred at room temperature or 90 ˚C for 24 h. The solvent was removed *in vacuo* and conversion was measured using ^{1}H NMR spectroscopy using 1,1,2,2-tetrachloroethane (21 µL, 0.20 mmol, 1.0 equiv) as an internal standard.

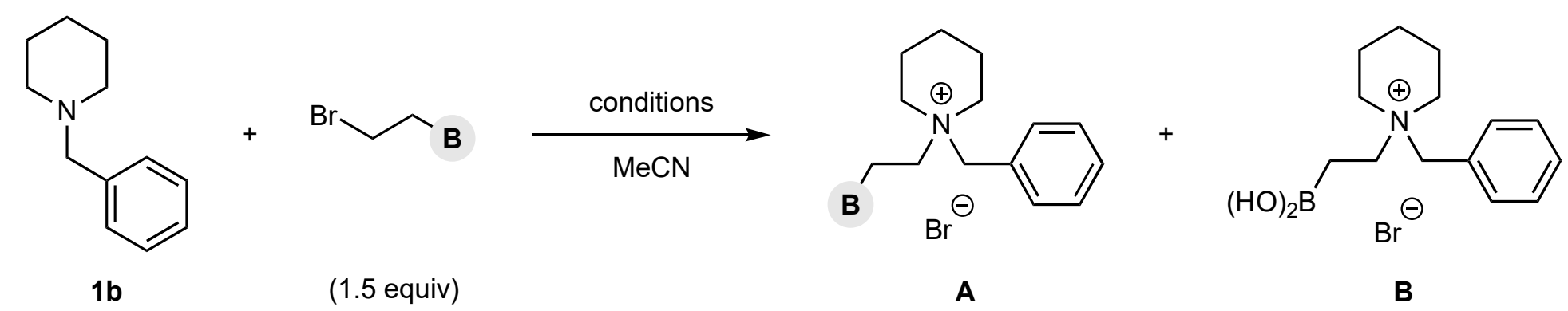

Entry	Boron Group	Temperature (˚C)	Yield A (%)	Yield B (%)
1	BPin	23	0[a]	0[a]
2	BPin	90	0[a]	0[a]
3	BPin	0 then 90	26[b]	56[b]
4	BF_3[c]	0 then 90	0[a]	0[a]
5	BMIDA	90	50[d]	0[d]

Table S8 | Optimisation attemps for the alkylation using 2-carbon reagents – assay yields were generated via quantitative ^{1}H NMR spectroscopy using 1,1,2,2-tetrachloroethane as an internal standard. [a]Only degradation of starting material was observed. [b]The formed compounds appeared to be very sensitive to moisture and only degradation was observed upon transfer or characterization. [c]Formed by addition of aqueous KHF_2 (3 M) after alkylation. [d] The product was formed with an equal amount of vinyl BMIDA. All purification attempts led to decomposition of the product to 1-benzylpiperidine and vinyl BMIDA.

Alkylation with a three-carbon linker

Experimental procedure: An oven-dried microwave vial was charged with a stirrer bar, Piribedil (60 mg, 0.20 mmol, 1.0 equiv) and iodide salt (0.60 mmol, 3.0 equiv) (if necessary). The flask was sealed and evacuated-refilled with nitrogen (3 cycles). Anhydrous acetonitrile (0.20 mL) and the bifunctional reagent (0.30 mmol, 1.5 equiv) were then added. The reaction was then stirred between 90 ˚C and 130 ˚C for 24–72 hours. The solvent was removed *in vacuo* and conversion was measured using ^{1}H NMR spectroscopy using 1,1,2,2-tetrachloroethane (21 µL, 0.20 mmol, 1.0 equiv) as an internal standard.

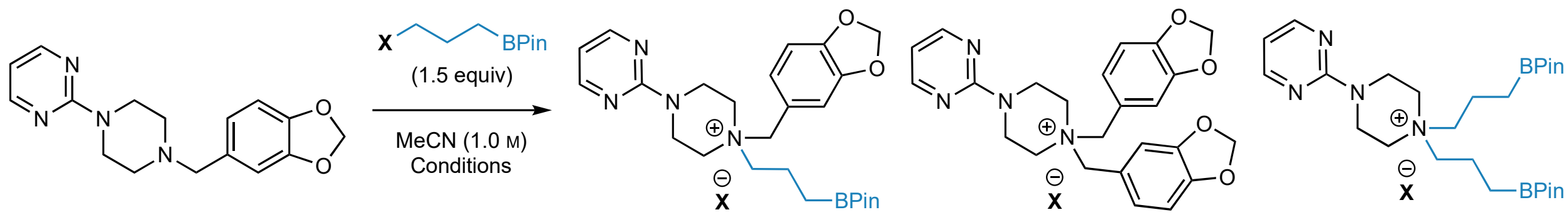

12a **B** **C**

Entry	X	Iodide Salt	Temperature (˚C)	Time (hours)	Yield 12a (%)	Yield B+C (%)
1	Br	none	100	24	9	29
2	Br	KI	100	24	26	74
3	Br	NaI	100	24	20	80
4	OTs	none	90	24	13	0
5	NMe_3	none	90	24	0	0
6	OTs	none	90	72	54	0
7	OTs	none	130	24	55	18
8	OMs	none	90–110	72	33	0

Table S9 | Optimisation of the *N*-alkylation between Piribedil and three-carbon, boronate-ester-containing, bifunctional reagents – assay yields were generated via quantitative ^{1}H NMR spectroscopy using 1,1,2,2-tetrachloroethane as an internal standard.

2.4.2. Optimisation of Palladium-Catalysed Multiple-Carbon Homologation Reaction using Boronic Acid Pinacol Esters

Experimental procedure: An oven-dried 4 mL PTFE/Silicone-lined septa screw cap clear glass vial equipped with a stirrer bar was charged with ammonium salt (**12a**) (32 mg or 64 mg, 0.05 mmol or 0.10 mmol, 1.0 equiv), palladium catalyst (5 mol%) and base (3.0–10 equiv) before being sealed and evacuated-refilled with nitrogen (3 cycles). A degassed mixture of 1,4-dioxane and HPLC grade water (and isopropanol if necessary) (0.25 mL or 0.50 mL) was added. The reaction mixture was heated at 90 ˚C overnight (16 hours) with vigorous stirring (1000 rpm). Upon completion the reaction mixture was cooled, filtered through Celite© and concentrated *in vacuo*. Conversion was measured using ^{1}H NMR spectroscopy using 1,1,2,2-tetrachloroethane (5 μL or 11 μL, 0.05 mmol or 0.10 mmol, 1.0 equiv) as an internal standard.

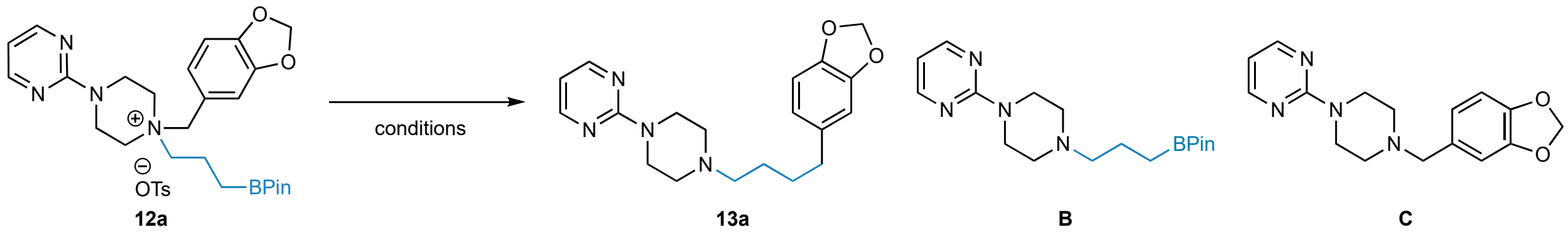

Entry	Catalyst	Isopropanol	Base	1,4-dioxane/H_2O ratio	Yield 13a (%)	Yield B (%)	Yield C (%)
1	Pd-PEPPSI™-IPent	20 mol%	*t*-BuOK (3.0 equiv)	4:1	trace	29	5
2	Pd-PEPPSI™-IPent	20 mol%	K_3PO_4 (3.0 equiv)	4:1	trace	51	17
3	RuPhos Pd G3	none	*t*-BuOK (3.0 equiv)	4:1	trace	47	13
4	XPhos Pd G3	none	*t*-BuOK (3.0 equiv)	4:1	trace	45	12
5	BrettPhos Pd G3	none	*t*-BuOK (3.0 equiv)	4:1	trace	86	8
6	DavePhos Pd G3	none	*t*-BuOK (3.0 equiv)	4:1	trace	24	6
7	SPhos Pd G3	none	*t*-BuOK (3.0 equiv)	4:1	31	41	11
8	CPhos Pd G3	none	*t*-BuOK (3.0 equiv)	4:1	28	46	13
9	SPhos Pd G3	none	K_3PO_4 (3.0 equiv)	4:1	42	41	11
10	SPhos Pd G3	none	K_3PO_4 (5.0 equiv)	4:1	39	28	9
11	SPhos Pd G3	none	K_3PO_4 (3.0 equiv)	9:1	52	47	13
12	SPhos Pd G3	none	K_3PO_4 (3.0 equiv)	3:2	23	15	9

Table S10 | Optimisation of the palladium-catalysed, three-carbon homologation of Piribedil – assay yields were generated via quantitative ^{1}H NMR spectroscopy using 1,1,2,2-tetrachloroethane as an internal standard. Yields marked as "trace" indicate observation of the product mass by LC-MS but not by ^{1}H NMR.

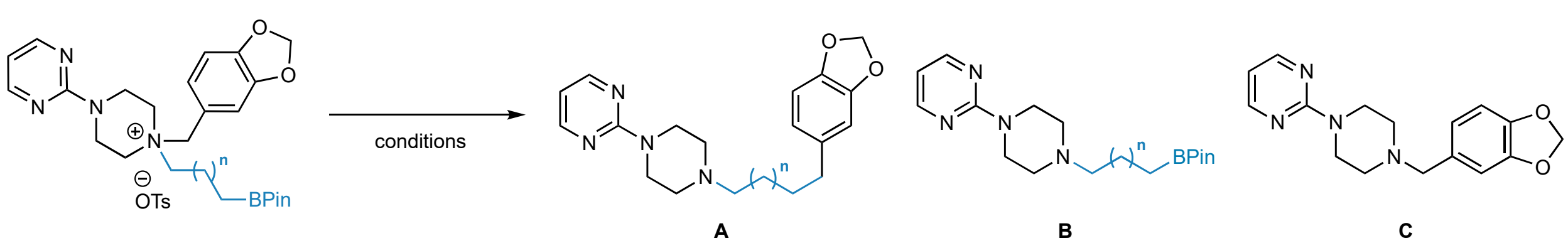

Entry	n	Catalyst	Base	1,4-dioxane/H_2O ratio	Yield A (%)	Yield B (%)	Yield C (%)
1	1	SPhos Pd G3	K_3PO_4 (3.0 equiv)	9:1	52	47	13
2	2	SPhos Pd G3	K_3PO_4 (3.0 equiv)	9:1	15	60	25
3	3	SPhos Pd G3	K_3PO_4 (3.0 equiv)	9:1	12	56	7

Table S11 | Extension of the optimised conditions for the palladium-catalysed, three-carbon homologation of Piribedil to the four- and five-carbon homologation – yields were generated via quantitaive ^{1}H NMR spectroscopy using 1,1,2,2-tetrachloroethane as an internal standard. Yields marked as "trace" indicate observation of the product mass by LC-MS but not by ^{1}H NMR.

2.4.3. Catalyst Screening for the Five-Carbon Linker Insertion

Experimental procedure: An oven-dried 4 mL PTFE/Silicone-lined septa screw cap clear glass vial equipped with a stirrer bar was charged with ammonium salt (86 mg, 0.10 mmol, 1.0 equiv), palladium catalyst (5 mol%) and potassium phosphate (3.0 equiv) before being sealed and evacuated-refilled with nitrogen (3 cycles). A degassed 9:1 mixture of 1,4-dioxane and HPLC grade water (0.50 mL) was added. The reaction mixture was heated at 90 ˚C overnight (16 hours) with vigorous stirring (1000 rpm). Upon completion the reaction mixture was cooled, filtered through Celite© and concentrated *in vacuo.* Conversion was measured using ^{1}H NMR spectroscopy using 1,1,2,2-tetrachloroethane (11 μL, 0.10 mmol, 1.0 equiv) as an internal standard.

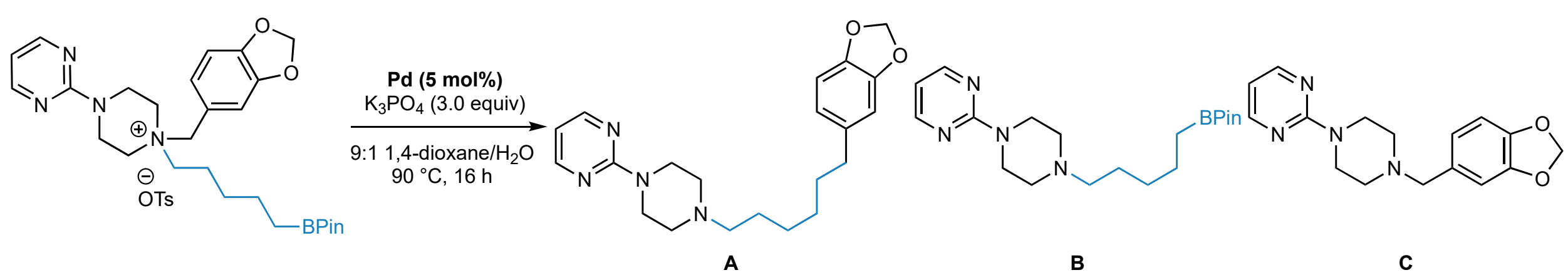

Entry	Ligand	Yield A (%)	Yield B (%)	Yield C (%)
1	SPhos[a]	12	56	7
2	PhCPhos[a]	15	70	15
3	JackiePhos[a]	11	42	13
4	L1[b,c]	9	26	13
5	L2[d,e]	10	54	6
6	BINAP[d]	16	66	11

Table S12 | Catalyst screening for cross coupling of the pentylboronic acid pinacol ester group – assay yields were generated via quantitative ^{1}H NMR spectroscopy using 1,1,2,2-tetrachloroethane as an internal standard. [a]Pd G3 complex was used. [b]Pd G4 complex was used. [c]Bis(3,5-bis(trifluoromethyl)phenyl)(2',6'-bis(dimethylamino)-3,6-dimethoxybiphenyl-2-yl)phosphine was used as L1. [d]$Pd(PhCN)_2Cl_2$ was used as a precatalyst. [e]Bis(3,5-bis(trifluoromethyl)phenyl)(2',6'-bis(isopropoxy)-3,6-dimethoxybiphenyl-2-yl)phosphine was used as L2.

Following these results, potential pathways for the formation of *N*-alkylated piperazine (B) and piribedil (C) were investigated. First, a control experiment was run, engaging ammonium salt in our standard reaction conditions and in the absence of a Pd-catalyst. No conversion to **B** or **C** of the starting ammonium was observed, thus ruling out a water-mediated S_N2-type process in these conditions. Careful analysis of the LC-MS traces collected during our Pd-catalyst screening revealed detectable quantities of 2-(piperazinyl)pyrimidine in the reaction mixture ([M+H$^+$] =

165.1). The previous control was thus repeated in the presence of 1.0 equiv. of 2-(piperazinyl)pyrimidine. In these conditions, 67% conversion of the starting ammonium salt was observed with the concomitant formation of **B** in 65% and **C** in 60%, supporting a potential S_N2 displacement if 2-(piperazinyl)pyrimidine is formed in the reaction mixture.

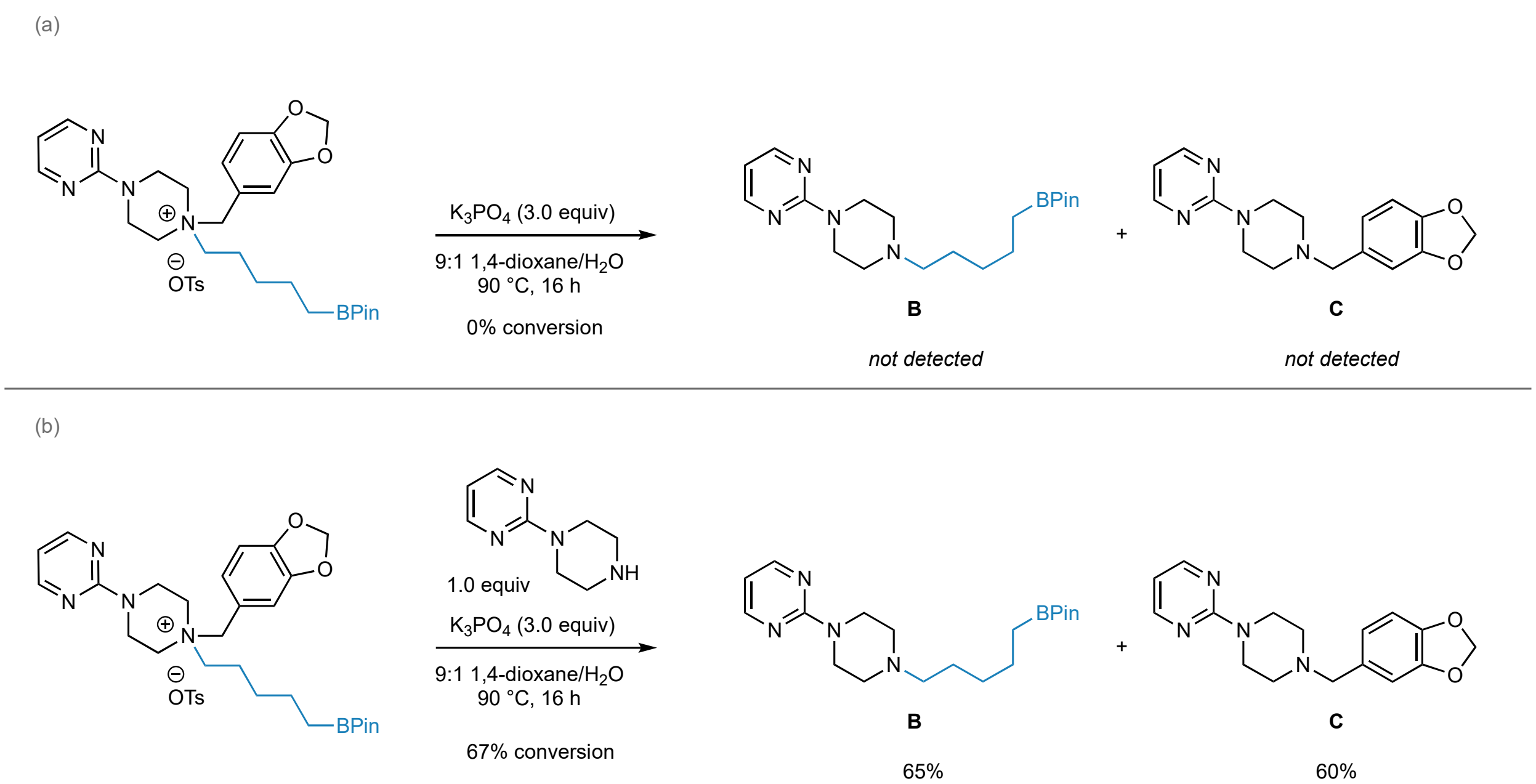

Figure S8 | Control experiment using benzyl ammonium salt – assay yields were generated via ^{1}H NMR spectroscopy using 1,1,2,2-tetrachloroethane as an internal standard.

Considering this, a plausible mechanism for the formation of *N*-alkylated piperazine **B** and piribedil **C** is proposed below. After oxidative addition into the benzylic C−N bond, the newly formed amine bound Pd-species can undergo β-hydride elimination if transmetallation is slow. This leads to the formation of a $Pd^{(II)}$−H species which can undergo reductive elimination to form 5-methyl-1,3-benzodioxole – observable by ^{1}H NMR, and an iminium, which undergoes basic hydrolysis under our standard reaction conditions, delivering *N*-(2-pyridinyl)piperazine. This newly formed secondary amine can undergo S_N2 with the starting ammonium salt to give *N*-alkylated piperazine **B** and piribedil **C**. Formation of piribedil **C** could also be explained by recombination of the formed piperazine with $Pd^{(II)}$-benzyl complex formed after oxidative addition.

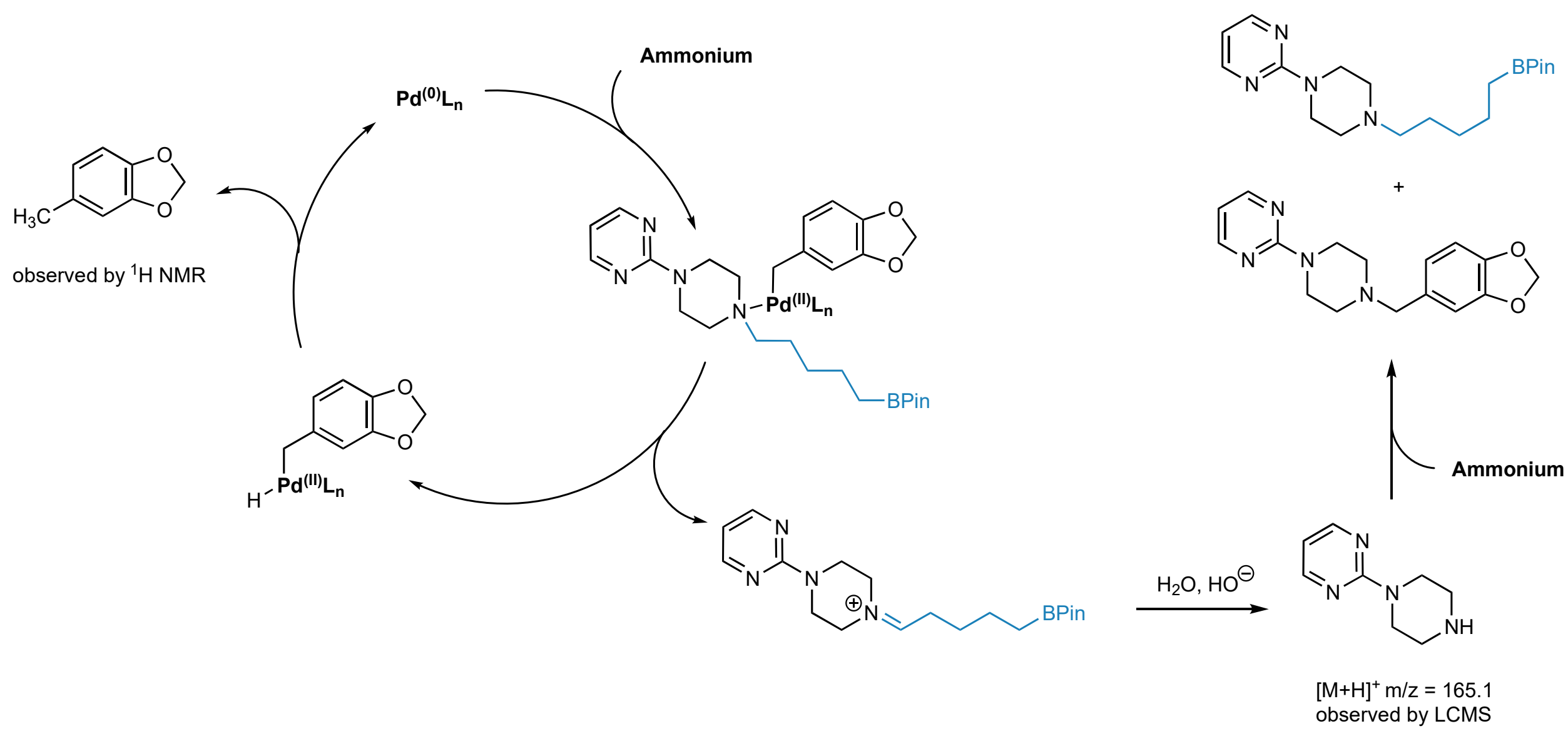

Figure S9 | Plausible mechanism for the formation of *N*-alkylated piperazine **B** and piribedil **C**.

2.4.4. Optimisation of the Csp^2-Csp^3 Cross-Coupling for Aryl Linker Insertion

Experimental procedure: An oven-dried 4 mL PTFE/Silicone-lined septa screw cap clear glass vial equipped with a stirrer bar was charged with ammonium salt (92 mg, 0.10 mmol, 1.0 equiv), palladium catalyst (5 mol%) and base (3.0 equiv) before being sealed and evacuated-refilled with nitrogen (3 cycles). A degassed 9:1 mixture of 1,4-dioxane and HPLC grade water was added (0.50 mL). The reaction mixture was heated at 90 ˚C overnight (16 hours) with vigorous stirring (1000 rpm). Upon completion the reaction mixture was cooled, filtered through Celite© and concentrated *in vacuo*. Yield was measured using ^{1}H NMR spectroscopy using 1,1,2,2-tetrachloroethane (11 µL, 0.10 mmol, 1.0 equiv) as an internal standard.

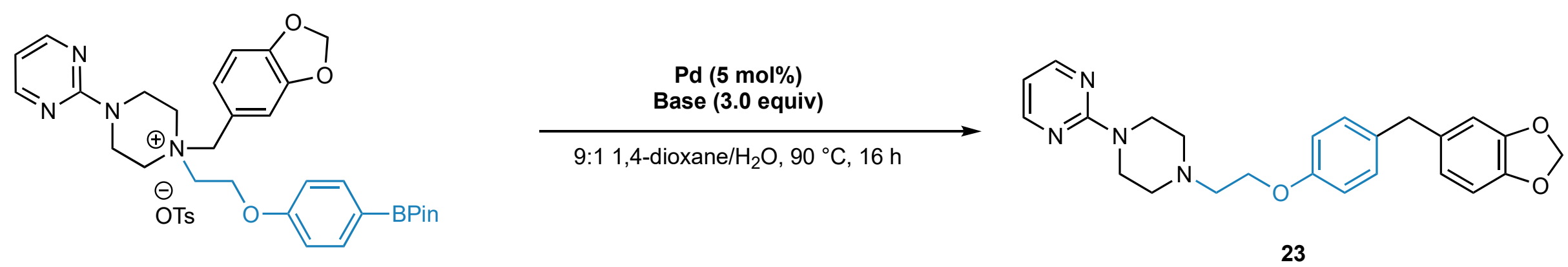

23

Entry	Catalyst	Base	Yield 23 (%)
1	BrettPhos Pd G3	*t*-BuOK	8
2	BrettPhos Pd G3	K_3PO_4	24
3	SPhos Pd G3	*t*-BuOK	47
4	SPhos Pd G3	K_3PO_4	50
5	RuPhos Pd G3	*t*-BuOK	58
6	RuPhos Pd G3	K_3PO_4	50

Table S13 | Optimisation of Csp^2-Csp^3 cross-coupling – assay yields were generated via quantitative ^1H NMR spectroscopy using 1,1,2,2-tetrachloroethane as an internal standard.

2.4.5. Optimisation of Alkylation-Hydroboration-Remodeling Sequence

Alkylation

Experimental procedure: An oven-dried microwave vial was charged with a stirrer bar and Piribedil (60 mg, 0.20 mmol, 1.0 equiv). The flask was sealed and evacuated-refilled with nitrogen (3 cycles). Anhydrous acetonitrile (0.20 mL) and the bifunctional reagent (0.30 mmol, 1.5 equiv) were then added. The reaction was then stirred at 110 ˚C for 72 hours. The solvent was removed *in vacuo* and conversion was measured using ^1H NMR spectroscopy using 1,3-dinitrobenzene (34 mg, 0.20 mmol, 1.0 equiv) as an internal standard.

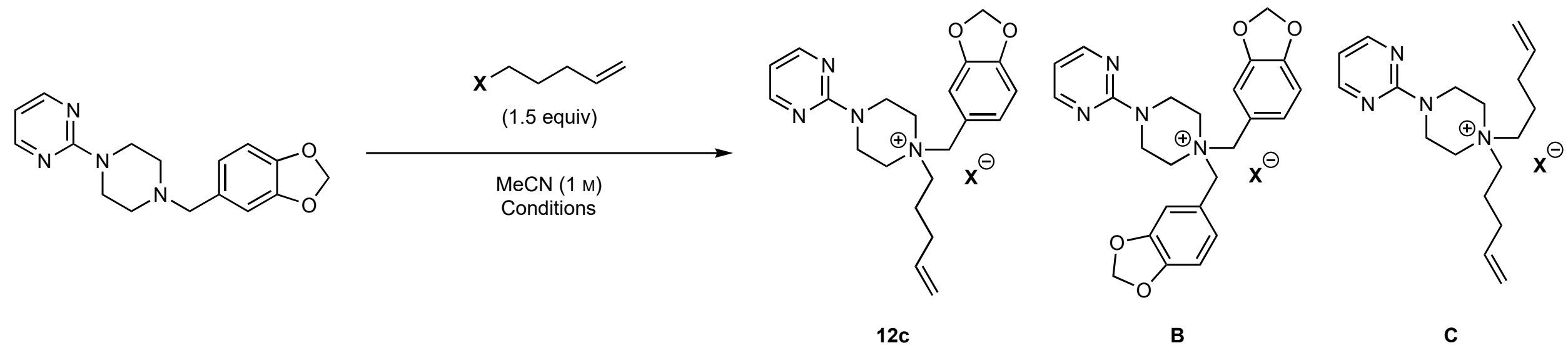

Entry	X	Yield 12c (%)	Yield B+C (%)
1	Br	trace	59
2	I	24	74
3	OTs	87	trace
4	ONs	53	trace
5	OTf	55	9

Table S14 | Optimisation of the *N*-alkylation between Piribedil and three-carbon, alkene-containing, bifunctional reagents – assay yields were generated via quantitative ^{1}H NMR spectroscopy using 1,3-dinitrobenzene as an internal standard. Yields marked as "trace" indicate observation of the product mass by LC-MS but not by ^{1}H NMR.

Hydroboration-Remodeling step

Experimental procedure using 9-borabicyclo[3.3.1]nonane (0.5 M in THF): An oven-dried microwave vial was charged with a stirrer bar and ammonium salt (0.20 mmol, 1.0 equiv). The flask was sealed and evacuated-refilled with nitrogen (3 cycles). 9-borabicyclo[3.3.1]nonane (0.5 M in THF, 0.40 or 0.80 mL, 0.20 mmol or 0.40 mmol, 1.0 equiv or 2.0 equiv) was then added. The reaction mixture was stirred at 60 ˚C overnight (16 hours) and allowed to cool to room temperature. A separate oven-dried microwave vial was charged with a stirrer bar, SPhos Pd G3 (4 mg, 0.005 mmol, 2.5 mol%) and potassium phosphate (52 mg, 0.30 mmol, 1.5 equiv). The flask was sealed and evacuated-refilled with nitrogen (3 cycles). Half of the hydroboration reaction mixture (0.40 mL, 0.10 mmol, 0.50 equiv) was added followed by a degassed 5:1 mixture of 1,4-dioxane and HPLC grade water (0.60 mL). The reaction mixture was heated at 90 ˚C overnight (16 hours) with vigorous stirring (1000 rpm). Upon completion the reaction mixture was cooled, filtered through Celite© and concentrated *in vacuo.* Conversion was measured using ^{1}H NMR spectroscopy using 1,1,2,2-tetrachloroethane (11 μL, 0.10 mmol, 1.0 equiv) as an internal standard.

Experimental procedure using 9-borabicyclo[3.3.1]nonane dimer: An oven-dried microwave vial was charged with a stirrer bar, ammonium salt (**12**) (0.20 mmol, 1.0 equiv), and 9-

borabicyclo[3.3.1]nonane dimer (49 mg or 98 mg, 0.20 mmol or 0.40 mmol, 1.0 equiv or 2.0 equiv). The flask was sealed and evacuated-refilled with nitrogen (3 cycles). Degassed 1,4-dioxane (0.80 mL) was then added. The reaction mixture was stirred at 60 ˚C overnight (16 hours) and allowed to cool to room temperature. A separate oven-dried microwave vial was charged with a stirrer bar, SPhos Pd G3 (4 mg, 0.005 mmol, 2.5 mol%) and potassium phosphate (52 mg, 0.30 mmol, 1.5 equiv). The flask was sealed and evacuated-refilled with nitrogen (3 cycles). Half of the hydroboration reaction mixture (0.40 mL, 0.10 mmol, 0.50 equiv) was added followed by a degassed 1:1 mixture of 1,4-dioxane and HPLC grade water (0.10 mL). The reaction mixture was heated at 90 ˚C overnight (16 hours) with vigorous stirring (1000 rpm). Upon completion the reaction mixture was cooled, filtered through Celite© and concentrated *in vacuo*. Conversion was measured using ^{1}H NMR spectroscopy using 1,1,2,2-tetrachloroethane (11 µL, 0.10 mmol, 1.0 equiv) as an internal standard.

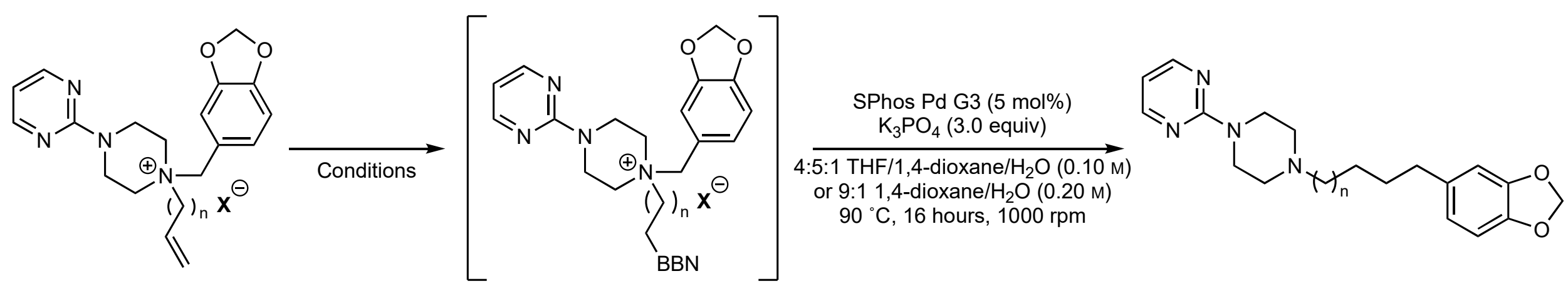

12b or 12c **14b or 14c** *Not Isolated* **13a, 13b or 13c**

Entry	n	9-BBN Source	9-BBN Reagent equiv.	Hydroboration Reaction Time (hours)	Hydroboration Reaction Temperature (˚C)	Yield 13 (%)
1	3	0.5 M IN THF	1.0	1	60	11
2	3	0.5 M IN THF	2.0	16	60	75
3	2	0.5 M IN THF	2.0	16	60	56
4	2	Dimer	1.0	16	60	64
5	1	0.5 M IN THF	2.0	16	60	19
6	1	Dimer	1.0	16	60	13
7	1	Dimer	2.0	16	60	32

Table S15 | Optimisation of the hydroboration-Remodeling of Piribedil – assay yields were generated via quantitative ^{1}H NMR spectroscopy using 1,1,2,2-tetrachloroethane as an internal standard.

2.5. General Procedures

2.5.1. General Procedure for Tertiary Benzylamine Synthesis

2.5.1.1. Synthesis via reductive amination: An oven-dried round-bottom flask was charged with a stirrer bar and any solid reactants/reagents. The flask was sealed and evacuated-refilled with nitrogen (3 cycles). Liquid reactants/reagents and anhydrous solvent were added. The reaction mixture was stirred for 30–120 minutes at room temperature and then sodium triacetoxyborohydride was added. The reaction mixture was stirred overnight, quenched with saturated, aqueous sodium bicarbonate or aqueous sodium hydroxide (1.0 M), extracted thrice with organic solvent, and washed once with brine. The combined organic layers were dried over anhydrous sodium sulfate, filtered and concentrated *in vacuo*. The crude mixtures were purified by automated flash column chromatography if necessary.

2.5.1.2. Synthesis via nucleophilic substitution: An oven-dried round-bottom flask was charged with a stirrer bar and any solid reactants/reagents. The flask was sealed and evacuated-refilled with nitrogen (3 cycles). Liquid reactants/reagents and anhydrous solvent were added. The reaction mixture was stirred at room temperature overnight. The crude reaction mixture was washed thrice with water and the organic layer was dried over anhydrous sodium sulfate, filtered and concentrated *in vacuo*. The crude mixtures were purified by automated flash column chromatography if necessary.

2.5.2. General Procedure for Tertiary Benzylamine Alkylation

2.5.2.1. Alkylation with (bromomethyl)boronic acid pinacol ester: An oven-dried round-bottom flask (or microwave vial) was charged with a stirrer bar and amine (1.0 equiv) (if solid). *In the case of amine hydrochloride salts K_2CO_3 (1.0 equiv) added*. The flask was sealed and evacuated-refilled with nitrogen (3 cycles). Anhydrous acetonitrile (0.25–0.50 M), amine (1.0 equiv) (if liquid), and then (bromomethyl)boronic acid pinacol ester (1.1–1.5 equiv) were then added. The reaction mixture was stirred at room temperature until full consumption of amine was observed via TLC (1–72 hours). Aqueous potassium hydrogenfluoride (3.0 M, 2.3–3.0 equiv) and hydrogen chloride (4.0 M in 1,4-dioxane, 1.0–2.0 equiv) were then added. The reaction

mixture was then diluted with anhydrous acetonitrile and HPLC grade water to give final concentration 4:1 MeCN/H_2O (0.10–0.20 M). The reaction mixture was stirred at room temperature until complete conversion was observed via TLC or LC-MS (4–24 hours). The reaction mixture was concentrated *in vacuo* and the crude material was purified via one of two methods:

Method I: The crude material was concentrated onto Celite© and purified by automated reverse phase flash column chromatography (100% water for 2–3 minutes, 0–100% acetonitrile in water for 10–20 minutes, 100% acetonitrile for 2–5 minutes).

Method II: The crude material was suspended in water (approximately 20 mL) and extracted with dichloromethane (3 x 15 mL). The combined organic phases were dried over anhydrous magnesium sulphate and concentrated *in vacuo*. The resultant solid was triturated with diethyl ether (3 x 10 mL) and then concentrated *in vacuo*.

2.5.2.2. Alkylation with potassium (bromomethyl)trifluoroborate: An oven-dried round-bottom flask (or microwave vial) was charged with a stirrer bar and amine (1.0 equiv) (if solid), and potassium (bromomethyl)trifluoroborate (1.1–1.5 equiv). The flask was sealed and evacuated-refilled with nitrogen (3 cycles). Anhydrous acetonitrile (1.0–2.0 M) and amine (1.0 equiv) (if liquid) were then added. The reaction mixture was then stirred at 80–110 ˚C until complete consumption of the amine was observed via TLC (4–48 hours). The reaction mixture was then cooled and purified by one of two methods:

Method I: The crude material was concentrated onto Celite© and purified by automated reverse phase flash column chromatography (100% water for 2–3 minutes, 0–100% acetonitrile in water for 10–20 minutes, 100% acetonitrile for 2–5 minutes).

Method II: The crude material was suspended in water (approximately 20 mL), acidified with hydrogen chloride (4.0 M in 1,4-dioxane, 1.0 equiv), and extracted with dichloromethane (3 x 15 mL). The combined organic phases were dried over anhydrous magnesium sulphate and concentrated *in vacuo*. The resultant solid was triturated with diethyl ether (3 x 10 mL) and then concentrated *in vacuo*.

2.5.3. General Procedure for Betaine Coupling:

2.5.3.1. Coupling using $Pd(PhCN)_2Cl_2$ and RuPhos: An oven-dried 4 mL PTFE/Silicone-lined septa screw cap clear glass vial equipped with a stirrer bar was charged with betaine (1.0 equiv), bis(benzonitrile)palladium(II) chloride (5 mol%), RuPhos (10 mol%), and potassium *tert*-butoxide (5.0 equiv) before being sealed and evacuated-refilled with nitrogen (3 cycles). A degassed 4:1 mixture of 1,4-dioxane and HPLC grade water was added (0.10–0.20 M). The reaction mixture was heated at 90 ˚C overnight (16 hours) with vigorous stirring (1000 rpm). Upon completion the reaction mixture was cooled, filtered through Celite© and concentrated *in vacuo.* The crude material was then purified by flash column chromatography.

2.5.3.2. Coupling using Pd-PEPPSI™-IPent: An oven-dried 4 mL PTFE/Silicone-lined septa screw cap clear glass vial equipped with a stirrer bar was charged with betaine (1.0 equiv), Pd-PEPPSI™-IPent (10 mol%), and potassium *tert*-butoxide (10 equiv) before being sealed and evacuated-refilled with nitrogen (3 cycles). A degassed 4:1 mixture of 1,4-dioxane and HPLC grade water was added (0.10–0.20 M). The reaction mixture was heated at 90 ˚C overnight (16 hours) with vigorous stirring (1000 rpm). Upon completion the reaction mixture was cooled, filtered through Celite© and concentrated *in vacuo.* The crude material was then purified by flash column chromatography.

2.5.3.3. Coupling using Pd-PEPPSI™-IPent with hydrolysable substrates: An oven-dried 4 mL PTFE/Silicone-lined septa screw cap clear glass vial equipped with a stirrer bar was charged with betaine (1.0 equiv), Pd-PEPPSI™-IPent (10 mol%), and potassium phosphate tribasic (10 equiv) before being sealed and evacuated-refilled with N_2 (3 cycles). A degassed 4:1 mixture of 1,4-dioxane and HPLC grade water was added (0.1–0.2 M). The reaction mixture was heated at 90 ˚C overnight (16 hours) with vigorous stirring (1000 rpm). Upon completion the reaction mixture was cooled, filtered through Celite© and concentrated *in vacuo.* The crude material was then purified by flash column chromatography.

2.5.4. General Procedure for Synthesis of Boronate-Ester-Substituted, Bifunctional Reagents

An oven-dried microwave vial was charged with a stirrer bar and silver *p*-toluenesulfonate (2.0 equiv). The flask was sealed and evacuated-refilled with nitrogen (3 cycles). The boronate-ester-substituted bromide (1.0 equiv) and anhydrous acetonitrile (0.33 M) were then added. The reaction mixture was then stirred at 90 ˚C overnight (16 hours). Upon completion, the reaction mixture was cooled and the solvent was removed *in vacuo*. The crude material was concentrated onto Celite© and purified by automated flash column chromatography (100% petroleum ether for 1 minute, 0–20% ethyl acetate in petroleum ether for 4 minutes, 20–50% ethyl acetate in petroleum ether for 4 minutes, 50–100% ethyl acetate in petroleum ether for 1 minute).

2.5.5. General Procedure for Tertiary Benzylamine Alkylation with Bifunctional Reagents

2.5.5.1. Alkylation with bifunctional reagents containing a boronate ester substituent: An oven-dried microwave vial was charged with a stirrer bar and Piribedil (1.0 equiv). The flask was sealed and evacuated-refilled with nitrogen (3 cycles). Anhydrous acetonitrile (1.0 M) and the bifunctional linker (1.5 equiv) were then added. The reaction mixture was then stirred at 110 ˚C for 72 hours. The reaction mixture was cooled and the crude material was concentrated onto Celite© and purified by automated reverse phase flash column chromatography (100% water for 1–2 minutes, 0–100% acetonitrile in water for 10–20 minutes, 100% acetonitrile for 2–5 minutes).

For compounds where hydrolysis was observed upon purification, the fractions containing the desired product as well as the boronic acid byproduct were then concentrated *in vacuo*. An oven-dried microwave vial was then charged with a stirrer bar, the mixture of boronate-ester- and boronic-acid-substituted salts, and pinacol (1.5 equiv). The flask was sealed and evacuated-refilled with nitrogen (3 cycles). Anhydrous acetonitrile (1.0 M) was then added. The reaction was then stirred at 40 ˚C for 24 hours. The crude material was concentrated *in vacuo* and the product:pinacol ratio was quantified using ^{1}H NMR spectroscopy.

2.5.5.2. Alkylation with bifunctional reagents containing an alkene substituent: An oven-dried microwave vial was charged with a stirrer bar and Piribedil (1.0 equiv). The flask was sealed and

evacuated-refilled with nitrogen (3 cycles). Anhydrous acetonitrile (1.0 M) and the bifunctional linker (1.5 equiv) were then added. The reaction mixture was then stirred at 110 ˚C for 72 hours. The reaction mixture was cooled and the crude material was concentrated onto Celite© and purified by automated reverse phase flash column chromatography (100% water for 1–2 minutes, 0–100% acetonitrile in water for 10–20 minutes, 100% acetonitrile for 2–5 minutes).

2.5.6. General Procedure for Palladium-Catalysed Remodeling of Tertiary Benzylamines

2.5.6.1. Three-carbon homologation: An oven-dried 4 mL PTFE/Silicone-lined septa screw cap clear glass vial equipped with a stirrer bar was charged with ammonium salt (64 mg, 0.10 mmol, 1.0 equiv), SPhos Pd G3 (4 mg, 0.005 mmol, 5 mol%) and potassium phosphate tribasic (64 mg, 0.30 mmol, 3 equiv) before being sealed and evacuated-refilled with nitrogen (3 cycles). A degassed 9:1 mixture of 1,4-dioxane and HPLC grade water (0.50 mL) was added. The reaction mixture was heated at 90 ˚C overnight (16 hours) with vigorous stirring (1000 rpm). Upon completion the reaction mixture was cooled, filtered through Celite© and concentrated *in vacuo.* Conversion was measured using ^{1}H NMR spectroscopy using 1,1,2,2-tetrachloroethane (11 μL, 0.10 mmol, 1.0 equiv) as an internal standard. The crude material was then purified by flash column chromatography (0–10% ethyl acetate with 5% triethylamine in petroleum ether for 10 minutes, 10-50% ethyl acetate with 5% triethylamine in petroleum ether for 10 minutes, 50-100% ethyl acetate with 5% triethylamine in petroleum ether for 5 minutes, and 100% ethyl acetate with 5% triethylamine for 5 minutes).

2.5.6.2. Csp^{2}-Csp^{3} coupling: An oven-dried 4 mL PTFE/Silicone-lined septa screw cap clear glass vial equipped with a stirrer bar was charged with ammonium salt 1.0 equiv), RuPhos Pd G3 (5 mol%) and base (3.0 equiv) before being sealed and evacuated-refilled with nitrogen (3 cycles). A degassed 9:1 mixture of 1,4-dioxane and HPLC grade water (0.50 mL) was added. The reaction mixture was heated at 90 ˚C overnight (16 hours) with vigorous stirring (1000 rpm). Upon completion the reaction mixture was cooled, filtered through Celite© and concentrated *in vacuo.* Conversion was measured using ^{1}H NMR spectroscopy using 1,1,2,2-tetrachloroethane (1.0 equiv) as an internal standard. The crude material was then purified by flash column chromatography.

2.5.6.3. Hydroboration-Remodeling with 9-borabicyclo[3.3.1]nonane (0.5 M in THF): An oven-dried microwave vial was charged with a stirrer bar and ammonium salt (0.2 mmol, 1.0 equiv). The flask was sealed and evacuated-refilled with nitrogen (3 cycles). 9-borabicyclo[3.3.1]nonane (0.5 M in THF, 0.80 mL, 0.40 mmol, 2.0 equiv) was then added. The reaction mixture was stirred at 60 ˚C overnight (16 hours) and allowed to cool to room temperature. A separate oven-dried microwave vial was charged with a stirrer bar, SPhos Pd G3 (4 mg, 0.005 mmol, 2.5 mol%) and potassium phosphate tribasic (52 mg, 0.30 mmol, 1.5 equiv). The flask was sealed and evacuated-refilled with nitrogen (3 cycles). Half of the hydroboration reaction mixture (0.40 mL, 0.10 mmol, 0.50 equiv) was added followed by a degassed 5:1 mixture of 1,4-dioxane and HPLC grade water (0.60 mL). The reaction mixture was heated at 90 ˚C overnight (16 hours) with vigorous stirring (1000 rpm). Upon completion the reaction mixture was cooled, filtered through Celite© and concentrated *in vacuo*. Conversion was measured using ^{1}H NMR spectroscopy using 1,1,2,2-tetrachloroethane (11 μL 0.10 mmol, 0.50 equiv) as an internal standard. The crude material was then purified by flash column chromatography.

2.5.6.4. Hydroboration-Remodeling with 9-borabicyclo[3.3.1]nonane dimer: An oven-dried microwave vial was charged with a stirrer bar, ammonium salt (0.10 mmol or 0.20 mmol, 1.0 equiv), and 9-borabicyclo[3.3.1]nonane dimer (24 mg or 49 mg, 0.10 mmol or 0.20 mmol, 1.0 equiv). The flask was sealed and evacuated-refilled with nitrogen (3 cycles). Degassed 1,4-dioxane (0.40 mL or 0.80 mL) was then added. The reaction mixture was stirred at 60 ˚C overnight (16 hours) and allowed to cool to room temperature. A separate oven-dried microwave vial was charged with a stirrer bar, SPhos Pd G3 (2 mg or 4 mg, 0.0025 mmol or 0.005 mmol, 2.5 mol%) and potassium phosphate tribasic (32 mg or 52 mg, 0.15 mmol or 0.30 mmol, 1.5 equiv). The flask was sealed and evacuated-refilled with nitrogen (3 cycles). Half of the hydroboration reaction mixture (0.20 mL or 0.40 mL, 0.05 mmol or 0.10 mmol, 0.5 equiv) was added followed by a degassed 1:1 mixture of 1,4-dioxane and HPLC grade water(0.05 mL or 0.10 mL). The reaction mixture was heated at 90 ˚C overnight (16 hours) with vigorous stirring (1000 rpm). Upon completion the reaction mixture was cooled, filtered through Celite© and concentrated *in vacuo*. Conversion was measured using ^{1}H NMR spectroscopy using 1,1,2,2-tetrachloroethane (5 μL or 11 μL, 0.05 mmol or 0.10 mmol, 0.50 equiv) as an internal standard. The crude material was then purified by flash column chromatography.

N-benzylmorpholine (**1a**)

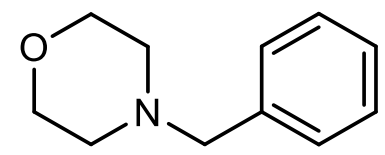

Prepared by general procedure **2.5.1.1.** morpholine (1.7 mL, 20 mmol, 1.3 equiv), benzaldehyde (1.5 mL, 15 mmol, 1.0 equiv), and sodium triacetoxyborohydride (6.4 g, 30 mmol, 3.0 equiv) in dichloromethane. The title compound was obtained as a yellow liquid (2.5 g, 14 mmol, 95% yield) and used in the next step without further purification. Data was in line with previous characterisation.[51] **^{1}H NMR** (400 MHz, $CDCl_3$) δ 7.38 – 7.26 (m, 5H), 3.76 – 3.68 (m, 4H), 3.51 (s, 2H), 2.45 (t, *J* = 4.6 Hz, 4H); **^{13}C NMR** (101 MHz, $CDCl_3$) δ 137.8, 129.3, 128.4, 127.3, 67.1, 63.6, 53.7

tert-butyl 4-benzylpiperazine-1-carboxylate (**1c**)

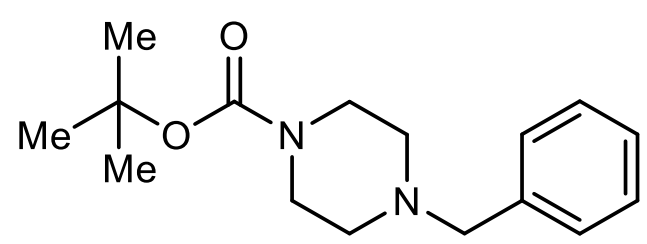

Prepared by general procedure **2.5.1.1.** using *N*-Boc-piperazine (3.8 g, 20 mmol, 1.3 equiv), benzaldehyde (1.5 mL, 15 mmol, 1.0 equiv), and sodium triacetoxyborohydride (6.4 g, 30 mmol, 3.0 equiv) in dichloromethane. The title compound was obtained as a white solid (4.1 g, 14 mmol, 96% yield) and used in the next step without further purification. Data was in line with previous characterisation.[52] **^{1}H NMR** (400 MHz, $CDCl_3$) δ 7.31 (d, *J* = 4.3 Hz, 4H), 7.28 – 7.24 (m, 1H), 3.51 (s, 2H), 3.42 (t, *J* = 5.0 Hz, 4H), 2.38 (t, *J* = 5.0 Hz, 4H), 1.45 (s, 9H); **^{13}C NMR** (101 MHz, $CDCl_3$) δ 154.9, 138.0, 129.3, 128.4, 127.3, 79.7, 63.2, 53.0, 28.6.

1-benzyl-4-tosylpiperazine (**1d**)

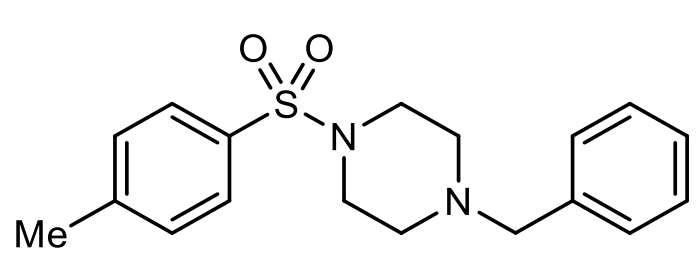

Prepared by general procedure **2.5.1.1.** using 1-tosylpiperazine (480 mg, 2.0 mmol, 1.0 equiv), benzaldehyde (0.24 mL, 2.4 mmol, 1.2 equiv), and sodium triacetoxyborohydride (843 mg, 4.0

mmol, 2.0 equiv) in tetrahydrofuran. Purification by automated flash column chromatography (10–50% acetone in petroleum ether) gave the title compound as a white solid (297 mg, 0.90 mmol, 44% yield). Data was in line with previous characterisation.[53] **^{1}H NMR** (500 MHz, $CDCl_3$) δ 7.65 – 7.59 (m, 1H), 7.34 – 7.20 (m, 4H), 3.48 (s, 1H), 3.01 (d, *J* = 5.8 Hz, 2H), 2.52 (t, *J* = 5.0 Hz, 2H), 2.43 (s, 2H); **^{13}C NMR** (126 MHz, $CDCl_3$) δ 143.7, 137.6, 132.6, 129.8, 129.2, 128.4, 128.0, 127.4, 62.7, 52.2, 46.2, 21.6.

1-benzyl-3,5-dimethylpiperidine (**1e**)

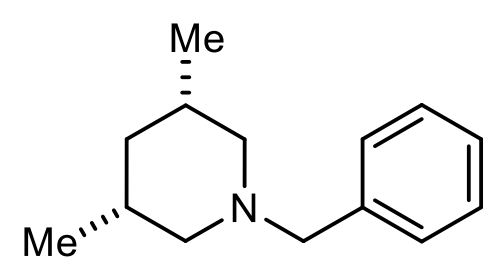

Prepared by general procedure **2.5.1.1.** using benzaldehyde (1.3 g, 12 mmol, 1.2 equiv), 3,5-dimethylpiperidine (1.1 g, 10 mmol, 1.0 equiv), and sodium triacetoxyborohydride (4.2 g, 20 mmol, 2.0 equiv) in tetrahydrofuran (40 mL). Purification by automated flash column chromatography (0–30% ethyl acetate in petroleum ether for 10 minutes, 30–50% ethyl acetate in petroleum ether for 2 minutes, 50–100% ethyl acetate in petroleum ether for 2 minutes, and 100% ethyl acetate for 2 minutes) gave the title compound as a colourless oil (808 mg, 4.0 mmol, 40% yield). **^{1}H NMR** (700 MHz, CDCl3) δ 7.37 – 7.31 (m, 4H), 7.27 (pt, *J* = 6.3, 2.8 Hz, 1H), 3.51 (s, 2H), 2.84 (ddd, *J* = 10.6, 3.7, 1.7 Hz, 2H), 1.78 – 1.68 (m, 3H), 1.48 (t, *J* = 10.8 Hz, 2H), 0.85 (d, *J* = 6.4 Hz, 6H), 0.58 – 0.50 (m, 1H); **^{13}C NMR** (176 MHz, $CDCl_3$) δ 138.8, 129.3, 128.2, 126.9, 63.5, 61.7, 42.4, 31.2, 19.8; **IR** (film, cm^{-1}) 2953, 2755, 1456, 1129, 1070, 880, 738, 699; **HRMS:** m/z calculated for $C_{14}H_{22}N$ $[M+H]^+$ 204.1747; found 204.1759.

8-benzyl-1,4-dioxa-8-azaspiro[4.5]decane (**1f**)

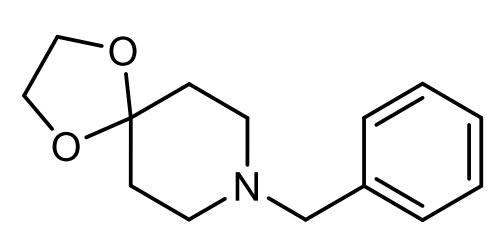

Prepared by general procedure **2.5.1.2.** using 1,4-dioxa-8-azaspiro[4.5]decane (1.4 g, 10 mmol, 1.0 equiv), benzyl bromide (1.2 mL, 10 mmol, 1.0 equiv) and potassium carbonate (2.7 g, 20 mmol, 2.0 equiv) in acetonitrile. Purification by automated flash column chromatography (0–20% ethyl acetate in petroleum ether) gave the title compound as a colourless oil (1.3 g, 5.7 mmol, 57%

yield). Data was in line with previous characterisation.[54] **^{1}H NMR** (500 MHz, $CDCl_3$) δ 7.28 – 7.23 (m, 4H), 7.20 – 7.16 (m, 1H), 3.88 (s, 4H), 3.47 (s, 2H), 2.47 (t, *J* = 5.5 Hz, 4H), 1.68 (t, *J* = 5.7 Hz, 4H); **^{13}C NMR** (126 MHz, $CDCl_3$) δ 138.8, 129.2, 128.3, 127.1, 107.5, 107.5, 64.3, 62.9, 62.8, 51.4, 35.0, 35.0.

1-benzyl-4-phenylpiperidin-4-ol (**1g**)

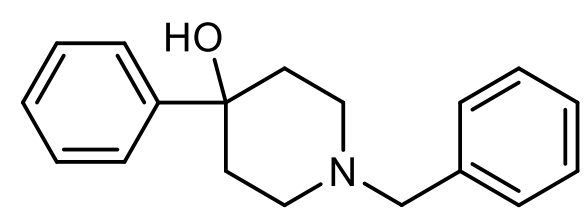

Prepared by general procedure **2.5.1.1.** using 4-phenylpiperidin-4-ol (709 mg, 4.0 mmol, 1.0 equiv), benzaldehyde (0.49 mL, 4.8 mmol, 1.2 equiv), and sodium triacetoxyborohydride (1.7 g, 8.0 mmol, 2 equiv) in tetrahydrofuran. Purification by automated flash column chromatography (10–50% acetone in petroleum ether) gave the title compound as a white solid (930 mg, 3.5 mmol, 88% yield). Data was in line with previous characterisation.[55] **^{1}H NMR** (500 MHz, $CDCl_3$) δ 7.56 – 7.51 (m, 2H), 7.40 – 7.31 (m, 6H), 7.30 – 7.25 (m, 2H), 3.60 (s, 2H), 2.85 – 2.76 (m, 2H), 2.49 (td, *J* = 12.0, 2.5 Hz, 2H), 2.23 – 2.13 (m, 2H), 1.79 – 1.71 (m, 2H); **^{13}C NMR** (126 MHz, $CDCl_3$) δ 148.6, 138.7, 129.3, 128.4, 128.3, 127.1, 127.1, 124.7, 71.4, 63.4, 49.6, 38.7.

1'-benzylspiro[chromane-2,4'-piperidin]-4-one (**1i**)

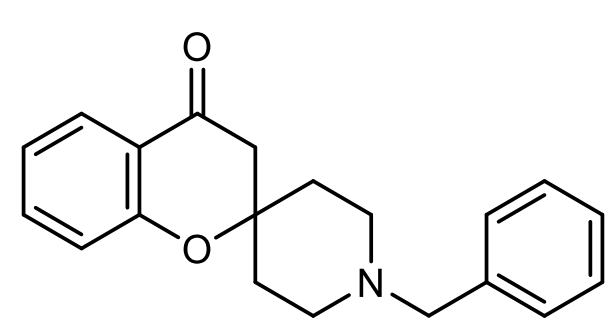

Prepared by general procedure **2.5.1.1.** using spiro[chromane-2,4'-piperidin]-4-one (868 mg, 3.4 mmol, 1.0 equiv), benzaldehyde (0.41 mL, 4.1 mmol, 1.2 equiv), and sodium triacetoxyborohydride (1.44 g, 6.8 mmol, 2.0 equiv) in tetrahydrofuran. Purification by automated flash column chromatography (20%–100% ethyl acetate in petroleum ether) gave the title compound as a yellow solid (782 mg, 2.5 mmol, 63% yield). **^{1}H NMR** (700 MHz, $CDCl_3$) δ 7.85 (dt, *J* = 8.2, 1.1 Hz, 1H), 7.50 – 7.46 (m, 1H), 7.33 – 7.30 (m, 4H), 7.26 (dq, *J* = 5.7, 3.0 Hz, 1H), 7.00 – 6.96 (m, 2H), 3.54 (d, *J* = 1.0 Hz, 2H), 2.71 (d, *J* = 1.0 Hz, 2H), 2.62 (dd, *J* = 11.1, 4.9 Hz,

2H), 2.47 (s, 1H), 2.03 (dq, J = 14.4, 2.7 Hz, 2H), 1.75 (ddd, J = 14.6, 11.4, 4.3 Hz, 2H); **^{13}C NMR** (176 MHz, $CDCl_3$) δ 192.3, 159.4, 138.5, 136.3, 129.2, 128.4, 127.2, 126.7, 121.0, 121.0, 118.5, 78.0, 63.2, 48.9, 48.1, 34.5; **IR** (film, cm^{-1}): 2944, 2812, 2771, 1688, 1607, 1463, 1324, 1299, 1230, 1149, 1115, 976, 892; **HRMS:** m/z calculated for $C_{20}H_{22}NO_2$ $[M + H]^+$ 308.1645; found 308.1648.

1-benzylpyrrolidine (**1j**)

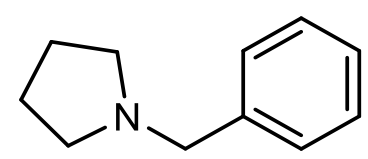

Prepared by general procedure **2.5.1.1.** using benzaldehyde (1.1 g, 10 mmol, 1.0 equiv), pyrrolidine (711 mg, 10 mmol, 1.0 equiv), and sodium triacetoxyborohydride (3.2 g, 15 mmol, 1.5 equiv) in 1,2-dichloroethane (35 mL). Purification by automated flash column chromatography (0–50% ethyl acetate in petroleum ether for 8 minutes, 50–100% ethyl acetate in petroleum ether for 5 minutes, and 100% ethyl acetate for 2 minutes) gave the title compound as a colourless liquid (340 mg, 2.1 mmol, 21% yield). Data was in line with previous characterisation.[56] **^{1}H NMR** (400 MHz, $CDCl_3$) δ 7.41 – 7.28 (m, 4H), 7.28 – 7.22 (m, 1H), 3.63 (s, 2H), 2.52 (tq, J = 5.3, 3.7 Hz, 4H), 1.80 (dq, J = 6.8, 3.2 Hz, 4H); **^{13}C NMR** (101 MHz, $CDCl_3$) δ 139.2, 128.6, 127.9, 126.6, 60.5, 53.9, 23.2.

tert-butyl 7-benzyl-2,7-diazaspiro[4.4]nonane-2-carboxylate (**1k**)

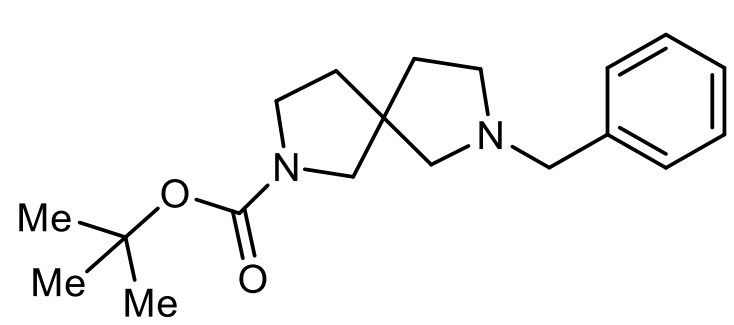

Prepared by general procedure **2.5.1.2.** using tert-butyl 2,7-diazaspiro[4.4]nonane-2-carboxylate (1.0 g, 4.5 mmol, 1.1 equiv), benzyl bromide (0.50 mL, 4.0 mmol, 1.0 equiv) and potassium carbonate (663 mg, 4.8 mmol, 2.0 equiv) in acetonitrile. Purification by automated flash column chromatography (10%–50% ethyl acetate in petroleum ether) gave the title compound as a colourless oil (650 mg, 2.1 mmol, 52% yield). **^{1}H NMR** (500 MHz, $CDCl_3$) δ 7.28 – 7.23 (m, 4H), 7.19 (dq, J = 9.4, 4.4 Hz, 1H), 3.62 – 3.48 (m, 2H), 3.38 – 3.09 (m, 4H), 2.58 (dddd, J = 35.2, 24.1, 8.7, 6.1 Hz, 2H), 2.48 – 2.29 (m, 2H), 1.86 – 1.64 (m, 4H), 1.39 (s, 9H); **^{13}C NMR** (126 MHz,

$CDCl_3$) δ 154.8 (major), 154.7 (minor), 139.3 (minor), 139.1 (major), 128.74 (major), 128.68 (minor), 128.3, 127.03 (major), 126.97 (minor), 79.16 (major), 79.10 (minor), 64.2, 60.4, 58.0 (major), 57.3 (minor), 53.8, 48.3 (major), 47.5 (minor), 45.6 (minor), 45.1 (major), 38.2 (minor), 37.3 (major), 35.73 (minor), 35.67 (major), 28.7; **IR** (film, cm^{-1}): 2974, 2871, 1692, 1397, 1366, 1170, 1143, 1105, 881; **HRMS:** m/z calculated for $C_{19}H_{29}N_2O_2$ $[M + H]^+$ 317.2224; found 317.2226.

Note: the compound was isolated as a mixture of rotamers, identified as minor and major where possible.

tert-butyl 2-benzyl-2,7-diazaspiro[3.5]nonane-7-carboxylate (**1l**)

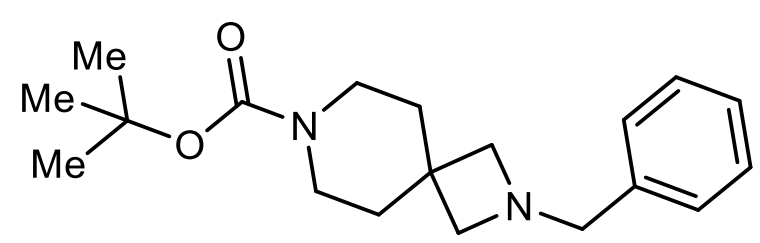

Prepared by general procedure **2.5.1.1.** using tert-butyl 2,7-diazaspiro[3.5]nonane-7-carboxylate (905 mg, 4.0 mmol, 1.0 equiv), benzaldehyde (0.49 mL, 4.8 mmol, 1.2 equiv), and sodium triacetoxyborohydride (1.7 g, 8.0 mmol, 2.0 equiv) in tetrahydrofuran. Purification by automated flash column chromatography (5%–50% acetone in petroleum ether) gave the title compound as a yellow solid (930 mg, 2.9 mmol, 73% yield). Data was in line with previous characterisation.[57]
^{1}H NMR (500 MHz, $CDCl_3$) δ 7.34 – 7.20 (m, 6H), 3.63 (s, 2H), 3.34 – 3.28 (m, 4H), 3.03 (s, 4H), 1.72 – 1.66 (m, 4H), 1.44 (s, 9H); **^{13}C NMR** (126 MHz, $CDCl_3$) δ 155.0, 138.5, 128.5, 128.4, 127.1, 79.5, 64.1, 63.9, 36.1, 34.6, 28.6.

tert-butyl 4-benzyl-1,4-diazepane-1-carboxylate (**1m**)

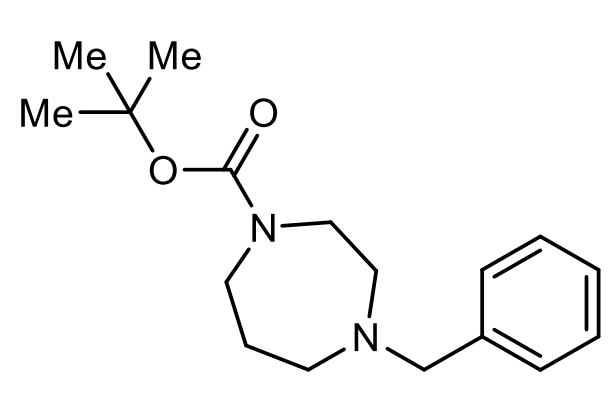

Prepared by general procedure **2.5.1.1.** using tert-butyl 1,4-diazepane-1-carboxylate (0.79 mL, 4.0 mmol, 1.0 equiv), benzaldehyde (0.49 mL, 4.8 mmol, 1.2 equiv), and sodium

triacetoxyborohydride (1.7 g, 8.0 mmol, 2.0 equiv) in tetrahydrofuran. Purification by automated flash column chromatography (20–40% ethyl acetate in petroleum ether) gave the title compound as a colourless liquid (1.0 g, 3.5 mmol, 87% yield). Data was in line with previous characterisation.[58] **^{1}H NMR** (500 MHz, $CDCl_3$) δ 7.34 – 7.28 (m, 4H), 7.26 – 7.22 (m, 1H), 3.62 (s, 2H), 3.50 (q, *J* = 5.9 Hz, 2H), 3.47 – 3.40 (m, 2H), 2.68 – 2.58 (m, 4H), 1.81 (dp, *J* = 24.2, 6.0 Hz, 3H), 1.46 (d, *J* = 3.9 Hz, 9H); **^{13}C NMR** (126 MHz, $CDCl_3$) δ 155.7, 155.6, 139.3, 128.8, 128.8, 128.2, 127.0, 127.0, 79.2, 62.3, 56.2, 55.8, 54.9, 54.7, 46.8, 46.3, 46.1, 45.3, 28.5, 28.1, 27.9.

Note: the compound was isolated as a 1:1 mixture of rotamers.

N-Benzyl-paroxetine (**1n**)

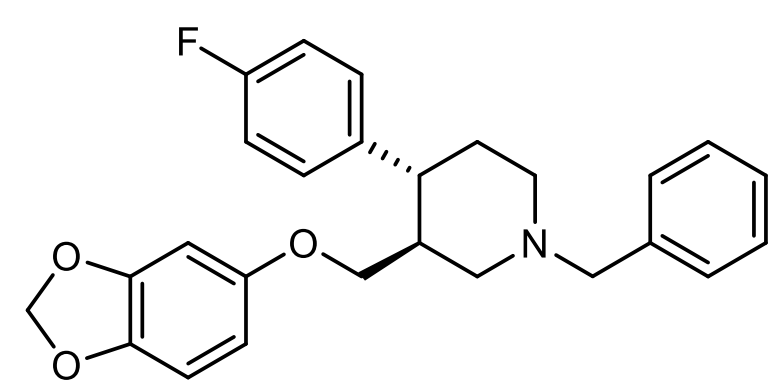

Prepared by general procedure **2.5.1.1.** using paroxetine (549 mg, 1.5 mmol, 1.0 equiv), benzaldehyde (0.18 mL, 1.8 mmol, 1.2 equiv), and sodium triacetoxyborohydride (633 mg, 3.0 mmol, 2.0 equiv). Purification by automated flash column chromatography (2–10% acetone in petroleum ether) gave the title compound as a white solid (390 mg, 0.93 mmol, 66% yield). Data was in line with previous characterisation.[59] **^{1}H NMR** (500 MHz, $CDCl_3$) δ 7.39 – 7.32 (m, 4H), 7.26 (s, 3H), 7.20 – 7.12 (m, 2H), 7.00 – 6.92 (m, 2H), 6.61 (d, *J* = 8.5 Hz, 1H), 6.32 (d, *J* = 2.5 Hz, 1H), 6.10 (dd, *J* = 8.5, 2.5 Hz, 1H), 5.87 (s, 2H), 3.64 (d, *J* = 13.1 Hz, 1H), 3.58 – 3.51 (m, 2H), 3.43 (dd, *J* = 9.4, 6.9 Hz, 1H), 3.24 (ddd, *J* = 11.2, 3.7, 1.8 Hz, 1H), 2.99 (d, *J* = 11.2 Hz, 1H), 2.48 (td, *J* = 11.6, 4.3 Hz, 1H), 2.21 (dtd, *J* = 10.8, 7.3, 3.4 Hz, 1H), 2.08 (dt, *J* = 14.9, 11.3 Hz, 2H), 1.91 – 1.75 (m, 2H); **^{13}C NMR** (126 MHz, $CDCl_3$) δ 161.6 (d, *J* = 244.1 Hz), 154.6, 148.3, 141.7, 140.0, 138.4, 129.4, 129.0, 129.0, 128.4, 127.2, 115.5 (d, *J* = 21.0 Hz)., 108.0, 105.7, 101.2, 98.1, 69.8, 63.6, 57.8, 54.0, 44.3, 42.3, 34.5; **^{19}F NMR** (471 MHz, $CDCl_3$) δ −116.7.

N-benzyl-*N*-methylpent-4-en-1-amine (**1p**)

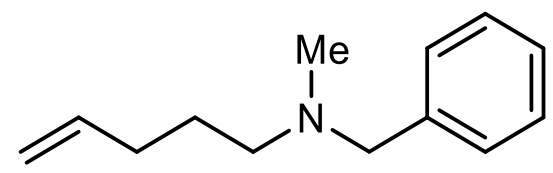

Prepared by general procedure **2.5.1.1.** using *N*-methyl-benzylamine (0.52 mL, 4.0 mmol, 1.0 equiv), 4-pentenal (403 mg, 4.8 mmol, 1.2 equiv), and sodium triacetoxyborohydride (1.7 g, 8.0 mmol, 2.0 equiv) in tetrahydrofuran. Purification by automated flash column chromatography (2% acetone in petroleum ether) gave the title compound as a colourless liquid (412 mg, 2.2 mmol, 55% yield). Data was in line with previous characterisation.[60] **^{1}H NMR** (400 MHz, $CDCl_3$) δ 7.31 (d, *J* = 4.4 Hz, 3H), 7.26 – 7.21 (m, 1H), 5.82 (ddt, *J* = 16.9, 10.1, 6.6 Hz, 1H), 5.01 (dq, *J* = 17.1, 1.7 Hz, 1H), 4.94 (ddt, *J* = 10.1, 2.2, 1.3 Hz, 1H), 3.48 (s, 2H), 2.43 – 2.34 (m, 2H), 2.19 (s, 3H), 2.16 – 2.03 (m, 2H), 1.62 (tt, *J* = 8.8, 6.7 Hz, 2H); **^{13}C NMR** (101 MHz, $CDCl_3$) δ 139.1, 138.7, 128.8, 128.1, 126.1, 114.7, 62.2, 56.8, 42.1, 31.6, 26.7.

2-(benzyl(methyl)amino)-*N,N*-dimethylacetamide (**1q**)

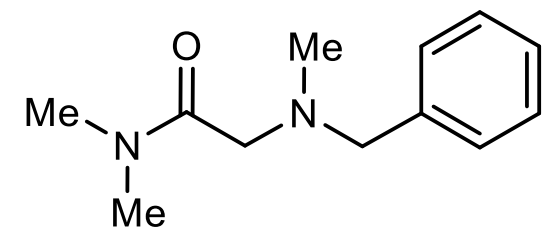

To a solution of 2-bromo-*N*,*N*-dimethylacetamide (0.54 mL, 5.0 mmol, 1.0 equiv) in acetonitrile (15 mL) was added potassium carbonate (3.2 g, 15 mmol, 3.0 equiv) followed by *N*-methyl-benzylamine (1.3 mL, 10 mmol, 2.0 equiv). The reaction mixture was left to stir at room temperature for 16 h. Following this, aqueous 10% NaOH (15 mL) was added and the reaction mixture was stirred for 5 min. The mixture was then extracted with ethyl acetate (25 mL × 3), organics were combined, dried over sodium sulphate, filtered and concentrated. Purification by automated flash column chromatography (5–10% ethyl acetate in petroleum ether) gave the title compound as a colourless oil (1.1 g, 4.5 mmol, 91% yield). **^{1}H NMR** (700 MHz, $CDCl_3$) δ 7.33 – 7.29 (m, 4H), 7.27 – 7.23 (m, 1H), 3.57 (s, 2H), 3.20 (s, 2H), 3.03 (s, 3H), 2.92 (s, 3H), 2.29 (s, 3H). **^{13}C NMR** (176 MHz, $CDCl_3$) δ 170.4, 138.6, 129.3, 128.4, 127.3, 62.2, 60.1, 42.6, 37.2, 35.6; **IR** (film, cm^{-1}): 2932, 2845, 2789, 1638, 1495, 1453, 1397, 1263, 1182, 1026, 996, 869, 740; **HRMS:** m/z calculated for $C_{12}H_{19}N_2O$ $[M + H]^+$ 207.1492; found 207.1492.

N-benzyl (*S*)-duloxetine (**1r**)

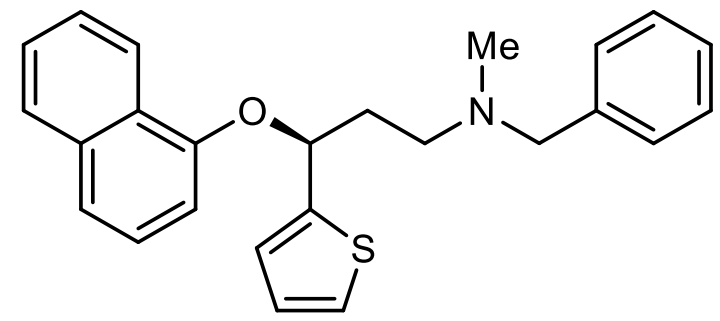

Prepared by general procedure **2.5.1.1.** (*S*)-duloxetine hydrochloride (668 mg, 2.0 mmol, 1.0 equiv), benzaldehyde (0.24 mL, 2.4 mmol, 1.2 equiv), and sodium triacetoxyborohydride (844 mg, 4.0 mmol, 2.0 equiv). Purification by automated flash column chromatography (5–40% ethyl acetate in petroleum ether) gave the title compound as a white solid (544 mg, 1.4 mmol, 68% yield). **^{1}H NMR** (700 MHz, $CDCl_3$) δ 8.27 (ddd, *J* = 8.0, 1.7, 0.8 Hz, 1H), 7.79 – 7.76 (m, 1H), 7.46 (dddd, *J* = 17.3, 8.2, 6.8, 1.5 Hz, 2H), 7.40 – 7.37 (m, 1H), 7.28 – 7.26 (m, 2H), 7.23 – 7.19 (m, 3H), 7.18 – 7.15 (m, 1H), 6.99 (ddd, *J* = 3.5, 1.2, 0.6 Hz, 1H), 6.91 (dd, *J* = 5.0, 3.5 Hz, 1H), 6.88 – 6.85 (m, 1H), 5.81 (dd, *J* = 7.7, 5.3 Hz, 1H), 3.58 – 3.49 (m, 2H), 2.70 (dt, *J* = 13.5, 7.1 Hz, 1H), 2.62 (dt, *J* = 12.9, 6.7 Hz, 1H), 2.49 (dtd, *J* = 13.5, 7.6, 5.9 Hz, 1H), 2.25 (s, 4H); **^{13}C NMR** (176 MHz, $CDCl_3$) δ 153.6, 145.6, 134.7, 129.2, 128.3, 128.2, 127.6, 127.1, 126.7, 126.4, 126.3, 125.9, 125.3, 124.8, 124.7, 122.3, 120.6, 107.1, 74.6, 62.5, 53.4, 42.2, 36.8; **IR** (film, cm^{-1}): 3060, 2940, 2842, 2793, 1580, 1509, 1397, 1265, 1237, 1095, 1068, 1019; **HRMS:** m/z calculated for $C_{25}H_{26}NOS$ $[M + H]^+$ 388.1730; found 388.1728.

2-benzyl-6,7-dimethoxy-1,2,3,4-tetrahydroisoquinoline (**1s**)

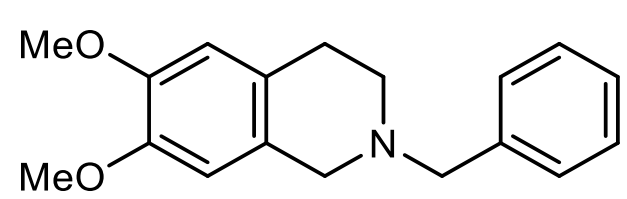

Prepared by general procedure **2.5.1.1.** 6,7-dimethoxy-1,2,3,4-tetrahydroisoquinoline hydrochloride (916 mg, 4.0 mmol, 1.0 equiv), benzaldehyde (0.49 mL, 4.8 mmol, 1.2 equiv), and sodium triacetoxyborohydride (1.7 g, 8.0 mmol, 2.0 equiv) in tetrahydrofuran. Purification by automated flash column chromatography (50% ethyl acetate in petroleum ether) gave the title compound as an orange solid (396 mg, 1.4 mmol, 35% yield). Data was in line with previous characterisation.[61] **^{1}H NMR** (700 MHz, $CDCl_3$) δ 7.40 (dt, *J* = 8.0, 1.0 Hz, 2H), 7.38 – 7.34 (m, 2H), 7.34 – 7.30 (m, 1H), 6.59 (s, 1H), 6.48 (s, 1H), 3.91 – 3.86 (m, 2H), 3.83 (d, *J* = 1.6 Hz, 3H), 3.80 (d, *J* = 1.6 Hz, 3H), 3.76 (d, *J* = 5.6 Hz, 2H), 2.99 – 2.94 (m, 2H), 2.89 (t, *J* = 6.1 Hz, 2H);

^{13}C NMR (176 MHz, $CDCl_3$) δ 148.1, 147.7, 134.9, 130.1, 128.7, 128.2, 125.0, 124.0, 111.4, 109.6, 60.5, 56.0, 56.0, 49.4, 26.8.

2-benzyl-2,3,4,9-tetrahydro-1H-pyrido[3,4-b]indole (**1t**)

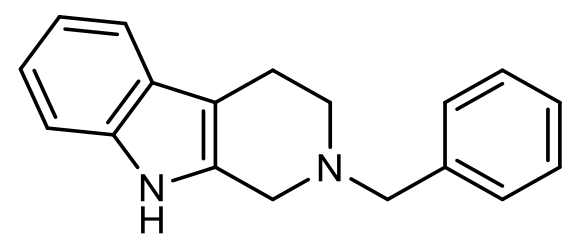

Prepared by general procedure **2.5.1.1.** using noreleagnine (345 mg, 2.0 mmol, 1.0 equiv), benzaldehyde (0.24 mL, 2.4 mmol, 1.2 equiv), and sodium triacetoxyborohydride (843 mg, 4.0 mmol, 2.0 equiv) in tetrahydrofuran. Purification by automated flash column chromatography (10–50% acetone in petroleum ether) gave the title compound as a brown solid (445 mg, 1.7 mmol, 85% yield). Data was in line with previous characterisation.[62] **^{1}H NMR** (500 MHz, $CDCl_3$) δ 7.69 (s, 1H), 7.49 – 7.46 (m, 1H), 7.44 – 7.40 (m, 2H), 7.39 – 7.34 (m, 2H), 7.33 – 7.28 (m, 1H), 7.27 – 7.25 (m, 2H), 7.15 – 7.07 (m, 2H), 3.78 (s, 2H), 3.62 (d, J = 1.6 Hz, 2H), 2.92 (t, J = 5.8 Hz, 2H), 2.84 (tt, J = 4.9, 1.5 Hz, 2H); **^{13}C NMR** (126 MHz, $CDCl_3$) δ 138.6, 136.1, 132.0, 129.3, 128.5, 127.4, 127.3, 121.4, 119.4, 118.1, 110.8, 108.5, 62.1, 51.0, 50.3, 21.3.

1-benzyl-4-phenylpiperazine (**1v**)

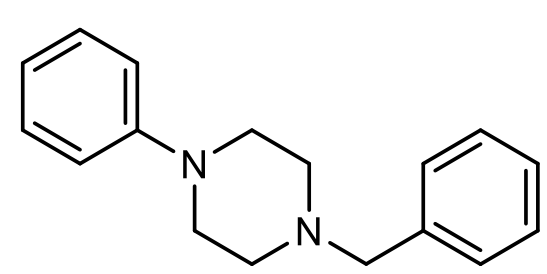

Prepared by general procedure **2.5.1.2.** using *N*-phenyl piperazine (1.6 mL, 10 mmol, 1.0 equiv), benzyl bromide (1.2 mL, 10 mmol, 1.0 equiv), and potassium carbonate (2.8 g, 20 mmol, 2.0 equiv) in acetonitrile. Purification by automated flash column chromatography (5–10% ethyl acetate in petroleum ether) gave the title compound as a yellow liquid (1.9 g, 7.7 mmol, 77% yield). Data was in line with previous characterisation.[63] **^{1}H NMR** (500 MHz, $CDCl_3$) δ 7.42 – 7.35 (m, 4H), 7.34 – 7.27 (m, 3H), 6.99 – 6.95 (m, 2H), 6.89 (tt, J = 7.3, 1.1 Hz, 1H), 3.61 (s, 2H), 3.28 – 3.22 (m, 4H), 2.68 – 2.61 (m, 4H); **^{13}C NMR** (126 MHz, $CDCl_3$) δ 151.4, 138.0, 129.3, 129.1, 128.3, 127.2, 119.6, 116.1, 63.1, 53.2, 49.9.

tert-butyl (*S*)-4-benzyl-3-methylpiperazine-1-carboxylate (**1w**)

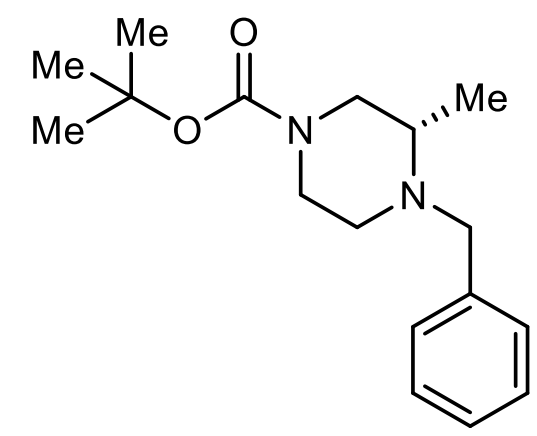

Prepared by general procedure **2.5.1.1.** using (*S*)-1-Boc-3-methylpiperazine (800 mg, 4.0 mmol, 1.0 equiv), benzaldehyde (0.49 mL, 4.8 mmol, 1.2 equiv), and sodium triacetoxyborohydride (1.7 g, 8.0 mmol, 2.0 equiv) in dichloromethane. Purification by automated flash column chromatography (0–40% acetone in 1:1 petroleum ether/dichloromethane) gave the title compound as a colourless liquid (1.1 g, 3.7 mmol, 91% yield). **^{1}H NMR** (700 MHz, $CDCl_3$) δ 7.33 – 7.29 (m, 4H), 7.26 – 7.23 (m, 1H), 3.99 (s, 1H), 3.86 – 3.57 (m, 2H), 3.19 (d, *J* = 13.3 Hz, 1H), 3.07 (s, 1H), 2.88 (d, *J* = 58.0 Hz, 1H), 2.64 (d, *J* = 11.7 Hz, 1H), 2.50 – 2.39 (m, 1H), 2.08 (s, 1H), 1.45 (s, 9H), 1.13 (d, *J* = 6.2 Hz, 3H); **^{13}C NMR** (176 MHz, $CDCl_3$) δ 154.9, 138.9, 129.2, 128.4, 127.1, 79.6, 58.2, 55.0, 50.7, 50.0, 44.5, 43.4, 28.6, 15.7, 15.3; **IR** (film, cm^{-1}): 2973, 2804, 1693, 1454, 1422, 1366, 1245, 1169, 1128; **HRMS:** m/z calculated for $C_{17}H_{27}N_2O_2$ $[M + H]^+$ 291.2067; found 291.2069.

4-(4-methoxybenzyl)morpholine (**1aa**)

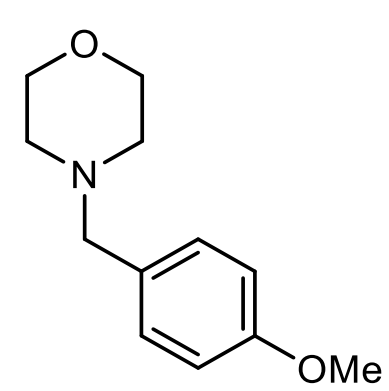

Prepared by general procedure **2.5.1.1.** using morpholine (0.21 mL, 2.4 mmol, 1.2 equiv), *p*-anisaldehyde (0.24 mL, 2.0 mmol, 1.0 equiv), and sodium triacetoxyborohydride (843 mg, 4.0 mmol, 2.0 equiv) in dichloromethane. The title compound was obtained as a yellow liquid (352 mg, 1.7 mmol, 85% yield) and used in the next step without further purification. Data in line with previous characterisation.[64] **^{1}H NMR** (500 MHz, $CDCl_3$) δ 7.23 (d, J = 8.5 Hz, 2H), 6.85 (d, J = 8.6 Hz, 2H), 3.83 – 3.76 (m, 3H), 3.74 – 3.66 (m, 4H), 3.46 (s, 2H), 2.50 – 2.39 (m, 4H); **^{13}C NMR** (126 MHz, CDCl3) δ 158.9, 130.6, 129.2, 113.7, 66.8, 62.7, 55.3, 53.3.

1-(4-(*tert*-butyl)benzyl)piperidine (**1ab**)

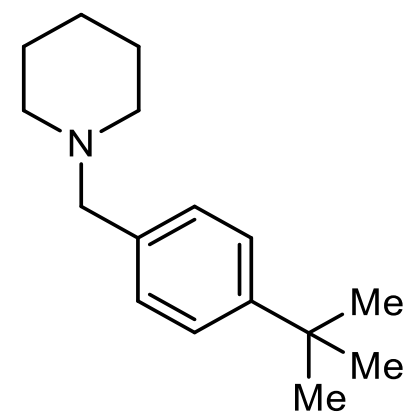

Prepared by general procedure **2.5.1.2.** using piperidine (3.4 g, 40 mmol, 4.0 equiv) and 4-*tert*-butylbenzyl bromide (2.3 g, 10 mmol, 1.0 equiv) in dichloromethane (20 mL). Purification by automated flash column chromatography (5–60% ethyl acetate in petroleum ether for 25 minutes) gave the title compound as a colourless oil (1.9 g, 8.1 mmol, 81% yield). Data was in line with previous characterisation.[65] **^{1}H NMR** (500 MHz, CDCl3) δ 7.36 – 7.30 (m, 2H), 7.28 – 7.22 (m, 2H), 3.45 (s, 2H), 2.38 (s, 4H), 2.05 (br s, 1H), 1.58 (p, *J* = 5.6 Hz, 4H), 1.43 (m, 2H), 1.32 (s, 9H); **^{13}C NMR** (101 MHz, $CDCl_3$) δ 149.8, 135.7, 129.1, 125.1, 63.7, 54.7, 34.6, 31.5, 26.1, 24.6.

4-(4-(trifluoromethyl)benzyl)morpholine (**1ac**)

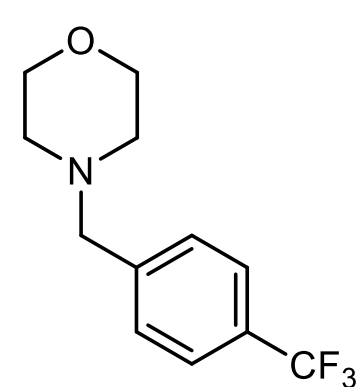

Prepared by general procedure **2.5.1.1.** using morpholine (174 mg, 2.0 mmol, 1.0 equiv), 4-trifluoromethylbenzaldehyde (348 mg, 2.0 mmol, 1.0 equiv), titanium (IV) isopropoxide (711 mg, 2.5 mmol, 1.3 equiv), and sodium triacetoxyborohydride (844 mg, 4.0 mmol, 2.0 equiv) in tetrahydrofuran (10 mL). Purification by automated flash column chromatography (0–100% ethyl acetate in petroleum ether for 15 minutes, 100% ethyl acetate for 5 minutes) gave the title compound as a colourless oil (437 mg, 1.8 mmol, 89% yield). Data was in line with previous characterisation.[51] **^{1}H NMR** (400 MHz, $CDCl_3$) δ 7.57 (d, *J* = 8.0 Hz, 2H), 7.46 (d, *J* = 8.0 Hz, 2H), 3.72 (m, 4H), 3.54 (s, 2H), 2.43 (m, 4H); **^{13}C NMR** (101 MHz, $CDCl_3$) δ 142.32, 142.30, 129.4, 125.3 (q, *J* = 4.0 Hz), 67.1, 63.0, 53.8; **^{19}F NMR** (471 MHz, $CDCl_3$) δ −63.2.

4-(2-methylbenzyl)morpholine (**1ad**)

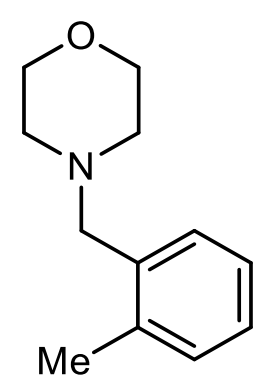

Prepared by general procedure **2.5.1.1.** using morpholine (174 mg, 2.0 mmol, 1.0 equiv), 2-methylbenzaldehyde (240 mg, 2.0 mmol, 1.0 equiv), titanium (IV) isopropoxide (711 mg, 2.5 mmol, 1.3 equiv), and sodium triacetoxyborohydride (844 mg, 4.0 mmol, 2.0 equiv). The title compound was obtained as a yellow liquid (302 mg, 1.5 mmol, 79% yield) and was used in the next step without further purification. Data was in line with previous characterization.[66] **^{1}H NMR** (500 MHz, $CDCl_3$) δ 7.26 (m, 1H), 7.22 – 7.11 (m, 3H), 3.69 (m, 4H), 3.46 (s, 2H), 2.44 (m, 4H), 2.39 (s, 3H); **^{13}C NMR** (126 MHz, $CDCl_3$) δ 137.7, 136.1, 130.4, 130.0, 127.3, 125.6, 67.3, 61.4, 53.8, 19.3.

1-(4-fluoro-2-methoxybenzyl)piperidine (**1ae**)

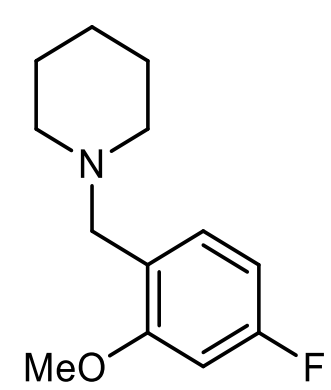

Prepared by general procedure **2.5.1.1.** using piperidine (0.40 mL, 4.0 mmol, 1.0 equiv), 4-fluoro-2-methoxybenzaldehyde (740 mg, 4.8 mmol, 1.2 equiv), and sodium triacetoxyborohydride (1.7 g, 8.0 mmol, 2.0 equiv) in tetrahydrofuran. Purification by automated flash column chromatography (10–40% acetone in petroleum ether) gave the title compound as a colourless oil (550 mg, 2.4 mmol, 60% yield).**^{1}H NMR** (500 MHz, $CDCl_3$) δ 7.29 (ddt, *J* = 8.4, 7.0, 0.7 Hz, 1H), 6.62 (td, *J* = 8.3, 2.5 Hz, 1H), 6.58 (dd, *J* = 11.0, 2.5 Hz, 1H), 3.79 (s, 3H), 3.46 (d, *J* = 0.8 Hz, 2H), 2.40 (s, 4H), 1.62 – 1.53 (m, 4H), 1.42 (q, *J* = 6.1 Hz, 2H); **^{13}C NMR** (126 MHz, $CDCl_3$) δ 162.7 (d, *J* = 243.8 Hz), 158.9 (d, *J* = 9.6 Hz), 131.3 (d, *J* = 9.7 Hz), 122.4 (d, *J* = 3.3 Hz), 106.5 (d, *J* = 20.8 Hz), 98.8 (d, *J* = 25.5 Hz), 56.4, 55.7, 54.6, 26.2, 24.5; **^{19}F NMR** (376 MHz, $CDCl_3$)

δ −114.7.; **IR** (film, cm^{-1}): 2936, 2819, 1518, 1467, 1431, 1343, 1279, 1246, 1159, 1038; **HRMS:** m/z calculated for $C_{13}H_{18}FNO$ $[M + H]^+$ 224.1445; found 224.1443.

1-(3,5-di-*tert*-butylbenzyl)piperidine (**1af**)

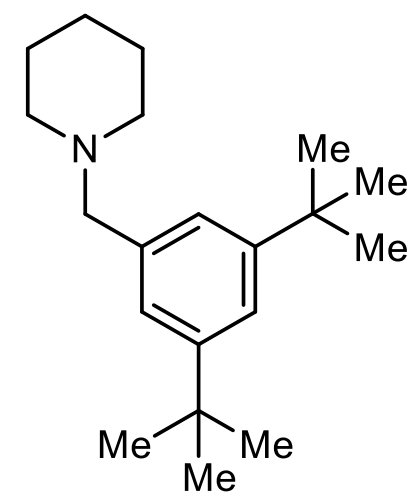

Prepared by general procedure **2.5.1.1.** using piperidine (852 mg, 10 mmol, 1.0 equiv), 3,5-di-*tert*-butylbenzaldehyde (2.6 g, 12 mmol, 1.0 equiv), acetic acid (240 mg, 4.0 mmol, 0.40 equiv), anhydrous sodium sulphate (1.1 mg, 8.0 mmol, 0.80 equiv), and sodium triacetoxyborohydride (4.2 mg, 20 mmol, 2.0 equiv) in dichloromethane (40 mL). Purification by automated flash column chromatography (0–50% ethyl acetate in petroleum ether for 8 minutes, 50–100% ethyl acetate in petroleum ether for 4 minutes, 100% ethyl acetate for 3 minutes) gave the title compound as a colourless oil (429 mg, 1.5 mmol, 15% yield). **^{1}H NMR** (500 MHz, $CDCl_3$) δ 7.42 (t, J = 2.2 Hz, 1H), 7.26 (d, J = 2.3 Hz, 2H), 3.59 (s, 2H), 2.48 (br s, 4H), 1.68 (q, J = 5.6 Hz, 4H), 1.55 – 1.49 (br m, 2H), 1.44 (s, 18H); **^{13}C NMR** (126 MHz, $CDCl_3$) δ 150.4, 137.5, 123.6, 120.8, 64.5, 54.5, 34.8, 31.7, 26.1, 24.6; **IR** (film, cm^{-1}): 2957, 2936, 1478, 1363, 1249, 1200, 1120, 1040, 874, 714; **HRMS:** m/z calculated for $C_{20}H_{34}N$ $[M + H]^+$ 288.2686; found 288.2689.

1-(naphthalen-2-ylmethyl)piperidine (**1ag**)

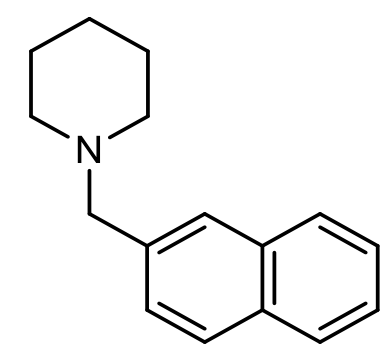

Prepared by general procedure **2.5.1.1.** using piperidine (426 mg, 5.0 mmol, 1.0 equiv), 2-naphthaldehyde (937 mg, 6.0 mmol, 1.2 equiv), and sodium triacetoxyborohydride (2.1 mg, 10 mmol, 2.0 equiv) in tetrahydrofuran (20 mL). Purification by automated flash column chromatography (0–100% ethyl acetate in petroleum ether for 15 minutes, 100% ethyl acetate for 5 minutes) to give the title compound as an orange solid (969 mg, 4.3 mmol, 86% yield). Data was in line with previous characterization.[67] **^{1}H NMR** (500 MHz, $CDCl_3$) δ 7.87 – 7.80 (m, 3H), 7.78 – 7.76 (m, 1H), 7.54 (dd, *J* = 8.4, 1.8 Hz, 1H), 7.51 – 7.44 (m, 2H), 3.66 (s, 2H), 2.46 (br s, 4H), 1.63 (p, *J* = 5.5 Hz, 4H), 1.47 (p, *J* = 5.9 Hz, 2H); **^{13}C NMR** (126 MHz, $CDCl_3$) δ 136.2, 133.4, 132.8, 127.8, 127.7, 127.7, 127.7, 125.9, 125.5, 64.0, 54.6, 26.0, 24.5.

1-(4-(piperidin-1-yl)benzyl)piperidine (**1ah**)

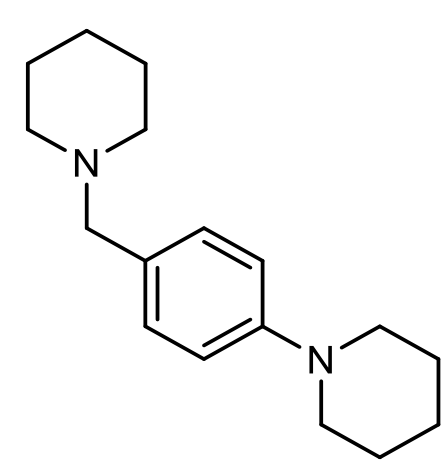

Prepared by general procedure **2.5.1.1.** using piperidine (0.40 mL, 4.0 mmol, 1.0 equiv), 4-(piperidin-1-yl)benzaldehyde (908 mg, 4.8 mmol, 1.2 equiv), and sodium triacetoxyborohydride (1.7 g, 8.0 mmol, 2.0 equiv) in tetrahydrofuran. Purification by automated flash column chromatography (1–10% acetone in 1:1 petroleum ether/dichloromethane) gave the title compound as a yellow oil (615 mg, 2.4 mmol, 60% yield). **^{1}H NMR** (500 MHz, $CDCl_3$) δ 7.2 – 7.1 (m, 2H), 6.9 – 6.9 (m, 2H), 3.4 (s, 2H), 3.2 – 3.1 (m, 4H), 2.3 (s, 4H), 1.7 – 1.7 (m, 4H), 1.6 – 1.5 (m, 6H), 1.4 (q, *J* = 6.1 Hz, 2H); **^{13}C NMR** (126 MHz, $CDCl_3$) δ 151.4, 130.2, 129.1, 116.3, 63.5, 54.5, 50.9, 26.1, 26.1, 24.6, 24.5; **IR** (film, cm^{-1}): 2932, 2853, 2794, 1612, 1514, 1467, 1236, 1220, 1120, 918; **HRMS:** m/z calculated for $C_{17}H_{26}N_2$ $[M + H]^+$ 259.2169; found 259.2168.

4-(2-oxopyrrolidin-1-yl)benzaldehyde

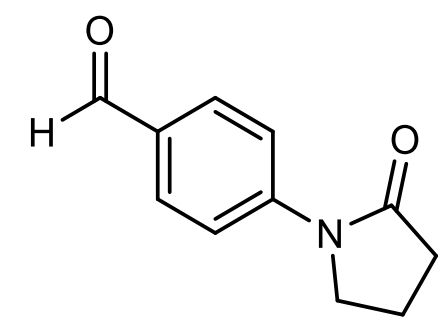

Following a reported procedure,[68] using 4-bromobenzaldehyde (3.5 g, 19 mmol, 1.0 equiv), 2-pyrrolidinone (1.8 mL, 23 mmol, 1.2 equiv), cesium carbonate (18 g, 58 mmol, 3.0 equiv), tris(dibenzylidineacetone)dipalladium (900 mg, 1.0 mmol, 5 mol%), and Xantphos (1.1 g, 2.0 mmol, 10 mol%) in toluene. Purification by automated column chromatography (5–30% ethyl acetate in petroleum ether) gave the title compound as a yellow solid (1.1 g, 6.0 mmol, 32% yield). Data was in line with previous characterisation.[69] **^{1}H NMR** (400 MHz, $CDCl_3$) δ 9.95 (s, 1H), 7.91 – 7.87 (m, 2H), 7.84 (d, J = 8.9 Hz, 2H), 3.93 (t, J = 7.0 Hz, 2H), 2.66 (t, J = 8.1 Hz, 2H), 2.27 – 2.15 (m, 2H); **^{13}C NMR** (δ 101 MHz, $CDCl_3$) δ 191.3, 174.9, 144.8, 132.2, 130.9, 119.2, 48.6, 33.0, 18.0.

1-(4-(piperidin-1-ylmethyl)phenyl)pyrrolidin-2-one (**1ai**)

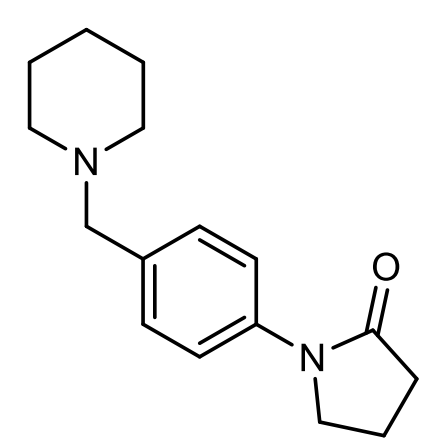

Prepared by general procedure **2.5.1.1.** using piperidine (0.40 mL, 4.0 mmol, 1.0 equiv), 4-(2-oxopyrrolidin-1-yl)benzaldehyde (908 mg, 4.8 mmol, 1.2 equiv), and sodium triacetoxyborohydride (1.7 g, 8.0 mmol, 2.0 equiv) in tetrahydrofuran. Purification by automated flash column chromatography (1%–30% acetone in 1:1 petroleum ether/dichloromethane) gave the title compound as a yellow solid (550 mg, 2.4 mmol, 60% yield). **^{1}H NMR** (500 MHz, $CDCl_3$) δ 7.56 – 7.52 (m, 2H), 7.33 – 7.28 (m, 2H), 3.86 (t, J = 6.9 Hz, 2H), 3.44 (s, 2H), 2.60 (t, J = 8.1 Hz, 2H), 2.35 (s, 4H), 2.21 – 2.10 (m, 2H), 1.56 (p, J = 5.6 Hz, 4H), 1.42 (q, J = 6.0 Hz, 2H); **^{13}C NMR** (126 MHz, $CDCl_3$) δ 174.3, 138.3, 135.0, 129.8, 119.8, 63.4, 54.5, 49.0, 32.9, 26.1, 24.5, 18.2; **IR** (film, cm^{-1}): 2932, 2795, 1692, 1613, 1514, 1389, 1299, 1222, 1112, 1099, 862, 838; **HRMS:** m/z calculated for $C_{16}H_{23}N_2O$ $[M + H]^+$ 259.1805; found 259.1806.

4-((2,3-dihydrobenzo[*b*][1,4]dioxin-6-yl)methyl)morpholine (**1aj**)

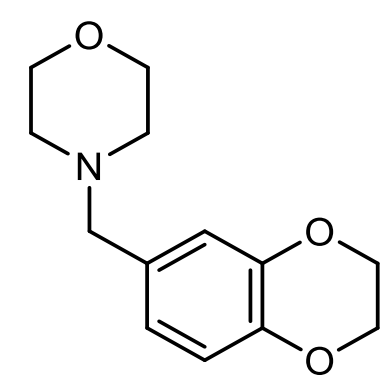

Prepared by General Procedure **2.5.1.1.** using morpholine (174 mg, 2.0 mmol, 1.0 equiv), 2,3-dihydrobenzo[*b*][1,4]dioxine-6-carbaldehyde (312 mg, 2.0 mmol, 1.0 equiv), titanium (IV) isopropoxide (711 mg, 2.5 mmol, 1.2 equiv), and sodium triacetoxyborohydride (844 mg, 4.0 mmol, 2.0 equiv) in tetrahydrofuran (10 mL). Purification by automated flash column chromatography (0–50% ethyl acetate in petroleum ether for 12 minutes, 50–100% ethyl acetate in petroleum ether for 1 minute, 100% ethyl acetate for 2 minutes) gave the title compound as a white solid (282 mg, 1.2 mmol, 60% yield). **^{1}H NMR** (500 MHz, $CDCl_3$) δ 6.84 (d, J = 1.8 Hz, 1H), 6.79 (s, 1H), 6.78 (d, J = 1.8 Hz, 1H), 4.26 – 4.22 (m, 4H), 3.70 (m, 4H), 3.38 (s, 2H), 2.42 (m, 4H); **^{13}C NMR** (126 MHz, $CDCl_3$) δ 143.4, 142.8, 131.2, 122.3, 118.0, 117.0, 67.2, 64.50, 64.48, 63.0, 53.7; **IR** (film, cm^{-1}) 2855, 2807, 1506, 1285, 1259, 1114, 1067, 1007, 886; **HRMS:** m/z calculated for $C_{13}H_{18}NO_3$ $[M+H]^+$ 236.1281; found 236.1280.

3-(4,4-difluoropiperidine-1-carbonyl)benzaldehyde

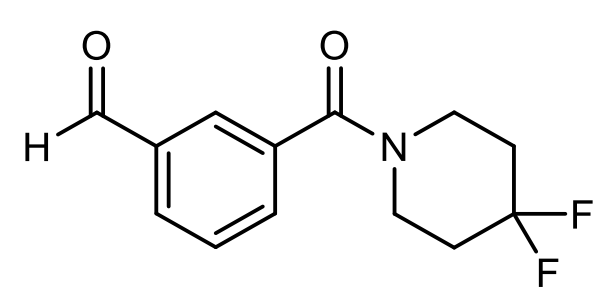

To a solution of 3-formylbenzoic acid (1.5 g, 10 mmol, 1.0 equiv) in dichloromethane (65 mL) was added triethylamine (3.5 mL, 25 mmol, 2.5 equiv), 1-hydroxybenzotriazole hydrate (1.8 g, 12 mmol, 1.2 equiv) and 1-(3-dimethylaminopropyl)-3-ethylcarbodiimide hydrochloride (2.3 g, 12 mmol, 1.2 equiv). The reaction mixture was stirred at room temperature for 1 hour. The mixture was then cooled to 0 °C, and 4,4-difluoropiperidine hydrochloride (1.7 g, 11 mmol, 1.1 equiv) was added. The resulting reaction mixture was stirred at room temperature for 16 h. Following this, dichloromethane (50 mL) was added and the mixture was washed with saturated aqueous citric acid (2 × 80 mL), followed by saturated aqueous sodium bicarbonate (2 × 80 mL), and brine (80

mL). The organic phase was dried over sodium sulphate and concentrated. The title compound was obtained as a yellow solid (1.9 g, 7.5 mmol, 75% yield) and used in the next step without further purification. **^{1}H NMR** (500 MHz, $CDCl_3$) δ 10.04 (s, 1H), 7.96 (dt, *J* = 7.6, 1.5 Hz, 1H), 7.92 (t, *J* = 1.7 Hz, 1H), 7.68 (dt, *J* = 7.6, 1.5 Hz, 1H), 7.62 (t, *J* = 7.6 Hz, 1H), 3.72 (d, *J* = 169.8 Hz, 4H), 2.28 – 1.74 (m, 4H); **^{13}C NMR** (126 MHz, $CDCl_3$) δ 191.4, 169.3, 136.7, 136.4, 132.8, 131.4, 129.7, 128.0, 121.4 (t, *J* = 242.5 Hz), 44.6, 39.4, 34.3 (d, *J* = 88.4 Hz); **^{19}F NMR** (376 MHz, $CDCl_3$) δ −99.0; **IR** (film, cm^{-1}): 2941, 2879, 1702, 1637, 1602, 1445, 1361, 1273, 1202, 1123, 1099, 972; **HRMS:** m/z calculated for $C_{13}H_{14}F_2NO_2$ $[M + H]^+$ 254.0987; found 254.0976.

(4,4-difluoropiperidin-1-yl)(3-(piperidin-1-ylmethyl)phenyl)methanone (**1ak**)

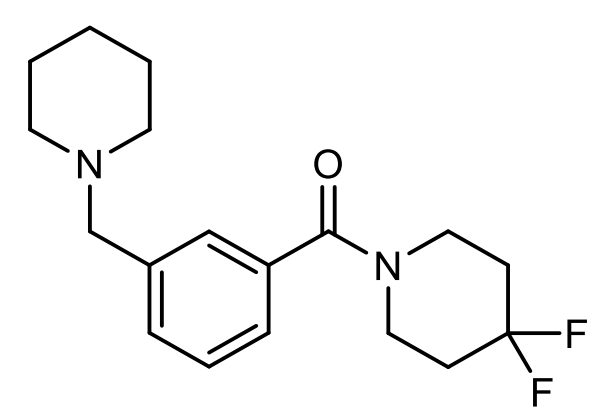

Prepared by general procedure **2.5.1.1.** using using piperidine (0.40 mL, 4.0 mmol, 1.0 equiv), 3-(4,4-difluoropiperidine-1-carbonyl)benzaldehyde (1.2 g, 4.8 mmol, 1.2 equiv), and sodium triacetoxyborohydride (1.7 g, 8.0 mmol, 2.0 equiv) in tetrahydrofuran. Purification by automated flash column chromatography (0–30% acetone in 1:1 petroleum ether/dichloromethane) gave the title compound as a beige solid (470 mg, 1.5 mmol, 38% yield). **^{1}H NMR** (700 MHz, $CDCl_3$) δ 7.41 – 7.37 (m, 2H), 7.35 (t, *J* = 7.5 Hz, 1H), 7.27 (dt, *J* = 7.5, 1.5 Hz, 1H), 3.96 – 3.50 (m, 4H), 3.48 (s, 2H), 2.36 (s, 4H), 2.00 (d, *J* = 89.6 Hz, 5H), 1.56 (p, *J* = 5.6 Hz, 4H), 1.43 (s, 2H). **^{13}C NMR** (176 MHz, $CDCl_3$) δ 170.9, 139.7, 135.2, 130.9, 128.6, 127.5, 125.5, 121.7 (t, *J* = 242.3 Hz), 63.5, 54.7, 44.6, 39.2, 34.7, 34.0, 26.1, 24.4; **^{19}F NMR** (376 MHz, $CDCl_3$) δ −98.77; **IR** (film, cm^{-1}): 2935, 2795, 2756, 1638, 1438, 1420, 1358, 1264, 1121, 1096, 952, 928; **HRMS:** m/z calculated for $C_{18}H_{25}N_2OF_2$ $[M + H]^+$ 323.1930; found 323.1931.

4-(3-(trifluoromethyl)-1H-pyrazol-1-yl)benzaldehyde

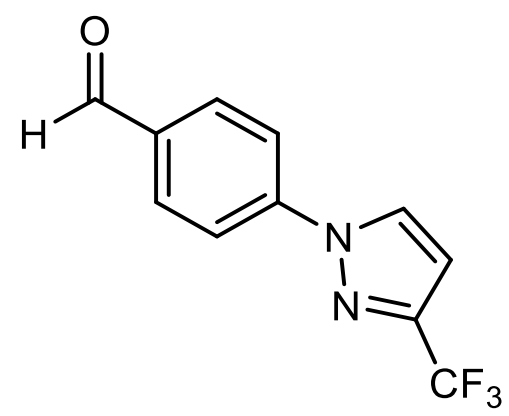

Following the procedure by Banothu and coworkers,[70] using 4-fluorobenzaldehyde (2.4 g, 20 mmol, 1.0 equiv), 3-trifluoromethyl-1H-pyrazole (3.4 g, 25 mmol, 1.3 equiv), and potassium carbonate (3.6 g, 58 mmol, 1.3 equiv), in *N*,*N*-dimethylformamide. Purification by automated flash column chromatography (10–50% ethyl acetate in petroleum ether) gave the title compound as a yellow solid (2.2 g, 9.2 mmol, 46% yield). Data was in line with previous characterisation.[74] **^{1}H NMR** (400 MHz, $CDCl_3$) δ 10.05 (s, 1H), 8.07 (dt, *J* = 2.7, 0.9 Hz, 1H), 8.04 – 8.00 (m, 2H), 7.95 – 7.89 (m, 2H), 6.78 (d, *J* = 2.6 Hz, 1H); **^{13}C NMR** (101 MHz, $CDCl_3$) δ 190.9, 145.2 (q, *J* = 38.6 Hz), 143.5, 135.3, 131.5, 128.6, 122.4 (q, *J* = 269.4 Hz), 119.8, 107.1 (d, *J* = 2.1 Hz); **^{19}F NMR** (376 MHz, $CDCl_3$) δ −62.42.

1-(4-(3-(trifluoromethyl)-1H-pyrazol-1-yl)benzyl)piperidine (**1al**)

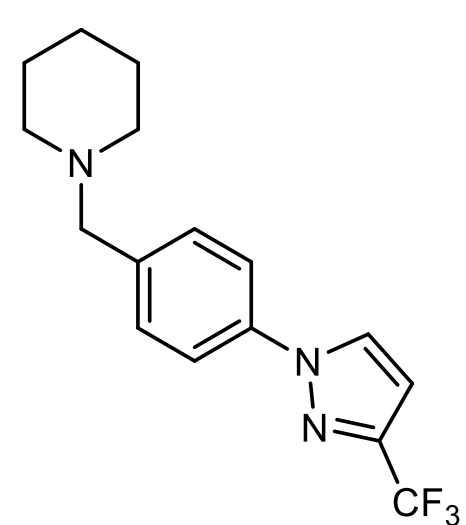

Prepared by general procedure **2.5.1.1.** using using piperidine (0.40 mL, 4.0 mmol, 1.0 equiv), 4-(3-(trifluoromethyl)-1H-pyrazol-1-yl)benzaldehyde (1.2 g, 4.8 mmol, 1.2 equiv), and sodium triacetoxyborohydride (1.7 g, 8.0 mmol, 2.0 equiv) in tetrahydrofuran. Purification by automated flash column chromatography (0%–40% acetone in 1:1 petroleum ether/dichloromethane) gave the title compound as a beige oil (490 mg, 1.6 mmol, 40% yield). **^{1}H NMR** (700 MHz, $CDCl_3$) δ 7.91 (dd, *J* = 2.4, 1.1 Hz, 1H), 7.66 – 7.60 (m, 2H), 7.44 – 7.39 (m, 2H), 6.69 (d, *J* = 2.4 Hz, 1H), 3.50 (s, 2H), 2.38 (s, 4H), 1.57 (p, *J* = 5.6 Hz, 4H), 1.43 (p, *J* = 5.9 Hz, 2H); **^{13}C NMR** (176 MHz, $CDCl_3$) δ 143.9 (q, *J* = 38.4 Hz), 138.8, 138.4, 130.3, 128.4, 122.1 (q, *J* = 267.8 Hz), 119.8, 105.9

(d, *J* = 2.1 Hz), 63.2, 54.6, 26.1, 24.4; **^{19}F NMR** (376 MHz, $CDCl_3$) δ −63.04; **IR** (film, cm^{-1}): 2937, 2800, 2760, 1513, 1483, 1389, 1272, 1125, 1054, 971, 863; **HRMS:** m/z calculated for $C_{16}H_{19}N_3F_3$ $[M + H]^+$ 310.1526; found 310.1527.

2-(3-(piperidin-1-ylmethyl)phenyl)pyridine (**1am**)

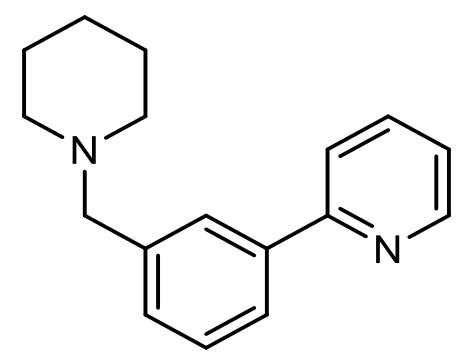

Prepared by general procedure **2.5.1.1.** using piperidine (0.40 mL, 4.0 mmol, 1.0 equiv), 3-(pyridin-2-yl)benzaldehyde (1.2 g, 4.8 mmol, 1.2 equiv), and sodium triacetoxyborohydride (1.7 g, 8.0 mmol, 2.0 equiv) in dichloromethane. Purification by automated flash column chromatography (5–20% acetone in petroleum ether) gave the title compound as a colourless oil (830 mg, 3.3 mmol, 82% yield). **^{1}H NMR** (700 MHz, $CDCl_3$) δ 8.69 (dt, *J* = 4.6, 1.3 Hz, 1H), 7.93 (d, *J* = 2.1 Hz, 1H), 7.86 (dq, *J* = 7.4, 1.6 Hz, 1H), 7.76 – 7.71 (m, 2H), 7.44 – 7.38 (m, 2H), 7.22 (h, *J* = 4.4 Hz, 1H), 3.56 (s, 2H), 2.41 (s, 4H), 1.58 (p, *J* = 5.6 Hz, 4H), 1.43 (q, *J* = 6.0 Hz, 2H); **^{13}C NMR** (176 MHz, $CDCl_3$) δ 157.8, 149.8, 139.4, 139.4, 136.8, 130.0, 128.7, 127.9, 125.7, 122.1, 120.9, 64.0, 54.7, 26.1, 24.5; **IR** (film, cm^{-1}): 2934, 2791, 1581, 1463, 1436, 1343, 1152, 1112, 1040, 992; **HRMS:** m/z calculated for $C_{17}H_{21}N_2$ $[M + H]^+$ 253.1699; found 253.1701.

2-methyl-6-(piperidin-1-ylmethyl)quinoline (**1an**)

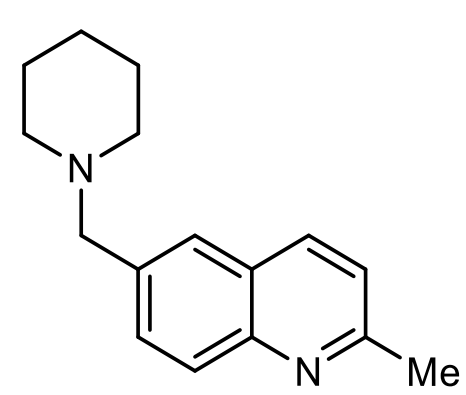

Prepared by general procedure **2.5.1.1.** using piperidine (0.40 mL, 4.0 mmol, 1.0 equiv), 2-methylquinoline-6-carboxaldehyde (822 mg, 4.8 mmol, 1.2 equiv), and sodium triacetoxyborohydride (1.7 g, 8.0 mmol, 2.0 equiv) in dichloromethane. Purification by automated flash column chromatography (0–20% acetone in 1:1 petroleum ether/dichloromethane) gave the

title compound as a brown oil (400 mg, 1.6 mmol, 40% yield). Data was in line with previous characterisation.[75] ^{1}**H NMR** (400 MHz, $CDCl_3$) δ 8.01 (d, J = 8.4 Hz, 1H), 7.98 – 7.93 (m, 1H), 7.68 (dd, J = 6.7, 2.2 Hz, 2H), 7.28 – 7.24 (m, 3H), 3.62 (s, 2H), 2.73 (s, 3H), 2.42 (s, 4H), 1.59 (p, J = 5.6 Hz, 4H), 1.45 (d, J = 5.9 Hz, 2H); ^{13}**C NMR** (101 MHz, $CDCl_3$) δ 158.4, 146.9, 136.2, 135.9, 131.2, 128.2, 126.8, 126.3, 121.9, 63.4, 54.5, 26.0, 25.3, 24.5.

5-(piperidin-1-ylmethyl)-2-(trifluoromethyl)pyridine (**1ao**)

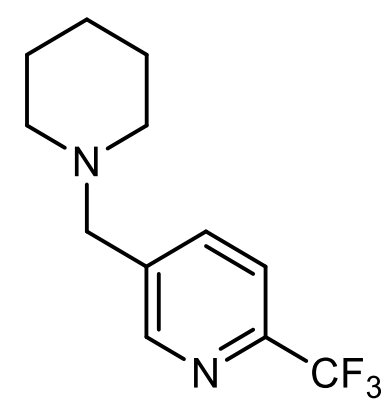

Prepared by general procedure **2.5.1.1.** using piperidine (0.40 mL, 4.0 mmol, 1.0 equiv), 2-(Trifluoromethyl)pyridine-5-carboxaldehyde (840 mg, 4.8 mmol, 1.2 equiv), and sodium triacetoxyborohydride (1.7 g, 8.0 mmol, 2.0 equiv) in dichloromethane. Purification by automated flash column chromatography (0–50% acetone in 1:1 petroleum ether/dichloromethane) gave the title compound as a colourless oil (750 mg, 3.0 mmol, 75% yield). Data was in line with previous characterisation.[76] ^{1}**H NMR** (500 MHz, $CDCl_3$) δ 8.67 – 8.61 (m, 1H), 7.85 (dd, J = 8.2, 2.0 Hz, 1H), 7.63 (d, J = 7.9 Hz, 1H), 3.53 (s, 2H), 2.43 – 2.30 (m, 4H), 1.57 (p, J = 5.6 Hz, H), 1.44 (q, J = 6.1 Hz, 2H); ^{13}**C NMR** (^{13}C NMR (126 MHz, $CDCl_3$) δ 150.6, 147.0 (q, J = 34.5 Hz), 138.2 – 138.1 (m), 137.8, 122.9 (q, J = 276.4 Hz), 120.2 (q, J = 2.8 Hz), 60.6, 54.7, 26.1, 24.3; ^{19}**F NMR** (376 MHz, $CDCl_3$) δ −67.74.

2-phenyl-5-(piperidin-1-ylmethyl)pyridine (**1ap**)

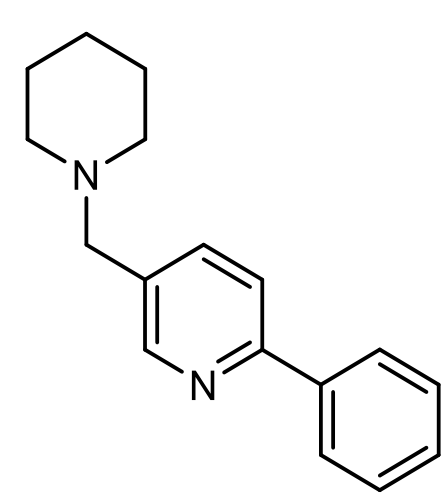

Prepared by general procedure **2.5.1.1.** using piperidine (341 mg, 4.0 mmol, 1.0 equiv), 6-phenylnicotinaldehyde (993 mg, 5.4 mmol, 1.4 equiv), acetic acid (3 drops), anhydrous sodium sulphate (one scoop), and sodium triacetoxyborohydride (1.7 g, 8.0 mmol, 2.0 equiv) in dichloromethane (16 mL). Purification by automated flash column chromatography (0–20% methanol in dichloromethane for 20 minutes, 20% methanol in dichloromethane for 5 minutes) gave the title compound as a yellow oil (945 mg, 3.7 mmol, 94% yield). **^{1}H NMR** (400 MHz, $CDCl_3$) δ 8.59 (d, J = 2.1 Hz, 1H), 8.02 – 7.95 (m, 2H), 7.79 – 7.66 (m, 2H), 7.53 – 7.42 (m, 2H), 7.44 – 7.37 (m, 1H), 3.53 (s, 2H), 2.42 (br s, 4H), 1.60 (p, J = 5.6 Hz, 4H), 1.45 (q, J = 6.0 Hz, 2H); **^{13}C NMR** (101 MHz, $CDCl_3$) δ 150.5, 139.5, 137.8, 128.9, 128.9, 127.0, 120.2, 60.8, 54.6, 26.1, 24.4; **IR** (film, cm^{-1}) 2934, 2778, 1474, 1340, 1119, 1110, 773, 740; **HRMS:** m/z calculated for $C_{17}H_{21}N_2$ $[M+H]^+$ 253.1699; found 253.1703.

5-(piperidin-1-ylmethyl)-2-(m-tolyl)pyrimidine (**1aq**)

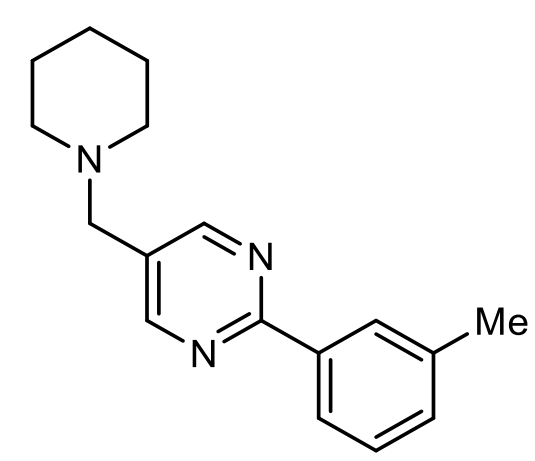

Prepared by general procedure **2.5.1.1.** using piperidine (0.40 mL, 4.0 mmol, 1.0 equiv), 2-(3-methylphenyl)pyrimidine-5-carbaldehyde (950 mg, 4.8 mmol, 1.2 equiv), and sodium triacetoxyborohydride (1.7 g, 8.0 mmol, 2.0 equiv) in tetrahydrofuran. Purification by automated flash column chromatography (0–40% acetone in 1:1 petroleum ether/dichloromethane) gave the title compound as a yellow solid (390 mg, 1.5 mmol, 38% yield). **^{1}H NMR** (500 MHz, $CDCl_3$) δ 8.73 (d, J = 0.6 Hz, 2H), 8.25 (td, J = 1.8, 1.0 Hz, 1H), 8.24 – 8.21 (m, 1H), 7.38 (t, J = 7.6 Hz, 1H), 7.32 – 7.27 (m, 1H), 3.50 (d, J = 0.8 Hz, 2H), 2.45 (s, 3H), 2.41 (s, 4H), 1.59 (p, J = 5.6 Hz, 4H), 1.45 (q, J = 6.1 Hz, 2H); **^{13}C NMR** (126 MHz, $CDCl_3$) δ 163.9, 158.0, 138.4, 137.6, 131.5, 129.3, 128.7, 128.7, 125.3, 58.5, 54.5, 26.1, 24.3, 21.6; **IR** (film, cm^{-1}): 2936, 2854, 2801, 1590, 1551, 1422, 1396, 1342, 1299, 1252, 1107, 1094, 993; **HRMS:** m/z calculated for $C_{17}H_{22}N_3$ $[M + H]^+$ 268.1808; found 268.1807.

2-phenyl-4-(piperidin-1-ylmethyl)thiazole (**1ar**)

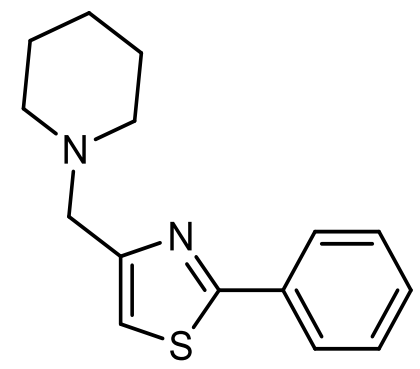

Prepared by general procedure **2.5.1.1.** piperidine (0.40 mL, 4.0 mmol, 1.0 equiv), 2-phenylthiazole-4-carbaldehyde (908 mg, 4.8 mmol, 1.2 equiv), and sodium triacetoxyborohydride (1.7 g, 8.0 mmol, 2.0 equiv) in tetrahydrofuran. Purification by automated flash column chromatography (0%–20% acetone in 1:1 petroleum ether/dichloromethane) gave the title compound as a yellow oil (357 mg, 1.4 mmol, 34% yield). **^{1}H NMR** (500 MHz, $CDCl_3$) δ 7.97 – 7.93 (m, 2H), 7.46 – 7.37 (m, 3H), 7.13 (s, 1H), 3.72 (d, *J* = 0.9 Hz, 2H), 2.61 – 2.42 (m, 4H), 1.66 – 1.58 (m, 5H), 1.45 (q, *J* = 6.3 Hz, 2H); **^{13}C NMR** (126 MHz, $CDCl_3$) δ 167.7, 155.5, 134.0, 129.9, 129.0, 126.7, 115.8, 59.3, 54.8, 26.1, 24.4; **IR** (film, cm^{-1}): 2853, 2759, 1517, 1501, 1459, 1371, 1265, 1154, 1040, 938, 790; **HRMS:** m/z calculated for $C_{15}H_{19}N_2S$ $[M + H]^+$ 259.1264; found 259.1267.

N-(2,4-dimethoxybenzyl)-*N*-methyl-1-(3-(trifluoromethyl)phenyl)methanamine (**1as**)

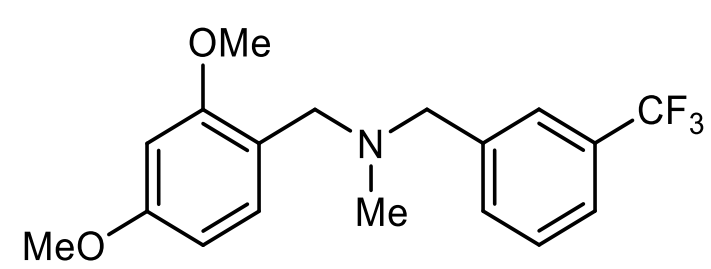

To an oven-dried round-bottom flask charged with a stirrer bar was added 2,4-dimethoxybenzaldehyde (8.31 g, 50 mmol, 1.0 equiv) and anhydrous methanol (100 mL) under nitrogen. The solution was cooled to 0 ˚C and methylamine (33 wt. % in absolute ethanol, 24.9 mL, 200 mmol, 4.0 equiv) was added. The reaction was stirred for 1 hour at room temperature and then cooled to 0 ˚C. Sodium borohydride (2.27 g, 60 mmol, 1.2 equiv) was added portion-wise and the reaction was stirred at room temperature for 12 hours. The reaction was acidified with saturated aqueous ammonium chloride (50 mL) and the methanol was removed *in vacuo*. The crude mixture was basified with aqueous sodium hydroxide (10 wt. %, 60 mL) and extracted with ethyl acetate (5 × 50 mL). The combined organic layers were dried over anhydrous sodium sulfate, filtered and concentrated *in vacuo* to afford 1-(2,4-dimethoxyphenyl)-*N*-methylmethanamine as a pale-yellow solid which was used without further purification. To an oven-dried round-bottom

flask charged with a stirrer bar was added 1-(2,4-dimethoxyphenyl)-*N*-methylmethanamine (1.09 g, 6.0 mmol, 1.0 equiv) and anhydrous sodium sulfate (426 mg, 3.0 mmol, 0.50 equiv). The flask was sealed and evacuated-refilled with nitrogen (3 cycles). Anhydrous tetrahydrofuran (20 mL) was added followed by 3-(trifluoromethyl)benzaldehyde (0.98 mL, 7.2 mmol, 1.2 equiv) and acetic acid (0.035 mL, 0.60 mmol, 10 mol%). The reaction was stirred for 1 hour at room temperature and then sodium triacetoxyborohydride (2.53 g, 12 mmol, 2.0 equiv) was added. The reaction was stirred overnight (16 hours) at room temperature and then aqueous sodium hydroxide (10 wt. %, 20 mL) was added. The layers were separated and the aqueous phase was extracted with ethyl acetate (3 × 20 mL). The combined organic layers were washed with brine (50 mL), dried over anhydrous sodium sulfate, and concentrated *in vacuo*. Purification by flash column chromatography (10% acetone in petroleum ether) gave the title compound as a colourless oil (1.75 g, 5.2 mmol, 86% yield). **^{1}H NMR** (400 MHz, $CDCl_3$) δ 7.68 (s, 1H), 7.59 – 7.55 (m, 1H), 7.53 – 7.49 (m, 1H), 7.43 (tt, *J* = 7.7, 0.8 Hz, 1H), 7.31 – 7.24 (m, 1H), 6.54 – 6.46 (m, 2H), 3.82 (s, 3H), 3.80 (s, 3H), 3.60 (s, 2H), 3.53 (s, 2H), 2.23 (s, 3H); **^{13}C NMR** (176 MHz, $CDCl_3$) δ 160.2, 159.0, 140.9, 132.3, 131.4, 130.6 (q, *J* = 31.9 Hz), 128.6, 125.6 (q, *J* = 3.8 Hz), 124.5 (q, *J* = 271.3 Hz), 123.7 (q, *J* = 3.9 Hz), 119.3, 104.1, 98.6, 61.6, 55.40, 55.39, 55.1, 42.4; **^{19}F NMR** (376 MHz, $CDCl_3$) δ –62.4; **IR** (film, cm^{-1}): 2838, 1508, 1327, 1292, 1208, 1157, 1116, 1036, 921, 833, 796; **HRMS:** m/z calculated for $C_{18}H_{21}F_3NO_2$ $[M+H]^+$ 340.1524; found 340.1520.

N-(3,5-difluorobenzyl)-1-(2,4-dimethoxyphenyl)-*N*-methylmethanamine (**1at**)

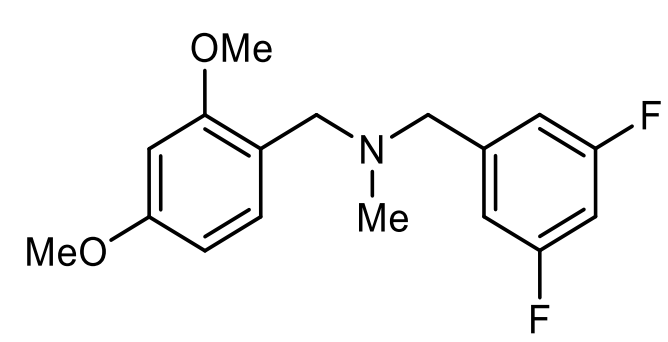

To an oven-dried round-bottom flask charged with a stirrer bar was added 2,4-dimethoxybenzaldehyde (8.31 g, 50 mmol, 1.0 equiv) and anhydrous methanol (100 mL) under nitrogen. The solution was cooled to 0 ˚C and methylamine (33 wt. % in absolute ethanol, 24.9 mL, 200 mmol, 4.0 equiv) was added. The reaction was stirred for 1 hour at room temperature and then cooled to 0 ˚C. Sodium borohydride (2.27 g, 60 mmol, 1.2 equiv) was added portion-wise and the reaction was stirred at room temperature for 12 hours. The reaction was acidified with saturated aqueous ammonium chloride (50 mL) and the methanol was removed *in vacuo*. The crude mixture was basified with aqueous sodium hydroxide (10 wt. %, 60 mL) and extracted with

ethyl acetate (5 × 50 mL). The combined organic layers were dried over anhydrous sodium sulfate, filtered and concentrated *in vacuo* to afford 1-(2,4-dimethoxyphenyl)-*N*-methylmethanamine as a pale-yellow solid which was used without further purification. To an oven-dried round-bottom flask charged with a stirrer bar was added 1-(2,4-dimethoxyphenyl)-*N*-methylmethanamine (906 mg, 5.0 mmol, 1.0 equiv) and anhydrous sodium sulfate (355 mg, 2.5 mmol, 0.50 equiv). The flask was sealed and evacuated-refilled with nitrogen (3 cycles). Anhydrous tetrahydrofuran (20 mL) was added followed by 3,5-difluorobenzaldehyde (0.66 mL, 6.0 mmol, 1.2 equiv) and acetic acid (0.029 mL, 0.50 mmol, 10 mol%). The reaction was stirred for 1 hour at room temperature and then sodium triacetoxyborohydride (2.11 g, 10 mmol, 2.0 equiv) was added. The reaction was stirred overnight (16 hours) at room temperature and then aqueous sodium hydroxide (10 wt. %, 20 mL) was added. The layers were separated and the aqueous phase was extracted with ethyl acetate (3 × 20 mL). The combined organic layers were washed with brine (50 mL), dried over anhydrous sodium sulfate, and concentrated *in vacuo*. Purification by flash column chromatography (10% acetone in petroleum ether) gave the title compound as a colourless oil (1.19 g, 3.9 mmol, 78% yield). **^{1}H NMR** (400 MHz, $CDCl_3$) δ 7.26 (dd, *J* = 8.0, 0.7 Hz, 1H), 7.00 – 6.90 (m, 2H), 6.68 (tt, *J* = 9.0, 2.4 Hz, 1H), 6.53 – 6.44 (m, 2H), 3.87 – 3.77 (m, 6H), 3.51 (s, 2H), 3.49 (s, 2H), 2.21 (s, 3H); **^{13}C NMR** (176 MHz, $CDCl_3$) δ 163.1 (dd, *J* = 247.4, 12.7 Hz), 160.2, 159.0, 144.4 (t, *J* = 8.7 Hz), 131.3, 119.2, 111.3 (dd, *J* = 20.4, 4.4 Hz), 104.0, 102.1 (t, *J* = 25.5 Hz), 98.5, 61.2 (t, *J* = 2.2 Hz), 55.39, 55.37, 55.1, 42.4; **^{19}F NMR** (376 MHz, $CDCl_3$) δ –110.9; **IR** (film, cm^{-1}): 2838, 1590, 1456, 1292, 1208, 1113, 1036, 960, 833, 787; **HRMS:** m/z calculated for $C_{17}H_{20}F_2NO_2$ $[M+H]^+$ 308.1462; found 308.1451.

N-(2,4-dimethoxybenzyl)-*N*-methyl-1-(6-(trifluoromethyl)pyridin-3-yl)methanamine (**1au**)

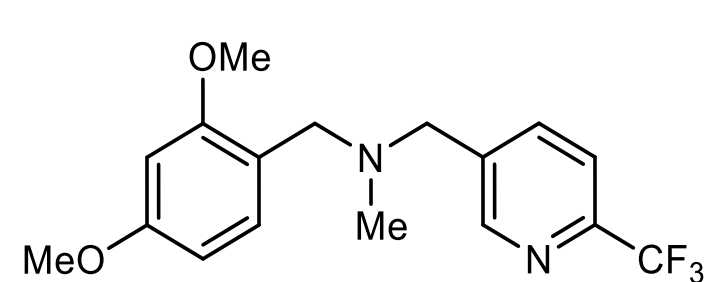

To an oven-dried round-bottom flask charged with a stirrer bar was added 2,4-dimethoxybenzaldehyde (8.31 g, 50 mmol, 1.0 equiv) and anhydrous methanol (100 mL) under nitrogen. The solution was cooled to 0 ˚C and methylamine (33 wt. % in absolute ethanol, 24.9 mL, 200 mmol, 4.0 equiv) was added. The reaction was stirred for 1 hour at room temperature and then cooled to 0 ˚C. Sodium borohydride (2.27 g, 60 mmol, 1.2 equiv) was added portion-

wise and the reaction was stirred at room temperature for 12 hours. The reaction was acidified with saturated aqueous ammonium chloride (50 mL) and the methanol was removed *in vacuo*. The crude mixture was basified with aqueous sodium hydroxide (10 wt. %, 60 mL) and extracted with ethyl acetate (5 × 50 mL). The combined organic layers were dried over anhydrous sodium sulfate, filtered and concentrated *in vacuo* to afford 1-(2,4-dimethoxyphenyl)-*N*-methylmethanamine as a pale-yellow solid which was used without further purification. To an oven-dried round-bottom flask charged with a stirrer bar was added 1-(2,4-dimethoxyphenyl)-*N*-methylmethanamine (906 mg, 5.0 mmol, 1.0 equiv), 6-(trifluoromethyl)nicotinaldehyde (1.05 g, 6.0 mmol, 1.2 equiv) and anhydrous sodium sulfate (355 mg, 2.5 mmol, 0.50 equiv). The flask was sealed and evacuated-refilled with nitrogen (3 cycles). Anhydrous tetrahydrofuran (20 mL) was added followed by acetic acid (0.029 mL, 0.50 mmol, 10 mol%). The reaction was stirred for 1 hour at room temperature and then sodium triacetoxyborohydride (2.11 g, 10 mmol, 2.0 equiv) was added. The reaction was stirred overnight (16 hours) at room temperature and then aqueous sodium hydroxide (10 wt. %, 20 mL) was added. The layers were separated and the aqueous phase was extracted with ethyl acetate (3 × 20 mL). The combined organic layers were washed with brine (50 mL), dried over anhydrous sodium sulfate, and concentrated *in vacuo*. Purification by flash column chromatography (10% acetone in petroleum ether) gave the title compound as a colourless oil (1.48 g, 4.4 mmol, 87% yield). **^{1}H NMR** (700 MHz, $CDCl_3$) δ 8.67 (s, 1H), 7.87 (d, *J* = 8.0 Hz, 1H), 7.61 (d, *J* = 8.0 Hz, 1H), 7.22 (d, *J* = 8.2 Hz, 1H), 6.46 (d, *J* = 8.2, 1H), 6.44 (s, 1H), 3.79 (s, 3H), 3.77 (s, 3H), 3.59 (s, 2H), 3.50 (s, 2H), 2.22 (s, 3H); **^{13}C NMR** (176 MHz, $CDCl_3$) δ 160.2, 158.9, 150.5, 146.8 (q, *J* = 34.5 Hz), 138.9 (app. d, *J* = 1.2 Hz), 137.6, 131.3, 121.8 (q, *J* = 273.3 Hz), 120.0 (q, *J* = 2.8 Hz), 118.8, 104.1, 98.5, 58.6, 55.37, 55.35, 55.1, 42.4; **^{19}F NMR** (376 MHz, $CDCl_3$) δ –67.7; **IR** (film, cm^{-1}): 2840, 1612, 1588, 1508, 1459, 1333, 1292, 1210, 1156, 1131, 1085, 1034, 911, 831, 731; **HRMS:** m/z calculated for $C_{17}H_{20}F_3N_2O_2$ $[M+H]^+$ 341.1477; found 341.1465.

N-(3,5-bis(trifluoromethyl)benzyl)-1-(4-methoxyphenyl)-*N*-methylmethanamine (**1av**)

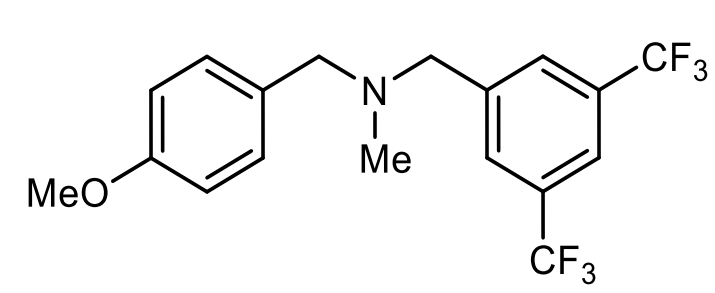

To an oven-dried round-bottom flask charged with a stirrer bar was added methylamine (33 wt. % in absolute ethanol, 1.24 mL, 10 mmol, 1.0 equiv), 4-methoxybenzaldehyde (1.40 mL, 12 mmol, 1.2 equiv) and anhydrous methanol (20 mL) under nitrogen. The reaction was stirred at room temperature for 30 minutes and then cooled to 0 ˚C. Sodium borohydride (757 mg, 20 mmol, 2.0 equiv) was added portion-wise and the reaction was stirred at room temperature for 1 hour. Aqueous sodium hydroxide (10 wt. %, 20 mL) was then added and the methanol was removed *in vacuo*. The crude mixture was extracted with ethyl acetate (5 × 20 mL) and the combined organic layers were dried over anhydrous sodium sulfate, filtered and concentrated *in vacuo* to afford 1-(4-methoxyphenyl)-*N*-methylmethanamine as a pale-yellow oil which was used without further purification. To an oven-dried flask charged with a stirrer bar was added potassium carbonate (829 mg, 6.0 mmol, 1.2 equiv). The flask was sealed and evacuated-refilled with nitrogen (3 cycles). 1-(4-methoxyphenyl)-*N*-methylmethanamine (756 mg, 5.0 mmol, 1.0 equiv), 1-(bromomethyl)-3,5-bis(trifluoromethyl)benzene (0.92 mL, 5.0 mmol, 1.0 equiv) and acetonitrile (5.0 mL) were then added and the reaction was stirred for 24 hours at room temperature. The solvent was removed *in vacuo* and the crude residue was redissolved in ethyl acetate (10 mL) and washed with water (10 mL). The aqueous phase was extracted with ethyl acetate (2 × 10 mL) and the combined organic layers were dried over anhydrous sodium sulfate, filtered, and concentrated *in vacuo*. Purification by flash column chromatography (10% acetone in petroleum ether) gave the title compound as a colourless oil (1.17 g, 3.1 mmol, 62% yield). **^{1}H NMR** (500 MHz, $CDCl_3$) δ 7.85 (s, 2H), 7.78 (s, 1H), 7.32 – 7.24 (m, 2H), 6.90 (d, *J* = 8.6 Hz, 2H), 3.82 (s, 3H), 3.61 (s, 2H), 3.52 (s, 2H), 2.22 (s, 3H); **^{13}C NMR** (176 MHz, $CDCl_3$) δ 159.1, 142.7, 131.6 (q, *J* = 33.1 Hz), 130.7, 130.2, 128.8 (d, *J* = 3.7), 123.6 (q, *J* = 272.6 Hz), 121.1 (app. p, *J* = 3.9 Hz), 113.9, 61.6, 60.6, 55.3, 42.4; **^{19}F NMR** (471 MHz, $CDCl_3$) δ –62.8; **IR** (film, cm^{-1}): 2954, 1513, 1387, 1277, 1249, 1169, 1124, 1036, 895, 706, 682; **HRMS:** m/z calculated for $C_{18}H_{18}F_6NO$ $[M+H]^+$ 378.1293; found 378.1281.

3-(5-bromo-1-oxoisoindolin-2-yl)piperidine-2,6-dione

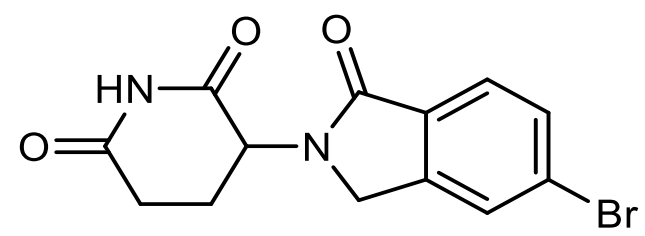

According to a procedure by Hayhow and co-workers,[73] an oven-dried 500 mL round bottom flask was charged with methyl 4-bromo-2-(bromomethyl)benzoate (6.2 g, 20 mmol, 1.0 equiv) and 3-aminopiperidine-2,6-dione hydrochloride (4.9 g, 30 mmol, 1.5 equiv). The flask was equipped with a reflux condenser, evacuated and refilled with nitrogen (3 cycles). Degassed acetonitrile (80 mL) was added via the top of the condenser, followed by *N*,*N*-diisopropylethylamine (11 mL, 60 mmol, 3.0 equiv). The resulting mixture was refluxed for 48 hours. Following this, the mixture was cooled down to 0 °C. The precipitate was filtered and washed with cold acetonitrile (50 mL) followed by diethyl ether (50 mL). The resulting solid was then collected, suspended in water (100 mL) and stirred for 30 minutes at room temperature. The solid was then filtered, washed with water (2 × 50 mL), followed by ethyl acetate (2 × 50 mL). The solid was collected and dried under high vacuum (<1 mbar) to give the title compound as a white blue solid (4.9 g, 15 mmol, 76% yield). Data was in line with previous characterisation.[73] **^{1}H NMR** (500 MHz, DMSO-d6) δ 11.00 (s, 1H), 7.89 (s, 1H), 7.76 – 7.69 (m, 1H), 7.67 (d, *J* = 8.1 Hz, 1H), 5.11 (dd, *J* = 13.3, 5.1 Hz, 1H), 4.47 (d, *J* = 17.6 Hz, 1H), 4.34 (d, *J* = 17.6 Hz, 1H), 3.32 (s, 1H), 2.90 (ddt, *J* = 13.7, 10.1, 5.4 Hz, 1H), 2.60 (ddd, *J* = 17.4, 4.5, 2.3 Hz, 1H), 2.39 (qd, *J* = 13.2, 4.5 Hz, 1H), 2.09 – 1.93 (m, 1H); **^{13}C NMR** (δ 176 MHz, DMSO-d6) δ 172.8, 170.9, 167.2, 144.3, 131.2, 130.9, 126.8, 125.4, 124.8, 51.7, 46.9, 31.2, 22.4.

Note: Thoroughly degassing the reaction mixture seemed to limit the formation of the dark blue dye side-product.

3-(1-oxo-5-(piperidin-4-yl)isoindolin-2-yl)piperidine-2,6-dione hydrochloride

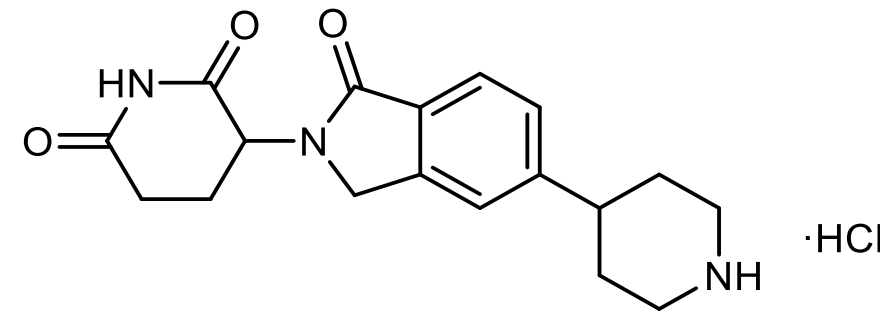

According to a procedure by Kappe, Williams, Eggenweiler and co-workers,[74] an oven-dried 100 mL round bottom flask was charged with 3-(5-bromo-1-oxoisoindolin-2-yl)piperidine-2,6-dione (1.6 g, 5.0 mmol, 1.0 equiv), *N*-Boc 4-bromo-piperidine (4.0 g, 15 mmol, 3.0 equiv), sodium carbonate (1.1 g, 10 mmol, 2.0 equiv), nickel dichloride ethylene glycol dimethyl ether complex (55 mg, 0.25 mmol, 5 mol%), 4,4'-di-tert-butyl-2,2'-dipyridyl (134 mg, 0.50 mmol, 10 mol%) and $[Ir[dF(CF_3)ppy]_2(dtbbpy)]PF_6$ (5.6 mg, 0.005 mmol, 1 mol%). The flask was evacuated and refilled with argon (3 cycles). Following this, degassed 1,2-dimethoxyethane (45 mL) was added followed by degassed *N,N*-dimethylacetamide (5.0 mL), followed by tris(trimethylsilyl)silane (1.5 mL, 5.0 mmol, 1.0 equiv). The flask was sealed with parafilm and placed in an oil bath at 45 °C, equipped with two 40W Kessil A160WE Tuna Blue LED, placed at a 3 cm distance from the flask. The reaction mixture was stirred at 800 rpm at 45 °C and irradiated for 48 hours. After that, the reaction mixture was transferred into a 250 mL round bottom flask and *n*-heptane (150 mL) was added. The reaction was left to precipitate at 4 °C overnight. Following this, the solid was filtered, redissolved in dichloromethane (200 mL) and filtered over a pad of silica. The silica pad was washed with acetone (3 × 50 mL). The filtrate was concentrated *in vacuo* to give 1.3 g of a beige solid. The residue was then redissolved in dichloromethane (300 mL) and hydrogen chloride (4.0 N in 1,4-dioxane, 10 mL, 40 mmol, 8.0 equiv) was added dropwise. The reaction mixture was left to stir for 18 h at room temperature, after which precipitation into a fine white powder was observed. The suspension was concentrated to a minimum of solvent (*c.a* 40 mL) and transferred into two 50 mL falcon tubes. The tubes were centrifugated for 30 seconds, the supernatant liquid was removed via pipette, dichloromethane (20 mL) was added to each tube, and this cycle was repeated twice. At the end of the last cycle, solids were transferred into a 6-dram glass vial and dried under high vacuum (<1 mbar) to give the title compound as a white solid (960 mg, 2.6 mmol, 53% yield over two steps). Data was in line with previous characterisation.[74] **^{1}H NMR** (700 MHz, DMSO-d6) δ 10.99 (s, 1H), 7.89 (d, *J* = 1.7 Hz, 1H), 7.72 (dd, *J* = 8.1, 1.7 Hz, 1H), 7.67 (d, *J* = 8.1 Hz, 1H), 5.11 (dd, *J* = 13.3, 5.2 Hz, 1H), 4.47 (d, *J* = 17.5 Hz, 1H), 4.34 (d, *J* = 17.5 Hz, 1H), 2.90 (ddd, *J* = 17.3, 13.7, 5.4 Hz, 1H), 2.60 (ddd, *J* = 17.3, 4.5, 2.3 Hz, 1H), 2.39 (qd, *J* = 13.3, 4.5 Hz, 1H), 2.01 (dtd, *J* = 12.6, 5.1, 2.1 Hz, 1H); **^{13}C NMR** (176 MHz, DMSO-d6) δ 172.8, 170.9, 167.2, 144.3, 131.2, 130.9, 126.8, 125.4, 124.8, 51.7, 46.9, 31.2, 22.4.

3-(5-(1-benzylpiperidin-4-yl)-1-oxoisoindolin-2-yl)piperidine-2,6-dione (NVP-DKY709)

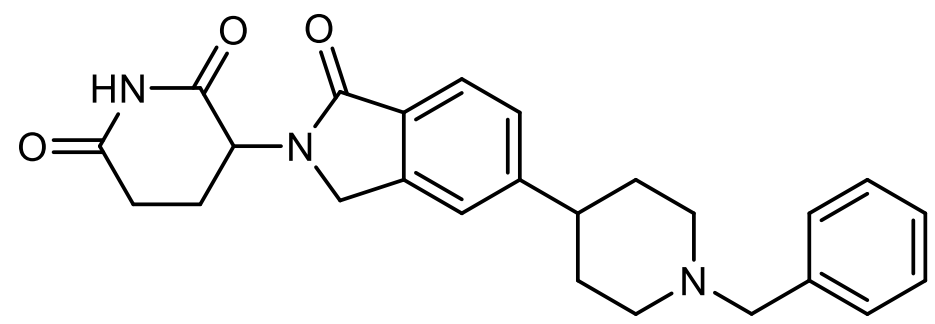

To a suspension of 3-(1-oxo-5-(piperidin-4-yl)isoindolin-2-yl)piperidine-2,6-dione hydrochloride (500 mg, 1.4 mmol, 1.0 equiv) in *N*,*N*-dimethylformamide (4.0 mL) was added triethylamine (0.52 mL, 3.7 mmol, 2.6 equiv). The reaction was stirred at room temperature for 15 min and benzyl bromide (0.19 mL, 1.6 mmol, 1.2 equiv) was then added. The reaction mixture was stirred at room temperature over 18 hours. Following this, the reaction mixture was concentrated and purified by manual column chromatography (silica gel, 5–20% triethylamine in ethyl acetate) to give the title compound as a beige solid (330 mg, 0.8 mmol, 56% yield). Data was in line with previous characterisation.[75] **^{1}H NMR** (700 MHz, DMSO-d6) δ 10.97 (s, 1H), 7.63 (d, *J* = 7.9 Hz, 1H), 7.49 (s, 1H), 7.40 (dd, *J* = 8.0, 1.4 Hz, 1H), 7.33 (d, *J* = 4.4 Hz, 4H), 7.25 (dt, *J* = 8.8, 4.2 Hz, 1H), 5.10 (dd, *J* = 13.3, 5.2 Hz, 1H), 4.41 (d, *J* = 17.0 Hz, 1H), 4.28 (d, *J* = 17.0 Hz, 1H), 3.32 (s, 1H), 2.95 – 2.87 (m, 3H), 2.67 – 2.56 (m, 2H), 2.38 (qd, *J* = 13.2, 4.5 Hz, 1H), 2.06 (td, *J* = 11.7, 2.5 Hz, 2H), 1.98 (dtd, *J* = 12.7, 5.4, 2.3 Hz, 1H), 1.78 – 1.74 (m, 2H), 1.70 (qd, *J* = 12.3, 3.7 Hz, 2H); **^{13}C NMR** (δ 176 MHz, DMSO-d6) δ 172.9, 171.1, 168.0, 150.6, 142.5, 138.5, 129.7, 128.8, 128.1, 126.9, 126.8, 122.9, 121.7, 62.4, 53.5, 51.5, 47.1, 42.2, 33.1, 33.0, 31.2, 22.5.

3-(5-(1-benzylpiperidin-4-yl)-1-oxoisoindolin-2-yl)-1-(4-methoxybenzyl)piperidine-2,6-dione

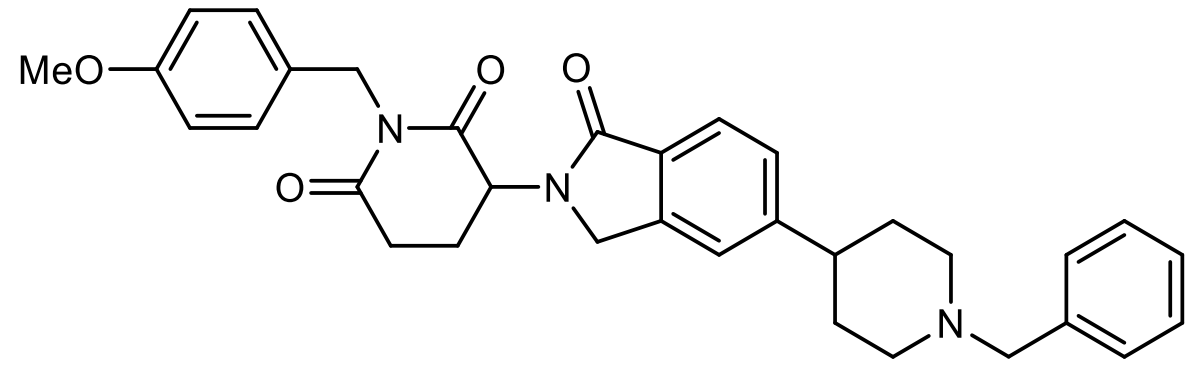

To a solution of NVP-DKY709 (250 mg, 0.60 mmol, 1.0 equiv) in THF (12 mL) was added 4-methoxybenzyl alcohol (0.11 mL, 0.90 mmol, 1.5 equiv), and triphenylphosphine (236 mg, 0.90 mmol, 1.5 equiv) at 0 °C. Following this, diisopropyl azodicarboxylate (0.18 mL, 0.90 mmol, 1.5 equiv) was added dropwise and the resulting reaction mixture was stirred at 55 °C for 18 hours.

The reaction mixture was then concentrated *in vacuo* and then purified by by automated flash column chromatography (5–30% acetone in dichloromethane) to give the title compound as a white solid (198 mg, 0.39 mmol, 65% yield). **^{1}H NMR** (500 MHz, $CDCl_3$) δ 7.80 (dd, *J* = 7.9, 0.7 Hz, 1H), 7.38 – 7.28 (m, 9H), 7.28 – 7.24 (m, 2H), 6.83 – 6.78 (m, 2H), 5.17 (dd, *J* = 13.5, 5.1 Hz, 1H), 4.89 (s, 2H), 4.38 (d, *J* = 15.8 Hz, 1H), 4.26 (d, *J* = 15.8 Hz, 1H), 3.77 (s, 3H), 3.56 (s, 2H), 3.06 – 3.01 (m, 2H), 2.97 (ddd, *J* = 17.7, 4.6, 2.4 Hz, 1H), 2.84 (ddd, *J* = 17.7, 13.5, 5.4 Hz, 1H), 2.65 – 2.56 (m, 1H), 2.27 (qd, *J* = 13.4, 4.6 Hz, 1H), 2.12 (dddd, *J* = 19.5, 11.5, 6.1, 3.8 Hz, 3H), 1.85 – 1.77 (m, 4H); **^{13}C NMR** (126 MHz, $CDCl_3$) δ 171.1, 170.1, 169.5, 159.2, 151.4, 142.0, 138.5, 130.8, 129.8, 129.3, 129.2, 128.3, 127.5, 127.1, 124.2, 121.3, 113.9, 63.5, 55.4, 54.2, 54.2, 52.6, 47.1, 43.3, 43.2, 33.6, 33.6, 32.3, 22.8; **IR** (film, cm^{-1}): 2937, 1727, 1678, 1619, 1514, 1454, 1367, 1340, 1247, 1165, 1109, 1032, 738; **HRMS:** m/z calculated for $C_{33}H_{36}N_3O_4$ $[M + H]^+$ 538.2700; found 538.2681.

(4-Benzylmorpholino-4-ium)methyl)trifluoroborate (**4a**)

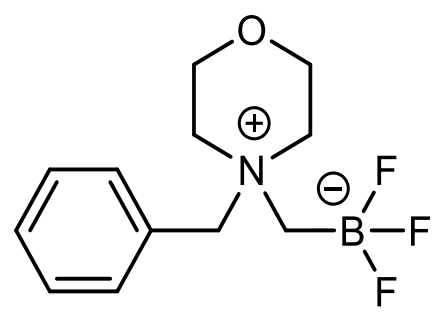

Prepared using general procedure **2.5.2.2. (method II)** using *N*-benzyl morpholine (**1a**) (1.8 g, 10 mmol, 1.0 equiv) and potassium (bromomethyl)trifluoroborate (3.0 g, 15 mmol, 1.5 equiv) in acetonitrile (10 mL). The reaction mixture was concentrated under reduced pressure before the addition of HCl (4 M in 1,4-dioxane, 2.5 mL, 10 mmol, 1.0 equiv). The reaction was then extracted with dichloromethane (3 x 20 mL) before being concentrated under reduced pressure. The resultant solid was then recrystalised from methanol to give the title compound as a white solid (2.5 g, 9.6 mmol, 96% yield). **^{1}H NMR** (500 MHz, DMSO-d6) δ 7.63 – 7.56 (m, 2H), 7.52 – 7.47 (m, 3H), 4.62 (s, 2H), 4.02 – 3.84 (m, 4H), 3.38 – 3.33 (m, 2H), 3.29 – 3.20 (m, 2H), 2.32 (br. q, *J* = 4.7 Hz, 2H); **^{13}C NMR** (126 MHz, DMSO-d6) δ 133.8, 130.4, 129.1, 128.7, 66.5, 60.6, 59.2, 50.0 (observed by HSQC); **^{19}F NMR** (471 MHz, DMSO-d6) δ −134.1; **IR** (film, cm^{-1}): 2902, 1485, 1456, 1434, 1419, 1392, 1362, 1327, 1311, 1287, 1258, 1216, 1157, 1119, 1088, 1040, 1024, 1009, 1000, 974, 946, 928, 912, 885, 853, 787, 763, 739, 712, 699; **HRMS:** m/z calculated for $C_{12}H_{17}BF_2NO$ $[M-F]^+$ 240.1366; found 240.1367.

((1-Benzylpiperidin-1-ium-1-yl)methyl)trifluoroborate (**4b**)

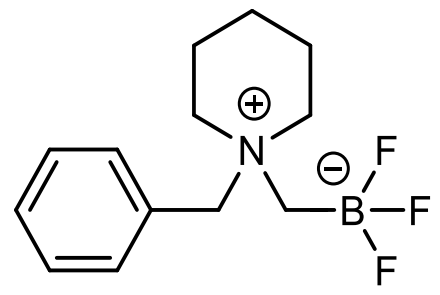

Prepared by general procedure **2.5.2.2 (method I)** using1-benzylpiperidine (**1b**) (184 µL, 1.0 mmol, 1.0 equiv) and potassium (bromomethyl)trifluoroborate (301 mg, 1.5 mmol, 1.5 equiv) in acetonitrile (1.0 mL). The title compound was obtained as a white solid (245 mg, 0.95 mmol, 95% yield). **^{1}H NMR** (400 MHz, DMSO-d6) δ 7.62 – 7.54 (m, 2H), 7.50 – 7.45 (m, 3H), 4.52 (s, 2H), 3.25 (dt, *J* = 12.5, 4.6 Hz, 2H), 3.13 (ddd, *J* = 12.9, 9.4, 4.1 Hz, 2H), 2.16 (q, *J* = 4.7 Hz, 2H), 1.97 – 1.73 (m, 4H), 1.66 – 1.53 (m, 1H), 1.48 – 1.34 (m, 1H); **^{13}C NMR** (101 MHz, DMSO-d6) δ 133.2, 129.7, 128.8, 128.6, 65.1, 59.8, 50.3, 20.9, 19.6; **^{19}F NMR** (376 MHz, DMSO-d6) δ −134.2; **IR** (film, cm^{-1}): 2947, 1697, 1527, 1480, 1547, 1437, 1383, 1362, 1348, 1323, 1298, 1278, 1214, 1174, 1103, 1052, 1035, 1014,

998, 976, 938, 928, 908, 885, 848, 828, 782, 760, 735, 711, 700; **HRMS:** m/z calculated for $C_{13}H_{19}BF_2N$ [M−F]$^+$ 238.1573; found 238.1576.

((1-Benzyl-4-(*tert*-butoxycarbonyl)piperazin-1-ium-1-yl)methyl)trifluoroborate **(4c)**

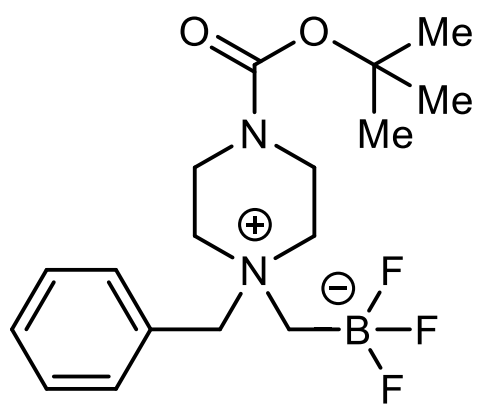

Prepared using general procedure **2.5.2.2. (method I)** using *tert*-butyl 4-benzylpiperazine-1-carboxylate (**1c**) (552 mg, 2.0 mmol, 1.0 equiv) and potassium (bromomethyl)trifluoroborate (301 mg, 3.0 mmol, 1.5 equiv) in acetonitrile (2.0 mL). The title compound was obtained as a white solid (688 mg, 1.9 mmol, 96% yield). Data was in line with previous characterisation.[32] **^{1}H NMR** (500 MHz, DMSO-d6) δ 7.61 – 7.57 (m, 2H), 7.52 – 7.46 (m, 3H), 4.59 (s, 2H), 3.85 (br dt, *J* = 15.0, 4.0 Hz, 2H), 3.60 – 3.39 (m, 2H), 3.31 – 3.20 (m, 4H), 2.23 (br q, *J* = 4.7 Hz, 2H), 1.41 (s, 9H); **^{13}C NMR** (126 MHz, DMSO-d6) δ 153.5, 133.3, 129.9, 128.7, 128.3, 79.7, 65.3, 58.4, 48.6, 37.9, 27.9; **^{19}F NMR** (471 MHz, DMSO-d6) δ −134.0; **IR** (film, cm^{-1}): 2972, 1694, 1471, 1451, 1417, 1395, 1361, 1325, 1295, 1275, 1262, 1243, 1171, 1140, 1107, 1086, 1073, 1053, 1018, 997, 979, 963, 930, 911, 891, 861, 840, 829, 787, 776, 761, 739, 714, 700; **HRMS:** m/z calculated for $C_{17}H_{26}BF_3N_2NaO_2$ [M + Na]$^+$ 381.1932; found 381.1938.

((1-Benzyl-4-tosylpiperazin-1-ium-1-yl)methyl)trifluoroborate (**4d**)

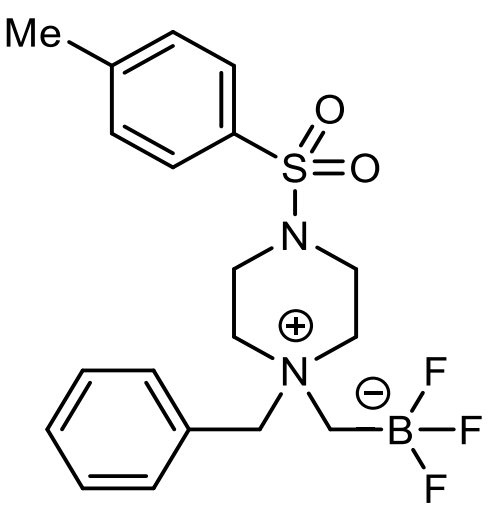

Prepared by general procedure **2.5.2.1. (method I)** using 1-benzyl-4-tosylpiperazine (**1d**) (330 mg, 1.0 mmol, 1.0 equiv) and 2-(Bromomethyl)-4,4,5,5,-tetramethyl-1,3,2-dioxaborolane (0.25 mL, 1.5 mmol, 1.2 equiv) in acetonitrile (4.0 mL). Fluorination was performed with aqueous potassium hydrogen fluoride (3 M, 0.84 mL, 2.5 mmol, 2.5 equiv) and HCl (4 M in 1,4-dioxane, 0.25 mL, 1.0 mmol, 1.0 equiv) in 4:1 acetonitrile/water (0.10 M). The title compound was obtained as a white solid (404 mg, 0.98 mmol, 98% yield). **^{1}H NMR** (500 MHz, DMSO-d6) δ 7.69 – 7.65 (m, 2H), 7.57 – 7.53 (m, 2H),

7.52 – 7.46 (m, 5H), 4.58 (s, 2H), 3.72 – 3.65 (m, 2H), 3.49 – 3.39 (m, 2H), 3.37 – 3.29 (m, 2H), 3.04 – 2.93 (m, 2H), 2.43 (s, 3H), 2.03 (br q, J = 4.6 Hz, 2H); **^{13}C NMR** (126 MHz, DMSO-d6) δ 144.7, 133.8, 132.7, 130.6, 130.5, 129.2, 128.5, 128.0, 66.9, 58.6, 48.7, 40.4, 21.6; **^{19}F NMR** δ (471 MHz, $CDCl_3$) δ −129.4; **IR** (film, cm^{-1}): 2867, 1486, 1459, 1450, 1354, 1335, 1306, 1267, 1187, 1165, 1090, 1044, 1027, 1015, 984, 966, 932, 916, 883, 819, 743, 727, 714, 702; **HRMS:** m/z calculated for $C_{19}H_{24}BF_2N_2O_2S$ [M−F]$^+$ 393.1614; found 393.1612.

((1-benzyl-3,5-dimethylpiperidin-1-ium-1-yl)methyl)trifluoroborate **(4e)**

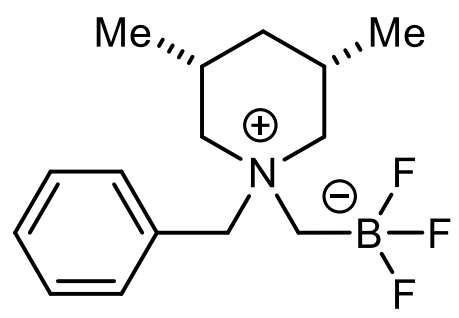

Prepared by general procedure **2.5.2.2. (method I)** using 1-benzyl-3,5-dimethylpiperidine (**1e**) (813 mg, 4.0 mmol, 1.0 equiv) and potassium (bromomethyl)trifluoroborate (1.2 g, 6.0 mmol, 1.5 equiv) in acetonitrile (4.0 mL). The title compound was obtained as a white solid (702 mg, 2.5 mmol, 62% yield, 4:1 d.r.); **^{1}H NMR** (700 MHz, $CDCl_3$) δ 7.57 – 7.40 (m, 5H), 4.54 (s, 2H, major), 4.51 (s, 2H, minor), 3.39 – 3.34 (m, 2H), 2.77 (t, J = 13.1 Hz, 2H, minor), 2.46 (d, J = 4.7 Hz, 2H, major), 2.42 (t, J = 12.0 Hz, 2H, major), 2.31 (q, J = 4.8 Hz, 2H, minor), 2.25 (qdd, J = 12.3, 9.9, 3.6 Hz, 2H, major), 2.02 – 1.98 (m, 1H, minor), 1.85 (dt, J = 13.4, 1.9 Hz, 1H, major), 1.00 (d, J = 6.5 Hz, 6H, minor), 0.93 (d, J = 6.5 Hz, 6H, major), 0.66 (q, J = 12.6 Hz, 1H, major); **^{13}C NMR** (176 MHz, $CDCl_3$) δ 133.6 (major), 132.8 (minor), 130.5 (minor), 130.4 (major), 129.3 (minor), 129.1 (major), 128.2 (minor), 127.8 (major), 70.1 (major), 66.4 (major), 66.0 (minor), 60.3 (minor), 39.7 (major), 39.5 (minor), 26.7 (minor), 26.0 (major), 18.8 (major), 18.7 (minor); **^{19}F NMR** (376 MHz, $CDCl_3$) δ −136.8 (minor), −138.7 (major); **IR** (neat, cm^{-1}) 2969, 1456, 1045, 991, 977, 889, 706, 609; **HRMS:** m/z calculated for $C_{15}H_{23}BF_2N$ [M−F]$^+$ 266.1886; found 266.1891.

((8-benzyl-1,4-dioxa-8-azaspiro[4.5]decan-8-ium-8-yl)methyl)trifluoroborate (**4f**)

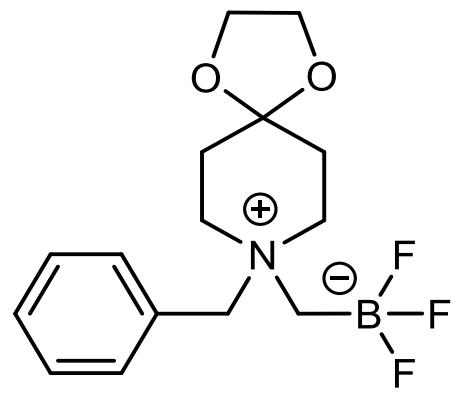

Prepared using general procedure **2.5.2.2. (method II)** using 8-benzyl-1,4-dioxa-8-azaspiro[4.5]decane (**1f)** (500 mg, 2.1 mmol, 1.0 equiv) and potassium (bromomethyl)trifluoroborate (1.1 g, 5.4 mmol, 2.5 equiv) in acetonitrile (2.1 mL). The title compound was obtained as a white solid (560 mg, 1.8 mmol, 84% yield). **^{1}H NMR** (700 MHz, Acetone-d6) δ 7.73 – 7.70 (m, 2H), 7.52 – 7.47 (m, 3H), 4.71 (s, 2H), 3.99 (d, *J* = 1.7 Hz, 4H), 3.54 – 3.50 (m, 2H), 3.40 (td, *J* = 12.2, 3.5 Hz, 2H), 2.34 (d, *J* = 6.2 Hz, 2H), 2.23 (ddd, *J* = 15.3, 11.6, 4.2 Hz, 2H), 1.98 – 1.91 (m, 2H); **^{13}C NMR** (176 MHz, Acetone-d6) δ 134.4, 130.9, 129.9, 129.6, 104.9, 65.5, 65.2, 59.6; **^{19}F NMR** (376 MHz, Acetone-d6) δ −138.0 (dd, *J* = 96.2, 44.2 Hz); **IR** (film, cm^{-1}): 2959, 2891, 1459, 1168, 1118, 1026, 1009, 971, 899, 874; **HRMS:** m/z calculated for $C_{15}H_{21}BF_2NO_2$ [M−F]$^+$ 296.1628; found 296.1635.

((1-benzyl-4-hydroxy-4-phenylpiperidin-1-ium-1-yl)methyl)trifluoroborate (**4g**)

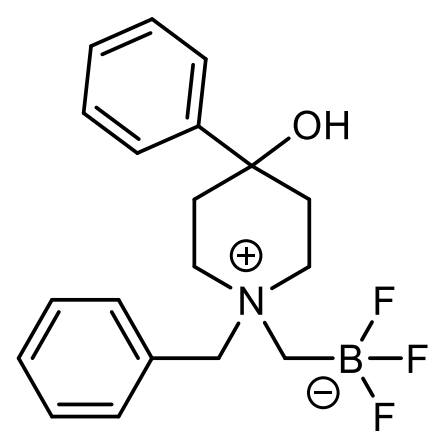

Prepared using general procedure **2.5.2.2. (method II)** using 1-benzyl-4-phenylpiperidin-4-ol (**1g)** (401 mg, 1.5 mmol, 1.0 equiv) and potassium (bromomethyl)trifluoroborate (753 mg, 3.8 mmol, 2.5 equiv) in acetonitrile (1.5 mL). The title compound was obtained as a white solid (412 mg, 1.2 mmol, 80% yield, 8:1 d.r.). **^{1}H NMR** (500 MHz, Acetone-d6) δ 7.77 – 7.74 (m, 2H, major), 7.75 – 7.71 (m, 2H, minor), 7.61 – 7.57 (m, 2H), 7.54 – 7.49 (m, 3H), 7.43 – 7.40 (m, 2H, minor), 7.40 – 7.35 (m, 2H, major), 7.33 – 7.29 (m, 1H, minor), 7.27 (ddt, *J* = 7.9, 6.7, 1.2 Hz, 1H, major), 4.82 (s, 2H, minor), 4.76 (s, 2H, major), 4.63 – 4.60 (m, 1H, minor), 4.42 (d, *J* = 1.0 Hz, 1H, major), 3.89 (td, *J* = 13.6, 3.2 Hz, 1H, minor), 3.72 (td, *J* = 12.7, 3.1 Hz, 2H, major), 3.47 – 3.40 (m, 2H), 2.65 – 2.57 (m, 2H), 2.45 (d, *J* = 5.3 Hz, 2H, major), 2.26 (d, *J* = 4.8 Hz, 2H, minor), 1.88 (d, *J* = 15.1 Hz, 2H); **^{13}C NMR** (126 MHz, Acetone-d6) δ 148.3, 134.6 (d, *J* = 1.0 Hz, major), 134.2 (minor), 130.9 (major), 130.8 (minor),

130.0, 129.65 (minor), 129.60 (major), 129.24 (major), 129.21 (minor), 128.19 (minor), 128.11 (major), 125.9 (minor), 125.6 (major), 69.6 (minor), 69.2 (major), 57.3, 34.05 (minor), 34.00 (major); **^{19}F NMR** (471 MHz, Acetone-d6) δ −136.5 (dd, *J* = 89.4, 36.9 Hz, minor), −137.9 (dd, *J* = 92.8, 41.5 Hz, major); **IR** (film, cm^{-1}): 3519, 2932, 1533, 1528, 1491, 1384, 1258, 1152, 1070, 1011, 975; **HRMS:** m/z calculated for $C_{19}H_{23}BF_2NO$ [M−F]$^+$ 330.1835; found 330.1839.

Note: The compound was isolated as mixture of diastereomers, identified as minor and major where possible.

((1-benzyl-3-(ethoxycarbonyl)piperidin-1-ium-1-yl)methyl)trifluoroborate (**4h**)

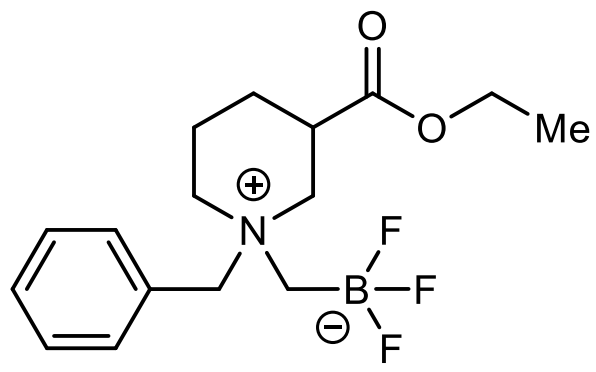

Prepared by procedure general procedure **2.5.2.1. (method I)** using ethyl 1-Benzylpiperidine-3-carboxylate (**1h**) (1.9 g, 7.5 mmol, 1.0 equiv) and 2-(Bromomethyl)-4,4,5,5,-tetramethyl-1,3,2-dioxaborolane (2.0 mL, 11 mmol, 1.5 equiv) in acetonitrile (30 mL). Fluorination was performed with aqueous potassium hydrogen fluoride (3 M, 7.5 mL, 23 mmol, 3.0 equiv) and HCl (4 M in 1,4-dioxane, 1.9 mL, 7.5 mmol, 1.0 equiv) in 4:1 acetonitrile/water (0.10 M). The title compound was obtained as a white solid (1.9 g, 5.8 mmol, 77% yield, 3.6:1 d.r.). **^{1}H NMR** (700 MHz, CD_3CN) δ 7.61 – 7.57 (m, 2H), 7.54 – 7.45 (m, 3H), 4.62 – 4.50 (m, 2H), 4.16 – 4.05 (m, 2H), 3.61 – 3.56 (m, 1H, major), 3.54 – 3.49 (m, 1H, minor), 3.36 – 3.28 (m, 1H), 3.23 (tt, *J* = 12.3, 3.7 Hz, 1H, minor), 3.10 (tt, *J* = 12.3, 3.5 Hz, 1H, major), 3.06 – 2.98 (m, 1H), 2.43 – 2.34 (m, 2H, minor), 2.31 – 2.22 (m, 2H, major), 2.22 – 2.14 (m, 1H,), 2.12 – 2.03 (m, 2H), 1.91 – 1.89 (m, 1H, minor), 1.82 – 1.74 (m, 1H, major), 1.62 (qd, *J* = 13.4, 3.8 Hz, 1H, minor), 1.41 (qd, *J* = 13.7, 4.8 Hz, 1H, major), 1.23 (t, *J* = 7.1 Hz, 3H, minor), 1.20 (t, *J* = 7.1 Hz, 3H, major); **^{13}C NMR** (176 MHz, $CDCl_3$) δ 172.7 (major), 172.3 (minor), 134.5 (major), 134.0 (minor), 131.1, 129.7 (major), 129.6 (minor), 129.3, 70.3 (major), 62.1 (minor), 61.9 (major), 61.2 (major), 61.0 (minor), 60.9 (major), 60.8 (minor), 60.5 (minor), 49.9, 37.5 (minor), 37.3 (major), 25.8 (major), 25.5 (minor), 25.2, 20.3 (major), 20.0 (minor), 14.4; **^{19}F NMR** (471 MHz, CD_3CN) δ −136.2, −137.7; **IR** (film, cm^{-1}): 2977, 1725, 1458, 1299, 1204, 1057, 1001, 918, 890, 863, 703; **HRMS:** m/z calculated for $C_{16}H_{23}BF_2NO_2$ [M−F]$^+$ 310.1784; found 310.1780.

((1'-benzyl-4-oxospiro[chromane-2,4'-piperidin]-1'-yl-1'-ium)methyl)trifluoroborate **(4i)**

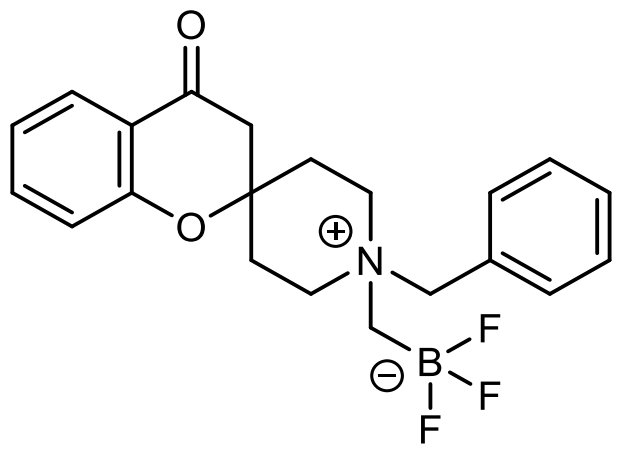

Prepared by general procedure **2.5.2.1. (method II)** using 1'-benzylspiro[chromane-2,4'-piperidin]-4-one (**1i)** (461 mg, 1.5 mmol, 1.0 equiv) and 2-(Bromomethyl)-4,4,5,5,-tetramethyl-1,3,2-dioxaborolane (0.38 mL, 2.3 mmol, 1.5 equiv) in acetonitrile (6.0 mL). Fluorination was performed with aqueous potassium hydrogen fluoride (3 M, 1.3 mL, 3.8 mmol, 2.5 equiv) and HCl (4 M in 1,4-dioxane, 0.38 mL, 1.5 mmol, 1.0 equiv) in 4:1 acetonitrile/water (0.10 M). The title compound was obtained as a white solid (433 mg, 1.1 mmol, 74% yield). **^{1}H NMR** (700 MHz, Acetone-d6) δ 7.80 (dd, *J* = 7.8, 1.7 Hz, 1H), 7.74 – 7.71 (m, 2H), 7.58 (ddd, *J* = 8.3, 7.2, 1.8 Hz, 1H), 7.54 – 7.48 (m, 3H), 7.08 (ddd, *J* = 7.8, 7.3, 1.1 Hz, 1H), 6.97 (dd, *J* = 8.3, 1.0 Hz, 1H), 4.76 (s, 2H), 3.66 (td, *J* = 13.0, 3.1 Hz, 2H), 3.43 (dp, *J* = 12.9, 2.3 Hz, 2H), 3.00 (s, 2H), 2.47 – 2.40 (m, 2H), 2.38 (d, *J* = 4.6 Hz, 2H), 2.22 – 2.16 (m, 2H); **^{13}C NMR** (176 MHz, Acetone-d6) δ 191.0, 159.1, 137.1, 134.5, 134.5, 130.9, 129.7, 129.5, 127.1, 122.5, 121.8, 119.3, 76.4, 69.4, 56.4, 47.4; **^{19}F NMR** (376 MHz, Acetone-d6) δ −138.1 (dd, *J* = 87.2, 39.3 Hz); **IR** (film, cm^{-1}): 2960, 1688, 1608, 1463, 1331, 1306, 1234, 1038, 1012, 997, 980; **HRMS:** m/z calculated for $C_{21}H_{23}BF_2NO_2$ [M−F]$^+$ 370.1784; found 370.1786.

((1-benzylpyrrolidin-1-ium-1-yl)methyl)trifluoroborate **(4j)**

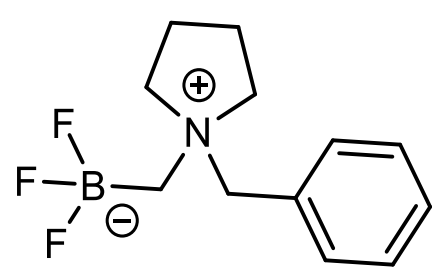

Prepared by general procedure **2.5.2.2. (method I)** using 1-benzylpyrrolidine (323 mg, 2.0 mmol, 1.0 equiv) and potassium (bromomethyl)trifluoroborate (603 mg, 3.0 mmol, 1.5 equiv) in acetonitrile (2.0 mL). The title compound was obtained as a white solid (375 mg, 1.5 mmol, 77% yield). **^{1}H NMR** (700 MHz, DMSO-d6) δ 7.61 – 7.56 (m, 2H), 7.52 – 7.45 (m, 3H), 4.43 (s, 2H), 3.36 (tq, *J* = 5.0, 3.2 Hz, 4H), 2.13 (q, *J* = 4.7 Hz, 2H), 2.06 (qd, *J* = 4.9, 2.4 Hz, 4H); **^{13}C NMR** (176 MHz, DMSO-d6) δ 132.8 (d, *J* = 1.0 Hz), 130.1, 129.9, 128.8, 63.3, 62.4 (d, *J* = 2.7 Hz), 30.8, 20.9; **^{19}F NMR** (376 MHz, DMSO-d6) δ −134.8. **IR** (neat, cm^{-1}): 2975, 1047, 993, 904, 729, 702; **HRMS:** m/z calculated for $C_{12}H_{17}BF_2N$ [M−F]$^+$ 224.1417; found 224.1419.

((2-benzyl-7-(tert-butoxycarbonyl)-2,7-diazaspiro[4.4]nonan-2-ium-2-yl)methyl)trifluoroborate **(4k)**

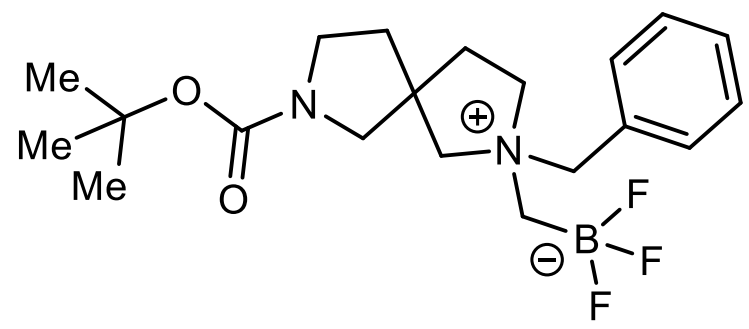

Prepared using general procedure **2.5.2.1. (method II)** using tert-butyl 7-benzyl-2,7-diazaspiro[4.4]nonane-2-carboxylate (**1k**) (316 mg, 1.0 mmol, 1.0 equiv) and 2-(Bromomethyl)-4,4,5,5,-tetramethyl-1,3,2-dioxaborolane (0.25 mL, 1.5 mmol, 1.5 equiv) in acetonitrile (4.0 mL). Fluorination was performed with aqueous potassium hydrogen fluoride (3 M, 0.84 mL, 2.5 mmol, 2.5 equiv) and HCl (4 M in 1,4-dioxane, 0.25 mL, 1.0 mmol, 1.0 equiv) in 4:1 acetonitrile/water (0.10 M). The title compound was obtained as a white solid (321 mg, 0.81 mmol, 81% yield). **^{1}H NMR** (700 MHz, Acetone-d6) δ 7.76 – 7.71 (m, 2H), 7.53 – 7.46 (m, 3H), 4.74 (dd, *J* = 14.9, 12.4 Hz, 1H), 4.66 (td, *J* = 14.5, 3.3 Hz, 1H), 3.73 (ttd, *J* = 24.1, 12.4, 8.3 Hz, 3H), 3.55 – 3.46 (m, 1H), 3.44 – 3.30 (m, 3H), 2.44 – 2.21 (m, 2H), 2.19 – 2.06 (m, 2H), 1.45 – 1.41 (m, 9H); **^{13}C NMR** (176 MHz, Acetone-d6) δ 154.78, 154.71, 154.6, 133.98, 133.97, 133.97, 133.95, 133.94, 133.94, 131.07, 131.04, 130.86, 130.85, 129.68, 129.67, 79.41, 79.39, 79.37, 79.3, 74.8, 74.7, 71.7, 71.6, 71.39, 71.37, 66.64, 66.58, 66.4, 66.2, 64.0, 63.8, 58.1, 57.9, 57.8, 57.5, 48.33, 48.29, 47.44, 47.40, 45.9, 45.7, 45.5, 45.3, 38.8, 38.4, 38.1, 37.8, 34.92, 34.86, 34.76, 34.70, 28.72, 28.68, 28.67, 25.31, 25.26, 25.2, 25.0; **^{19}F NMR** (376 MHz, Acetone-d6) δ −138.5; **IR** (film, cm^{-1}): 2978, 2939, 2880, 1687, 1402, 1366, 1172, 1146, 1051, 998, 971, 883; **HRMS:** m/z calculated for $C_{20}H_{30}BF_2N_2O_2$ [M−F]$^+$ 379.2363; found 379.2365.
Note: The compound was isolated as mixture of diastereomers and rotamers.

((2-benzyl-7-(tert-butoxycarbonyl)-2,7-diazaspiro[3.5]nonan-2-ium-2-yl)methyl)trifluoroborate (**4l**)

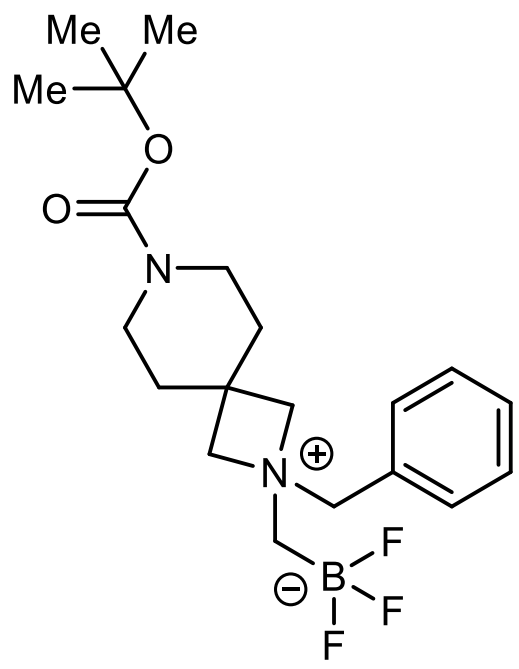

Prepared using general procedure **2.5.2.1. (method II)** using tert-butyl 2-benzyl-2,7-diazaspiro[3.5]nonane-7-carboxylate (**1l**) (400 mg, 1.3 mmol, 1.0 equiv) and 2-(Bromomethyl)-4,4,5,5,-tetramethyl-1,3,2-dioxaborolane (0.38 mL, 2.3 mmol, 1.8 equiv) in acetonitrile (5.2 mL).

Fluorination was performed with aqueous potassium hydrogen fluoride (3 M, 1.3 mL, 3.8 mmol, 3.0 equiv) and HCl (4 M in 1,4-dioxane, 0.38 mL, 1.5 mmol, 1.2 equiv) in 4:1 acetonitrile/water (0.10 M). The title compound was obtained as a white solid (430 mg, 1.1 mmol, 87% yield). **^{1}H NMR** (700 MHz, Acetone-d6) δ 7.91 (d, *J* = 7.0 Hz, 2H, minor), 7.83 – 7.81 (m, 2H, minor), 7.77 (dd, *J* = 7.5, 1.8 Hz, 2H, major), 7.52 (dtd, *J* = 15.7, 7.3, 5.3 Hz, 3H), 4.74 (s, 2H, minor), 4.65 (s, 2H, major), 4.42 (d, *J* = 11.8 Hz, 1H), 4.35 – 4.28 (m, 1H), 4.22 (d, *J* = 11.7 Hz, 1H), 3.43 – 3.32 (m, 4H), 2.53 (d, *J* = 22.0 Hz, 2H), 1.95 (dt, *J* = 12.7, 5.7 Hz, 2H), 1.71 (s, 2H), 1.43 (s, 9H); **^{13}C NMR** (176 MHz, Acetone-d6) δ 155.1, 134.3 (minor), 134.1 (d, *J* = 1.2 Hz, minor), 134.0 (q, *J* = 1.2 Hz, major), 131.5, 131.1 (minor), 131.0 (major), 130.8, 130.7, 129.84 (major), 129.7 (minor), 129.5 (minor), 79.71 (major), 79.68 (minor), 79.67 (minor), 73.1 (major), 72.9 (minor), 67.4, 67.11, 41.7, 40.8, 36.8, 36.2, 36.0, 31.32 (major), 31.31 (minor), 28.6, 26.65, 26.52; **^{19}F NMR** (471 MHz, Acetone-d6) δ −132.4 (dd, *J* = 112.8, 50.3 Hz), −137.8 (dd, *J* = 92.3, 41.0 Hz); **IR** (film, cm^{-1}): 2978, 2935, 2855, 1686, 1425, 1367, 1272, 1245, 1152, 1064, 968, 918; **HRMS:** m/z calculated for $C_{20}H_{30}BF_3N_2O_2Na$ [M+Na]$^+$ 421.2245; found 421.2246.

Note: The compound was isolated as mixture of rotamers, identified as major and minor where possible.

((1-benzyl-4-(tert-butoxycarbonyl)-1,4-diazepan-1-ium-1-yl)methyl)trifluoroborate (**4m**)

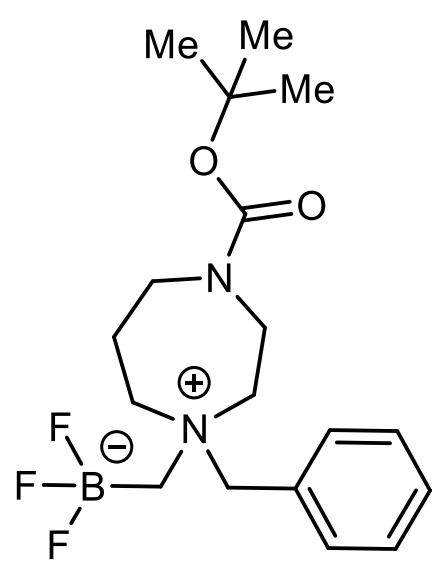

Prepared using general procedure **2.5.2.1. (method II)** using tert-butyl 4-benzyl-1,4-diazepane-1-carboxylate (**1m)** (435 mg, 1.5 mmol, 1.0 equiv) and 2-(Bromomethyl)-4,4,5,5,-tetramethyl-1,3,2-dioxaborolane (0.38 mL, 2.3 mmol, 1.5 equiv) in acetonitrile (6.0 mL). Fluorination was performed with aqueous potassium hydrogen fluoride (3 M, 1.3 mL, 3.8 mmol, 2.5 equiv) and HCl (4 M in 1,4-dioxane, 0.38 mL, 1.5 mmol, 1.0 equiv) in 4:1 acetonitrile/water (0.10 M). The title compound was obtained as a white solid (433 mg, 1.2 mmol, 77% yield). **^{1}H NMR** (700 MHz, Acetone-d6) δ 7.72 – 7.68 (m, 2H), 7.50 (d, *J* = 8.5 Hz, 3H), 4.81 – 4.63 (m, 2H), 3.91 (dd, *J* = 68.2, 16.6 Hz, 1H), 3.82 – 3.45 (m, 6H), 3.37 (dt, *J* = 26.6, 12.1 Hz, 1H), 2.33 (d, *J* = 46.3 Hz, 4H), 2.21 – 2.11 (m, 1H), 1.42 (s,

9H); **13C NMR** (176 MHz, Acetone-d6) δ 154.9, 154.2, 133.6, 133.6, 130.0, 129.2, 128.7, 79.4, 67.9, 67.6, 64.6, 63.9, 62.6, 62.5, 45.0, 43.7, 39.9, 38.7, 27.6, 22.2. **19F NMR** (376 MHz, Acetone-d6) δ −137.13 (d, *J* = 43.2 Hz); **IR** (film, cm^{-1}): 2952, 2927, 2857, 1687, 1459, 1412, 1368, 1248, 1169, 1135, 1046, 1004, 981, 923; **HRMS:** m/z calculated for $C_{18}H_{28}BF_3N_2O_2Na$ $[M+Na]^+$ 395.2088; found 395.2088.

Note: The compound was isolated as mixture of rotamers.

((1-benzyl-4-hydroxy-4-phenylpiperidin-1-ium-1-yl)methyl)trifluoroborate **(4n)**

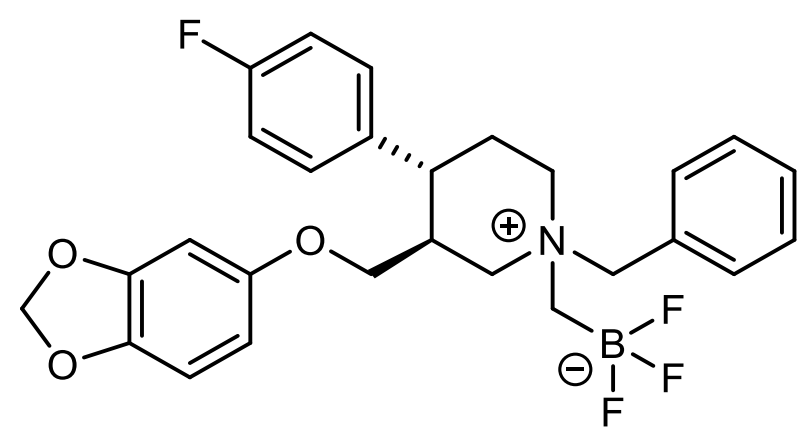

Prepared using general procedure **2.5.2.2. (method II)** using *N*-benzyl paroxetine (**1n**) (300 mg, 0.70 mmol, 1.0 equiv), potassium (bromomethyl)trifluoroborate (325 g, 1.6 mmol, 2.3 equiv) and potassium carbonate (0.70 mmol, 97 mg, 1.0 equiv) in MeCN (1.6 mL). The title compound was obtained as a yellow solid (235 mg, 0.47 mmol, 67% yield, 2:1 d.r.). **1H NMR** (700 MHz, Acetone-d6) δ 7.79 – 7.77 (m, 2H, minor), 7.75 – 7.72 (m, 2H, major), 7.64 – 7.60 (m, 2H, minor), 7.54 – 7.48 (m, 3H), 7.43 – 7.39 (m, 2H, major), 7.16 – 7.12 (m, 2H, minor), 7.07 (m, 2H, major), 6.67 (dd, *J* = 8.5, 1.2 Hz, 1H), 6.42 (d, *J* = 2.5 Hz, 1H, major), 6.41 (d, *J* = 2.5 Hz, 1H, minor), 6.23 (td, *J* = 8.5, 2.5 Hz, 1H), 5.91 (s, 2H, major), 5.90 (s, 2H, minor), 5.03 (d, *J* = 12.9 Hz, 1H, minor), 4.85 (d, *J* = 12.9 Hz, 1H, minor), 4.82 (d, *J* = 12.7 Hz, 1H, major), 4.77 (d, *J* = 12.7 Hz, 1H, major), 3.80 – 3.74 (m, 1H), 3.74 – 3.68 (m, 2H), 3.63 – 3.55 (m, 2H), 3.54 – 3.46 (m, 1H), 3.17 – 3.06 (m, 1H, min), 3.02 (td, *J* = 12.4, 4.3 Hz, 1H), 2.86 – 2.76 (m, 1H), 2.69 – 2.62 (m, 1H, minor), 2.62 – 2.51 (m, 2H, major), 2.46 (dtd, *J* = 15.0, 13.0, 4.0 Hz, 1H, major), 2.39 – 2.33 (m, 1H, minor), 2.30 – 2.23 (m, 1H, minor), 2.13 (dt, *J* = 15.2, 2.5 Hz, 1H, minor), 2.01 (dq, *J* = 15.1, 3.4 Hz, 1H, major); **13C NMR** (176 MHz, Acetone-d6) δ 163.5, 163.3, 163.0, 162.1, 161.9, 161.6, 155.4, 155.1, 154.8, 149.20, 149.16, 149.11, 142.89, 142.85, 142.5, 138.92, 138.90, 138.84, 138.83, 134.52, 134.52, 134.22, 134.21, 130.86, 130.84, 130.56, 130.51, 130.41, 130.37, 130.07, 130.02, 129.76, 129.67, 129.55, 129.54, 128.98, 128.92, 127.7, 127.3, 116.3, 116.26, 116.25, 116.19, 116.13, 116.0, 115.8, 108.66, 108.64, 108.57, 106.9, 106.6, 106.4, 102.11, 102.10, 102.0, 98.87, 98.85, 98.6, 98.5, 70.5, 69.94, 69.92, 69.90, 69.1, 68.9, 63.7, 63.65,

63.63, 63.19, 63.18, 61.20, 61.19, 61.14, 61.13, 60.36, 60.35, 58.4, 54.6, 44.8, 42.9, 42.62, 42.07, 38.0, 28.9; **^{19}F NMR** (376 MHz, Acetone-d6) δ −118.1 (minor), −118.2 (major), −137.1 (minor), −138.7 (major); **IR** (film, cm^{-1}): 2951, 2927, 2859, 1631, 1606, 1511, 1489, 1469, 1182, 1034, 1016, 983, 926, 917; **HRMS:** m/z calculated for $C_{27}H_{28}BF_2NO_3$ $[M-F]^+$ 482.2109; found 482.2120.

Note: The compound was isolated as mixture of diastereomers and conformers, identified as minor and major where possible.

trifluoro((4-(4-methoxybenzyl)morpholino-4-ium)methyl)borate (**4o**)

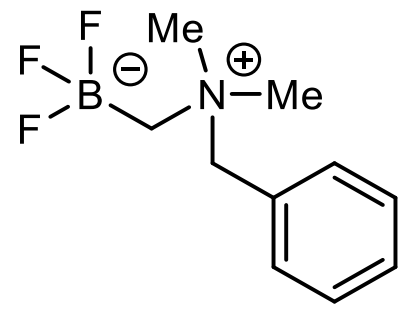

Prepared using general procedure **2.5.2.2. (method II)** using *N*,*N*-dimethylbenzylamine (**1o**) (0.30 mL, 2.0 mmol. 1.0 equiv) and potassium (bromomethyl)trifluoroborate (603 mg, 3.0 mmol, 1.5 equiv) in acetonitrile (2.0 mL). The title compound was obtained as a white solid (433 mg, 2.0 mmol, 99% yield). **^{1}H NMR** (500 MHz, DMSO-d6) δ 7.59 – 7.53 (m, 2H, H_6), 7.52 – 7.45 (m, 3H, H_5 + H_7), 4.43 (s, 2H, H_3), 2.91 (s, 6H, H_1), 2.29 – 2.19 (m, 2H, H_2); **^{13}C NMR** (126 MHz, DMSO-d6) δ 133.4 (C_4), 130.4 (C_7), 129.7 (C_5), 129.1 (C_6), 69.4 (C_3), 59.1 (C_1), 52.0 (C_2); **^{19}F NMR** (471 MHz, DMSO-d6) δ −135.0; **IR** (film, cm^{-1}): 1689, 1527, 1477, 1455, 1428, 1386, 1348, 1335, 1318, 1214, 1135, 1110, 1061, 1031, 1015, 989, 972, 926, 871, 806, 788, 739, 710; **HRMS:** m/z calculated for $C_{10}H_{15}BF_2N$ $[M-F]^+$ 198.1260; found 198.1262.

((benzyl(but-3-en-1-yl)(methyl)ammonio)methyl)trifluoroborate (**4p**)

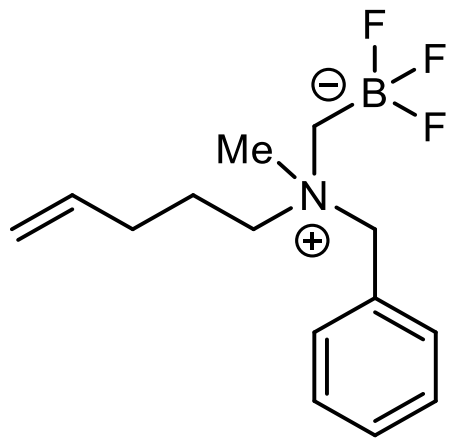

Prepared using general procedure **2.5.2.1. (method II)** using *N*-benzyl-*N*-methylpent-4-en-1-amine (**1p**) (284 mg, 1.5 mmol, 1.0 equiv) and 2-(Bromomethyl)-4,4,5,5,-tetramethyl-1,3,2-dioxaborolane (0.38 mL, 2.3 mmol, 1.5 equiv) in acetonitrile (6.0 mL). Fluorination was performed with aqueous potassium hydrogen fluoride (3 M, 1.3 mL, 3.8 mmol, 2.5 equiv) and HCl (4 M in 1,4-dioxane, 0.38 mL, 1.5 mmol, 1.0 equiv) in 4:1 acetonitrile/water (0.10 M). The title compound was obtained as a

colourless oil (385 mg, 1.4 mmol, 95% yield). **^{1}H NMR** (400 MHz, Acetone-d6) δ 7.66 (dd, *J* = 7.5, 2.1 Hz, 2H), 7.55 – 7.43 (m, 3H), 5.85 (ddt, *J* = 16.9, 10.2, 6.3 Hz, 1H), 5.08 (dd, *J* = 17.2, 1.9 Hz, 1H), 4.98 (dd, *J* = 10.3, 1.9 Hz, 1H), 4.61 (d, *J* = 12.8 Hz, 1H), 4.52 (d, *J* = 12.8 Hz, 1H), 3.39 – 3.22 (m, 2H), 2.96 (s, 3H), 2.41 – 2.23 (m, 2H), 2.17 – 1.93 (m, 2H); **^{13}C NMR** (101 MHz, Acetone-d6) δ 137.9, 134.0, 130.8, 130.1, 129.5, 116.1, 68.0, 67.9, 63.9, 50.5, 22.6; **^{19}F NMR** (376 MHz, Acetone-d6) δ −140.3 (d, *J* = 79.2 Hz); **IR** (film, cm^{-1}): 2923, 2857, 1475, 1459, 1049, 1024, 990, 907, 736; **HRMS:** m/z calculated for $C_{14}H_{21}BF_2N$ $[M-F]^+$ 252.1729; found 252.1733.

((benzyl(but-3-en-1-yl)(methyl)ammonio)methyl)trifluoroborate (**4q**)

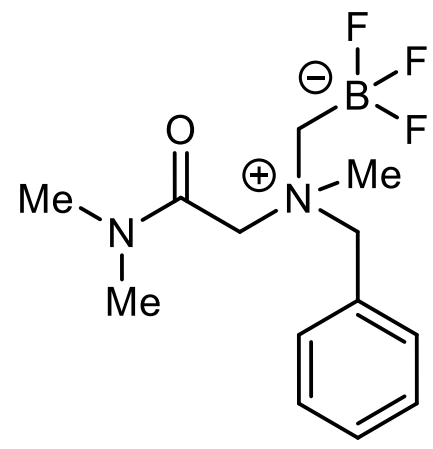

Prepared using general procedure **2.5.2.1. (method II)** using 2-(benzyl(methyl)amino)-N,N-dimethylacetamide (**1q)** (309 mg, 1.5 mmol, 1.0 equiv) and 2-(Bromomethyl)-4,4,5,5,-tetramethyl-1,3,2-dioxaborolane (0.38 mL, 2.3 mmol, 1.5 equiv) in acetonitrile (6.0 mL). Fluorination was performed with aqueous potassium hydrogen fluoride (3 M, 1.3 mL, 3.8 mmol, 2.5 equiv) and HCl (4 M in 1,4-dioxane, 0.38 mL, 1.5 mmol, 1.0 equiv) in 4:1 acetonitrile/water (0.10 M). The title compound was obtained as a white solid (282 mg, 0.98 mmol, 79% yield). **^{1}H NMR** (700 MHz, Acetone-d6) δ 7.60 – 7.57 (m, 2H), 7.54 – 7.47 (m, 3H), 5.02 (d, *J* = 12.5 Hz, 1H), 4.88 (d, *J* = 12.5 Hz, 1H), 4.20 (d, *J* = 16.4 Hz, 1H), 4.09 (d, *J* = 16.4 Hz, 1H), 3.26 (s, 3H), 3.00 (d, *J* = 1.4 Hz, 6H), 2.64 (m, 1H), 2.47 (m, 1H); **^{13}C NMR** (176 MHz, Acetone-d6) δ 165.5, 133.9, 131.0, 130.3, 129.7, 67.7, 67.7, 60.0, 51.3, 36.6, 35.6; **^{19}F NMR** (376 MHz, Acetone-d6) δ −137.8 (dd, *J* = 95.9, 44.3 Hz); **IR** (film, cm^{-1}): 2939, 1656, 1498, 1459, 1365, 1151, 1053, 1016, 986, 897; **HRMS:** m/z calculated for $C_{13}H_{20}BF_2N_2O$ $[M-F]^+$ 269.1631; found 269.1636.

((Benzyl(methyl)((*S*)-3-(naphthalen-1-yloxy)-3-(thiophen-2-yl)propyl)ammonio)methyl) trifluoroborate (**4r**)

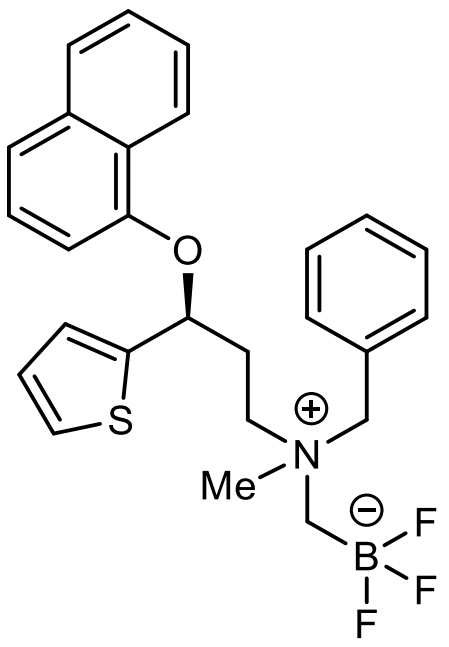

Prepared by general procedure **2.5.2.1. (method I)** using (*S*)-*N*-benzyl-*N*-methyl-3-(naphthalen-1-yloxy)-3-(thiophen-2-yl)propan-1-amine (**1r**) (401 mg, 1.0 mmol, 1.0 equiv) and 2-(Bromomethyl)-4,4,5,5,-tetramethyl-1,3,2-dioxaborolane (0.25 mL, 1.5 mmol, 1.5 equiv) in acetonitrile (4.0 mL). Fluorination was performed with aqueous potassium hydrogen fluoride (3 M, 0.90 mL, 2.5 mmol, 2.5 equiv) and HCl (4 M in 1,4-dioxane, 0.25 mL, 1.0 mmol, 1.0 equiv) in 4:1 acetonitrile/water (0.20 M). The title compound was obtained as a white solid (343 mg, 0.73 mmol, 73% yield, 1:1 d.r.). **^{1}H NMR** (400 MHz, Acetone-d6) δ 8.44 – 8.28 (m, 1H), 7.87 – 7.80 (m, 1H), 7.73 – 7.62 (m, 2H), 7.53 – 7.42 (m, 6H), 7.39 (dt, *J* = 5.1, 1.4 Hz, 1H), 7.32 (dd, *J* = 7.8, 2.1 Hz, 1H), 7.29 (d, *J* = 3.5 Hz, 1H), 7.05 (d, *J* = 7.8 Hz, 1H), 6.99 (ddd, *J* = 5.1, 3.5, 1.4 Hz, 1H), 6.10 – 5.99 (m, 1H), 4.77 – 4.55 (m, 2H,), 3.86 – 3.70 (m, 1H, dia 1), 3.70 – 3.48 (m, 1H, dia 2), 3.10 – 3.04 (m, 3H), 3.04 – 2.90 (m, 1H, dia 1), 2.87 – 2.69 (m, 1H, dia 2), 2.59 – 2.42 (m, 1H, dia 1), 2.42 – 2.29 (m, 1H, dia 2); **^{13}C NMR** (101 MHz, Acetone-d6) δ 152.8 (dia 1), 152.7 (dia 2), 143.9, 134.8, 133.2, 133.2, 130.0, 130.0, 129.1, 128.8, 128.8, 127.5, 126.7, 126.4, 126.0, 125.8, 125.8, 125.4, 125.3, 122.0 (dia 1), 122.0 (dia 2), 120.8, 120.8, 107.3, 73.9 (dia 1), 73.9 (dia 2), 67.5, 60.5, 56.6, 50.0, 32.2 (dia 1), 32.1 (dia 2); **^{19}F NMR** (471 MHz, Acetone-d6) δ −138.0; **IR** (film, cm^{-1}): 1688, 1681, 1646, 1531, 1475, 1456, 1433, 1395, 1379, 1348, 1234, 1212, 1172, 1092, 1048, 1016, 989, 973, 953, 928, 877, 793, 772, 755, 739, 700; **HRMS:** m/z calculated for $C_{26}H_{27}BF_2NOS$ [M−F]$^+$ 450.1869; found 450.1887.

(2-Benzyl-6,7-dimethoxy-1,2,3,4-tetrahydroisoquinolin-2-ium-2-yl)methyl)trifluoroborate (**4s**)

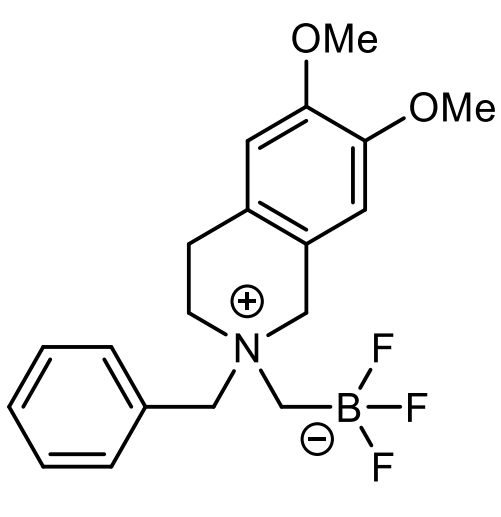

Prepared by general procedure **2.5.2.1. (method I)** using 2-benzyl-6,7-dimethoxy-1,2,3,4-tetrahydroisoquinoline (**1s**) (425 mg, 1.5 mmol, 1.0 equiv) and 2-(Bromomethyl)-4,4,5,5,-tetramethyl-1,3,2-dioxaborolane (0.38 mL, 2.3 mmol, 1.5 equiv) in acetonitrile (6.0 mL). The reaction mixture was stirred at room temperature until consumption of the amine starting material. Fluorination was performed with aqueous potassium hydrogen fluoride (3 M, 1.3 mL, 3.8 mmol, 2.5 equiv) and HCl (4 M in 1,4-dioxane, 0.38 mL, 1.5 mmol, 1.0 equiv) in 4:1 acetonitrile/water (0.10 M). The title compound was obtained as a pale brown solid (360 mg, 1.0 mmol, 66% yield). **^{1}H NMR** (500 MHz, DMSO-d6) δ 7.66 – 7.58 (m, 2H), 7.54 – 7.48 (m, 3H), 6.85 (s, 1H), 6.75 (s, 1H), 4.68 (d, *J* = 12.6 Hz, 1H), 4.50 (d, *J* = 12.6 Hz, 1H), 4.43 (d, *J* = 14.9 Hz, 1H), 4.04 (d, *J* = 14.9 Hz, 1H), 3.77 – 3.71 (m, 4H), 3.70 (s, 3H), 3.50 (ddd, *J* = 12.3, 9.4, 5.8 Hz, 1H), 3.21 (ddd, *J* = 16.5, 9.4, 6.4 Hz, 1H), 3.06 (ddd, *J* = 17.9, 5.0, 5.0 Hz, 1H), 2.18 – 2.08 (m, 2H); **^{13}C NMR** (126 MHz, DMSO-d6) δ 148.5, 147.8, 133.2, 129.9, 128.7, 128.6, 122.1, 119.3, 111.3, 110.3, 64.8, 61.1, 57.0, 55.5, 55.5, 50.7, 23.4; **^{19}F NMR** (376 MHz, DMSO-d6) δ −134.2; **IR** (film, cm^{-1}): 1697, 1519, 1465, 1453, 1422, 1370, 1348, 1329, 1266, 1239, 1216, 1119, 1081, 1056, 1045, 1020, 1005, 999, 990, 971, 903, 888, 871, 830, 790, 757, 743, 713, 698; **HRMS:** m/z calculated for $C_{19}H_{23}BF_2NO_2$ [M−F]$^+$ 346.1784; found 346.1796.

((2-Benzyl-2,3,4,9-tetrahydro-1*H*-pyrido[3,4-*b*]indol-2-ium-2-yl)methyl)trifluoroborate (**4t**)

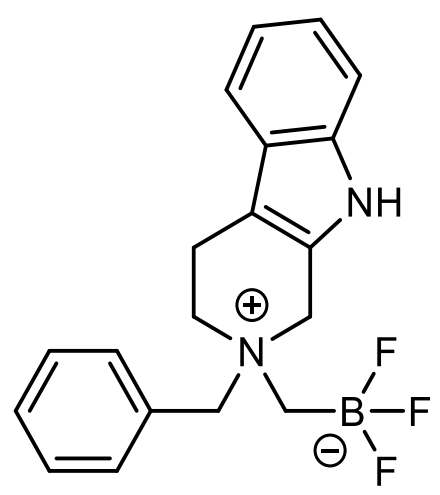

Prepared by general procedure **2.5.2.1. (method I)** using 2-benzyl-2,3,4,9-tetrahydro-1H-pyrido[3,4-b]indole (**1t**) (262 mg, 1.0 mmol, 1.0 equiv) and 2-(Bromomethyl)-4,4,5,5,-tetramethyl-1,3,2-dioxaborolane (0.20 mL, 1.2 mmol, 1.2 equiv) in acetonitrile (4.0 mL). Fluorination was performed with aqueous potassium hydrogen fluoride (3 M, 1.0 mL, 3.0 mmol, 3.0 equiv) and HCl (4 M in 1,4-dioxane, 0.50 mL, 2.0 mmol, 2.0 equiv) in 4:1 acetonitrile/water (0.20 M). The title compound was obtained as a pale orange solid (295 mg, 0.86 mmol, 86% yield). **^{1}H NMR** (400 MHz, Acetone-d6) δ 10.12 (s, 1H), 7.74 – 7.66 (m, 2H), 7.58 – 7.47 (m, 4H), 7.43 (dt, *J* = 8.1, 1.0 Hz, 1H), 7.16 (ddd, *J* = 8.3, 7.1, 1.2 Hz, 1H), 7.09 (ddd, *J* = 8.1, 7.0, 1.1 Hz, 1H), 4.87 (d, *J* = 12.7 Hz, 1H), 4.72 (d, *J* = 12.7 Hz, 1H), 4.66 (d, *J* = 15.4 Hz, 1H), 4.53 (d, *J* = 15.4 Hz, 1H), 4.00 (dt, *J* = 11.6, 5.8 Hz, 1H), 3.74 (dt, *J* = 12.8, 6.5 Hz, 1H), 3.38 – 3.29 (m, 1H), 3.28 – 3.19 (m, 1H), 2.52 – 2.37 (m, 2H); **^{13}C NMR** (101 MHz, Acetone-d6) δ 138.0, 134.3, 130.9, 129.8, 129.7, 127.1, 126.7, 123.0, 120.4, 119.1, 112.4, 105.6,

65.7, 59.8, 55.0, 18.8; **^{19}F NMR (**376 MHz, Acetone-d6) δ −136.7; **IR** (film, cm^{-1}): 1682, 1646, 1526, 1496, 1454, 1378, 1348, 1328, 1227, 1242, 1212, 1161, 1086, 1048, 993, 973, 952, 921, 894, 878, 771, 749, 700, 649; **HRMS:** m/z calculated for $C_{19}H_{20}BF_2N_2$ $[M-F]^+$ 325.1682; found 325.1686.

((Benzyl(methyl)(phenyl)ammonio)methyl)trifluoroborate (**4u**)

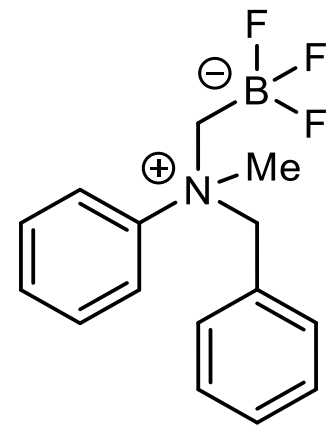

Prepared by general procedure **2.5.2.1. (method I)** using *N*-benzyl-*N*-methylaniline (**1u**) (296 mg, 1.5 mmol, 1.0 equiv) and 2-(Bromomethyl)-4,4,5,5,-tetramethyl-1,3,2-dioxaborolane (0.38 mL, 2.3 mmol, 1.5 equiv) in acetonitrile (6.0 mL). Fluorination was performed with aqueous potassium hydrogen fluoride (3 M, 1.3 mL, 3.8 mmol, 2.5 equiv) and HCl (4 M in 1,4-dioxane, 0.38 mL, 1.5 mmol, 1.0 equiv) in 4:1 acetonitrile/water (0.10 M). The title compound was obtained as a white solid (326 mg, 1.2 mmol, 77% yield). **^{1}H NMR** (700 MHz, Acetone-d6) δ 7.77 – 7.72 (m, 2H), 7.56 – 7.48 (m, 3H), 7.38 (t, *J* = 7.4 Hz, 1H), 7.28 – 7.24 (m, 2H), 7.06 – 7.01 (m, 2H), 5.10 (d, *J* = 12.9 Hz, 1H), 4.96 (d, *J* = 12.9 Hz, 1H), 3.59 (s, 3H), 3.10 – 2.95 (m, 1H); **^{13}C NMR** (176 MHz, Acetone-d6) δ 133.6, 130.9, 130.3, 130.2, 130.2, 129.3, 129.3, 123.0, 74.5, 64.1, 50.6 ; **^{19}F NMR** (471 MHz, Acetone-d6) δ −137.3; **IR** (film, cm^{-1}): 1681, 1646, 1527, 1496, 1480, 1456, 1430, 1378, 1347, 1212, 1166, 1048, 1014, 986, 952, 927, 876, 771, 755, 736, 691; **HRMS:** m/z calculated for $C_{15}H_{17}BF_2N$ $[M-F]^+$ 260.1417; found 260.1422.

((1-Benzyl-4-phenylpiperazin-1-ium-1-yl)methyl)trifluoroborate (**4v**)

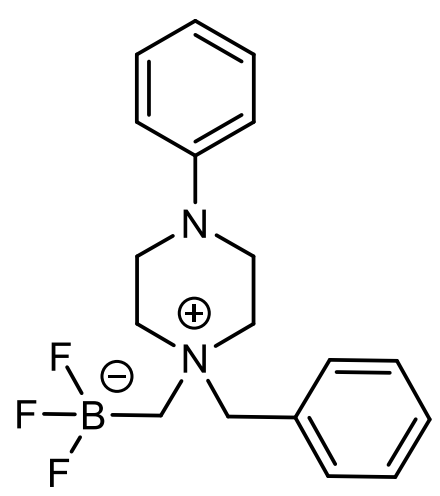

Prepared by general procedure **2.5.2.1. (method I)** using 1-benzyl-4-phenylpiperazine (**1v**) (252 mg, 1.0 mmol, 1.0 equiv) and 2-(Bromomethyl)-4,4,5,5,-tetramethyl-1,3,2-dioxaborolane (0.20 mL, 1.2 mmol, 1.2 equiv) in acetonitrile (4.0 mL). Fluorination was performed with aqueous potassium hydrogen fluoride (3 M, 1.0 mL, 3.0 mmol, 3.0 equiv) and HCl (4 M in 1,4-dioxane, 0.50 mL, 2.0 mmol, 2.0 equiv) in 4:1 acetonitrile/water (0.10 M). The title compound was afforded as a white solid

(321 mg, 0.96 mmol, 96% yield). **^{1}H NMR** (700 MHz, DMSO-d6) δ 7.67 – 7.61 (m, 2H), 7.51 (dd, *J* = 5.1, 1.9 Hz, 3H), 7.26 (dd, *J* = 8.7, 7.2 Hz, 2H), 7.04 – 6.94 (m, 2H), 6.92 – 6.80 (m, 1H), 4.66 (s, 2H), 3.68 (dt, *J* = 14.1, 4.2 Hz, 2H), 3.44 (t, *J* = 5.1 Hz, 4H), 3.38 – 3.35 (m, 4H), 2.28 (q, *J* = 4.6 Hz, 2H); **^{13}C NMR** (176 MHz, DMSO-d6) δ 150.0, 133.8, 130.4, 129.5, 129.2, 128.9, 120.2, 116.0, 65.6, 58.9, 55.4, 42.6; **^{19}F NMR** (471 MHz, DMSO-d6) δ −129.1; **IR** (film, cm^{-1}): 2869, 1481, 1456, 1453, 1351, 1339,1306, 1277, 1183, 1167, 1090, 1027, 1013, 966, 932, 916, 883, 819, 743, 727, 712; **HRMS:** m/z calculated for $C_{18}H_{22}BF_2N_2$ [M−F]$^+$ 315.1839; found 315.1848.

(((2S)-1-benzyl-4-(tert-butoxycarbonyl)-2-methylpiperazin-1-ium-1-yl)methyl)trifluoroborate (**4w**)

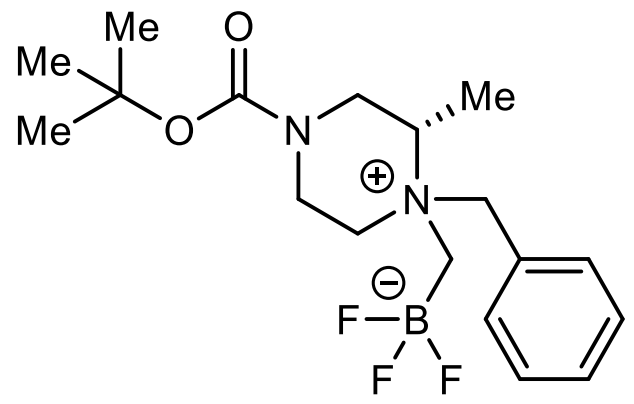

Prepared using general procedure **2.5.2.1. (method I)** using using *tert*-butyl (*S*)-4-benzyl-3-methylpiperazine-1-carboxylate (**1w**) (290 mg, 1.0 mmol, 1.0 equiv) and 2-(Bromomethyl)-4,4,5,5,-tetramethyl-1,3,2-dioxaborolane (0.20 mL, 1.2 mmol, 1.2 equiv) in acetonitrile (4.0 mL). Fluorination was performed with aqueous potassium hydrogen fluoride (3 M, 0.83 mL, 2.5 mmol, 2.5 equiv) and HCl (4 M in 1,4-dioxane, 0.25 mL, 1.0 mmol, 1.0 equiv) in 4:1 acetonitrile/water (0.10 M). The title compound was obtained as a white solid (190 mg, 0.51 mmol, 51% yield, d.r. 3:1). **^{1}H NMR** (700 MHz, Acetone-d6) δ 7.69 – 7.65 (m, 2H, minor), 7.62 – 7.57 (m, 2H, major), 7.55 – 7.47 (m, 3H), 4.92 (dq, *J* = 14.0, 2.0 Hz, 1H, major), 4.82 (s, 1H, minor), 4.70 (d, *J* = 14.0 Hz, 1H, major), 4.40 (d, *J* = 12.8 Hz, 1H, minor), 4.17 (s, 1H, minor), 4.02 (dd, *J* = 15.2, 3.5 Hz, 1H, minor), 3.96 (dd, *J* = 25.6, 13.9 Hz, 2H), 3.81 (d, *J* = 12.6 Hz, 1H, major), 3.56 (s, 2H), 3.38 – 3.19 (m, 2H), 2.97 (t, *J* = 11.6 Hz, 1H, major), 2.80 (s, 1H, minor), 2.69 (d, *J* = 13.7 Hz, 1H, major), 2.51 (s, 1H, minor), 2.30 – 2.12 (m, 1H), 1.57 (dd, *J* = 12.1, 6.2 Hz, 3H), 1.47 (s, 3H), 1.35 (s, 9H); **^{13}C NMR** (176 MHz, Acetone-d6) δ 154.8 (minor), 154.4 (major), 134.4 (minor), 134.1 (major), 131.0 (major), 130.8 (minor), 129.9 (major), 129.6 (minor), 129.3 (minor), 129.1 (major), 80.8 (minor), 80.6 (major), 65.1 (minor), 62.1 (major), 57.5 (major), 55.6 (minor), 44.8, 43.9, 39.5, 38.4, 30.6, 28.4 (minor), 28.3 (major), 13.1 (minor), 12.7 (major); **^{19}F NMR** (471 MHz, Acetone-d6) δ −137.03 (dd, *J* = 92.5, 39.7 Hz, major), −137.03 (m, minor); **IR** (film, cm^{-1}): 2980, 1693, 1455, 1413, 1368, 1395, 1367, 1269, 1247, 1145, 1047, 999, 970, 897; **HRMS:** m/z calculated for $C_{18}H_{28}BF_3N_2O_2Na$ [M +Na]$^+$ 395.2088; found 395.2089.

Note: The compound was isolated as mixture of diastereomers, identified as major and minor where possible.

(*S*)-((dimethyl(1-phenylethyl)ammonio)methyl)trifluoroborate (**4x**)

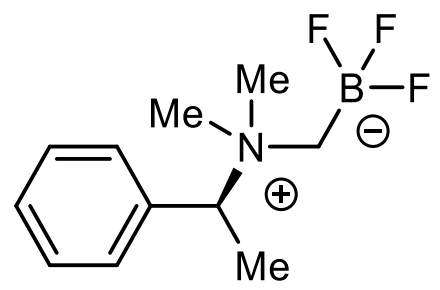

Prepared by general procedure **2.5.2.1. (method I)** using (*S*)-*N*,*N*-dimethyl-1-phenylethan-1-amine (**1x**) (0.49 mL, 3.0 mmol, 1.0 equiv), and 2-(Bromomethyl)-4,4,5,5,-tetramethyl-1,3,2-dioxaborolane (0.76 mL, 4.5 mmol, 1.5 equiv) in acetonitrile (6.0 mL). Fluorination was performed with aqueous potassium hydrogen fluoride (3 M, 4.5 mL, 14 mmol, 4.5 equiv) and HCl (4 M in 1,4-dioxane, 0.75 mL, 3.0 mmol, 2.0 equiv) in 4:1 acetonitrile/water (0.10 M). The title compound was obtained as a white solid (497 mg, 2.2 mmol, 72% yield). **^{1}H NMR** (500 MHz, DMSO-d6) δ 7.59 – 7.55 (m, 2H), 7.49 – 7.45 (m, 3H), 4.67 (q, *J* = 7.0 Hz, 1H), 2.86 (app d, *J* = 11.5 Hz, 6H), 2.25 – 2.13 (m, 2H), 1.67 (d, *J* = 6.9 Hz, 3H); **^{13}C NMR** (126 MHz, DMSO-d6) δ 134.5, 130.8, 129.7, 128.6, 73.5, 55.7, 49.7, 49.3, 14.6; **^{19}F NMR** (471 MHz, DMSO-d6) δ −134.4 (m); **IR** (film, cm^{-1}): 1467, 1454, 1406, 1319, 1110, 1088, 1072, 1031, 1016, 1003, 971, 943, 849, 800, 782, 732, 719, 706, 631; **HRMS:** m/z calculated for $C_{11}H_{17}BF_2N$ [M −F]$^+$ 212.1417; found 212.1418.

Trifluoro((4-(4-methoxybenzyl)morpholino-4-ium)methyl)borate (**4aa**)

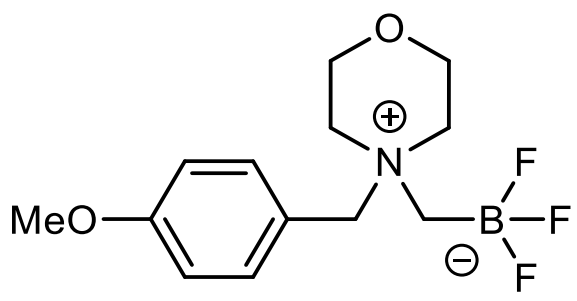

Prepared by general procedure **2.5.2.1. (method I)** using 4-(4-methoxybenzyl)morpholine (207 mg, 1.0 mmol, 1.0 equiv) and 2-(Bromomethyl)-4,4,5,5,-tetramethyl-1,3,2-dioxaborolane (0.25 mL, 1.5 mmol, 1.5 equiv) in acetonitrile (4.0 mL). Fluorination was performed with aqueous potassium hydrogen fluoride (3 M, 1.3 mL, 3.8 mmol, 3.8 equiv) and HCl (4 M in 1,4-dioxane, 0.25 mL, 1.0 mmol, 1.0 equiv) in 4:1 acetonitrile/water (0.10 M). The title compound was obtained as a white solid (275 mg, 0.95 mmol, 95% yield). **^{1}H NMR** (700 MHz, DMSO-d6) δ 7.55 – 7.46 (m, 2H), 7.09 – 7.00 (m, 2H), 4.55 (s, 2H), 3.99 – 3.86 (m, 4H), 3.79 (s, 3H), 3.33 – 3.27 (m, 2H), 3.25 – 3.18 (m, 2H), 2.34 – 2.25 (m, 2H); **^{13}C NMR** (176 MHz, DMSO-d6) δ 160.4, 134.7, 119.9, 114.0, 65.7, 60.2, 58.5, 55.2, 48.9; **^{19}F NMR** (471 MHz, DMSO-d6) δ −137.3; **IR** (film, cm^{-1}): 2938, 1611, 1515, 1458, 1447, 1292,

1253, 1236, 1222, 1181, 1126, 1096, 1069, 1050, 1020, 997, 959, 939, 910, 883, 846, 835, 923, 775, 763, 727; **HRMS:** m/z calculated for $C_{13}H_{19}BF_2NO_2$ [M−F]$^+$ 270.1477; found 270.1474.

((1-(4-(*tert*-butyl)benzyl)piperidin-1-ium-1-yl)methyl)trifluoroborate (**4ab**)

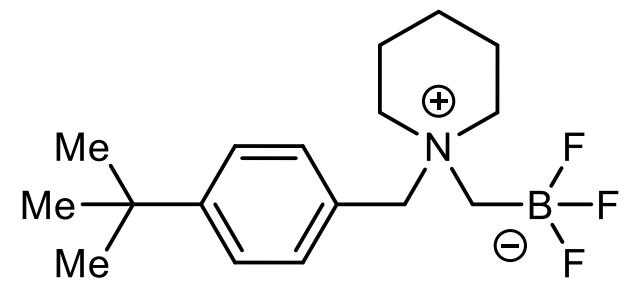

Prepared by general procedure **2.5.2.2. (method I)** using 1-(4-(*tert*-butyl)benzyl)piperidine (**1ab**) (210 mg, 0.91 mmol, 1.0 equiv) and potassium (bromomethyl)trifluoroborate (301 mg, 1.5 mmol, 1.6 equiv) in acetonitrile (1.0 mL). The title compound was obtained as a white solid (243 mg, 0.77 mmol, 85% yield); **^{1}H NMR** (500 MHz, DMSO-d6) δ 7.49 (br s, 4H), 4.47 (s, 2H), 3.24 (m, 2H), 3.19 – 3.07 (m, 2H), 2.14 (q, *J* = 4.8 Hz, 2H), 1.81 (br m, 4H), 1.59 (br m, 1H), 1.47 – 1.37 (br m, 1H), 1.29 (s, 9H); **^{13}C NMR** (126 MHz, DMSO-d6) δ 206.5, 152.2, 132.9, 125.8, 125.4, 59.6, 34.5, 31.0, 30.7, 21.0, 19.6; **^{19}F NMR** (471 MHz, DMSO-d6) δ −134.3; **IR** (neat, cm^{-1}): 2965, 2945, 1074, 1063, 971, 935, 912, 888, 843, 603, 593; **HRMS:** m/z calculated for $C_{17}H_{27}BF_2N$ [M−F]$^+$ 294.2199; found 294.2201.

((1-(4-(*tert*-butyl)benzyl)piperidin-1-ium-1-yl)methyl)trifluoroborate (**4ac**)

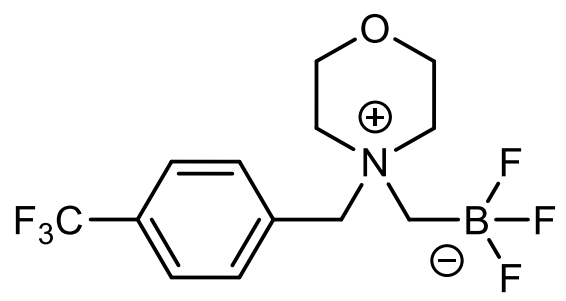

Prepared by general procedure **2.5.2.2. (method I)** using 4-(4-(trifluoromethyl)benzyl)morpholine (**1ac**) (437 mg, 1.8 mmol, 1.0 equiv) and potassium (bromomethyl)trifluoroborate (301 mg, 1.5 mmol, 1.5 equiv) in acetonitrile (1.0 mL). The title compound was obtained as a white solid (263 mg, 0.81 mmol, 81% yield). **^{1}H NMR** (500 MHz, DMSO-d6) δ 7.89 (d, *J* = 8.1 Hz, 2H), 7.83 (d, *J* = 8.1 Hz, 2H), 4.71 (s, 2H), 3.99 – 3.87 (m, 4H), 3.44 – 3.35 (m, 2H), 3.27 (d, *J* = 12.6 Hz, 2H), 2.32 (q, *J* = 4.7 Hz, 2H); **^{13}C NMR** (176 MHz, DMSO-d6) δ 134.2, 132.8, 130.2 (q, *J* = 32.0 Hz), 125.5 (q, *J* = 3.7 Hz), 124.0 (q, *J* = 272.3 Hz), 65.1, 60.1, 59.1; **^{19}F NMR** (471 MHz, DMSO-d6) δ −61.3, −134.0. **IR** (neat, cm^{-1}): 2881, 1322, 1120, 1058, 1044, 1021, 1001, 984, 916, 892, 827, 645, 633, 595; **HRMS**: m/z calculated for $C_{13}H_{16}BF_5NO$ [M−F]$^+$ 308.1240; found 308.1245.

trifluoro((4-(2-methylbenzyl)morpholino-4-ium)methyl)borate (**4ad**)

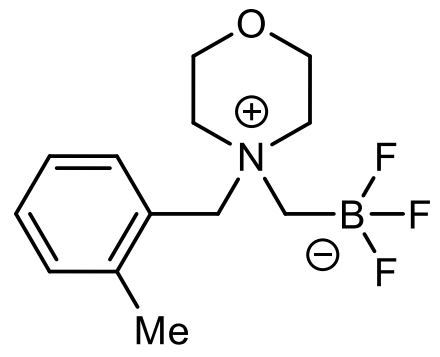

Prepared by general procedure **2.5.2.2. (method I)** using 4-(2-methylbenzyl)morpholine (**1ad**) (191 mg, 1.0 mmol, 1.0 equiv) and potassium (bromomethyl)trifluoroborate (301 mg, 1.5 mmol, 1.5 equiv) in acetonitrile (1.0 mL). The title compound was obtained as a white solid (248 mg, 0.91 mmol, 91% yield). **^{1}H NMR** (500 MHz, DMSO-d6) δ 7.51 (d, *J* = 7.6 Hz, 1H), 7.45 – 7.27 (m, 3H), 4.67 (s, 2H), 3.98 (t, *J* = 2.0 Hz, 2H), 3.83 (d, *J* = 3.1 Hz, 2H), 3.32 (d, 2H, 3.19 (t, *J* = 3.7 Hz, 2H), 2.55 (q, *J* = 4.8 Hz, 2), 2.44 (s, 3H); **^{13}C NMR** (176 MHz, DMSO-d6) δ 140.4, 134.9, 131.5, 130.1, 126.7, 126.0, 64.9, 60.2, 57.3, 19.9; **^{19}F NMR** (471 MHz, DMSO-d6) δ −134.3; **IR** (neat, cm^{-1}): 2896, 1443, 1102, 1061, 1020, 1003, 975, 937, 907, 888; **HRMS**: m/z calculated for $C_{13}H_{19}BF_2NO$ [M−F]$^+$ 254.1522; found 254.1527.

trifluoro((1-(4-fluoro-2-methoxybenzyl)piperidin-1-ium-1-yl)methyl)borate (**4ae**)

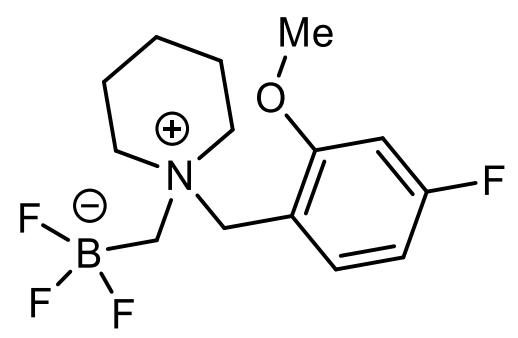

Prepared by general procedure **2.5.2.1. (method II)** using 1-(4-fluoro-2-methoxybenzyl)piperidine (**1ae**) (221 g, 1.0 mmol, 1.0 equiv) and 2-(Bromomethyl)-4,4,5,5,-tetramethyl-1,3,2-dioxaborolane (0.25 mL, 1.5 mmol, 1.5 equiv) in acetonitrile (4.0 mL). Fluorination was performed with aqueous potassium hydrogen fluoride (3 M, 0.83 mL, 2.5 mmol, 2.5 equiv) and HCl (4 M in 1,4-dioxane, 0.25 mL, 1.0 mmol, 1.0 equiv) in 4:1 acetonitrile/water (0.10 M). The title compound was obtained as a beige solid (260 mg, 0.85 mmol, 85% yield). **^{1}H NMR** (500 MHz, Acetone-d6) δ 7.72 (dd, *J* = 8.5, 6.8 Hz, 1H), 6.96 (dd, *J* = 11.1, 2.6 Hz, 1H), 6.83 (td, *J* = 8.4, 2.6 Hz, 1H), 4.59 (s, 2H), 3.94 (s, 3H), 3.43 (dt, *J* = 13.5, 4.3 Hz, 2H), 3.17 (td, *J* = 12.1, 3.4 Hz, 2H), 2.34 (d, *J* = 6.1 Hz, 2H), 2.10 – 1.97 (m, 2H), 1.84 – 1.76 (m, 2H), 1.72 (dq, *J* = 12.2, 3.9 Hz, 1H), 1.53 – 1.40 (m, 1H); **^{13}C NMR** (126 MHz, Acetone-d6) δ 165.7 (d, *J* = 24.7 Hz), 161.83 (d, *J* = 10.5 Hz), 137.8 (dd, *J* = 10.5, 1.6 Hz), 114.3 (d, *J* = 3.4 Hz), 107.9 (d, *J* = 21.6 Hz), 100.6 (d, *J* = 26.1 Hz), 62.0, 60.8, 56.5, 22.0, 20.9; **^{19}F NMR** (376 MHz, Acetone-d6) δ −110.0, −138.5 (dd, *J* = 96.1, 45.5 Hz); **IR** (film, cm^{-1}): 2946, 1614,

1601, 1507, 1465, 1420, 1287, 1198, 1156, 1117, 1062, 1032, 1017, 1004, 953; **HRMS:** m/z calculated for $C_{14}H_{20}BF_3NO$ [M−F]$^+$ 286.1585; found 286.1587

((1-(3,5-di-*tert*-butylbenzyl)piperidin-1-ium-1-yl)methyl)trifluoroborate (**4af**)

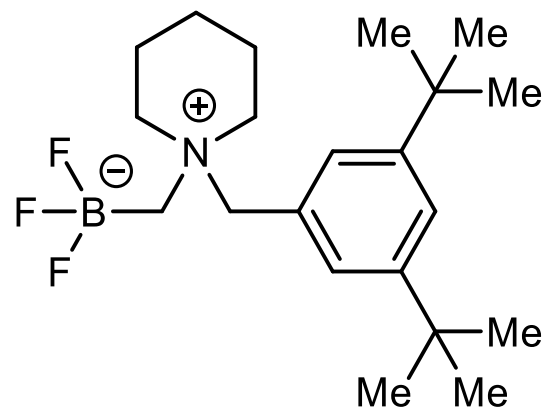

Prepared by general procedure **2.5.2.1. (method I)** using 1-(3,5-di-*tert*-butylbenzyl)piperidine (**1af**) (862 mg, 3.0 mmol, 1.0 equiv) and 2-(Bromomethyl)-4,4,5,5,-tetramethyl-1,3,2-dioxaborolane (994 mg, 4.5 mmol, 1.5 equiv) in acetonitrile (12 mL). Fluorination was performed with aqueous potassium hydrogen fluoride (3 M, 2.3 mL, 6.8 mmol, 2.3 equiv) and HCl (4 M in 1,4-dioxane, 1.1 mL, 4.5 mmol, 1.5 equiv) in 4:1 acetonitrile/water (0.20 M). The title compound was obtained as a white solid (621 mg, 1.7 mmol, 60% yield). **^{1}H NMR** (700 MHz, DMSO-d6) δ 7.47 (t, *J* = 1.8 Hz, 1H), 7.44 (d, *J* = 1.8 Hz, 2H), 4.49 (s, 2H), 3.20 (dt, *J* = 9.8, 4.5 Hz, 2H), 3.14 (ddd, *J* = 12.9, 9.3, 4.0 Hz, 2H), 2.13 (q, *J* = 4.8 Hz, 2H), 1.82 (dtt, *J* = 15.1, 10.1, 4.7 Hz, 2H), 1.61 (dt, *J* = 13.7, 4.9 Hz, 1H), 1.44 (td, *J* = 9.3, 4.6 Hz, 1H), 1.29 (s, 18H); **^{13}C NMR** (176 MHz, DMSO-d6) δ 150.4, 127.9, 127.5, 122.9, 60.0, 34.5, 31.1, 21.1, 19.6; **^{19}F NMR** (471 MHz, $CDCl_3$) δ −138.5; **IR** (neat, cm^{-1}): 2949, 2901, 2868, 1477, 1363, 1069, 1057, 1018, 1004, 985, 892; **HRMS**: m/z calculated for $C_{21}H_{35}BF_2N$ [M−F]$^+$ 350.2825; found 350.2826.

trifluoro((1-(naphthalen-2-ylmethyl)piperidin-1-ium-1-yl)methyl)borate (**4ag**)

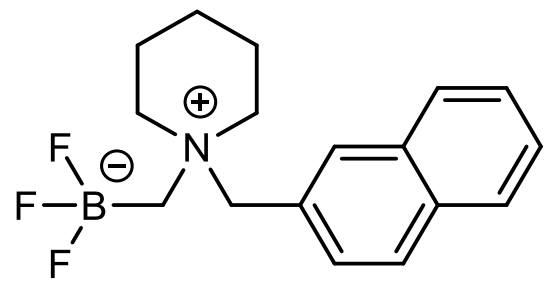

Prepared using general procedure **2.5.2.2. (method I)** using 1-(naphthalen-2-ylmethyl)piperidine (**1ag**) (225 mg, 1.0 mmol, 1.0 equiv) and potassium (bromomethyl)trifluoroborate (301 mg, 1.5 mmol, 1.5 equiv) in acetonitrile (1.0 mL). The title compound was obtained as a white solid (230 mg, 0.75 mmol, 75% yield). **^{1}H NMR** (700 MHz, DMSO-d6) δ 8.14 – 8.12 (m, 1H), 8.01 (d, *J* = 8.4 Hz, 1H), 7.99 – 7.95 (m, 2H), 7.68 (dd, *J* = 8.4, 1.8 Hz, 1H), 7.63 – 7.58 (m, 2H), 4.69 (s, 2H), 3.34 (m, 2H), 3.18 (ddd, *J* = 13.1, 9.9, 3.6 Hz, 2H), 2.23 (q, *J* = 4.7 Hz, 2H), 1.92 – 1.78 (m, 4H), 1.60 (dt, *J* = 13.6,

4.8 Hz, 1H), 1.45 – 1.38 (m, 1H); **^{13}C NMR** (176 MHz, DMSO-d6) δ 133.3, 133.1, 132.4, 129.8, 128.2, 128.0, 127.6, 127.3, 126.7, 126.4, 65.4, 59.7, 21.0, 19.6; **^{19}F NMR** (471 MHz, $CDCl_3$) δ −137.7; **IR** (neat, cm^{-1}): 2949, 2875, 1460, 1059, 1044, 1022, 996, 981, 935, 820, 757, 573, 480; **HRMS**: m/z calculated for $C_{17}H_{21}BF_2N$ [M−F]$^+$ 288.1730; found 288.1733.

trifluoro((1-(4-(piperidin-1-yl)benzyl)piperidin-1-ium-1-yl)methyl)borate (**4ah**)

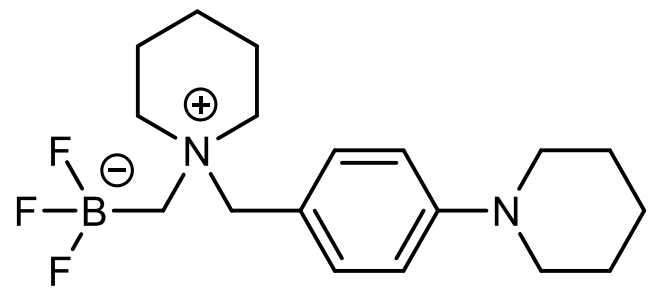

Prepared using general procedure **2.5.2.1. (method II)** using 1-(4-(piperidin-1-yl)benzyl)piperidine (**1ah**) (221 g, 1.0 mmol, 1.0 equiv) and 2-(Bromomethyl)-4,4,5,5,-tetramethyl-1,3,2-dioxaborolane (0.25 mL, 1.5 mmol, 1.5 equiv) in acetonitrile (4.0 mL). Fluorination was performed with aqueous potassium hydrogen fluoride (3 M, 0.83 mL, 2.5 mmol, 2.5 equiv) and HCl (4 M in 1,4-dioxane, 0.25 mL, 1.0 mmol, 1.0 equiv) in 4:1 acetonitrile/water (0.10 M). The title compound was obtained as a beige solid (317 mg, 0.93 mmol, 93% yield). **^{1}H NMR** (700 MHz, Acetone-d6) δ 7.47 – 7.44 (m, 2H), 6.99 – 6.95 (m, 2H), 4.50 (s, 2H), 3.38 (dt, *J* = 12.5, 4.4 Hz, 2H), 3.27 – 3.24 (m, 4H), 3.16 (ddd, *J* = 13.1, 10.6, 3.5 Hz, 2H), 2.25 (q, *J* = 4.6 Hz, 2H), 2.00 (qt, *J* = 10.6, 5.3 Hz, 2H), 1.85 (dt, *J* = 15.6, 4.5 Hz, 2H), 1.74 (dp, *J* = 14.1, 4.7 Hz, 1H), 1.68 – 1.64 (m, 4H), 1.60 (dddd, *J* = 10.5, 7.2, 4.1, 1.3 Hz, 2H), 1.55 – 1.48 (m, 1H); **^{13}C NMR** (176 MHz, Acetone-d6) δ 153.6, 135.1, 135.1, 118.4, 115.7, 67.1, 60.8, 49.9, 26.3, 25.0, 22.3, 20.9; **^{19}F NMR** (376 MHz, Acetone-d6) δ −138.5 (dd, *J* = 95.1, 44.7 Hz); **IR** (film, cm^{-1}): 2855, 1702, 1612, 1465, 1385, 1333, 1323, 1200, 1100, 948; **HRMS:** m/z calculated for $C_{18}H_{28}BF_3N_2Na$ [M+Na]$^+$ 363.2190; found 363.2190

trifluoro((1-(4-(2-oxopyrrolidin-1-yl)benzyl)piperidin-1-ium-1-yl)methyl)borate (**4ai**)

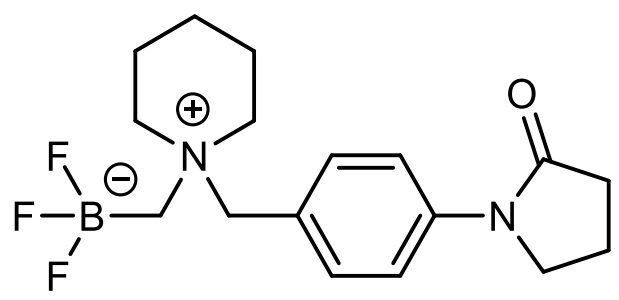

Prepared using general procedure **2.5.2.1. (method II)** using 1-(4-(piperidin-1-ylmethyl)phenyl)pyrrolidin-2-one (**1ai)** (388 mg, 1.5 mmol, 1.0 equiv) and 2-(Bromomethyl)-4,4,5,5,-tetramethyl-1,3,2-dioxaborolane (0.38 mL, 2.25 mmol, 1.5 equiv) in acetonitrile (6.0 mL). Fluorination was performed with aqueous potassium hydrogen fluoride (3 M, 1.3 mL, 3.8 mmol, 2.5 equiv) and HCl (4 M in 1,4-dioxane, 0.38 mL, 1.5 mmol, 1.0 equiv) in 4:1 acetonitrile/water (0.10 M). The title compound was obtained as a beige solid (460 mg, 1.4 mmol, 90% yield). **^{1}H NMR** (700

MHz, Acetone-d6) δ 7.86 (d, *J* = 8.4 Hz, 2H), 7.68 (d, *J* = 8.4 Hz, 2H), 4.64 (s, 2H), 3.99 – 3.94 (m, 2H), 3.45 (dt, *J* = 12.4, 4.5 Hz, 2H), 3.26 (ddd, *J* = 13.2, 10.5, 3.5 Hz, 2H), 2.58 – 2.55 (m, 2H), 2.30 (d, *J* = 5.2 Hz, 2H), 2.21 (p, *J* = 7.6 Hz, 2H), 2.08 (m, *J* = 3.2, 2.1 Hz, 2H), 1.91 (dt, *J* = 15.7, 4.5 Hz, 2H), 1.79 (dp, *J* = 14.5, 4.9 Hz, 1H), 1.58 (m, 1H); **^{13}C NMR** (176 MHz, Acetone-d6) δ 174.9, 142.4, 134.6, 124.6, 119.5, 66.6, 61.2, 48.9, 33.3, 22.2, 20.9, 18.5; **^{19}F NMR** (376 MHz, Acetone-d6) δ −138.5 (dd, *J* = 95.2, 43.2 Hz); **IR** (film, cm^{-1}): 2955, 2878, 1699, 1612, 1519, 1463, 1392, 1324, 1306, 1223, 1060, 1017, 1004, 909; **HRMS:** m/z calculated for $C_{17}H_{24}BF_2N_2O$ [M−F]$^+$ 321.1944; found 321.1947.

((4-((2,3-dihydrobenzo[*b*][1,4]dioxin-6-yl)methyl)morpholino-4-ium)methyl)trifluoroborate (**4aj**)

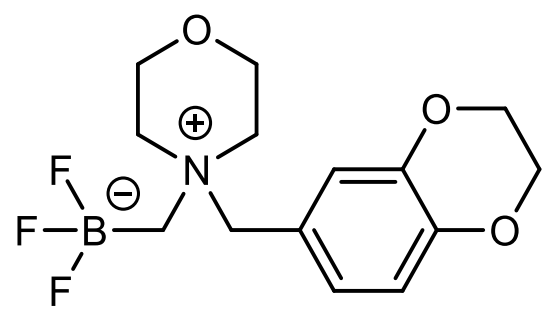

Prepared by general procedure **2.5.2.1. (method I)** using 4-((2,3-dihydrobenzo[*b*][1,4]dioxin-6-yl)methyl)morpholine (**1aj**) (284 mg, 1.2 mmol, 1.0 equiv) and 2-(Bromomethyl)-4,4,5,5,-tetramethyl-1,3,2-dioxaborolane (401 mg, 1.8 mmol, 1.5 equiv) in acetonitrile (4.8 mL). Fluorination was performed with aqueous potassium hydrogen fluoride (3 M, 0.60 mL, 2.7 mmol, 2.3 equiv) and HCl (4 M in 1,4-dioxane, 0.30 mL, 1.8 mmol, 1.5 equiv) in 4:1 acetonitrile/water (0.20 M). The title compound was obtained as a white solid (236 mg, 0.74 mmol, 62% yield). **^{1}H NMR** (500 MHz, DMSO-d6) δ 7.12 (d, *J* = 2.0 Hz, 1H), 7.04 (dd, *J* = 8.3, 2.1 Hz, 1H), 6.94 (d, *J* = 8.3 Hz, 1H), 4.48 (s, 2H), 4.30 – 4.23 (m, 4H), 3.97 – 3.85 (m, 4H), 3.35 – 3.26 (m, 2H), 3.21 (d, *J* = 12.9 Hz, 2H), 2.32 (q, *J* = 4.6 Hz, 2H); **^{13}C NMR** (176 MHz, DMSO-d6) δ 144.8, 143.1, 126.3, 122.0, 120.8, 117.1, 64.2, 64.0, 60.2, 58.6; **^{19}F NMR** (471 MHz, DMSO-d6) δ −134.1; **IR** (neat, cm^{-1}): 2937, 2041, 1511, 1294, 1263, 1121, 1067, 1049, 1028, 996, 984, 942, 924, 912, 874, 826; **HRMS**: m/z calculated for $C_{14}H_{19}BF_2NO_3$ [M−F]$^+$ 298.1421; found 298.1425.

((1-(3-(4,4-difluoropiperidine-1-carbonyl)benzyl)piperidin-1-ium-1-yl)methyl)trifluoroborate (**4ak**)

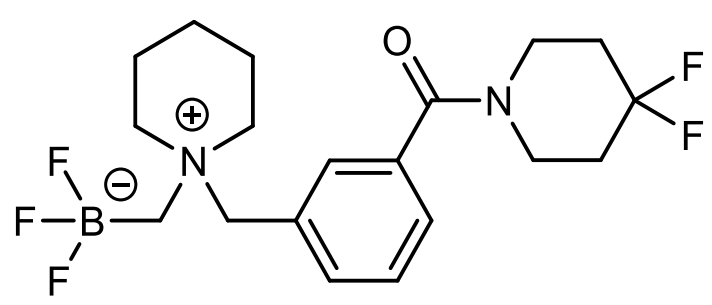

Prepared by general procedure **2.5.2.1. (method I)** using (4,4-difluoropiperidin-1-yl)(3-(piperidin-1-ylmethyl)phenyl)methanone (**1ak**) (322 mg, 1.0 mmol, 1.0 equiv) and 2-(Bromomethyl)-4,4,5,5,-tetramethyl-1,3,2-dioxaborolane (331 mg, 1.5 mmol, 1.5 equiv) in acetonitrile (4.0 mL). Fluorination was performed with aqueous potassium hydrogen fluoride (3 M, 0.80 mL, 2.3 mmol, 2.3 equiv) and HCl (4 M in 1,4-dioxane, 0.40 mL, 1.5 mmol, 1.5 equiv) in 4:1 acetonitrile/water (0.20 M). The title compound was obtained as a white solid (230 mg, 0.57 mmol, 57% yield). **^{1}H NMR** (400 MHz, CD_3CN) δ 7.59 (dt, *J* = 7.1, 1.9 Hz, 1H), 7.56 – 7.49 (m, 3H), 4.26 (d, *J* = 4.9 Hz, 2H), 3.88 – 3.33 (m, 6H), 2.91 (tdd, *J* = 12.4, 9.5, 3.2 Hz, 2H), 2.30 – 2.21 (m, 2H), 2.14 – 1.95 (br s, 4H), 1.86 (dt, *J* = 15.5, 3.7 Hz, 2H), 1.80 – 1.63 (m, 3H), 1.51 – 1.37 (m, 1H); **^{13}C NMR** (176 MHz, CD_3CN) δ 170.1, 137.6, 133.5, 130.4, 130.3, 130.3, 129.2, 123.3 (t, *J* = 241 Hz), 60.9, 53.9, 45.1, 39.6, 34.6, 34.1, 23.5, 22.0; **^{19}F NMR** (376 MHz, CD_3CN) δ −98.0, −143.2 (dd, *J* = 103.9, 51.7 Hz); **IR** (neat, cm^{-1}) 2969, 1605, 1457, 1364, 1260, 1101, 979, 951, 746; **HRMS:** m/z calculated for $C_{19}H_{26}BF_5N_2$ $[M]^+$ 404.2045; found 404.2053.

1-((trifluoro-l4-boraneyl)methyl)-1-(4-(3-(trifluoromethyl)-1H-pyrazol-1-yl)benzyl)-1l4-piperidine (**4ai**)

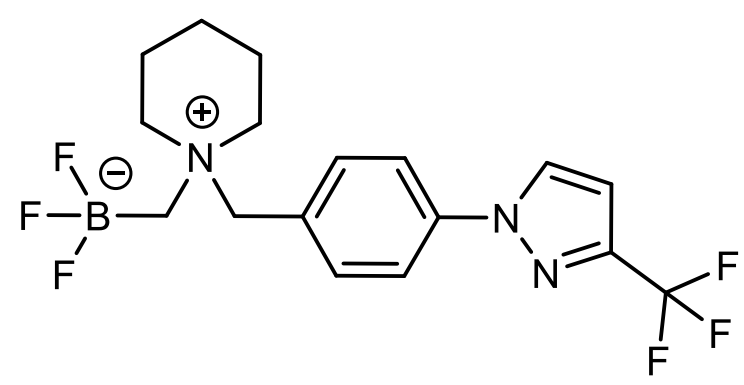

Prepared by general procedure **2.5.2.1. (method II)** using 1-(4-(3-(trifluoromethyl)-1H-pyrazol-1-yl)benzyl)piperidine (**1ai)** (309 mg, 1.0 mmol, 1.0 equiv) and 2-(Bromomethyl)-4,4,5,5,-tetramethyl-1,3,2-dioxaborolane (0.25 mL, 1.5 mmol, 1.5 equiv) in acetonitrile (4.0 mL). Fluorination was performed with aqueous potassium hydrogen fluoride (3 M, 0.83 mL, 2.5 mmol, 2.5 equiv) and HCl (4 M in 1,4-dioxane, 0.25 mL, 1.0 mmol, 1.0 equiv) in 4:1 acetonitrile/water (0.10 M). The title compound was obtained as beige crystals (402 mg, 1.0 mmol, 99% yield). **^{1}H NMR** (700 MHz, Acetone-d6) δ 8.61 (dd, *J* = 2.5, 1.1 Hz, 1H), 8.01 – 7.98 (m, 2H), 7.91 – 7.87 (m, 2H), 6.96 (d, *J* = 2.6 Hz, 1H), 4.72 (s, 2H), 3.47 (dt, *J* = 12.3, 4.4 Hz, 2H), 3.32 (ddd, *J* = 13.0, 10.7, 3.5 Hz, 2H), 2.31 (q, *J* = 4.6 Hz, 2H), 2.08 – 2.00 (m, 2H), 1.89 (dp, *J* = 13.7, 4.3 Hz, 2H), 1.77 (dp, *J* = 14.0, 4.7 Hz, 1H), 1.57zz (m, 1H); **^{13}C NMR** (176 MHz, Acetone-d6) δ 144.5 (q, *J* = 38.1 Hz), 141.2, 135.8 (q, *J* = 1.2 Hz), 130.8, 129.3, 122.4 (q, *J* = 267.9 Hz), 120.2, 107.2 (d, *J* = 2.1 Hz), 66.3, 61.6 (d, *J* = 2.0 Hz), 22.2, 20.9; **^{19}F NMR** ^{19}F NMR (376 MHz, Acetone-d6) δ −63.7, −138.4 (q, *J* = 79.9 Hz); **IR** (film, cm^{-1}): 2968, 1518, 1392, 1279, 1131, 1054, 1005, 971, 910; **HRMS:** m/z calculated for $C_{17}H_{20}BF_5N3$ $[M - F]^+$ 372.1665; found 372.1665.

2-(3-((1-((trifluoro-l4-boraneyl)methyl)-1l4-piperidin-1-yl)methyl)phenyl)pyridine (**4am**)

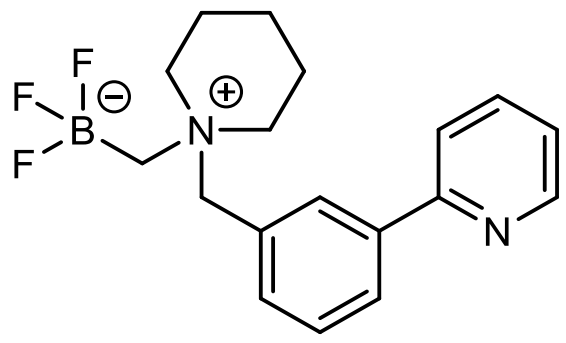

Prepared by general procedure **2.5.2.1. (method II)** using 2-(3-(piperidin-1-ylmethyl)phenyl)pyridine (**1am**) (252 mg, 1.0 mmol, 1.0 equiv) and 2-(Bromomethyl)-4,4,5,5,-tetramethyl-1,3,2-dioxaborolane (0.19 mL, 1.1 mmol, 1.1 equiv) in acetonitrile (4.0 mL). Fluorination was performed with aqueous potassium hydrogen fluoride (3 M, 0.83 mL, 2.5 mmol, 2.5 equiv) and HCl (4 M in 1,4-dioxane, 0.25 mL, 1.0 mmol, 1.0 equiv) in 4:1 acetonitrile/water (0.10 M). The title compound was obtained as a white solid (223 mg, 0.67 mmol, 67% yield). **^{1}H NMR** (700 MHz, Acetone-d6) δ 8.69 (ddd, *J* = 4.8, 1.9, 1.0 Hz, 1H), 8.40 (t, *J* = 1.8 Hz, 1H), 8.26 (ddd, *J* = 7.9, 1.8, 1.1 Hz, 1H), 7.98 (dt, *J* = 8.0, 1.1 Hz, 1H), 7.89 (td, *J* = 7.7, 1.8 Hz, 1H), 7.76 (dt, *J* = 7.6, 1.5 Hz, 1H), 7.60 (t, *J* = 7.7 Hz, 1H), 7.36 (ddd, *J* = 7.5, 4.7, 1.1 Hz, 1H), 4.75 (s, 2H), 3.50 (dt, *J* = 12.6, 4.3 Hz, 2H), 3.32 (ddd, *J* = 12.6, 10.7, 3.5 Hz, 2H), 2.36 (q, *J* = 4.5 Hz, 2H), 2.09 – 2.02 (m, 2H), 1.90 (dt, *J* = 15.5, 4.4 Hz, 2H), 1.77 (dp, *J* = 13.8, 4.6 Hz, 1H), 1.62 – 1.49 (m, 1H); **^{13}C NMR** (176 MHz, Acetone-d6) δ 156.9, 150.6, 140.6, 138.0, 134.7 (t, *J* = 1.1 Hz), 132.6 (q, *J* = 1.1 Hz), 130.4, 129.9, 128.9, 123.6, 121.2, 67.3, 61.5 (d, *J* = 2.6 Hz), 22.2, 20.9; **^{19}F NMR** (471 MHz, Acetone-d6) δ −137.3 (dd, *J* = 97.7, 42.6 Hz); **IR** (film, cm^{-1}): 2876, 1585, 1438, 1247, 1051, 1003, 957, 933; **HRMS:** m/z calculated for $C_{18}H_{23}BF_3N_2O$ [M+H]$^+$ 335.1901; found 335.1902

2-methyl-6-((1-((trifluoro-l4-boraneyl)methyl)-1l4-piperidin-1-yl)methyl)quinoline (**4an**)

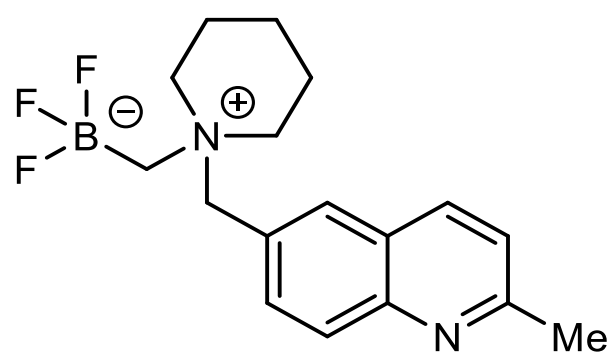

Prepared by general procedure **2.5.2.1. (method II)** using 2-methyl-6-(piperidin-1-ylmethyl)quinoline (**1an**) (240 mg, 1.0 mmol, 1.0 equiv) and 2-(Bromomethyl)-4,4,5,5,-tetramethyl-1,3,2-dioxaborolane (0.25 mL, 1.5 mmol, 1.5 equiv) in acetonitrile (4.0 mL). Monitoring by ^{11}B NMR showed a single peak at 31.1 ppm. Fluorination was performed with aqueous potassium hydrogen fluoride (3 M, 0.83 mL, 2.5 mmol, 2.5 equiv) and HCl (4 M in 1,4-dioxane, 0.25 mL, 1.0 mmol, 1.0 equiv) in 4:1 acetonitrile/water (0.10 M). The title compound was obtained as a brown solid (240 mg, 0.75 mmol, 75% yield). **^{1}H NMR** (700 MHz, Acetone-d6) δ 8.2 (d, *J* = 8.4 Hz, 1H), 8.2 (s, 1H), 8.0 (d, *J* = 8.7 Hz, 1H), 8.0 (dt, *J* = 8.8, 1.6 Hz, 1H), 7.5 (d, *J* = 8.4 Hz, 1H), 4.8 (s, 2H), 3.5 – 3.4 (m, 2H), 3.3 (td, *J* =

12.1, 3.7 Hz, 2H), 2.8 (d, J = 22.7 Hz, 5H), 2.7 (d, J = 1.4 Hz, 3H), 2.3 (d, J = 6.2 Hz, 2H), 2.1 – 2.0 (m, 3H), 1.9 (d, J = 15.2 Hz, 2H), 1.8 (dt, J = 13.9, 4.8 Hz, 1H), 1.5 (td, J = 12.7, 8.6 Hz, 1H); **^{13}C NMR** (176 MHz, Acetone-d6) δ 161.4, 149.0, 137.3, 134.5, 134.2 (d, J = 1.2 Hz), 134.2, 129.8, 127.1, 127.0, 123.5, 67.0, 61.4, 25.4, 22.2, 20.9.; **^{19}F NMR** (376 MHz, Acetone-d6) δ 138.3 (dd, J = 95.6, 43.1 Hz); **IR** (film, cm^{-1}): 2967, 2647, 1700, 1603, 1502, 1469, 1455, 1062, 1023, 1007, 937; **HRMS:** m/z calculated for $C_{17}H_{23}BF_3N_2$ [M+H]$^+$ 323.1901; found 323.1902.

Trifluoro((1-((6-(trifluoromethyl)pyridin-3-yl)methyl)piperidin-1-ium-1-yl)methyl)borate (**4ao**)

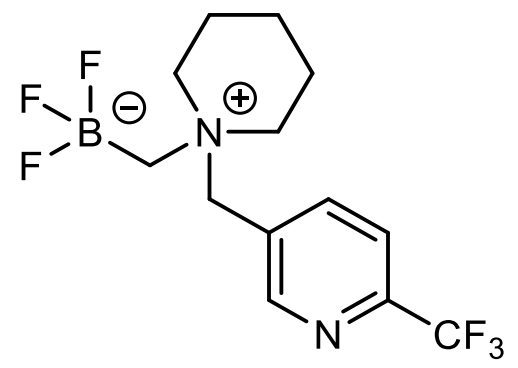

Prepared by general procedure **2.5.2.1. (method II)** using 5-(piperidin-1-ylmethyl)-2-(trifluoromethyl)pyridine (**1ao)** (488 mg, 2.0 mmol, 1.0 equiv) and 2-(Bromomethyl)-4,4,5,5,-tetramethyl-1,3,2-dioxaborolane (0.41 mL, 2.4 mmol, 1.2 equiv) in acetonitrile (6.0 mL). Fluorination was performed with aqueous potassium hydrogen fluoride (3 M, 1.7 mL, 5.0 mmol, 2.5 equiv) and HCl (4 M in 1,4-dioxane, 0.90 mL, 3.6 mmol, 1.8 equiv) in 4:1 acetonitrile/water (0.10 M). The title compound was obtained as a white solid (610 mg, 1.9 mmol, 94% yield). **^{1}H NMR** (700 MHz, DMSO-d6) δ 8.92 (d, J = 2.0 Hz, 1H), 8.29 (dd, J = 8.1, 2.1 Hz, 1H), 8.06 (d, J = 8.1 Hz, 1H), 4.68 (s, 2H), 3.32 (dt, J = 12.4, 4.5 Hz, 2H), 3.19 (ddd, J = 12.9, 10.2, 3.6 Hz, 2H), 2.17 (q, J = 4.7 Hz, 2H), 1.87 (tdd, J = 14.3, 7.9, 3.9 Hz, 2H), 1.79 (dt, J = 15.5, 4.4 Hz, 2H), 1.62 (dp, J = 14.1, 4.8 Hz, 1H), 1.44 (dtt, J = 14.6, 9.8, 4.2 Hz, 1H); **^{13}C NMR** (176 MHz, DMSO-d6) δ 153.7, 147.2 (q, J = 34.2 Hz), 143.0 (d, J = 1.4 Hz), 128.8, 121.5 (q, J = 274.2 Hz), 120.8 (q, J = 2.6 Hz), 66.3, 61.9, 60.2 (d, J = 1.9 Hz), 20.2 (d, J = 236.2 Hz); **^{19}F NMR** (471 MHz, DMSO-d6) δ −66.7, −134.1; **IR** (film, cm^{-1}): 2953, 1470, 1339, 1173, 1139, 1088, 1062, 1019, 979, 915; **HRMS:** m/z calculated for $C_{13}H_{17}BF_5N_2$ [M−F]$^+$ 307.1400; found 307.1402.

trifluoro((1-((6-phenylpyridin-3-yl)methyl)piperidin-1-ium-1-yl)methyl)borate (**4ap**)

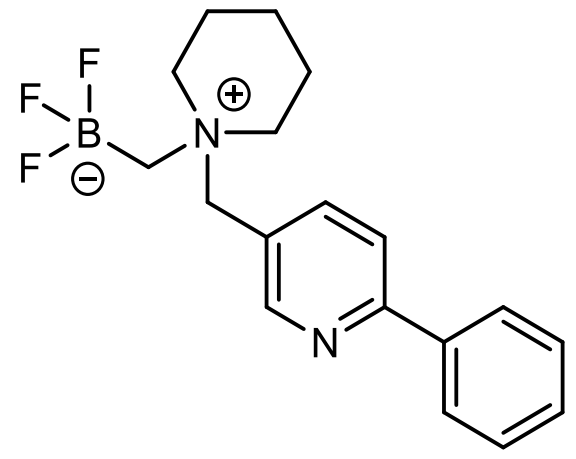

Prepared by general procedure **2.5.2.1. (method I)** using 2-phenyl-5-(piperidin-1-ylmethyl)pyridine (**1ap**) (167 mg, 0.70 mmol, 1.0 equiv) and 2-(Bromomethyl)-4,4,5,5,-tetramethyl-1,3,2-dioxaborolane (219 mg, 0.99 mmol, 1.5 equiv) in acetonitrile (2.7 mL). Monitoring by ^{11}B NMR showed peaks at 31.0 and 22.5 ppm. Fluorination was performed with aqueous potassium hydrogen fluoride (3 M, 0.50 mL, 2.3 mmol, 2.3 equiv) and HCl (4 M in 1,4-dioxane, 0.20 mL, 1.0 mmol, 1.5 equiv) in 4:1 acetonitrile/water (0.20 M). The title compound was obtained as a yellow solid (115 mg, 0.34 mmol, 52% yield). **^{1}H NMR** (700 MHz, $CDCl_3$) δ 8.72 (dd, *J* = 2.5, 0.9 Hz, 1H), 8.18 (dd, *J* = 8.3, 2.4 Hz, 1H), 8.04 – 7.99 (m, 2H), 7.85 (dd, *J* = 8.2, 0.9 Hz, 1H), 7.51 – 7.45 (m, 3H), 4.62 (s, 2H), 3.49 (dt, *J* = 11.8, 4.4 Hz, 2H), 3.11 (ddd, *J* = 13.2, 10.4, 3.4 Hz, 2H), 2.43 (q, *J* = 4.6 Hz, 2H), 2.09 (tdd, *J* = 14.5, 7.9, 3.9 Hz, 2H), 1.85 (ddt, *J* = 42.3, 13.9, 4.6 Hz, 3H), 1.55 (dtd, *J* = 14.0, 10.4, 5.0 Hz, 1H); **^{13}C NMR** (176 MHz, $CDCl_3$) δ 159.3, 153.0, 142.1 (d, *J* = 1.7 Hz), 138.1, 130.0, 129.1, 127.2, 122.2, 120.7, 61.5 (d, *J* = 2.1 Hz), 21.6, 20.4; **^{19}F NMR** (471 MHz, $CDCl_3$) δ −138.0. **IR** (neat, cm^{-1}): 2939, 1478, 1060, 1016, 997, 982, 930, 910, 884, 751, 701; **HRMS**: m/z calculated for $C_{18}H_{22}BF_2N_2$ [M−F]$^+$ 315.1839; found 315.1840.

trifluoro((1-((2-(m-tolyl)pyrimidin-5-yl)methyl)piperidin-1-ium-1-yl)methyl)borate (**4aq**)

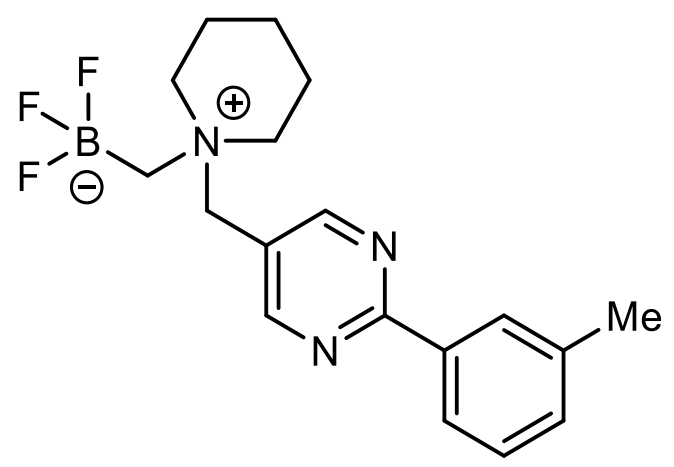

Prepared by general procedure **2.5.2.1. (method II)** using 5-(piperidin-1-ylmethyl)-2-(*m*-tolyl)pyrimidine (**1aq**) (267 mg, 1.0 mmol, 1.0 equiv) and 2-(Bromomethyl)-4,4,5,5,-tetramethyl-1,3,2-dioxaborolane (0.18 mL, 1.1 mmol, 1.1 equiv) in acetonitrile (4.0 mL). Monitoring by ^{11}B NMR showed a single peak at 31.2 ppm. Fluorination was performed with aqueous potassium hydrogen fluoride (3 M, 0.83 mL, 2.5 mmol, 2.5 equiv) and HCl (4 M in 1,4-dioxane, 0.50 mL, 2.0 mmol, 2.0 equiv) in 4:1 acetonitrile/water (0.10 M). The title compound was obtained as a white solid (186 mg, 0.53 mmol, 53% yield). **^{1}H NMR** (700 MHz, Acetone-d6) δ 9.13 (s, 2H), 8.36 (tt, *J* = 1.7, 0.8 Hz, 1H), 8.32 (dd, *J* = 7.6, 1.7 Hz, 1H), 7.42 (t, *J* = 7.6 Hz, 1H), 7.38 (dq, *J* = 7.4, 1.2 Hz, 1H), 4.79 (s, 2H), 3.59 – 3.53 (m, 2H), 3.42 – 3.36 (m, 2H), 2.44 (s, 3H), 2.33 (s, 2H), 2.09 (tq, *J* = 10.9, 4.0 Hz, 2H), 1.96 – 1.89 (m, 2H), 1.81 (dp, *J* = 13.8, 4.5 Hz, 1H), 1.65 – 1.56 (m, 1H); **^{13}C NMR** (176 MHz, Acetone-d6) δ 165.6, 162.1 (q, *J* = 1.5 Hz), 139.1, 137.9, 132.9, 129.7, 129.4, 126.4, 122.2, 62.2, 61.8 (d, *J* = 2.1 Hz); **^{19}F NMR** (471 MHz, Acetone-d6) δ −137.2 (dd, *J* = 96.5, 43.6 Hz); **IR** (film, cm^{-1}):

2949, 1590, 1545, 1440, 1062, 1040, 1020, 1008, 934, 913, 883; **HRMS:** m/z calculated for $C_{18}H_{23}BF_3N_3$ [M−F]$^+$ 350.2009; found 350.2011.

trifluoro((1-((2-phenylthiazol-4-yl)methyl)piperidin-1-ium-1-yl)methyl)borate (**4ar**)

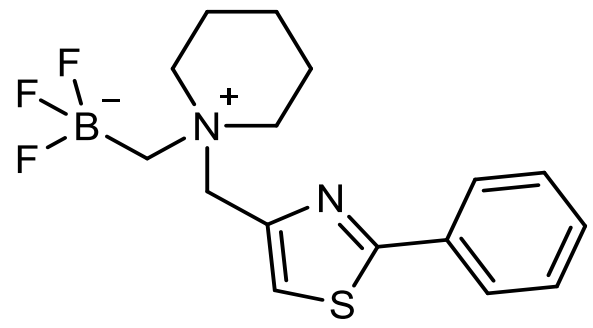

Prepared by general procedure **2.5.2.1. (method II)** using 2-phenyl-4-(piperidin-1-ylmethyl)thiazole (**1ar**) (267 mg, 1.0 mmol, 1.0 equiv) and 2-(Bromomethyl)-4,4,5,5,-tetramethyl-1,3,2-dioxaborolane (0.18 mL, 1.1 mmol, 1.1 equiv) in acetonitrile (4.0 mL). Monitoring by ^{11}B NMR showed a single peak at 31.0 ppm. Fluorination was performed with aqueous potassium hydrogen fluoride (3 M, 0.83 mL, 2.5 mmol, 2.5 equiv) and HCl (4 M in 1,4-dioxane, 0.25 mL, 1.0 mmol, 1.0 equiv) in 4:1 acetonitrile/water (0.10 M). The title compound was obtained as a beige solid (226 mg, 0.67 mmol, 67% yield). **^{1}H NMR** (700 MHz, CD_2Cl_2) δ 7.97 – 7.93 (m, 2H), 7.87 (s, 1H), 7.49 – 7.46 (m, 3H), 4.66 (s, 2H), 3.39 (dhept, *J* = 17.6, 4.6 Hz, 4H), 2.36 (q, *J* = 4.7 Hz, 2H), 1.99 (hd, *J* = 10.1, 6.0 Hz, 4H), 1.75 – 1.68 (m, 1H), 1.64 (tdd, *J* = 13.3, 8.5, 5.6 Hz, 1H); **^{13}C NMR** (176 MHz, CD_2Cl_2) δ 168.9, 145.6, 133.4, 130.9, 129.4, 126.9, 125.8f (q, *J* = 2.4 Hz), 62.3 (d, *J* = 2.3 Hz), 59.9, 21.8, 20.7; **^{19}F NMR** (471 MHz, CD_2Cl_2) δ −137.9 (m); **IR** (film, cm^{-1}): 3121, 2947, 2878, 1464, 1440, 1060, 1006, 954, 767; **HRMS:** m/z calculated for $C_{16}H_{20}BF_2N_2S$ [M−F]$^+$ 321.1403; found 321.1405.

(((2,4-dimethoxybenzyl)(methyl)(3-(trifluoromethyl)benzyl)ammonio)methyl)trifluoroborate (**4as**)

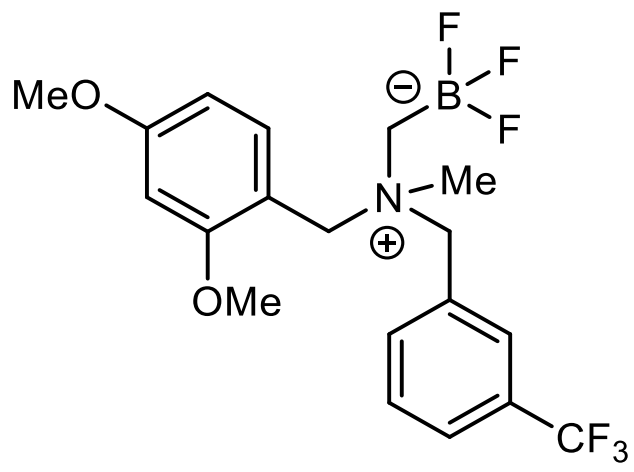

Prepared by general procedure **2.5.2.1. (method I)** using *N*-(2,4-dimethoxybenzyl)-*N*-methyl-1-(3-(trifluoromethyl)phenyl)methanamine (**1as**) (679 mg, 2.0 mmol, 1.0 equiv) and 2-(Bromomethyl)-4,4,5,5-tetramethyl-1,3,2-dioxaborolane (663 mg, 3.0 mmol, 1.5 equiv) in acetonitrile (8.0 mL). Fluorination was performed with aqueous potassium hydrogen fluoride (3 M, 1.5 mL, 4.5 mmol, 2.3 equiv) and HCl (4 M in 1,4-dioxane, 0.75 mL, 3.0 mmol, 1.5 equiv) in 4:1 acetonitrile/water (0.2 M). The title compound was obtained as a white solid (507 mg, 1.2 mmol, 60% yield). **^{1}H NMR** (500 MHz, $CDCl_3$) δ 7.85 (dd, *J* = 7.9, 1.7 Hz, 1H), 7.75 (ddt, *J* = 7.7, 1.8, 1.0 Hz, 1H), 7.66 (d, *J* = 1.9 Hz,

1H), 7.61 (t, J = 7.8 Hz, 1H), 7.45 (d, J = 8.5 Hz, 1H), 6.57 (dd, J = 8.5, 2.4 Hz, 1H), 6.52 (d, J = 2.4 Hz, 1H), 4.61 (dd, J = 19.9, 12.9 Hz, 2H), 4.47 (d, J = 12.9 Hz, 1H), 4.35 (d, J = 12.9 Hz, 1H), 3.85 (s, 3H), 3.82 (s, 3H), 2.75 (s, 3H), 2.38 (tt, J = 13.9, 7.0 Hz, 2H); **^{13}C NMR** (176 MHz, $CDCl_3$) δ 163.2, 160.1, 137.0, 136.3, 131.6 (q, J = 32.8 Hz), 130.0, 129.67, 129.65, 127.3 (q, J = 3.7 Hz), 123.7 (q, J = 272.5 Hz), 108.7, 105.4, 99.2, 66.3, 62.9, 55.8, 55.7, 49.2; **^{19}F NMR** (471 MHz, $CDCl_3$) δ –62.7, –137.5; **IR** (film, cm^{-1}): 2974, 1511, 1467, 1331, 1211, 1124, 1027, 982, 706; **HRMS:** m/z calculated for $C_{19}H_{22}BF_5NO_2$ $[M–F]^+$ 402.1658; found 402.1661.

(((3,5-difluorobenzyl)(2,4-dimethoxybenzyl)(methyl)ammonio)methyl)trifluoroborate (**4at**)

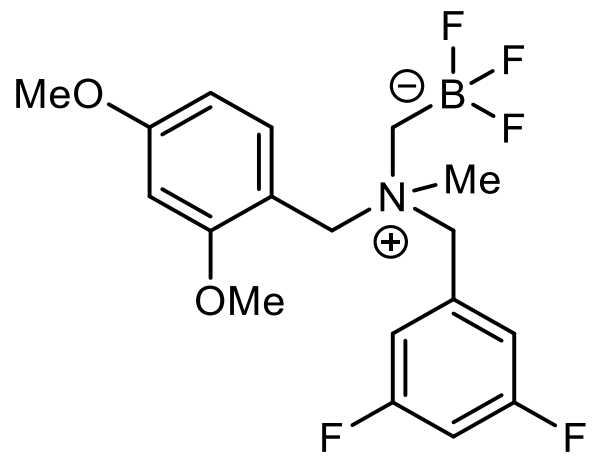

Prepared by general procedure **2.5.2.1. (method I)** using *N*-(3,5-difluorobenzyl)-1-(2,4-dimethoxyphenyl)-*N*-methylmethanamine (**1at**) (615 mg, 2.0 mmol, 1.0 equiv) and 2-(Bromomethyl)-4,4,5,5-tetramethyl-1,3,2-dioxaborolane (663 mg, 3.0 mmol, 1.5 equiv) in acetonitrile (8.0 mL). Fluorination was performed with aqueous potassium hydrogen fluoride (3 M, 1.5 mL, 4.5 mmol, 2.3 equiv) and HCl (4 M in 1,4-dioxane, 0.75 mL, 3.0 mmol, 1.5 equiv) in 4:1 acetonitrile/water (0.2 M). The title compound was obtained as a white solid (481 mg, 1.2 mmol, 62% yield). **^{1}H NMR** (500 MHz, $CDCl_3$) δ 7.44 (d, J = 8.5 Hz, 1H), 7.11 (h, J = 5.0 Hz, 2H), 6.94 (tt, J = 8.7, 2.3 Hz, 1H), 6.57 (dd, J = 8.5, 2.4 Hz, 1H), 6.51 (d, J = 2.4 Hz, 1H), 4.56 (dd, J = 12.9, 4.7 Hz, 2H), 4.44 (d, J = 12.9 Hz, 1H), 4.28 (d, J = 12.9 Hz, 1H), 3.85 (s, 3H), 3.83 (s, 3H), 2.76 (s, 3H), 2.41 (h, J = 4.6 Hz, 2H); **^{13}C NMR** (126 MHz, $CDCl_3$) δ 163.2, 163.1 (dd, J = 251.9, 12.6 Hz), 160.1, 136.3, 131.9 (t, J = 9.3 Hz), 108.6, 106.2 (t, J = 24.9 Hz), 116.4 (dd, J = 19.1, 6.7 Hz), 105.4, 99.2, 65.9, 63.1, 55.8, 55.7, 49.5; **^{19}F NMR** (471 MHz, $CDCl_3$) δ –107.4, –137.5; **IR** (film, cm^{-1}): 2943, 1599, 1459, 1295, 1213, 1122, 1025, 971, 861, 731; **HRMS:** m/z calculated for $C_{18}H_{21}BF_4NO_2$ $[M–F]^+$ 370.1596; found 370.1601.

(((2,4-dimethoxybenzyl)(methyl)((6-(trifluoromethyl)pyridin-3-yl)methyl)ammonio)methyl)trifluoroborate (**4au**)

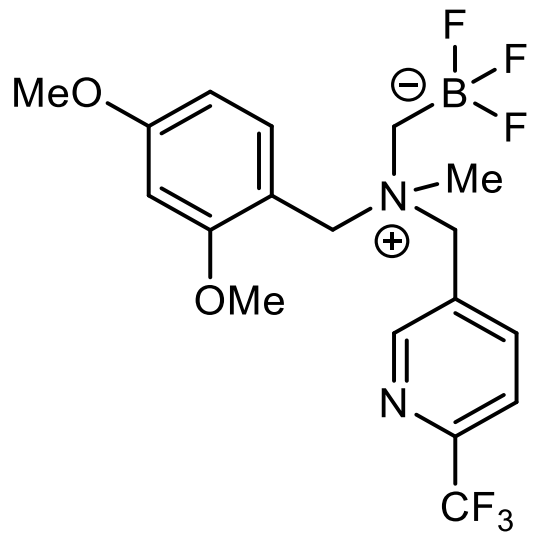

Prepared by general procedure **2.5.2.1. (method I)** using *N*-(2,4-dimethoxybenzyl)-*N*-methyl-1-(6-(trifluoromethyl)pyridin-3-yl)methanamine (**1au**) (681 mg, 2.0 mmol, 1.0 equiv) and 2-(Bromomethyl)-4,4,5,5-tetramethyl-1,3,2-dioxaborolane (663 mg, 3.0 mmol, 1.5 equiv) in acetonitrile (8.0 mL). Fluorination was performed with aqueous potassium hydrogen fluoride (3 M, 1.5 mL, 4.5 mmol, 2.3 equiv) and HCl (4 M in 1,4-dioxane, 0.75 mL, 3.0 mmol, 1.5 equiv) in 4:1 acetonitrile/water (0.2 M). The title compound was obtained as a white solid (539 mg, 1.3 mmol, 64% yield). **^{1}H NMR** (500 MHz, $CDCl_3$) δ 8.73 (d, *J* = 2.2 Hz, 1H), 8.43 – 8.38 (m, 1H), 7.82 (d, *J* = 8.2, 1H), 7.45 (d, *J* = 8.5 Hz, 1H), 6.59 (dd, *J* = 8.5, 2.4 Hz, 1H), 6.54 (d, *J* = 2.4 Hz, 1H), 4.78 (d, *J* = 13.0 Hz, 1H), 4.63 (d, *J* = 12.9 Hz, 1H), 4.44 (dd, *J* = 22.4, 12.9 Hz, 2H), 3.86 (s, 3H), 3.85 (s, 3H), 2.76 (s, 3H), 2.36 (q, *J* = 4.5 Hz, 2H); **^{13}C NMR** (176 MHz, $CDCl_3$) δ 163.4, 160.1, 153.3, 149.9 (q, *J* = 35.8 Hz), 143.0, 136.3, 128.1, 121.2 (q, *J* = 274.1 Hz), 121.0 (q, *J* = 2.7 Hz), 108.2, 105.6, 99.3, 63.5, 63.1, 55.9, 55.7, 49.7; **^{19}F NMR** (471 MHz, $CDCl_3$) δ –68.2, –137.1; **IR** (film, cm^{-1}): 2973, 1612, 1599, 1511, 1461, 1321, 1295, 1211, 1122, 1025, 971, 861, 732; **HRMS:** m/z calculated for $C_{18}H_{21}BF_6N_2O_2Na$ $[M+Na]^+$ 445.1498; found 445.1486.

(((3,5-bis(trifluoromethyl)benzyl)(4-methoxybenzyl)(methyl)ammonio)methyl)trifluoroborate (**4av**)

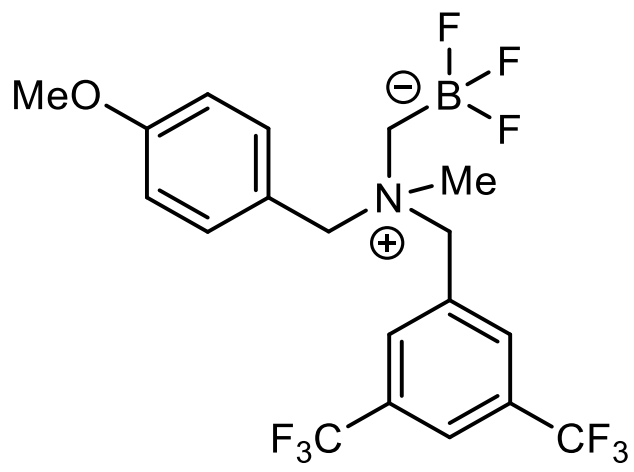

Prepared by general procedure **2.5.2.1. (method I)** using *N*-(3,5-bis(trifluoromethyl)benzyl)-1-(4-methoxyphenyl)-*N*-methylmethanamine (**1av**) (755 mg, 2.0 mmol, 1.0 equiv) and 2-(Bromomethyl)-4,4,5,5-tetramethyl-1,3,2-dioxaborolane (663 mg, 3.0 mmol, 1.5 equiv) in acetonitrile (8.0 mL). Fluorination was performed with aqueous potassium hydrogen fluoride (3 M, 1.5 mL, 4.5 mmol, 2.3 equiv) and HCl (4 M in 1,4-dioxane, 0.75 mL, 3.0 mmol, 1.5 equiv) in 4:1 acetonitrile/water (0.2 M).

The title compound was obtained as a white solid (542 mg, 1.2 mmol, 59% yield). **^{1}H NMR** (400 MHz, $CDCl_3$) δ 8.16 (s, 2H), 7.98 (s, 1H), 7.50 (d, *J* = 8.7 Hz, 2H), 6.95 (d, *J* = 8.7 Hz, 2H), 4.77 (d, *J* = 12.8 Hz, 1H), 4.61 (d, *J* = 12.9 Hz, 1H), 4.56 (s, 2H), 3.83 (s, 3H), 2.85 (s, 3H), 2.35 (dhept, *J* = 9.6, 4.7 Hz, 2H); **^{13}C NMR** (126 MHz, $CDCl_3$) δ 161.6, 134.8, 133.5 (br. s), 132.9 (d, *J* = 34.0 Hz), 131.0, 124.6 (br s), 122.8 (d, *J* = 272.9 Hz), 119.2, 114.8, 68.8, 65.7, 55.6, 49.8; **^{19}F NMR** (471 MHz, $CDCl_3$) δ –62.9, –136.2; **IR** (film, cm^{-1}): 2976, 1517, 1370, 1279, 1129, 1027, 900, 684; **HRMS:** m/z calculated for $C_{19}H_{21}BF_9NO_2$ $[M+H_2O]^+$ 477.1522; found 477.1788.

((1-Benzyl-4-((5,6-dimethoxy-1-oxo-2,3-dihydro-1*H*-inden-2-yl)methyl)piperidin-1-ium-1-yl)methyl)trifluoroborate

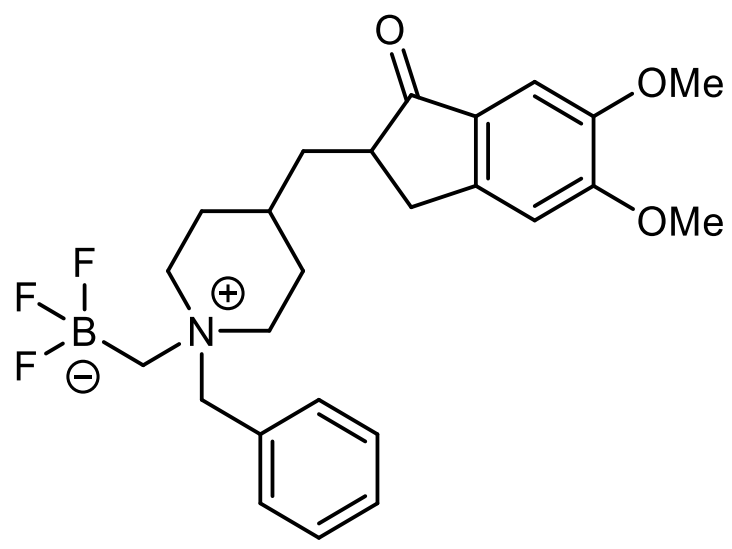

Prepared by general procedure **2.5.2.1. (method I)** using donepezil (569 mg, 1.5 mmol, 1.0 equiv) and 2-(Bromomethyl)-4,4,5,5,-tetramethyl-1,3,2-dioxaborolane (0.38 mL, 2.3 mmol, 1.5 equiv) in acetonitrile (6.0 mL). Fluorination was performed with aqueous potassium hydrogen fluoride (3 M, 1.3 mL, 3.8 mmol, 2.5 equiv) and HCl (4 M in 1,4-dioxane, 0.38 mL, 1.5 mmol, 1.0 equiv) in 4:1 acetonitrile/water (0.10 M). The title compound was obtained as an off-white solid (637 mg, 1.4 mmol, 92% yield, 2:1 d.r.). **^{1}H NMR** (700 MHz, DMSO-d6) δ 7.62 – 7.57 (m, 2H), 7.52 – 7.46 (m, 3H), 7.11 (s, 1H, minor), 7.08 (s, 1H, major), 7.08 (s, 1H, minor), 7.05 (s, 1H), 4.56 (s, 2H minor), 4.53 (s, 2H, major), 3.87 (s, 3H, minor), 3.86 (s, 3H, major), 3.79 (s, 3H, minor), 3.78 (s, 3H, major), 3.40 – 3.36 (m, 1H), 3.31 – 3.15 (m, 4H), 2.75 – 2.68 (m, 2H, minor), 2.68 – 2.62 (m, 2H, major), 2.21 (br. q, *J* = 4.5 Hz, 2H, minor), 2.10 (br. q, *J* = 4.8 Hz, 2H), 1.99 – 1.60 (m, 6H), 1.39 (ddd, *J* = 13.6, 10.6, 5.1 Hz, 1H, minor), 1.33 (ddd, *J* = 14.0, 9.8, 4.6 Hz, 1H, major); **^{13}C NMR** (176 MHz, DMSO-d6) δ 206.4 (minor), 206.2 (major), 155.3 (minor), 155.3 (major), 149.2 (minor), 149.1 (major), 148.8 (minor), 148.7 (major), 133.3 (major), 133.1 (minor), 129.8 (major), 129.7 (minor), 129.2 (minor), 128.8 (major), 128.7 (minor), 128.6 (major), 128.4 (minor), 128.3 (major), 108.20 (minor), 108.17 (major), 103.98 (minor), 103.96 (major), 67.4 (major), 60.0, 58.7 (minor), 55.97 (minor), 55.95 (major), 55.61 (minor), 55.60 (major), 47.2, 44.6 (minor), 44.5 (major), 37.2 (major), 36.6 (minor), 31.5 (major), 31.1 (minor), 26.6 (minor), 26.3 (major), 25.5 (major), 25.1 (minor); **^{19}F NMR** (376 MHz, DMSO-d6) δ −133.4 (minor), −134.6 (major); **IR** (film, cm^{-1}): 2932, 1688, 1605, 1591, 1497, 1457, 1365, 1312,

1260, 1221, 1187, 1126, 1104, 1063, 1031, 1015, 992, 978, 914, 887, 856, 837, 823, 796, 761, 736, 709, 697; **HRMS:** m/z calculated for $C_{25}H_{31}BF_2NO_3$ [M−F]$^+$ 442.2360; found 442.2374.

((1-Benzyl-4-(bis(4-fluorophenyl)methyl)piperazin-1-ium-1-yl)methyl)trifluoroborate

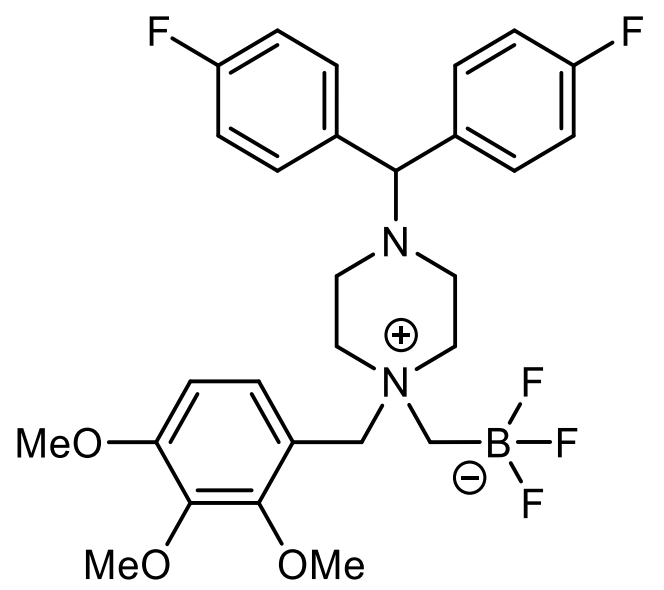

Prepared using general procedure **2.5.2.2. (method I)** using lomerizine (93.6 mg, 0.20 mmol, 1.0 equiv) and potassium (bromomethyl)trifluoroborate (80.3 mg, 0.40 mmol, 2.0 equiv) in acetonitrile (0.20 mL), heating at 110 ˚C for 24 hours. The title compound was obtained as a white solid (96.8 mg, 0.17 mmol, 88% yield). **^{1}H NMR** (700 MHz, CD_3CN) δ 7.44 – 7.39 (m, 4H), 7.27 (d, J = 8.7 Hz, 1H), 7.06 – 7.02 (m, 4H), 6.85 (d, J = 8.7 Hz, 1H), 4.49 (s, 2H), 4.48 (s, 1H), 3.90 (s, 3H), 3.86 (s, 3H), 3.81 (s, 3H), 3.33 – 3.27 (m, 2H), 3.26 – 3.20 (m, 2H), 2.72 – 2.67 (m, 2H), 2.65 – 2.59 (m, 2H), 2.39 – 2.34 (m, 2H); **^{13}C NMR** (176 MHz, CD_3CN) δ 162.9 (d, J = 243.8 Hz), 156.8, 154.9, 142.9, 139.1, 139.0, 130.7, 130.4 (d, J = 8.1 Hz), 116.5 (d, J = 21.5 Hz), 114.8, 108.5, 73.8, 73.8, 61.9 , 61.2 , 59.9 , 56.7, 46.3; **^{19}F NMR** (471 MHz, CD_3CN) δ −115.3, −134.1; **IR** (film, cm^{-1}): 2937, 1610, 1514, 1459, 1447, 1291, 1253, 1220, 1181, 1096, 1068, 1019, 998, 980, 959, 940, 909, 883, 822; **HRMS:** m/z calculated for $C_{28}H_{32}BF_5N_2NaO_3$ [M+Na]$^+$ 573.2318; found 573.2322.

((Benzyl(2-((5-(methoxycarbonyl)-2,6-dimethyl-4-(3-nitrophenyl)-1,4-dihydropyridine-3-carbonyl)oxy)ethyl)(methyl)ammonio)methyl)trifluoroborate

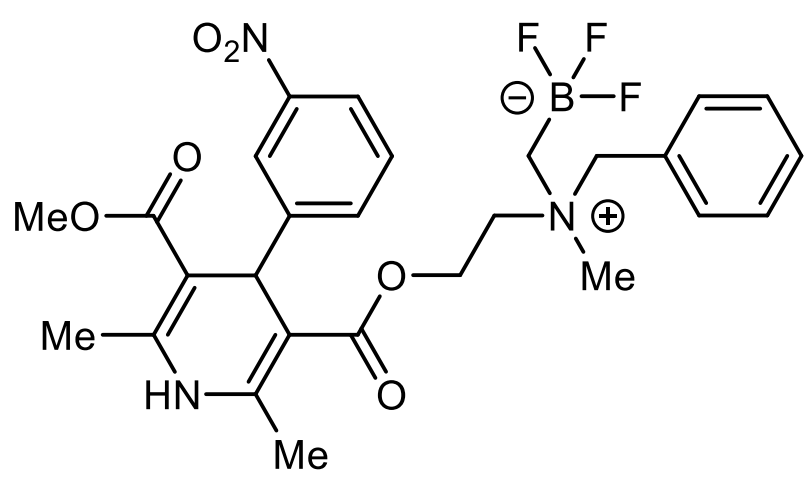

Prepared by general procedure general procedure **2.5.2.1. (method I)** using nicardipine.HCl (515 mg, 1.0 mmol, 1.0 equiv), 2-(Bromomethyl)-4,4,5,5,-tetramethyl-1,3,2-dioxaborolane (0.20 mL, 1.2 mmol, 1.2 equiv), and potassium carbonate (138 mg, 1.0 equiv, 1.0 equiv) in acetonitrile (4.0 mL).

Fluorination was performed with aqueous potassium hydrogen fluoride (3 M, 1.7 mL, 5.0 mmol, 5.0 equiv) and HCl (4 M in 1,4-dioxane, 0.50 mL, 2.0 mmol, 2.0 equiv) in 4:1 acetonitrile/water (0.10 M). The title compound was obtained as a pale yellow solid (522 mg, 0.93 mmol, 93% yield, 1.2:1 d.r.) – *isolated with 10% impurity of N-alkylated product (9:1 r.r.).* **^{1}H NMR** (700 MHz, DMSO-d6) δ 9.17 (s, 1H), 7.94 – 7.89 (m, 2H), 7.58 (ddt, *J* = 13.3, 7.7, 1.4 Hz, 1H), 7.53 – 7.48 (m, 6H), 5.00 (s, 1H), 4.56 – 4.45 (m, 2H), 4.38 (dd, *J* = 12.6, 8.9 Hz, 1H), 4.33 (dd, *J* = 12.6, 7.8 Hz, 1H), 3.61 – 3.55 (m, 1H,), 3.54 (s, 3H), 3.50 – 3.45 (m, 1H), 2.77 (s, 3H), 2.74 (s, 3H), 2.29 – 2.25 (m, 3H), 2.35 (s, 3H), 2.29 – 2.25 (m, 4H), 2.12 – 2.06 (m, 1H); **^{13}C NMR** (176 MHz, DMSO-d6) δ 166.94 (major), 166.93 (minor), 166.00 (minor), 165.98 (major), 149.5 (minor), 149.4 (major), 148.09 (major), 148.08 (minor), 147.74 (minor), 147.73 (major), 146.23 (major), 146.22 (minor), 133.9 (minor), 133.8 (major), 133.09 (minor), 133.06 (major), 129.9, 129.62 (major), 129.60 (minor), 128.73 (minor), 128.72 (major), 128.6, 121.44 (minor), 121.42 (major), 121.2, 101.38 (minor), 101.36 (major), 99.8 (minor), 99.7 (major), 67.0, 61.7, 59.2, 55.0, 50.8, 49.6 (major), 49.5 (minor), 38.63 (minor), 38.59 (major), 18.5 (minor), 18.4 (major), 18.2 (major), 18.2 (minor); **^{19}F NMR** (471 MHz, DMSO-d6) δ −135.4, −135.5; **IR** (film, cm^{-1}): 1694, 1657, 1526, 1477, 1432, 1347, 1317, 1274, 1209, 1115, 1092, 1045, 1009, 898, 881, 778, 740, 701, 680; **HRMS:** m/z calculated for $C_{27}H_{31}BF_3N_3NaO_6$ $[M+Na]^+$ 584.2157; found 584.2157.

((2-amino-6-benzyl-3-(ethoxycarbonyl)-4,5,6,7-tetrahydrothieno[2,3-c]pyridin-6-ium-6-yl)methyl)trifluoroborate

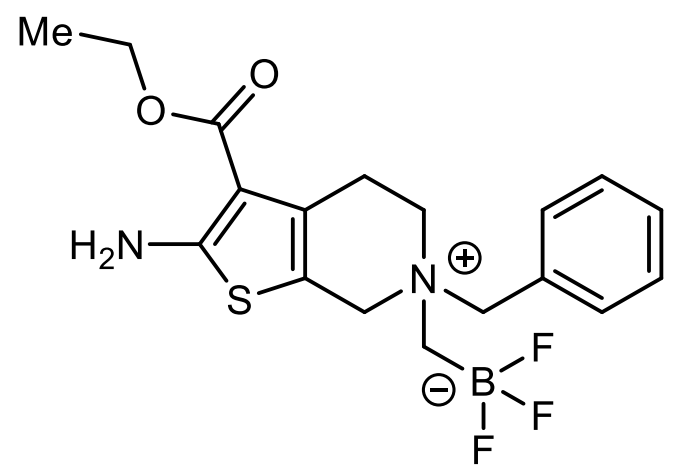

Prepared using general procedure **2.5.2.1. (method I)** using tinoridine (316 mg, 1.0 mmol, 1.0 equiv) and 2-(Bromomethyl)-4,4,5,5,-tetramethyl-1,3,2-dioxaborolane (0.25 mL, 1.5 mmol, 1.5 equiv) in acetonitrile (4.0 mL). Fluorination was performed with aqueous potassium hydrogen fluoride (3 M, 0.83 mL, 2.5 mmol, 2.5 equiv) and HCl (4 M in 1,4-dioxane, 0.25 mL, 1.0 mmol, 1.0 equiv) in 4:1 acetonitrile/water (0.10 M). The title compound was obtained as a brown solid (390 mg, 0.98 mmol, 98% yield). **^{1}H NMR** (400 MHz, CD_2Cl_2) δ 7.59 – 7.42 (m, 5H), 6.24 (s, 2H), 4.63 (d, *J* = 12.7 Hz, 1H), 4.46 (d, *J* = 12.7 Hz, 1H), 4.28 (q, *J* = 7.1 Hz, 2H), 4.16 – 4.05 (m, 2H), 3.76 (dt, *J* = 13.0, 6.5

Hz, 1H), 3.50 (dt, J = 12.6, 6.1 Hz, 1H), 3.30 (dt, J = 18.9, 6.1 Hz, 1H), 3.18 (dt, J = 18.7, 6.4 Hz, 1H), 2.49 – 2.36 (m, 2H), 1.35 (t, J = 7.1 Hz, 3H); **^{13}C NMR** (101 MHz, CD_2Cl_2) δ 165.5, 163.8, 133.6, 130.9, 129.7, 129.5, 128.1, 107.3, 104.9, 64.8, 60.5, 58.4, 58.3, 24.3, 14.6; **^{19}F NMR** (376 MHz, CD_2Cl_2) δ −137.8; **IR** (film, cm^{-1}): 3449, 3332, 2986, 1617, 1588, 1496, 1433, 1267, 1174, 1058, 1041, 997; **HRMS:** m/z calculated for $C_{18}H_{22}BF_3N_2NaO_2S$ $[M+Na]^+$ 421.1339; found 421.1339.

((1-Benzyl-4-(pyrimidin-2-yl)piperazin-1-ium-1-yl)methyl)trifluoroborate

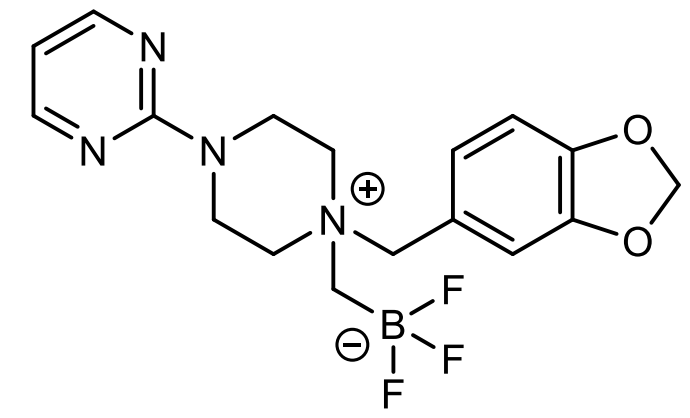

Prepared by general procedure **2.5.2.1. (method I)** using piribedil (894 mg, 3.0 mmol, 1.0 equiv) and 2-(Bromomethyl)-4,4,5,5,-tetramethyl-1,3,2-dioxaborolane (0.61 mL, 3.6 mmol, 1.2 equiv) in acetonitrile (6.0 mL). The reaction mixture was stirred at room temperature for 30 minutes enabling the formation of a white precipitate. The reaction mixture was diluted in acetonitrile (0.25 M) to enable efficient mixing and was stirred until consumption of the amine starting material was observed. Fluorination was performed with aqueous potassium hydrogen fluoride (3 M, 2.4 mL, 7.2 mmol, 2.5 equiv) and HCl (4 M in 1,4-dioxane, 1.5 mL, 6.0 mmol, 2.0 equiv) in 4:1 acetonitrile/water (0.20 M). The title compound was obtained as a white solid (985 mg, 2.6 mmol, 86% yield). **^{1}H NMR** (500 MHz, DMSO-d6) δ 8.44 (d, J = 4.8 Hz, 2H), 7.15 (d, J = 1.7 Hz, 1H), 7.10 (dd, J = 8.0, 1.7 Hz, 1H), 7.03 (d, J = 8.0 Hz, 1H), 6.74 (t, J = 4.8 Hz, 1H), 6.08 (s, 2H), 4.53 (s, 2H), 4.42 (dt, J = 15.3, 3.8 Hz, 2H), 3.71 (ddd, J = 15.3, 9.7, 3.3 Hz, 2H), 3.41 – 3.27 (m, 4H), 2.38 – 2.24 (m, 2H); **^{13}C NMR** (126 MHz, DMSO-d6) δ 160.7, 158.1, 148.5, 147.4, 127.6, 121.6, 112.9, 111.2, 108.4, 101.6, 65.4, 58.4, 47.9, 37.5; **^{19}F NMR** (471 MHz, DMSO-d6) δ −133.9; **IR** (film, cm^{-1}): 2913, 1585, 1557, 1507, 1493, 1461, 1447, 1438, 1383, 1364, 1340, 1315, 1299. 1266, 1240, 1220. 1182, 1107, 1059, 1035, 997, 969, 930, 894, 871, 822, 814, 798, 790, 756, 698; **HRMS:** m/z calculated for $C_{16}H_{20}BF_2N_4$ $[M-F]^+$ 361.1641; found 361.1649.

2-(bromomethyl-d2)-4,4,5,5-tetramethyl-1,3,2-dioxaborolane

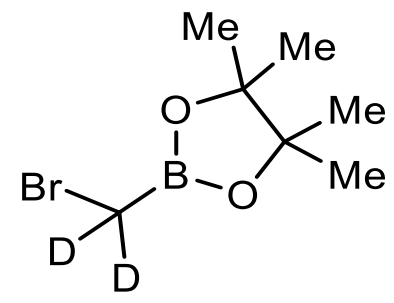

According to the procedure from Wang and coworkers,[74] to a solution of triisopropylborate (1.6 mL, 6.9 mmol, 1.4 equiv) and dibromomethane-*d*2 (0.50 mL, 7.1 mmol, 1.4 equiv) in anhydrous tetrahydrofuran (10 mL) at −78 °C was added *n*-butyllithium (titrated at 1.9 M in hexane, 3.4 mL, 6.4 mmol, 1.3 equiv) over a period of 20 minutes, ensuring the temperature remained below −72 °C. The resulting mixture was stirred at −78 °C for 1 hour, followed by 2 hours at room temperature. Following this, the reaction mixture was cooled down to 0 °C and methanesulfonic acid (0.40 mL, 6.2 mmol, 1.2 equiv) was added over 5 minutes. The reaction mixture was then stirred at room temperature for 1 hour, and then cooled again to 0 °C. Following this, pinacol (603 mg, 5.1 mmol, 1.0 equiv) was added and the reaction was stirred at room temperature for 1 hour. After this, the reaction mixture was concentrated *in vacuo.* The residue was then suspended in dichloromethane (15 mL), filtered on celite and the filtrate was concentrated *in vacuo* to give the title compound as a yellow liquid (1.1 g, 5.1 mmol, 99% yield) which was used in the next step without further purification. **^{1}H NMR** (700 MHz, $CDCl_3$) δ 1.28 (s, 12H); **^{13}C NMR** (176 MHz, $CDCl_3$) δ 84.6, 24.7; **^{2}H NMR** (107 MHz, $CDCl_3$) δ 2.55 (s, 2H); **^{11}B NMR** (160 MHz, $CDCl_3$) δ 31.15; **IR** (film, cm^{-1}): 2980, 2931, 1382, 1384, 1331, 1142, 969, 857; **HRMS:** not detected (compound appeared not to be stable in HRMS conditions)

((1-(benzo[d][1,3]dioxol-5-ylmethyl)-4-(pyrimidin-2-yl)piperazin-1-ium-1-yl)methyl-d2)trifluoroborate

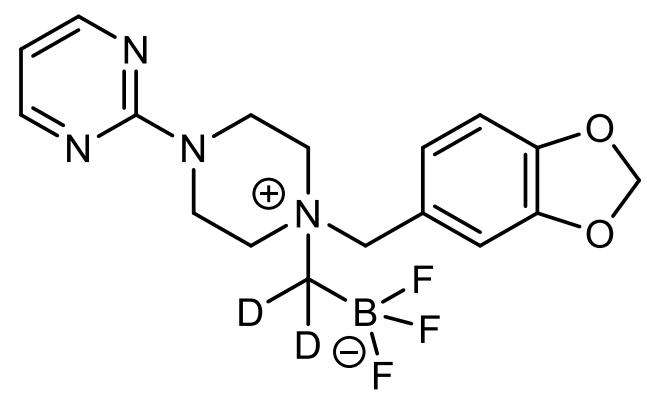

Prepared by general procedure **2.5.2.1. (method II)** using piribedil (298 mg, 1.0 mmol, 1.0 equiv) and 2-(bromomethyl-d2)-4,4,5,5-tetramethyl-1,3,2-dioxaborolane (334 mg, 1.5 mmol, 1.5 equiv) in acetonitrile (4.0 mL). Fluorination was performed with aqueous potassium hydrogen fluoride (3 M, 0.83 mL, 2.5 mmol, 2.5 equiv) and HCl (4 M in 1,4-dioxane, 0.25 mL, 1.0 mmol, 1.0 equiv) in 4:1

acetonitrile/water (0.20 M). The title compound was obtained as a white solid (315 mg, 0.82 mmol, 82% yield). **^{1}H NMR** (500 MHz, CD_2Cl_2) δ 8.36 (d, *J* = 4.8 Hz, 2H), 7.04 (d, *J* = 1.8 Hz, 1H), 7.02 (dd, *J* = 7.9, 1.8 Hz, 1H), 6.89 (dd, *J* = 7.9, 0.4 Hz, 1H), 6.64 (t, *J* = 4.8 Hz, 1H), 6.03 (s, 2H), 4.64 (s, 1H), 4.61 (s, 1H), 4.51 (s, 2H), 3.71 (ddd, *J* = 15.3, 10.9, 2.8 Hz, 2H), 3.52 – 3.44 (m, 2H), 3.15 (ddd, *J* = 12.4, 10.9, 3.8 Hz, 2H); **^{13}C NMR** (176 MHz, CD_2Cl_2) δ 161.4, 158.3, 150.0, 148.7, 128.2, 125.3, 120.9, 113.3, 112.0, 109.0, 102.4, 68.8, 59.8, 38.3; **^{2}H NMR** (107 MHz, CD_2Cl_2) δ 2.09 (s, 2H); **^{19}F NMR** (471 MHz, CD_2Cl_2) δ −137.2 (m); **IR** (film, cm^{-1}): 2928, 2857, 1586, 1556, 1506, 1492, 1445, 1360, 1258, 1238, 1031, 929; **HRMS:** m/z calculated for $C_{17}H_{18}D_2BF_2N_4O_2$ $[M-F]^+$ 363.1767; found 363.1768.

((1-benzyl-4-(2-(1-(4-methoxybenzyl)-2,6-dioxopiperidin-3-yl)-1-oxoisoindolin-5-yl)piperidin-1-ium-1-yl)methyl)trifluoroborate

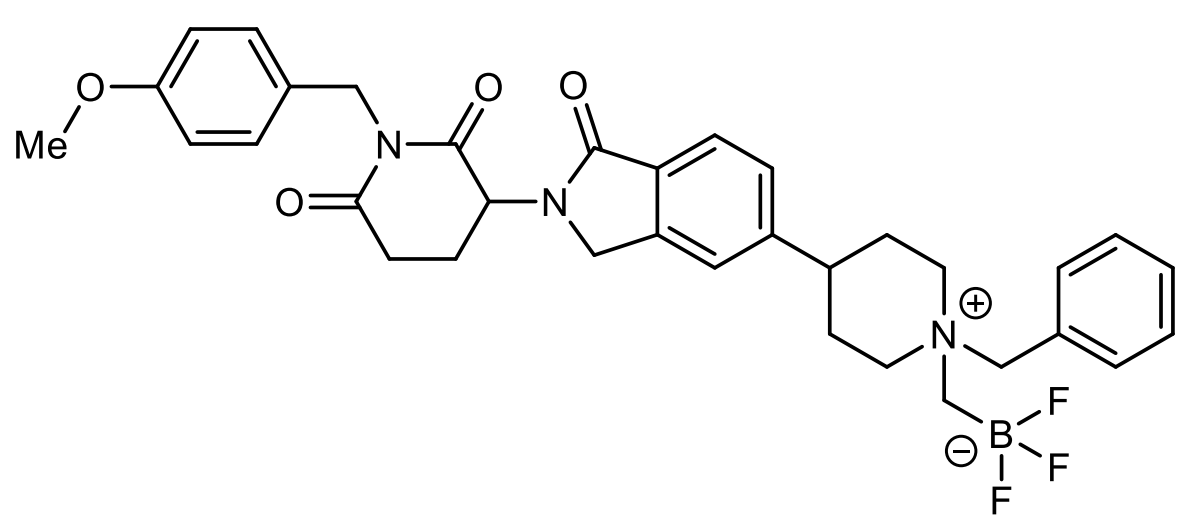

Prepared by general procedure **2.5.2.1. (method II)** using *N*-PMB NVP-DKY709 (60 mg, 0.12 mmol, 1.0 equiv) and 2-(Bromomethyl)-4,4,5,5,-tetramethyl-1,3,2-dioxaborolane (25 µL, 0.15 mmol, 1.3 equiv) in acetonitrile (0.24 mL). Fluorination was performed with aqueous potassium hydrogen fluoride (3 M, 0.13 mL, 0.25 mmol, 2.1 equiv) and HCl (4 M in 1,4-dioxane, 38 µL, 0.12 mmol, 1.2 equiv) in 4:1 acetonitrile/water (0.20 M). The title compound was obtained as a white solid (62 mg, 0.10 mmol, 87% yield, d.r. 4:1) and used in the homologation step without further purification. **^{1}H NMR** ((700 MHz, CD_2Cl_2) δ 7.84 (d, *J* = 7.8 Hz, 1H, minor), 7.78 – 7.76 (m, 1H, major), 7.59 – 7.57 (m, 2H), 7.54 – 7.45 (m, 5H), 7.44 (s, 1H, minor), 7.40 (s, 1H, major), 7.35 (dd, *J* = 8.0, 1.5 Hz, 1H), 7.29 – 7.26 (m, 2H), 5.14 (m, 1H, minor), 5.11 (dd, *J* = 13.6, 5.2 Hz, 1H, major), 4.89 – 4.83 (m, 2H), 4.61 (s, 2H, major), 4.59 (s, 2H, minor), 4.37 (d, *J* = 2.8 Hz, 2H, minor), 4.33 (d, *J* = 1.9 Hz, 2H, major), 3.76 (s, 3H, minor), 3.76 (s, 3H, major), 3.61 (td, *J* = 10.9, 3.0 Hz, 2H, major), 3.56 (dt, *J* = 11.5, 3.6 Hz, 2H, minor), 3.16 – 3.10 (m, 2H, major), 3.08 (dq, *J* = 12.0, 4.1 Hz, 2H, minor), 3.00 – 2.93 (m, 1H), 2.85 (m, 1H), 2.80f (m, 1H), 2.56 – 2.52 (m, 2H), 2.39 – 2.27 (m, 4H), 2.16 (ddtd, *J* = 15.2, 12.8, 5.3, 2.4 Hz, 2H), 1.99 (d, *J* = 15.1 Hz, 2H); **^{13}C NMR** (176 MHz, CD_2Cl_2) δ 171.40 (major), 171.4 (minor), 170.54 (minor), 170.52 (major), 169.10 (major), 169.07 (minor), 159.47 (minor), 159.45 (major), 147.7 (major), 147.5 (minor), 142.96 (minor), 142.89 (major), 133.9 (major), 133.3 (minor), 131.35 (minor), 131.2 (major), 130.9 (minor), 130.8 (major), 130.52 (minor), 130.51 (major),

129.65, 129.56, 129.4, 128.3 (minor), 128.1 (major), 127.7, 127.1, 124.6 (minor), 124.4 (major), 121.9 (minor), 121.5 (major), 114.1 (minor), 114.05 (major), 85.0 (minor), 83.3 (major), 70.40 (d, J = 3.1 Hz, minor), 67.4 (major), 60.98 (d, J = 2.2 Hz, major), 60.46 (d, J = 1.7 Hz, minor), 55.60 (minor), 55.59 (major), 53.10 (minor), 53.07 (major), 47.60 (minor), 47.56 (major), 43.27, 43.24, 40.6, 32.6, 32.6, 28.3, 27.8, 27.7, 24.9 (minor), 24.8 (major), 22.9 (major); **^{19}F NMR** (471 MHz, CD_2Cl_2) δ −137.1, −138.5; **IR** (film, cm^{-1}): 2930, 1682, 1515, 1456, 1369, 1343, 1249, 1167, 1061, 1010, 915; **HRMS:** m/z calculated for $C_{34}H_{37}BF_2N_3O_4$ $[M-F]^+$ 600.2840; found 600.2827.

4-Phenethylmorpholine (**2a**)

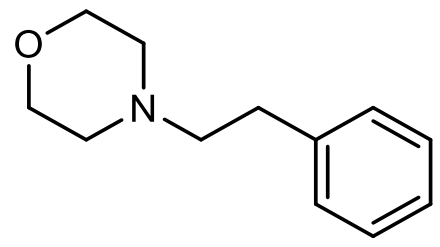

Prepared by general procedure **2.5.3.1.** ((4-benzylmorpholino-4-ium)methyl)trifluoroborate (**4a**) (104 mg, 0.40 mmol, 1.0 equiv), bis(benzonitrile)palladium(II) chloride (7.7 mg, 0.02 mmol, 5 mol%), RuPhos (19 mg, 0.04 mmol, 10 mol%) and potassium *tert*-butoxide (224 mg, 2.0 mmol, 5.0 equiv) in 4:1 1,4-dioxane/water (2.0 mL). Purification by automated flash column chromatography (0–10% methanol in dichloromethane)) gave the title compound as a colourless oil (59 mg, 0.31 mmol, 77% yield). Data was in line with previous characterisation.[77] **^{1}H NMR** (700 MHz, $CDCl_3$) δ 7.31 – 7.27 (m, 1H), 7.23 – 7.18 (m, 1H), 3.74 (t, J = 4.7 Hz, 2H), 2.85 – 2.78 (m, 1H), 2.64 – 2.57 (m, 1H), 2.57 – 2.45 (m, 2H); **^{13}C NMR** (176 MHz, $CDCl_3$) δ 140.2, 128.7, 128.4, 126.1, 67.0, 60.9, 53.7, 33.4.

1-Phenethylpiperidine (**2b**)

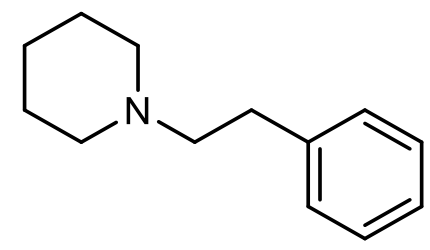

Prepared by general procedure **2.5.3.1.** using ((1-benzyl-4-(tert-butoxycarbonyl)piperazin-1-ium-1-yl)methyl)trifluoroborate (**4b**) (51 mg, 0.20 mmol, 1.0 equiv), bis(benzonitrile)palladium(II) chloride (3.8 mg, 0.01 mmol, 5 mol%), RuPhos (9.3 mg, 0.02 mmol, 10 mol%), and potassium *tert*-butoxide (112 mg, 1.0 mmol, 5.0 equiv), in 4:1 1,4-dioxane/water (1.0 mL). Purification by automated flash column chromatography (0–10% methanol in dichloromethane) gave the title compound as a colourless oil (32 mg, 0.17 mmol, 86% yield). Data was in line with previous characterisation.[77] **^{1}H NMR** (700 MHz, $CDCl_3$) δ 7.33 – 7.28 (m, 2H), 7.26 – 7.19 (m, 3H) 2.88 – 2.82 (m, 2H), 2.62 – 2.57 (m, 2H) , 2.57 – 2.37 (m, 4H), 1.65 (p, J = 5.7 Hz, 4H), 1.55 – 1.43 (m, 2H); **^{13}C NMR** (176 MHz, $CDCl_3$) δ 140.7, 128.7, 128.4, 126.0, 61.5, 54.6, 33.7, 26.0, 24.5.

tert-butyl 4-phenethylpiperazine-1-carboxylate (**2c**)

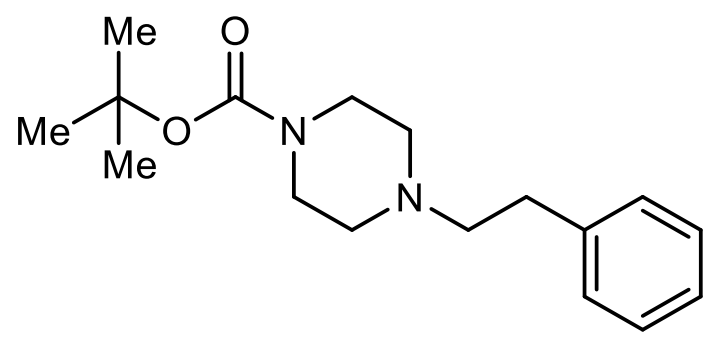

Prepared by general procedure **2.5.3.1.** using ((1-benzylpiperidin-1-ium-1-yl)methyl)trifluoroborate (**4c**) (72 mg, 0.20 mmol, 1.0 equiv), bis(benzonitrile)palladium(II) chloride (3.8 mg, 0.01 mmol, 5 mol%), RuPhos (9.3 mg, 0.02 mmol, 10 mol%) and potassium *tert*-butoxide (112 mg, 1.0 mmol, 5.0 equiv) in 4:1 1,4-dioxane/water (1.0 mL). Purification by automated flash column chromatography (0–10% methanol in dichloromethane) gave the title compound as a colourless oil (52 mg, 0.18 mmol, 89% yield). Data was in line with previous characterisation.[78] **^{1}H NMR** (700 MHz, $CDCl_3$) δ 7.31 – 7.26 (m, 2H), 7.20 (ddd, *J* = 6.1, 3.1, 1.7 Hz, 3H), 3.46 (dd, *J* = 7.4, 2.9 Hz, 4H), 2.84 – 2.77 (m, 2H), 2.65 – 2.58 (m, 2H), 2.50 – 2.44 (m, 4H), 1.46 (s, 9H); **^{13}C NMR** (176 MHz, $CDCl_3$) δ 154.9, 140.3, 128.8, 128.6, 126.2, 79.8, 60.6, 53.2, 44.3, 33.7, 28.6.

1-Phenethyl-4-tosylpiperazine (**2d**)

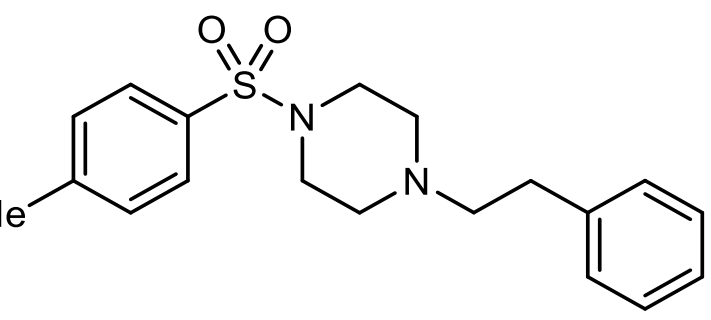

Prepared by general procedure **2.5.3.1.** using ((1-benzyl-4-tosylpiperazin-1-ium-1-yl)methyl)trifluoroborate (**4d**) (73 mg, 0.40 mmol, 1.0 equiv), bis(benzonitrile)palladium(II) chloride (7.7 mg, 0.02 mmol, 5 mol%), RuPhos (19 mg, 0.04 mmol, 10 mol%) and potassium *tert*-butoxide (224 mg, 2.0 mmol, 5.0 equiv) in 4:1 1,4-dioxane/water (2.0 mL). Purification by automated flash column chromatography (30% ethyl acetate in petroleum ether) gave the title compound as a colourless oil (66 mg, 0.19 mmol, 48% yield). **^{1}H NMR** (700 MHz, $CDCl_3$) δ 7.67 (d, *J* = 7.9 Hz, 2H), 7.35 (d, *J* = 7.9 Hz, 2H), 7.29 (t, *J* = 7.5 Hz, 2H), 7.21 (t, *J* = 7.4 Hz, 1H), 7.17 (d, *J* = 7.5 Hz, 2H), 3.16 – 2.98 (m, 4H), 2.80 – 2.71 (m, 2H), 2.67 – 2.57 (m, 6H), 2.45 (s, 3H); **^{13}C NMR** (176 MHz, $CDCl_3$) δ 143.7, 139.8, 132.3, 129.7, 128.6, 128.5, 127.9, 126.2, 59.9, 52.2, 46.1, 33.5, 21.5; **IR** (film, cm^{-1}): 2832, 1595, 1492, 1464, 1453, 1372, 1343, 1328, 1306, 1291, 1258, 1171, 1160, 1135, 1117, 1091, 1059, 1042, 1018, 1000, 952, 908, 815, 801, 776, 748, 728, 698, 652; **HRMS:** m/z calculated for $C_{19}H_{24}N_2O_2S$ $[M + H]^+$ 345.1637; found 345.1636.

3,5-dimethyl-1-phenethylpiperidine (**2e**)

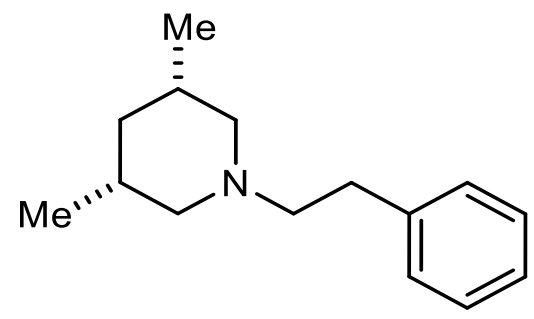

Prepared by general procedure **2.5.3.1.** using ((1-benzyl-3,5-dimethylpiperidin-1-ium-1-yl)methyl)trifluoroborate (**4e**) (49 mg, 0.20 mmol, 1.0 equiv), bis(benzonitrile)palladium(II) chloride (3.8 mg, 0.01 mmol, 5 mol%), RuPhos (9.3 mg, 0.02 mmol, 10 mol%), and potassium *tert*-butoxide (112 mg, 1.0 mmol, 5.0 equiv) in 4:1 1,4-dioxane/water (1.0 mL). Purification by automated flash column chromatography (0–30% acetone in petroleum ether for 15 minutes, 30% acetone in petroleum ether for 5 minutes, 0–20% methanol in dichloromethane for 5 minutes, 20% methanol in dichloromethane for 5 minutes) gave the title compound as a colourless oil (33 mg, 0.15 mmol, 76% yield). **^{1}H NMR** (700 MHz, $CDCl_3$) δ 7.33 – 7.25 (m, 2H), 7.23 – 7.17 (m, 3H), 2.94 (ddt, *J* = 10.4, 3.4, 1.5 Hz, 2H), 2.85 – 2.80 (m, 2H), 2.60 – 2.55 (m, 2H), 1.78 – 1.68 (m, 3H), 1.51 (t, *J* = 10.9 Hz, 2H), 0.88 (d, *J* = 6.4 Hz, 6H), 0.59 – 0.50 (m, 1H); **^{13}C NMR** (176 MHz, $CDCl_3$) δ 140.8, 128.8, 128.5, 126.1, 61.8, 61.1, 42.3, 33.8, 31.3, 19.8; **IR (**thin film, cm^{-1}) 2952, 2756, 1456, 1125, 1081, 747, 698; **HRMS:** m/z calculated for $C_{15}H_{24}N$ $[M+H]^+$ 218.1903; found 218.1907.

8-phenethyl-1,4-dioxa-8-azaspiro[4.5]decane (**2f**)

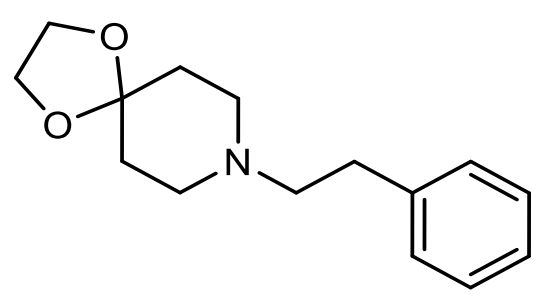

Prepared by general procedure **2.5.3.1.** using ((8-benzyl-1,4-dioxa-8-azaspiro[4.5]decan-8-ium-8-yl)methyl)trifluoroborate (**4f**) (315 mg, 1.0 mmol, 1.0 equiv), bis(benzonitrile)palladium(II) chloride (19 mg, 0.05 mmol, 5 mol%), RuPhos (47 mg, 0.10 mmol, 10 mol%) and potassium *tert*-butoxide (561 mg, 5.0 mmol, 5.0 equiv) in 4:1 dioxane/water (5.0 mL). Purification by automated flash column chromatography (5–30% acetone in petroleum ether) gave the title compound as a white solid (201 mg, 0.81 mmol, 81% yield). Data was in line with previous characterisation.[79] **^{1}H NMR** (500 MHz, $CDCl_3$) δ 7.30 – 7.26 (m, 2H), 7.22 – 7.17 (m, 3H), 3.96 (s, 4H), 2.84 – 2.77 (m, 2H), 2.70 – 2.59 (m, 4H), 1.79 (t, *J* = 5.7 Hz, 4H); **^{13}C NMR** (126 MHz, $CDCl_3$) δ 140.6, 128.8, 128.5, 126.2, 107.4, 64.4, 60.3, 51.5, 35.0, 34.1.

1-phenethyl-4-phenylpiperidin-4-ol (**2g**)

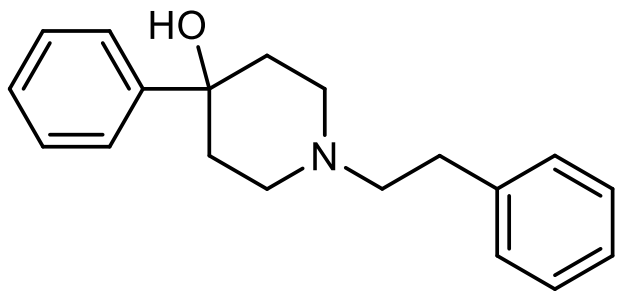

Prepared by general procedure **2.5.3.1.** using ((1-benzyl-4-hydroxy-4-phenylpiperidin-1-ium-1-yl)methyl)trifluoroborate (**4g**) (140 mg, 0.40 mmol, 1.0 equiv), bis(benzonitrile)palladium(II) chloride (7.8 mg, 0.02 mmol, 5 mol%), RuPhos (19 mg, 0.04 mmol, 10 mol%) and potassium *tert*-butoxide (224 mg, 2.0 mmol, 5.0 equiv) in 4:1 1,4-dioxane/water (2.0 mL). Purification by automated flash column chromatography (5–50% acetone in petroleum ether) gave the title compound as a white solid (55 mg, 0.20 mmol, 50% yield). **^{1}H NMR** (500 MHz, $CDCl_3$) δ 7.56 – 7.51 (m, 2H), 7.37 (dd, *J* = 8.4, 7.0 Hz, 2H), 7.33 – 7.25 (m, 3H), 7.25 – 7.19 (m, 3H), 2.92 (dt, *J* = 11.4, 2.4 Hz, 2H), 2.89 – 2.84 (m, 2H), 2.73 – 2.64 (m, 2H), 2.56 (td, *J* = 11.9, 2.5 Hz, 2H), 2.21 (td, *J* = 13.2, 4.5 Hz, 2H), 1.84 – 1.76 (m, 2H), 1.69 (br s, 1H); **^{13}C NMR** (126 MHz, $CDCl_3$) δ 148.50, 140.59, 128.85, 128.54, 128.50, 127.16, 126.17, 124.67, 124.66, 71.42, 71.41, 60.90, 49.66, 38.7, 33.9; **IR** (film, cm^{-1}): 3314, 2923, 2817, 1671, 1602, 1494, 1447, 1374, 1132, 1117, 1031; **HRMS:** m/z calculated for $C_{19}H_{24}NO$ $[M + H]^+$ 282.1852; found 282.1854.

ethyl 1-phenethylpiperidine-3-carboxylate **(2h)**

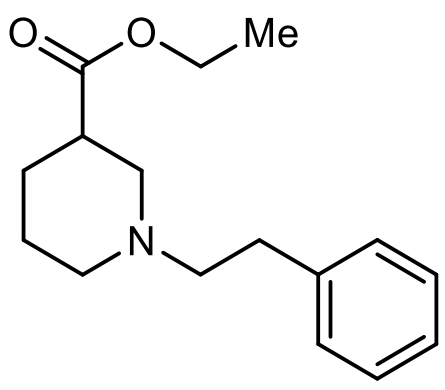

Prepared by general procedure **2.5.3.3.** using ((1-benzyl-3-(ethoxycarbonyl)piperidin-1-ium-1-yl)methyl)trifluoroborate (**4h**) (66 mg, 0.20 mmol, 1.0 equiv), Pd-PEPPSI^{TM}-IPent (7.9 mg, 0.01 mmol, 5 mol%), and potassium phosphate tribasic (424 mg, 2.0 mmol, 10 equiv) in 4:1 1,4-dioxane/water (1.0 mL). Purification by reverse phase automated flash column chromatography (0–100% acetonitrile in water) afforded the title compound as a pale yellow solid (24 mg, 0.09 mmol, 45% yield). Data was in line with previous characterisation.[80] **^{1}H NMR** (700 MHz, $CDCl_3$) δ 7.31 – 7.28 (m, 2H), 7.22 – 7.18 (m, 3H), 4.14 (q, *J* = 7.1 Hz, 2H), 3.09 (d, *J* = 11.1 Hz, 1H), 2.88 – 2.83 (m, 1H), 2.83 – 2.77 (m, 2H), 2.65 – 2.54 (m, 3H), 2.24 (t, *J* = 11.1 Hz, 1H), 2.07 (t, *J* = 11.1 Hz, 1H), 1.96 (dd, *J* = 13.1, 4.1 Hz, 1H), 1.78 – 1.73 (m, 1H), 1.67 – 1.58 (m, 1H), 1.51 – 1.42 (m, 1H), 1.26 (t,

J = 7.1 Hz, 3H); **^{13}C NMR** (176 MHz, $CDCl_3$) δ 174.4, 140.6, 128.9, 128.5, 126.1, 63.4, 61.0, 60.5, 55.5, 53.9, 42.1, 33.7, 27.2, 24.8, 14.4.

1'-phenethylspiro[chromane-2,4'-piperidin]-4-one (**2i**)

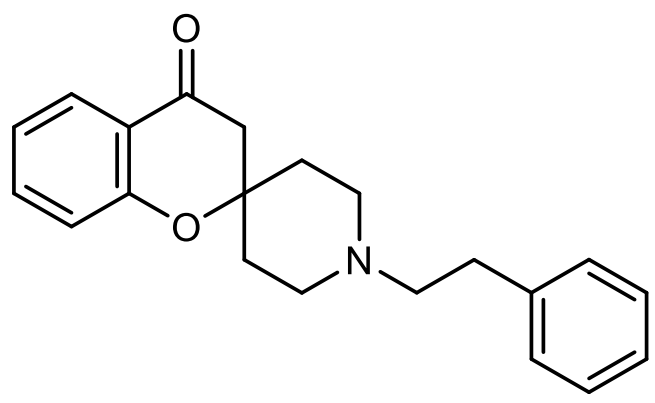

Prepared by general procedure **2.5.3.3.** using ((1'-benzyl-4-oxospiro[chromane-2,4'-piperidin]-1'-yl-1'-ium)methyl)trifluoroborate (**4i**) (79 mg, 0.20 mmol, 1.0 equiv), Pd-PEPPSI^{TM}-IPent (7.9 mg, 0.01 mmol, 5 mol%), and potassium phosphate tribasic (424 mg, 2.0 mmol, 10 equiv) in 4:1 1,4-dioxane/water (1.0 mL). Purification by automated flash column chromatography (10–30% acetone in petroleum ether) gave the title compound as a yellow oil (31 mg, 0.09 mmol, 48% yield). **^{1}H NMR** (500 MHz, $CDCl_3$) δ 7.86 (ddd, J = 8.1, 1.8, 0.8 Hz, 1H), 7.48 (dddd, J = 8.1, 7.1, 1.8, 0.8 Hz, 1H), 7.31 – 7.27 (m, 2H), 7.23 – 7.18 (m, 3H), 6.99 (dddd, J = 7.1, 6.1, 1.9, 1.0 Hz, 2H), 2.85 – 2.79 (m, 2H), 2.73 (d, J = 0.8 Hz, 4H), 2.68 – 2.63 (m, 2H), 2.52 (td, J = 11.5, 2.7 Hz, 2H), 2.08 (dq, J = 14.5, 3.0 Hz, 2H), 1.78 (ddd, J = 14.0, 11.6, 4.4 Hz, 2H); **^{13}C NMR** (126 MHz, $CDCl_3$) δ 192.3, 159.4, 140.4, 136.4, 128.8, 128.6, 126.7, 126.2, 121.1, 121.0, 118.5, 77.9, 60.6, 48.9, 48.2, 34.5, 34.0; **IR** (film, cm^{-1}): 2944, 2811, 1690, 1608, 1463, 1326, 1299, 1231, 1115, 736; **HRMS:** m/z calculated for $C_{21}H_{24}NO_2$ $[M + H]^+$ 322.1802; found 322.1793.

1-phenethylpyrrolidine (**2j**)

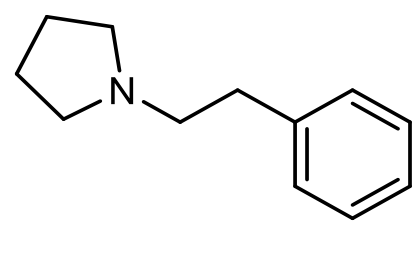

Prepared by general procedure **2.5.3.2.** using ((1-benzylpyrrolidin-1-ium-1-yl)methyl)trifluoroborate (**4j**) (49 mg, 0.20 mmol, 1.0 equiv), Pd-PEPPSI^{TM}-IPent (16 mg, 0.02 mmol, 10 mol%), isopropanol (2.4 mg, 0.04 mmol, 20 mol%), and potassium *tert*-butoxide (224 mg, 2.0 mmol, 10 equiv) in 4:1 1,4-dioxane/water (1.0 mL). Purification by automated flash column chromatography (100% dichloromethane for 1 minute, 0–20% methanol in dichloromethane for 19 minutes, 20% methanol in dichloromethane for 5 minutes) gave the title compound as a colourless oil (21 mg, 0.12 mmol, 61% yield). Data was in line with previous characterisation.[81] **^{1}H NMR** (700 MHz, $CDCl_3$) δ 7.30 – 7.23

(m, 2H), 7.18 (m, 3H), 2.85 – 2.80 (m, 2H), 2.73 – 2.68 (m, 2H), 2.61 – 2.58 (m, 4H), 1.80 (h, *J* = 3.4 Hz, 4H); **^{13}C NMR** (176 MHz, $CDCl_3$) δ 140.2, 128.7, 128.5, 126.2, 58.4, 54.2, 35.5, 23.4.

tert-butyl 7-phenethyl-2,7-diazaspiro[4.4]nonane-2-carboxylate (**2k**)

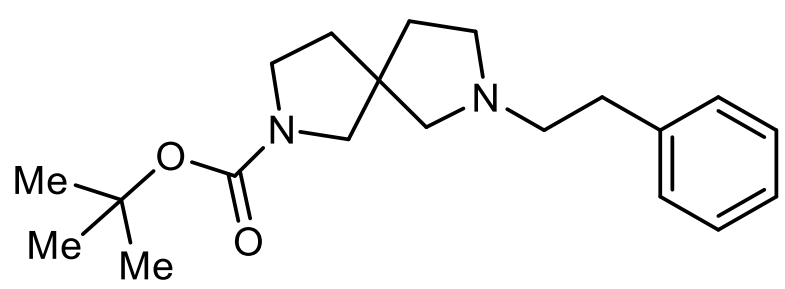

Prepared by general procedure **2.5.3.2.** using ((2-benzyl-7-(tert-butoxycarbonyl)-2,7-diazaspiro[4.4]nonan-2-ium-2-yl)methyl)trifluoroborate (**4k**) (80 mg, 0.20 mmol, 1.0 equiv), Pd-PEPPSI^{TM}-IPent (7.9 mg, 0.01 mmol, 5 mol%), and potassium *tert*-butoxide (224 mg, 2.0 mmol, 10 equiv) in 4:1 1,4-dioxane/water (1.0 mL). Purification by automated flash column chromatography (10–50% ethyl acetate in petroleum ether) gave the title compound as a colourless oil (25 mg, 0.08 mmol, 38% yield). **^{1}H NMR** (500 MHz, $CDCl_3$) δ δ 7.37 – 7.30 (m, 2H), 7.25 (d, *J* = 7.4 Hz, 3H), 3.52 – 3.23 (m, 4H), 2.91 – 2.48 (m, 9H), 1.86 (dtq, *J* = 19.0, 12.1, 6.2 Hz, 4H), 1.51 (d, *J* = 2.3 Hz, 9H); **^{13}C NMR** (126 MHz, $CDCl_3$) δ 154.9 (major), 154.8 (minor), 140.5, 128.8, 128.5, 126.2, 79.3 (major), 79.2 (minor), 64.5 (major), 64.4 (minor), 58.5, 58.2 (major), 57.2 (minor), 54.1, 48.2 (major), 47.4 (minor), 45.6 (minor), 45.2 (major), 38.4 (minor), 37.4 (major), 35.9 (minor), 35.7 (major), 35.6, 28.7; **IR** (film, cm^{-1}): 2970, 2935, 2870, 1688, 1478, 1397, 1366, 1170, 1142, 1106; **HRMS:** m/z calculated for $C_{20}H_{31}N_2O_2$ $[M + H]^+$ 331.2380; found 331.2382.

Note: the compound was isolated as a mixture of rotamers, identified as minor and major where possible.

tert-butyl 2-phenethyl-2,7-diazaspiro[3.5]nonane-7-carboxylate (**2l**)

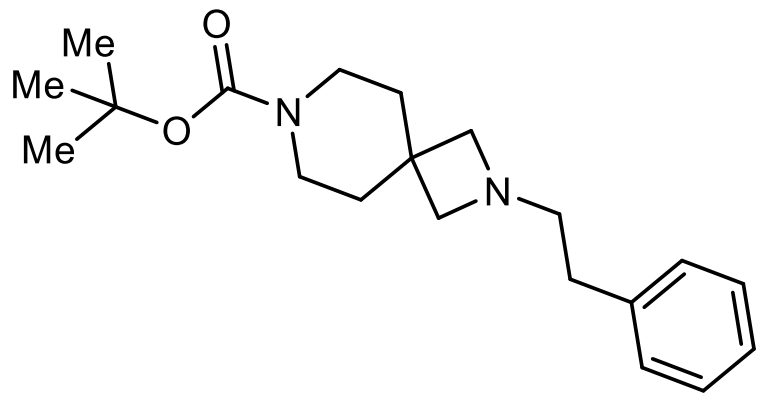

Prepared by general procedure **2.5.3.1.** using ((2-benzyl-7-(tert-butoxycarbonyl)-2,7-diazaspiro[3.5]nonan-2-ium-2-yl)methyl)trifluoroborate (**4l**) (159 mg, 0.40 mmol, 1.0 equiv), bis(benzonitrile)palladium(II) chloride (7.7 mg, 0.02 mmol, 5 mol%), RuPhos (19 mg, 0.04 mmol, 10 mol%) and potassium *tert*-butoxide (224 mg, 2.0 mmol, 5.0 equiv) in 4:1 1,4-dioxane/water (2.0 mL).

Purification by automated column chromatography (5–30% acetone in petroleum ether) gave the title compound as a colourless oil (57 mg, 0.17 mmol, 43% yield). **^{1}H NMR** (500 MHz, $CDCl_3$) 7.29 – 7.25 (m, 2H), 7.19 (t, J = 7.9 Hz, 3H), 3.32 (dd, J = 6.8, 4.6 Hz, 4H), 3.02 (s, 4H), 2.71 – 2.67 (m, 2H), 2.63 (dd, J = 9.5, 6.2 Hz, 2H), 1.72 – 1.66 (m, 4H), 1.45 (s, 9H); **^{13}C NMR** (126 MHz, $CDCl_3$) δ 155.0, 140.2, 128.8, 128.5, 126.2, 79.5, 64.4, 62.0, 36.2, 34.7, 34.6, 28.6; **IR** (film, cm^{-1}): 2976, 2925, 2852, 1687, 1617, 1422, 1366, 1277, 1246, 1156, 1042, 1026, 1014; **HRMS:** m/z calculated for $C_{20}H_{31}N_2O_2$ $[M + H]^+$ 331.2380; found 331.2382.

Note: ring-opening of the azetidinium by hydroxide ion was also observed as a side product by LCMS.

tert-butyl 4-phenethyl-1,4-diazepane-1-carboxylate (**2m**)

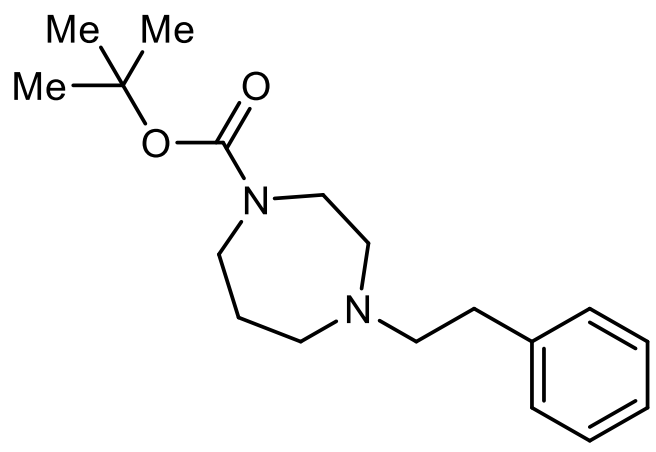

Prepared by general procedure **2.5.3.1.** using ((1-benzyl-4-(tert-butoxycarbonyl)-1,4-diazepan-1-ium-1-yl)methyl)trifluoroborate (**4m**) (142 mg, 0.38 mmol, 1.0 equiv), bis(benzonitrile)palladium(II) chloride (7.3 mg, 0.02 mmol, 5 mol%), RuPhos (18 mg, 0.04 mmol, 10 mol%) and potassium *tert*-butoxide (213 mg, 1.9 mmol, 5.0 equiv) in 4:1 1,4-dioxane/water (1.9 mL). Purification by automated flash column chromatography (10–40% acetone in petroleum ether) gave the title compound as a colourless oil (81 mg, 0.27 mmol, 71% yield). Data was in line with previous characterisation.[82] **^{1}H NMR** (700 MHz, $CDCl_3$) δ 7.37 – 7.32 (m, 2H), 7.26 (q, J = 3.5 Hz, 3H), 3.59 (dd, J = 7.2, 3.3 Hz, 1H), 3.57 – 3.48 (m, 3H), 2.87 – 2.78 (m, 5H), 2.76 (q, J = 5.4 Hz, 2H), 1.90 (dp, J = 24.4, 6.0 Hz, 2H), 1.53 (d, J = 1.5 Hz, 9H); **^{13}C NMR** (176 MHz, $CDCl_3$) δ 155.74, 155.62, 140.53, 140.51, 140.48, 128.82, 128.80, 128.48, 128.47, 126.12, 126.08, 79.37, 79.34, 59.96, 59.88, 56.13, 55.95, 54.96, 54.71, 46.98, 46.42, 46.10, 45.16, 34.22, 34.18, 28.63, 28.05, 27.92.

Note: the compound was isolated as a mixture of rotamers.

(4R)-3-((benzo[d][1,3]dioxol-5-yloxy)methyl)-4-(4-fluorophenyl)-1-phenethylpiperidine (**2n**)

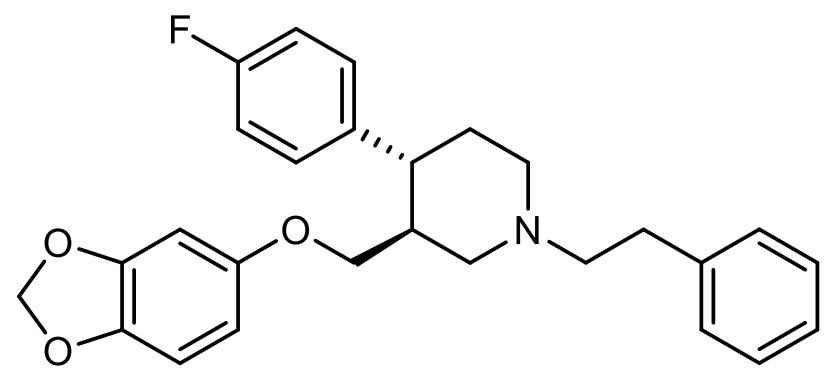

Prepared by general procedure **2.5.3.1.** using (3S,4R)-3-((benzo[d][1,3]dioxol-5-yloxy)methyl)-1-benzyl-4-(4-fluorophenyl)piperidin-1-ium-1-yl)methyl)trifluoroborate (**4n**) (100 mg, 0.20 mmol, 1.0 equiv), bis(benzonitrile)palladium (3.8 mg, 0.01 mmol, 5.0 mol%), RuPhos (9.3 mg, 0.02 mmol, 10 mol%) and potassium *tert*-butoxide (112 mg, 1.0 mmol, 5.0 equiv) in 4:1 1,4-dioxane/water (1.0 mL). Purification by automated column chromatography (5%–30% ethyl acetate in petroleum ether) gave the title compound as a colourless oil (60 mg, 0.14 mmol, 70% yield). **^{1}H NMR** (500 MHz, $CDCl_3$) δ 7.30 (t, *J* = 7.6 Hz, 2H), 7.25 – 7.20 (m, 3H), 7.18 (ddd, *J* = 8.5, 5.3, 2.5 Hz, 2H), 7.02 – 6.94 (m, 2H), 6.63 (d, *J* = 8.5 Hz, 1H), 6.36 (d, *J* = 2.4 Hz, 1H), 6.14 (dd, *J* = 8.5, 2.5 Hz, 1H), 5.88 (s, 2H), 3.60 (dd, *J* = 9.4, 2.9 Hz, 1H), 3.47 (dd, *J* = 9.4, 7.1 Hz, 1H), 3.36 (ddd, *J* = 11.2, 3.6, 1.8 Hz, 1H), 3.13 (ddt, *J* = 11.2, 3.9, 2.4 Hz, 1H), 2.93 – 2.81 (m, 2H), 2.72 – 2.65 (m, 2H), 2.49 (td, *J* = 11.4, 4.7 Hz, 1H), 2.24 (tdt, *J* = 10.7, 6.7, 3.2 Hz, 1H), 2.19 – 2.09 (m, 2H), 1.94 – 1.82 (m, 2H); **^{13}C NMR** (126 MHz, $CDCl_3$) δ 161.7 (d, *J* = 244.3 Hz), 154.5, 148.3, 141.7, 140.5, 139.8 (d, *J* = 3.2 Hz), 129.0 (d, *J* = 7.8 Hz), 128.9, 128.6, 126.2, 115.6 (d, *J* = 21.1 Hz), 108.0, 105.7, 101.2, 98.1, 69.8, 61.1, 57.7, 54.3, 44.3, 42.3, 34.6, 33.9; **^{19}F NMR** (471 MHz, $CDCl_3$) δ −116.5.; **IR** (film, cm^{-1}): 2917, 2805, 2771, 1630, 1604, 1509, 1487, 1180, 1159, 1038, 1016, 831; **HRMS:** m/z calculated for $C_{27}H_{29}NO_3F$ $[M + H]^+$ 434.2126; found 434.2126.

N,*N*-dimethyl-2-phenylethan-1-amine (**2o**)

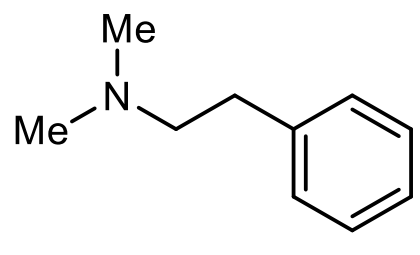

Prepared by general procedure **2.5.3.1.** trifluoro((4-(4-methoxybenzyl)morpholino-4-ium)methyl)borate (**4o**) (87 mg, 0.40 mmol, 1.0 equiv), bis(benzonitrile)palladium(II) chloride (7.7 mg, 0.02 mmol, 5.0 mol%), RuPhos (19 mg, 0.04 mmol, 10 mol%) and potassium *tert*-butoxide (224 mg, 2.0 mmol, 5.0 equiv) in 4:1 1,4-dioxane/water (2.0 mL). Reaction mixture was concentrated under reduced pressure and an assay yield was taken using 1,1,2,2-tetrachloroethane (44 µL, 0.40 mmol) as an internal standard (83% assay yield). Further purification was not possible due to the product's high volatility. Data was in line with previous characterisation.[83] **^{1}H NMR** (400 MHz, $CDCl_3$) δ 7.34 – 7.26

(m, 2H), 7.26 – 7.16 (m, 3H), 2.89 – 2.73 (m, 2H), 2.61 – 2.52 (m, 2H), 2.32 (s, 6H); **HRMS:** m/z calculated for $C_{10}H_{16}N$ $[M + H]^+$ 150.1283; found 150.1285.

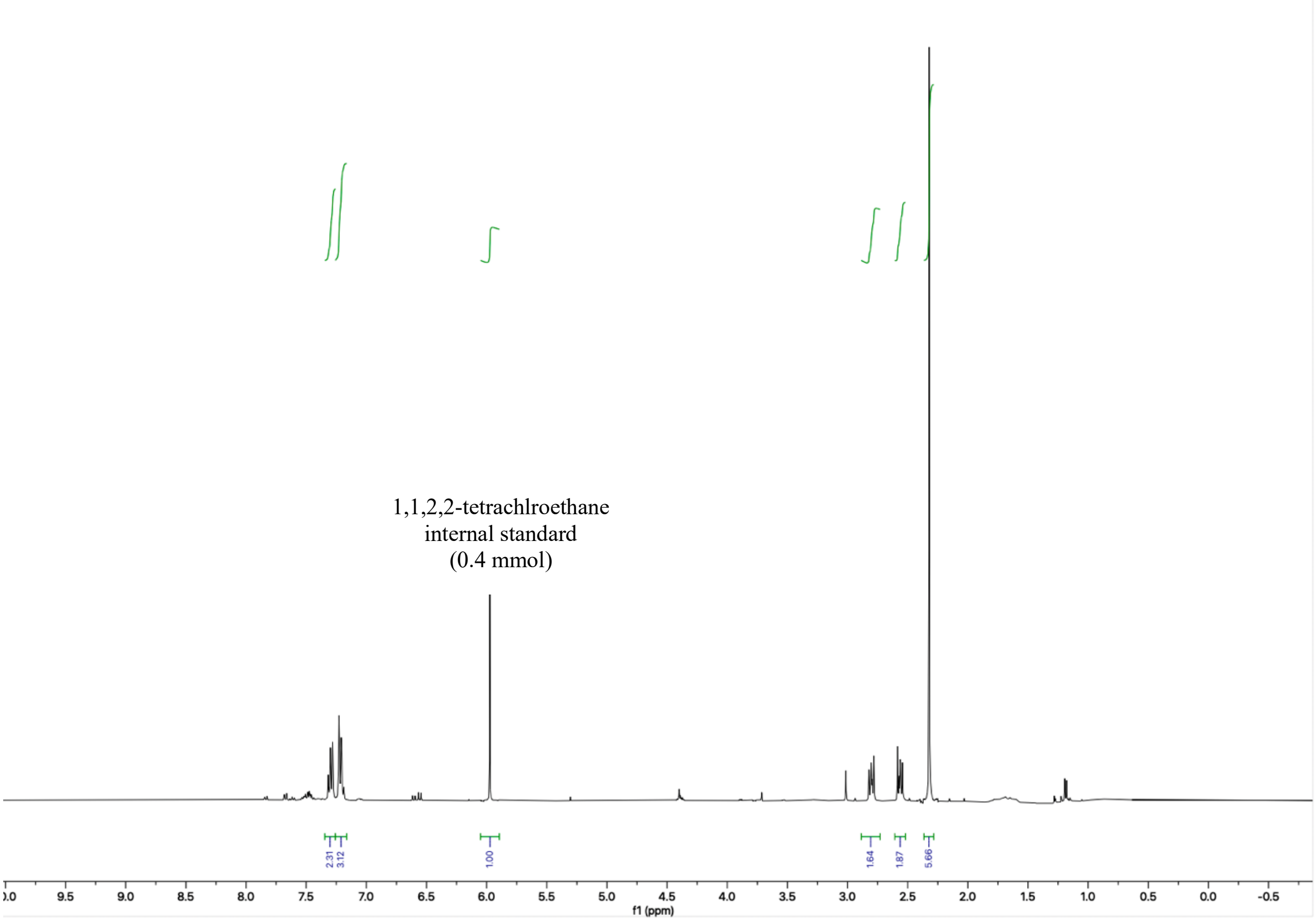

N-methyl-*N*-phenethylbut-3-en-1-amine (**2p**)

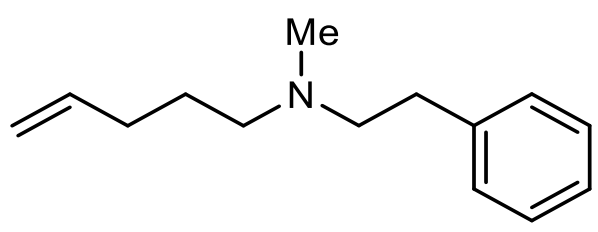

Prepared by general procedure **2.5.3.3.** using ((benzyl(methyl)(pent-4-en-1-yl)ammonio)methyl)trifluoroborate (**4p**) (54 mg, 0.20 mmol, 1.0 equiv), Pd-PEPPSI^{TM}-IPent (7.9 mg, 0.01 mmol, 5 mol%), isopropanol (3.1 µL, 0.04 mmol, 0.20 equiv), and potassium phosphate tribasic (424 mg, 2.0 mmol, 10 equiv) in 4:1 1,4-dioxane/water (1.0 mL). Purification by automated flash column chromatography (1%–10% acetone in petroleum ether) followed by purification by SCX chromatography (eluting with 4 N NH_3 in methanol) gave the title compound as a colourless oil (18 mg, 0.09 mmol, 45% yield). **^{1}H NMR** (700 MHz, $CDCl_3$) δ 7.28 (t, *J* = 7.7 Hz, 2H), 7.22 – 7.18 (m, 3H), 5.82 (ddt, *J* = 16.9, 10.0, 6.6 Hz, 1H), 5.02 (dq, *J* = 17.1, 1.7 Hz, 1H), 4.96 (ddd, *J* = 10.2, 2.2, 1.1 Hz, 1H), 2.80 – 2.76 (m, 2H), 2.63 – 2.59 (m, 2H), 2.43 – 2.39 (m, 2H), 2.31 (s, 3H), 2.09 – 2.04 (m, 2H), 1.59 (p, *J* = 7.4 Hz, 2H); **^{13}C NMR** (176 MHz, $CDCl_3$) δ 140.8, 138.7, 128.8, 128.5, 126.1,

114.7, 59.8, 57.2, 42.4, 34.0, 31.8, 26.7; **IR** (film, cm^{-1}): 2935, 2789, 1641, 1496, 1454, 1123, 1064, 993, 909; **HRMS:** m/z calculated for $C_{14}H_{22}N$ $[M+H]^+$ 204.1747; found 204.1749.

N,N-dimethyl-2-(methyl(phenethyl)amino)acetamide (**2q**)

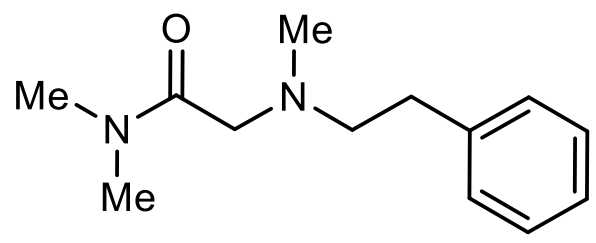

Prepared by general procedure **2.5.3.3.** using ((benzyl(2-(dimethylamino)-2-oxoethyl)(methyl)ammonio)methyl)trifluoroborate (**4q**) (115 mg, 0.40 mmol, 1.0 equiv), Pd-PEPPSI™-IPent (7.9 mg, 0.01 mmol, 5 mol%), isopropanol (3.1 µL, 0.04 mmol, 0.20 equiv), and potassium phosphate tribasic (849 mg, 4.0 mmol, 10 equiv) in 4:1 1,4-dioxane/water (2.0 mL). Purification by automated flash column chromatography (5–50% acetone in petroleum ether) gave the title compound as a colourless oil (57 mg, 0.26 mmol, 65% yield). **^{1}H NMR** (500 MHz, $CDCl_3$) δ 7.29 – 7.24 (m, 2H), 7.20 – 7.17 (m, 3H), 3.20 (s, 2H), 2.89 (d, *J* = 6.2 Hz, 6H), 2.79 (dd, *J* = 8.4, 5.7 Hz, 2H), 2.71 (ddd, *J* = 8.2, 6.4, 1.2 Hz, 2H), 2.36 (s, 3H); **^{13}C NMR** (126 MHz, $CDCl_3$) 170.3, 140.6, 128.9, 128.4, 126.0, 61.0, 59.2, 42.3, 37.0, 35.6, 33.9; **IR** (film, cm^{-1}): 2931, 2853, 1642, 1497, 1455, 1399, 1264, 1120, 1053, 749; **HRMS:** m/z calculated for $C_{13}H_{21}N_2O$ $[M + H]^+$ 221.1648; found 221.1642.

(*S*)-*N*-methyl-3-(naphthalen-1-yloxy)-*N*-phenethyl-3-(thiophen-2-yl)propan-1-amine (**2r**)

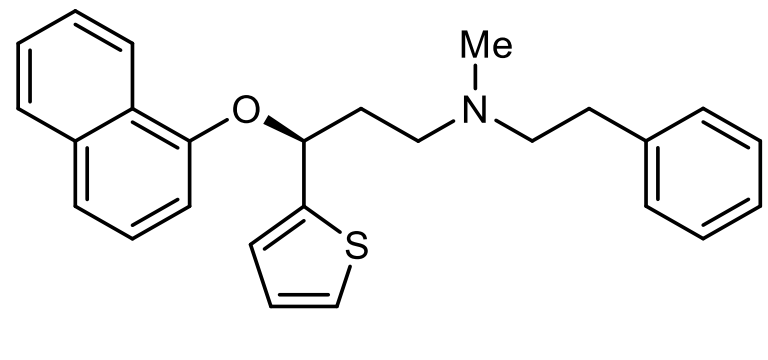

Prepared by general procedure **2.5.3.1.** ((benzyl(methyl)((*S*)-3-(naphthalen-1-yloxy)-3-(thiophen-2-yl)propyl)ammonio)methyl) trifluoroborate (**4r**) (94 mg, 0.20 mmol, 1.0 equiv), bis(benzonitrile)palladium(II) chloride (3.8 mg, 0.01 mmol, 5 mol%), RuPhos (9.3 mg, 0.02 mmol, 10 mol%), and potassium *tert*-butoxide (112 mg, 1.0 mmol, 5.0 equiv) in 4:1 1,4-dioxane/water (1.0 mL). Purification by automated column chromatography (40% ethyl acetate in petroleum ether) gave the title compound as a colourless oil (44 mg, 0.11 mmol, 54% yield). **^{1}H NMR** (700 MHz, $CDCl_3$) δ 8.41 – 8.34 (m, 1H), 7.83 – 7.77 (m, 1H), 7.50 (dt, *J* = 6.8, 3.4 Hz, 2H), 7.42 (d, *J* = 8.7 Hz, 1H), 7.31 – 7.27 (m, 3H), 7.23 (dd, *J* = 5.0, 1.2 Hz, 1H), 7.22 – 7.19 (m, 1H), 7.18 – 7.14 (m, 2H), 7.02 (dt, *J* = 3.5, 1.2 Hz, 1H), 6.95 (dd, *J* = 5.0, 3.5 Hz, 1H), 6.84 (d, *J* = 7.4 Hz, 1H), 5.74 (dd, *J* = 7.7, 5.4 Hz,

1H), 2.76 (dd, J = 9.2, 6.4 Hz, 2H), 2.70 (ddd, J = 12.6, 7.7, 6.6 Hz, 1H), 2.68 – 2.60 (m, 3H), 2.46 (dtd, J = 13.6, 7.7, 5.9 Hz, 1H), 2.36 (s, 3H), 2.25 – 2.17 (m, 1H); **^{13}C NMR** (176 MHz, $CDCl_3$) δ 153.5, 145.4, 140.6, 134.6, 128.8, 128.3, 127.5, 126.5, 126.3, 126.2, 126.0, 125.8, 125.2, 124.67, 124.66, 122.2, 120.5, 107.1, 74.5, 59.5, 53.6, 42.2, 36.7, 33.8; **IR** (film, cm^{-1}): 2789, 1594, 1577, 1506, 1461, 1450, 1395, 1369, 1263, 1234, 1192, 1154, 1093, 822, 789, 770, 748, 729, 696, 657; **HRMS:** m/z calculated for $C_{26}H_{28}NOS$ $[M + H]^+$ 402.1892; found 402.1891.

6,7-Dimethoxy-2-phenethyl-1,2,3,4-tetrahydroisoquinoline (**2s**)

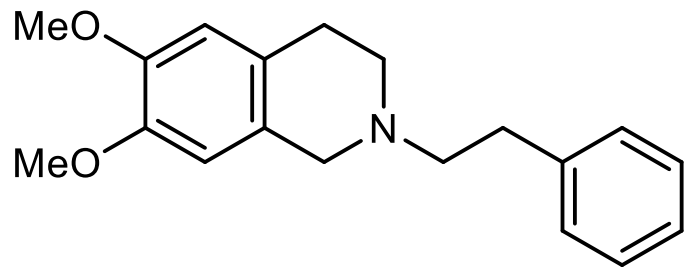

Prepared by general procedure **2.5.3.1.** using ((2-benzyl-6,7-dimethoxy-1,2,3,4-tetrahydroisoquinolin-2-ium-2-yl)methyl)trifluoroborate (**4s**) (73 mg, 0.20 mmol, 1.0 equiv), bis(benzonitrile)palladium(II) chloride (3.8 mg, 0.01 mmol, 5.0 mol%), RuPhos (9.3 mg, 0.02 mmol, 10 mol%) and potassium *tert*-butoxide (112 mg, 1.0 mmol, 5.0 equiv) in 4:1 1,4-dioxane/water (1.0 mL). Purification by automated flash column chromatography (0–10% methanol in dichloromethane) gave the title compound as a colourless oil (43 mg, 0.14 mmol, 73% yield). Data was in line with previous characterisation.[88] **^{1}H NMR** (700 MHz, $CDCl_3$) δ 7.34 – 7.31 (m, 2H), 7.29 – 7.26 (m, 2H), 7.24 (d, J = 7.3 Hz, 1H), 6.63 (s, 1H), 6.57 (s, 1H), 3.87 (s, 3H), 3.87 (s, 3H), 3.68 (s, 2H), 2.98 – 2.92 (m, 2H), 2.88 (t, J = 5.9 Hz, 2H), 2.84 – 2.78 (m, 4H); **^{13}C NMR** (176 MHz, $CDCl_3$) δ 147.6, 147.2, 140.4, 128.8, 128.4, 126.5, 126.2, 126.1, 111.4, 109.5, 60.3, 56.0, 55.9, 55.7, 51.1, 34.1, 28.7.

2-phenethyl-2,3,4,9-tetrahydro-1*H*-pyrido[3,4-*b*]indole (**2t**)

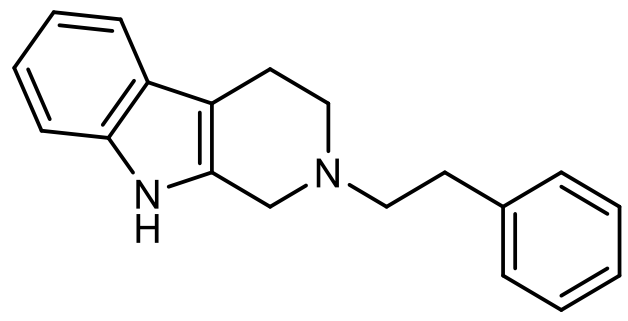

Prepared by general procedure **2.5.3.1.** ((2-benzyl-2,3,4,9-tetrahydro-1*H*-pyrido[3,4-*b*]indol-2-ium-2-yl)methyl)trifluoroborate (**4t**) (67 mg, 0.20 mmol, 1.0 equiv), bis(benzonitrile)palladium(II) chloride (3.8 mg, 0.01 mmol, 5.0 mol%), RuPhos (9.3 mg, 0.02 mmol, 10 mol%), and potassium *tert*-butoxide (112 mg, 1.0 mmol, 5.0 equiv) in 4:1 1,4-dioxane/water (1.0 mL). Purification by automated flash column chromatography (70–100% ethyl acetate in petroleum ether) gave the title compound as a

white solid (29 mg, 0.10 mmol, 52% yield). **^{1}H NMR** (500 MHz, $CDCl_3$) δ 7.89 – 7.80 (m, 1H), 7.51 (d, *J* = 1.3 Hz, 1H), 7.37 – 7.23 (m, 6H), 7.19 – 7.09 (m, 2H), 3.77 (s, 2H), 3.01 – 2.94 (m, 4H), 2.93 – 2.86 (m, 4H); **^{13}C NMR** (126 MHz, $CDCl_3$) δ 140.3, 136.1, 131.8, 128.8, 128.5, 127.3, 126.2, 121.4, 119.4, 118.0, 110.7, 108.5, 59.7, 51.1, 50.5, 34.3, 21.4; **IR** (film, cm^{-1}): 2835, 1666, 1452, 1380, 1312, 1162, 903, 735, 710, 694; **HRMS:** m/z calculated for $C_{19}H_{21}N_2$ $[M + H]^+$ 277.1705; found 277.1704.

N-Methyl-*N*-phenethylaniline (**2u**)

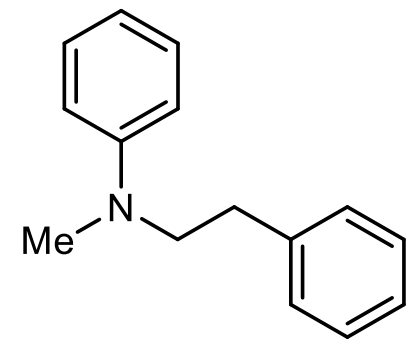

Prepared by general procedure **2.5.3.1.** using ((benzyl(methyl)(phenyl)ammonio)methyl)trifluoroborate (**4u**) (112 mg, 0.40 mmol, 1.0 equiv), bis(benzonitrile)palladium(II) chloride (7.7 mg, 0.02 mmol, 5.0 mol%), RuPhos (19 mg, 0.04 mmol, 10 mol%) and potassium *tert*-butoxide (224 mg, 2.0 mmol, 5.0 equiv) in 4:1 1,4-dioxane/water (2.0 mL). Purification by automated flash column chromatography (0–10% ethyl acetate in petroleum ether) gave the title compound as a colourless oil (67 mg, 0.32 mmol, 79% yield). Data was in line with previous characterisation.[85] **^{1}H NMR** (500 MHz, $CDCl_3$) δ 7.38 – 7.32 (m, 2H), 7.32 – 7.24 (m, 5H), 6.83 – 6.72 (m, 3H), 3.64 – 3.57 (m, 2H), 2.94 (s, 3H), 2.92 – 2.86 (m, 2H); **^{13}C NMR** (126 MHz, $CDCl_3$) δ 148.8, 139.9, 129.3, 128.8, 128.6, 126.2, 116.2, 112.2, 54.8, 38.5, 32.9.

1-Phenethyl-4-phenylpiperazine (**2v**)

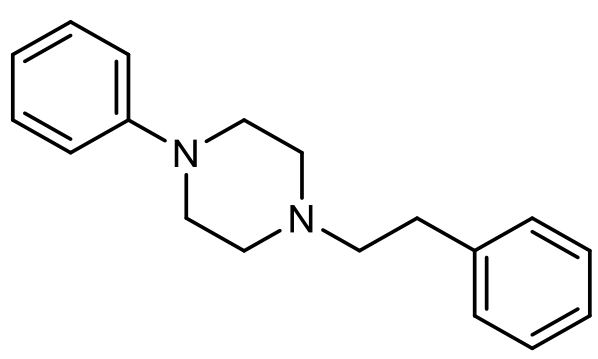

Prepared by general procedure **2.5.3.1.** ((1-benzyl-4-phenylpiperazin-1-ium-1-yl)methyl)trifluoroborate (**4v**) (67 mg, 0.20 mmol, 1.0 equiv), bis(benzonitrile)palladium(II) chloride (3.8 mg, 0.01 mmol, 5 mol%), RuPhos (9.3 mg, 0.02 mmol, 10 mol%), and potassium *tert*-butoxide (112 mg, 1.0 mmol, 5.0 equiv) in 4:1 1,4-dioxane/H_2O (1.0 mL). Purification by automated flash column chromatography (30% ethyl acetate in petroleum ether) gave the title compound as a white solid (48 mg, 0.18 mmol, 89% yield). Data was in line with previous characterisation.[86] **^{1}H NMR** (700 MHz, $CDCl_3$) δ 7.36 – 7.28 (m, 4H), 7.28 – 7.22 (m, 3H), 6.98 (dt, *J* = 7.8, 1.0 Hz, 2H), 6.91 – 6.88

(m, 1H), 3.31 – 3.24 (m, 4H), 2.91 – 2.87 (m, 2H), 2.77 – 2.72 (m, 4H), 2.72 – 2.68 (m, 2H); **^{13}C NMR** (176 MHz, $CDCl_3$) δ 151.4, 140.3, 129.1, 128.7, 128.4, 126.1, 119.7, 116.1, 60.6, 53.3, 49.2, 33.7.

tert-butyl (*S*)-3-methyl-4-phenethylpiperazine-1-carboxylate (**2w**)

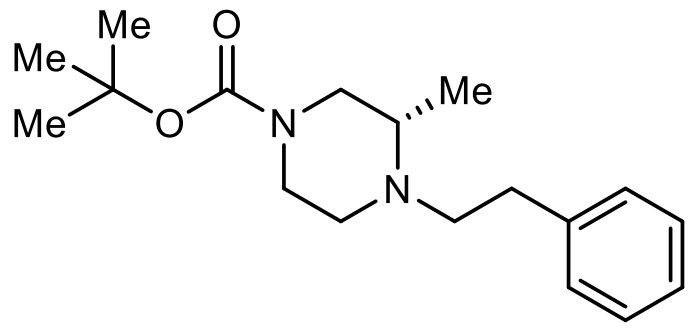

Prepared by general procedure **2.5.3.1.** using (((2S)-1-benzyl-4-(tert-butoxycarbonyl)-2-methylpiperazin-1-ium-1-yl)methyl)trifluoroborate (**4w**) (62 mg, 0.15 mmol, 1.0 equiv), bis(benzonitrile)palladium(II) chloride (3.8 mg, 0.01 mmol, 6.7 mol%), RuPhos (9.3 mg, 0.02 mmol, 13 mol%), and potassium *tert*-butoxide (112 mg, 1.0 mmol, 6.7 equiv) in 4:1 1,4-dioxane/water (0.75 mL). Purification by automated flash column chromatography (1–20% ethyl acetate in petroleum ether) gave the title compound as a colourless oil (36 mg, 0.12 mmol, 80% yield). **^{1}H NMR** (500 MHz, $CDCl_3$) δ 7.29 (dd, *J* = 8.2, 6.8 Hz, 2H), 7.22 – 7.17 (m, 3H), 3.87 – 3.57 (m, 2H), 3.15 (ddd, *J* = 13.3, 10.0, 3.1 Hz, 1H), 2.94 – 2.68 (m, 3H), 2.64 (ddd, *J* = 12.3, 10.4, 5.4 Hz, 1H), 2.48 (dqd, *J* = 9.3, 6.1, 3.1 Hz, 1H), 2.43 – 2.35 (m, 1H), 1.46 (s, 9H), 1.06 (d, *J* = 6.3 Hz, 3H); **^{13}C NMR** (126 MHz, $CDCl_3$) δ 154.8, 140.5, 128.8, 128.6, 126.2, 79.7, 55.7, 54.3, 50.6, 49.8, 44.5, 43.4, 32.4, 28.6, 15.3; **IR** (film, cm^{-1}): 2975, 2930, 2857, 2811, 1693, 1454, 1422, 1366, 1266, 1245, 1169, 1128, 887; **HRMS:** m/z calculated for $C_{18}H_{29}N_2O_2$ $[M + H]^+$ 305.2224; found 305.2225.
Note: the compound was isolated as a mixture of rotamers.

(*R*)-*N*,*N*-dimethyl-2-phenylpropan-1-amine (**2x**)

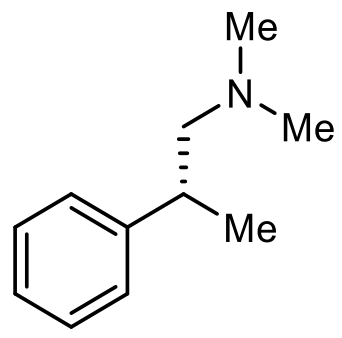

Prepared by general procedure **2.5.3.2.** using (*S*)-((dimethyl(1-phenylethyl)ammonio)methyl)trifluoroborate (**4x**) (93 mg, 0.40 mmol, 1.0 equiv), Pd-PEPPSI^{TM}-IPent (7.9 mg, 0.01 mmol, 5 mol%), and potassium *tert*-butoxide (224 mg, 2.0 mmol, 5.0 equiv), in 4:1 1,4-dioxane/water (2.0 mL). Purification by automated flash column chromatography (0–10% methanol in dichloromethane) gave the title compound as a colourless oil (25.0 mg, 0.15 mmol, 42% yield). Data was in line with previous characterisation.[87] **^{1}H NMR** (500 MHz, $CDCl_3$) δ 7.38 – 7.33 (m, 2H), 7.29

– 7.22 (m, 3H), 2.95 (app h, J = 7.1 Hz, 1H), 2.46 (dd, J = 7.5, 1.8 Hz, 2H), 2.28 (s, 6H), 1.32 (d, J = 6.9 Hz, 3H); **^{13}C NMR** (176 MHz, $CDCl_3$) δ 146.5, 128.7, 127.4, 126.5, 67.9, 46.2, 38.4, 20.5; **Enantiomeric excess** of >99% was determined by GC-MS using chiral column Astec CHIRALDEX^{TM} B-DM (20 m x 0.25 mmID, 0.12 μm df); method: 25 to 70 °C for 1 min; ramp 3°C/min up to 120 °C, hold 5 min; ramp 15 °C/min up to 200 °C; major enantiomer t_R = 6.454 min, minor enantiomer not detected]. The absolute configuration of **(2y)** was assigned by chiral GC-MS using an authentic standard with known configuration.

GC traces:

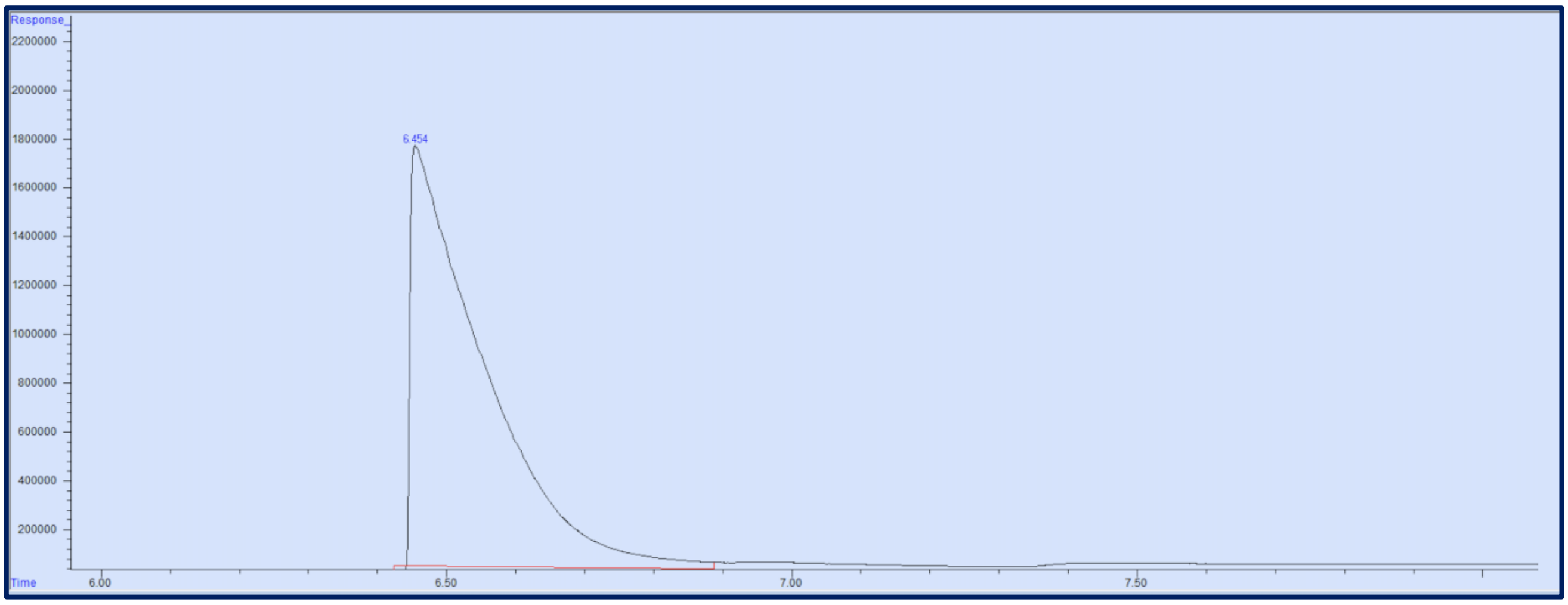

Peak	Retention Time (min)	Peak Height	Peak Area	Area%
1	6.454	1723550	125983077	100

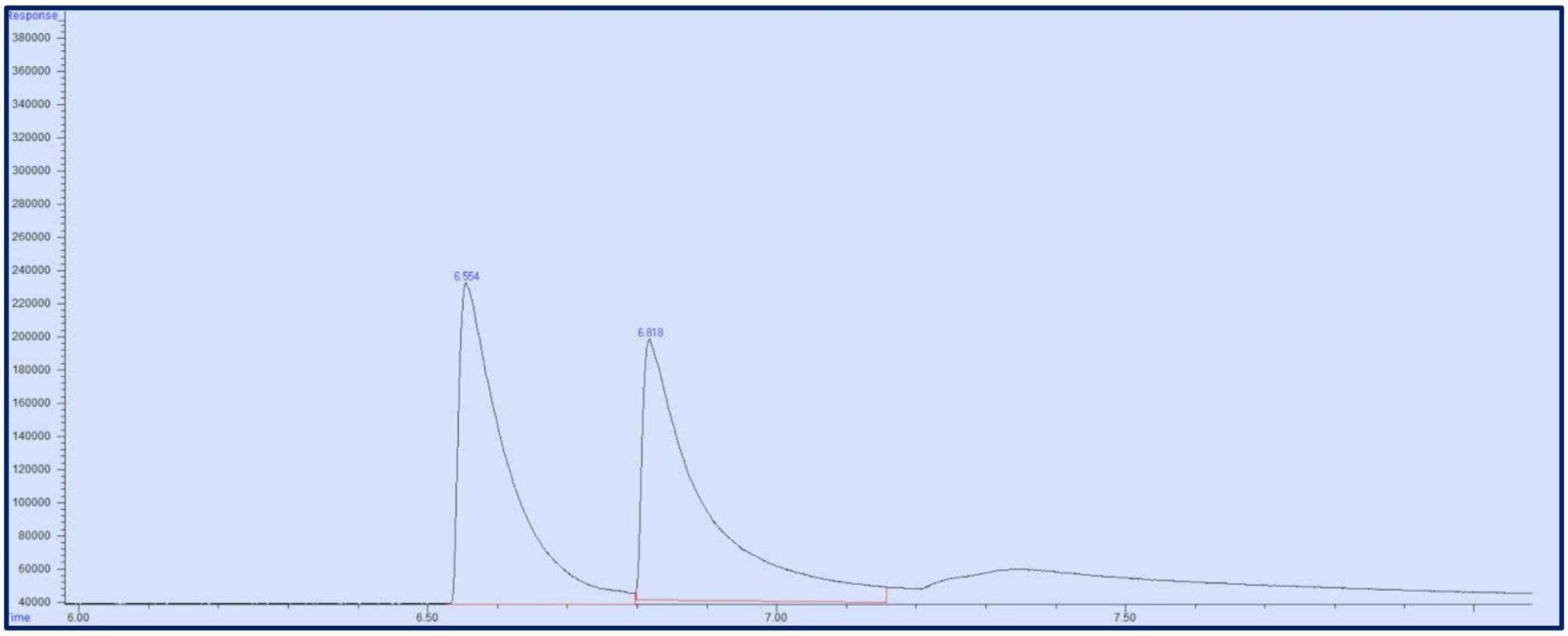

Peak	Retention Time (min)	Peak Height	Peak Area	Area%
1	6.554	193728	9218196	49.7%
2	6.818	157367	9327249	50.3%

Analytical standard GC trace: (*R*)-*N*,*N*-dimethyl-2-phenylpropan-1-amine

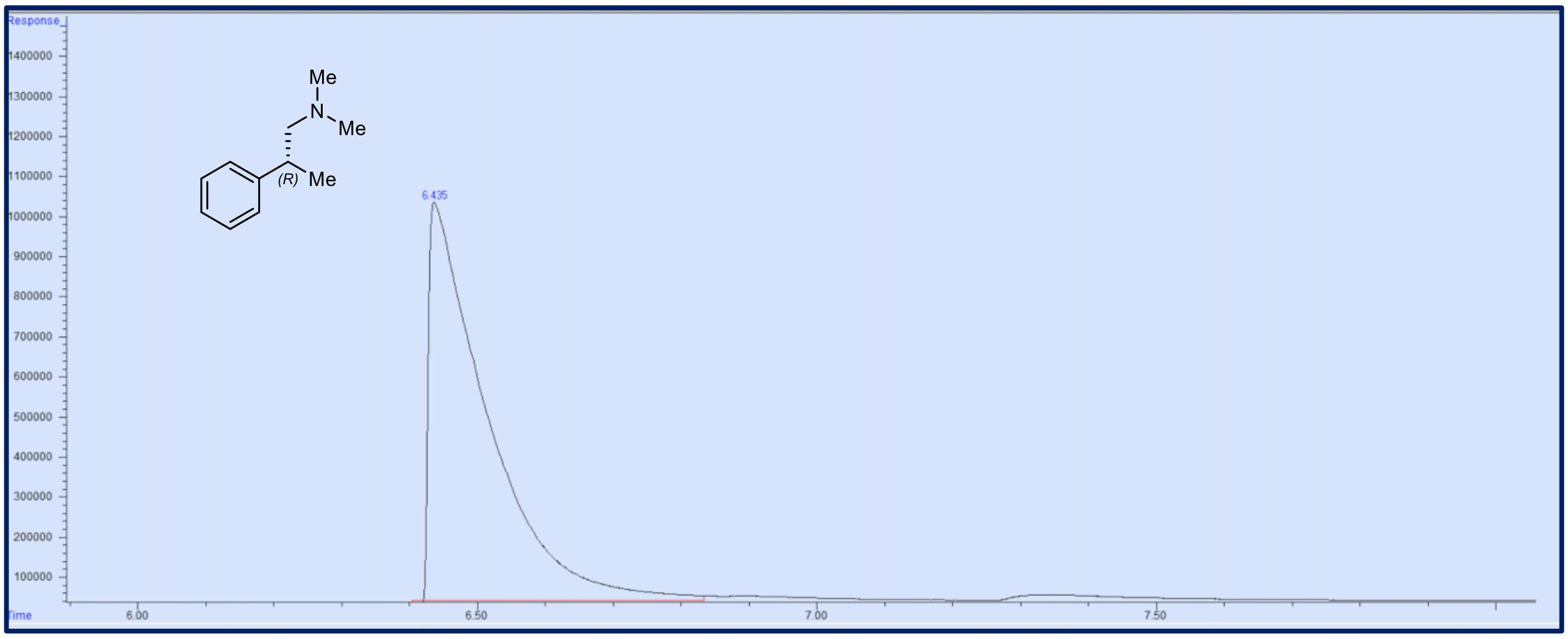

Peak	Retention Time (min)	Peak Height	Peak Area	Area%
1	6.435	988472	59213017	100

4-(4-methoxyphenethyl)morpholine (**2aa**)

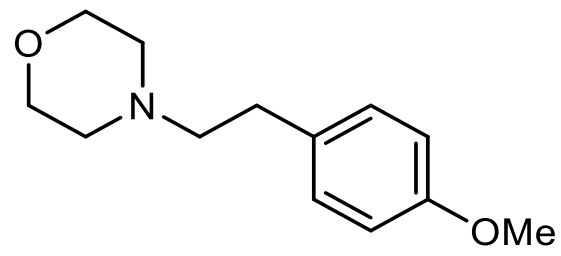

Prepared by general procedure **2.5.3.1.** using trifluoro((4-(4-methoxybenzyl)morpholino-4-ium)methyl)borate (**4aa**) (58 mg, 0.20 mmol, 1.0 equiv), bis(benzonitrile)palladium(II) chloride (3.8 mg, 0.01 mmol, 5 mol%), RuPhos (9.3 mg, 0.02 mmol, 10 mol%), and potassium *tert*-butoxide (112 mg, 1.0 mmol, 5.0 equiv) in 4:1 1,4-dioxane/water (1.0 mL). Purification by automated flash column chromatography (0–10% methanol in dichloromethane) gave the title compound as a colourless oil (36 mg, 0.16 mmol, 82% yield). Data in line with previous characterisation.[87] **^{1}H NMR** (700 MHz, $CDCl_3$) δ 7.16 – 7.08 (m, 2H), 6.87 – 6.79 (m, 2H), 3.78 (s, 3H), 3.77 – 3.71 (m, 4H), 2.77 – 2.72 (m, 2H), 2.59 – 2.55 (m, 2H), 2.55 – 2.46 (m, 4H); **^{13}C NMR** (176 MHz, $CDCl_3$) δ 158.0, 132.2, 129.6, 113.9, 67.0, 61.2, 55.3, 53.8, 32.4.

1-(4-(*tert*-butyl)phenethyl)piperidine (**2ab**)

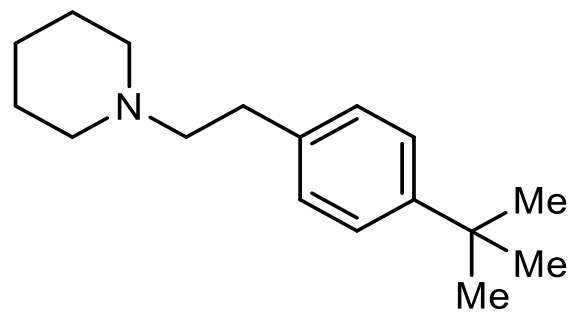

Prepared by general procedure **2.5.3.1.** using ((1-(4-(*tert*-butyl)benzyl)piperidin-1-ium-1-yl)methyl)trifluoroborate (**4ab**) (63 mg, 0.20 mmol, 1.0 equiv), bis(benzonitrile)palladium(II) chloride (3.8 mg, 0.01 mmol, 5 mol%), RuPhos (9.3 mg, 0.02 mmol, 10 mol%), and potassium *tert*-butoxide (112 mg, 1.0 mmol, 5.0 equiv) in 4:1 1,4-dioxane/water (1.0 mL). Purification by automated flash column chromatography (100% petroleum ether for 1 minute, 0–30% ethyl acetate in petroleum ether for 15 minutes, 30–50% ethyl acetate in petroleum ether for 2 minutes, and 50–100% ethyl acetate in petroleum ether for 2 minutes) gave the title compound as a colourless oil (32 mg, 0.13 mmol, 64% yield). Data was in line with previous characterisation.[88] **^{1}H NMR** (700 MHz, $CDCl_3$) δ 7.33 – 7.28 (m, 2H), 7.16 – 7.12 (m, 2H), 2.82 – 2.77 (m, 2H), 2.59 – 2.54 (m, 2H), 2.48 (br s, 4H), 1.63 (p, J = 5.7 Hz, 4H), 1.47 (br s, 2H), 1.31 (s, 9H); **^{13}C NMR** (176 MHz, $CDCl_3$) δ 148.9, 137.6, 128.5, 125.4, 61.6, 54.7, 34.5, 33.1, 31.5, 26.1, 24.5.

4-(4-(trifluoromethyl)phenethyl)morpholine (**2ac**)

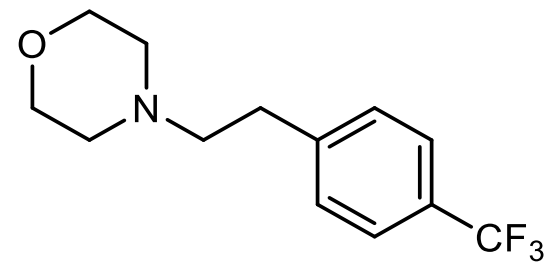

Prepared by general procedure **2.5.3.2.** using trifluoro((4-(4-(trifluoromethyl)benzyl)morpholino-4-ium)methyl)borate (**4ac**) (33 mg, 0.10 mmol, 1.0 equiv), Pd-PEPPSI^{TM}-IPent (7.9 mg, 0.01 mmol, 10 mol%), and potassium *tert*-butoxide (112 mg, 1.0 mmol, 10 equiv) in 4:1 1,4-dioxane/water (0.50 mL). Purification by automated flash column chromatography (100% petroleum ether for 2 minutes, 0–100% ethyl acetate in petroleum ether for 21 minutes, 100% ethyl acetate for 2 minutes) gave the title compound as a yellow oil (19 mg, 0.07 mmol, 75% yield). Data was in line with previous characterization.[79] **^{1}H NMR** (500 MHz, $CDCl_3$) δ 7.54 (d, J = 8.0 Hz, 2H), 7.32 (d, J = 7.9 Hz, 2H), 3.74 (m, 4H), 2.85 (t, J = 6.4 Hz, 2H), 2.60 (t, J = 6.4 Hz, 2H), 2.54 – 2.49 (m, 4H); **^{13}C NMR** (101 MHz, $CDCl_3$) δ 144.4, 129.1, 128.8, 128.5, 125.5 (q, J = 4.0 Hz), 67.0, 60.3, 53.7, 33.2; **^{19}F NMR** (376 MHz, $CDCl_3$) δ −62.4.

4-(4-(trifluoromethyl)phenethyl)morpholine (**2ad**)

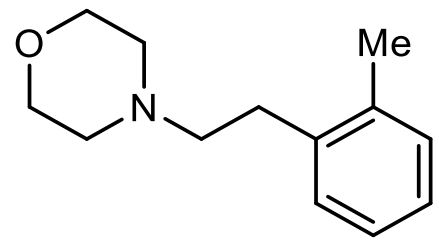

Prepared by general procedure **2.5.3.1.** using trifluoro((4-(2-methylbenzyl)morpholino-4-ium)methyl)borate (**4ad**) (109 mg, 0.40 mmol, 1.0 equiv), bis(benzonitrile)palladium (II) chloride (7.6 mg, 0.02 mmol, 5 mol%), RuPhos (19 mg, 0.04 mmol, 10 mol%), and potassium *tert*-butoxide (224 mg, 2.0 mmol, 5.0 equiv) in 4:1 1,4-dioxane/water (4.0 mL). Purification by automated flash column chromatography (100% petroleum ether for 2 minutes, 0–100% ethyl acetate in petroleum ether for 21 minutes, 100% ethyl acetate for 2 minutes) gave the title compound as a clear oil (51 mg, 0.25 mmol, 62% yield). Data was in line with previous characterization.[89] **^{1}H NMR** (400 MHz, $CDCl_3$) δ 7.18 – 7.06 (m, 4H), 3.75 (m, 4H), 2.85 – 2.76 (m, 2H), 2.62 – 2.46 (m, 6H), 2.32 (s, 3H); **^{13}C NMR** (176 MHz, $CDCl_3$) δ 138.2, 136.1, 130.4, 129.4, 126.4, 126.2, 67.0, 59.7, 53.8, 30.5, 19.4.

1-(4-fluoro-2-methoxyphenethyl)piperidine (**2ae**)

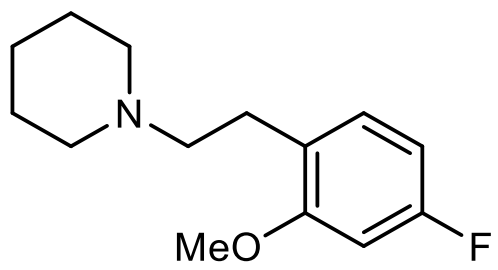

Prepared by general procedure **2.5.3.1.** using trifluoro((1-(4-fluoro-2-methoxybenzyl)piperidin-1-ium-1-yl)methyl)borate (**4ae**) (61 mg, 0.20 mmol, 1.0 equiv), bis(benzonitrile)palladium(II) chloride (3.8 mg, 0.01 mmol, 5 mol%), RuPhos (9.3 mg, 0.02 mmol, 10 mol%), and potassium *tert*-butoxide (112 mg, 1.0 mmol, 5 equiv) in 4:1 1,4-dioxane/water (1.0 mL). Purification by automated flash column chromatography (5–30% acetone in petroleum ether) gave the title compound as a yellow oil (28 mg, 0.12 mmol, 60% yield). **^{1}H NMR** (400 MHz, $CDCl_3$) δ 7.09 – 7.02 (m, 1H), 6.57 (dt, *J* = 6.4, 2.3 Hz, 1H), 6.54 (d, *J* = 2.1 Hz, 1H), 3.78 (s, 3H), 2.80 – 2.73 (m, 2H), 2.50 – 2.43 (m, 4H), 1.61 (p, *J* = 5.6 Hz, 4H), 1.44 (p, *J* = 6.0 Hz, 2H); **^{13}C NMR** (126 MHz, $CDCl_3$) δ 162.4 (d, *J* = 243.0 Hz), 158.6 (d, *J* = 9.5 Hz), 130.7 (d, *J* = 9.7 Hz), 124.6 (d, *J* = 3.3 Hz), 106.5 (d, *J* = 20.7 Hz), 98.8 (d, *J* = 25.5 Hz), 59.7 (d, *J* = 1.3 Hz), 55z.6, 54.6, 27.4, 26.1, 24.6; **^{19}F NMR** (376 MHz, $CDCl_3$) δ −115.5; **IR** (film, cm^{-1}): 2928, 2860, 1669, 1603, 1505, 1464, 1414, 1277, 1151, 1036, 954; **HRMS:** m/z calculated for $C_{14}H_{21}FNO$ $[M + H]^+$ 238.1614; found 238.1607.

1-(3,5-di-*tert*-butylphenethyl)piperidine (**2af**)

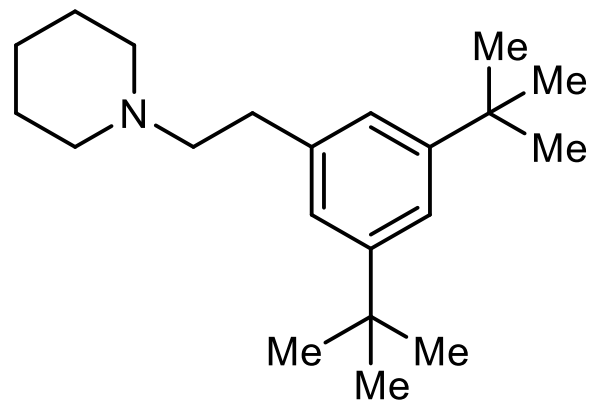

Prepared by general procedure **2.5.3.2.** using ((1-(3,5-di-*tert*-butylbenzyl)piperidin-1-ium-1-yl)methyl)trifluoroborate (**4af**) (40 mg, 0.10 mmol, 1.0 equiv), Pd-PEPPSI^{TM}-IPent (7.9 mg, 0.01 mmol, 10 mol%), isopropanol (1.2 mg, 0.02 mmol, 20 mol%), and potassium *tert*-butoxide (112 mg, 1.0 mmol, 10 equiv) in 4:1 1,4-dioxane/water (0.50 mL). Purification by automated flash column chromatography (0–30% acetone in petroleum ether for 15 minutes, 30% acetone in petroleum ether for 5 minutes, 0–20% methanol in dichloromethane for 5 minutes, 20% methanol in dichloromethane for 5 minutes) gave the title compound as a yellow oil (18 mg, 0.06 mmol, 60% yield). **^{1}H NMR** (700 MHz, CDCl3) δ 7.27 (d, *J* = 1.8 Hz, 1H), 7.06 (d, *J* = 1.8 Hz, 2H), 2.83 – 2.78 (m, 2H), 2.60 – 2.55 (m, 2H), 2.49 (br s, 4H), 1.64 (p, *J* = 5.6 Hz, 4H), 1.47 (br s, 2H), 1.32 (s, 18H); **^{13}C NMR** (176 MHz,

$CDCl_3$) δ 150.8, 139.8, 123.1, 120.1, 61.8, 54.7, 34.9, 34.4, 31.6, 26.2, 24.6; **IR** (thin film, cm^{-1}) 2933, 1474, 1443, 1108, 1020, 833, 739, 692; **HRMS:** m/z calculated for $C_{21}H_{36}N$ $[M+H]^+$ 302.2842; found 302.2846.

1-(2-(naphthalen-2-yl)ethyl)piperidine (**2ag**)

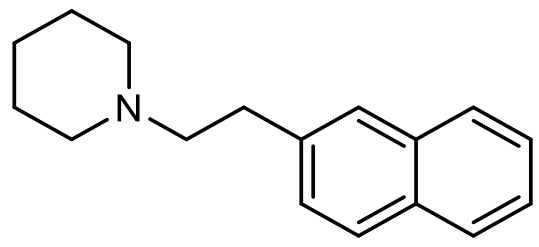

Prepared using general procedure **2.5.3.1.** using trifluoro((1-(naphthalen-2-ylmethyl)piperidin-1-ium-1-yl)methyl)borate (**4ag**) (61 mg, 0.20 mmol, 1.0 equiv), bis(benzonitrile)palladium (II) chloride (3.8 mg, 0.01 mmol, 5 mol%), RuPhos (9.3 mg, 0.02 mmol, 10 mol%), and potassium *tert*-butoxide (112 mg, 1.0 mmol, 5.0 equiv) in 4:1 1,4-dioxane/water (1.0 mL). Crude material was purified by automated flash column chromatography (100% petroleum ether for 2 minutes, 0–100% ethyl acetate in petroleum ether for 21 minutes, 100% ethyl acetate for 2 minutes) gave the title compound as a pale yellow oil (29 mg, 0.12 mmol, 60% yield). Data was in line with previous characterization.[88] **^{1}H NMR** (500 MHz, $CDCl_3$) δ 7.81 (m, 3H), 7.67 (s, 1H), 7.51 – 7.42 (m, 2H), 7.38 (dd, J = 8.4, 1.8 Hz, 1H), 3.05 – 2.97 (m, 2H), 2.72 – 2.65 (m, 2H), 2.54 (br s, 4H), 1.67 (p, J = 5.6 Hz, 4H), 1.50 (p, J = 6.1 Hz, 2H); **^{13}C NMR** (126 MHz, $CDCl_3$) δ 138.3, 133.7, 132.2, 128.0, 127.7, 127.7, 127.5, 126.9, 126.0, 125.3, 61.4, 54.7, 33.9, 26.1, 24.5.

1-(4-(2-(piperidin-1-yl)ethyl)phenyl)piperidine (**2ah**)

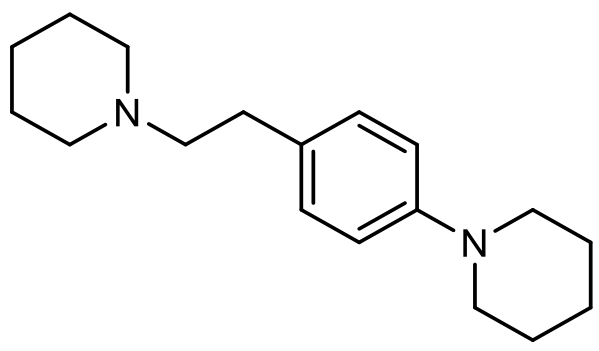

Prepared by general procedure **2.5.3.3.** using trifluoro((1-(4-(piperidin-1-yl)benzyl)piperidin-1-ium-1-yl)methyl)borate (**4ah**) (68 mg, 0.20 mmol, 1.0 equiv), Pd-PEPPSI™-IPent (7.9 mg, 0.01 mmol, 5 mol%), isopropanol (3.1 µL, 0.04 mmol, 0.20 equiv), and potassium phosphate tribasic (424 mg, 2.0 mmol, 10 equiv) in 4:1 1,4-dioxane/water (1.0 mL). Purification by automated flash column chromatography (5–30% acetone in petroleum ether) gave the title compound as a yellow oil (28 mg,

0.10 mmol, 50% yield). **^{1}H NMR** (400 MHz, $CDCl_3$) δ 7.10 – 7.06 (m, 2H), 6.89 – 6.84 (m, 2H), 3.13 – 3.07 (m, 4H), 2.76 – 2.70 (m, 2H), 2.56 – 2.50 (m, 2H), 2.46 (br s, 4H), 1.74 – 1.67 (m, 4H), 1.62 (p, *J* = 5.6 Hz, 4H), 1.58 – 1.52 (m, 2H), 1.46 (d, *J* = 5.5 Hz, 2H); **^{13}C NMR** (126 MHz, $CDCl_3$) δ 150.8, 131.4, 129.3, 116.9, 61.8, 54.7, 51.2, 32.7, 26.1, 26.1, 24.6, 24.4; **IR** (film, cm^{-1}): 2931, 2853, 2794, 1612, 1514, 1451, 1342, 1235, 1220, 1129, 1103, 918; **HRMS:** m/z calculated for $C_{18}H_{29}N_2$ [M + H]$^+$ 273.2325; found 273.2325.

1-(4-(2-(piperidin-1-yl)ethyl)phenyl)pyrrolidin-2-one (**2ai**)

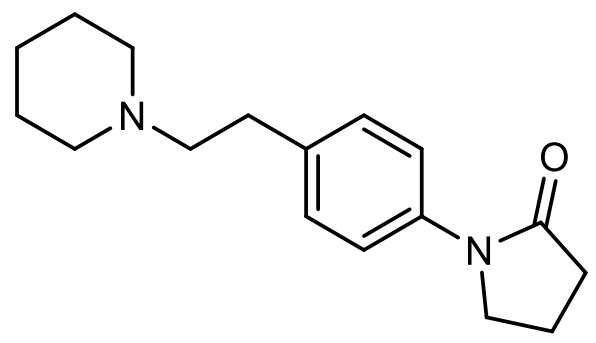

Prepared by general procedure **2.5.3.1.** using trifluoro((1-(4-(2-oxopyrrolidin-1-yl)benzyl)piperidin-1-ium-1-yl)methyl)borate (**4ai**) (68 mg, 0.20 mmol, 1.0 equiv), bis(benzonitrile)palladium(II) chloride (3.8 mg, 0.01 mmol, 5 mol%), RuPhos (9.3 mg, 0.02 mmol, 10 mol%), and potassium *tert*-butoxide (112 mg, 1.0 mmol, 5.0 equiv) in 4:1 1,4-dioxane/water (1.0 mL). Purification by automated flash column chromatography (10–70% acetone in dichloromethane) gave the title compound as a yellow solid (24 mg, 0.09 mmol, 44% yield). **^{1}H NMR** (500 MHz, $CDCl_3$) δ 7.52 – 7.48 (m, 2H), 7.22 – 7.17 (m, 2H), 3.84 (t, *J* = 7.0 Hz, 2H), 2.83 – 2.75 (m, 2H), 2.59 (t, *J* = 8.1 Hz, 2H), 2.56 – 2.50 (m, 2H), 2.46 (s, 4H), 2.15 (p, *J* = 7.6 Hz, 2H), 1.61 (p, *J* = 5.6 Hz, 4H), 1.46 (q, *J* = 6.0 Hz, 2H); **^{13}C NMR** (126 MHz, $CDCl_3$) δ 174.2, 137.5, 137.1, 129.2, 120.3, 61.5, 54.7, 49.0, 33.1, 32.8, 26.1, 24.5, 18.2; **IR** (film, cm^{-1}): 2935, 2853, 2766, 1639, 1515, 1394, 1303, 1226, 1112, 1041; **HRMS:** m/z calculated for $C_{17}H_{25}N_2O$ [M + H]$^+$ 273.1961; found 273.1962.

4-(2-(2,3-dihydrobenzo[*b*][1,4]dioxin-6-yl)ethyl)morpholine (**2aj**)

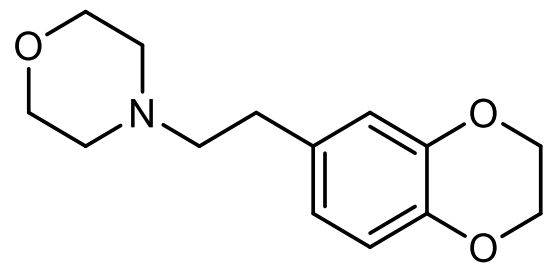

Prepared by general procedure **2.5.3.2.** using ((4-((2,3-dihydrobenzo[*b*][1,4]dioxin-6-yl)methyl)morpholino-4-ium)methyl)trifluoroborate (**4aj**) (63 mg, 0.20 mmol, 1.0 equiv), Pd-PEPPSI^{TM}-IPent (16 mg, 0.02 mmol, 10 mol%), isopropanol (3.1 μL, 0.04 mmol, 0.20 equiv) and

potassium *tert*-butoxide (224 mg, 2.0 mmol, 10 equiv) 4:1 1,4-dioxane/water (1.0 mL). Purification by automated flash column chromatography (0–30% acetone in petroleum ether for 15 minutes, 30% acetone in petroleum ether for 5 minutes, 0–20% methanol in dichloromethane for 3 minutes, 20% methanol in dichloromethane for 2 minutes) gave the title compound as a white solid (27 mg, 0.11 mmol, 55% yield). **^{1}H NMR** (700 MHz, $CDCl_3$) δ 6.77 (d, *J* = 8.2 Hz, 1H), 6.71 (d, *J* = 2.1 Hz, 1H), 6.66 (dd, *J* = 8.2, 2.1 Hz, 1H), 4.25 – 4.20 (m, 4H), 3.73 (t, *J* = 4.7 Hz, 4H), 2.72 – 2.67 (m, 2H), 2.57 – 2.53 (m, 2H), 2.51 (br s, 4H); **^{13}C NMR** (176 MHz, $CDCl_3$) δ 143.4, 142.0, 133.5, 121.7, 117.4, 117.2, 67.1, 64.5, 64.4, 61.1, 53.8, 32.6; **IR:** (thin film, cm^{-1}) 2857, 2807, 1508, 1282, 1258, 1115, 1068, 864, 805; **HRMS:** m/z calculated for $C_{14}H_{20}NO_3$ $[M+H]^+$ 250.1438; found 250.1443.

(4,4-difluoropiperidin-1-yl)(3-(2-(piperidin-1-yl)ethyl)phenyl)methanone (**2ak**)

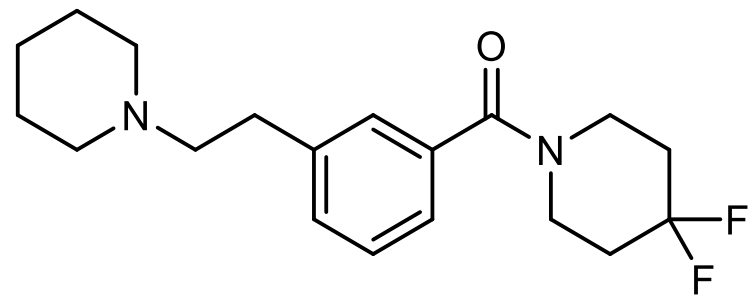

Prepared by general procedure **5.2.3.2.** using ((1-(3-(4,4-difluoropiperidine-1-carbonyl)benzyl)piperidin-1-ium-1-yl)methyl)trifluoroborate (**4ak**) (81 mg, 0.20 mmol, 1.0 equiv), Pd-PEPPSI^{TM}-IPent (16 mg, 0.02 mmol, 10 mol%), 2,6-di-*tert*-butyl-4-methylphenol (8.8 mg, 0.04 mmol, 20 mol%), and potassium *tert*-butoxide (112 mg, 1.0 mmol, 10 equiv) in 4:1 1,4-dioxane/water (1.0 mL). Purification by automated flash column chromatography (0–20% methanol in dichloromethane, 20% methanol in dichloromethane) gave the title compound as a pale yellow solid (15 mg, 0.04 mmol, 22% yield). **^{1}H NMR** (700 MHz, $CDCl_3$) δ 7.32 (t, *J* = 7.5 Hz, 1H), 7.28 (d, *J* = 7.9 Hz, 1H), 7.24 (s, 1H), 7.21 (dd, *J* = 7.6, 1.7 Hz, 1H), 3.70 (br d, 4H), 2.85 – 2.80 (m, 2H), 2.57 – 2.52 (m, 2H), 2.45 (br s, 4H), 2.14 – 1.89 (br d, 4H), 1.68 – 1.58 (m, 4H), 1.45 (br s, 2H); **^{13}C NMR** (176 MHz, $CDCl_3$) δ 170.9, 141.6, 135.4, 130.6, 128.7, 127.3, 124.5, 61.2, 54.7, 33.6, 26.2, 24.5; **^{19}F NMR** (376 MHz, $CDCl_3$) δ −97.9; **IR** (film, cm^{-1}): 2938, 1641, 1444, 1426, 1361, 1102, 952; **HRMS:** m/z calculated for $C_{19}H_{27}F_2N_2O$ $[M+H]^+$ 337.2086; found 337.2090.

1-(4-(3-(trifluoromethyl)-1H-pyrazol-1-yl)phenethyl)piperidine (**2al**)

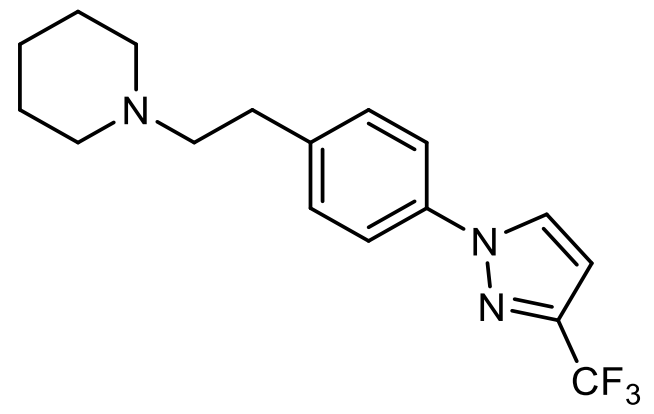

Prepared by general procedure **2.5.3.2.** using trifluoro((1-(4-(3-(trifluoromethyl)-1H-pyrazol-1-yl)benzyl)piperidin-1-ium-1-yl)methyl)borate (**4al**) (78 mg, 0.20 mmol, 1.0 equiv), Pd-PEPPSI^{TM}-IPent (7.9 mg, 0.01 mmol, 5 mol%), isopropanol (3.1 µL, 0.04 mmol, 0.20 equiv), and potassium *tert*-butoxide (112 mg, 1.0 mmol, 5.0 equiv) in 4:1 1,4-dioxane/water (1.0 mL). Purification by automated flash column chromatography (5–50% acetone in petroleum ether) gave the title compound as a yellow solid (47 mg, 0.15 mmol, 75% yield). **^{1}H NMR** (700 MHz, $CDCl_3$) δ 7.9 (dq, *J* = 2.6, 0.9 Hz, 1H), 7.6 – 7.6 (m, 2H), 7.3 – 7.3 (m, 2H), 6.7 (d, *J* = 2.5 Hz, 1H), 2.9 – 2.8 (m, 2H), 2.6 – 2.5 (m, 2H), 2.5 (s, 4H), 1.6 – 1.6 (m, 4H), 1.5 (q, *J* = 6.0 Hz, 2H); **^{13}C NMR** (176 MHz, $CDCl_3$) δ 143.9 (q, *J* = 38.4 Hz), 140.8, 137.7, 129.9, 128.4, 121.4 (q, *J* = 268.7 Hz), 120.1, 105.9 (q, *J* = 2.1 Hz), 61.2, 54.7, 33.3, 26.2, 24.6.; **^{19}F NMR** (376 MHz, $CDCl_3$) δ −63.03.; **IR** (film, cm^{-1}): 2937, 2856, 2803, 1513, 1484, 1390, 1274, 1131, 1054, 971, 951; **HRMS:** m/z calculated for $C_{17}H_{21}N_3F_3$ $[M + H]^+$ 324.1682; found 324.1681.

2-(3-(2-(piperidin-1-yl)ethyl)phenyl)pyridineone (**2am**)

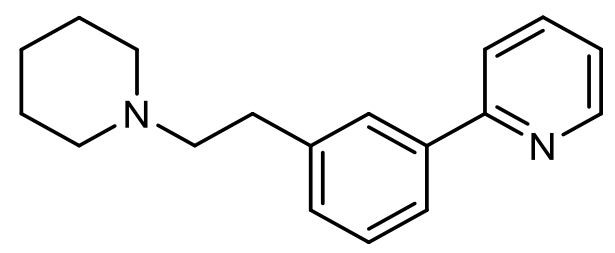

Prepared by general procedure **2.5.3.1.** using trifluoro((1-(3-(pyridin-2-yl)benzyl)piperidin-1-ium-1-yl)methyl)borate (**4am**) (66 mg, 0.20 mmol, 1.0 equiv), bis(benzonitrile)palladium(II) chloride (3.8 mg, 0.01 mmol, 5 mol%), RuPhos (9.3 mg, 0.02 mmol, 10 mol%), and potassium *tert*-butoxide (112 mg, 1.0 mmol, 5.0 equiv) in 4:1 1,4-dioxane/water (1.0 mL). Purification by automated flash column chromatography (5–50% acetone in petroleum ether) gave the title compound as a colourless oil (23 mg, 0.09 mmol, 43% yield). **^{1}H NMR** (700 MHz, $CDCl_3$) δ 8.69 (ddt, *J* = 4.8, 1.7, 0.8 Hz, 1H), 7.86 (t, *J* = 1.9 Hz, 1H), 7.79 (dt, *J* = 7.7, 1.5 Hz, 1H), 7.75 (dddd, *J* = 7.9, 7.2, 1.8, 0.7 Hz, 1H), 7.73 – 7.71 (m, 1H), 7.39 (t, *J* = 7.6 Hz, 1H), 7.27 (d, *J* = 7.9 Hz, 2H), 7.22 (dddd, *J* = 6.1, 4.8, 1.4, 0.7 Hz, 1H), 2.92 – 2.88 (m, 2H), 2.64 – 2.60 (m, 2H), 2.49 (s, 4H), 1.63 (p, *J* = 5.7 Hz, 4H), 1.46 (s, 2H); **^{13}C NMR** (176 MHz, $CDCl_3$) δ 157.7, 149.8, 141.4, 139.7, 136.9, 129.6, 128.9, 127.5, 124.8, 122.2, 120.8, 61.6,

54.7, 33.8, 26.2, 24.6; **IR** (film, cm^{-1}): 2935, 2854, 2797, 2761, 1586, 1463, 1437, 1155, 1113, 991; **HRMS:** m/z calculated for $C_{18}H_{23}N_2$ $[M + H]^+$ 267.1856; found 267.1855.

2-methyl-6-(2-(piperidin-1-yl)ethyl)quinoline (**2an**)

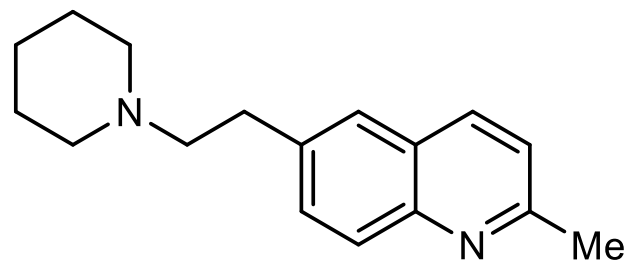

Prepared by general procedure **2.5.3.2.** using trifluoro((1-((2-methylquinolin-6-yl)methyl)piperidin-1-ium-1-yl)methyl)borate (**4an**) (62 mg, 0.20 mmol, 1.0 equiv), Pd-PEPPSI^{TM}-IPent (7.9 mg, 0.01 mmol, 5 mol%), isopropanol (3.1 µL, 0.04 mmol, 0.20 equiv), and potassium *tert*-butoxide (112 mg, 1.0 mmol, 5.0 equiv) in 4:1 1,4-dioxane/water (1.0 mL). Purification by automated flash column chromatography (5–50% acetone in petroleum ether) gave the title compound as a brown oil (12 mg, 0.05 mmol, 25% yield). **^{1}H NMR** (700 MHz, $CDCl_3$) δ 7.98 (d, *J* = 8.4 Hz, 1H), 7.93 (d, *J* = 8.6 Hz, 1H), 7.56 (d, *J* = 1.8 Hz, 1H), 7.54 (dd, *J* = 8.7, 2.0 Hz, 1H), 7.25 (d, 1H), 3.01 – 2.96 (m, 2H), 2.73 (s, 3H), 2.68 – 2.63 (m, 2H), 2.51 (s, 4H), 1.64 (p, *J* = 5.7 Hz, 4H), 1.47 (s, 2H); **^{13}C NMR** (176 MHz, $CDCl_3$) δ 158.4, 146.9, 138.2, 135.9, 131.2, 128.7, 126.6, 126.5, 122.2, 61.3, 54.7, 33.6, 26.1, 25.4, 24.5; **IR** (film, cm^{-1}): 2926, 2856, 1602, 1498, 1467, 1454, 1376, 1120, 833; **HRMS:** m/z calculated for $C_{17}H_{23}N_2$ $[M + H]^+$ 255.1856; found 255.1855.

5-(2-(piperidin-1-yl)ethyl)-2-(trifluoromethyl)pyridine (**2ao**)

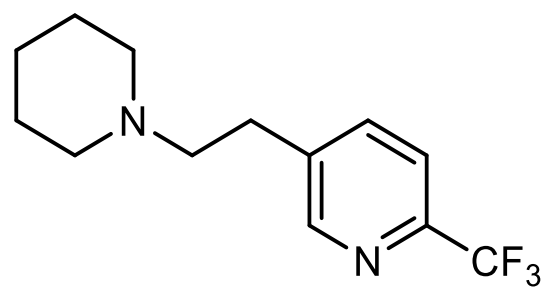

Prepared by general procedure **2.5.3.2.** using trifluoro((1-((6-(trifluoromethyl)pyridin-3-yl)methyl)piperidin-1-ium-1-yl)methyl)borate (**4ao**) (144 mg, 0.40 mmol, 1.0 equiv), Pd-PEPPSI^{TM}-IPent (32 mg, 0.04 mmol, 10 mol%), isopropanol (6.2 µL, 0.08 mmol, 0.20 equiv), and potassium *tert*-butoxide (448 mg, 4.0 mmol, 10 equiv) in 9:1 1,4-dioxane/water (4.0 mL). Purification by automated flash column chromatography (0–60% acetone in petroleum ether) gave the title compound as a brown oil (31 mg, 0.12 mmol, 30% yield). **^{1}H NMR** (700 MHz, $CDCl_3$) δ 8.58 (s, 1H), 7.71 (d, *J* = 8.0 Hz, 1H), 7.59 (d, *J* = 8.0 Hz, 1H), 2.87 (t, *J* = 7.8 Hz, 2H), 2.61 – 2.55 (m, 2H), 2.46 (s, 4H), 1.60 (p, *J* = 5.7 Hz, 4H), 1.46 (d, *J* = 8.0 Hz, 2H). **^{13}C NMR** (176 MHz, $CDCl_3$) δ 150.52, 146.20 (q, *J* = 34.7 Hz),

139.71, 137.51, 121.86 (q, J = 273.6 Hz), 120.20 (q, J = 2.8 Hz), 60.16, 54.61, 30.67, 26.06, 24.45; **^{19}F NMR** (471 MHz, $CDCl_3$) δ −67.7; **IR** (film, cm^{-1}): 2937, 2857, 2798, 2763, 1617, 1455, 1335, 1170, 1129, 1086, 1029, 839; **HRMS:** m/z calculated for $C_{13}H_{18}N_2F_3$ $[M+H]^+$ 259.1417; found 259.1417.

5-(2-(piperidin-1-yl)ethyl)-2-(trifluoromethyl)pyridine (**2ap**)

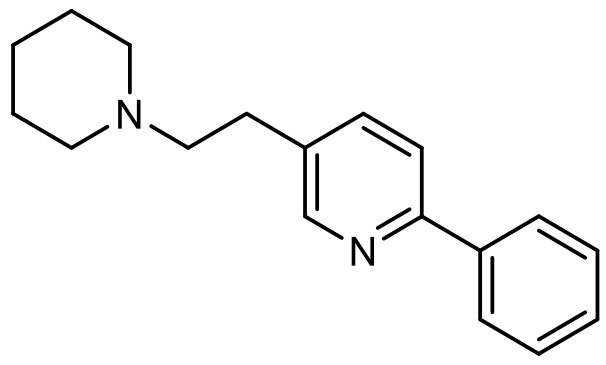

Prepared using general procedure **2.5.3.2.** using trifluoro((1-((6-phenylpyridin-3-yl)methyl)piperidin-1-ium-1-yl)methyl)borate (**4ap**) (33 mg, 0.10 mmol, 1.0 equiv), Pd-PEPPSI^{TM}-IPent (8 mg, 0.01 mmol, 10 mol%), isopropanol (1.6 µL, 0.02 mmol, 0.20 equiv), and potassium *tert*-butoxide (112 mg, 1.0 mmol, 10 equiv) in 4:1 1,4-dioxane/water (0.50 mL). Purification by automated flash column chromatography (0–20% methanol in dichloromethane for 20 minutes, 20% methanol in dichloromethane for 5 minutes) gave the title compound as a pale yellow oil (19 mg, 0.07 mmol, 72% yield). **^{1}H NMR** (500 MHz, $CDCl_3$) δ 8.54 (d, J = 2.3 Hz, 1H), 7.99 – 7.93 (m, 2H), 7.64 (d, J = 8.1 Hz, 1H), 7.60 – 7.55 (m, 1H), 7.48 – 7.42 (m, 2H), 7.41 – 7.35 (m, 1H), 2.87 – 2.78 (m, 2H), 2.61 – 2.53 (m, 2H), 2.46 (br s, 4H), 1.61 (p, J = 5.6 Hz, 4H), 1.45 (t, J = 6.0 Hz, 2H); **^{13}C NMR** (126 MHz, $CDCl_3$) δ 155.4, 150.1, 139.5, 137.1, 134.6, 128.8, 128.7, 126.8, 120.2, 60.9, 54.6, 30.6, 26.1, 24.5; **IR** (thin film, cm^{-1}) 2976, 2905, 1396, 1251, 1068, 881. **HRMS:** m/z calculated for $C_{18}H_{23}N_2$ $[M+H]^+$ 267.1856; found 267.1859.

5-(2-(piperidin-1-yl)ethyl)-2-(m-tolyl)pyrimidine (**2aq**)

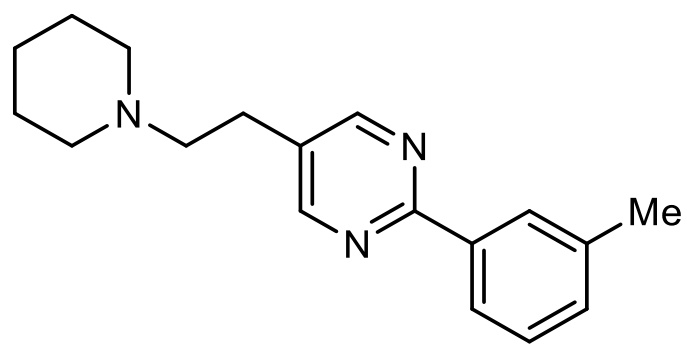

Prepared by general procedure **2.5.3.2.** using trifluoro((1-((2-(m-tolyl)pyrimidin-5-yl)methyl)piperidin-1-ium-1-yl)methyl)borate (**4aq**) (59 mg, 0.17 mmol, 1.0 equiv), Pd-PEPPSI^{TM}-IPent (6.7 mg, 0.01 mmol, 5 mol%), and potassium *tert*-butoxide (95 mg, 0.85 mmol, 5.0 equiv) in 4:1 1,4-dioxane/water (0.85 mL). Purification by automated flash column chromatography (5–50%

acetone in petroleum ether) gave the title compound as a yellow oil (34 mg, 0.12 mmol, 60% yield). **^{1}H NMR** (500 MHz, $CDCl_3$) δ 8.66 (s, 2H), 8.26 – 8.17 (m, 2H), 7.37 (t, *J* = 7.6 Hz, 1H), 7.31 – 7.26 (m, 1H), 2.79 (dd, *J* = 8.5, 6.7 Hz, 2H), 2.57 (dd, *J* = 8.5, 6.8 Hz, 2H), 2.51 – 2.39 (m, 7H), 1.61 (p, *J* = 5.6 Hz, 4H), 1.46 (q, *J* = 6.1 Hz, 2H); **^{13}C NMR** (126 MHz, $CDCl_3$) δ (126 MHz, $CDCl_3$) δ 163.1, 157.4, 138.4, 137.7, 131.4, 131.3, 128.7, 128.6, 125.2, 60.0, 54.6, 28.1, 26.1, 24.5, 21.6; **IR** (film, cm^{-1}): 2933, 2854, 2802, 1586, 1545, 1433, 1422, 1353, 1155, 1041, 775; **HRMS:** m/z calculated for $C_{18}H_{24}N_3$ $[M + H]^+$ 282.1965; found 282.1972.

2-phenyl-4-(2-(piperidin-1-yl)ethyl)thiazole (**2ar**)

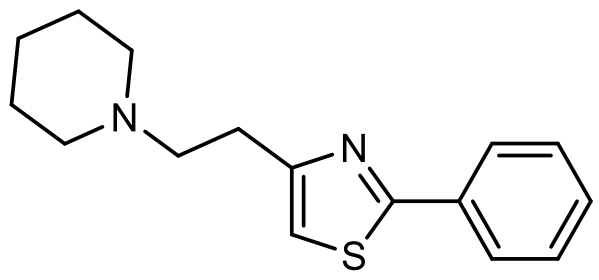

Prepared by general procedure **2.5.3.1.** using trifluoro((1-((2-phenylthiazol-4-yl)methyl)piperidin-1-ium-1-yl)methyl)borate (**4ar**) (52 mg, 0.20 mmol, 1.0 equiv), bis(benzonitrile)palladium(II) chloride (3.8 mg, 0.01 mmol, 5 mol%), RuPhos (9.3 mg, 0.02 mmol, 10 mol%), and potassium *tert*-butoxide (112 mg, 1.0 mmol, 5.0 equiv) in 4:1 1,4-dioxane/water (1.0 mL). Purification by automated flash column chromatography (0–20% acetone in petroleum ether) gave the title compound as a colourless oil (25 mg, 0.09 mmol, 45% yield). **^{1}H NMR** (500 MHz, $CDCl_3$) δ 7.95 – 7.91 (m, 2H), 7.46 – 7.37 (m, 3H), 6.96 (d, *J* = 0.8 Hz, 1H), 3.07 – 3.00 (m, 2H), 2.79 – 2.72 (m, 2H), 2.54 – 2.44 (m, 4H), 1.62 (p, *J* = 5.6 Hz, 4H), 1.46 (q, *J* = 6.0 Hz, 2H); **^{13}C NMR** (126 MHz, $CDCl_3$) δ 167.6, 157.0, 134.0, 129.9, 129.0, 126.7, 113.6, 58.8, 54.6, 29.5, 26.2, 24.6; **IR** (film, cm^{-1}): 2933, 2851, 2799, 2763, 1517, 1459, 1438, 1307, 1155, 1110, 1003, 763; **HRMS:** m/z calculated for $C_{16}H_{21}N_2S$ $[M + H]^+$ 273.1420; found 273.1422.

2-(2,4-dimethoxyphenyl)-*N*-methyl-*N*-(3-(trifluoromethyl)benzyl)ethan-1-amine (**2as**)

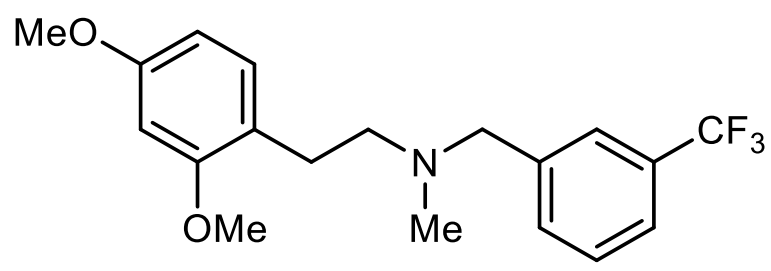

Prepared by general procedure **2.5.3.2.** using (((2,4-dimethoxybenzyl)(methyl)(3-(trifluoromethyl)benzyl)ammonio)methyl)trifluoroborate (**4as**) (42.1 mg, 0.10 mmol, 1.0 equiv), Pd-PEPPSI^{TM}-IPent (7.9 mg, 0.01 mmol, 10 mol%), isopropanol (1.2 mg, 0.02 mmol, 20 mol%), and potassium *tert*-butoxide (121 mg, 1.0 mmol, 10 equiv) in 4:1 1,4-dioxane/water (0.50 mL). Purification by automated flash column chromatography (0–10% acetone in petroleum ether for 15

minutes, 10–30% acetone in petroleum ether for 3 minutes, 50% acetone in petroleum ether for 2 minutes) gave the title compound as a colourless oil (22.9 mg, 0.065 mmol, 65% yield). **^{1}H NMR** (700 MHz, $CDCl_3$) δ 7.57 (s, 1H), 7.51 – 7.45 (m, 2H), 7.40 (t, *J* = 7.7 Hz, 1H), 7.02 (d, *J* = 8.0 Hz, 1H), 6.44 – 6.39 (m, 2H), 3.79 (s, 3H), 3.74 (s, 3H), 3.60 (s, 2H), 2.79 – 2.74 (m, 2H), 2.60 – 2.55 (m, 2H), 2.29 (s, 3H); **^{13}C NMR** (176 MHz, $CDCl_3$) δ 159.5, 158.5, 140.8, 132.3, 130.6 (q, *J* = 32.0 Hz), 130.5, 128.7, 125.7 (q, *J* = 3.8 Hz), 124.4 (q, *J* = 272.1 Hz), 123.8 (q, *J* = 3.9 Hz), 121.2, 104.0, 98.6, 61.6, 57.7, 55.5, 55.3, 42.4, 27.7; **^{19}F NMR** (376 MHz, $CDCl_3$) δ –62.4; **IR** (film, cm^{-1}): 2932, 1508, 1457, 1329, 1210, 1157, 1124, 1040, 703; **HRMS:** m/z calculated for $C_{19}H_{23}F_3NO_2$ $[M+H]^+$ 354.1675; found 354.1677.

N-(3,5-difluorobenzyl)-2-(2,4-dimethoxyphenyl)-*N*-methylethan-1-amine (**2at**)

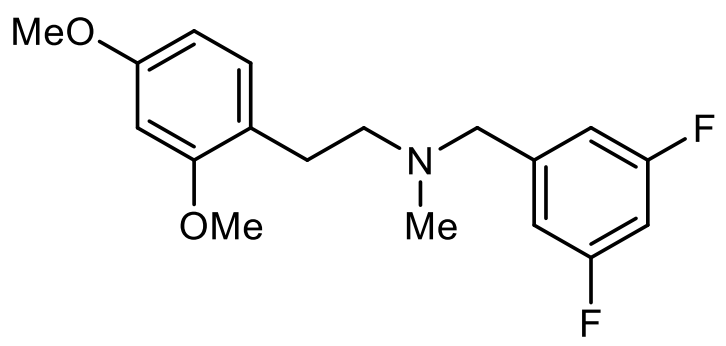

Prepared by general procedure **2.5.3.2.** using (((3,5-difluorobenzyl)(2,4-dimethoxybenzyl)(methyl)ammonio)methyl)trifluoroborate (**4at**) (38.9 mg, 0.10 mmol, 1.0 equiv), Pd-PEPPSI^{TM}-IPent (7.9 mg, 0.01 mmol, 10 mol%), isopropanol (1.2 mg, 0.02 mmol, 20 mol%), and potassium *tert*-butoxide (121 mg, 1.0 mmol, 10 equiv) in 4:1 1,4-dioxane/water (0.50 mL). Purification by automated flash column chromatography (0–10% acetone in petroleum ether for 15 minutes, 10–30% acetone in petroleum ether for 3 minutes, 50% acetone in petroleum ether for 2 minutes) gave the title compound as a colourless oil (22.4 mg, 0.070 mmol, 70% yield). The structure was confirmed by 2D NMR spectroscopy. **^{1}H NMR** (400 MHz, $CDCl_3$) δ 7.05 – 6.99 (m, 1H), 6.88 – 6.77 (m, 2H), 6.66 (tt, *J* = 8.9, 2.4 Hz, 1H), 6.46 – 6.38 (m, 2H), 3.79 (s, 3H), 3.75 (s, 3H), 3.52 (s, 2H), 2.75 (dd, *J* = 9.2, 6.1 Hz, 2H), 2.59 – 2.51 (m, 2H), 2.29 (s, 3H); **^{13}C NMR** (176 MHz, $CDCl_3$) δ 163.1 (dd, *J* = 247.5, 12.7 Hz), 159.5, 158.5, 144.1 (t, *J* = 8.6 Hz), 130.6, 121.1, 111.4 (dd, *J* = 20.4, 4.3 Hz), 104.0, 102.2 (t, *J* = 25.5 Hz), 98.6, 61.3, 57.5, 55.5, 55.3, 42.4, 27.8; **^{19}F NMR** (376 MHz, $CDCl_3$) δ –110.9; **IR** (film, cm^{-1}): 2941, 1593, 1508, 1459, 1290, 1210, 1157, 1115, 1040, 848; **HRMS:** m/z calculated for $C_{18}H_{22}F_2NO_2$ $[M+H]^+$ 322.1619; found 322.1616.

2-(2,4-dimethoxyphenyl)-*N*-methyl-*N*-((6-(trifluoromethyl)pyridin-3-yl)methyl)ethan-1-amine (**2au**)

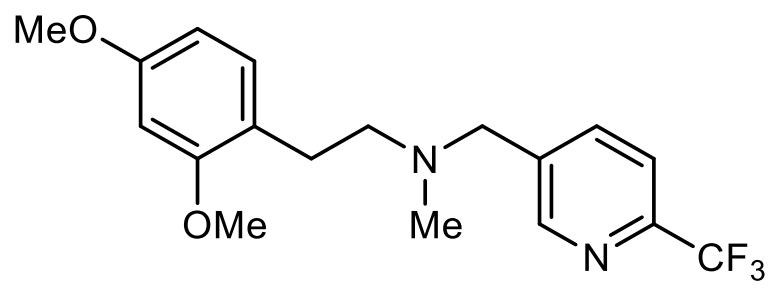

Prepared by general procedure **2.5.3.2.** using (((2,4-dimethoxybenzyl)(methyl)((6-(trifluoromethyl)pyridin-3-yl)methyl)ammonio)methyl)trifluoroborate (**4au**) (42.2 mg, 0.10 mmol, 1.0 equiv), Pd-PEPPSI^{TM}-IPent (7.9 mg, 0.01 mmol, 10 mol%), isopropanol (1.2 mg, 0.02 mmol, 20 mol%), and potassium *tert*-butoxide (121 mg, 1.0 mmol, 10 equiv) in 4:1 1,4-dioxane/water (0.50 mL). Purification by automated flash column chromatography (0–10% acetone in petroleum ether for 15 minutes, 10–30% acetone in petroleum ether for 3 minutes, 50% acetone in petroleum ether for 2 minutes) gave the title compound as a colourless oil (25.4 mg, 0.072 mmol, 72% yield). **^{1}H NMR** (500 MHz, $CDCl_3$) δ 8.61 (s, 1H), 7.77 (d, *J* = 8.0, 1H), 7.59 (d, *J* = 8.0 Hz, 1H), 7.03 – 6.98 (m, 1H), 6.43 – 6.39 (m, 2H), 3.79 (s, 3H), 3.72 (s, 3H), 3.62 (s, 2H), 2.75 (dd, *J* = 9.0, 6.2 Hz, 2H), 2.60 – 2.53 (m, 2H), 2.31 (s, 3H); **^{13}C NMR** (176 MHz, $CDCl_3$) δ 159.5, 158.5, 150.5, 146.9 (q, *J* = 34.5 Hz), 138.7, 137.8, 130.6, 121.9 (q, *J* = 274.0 Hz), 120.9, 120.1 (q, *J* = 2.8 Hz), 104.0, 98.6, 58.8, 57.4, 55.5, 55.3, 42.4, 27.8; **^{19}F NMR** (376 MHz, $CDCl_3$) δ –67.7; **IR** (film, cm^{-1}): 2943, 1616, 1508, 1333, 1292, 1210, 1174, 1137, 1087, 835; **HRMS:** m/z calculated for $C_{18}H_{22}F_3N_2O_2$ $[M+H]^+$ 355.1633; found 355.1626.

N-(3,5-bis(trifluoromethyl)benzyl)-2-(4-methoxyphenyl)-*N*-methylethan-1-amine (**2av**)

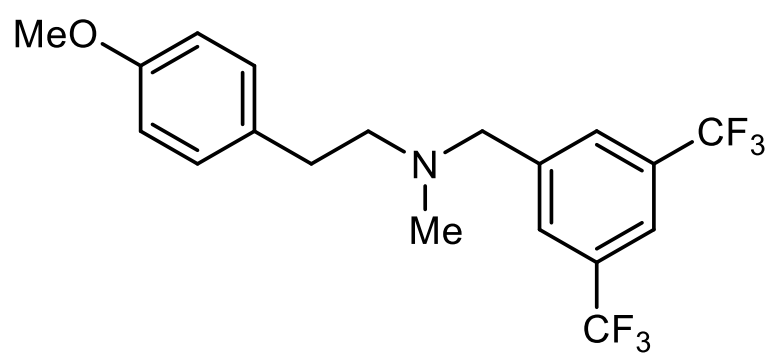

Prepared by general procedure **2.5.3.2.** using (((3,5-bis(trifluoromethyl)benzyl)(4-methoxybenzyl)(methyl)ammonio)methyl)trifluoroborate (**4av**) (45.9 mg, 0.10 mmol, 1.0 equiv), Pd-PEPPSI^{TM}-IPent (7.9 mg, 0.01 mmol, 10 mol%), isopropanol (1.2 mg, 0.02 mmol, 20 mol%), and potassium *tert*-butoxide (121 mg, 1.0 mmol, 10 equiv) in 4:1 1,4-dioxane/water (0.50 mL). Purification by automated flash column chromatography (0–10% acetone in petroleum ether for 15 minutes, 10–30% acetone in petroleum ether for 3 minutes, 50% acetone in petroleum ether for 2 minutes) gave the title compound as a colourless oil (32.5 mg, 0.083 mmol, 83% yield). **^{1}H NMR** (500 MHz, $CDCl_3$) δ 7.74 (s, 3H), 7.09 (d, *J* = 8.6 Hz, 2H), 6.83 (d, *J* = 8.6 Hz, 2H), 3.79 (s, 3H), 3.63 (s, 2H), 2.77 (dd, *J* = 8.9, 6.2 Hz, 2H), 2.64 (dd, *J* = 8.5, 6.3 Hz, 2H), 2.29 (s, 3H); **^{13}C NMR** (126

MHz, $CDCl_3$) δ 158.2, 142.6, 132.2, 131.6 (q, *J* = 33.1 Hz), 129.7, 128.8 (br. s), 121.1 (br s), 114.0, 61.4, 59.4, 55.4, 42.3, 33.1; **^{19}F NMR** (376 MHz, $CDCl_3$) δ –62.8; **IR** (film, cm^{-1}): 2950, 1515, 1357, 1277, 1131, 1040, 826, 684; **HRMS:** m/z calculated for $C_{19}H_{20}F_6NO$ $[M+H]^+$ 392.1449; found 392.1442.

5,6-dimethoxy-2-((1-phenethylpiperidin-4-yl)methyl)-2,3-dihydro-1*H*-inden-1-one (**5**)

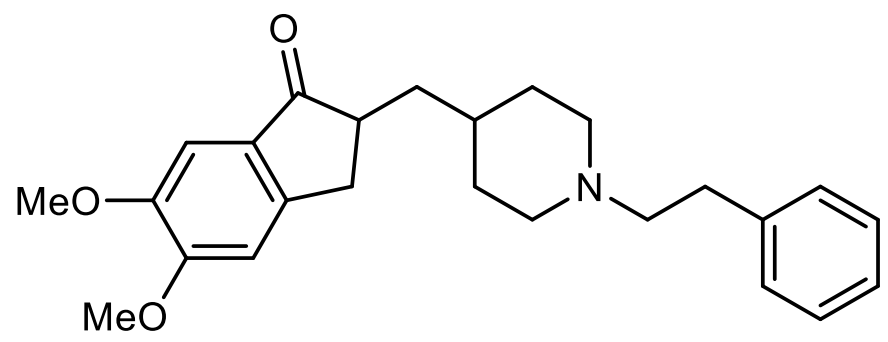

Prepared by general procedure **2.5.3.1.** using ((1-benzyl-4-((5,6-dimethoxy-1-oxo-2,3-dihydro-1*H*-inden-2-yl)methyl)piperidin-1-ium-1-yl)methyl)trifluoroborate (92 mg, 0.20 mmol, 1.0 equiv), bis(benzonitrile)palladium(II) chloride (3.8 mg, 0.01 mmol, 5 mol%), RuPhos (9.3 mg, 0.02 mmol, 10 mol%), and potassium *tert*-butoxide (112 mg, 1.0 mmol, 5.0 equiv) in 4:1 1,4-dioxane/water (1.0 mL). Purification by automated flash column chromatography (40–60% ethyl acetate in petroleum ether) gave the title compound as a white solid (49 mg, 0.12 mmol, 62% yield). **^{1}H NMR** (500 MHz, $CDCl_3$) δ 7.33 – 7.27 (m, 2H), 7.25 – 7.20 (m, 3H), 7.19 (s, 1H), 6.88 (s, 1H), 3.98 (s, 3H,), 3.92 (s, 3H), 3.27 (dd, *J* = 17.5, 8.1 Hz, 1H), 3.06 (ddd, *J* = 11.6, 7.1, 4.7 Hz, 2H), 2.88 – 2.82 (m, 2H), 2.79 – 2.70 (m, 2H), 2.68 – 2.56 (m, 2H), 2.05 (tdd, *J* = 11.6, 7.3, 2.7 Hz, 2H), 1.95 (ddd, *J* = 13.6, 8.0, 4.3 Hz, 1H), 1.81 (dt, *J* = 13.2, 3.1 Hz, 1H), 1.75 (dt, *J* = 12.8, 3.1 Hz, 1H), 1.62 – 1.51 (m, 1H), 1.49 – 1.30 (m, 3H); **^{13}C NMR** (126 MHz, $CDCl_3$) δ 207.8, 155.5, 149.5, 148.8, 140.5, 129.3, 128.7, 128.4, 126.0, 107.4, 104.4, 61.0, 56.2, 56.1, 53.9, 53.9, 45.4, 38.8, 34.5, 33.8, 33.4, 33.0, 31.8; **IR** (film, cm^{-1}): 2915, 1618, 1590, 1498, 1467, 1452, 1421, 1313, 1261, 1221, 1190, 1130, 1105, 1032, 856, 768, 746, 726, 699; **HRMS:** m/z calculated for $C_{25}H_{32}NO_3$ $[M + H]^+$ 394.2377; found 394.2369.

1-(Bis(4-fluorophenyl)methyl)-4-(2,3,4-trimethoxyphenethyl)piperazine (**6**)

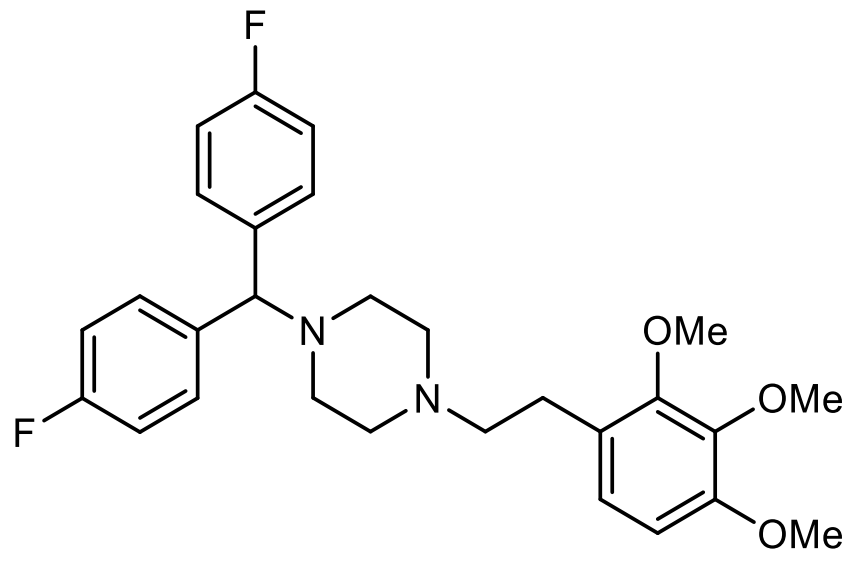

Prepared by general procedure **2.5.3.1.** ((1-benzyl-4-(bis(4-fluorophenyl)methyl)piperazin-1-ium-1-yl)methyl)trifluoroborate (28 mg, 0.05 mmol, 1.0 equiv), bis(benzonitrile)palladium(II) chloride (1.9 mg, 0.005 mmol, 10 mol%), RuPhos (4.7 mg, 0.01 mmol, 20 mol%), and potassium *tert*-butoxide (28 mg, 0.25 mmol, 5.0 equiv) in 4:1 1,4-dioxane/water (0.25 mL). Purification by reverse phase automated flash column chromatography (0–100% acetonitrile in water) gave the title compound as a white solid (23 mg, 0.048 mmol, 96% yield). **^{1}H NMR** (700 MHz, $CDCl_3$) δ 7.40 – 7.31 (m, 4H), 7.03 – 6.93 (m, 4H), 6.83 (d, *J* = 8.4 Hz, 1H), 6.59 (d, *J* = 8.5 Hz, 1H), 4.22 (s, 1H), 3.87 (s, 3H), 3.86 (s, 3H), 3.83 (s, 3H), 2.75 – 2.72 (m, 2H), 2.68 – 2.17 (m, 10H); **^{13}C NMR** (176 MHz, $CDCl_3$) δ 161.8 (d, *J* = 245.4 Hz), 152.2, 152.0, 142.3, 138.3 (d, *J* = 3.20), 129.3 (d, *J* = 7.8 Hz), 126.2, 124.1, 115.4 (d, *J* = 21.3 Hz), 107.3, 74.5, 61.1, 60.7, 59.8, 56.0, 53.4, 51.8, 27.4; **IR** (film, cm^{-1}): 2933, 2808, 1601, 1504, 1493, 1464, 1416, 1276, 1219, 1198, 1150, 1132, 1095, 1054, 1005, 869, 824, 786; **HRMS:** m/z calculated for $C_{28}H_{33}F_2N_2O_3$ $[M + H]^+$ 483.2459; found 483.2460.

3-methyl 5-(2-(methyl(phenethyl)amino)ethyl) 2,6-dimethyl-4-(3-nitrophenyl)-1,4-dihydropyridine-3,5-dicarboxylate (**7**)

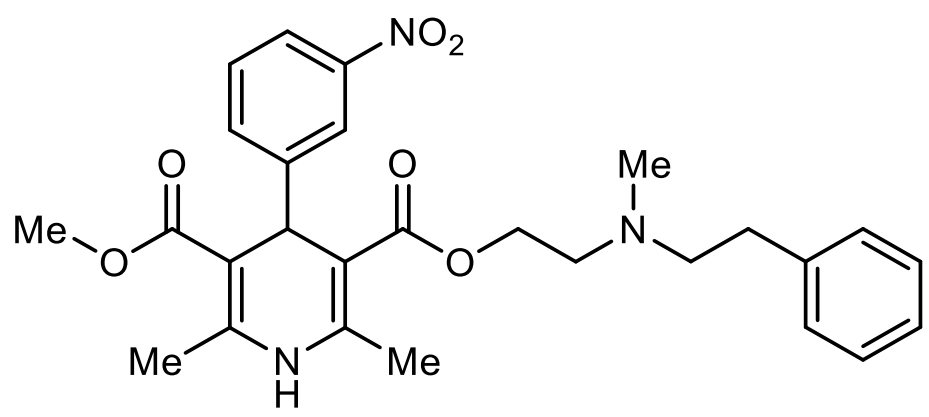

Prepared by general procedure **2.5.3.3.** using ((benzyl(2-((5-(methoxycarbonyl)-2,6-dimethyl-4-(3-nitrophenyl)-1,4-dihydropyridine-3-carbonyl)oxy)ethyl)(methyl)ammonio)methyl) trifluoroborate (56 mg, 0.1 mmol, 1.0 equiv, 90% purity), Pd-PEPPSI^{TM}-IPent (7.9 mg, 0.01 mmol, 10 mol%), and potassium phosphate tribasic (212 mg, 1.0 mmol, 10 equiv) in 4:1 1,4-dioxane/water (0.50 mL). Purification by reverse phase automated flash column chromatography (0–100% acetonitrile in water) gave the title compound as a pale yellow solid (23 mg, 0.05 mmol, 45% yield). **^{1}H NMR** (700 MHz,

CDCl3) δ 8.13 (app t, *J* = 2.1 Hz, 1H), 8.01 (ddd, *J* = 8.1, 2.1, 1.1 Hz, 1H), 7.67 (d, *J* = 7.9 Hz, 1H), 7.38 (app t, *J* = 7.9 Hz, 1H), 7.31 – 7.29 (m, 2H), 7.23 – 7.18 (m, 3H), 5.73 (s, 1H), 5.14 (s, 1H), 4.17 (td, *J* = 6.2, 1.9 Hz, 2H), 3.65 (s, 3H), 2.80 – 2.74 (m, 2H), 2.74 – 2.68 (m, 1H), 2.70 – 2.63 (m, 3H), 2.40 (s, 3H), 2.38 (s, 3H), 2.34 (s, 3H); **^{13}C NMR** (176 MHz, $CDCl_3$) δ 167.5, 167.0, 149.6, 148.3, 144.9, 144.8, 140.3, 134.4, 128.7, 128.7, 128.4, 126.0, 122.9, 121.4, 103.3, 103.2, 61.9, 59.8, 55.6, 51.2, 42.5, 39.7, 33.9, 19.8, 19.7; **IR** (film, cm^{-1}): 2923, 1681, 1524, 1489, 1378, 1345, 1298, 1274, 1206, 1119, 1096, 1016, 805, 781, 747, 696, 676; **HRMS:** m/z calculated for $C_{27}H_{31}BF_2N_3O_6$ [M + H]$^+$ 494.2291; found 494.2292.

ethyl 2-amino-6-phenethyl-4,5,6,7-tetrahydrothieno[2,3-c]pyridine-3-carboxylate (**8**)

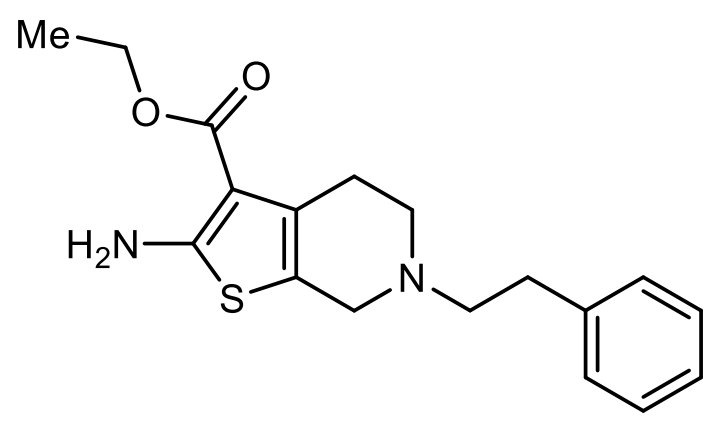

Prepared by general procedure **2.5.3.3.** using ((2-amino-6-benzyl-3-(ethoxycarbonyl)-4,5,6,7-tetrahydrothieno[2,3-c]pyridin-6-ium-6-yl)methyl)trifluoroborate (80 mg, 0.20 mmol, 1.0 equiv), Pd-PEPPSI^{TM}-IPent (7.9 mg, 0.01 mmol, 5 mol%), and potassium phosphate tribasic (424 mg, 2.0 mmol, 10 equiv) in 4:1 1,4-dioxane/water (1.0 mL). Purification by automated flash column chromatography (10–30% ethyl acetate in petroleum ether) gave the title compound as a brown oil (17 mg, 0.05 mmol, 25% yield). **^{1}H NMR** (500 MHz, $CDCl_3$) δ 7.31 – 7.27 (m, 2H), 7.24 – 7.19 (m, 3H), 5.95 (s, 2H), 4.26 (q, *J* = 7.1 Hz, 2Hz), 3.52 (t, *J* = 2.0 Hz, 2H), 2.91 – 2.83 (m, 3H), 2.82 – 2.74 (m, 4H), 1.33 (t, *J* = 7.1 Hz, 3H); **^{13}C NMR** (126 MHz, $CDCl_3$) δ 166.1, 162.2, 140.3, 131.3, 128.9, 128.6, 126.3, 114.9, 105.6, 59.7, 59.6, 51.6, 50.7, 34.4, 27.5, 14.6; **IR** (film, cm^{-1}): 3427, 3321, 2931, 1665, 1655, 1579, 1491, 1259, 1114, 1028, 734; **HRMS:** m/z calculated for $C_{18}H_{23}N_2O_2S$ [M + H]$^+$ 331.1475; found 331.1476.

2-(4-(2-(Benzo[*d*][1,3]dioxol-5-yl)ethyl)piperazin-1-yl)pyrimidine (**9a**)

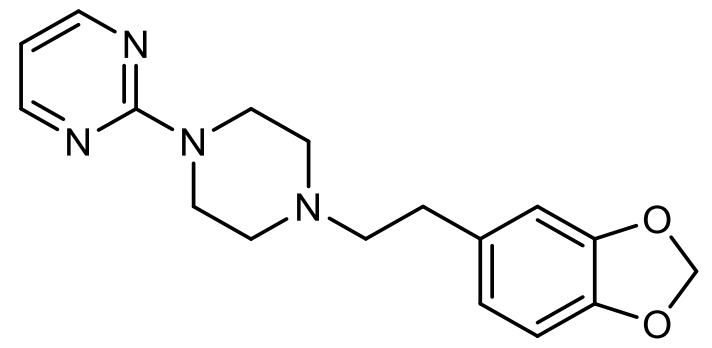

Prepared by general procedure **2.5.3.1.** ((1-benzyl-4-(pyrimidin-2-yl)piperazin-1-ium-1-yl)methyl)trifluoroborate (76 mg, 0.20 mmol, 1.0 equiv), bis(benzonitrile)palladium(II) chloride (3.8 mg, 0.01 mmol, 5 mol%), RuPhos (9.3 mg, 0.02 mmol, 10 mol%), and potassium *tert*-butoxide (112

mg, 1.0 mmol, 5.0 equiv) in 4:1 1,4-dioxane/water (1.0 mL). Purification by reverse phase automated flash column chromatography (0–100% acetonitrile in water) gave the title compound as a colourless oil (60 mg, 0.19 mmol, 95% yield). Data in line with previous characterisation.[90] **^{1}H NMR** (500 MHz, $CDCl_3$) δ 8.30 (d, *J* = 4.7 Hz, 2H), 6.76 – 6.70 (m, 2H), 6.66 (dd, *J* = 7.9, 1.7 Hz, 1H), 6.48 (t, *J* = 4.7 Hz, 1H), 5.91 (s, 2H), 3.92 – 3.79 (m, 4H), 2.81 – 2.72 (m, 2H), 2.63 – 2.52 (m, 6H); **^{13}C NMR** (126 MHz, $CDCl_3$) δ 161.7, 157.7, 147.6, 145.8, 134.0, 121.5, 109.9, 109.2, 108.2, 100.8, 60.8, 53.1, 43.7, 33.3.

2-(4-(2-(benzo[d][1,3]dioxol-5-yl)ethyl-1,1-d2)piperazin-1-yl)pyrimidine (**9b**)

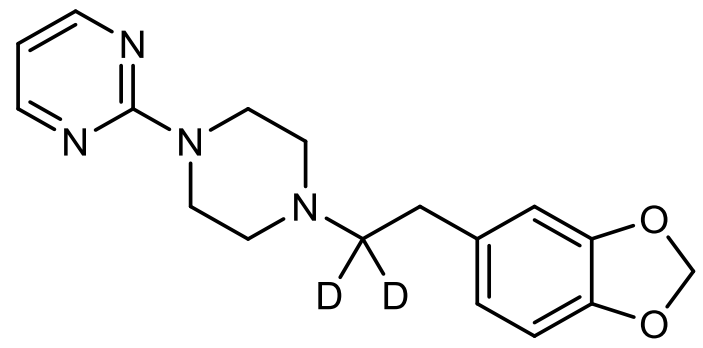

Prepared by general procedure **2.5.3.1.** ((1-(benzo[d][1,3]dioxol-5-ylmethyl)-4-(pyrimidin-2-yl)piperazin-1-ium-1-yl)methyl-d2)trifluoroborate (38 mg, 0.10 mmol, 1.0 equiv), bis(benzonitrile)palladium(II) chloride (3.8 mg, 0.01 mmol, 10 mol%), RuPhos (9.3 mg, 0.02 mmol, 20 mol%), and potassium *tert*-butoxide (56 mg, 0.50 mmol, 5.0 equiv) in 4:1 1,4-dioxane/water (0.50 mL). Purification by automated flash column chromatography (1–20% acetone in petroleum ether) gave the title compound as a colourless oil (21 mg, 0.07 mmol, 68% yield). **^{1}H NMR** ((700 MHz, $CDCl_3$) δ 8.30 (d, *J* = 4.7 Hz, 2H), 6.74 – 6.70 (m, 2H), 6.66 (ddd, *J* = 7.9, 1.7, 0.6 Hz, 1H), 6.48 (t, *J* = 4.7 Hz, 1H), 5.92 (s, 2H), 3.88 – 3.82 (m, 4H), 2.75 (s, 2H), 2.58 – 2.55 (m, 4H); **^{13}C NMR** (176 MHz, $CDCl_3$) δ 161.8, 157.9, 147.7, 145.9, 134.1, 121.6, 110.0, 109.3, 108.4, 100.9, 60.1 (p, *J* = 20.1 Hz), 53.2, 43.8, 33.2; **^{2}H NMR** (107 MHz, $CDCl_3$) δ 2.58 (s, 1H). **IR** (film, cm^{-1}): 2935, 2805, 1584, 1502, 1488, 1441, 1358, 1245, 1153, 1038, 983; **HRMS:** m/z calculated for $C_{17}H_{19}D_2N_4O_2$ $[M + H]^+$ 315.1785; found 315.1786.

1-(4-methoxybenzyl)-3-(1-oxo-5-(1-phenethylpiperidin-4-yl)isoindolin-2-yl)piperidine-2,6-dione (**10**)

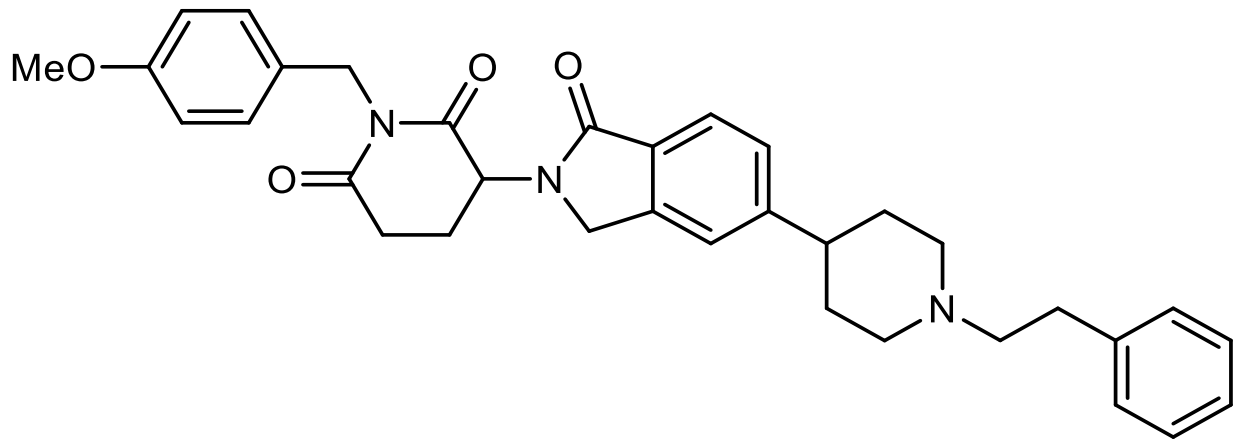

Prepared by general procedure **2.5.3.3.** using ((1-benzyl-4-(2-(1-(4-methoxybenzyl)-2,6-dioxopiperidin-3-yl)-1-oxoisoindolin-5-yl)-1l4-piperidin-2-ylium-1-yl)methyl)trifluoroborate (50 mg, 0.08 mmol, 1.0 equiv), Pd-PEPPSI^{TM}-IPent (6.3 mg, 0.008 mmol, 10 mol%), and potassium phosphate tribasic (90 mg, 0.80 mmol, 10 equiv) in 4:1 1,4-dioxane/water (0.40 mL). After 20 hours, the reaction mixture was diluted with ethyl acetate (5.0 mL) and H_2O (2.0 mL). Acetic acid was added dropwise until pH = 7 was reached. Phases were separated and the aqueous phase was washed with 5:1 chloroform/isopropanol (3 × 5 mL), the organic phases were then combined, dried over sodium sulphate and concentrated *in vacuo*. The residue was redissolved in dry chloroform (1.0 mL), transferred into a 4 mL PTFE/Silicone-lined septa screw cap clear glass vial equipped with a stir bar. Carbonyldiimidazole was added (16 mg, 0.10 mmol, 1.3 equiv) was added and the reaction mixture was heated at 65 °C for 18 hours. Following this, the reaction mixture was concentrated and the crude residue was purified by manual column chromatography on silica gel (20–40% acetone in dichloromethane) to give the title compound as a beige solid (24 mg, 0.0044 mmol, 54% yield over two steps). **^{1}H NMR** (700 MHz, $CDCl_3$) δ 7.81 (d, *J* = 7.9 Hz, 1H), 7.37 – 7.28 (m, 7H), 7.24 – 7.20 (m, 4H), 6.84 – 6.80 (m, 2H), 5.18 (dd, *J* = 13.6, 5.1 Hz, 1H), 4.90 (s, 2H), 4.40 (d, *J* = 15.7 Hz, 1H), 4.28 (d, *J* = 15.7 Hz, 1H), 3.78 (s, 3H), 3.17 (d, *J* = 11.4 Hz, 3H), 2.99 (ddd, *J* = 17.7, 4.5, 2.4 Hz, 1H), 2.89 – 2.82 (m, 4H), 2.69 – 2.60 (m, 3H), 2.28 (qd, *J* = 13.4, 4.5 Hz, 1H), 2.19 – 2.13 (m, 4H), 1.87 (q, *J* = 11.8 Hz, 4H). **^{13}C NMR** (176 MHz, $CDCl_3$) δ 171.1, 170.1, 169.5, 159.2, 151.3, 142.1, 140.6, 130.8, 129.9, 129.2, 128.9, 128.6, 127.6, 126.2, 124.3, 121.3, 114.0, 61.1, 55.4, 54.3, 52.6, 47.2, 43.3, 43.3, 33.9, 33.7, 32.4, 22.9; **IR** (film, cm^{-1}): 2952, 2926, 2851, 1638, 1515, 1456, 1368, 1345, 1249, 1164; **HRMS:** m/z calculated for $C_{34}H_{37}N_3O_4$ $[M + H]^+$ 552.2857; found 552.2838.

2-(2-bromoethyl)-4,4,5,5-tetramethyl-1,3,2-dioxaborolane

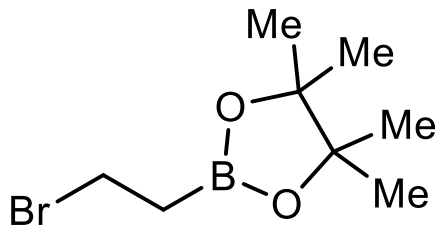

In a two-neck round bottom flask, equipped with an addition funnel, 1,2,3,4-tetrahydronaphthalene (4.1 mL, 30 mmol, 1.5 equiv) was stirred at room temperature. The second neck was connected to a Dreschel head directly bubbling into a three necked 500 mL round bottom flask containing 2-ethenyl-4,4,5,5-tetramethyl-1,3,2-dioxaborolane (3.4 mL, 20 mmol, 1.0 equiv) and dibenzoyl peroxide (48 mg, 0.20 mmol, 10 mol%) in heptane and maintained at 5 °C. Bromine (3.1 mL, 60 mmol, 3.0 equiv) was slowly added to tetrahydronaphthalene, ensuring a continuous bubbling of HBr for 3 h. After consumption of the starting material, the solution was sparged with nitrogen for 20 minutes. The reaction was then quenched with aqueous sodium bicarbonate (50 mL) and quickly transferred into a separating funnel, without shaking.[a] Phases were separated and the organic was washed with aqueous sodium thiosulphate (50 mL), dried over magnesium sulphate, filtered and concentrated to give a dark green liquid. To this residue, diethyl ether (20 mL) was added followed by a spatula of activated charcoal. The suspension was filtered over celite and the filtrate was concentrated *in vacuo* to give the title compound as a colourless liquid (3.2 g, 14 mmol, 70% yield). The product appeared to be unstable under standard storage condition and was used directly, without further purification. **^{1}H NMR** (δ 400 MHz, $CDCl_3$) δ 3.55 (t, *J* = 7.9 Hz, 2H), 1.58 (t, *J* = 7.8 Hz, 2H), 1.25 (s, 12H). Instability of the product precluded the collection of further characterisation data. The corresponding BMIDA was prepared following the same procedure, using vinyl BMIDA in dichloromethane. The product was obtained as a white solid (100mg, 0.38 mmol, 19% yield) and was directly used in the next step. Any purification attempt led to decomposition of the desired product. **^{1}H NMR** (500 MHz, CD_3CN) δ 3.96 (d, *J* = 17.0 Hz, 2H), 3.81 (d, *J* = 17.0 Hz, 2H), 3.63 – 3.56 (m, 2H), 2.87 (s, 3H), 1.43 – 1.36 (m, 2H).

Note:[a] Extended stirring of the quenching solution and the reaction mixture led to decomposition of the desired product.

3-(4,4,5,5-tetramethyl-1,3,2-dioxaborolan-2-yl)propyl 4-methylbenzenesulfonate

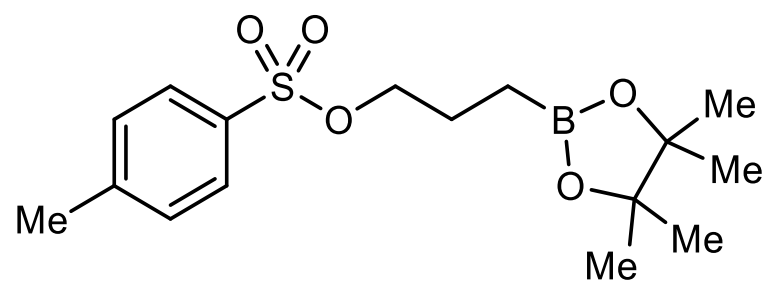

Prepared by general procedure **2.5.4.** using 2-(3-bromopropyl)-4,4,5,5-tetramethyl-1,3,2-dioxaborolane (2.0 g, 8.0 mmol, 1.0 equiv) and silver *p*-toluenesulfonate (4.5 g, 16 mmol, 2.0 equiv) in acetonitrile (24 mL). Purification by automated flash column chromatography (100% petroleum ether for 1 minute, 0–20% ethyl acetate in petroleum ether for 4 minutes, 20–50% ethyl acetate in petroleum ether for 4 minutes, 50–100% ethyl acetate in petroleum ether for 1 minute) gave the title compound as a clear liquid (2.1 g, 6.3 mmol, 78% yield). **^{1}H NMR** (700 MHz, $CDCl_3$) δ 7.80 – 7.75 (m, 2H), 7.34 – 7.30 (m, 2H), 3.99 (t, *J* = 6.6 Hz, 2H), 2.43 (s, 3H), 1.74 (dq, *J* = 7.8, 6.7 Hz, 2H), 1.19 (s, 12H), 0.75 (t, *J* = 7.8 Hz, 2H); **^{13}C NMR** (176 MHz, $CDCl_3$) δ 144.6, 133.4, 129.9, 128.0, 83.3, 72.3, 24.9, 23.6, 21.7; **IR** (thin film, cm^{-1}) 2978, 1359, 1176, 1143, 964, 912, 814, 663; **HRMS:** m/z calculated for $C_{16}H_{26}BO_5S$ $[M+H]^+$ 340.1630; found 340.1617.

4-(4,4,5,5-tetramethyl-1,3,2-dioxaborolan-2-yl)butyl 4-methylbenzenesulfonate

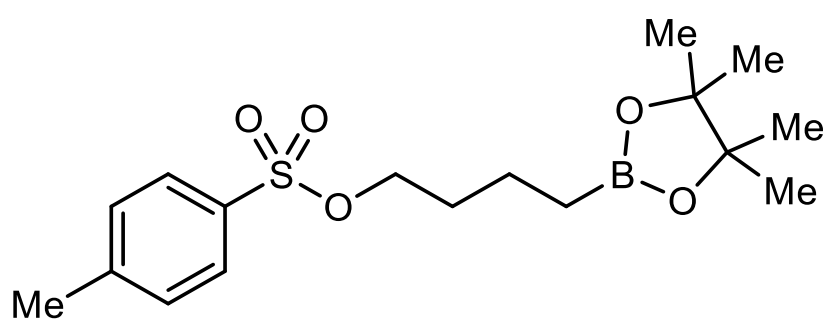

Prepared by general procedure **2.5.4.** using 2-(4-bromobutyl)-4,4,5,5-tetramethyl-1,3,2-dioxaborolane (2.1 g, 8.0 mmol, 1.0 equiv) and silver *p*-toluenesulfonate (4.5 g, 16 mmol, 2.0 equiv) in acetonitrile (24 mL). Purification by automated flash column chromatography (100% petroleum ether for 1 minute, 0–20% ethyl acetate in petroleum ether for 4 minutes, 20–50% ethyl acetate in petroleum ether for 4 minutes, 50–100% ethyl acetate in petroleum acetate for 1 minute) gave the title compound as a clear liquid (2.0 g, 5.6 mmol, 70% yield). **^{1}H NMR** (400 MHz, $CDCl_3$) δ 7.75 (dq, *J* = 8.5, 1.6 Hz, 2H), 7.34 – 7.27 (m, 2H), 3.98 (tq, *J* = 6.7, 1.4 Hz, 2H), 2.41 (s, 3H), 1.71 – 1.55 (m, 2H), 1.39 (p, *J* = 8.1 Hz, 2H), 1.19 (s, 12H), 0.68 (t, *J* = 7.9 Hz, 2H); **^{13}C NMR** (176 MHz, $CDCl_3$) δ 144.7, 133.3, 129.9, 128.0, 83.1, 70.6, 31.3, 24.9, 24.9, 21.7, 19.9; **IR** (neat, cm^{-1}) 2981, 1358, 1176, 1144, 925, 814, 663; **HRMS:** m/z calculated for $C_{17}H_{28}BO_5S$ $[M+H]^+$ 355.1745; found 355.1746.

5-(4,4,5,5-tetramethyl-1,3,2-dioxaborolan-2-yl)pentyl 4-methylbenzenesulfonate

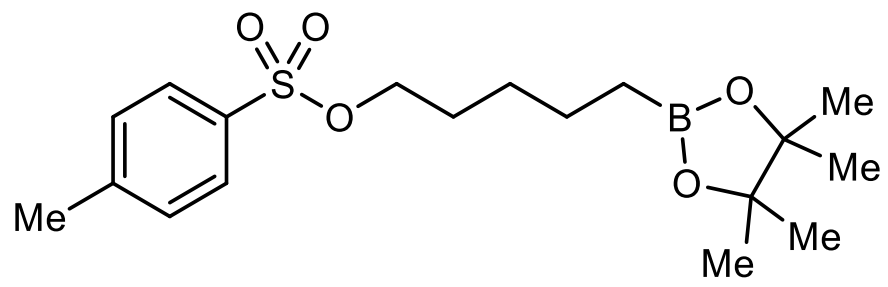

Prepared by general procedure **2.5.4.** using 2-(5-bromopentyl)-4,4,5,5-tetramethyl-1,3,2-dioxaborolane (2.2 g, 8.0 mmol, 1.0 equiv) and silver *p*-toluenesulfonate (4.5 g, 16 mmol, 2.0 equiv) in acetonitrile (24 mL). Purification by automated flash column chromatography (100% petroleum ether for 1 minute, 0–20% ethyl acetate in petroleum ether for 4 minutes, 20–50% ethyl acetate in petroleum ether for 4 minutes, 50–100% ethyl acetate in petroleum ether for 1 minute) gave the title compound as a clear liquid (2.7 g, 7.4 mmol, 93% yield). **^{1}H NMR** (700 MHz, $CDCl_3$) δ 7.79 – 7.75 (m, 2H), 7.35 – 7.31 (m, 2H), 4.00 (t, *J* = 6.6 Hz, 2H), 2.44 (s, 3H), 1.65 – 1.59 (m, 2H), 1.38 – 1.24 (m, 4H), 1.22 (s, 12H), 0.71 (t, *J* = 7.7 Hz, 2H); **^{13}C NMR** (176 MHz, $CDCl_3$) δ 144.7, 133.4, 129.9, 128.0, 83.1, 70.8, 28.7, 28.1, 24.9, 23.5, 21.7; **IR** (neat, cm^{-1}) 2980, 2933, 1359, 1176, 1144, 936, 814, 663; **HRMS:** m/z calculated for $C_{18}H_{29}BO_5S$ $[M+H]^+$ 369.1902; found 369.1905.

2-(allyloxy)ethyl 4-methylbenzenesulfonate (**15**)

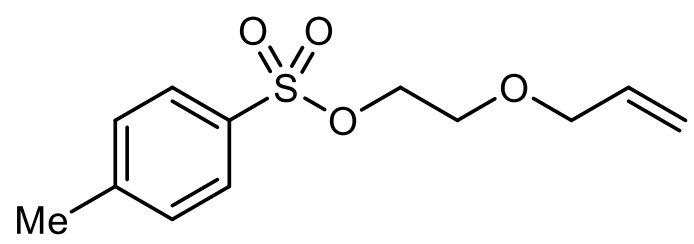

To a suspension of sodium hydride (60% in mineral oil, 1.2 g, 30 mmol, 1.5 equiv) in diethyl ether (45 mL) at 0 °C was added 2-allyloxy ethanol (2.1 mL, 20 mmol, 1.0 equiv) dropwise. After the gas evolution ceased, the reaction mixture was stirred at room temperature for 30 minutes and cooled down again to 0 °C. A solution of tosyl chloride (3.8 g, 20 mmol, 1.0 equiv) in diethyl ether (40 mL) was added dropwise over a period of 30 minutes. After addition, the reaction mixture was stirred at room temperature for 2 hours. Following this, the reaction mixture was quenched with saturated aqueous ammonium chloride solution (50 mL). The aqueous phase was extracted with diethyl ether (2 × 40 mL), the organic phases were combined, washed with brine (40 mL), dried over sodium sulphate, filtered and concentrated *in vacuo*. The title compound was obtained as a yellow liquid (3.7 g, 14 mmol, 72% yield) and was used without further purification. Data was in line with previous characterisation.[91] **^{1}H NMR** (500 MHz, $CDCl_3$) δ 7.82 – 7.76 (m, 2H), 7.37 – 7.30 (m, 2H), 5.82 (ddt, *J* = 17.3, 10.4, 5.6 Hz, 1H), 5.22 (dq, *J* = 17.2, 1.6 Hz, 1H), 5.16 (dq, *J* = 10.4, 1.4 Hz, 1H), 4.19 –

4.15 (m, 2H), 3.94 (dt, *J* = 5.6, 1.4 Hz, 2H), 3.66 – 3.59 (m, 2H), 2.44 (s, 3H); **^{13}C NMR** (126 MHz, $CDCl_3$) δ 144.9, 134.2, 133.2, 129.9, 128.1, 117.6, 72.3, 69.4, 67.6, 21.8.

2-(2-(but-3-en-1-yloxy)ethoxy)ethan-1-ol

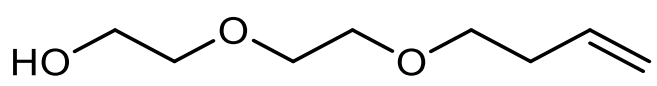

According to a procedure by Grubbs and co-workers,[92] diethylene glycol (5.2 mL, 40 mmol, 20 equiv) and 4-bromo-1-butene (0.20 mL, 2.0 mmol, 1.0 equiv) were added to an oven-dried 50 mL round-bottom flask. A solution of sodium hydroxide (400 mg, 10 mmol, 5.0 equiv) in water (2.0 mL) was slowly added over a period of 10 minutes. The resulting reaction mixture was stirred at 80 °C for 18 h. Following this, the reaction mixture was diluted with dichloromethane (10 mL), water (10 mL) and brine (10 mL). Phases were separated and the aqueous phase was extracted with dichloromethane (4 × 15 mL). Organic phases were combined, dried over magnesium sulphate, filtered and concentrated *in vacuo*. The crude liquid was purified by automated flash column chromatography (10–50% ethyl acetate in petroleum ether) to give the title compound as a colourless liquid (250 mg, 1.6 mmol, 80% yield). **^{1}H NMR** (500 MHz, $CDCl_3$) δ 5.81 (ddt, *J* = 17.1, 10.2, 6.7 Hz, 1H), 5.09 (dq, *J* = 17.1, 1.6 Hz, 1H), 5.04 (ddt, *J* = 10.2, 2.0, 1.2 Hz, 1H), 3.74 – 3.71 (m, 2H), 3.69 – 3.66 (m, 2H), 3.63 – 3.59 (m, 4H), 3.53 (t, *J* = 6.8 Hz, 2H), 2.48 (s, 1H), 2.36 (qt, *J* = 6.8, 1.4 Hz, 2H); **^{13}C NMR** (176 MHz, $CDCl_3$) δ 135.0, 116.7, 72.5, 70.8, 70.4, 70.3, 61.8, 34.1; **IR** (film, cm^{-1}): 3356, 2921, 2876, 1459, 1356, 1126, 1054, 896; **HRMS:** m/z calculated for $C_8H_{17}O_3$ $[M + H]^+$ 161.1172; found 161.1172.

2-(2-(but-3-en-1-yloxy)ethoxy)ethyl 4-methylbenzenesulfonate (**16**)

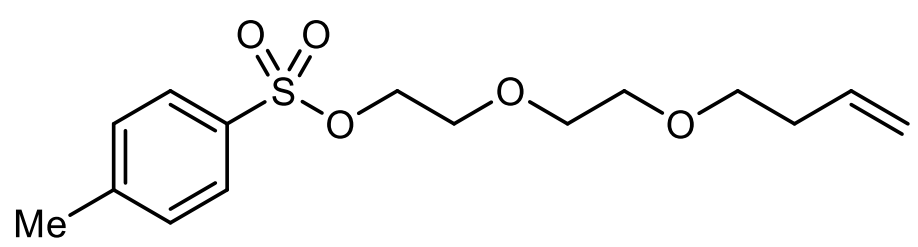

To a suspension of sodium hydride (60% in mineral oi, 141 mg, 3.5 mmol, 2.2 equiv) in diethyl ether (4.0 mL) at 0 °C was added 2-(2-(but-3-en-1-yloxy)ethoxy)ethan-1-ol (250 mg, 1.6 mmol, 1.0 equiv) in diethyl ether (2.0 mL) dropwise. After the gas evolution ceased, the reaction mixture was stirred at room temperature for 30 minutes and cooled down again to 0 °C. A solution of tosyl chloride (429 mg, 2.3 mmol, 1.5 equiv) in diethyl ether (4.0 mL) was added dropwise over a period of 30 minutes. After addition, the reaction mixture was stirred at room temperature for 3 hours. Following this, the reaction mixture was quenched with saturated aqueous ammonium chloride solution (10 mL). The aqueous

phase was extracted with diethyl ether (2 × 10 mL), the organic phases were combined, washed with brine (10 mL), dried over sodium sulphate, filtered and concentrated *in vacuo*. The liquid residue was purified by automated flash column chromatography (5–50% ethyl acetate in petroleum ether) to give the title compound as a colourless liquid (450 mg, 1.4 mmol, 92% yield). **^{1}H NMR** (400 MHz, $CDCl_3$) δ 7.79 (d, *J* = 8.1 Hz, 2H), 7.33 (d, *J* = 8.0 Hz, 2H), 5.79 (ddt, *J* = 17.1, 10.2, 6.8 Hz, 1H), 5.12 – 4.98 (m, 2H), 4.20 – 4.10 (m, 2H), 3.72 – 3.65 (m, 2H), 3.59 – 3.55 (m, 2H), 3.55 – 3.51 (m, 2H), 3.48 (t, *J* = 6.8 Hz, 2H), 2.44 (s, 3H), 2.32 (qt, *J* = 6.8, 1.4 Hz, 2H); **^{13}C NMR** (101 MHz, $CDCl_3$) δ 144.9, 135.2, 133.2, 129.9, 128.1, 116.5, 70.9, 70.8, 70.2, 69.4, 68.8, 34.2, 21.8; **IR** (film, cm^{-1}): 2876, 1727, 1599, 1355, 1176, 1096, 919, 817; **HRMS:** m/z calculated for $C_{15}H_{23}O_5S$ $[M + H]^+$ 315.1261; found 315.1262.

1-(4-allylpiperidin-1-yl)-2-hydroxyethan-1-one

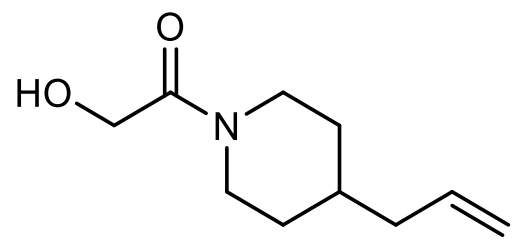

Glycolic acid (988 mg, 13 mmol, 1.4 equiv) and hexafluorophosphate benzotriazole tetramethyl uronium (HBTU) (4.9 g, 13 mmol, 1.4 equiv) were dissolved in *N,N*-dimethylformamide (28 mL) under an atmosphere of nitrogen and allowed to stir at room temperature. In a separate flask, 4-allylpiperidine hydrochloride (1.5 g, 9.3 mmol, 1.0 equiv) was stirred with *N,N*-diisopropylethylamine (DIPEA) (6.5 mL, 37 mmol, 4.0 equiv) at room temperature under an atmosphere of nitrogen. After 30 minutes, the mixture of 4-allylpiperidine hydrochloride and DIPEA was transferred to the solution of HBTU and glycolic acid and stirred at room temperature under nitrogen overnight (16 hours). The *N,N*-dimethylformamide was removed *in vacuo* and the crude mixture was dissolved in ethyl acetate (25 mL). The organic solution was then washed with water (2 × 25 mL) followed by brine (25 mL). The organic layer was dried over anhydrous sodium sulphate, filtered, and the solvent removed *in vacuo*. Purification by automated flash column chromatography (0–10% methanol in dichloromethane for 10 minutes, 10–20% methanol in dichloromethane for 4 minutes, 20% methanol in dichloromethane for 1 minute) gave the title compound as a colourless oil (819 mg, 4.5 mmol, 48% yield). **^{1}H NMR** (700 MHz, $CDCl_3$) δ 5.72 (ddt, *J* = 16.0, 11.0, 7.2 Hz, 1H), 5.02 – 4.96 (m, 2H), 4.52 (ddt, *J* = 13.3, 4.6, 2.3 Hz, 1H), 4.14 – 4.06 (m, 2H), 3.68 (t, *J* = 4.3 Hz, 1H), 3.44 (ddt, *J* = 13.5, 4.5, 2.3 Hz, 1H), 2.94 – 2.87 (m, 1H), 2.64 (td, *J* = 13.0, 2.9 Hz, 1H), 1.99 (tdt, *J* = 6.8, 4.2, 1.3 Hz, 2H), 1.73 (dddd, *J* = 13.4, 6.6, 3.1, 1.5 Hz, 2H), 1.56 (dddd, *J* = 14.7, 11.4, 7.1, 3.3 Hz, 1H), 1.15 – 1.04

(m, 2H); **^{13}C NMR** (176 MHz, $CDCl_3$) δ 169.7, 135.9, 116.7, 59.7, 40.6, 35.9, 32.0, 31.4; **IR** (thin film, cm^{-1}) 3414, 2915, 2857, 1638, 1447, 1397, 1377, 1275, 1083, 976, 912; **HRMS:** m/z calculated for $C_{10}H_{18}NO_2$ $[M+H]^+$ 184.1332; found 184.1336.

2-(4-allylpiperidin-1-yl)-2-oxoethyl 4-methylbenzenesulfonate (**17**)

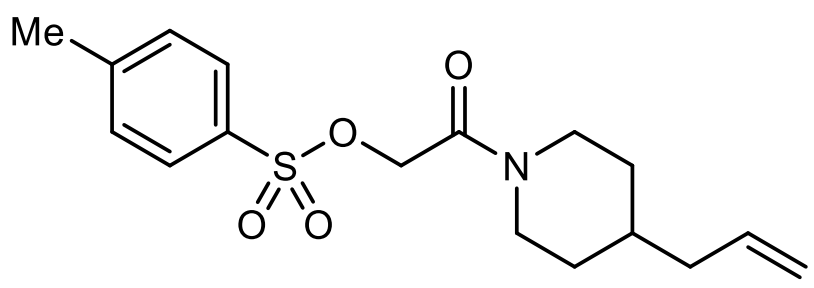

1-(4-allylpiperidin-1-yl)-2-hydroxyethan-1-one (819 mg, 4.5 mmol, 1.0 equiv), *p*-toluenesulfonic anhydride (1.7 g, 5.4 mmol, 1.2 equiv), 4-dimethylaminopyridine (164 mg, 1.3 mmol, 0.30 equiv), and pyridine (1.2 mL, 15.2 mmol, 3.4 equiv) were dissolved in dichloromethane (18 mL) under an atmosphere of nitrogen. The mixture was stirred at room temperature for 18 hours. Aqueous hydrogen chloride (1 M, 15 mL) was added and the mixture was stirred for an additional hour. The layers were separated and the organic layer was washed with brine (15 mL), collected, dried over anhydrous sodium sulphate, filtered and the solvent removed *in vacuo*. Purification by automated flash column chromatography (0–20% ethyl acetate in petroleum ether for 10 minutes, 20–50% ethyl acetate in petroleum ether for 6 minutes, 50–100% ethyl acetate in petroleum ether for 2 minutes, 100% ethyl acetate for 2 minutes) gave the title compound as a colourless oil (257 mg, 0.76 mmol, 17% yield). **^{1}H NMR** (700 MHz, $CDCl_3$) δ 7.79 (d, *J* = 8.1 Hz, 2H), 7.32 (d, *J* = 8.1 Hz, 2H), 5.70 (ddt, *J* = 14.7, 10.7, 7.2 Hz, 1H), 5.01 – 4.94 (m, 2H), 4.67 – 4.56 (m, 2H), 4.39 (ddt, *J* = 13.3, 4.7, 2.4 Hz, 1H), 3.63 (ddt, *J* = 13.6, 4.8, 2.5 Hz, 1H), 2.94 (td, *J* = 13.0, 2.8 Hz, 1H), 2.49 (td, *J* = 12.9, 3.0 Hz, 1H), 2.41 (s, 3H), 1.95 (t, *J* = 7.0 Hz, 2H), 1.73 – 1.63 (m, 2H), 1.51 (ttt, *J* = 10.8, 7.1, 3.7 Hz, 1H), 1.03 (dqd, *J* = 51.8, 12.5, 4.4 Hz, 2H); **^{13}C NMR** (176 MHz, $CDCl_3$) δ 162.7, 145.3, 136.0, 132.4, 129.9, 128.2, 116.5, 66.9, 45.6, 42.5, 40.5, 35.7, 32.1, 31.3, 21.7; **IR** (thin film, cm^{-1}) 2927, 1641, 1374, 1177, 977. **HRMS:** m/z calculated for $C_{17}H_{24}NO_4S$ $[M+H]^+$ 338.1426; found 338.1414.

2-(4-(4,4,5,5-tetramethyl-1,3,2-dioxaborolan-2-yl)phenoxy)ethyl 4-methylbenzenesulfonate (**18**)

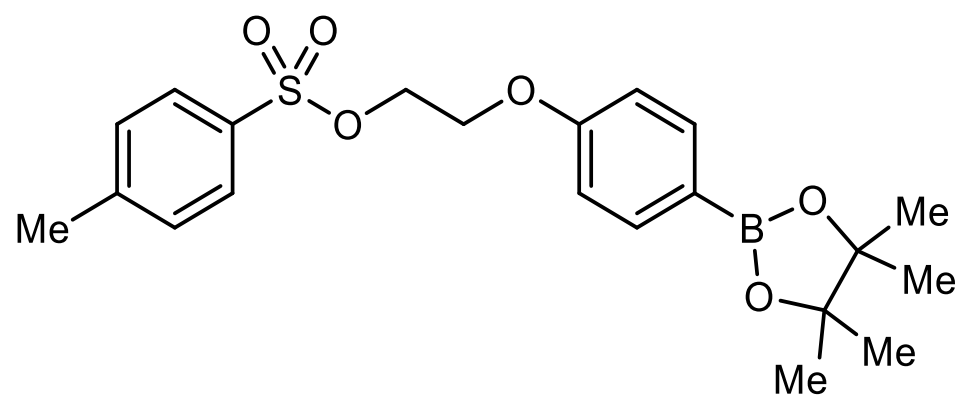

Prepared by general procedure **2.5.4.** using 4-(2-Bromoethoxy)benzene boronic acid pinacol ester (2.6 g, 8.0 mmol, 1.0 equiv) and silver *p*-toluenesulfonate (4.5 g, 16 mmol, 2.0 equiv) in acetonitrile (24 mL). Purification by manual column chromatography (silica gel, 5–20% ethyl acetate in petroleum ether) gave the title compound as a white solid (2.9 g, 6.9 mmol, 87% yield). **^{1}H NMR** (700 MHz, $CDCl_3$) δ 7.82 – 7.79 (m, 2H), 7.73 – 7.69 (m, 2H), 7.34 – 7.31 (m, 2H), 6.76 – 6.73 (m, 2H), 4.39 – 4.35 (m, 2H), 4.23 – 4.10 (m, 2H), 2.44 (s, 3H), 1.33 (s, 12H); **^{13}C NMR** (176 MHz, $CDCl_3$) δ 160.6, 145.1, 136.6, 133.0, 130.0, 128.1, 113.9, 83.8, 68.2, 65.2, 25.0, 21.8; **IR** (film, cm^{-1}): 2978, 1604, 1356, 1319, 1246, 1176, 1142, 1094, 928, 815; **HRMS:** m/z calculated for $C_{21}H_{28}BO_6S$ $[M + H]^+$ 418.1694; found 418.1694.

1-(4-(4-bromophenyl)piperazin-1-yl)-2-hydroxyethan-1-one

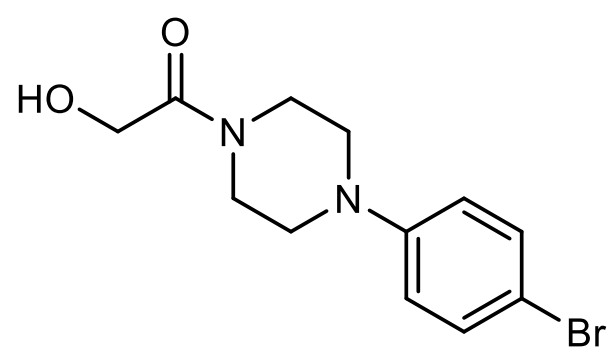

To an oven-dried 250 mL round-bottom flask, glycolic acid (1.7 g, 22 mmol, 1.4 equiv) was added to a solution of HBTU (8.5 g, 22 mmol, 1.4 equiv) in dimethylformamide (48 mL) at 0 °C. The resulting reaction mixture was stirred at room temperature for 30 minutes. Following this, 1-(4-bromophenyl)piperazine (3.9 g, 16 mmol, 1.0 equiv) was added, followed by *N*,*N*-diisopropylethylamine (8.4 mL, 48 mmol, 3.0 equiv) and the resulting reaction mixture was stirred for 18 h at room temperature. The reaction mixture was then concentrated to a minimum and then redissolved in ethyl acetate (150 mL). The solution was washed with water (2 × 100 mL), followed by brine (2 × 100 mL), dried over sodium sulphate, filtered and concentrated. The residue was purified by manual flash column chromatography (20–80% ethyl acetate in petroleum ether) to give the title compound as a beige solid (3.2 g, 11 mmol, 68% yield). **^{1}H NMR** (500 MHz, $CDCl_3$) δ 7.40 – 7.36 (m, 2H), 6.82 – 6.76 (m, 2H), 4.21 (s, 2H), 3.83 (d, *J* = 5.2 Hz, 2H), 3.49 – 3.39 (m, 2H), 3.19 – 3.12 (m, 4H). **^{13}C NMR** (126 MHz, $CDCl_3$) δ 170.3, 149.9, 132.3, 118.6, 113.4, 59.9, 49.5, 49.4, 43.4, 42.3.

IR (film, cm^{-1}): 3373, 2913, 2833, 1649, 1590, 1495, 1444, 1388, 1234, 1028, 818; **HRMS:** m/z calculated for $C_{12}H_{16}N_2O_2Br$ $[M + H]^+$ 299.0390; found 299.0406.

2-hydroxy-1-(4-(4-(4,4,5,5-tetramethyl-1,3,2-dioxaborolan-2-yl)phenyl)piperazin-1-yl)ethan-1-one

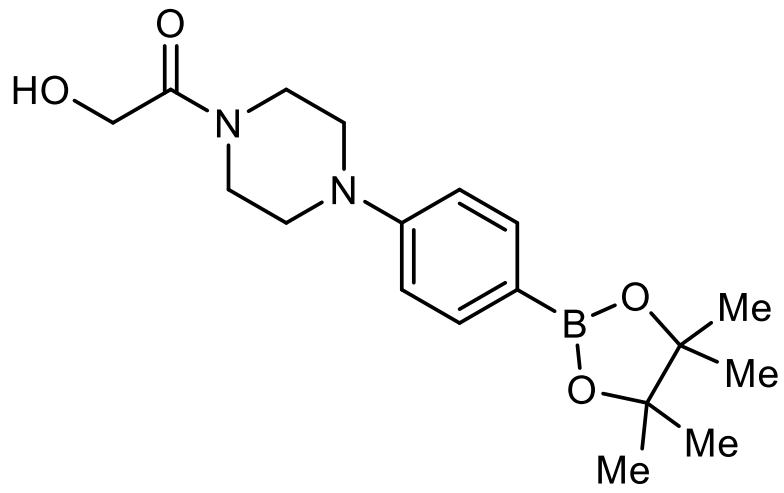

An oven-dried 250 mL three-neck round-bottom flask was charged with 1-(4-(4-bromophenyl)piperazin-1-yl)-2-hydroxyethan-1-one (3.0 g, 10 mmol, 1.0 equiv), potassium acetate (4.0 g, 41 mmol, 4.1 equiv), bis(pinacolato)diboron (5.2 g, 20 mmol, 2.0 equiv) and [1,1′-Bis(diphenylphosphino)ferrocene]dichloropalladium, complexed with dichloromethane (416 mg, 0.50 mmol, 5 mol%). The flask was equipped with a condenser, evacuated and backfilled with N_2 (three cycles). Anhydrous, degassed 1,4-dioxane (100 mL) was then added via syringe. The resulting reaction mixture was heated at 110 °C for 18 h. Following this, the reaction mixture was cooled down and concentrated *in vacuo.* The crude residue was then purified by automated flash column chromatography (10–70% ethyl acetate in petroleum ether) to give the title compound as a white solid (2.0 g, 5.8 mmol, 59% yield). **^{1}H NMR** (500 MHz, $CDCl_3$) δ 7.76 – 7.71 (m, 2H), 6.91 – 6.86 (m, 2H), 4.21 (d, J = 4.1 Hz, 2H), 3.82 (dd, J = 6.5, 4.1 Hz, 2H), 3.60 (t, J = 4.3 Hz, 1H), 3.45 – 3.40 (m, 2H), 3.28 (q, J = 4.7 Hz, 4H), 1.33 (s, 12H); **^{13}C NMR** (126 MHz, $CDCl_3$) δ 170.3, 152.9, 136.4, 115.2, 83.7, 59.9, 48.6, 48.5, 43.4, 42.2, 25.0; **IR** (film, cm^{-1}): 3317, 2979, 2926, 1647, 1604, 1447, 1361, 1231, 1142, 1092, 1028, 963, 861; **HRMS:** m/z calculated for $C_{18}H_{28}BN_2O_4$ $[M+H]^+$ 347.2137; found 347.2137.

2-oxo-2-(4-(4-(4,4,5,5-tetramethyl-1,3,2-dioxaborolan-2-yl)phenyl)piperazin-1-yl)ethyl 4-methylbenzenesulfonate (**19**)

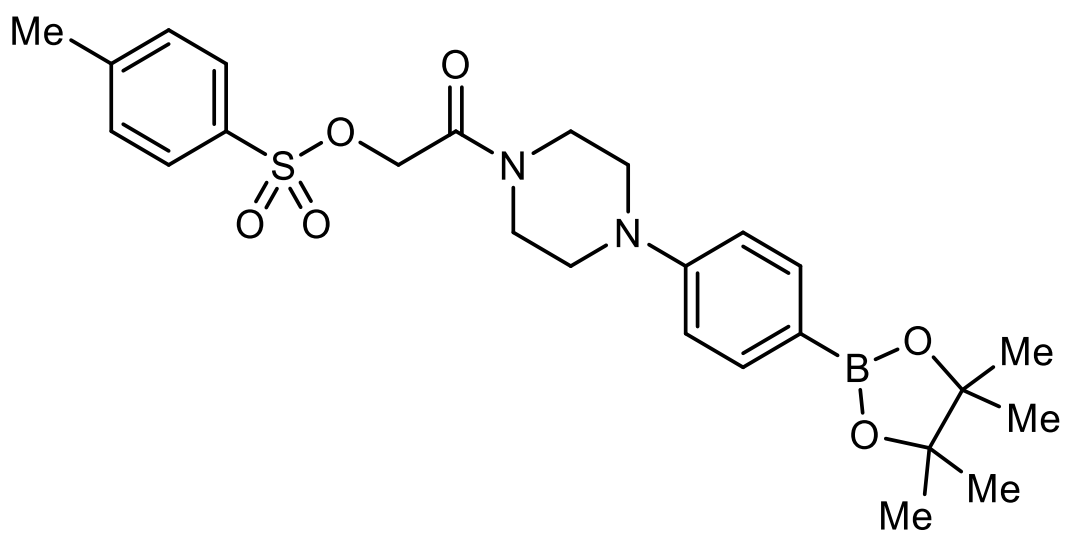

In an oven-dried 100 mL round bottom flask, 2-hydroxy-1-(4-(4-(4,4,5,5-tetramethyl-1,3,2-dioxaborolan-2-yl)phenyl)piperazin-1-yl)ethan-1-one (500 mg, 1.5 mmol, 1.0 equiv) was dissolved in toluene (10 mL) and cooled to 0 °C. Following this, triethylamine was added (0.44 mL, 3.2 mmol, 2.1 equiv) followed by a solution tosyl chloride (419 mg, 2.2 mmol, 1.5 equiv) in toluene (5.0 mL) over 5 minutes. The reaction mixture was brought back to room temperature and stirred over 18 h at this temperature. Following this, the reaction mixture was diluted with ethyl acetate (30 mL) and washed with water (2 × 50 mL), dried over sodium sulphate, filtered and concentrated *in vacuo*. The residue was purified by automated column chromatography (0–50% ethyl acetate in petroleum ether) to give the title compound as a white solid (460 mg, 0.92 mmol, 66% yield). **^{1}H NMR** (700 MHz, $CDCl_3$) δ 7.85 – 7.82 (m, 2H), 7.74 – 7.71 (m, 2H), 7.37 – 7.34 (m, 2H), 6.88 – 6.85 (m, 2H), 4.68 (s, 2H), 3.64 (dt, J = 65.7, 5.3 Hz, 4H), 3.23 (dt, J = 39.0, 5.2 Hz, 4H), 2.45 (s, 3H), 1.33 (s, 12H). **^{13}C NMR** (176 MHz, $CDCl_3$) δ 163.3, 152.8, 145.7, 136.4, 132.3, 130.2, 130.1, 128.4, 115.1, 83.7, 66.9, 48.6, 48.2, 45.3, 42.1, 25.0, 21.9.; **IR** (film, cm^{-1}): 2933, 1673, 1606, 1401, 1364, 1233, 1177, 1096, 1010, 812; **HRMS:** m/z calculated for $C_{25}H_{33}BN_2NaO_6S$ $[M+Na]^+$ 523.2047; found 523.2045.
Note: c.a 10% of alpha-chloroamide was also observed by ^{1}H crude NMR.

1-(benzo[*d*][1,3]dioxol-5-ylmethyl)-4-(pyrimidin-2-yl)-1-(3-(4,4,5,5-tetramethyl-1,3,2-dioxaborolan-2-yl)propyl)piperazin-1-ium 4-methylbenzenesulfonate (**12a**)

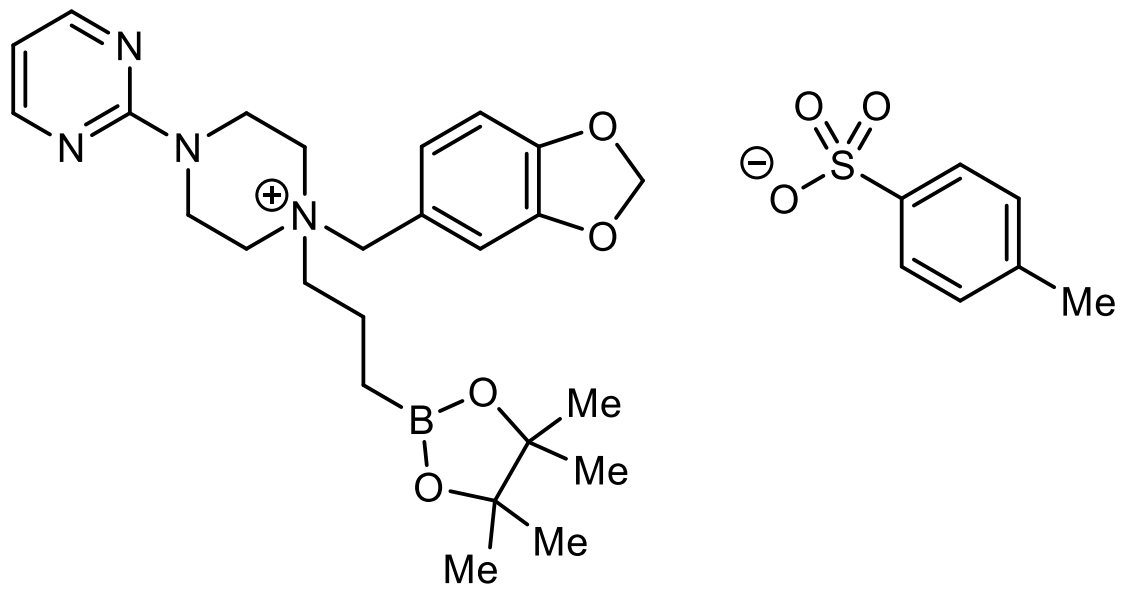

Prepared by general procedure **2.5.5.1.** using piribedil (597 mg, 2.0 mmol, 1.0 equiv) and 3-(4,4,5,5-tetramethyl-1,3,2-dioxaborolan-2-yl)propyl 4-methylbenzenesulfonate (**11a**) (1.0 g, 3.0 mmol, 1.5 equiv) in acetonitrile (2.0 mL). Upon completion, the reaction was purified by automated reverse phase flash column chromatography then reaction with pinacol (355 mg, 3.0 mmol, 1.5 equiv) in acetonitrile (2.0 mL) gave the title compound as an orange, viscous liquid (68% by mass in pinacol, 574 mg, 0.64 mmol, 32% yield). **^{1}H NMR** (700 MHz, CD_3CN) δ 8.39 (d, *J* = 4.7, 2H), 7.60 (d, *J* = 8.0, 2H), 7.12 (d, *J* = 8.0, 2H), 7.04 (d, *J* = 1.7 Hz, 1H), 7.01 (dd, *J* = 7.9, 1.6 Hz, 1H), 6.92 (dd, *J* = 8.0, 1.3 Hz, 1H), 6.71 (t, *J* = 4.7, 1H), 6.04 – 6.01 (m, 2H), 4.50 (br s, 2H), 4.43 (dt, *J* = 15.6, 4.0 Hz, 2H), 3.68 (dt, *J* = 14.7, 6.6 Hz, 2H), 3.46 – 3.41 (m, 4H), 3.33 – 3.28 (m, 2H), 2.31 (s, 3H), 1.87 (p, *J* = 7.5 Hz, 2H), 1.20 (s, 12H), 0.81 – 0.76 (m, 2H); **^{13}C NMR** (176 MHz, CD_3CN) δ 162.1, 159.0, 150.5, 149.3, 146.1, 139.8, 129.4, 128.6, 126.6, 121.0, 113.6, 112.6, 109.6, 103.1, 84.4, 65.4, 57.7, 57.3, 38.2, 25.2, 21.3, 17.1; **IR** (thin film, cm^{-1}) 2979, 1450, 1372, 1142, 1121, 1034, 1012, 733, 680; **HRMS:** m/z calculated for $C_{19}H_{26}BN_4O_4$ $[M{-}C_6H_{10}]^+$ 385.2042; found 385.2043.

1-(benzo[*d*][1,3]dioxol-5-ylmethyl)-4-(pyrimidin-2-yl)-1-(4-(4,4,5,5-tetramethyl-1,3,2-dioxaborolan-2-yl)butyl)piperazin-1-ium 4-methylbenzenesulfonate

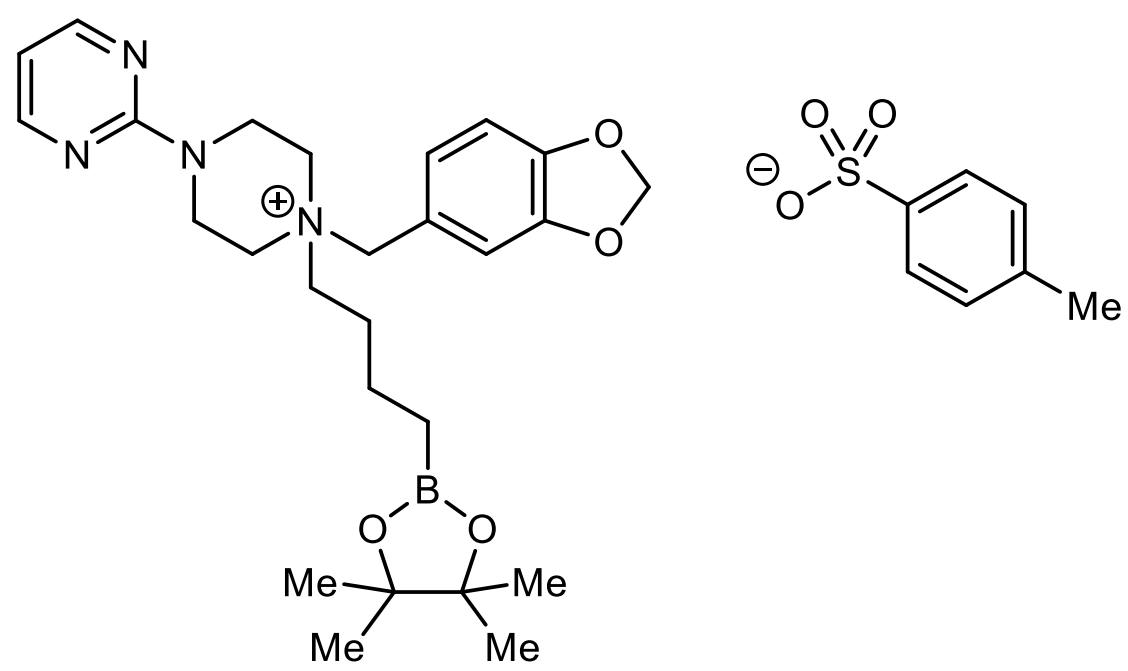

Prepared using general procedure **2.5.5.1.** using piribedil (1.2 g, 4.0 mmol, 1.0 equiv) and 4-(4,4,5,5-tetramethyl-1,3,2-dioxaborolan-2-yl)butyl 4-methylbenzenesulfonate (2.1 g, 6.0 mmol, 1.5 equiv) in acetonitrile (4.0 mL). Upon completion, the reaction was purified by automated reverse phase flash column chromatography then reaction with pinacol (709 mg, 6.0 mmol, 1.5 equiv) in acetonitrile (4.0 mL) to give the title compound as a yellow, viscous liquid (72% by mass in pinacol, 1.6 g, 1.7 mmol, 43% yield). **^{1}H NMR** (700 MHz, CD_3CN) δ 8.40 (d, *J* = 4.8 Hz, 2H), 7.60 – 7.58 (m, 2H), 7.13 – 7.11 (m, 2H), 7.05 (d, *J* = 1.8 Hz, 1H), 7.02 (dd, *J* = 7.9, 1.9 Hz, 1H), 6.93 (d, *J* = 8.0 Hz, 1H), 6.71 (t, *J* = 4.8 Hz, 1H), 6.04 (s, 2H), 4.49 (s, 2H), 4.42 (dt, *J* = 15.4, 4.1 Hz, 2H), 3.70 (ddd, *J* = 15.3, 9.6, 3.5 Hz,

2H), 3.47 – 3.38 (m, 4H), 3.28 – 3.22 (m, 2H), 2.31 (s, 3H), 1.86 – 1.79 (m, 2H), 1.39 (p, *J* = 7.7 Hz, 2H), 1.23 (s, 12H), 0.81 (t, *J* = 7.6 Hz, 2H); **^{13}C NMR** (176 MHz, CD_3CN) δ 162.1, 159.0, 150.6, 149.4, 146.2, 139.8, 129.4, 128.6, 126.6, 120.9, 113.5, 112.6, 109.6, 103.2, 84.1, 65.0, 57.6, 55.9, 38.2, 25.2, 24.5, 21.6, 21.3; **IR** (thin film, cm^{-1}) 2980, 1449, 1366, 1143, 1121, 1033, 1012, 680; **HRMS:** m/z calculated for $C_{20}H_{28}BN_4O_4$ $[M-C_6H_{10}]^+$ 399.2198; found 399.2189.

1-(benzo[*d*][1,3]dioxol-5-ylmethyl)-4-(pyrimidin-2-yl)-1-(5-(4,4,5,5-tetramethyl-1,3,2-dioxaborolan-2-yl)pentyl)piperazin-1-ium 4-methylbenzenesulfonate

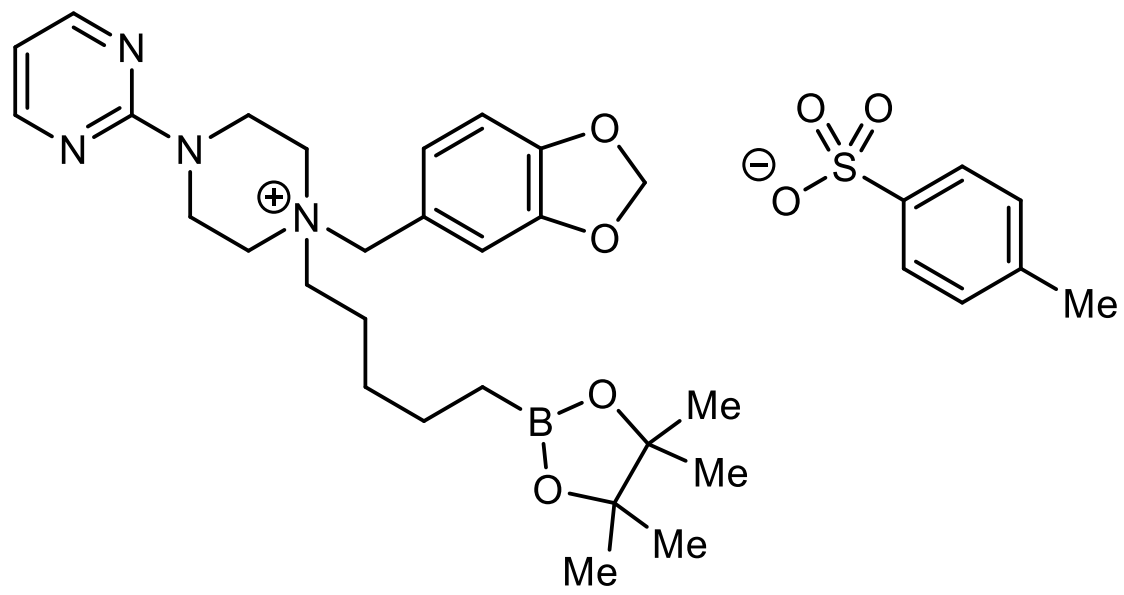

Prepared using general procedure **2.5.5.1.** using piribedil (1.2 g, 4.0 mmol, 1.0 equiv) and 5-(4,4,5,5-tetramethyl-1,3,2-dioxaborolan-2-yl)pentyl 4-methylbenzenesulfonate (2.2 g, 6.0 mmol, 1.5 equiv) in acetonitrile (4.0 mL). Upon completion, the reaction was purified by automated reverse phase flash column chromatography then reaction with pinacol (709 mg, 6.0 mmol, 1.5 equiv) in acetonitrile (4.0 mL) to give the title compound as a yellow, viscous liquid (65% by mass in pinacol, 2.1 g, 2.0 mmol, 50% yield). **^{1}H NMR** (700 MHz, CD_3CN) δ 8.39 (d, *J* = 4.8 Hz, 2H), 7.62 – 7.58 (m, 2H), 7.14 – 7.11 (m, 2H), 6.99 – 6.97 (m, 2H), 6.92 (d, *J* = 7.9 Hz, 1H), 6.71 (t, *J* = 4.7 Hz, 1H), 6.03 (s, 2H), 4.48 (s, 2H), 4.41 (dt, *J* = 15.5, 4.1 Hz, 2H), 3.70 (ddd, *J* = 15.3, 8.5, 4.7 Hz, 2H), 3.45 – 3.40 (m, 4H), 3.27 – 3.22 (m, 2H), 2.31 (s, 3H), 1.82 – 1.77 (m, 2H), 1.43 (p, *J* = 7.8 Hz, 2H), 1.33 – 1.28 (m, 2H), 1.20 (s, 12H), 0.74 (t, *J* = 7.9 Hz, 2H); **^{13}C NMR** (176 MHz, CD_3CN) δ 162.1, 159.0, 150.6, 149.3, 145.6, 140.1, 129.4, 128.6, 126.7, 120.9, 113.4, 112.6, 109.6, 103.1, 83.9, 65.1, 57.5, 56.4, 38.2, 29.4, 25.2, 24.4, 22.1, 21.3; **IR** (thin film, cm^{-1}) 2980, 1450, 1368, 1143, 1121, 1034, 1012, 949, 682; **HRMS:** m/z calculated for $C_{21}H_{30}BN_4O_4$ $[M-C_6H_{10}]^+$ 413.2355; found 413.2357.

1-allyl-1-(benzo[*d*][1,3]dioxol-5-ylmethyl)-4-(pyrimidin-2-yl)piperazin-1-ium 4-methylbenzenesulfonate

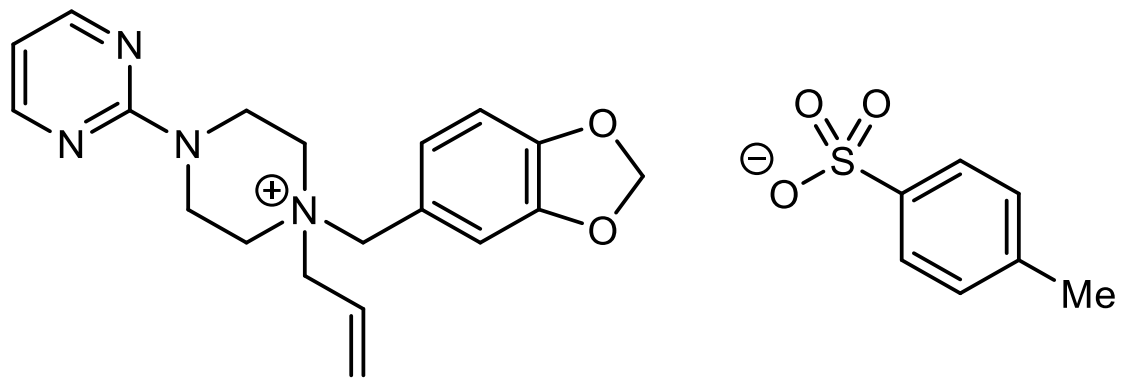

Prepared by general procedure **2.5.5.2.** using piribedil (298 mg, 1.0 mmol, 1.0 equiv) and allyl 4-methylbenzenesulfonate (318 mg, 1.5 mmol, 1.5 equiv) in acetonitrile (1.0 mL) at 60 ˚C over 72 h to give the title compound as a white solid (227 mg, 0.44 mmol, 44% yield). **^{1}H NMR** (700 MHz, CD_3CN) δ 8.37 (d, *J* = 4.7 Hz, 2H), 7.61 – 7.56 (m, 2H), 7.10 (d, *J* = 7.8 Hz, 2H), 7.05 (d, *J* = 1.8 Hz, 1H), 7.02 (dd, *J* = 8.0, 1.9 Hz, 1H), 6.90 (d, *J* = 8.0 Hz, 1H), 6.69 (t, *J* = 4.8 Hz, 1H), 6.11 (ddt, *J* = 17.3, 10.2, 7.2 Hz, 1H), 5.73 (dt, *J* = 16.8, 1.2 Hz, 1H), 5.69 (dd, *J* = 10.2, 1.2 Hz, 1H), 4.54 (s, 2H), 4.35 (dt, *J* = 15.5, 4.3 Hz, 2H), 4.05 (d, *J* = 7.2 Hz, 2H), 3.80 (ddd, *J* = 15.4, 9.2, 3.4 Hz, 2H), 3.47 – 3.36 (m, 4H), 2.30 (s, 3H); **^{13}C NMR** (176 MHz, CD_3CN) δ 162.0, 159.0, 150.6, 149.2, 146.1, 139.9, 129.5, 129.4, 128.9, 126.6, 125.4, 120.9, 118.4, 113.8, 112.6, 109.5, 103.1, 65.2, 59.7, 56.6, 38.1, 21.3; **IR** (film, cm^{-1}) 3004, 2896, 1450, 1193, 1034, 1012, 681; **HRMS:** m/z calculated for $C_{19}H_{23}N_4O_2$ $[M]^+$ 339.1816; found 339.1817.

1-(benzo[*d*][1,3]dioxol-5-ylmethyl)-1-(but-3-en-1-yl)-4-(pyrimidin-2-yl)piperazin-1-ium 4-methylbenzenesulfonate (**12a**)

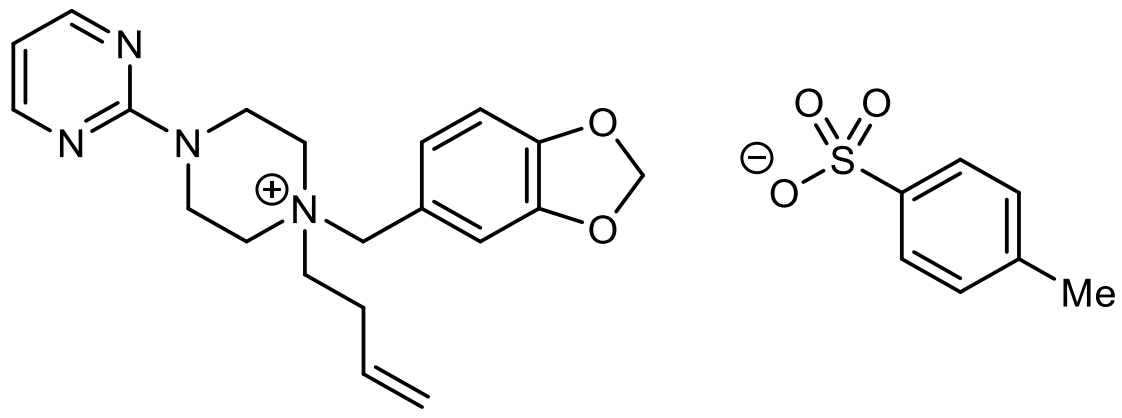

Prepared by general procedure **2.5.5.2.** using piribedil (1.2 g, 4.0 mmol, 1.0 equiv) and but-3-en-1-yl 4-methylbenzenesulfonate (**11a**) (1.4 g, 6.0 mmol, 1.5 equiv) in acetonitrile (4.0 mL) to give the title compound as a yellow, solid (1.2 g, 2.4 mmol, 59% yield). **^{1}H NMR** (500 MHz, DMSO-d6) δ 8.47 (d, *J* = 4.8 Hz, 2H), 7.50 – 7.44 (m, 2H), 7.13 – 6.99 (m, 5H), 6.79 (t, *J* = 4.8 Hz, 1H), 6.11 (s, 2H), 5.76 (ddt, *J* = 16.9, 10.2, 6.5 Hz, 1H), 5.28 (dq, *J* = 17.2, 1.5 Hz, 1H), 5.17 (dq, *J* = 10.2, 1.2 Hz, 1H),

4.63 (s, 2H), 4.43 – 4.36 (m, 2H), 3.80 (ddd, *J* = 14.9, 9.3, 3.1 Hz, 2H), 3.56 – 3.42 (m, 2H), 3.39 – 3.34 (m, 2H), 2.65 (q, *J* = 7.1 Hz, 2H), 2.28 (s, 3H). **^{13}C NMR** (126 MHz, DMSO-d6) δ 160.8, 158.1, 148.9, 147.7, 145.8, 137.5, 132.6, 128.0, 127.4, 125.5, 120.4, 118.5, 112.5, 111.5, 108.7, 101.8, 63.0, 56.3, 37.2, 25.4, 20.8; **IR** (thin film, cm^{-1}) 3010, 2916, 1450, 1187, 1033, 1012, 680. **HRMS**: m/z calculated for $C_{20}H_{25}N_4O_2$ [M]$^+$ 353.1972; found 353.1972.

1-(benzo[*d*][1,3]dioxol-5-ylmethyl)-1-(pent-4-en-1-yl)-4-(pyrimidin-2-yl)piperazin-1-ium 4-methylbenzenesulfonate (**12b**)

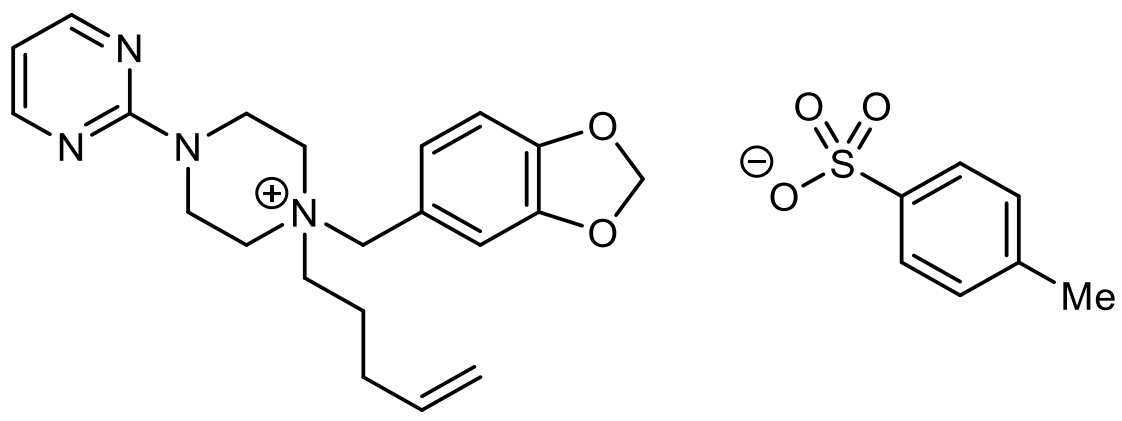

Prepared using general procedure **2.5.5.2.** using piribedil (597 mg, 2.0 mmol, 1.0 equiv) and pent-4-en-1-yl 4-methylbenzenesulfonate (**11b**) (721 mg, 3.0 mmol, 1.5 equiv) in acetonitrile (2.0 mL) to give the title compound as a pale yellow, solid (570 mg, 1.1 mmol, 53% yield). **^{1}H NMR** (500 MHz, DMSO-d6) δ 8.46 (d, *J* = 4.7 Hz, 2H), 7.50 – 7.44 (m, 2H), 7.10 (ddd, *J* = 7.9, 1.3, 0.7 Hz, 3H), 7.06 (d, *J* = 8.0 Hz, 1H), 7.02 (dd, *J* = 8.0, 1.7 Hz, 1H), 6.79 (t, *J* = 4.7 Hz, 1H), 6.11 (s, 2H), 5.86 (ddt, *J* = 16.8, 10.2, 6.5 Hz, 1H), 5.12 (dq, *J* = 17.1, 1.6 Hz, 1H), 5.05 (ddt, *J* = 10.2, 2.2, 1.2 Hz, 1H), 4.60 (s, 2H), 4.40 (d, *J* = 15.4 Hz, 2H), 3.76 (ddd, *J* = 15.1, 9.9, 3.3 Hz, 2H), 3.54 – 3.39 (m, 4H), 3.30 (d, *J* = 8.8 Hz, 1H), 2.28 (s, 3H), 2.08 (q, *J* = 7.1 Hz, 2H), 1.98 – 1.88 (m, 1H); **^{13}C NMR** (126 MHz, DMSO-d6) δ 160.8, 158.1, 148.9, 147.7, 145.8, 137.5, 136.9, 128.0, 127.4, 125.5, 120.4, 116.2, 112.6, 111.5, 108.7, 101.7, 63.2, 56.2, 37.2, 29.7, 20.8, 20.1; **IR** (thin film, cm^{-1}) 3008, 2922, 1446, 1187, 1033, 1012, 679; **HRMS**: m/z calculated for $C_{21}H_{27}N_4O_2$ [M]$^+$ 367.2129; found 367.2128.

1-(2-(allyloxy)ethyl)-1-(benzo[d][1,3]dioxol-5-ylmethyl)-4-(pyrimidin-2-yl)piperazin-1-ium 4-methylbenzenesulfonate

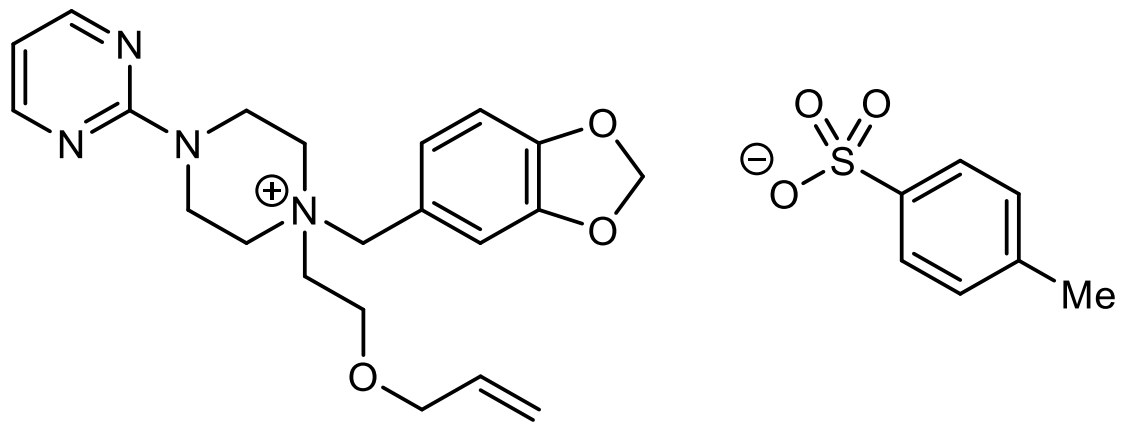

Prepared by general procedure **2.5.5.2.** using piribedil (895 mg, 3.0 mmol, 1.0 equiv), 2-(allyloxy)ethyl 4-methylbenzenesulfonate (**15**) (1.2 g, 4.5 mmol, 1.5 equiv) in acetonitrile (3.0 mL) at 90 °C over 72 h. Purification by reverse phase automated flash column chromatography (0–100% acetonitrile in water) gave the title compound as a light yellow solid (830 mg, 1.5 mmol, 50% yield). **^{1}H NMR** (700 MHz, DMSO-d6) δ 8.46 (d, *J* = 4.8 Hz, 2H), 7.50 – 7.46 (m, 2H), 7.15 (d, *J* = 1.7 Hz, 1H), 7.11 – 7.09 (m, 1H), 7.08 (dd, *J* = 8.0, 1.8 Hz, 1H), 7.05 (d, *J* = 8.0 Hz, 1H), 6.78 (t, *J* = 4.8 Hz, 1H), 6.10 (s, 2H), 5.96 (ddt, *J* = 17.2, 10.5, 5.3 Hz, 1H), 5.30 (dq, *J* = 17.3, 1.8 Hz, 1H), 5.21 (dq, *J* = 10.5, 1.5 Hz, 1H), 4.72 (s, 2H), 4.42 (dt, *J* = 15.3, 4.0 Hz, 2H), 4.08 (dt, *J* = 5.3, 1.6 Hz, 2H), 4.00 – 3.97 (m, 2H), 3.80 (ddd, *J* = 15.1, 9.9, 3.0 Hz, 2H), 3.68 – 3.64 (m, 2H), 3.57 (dt, *J* = 13.3, 3.6 Hz, 2H), 3.46 (ddd, *J* = 13.3, 9.9, 3.7 Hz, 2H), 2.28 (s, 3H); **^{13}C NMR** (176 MHz, DMSO) δ 160.7, 158.1, 148.9, 147.6, 145.8, 137.6, 134.5, 128.0, 127.9, 125.5, 120.4, 116.9, 113.0, 111.4, 108.6, 101.7, 71.2, 64.5, 62.9, 56.7, 54.3, 37.2, 20.8; **IR** (film, cm^{-1}): 3030, 2890, 1585, 1504, 1494, 1450, 1358, 1193, 1121, 1064, 1012; **HRMS:** m/z calculated for $C_{21}H_{27}N_4O_3$ $[M]^+$ 383.2078; found 383.2068.

1-(benzo[d][1,3]dioxol-5-ylmethyl)-1-(2-(2-(but-3-en-1-yloxy)ethoxy)ethyl)-4-(pyrimidin-2-yl)piperazin-1-ium 4-methylbenzenesulfonate

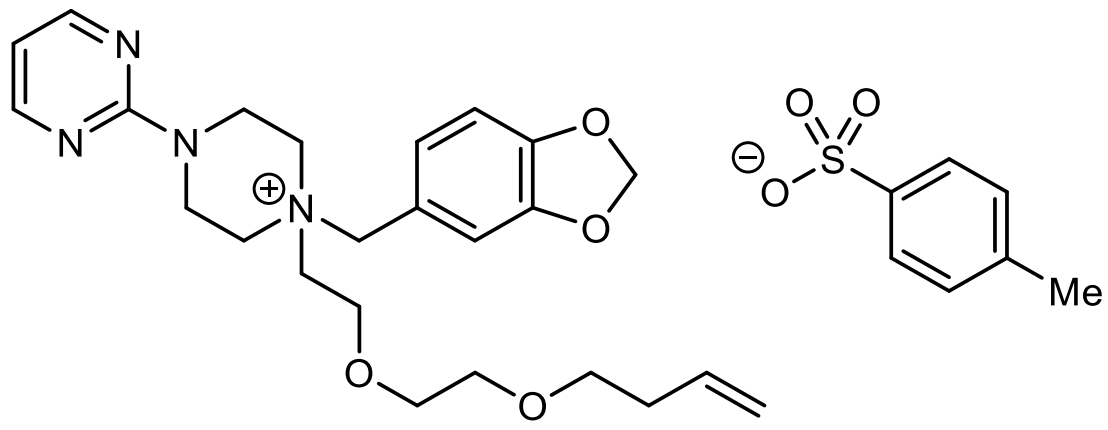

Prepared by general procedure **2.5.5.2.** using piribedil (149 mg, 0.50 mmol, 1.0 equiv), 2-(2-(but-3-en-1-yloxy)ethoxy)ethyl 4-methylbenzenesulfonate (**16**) (314 mg, 1.0 mmol, 2.0 equiv) in acetonitrile (0.50 mL) at 90 °C over 72 h. Purification by reverse phase automated flash column chromatography (0–100% acetonitrile in water) gave the title compound as a beige solid (192 mg, 0.31 mmol, 62% yield). **^{1}H NMR** (700 MHz, CD_3CN) δ 8.39 (dd, *J* = 4.8, 0.8 Hz, 2H), 7.57 – 7.55 (m, 2H), 7.15 (t, *J*

= 2.0 Hz, 1H), 7.11 – 7.07 (m, 3H), 6.91 (dd, *J* = 8.0, 1.2 Hz, 1H), 6.70 (td, *J* = 4.8, 0.8 Hz, 1H), 6.03 (s, 2H), 5.81 (ddt, *J* = 17.1, 10.2, 6.7 Hz, 1H), 5.06 (ddt, *J* = 17.2, 2.1, 1.6 Hz, 1H), 4.99 (ddt, *J* = 10.3, 2.3, 1.3 Hz, 1H), 4.71 (d, *J* = 3.4 Hz, 2H), 4.47 (dt, *J* = 15.6, 4.0 Hz, 2H), 3.99 – 3.95 (m, 2H), 3.70 (ddd, *J* = 15.3, 10.2, 3.1 Hz, 2H), 3.67 – 3.64 (m, 2H), 3.59 (ddt, *J* = 6.4, 2.9, 1.5 Hz, 4H), 3.56 (dt, *J* = 12.8, 3.4 Hz, 1H), 3.49 (t, *J* = 6.7 Hz, 3H), 2.30 (s, 3H), 2.28 (tt, *J* = 6.7, 1.4 Hz, 2H); **^{13}C NMR** (176 MHz, CD_3CN) δ 162.1, 159.0, 150.5, 149.2, 146.9, 139.4, 136.7, 129.3, 129.3, 126.6, 121.3, 118.3, 116.8, 114.3, 112.6, 109.4, 103.1, 71.2, 71.1, 70.4, 67.2, 65.0, 58.1, 55.3, 38.5, 34.9, 21.3; **IR** (film, cm^{-1}): 2922; 1586, 1554, 1494,1451, 1359, 1257, 1193, 1122, 1034, 1012; **HRMS:** m/z calculated for $C_{24}H_{33}N_4O_4$ $[M]^+$ 441.2496; found 441.2502.

1-(2-(4-allylpiperidin-1-yl)-2-oxoethyl)-1-(benzo[*d*][1,3]-dioxol-5-ylmethyl)-4-(pyrimidin-2-yl)piperazin-1-ium 4-methylbenzenesulfonate

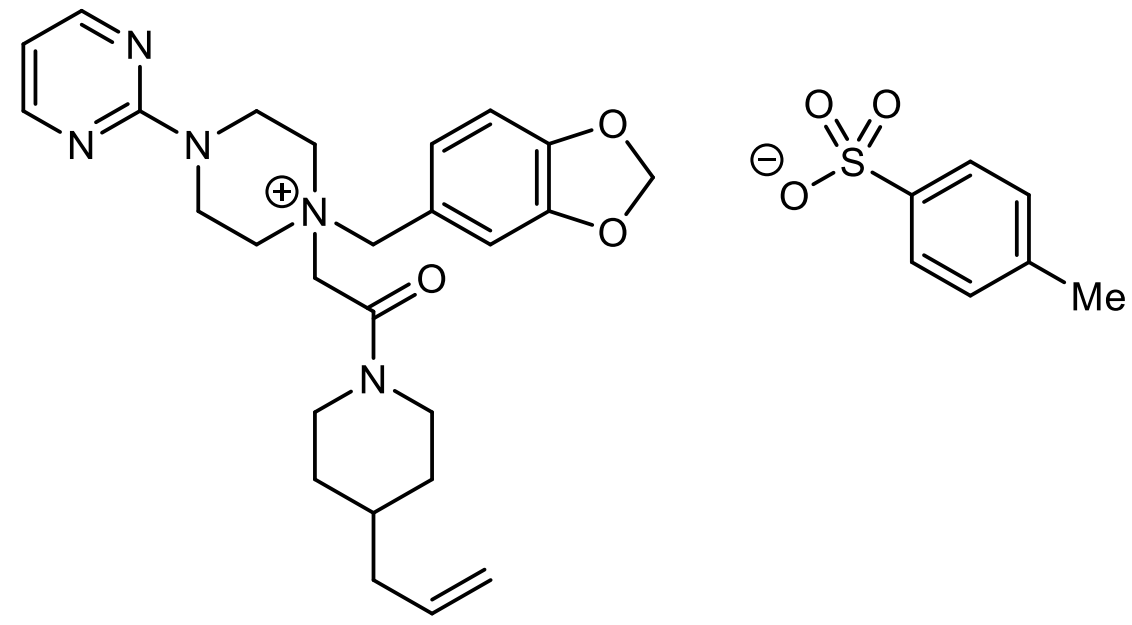

Started according to general procedure **2.5.5.2.** using piribedil (149 mg, 0.50 mmol, 1.0 equiv) and 2-(4-allylpiperidin-1-yl)-2-oxoethyl 4-methylbenzenesulfonate (**17**) (257 mg, 0.76 mmol, 1.5 equiv) in acetonitrile (0.50 mL) at 60 °C over 72 h. Following this, the reaction was cooled down and the solvent was blown down with a flow of nitrogen. The residue was suspended in ethyl acetate (3.0 mL), transferred into two 2 mL eppendorfs and centrifugated (2 minutes, 3800 rpm). The supernatants were pipetted out and 1.5 mL of ethyl acetate was added to each eppendorf. Each of these were vortexed for 10 seconds and centrifugated again (2 minutes, 3800 rpm). The supernatants were pipetted out, and the solid residues were dissolved in dichloromethane, combined and the solvent was blown down with a flow of nitrogen to give the title compound as a white solid (172 mg, 0.27 mmol, 54% yield). **^{1}H NMR** (700 MHz, MeOD) δ 8.43 (d, *J* = 4.8 Hz, 2H), 7.70 – 7.66 (m, 2H), 7.22 – 7.18 (m, 2H), 7.00 – 6.94 (m, 2H), 6.92 – 6.89 (m, 1H), 6.75 (t, *J* = 4.8 Hz, 1H), 6.08 – 6.05 (m, 2H), 5.81 (ddt, *J* = 16.7, 10.5, 7.1 Hz, 1H), 5.12 (d, *J* = 13.1 Hz, 1H), 5.07 – 4.99 (m, 2H), 4.83 (d, *J* = 13.6 Hz, 1H), 4.61 (ddt, *J* = 13.2, 4.6, 2.4 Hz, 1H), 4.49 (ddt, *J* = 20.1, 15.8, 4.7 Hz, 2H), 4.43 (d, *J* = 17.0 Hz, 1H), 4.23 (d, *J*

= 12.4 Hz, 1H), 4.06 (d, *J* = 16.9 Hz, 1H), 3.97 (tdd, *J* = 15.4, 9.1, 3.2 Hz, 2H), 3.84 (ddd, *J* = 12.8, 9.2, 3.5 Hz, 2H), 3.75 (ddd, *J* = 12.8, 9.1, 3.6 Hz, 1H), 3.64 (ddt, *J* = 14.0, 4.6, 2.4 Hz, 1H), 3.02 – 2.94 (m, 1H), 2.71 (td, *J* = 12.9, 2.9 Hz, 1H), 2.36 (s, 3H), 2.09 – 2.04 (m, 2H), 1.87 – 1.81 (m, 1H), 1.74 – 1.60 (m, 2H), 1.19 (tdd, *J* = 13.0, 11.5, 4.3 Hz, 1H), 1.05 – 0.97 (m, 1H); **^{13}C NMR** (176 MHz, MeOD) δ 163.2, 162.4, 159.3, 151.4, 150.0, 143.7, 141.6, 137.5, 129.8, 128.5, 126.9, 121.5, 116.9, 113.2, 112.9, 110.0, 103.4, 65.5, 60.8, 60.2, 54.8, 46.4, 43.4, 41.7, 38.8, 38.7, 36.9, 33.3, 32.7, 21.3; **IR** (thin film, cm^{-1}) 2927, 1649, 1459, 1191, 1013; **HRMS**: m/z calculated for $C_{26}H_{34}N_5O_3^+$ [M]$^+$ 464.2656; found 464.2645.

1-(benzo[d][1,3]dioxol-5-ylmethyl)-4-(pyrimidin-2-yl)-1-(2-(4-(4,4,5,5-tetramethyl-1,3,2-dioxaborolan-2-yl)phenoxy)ethyl)piperazin-1-ium 4-methylbenzenesulfonate

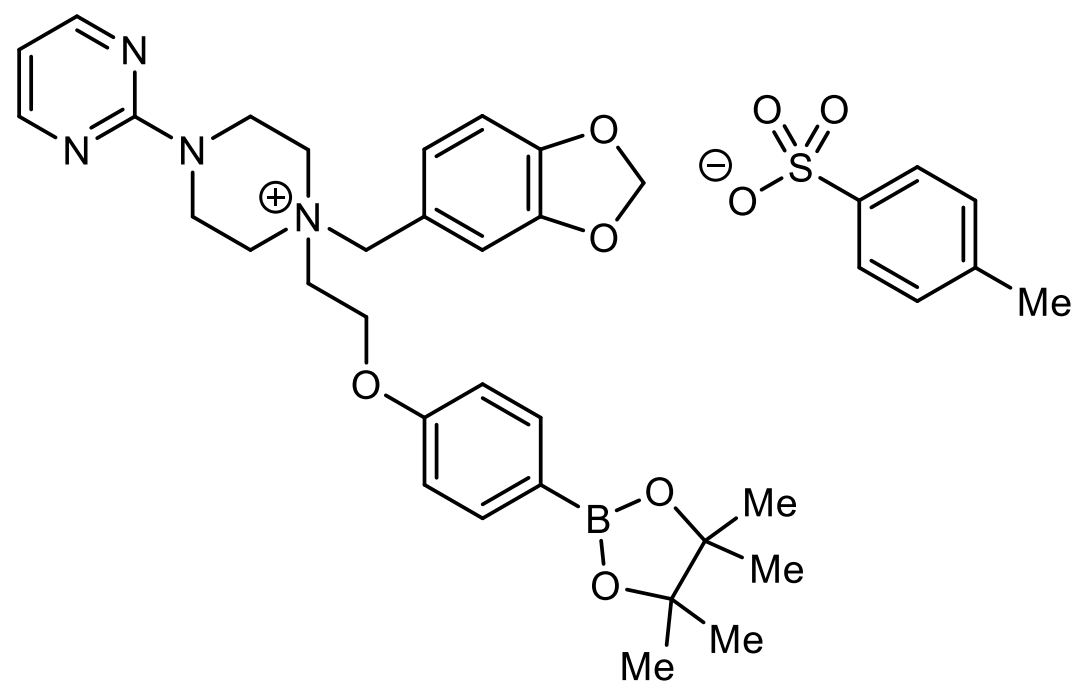

Prepared by general procedure **2.5.5.1.** using piribedil (149 mg, 0.50 mmol, 1.0 equiv), 2-(4-(4,4,5,5-tetramethyl-1,3,2-dioxaborolan-2-yl)phenoxy)ethyl 4-methylbenzenesulfonate (**18**) (628 mg, 1.5 mmol, 3.0 equiv) in acetonitrile (0.50 mL) at 90 °C over 72 h. Purification by automated column chromatography (2–15% methanol in dichloromethane) gave the title compound as a beige solid (142 mg, 0.20 mmol, 40% yield). **^{1}H NMR** (700 MHz, $CDCl_3$) δ 8.33 (d, *J* = 4.8 Hz, 2H), 7.79 – 7.77 (m, 2H), 7.75 – 7.73 (m, 2H), 7.15 (dd, *J* = 8.1, 1.8 Hz, 1H), 7.12 – 7.09 (m, 3H), 6.89 – 6.86 (m, 2H), 6.83 (d, *J* = 8.0 Hz, 1H), 6.63 (t, *J* = 4.8 Hz, 1H), 5.99 (s, 2H), 5.08 (s, 2H), 4.67 – 4.62 (m, 2H), 4.58 (d, *J* = 15.5 Hz, 2H), 4.52 – 4.45 (m, 2H), 3.95 (d, *J* = 12.9 Hz, 2H), 3.92 – 3.86 (m, 2H), 3.50 (ddd, *J* = 13.4, 10.1, 3.8 Hz, 2H), 2.33 (s, 3H), 1.34 (s, 12H); **^{13}C NMR** (176 MHz, $CDCl_3$) δ 160.8, 159.4, 158.0, 150.0, 148.6, 143.7, 139.4, 137.0, 128.8, 128.7, 126.1, 119.5, 113.8, 113.5, 112.1, 109.2, 102.0, 83.9, 66.2, 61.9, 56.5, 37.8, 25.0, 21.5; **IR** (film, cm^{-1}): 2980, 1606, 1586, 1450, 1362, 1259, 1241, 1215, 1034, 1013; **HRMS:** m/z calculated for $C_{30}H_{38}BN_4O_5$ [M]$^+$ 545.2929; found 545.2935.

1-(benzo[d][1,3]dioxol-5-ylmethyl)-1-(2-oxo-2-(4-(4-(4,4,5,5-tetramethyl-1,3,2-dioxaborolan-2-yl)phenyl)piperazin-1-yl)ethyl)-4-(pyrimidin-2-yl)piperazin-1-ium 4-methylbenzenesulfonate

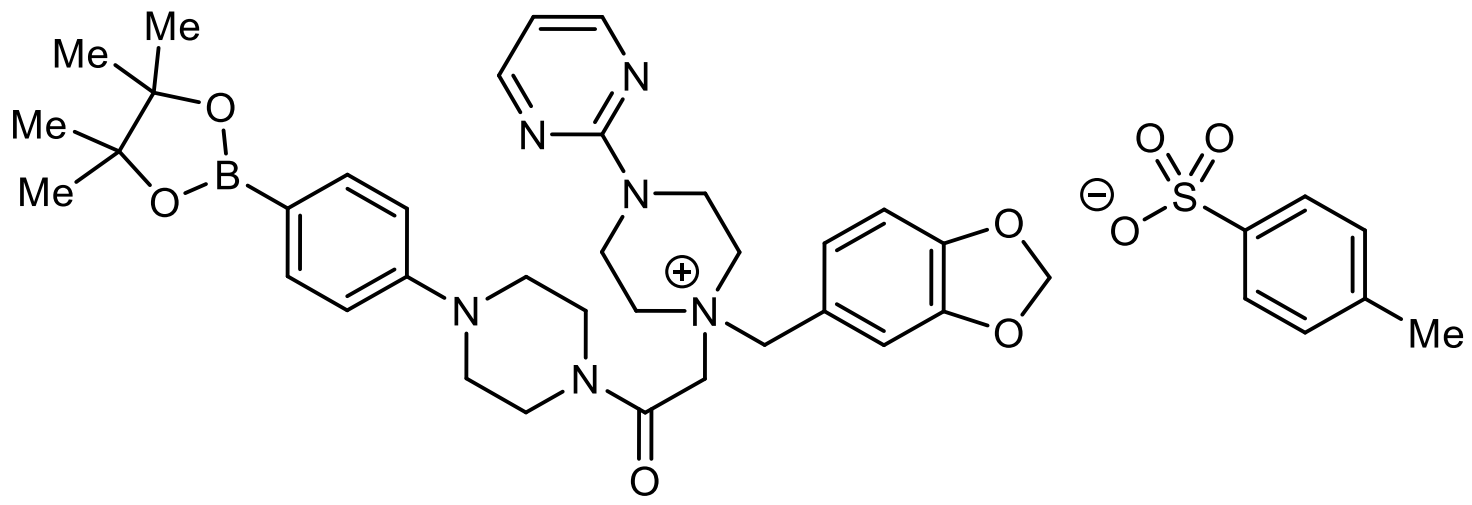

Started according to general procedure **2.5.5.1.** using piribedil (149 mg, 0.50 mmol, 1.0 equiv), 2-oxo-2-(4-(4-(4,4,5,5-tetramethyl-1,3,2-dioxaborolan-2-yl)phenyl)piperazin-1-yl)ethyl 4 methylbenzene-sulfonate (**19**) (375 mg, 0.75 mmol, 1.5 equiv) in acetonitrile (1.0 mL) at 60 °C over 72 h. Following this, the reaction was cooled down and the solvent was blown down with a flow of nitrogen. The residue was suspended in ethyl acetate (3.0 mL) and transferred into two 2 mL eppendorfs and centrifugated (2 minutes, 3800 rpm). The supernatants were pipetted out and 1.5 mL of ethyl acetate was added to each eppendorf. Each of these were vortexed for 10 seconds and centrifugated again (2 minutes, 3800 rpm). The supernatants were pipetted out, and the solid residues were dissolved in dichloromethane, combined and the solvent was blown down with a flow of nitrogen to give the title compound as a white solid (240 mg, 0.30 mmol, 60% yield). **^{1}H NMR** (700 MHz, CD_3CN) δ 8.42 (dd, *J* = 4.7, 0.6 Hz, 2H), 7.62 – 7.59 (m, 2H), 7.58 – 7.54 (m, 2H), 7.11 (dtd, *J* = 7.3, 1.7, 0.9 Hz, 2H), 6.93 – 6.90 (m, 5H), 6.74 (t, *J* = 4.8 Hz, 1H), 5.97 (s, 2H), 4.90 (s, 2H), 4.40 (dt, *J* = 15.6, 4.6 Hz, 2H), 4.15 (s, 1H), 3.99 (d, *J* = 12.7 Hz, 2H), 3.92 (ddd, *J* = 15.6, 9.1, 3.2 Hz, 2H), 3.76 (t, *J* = 5.3 Hz, 2H), 3.69 (ddd, *J* = 12.8, 9.0, 3.6 Hz, 2H), 3.47 – 3.40 (m, 2H), 3.32 (t, *J* = 5.3 Hz, 2H), 3.15 (t, *J* = 5.2 Hz, 2H), 2.31 (s, 3H), 1.30 (s, 12H); **^{13}C NMR** (176 MHz, CD_3CN) δ 163.0, 162.1, 159.1, 153.9, 150.9, 149.5, 146.9, 139.4, 136.8, 129.2, 128.3, 126.6, 121.0, 118.3, 115.6, 113.3, 112.7, 109.8, 103.2, 84.4, 65.3, 59.9, 54.6, 48.6, 48.5, 45.5, 42.4, 25.2, 21.3; **IR** (film, cm^{-1}): 2977, 1654, 1606, 1586, 1451, 1364, 1231, 1193, 1146, 1034, 1013; **HRMS:** m/z calculated for $C_{34}H_{44}BN_6O_5$ $[M]^+$ 627.3461; found 627.3464.

2-(4-(4-(benzo[*d*][1,3]dioxol-5-yl)butyl)piperazin-1-yl)pyrimidine

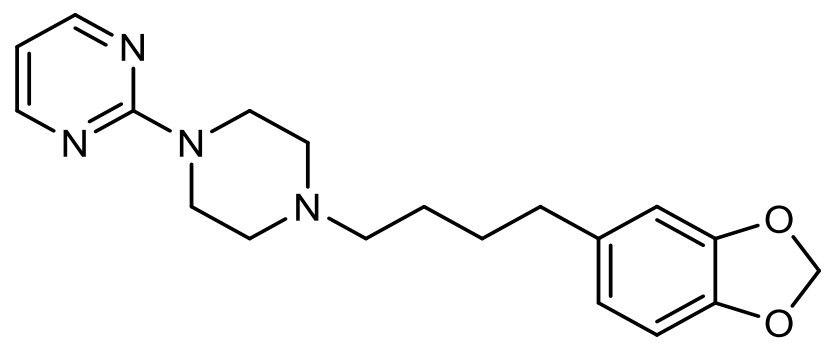

Prepared using general procedure **2.5.6.1.** using 1-(benzo[*d*][1,3]dioxol-5-ylmethyl)-4-(pyrimidin-2-yl)-1-(3-(4,4,5,5-tetramethyl-1,3,2-dioxaborolan-2-yl)propyl)piperazin-1-ium 4-methylbenzenesulfonate (53% by mass in pinacol, 370 mg, 0.31 mmol, 1.0 equiv), SPhos Pd G3 (8.6 mg, 0.01 mmol, 3 mol%), and potassium phosphate tribasic (212 mg, 1.0 mmol, 3.2 equiv) in 4:1 1,4-dioxane/water (0.20 M). Purification by automated flash column chromatography (0–10% ethyl acetate with 5% triethylamine in petroleum ether for 10 minutes, 10–50% ethyl acetate with 5% triethylamine for 10 minutes, 50–100% ethyl acetate with 5% triethylamine in petroleum ether for 5 minutes, 100% ethyl acetate with 5% triethylamine for 5 minutes) gave the title compound as a colourless oil (30 mg, 0.09 mmol, 28% yield). **^{1}H NMR** (700 MHz, $CDCl_3$) δ 8.28 (d, *J* = 4.7 Hz, 2H), 6.70 (d, *J* = 7.9 Hz, 1H), 6.66 (d, *J* = 1.6 Hz, 1H), 6.60 (dd, *J* = 7.9, 1.7 Hz, 1H), 6.45 (t, *J* = 4.7 Hz, 1H), 5.89 (s, 2H), 3.82 – 3.78 (m, 4H), 2.54 (t, *J* = 7.5 Hz, 2H), 2.50 – 2.44 (m, 4H), 2.38 – 2.34 (m, 2H), 1.63 – 1.57 (m, 2H), 1.57 – 1.50 (m, 2H); **^{13}C NMR** (176 MHz, $CDCl_3$) δ 161.8, 157.8, 147.6, 145.6, 136.4, 121.2, 109.9, 109.0, 108.2, 100.8, 58.8, 53.3, 43.8, 35.6, 29.7, 26.4; **IR** (thin film, cm^{-1}) 2939, 1489, 1360, 1243, 1040, 984, 798; **HRMS**: m/z calculated for $C_{19}H_{25}N_4O_2$ $[M+H]^+$ 341.1972; found 341.1980.

2-(4-(5-(benzo[*d*][1,3]dioxol-5-yl)pentyl)piperazin-1-yl)pyrimidine (**14a**)

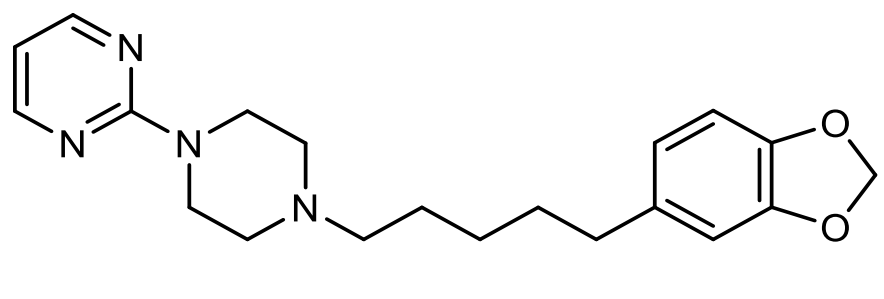

Prepared using general procedure **2.5.6.4.** using 1-(benzo[*d*][1,3]dioxol-5-ylmethyl)-1-(but-3-en-1-yl)-4-(pyrimidin-2-yl)piperazin-1-ium 4-methylbenzenesulfonate (**12a**) (52 mg, 0.10 mmol, 1.0 equiv) and 9-borabicyclo[3.3.1]nonane dimer (24 mg, 0.10 mmol, 1.0 equiv) in 1,4-dioxane (0.40 mL) then using SPhos Pd G3 (4.3 mg, 0.01 mmol, 5 mol%), and potassium phosphate tribasic (64 mg, 0.30 mmol, 3.0 equiv) in 9:1 1,4-dioxane/water (0.20 M). Purification by automated flash column chromatography (0–30% acetone in petroleum ether for 15 minutes, 30–50% acetone in petroleum

ether for 4 minutes, 50% acetone in petroleum ether for 1 minute) gave the title compound as a white solid (17 mg, 0.05 mmol, 47% yield). **^{1}H NMR** (700 MHz, $CDCl_3$) δ 8.30 (d, *J* = 4.7 Hz, 2H), 6.72 (d, *J* = 7.8 Hz, 1H), 6.67 (d, *J* = 1.7 Hz, 1H), 6.62 (dd, *J* = 7.8, 1.7 Hz, 1H), 6.47 (t, *J* = 4.7 Hz, 1H), 5.91 (s, 2H), 3.83 (dd, *J* = 6.1, 4.2 Hz, 4H), 2.56 – 2.51 (m, 2H), 2.49 (t, *J* = 5.2 Hz, 4H), 2.38 – 2.33 (m, 2H), 1.64 – 1.52 (m, 4H), 1.38 – 1.31 (m, 2H); **^{13}C NMR** (176 MHz, $CDCl_3$) δ 161.8, 157.8, 147.6, 145.6, 136.7, 121.2, 109.9, 109.0, 108.2, 100.8, 59.0, 53.3, 43.8, 35.7, 31.8, 27.2, 26.9; **IR** (thin film, cm^{-1}) 2937, 1490, 1361, 1260, 1249, 1040, 984, 798; **HRMS**: m/z calculated for $C_{20}H_{26}N_4O_2$ $[M+H]^+$ 355.2129; found 355.2129.

2-(4-(6-(benzo[*d*][1,3]dioxol-5-yl)hexyl)piperazin-1-yl)pyrimidine (**14b**)

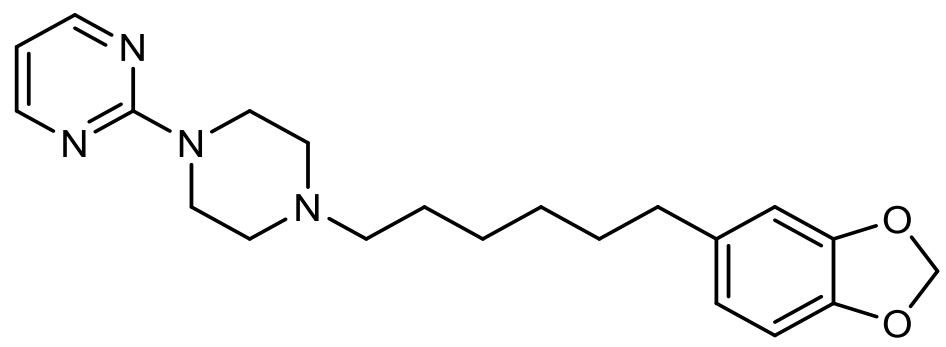

Prepared using general procedure **2.5.6.3.** using 1-(benzo[*d*][1,3]dioxol-5-ylmethyl)-1-(pent-4-en-1-yl)-4-(pyrimidin-2-yl)piperazin-1-ium 4-methylbenzenesulfonate (**12b**) (108 mg, 0.20 mmol, 1.0 equiv) and 9-borabicyclo[3.3.1]nonane (0.5 M in THF, 0.80 mL, 0.40 mmol, 2.0 equiv) then using SPhos Pd G3 (4.3 mg, 0.01 mmol, 3 mol%), and potassium phosphate tribasic (64 mg, 0.15 mmol, 3.0 equiv) in 4:5:1 tetrahydrofuran/1,4-dioxane/water (0.10 M). Purification by automated flash column chromatography (0–20% ethyl acetate in petroleum ether for 10 minutes, 20–50% ethyl acetate in petroleum ether for 10 minutes, 50–100% ethyl acetate in petroleum acetate for 3 minutes, 100% ethyl acetate for 2 minutes) gave the title compound as a white solid (22 mg, 0.06 mmol, 58% yield). **^{1}H NMR** (700 MHz, $CDCl_3$) δ 8.30 (d, *J* = 4.7 Hz, 2H), 6.72 (d, *J* = 7.9 Hz, 1H), 6.67 (d, *J* = 1.6 Hz, 1H), 6.61 (dd, *J* = 7.9, 1.7 Hz, 1H), 6.47 (t, *J* = 4.7 Hz, 1H), 5.91 (s, 2H), 3.85 – 3.81 (m, 4H), 2.55 – 2.51 (m, 2H), 2.48 (t, *J* = 5.1 Hz, 4H), 2.38 – 2.33 (m, 2H), 1.58 (d, *J* = 14.6 Hz, 2H), 1.54 – 1.49 (m, 2H), 1.34 (p, *J* = 3.5 Hz, 4H); **^{13}C NMR** (176 MHz, CDCl3) δ 161.8, 157.8, 147.6, 145.6, 136.8, 121.2, 109.9, 109.0, 108.2, 100.8, 59.0, 53.3, 43.8, 35.8, 31.8, 31.1, 29.2, 27.6, 27.0; **IR** (thin film, cm^{-1}) 2936, 1491, 1361, 1261, 1040, 984, 798; **HRMS:** m/z calculated for $C_{21}H_{28}N_4O_2$ $[M+H]^+$ 369.2285; found 369.2284.

2-(4-(2-(4-(benzo[d][1,3]dioxol-5-yl)butoxy)ethyl)piperazin-1-yl)pyrimidine (**20**)

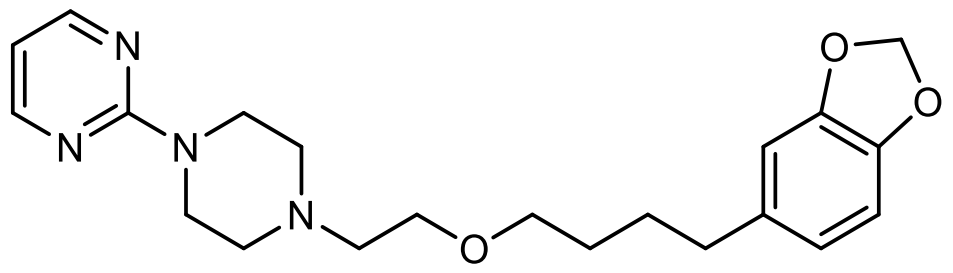

Prepared by general procedure **2.5.6.3.** using 1-(2-(allyloxy)ethyl)-1-(benzo[d][1,3]dioxol-5-ylmethyl)-4-(pyrimidin-2-yl)piperazin-1-ium 4-methylbenzenesulfonate (56 mg, 0.10 mmol, 1.0 equiv), 9-BBN (0.5 M in THF, 0.40 mL, 0.20 mmol, 2.0 equiv), then using SPhos Pd G3 (4.3 mg, 0.005 mmol, 5 mol%) and potassium phosphate tribasic (64 mg, 0.30 mmol, 3.0 equiv) in 4:5:1 tetrahydrofuran/1,4-dioxane/water (0.10 M). Purification by automated flash column chromatography (10–50% ethyl acetate in petroleum ether) gave the title compound as a colourless oil (23 mg, 0.06 mmol, 59% yield). **^{1}H NMR** (700 MHz, $CDCl_3$) δ 8.30 (d, *J* = 4.7 Hz, 2H), 6.71 (d, *J* = 7.9 Hz, 1H), 6.67 (d, *J* = 1.7 Hz, 1H), 6.61 (dd, *J* = 7.9, 1.7 Hz, 1H), 6.47 (t, *J* = 4.7 Hz, 1H), 5.91 (s, 2H), 3.85 – 3.81 (m, 4H), 3.58 (t, *J* = 5.9 Hz, 2H), 3.45 (t, *J* = 6.2 Hz, 2H), 2.62 (t, *J* = 5.9 Hz, 2H), 2.57 – 2.54 (m, 6H), 1.67 – 1.57 (m, 4H); **^{13}C NMR** (176 MHz, $CDCl_3$) δ 161.8, 157.8, 147.6, 145.6, 136.4, 121.2, 109.9, 109.0, 108.2, 100.8, 71.2, 68.7, 58.1, 53.7, 43.8, 35.5, 29.2, 28.4; **IR** (film, cm^{-1}): 2926, 2858, 1586, 1548, 1503, 1489, 1445, 1360, 1244, 1110, 1039, 984; **HRMS:** m/z calculated for $C_{21}H_{29}N_4O_3$ $[M + H]^+$ 385.2234; found 385.2232.

2-(4-(2-(2-((5-(benzo[d][1,3]dioxol-5-yl)pentyl)oxy)ethoxy)ethyl)piperazin-1-yl)pyrimidine (**21**)

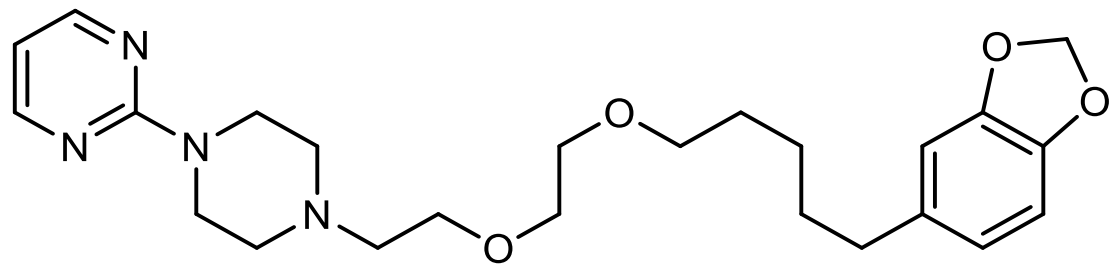

Prepared by general procedure **2.5.6.3.** using 1-(benzo[d][1,3]dioxol-5-ylmethyl)-1-(2-(2-(but-3-en-1-yloxy)ethoxy)ethyl)-4-(pyrimidin-2-yl)piperazin-1-ium 4-methylbenzenesulfonate (62 mg, 0.10 mmol, 1.0 equiv), 9-BBN (0.5 M in THF, 0.40 mL, 0.20 mmol, 2.0 equiv), then using SPhos Pd G3 (4.3 mg, 0.005 mmol, 5 mol%) and potassium phosphate tribasic (64 mg, 0.30 mmol, 3.0 equiv) in 4:5:1 tetrahydrofuran/1,4-dioxane/water (0.10 M). Purification by automated flash column chromatography (10–50% acetone in petroleum ether) gave the title compound as a colourless oil (27 mg, 0.61 mmol, 61% yield). **^{1}H NMR** (500 MHz, $CDCl_3$) δ 8.29 (d, *J* = 4.7 Hz, 2H), 6.71 (d, *J* = 7.9 Hz, 1H), 6.66 (d, *J* = 1.7 Hz, 1H), 6.61 (dd, *J* = 7.9, 1.7 Hz, 1H), 6.47 (t, *J* = 4.7 Hz, 1H), 5.90 (s, 2H), 3.85 – 3.82 (m, 5H), 3.66 (t, *J* = 5.9 Hz, 2H), 3.64 – 3.60 (m, 2H), 3.59 – 3.56 (m, 2H), 3.45 (t, *J* = 6.8 Hz, 2H), 2.64 (t, *J* = 5.9 Hz, 2H), 2.59 – 2.55 (m, 4H), 2.55 – 2.50 (m, 2H), 1.64 – 1.54 (m, 4H), 1.41

– 1.32 (m, 2H); **^{13}C NMR** (126 MHz, $CDCl_3$) δ 161.8, 157.8, 147.6, 145.6, 136.7, 121.2, 109.9, 109.0, 108.2, 100.8, 71.6, 70.6, 70.3, 69.1, 58.1, 53.6, 43.7, 35.8, 31.7, 29.7, 25.8; **IR** (film, cm^{-1}): 2926, 2856, 1585, 1547, 1489, 1443, 1359, 1259, 1244, 1111, 1038, 984; **HRMS:** m/z calculated for $C_{24}H_{35}N_4O_4$ [M + H]$^+$ 443.2653; found 443.2665.

1-(4-(4-(benzo[*d*][1,3]dioxol-5-yl)butyl)piperidin-1-yl)-2-(4-(pyrimidin-2-yl)piperazin-1-yl)ethan-1-one (**22**)

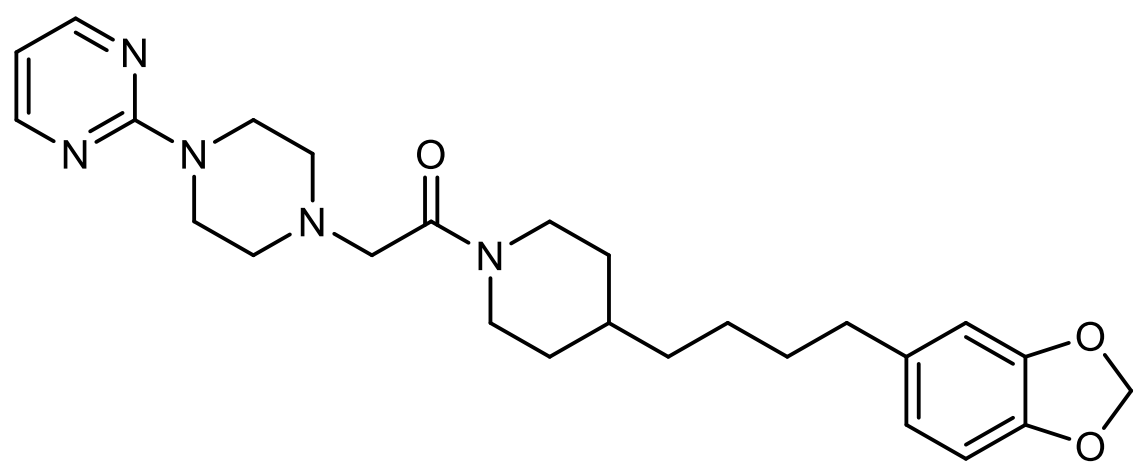

Prepared using general procedure **2.5.6.4.** using 1-(2-(4-allylpiperidin-1-yl)-2-oxoethyl)-1-(benzo[*d*][1,3]-dioxol-5-ylmethyl)-4-(pyrimidin-2-yl)piperazin-1-ium 4-methylbenzenesulfonate (64 mg, 0.10 mmol, 1.0 equiv) and 9-borabicyclo[3.3.1]nonane dimer (24 mg, 0.10 mmol, 1.0 equiv) in 1,4-dioxane (0.40 mL), then using SPhos Pd G3 (2.0 mg, 0.003 mmol, 3 mol%) and potassium phosphate tribasic (32 mg, 0.15 mmol, 1.5 equiv) 9:1 mixture 1,4-dioxane/water (0.20 M). Purification by automated flash column chromatography (0–30% acetone in petroleum ether for 20 minutes, 30–50% acetone in petroleum ether for 4 minutes, 50% acetone in petroleum ether for 6 minutes) gave the title compound as a white solid (14 mg, 0.03 mmol, 62% yield). **^{1}H NMR** (700 MHz, $CDCl_3$) δ 8.30 (d, J = 4.7 Hz, 2H), 6.71 (d, J = 7.8 Hz, 1H), 6.66 (d, J = 1.7 Hz, 1H), 6.60 (dd, J = 7.9, 1.7 Hz, 1H), 6.49 (t, J = 4.7 Hz, 1H), 5.91 (s, 2H), 4.56 (d, J = 13.1 Hz, 1H), 4.05 (d, J = 13.2 Hz, 1H), 3.86 (br s, 4H), 3.31 (d, J = 13.6 Hz, 1H), 3.18 (d, J = 13.7 Hz, 1H), 2.99 – 2.93 (m, 1H), 2.68 – 2.57 (br m, 4H), 2.53 (dt, J = 15.3, 9.1 Hz, 3H), 1.72 (t, J = 10.9 Hz, 2H), 1.55 (p, J = 7.6 Hz, 2H), 1.46 (ddt, J = 12.9, 9.6, 4.8 Hz, 1H), 1.32 (q, J = 7.9 Hz, 2H), 1.25 (dt, J = 9.0, 5.9 Hz, 2H), 1.16 – 1.01 (m, 2H); **^{13}C NMR** (176 MHz, $CDCl_3$) δ 161.8, 157.8, 147.6, 145.6, 136.6, 121.2, 110.1, 108.9, 108.2, 100.8, 53.1, 46.2, 43.7, 42.5, 36.4, 36.1, 35.7, 33.2, 32.3, 32.0, 26.2; **IR** (thin film, cm^{-1}) 2927, 2853, 1643, 1490, 1446, 1361, 1258, 1039, 984; **HRMS:** m/z calculated for $C_{26}H_{36}N_5O_3$ [M+H]$^+$ 466.2813; found 466.2811.

2-(4-(2-(4-(benzo[d][1,3]dioxol-5-ylmethyl)phenoxy)ethyl)piperazin-1-yl)pyrimidine (**23**)

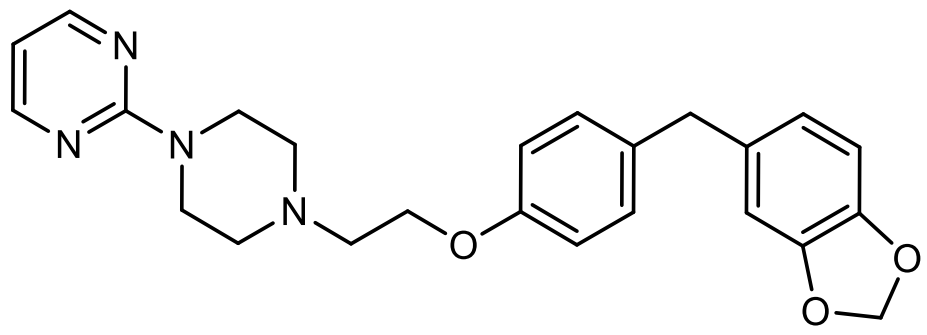

Prepared by general procedure **2.5.6.2.** using 1-(benzo[d][1,3]dioxol-5-ylmethyl)-4-(pyrimidin-2-yl)-1-(2-(4-(4,4,5,5-tetramethyl-1,3,2-dioxaborolan-2-yl)phenoxy)ethyl)piperazin-1-ium 4-methylbenzenesulfonate (92 mg, 0.10 mmol, 1.0 equiv), RuPhos Pd G3 (3.9 mg, 0.005 mmol, 5 mol%), and potassium *tert*-butoxide (34 mg, 0.30 mmol, 3.0 equiv) in 4:1 1,4-dioxane/water (0.50 mL). Purification by automated flash column chromatography (1–20% acetone in petroleum ether) gave the title compound as a white solid (24 mg, 0.06 mmol, 57% yield). **^{1}H NMR** (700 MHz, $CDCl_3$) δ 8.30 (d, *J* = 4.7 Hz, 2H), 7.10 – 7.06 (m, 2H), 6.85 – 6.83 (m, 2H), 6.72 (d, *J* = 8.3 Hz, 1H), 6.65 – 6.62 (m, 2H), 6.48 (t, *J* = 4.7 Hz, 1H), 5.90 (s, 2H), 4.12 (t, *J* = 5.8 Hz, 2H), 3.87 – 3.84 (m, 4H), 3.83 (s, 2H), 2.84 (t, *J* = 5.8 Hz, 2H), 2.64 (t, *J* = 5.1 Hz, 4H); **^{13}C NMR** (176 MHz, $CDCl_3$) δ 161.8, 157.9, 157.3, 147.8, 145.9, 135.6, 133.8, 129.9, 121.7, 114.8, 110.0, 109.4, 108.3, 100.9, 66.0, 57.5, 53.6, 43.8, 40.9; **IR** (film, cm^{-1}): 2926, 2859, 1586, 1509, 1488, 1444, 1360, 1244, 1040, 984, 799; **HRMS:** m/z calculated for $C_{24}H_{27}$N4O3 $[M + H]^+$ 419.2078; found 419.2075.

1-(4-(4-(benzo[d][1,3]dioxol-5-ylmethyl)phenyl)piperazin-1-yl)-2-(4-(pyrimidin-2-yl)piperazin-1-yl)ethan-1-one (**24**)

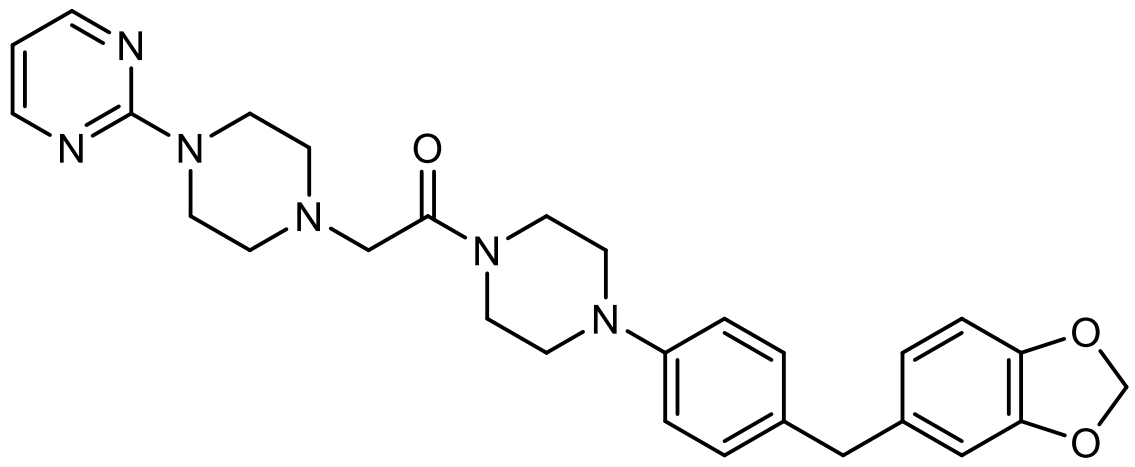

Prepared by general procedure **2.5.6.2.** using 1-(benzo[d][1,3]dioxol-5-ylmethyl)-1-(2-oxo-2-(4-(4-(4,4,5,5-tetramethyl-1,3,2-dioxaborolan-2-yl)phenyl)piperazin-1-yl)ethyl)-4-(pyrimidin-2-yl)piperazin-1-ium 4-methylbenzenesulfonate (79 mg, 0.10 mmol, 1.0 equiv), RuPhos Pd G3 (8.4 mg, 0.01 mmol, 10 mol%), and potassium phosphate tribasic (64 mg, 0.30 mmol, 3.0 equiv) in 4:1 1,4-dioxane/water (1.0 mL). Purification by automated flash column chromatography (0–5% methanol in dichloromethane) gave the title compound as a beige solid (26 mg, 0.052 mmol, 52% yield). **^{1}H NMR** (700 MHz, $CDCl_3$) δ 8.30 (d, *J* = 4.7 Hz, 2H), 7.10 – 7.07 (m, 2H), 6.88 – 6.84 (m, 2H), 6.73 – 6.71 (m, 1H), 6.65 – 6.62 (m, 2H), 6.49 (t, *J* = 4.7 Hz, 1H), 5.90 (s, 2H), 3.84 (dd, *J* = 6.0, 4.0 Hz, 4H), 3.82

(s, 2H), 3.78 (dt, J = 11.0, 5.1 Hz, 4H), 3.27 (s, 2H), 3.16 (t, J = 5.1 Hz, 2H), 3.12 (t, J = 5.2 Hz, 2H), 2.58 (t, J = 5.1 Hz, 4H); **^{13}C NMR** (176 MHz, $CDCl_3$) δ 168.0, 161.8, 157.8, 149.5, 147.8, 145.9, 135.5, 133.6, 129.7, 121.7, 117.0, 110.1, 109.5, 108.2, 100.9, 61.6, 53.1, 50.4, 49.9, 45.8, 43.8, 41.9, 40.8; **IR** (film, cm^{-1}): 2909, 2818, 1646, 1586, 1514, 1489, 1444, 1361, 1258, 1244, 1038, 984, 799; **HRMS:** m/z calculated for $C_{28}H_{33}N_6O_3$ $[M + H]^+$ 501.2609; found 501.2607.

2-(allyloxy)-*N*,*N*-dibenzyl-*N*-methylethan-1-aminium 4-methylbenzenesulfonate

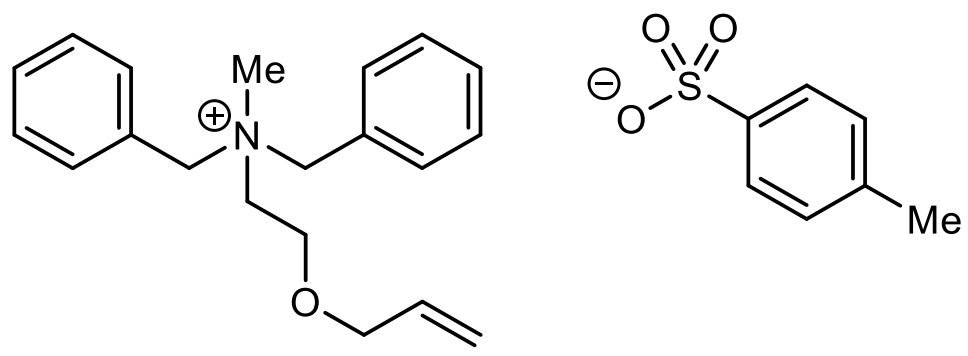

Prepared by general procedure **2.5.5.2.** using *N*-benzyl-*N*-methyl-1-phenylmethanamine (634 mg, 3.0 mmol, 1.0 equiv), 2-(allyloxy)ethyl 4-methylbenzenesulfonate (**15**) (1.15 g, 4.5 mmol, 1.5 equiv) in acetonitrile (3.0 mL) at 90 °C over 72 h. Following this, the reaction was cooled down and the solvent was blown down with a flow of nitrogen. Diethyl ether (10 mL) was added to the crude oil and the resulting mixture was sonicated for 5 min. The mixture was decanted and the supernatant was then removed via pipette. The cycle was repeated twice and the final light-yellow solid was dried to give the title compound (681 mg, 1.5 mmol, 49% yield). **^{1}H NMR** (500 MHz, $CDCl_3$) δ 7.87 – 7.84 (m, 2H), 7.62 – 7.59 (m, 3H), 7.48 – 7.39 (m, 7H), 7.17 – 7.13 (m, 2H), 5.86 (ddt, J = 17.2, 10.4, 5.8 Hz, 1H), 5.27 (dq, J = 17.3, 1.6 Hz, 1H), 5.24 – 5.21 (m, 1H), 5.00 (d, J = 12.6 Hz, 2H), 4.77 (d, J = 12.7 Hz, 2H), 4.03 (dt, J = 5.8, 1.3 Hz, 2H), 3.99 (t, J = 4.9 Hz, 2H), 3.78 – 3.74 (m, 2H), 3.03 (s, 3H), 2.33 (s, 3H); **^{13}C NMR** (126 MHz, $CDCl_3$) δ 143.9, 139.3, 133.8, 133.3, 130.7, 129.4, 128.7, 127.5, 126.2, 118.8, 72.6, 66.4, 64.1, 59.3, 47.3, 21.4; **IR** (film, cm^{-1}): 3377, 3037, 3011, 2922, 1497, 1457, 1215, 1193, 1121, 1034, 1012; **HRMS:** m/z calculated for $C_{20}H_{26}NO$ $[M]^+$ 296.2014; found 296.2025.

N-benzyl-*N*-methyl-2-(4-phenylbutoxy)ethan-1-amine (**25**)

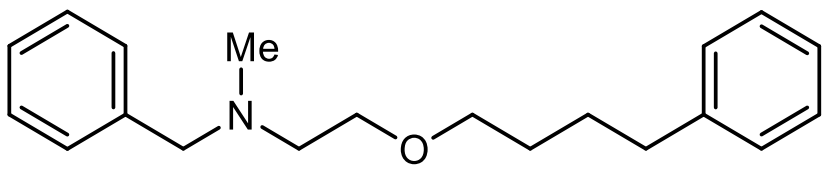

Prepared by general procedure **2.5.6.3.** using 2-(allyloxy)-*N*,*N*-dibenzyl-*N*-methylethan-1-aminium 4-methylbenzenesulfonate (234 mg, 0.50 mmol, 1.0 equiv), 9-BBN (0.5 M in THF, 2.0 mL, 1.0 mmol, 2.0 equiv), then using SPhos Pd G3 (21.7 mg, 0.025 mmol, 5 mol%) and potassium phosphate tribasic (318 mg, 1.5 mmol, 3.0 equiv) in 4:5:1 tetrahydrofuran/1,4-dioxane/water (0.10 M). Purification by

automated flash column chromatography (0–20% ethyl acetate in petroleum ether) gave the title compound as a colourless oil (81.0 mg, 0.28 mmol, 56% yield). **^{1}H NMR** (700 MHz, $CDCl_3$) δ 7.33 – 7.29 (m, 4H), 7.28 – 7.24 (m, 3H), 7.18 (ddt, *J* = 7.5, 5.0, 2.6 Hz, 3H), 3.58 – 3.54 (m, 4H), 3.43 (t, *J* = 6.6 Hz, 2H), 2.65 – 2.60 (m, 4H), 2.27 (s, 3H), 1.68 (dddd, *J* = 14.2, 8.4, 6.6, 3.8 Hz, 2H), 1.65 – 1.59 (m, 2H); **^{13}C NMR** (176 MHz, $CDCl_3$) δ 142.6, 138.9, 129.3, 128.6, 128.41, 128.35, 127.1, 125.8, 71.2, 69.3, 62.8, 56.6, 43.0, 35.9, 29.5, 28.2; **IR** (film, cm^{-1}): 3028, 2937, 2860, 2786, 1497, 1453, 1116, 1051, 1026, 736, 698; **HRMS:** m/z calculated for $C_{20}H_{28}NO$ $[M + H]^+$ 298.2171; found 298.2164.

N-methyl-2-(4-phenylbutoxy)ethan-1-amine (**26**)

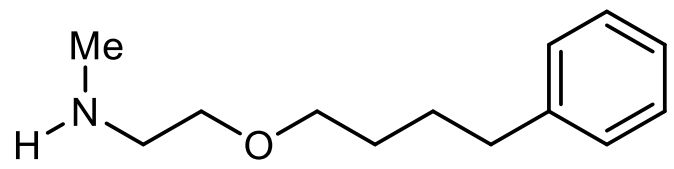

To a solution of *N*-benzyl-*N*-methyl-2-(4-phenylbutoxy)ethan-1-amine (**25**) (29.7 mg, 0.10 mmol, 1.0 equiv) in methanol (1.0 mL) was added $Pd(OH)_2/C$ (20 wt. %; 50% wet, 14.1 mg, 0.01 mmol, 10 mol%). A balloon of hydrogen was connected and the reaction mixture was evacuated and backfilled with hydrogen (3 cycles). The reaction was left to stir at room temperature for 3 hours. Following this, the reaction mixture was sparged with nitrogen for 10 minutes, filtered through Celite© and concentrated *in vacuo* to give the title compound as a colourless liquid (17.9 mg, 0.09 mmol, 87% yield). **^{1}H NMR** (700 MHz, $CDCl_3$) δ 7.28 (d, *J* = 7.6 Hz, 2H), 7.17 (dd, *J* = 7.6, 4.7 Hz, 3H), 3.58 (t, *J* = 5.1 Hz, 2H), 3.46 (t, *J* = 6.4 Hz, 2H), 2.83 (t, *J* = 5.2 Hz, 2H), 2.63 (t, *J* = 7.6 Hz, 2H), 2.51 (s, 3H), 1.68 (qd, *J* = 8.7, 5.6 Hz, 2H), 1.64 – 1.60 (m, 2H); **^{13}C NMR** (176 MHz, $CDCl_3$) δ 142.4, 128.4, 128.3, 125.7, 71.2, 68.9, 50.8, 35.71, 35.69, 29.3, 28.0; **IR** (film, cm^{-1}): 2931, 2859, 2801, 1454, 1114, 1053, 747; **HRMS:** m/z calculated for $C_{13}H_{22}NO$ $[M + H]^+$ 208.1701; found 208.1698.

((dibenzyl(methyl)ammonio)methyl)trifluoroborate

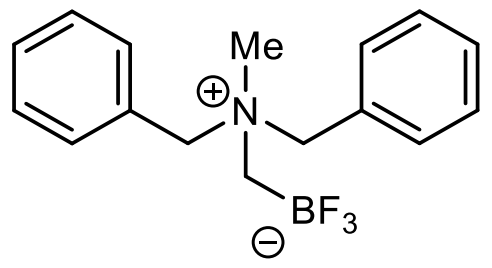

Prepared by general procedure **2.5.2.1. (method I)** using *N*-benzyl-*N*-methyl-1-phenethylmethanamine (1.27 g, 6.0 mmol, 1.0 equiv) and 2-(Bromomethyl)-4,4,5,5-tetramethyl-1,3,2-dioxaborolane (1.2 mL, 7.2 mmol, 1.5 equiv) in acetonitrile (24 mL). Fluorination was performed with aqueous potassium hydrogen fluoride (3 M, 5 mL, 15 mmol, 2.5 equiv) and HCl (4 M in 1,4-

dioxane, 1.5 mL, 6 mmol, 1.0 equiv) in 4:1 acetonitrile/water (0.25 M). The title compound was obtained as a white solid (1.42 g, 4.8 mmol, 81% yield). **^{1}H NMR** (700 MHz, $CDCl_3$) δ 7.50 – 7.47 (m, 6H), 7.47 – 7.43 (m, 4H), 4.56 (d, *J* = 12.8 Hz, 2H), 4.42 (d, *J* = 12.8 Hz, 2H), 2.78 (s, 3H), 2.44 (q, *J* = 4.7 Hz, 2H); **^{13}C NMR** (176 MHz, $CDCl_3$) δ 133.3, 130.7, 129.3, 128.1, 68.2, 49.5; **^{19}F NMR** (471 MHz, $CDCl_3$) δ –137.4; **IR** (film, cm^{-1}): 3038, 1470, 1327, 1025, 945, 910, 880, 736, 703; **HRMS:** m/z calculated for $C_{16}H_{19}BF_2N$ $[M–F]^+$ 274.1573; found 274.1576.

N-benzyl-*N*-methyl-2-phenylethan-1-amine (**27**)

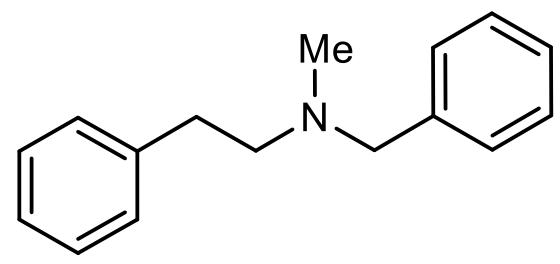

Prepared by general procedure **2.5.3.1.** using ((dibenzyl(methyl)ammonio)methyl)trifluoroborate (586 mg, 2.0 mmol, 1.0 equiv), bis(benzonitrile)palladium(II) chloride (38.4 mg, 0.1 mmol, 5 mol%), RuPhos (93.3 mg, 0.2 mmol, 10 mol%) and potassium *tert*-butoxide (1.12 g, 10 mmol, 5.0 equiv) in 4:1 1,4-dioxane/water (10 mL). Purification by automated flash column chromatography (0–10% acetone in petroleum ether) gave the title compound as a colourless oil (351 mg, 1.6 mmol, 78% yield). Data was in line with previous characterisation.[93] **^{1}H NMR** (700 MHz, $CDCl_3$) δ 7.28 – 7.19 (m, 7H), 7.17 – 7.14 (m, 3H), 3.53 (s, 2H), 2.82 – 2.77 (m, 2H), 2.65 – 2.60 (m, 2H), 2.25 (s, 3H); **^{13}C NMR** (176 MHz, $CDCl_3$) δ 140.7, 139.2, 129.1, 128.9, 128.4, 128.3, 127.1, 126.1, 62.3, 59.3, 42.3, 34.1.

N-benzyl-*N*-methyl-2-oxo-*N*-phenethyl-2-(4-(4-(4,4,5,5-tetramethyl-1,3,2-dioxaborolan-2-yl)phenyl)piperazin-1-yl)ethan-1-aminium 4-methylbenzenesulfonate

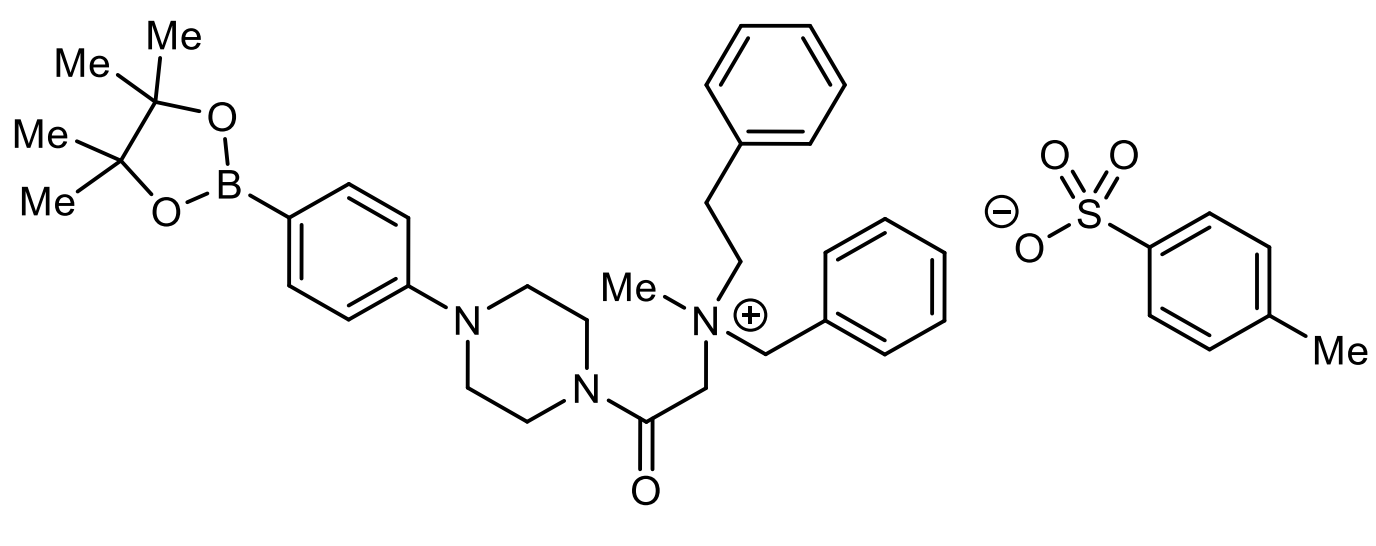

Started according to general procedure **2.5.5.1.** using *N*-benzyl-*N*-methyl-2-phenylethan-1-amine (**27**) (169 mg, 0.75 mmol, 1.0 equiv), 2-oxo-2-(4-(4-(4,4,5,5-tetramethyl-1,3,2-dioxaborolan-2-yl)phenyl)piperazin-1-yl)ethyl 4 methylbenzene-sulfonate (**19**) (565 mg, 1.1 mmol, 1.5 equiv) in acetonitrile (1.0 mL) at 60 °C over 72 h. Following this, the reaction was concentrated to dryness. The residue was redissolved in dichloromethane (3.0 mL) and loaded on a silica plug (h 4 cm; ø 2 cm), eluted with 10% acetone in dichloromethane (4 column volumes) followed by 30% 2-propanol in

dichloromethane (4 column volumes) to give the title compound as a white solid (345 mg, 0.48 mmol, 64% yield). **^{1}H NMR** (700 MHz, $CDCl_3$) δ 7.78 – 7.75 (m, 2H), 7.70 – 7.66 (m, 2H), 7.58 – 7.56 (m, 2H), 7.47 – 7.43 (m, 1H), 7.40 (dd, *J* = 8.1, 6.8 Hz, 2H), 7.26 – 7.17 (m, 5H), 7.08 (d, *J* = 7.9 Hz, 2H), 6.74 – 6.69 (m, 2H), 5.22 (d, *J* = 12.7 Hz, 1H), 5.08 – 5.00 (m, 2H), 4.84 (d, *J* = 16.9 Hz, 1H), 4.06 – 3.93 (m, 1H), 3.76 (td, *J* = 12.2, 5.0 Hz, 1H), 3.73 – 3.66 (m, 2H), 3.64 (t, *J* = 5.3 Hz, 2H), 3.42 (s, 3H), 3.26 – 3.19 (m, 3H), 3.09 (td, *J* = 12.5, 5.3 Hz, 1H), 3.06 – 2.97 (m, 2H), 2.28 (s, 3H), 1.33 (s, 12H); **^{13}C NMR** (176 MHz, $CDCl_3$) δ 162.8, 152.6, 143.8, 139.6, 136.2, 135.4, 133.4, 131.0, 129.5, 129.3, 129.1, 129.0, 128.9, 127.4, 127.3, 126.0, 114.8, 83.6, 67.0, 61.4, 58.8, 49.3, 48.1, 48.0, 44.9, 41.6, 29.4, 25.0, 21.4; **IR** (film, cm^{-1}): 2980, 1656, 1604, 1399, 1362, 1232, 1215, 1194, 1144, 1121, 1034, 1012, 730; **HRMS:** m/z calculated for $C_{34}H_{45}BN_3O_3$ $[M]^+$ 554.3554; found 554.3530.

1-(4-(4-benzylphenyl)piperazin-1-yl)-2-(methyl(phenethyl)amino)ethan-1-one (**28**)

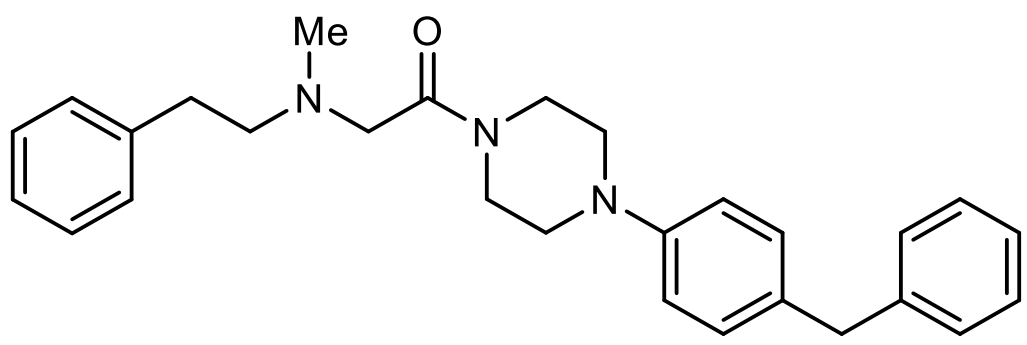

Prepared by general procedure **2.5.6.2.** using *N*-benzyl-*N*-methyl-2-oxo-*N*-phenethyl-2-(4-(4-(4,4,5,5-tetramethyl-1,3,2-dioxaborolan-2-yl)phenyl)piperazin-1-yl)ethan-1-aminium 4-methylbenzenesulfonate (72.6 mg, 0.10 mmol, 1.0 equiv), RuPhos Pd G3 (8.4 mg, 0.01 mmol, 10 mol%), and potassium phosphate tribasic (63.7 mg, 0.30 mmol, 3.0 equiv) in 4:1 1,4-dioxane/water (1.0 mL). Purification by automated flash column chromatography (10–30% acetone in petroleum ether) gave a colourless oil, which was further purified by preparative TLC (10% methanol in dichloromethane) to give the title compound as colourless oil (32.2 mg, 0.075 mmol, 75% yield). **^{1}H NMR** (700 MHz, $CDCl_3$) δ 7.27 (d, *J* = 7.5 Hz, 4H), 7.21 – 7.16 (m, 6H), 7.10 (d, *J* = 8.2 Hz, 2H), 6.83 – 6.80 (m, 2H), 3.92 (s, 2H), 3.68 (t, *J* = 5.2 Hz, 2H), 3.51 (t, *J* = 5.1 Hz, 2H), 3.22 (s, 2H), 3.05 (t, *J* = 5.2 Hz, 2H), 2.86 (t, *J* = 5.1 Hz, 2H), 2.79 (t, *J* = 7.3 Hz, 2H), 2.71 (t, *J* = 7.3 Hz, 2H), 2.33 (s, 3H); **^{13}C NMR** (176 MHz, $CDCl_3$) δ 168.7, 149.4, 141.6, 140.3, 133.1, 129.7, 128.8, 128.7, 128.43, 128.36, 126.1, 126.0, 116.7, 61.6, 59.0, 49.8, 49.7, 45.4, 41.8, 41.7, 41.0, 33.6; **IR** (film, cm^{-1}): 3028, 2920, 2853, 1647, 1515, 1455, 1233, 978; **HRMS:** m/z calculated for $C_{28}H_{34}N_3O$ $[M + H]^+$ 428.2702; found 428.2690.

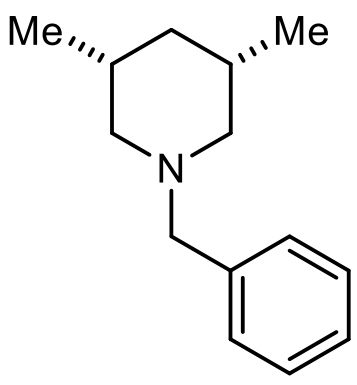

1e

^{1}H NMR, 700 MHz, $CDCl_3$

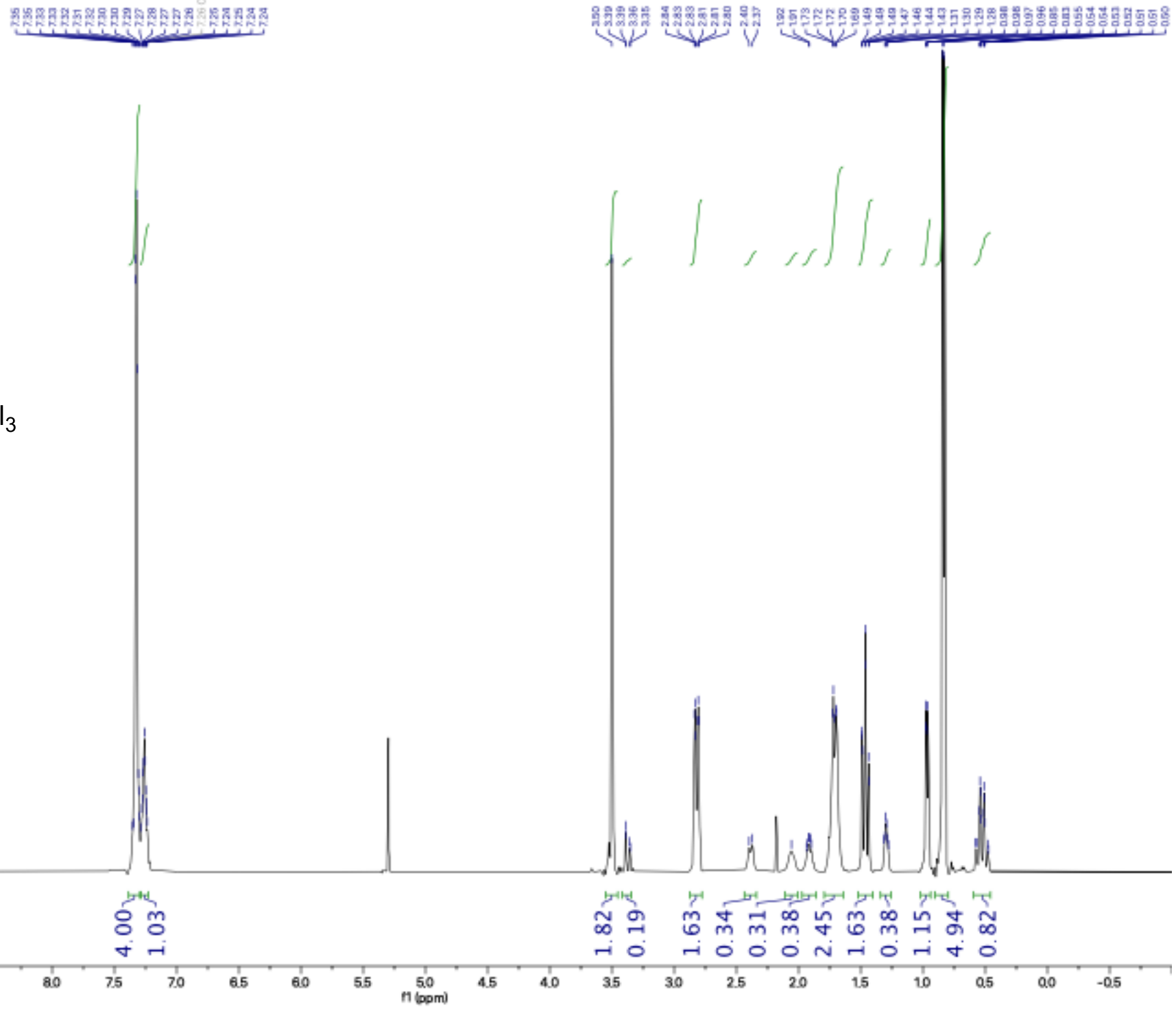

1e

^{13}C NMR, 176 MHz, $CDCl_3$

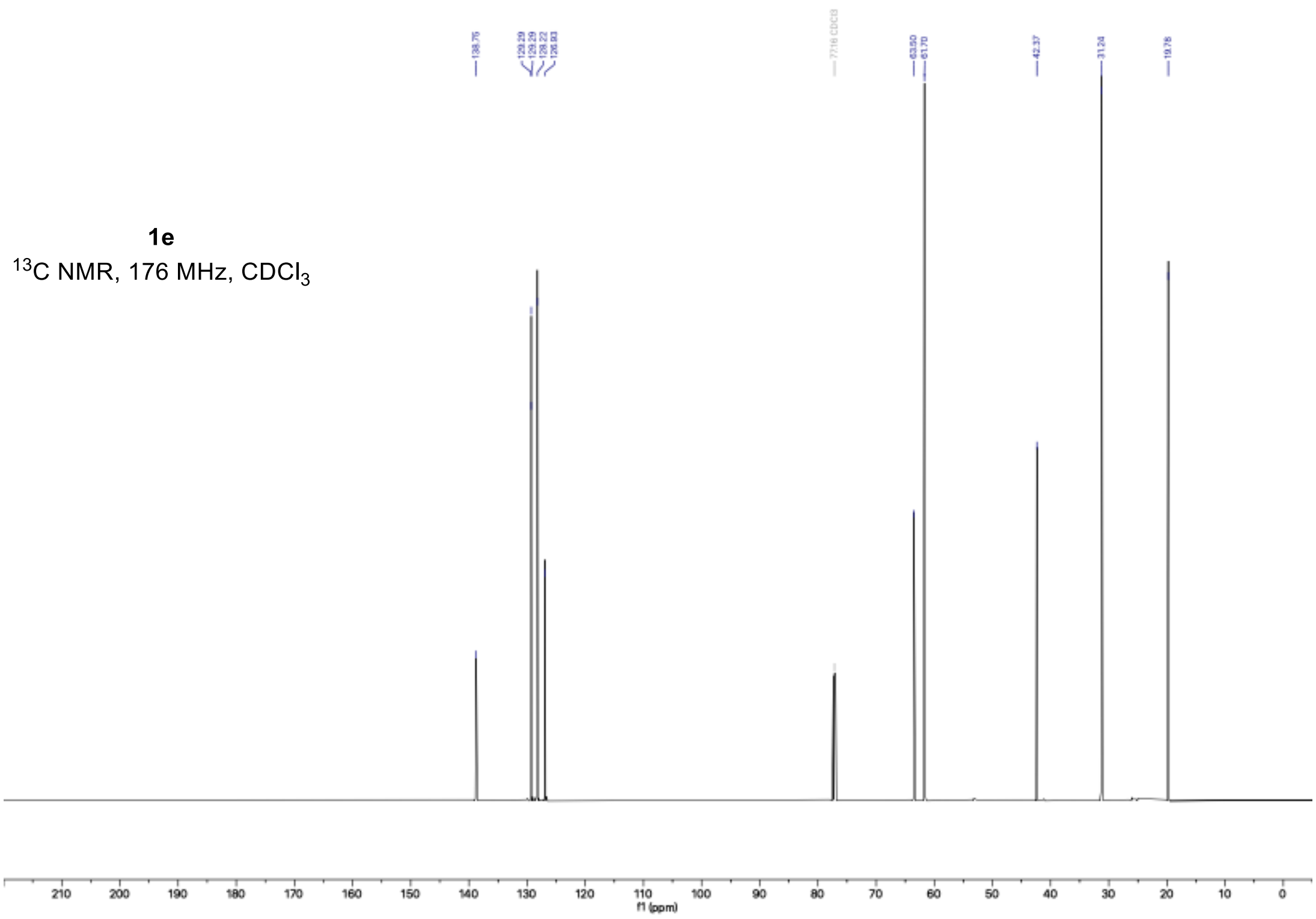

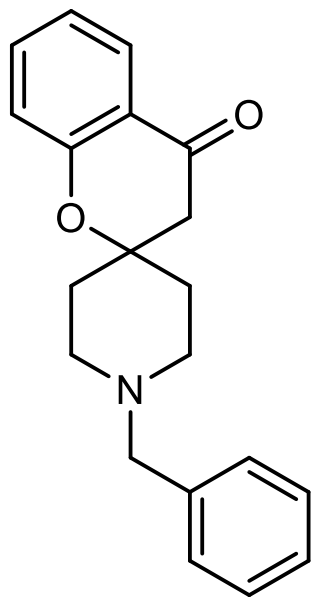

1i

^{1}H NMR, 700 MHz, $CDCl_3$

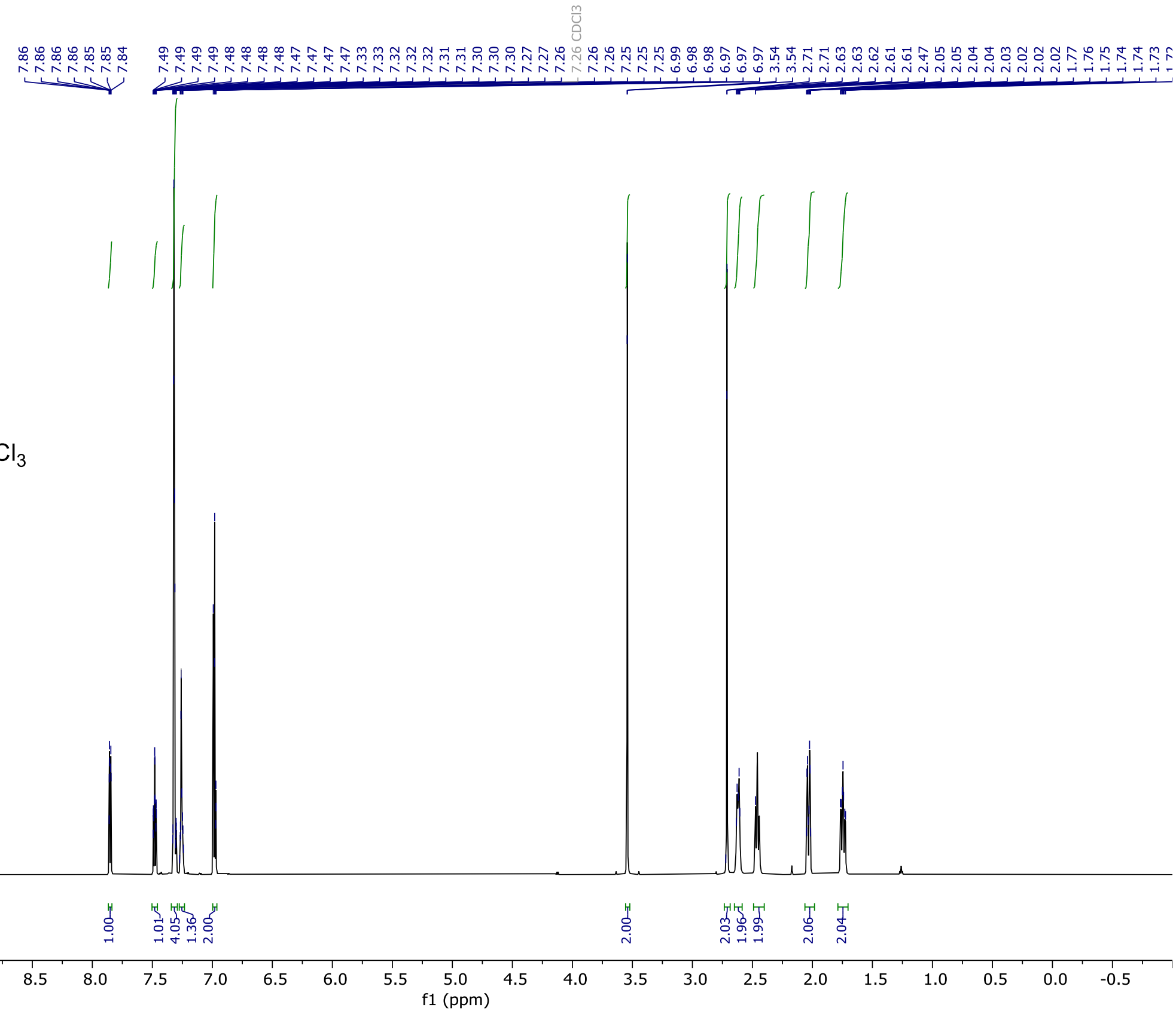

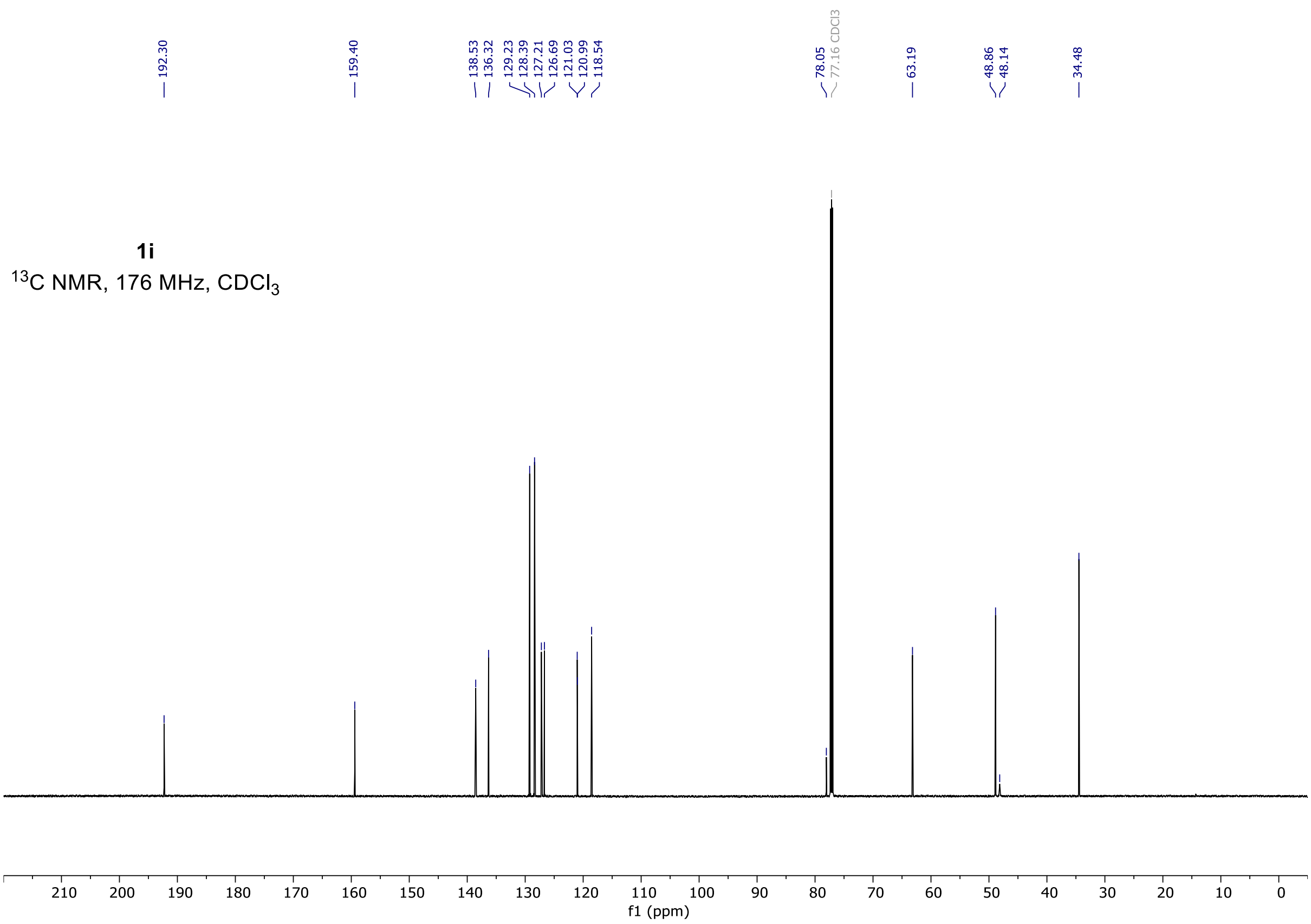

1i

^{13}C NMR, 176 MHz, $CDCl_3$

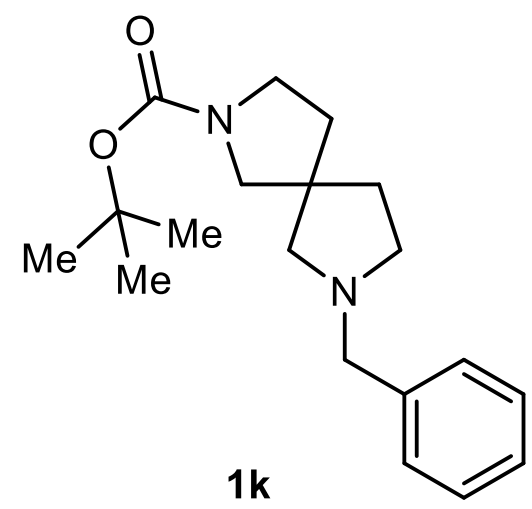

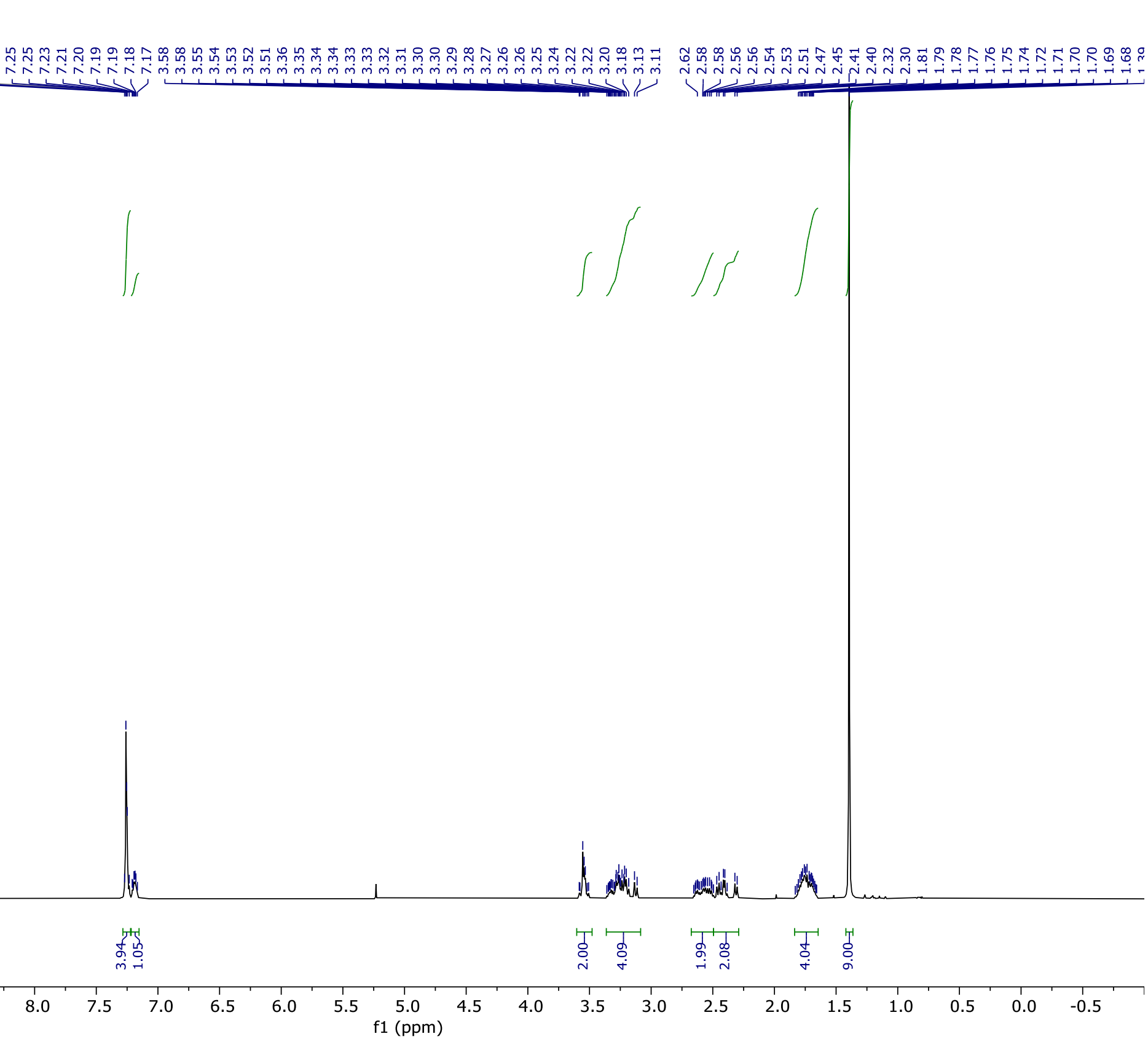

1k

^{1}H NMR, 500 MHz, $CDCl_3$

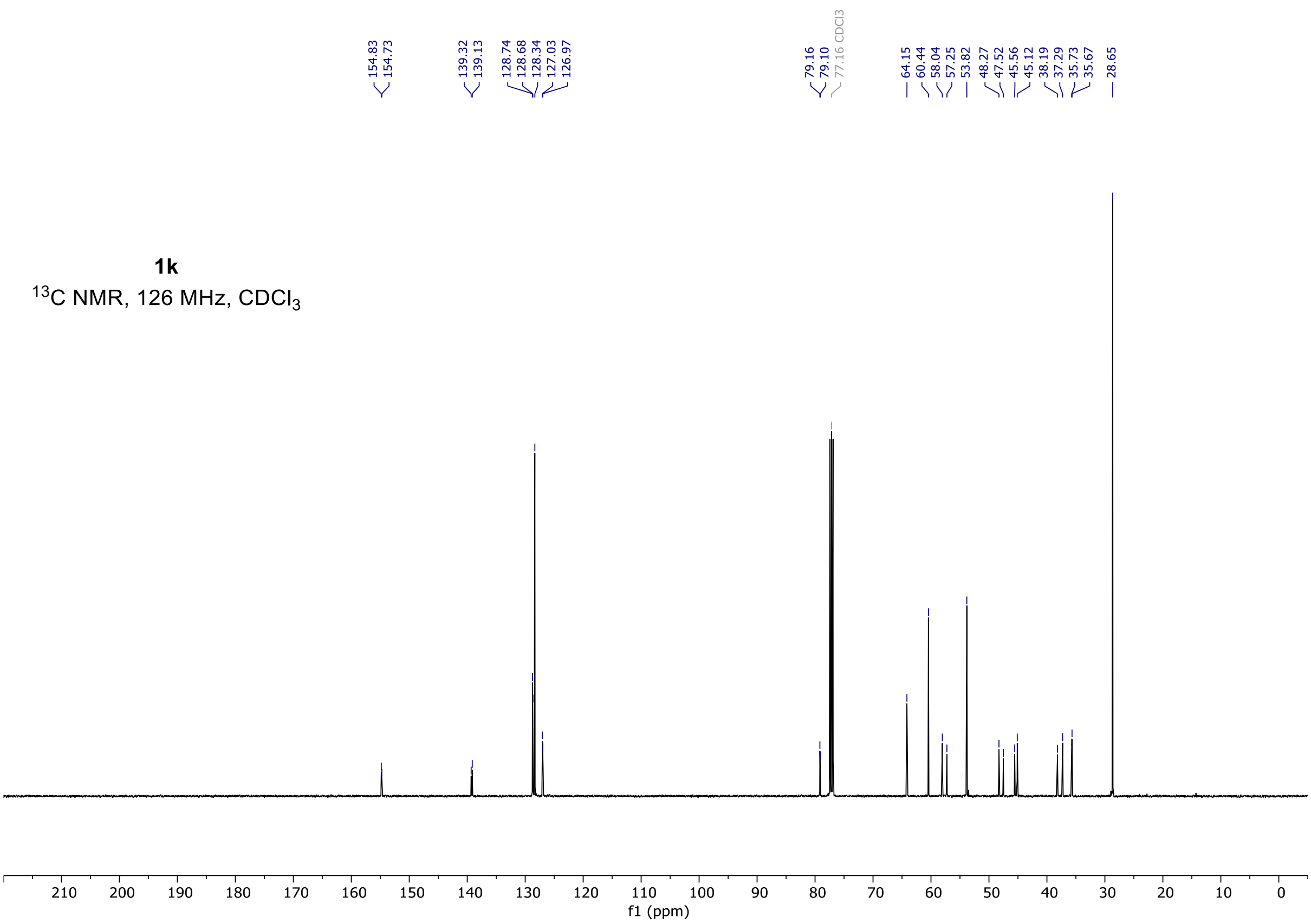

1k

^{13}C NMR, 126 MHz, $CDCl_3$

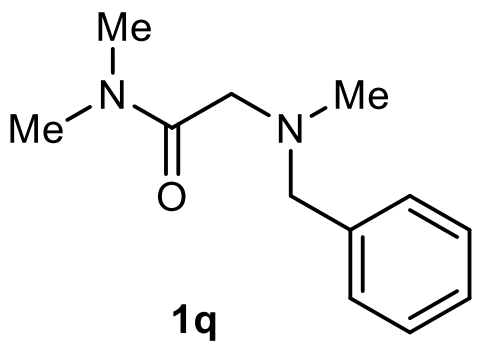

1q

^{1}H NMR, 700 MHz, $CDCl_3$

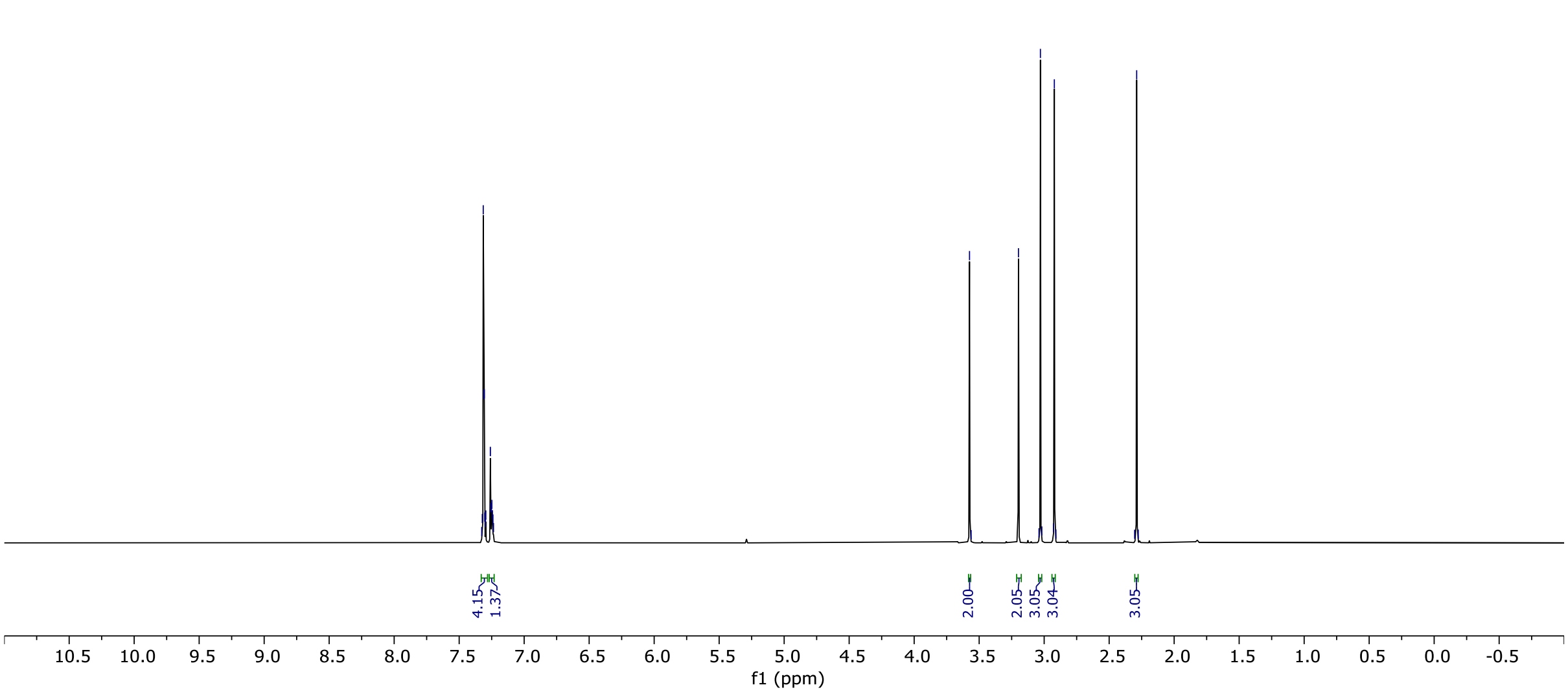

1q

^{13}C NMR, 176 MHz, $CDCl_3$

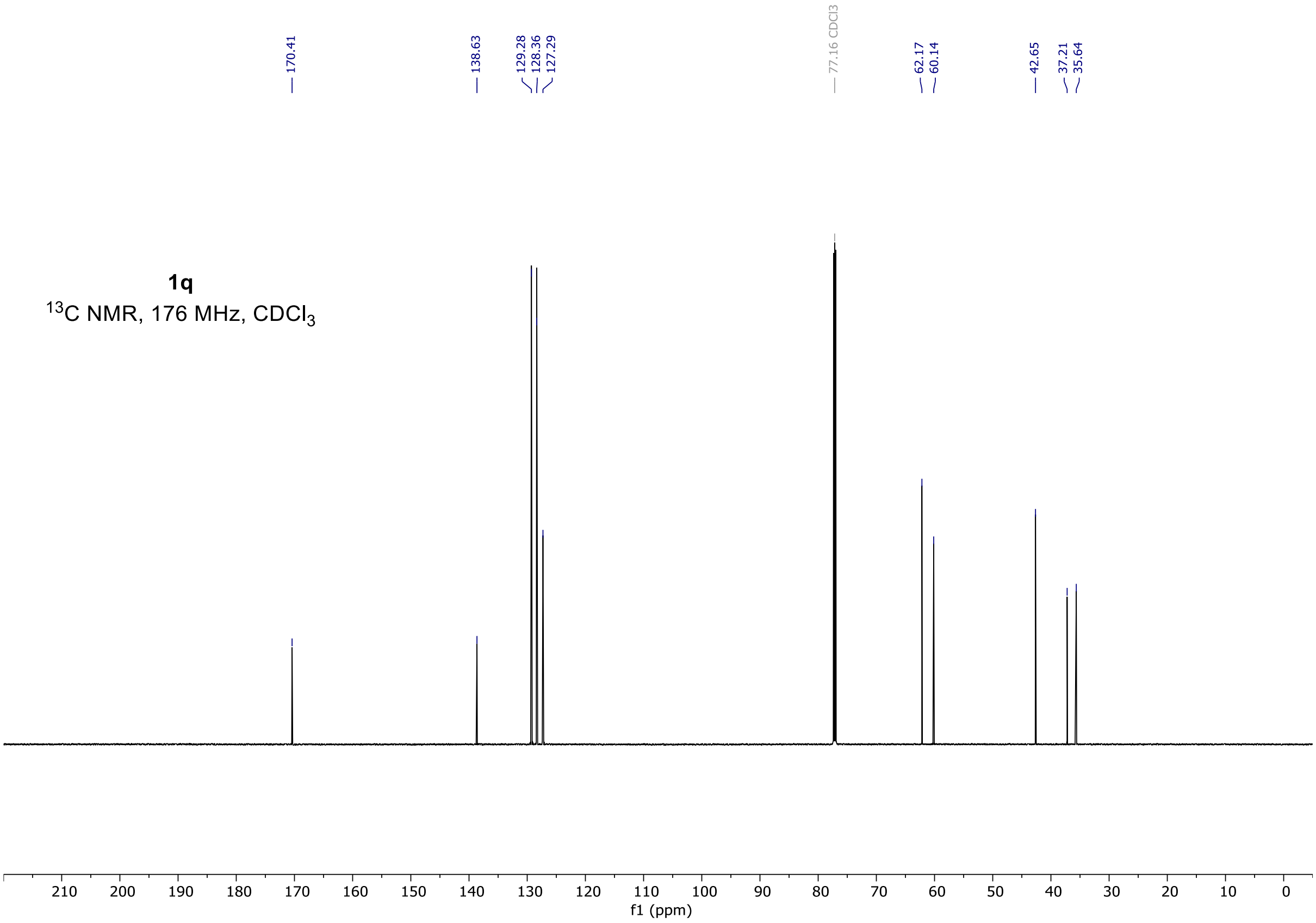

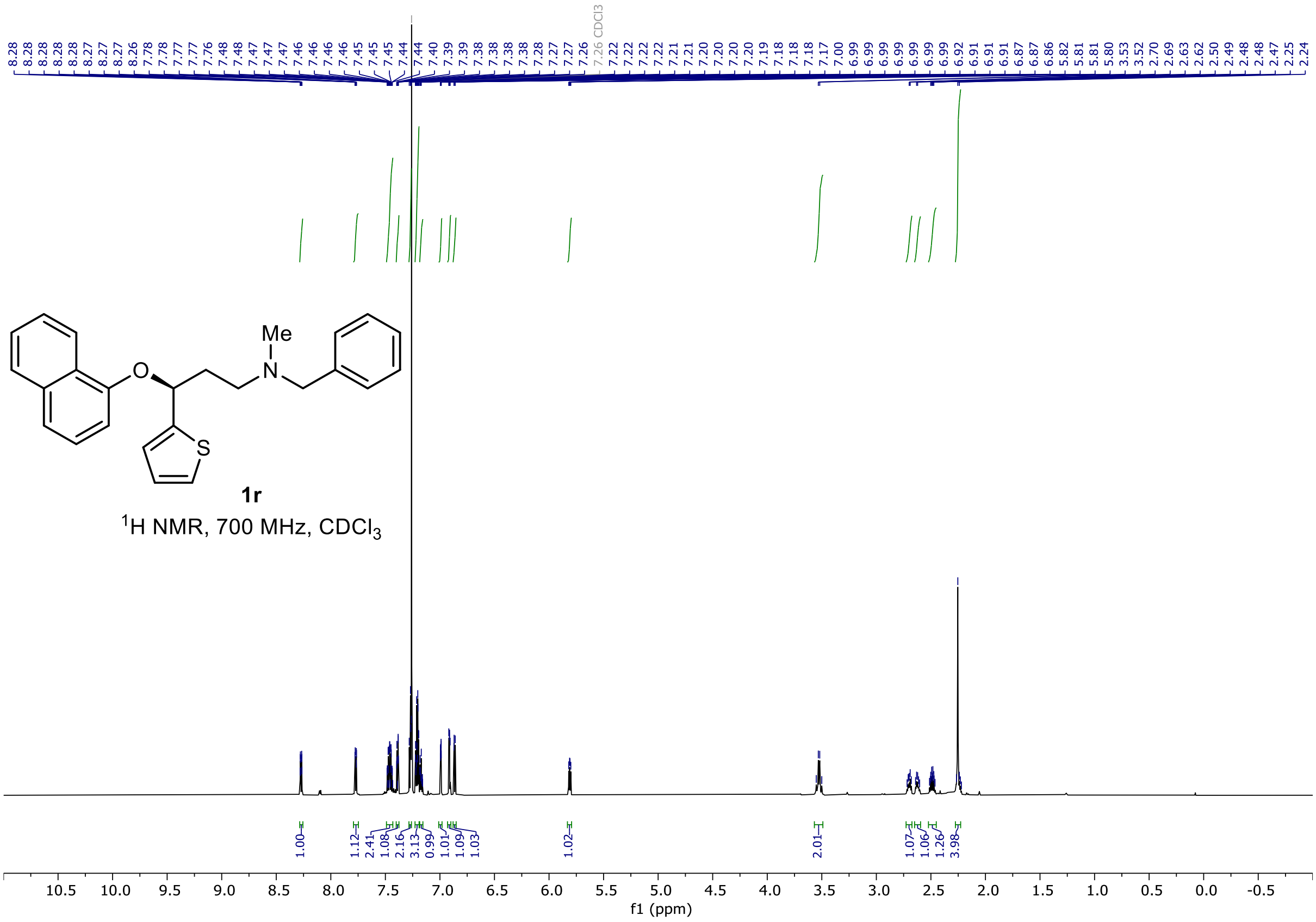
Me
N
O
S
1r
^{1}H NMR, 700 MHz, CDCl$_3$
f1 (ppm)

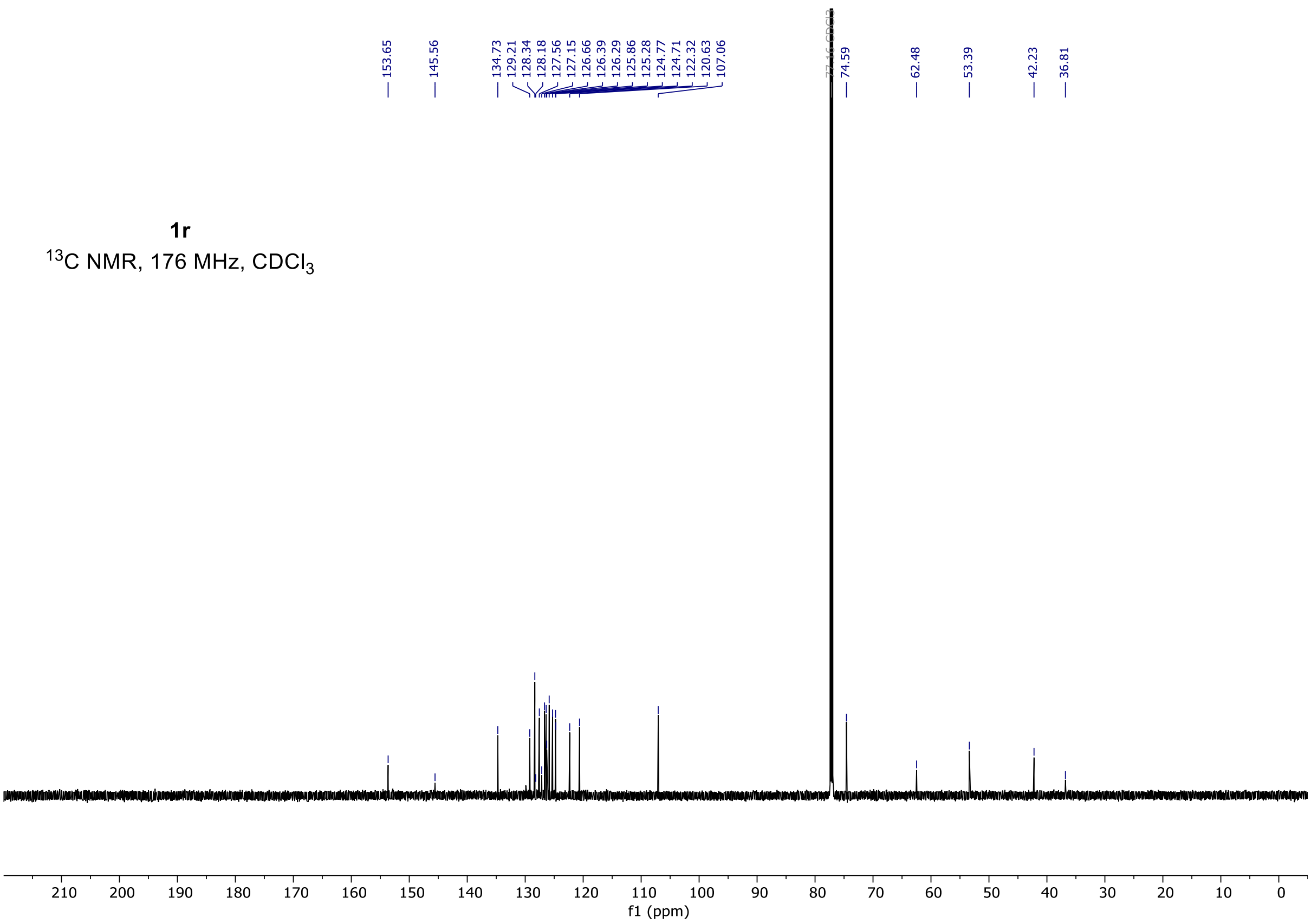
153.65
145.56
134.73
129.21
128.34
128.18
127.56
127.15
126.66
126.39
126.29
125.86
125.28
124.77
124.71
122.32
120.63
107.06
74.59
62.48
53.39
42.23
36.81
1r
^{13}C NMR, 176 MHz, CDCl$_3$
f1 (ppm)

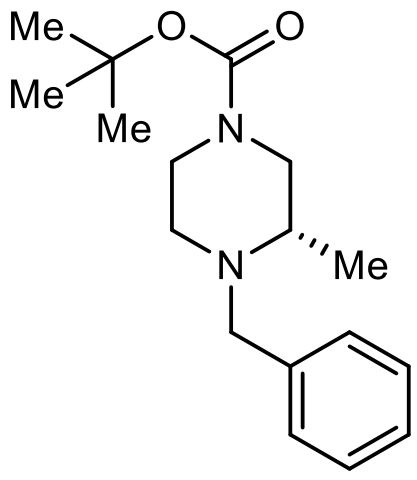

1w

^{1}H NMR, 700 MHz, $CDCl_3$

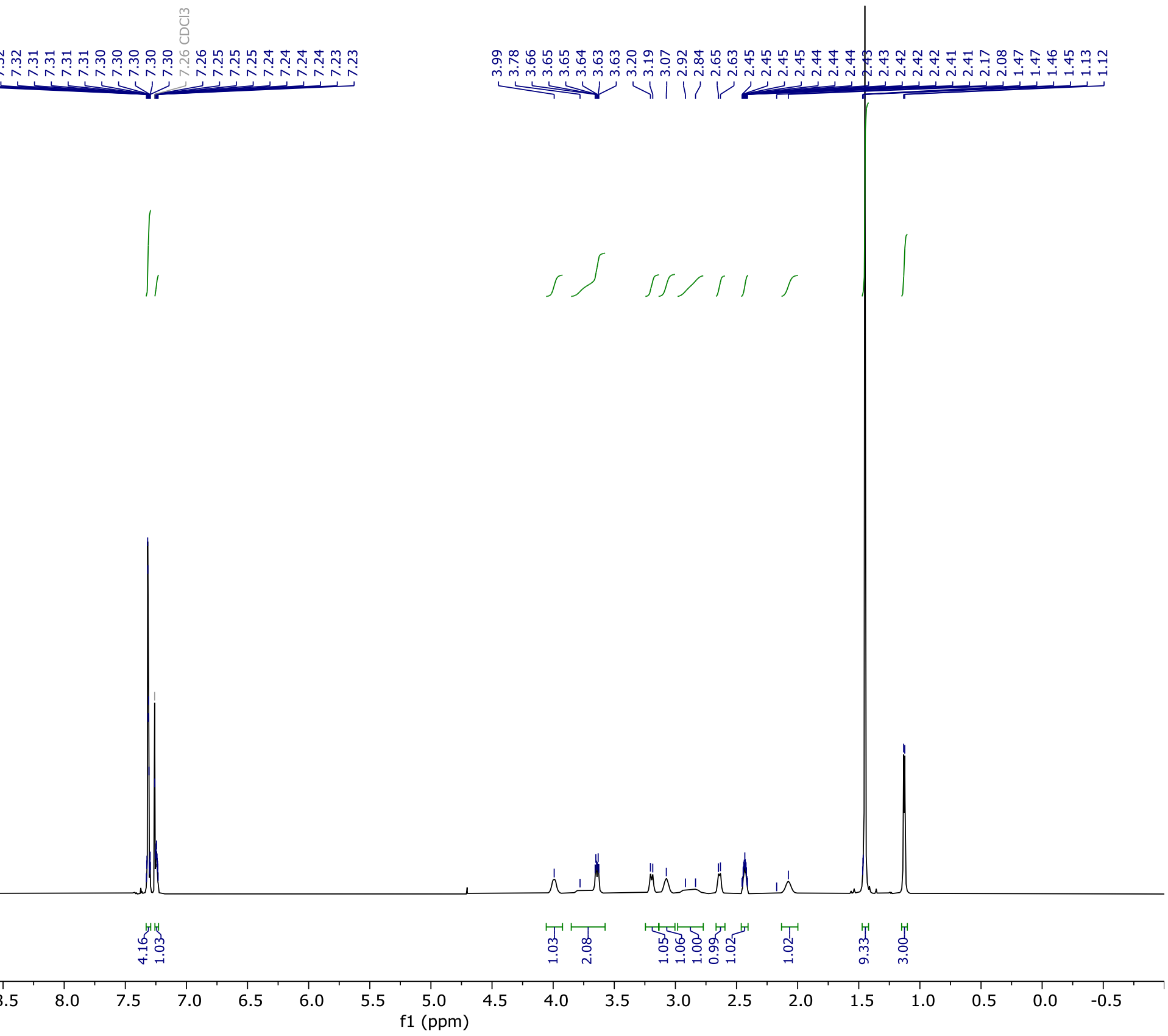

1w

^{13}C NMR, 176 MHz, $CDCl_3$

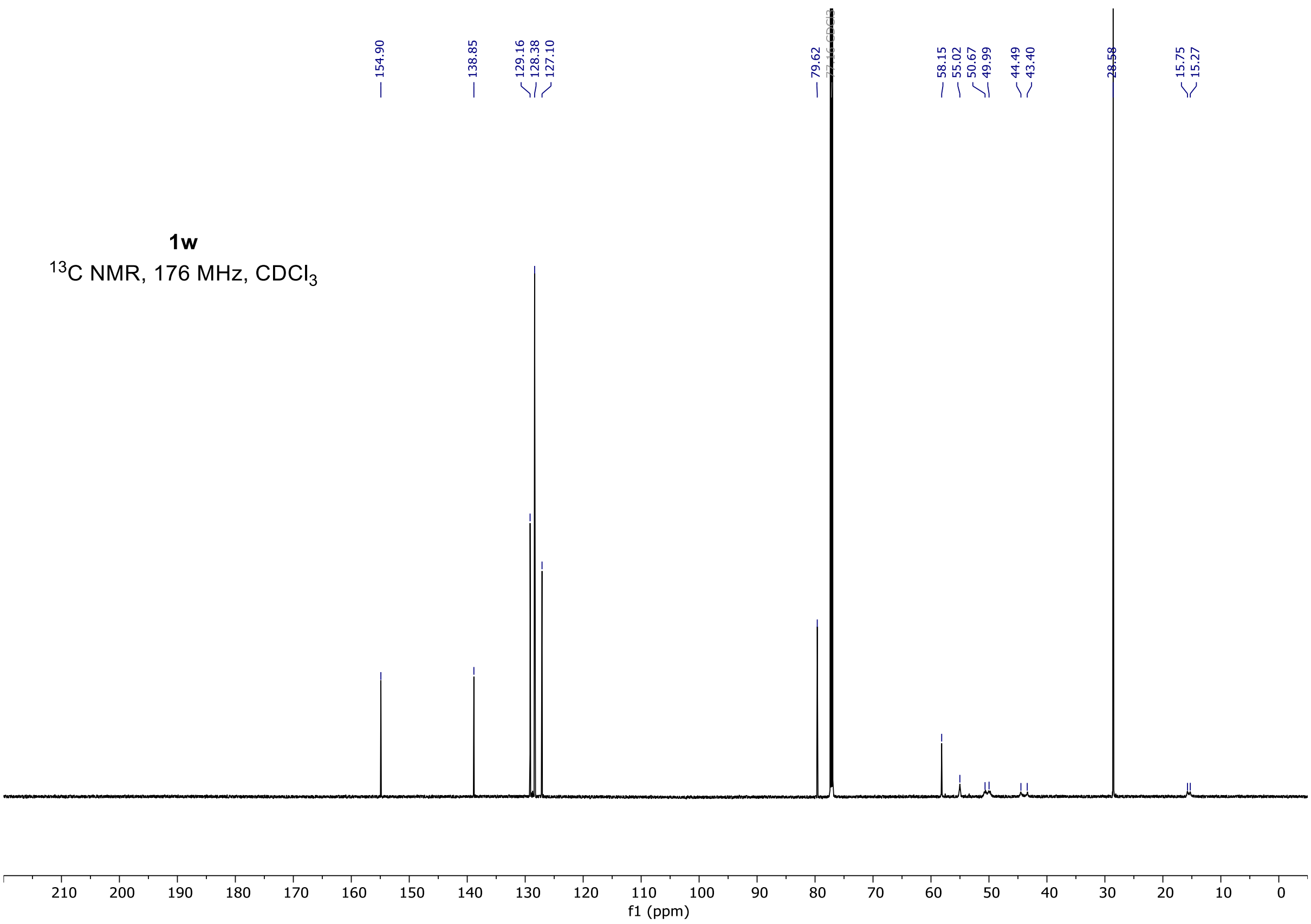

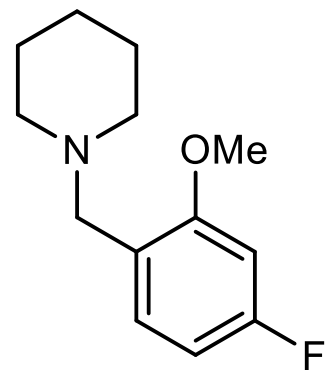

1ae

^{1}H NMR, 500 MHz, $CDCl_3$

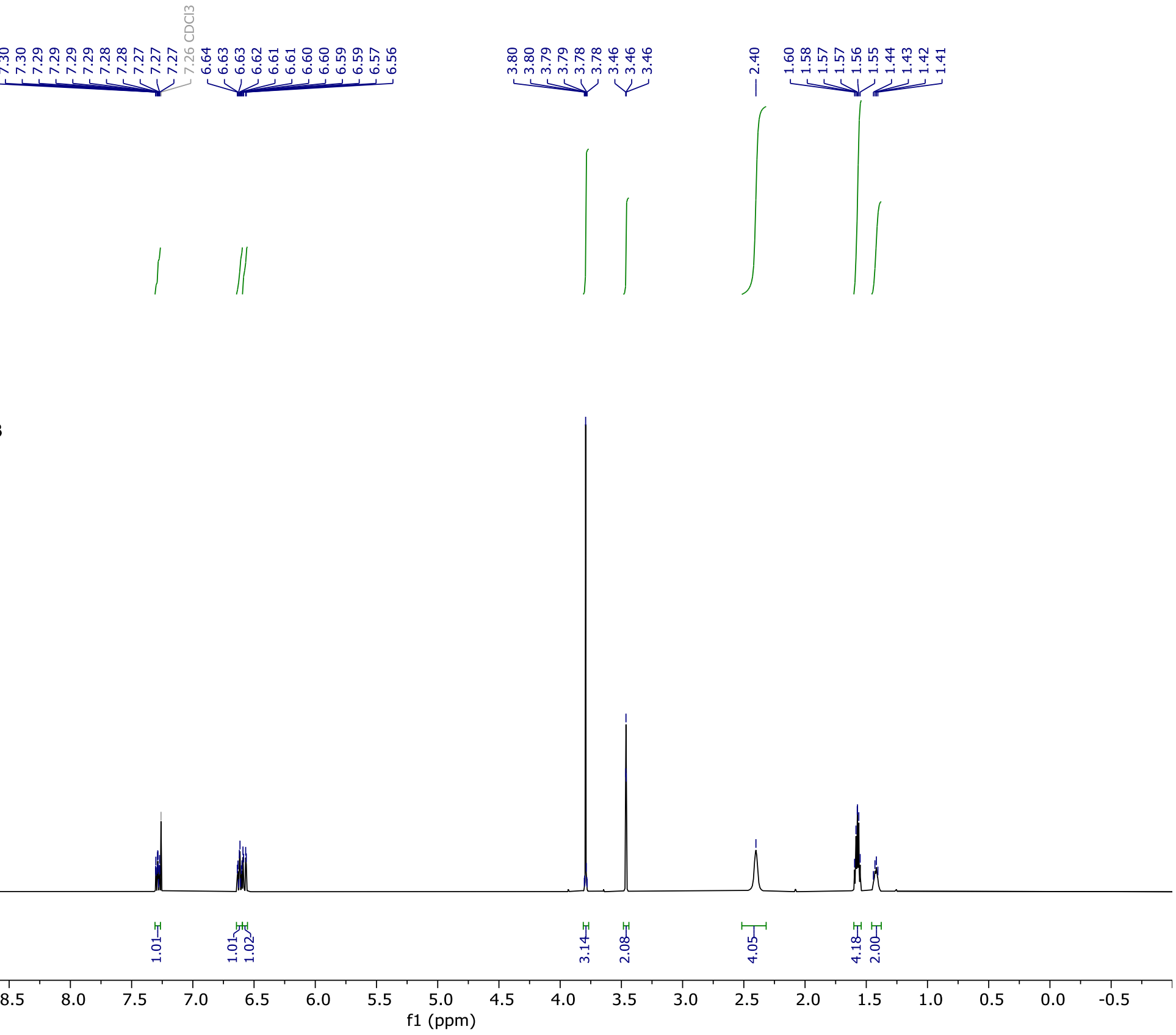

1ae

^{13}C NMR, 126 MHz, $CDCl_3$

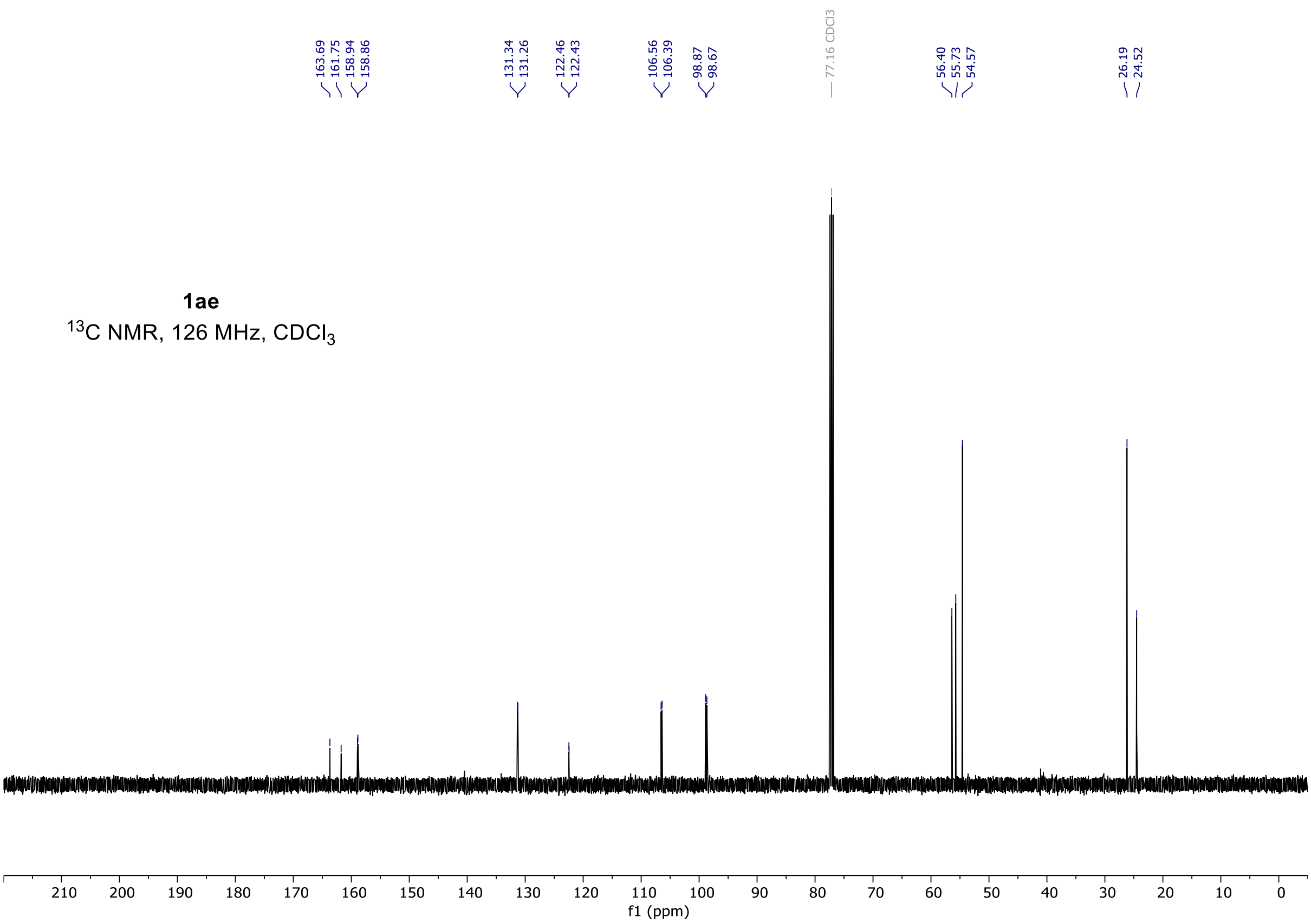

1ae

^{19}F NMR, 376 MHz, $CDCl_3$

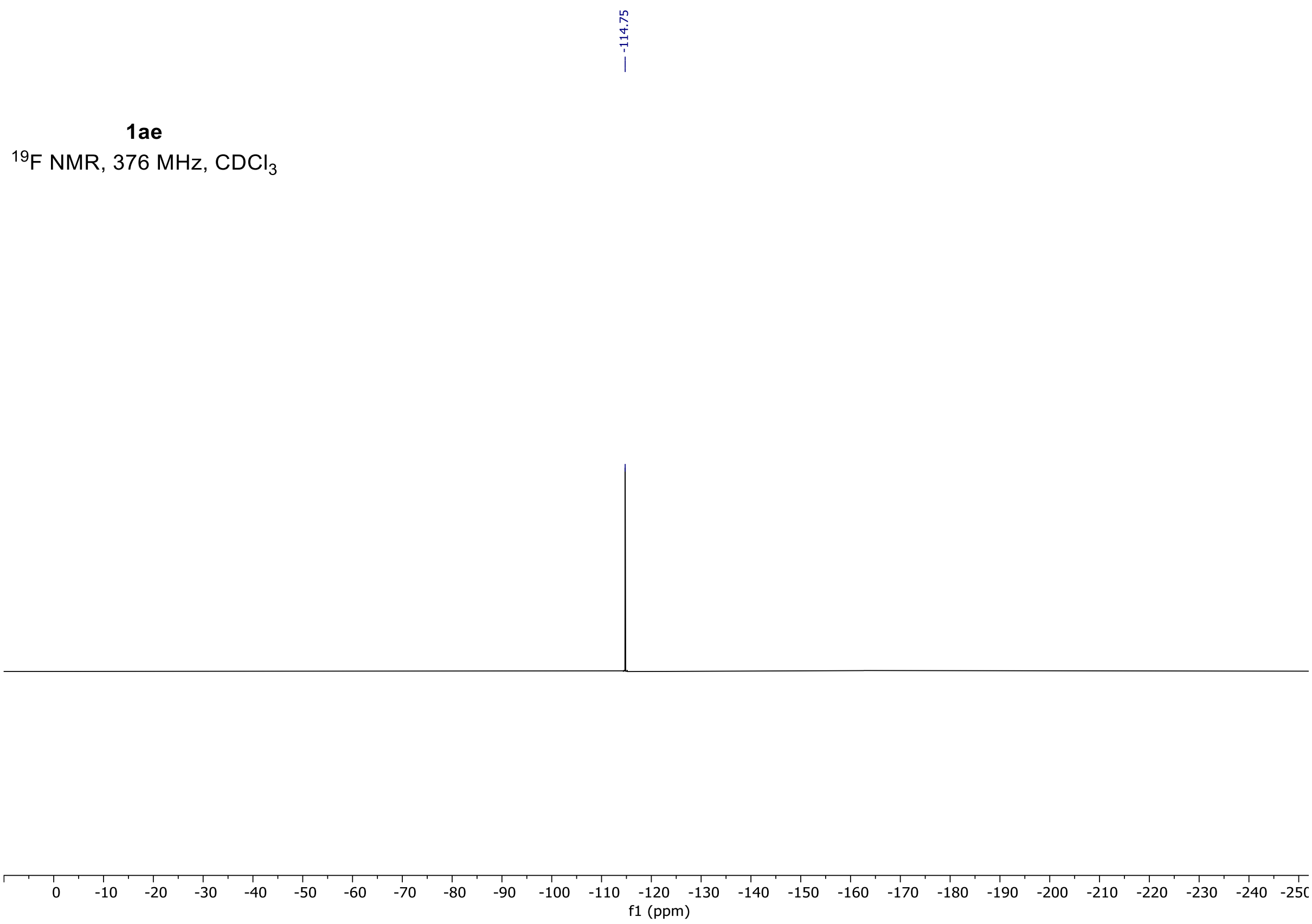

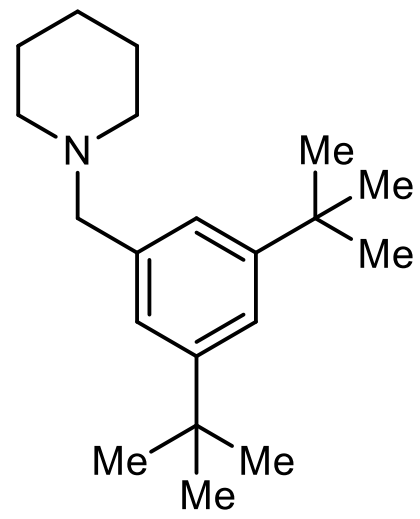

1af
^{1}H NMR, 500 MHz, $CDCl_3$

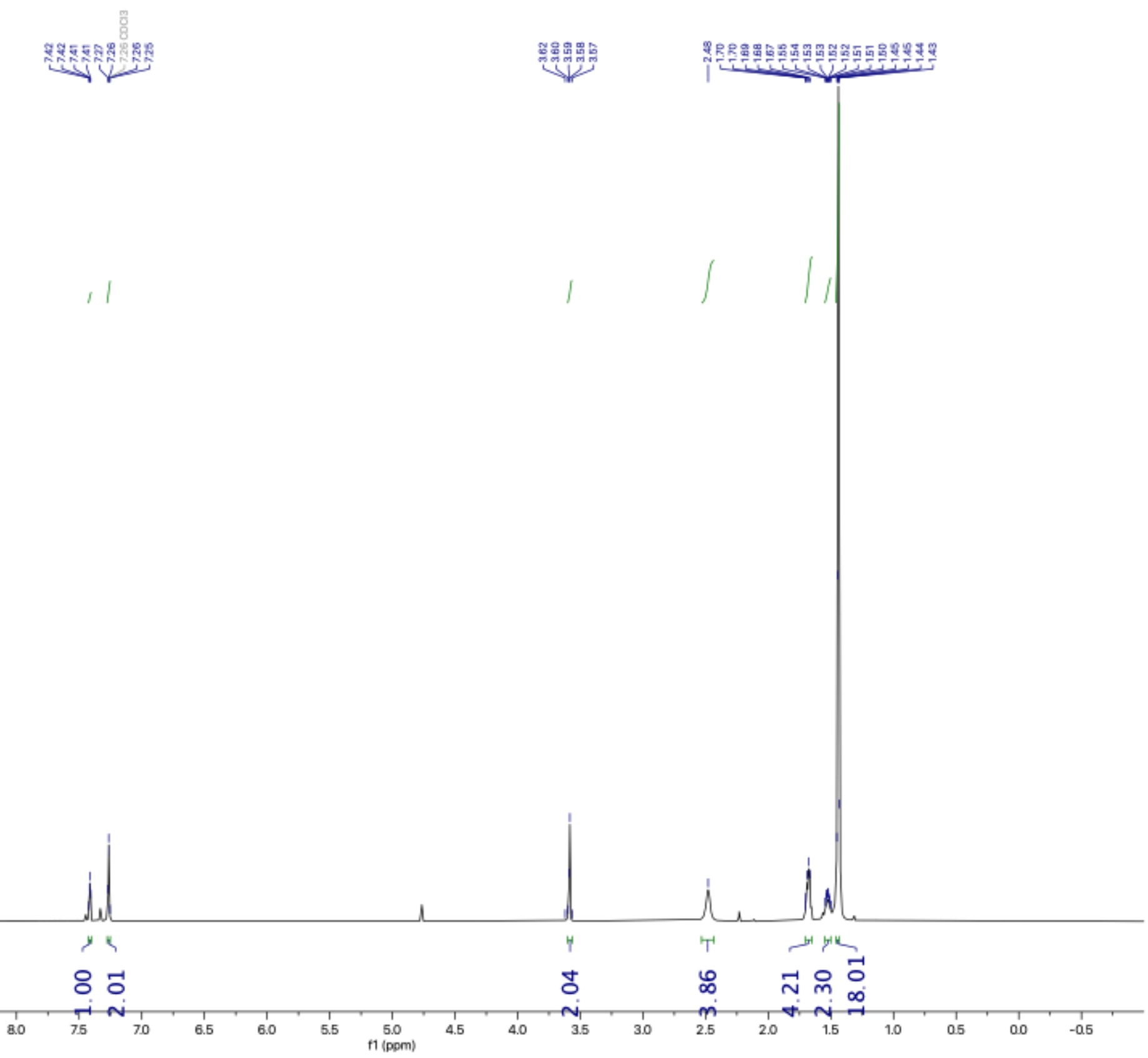

1af
^{13}C NMR, 126 MHz, $CDCl_3$

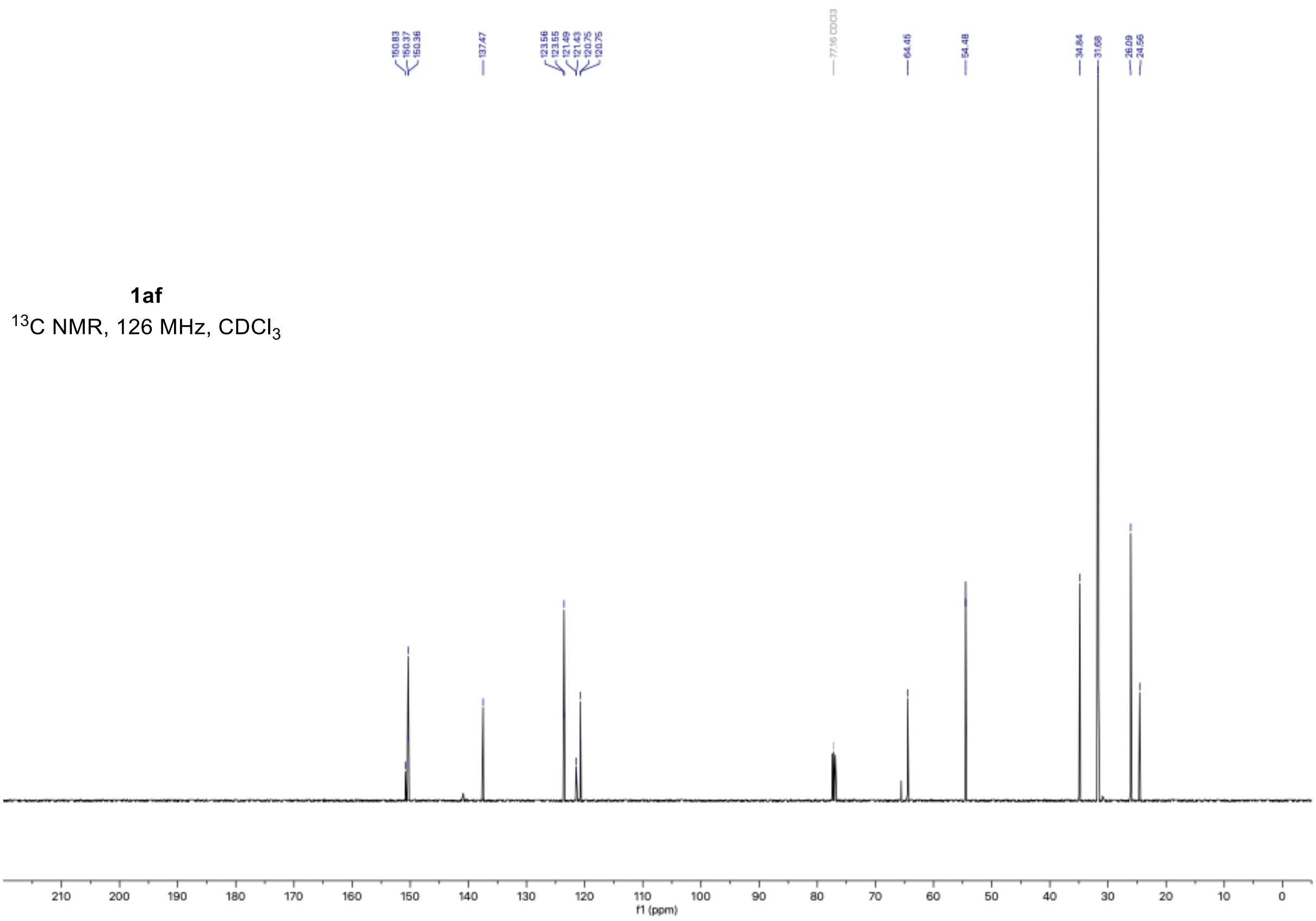

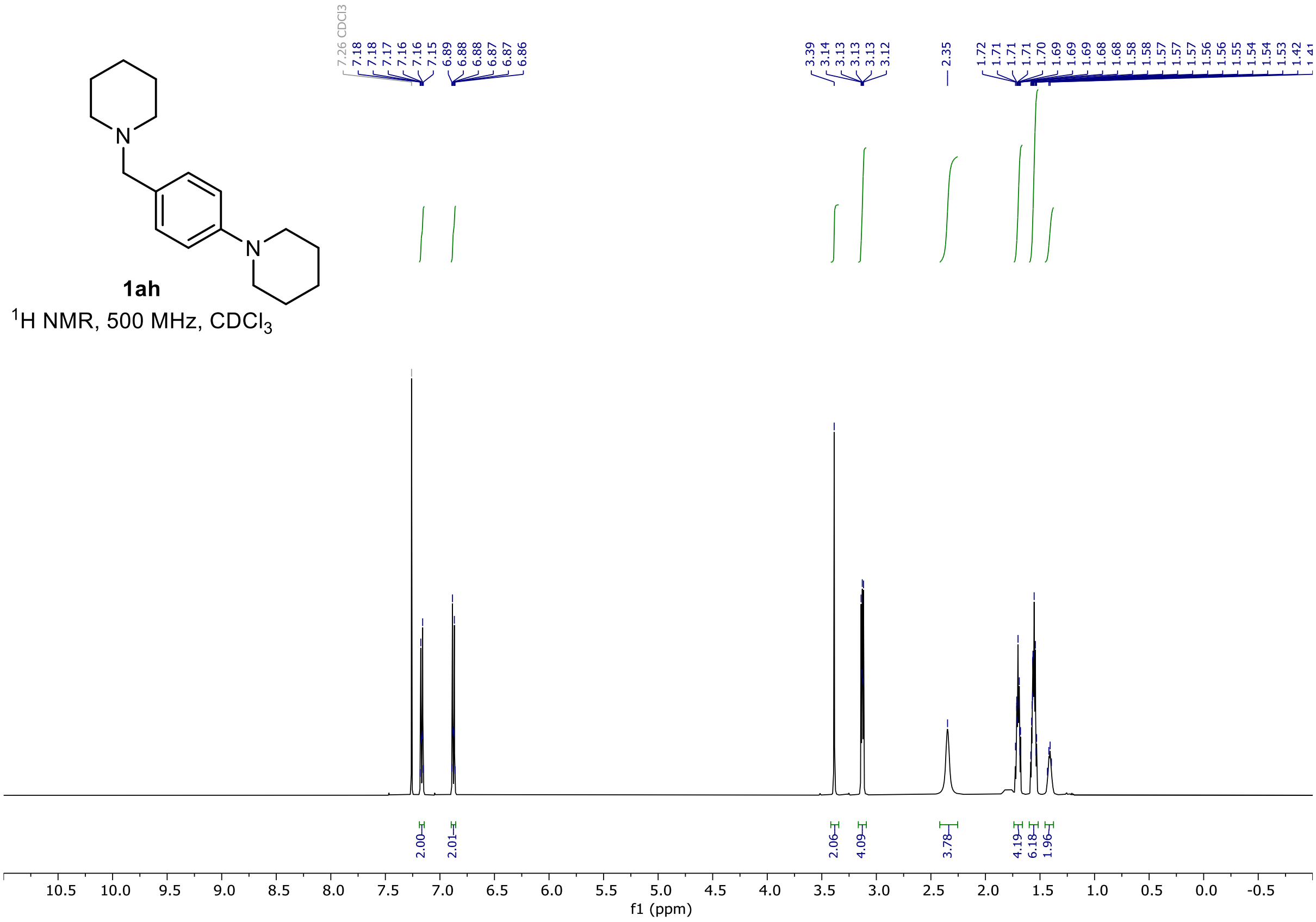

1ah

^{1}H NMR, 500 MHz, $CDCl_3$

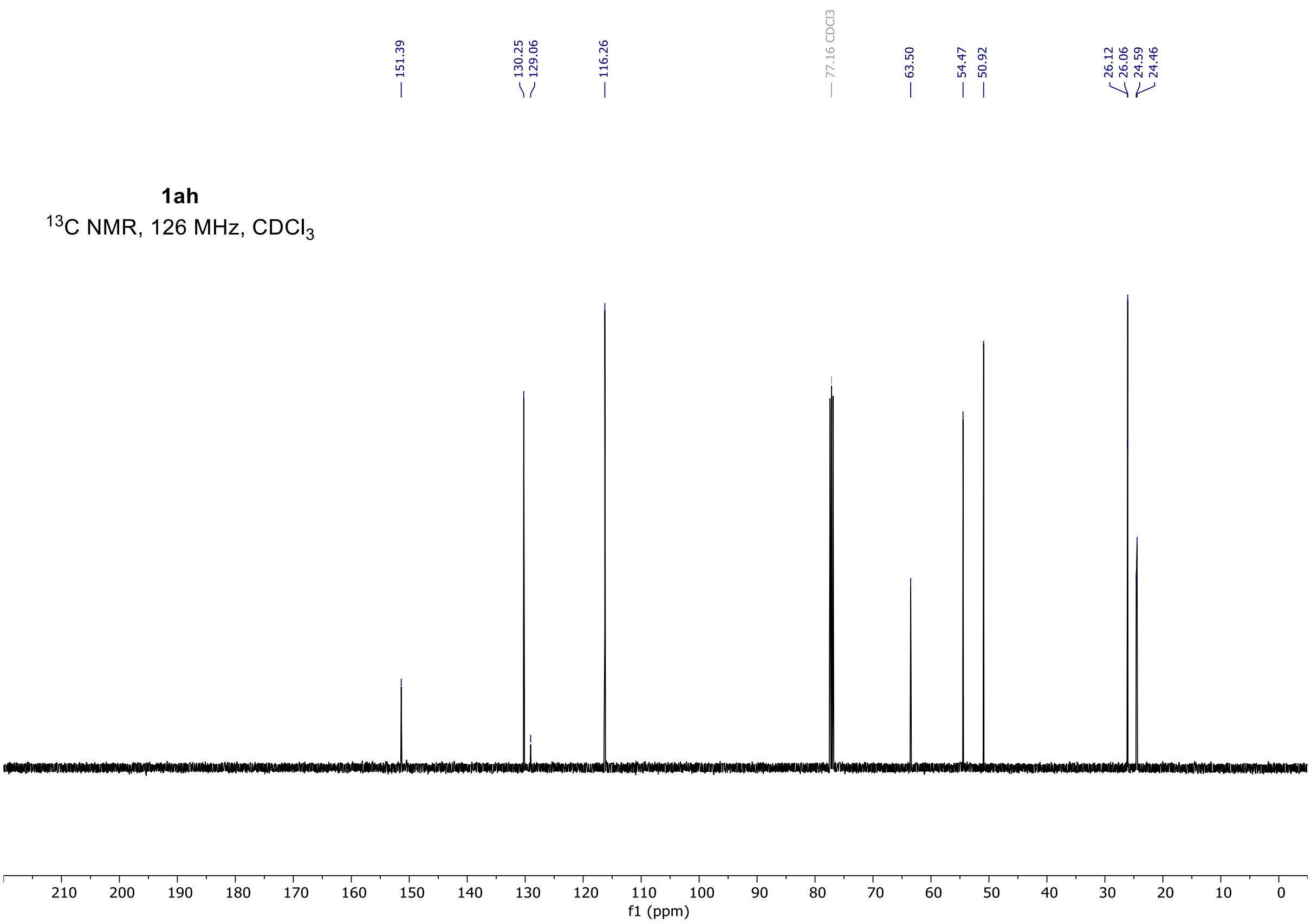

1ah

^{13}C NMR, 126 MHz, $CDCl_3$

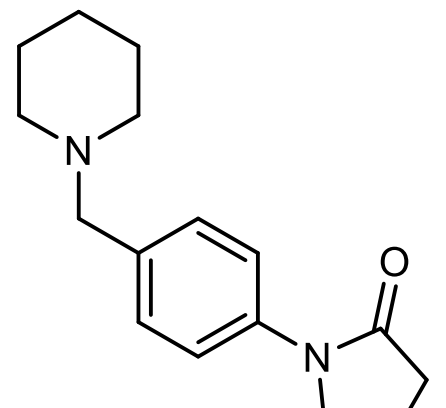

1ai

^{1}H NMR, 500 MHz, $CDCl_3$

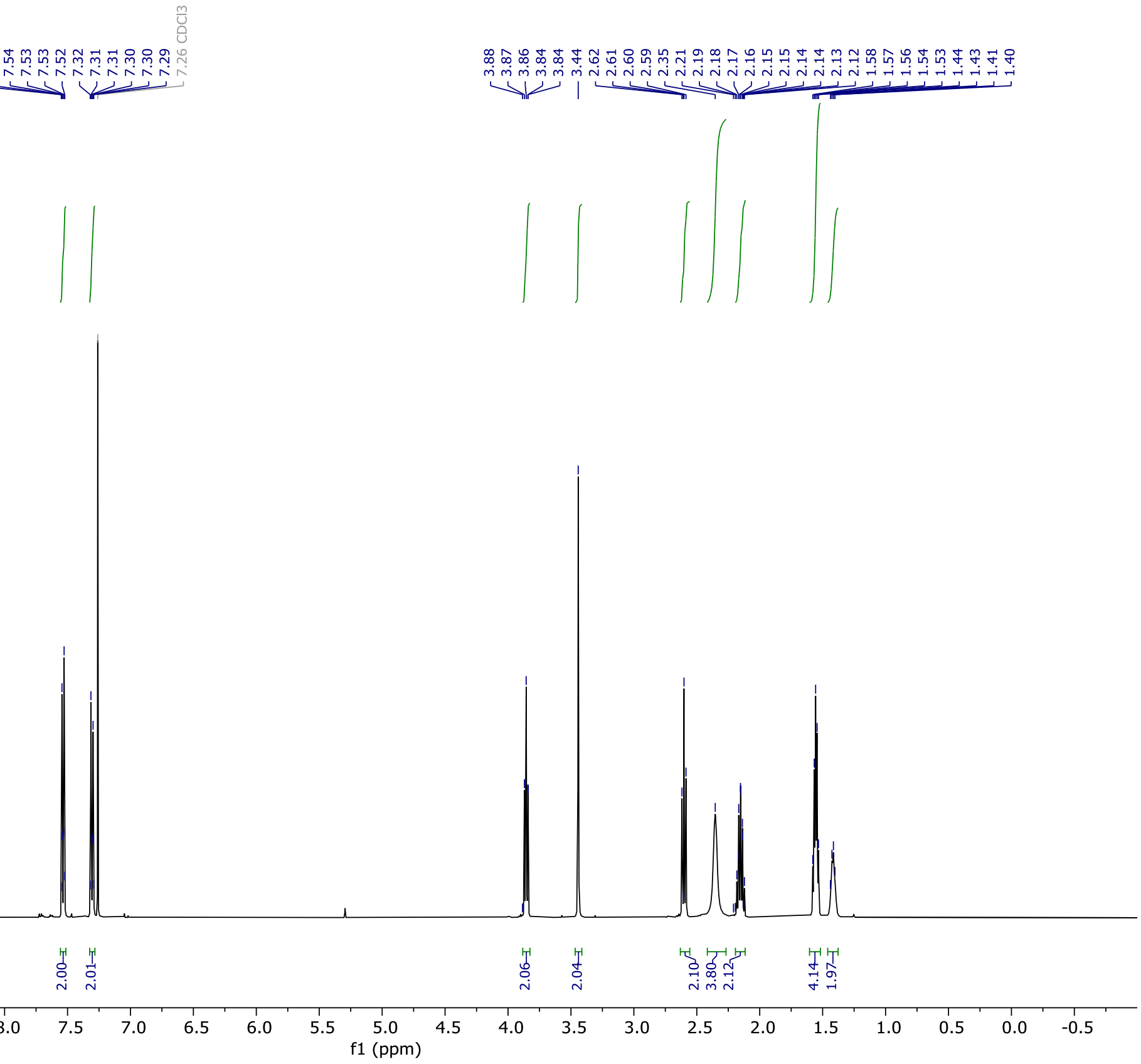

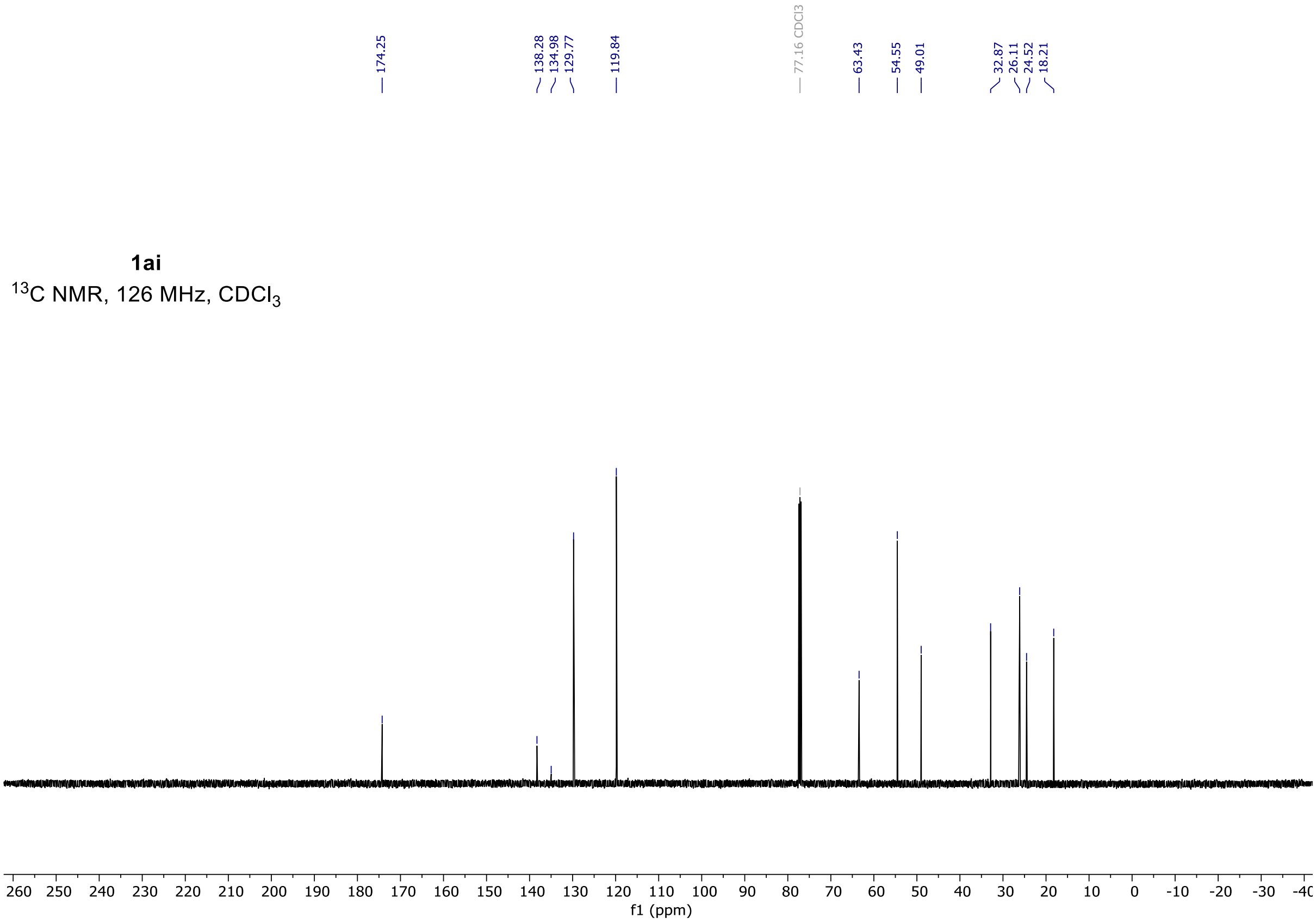

1ai

^{13}C NMR, 126 MHz, $CDCl_3$

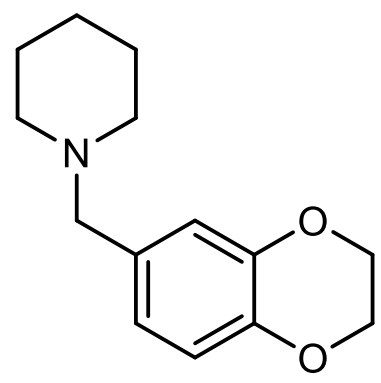

1aj

^{1}H NMR, 500 MHz, $CDCl_3$

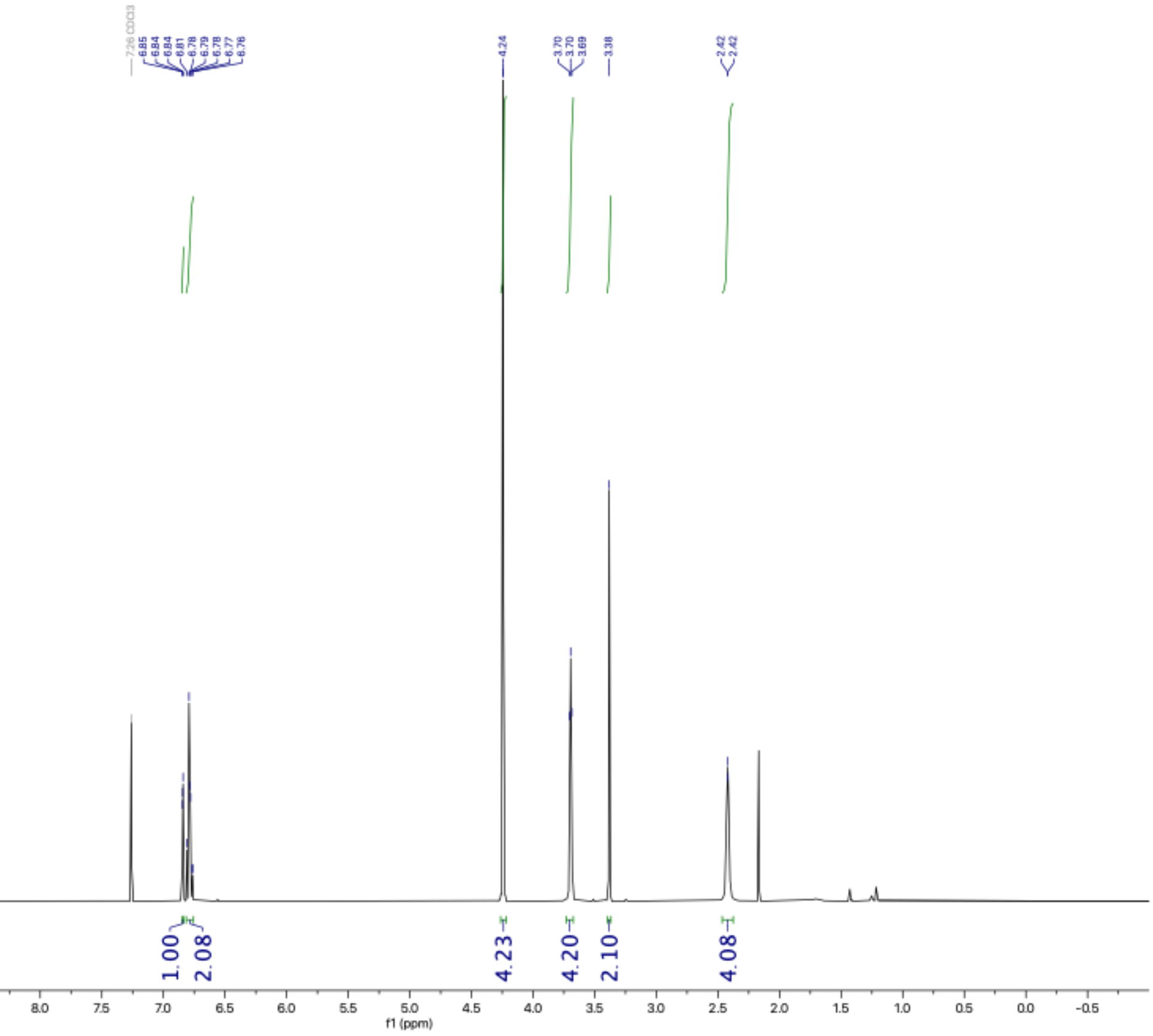

1aj

^{13}C NMR, 126 MHz, $CDCl_3$

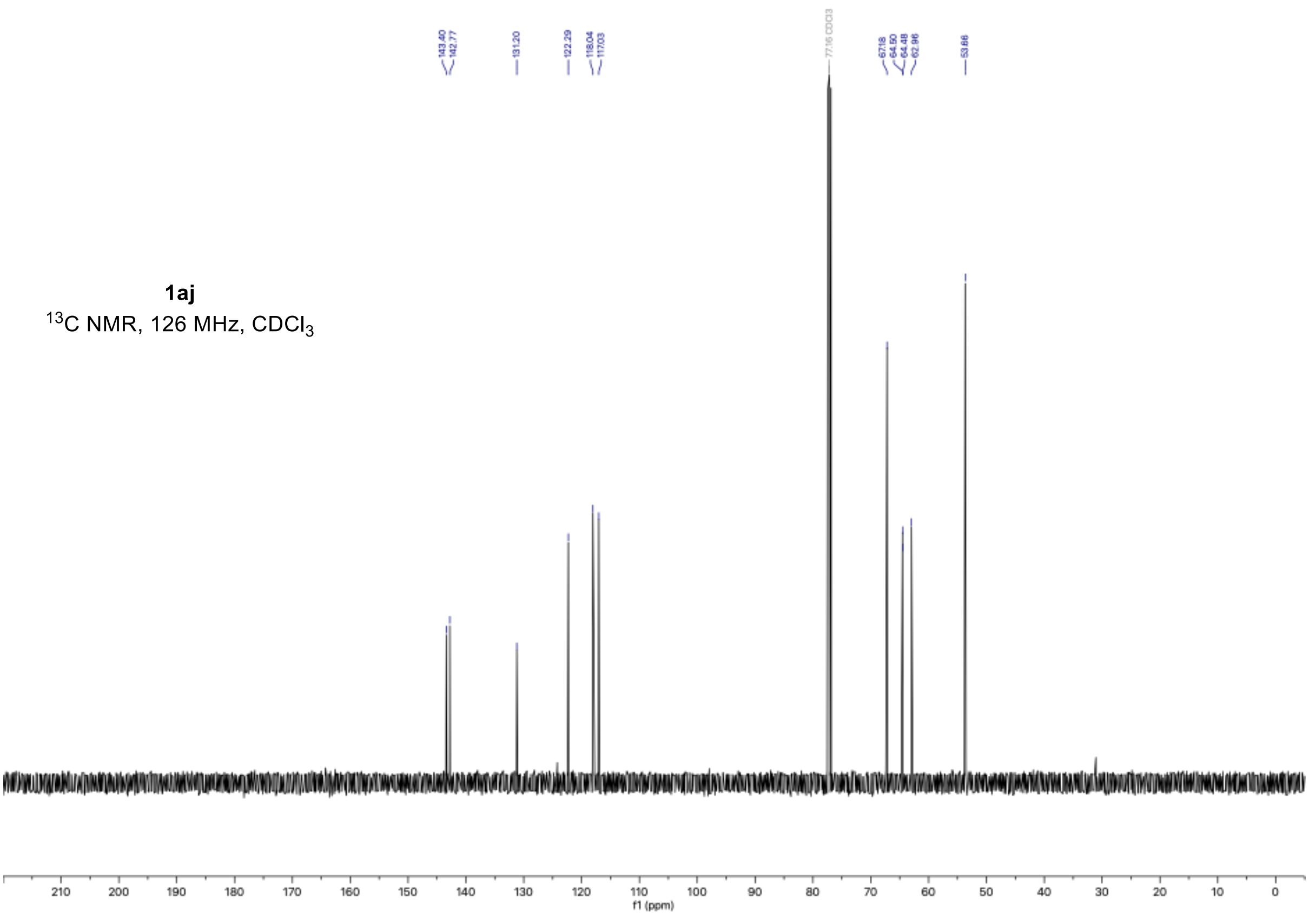

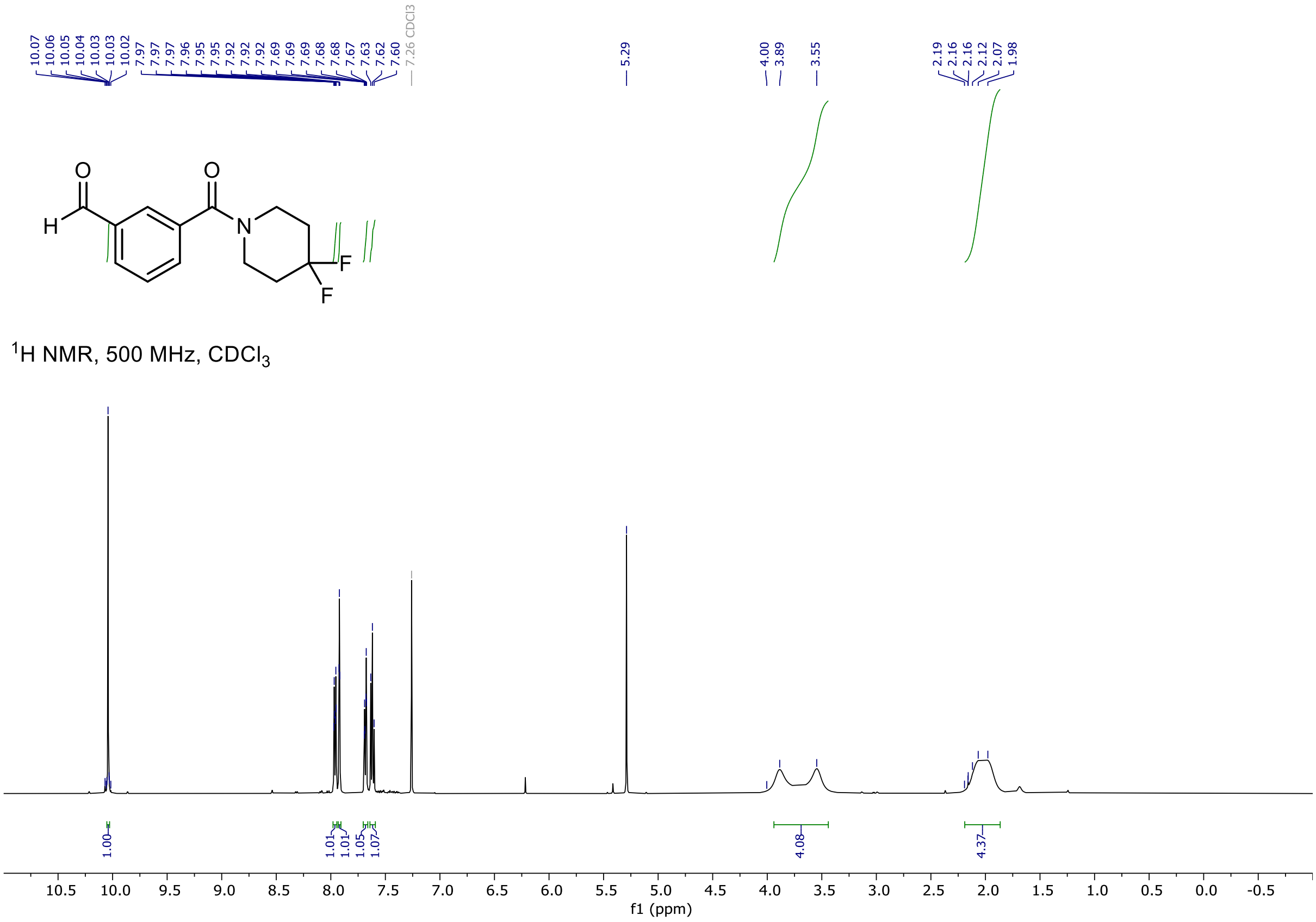

^{1}H NMR, 500 MHz, $CDCl_3$

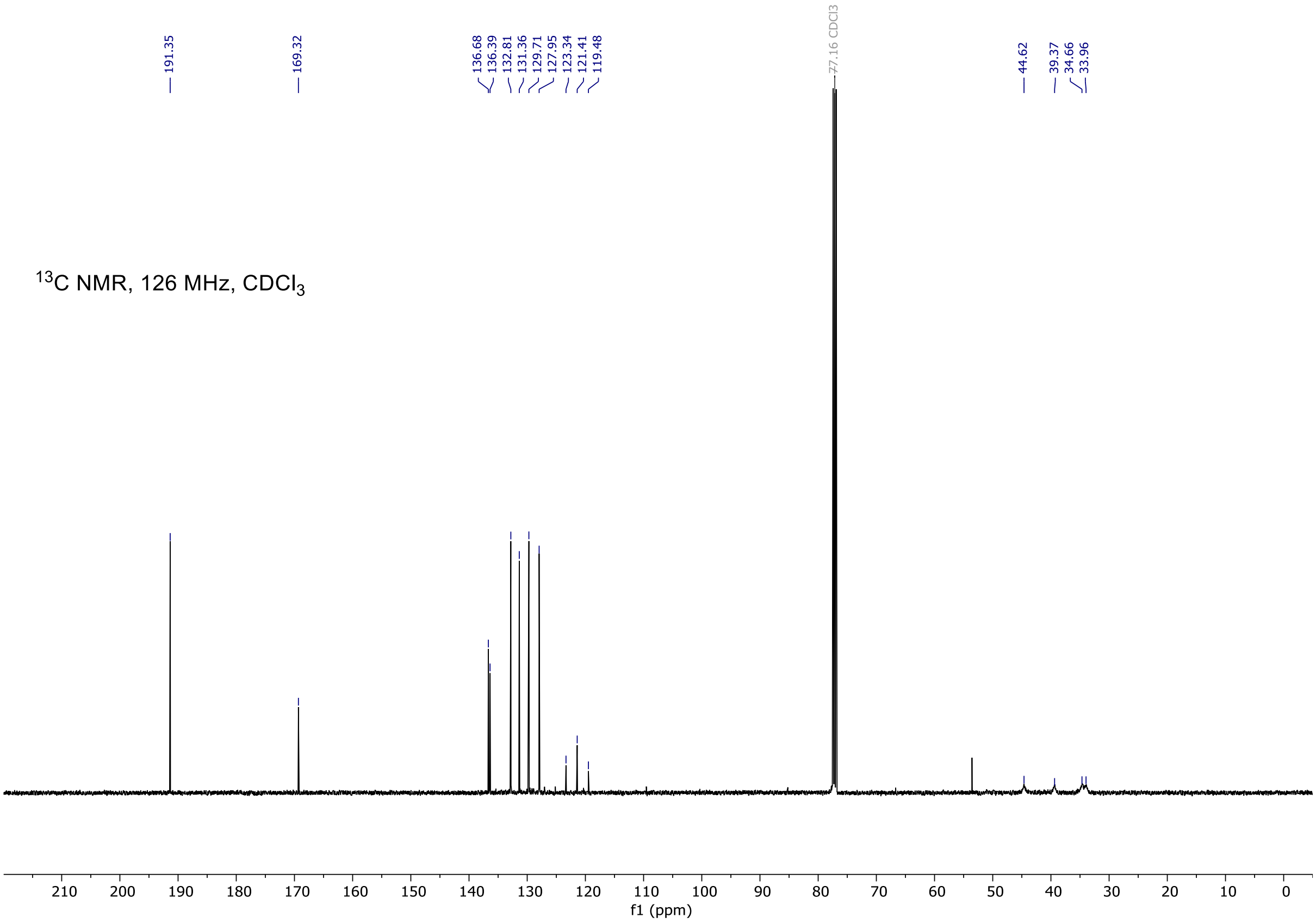

^{13}C NMR, 126 MHz, $CDCl_3$

^{19}F NMR, 376 MHz, $CDCl_3$

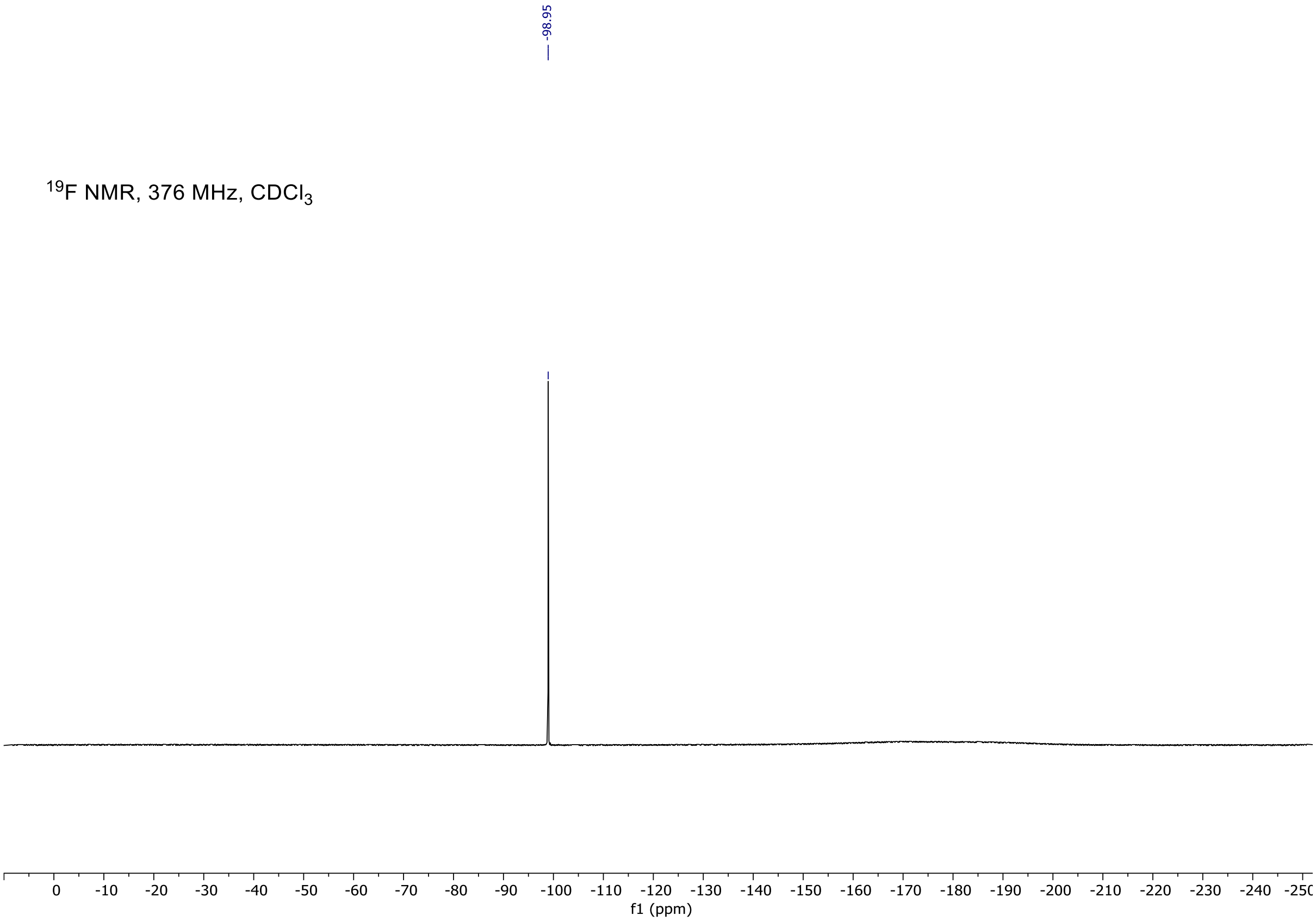

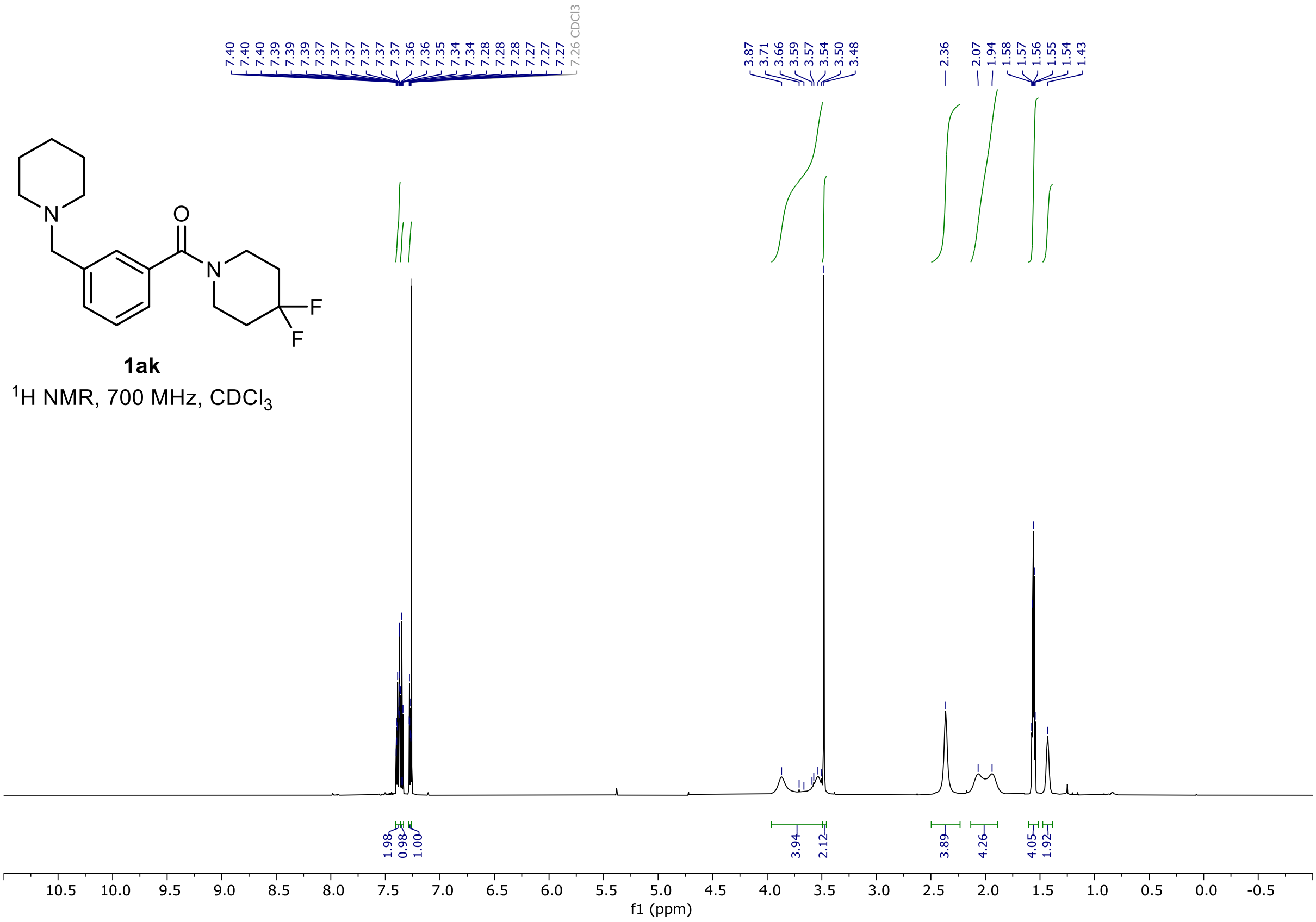

1ak

^{1}H NMR, 700 MHz, $CDCl_3$

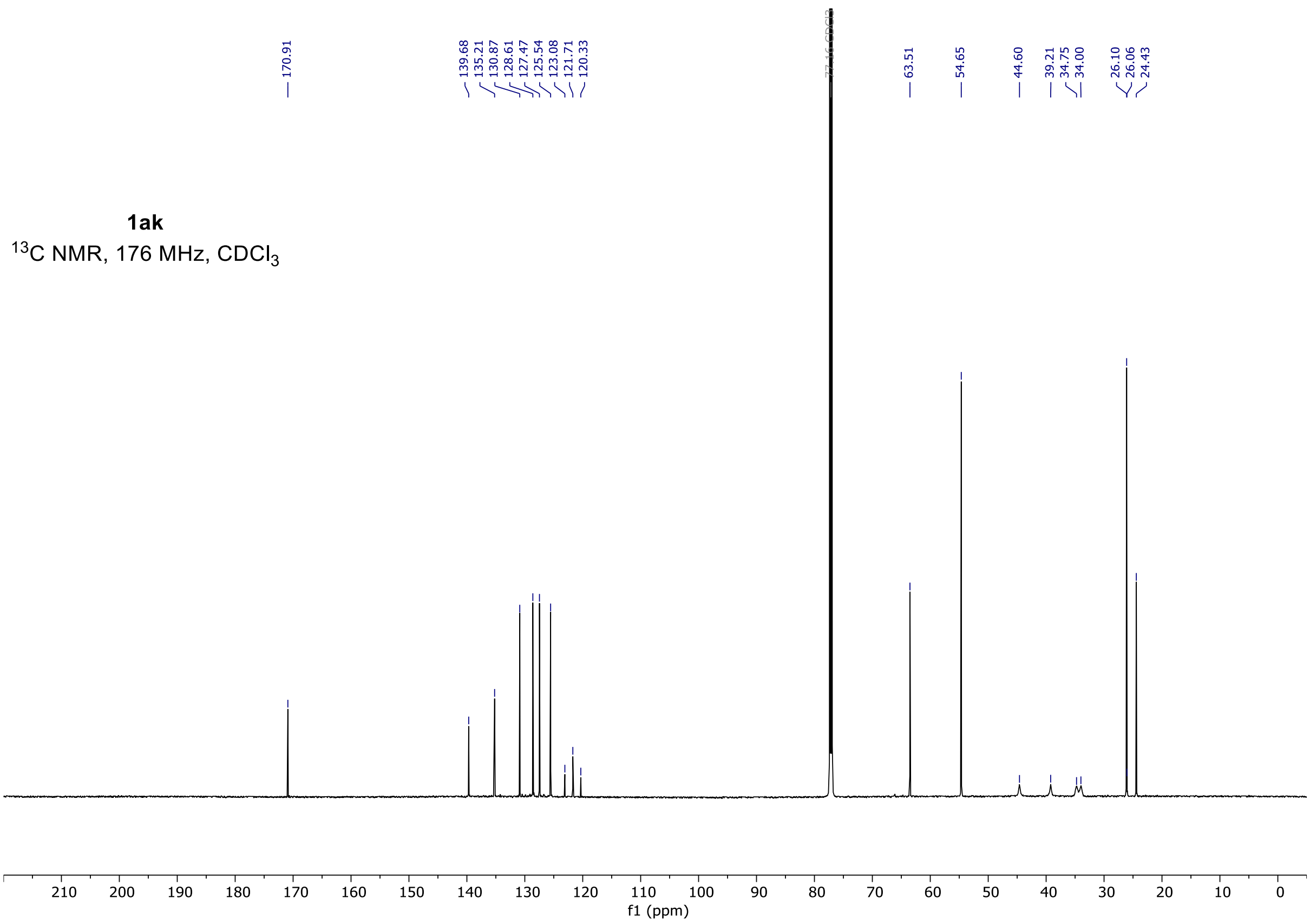

1ak

^{13}C NMR, 176 MHz, $CDCl_3$

1ak
^{19}F NMR, 376 MHz, $CDCl_3$

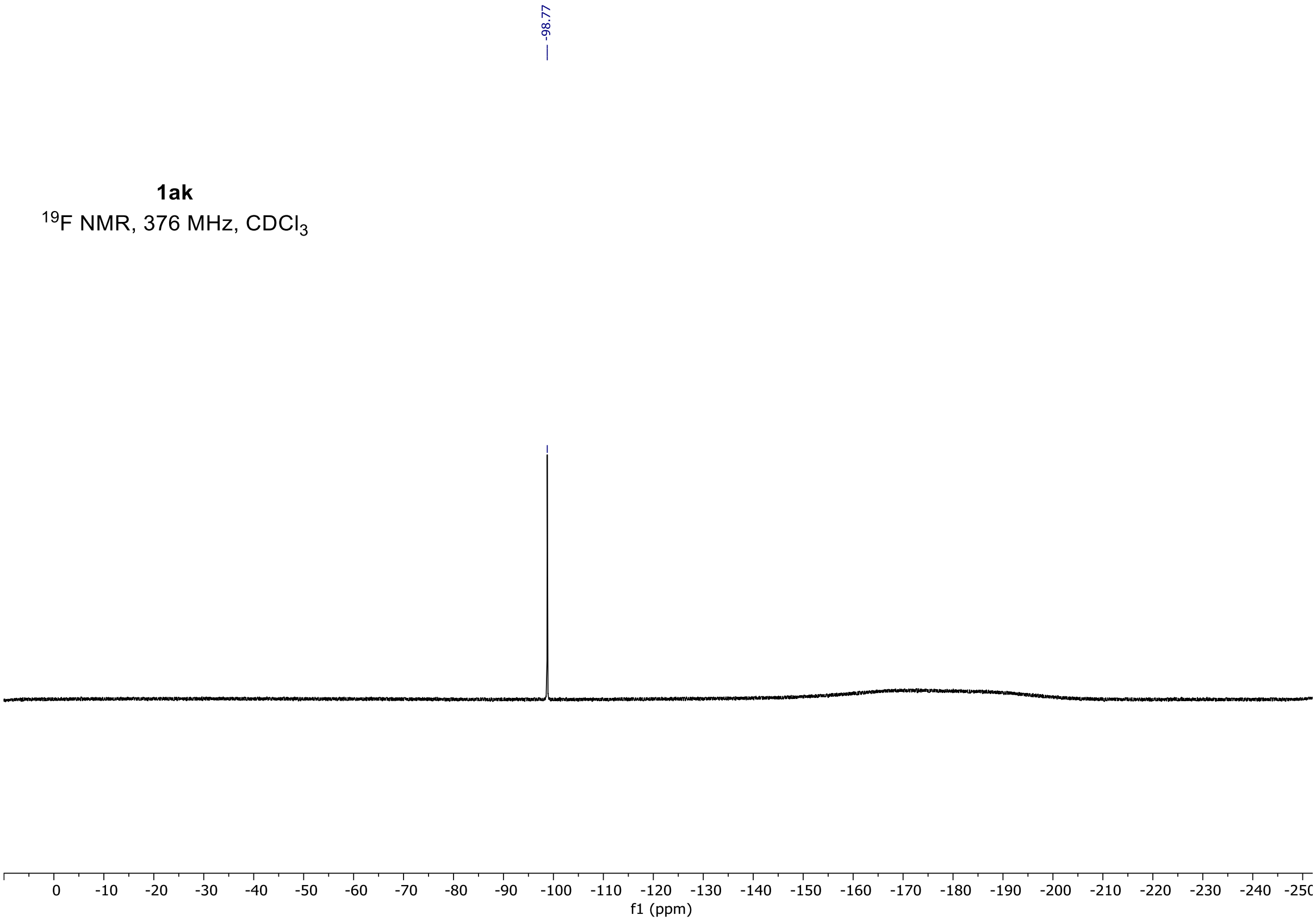

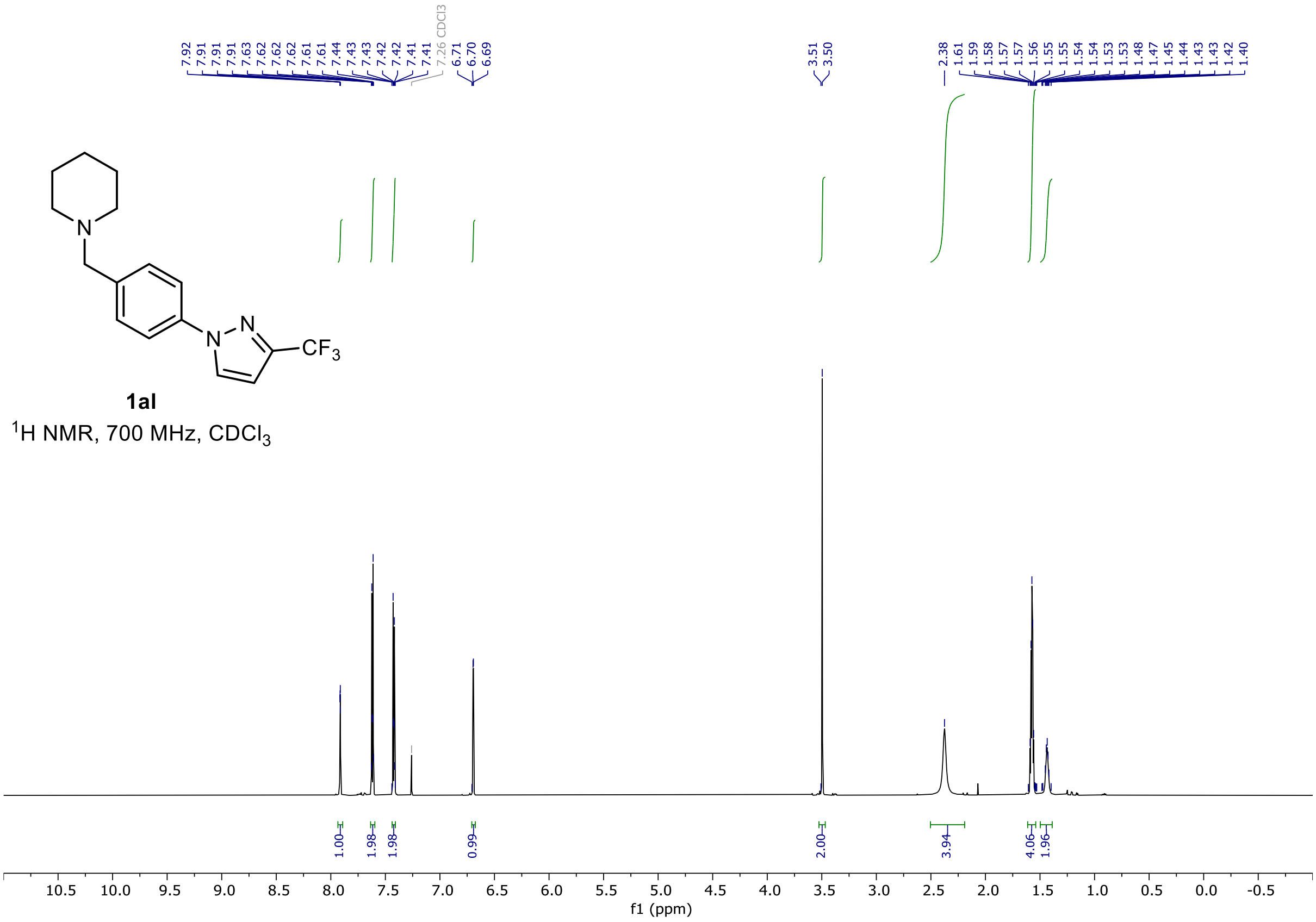

1al

^{1}H NMR, 700 MHz, $CDCl_3$

1al

^{13}C NMR, 176 MHz, $CDCl_3$

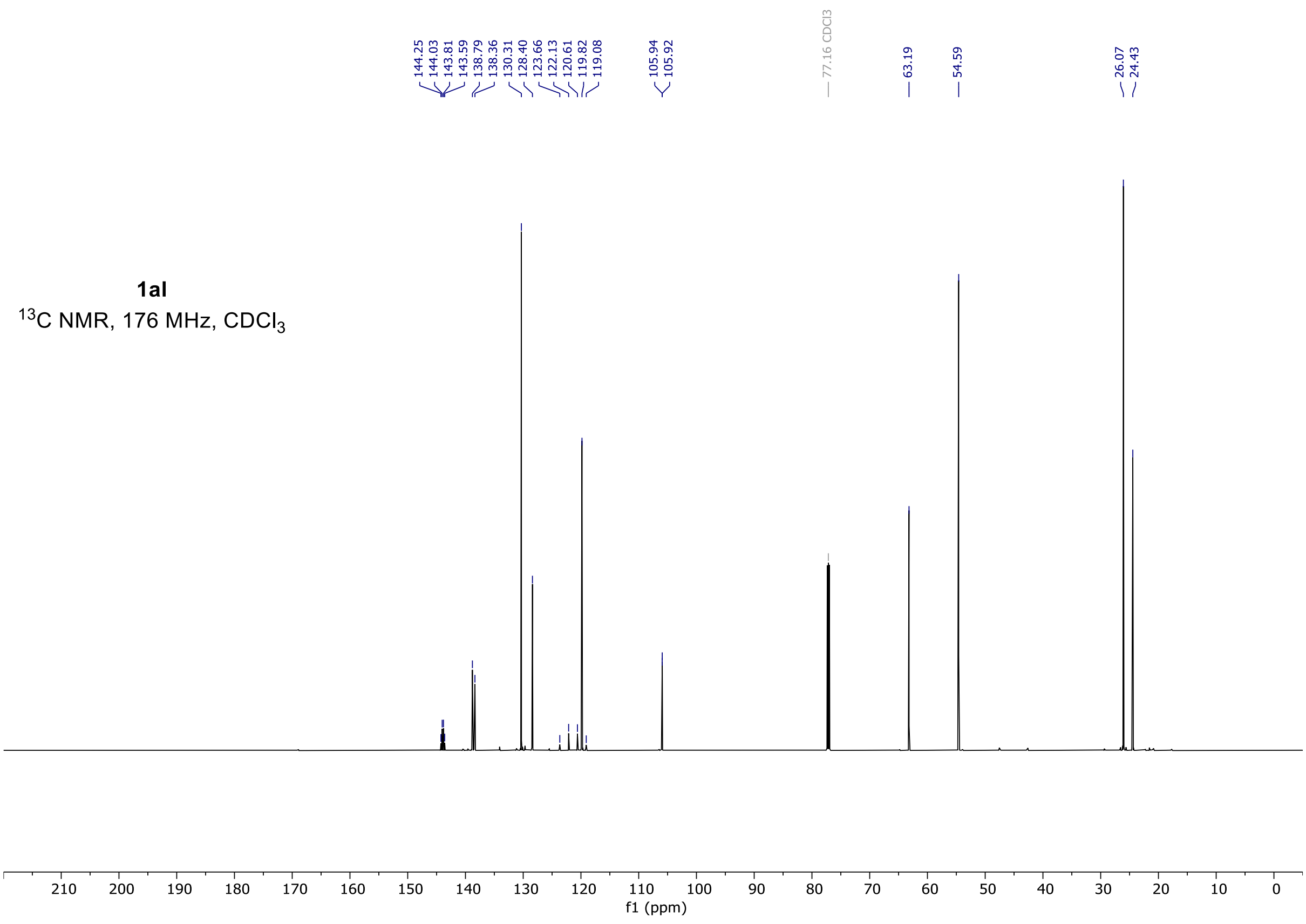

1al

^{19}F NMR, 376 MHz, $CDCl_3$

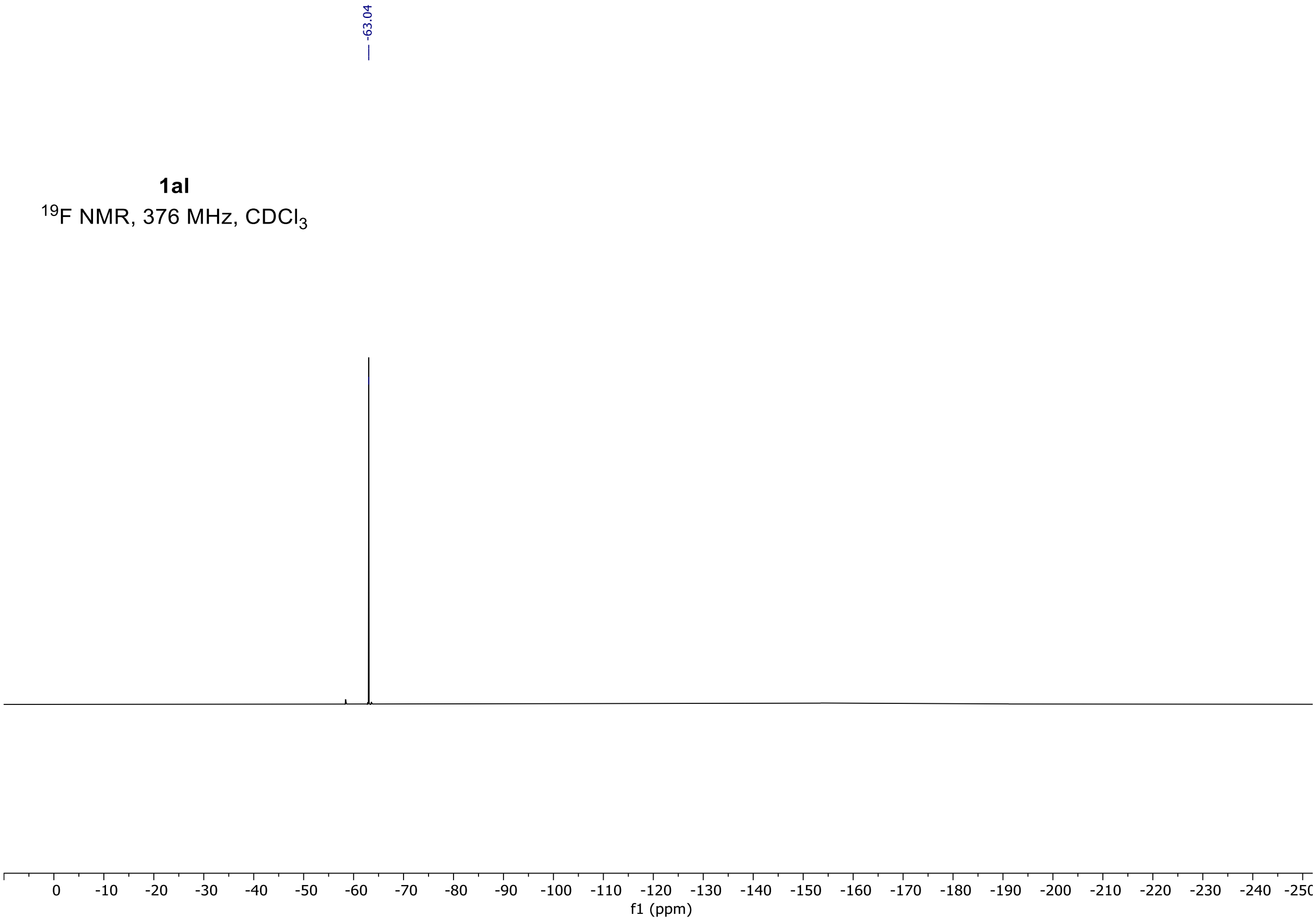

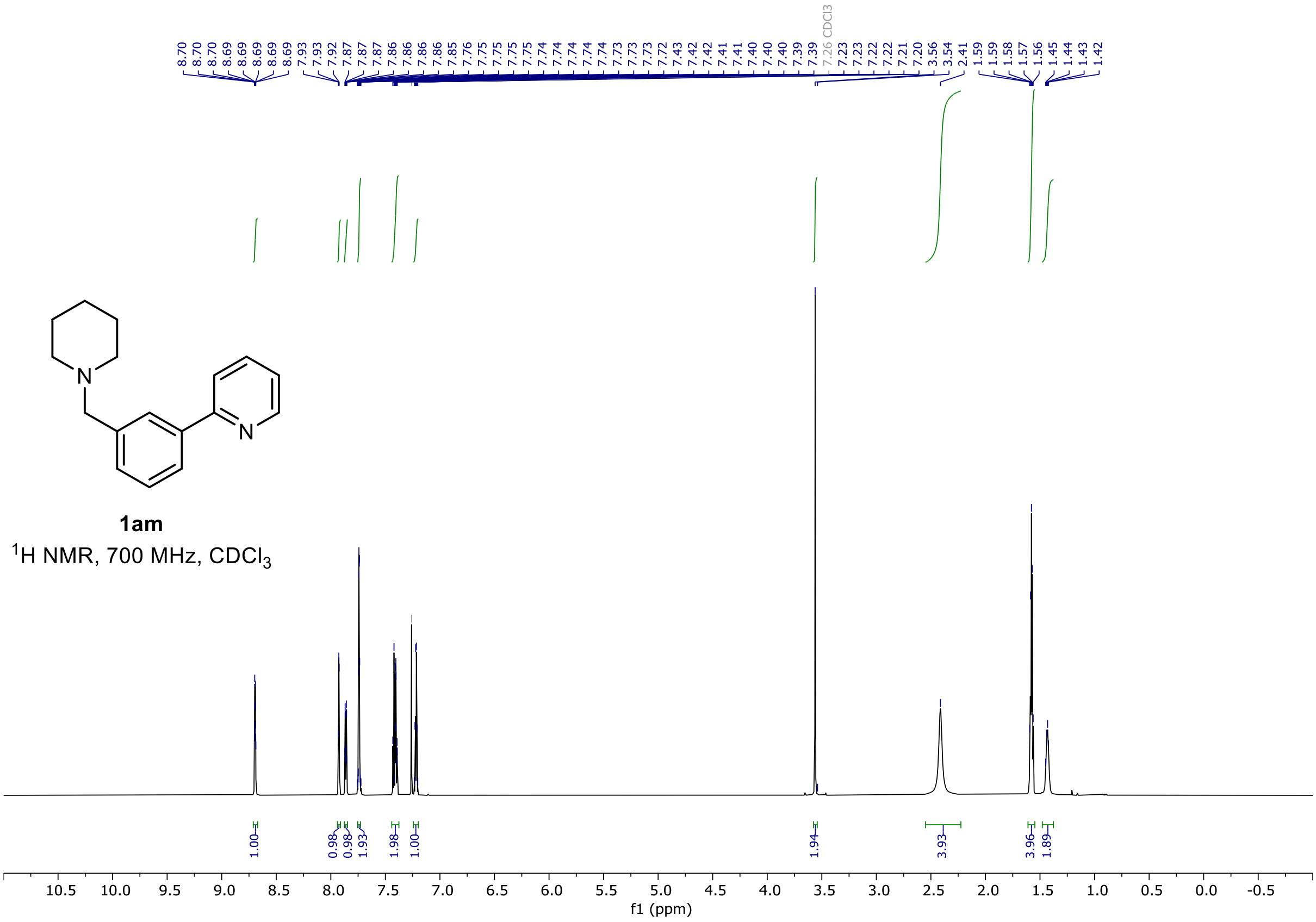

1am

^{1}H NMR, 700 MHz, $CDCl_3$

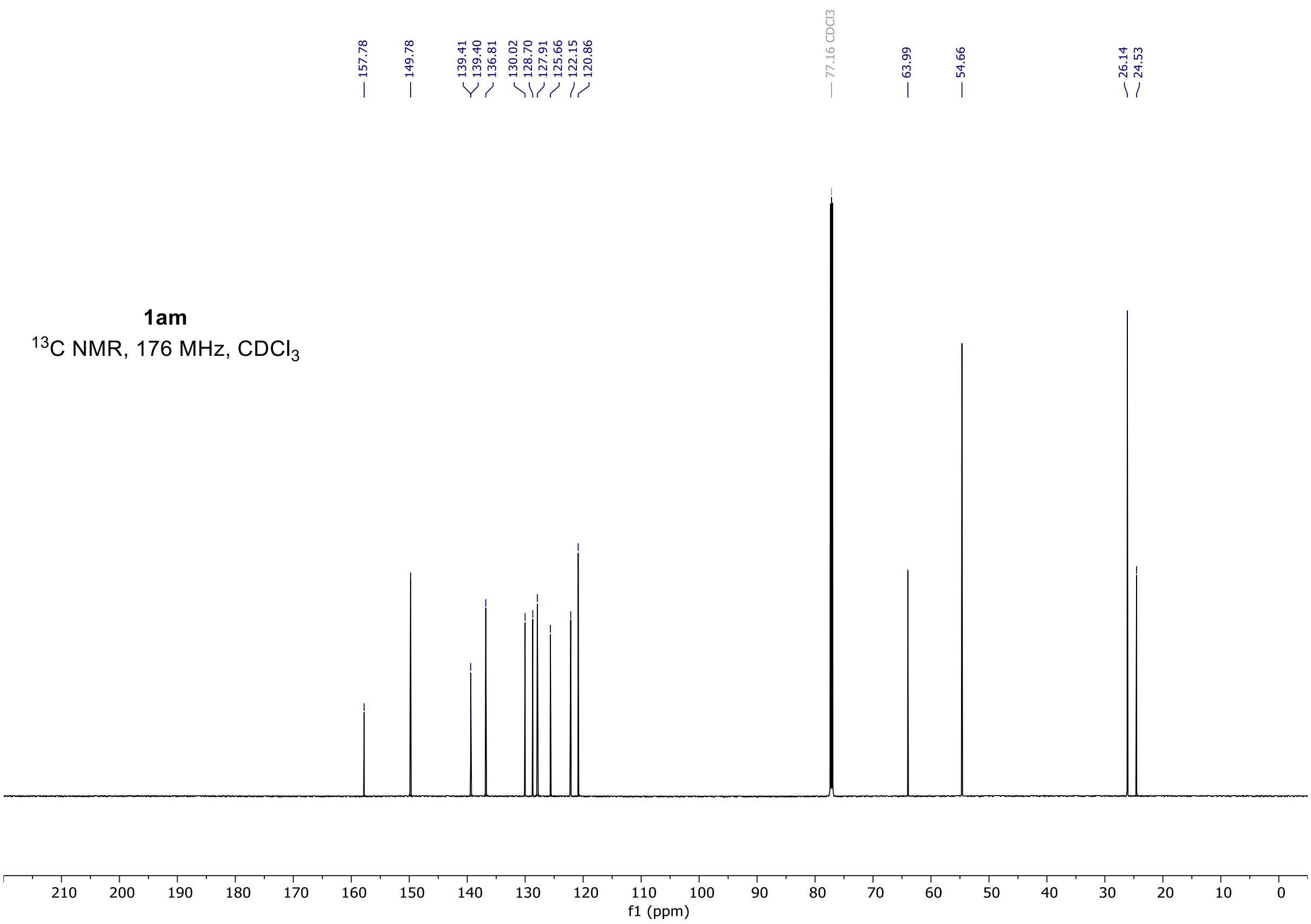

1am

^{13}C NMR, 176 MHz, $CDCl_3$

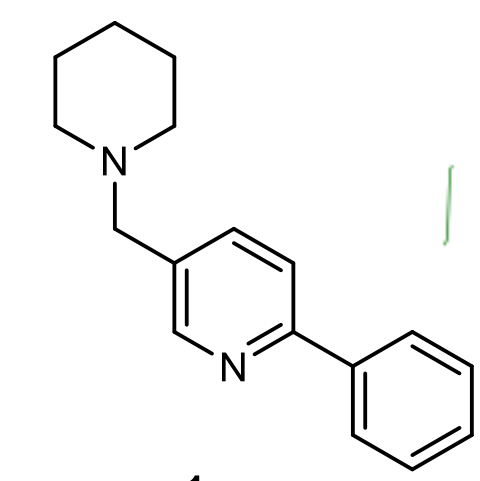

1ap

^{1}H NMR, 400 MHz, $CDCl_3$

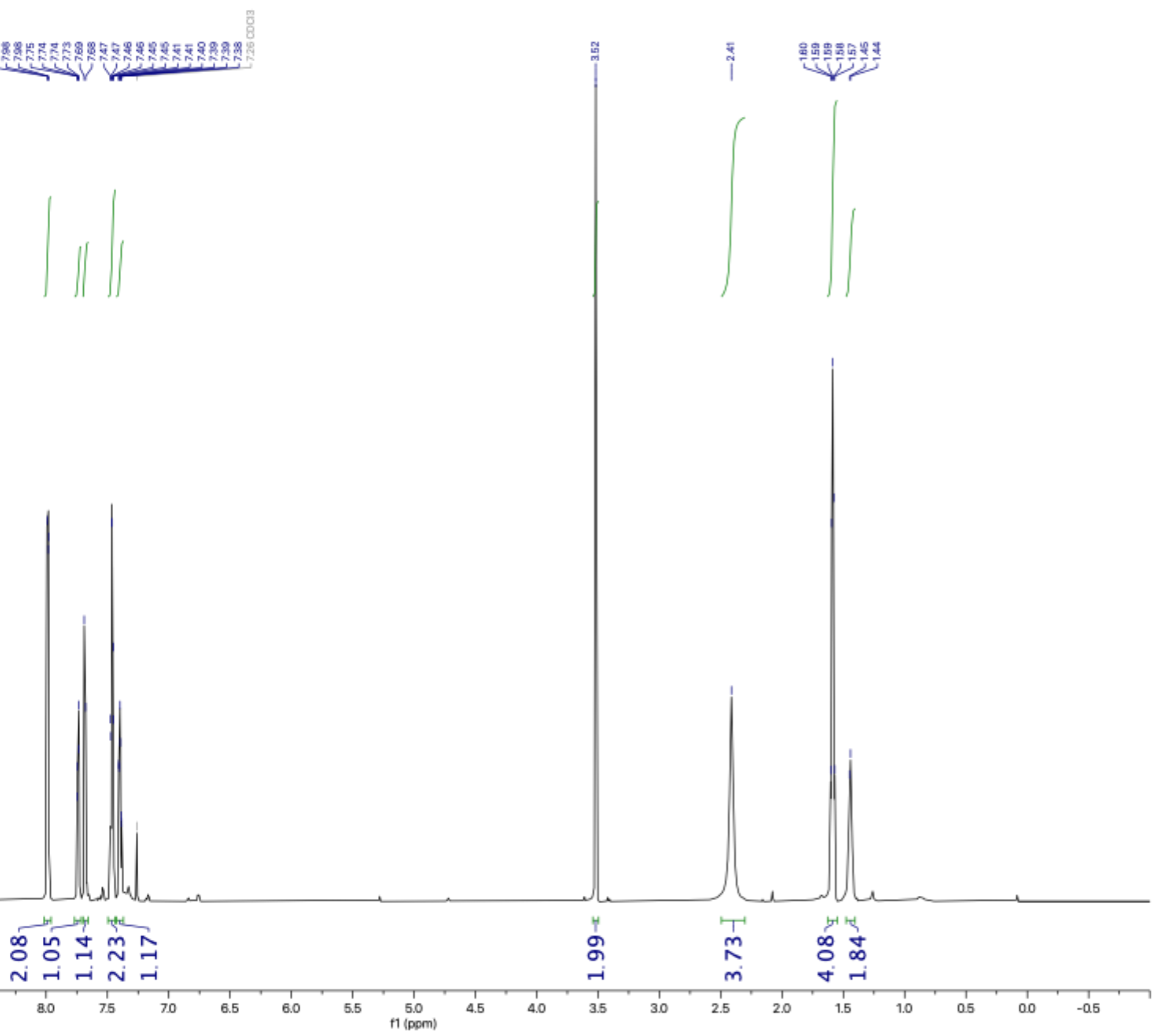

1ap

^{13}C NMR, 101 MHz, $CDCl_3$

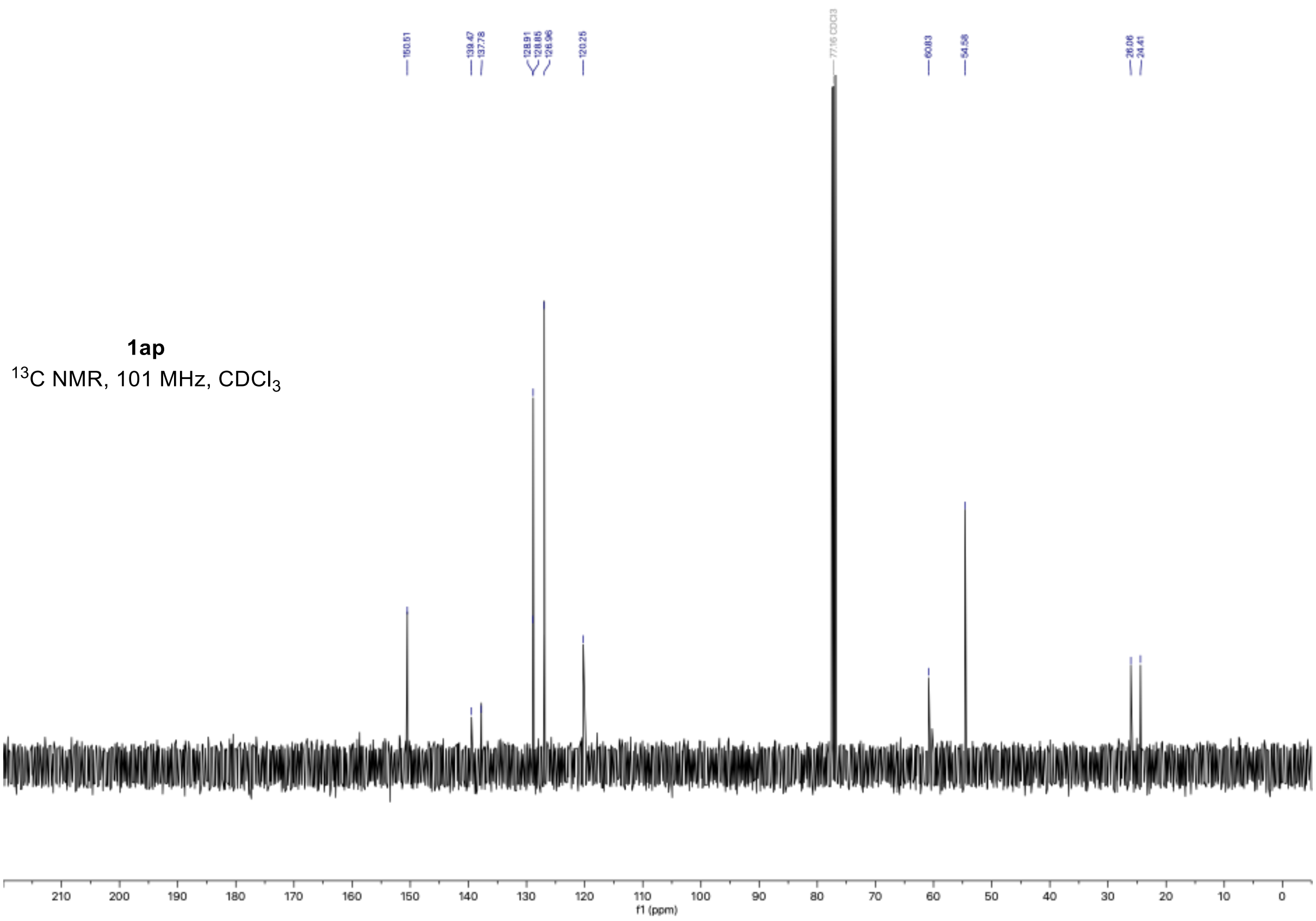

1aq

^{1}H NMR, 500 MHz, $CDCl_3$

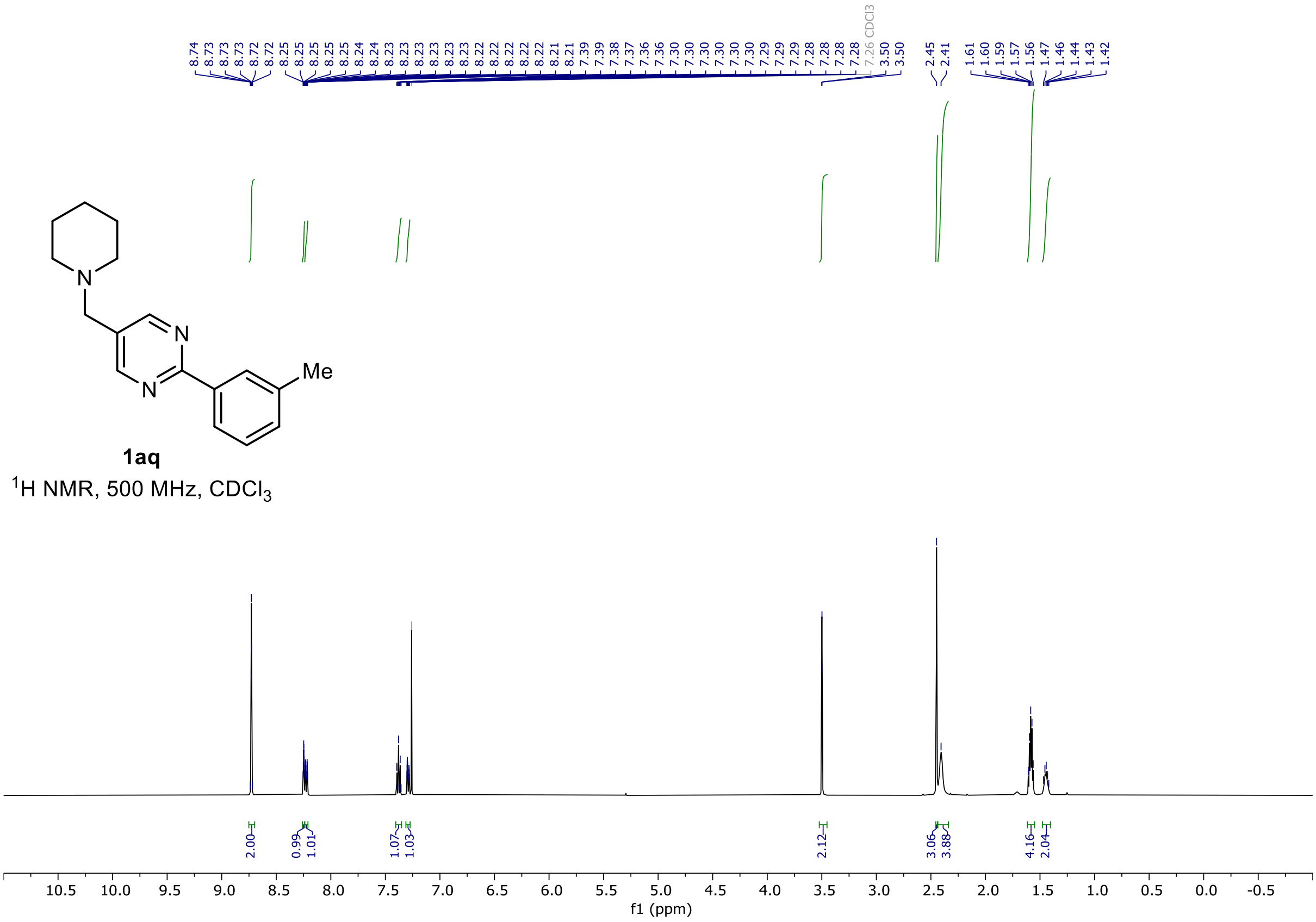

1aq

^{13}C NMR, 126 MHz, $CDCl_3$

1aq

^{1}H NMR, 500 MHz, $CDCl_3$

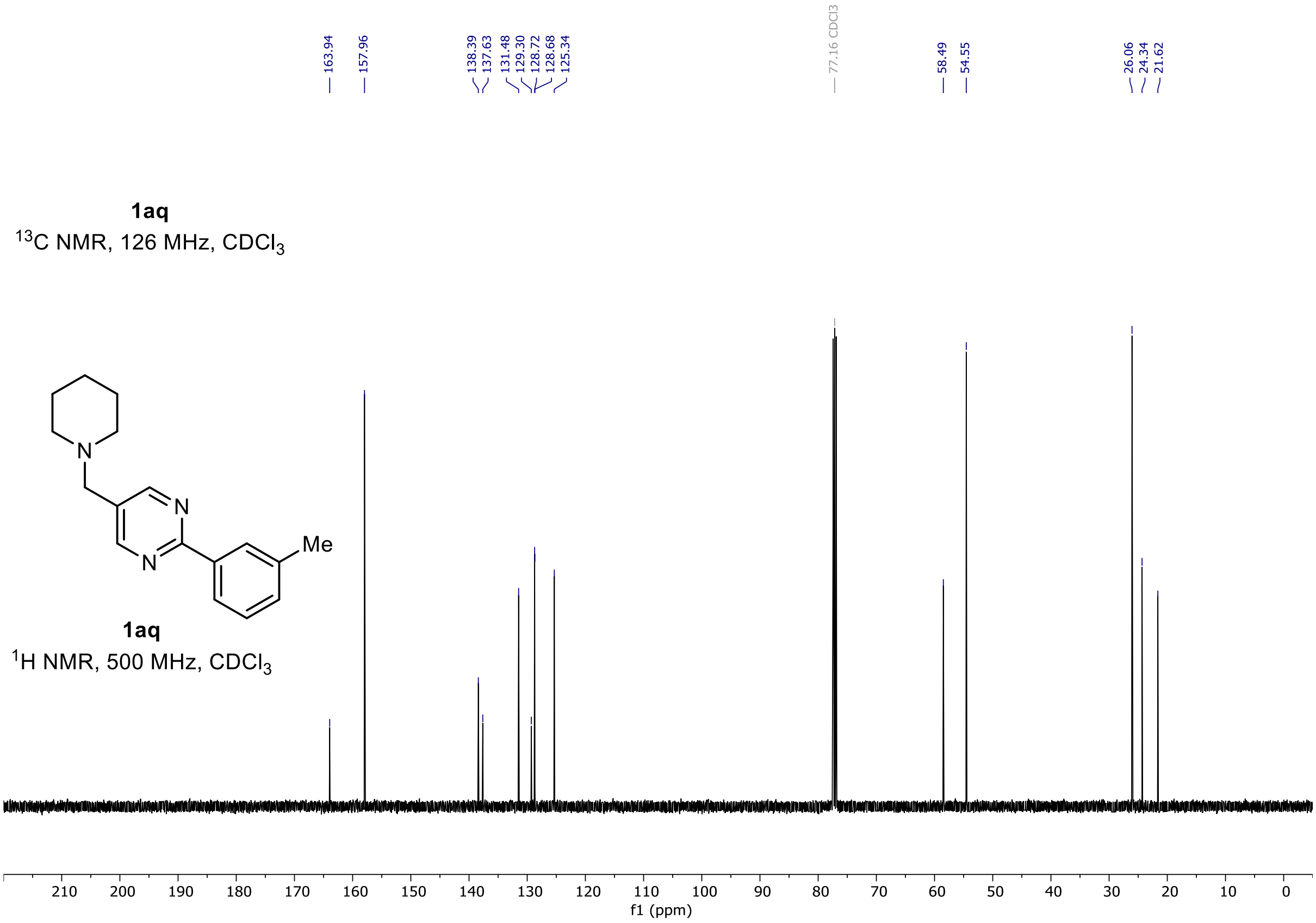

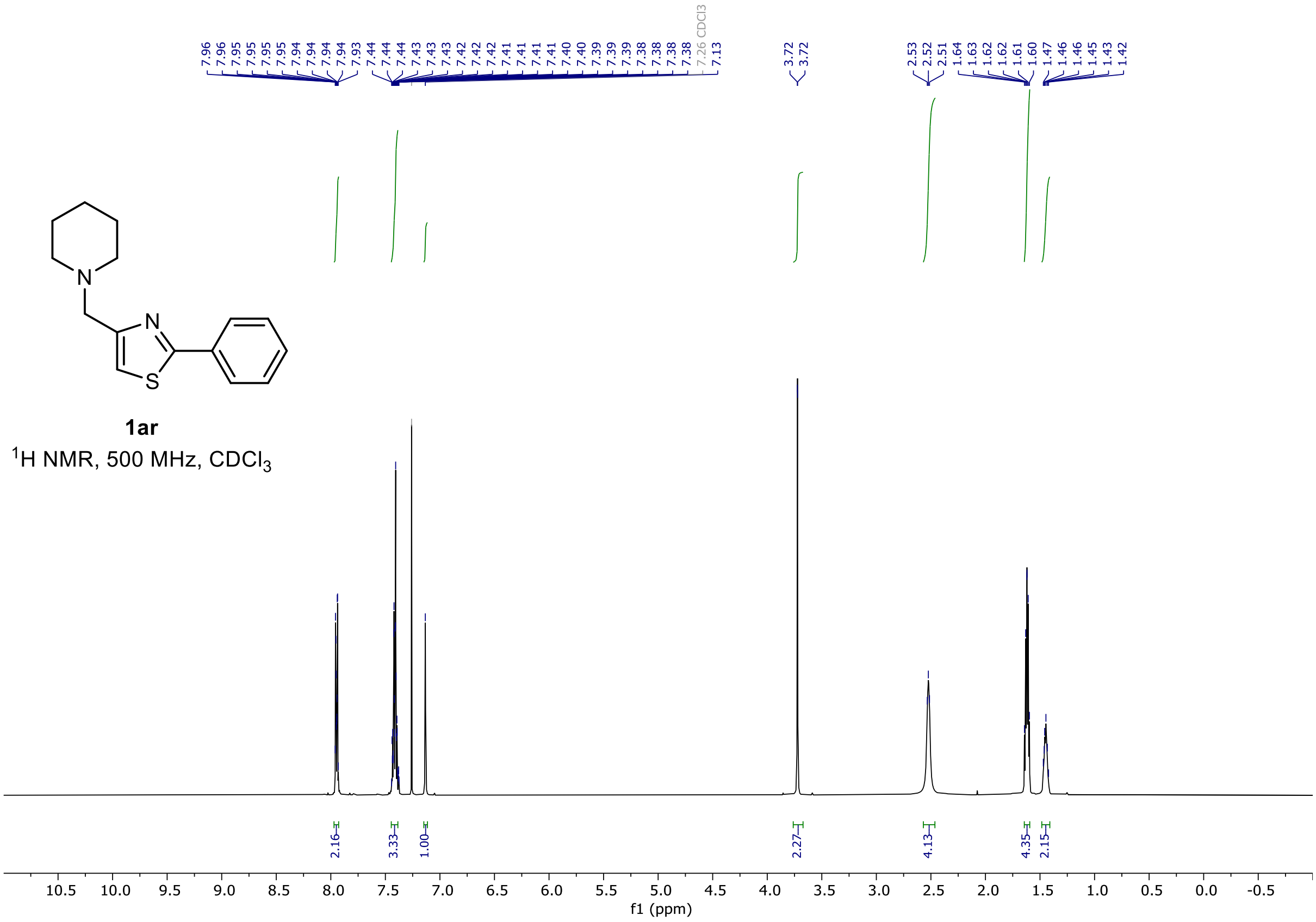
7.96 7.96 7.95 7.95 7.95 7.95 7.94 7.94 7.94 7.94 7.93 7.44 7.44 7.43 7.43 7.43 7.42 7.42 7.42 7.41 7.41 7.41 7.41 7.40 7.39 7.39 7.39 7.38 7.38 7.38 7.38 7.26 CDCl3 7.13
3.72 3.72
2.53 2.52 2.51 1.64 1.63 1.62 1.62 1.61 1.60 1.47 1.46 1.45 1.43 1.42
N
N
S
1ar
1H NMR, 500 MHz, CDCl3
2.16 3.33 1.00 2.27 4.13 4.35 2.15
10.5 10.0 9.5 9.0 8.5 8.0 7.5 7.0 6.5 6.0 5.5 5.0 4.5 4.0 3.5 3.0 2.5 2.0 1.5 1.0 0.5 0.0 -0.5
f1 (ppm)

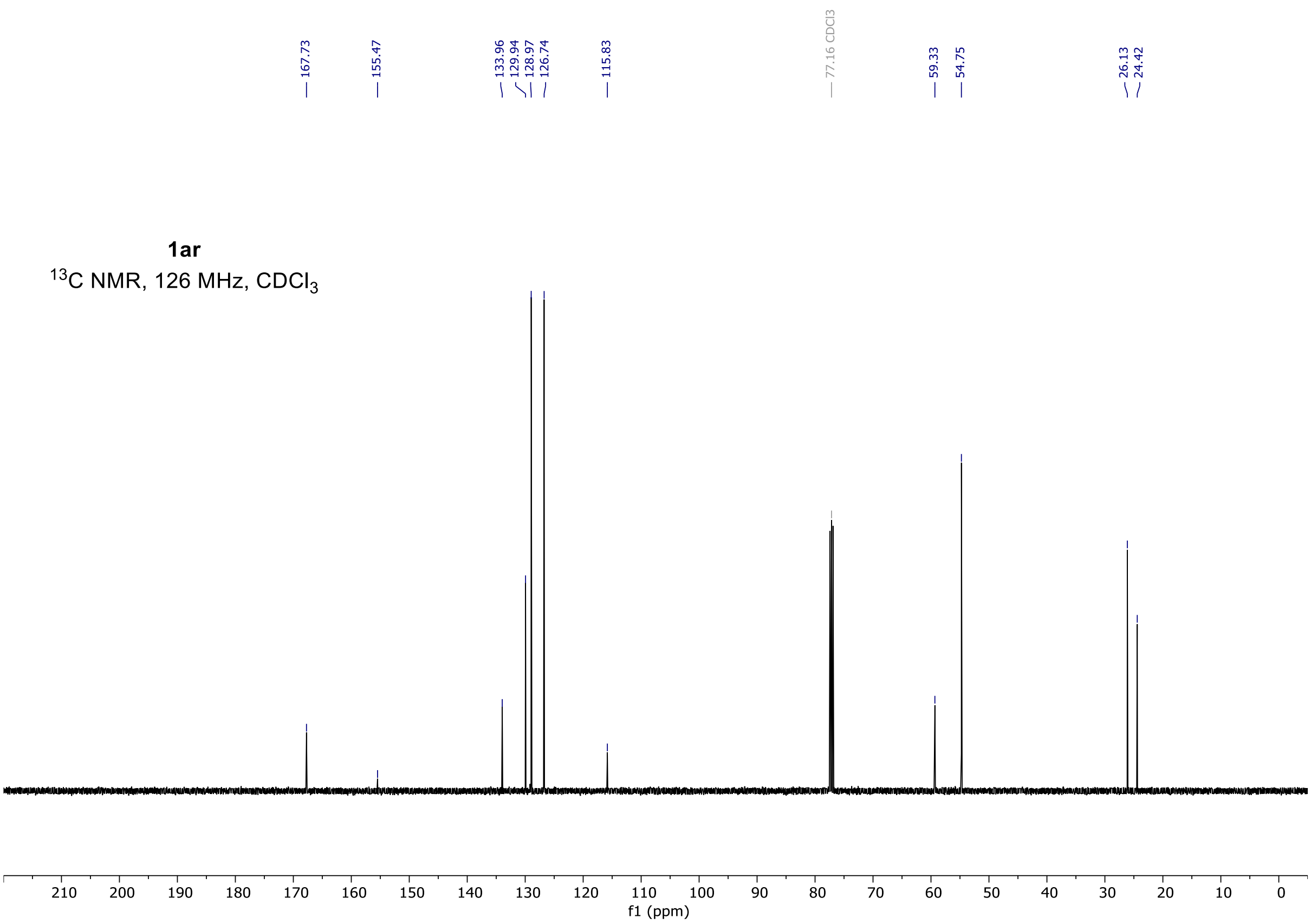
167.73
155.47
133.96 129.94 128.97 126.74
115.83
77.16 CDCl3
59.33
54.75
26.13 24.42
1ar
13C NMR, 126 MHz, CDCl3
210 200 190 180 170 160 150 140 130 120 110 100 90 80 70 60 50 40 30 20 10 0
f1 (ppm)

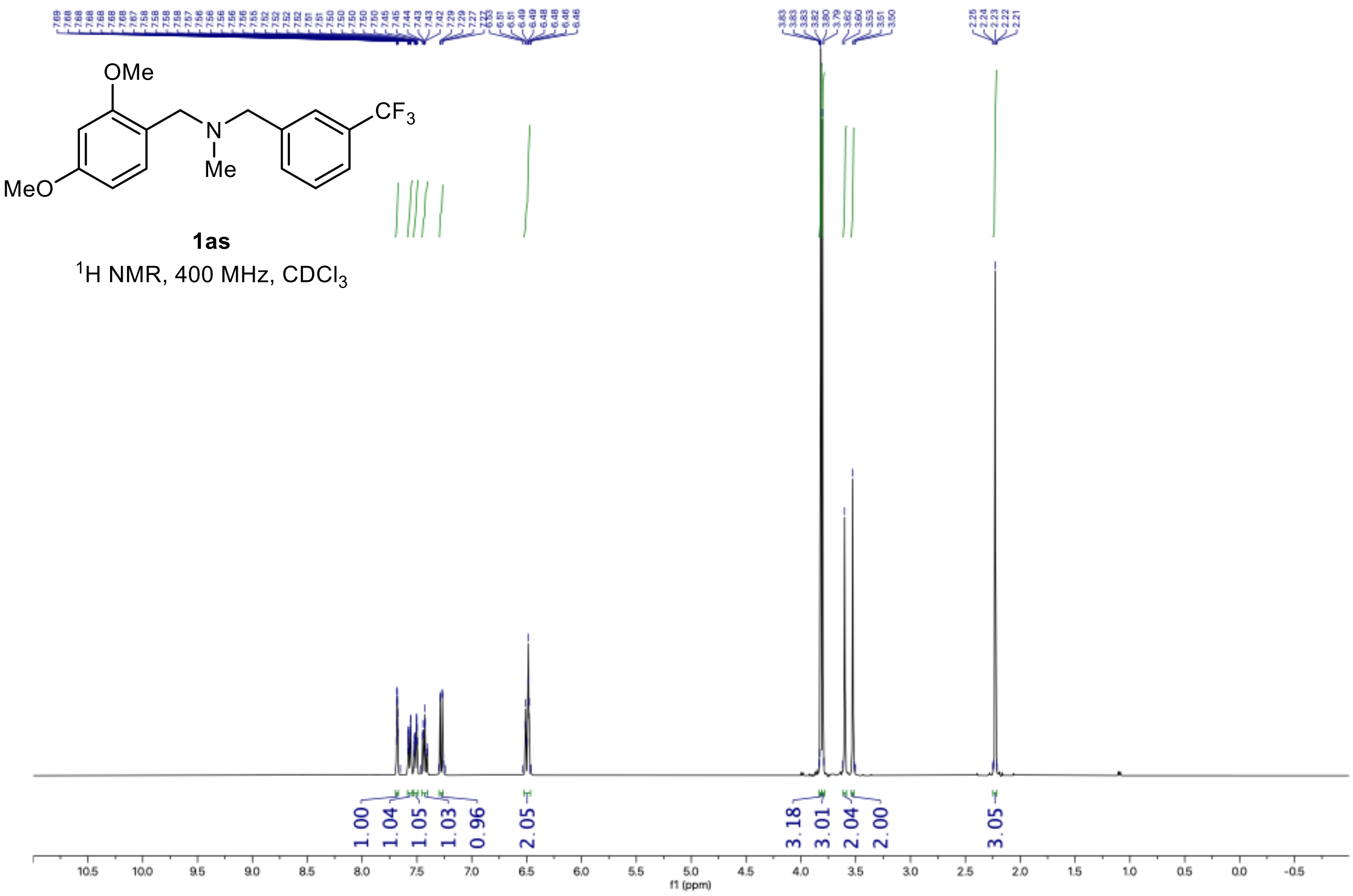

1as
^{1}H NMR, 400 MHz, $CDCl_3$

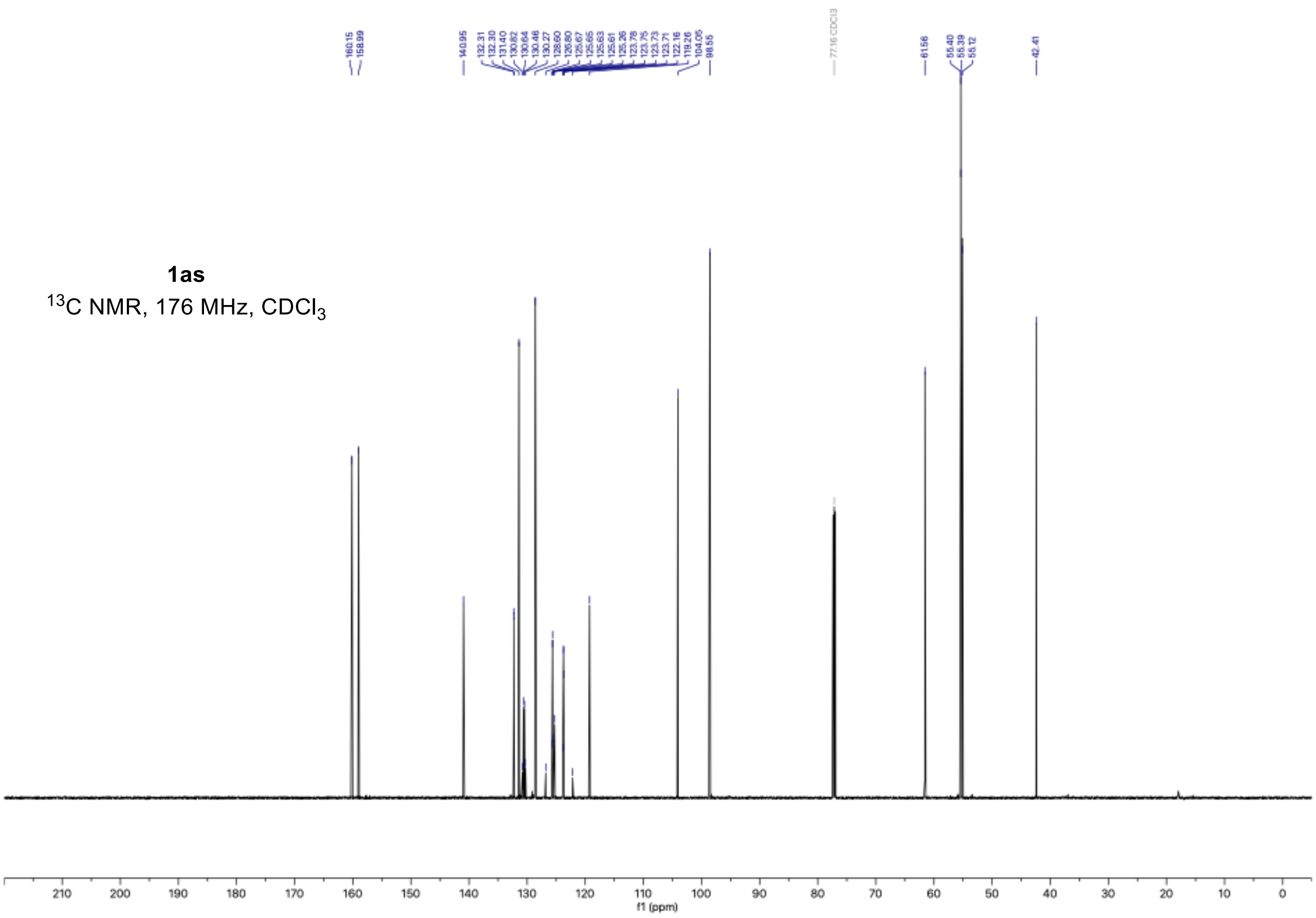

1as
^{13}C NMR, 176 MHz, $CDCl_3$

1as

^{13}C NMR, 376 MHz, $CDCl_3$

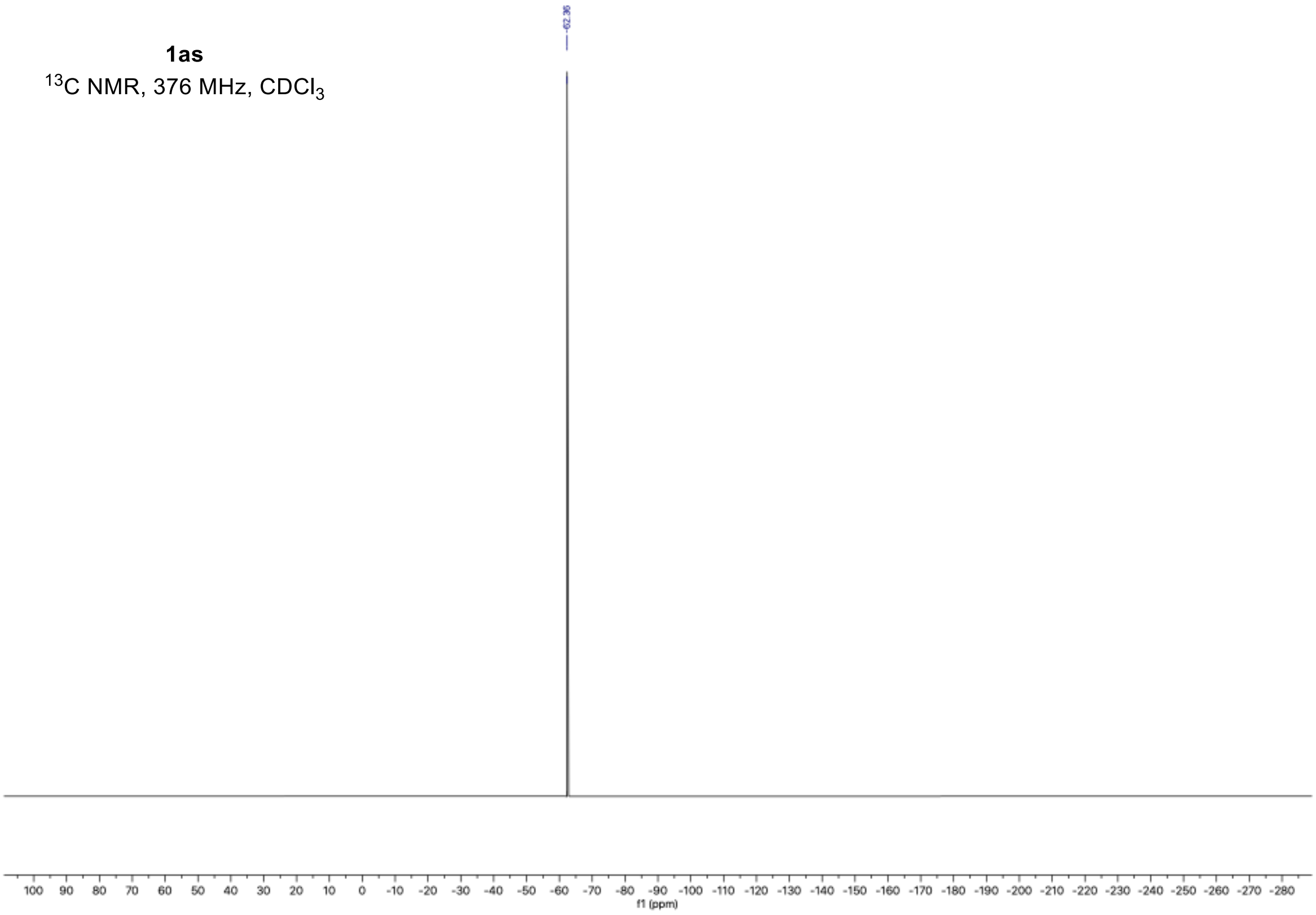

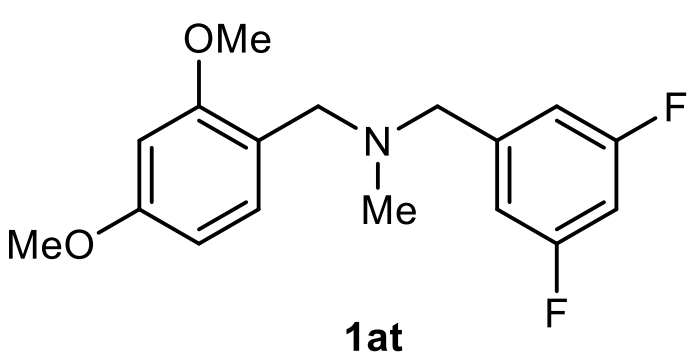

1at

^{1}H NMR, 400 MHz, $CDCl_3$

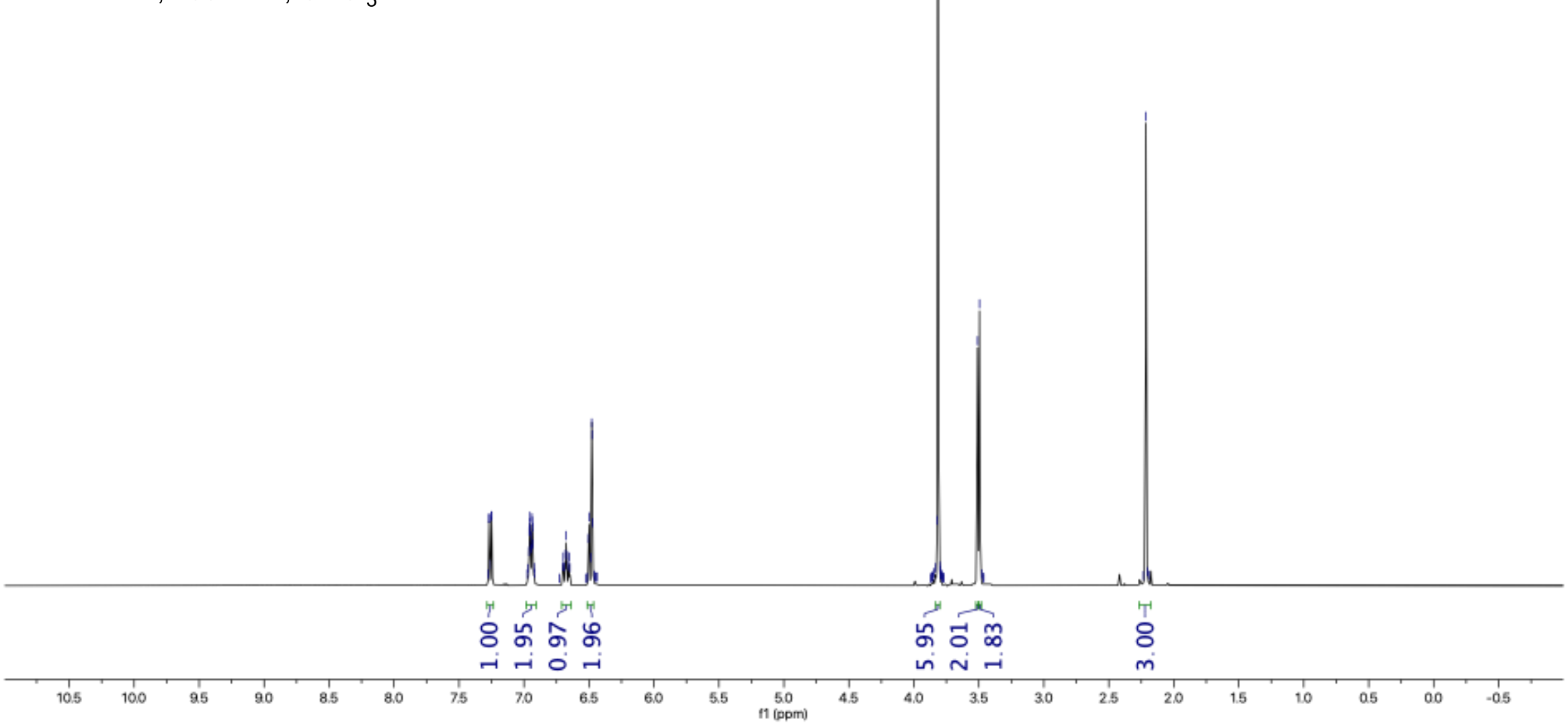

1at

^{13}C NMR, 176 MHz, $CDCl_3$

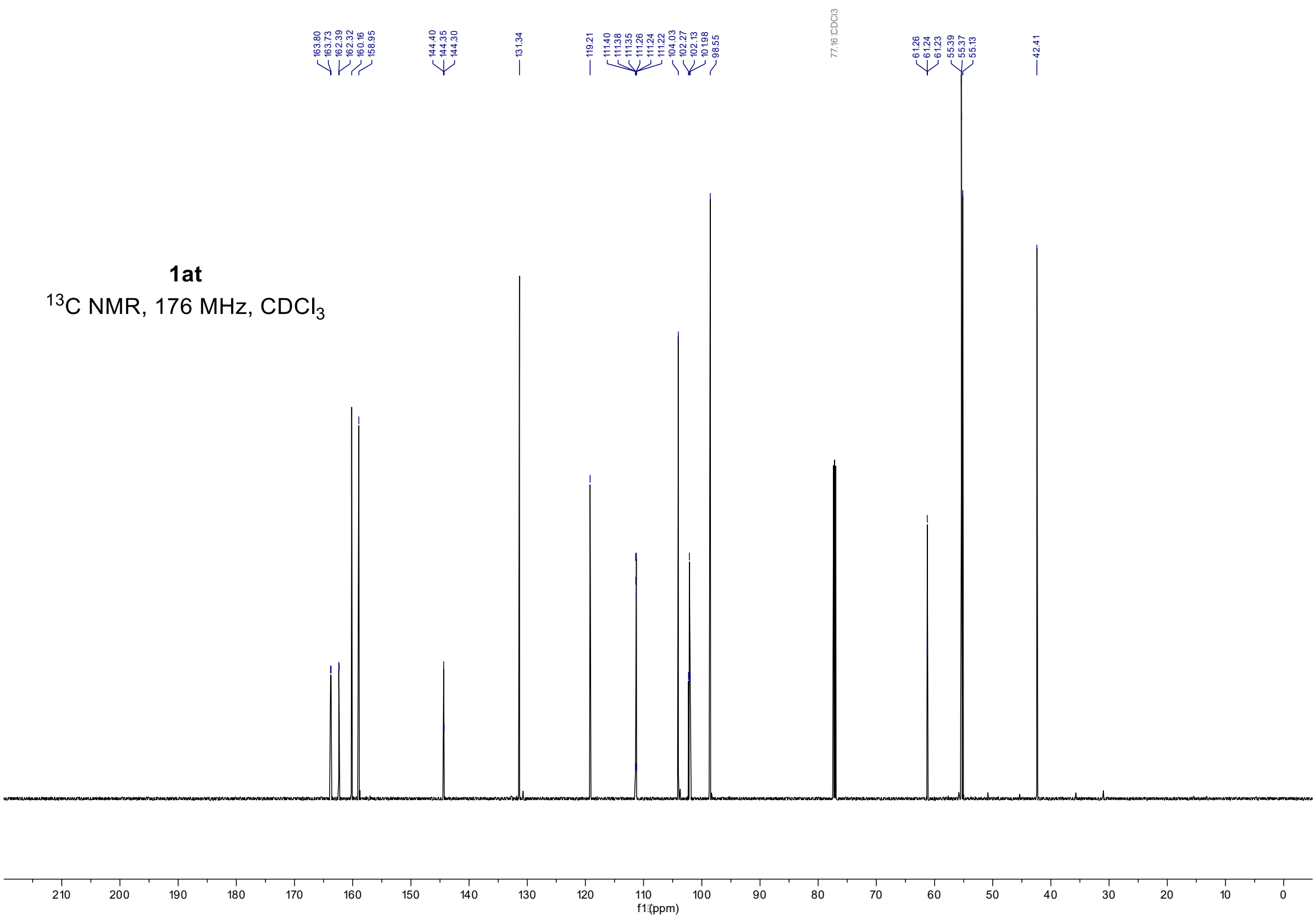

1at

^{13}C NMR, 376 MHz, $CDCl_3$

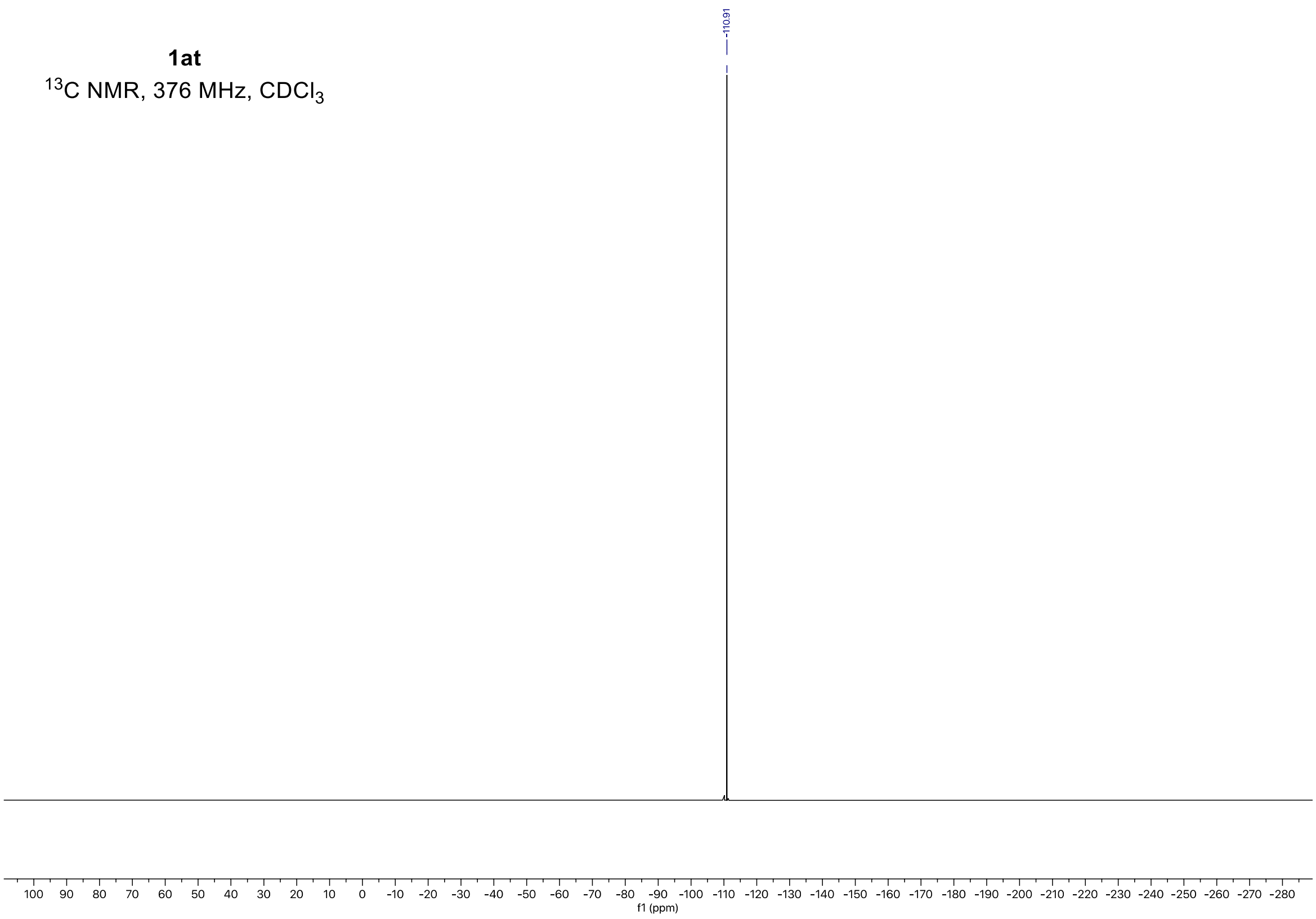

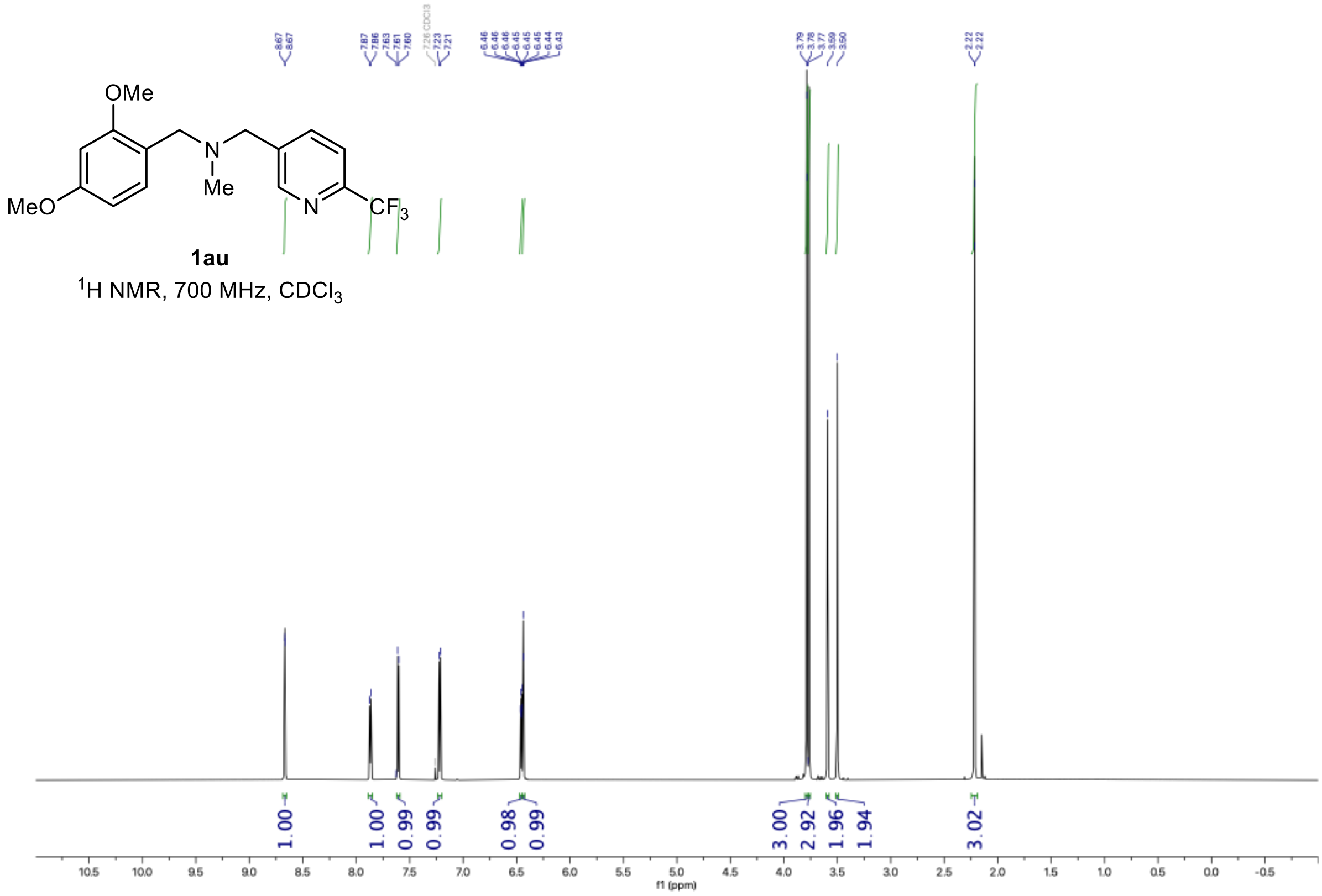

1au

^{1}H NMR, 700 MHz, $CDCl_3$

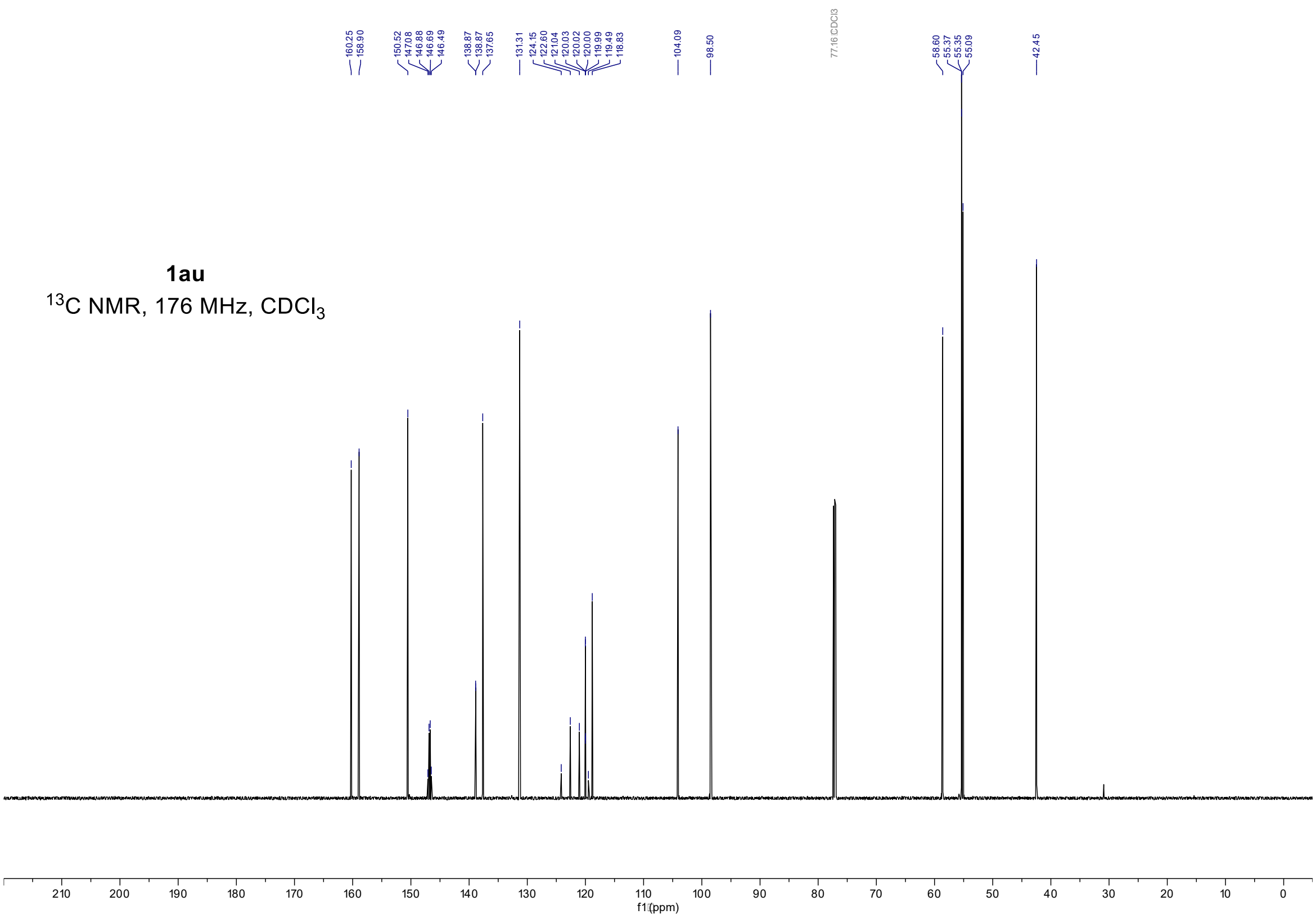

1au

^{13}C NMR, 176 MHz, $CDCl_3$

1au

^{13}C NMR, 376 MHz, $CDCl_3$

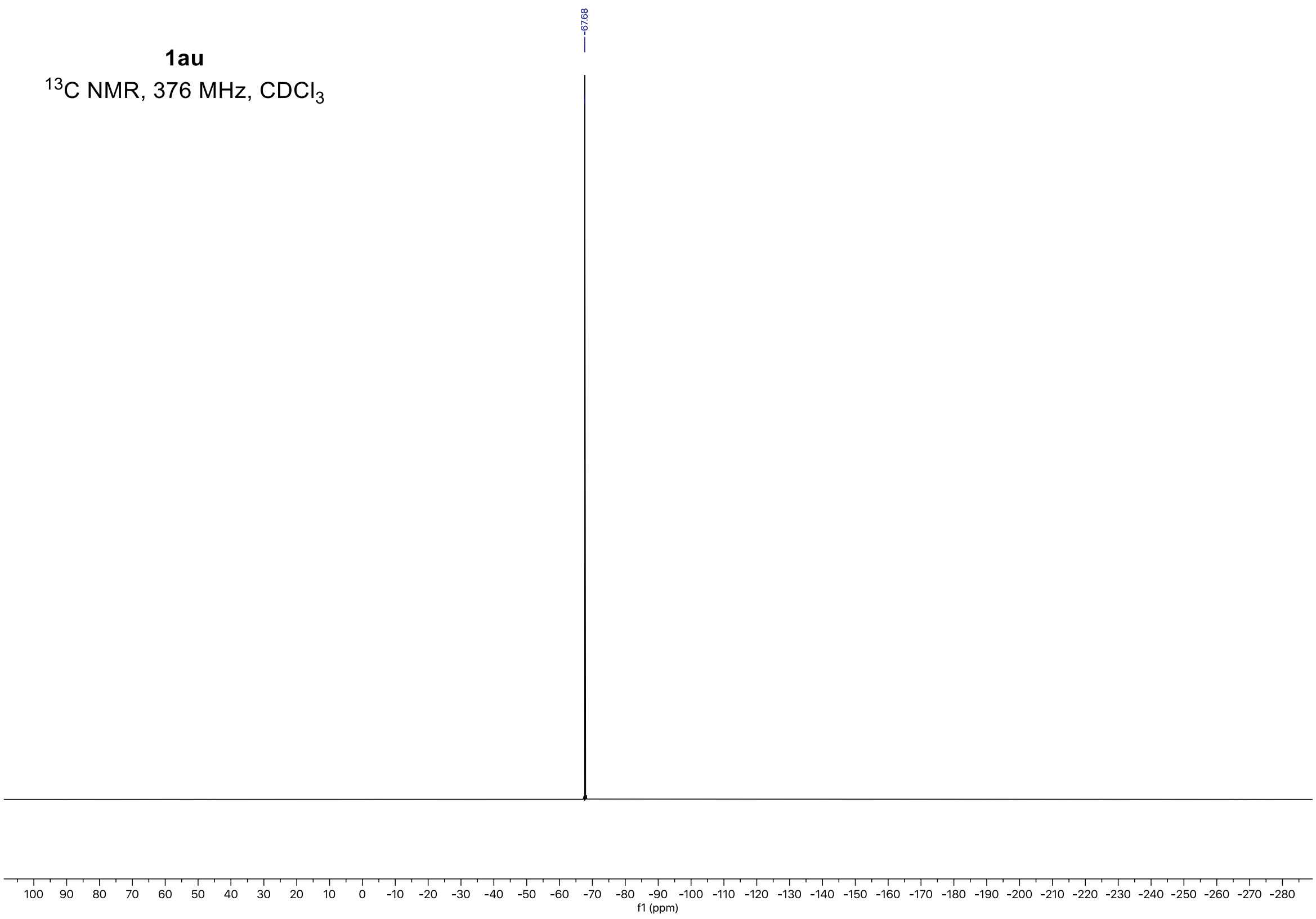

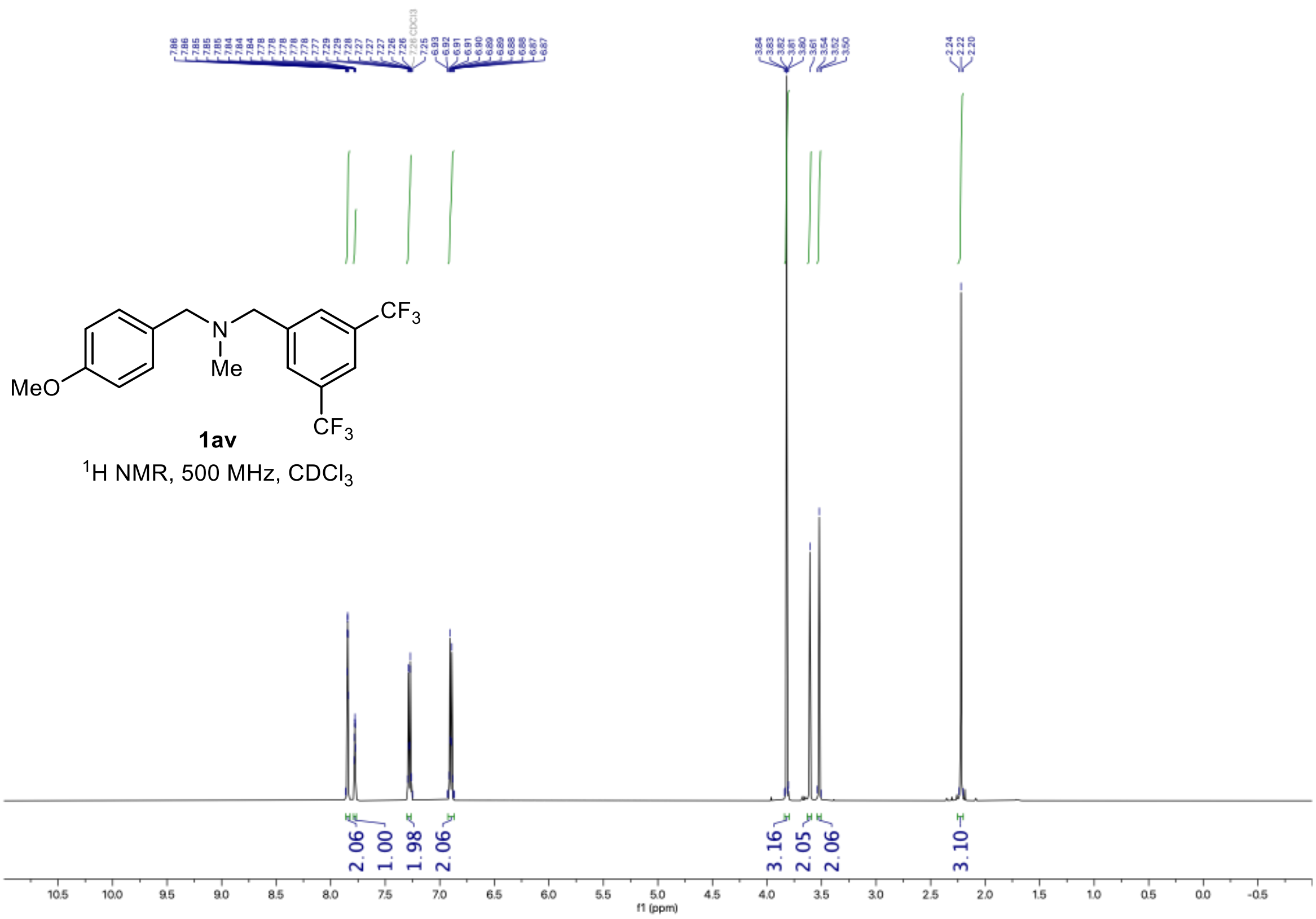

1av

^{1}H NMR, 500 MHz, $CDCl_3$

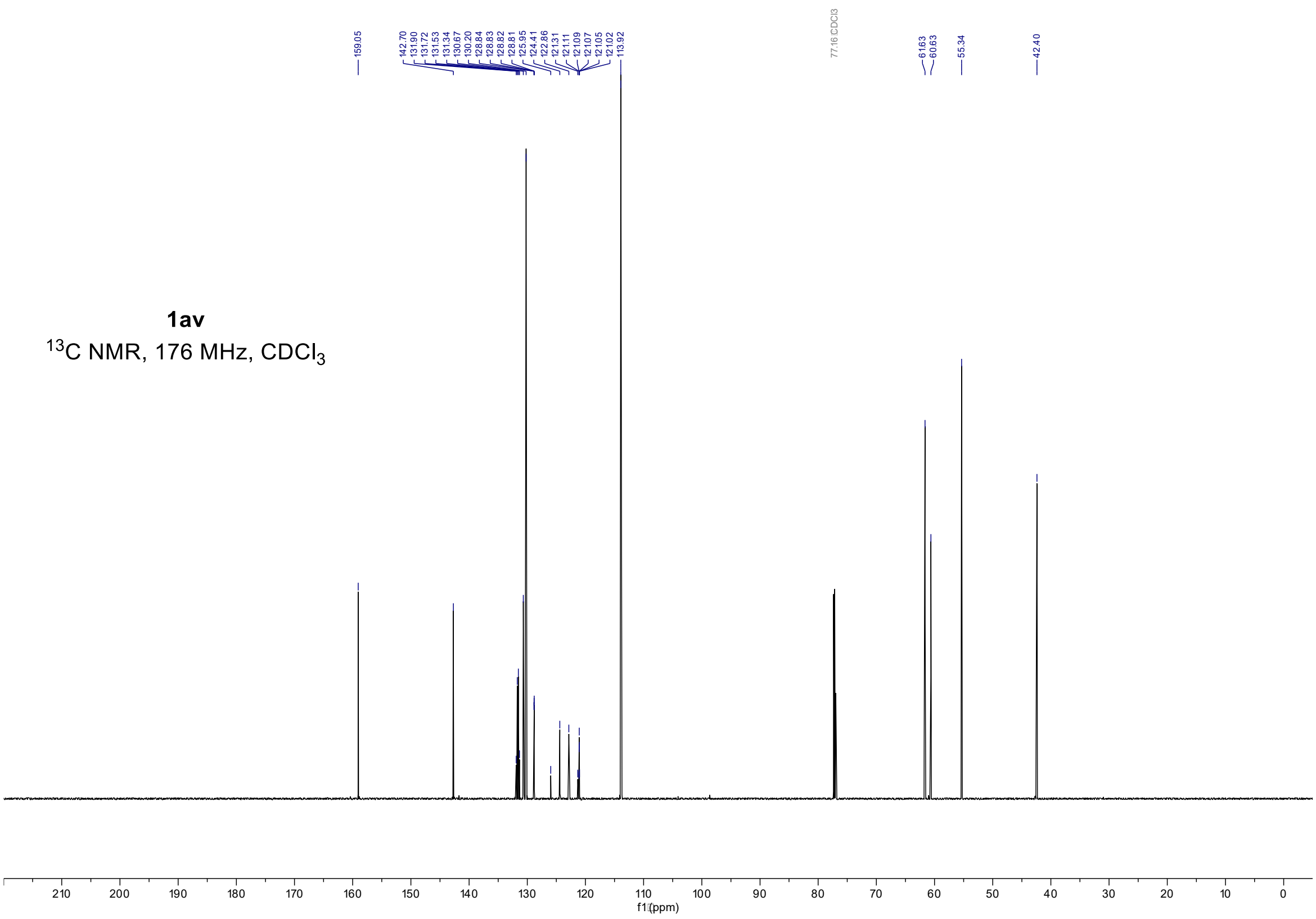

1av

^{13}C NMR, 176 MHz, $CDCl_3$

1av

^{19}F NMR, 471 MHz, $CDCl_3$

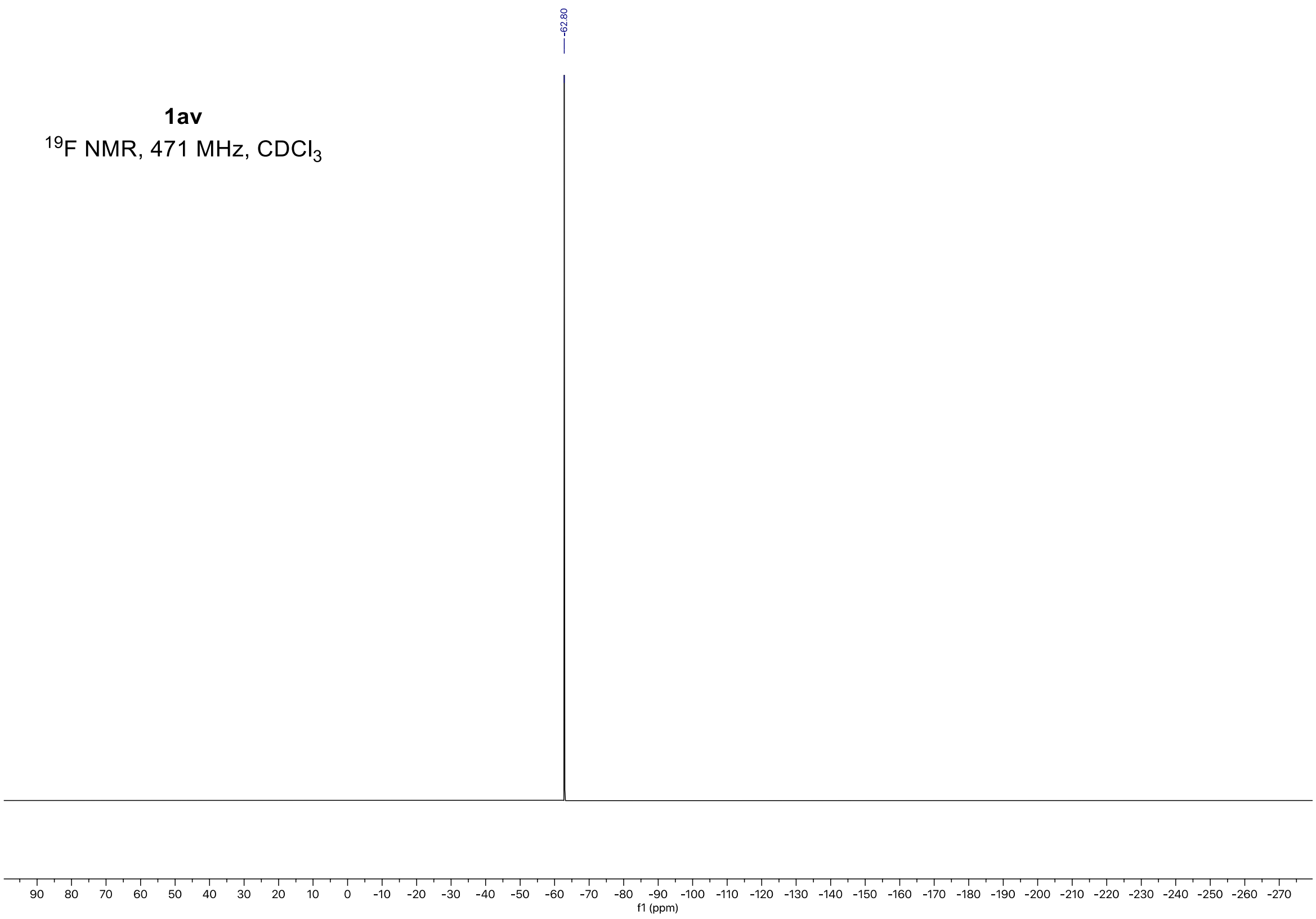

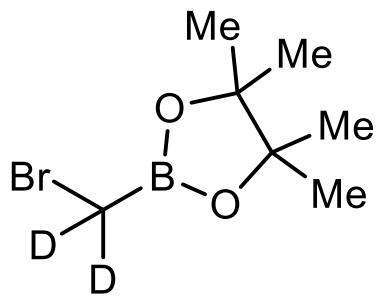

^{1}H NMR, 700 MHz, $CDCl_3$

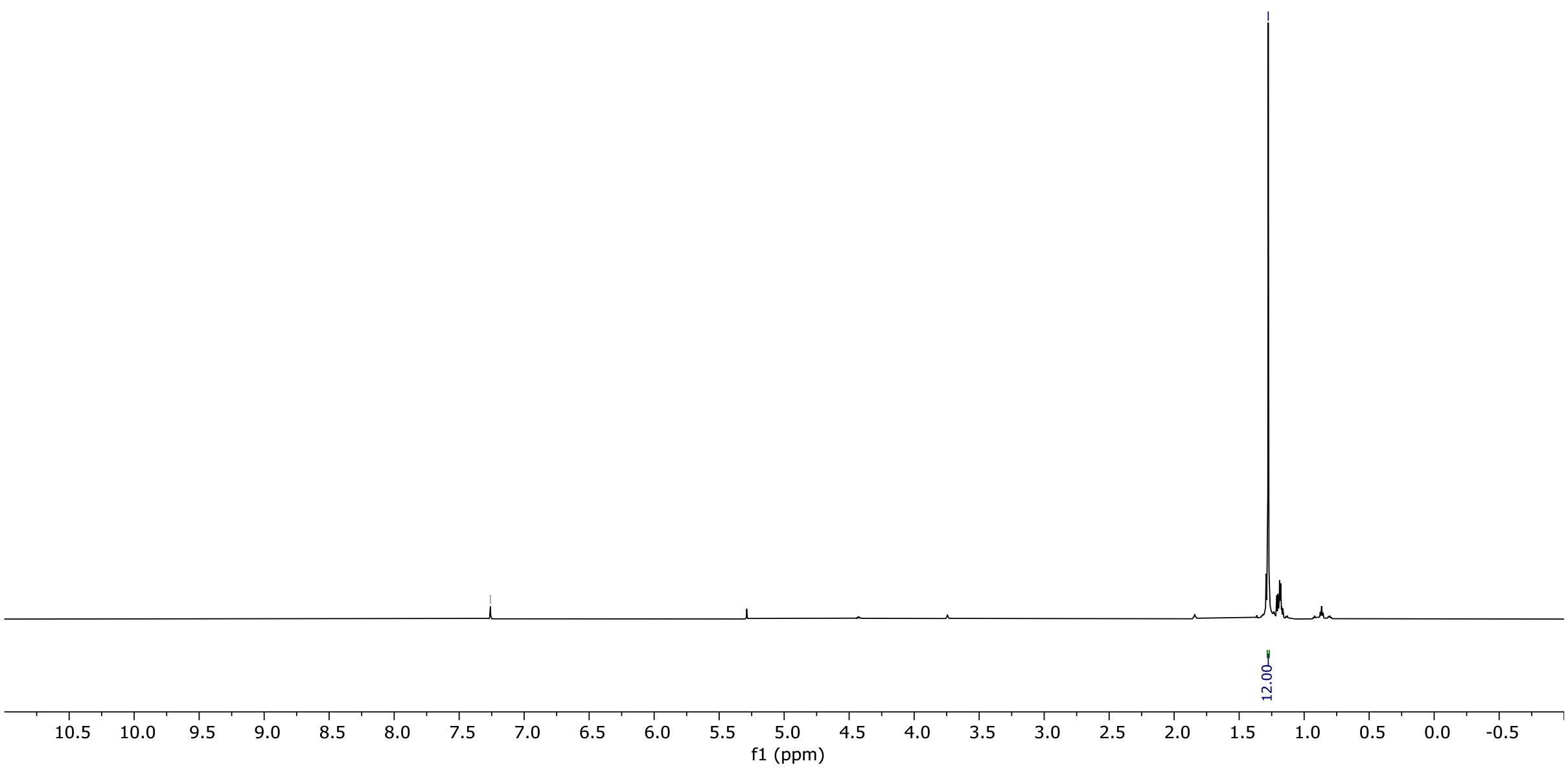

^{13}C NMR, 176 MHz, $CDCl_3$

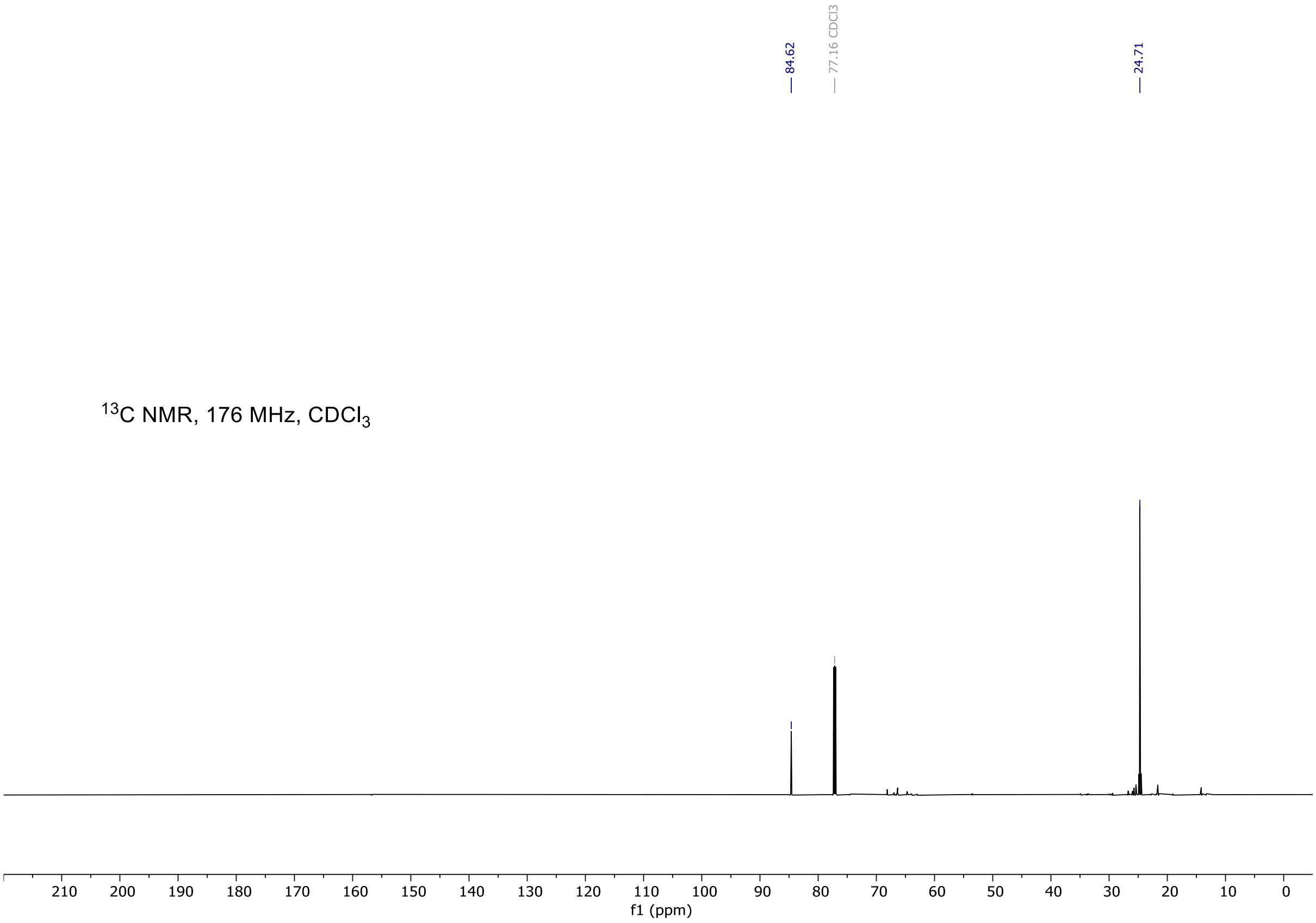

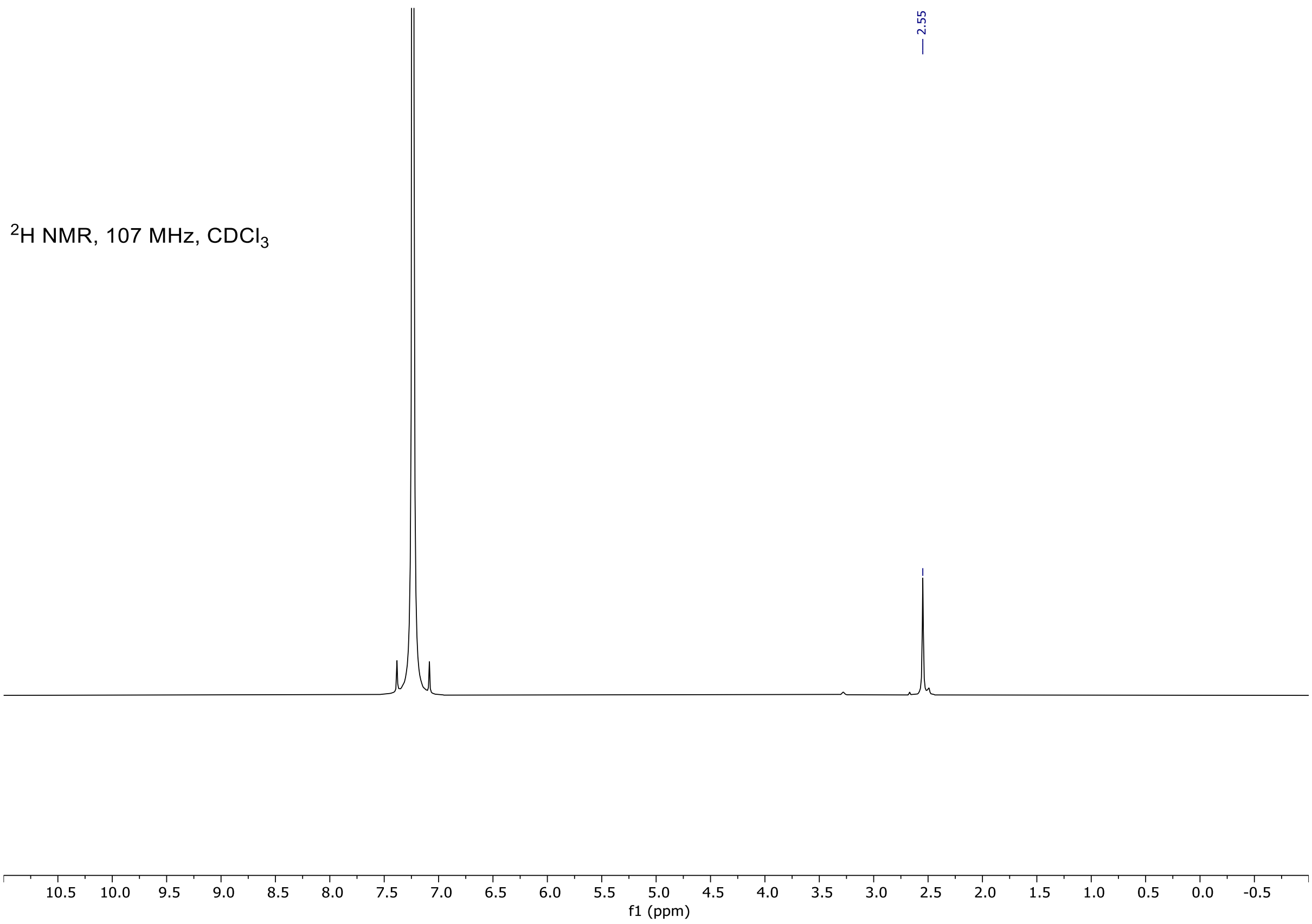

2.55
^{2}H NMR, 107 MHz, $CDCl_3$
10.5 10.0 9.5 9.0 8.5 8.0 7.5 7.0 6.5 6.0 5.5 5.0 4.5 4.0 3.5 3.0 2.5 2.0 1.5 1.0 0.5 0.0 -0.5
f1 (ppm)

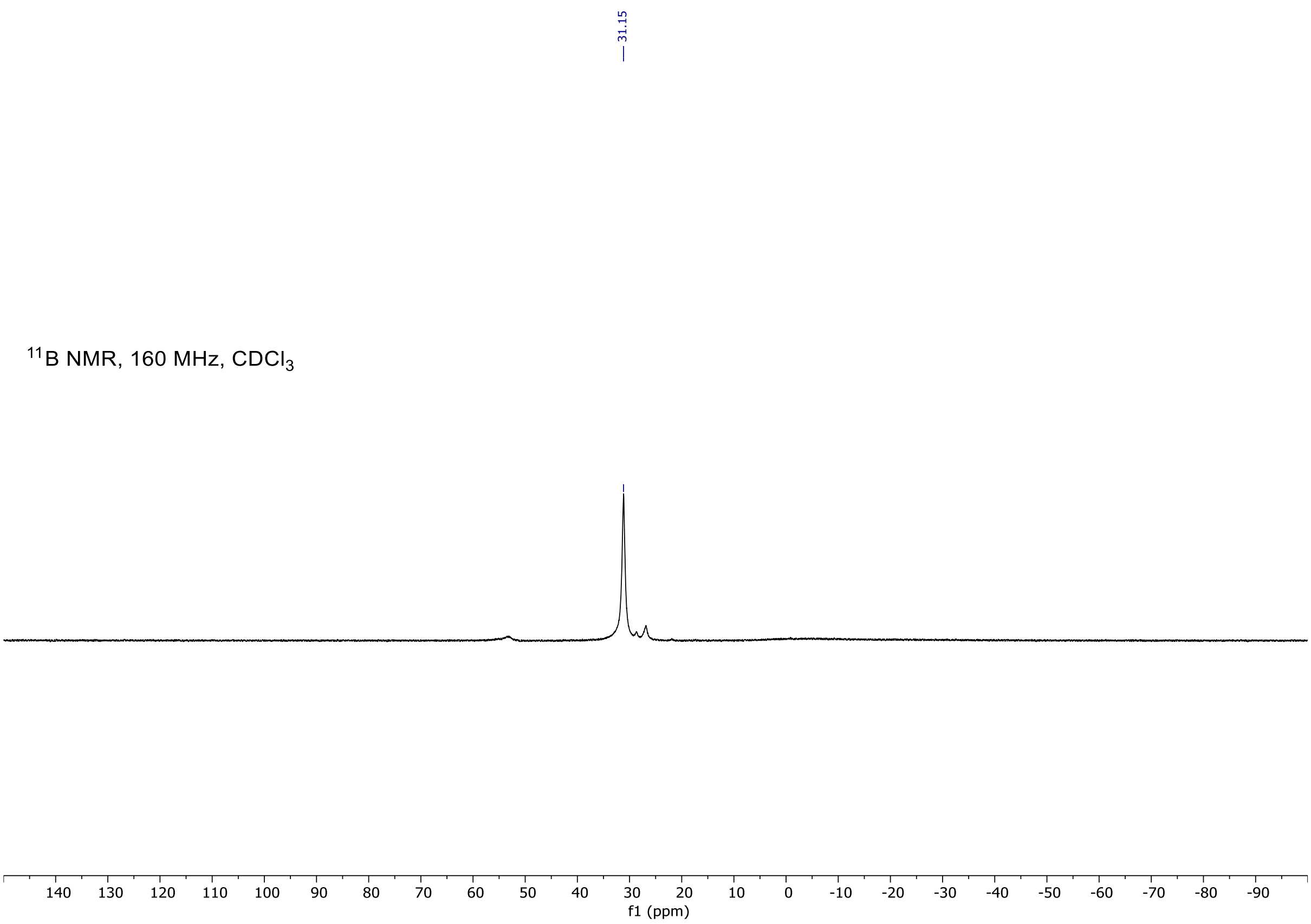

31.15
^{11}B NMR, 160 MHz, $CDCl_3$
140 130 120 110 100 90 80 70 60 50 40 30 20 10 0 -10 -20 -30 -40 -50 -60 -70 -80 -90
f1 (ppm)

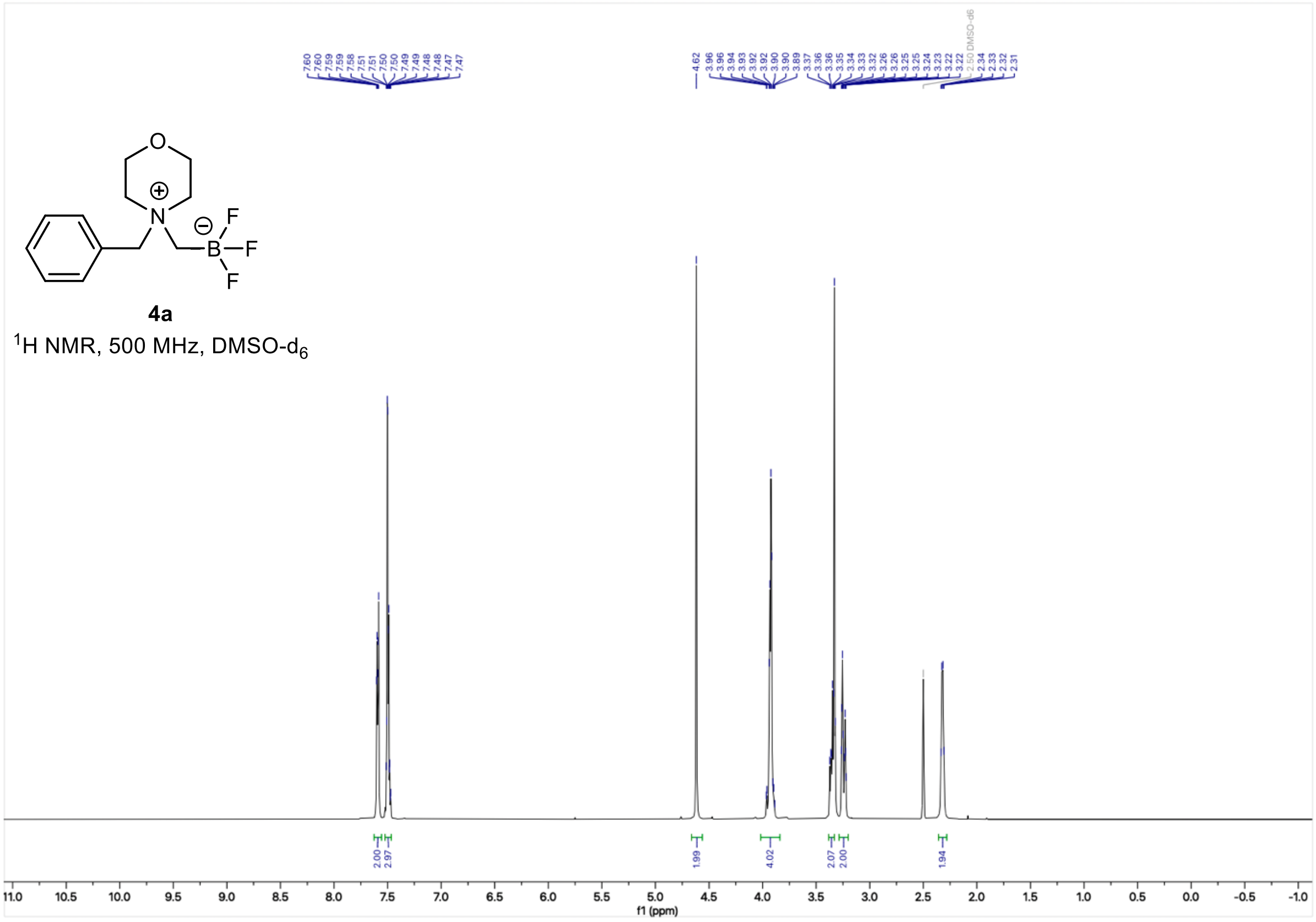

4a

^{1}H NMR, 500 MHz, DMSO-d_6

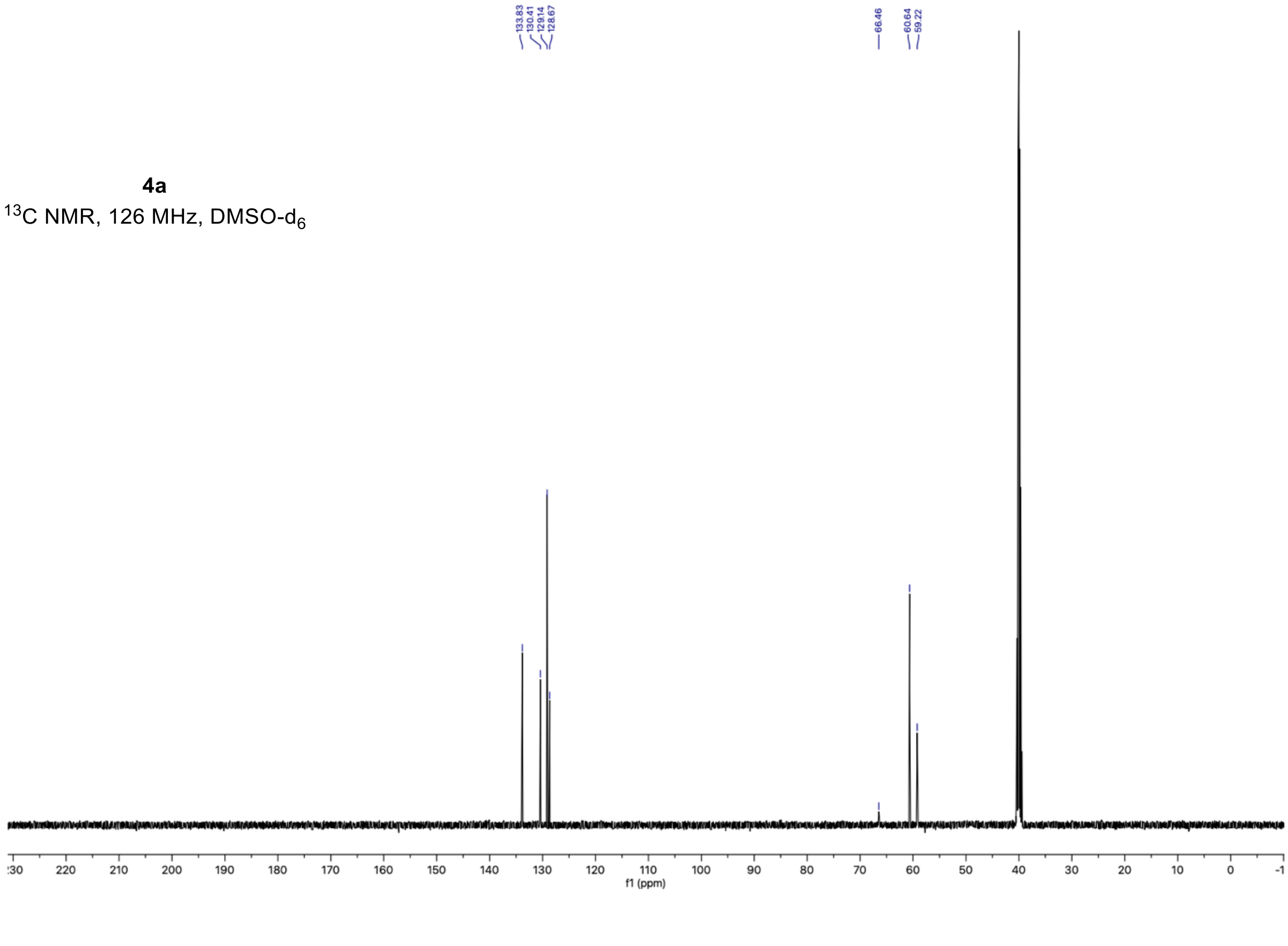

4a

^{13}C NMR, 126 MHz, DMSO-d_6

4a

^{19}F NMR, 471 MHz, DMSO-d_6

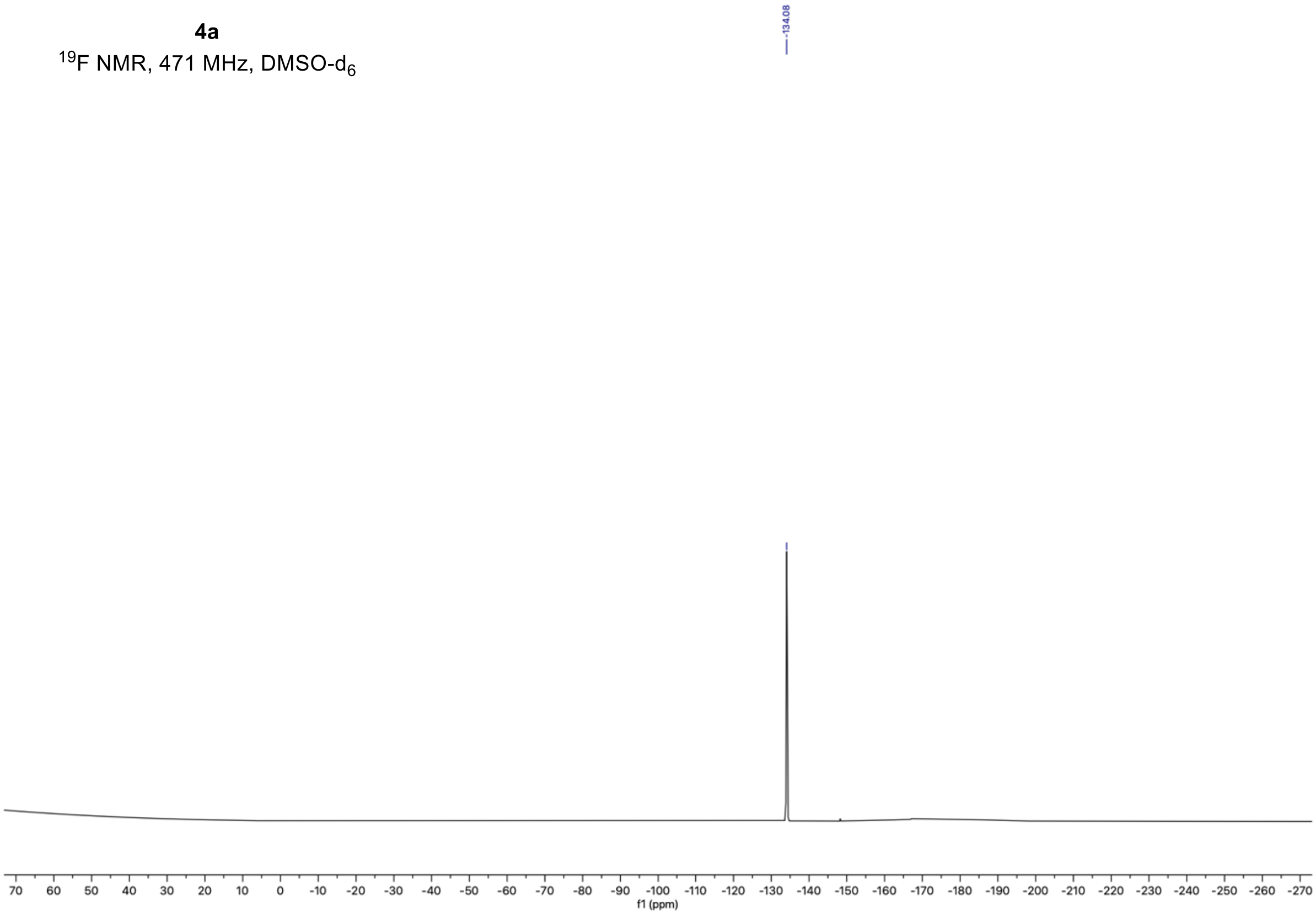

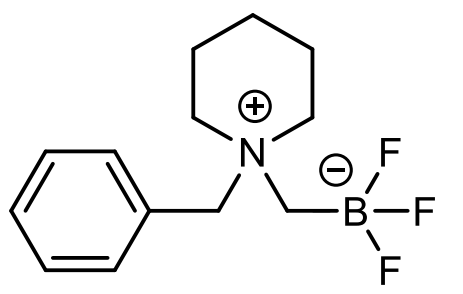

4b

^{1}H NMR, 400 MHz, DMSO-d_6

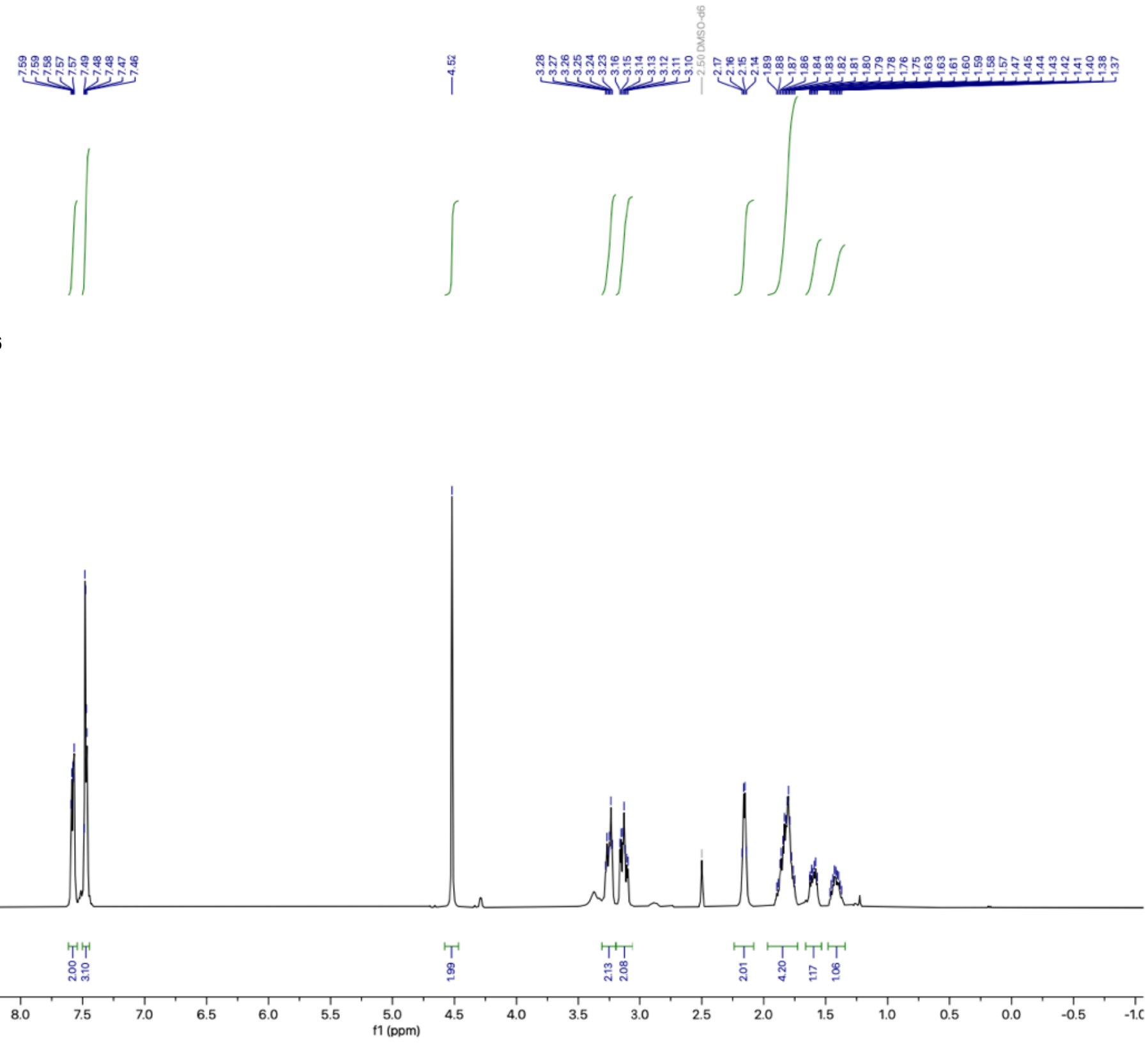

4b

^{13}C NMR, 101 MHz, DMSO-d_6

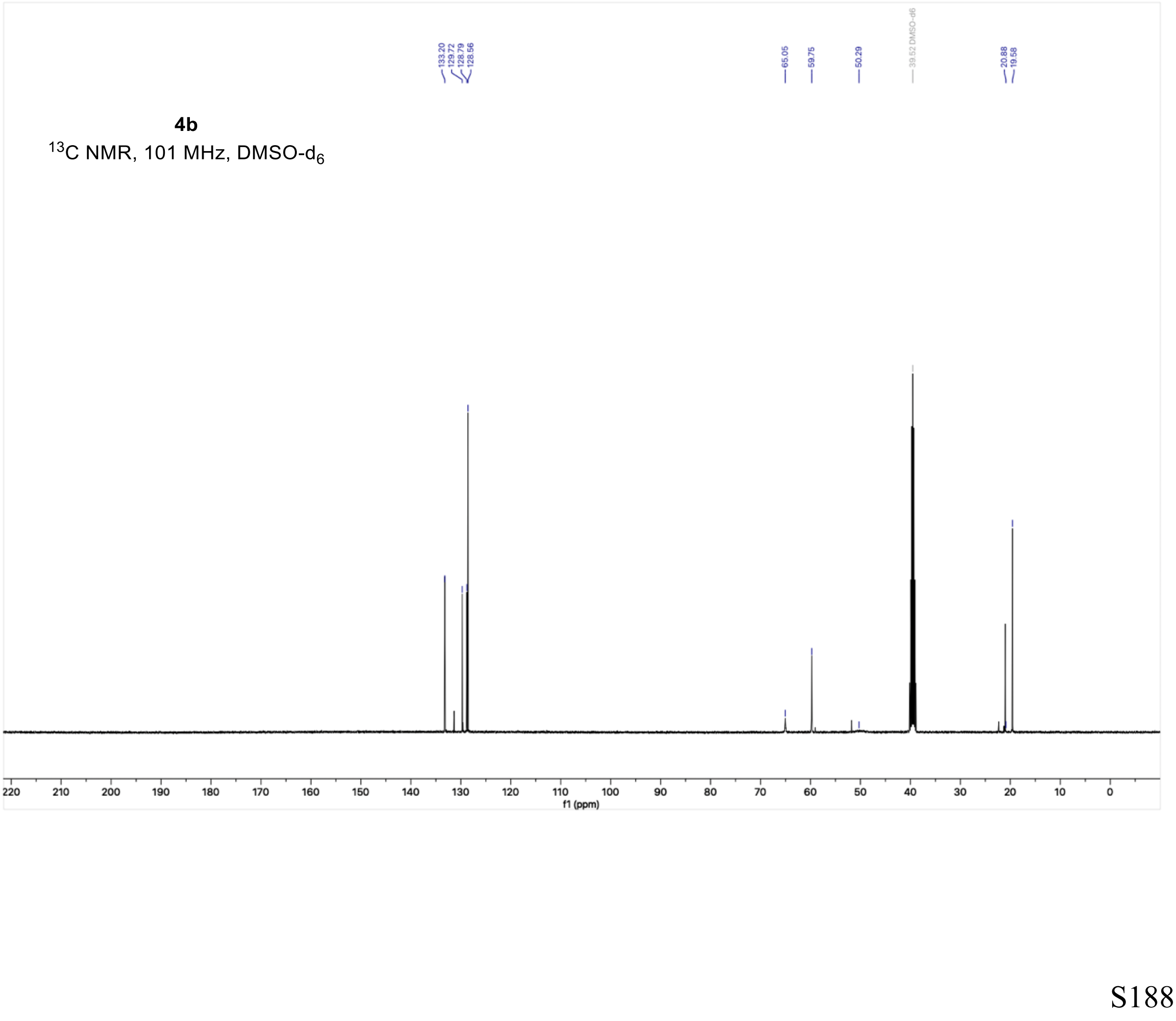

4b

^{19}F NMR, 376 MHz, DMSO-d_6

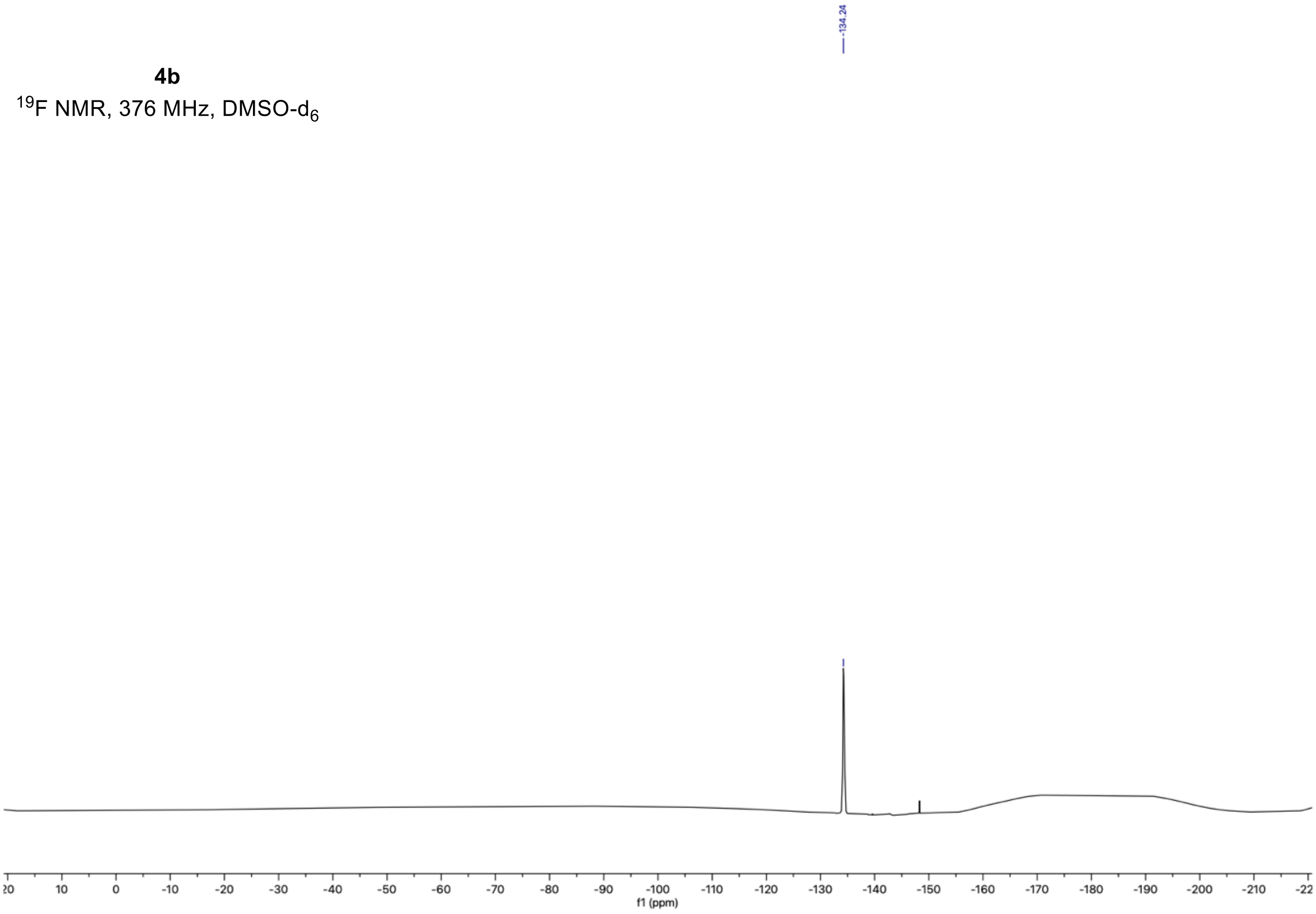

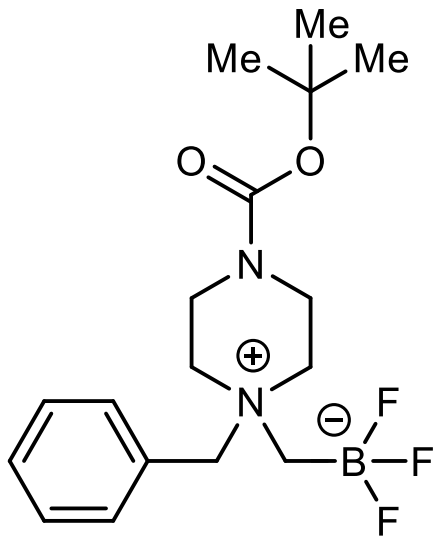

4c

^{1}H NMR, 500 MHz, DMSO-d_6

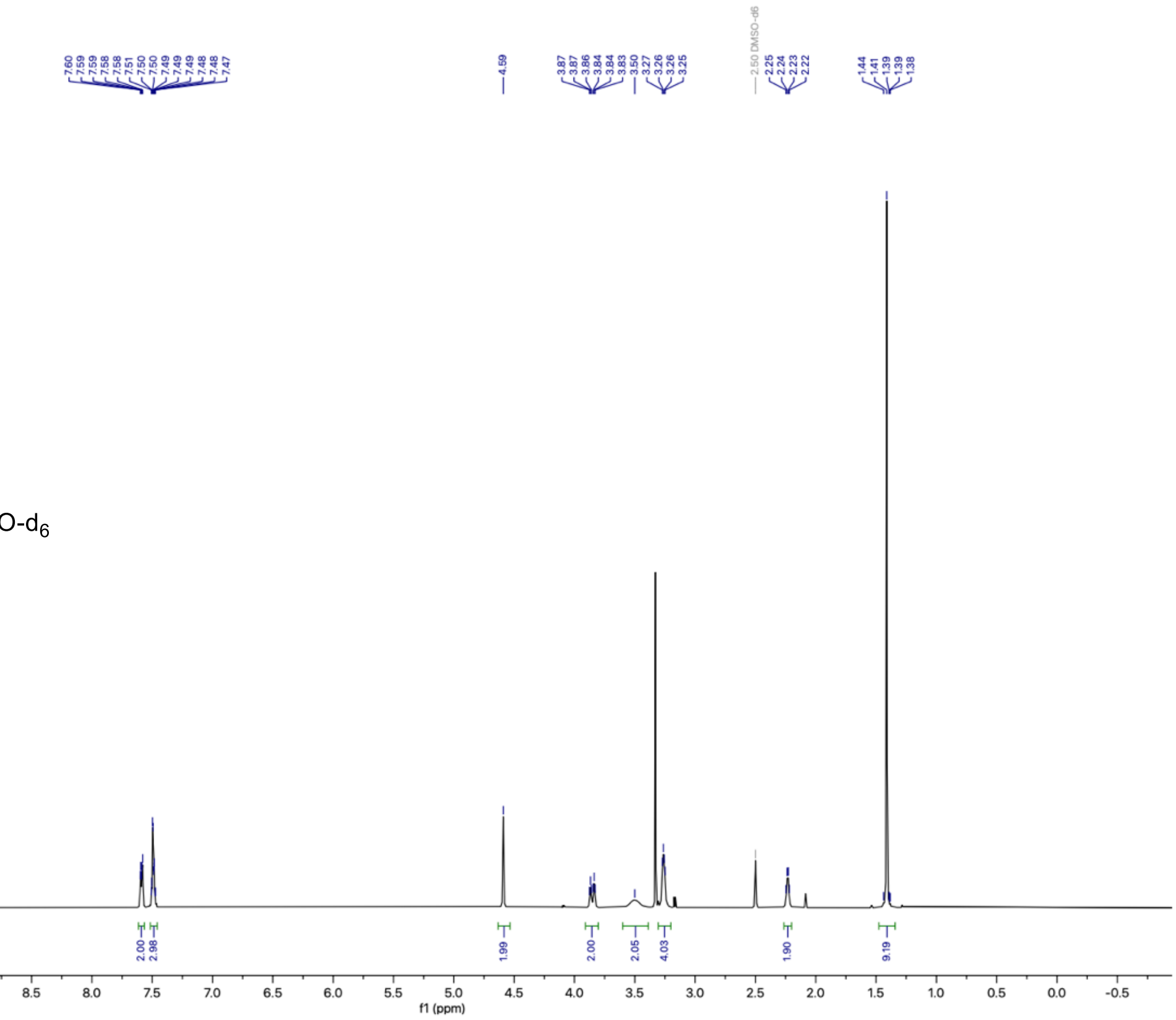

4c

^{13}C NMR, 126 MHz, DMSO-d_6

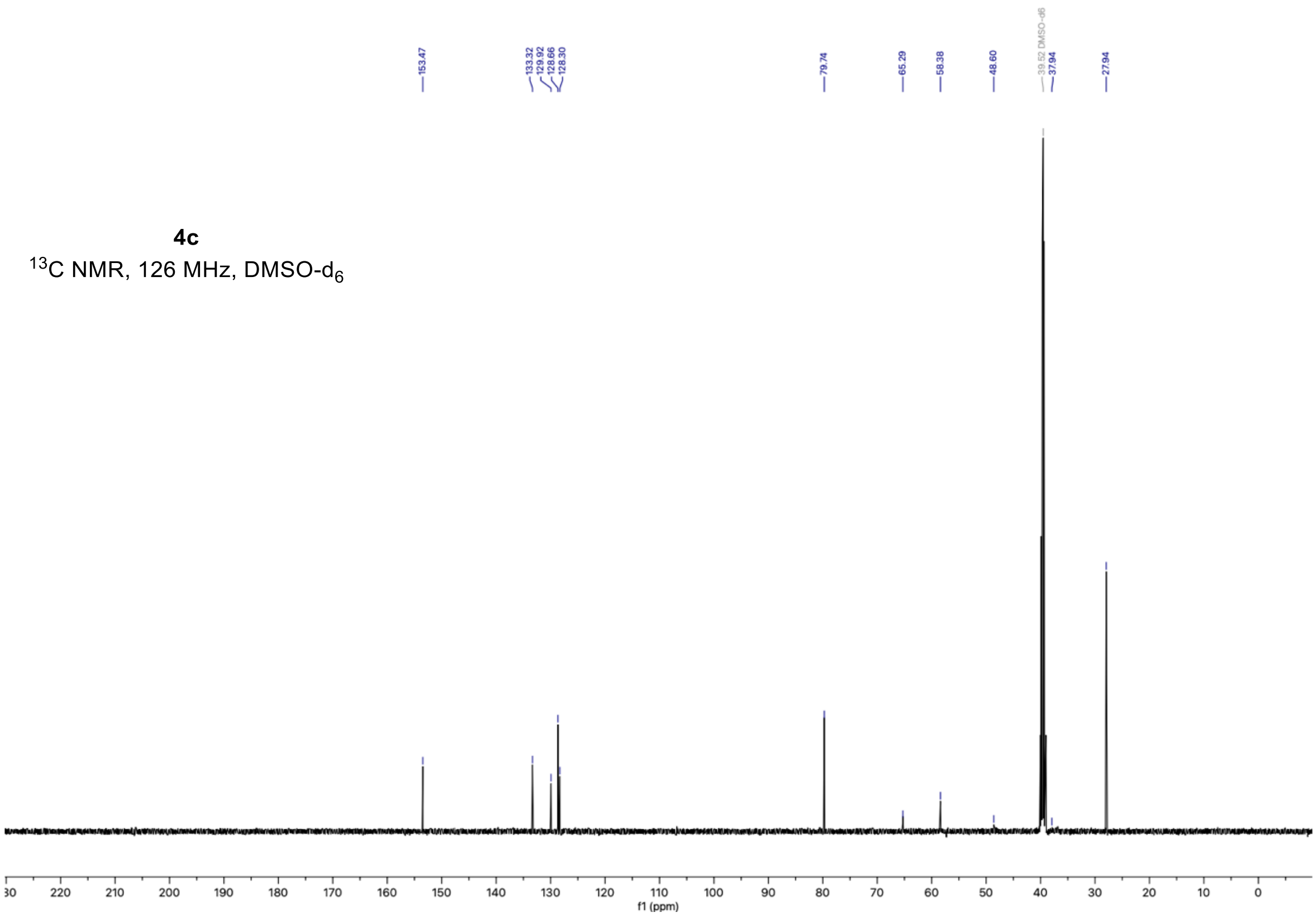

4c

^{19}F NMR, 471 MHz, DMSO-d_6

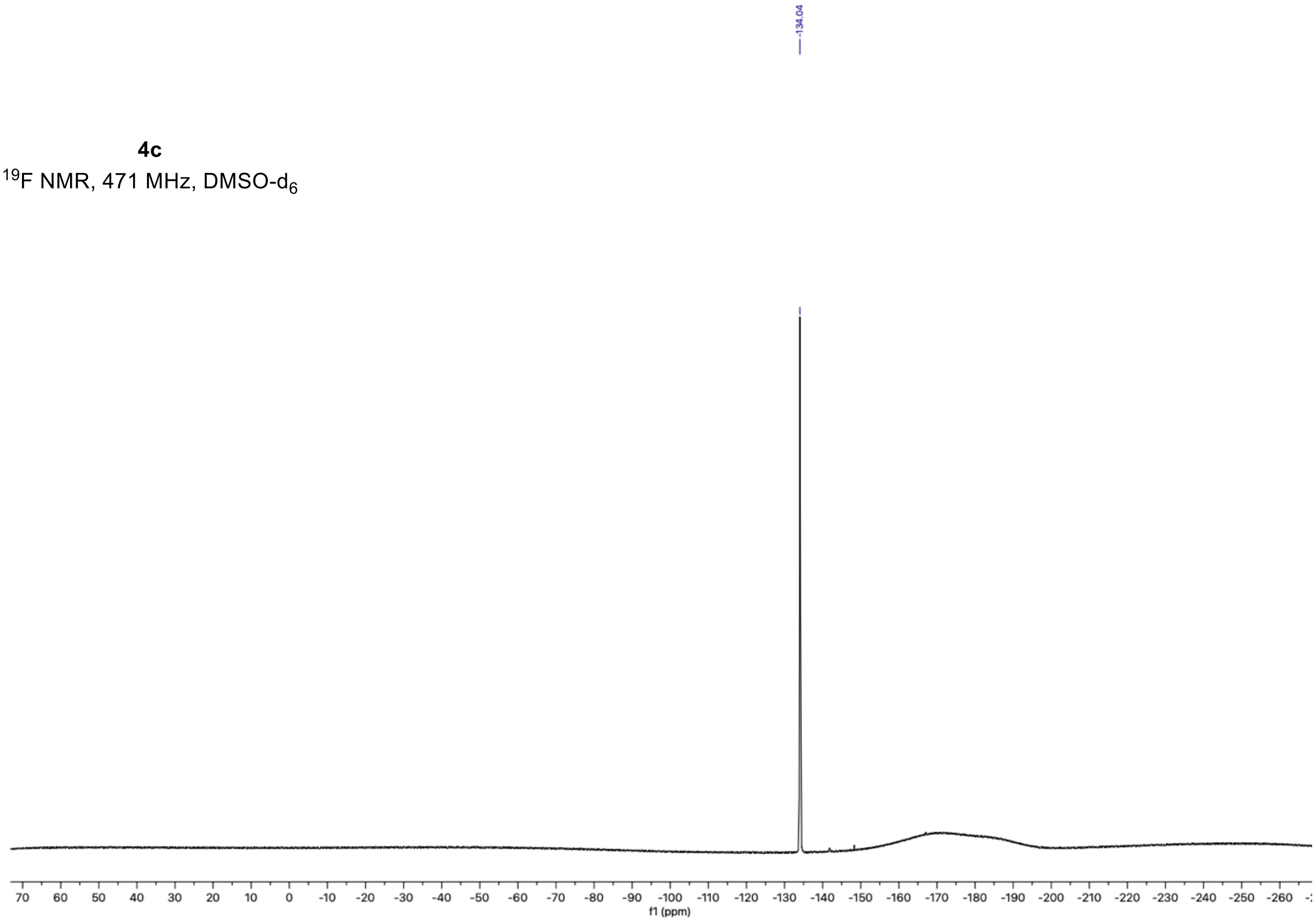

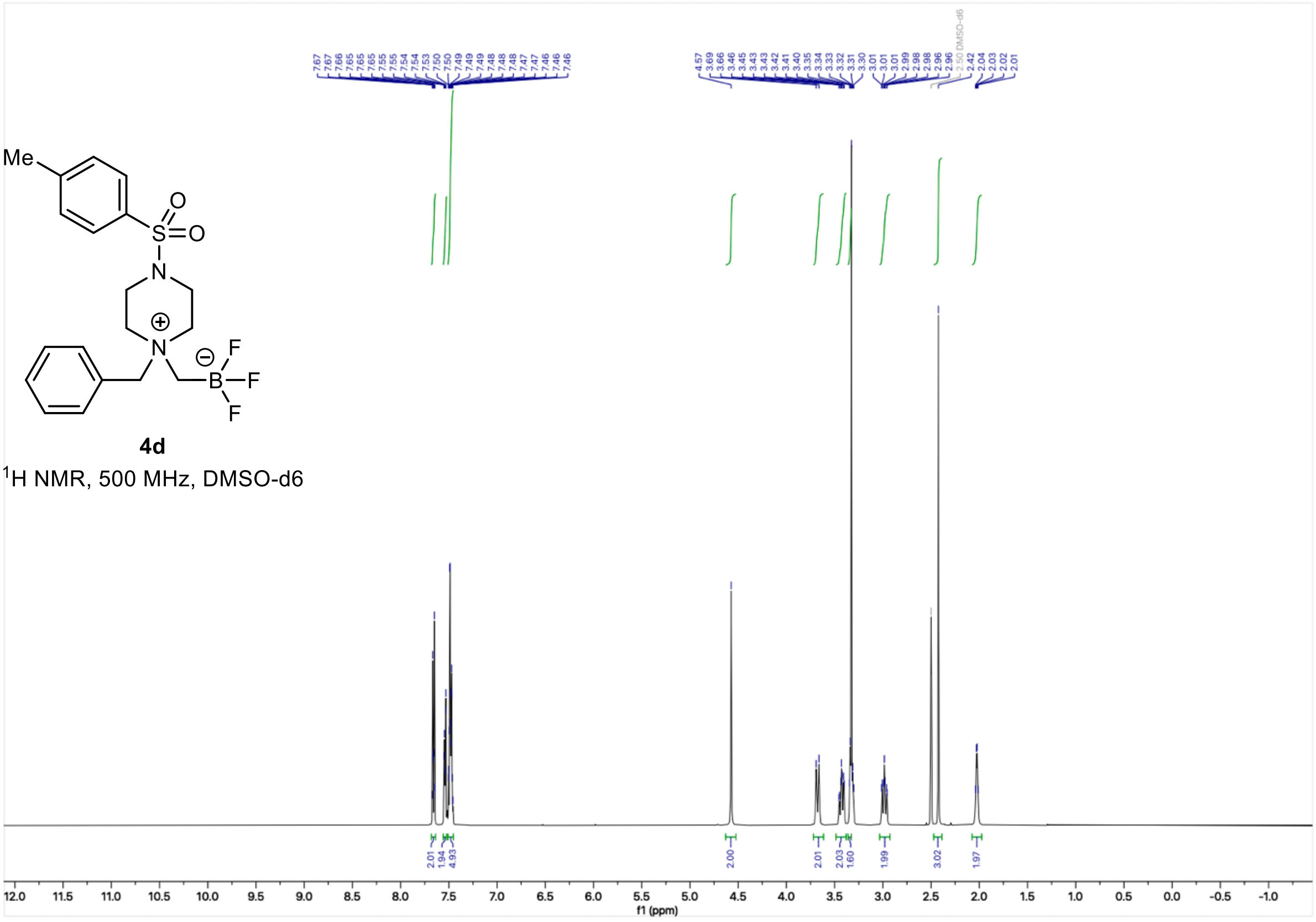
Me
4d
1H NMR, 500 MHz, DMSO-d6
f1 (ppm)

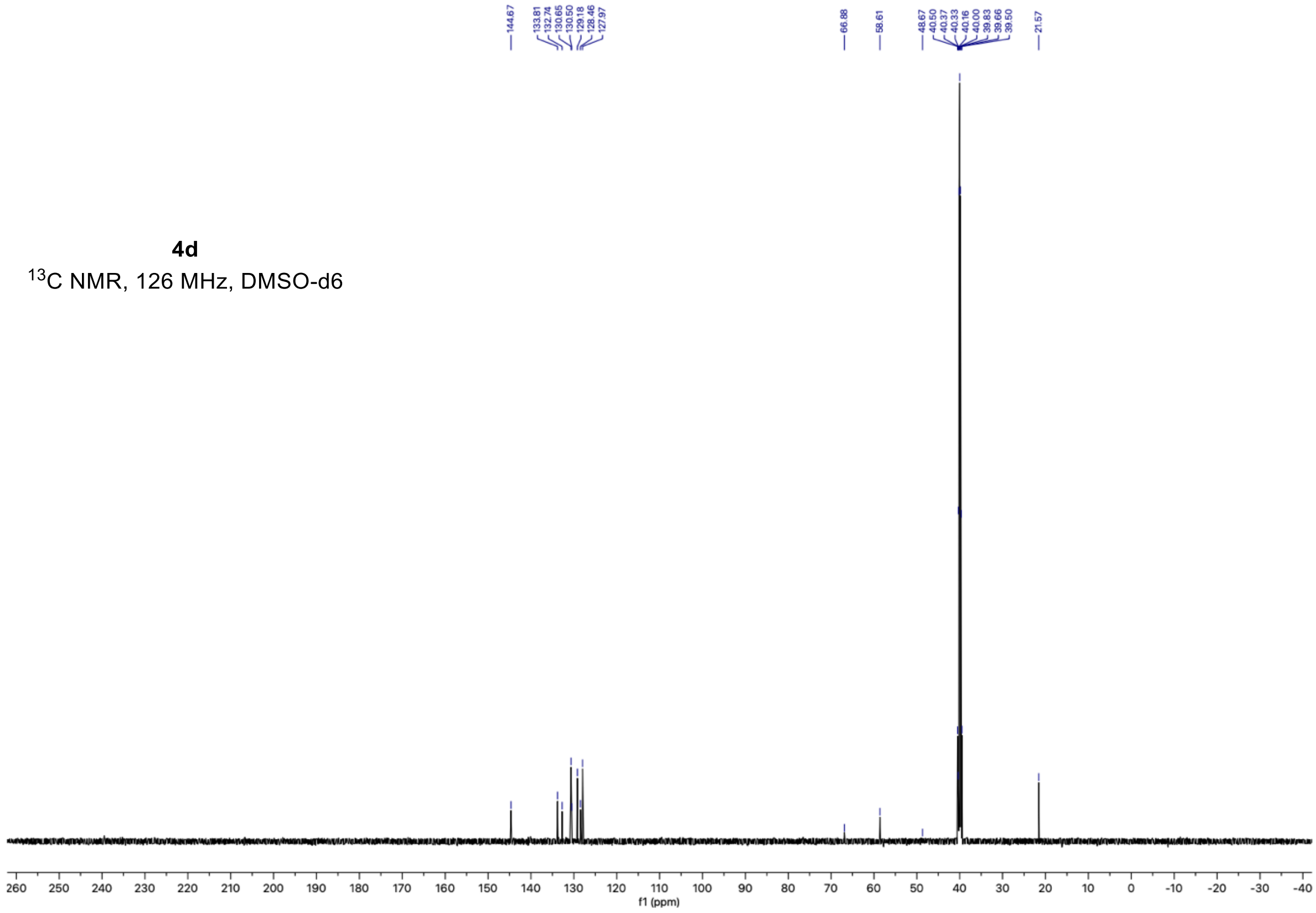
4d
13C NMR, 126 MHz, DMSO-d6
f1 (ppm)

4d

^{19}F NMR, 471 MHz, DMSO-d6

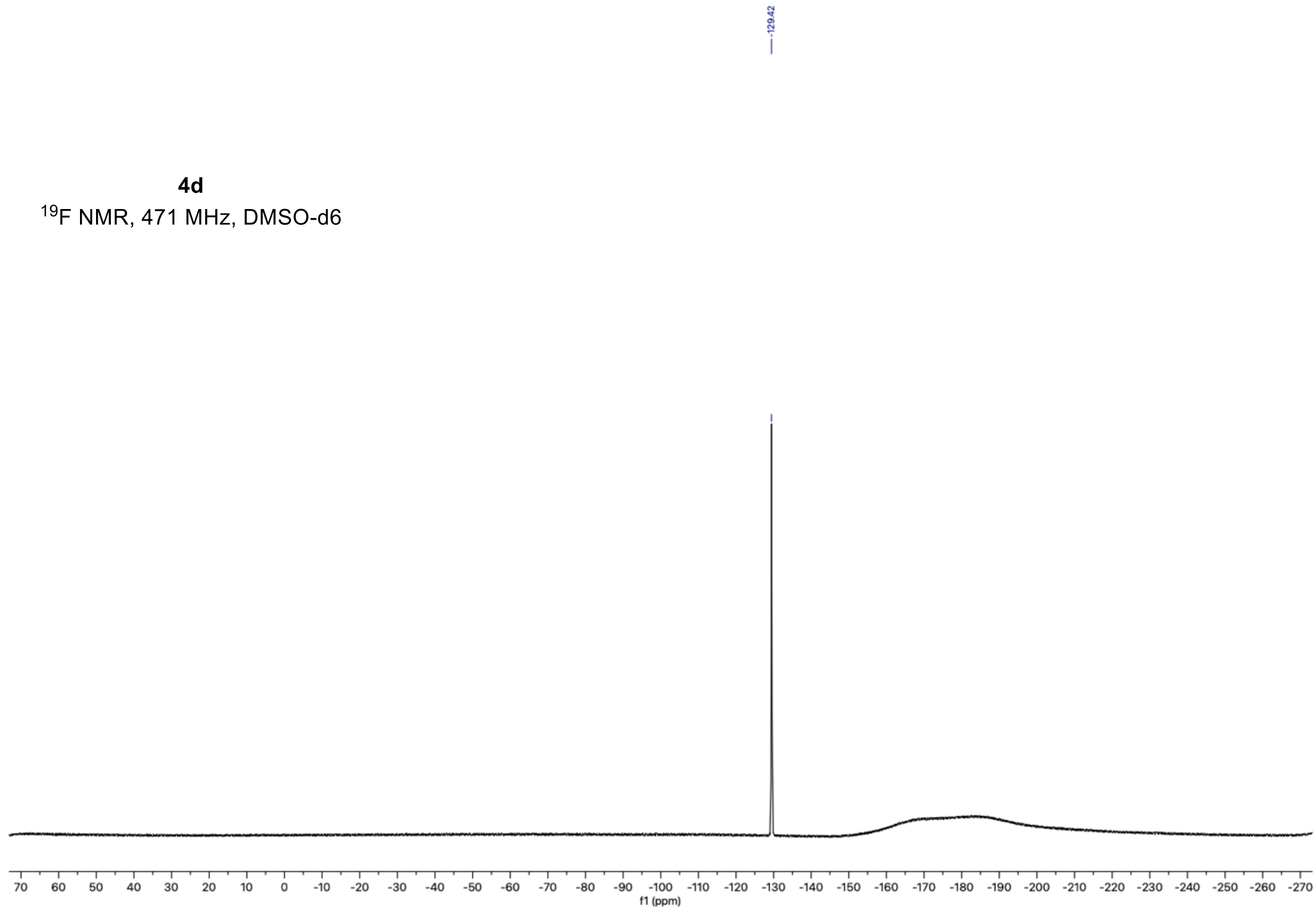

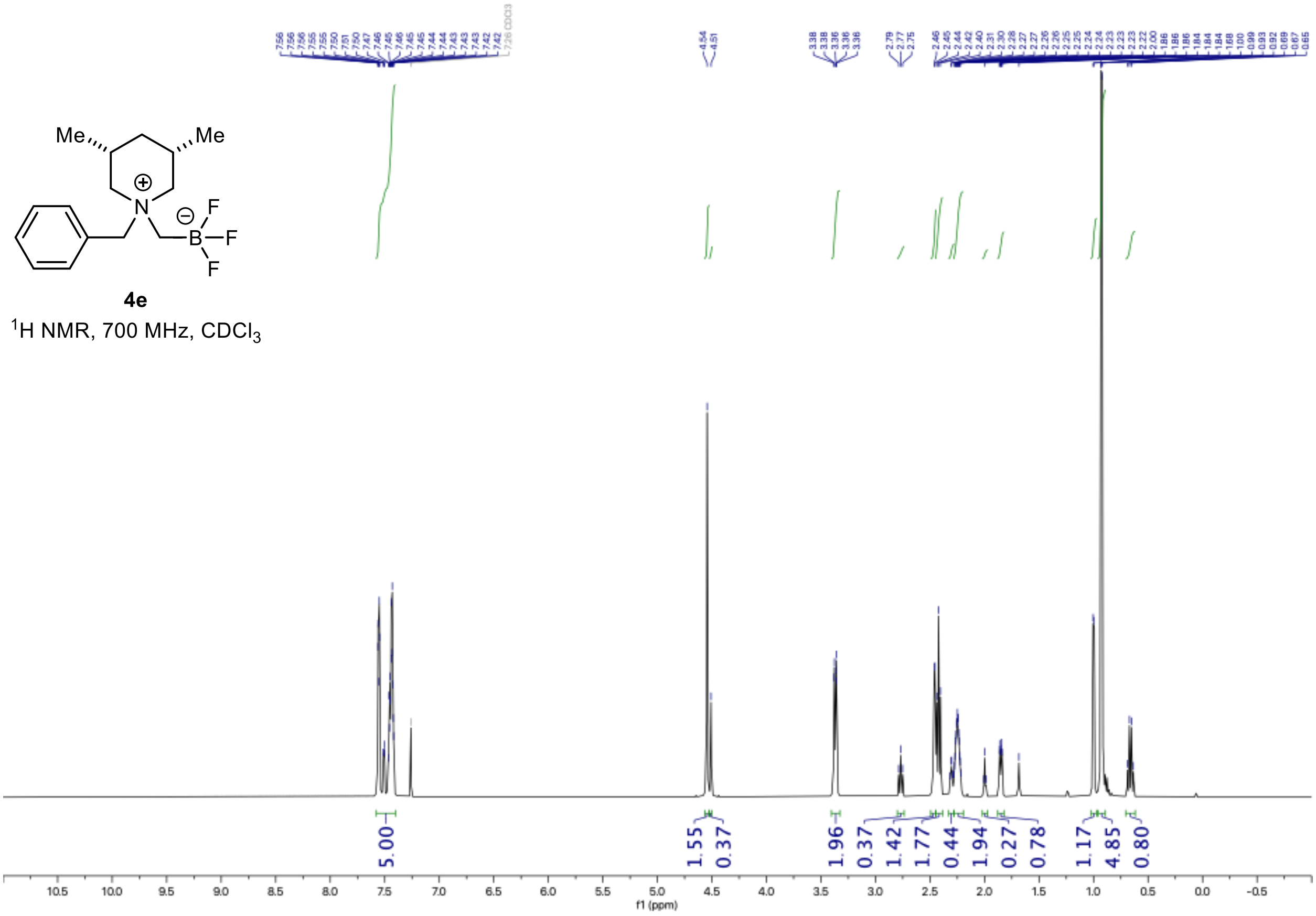

4e

^{1}H NMR, 700 MHz, $CDCl_3$

4e

^{13}C NMR, 176 MHz, $CDCl_3$

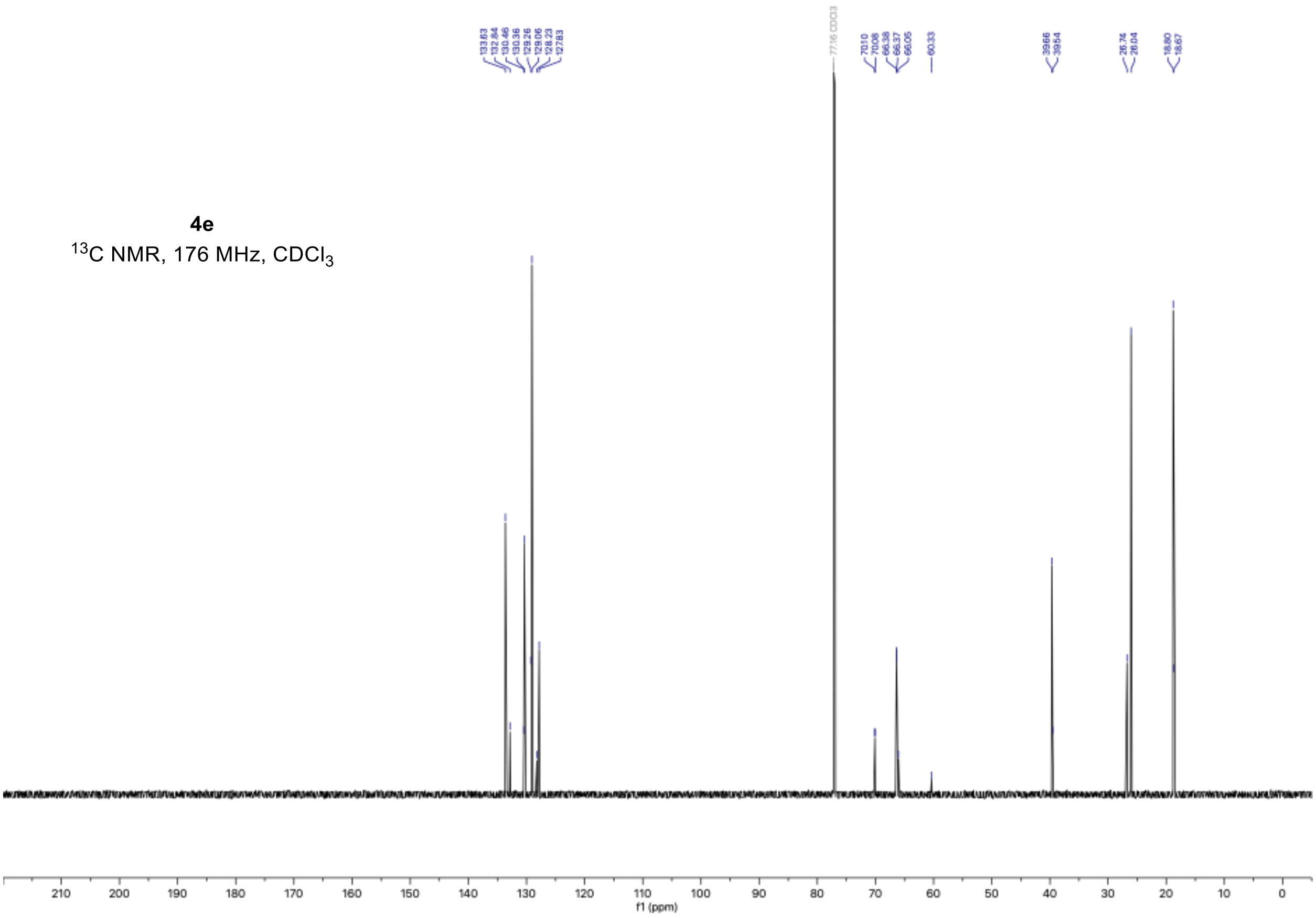

4e
^{19}F NMR, 376 MHz, $CDCl_3$

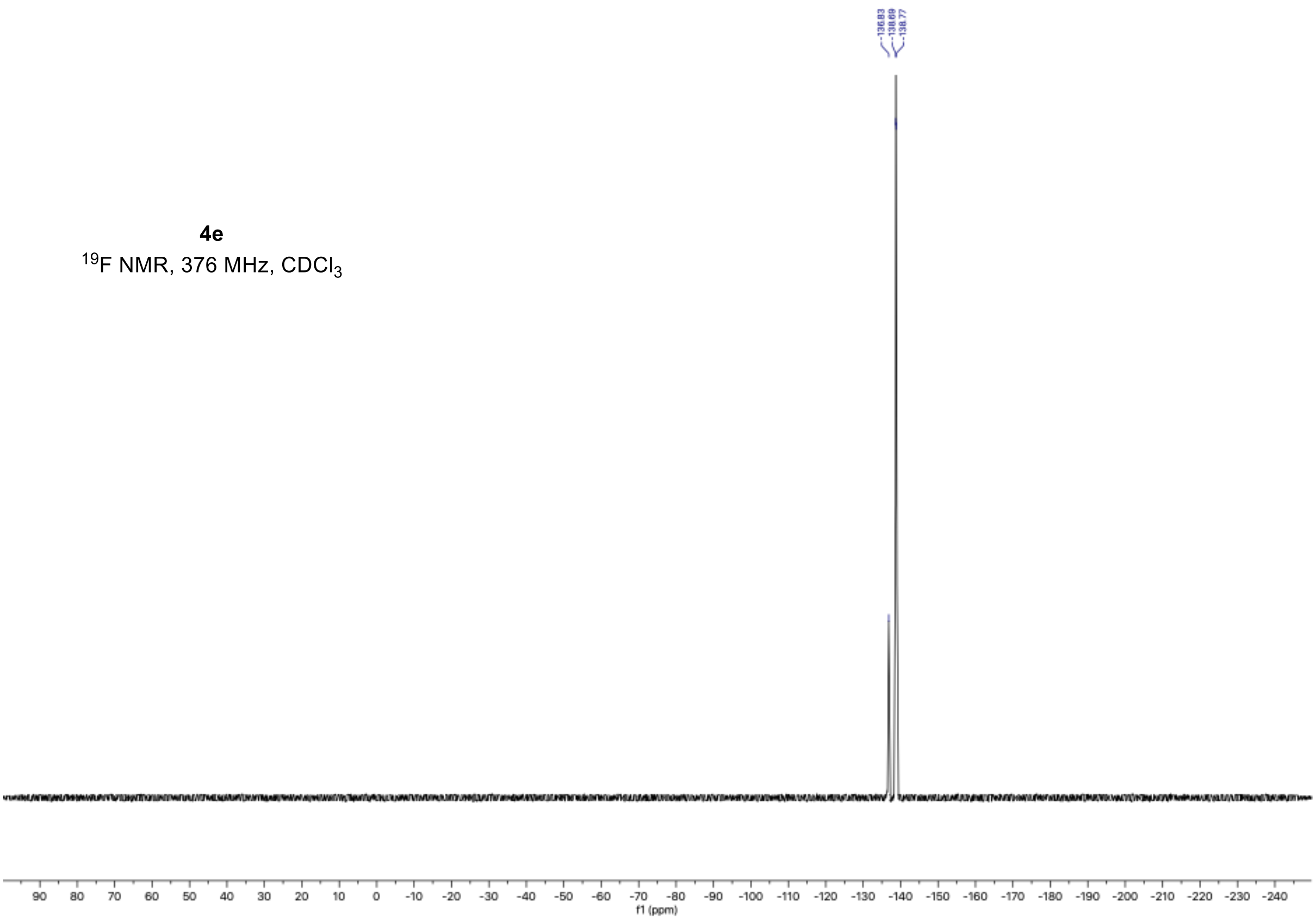

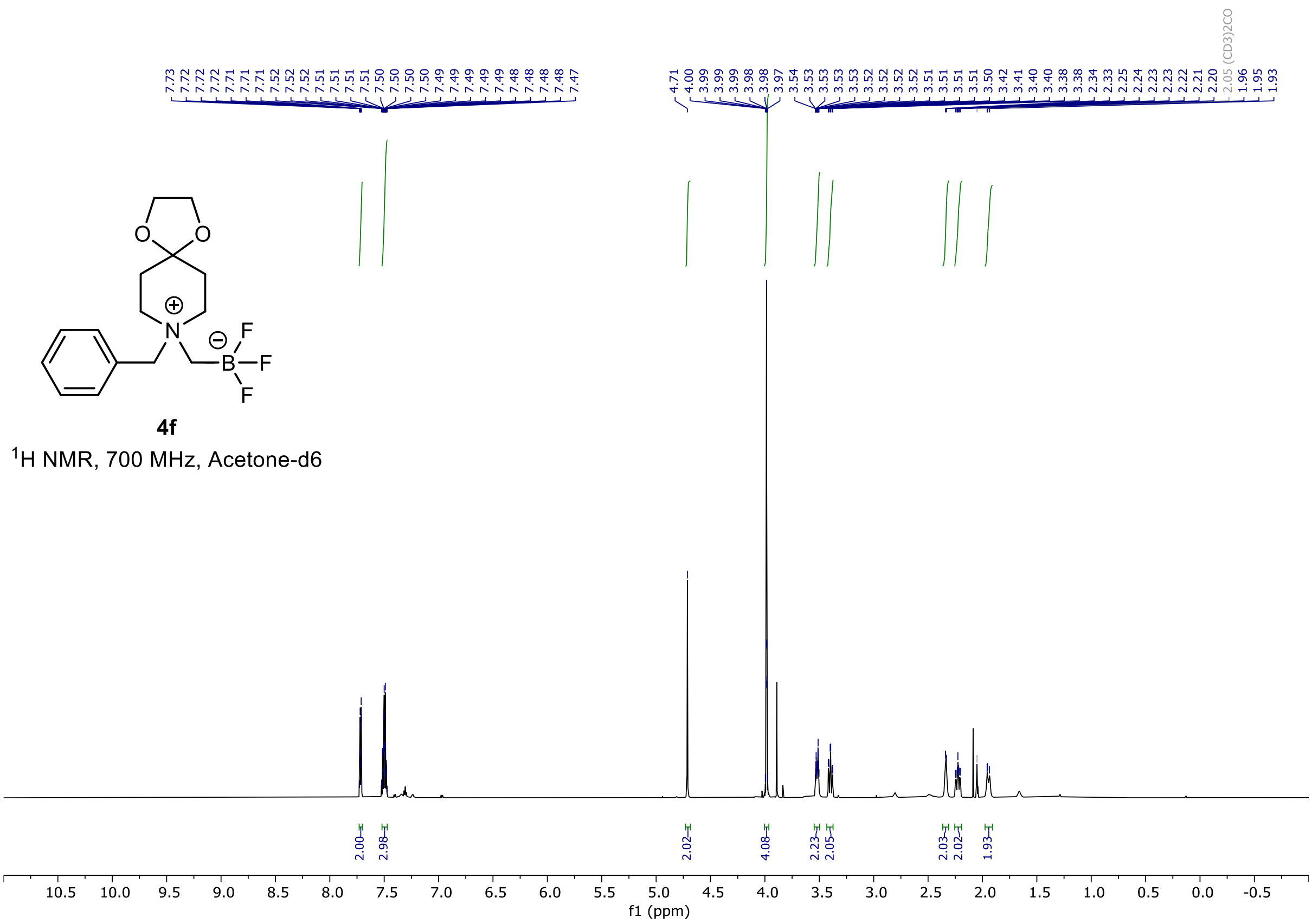
4f
^{1}H NMR, 700 MHz, Acetone-d6
f1 (ppm)

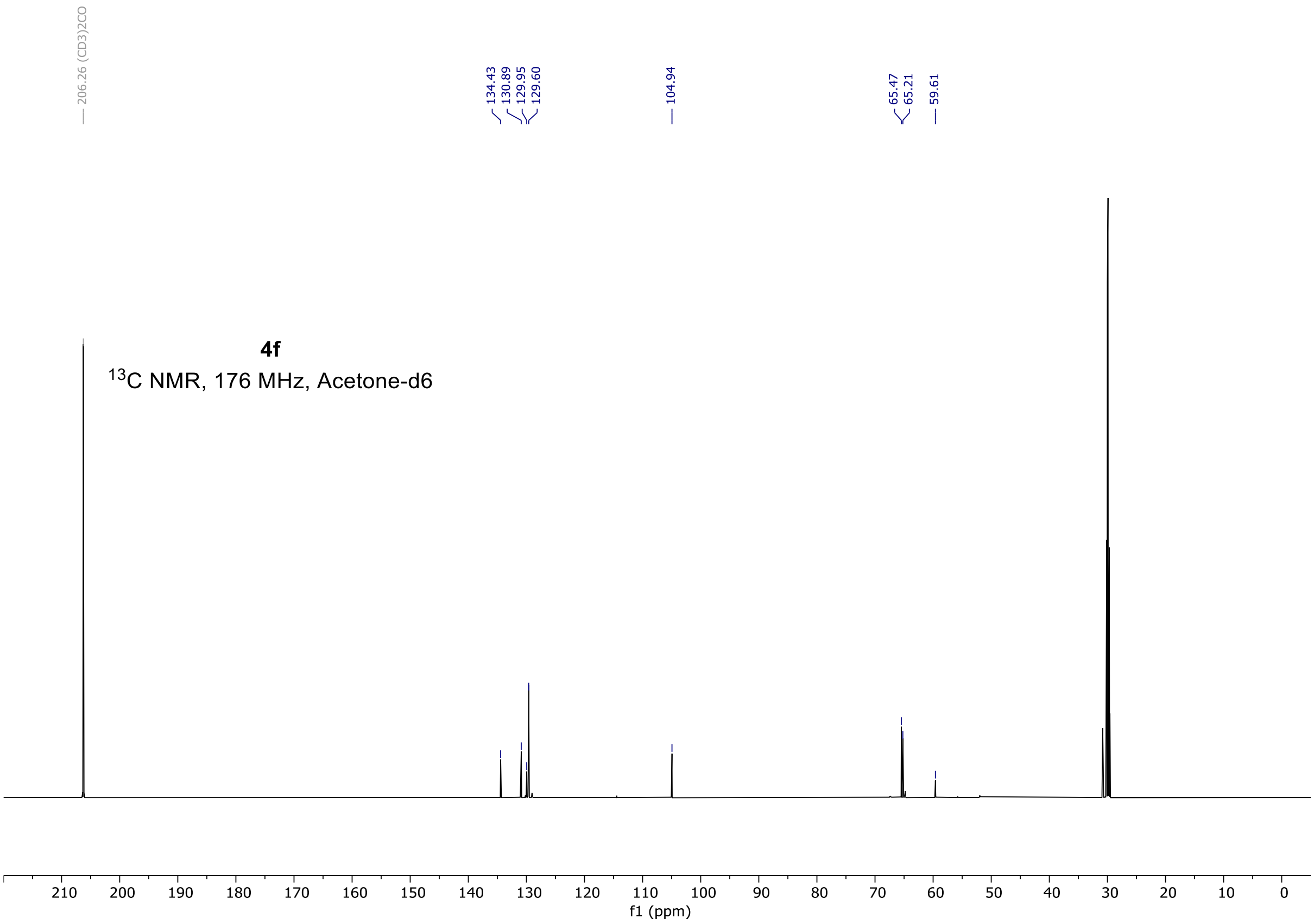
4f
^{13}C NMR, 176 MHz, Acetone-d6
206.26 (CD3)2CO
134.43
130.89
129.95
129.60
104.94
65.47
65.21
59.61
f1 (ppm)

4f

^{19}F NMR, 376 MHz, Acetone-d6

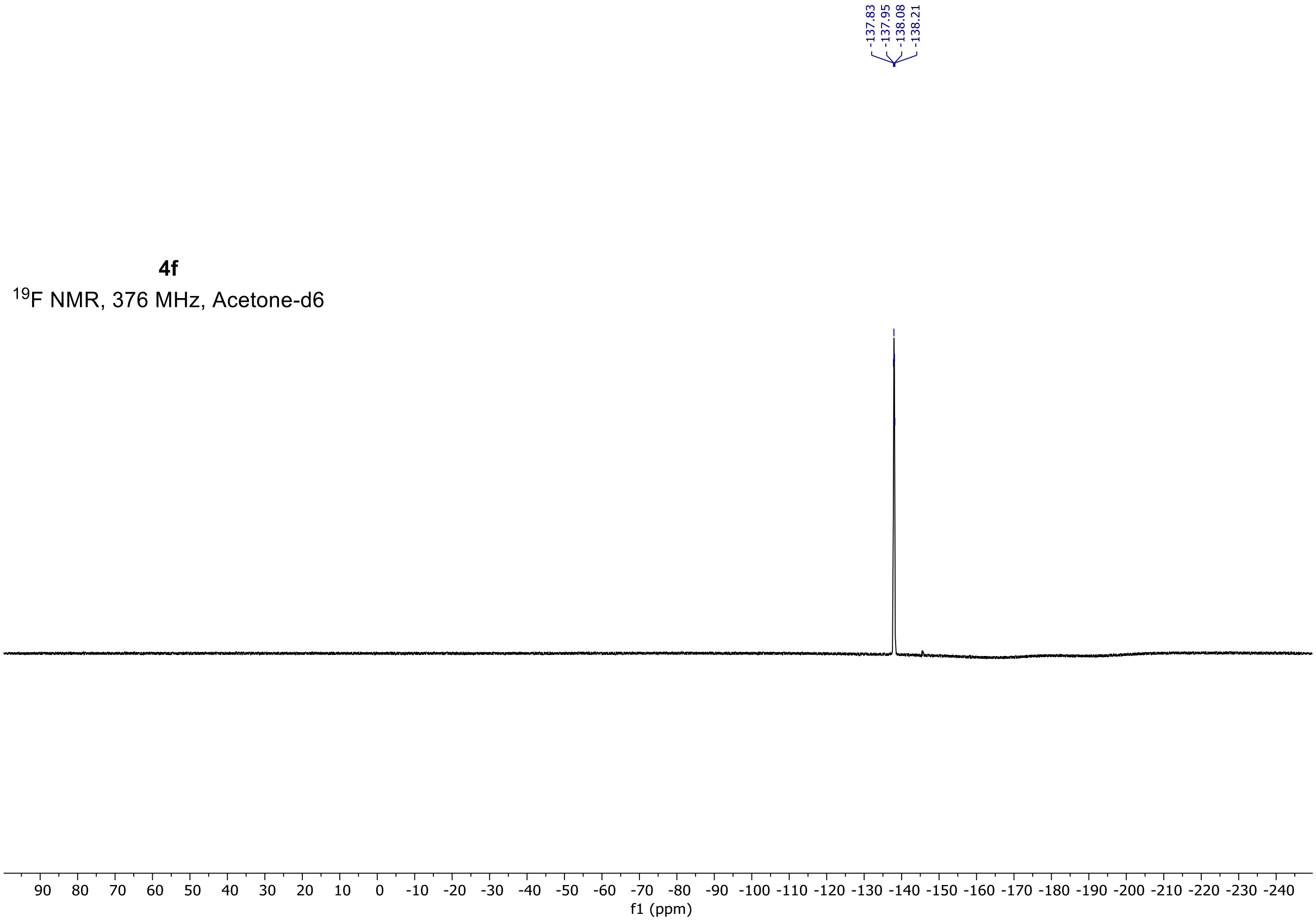

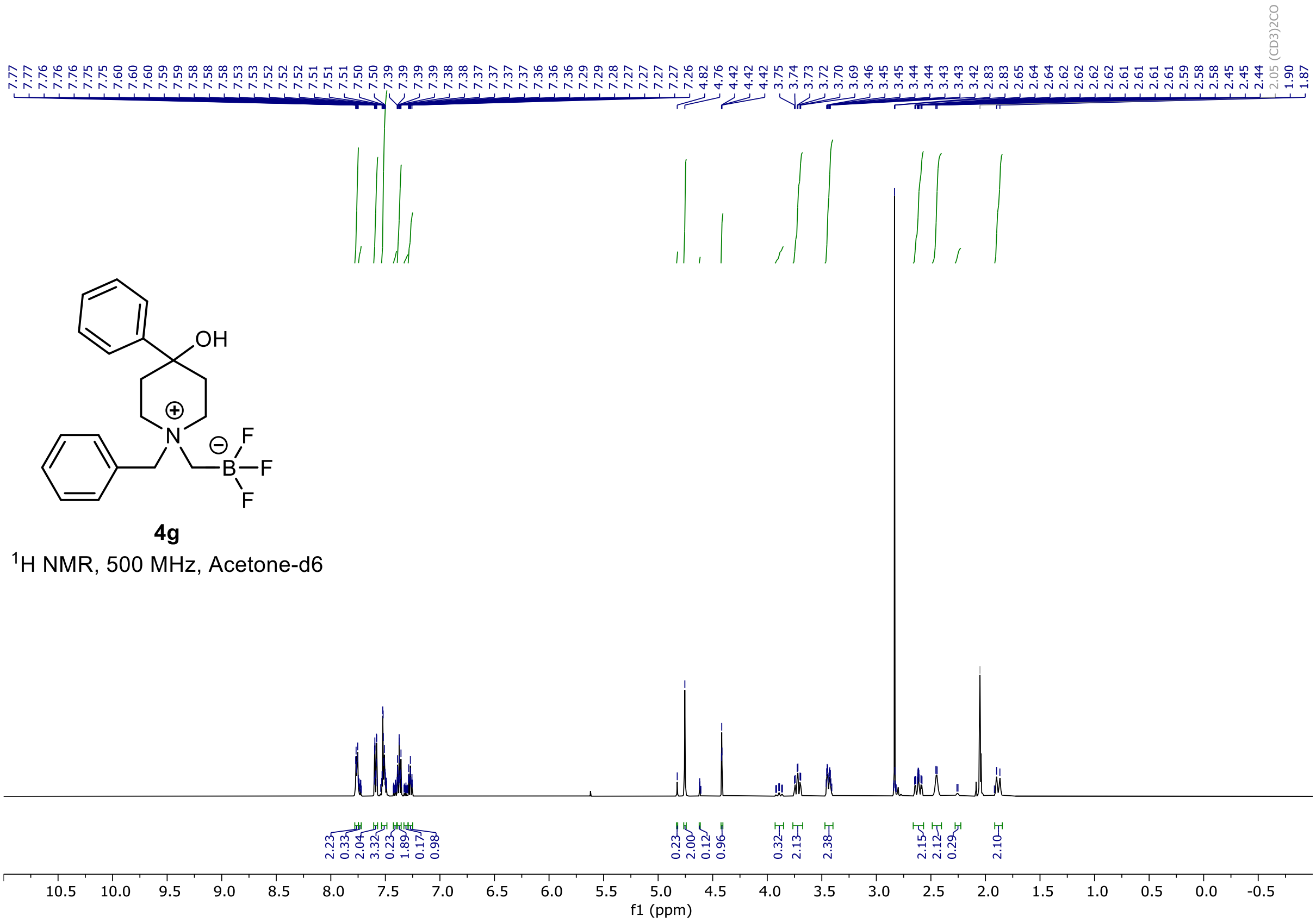
7.77 7.77 7.76 7.76 7.76 7.75 7.75 7.60 7.60 7.59 7.59 7.58 7.58 7.58 7.53 7.52 7.52 7.51 7.51 7.50 7.50 7.39 7.39 7.39 7.38 7.38 7.37 7.37 7.37 7.36 7.36 7.29 7.29 7.28 7.27 7.27 7.27 7.26 4.82 4.76 4.42 4.42 4.42 3.75 3.74 3.73 3.72 3.70 3.69 3.46 3.45 3.45 3.44 3.43 3.42 2.83 2.83 2.65 2.64 2.62 2.62 2.62 2.62 2.61 2.61 2.61 2.59 2.58 2.58 2.45 2.45 2.44 2.05 (CD3)2CO 1.90 1.87
OH
F
F
F
B
N
4g
1H NMR, 500 MHz, Acetone-d6
2.23 0.33 2.04 3.32 0.23 1.89 0.17 0.98 0.23 2.00 0.12 0.96 0.32 2.13 2.38 2.15 2.12 0.29 2.10
10.5 10.0 9.5 9.0 8.5 8.0 7.5 7.0 6.5 6.0 5.5 5.0 4.5 4.0 3.5 3.0 2.5 2.0 1.5 1.0 0.5 0.0 -0.5
f1 (ppm)

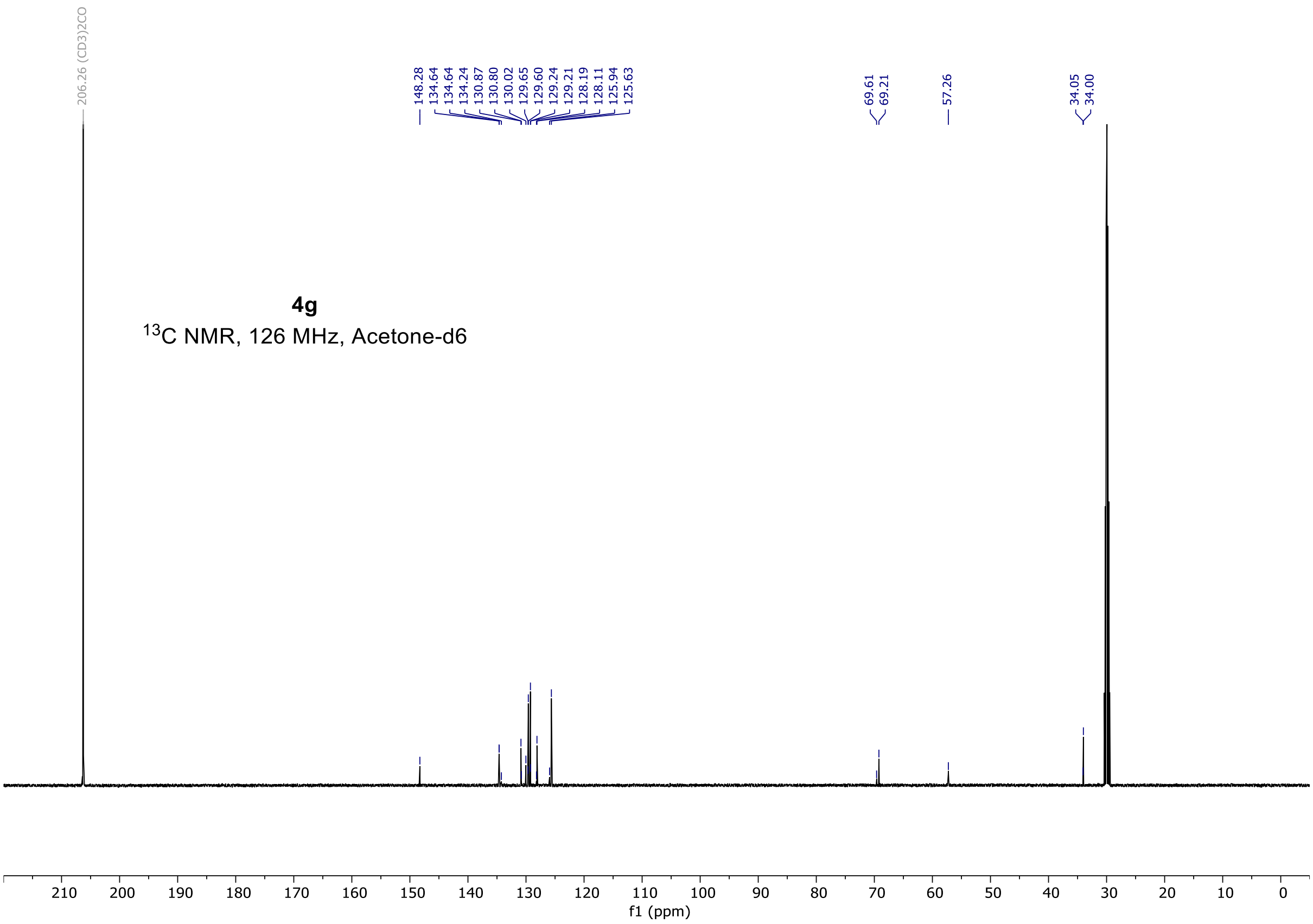
206.26 (CD3)2CO
148.28 134.64 134.64 134.24 130.87 130.80 130.02 129.65 129.60 129.24 129.21 128.19 128.11 125.94 125.63
69.61 69.21
57.26
34.05 34.00
4g
13C NMR, 126 MHz, Acetone-d6
210 200 190 180 170 160 150 140 130 120 110 100 90 80 70 60 50 40 30 20 10 0
f1 (ppm)

4g
^{19}F NMR, 471 MHz, Acetone-d6

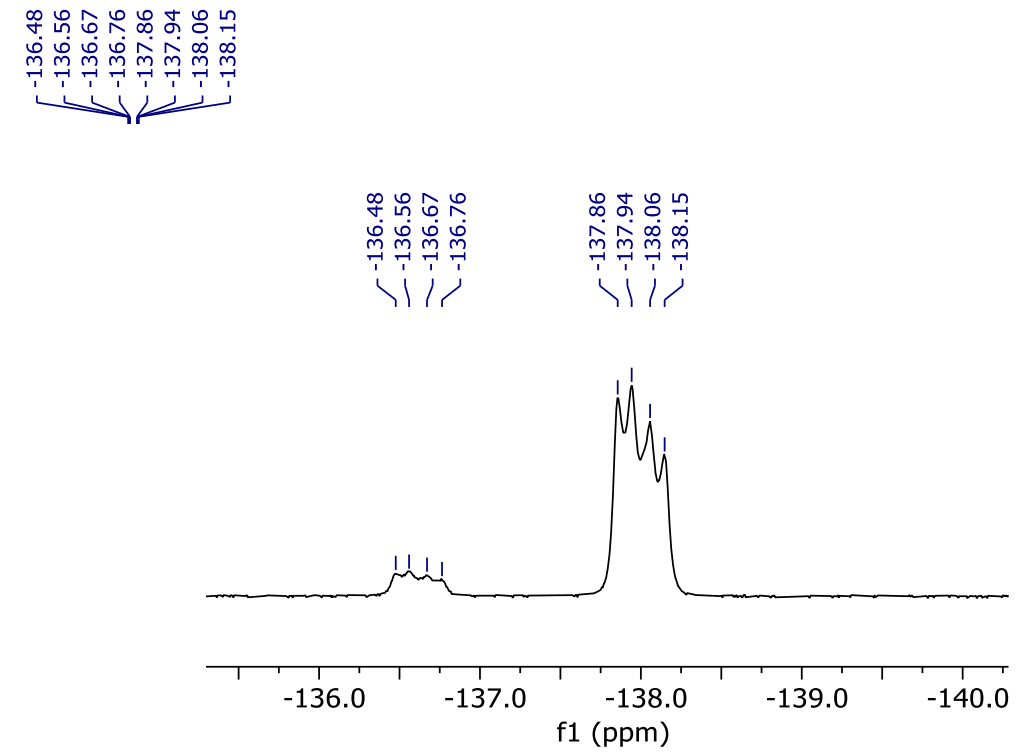

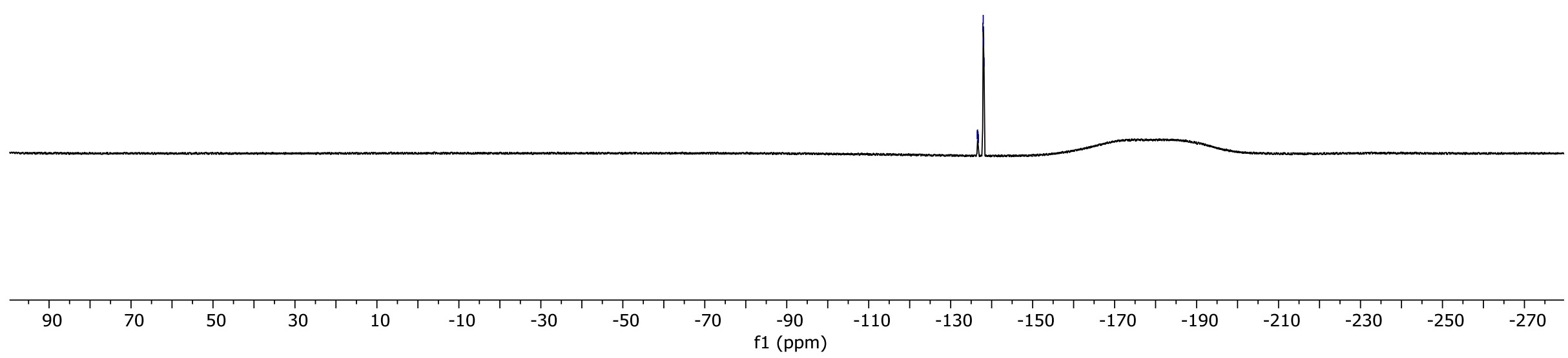

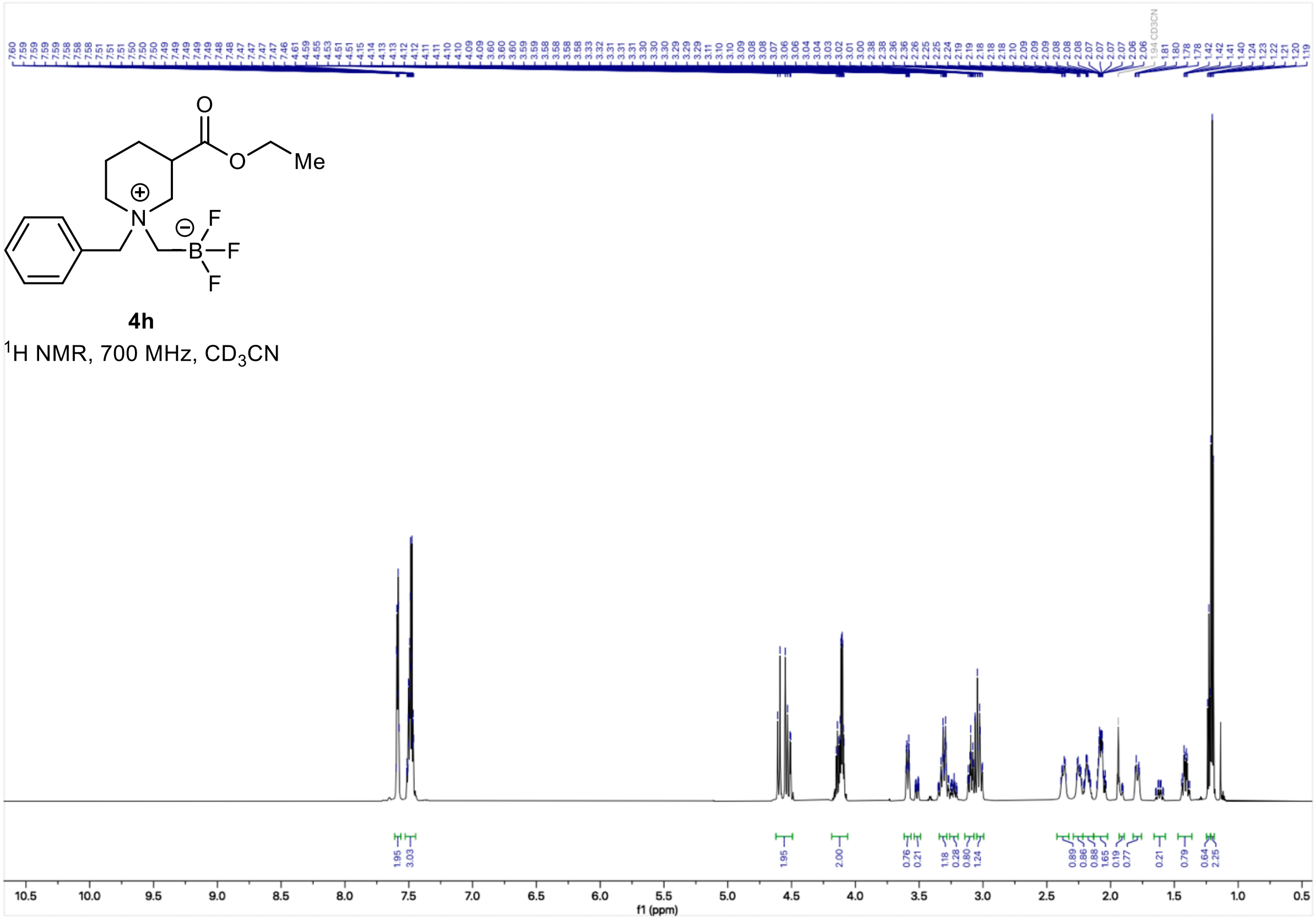
O
O
Me
N
B
F
F
F
4h
1H NMR, 700 MHz, CD3CN
f1 (ppm)

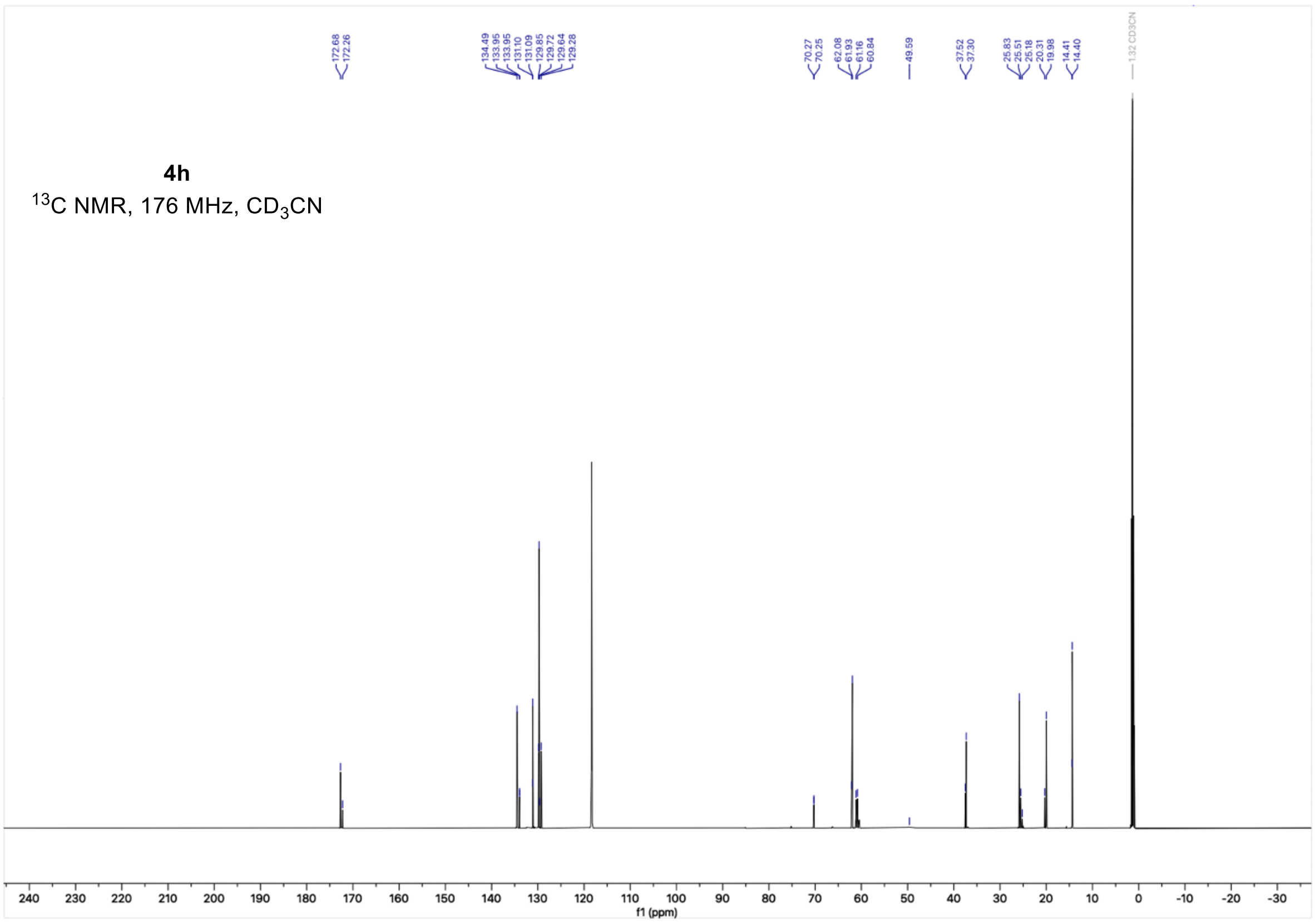
4h
13C NMR, 176 MHz, CD3CN
f1 (ppm)

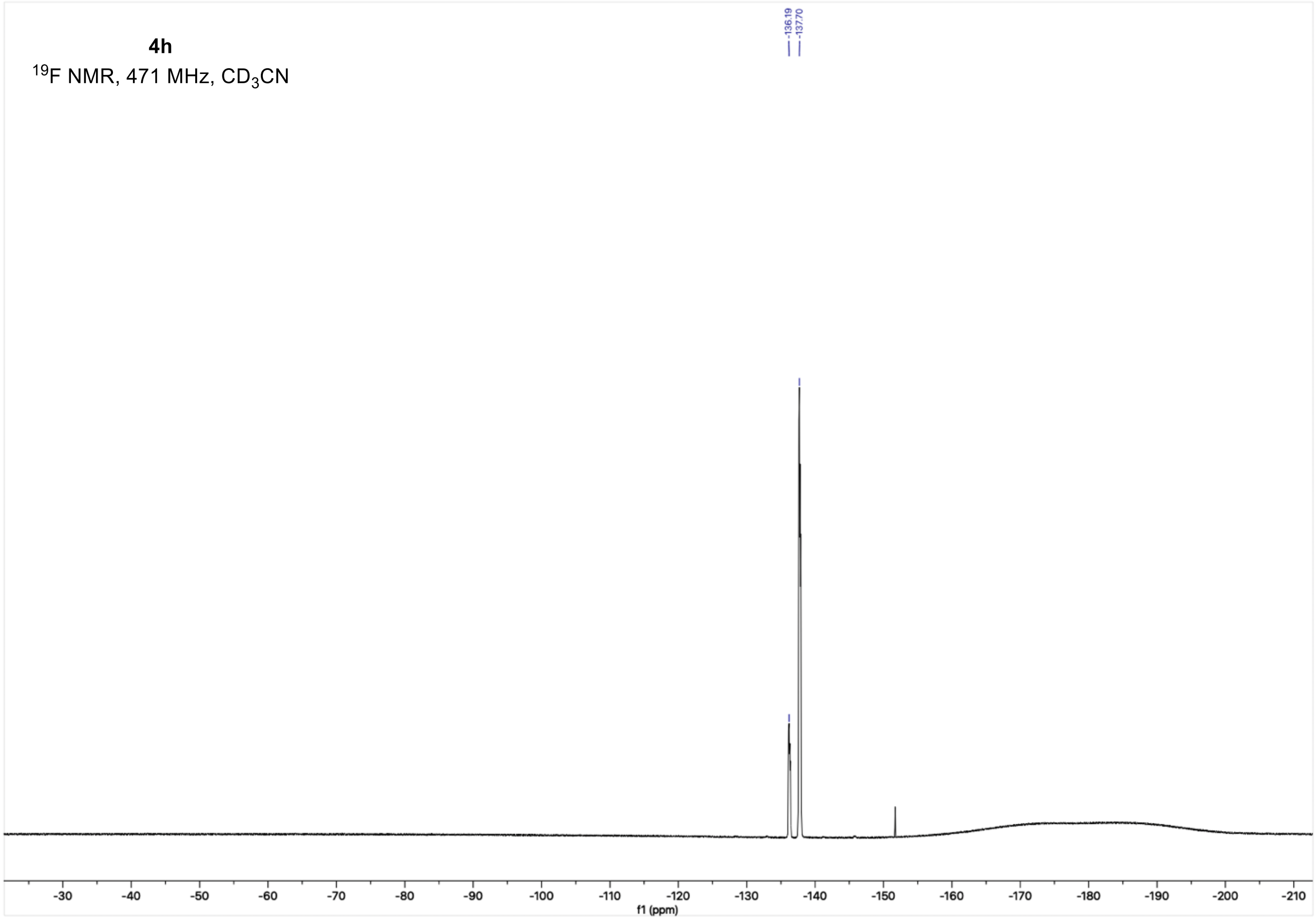
4h
19F NMR, 471 MHz, CD3CN
-136.19
-137.70
-30 -40 -50 -60 -70 -80 -90 -100 -110 -120 -130 -140 -150 -160 -170 -180 -190 -200 -210
f1 (ppm)

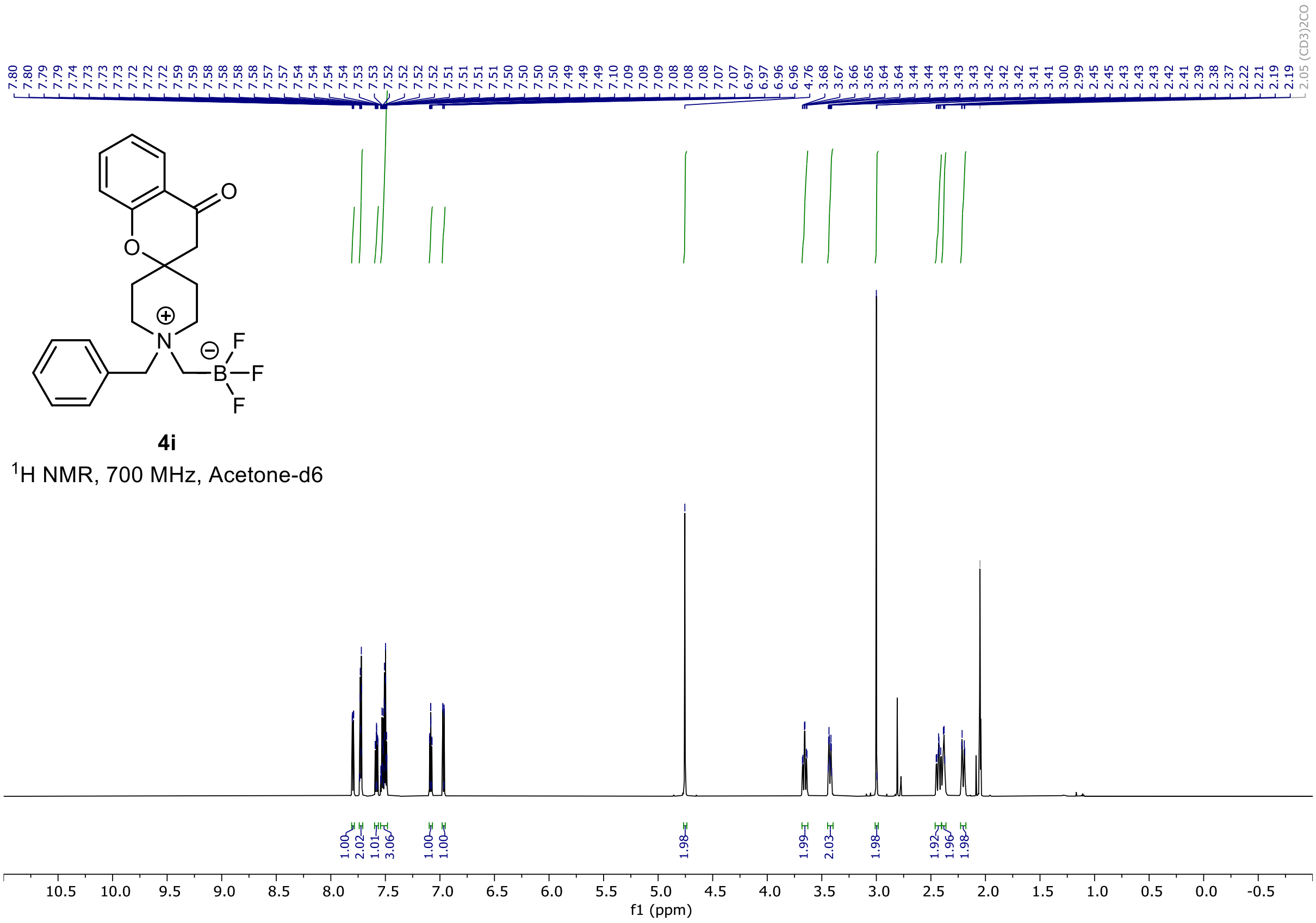
4i
¹H NMR, 700 MHz, Acetone-d6
f1 (ppm)

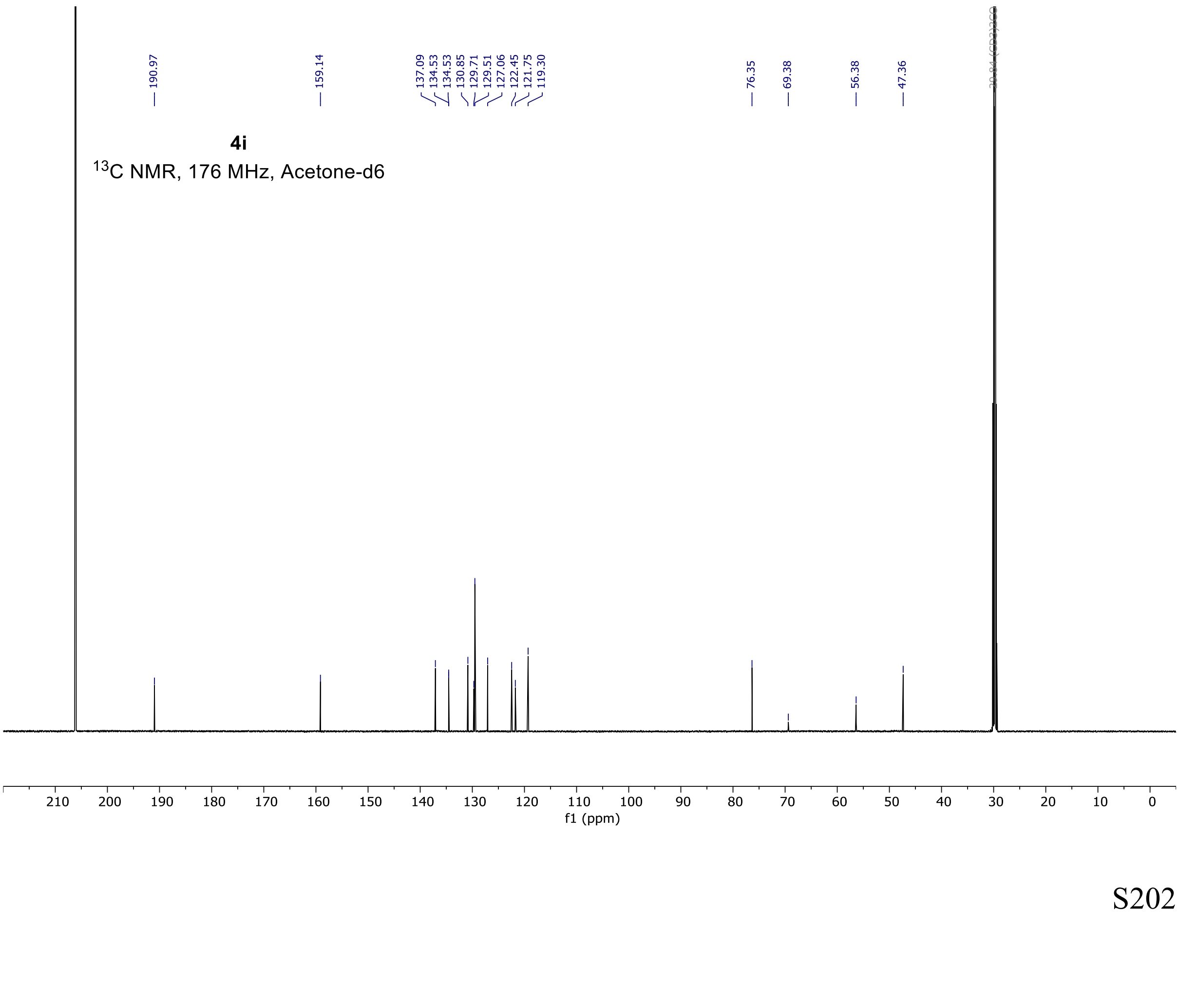
4i
¹³C NMR, 176 MHz, Acetone-d6
190.97
159.14
137.09
134.53
134.53
130.85
129.71
129.51
127.06
122.45
121.75
119.30
76.35
69.38
56.38
47.36
f1 (ppm)

4i

^{19}F NMR, 376 MHz, Acetone-d6

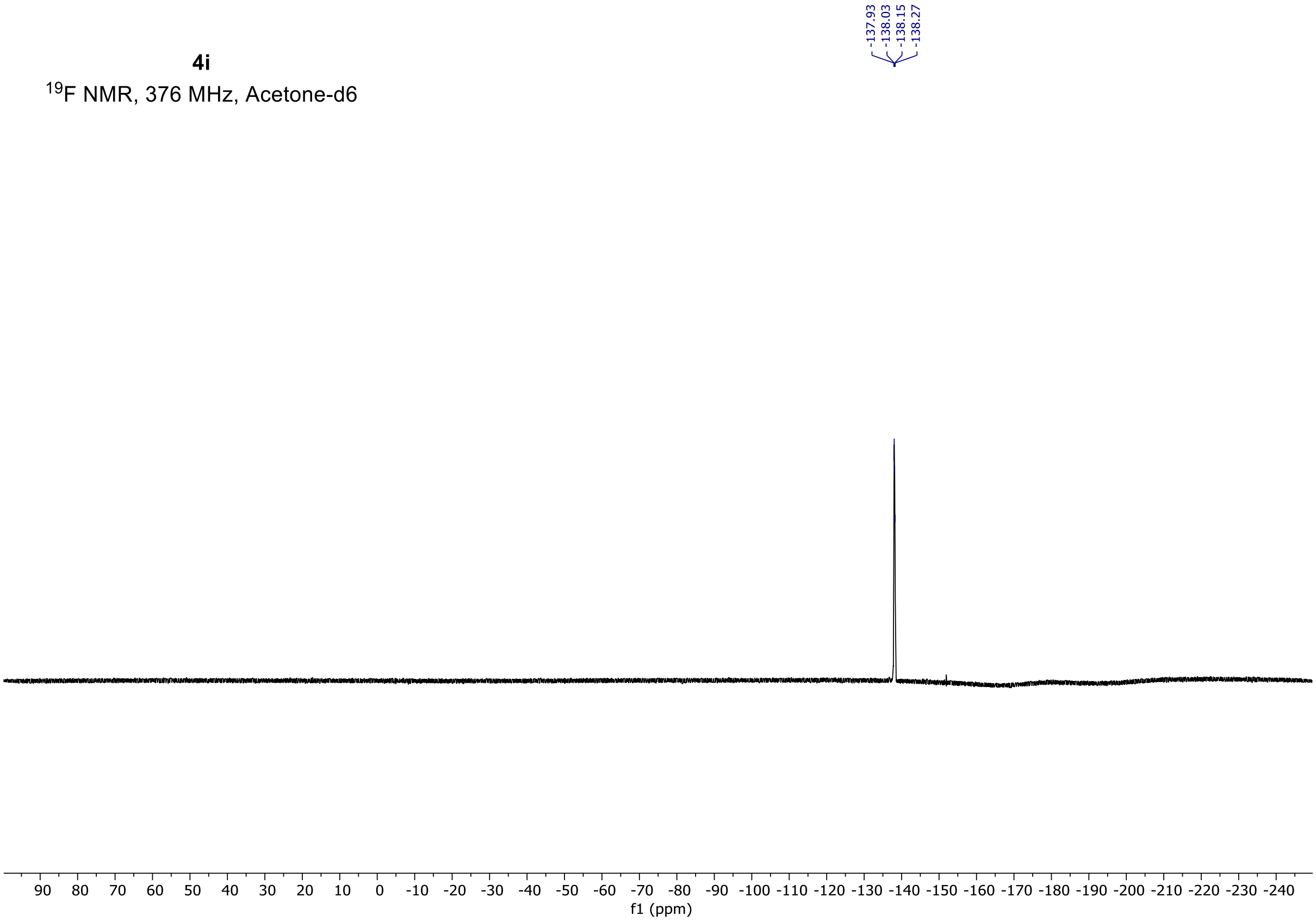

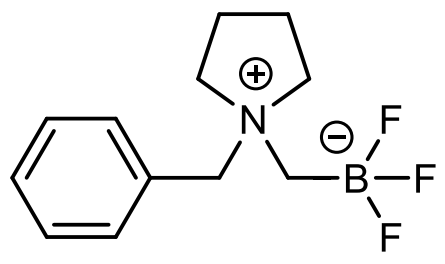

4j

^{1}H NMR, 700 MHz, DMSO-d6

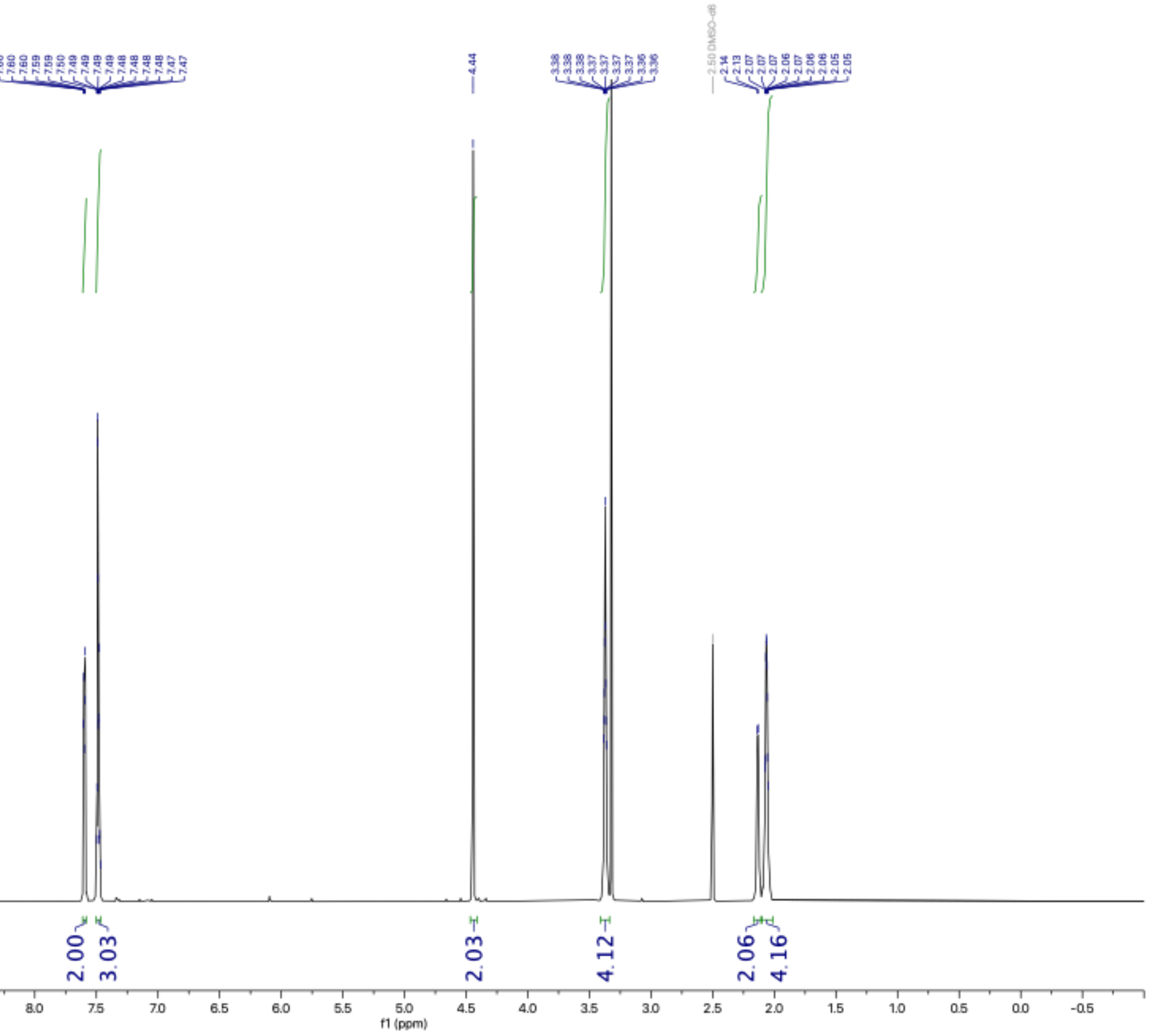

4j

^{13}C NMR, 176 MHz, DMSO-d6

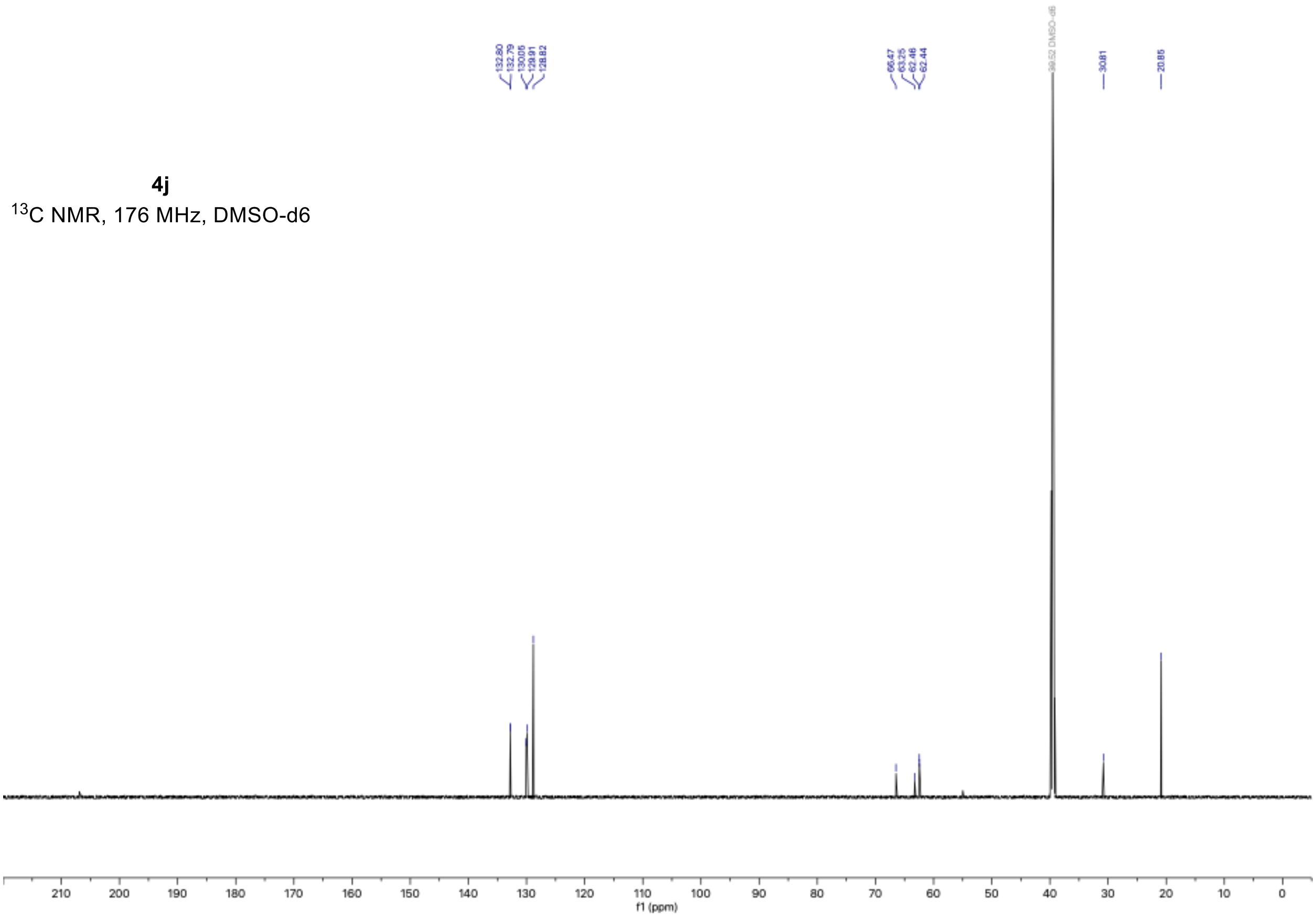

4j
^{19}F NMR, 376 MHz, DMSO-d6

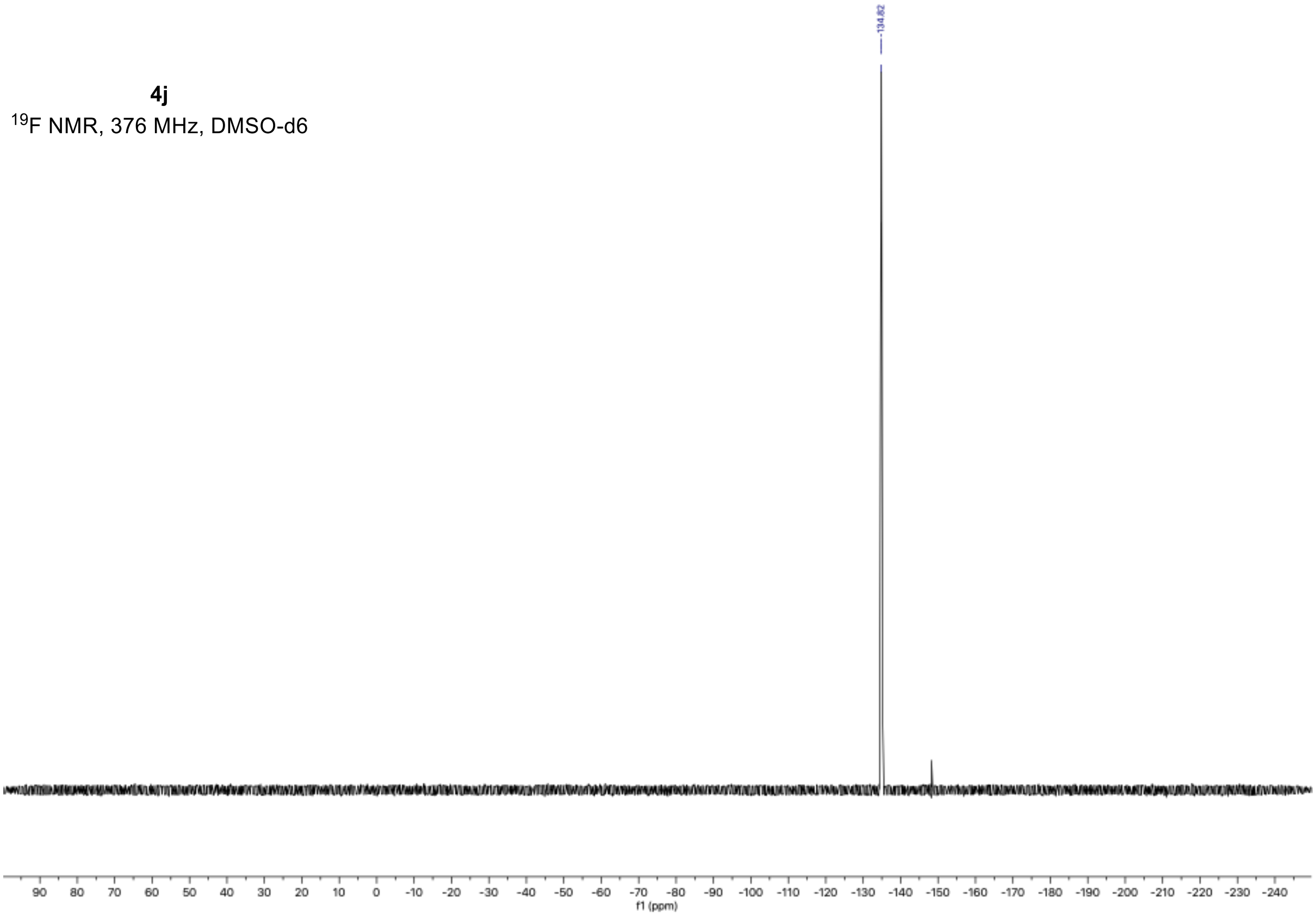

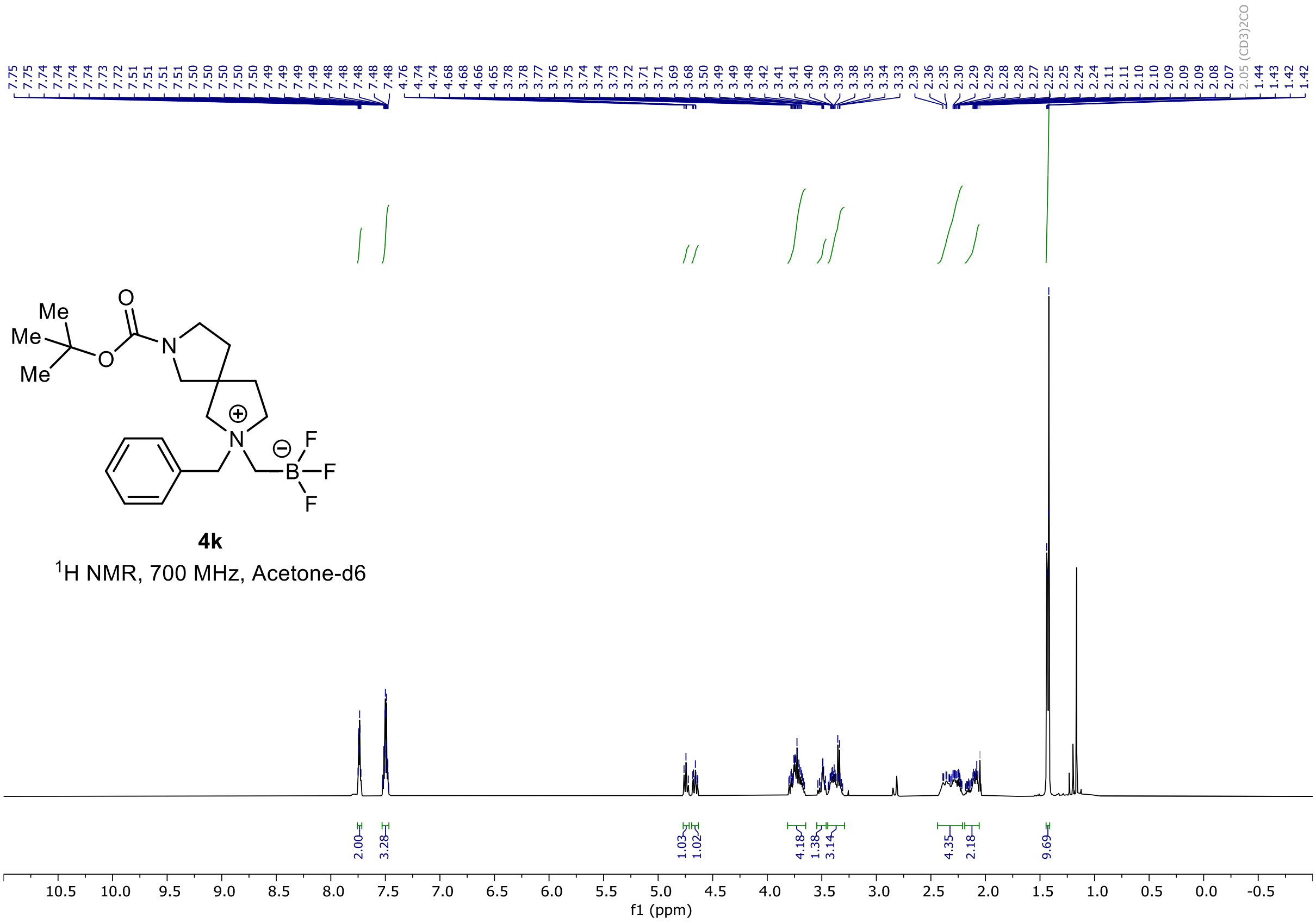

4k

^{1}H NMR, 700 MHz, Acetone-d6

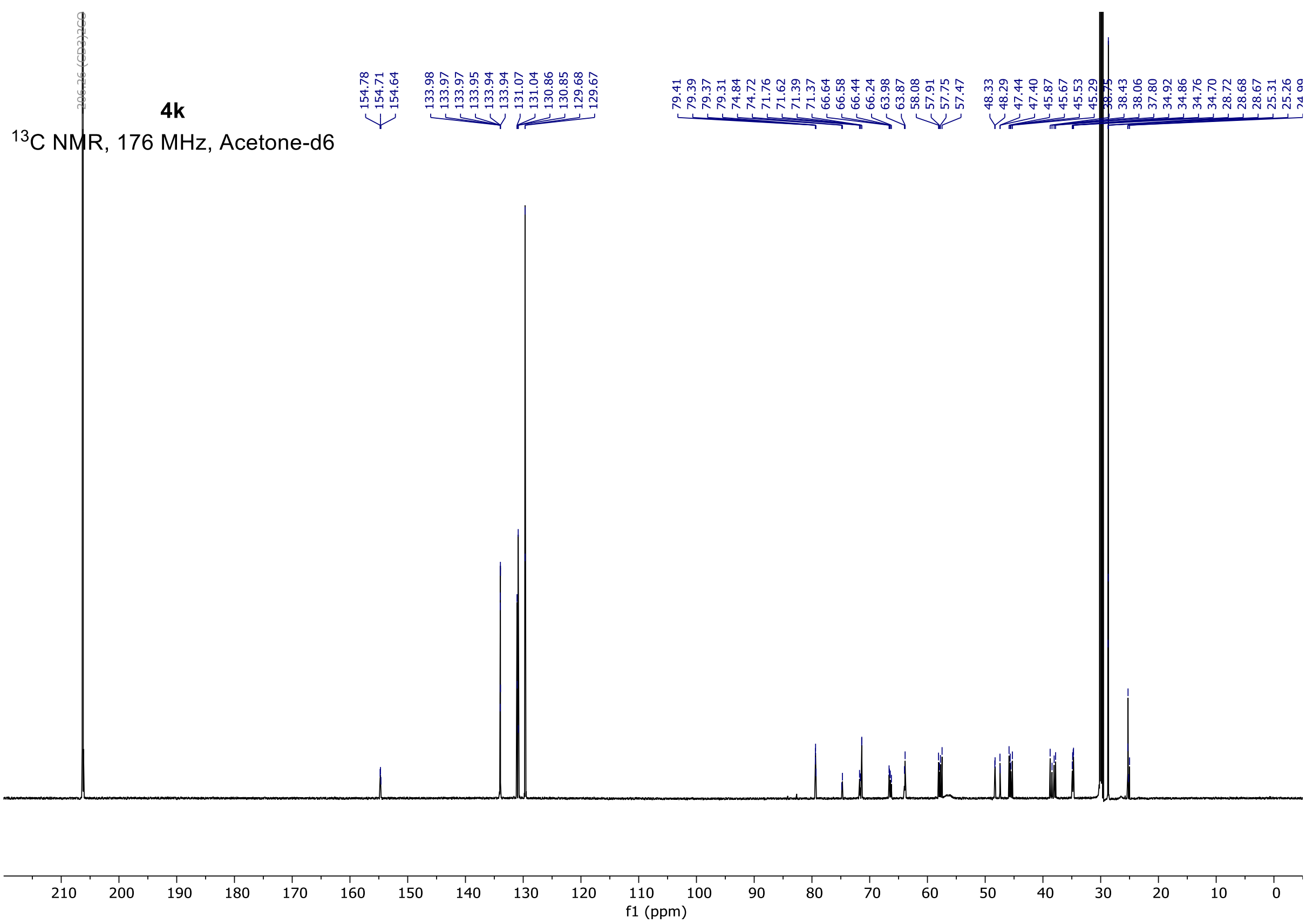

4k

^{13}C NMR, 176 MHz, Acetone-d6

4k

^{19}F NMR, 376 MHz, Acetone-d6

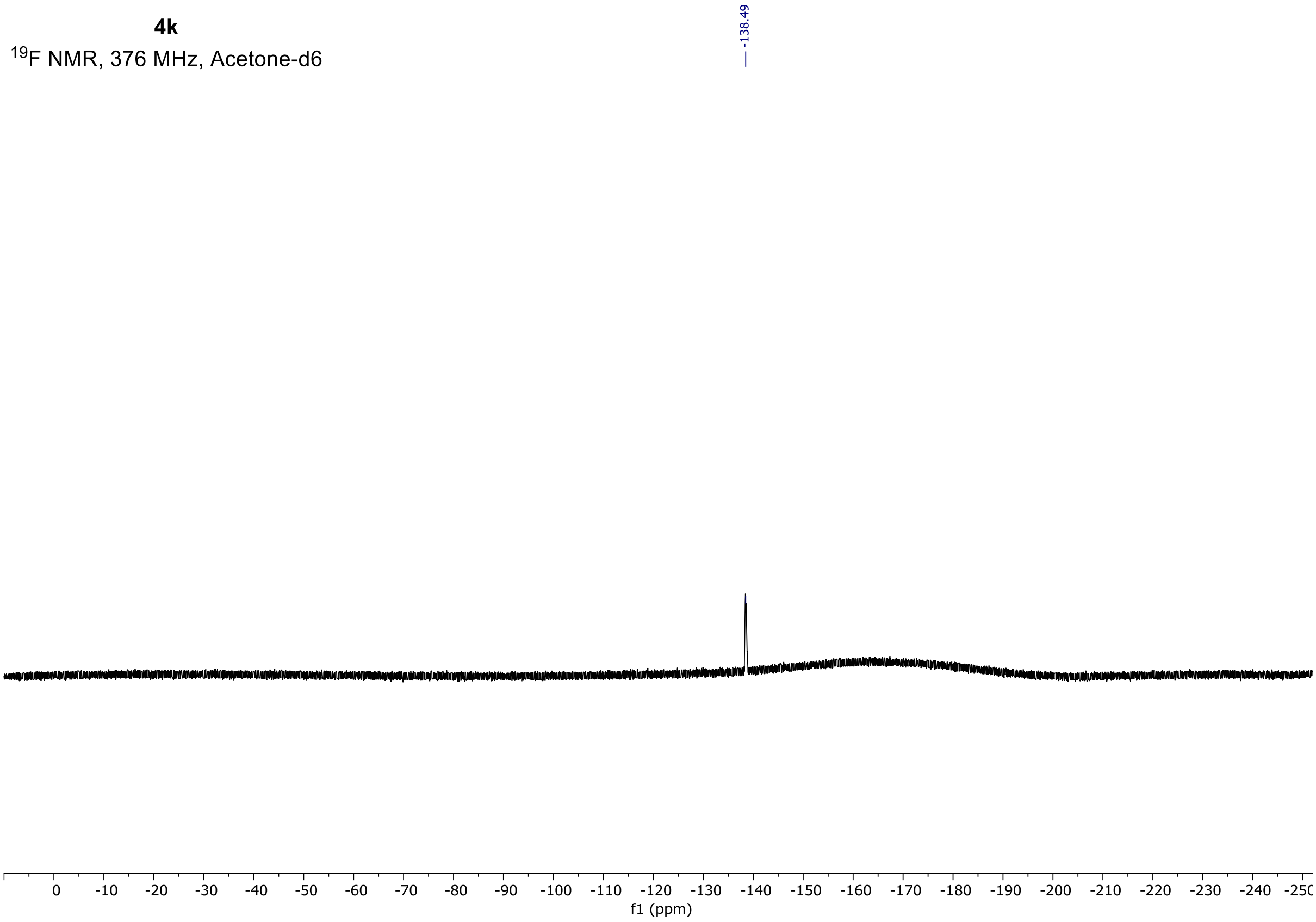

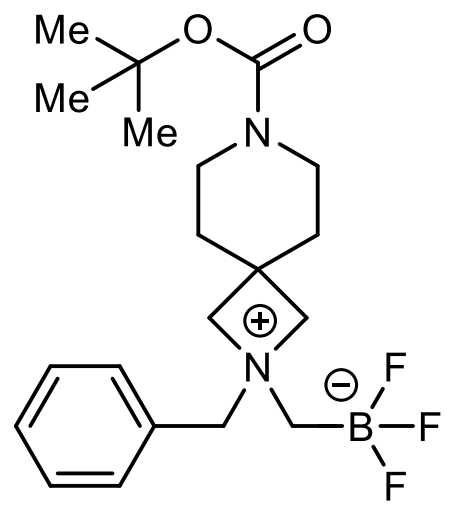

4l

^{1}H NMR, 700 MHz, Acetone-d6

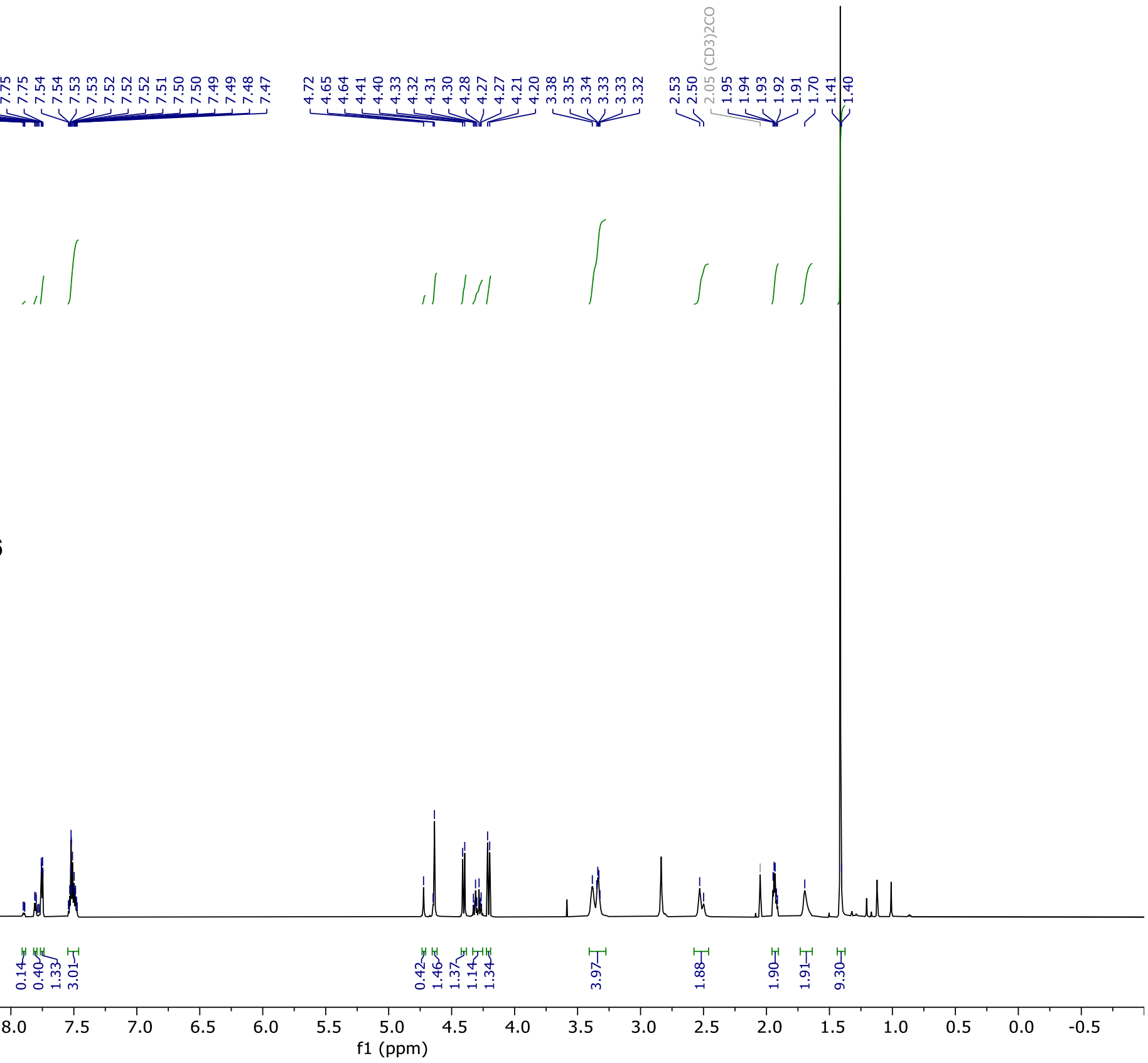

4l

^{13}C NMR, 176 MHz, Acetone-d6

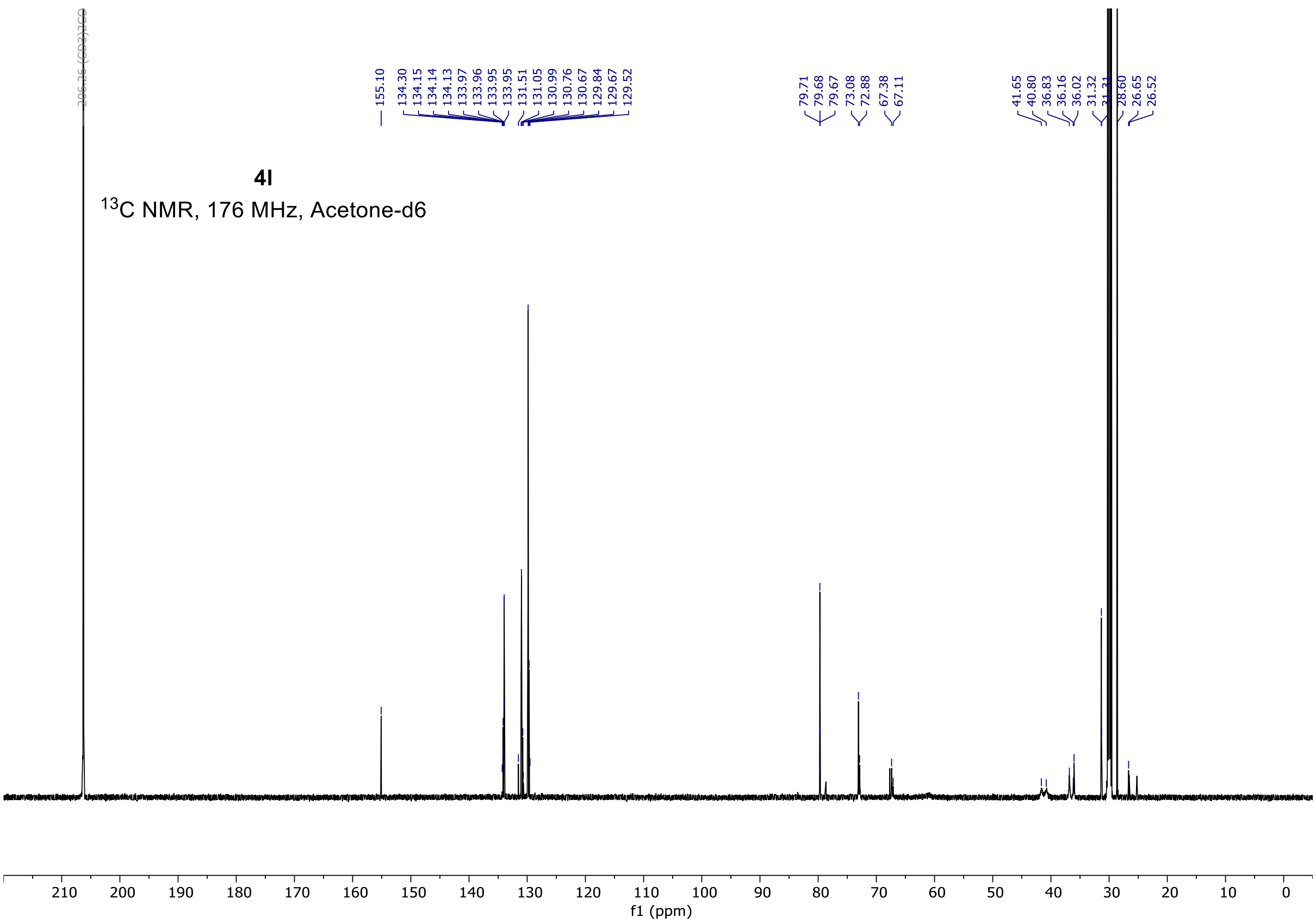

4l

^{19}F NMR, 471 MHz, Acetone-d6

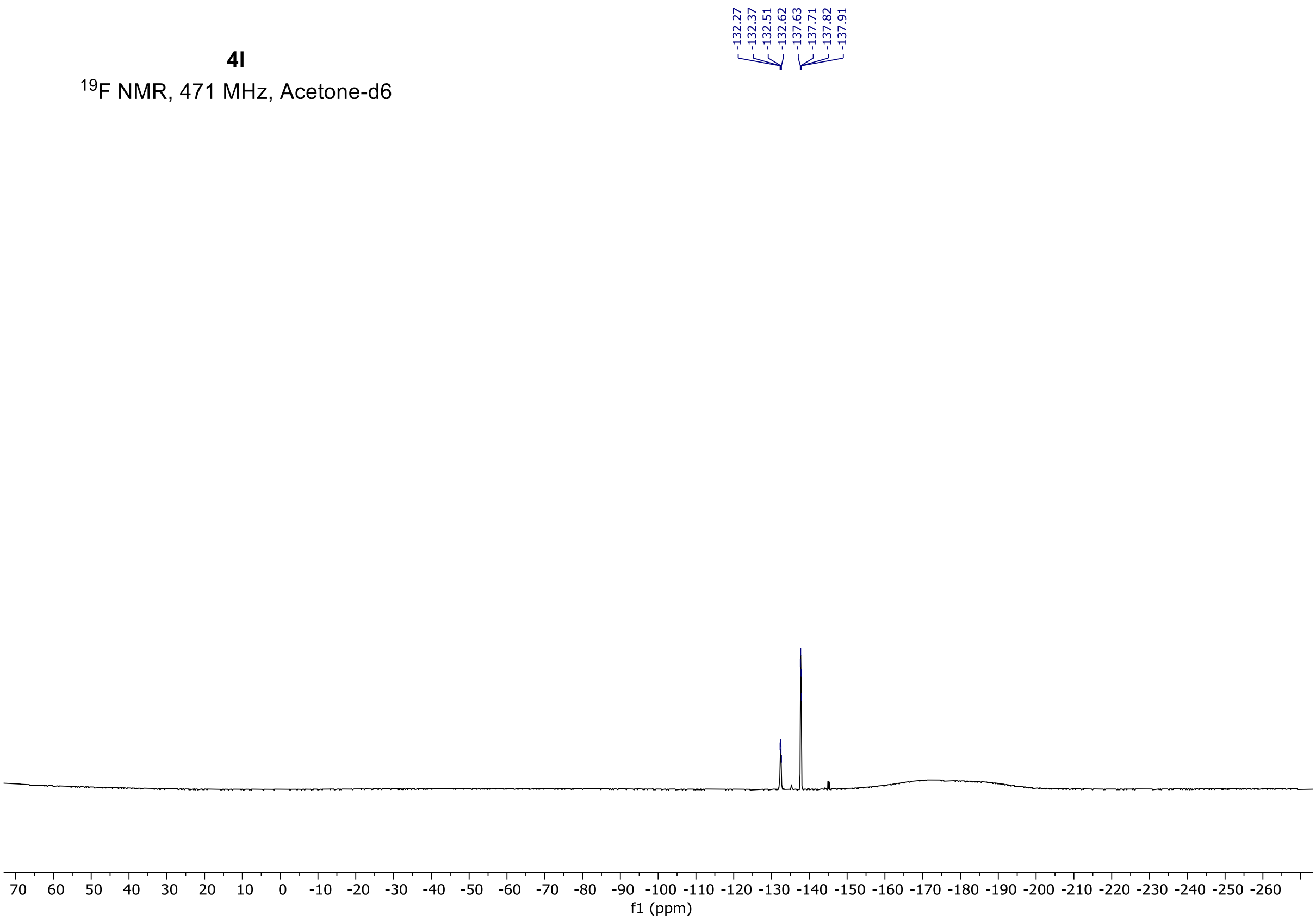

4m

^{1}H NMR, 700 MHz, Acetone-d6

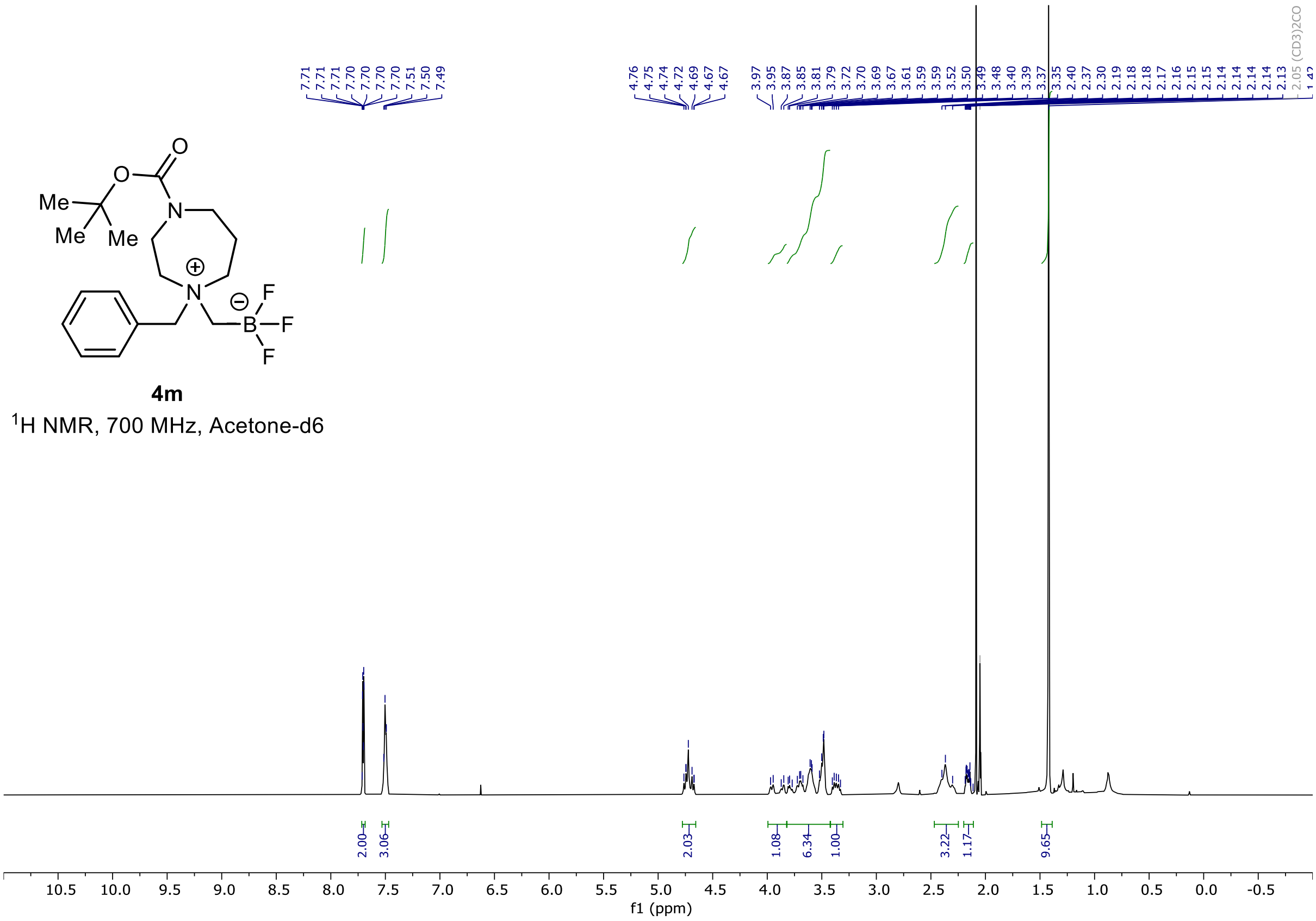

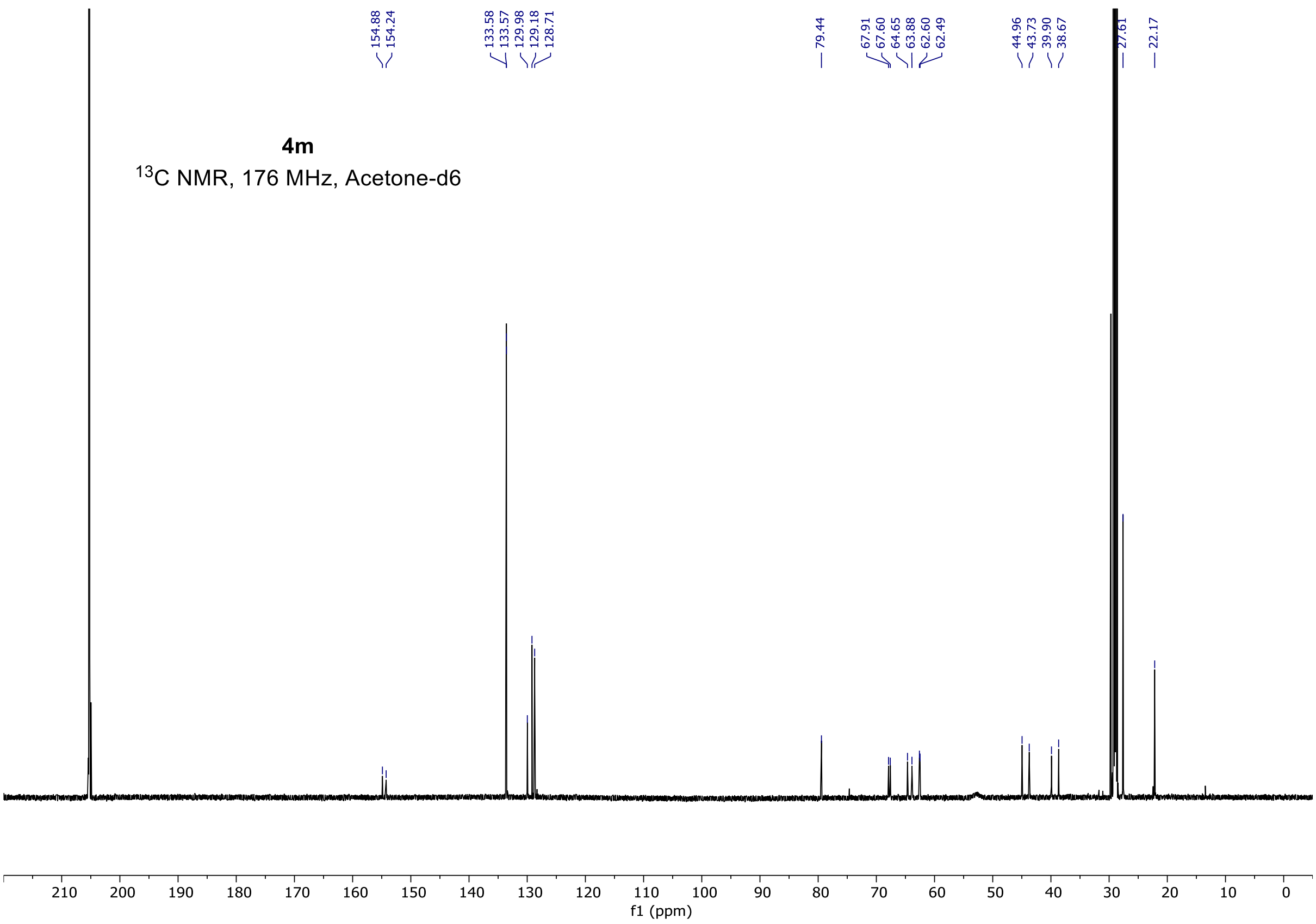

4m

^{19}F NMR, 376 MHz, Acetone-d6

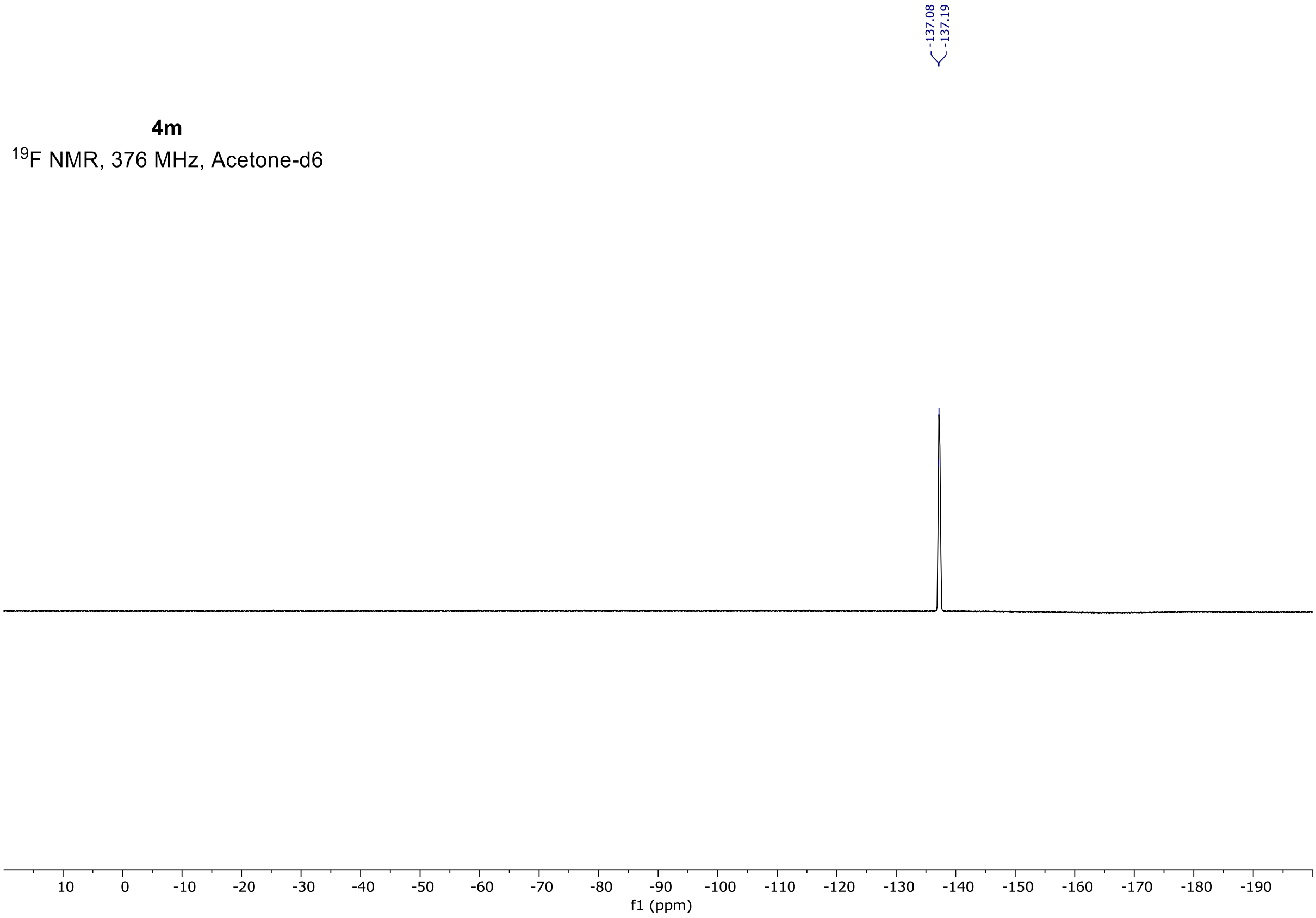

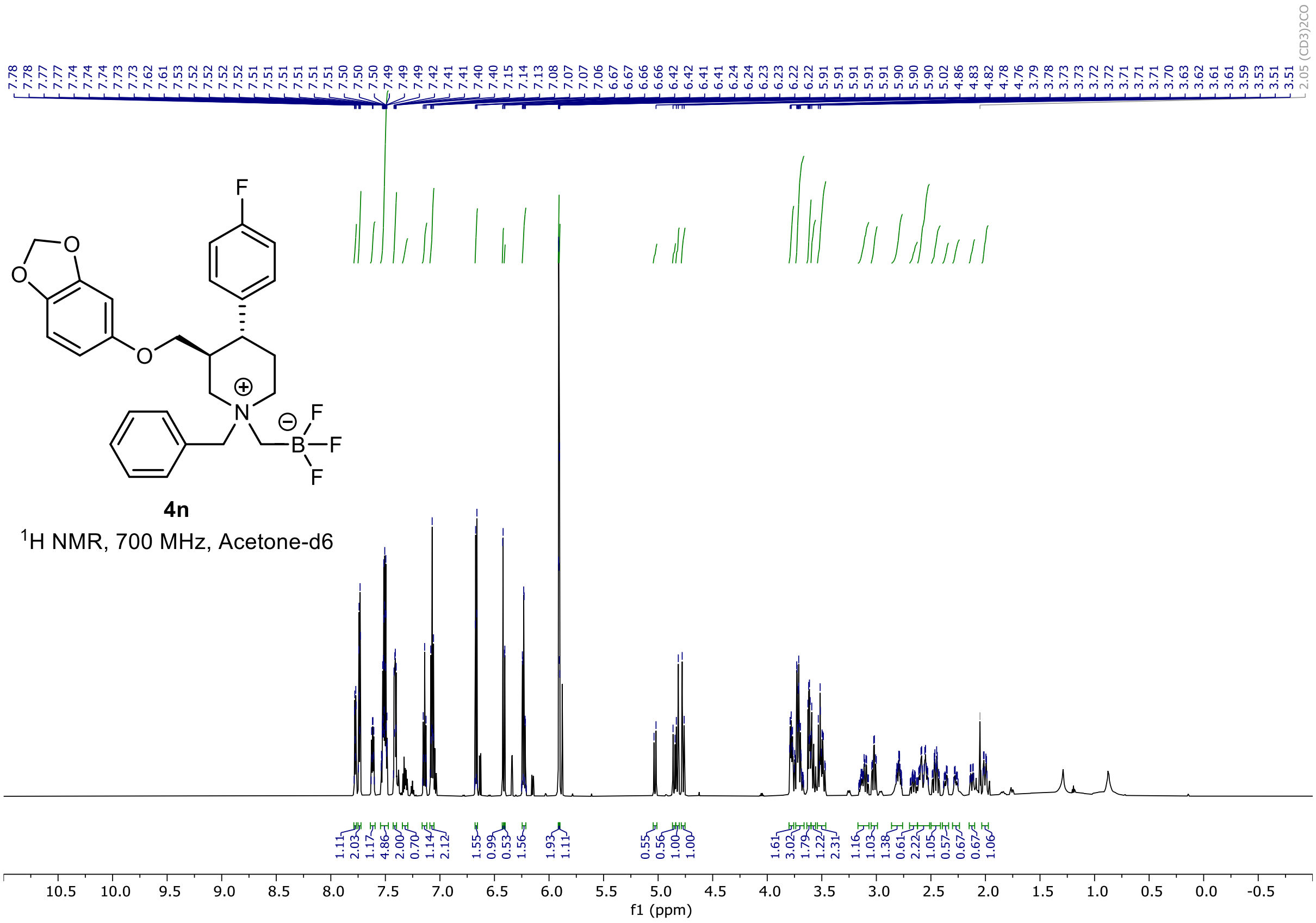
7.78 7.78 7.77 7.77 7.74 7.74 7.74 7.73 7.73 7.62 7.61 7.53 7.52 7.52 7.52 7.52 7.51 7.51 7.51 7.51 7.51 7.51 7.50 7.50 7.50 7.49 7.49 7.49 7.42 7.41 7.41 7.40 7.40 7.15 7.14 7.13 7.08 7.07 7.07 7.06 6.67 6.67 6.66 6.66 6.42 6.42 6.41 6.41 6.24 6.24 6.23 6.23 6.22 6.22 5.91 5.91 5.91 5.91 5.91 5.90 5.90 5.90 5.02 4.86 4.83 4.82 4.78 4.76 3.79 3.78 3.73 3.73 3.72 3.72 3.71 3.71 3.71 3.70 3.63 3.62 3.61 3.61 3.59 3.53 3.51 3.51
2.05 (CD3)2CO
F
O
O
O
N
B
F
F
F
4n
1H NMR, 700 MHz, Acetone-d6
1.11 2.03 1.17 4.86 2.00 0.70 1.14 2.12 1.55 0.99 0.53 1.56 1.93 1.11 0.55 0.56 1.00 1.00 1.61 3.02 1.79 1.22 2.31 1.16 1.03 1.38 0.61 2.22 1.05 0.57 0.67 0.67 1.06
10.5 10.0 9.5 9.0 8.5 8.0 7.5 7.0 6.5 6.0 5.5 5.0 4.5 4.0 3.5 3.0 2.5 2.0 1.5 1.0 0.5 0.0 -0.5
f1 (ppm)

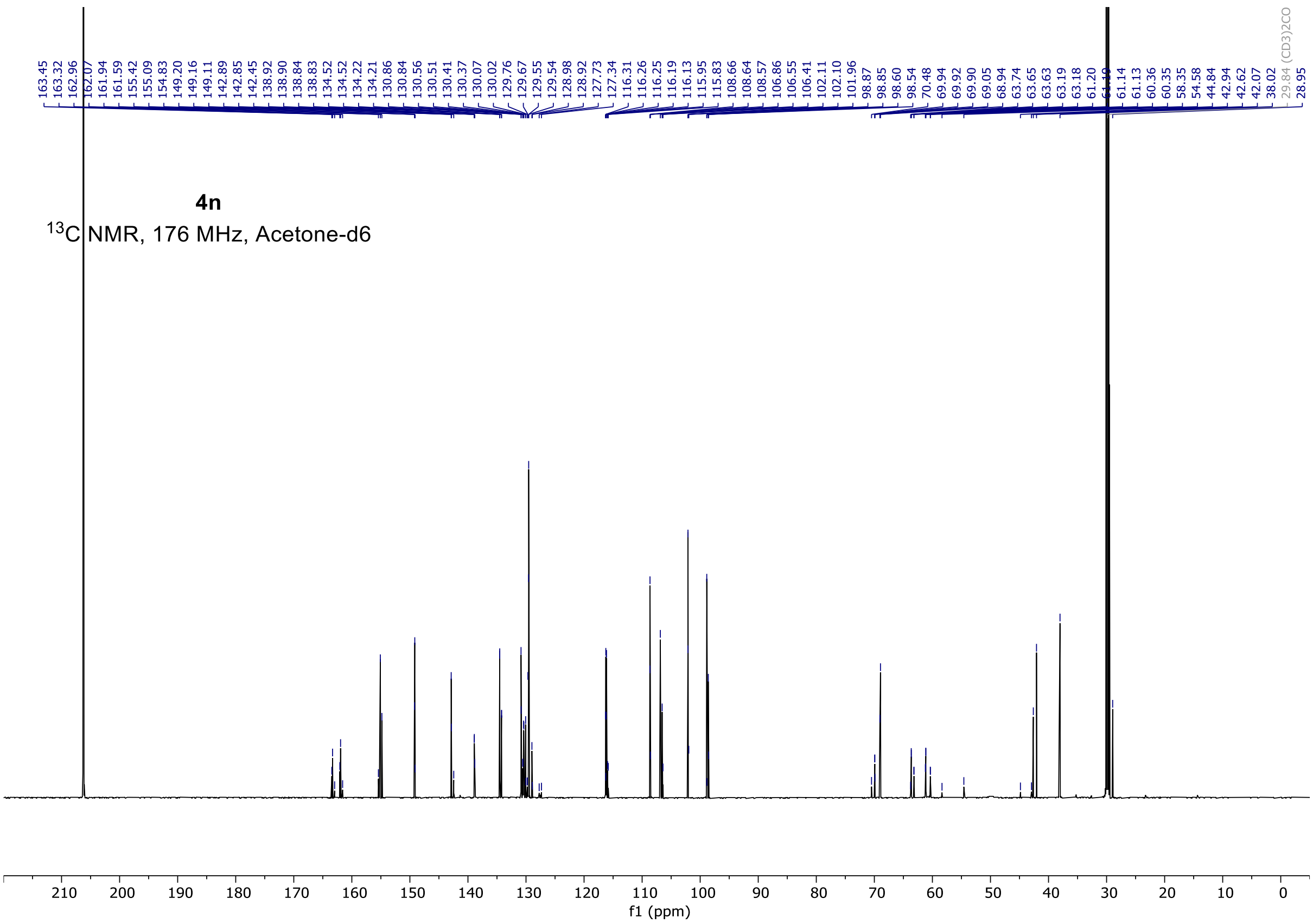
163.45 163.32 162.96 162.07 161.94 161.59 155.42 155.09 154.83 149.20 149.16 149.11 142.89 142.85 142.45 138.92 138.90 138.84 138.83 134.52 134.52 134.22 134.21 130.86 130.84 130.56 130.51 130.41 130.37 130.07 130.02 129.76 129.67 129.55 129.54 128.98 128.92 127.73 127.34 116.31 116.26 116.25 116.19 116.13 115.95 115.83 108.66 108.64 108.57 106.86 106.55 106.41 102.11 102.10 101.96 98.87 98.85 98.60 98.54 70.48 69.94 69.92 69.90 69.05 68.94 63.74 63.65 63.63 63.19 63.18 61.20 61.14 61.13 60.36 60.35 58.35 54.58 44.84 42.94 42.62 42.07 38.02 29.84 (CD3)2CO 28.95
4n
13C NMR, 176 MHz, Acetone-d6
210 200 190 180 170 160 150 140 130 120 110 100 90 80 70 60 50 40 30 20 10 0
f1 (ppm)

4n

^{19}F NMR, 376 MHz, Acetone-d6

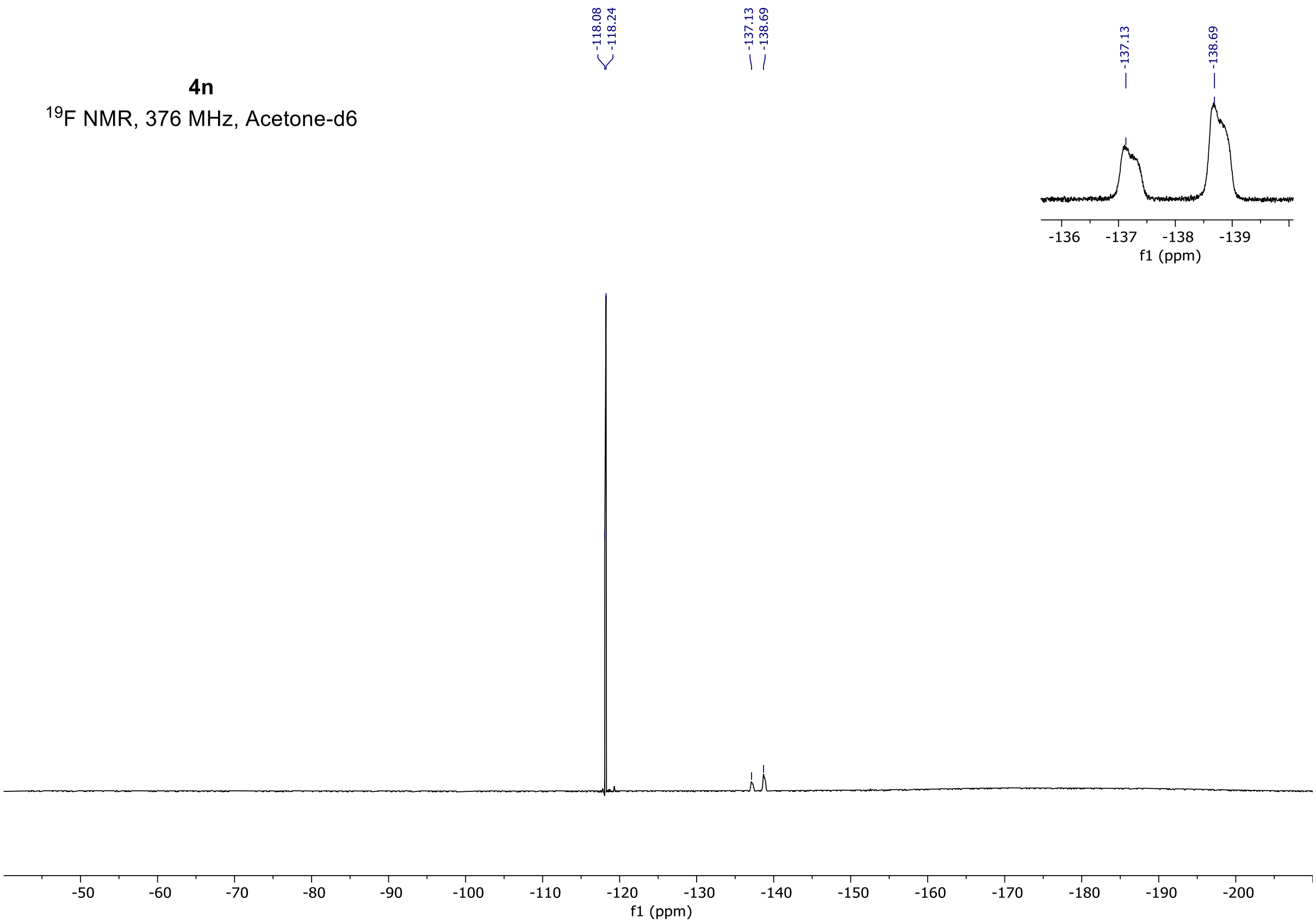

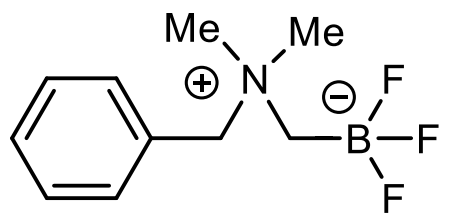

4o

^{1}H NMR, 500 MHz, DMSO-d6

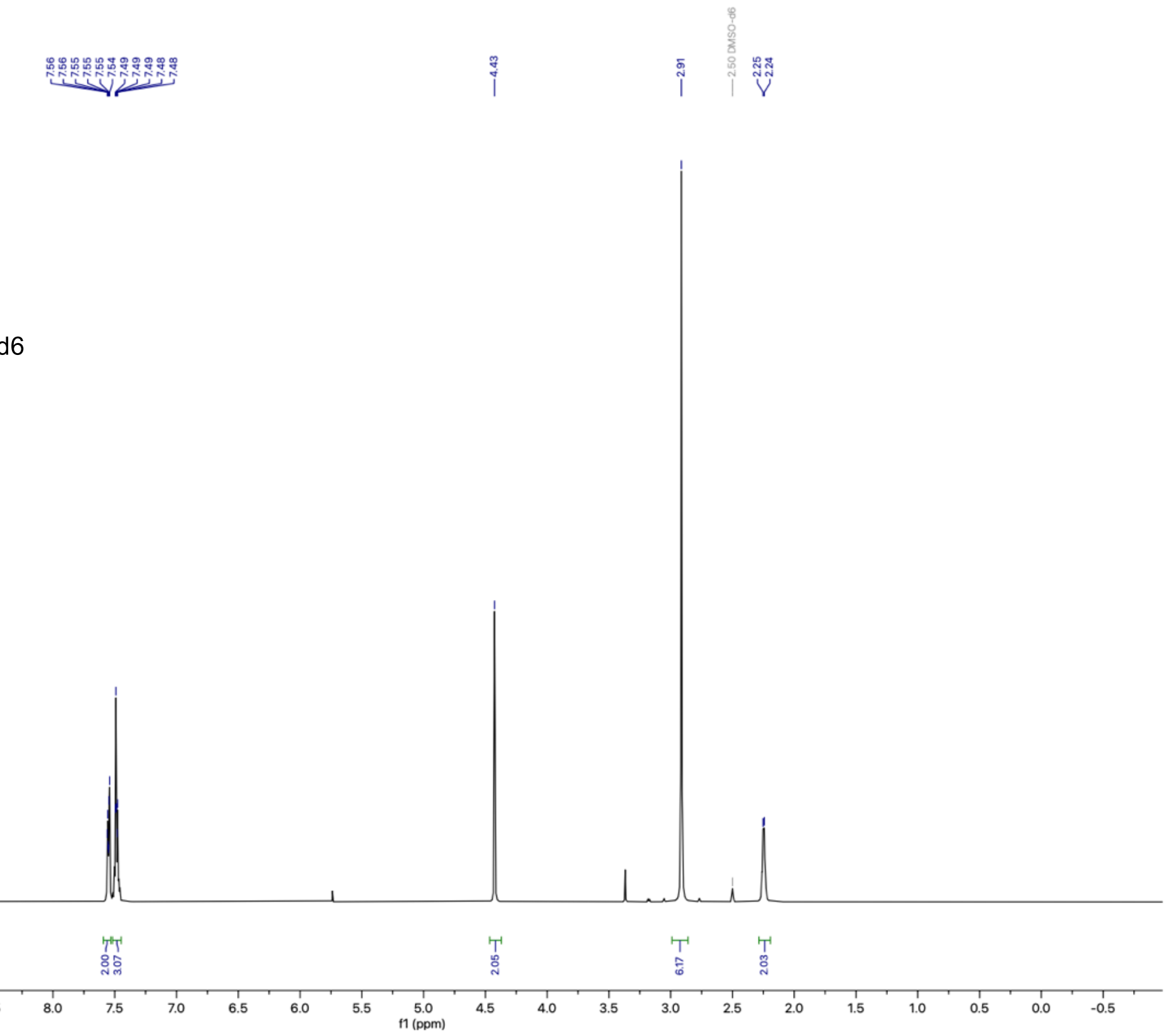

4o

^{13}C NMR, 126 MHz, DMSO-d6

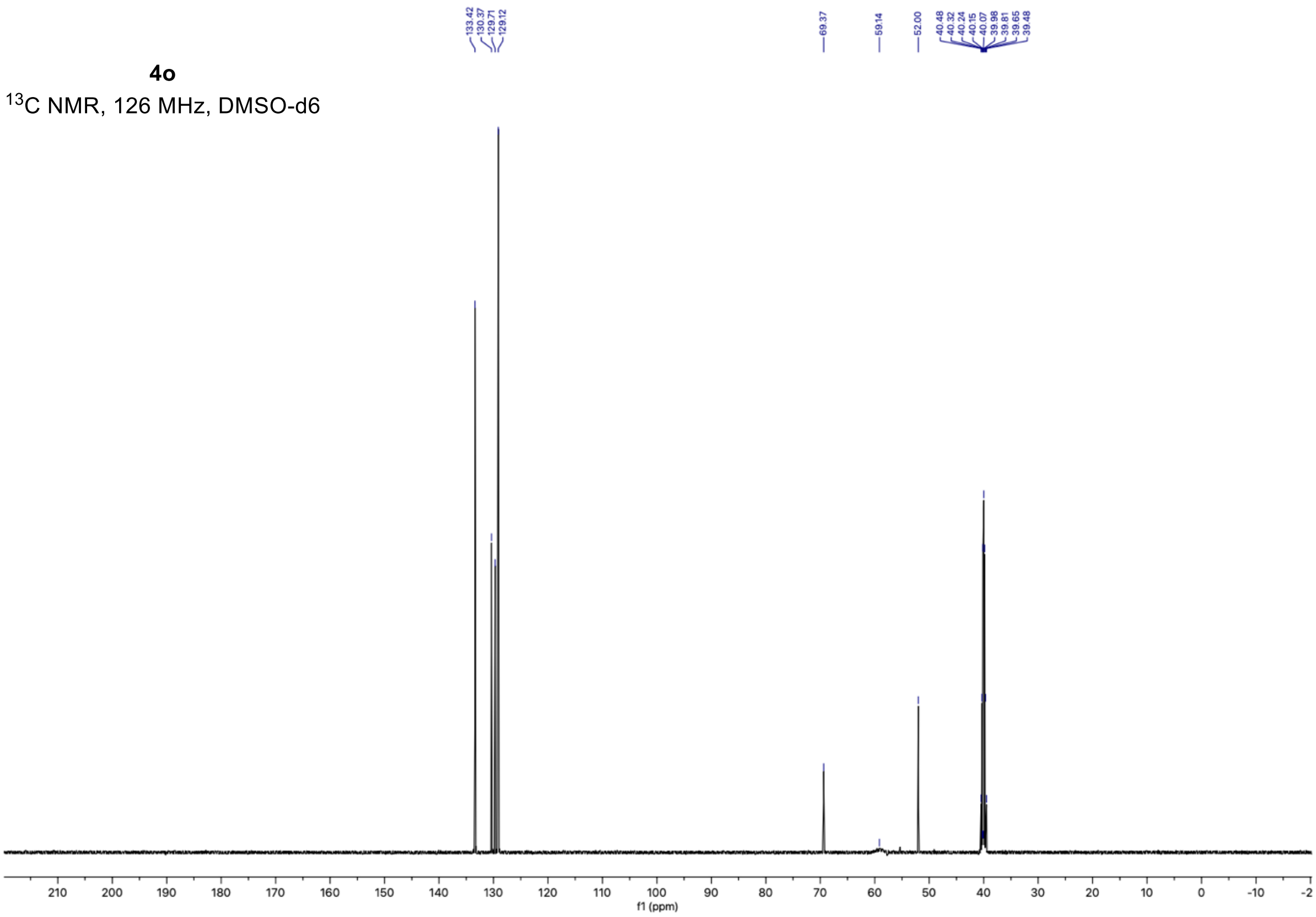

4o

^{19}F NMR, 471 MHz, DMSO-d6

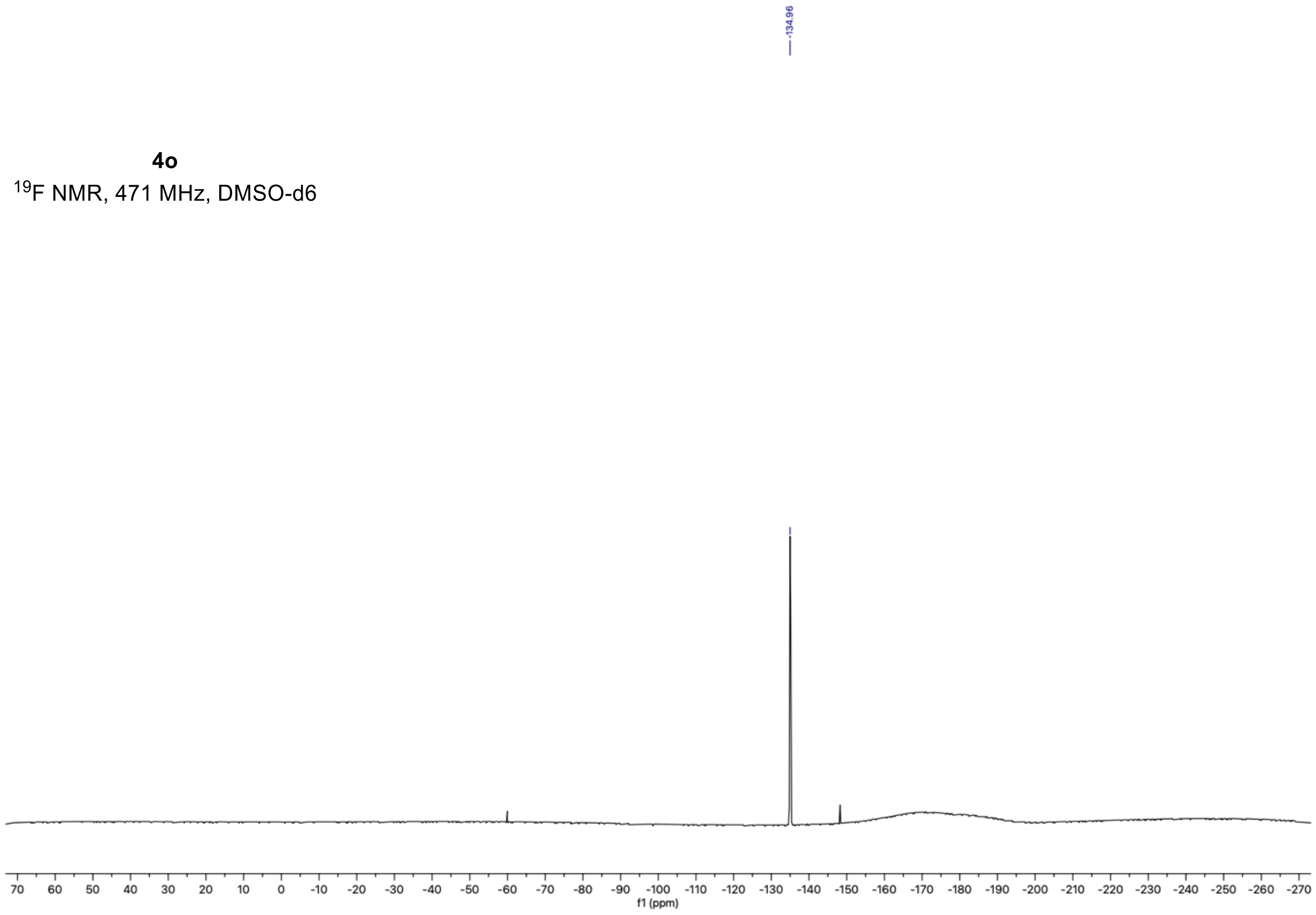

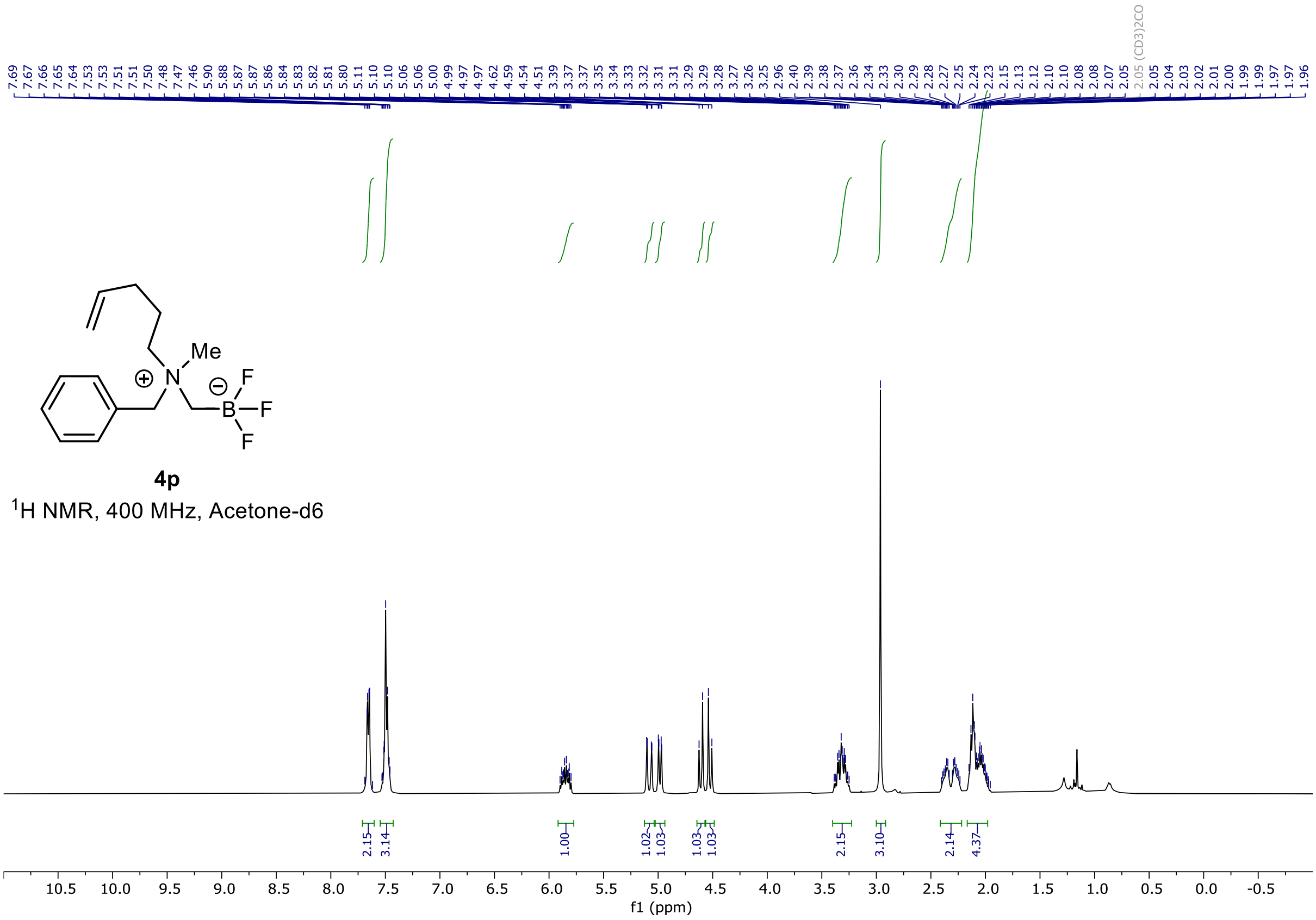

4p

^{1}H NMR, 400 MHz, Acetone-d6

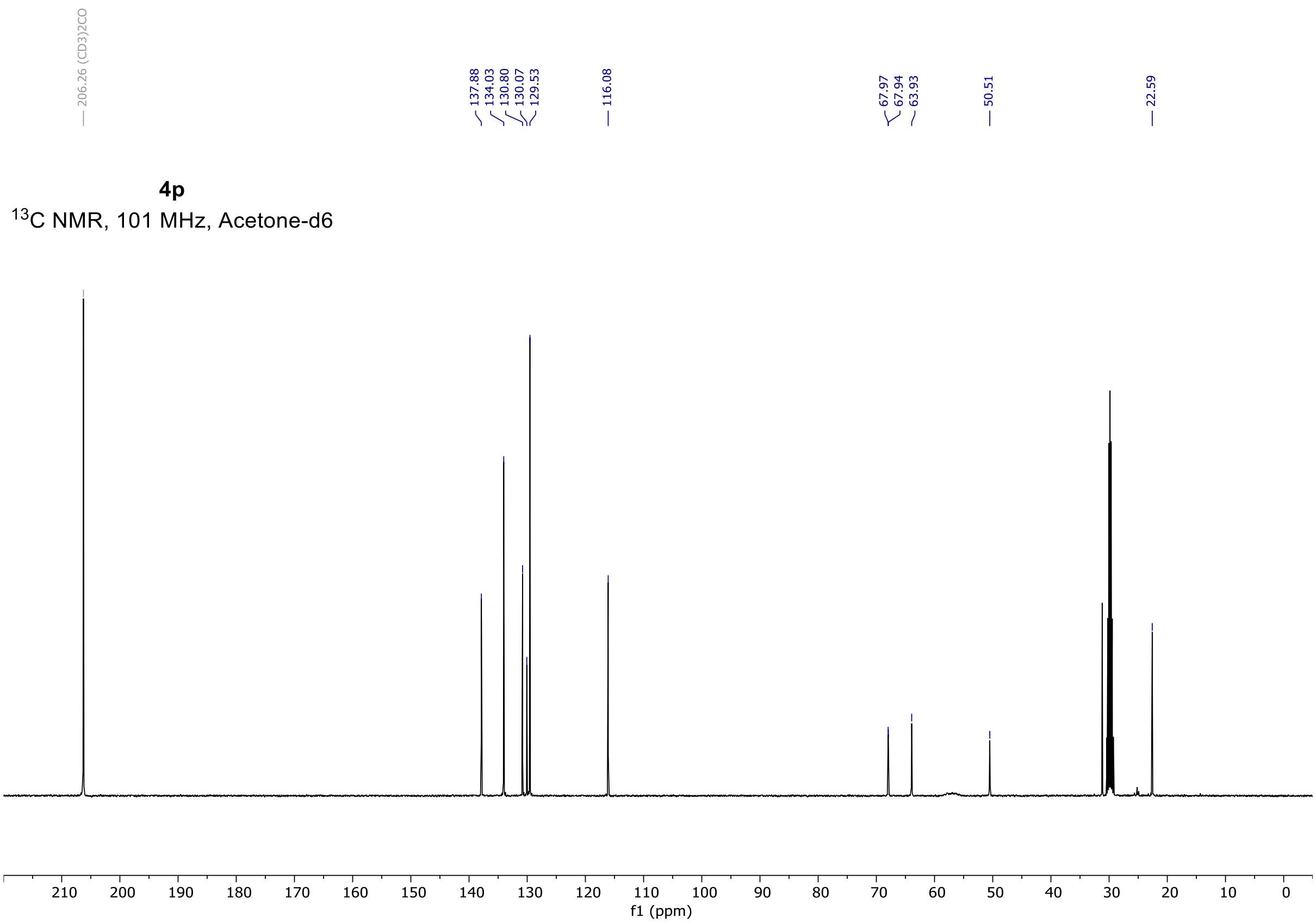

4p

^{13}C NMR, 101 MHz, Acetone-d6

4p

^{19}F NMR, 376 MHz, Acetone-d6

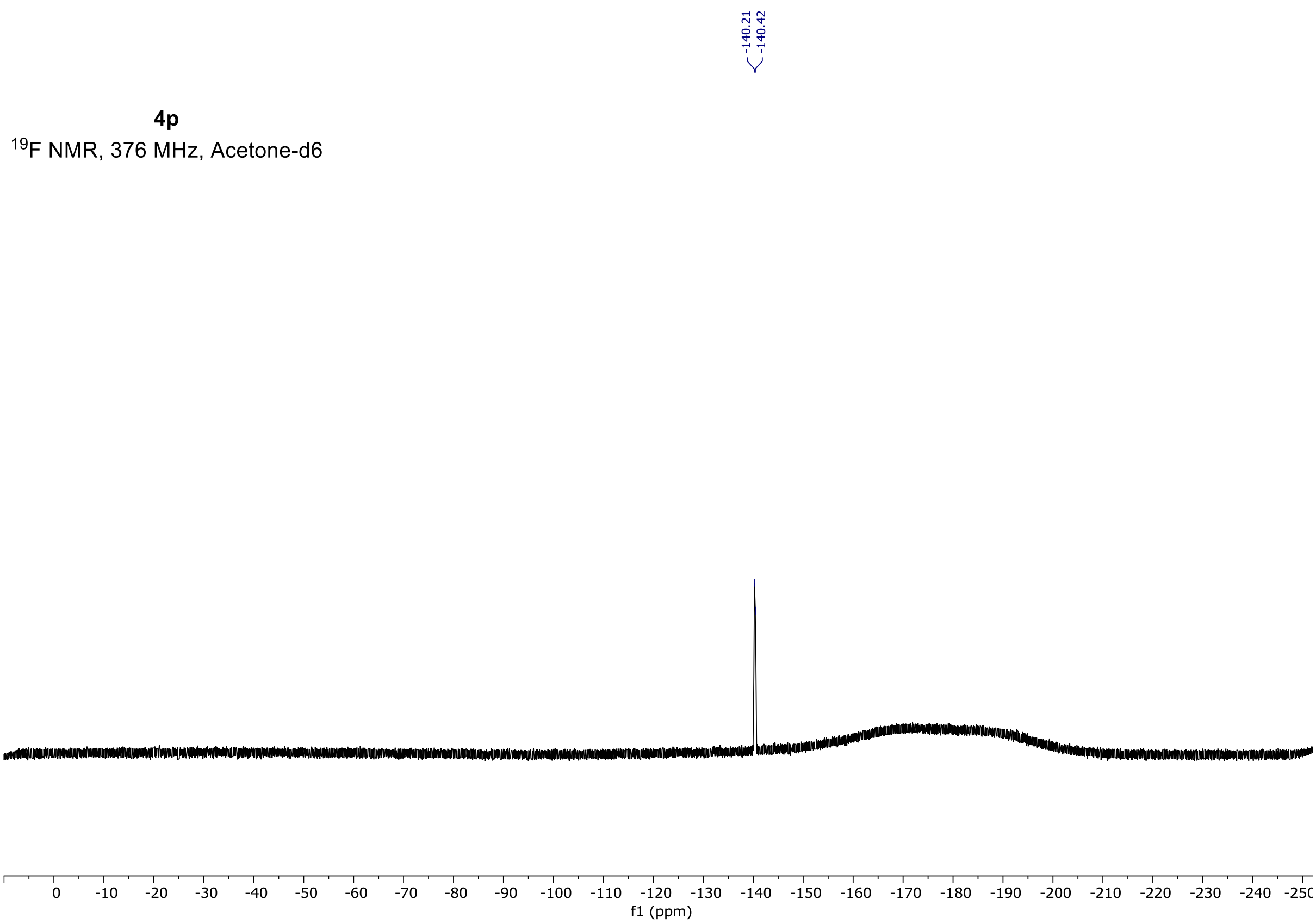

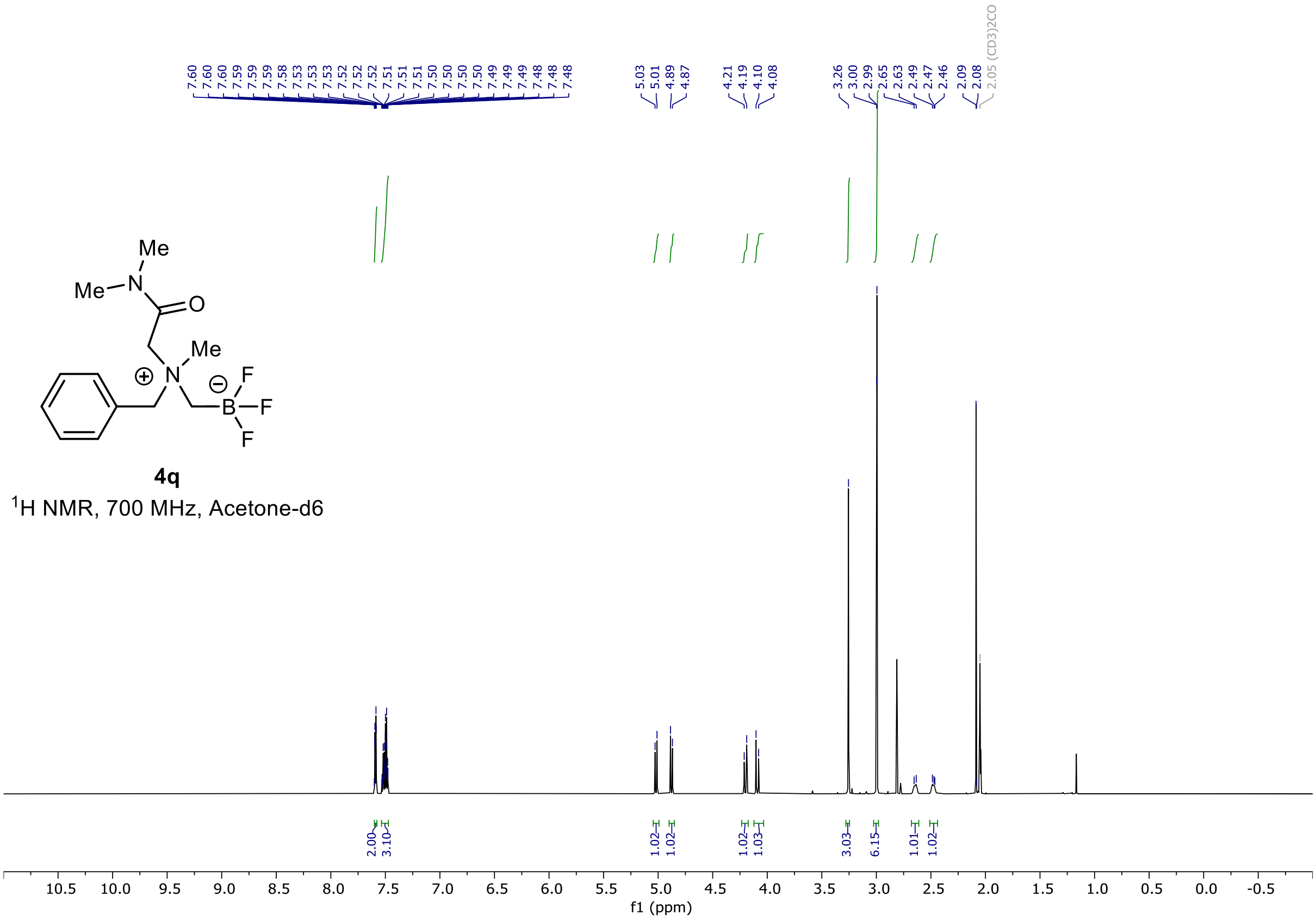
Me
Me
N
O
Me
N
F
B
F
F
4q
^{1}H NMR, 700 MHz, Acetone-d6
7.60 7.60 7.60 7.59 7.59 7.59 7.58 7.53 7.53 7.53 7.52 7.52 7.52 7.51 7.51 7.51 7.50 7.50 7.50 7.50 7.49 7.49 7.49 7.48 7.48 7.48
5.03 5.01 4.89 4.87
4.21 4.19 4.10 4.08
3.26 3.00 2.99 2.65 2.63 2.49 2.47 2.46
2.09 2.08
2.05 (CD3)2CO
2.00 3.10 1.02 1.02 1.02 1.03 3.03 6.15 1.01 1.02
f1 (ppm)

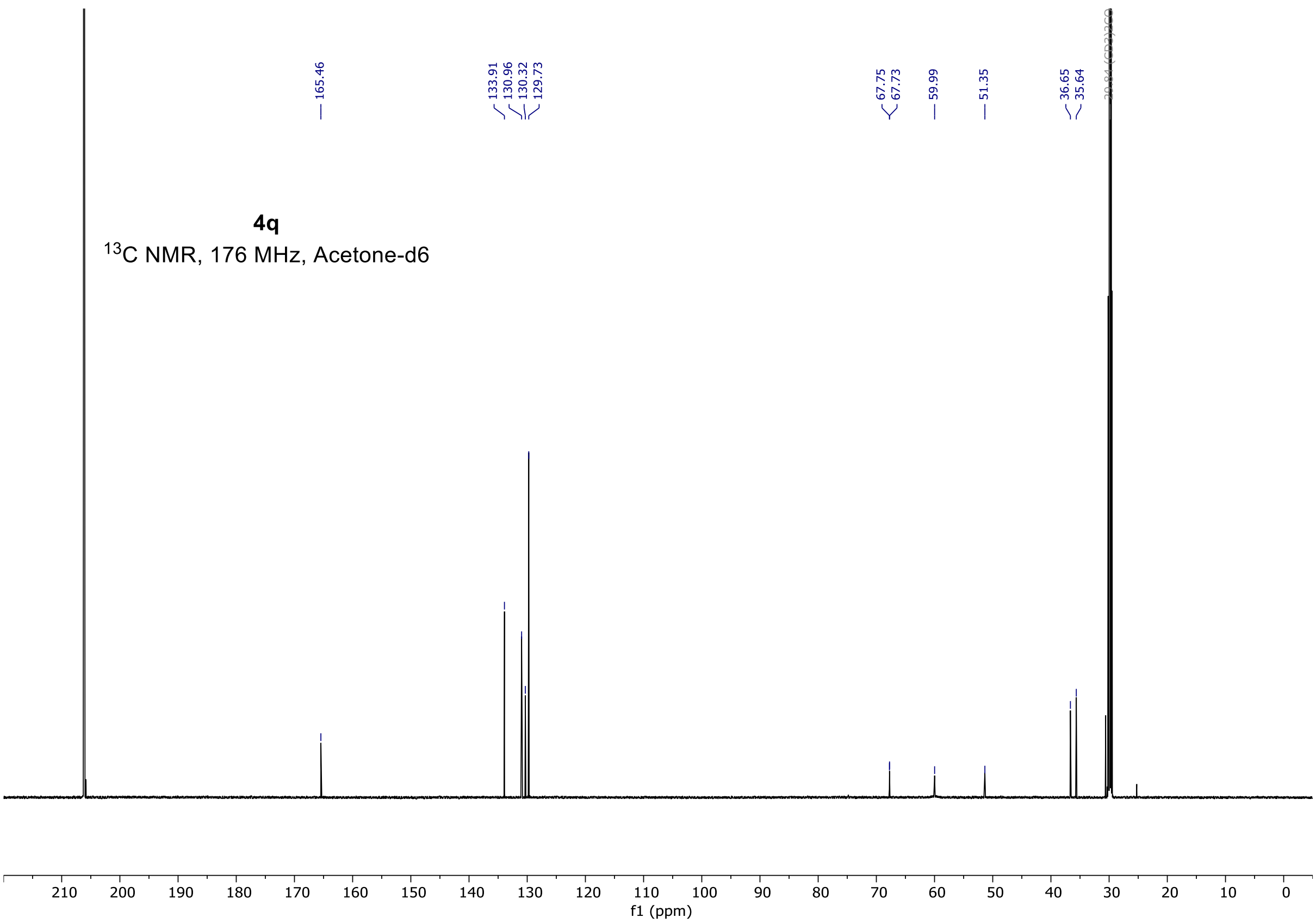
4q
^{13}C NMR, 176 MHz, Acetone-d6
165.46
133.91 130.96 130.32 129.73
67.75 67.73
59.99
51.35
36.65 35.64
f1 (ppm)

4q

^{19}F NMR, 376 MHz, Acetone-d6

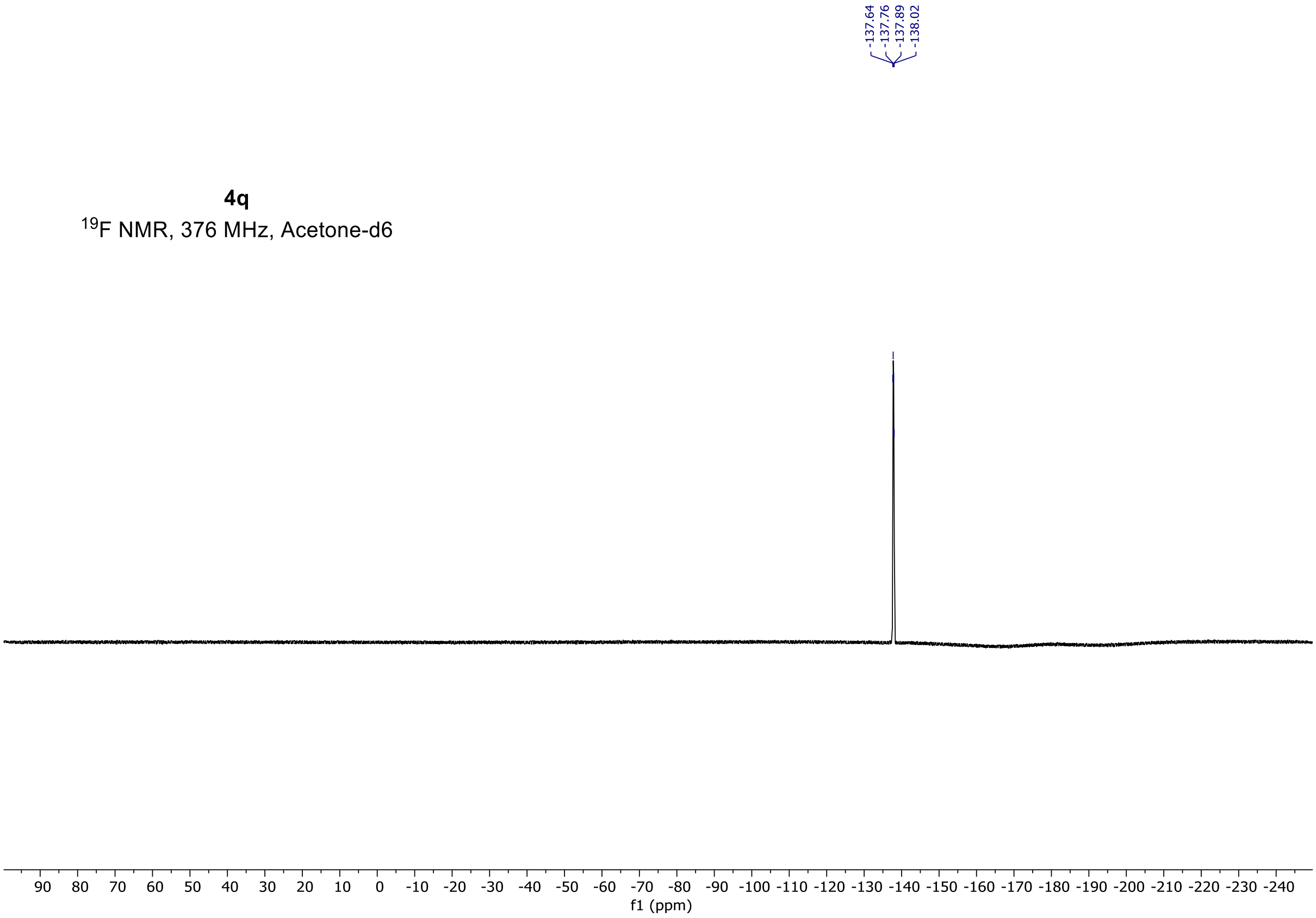

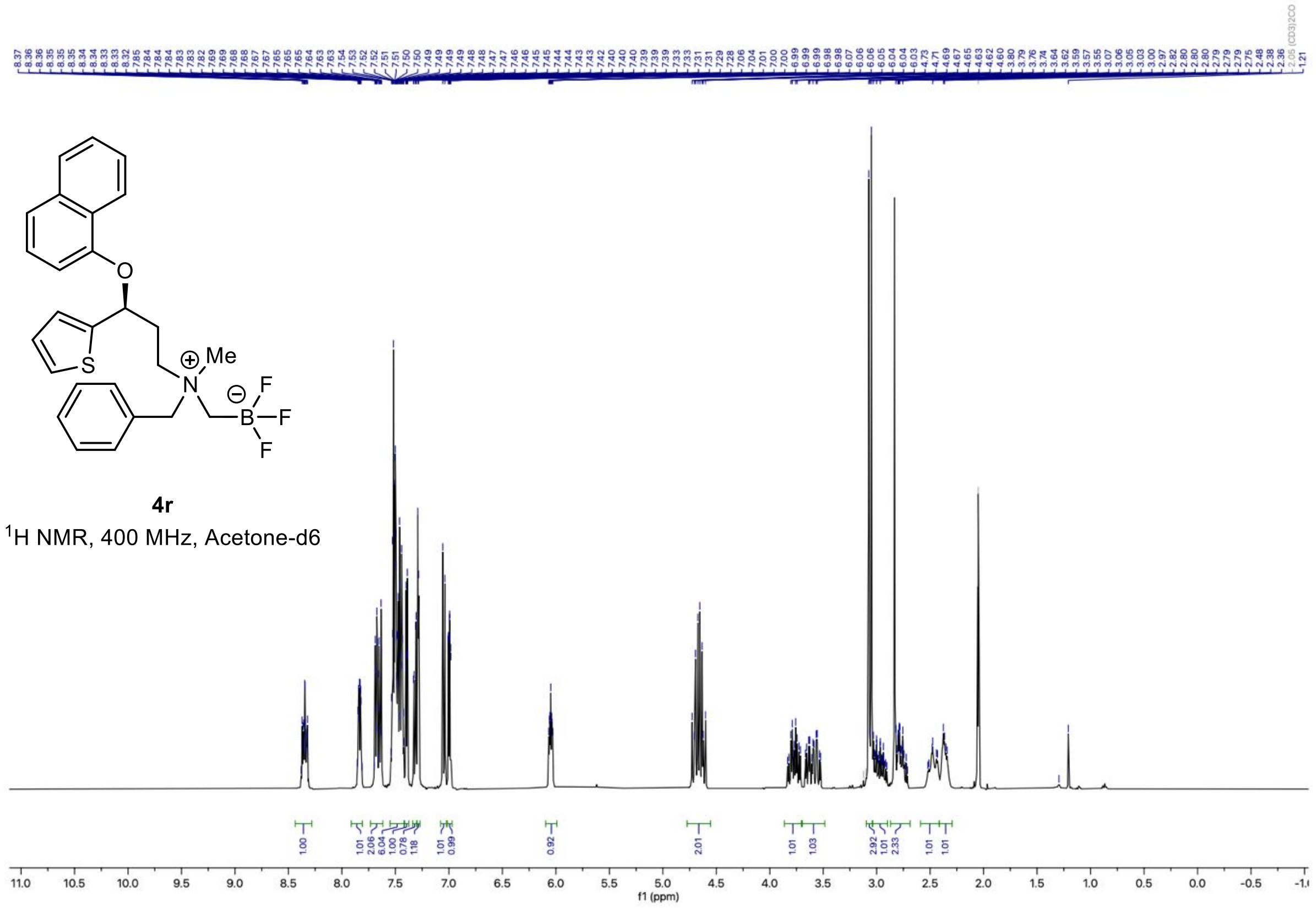

4r

^{1}H NMR, 400 MHz, Acetone-d6

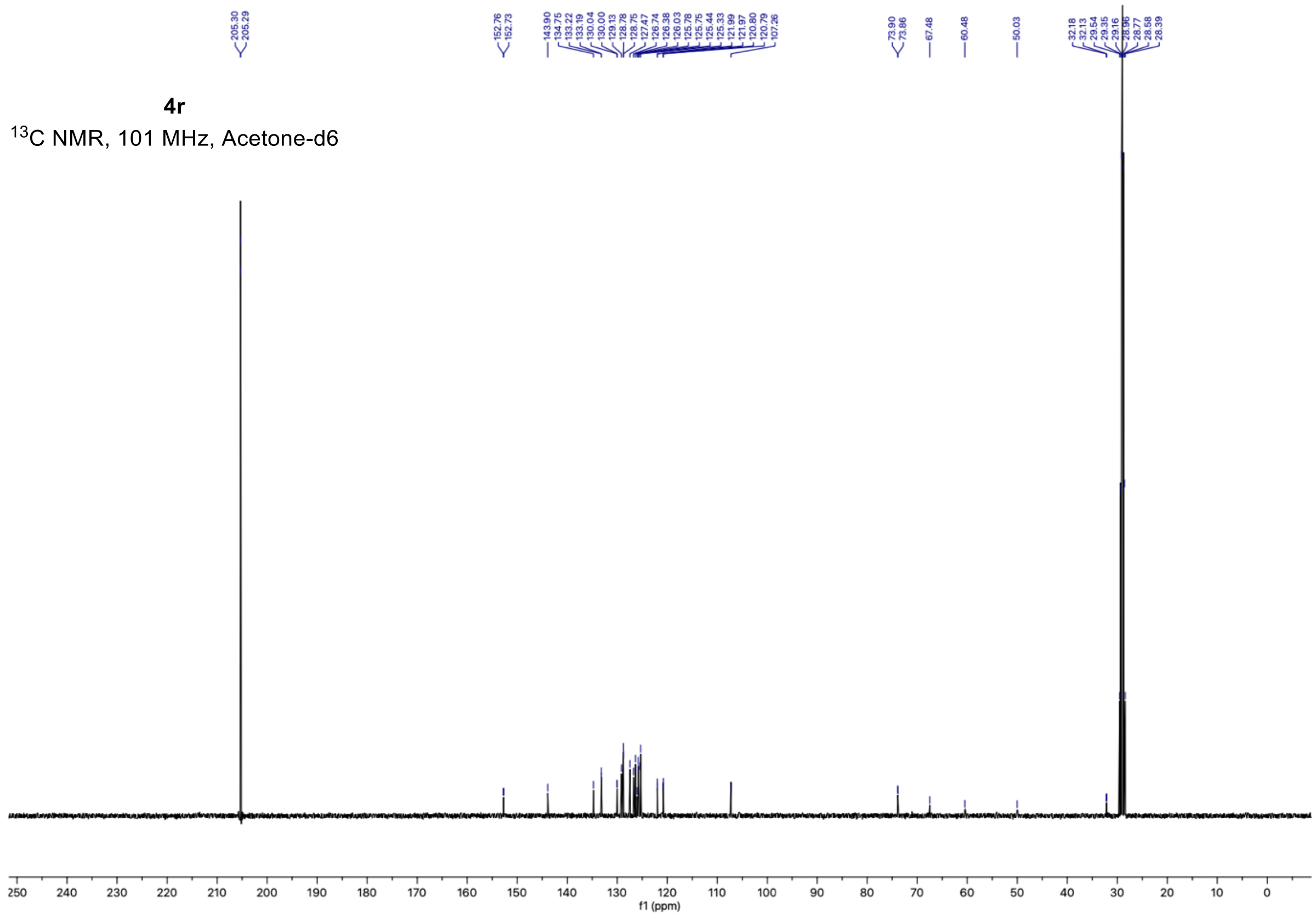

4r

^{13}C NMR, 101 MHz, Acetone-d6

4r

^{19}F NMR, 471 MHz, Acetone-d6

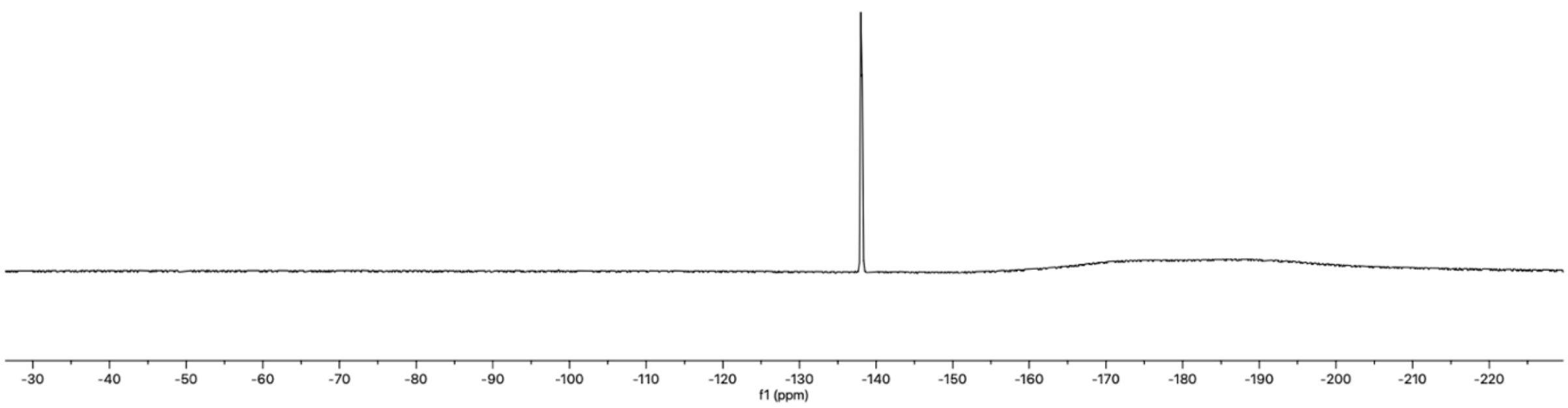

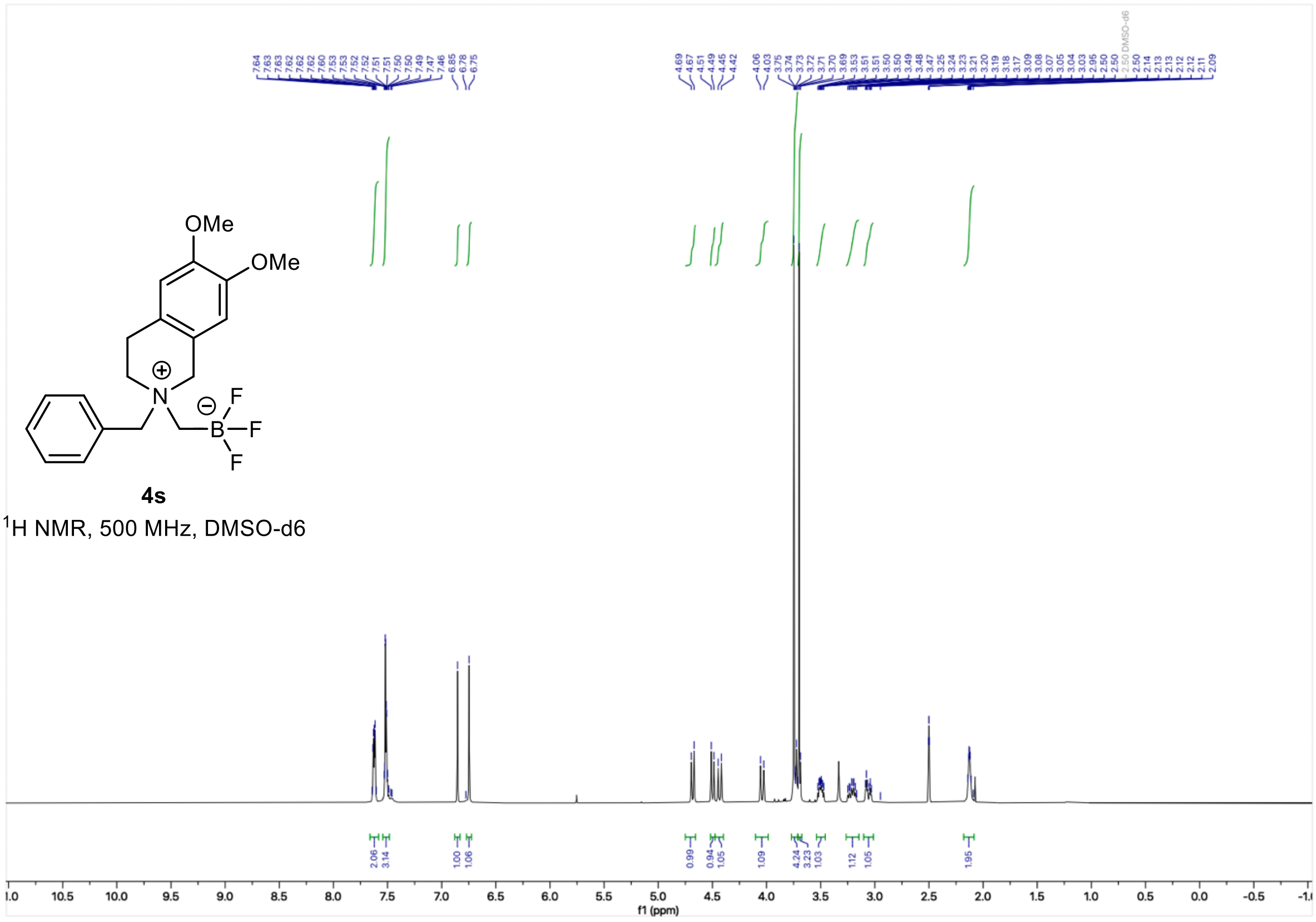

4s

^{1}H NMR, 500 MHz, DMSO-d6

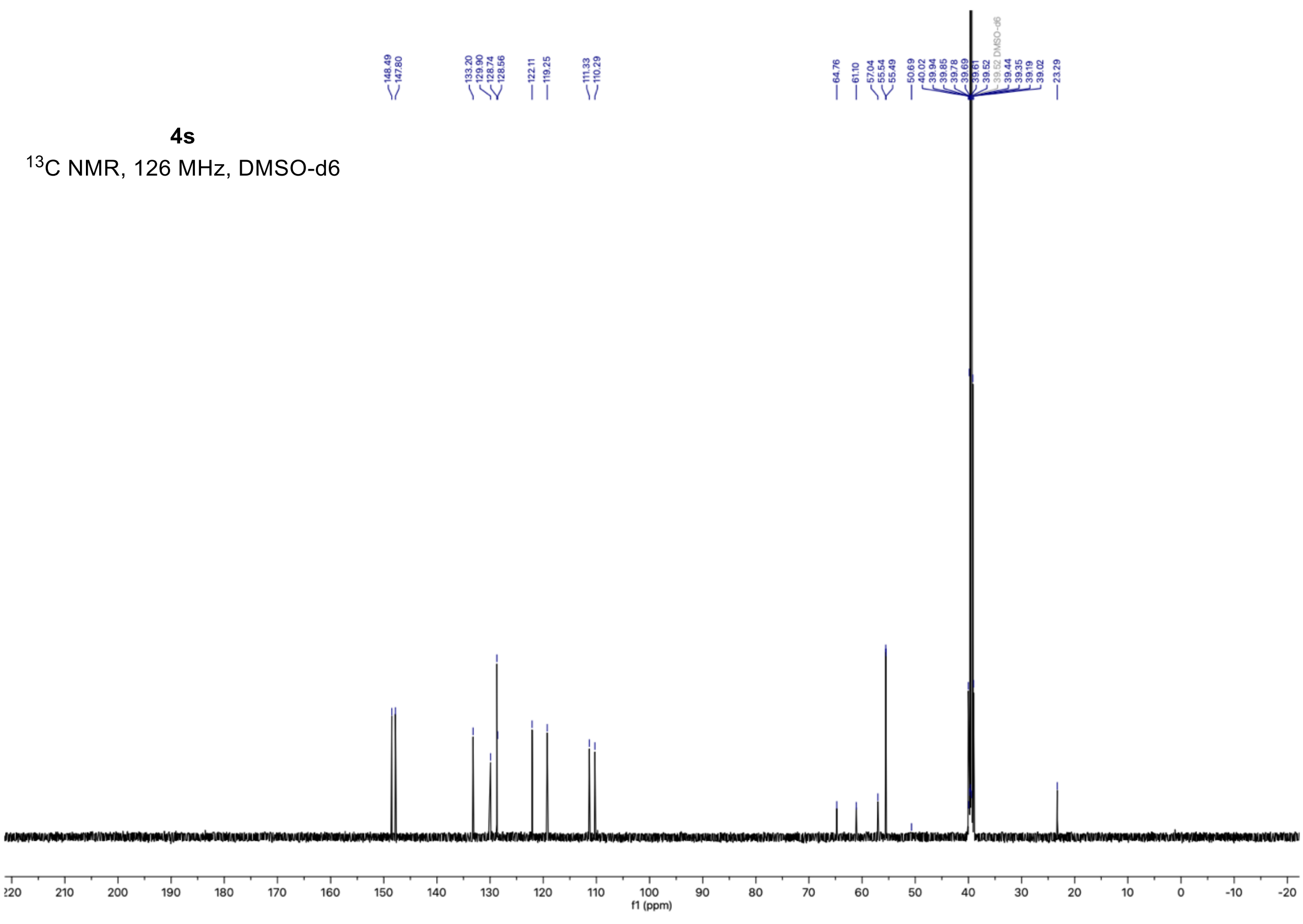

4s

^{13}C NMR, 126 MHz, DMSO-d6

4s

^{19}F NMR, 376 MHz, DMSO-d6

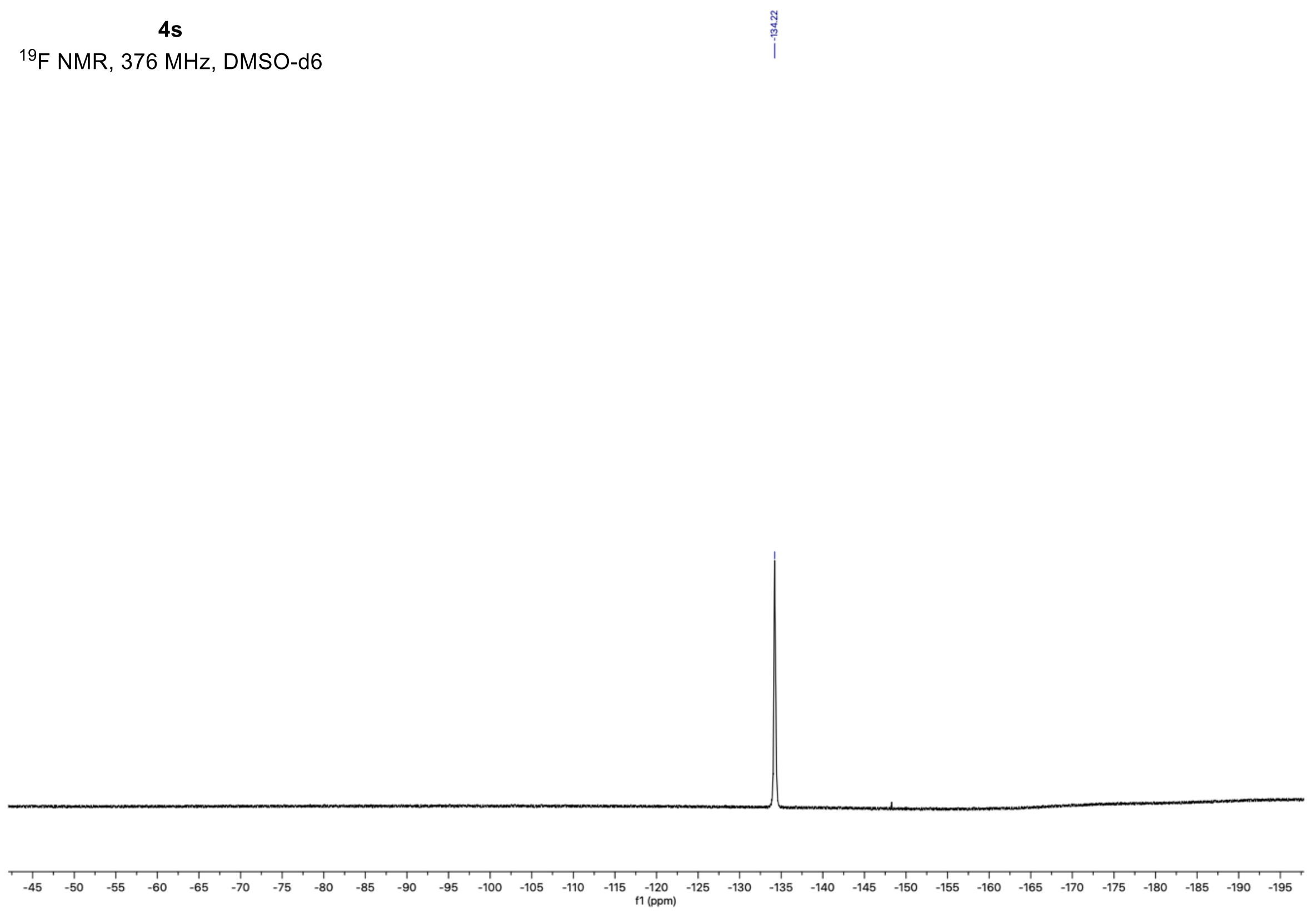

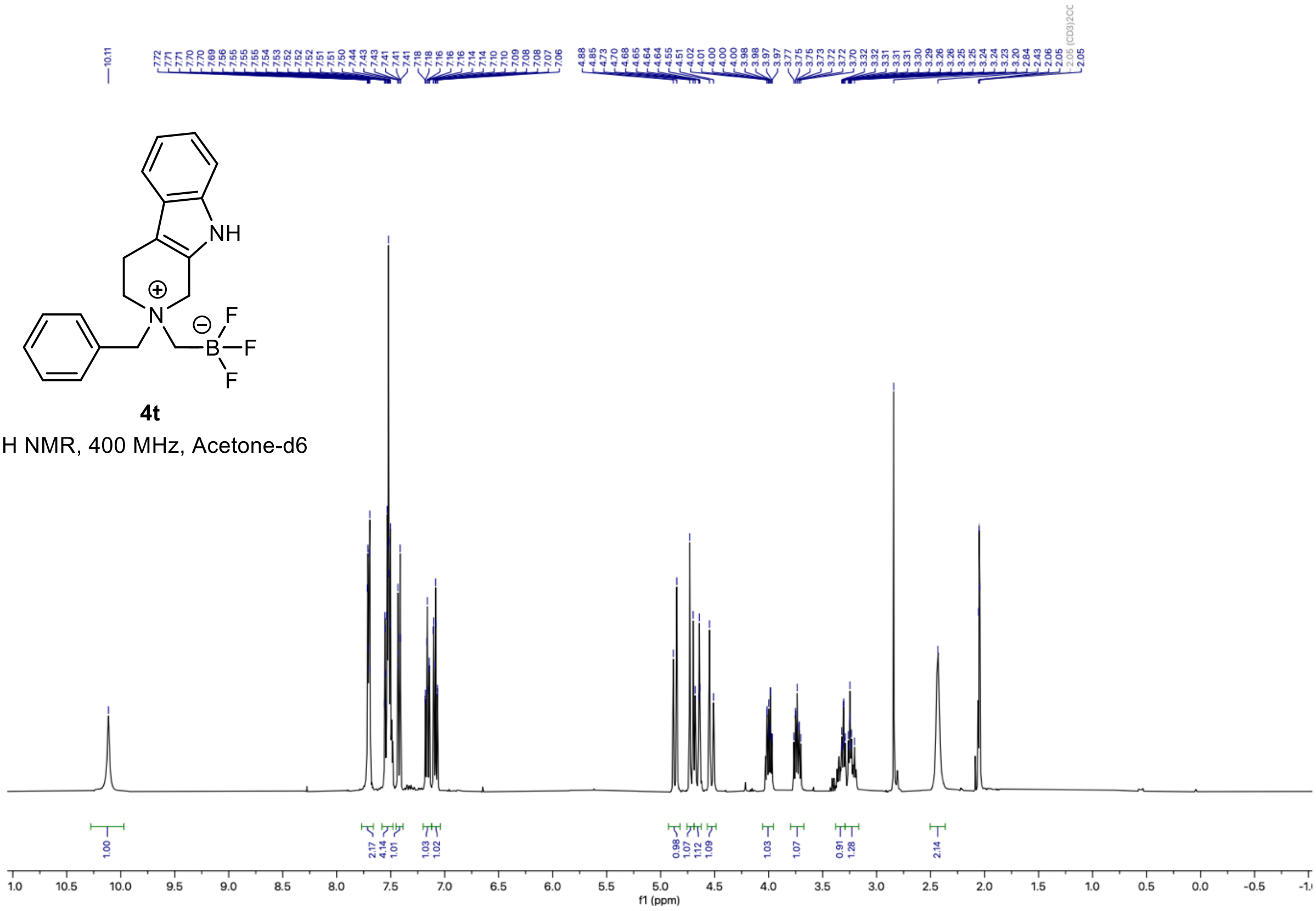

4t

^1H NMR, 400 MHz, Acetone-d6

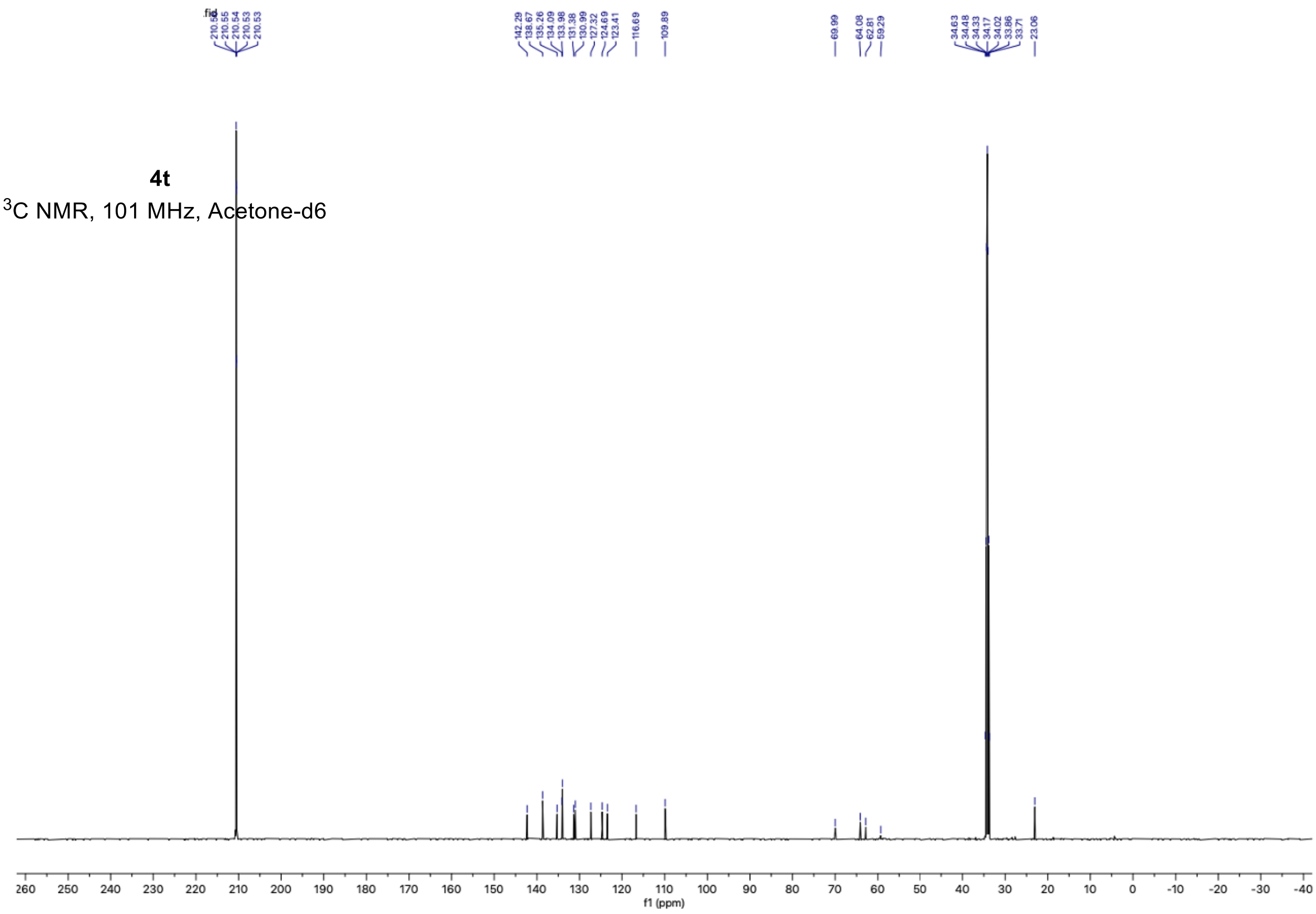

4t

^{13}C NMR, 101 MHz, Acetone-d6

4t
^{19}F NMR, 376 MHz, Acetone-d6

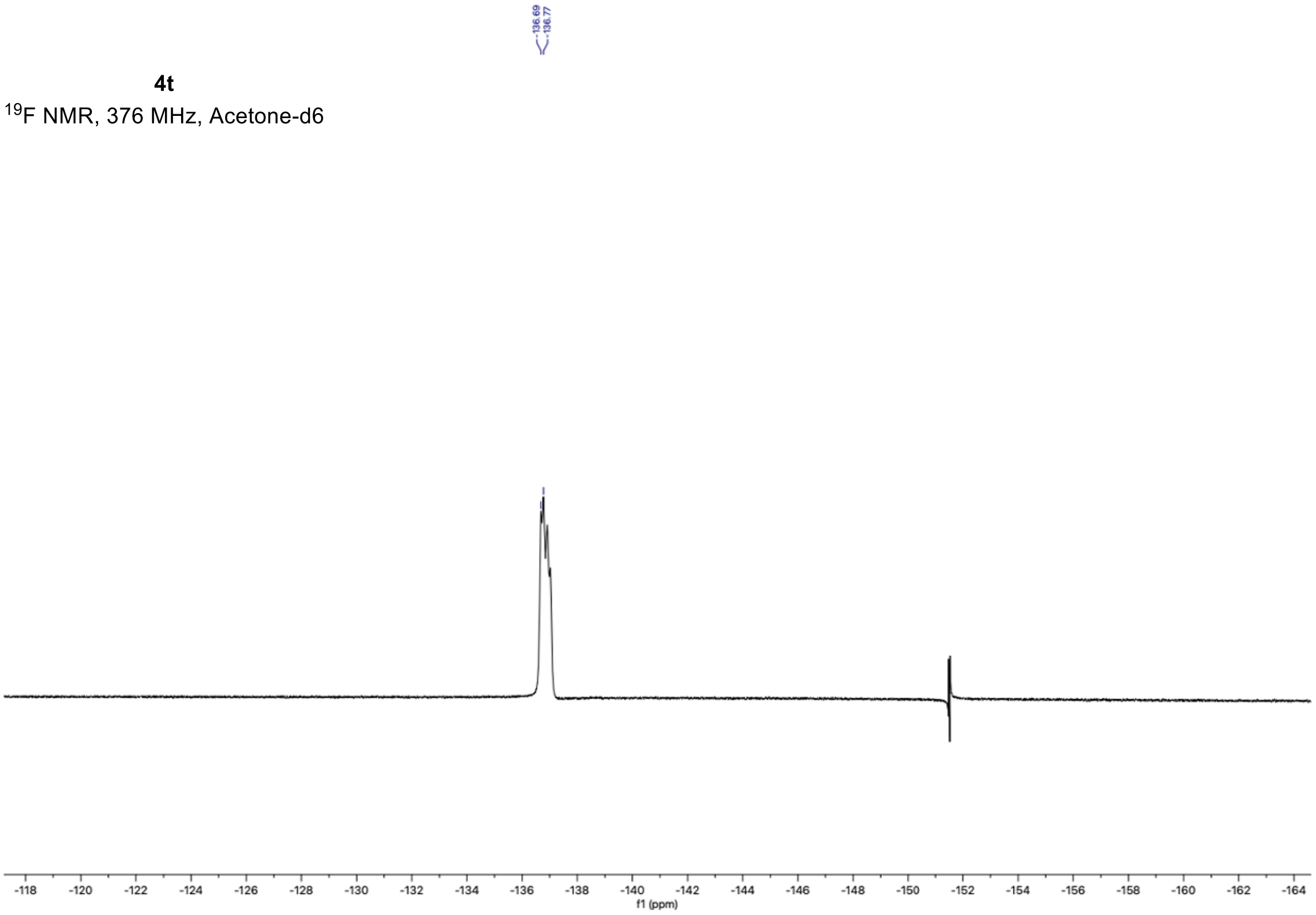

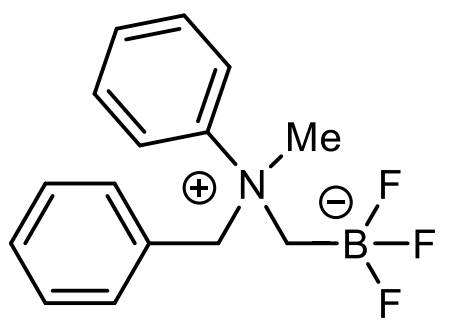

4u

^{1}H NMR, 700 MHz, Acetone-d6

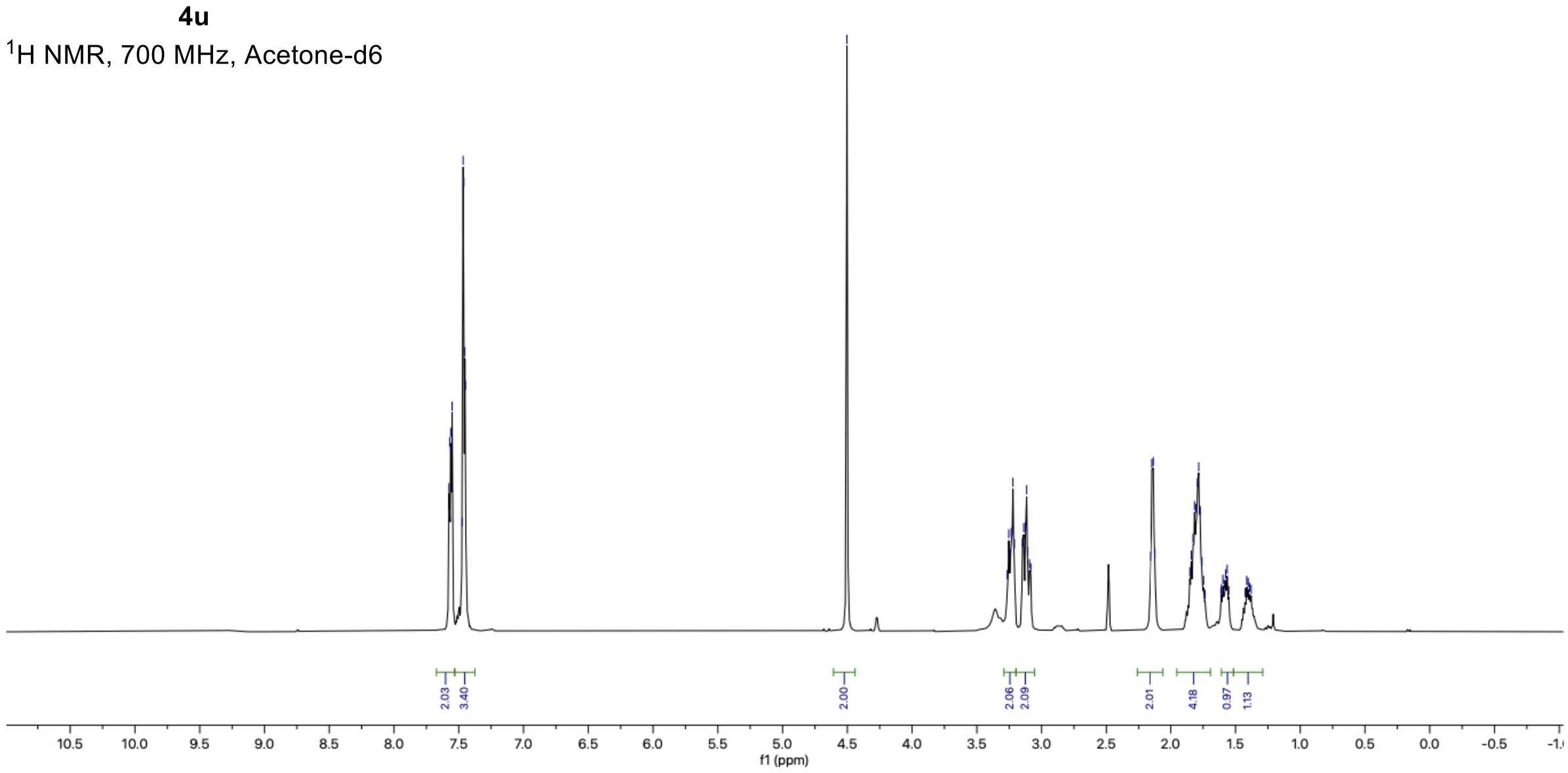

4u

^{13}C NMR, 176 MHz, Acetone-d6

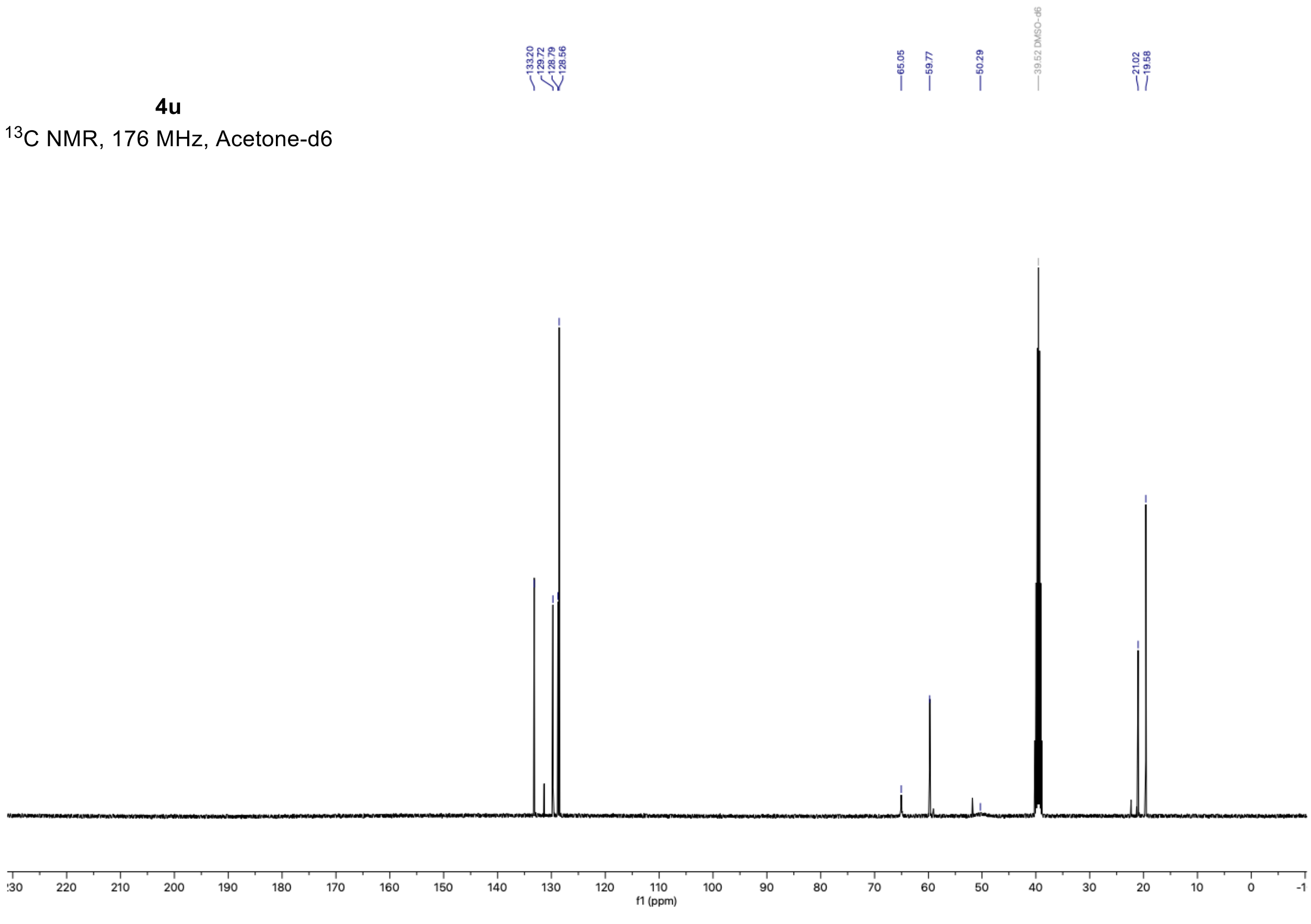

4u

^{19}F NMR, 471 MHz, Acetone-d6

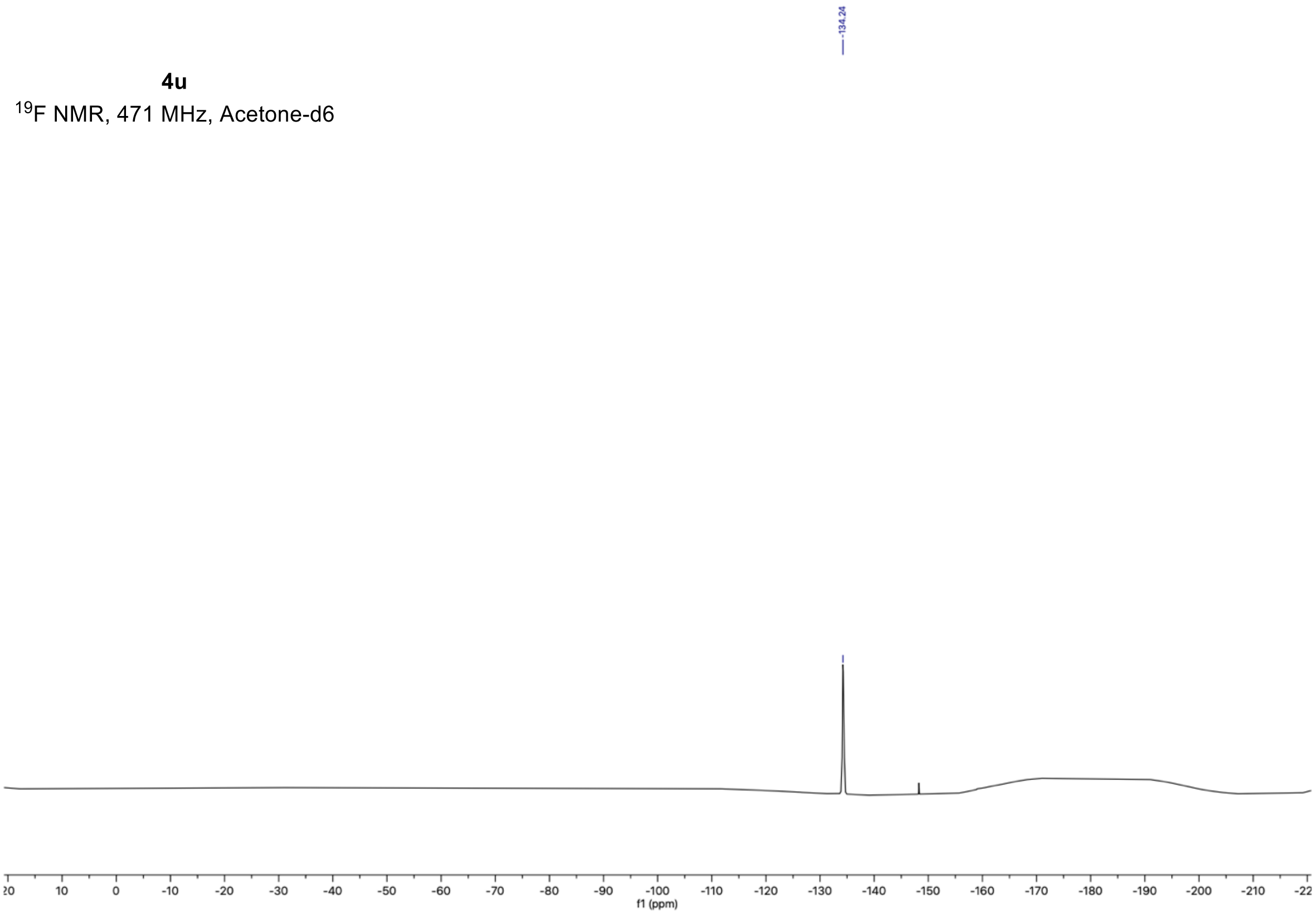

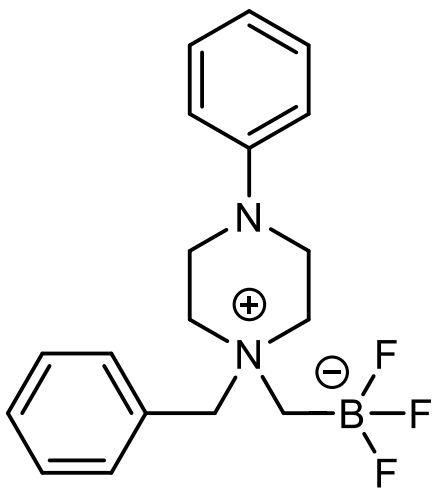

4v

^{1}H NMR, 700 MHz, DMSO-d6

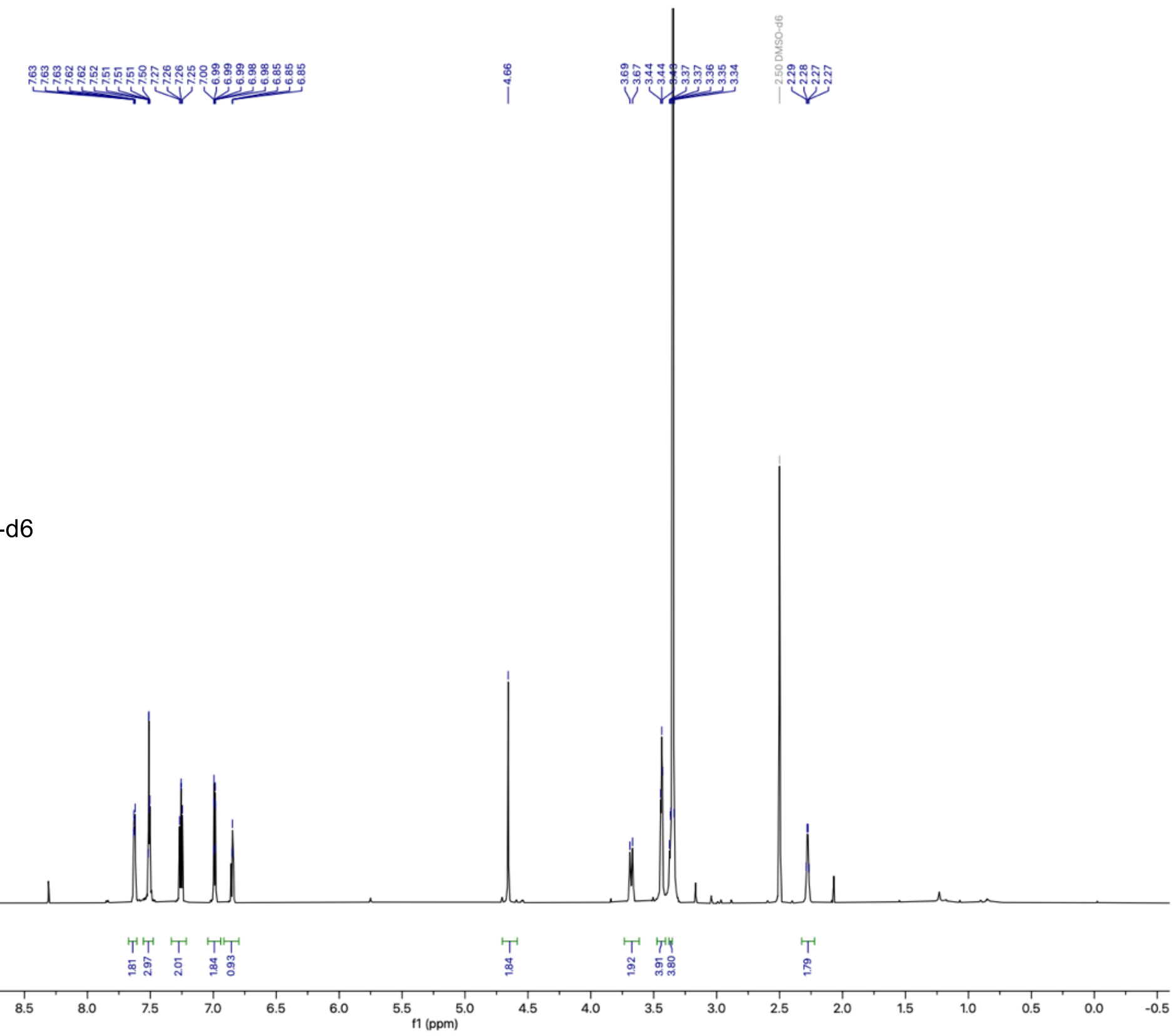

4v

^{13}C NMR, 176 MHz, DMSO-d6

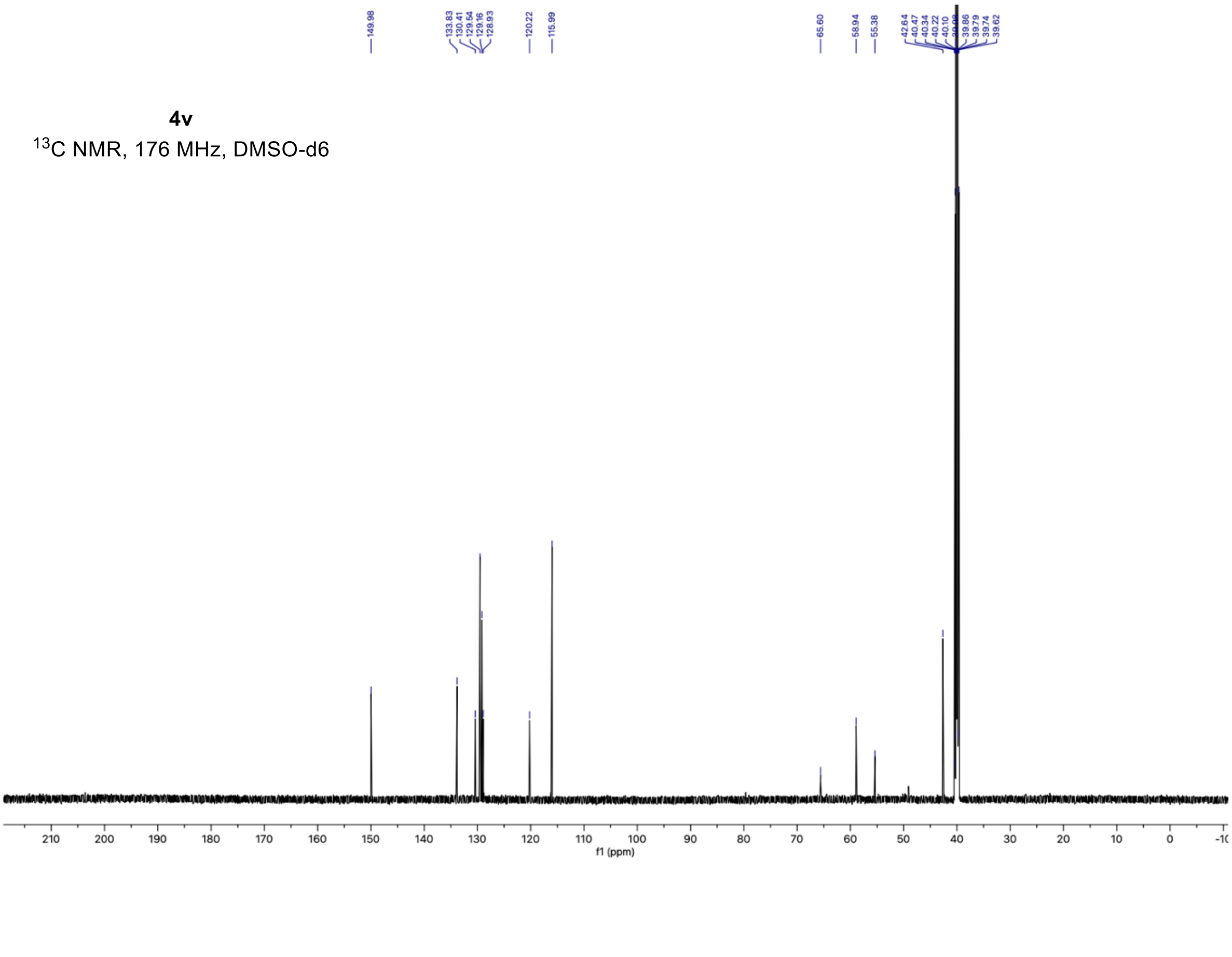

4v
^{19}F NMR, 471 MHz, DMSO-d6

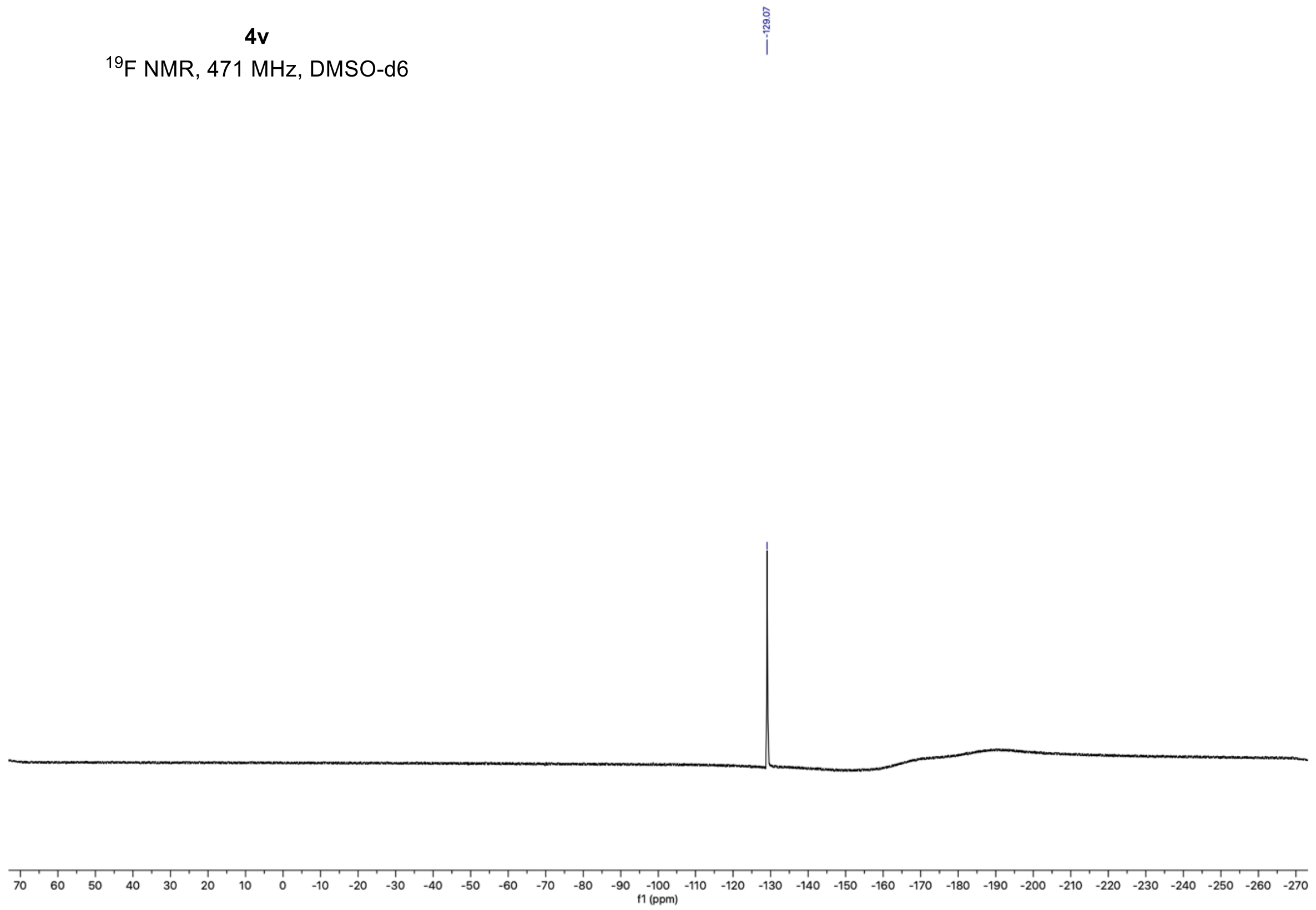

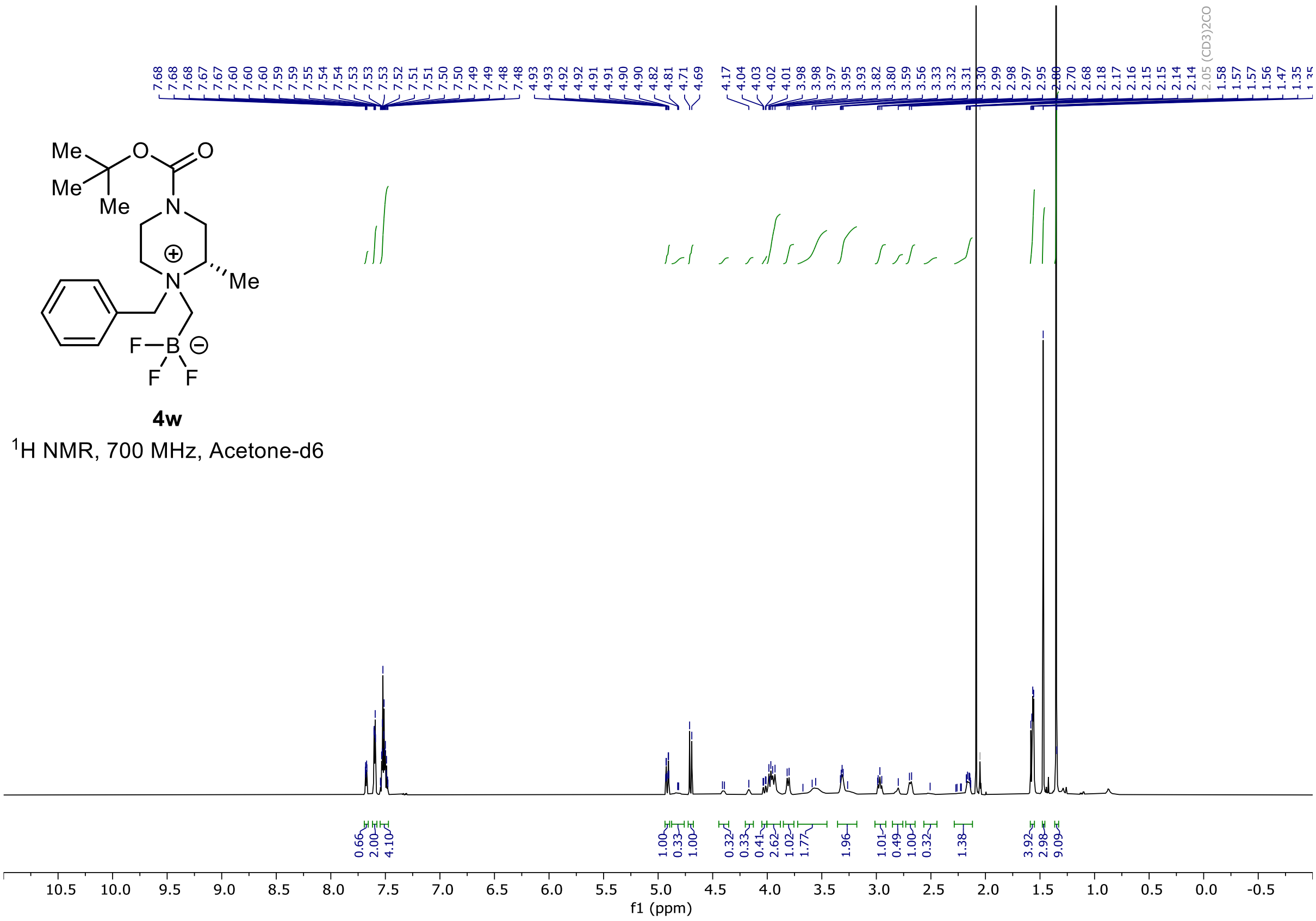
4w
1H NMR, 700 MHz, Acetone-d6
f1 (ppm)

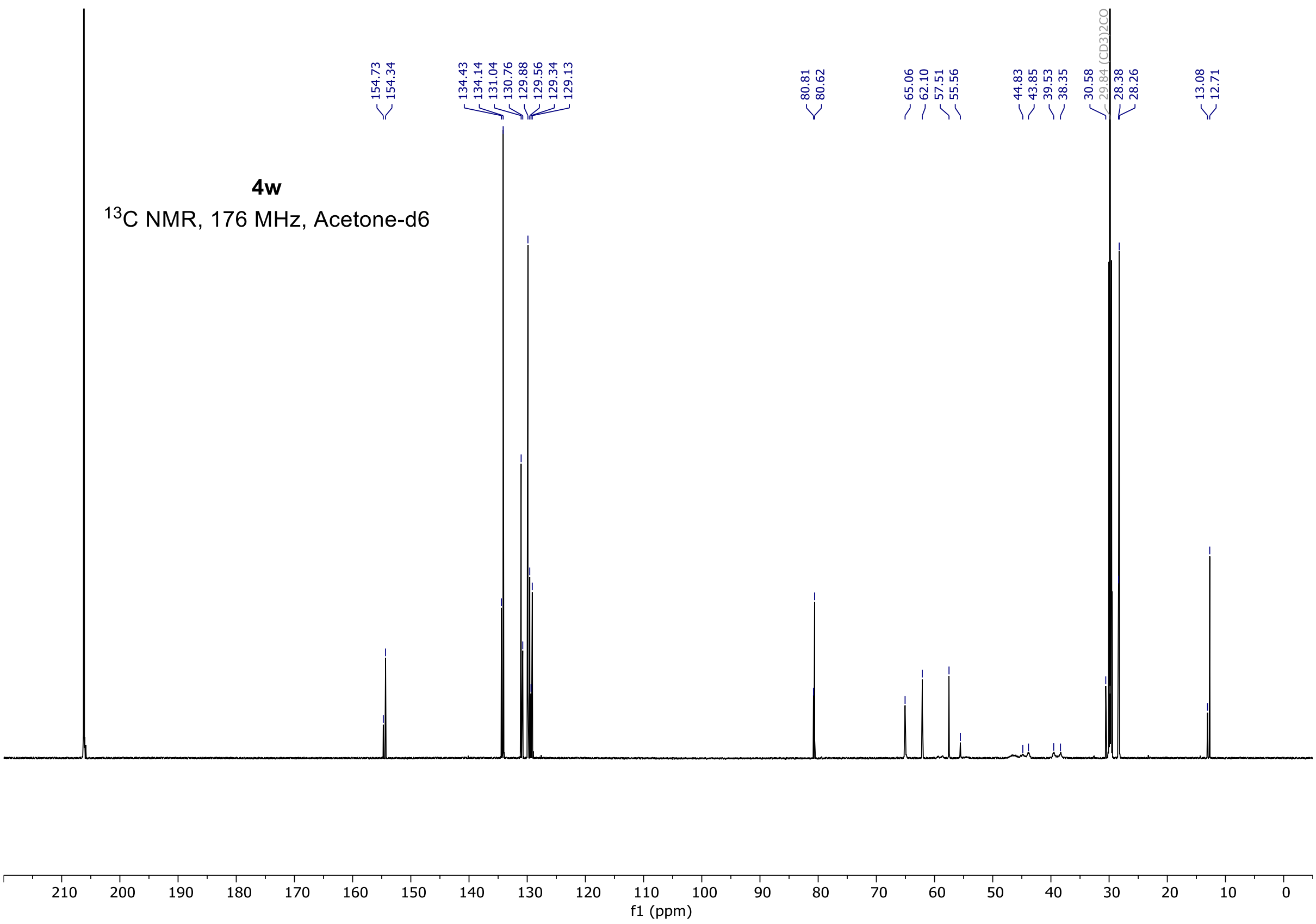
4w
13C NMR, 176 MHz, Acetone-d6
f1 (ppm)

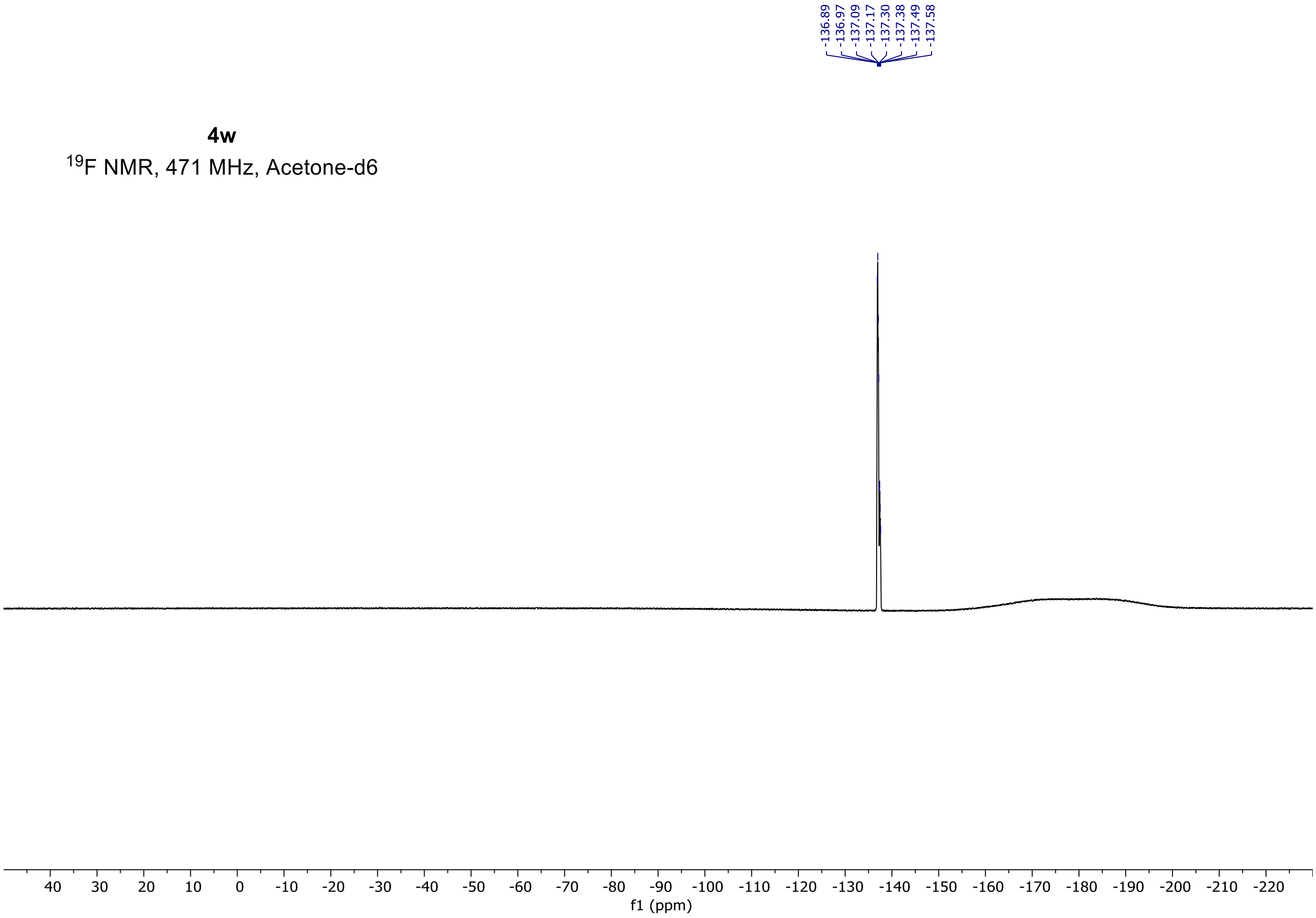

-136.89
-136.97
-137.09
-137.17
-137.30
-137.38
-137.49
-137.58
4w
19F NMR, 471 MHz, Acetone-d6
40 30 20 10 0 -10 -20 -30 -40 -50 -60 -70 -80 -90 -100 -110 -120 -130 -140 -150 -160 -170 -180 -190 -200 -210 -220
f1 (ppm)

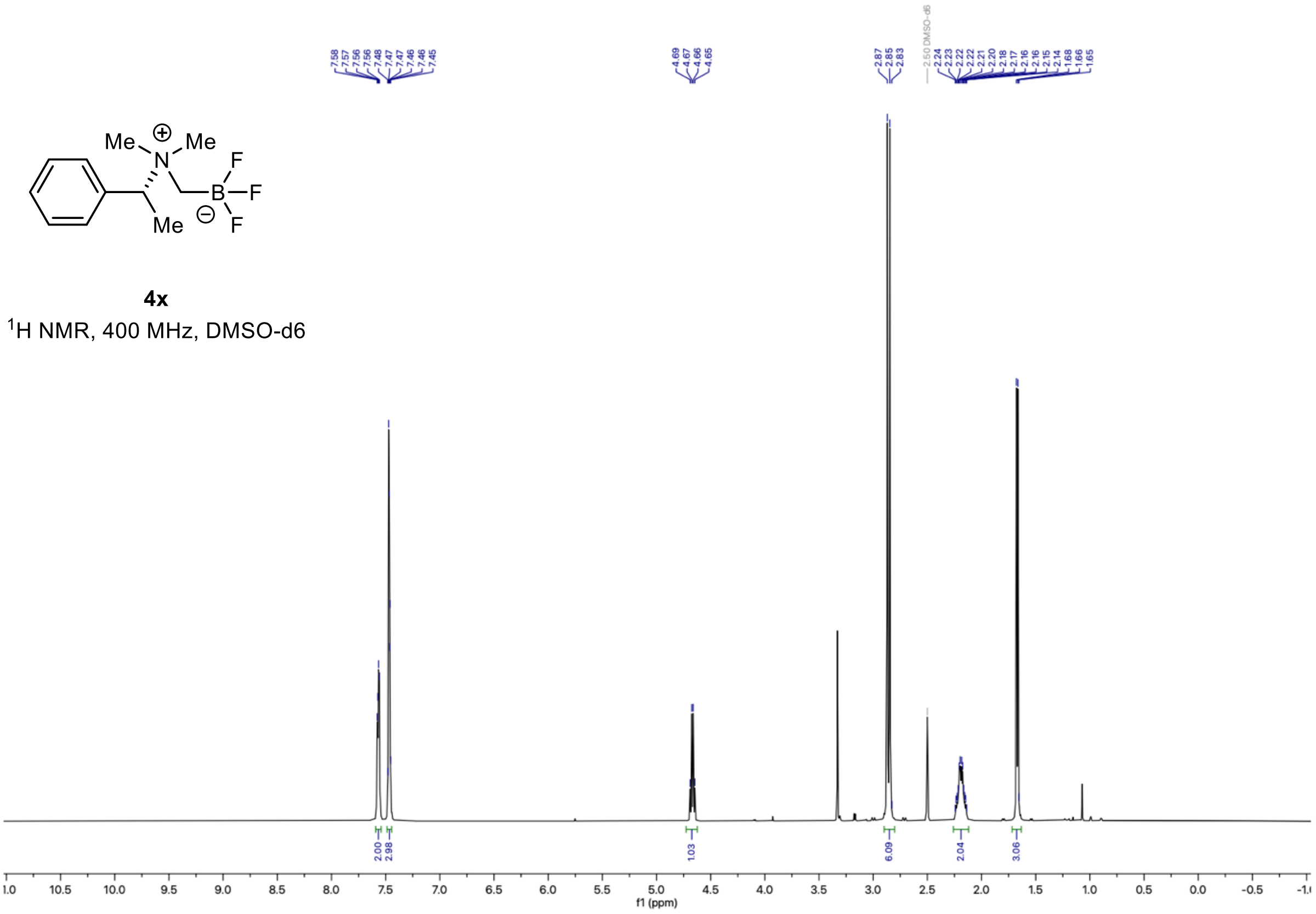

4x

^{1}H NMR, 400 MHz, DMSO-d6

4x

^{13}C NMR, 126 MHz, DMSO-d6

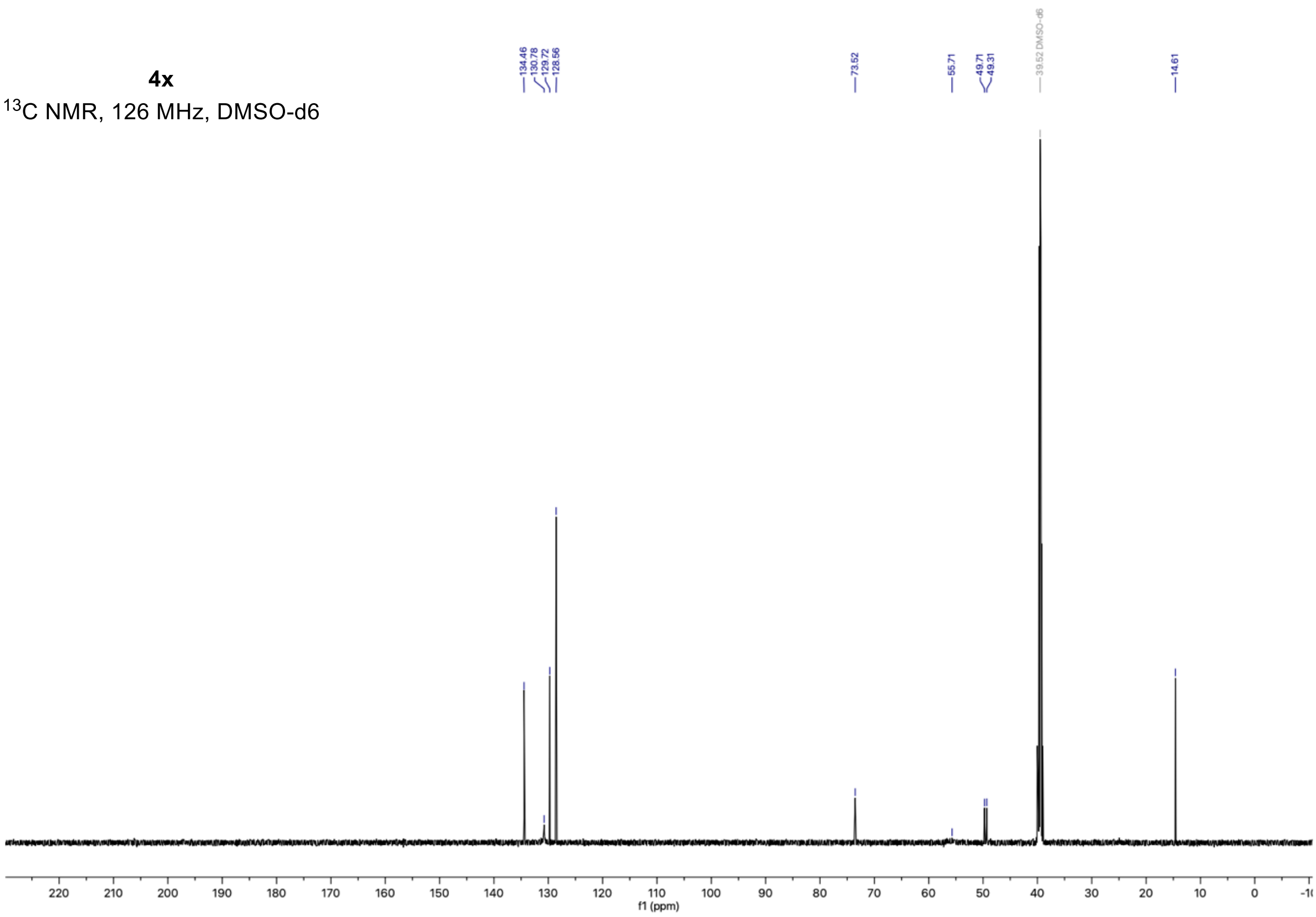

4x

^{19}F NMR, 471 MHz, DMSO-d6

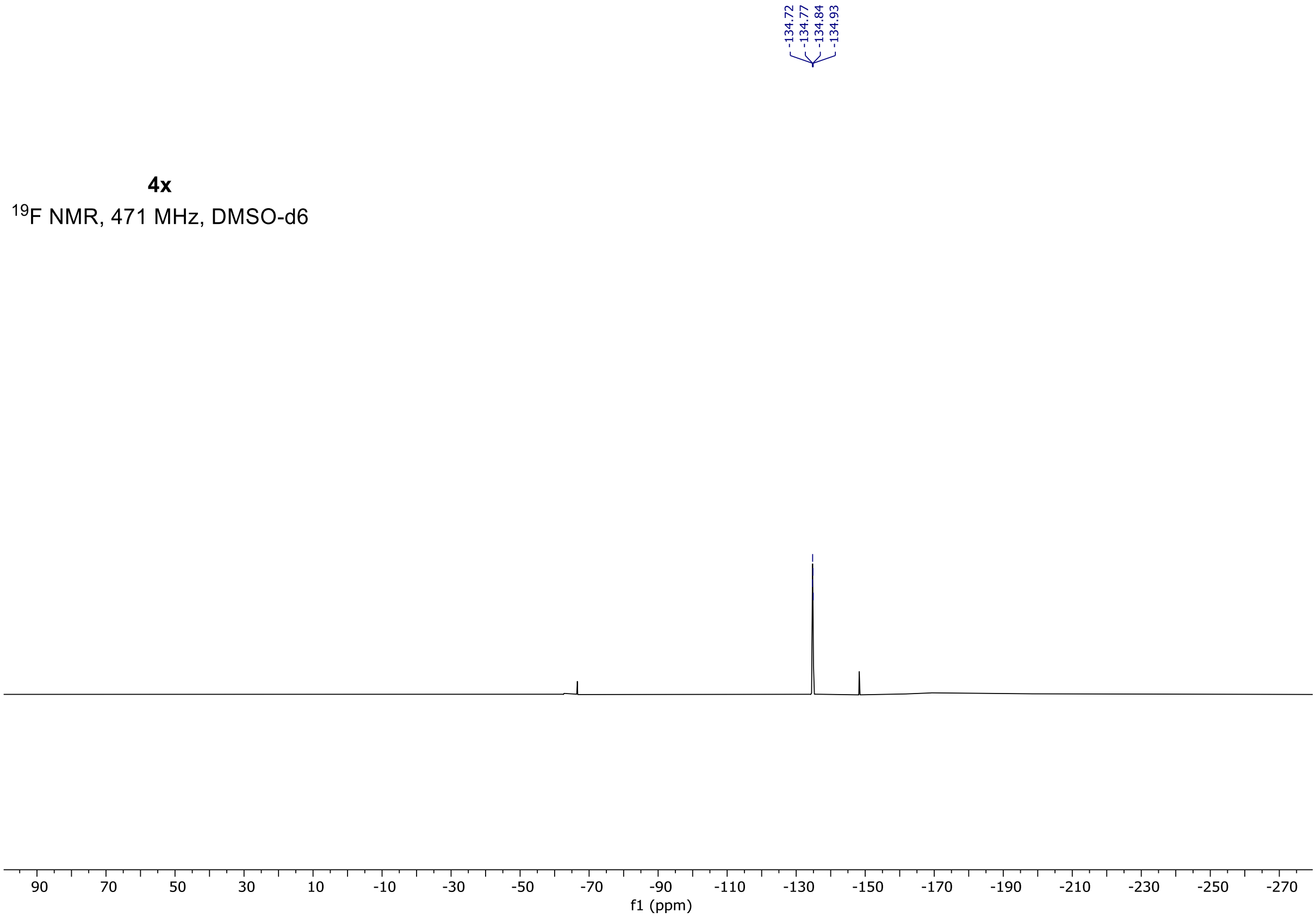

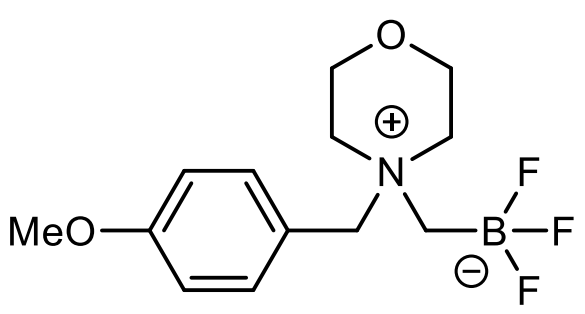

4aa

^{1}H NMR, 700 MHz, DMSO-d6

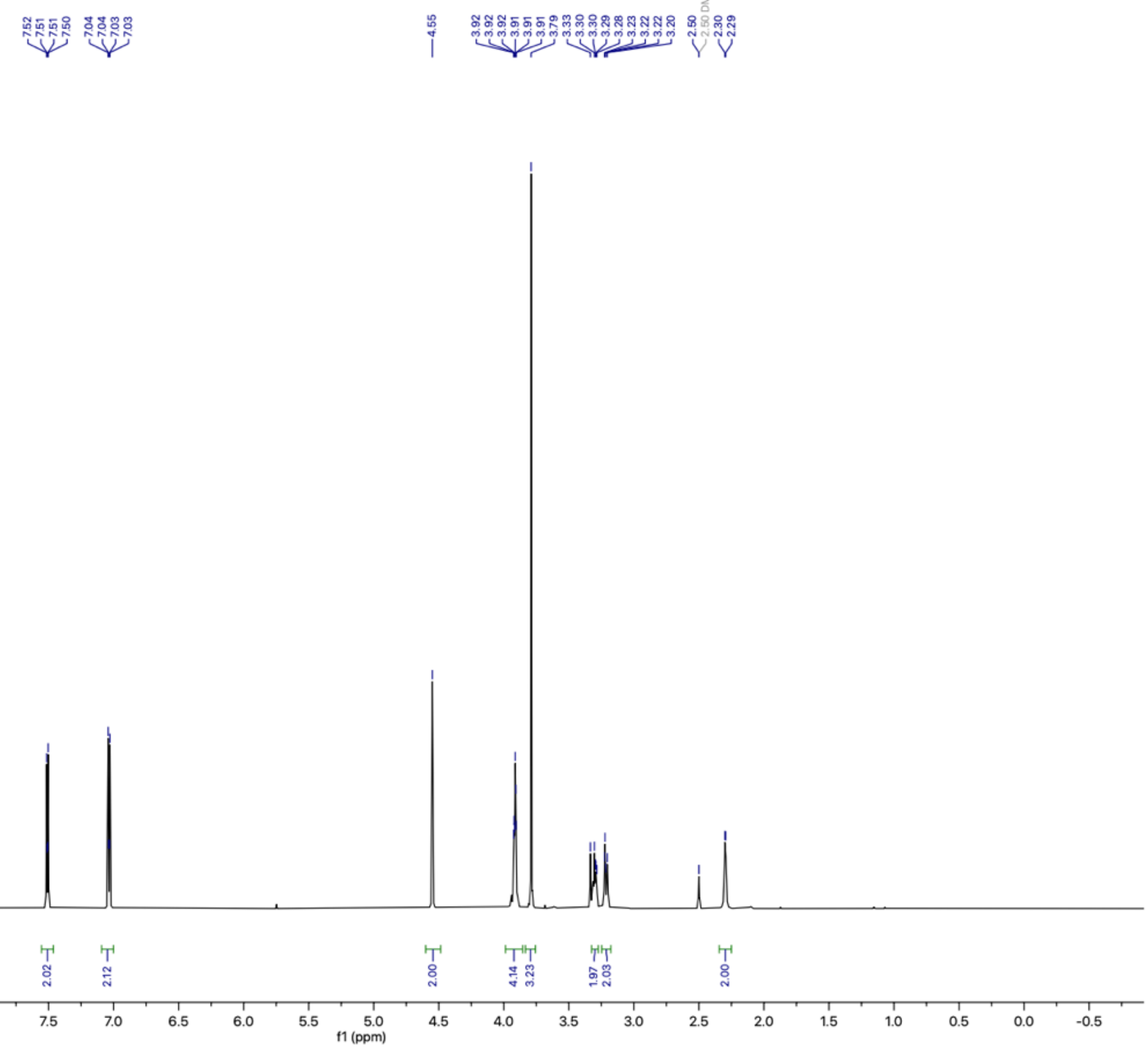

4aa

^{13}C NMR, 176 MHz, DMSO-d6

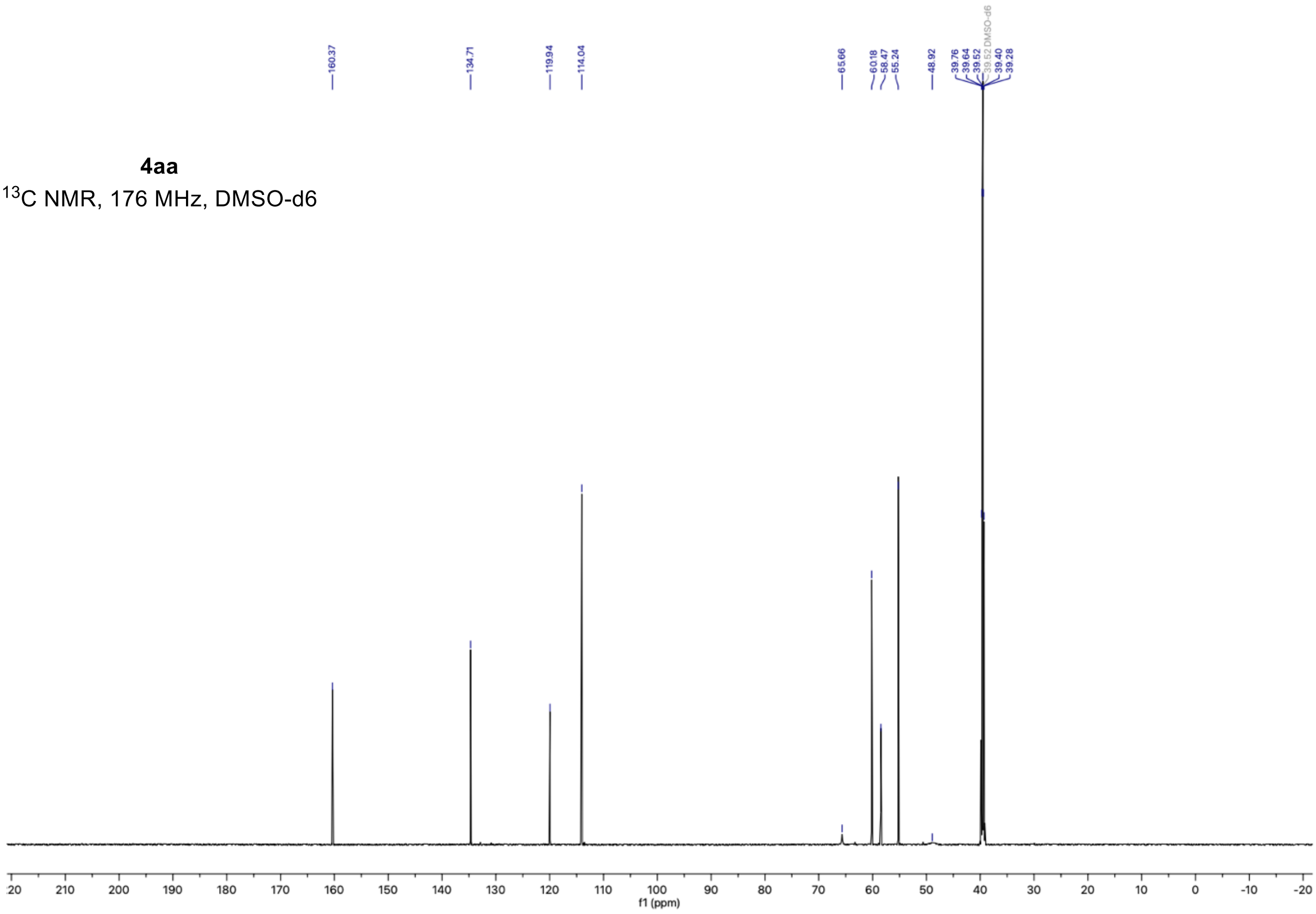

4aa

^{19}F NMR, 471 MHz, DMSO-d6

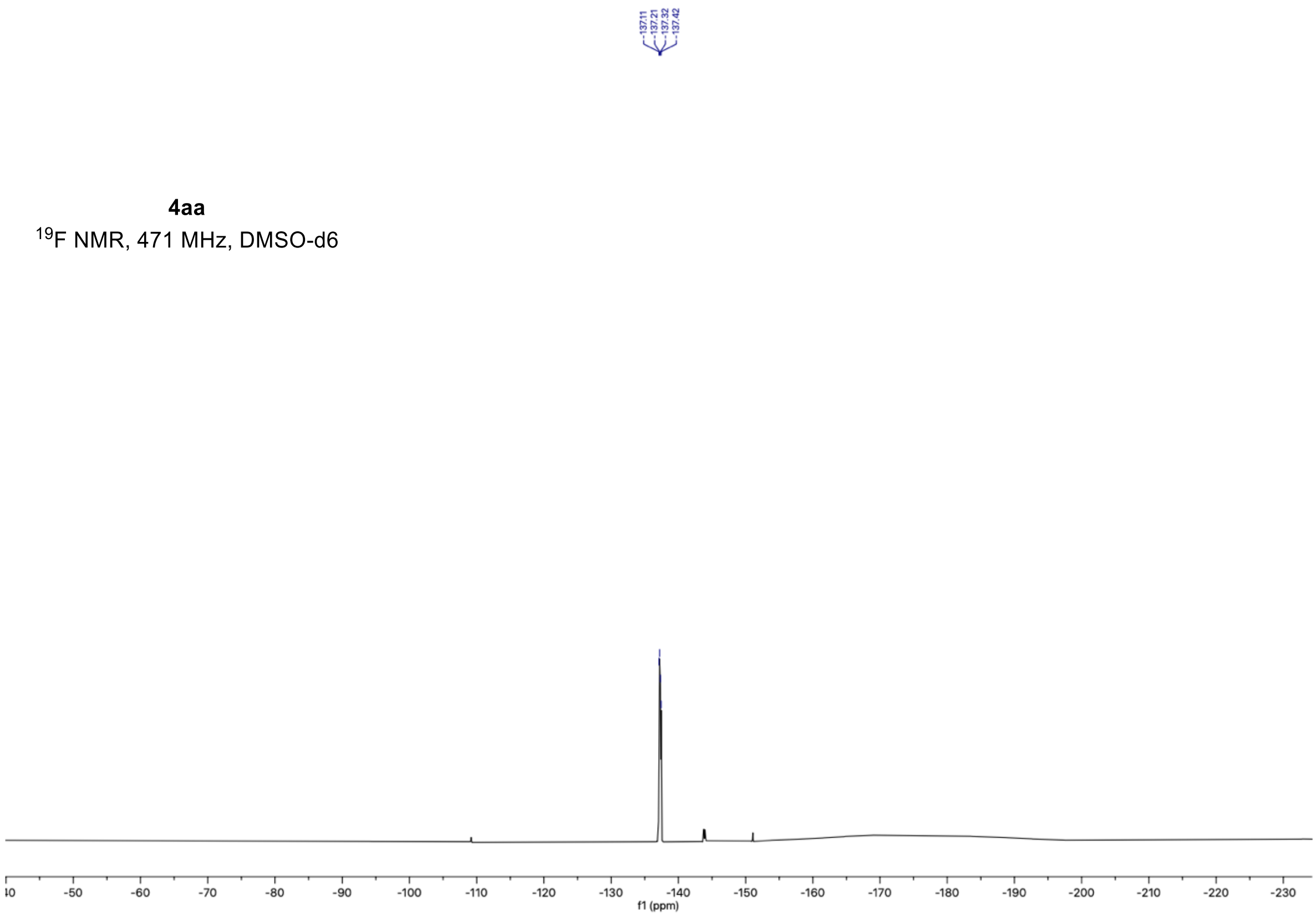

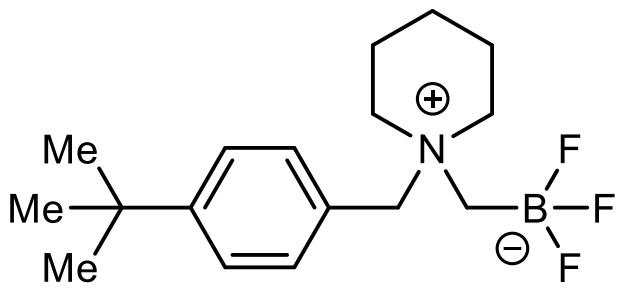

4ab

^{1}H NMR, 500 MHz, DMSO-d6

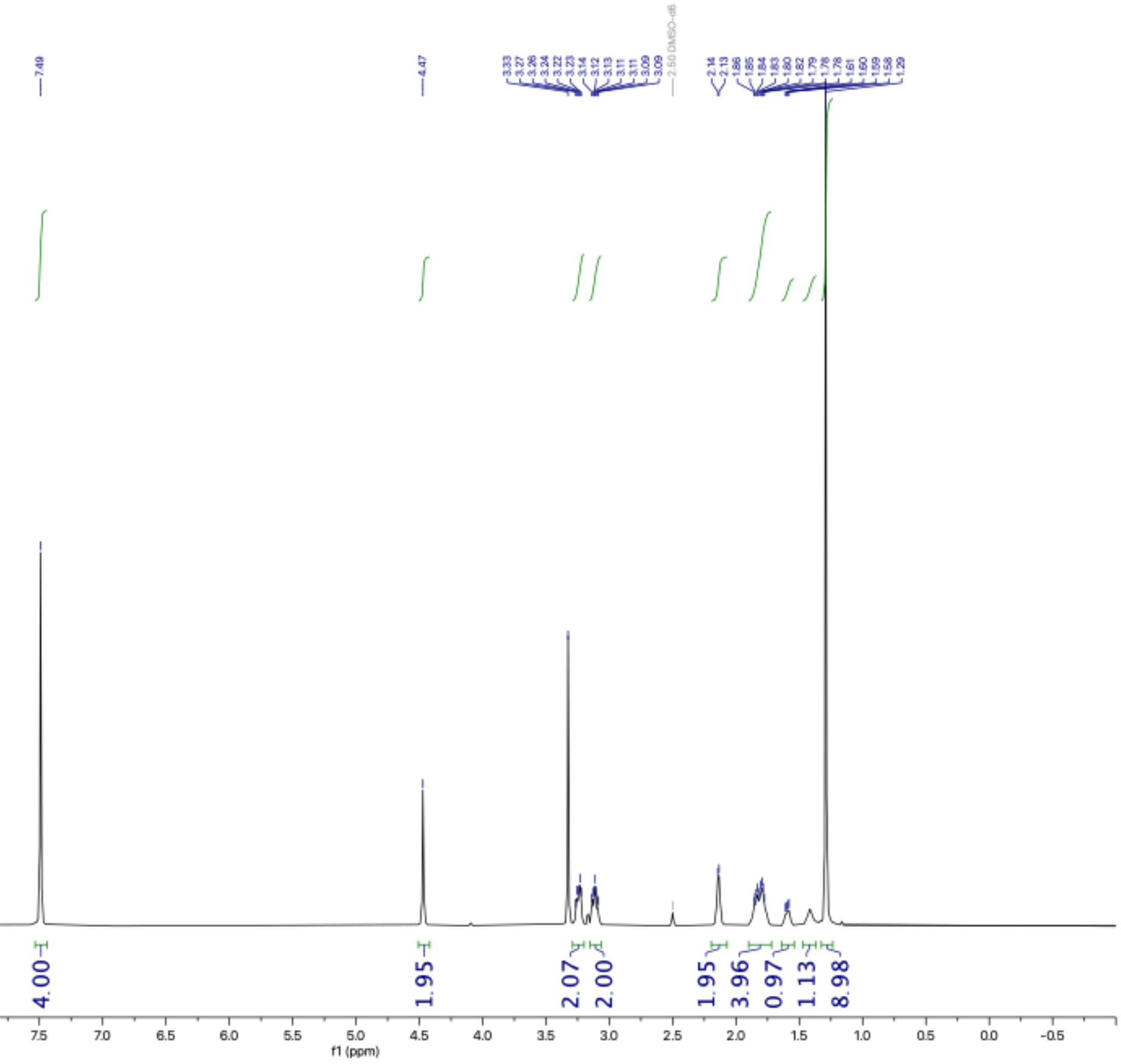

4ab

^{13}C NMR, 126 MHz, DMSO-d6

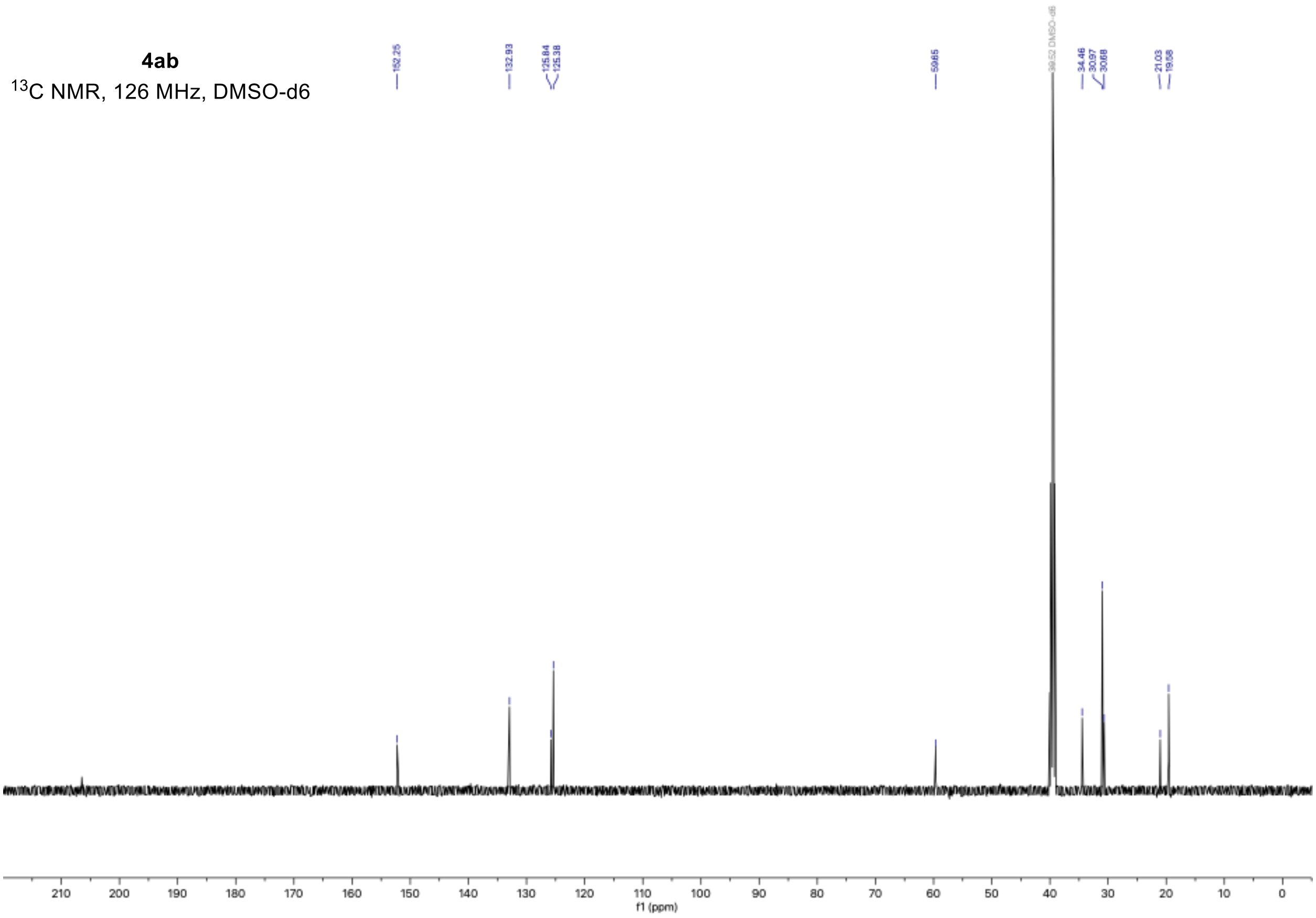

4ab
^{19}F NMR, 471 MHz, DMSO-d6

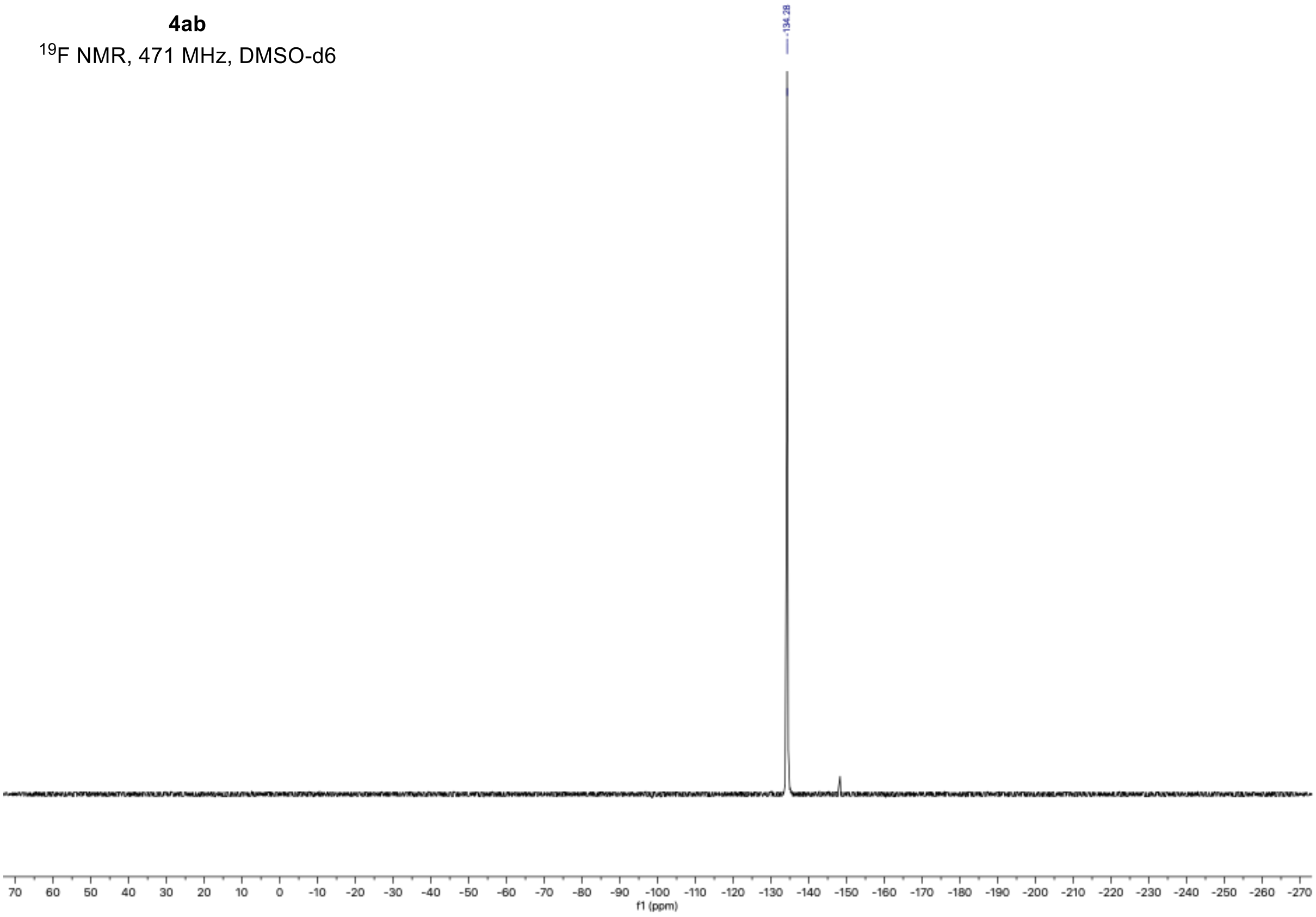

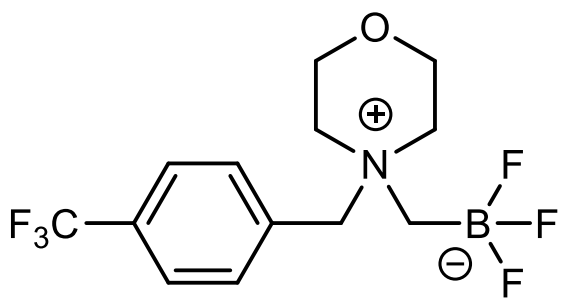

4ac

^{1}H NMR, 500 MHz, DMSO-d6

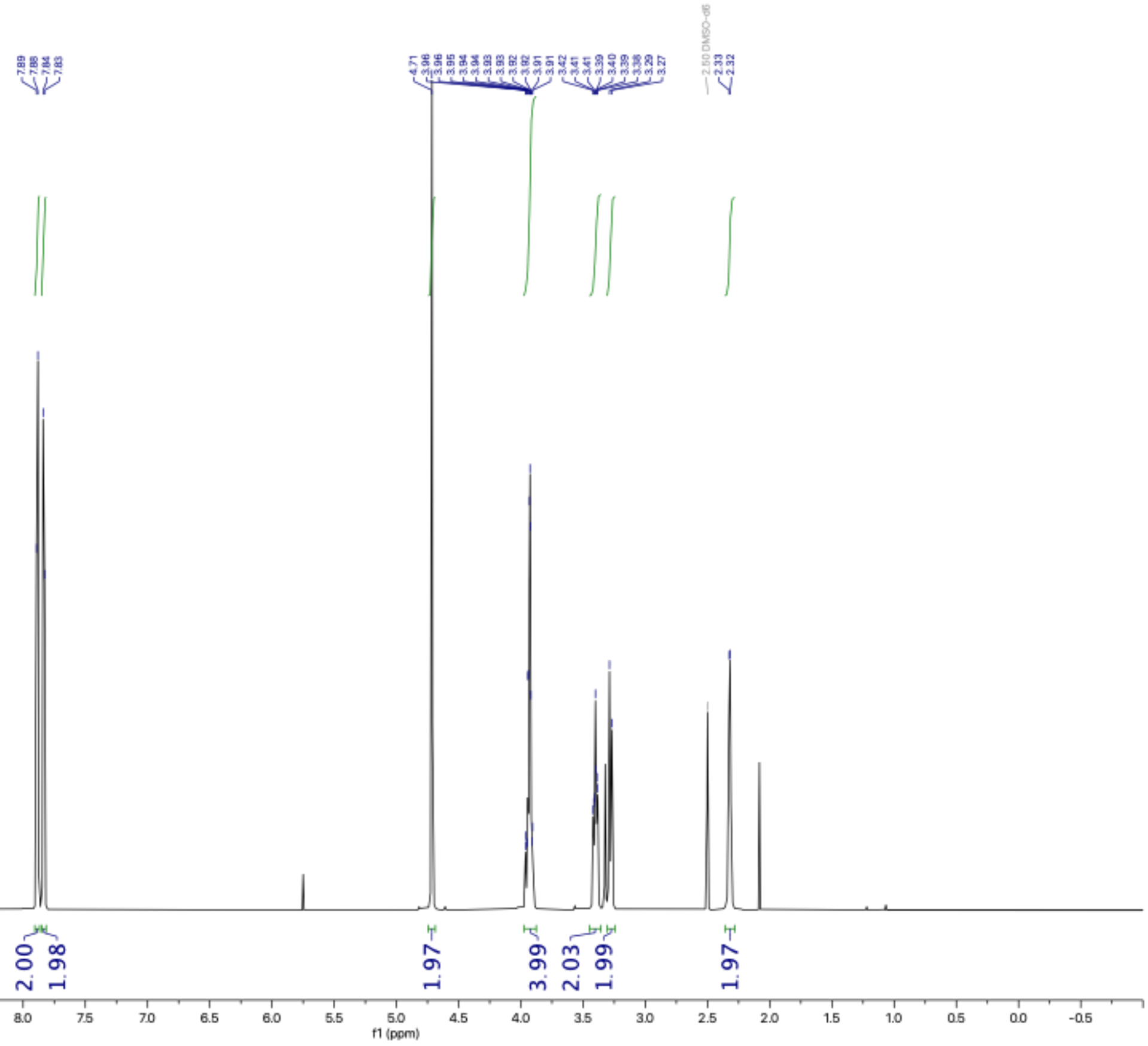

4ac

^{13}C NMR, 126 MHz, DMSO-d6

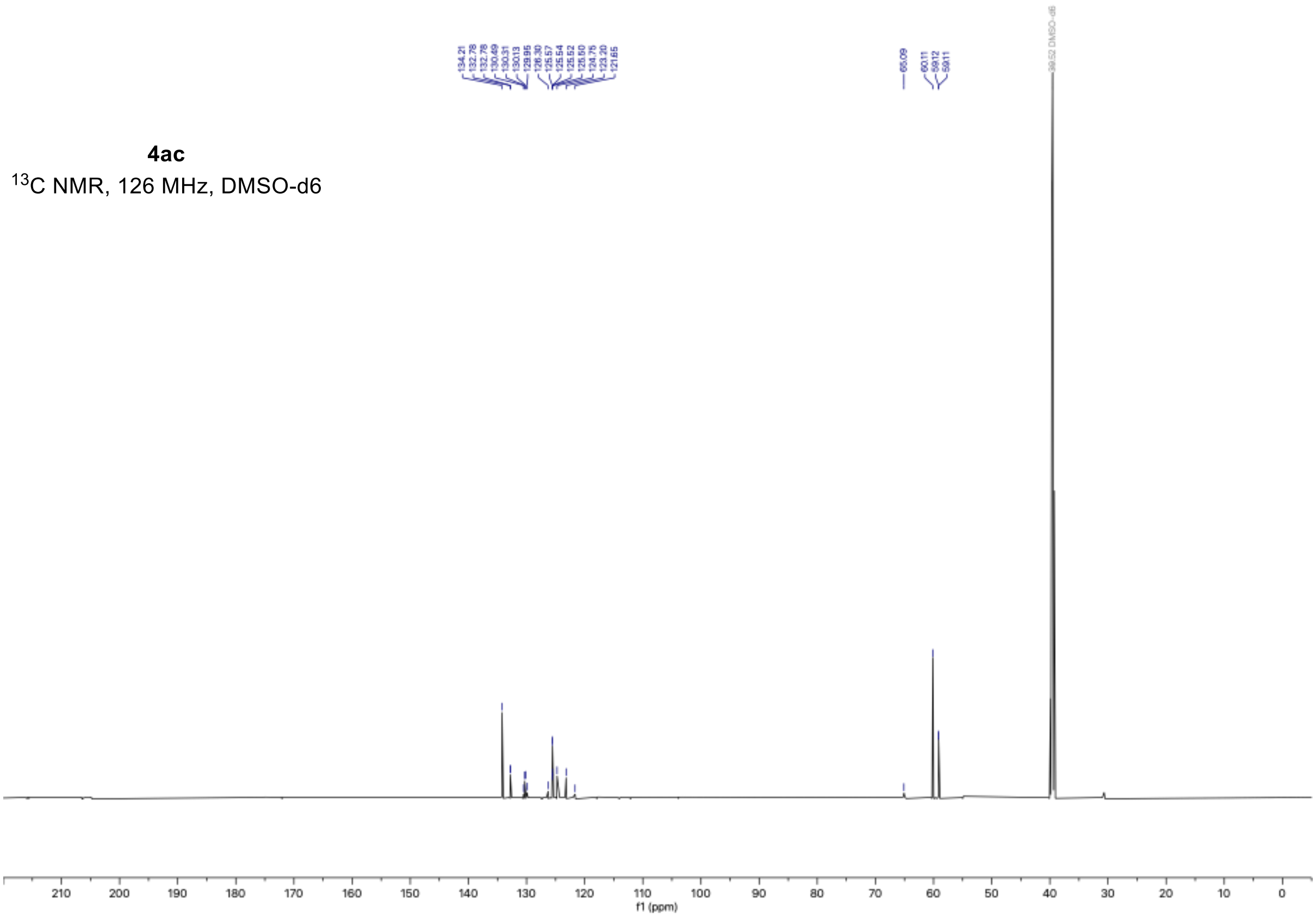

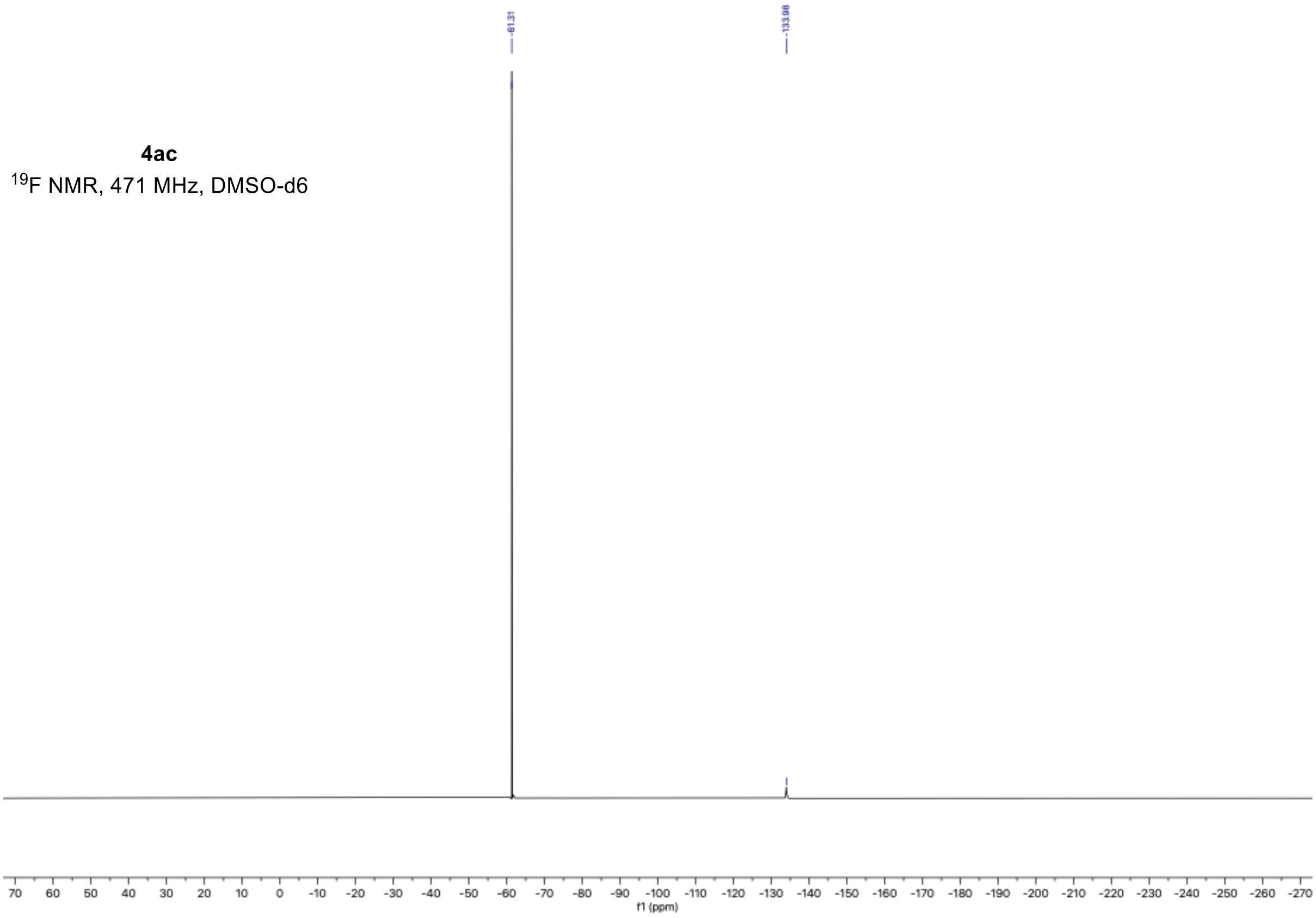
-61.31
-133.98
4ac
19F NMR, 471 MHz, DMSO-d6
70 60 50 40 30 20 10 0 -10 -20 -30 -40 -50 -60 -70 -80 -90 -100 -110 -120 -130 -140 -150 -160 -170 -180 -190 -200 -210 -220 -230 -240 -250 -260 -270
f1 (ppm)

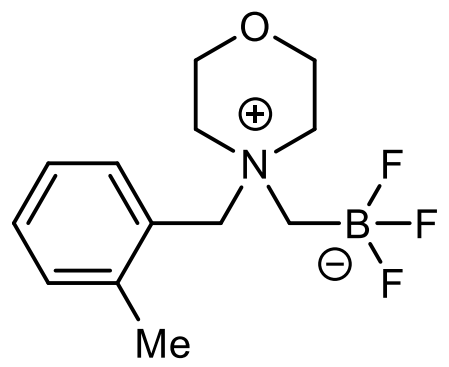

4ad

^{1}H NMR, 500 MHz, DMSO-d6

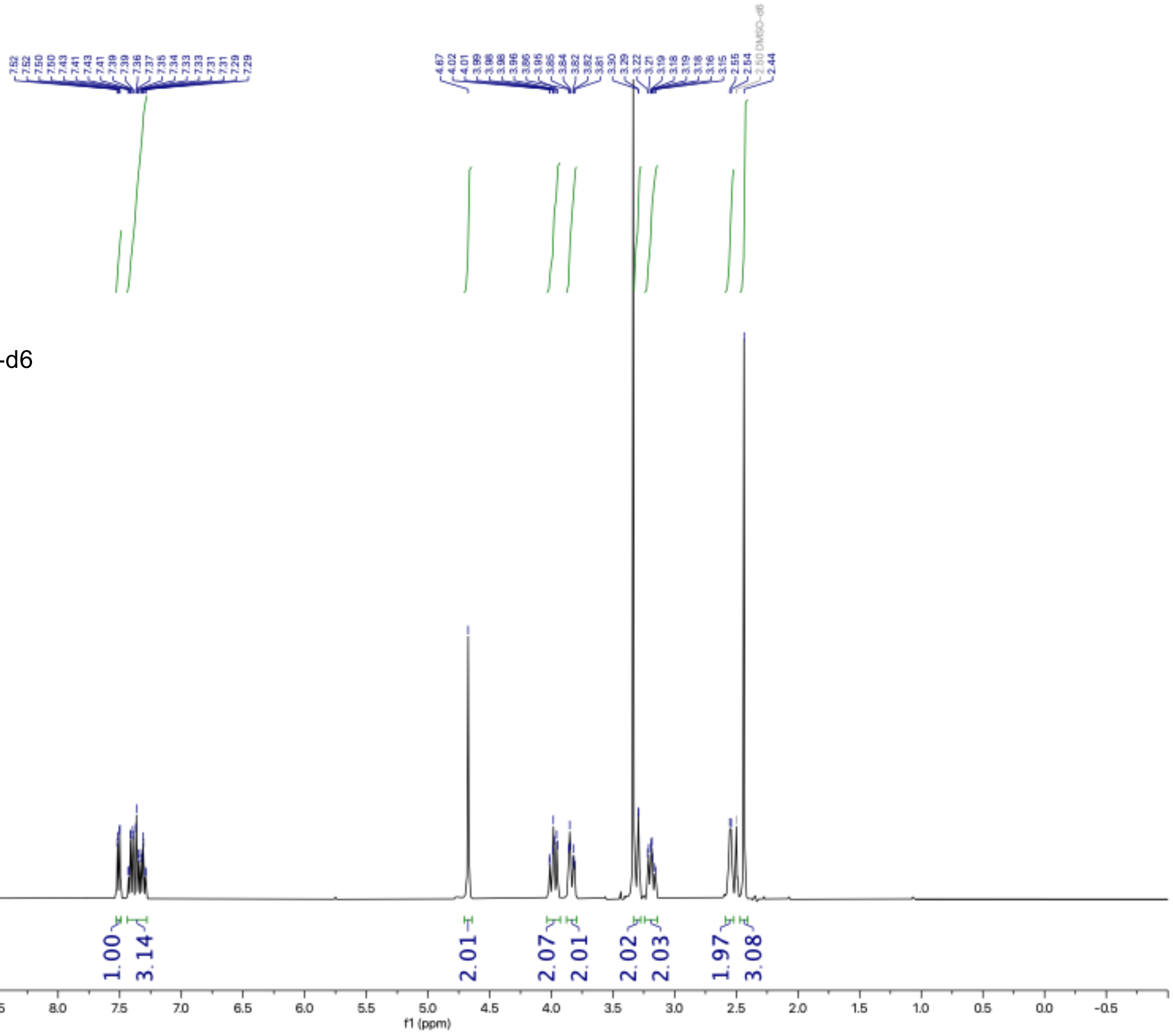

4ad

^{13}C NMR, 176 MHz, DMSO-d6

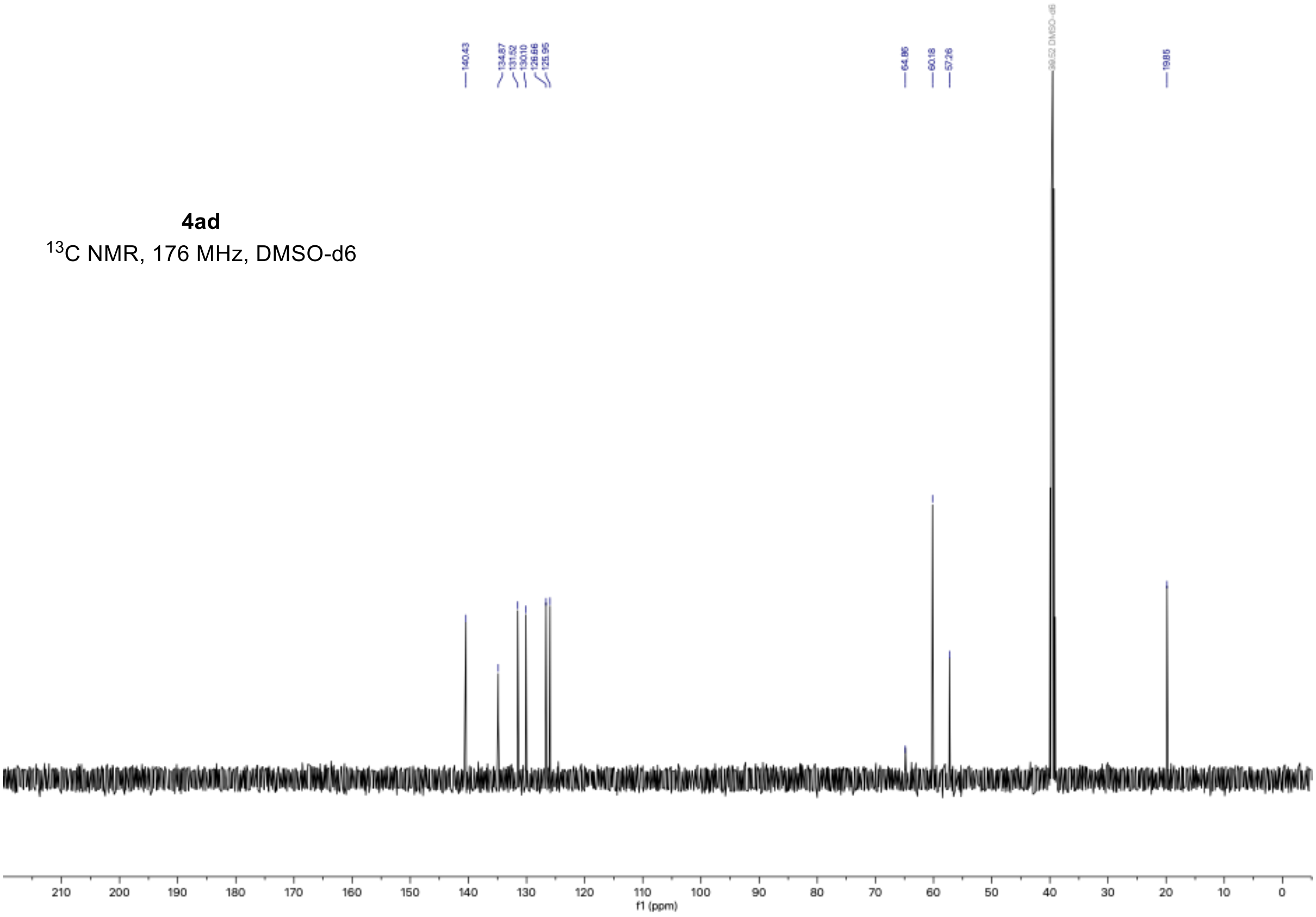

4ad
^{19}F NMR, 471 MHz, DMSO-d6

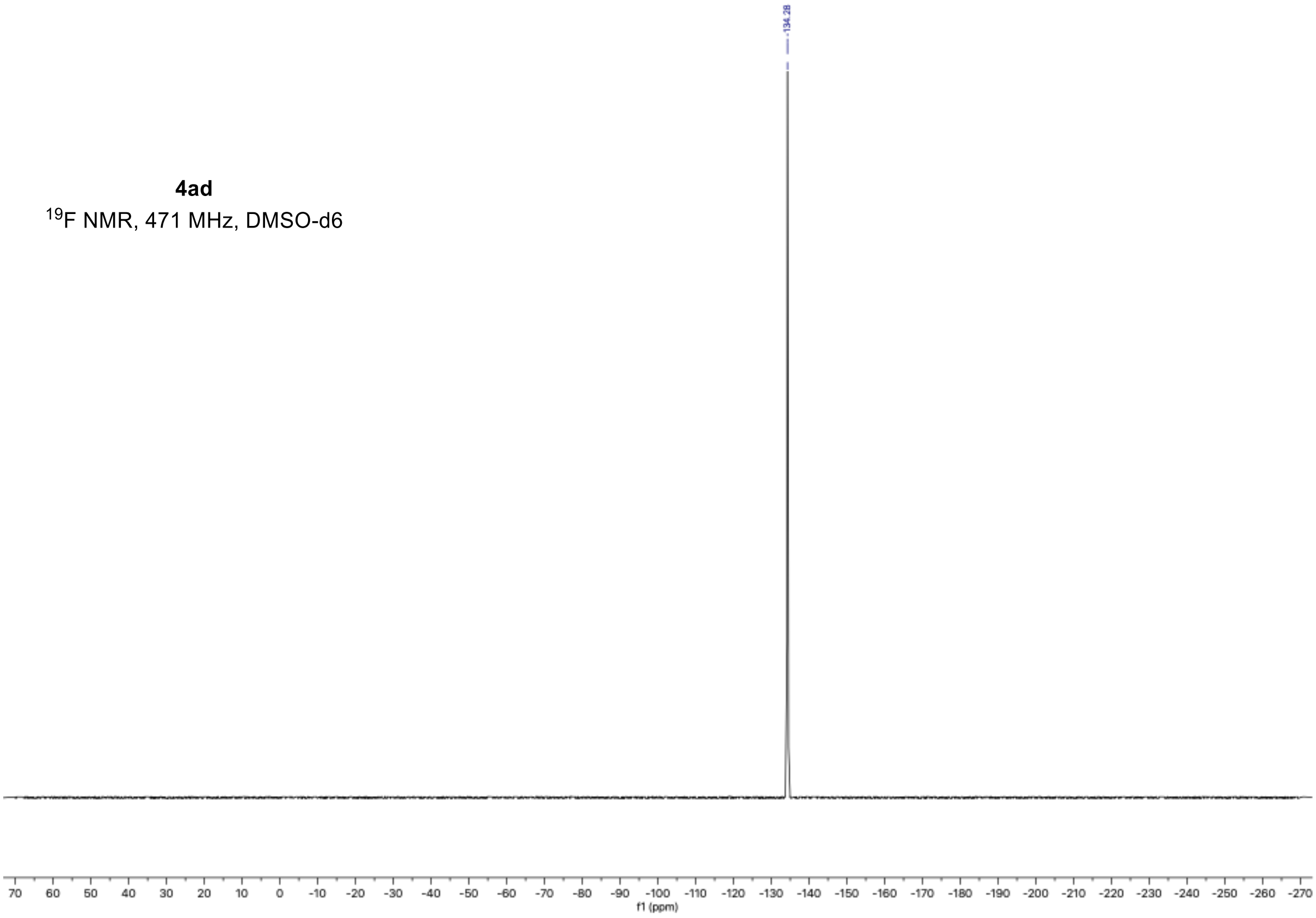

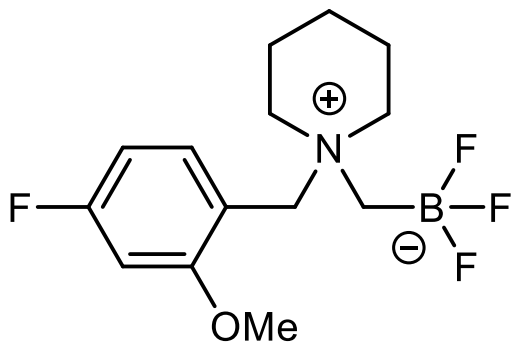

4ae

^{1}H NMR, 500 MHz, Acetone-d6

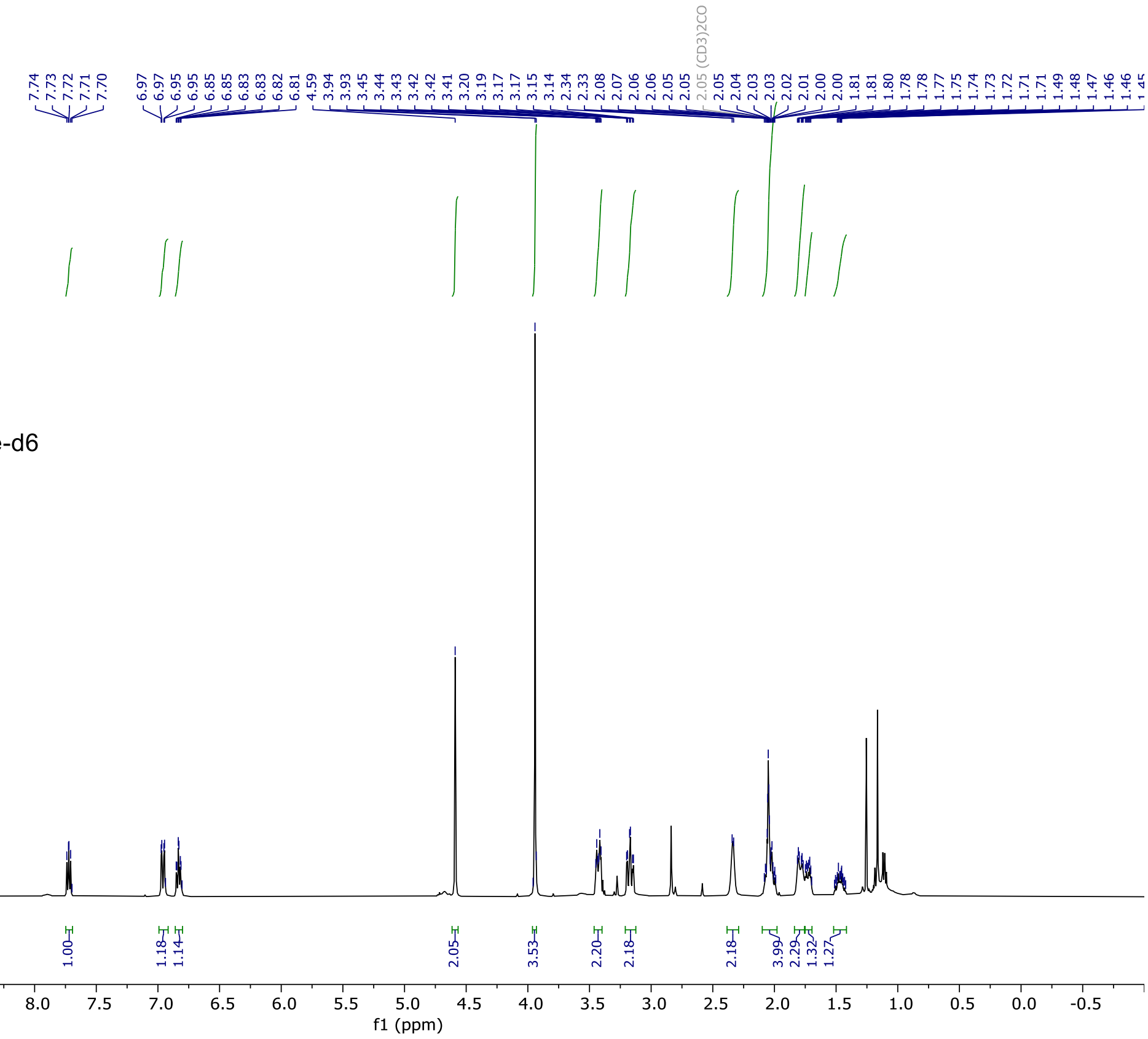

4ae

^{13}C NMR, 126 MHz, Acetone-d6

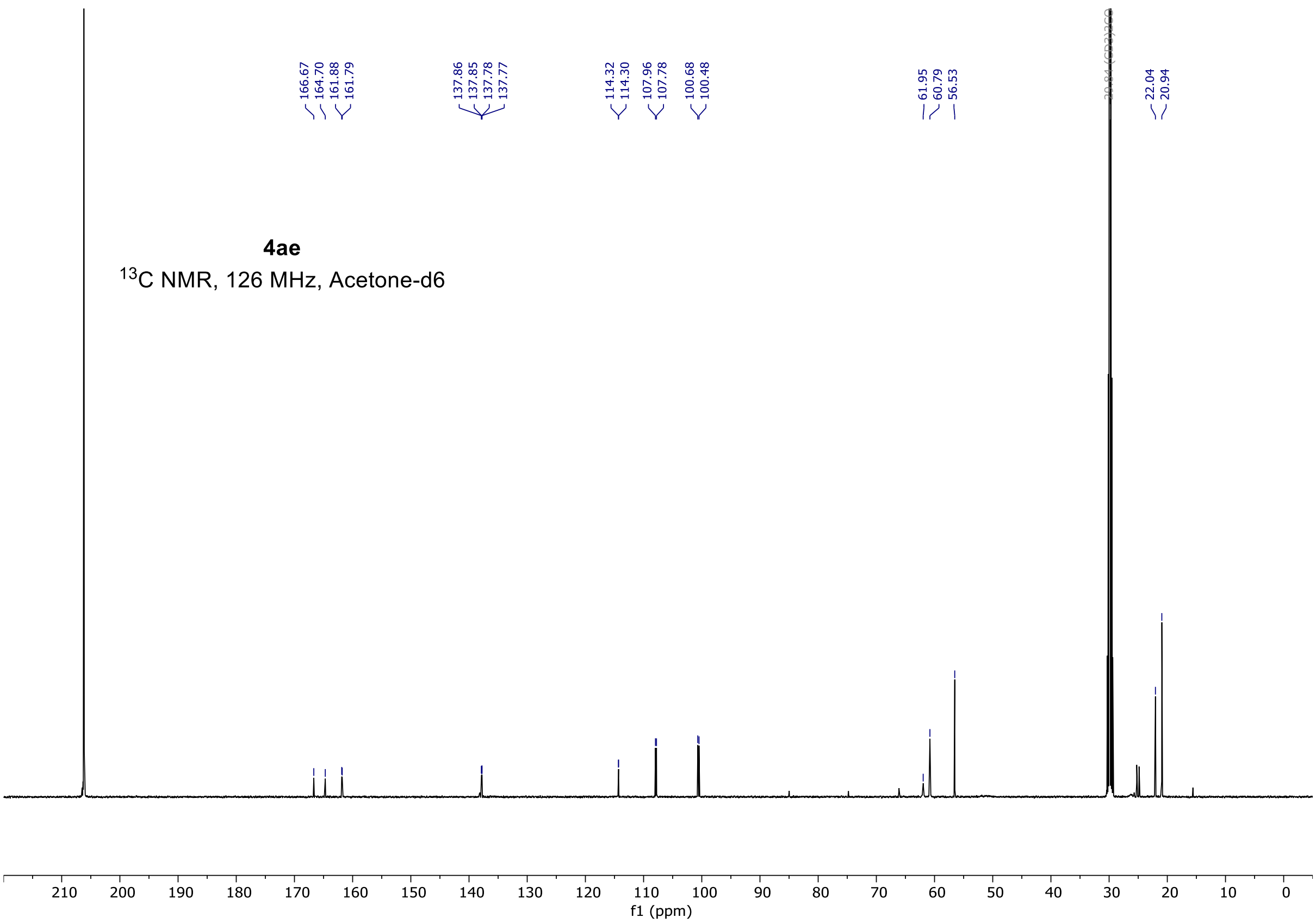

4ae

^{19}F NMR, 376 MHz, Acetone-d6

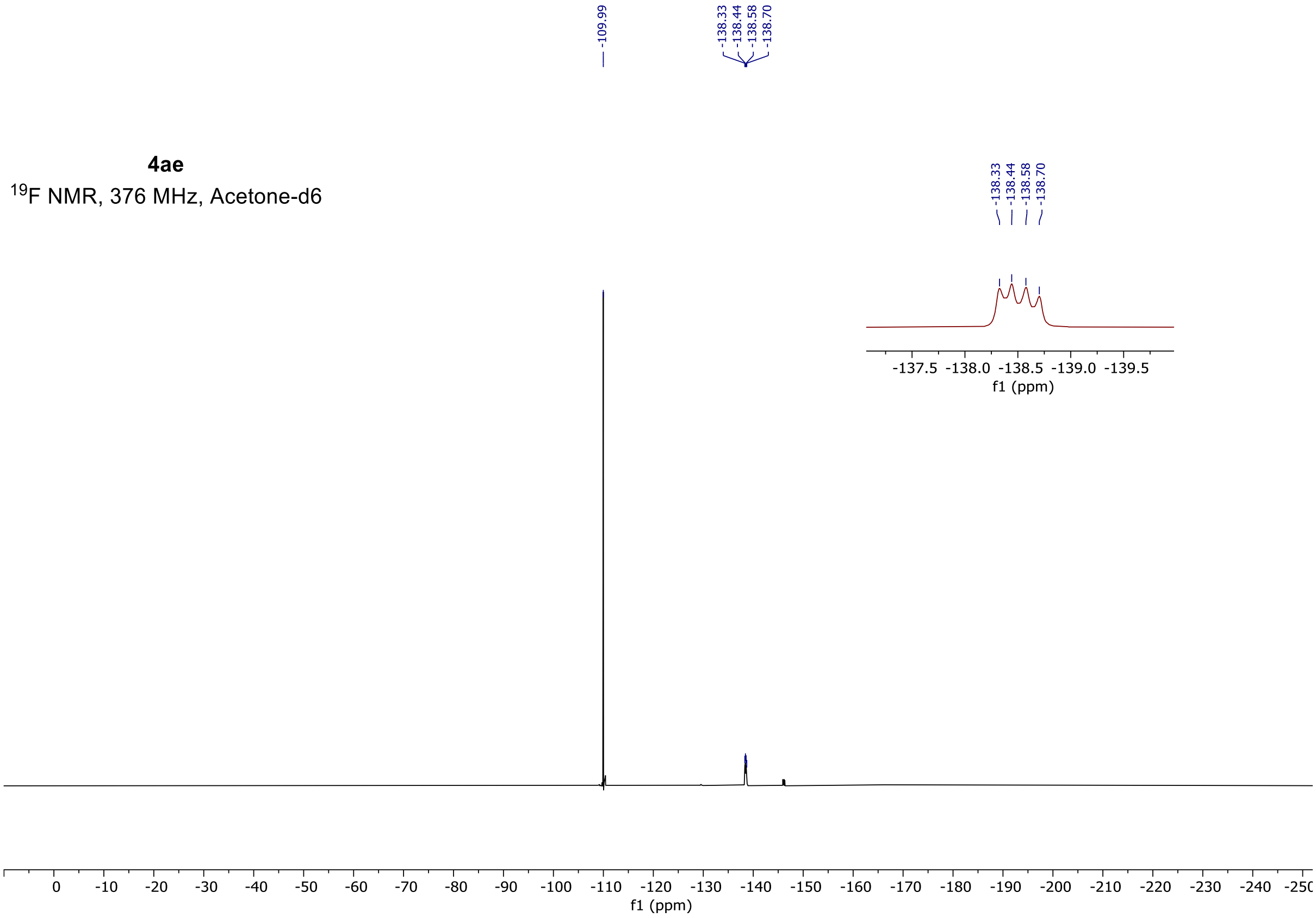

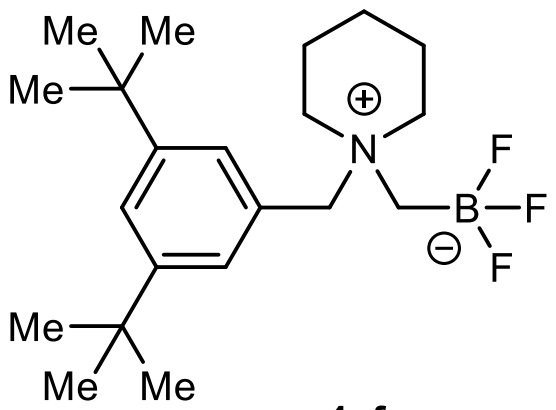

4af

^{1}H NMR, 700 MHz, DMSO-d6

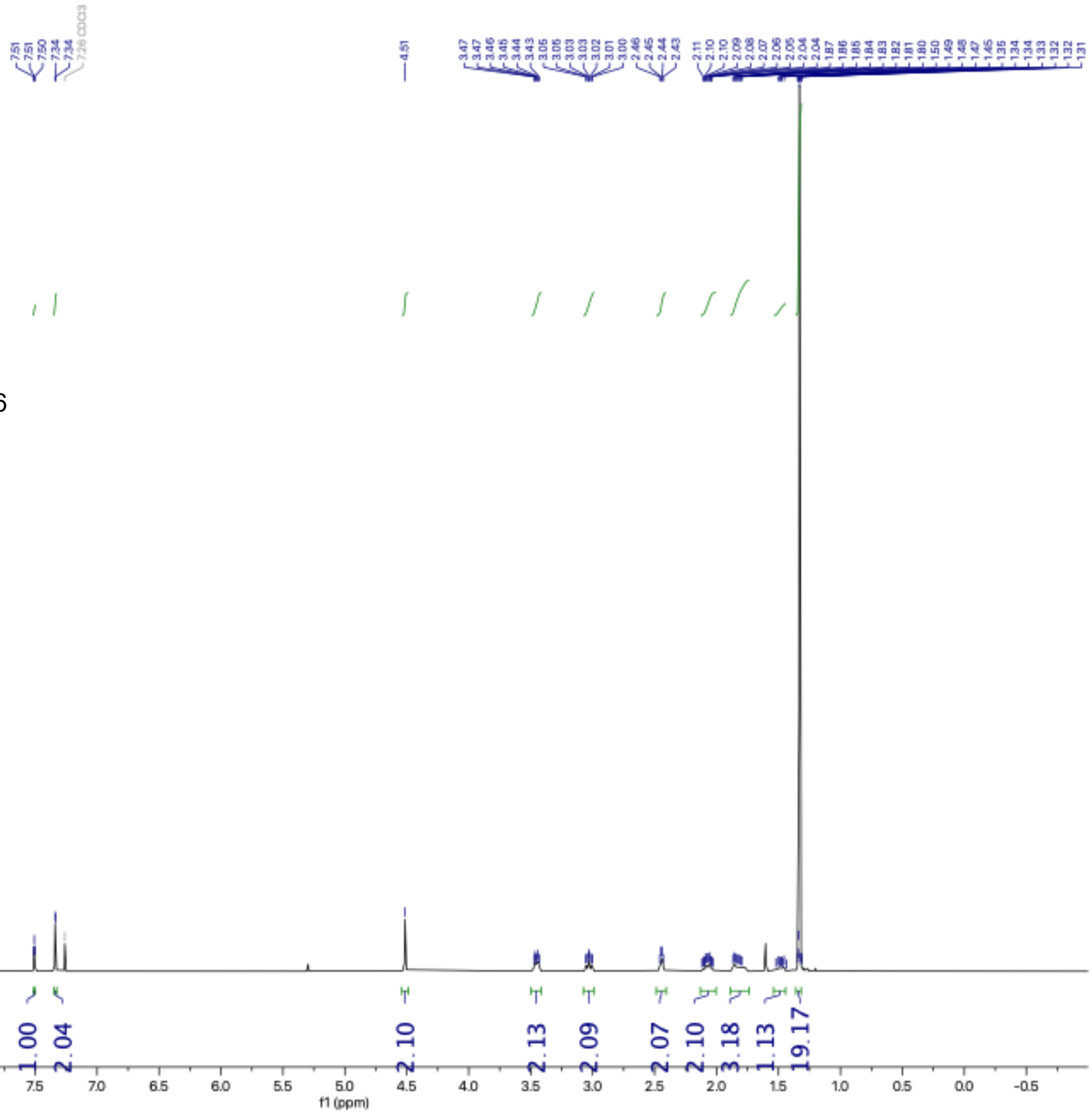

4af

^{13}C NMR, 176 MHz, DMSO-d6

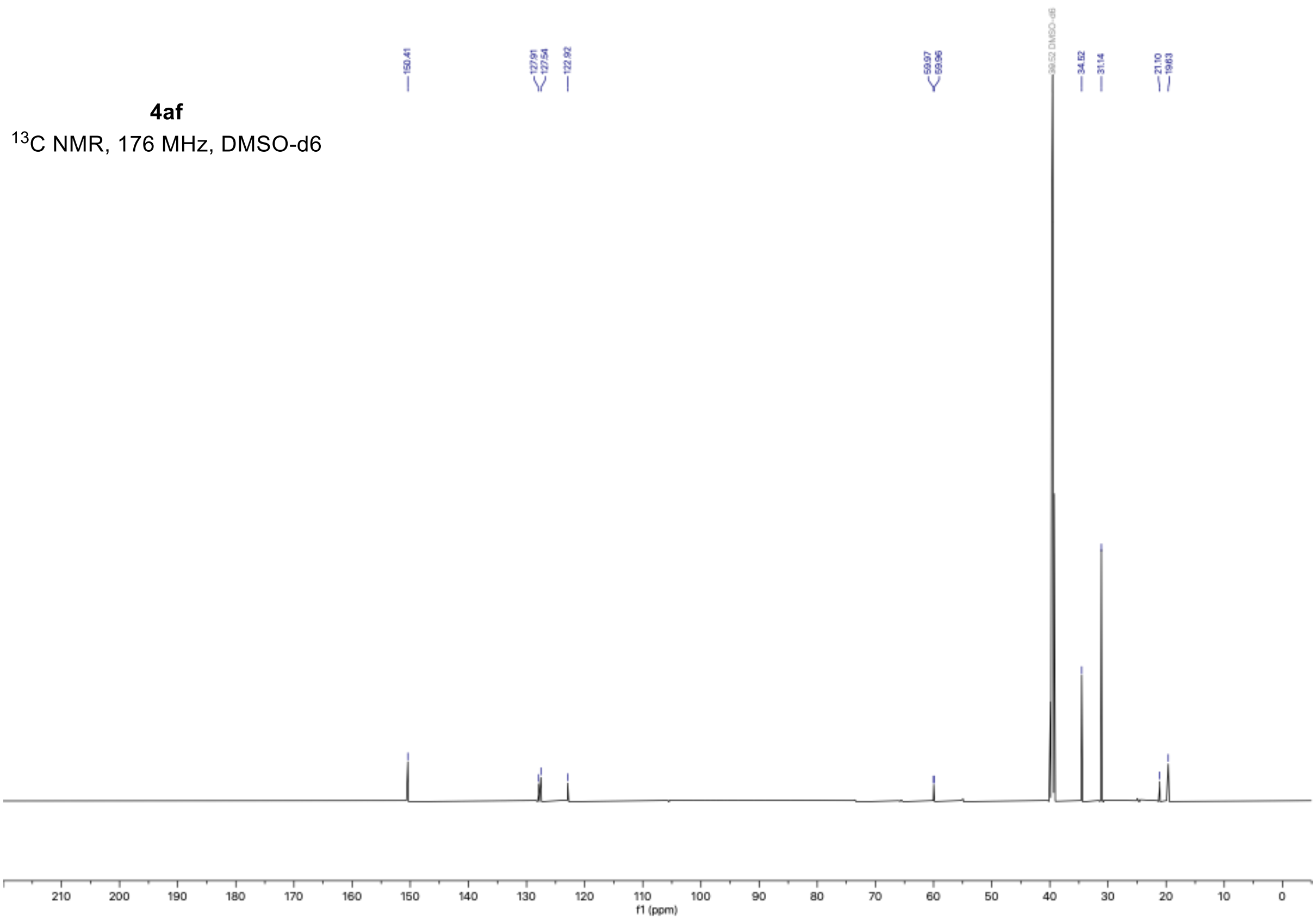

4af

^{19}F NMR, 471 MHz, DMSO-d6

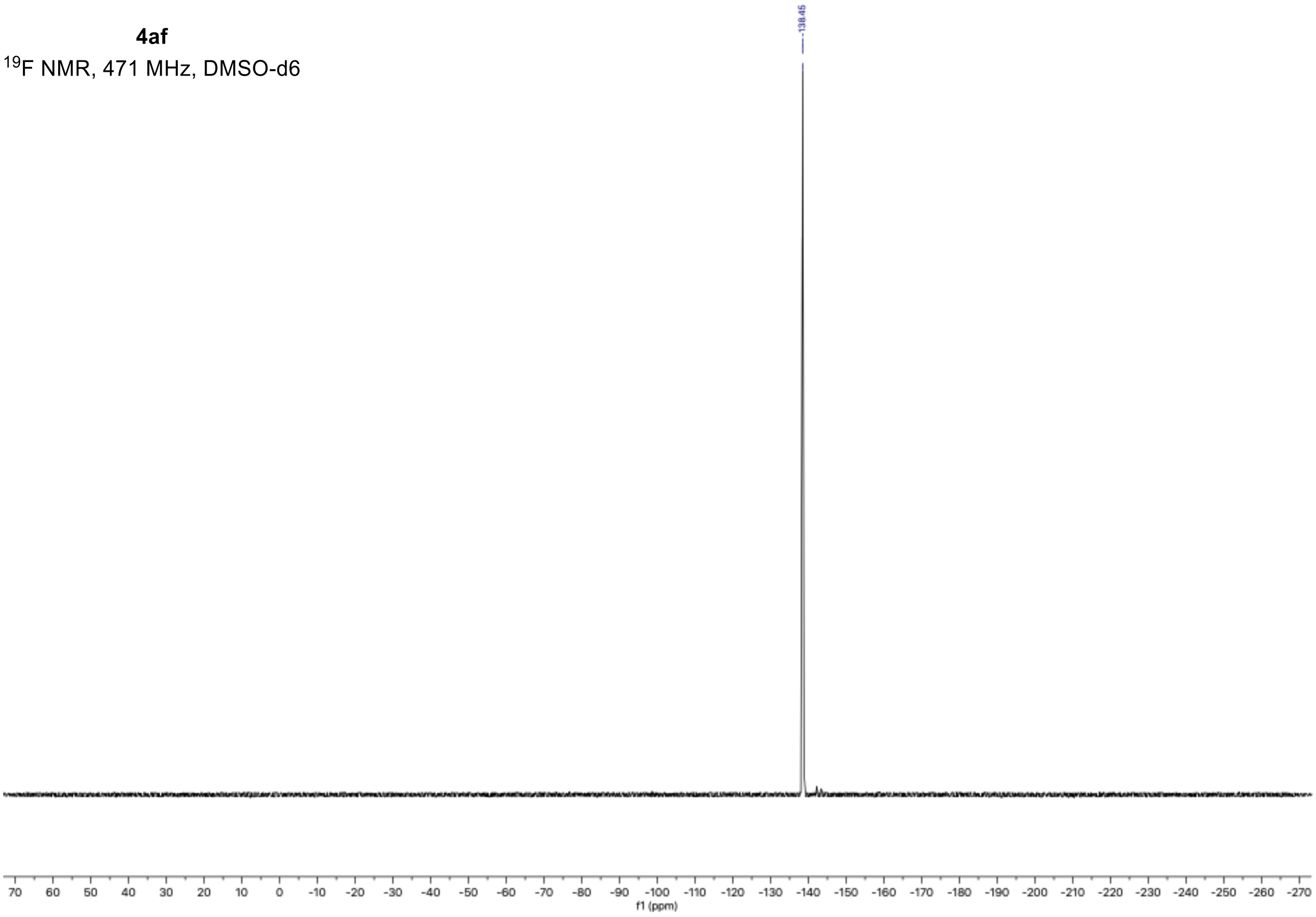

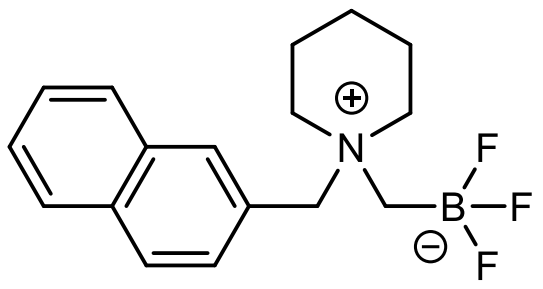

4ag

^{1}H NMR, 700 MHz, DMSO-d6

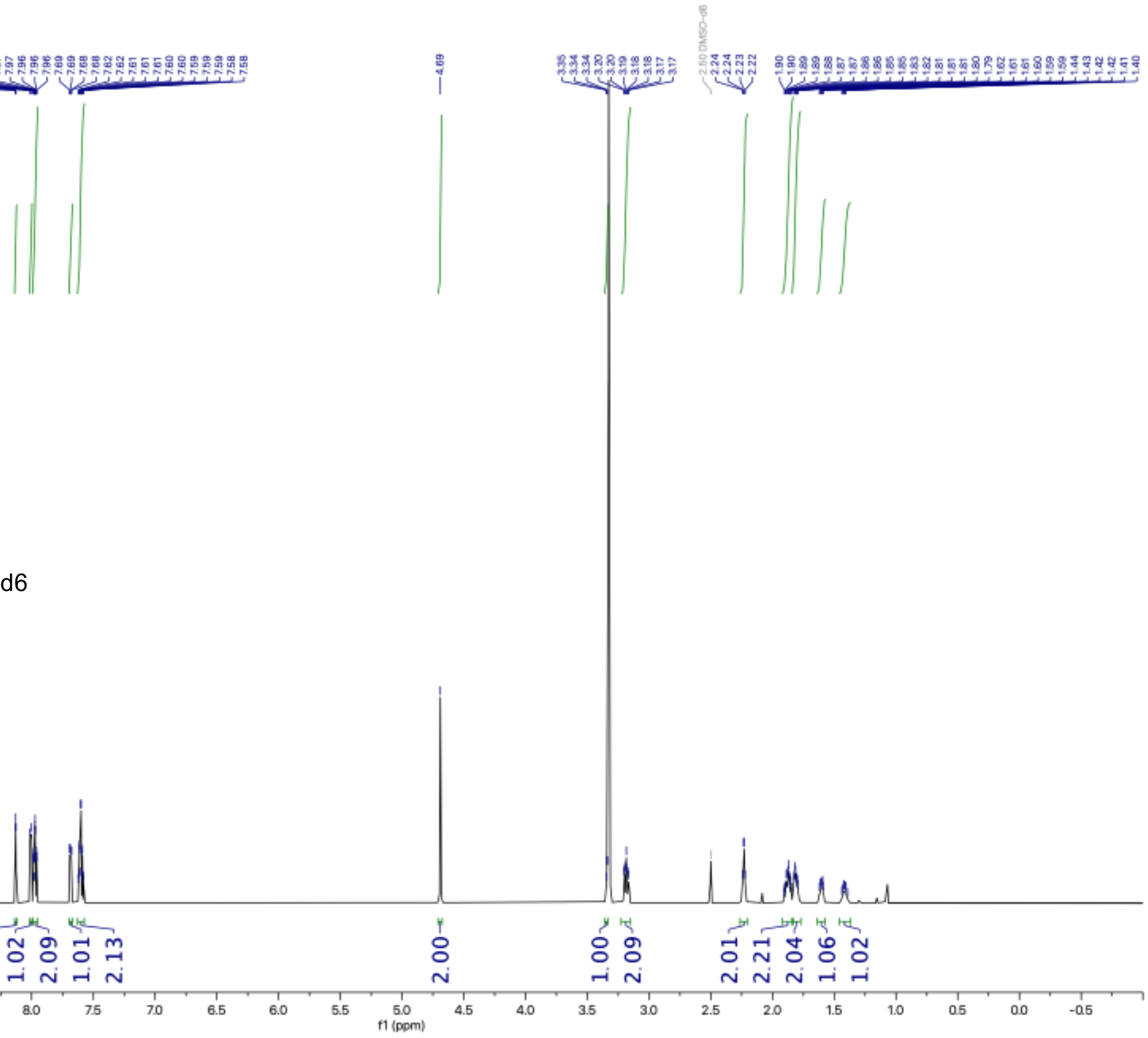

4ag

^{13}C NMR, 176 MHz, DMSO-d6

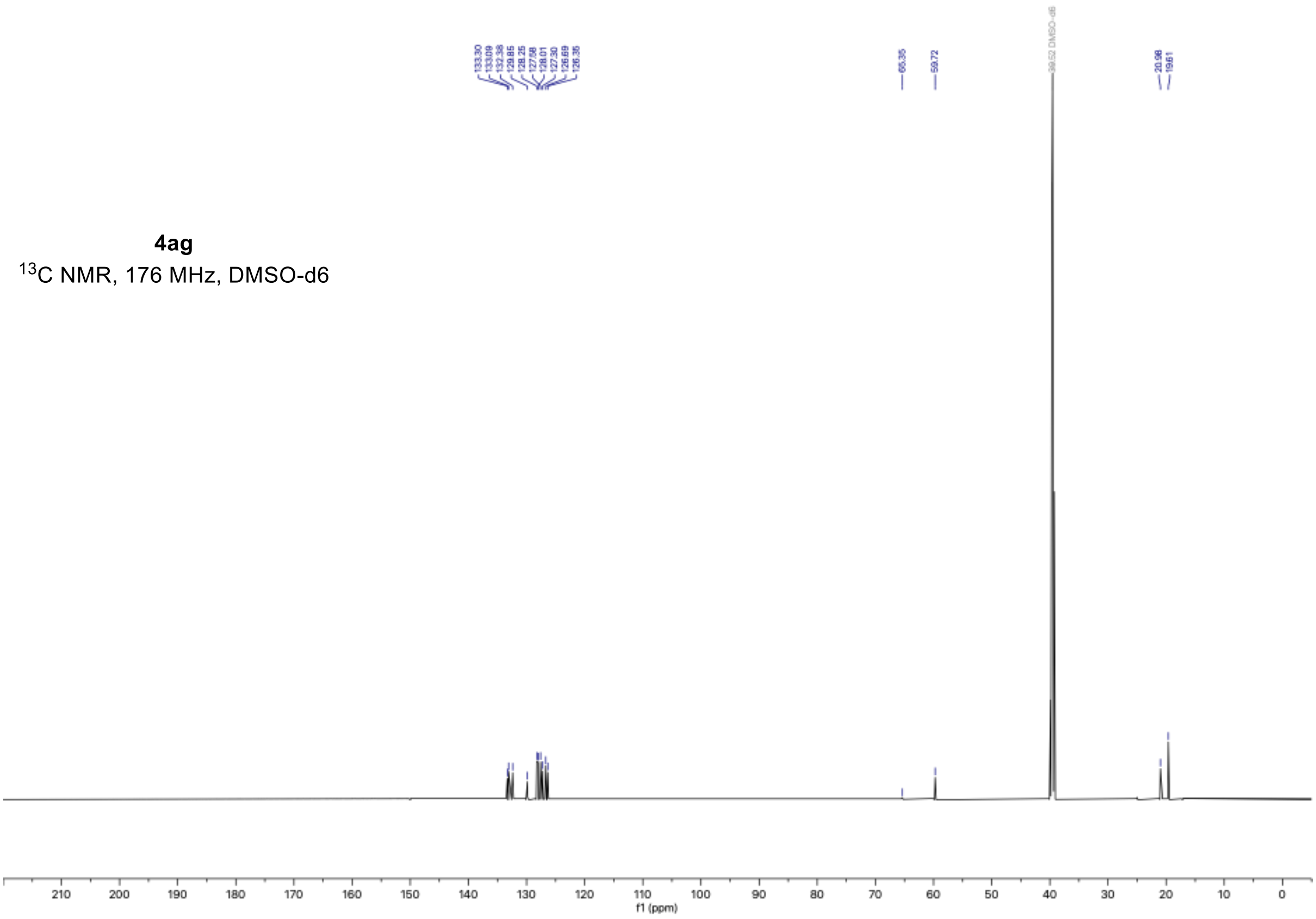

4ag

^{19}F NMR, 471 MHz, DMSO-d6

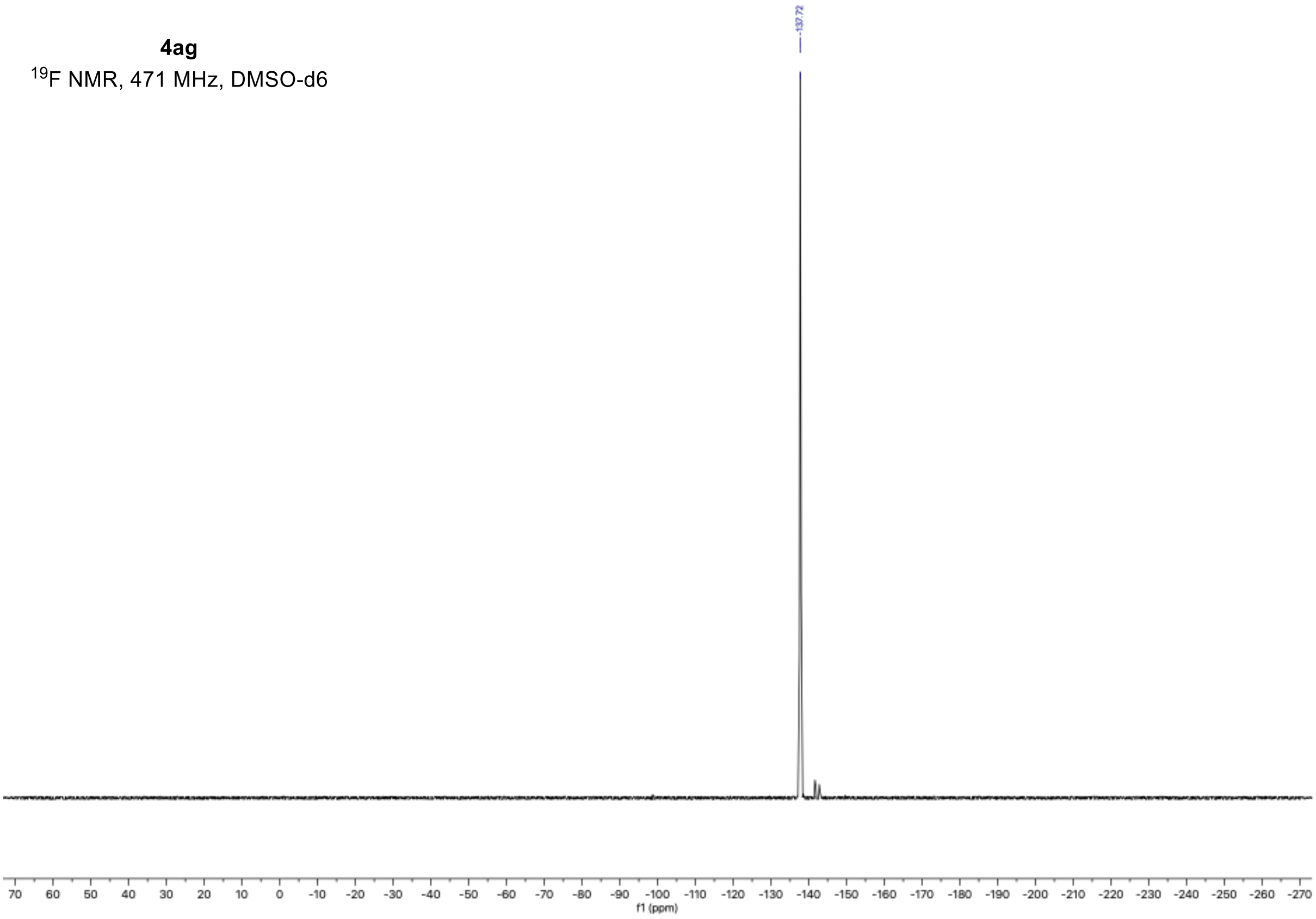

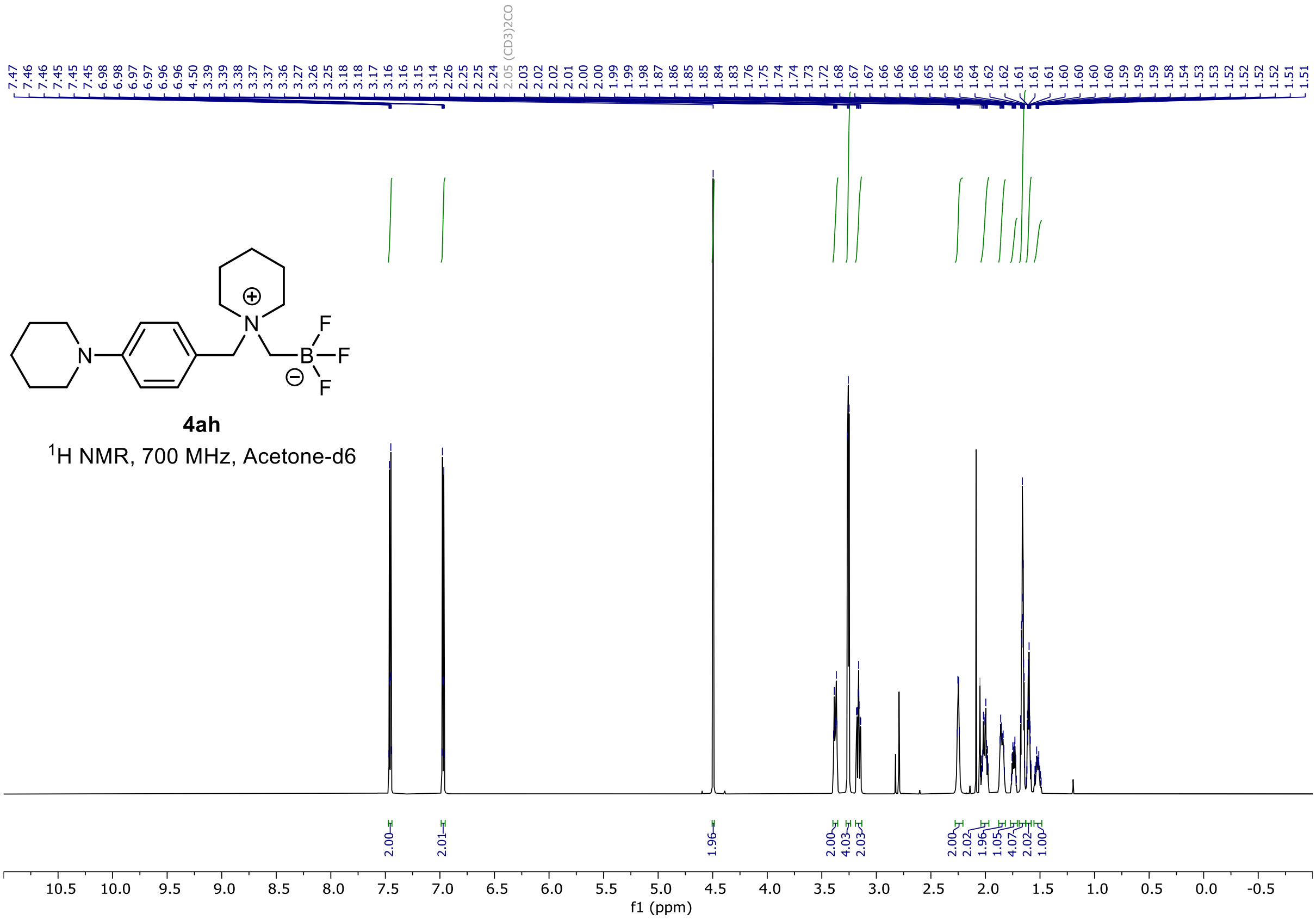

4ah
1H NMR, 700 MHz, Acetone-d6
f1 (ppm)

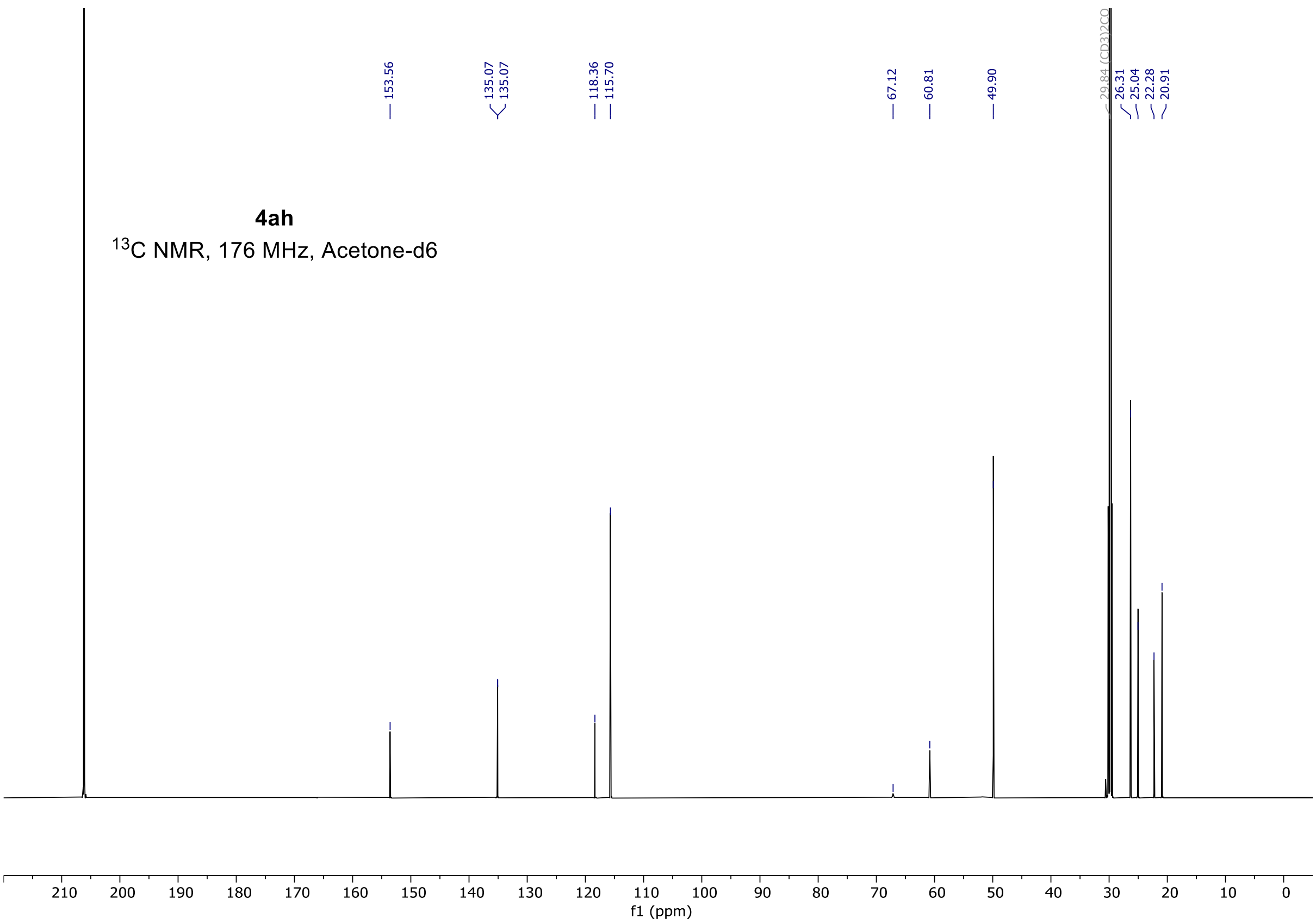

4ah
13C NMR, 176 MHz, Acetone-d6
153.56
135.07
135.07
118.36
115.70
67.12
60.81
49.90
29.84 (CD3)2CO
26.31
25.04
22.28
20.91
f1 (ppm)

4ah

^{19}F NMR, 376 MHz, Acetone-d6

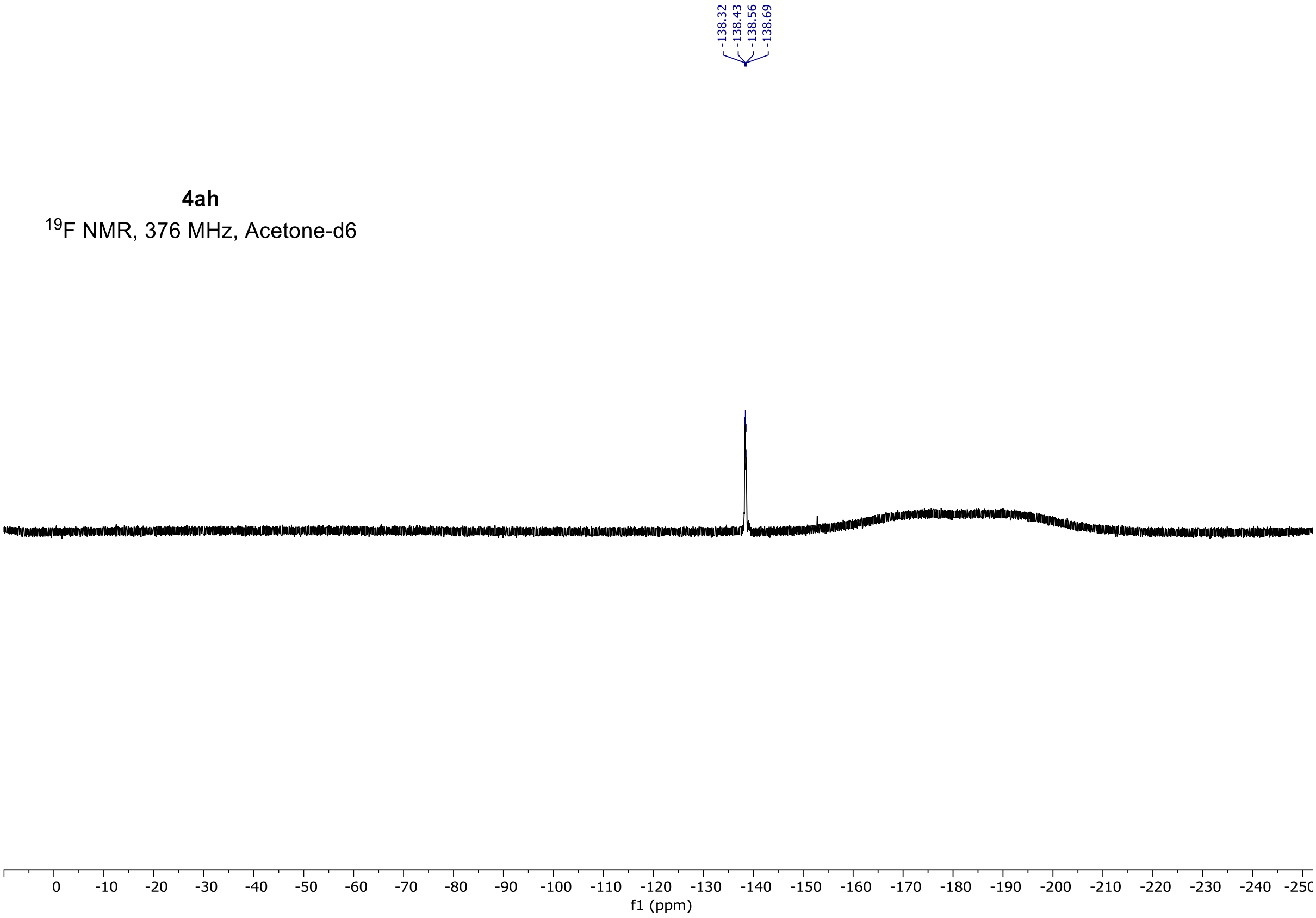

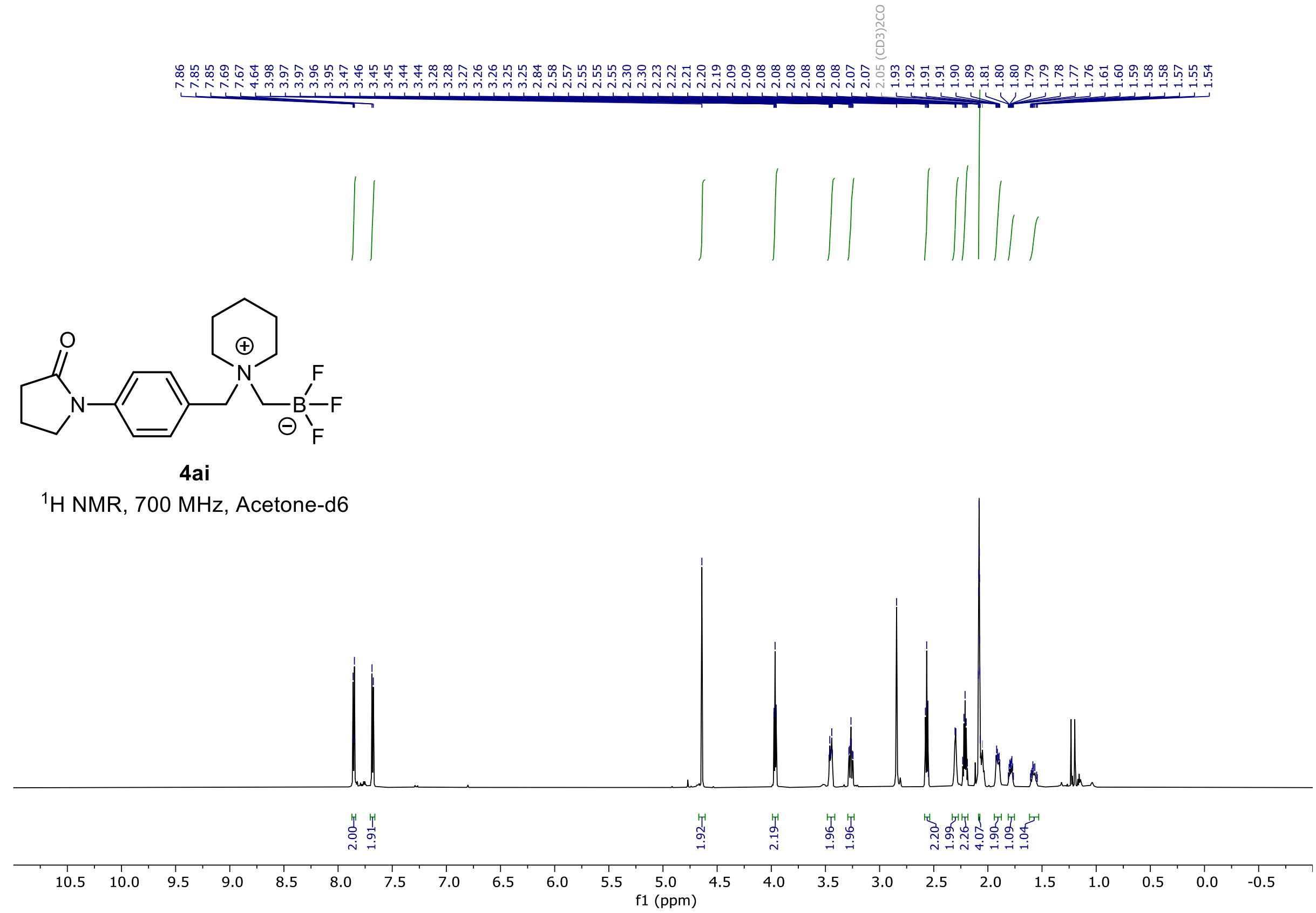

4ai

^{1}H NMR, 700 MHz, Acetone-d6

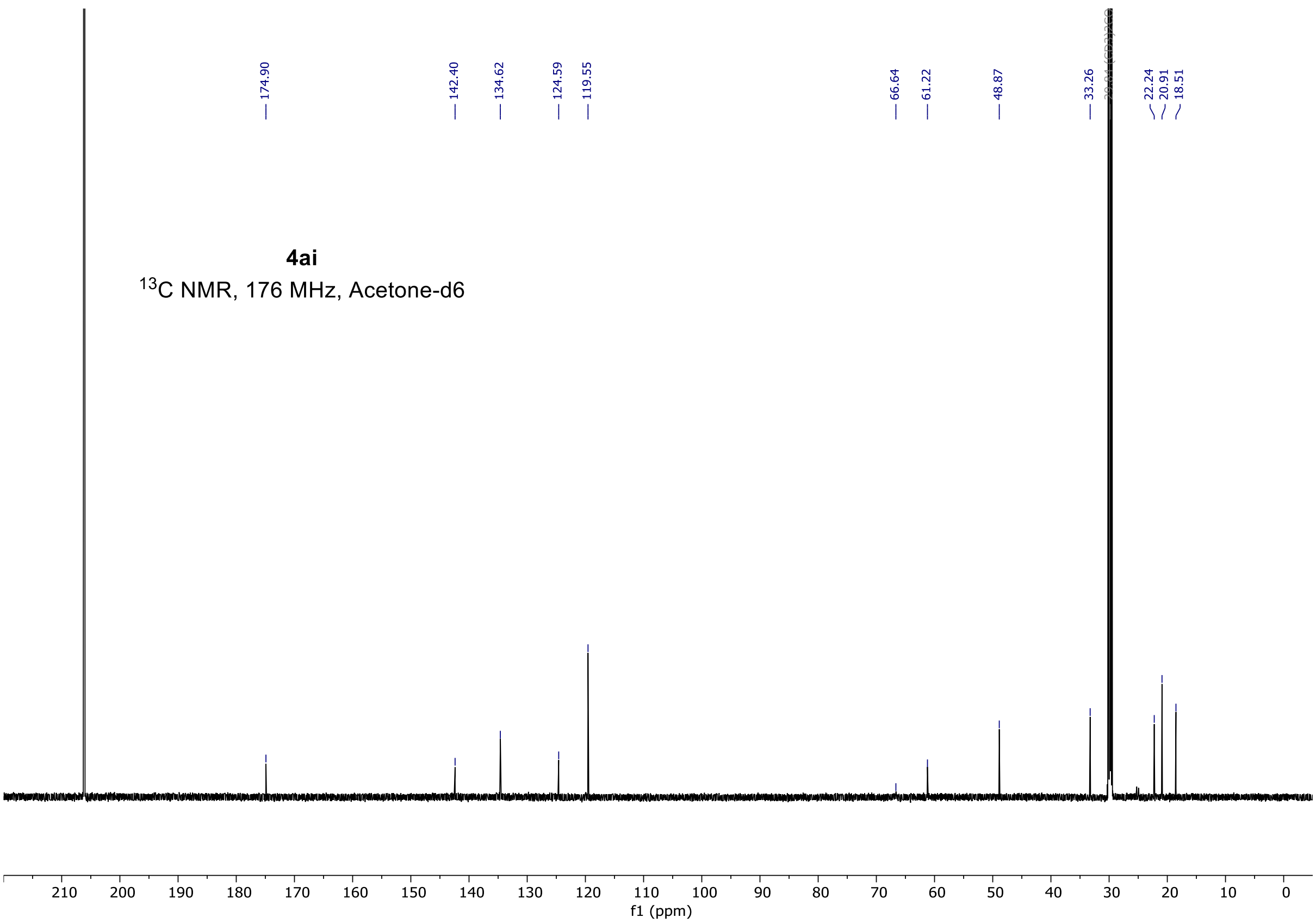

4ai

^{13}C NMR, 176 MHz, Acetone-d6

4ai
^{19}F NMR, 376 MHz, Acetone-d6

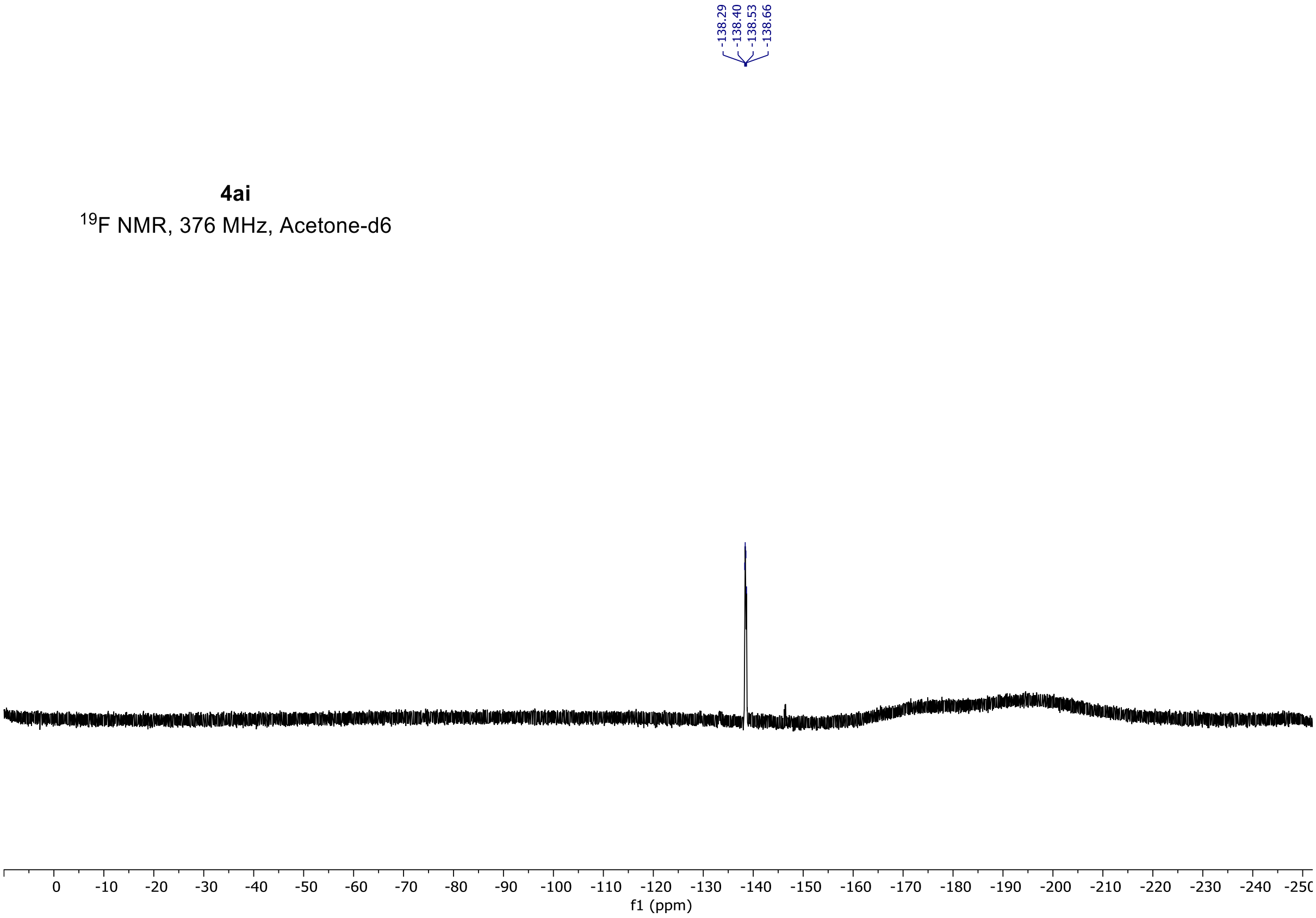

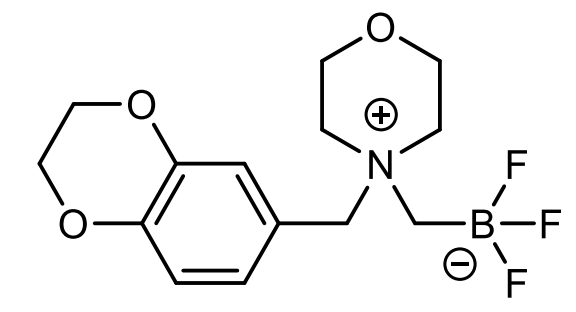

4aj

^{1}H NMR, 500 MHz, DMSO-d6

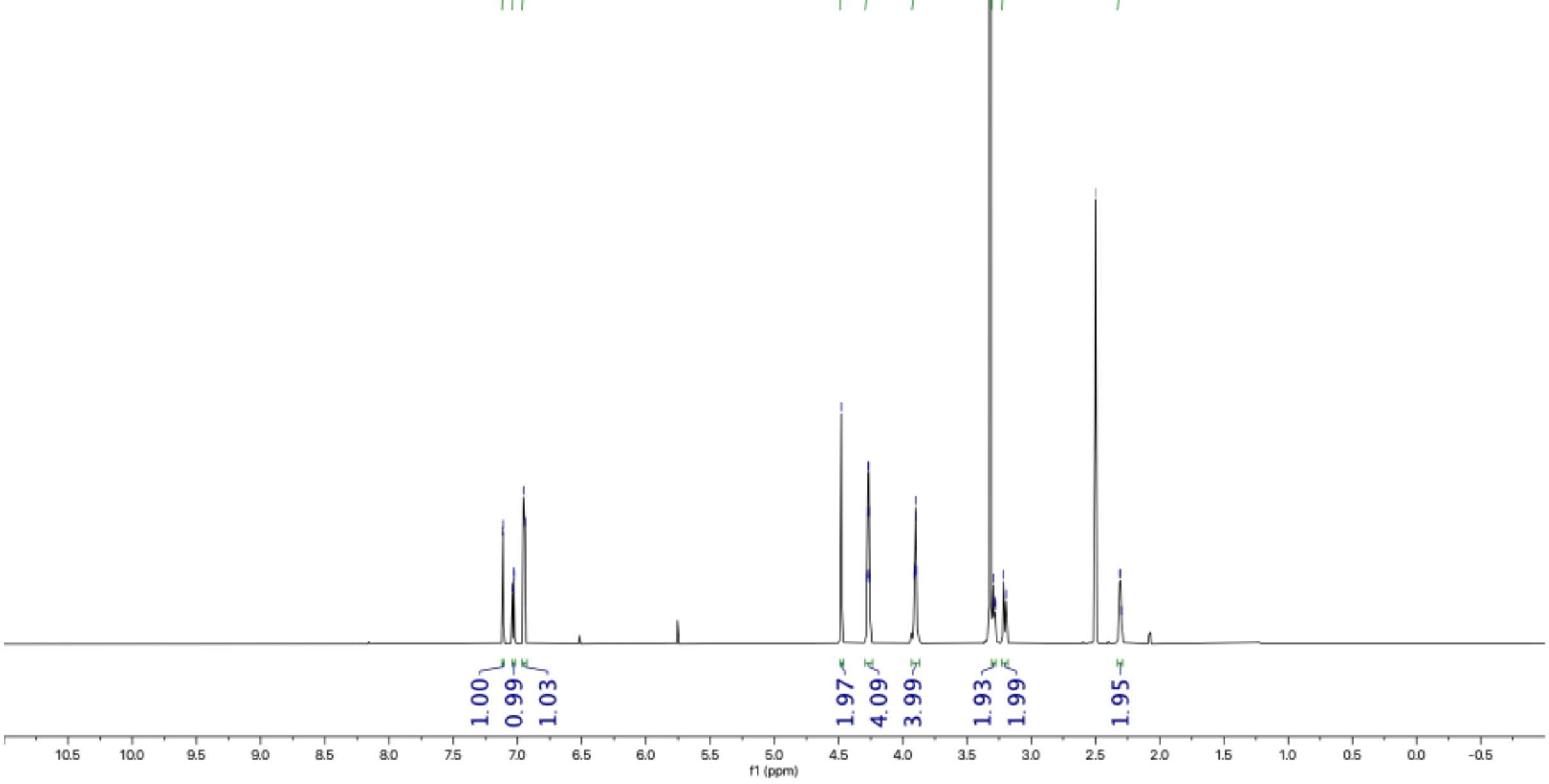

4aj

^{13}C NMR, 176 MHz, DMSO-d6

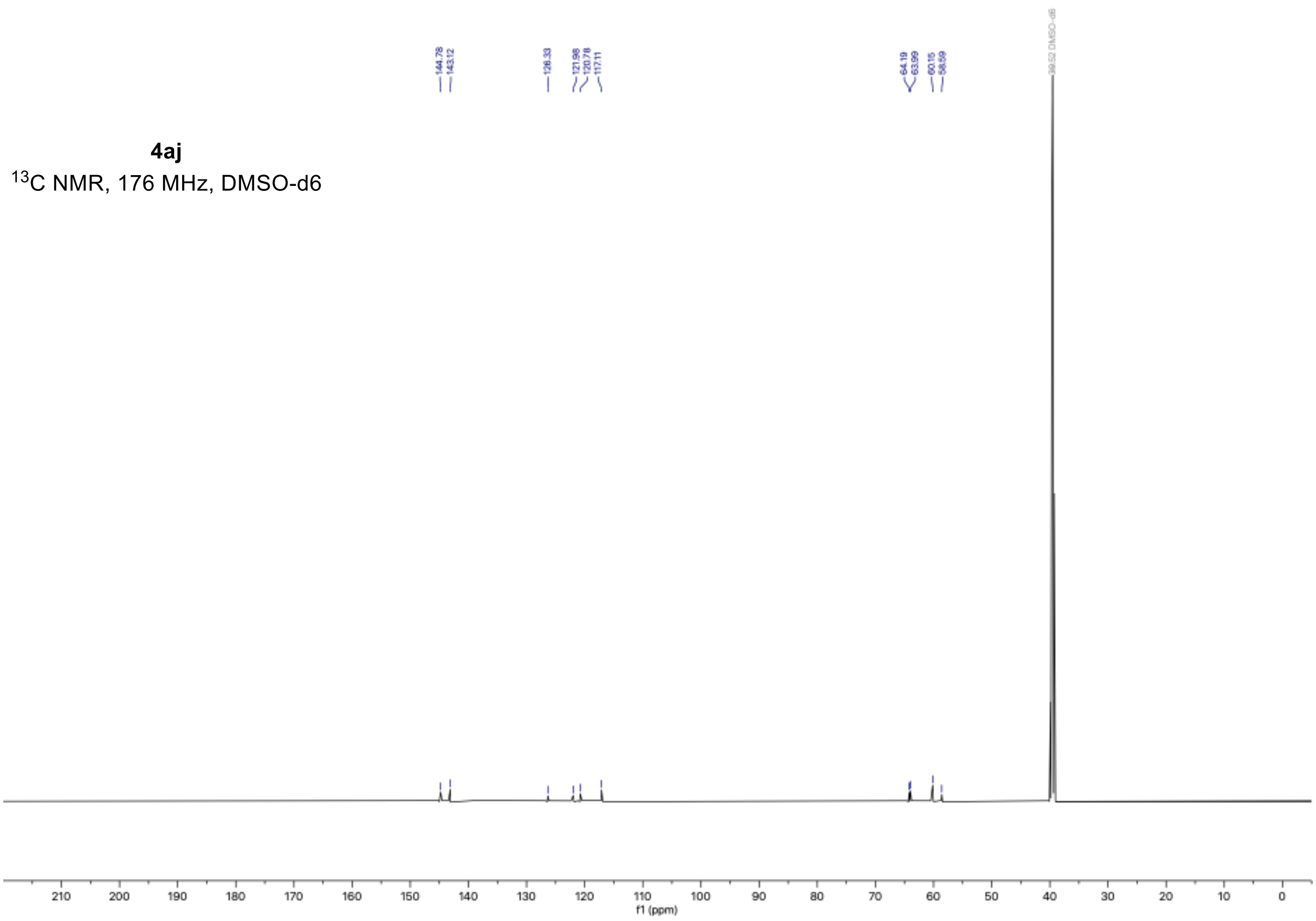

4aj

^{19}F NMR, 471 MHz, DMSO-d6

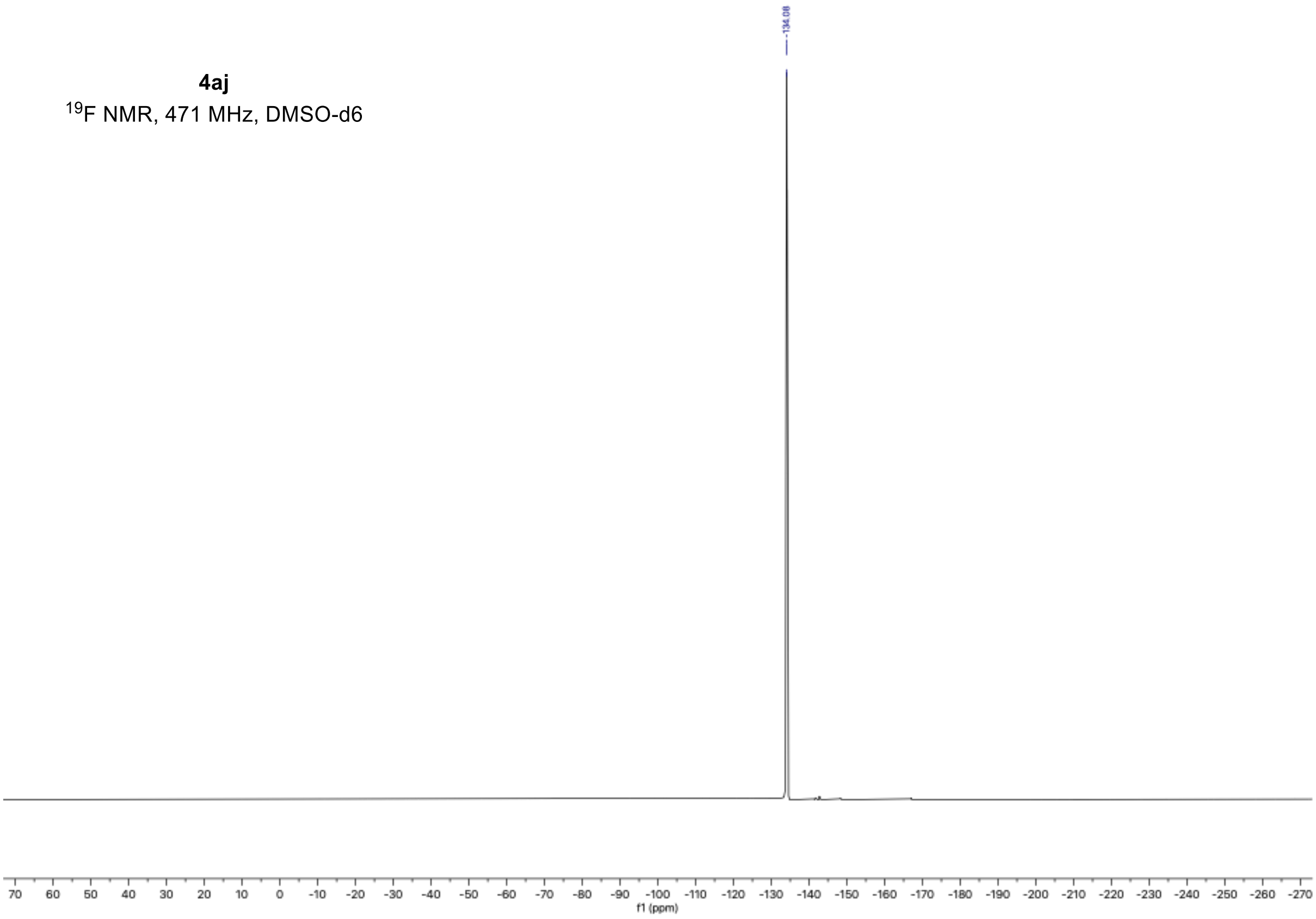

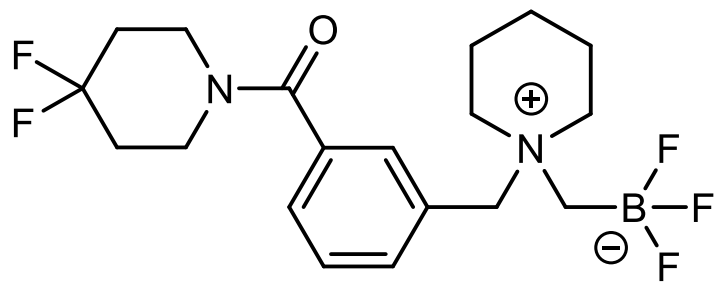

4ak

^{1}H NMR, 400 MHz, CD_3CN

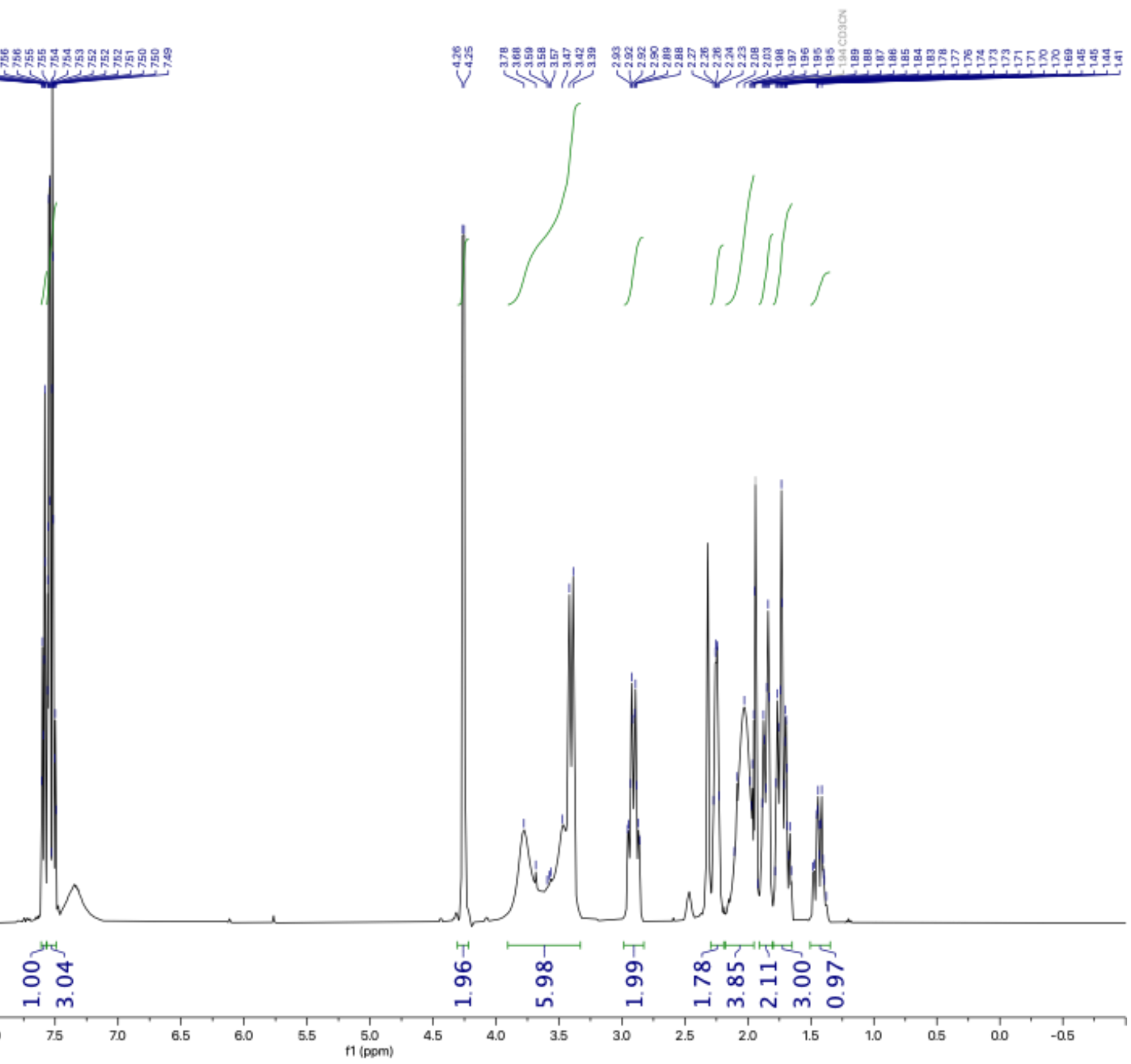

4ak

^{13}C NMR, 176 MHz, CD_3CN

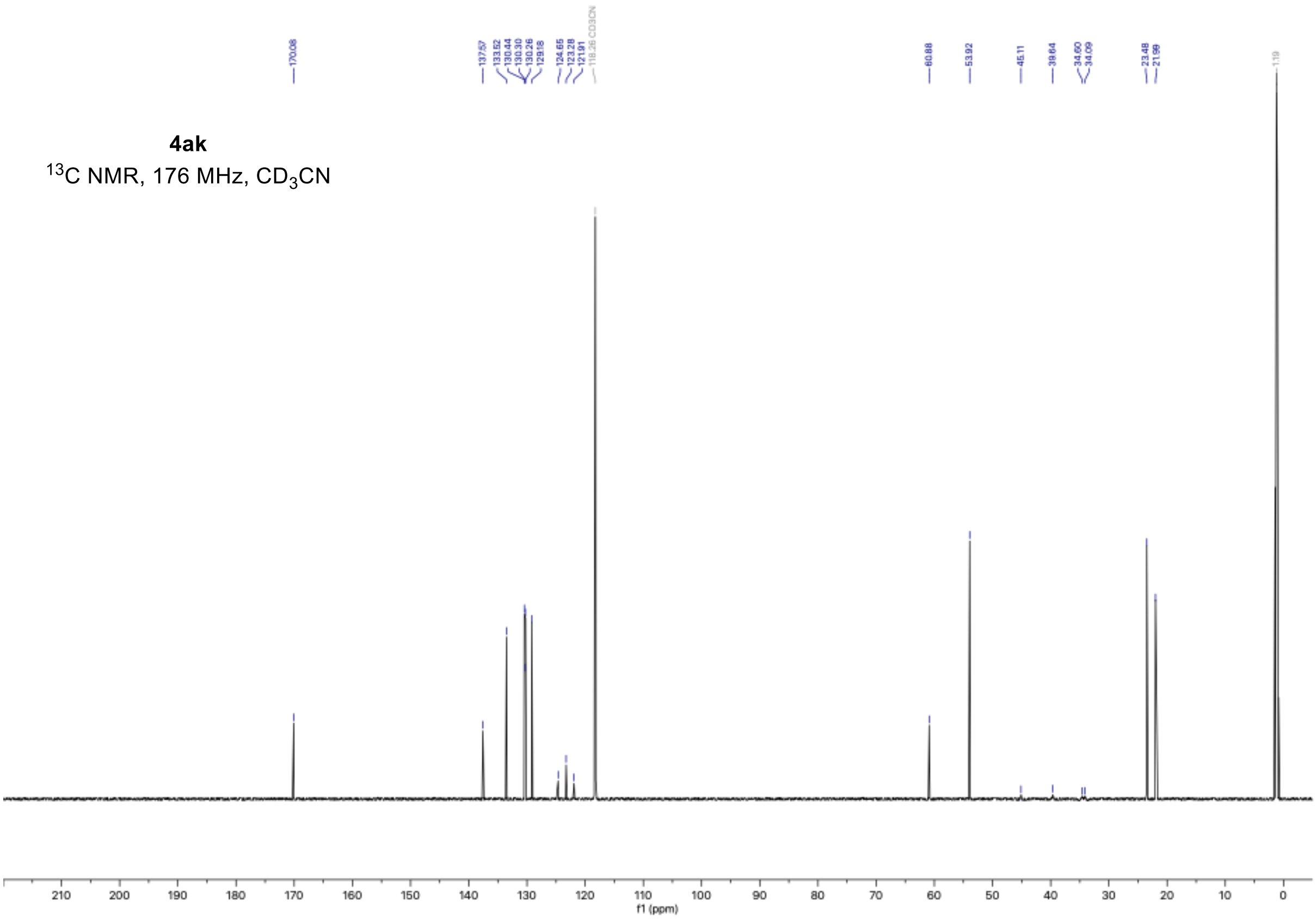

4ak

^{19}F NMR, 376 MHz, CD_3CN

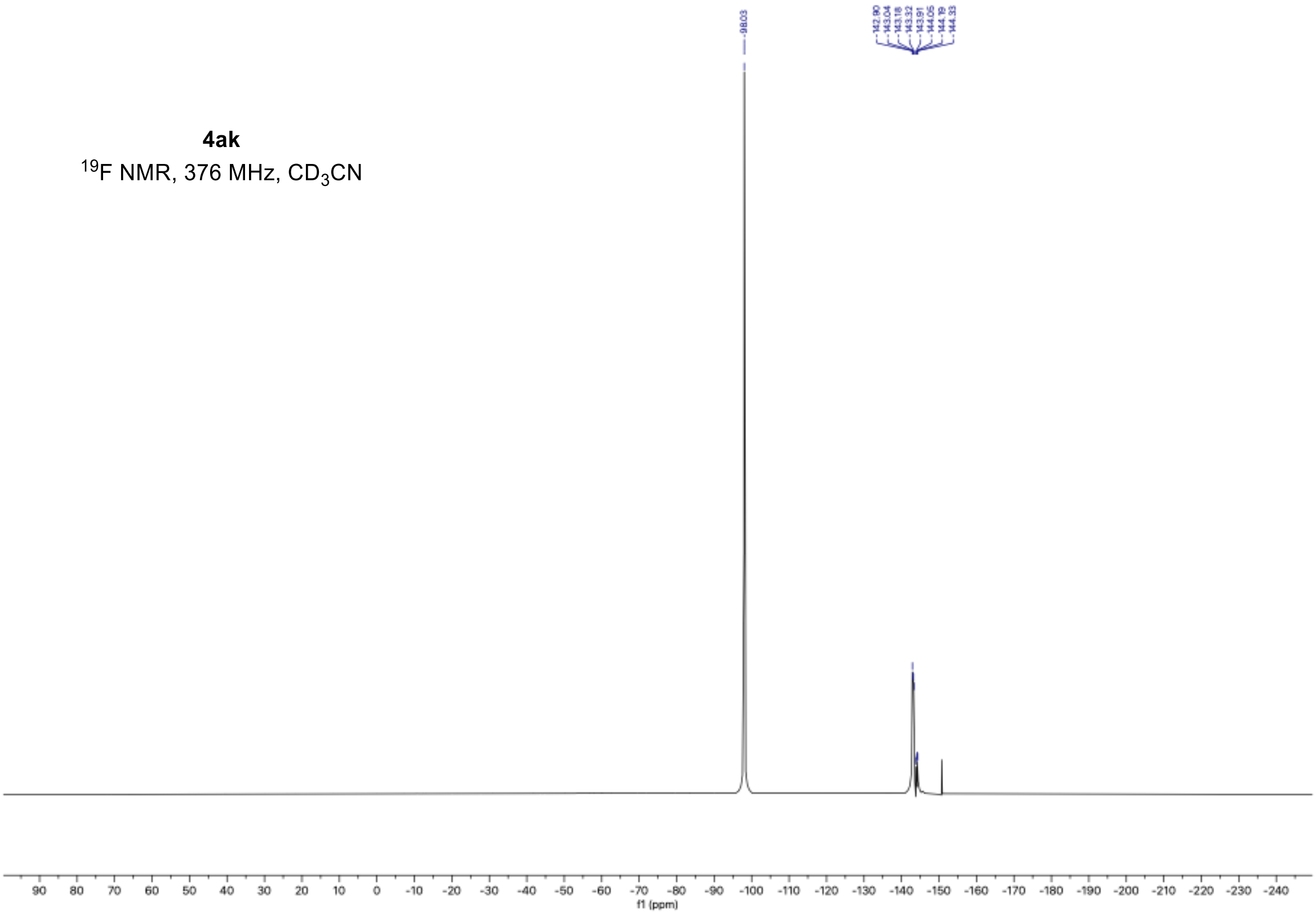

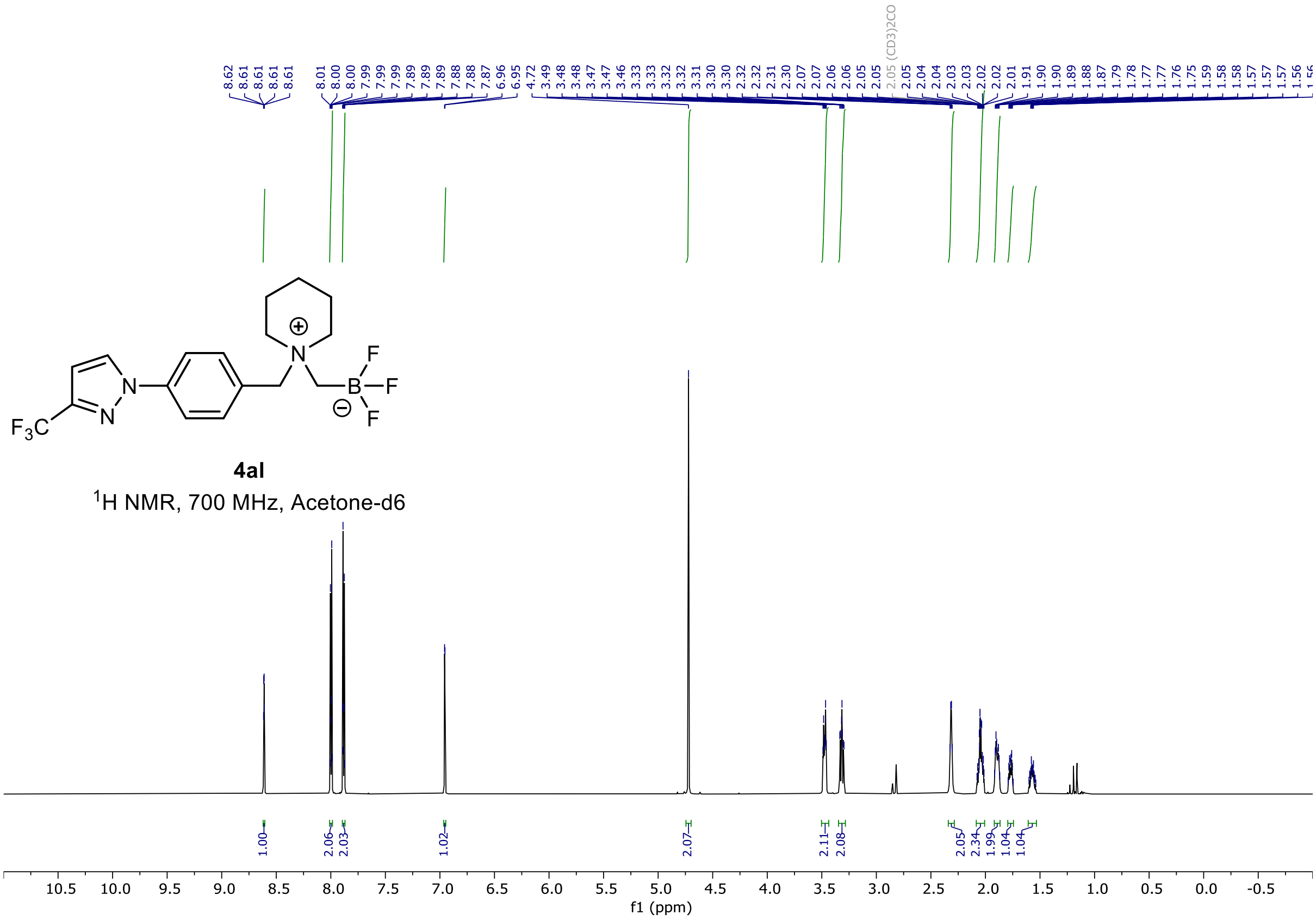

4al
^{1}H NMR, 700 MHz, Acetone-d6
f1 (ppm)

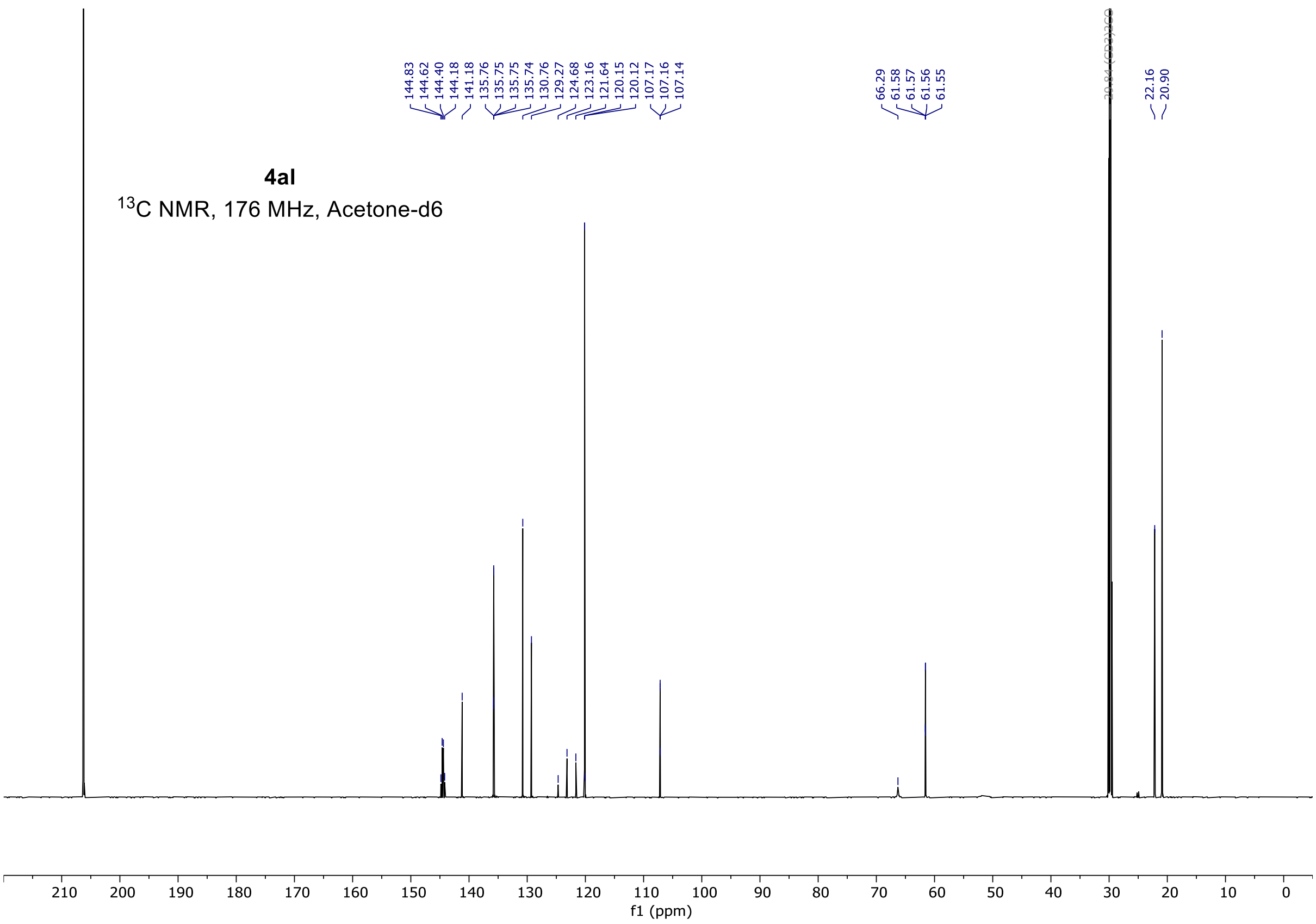

4al
^{13}C NMR, 176 MHz, Acetone-d6
f1 (ppm)

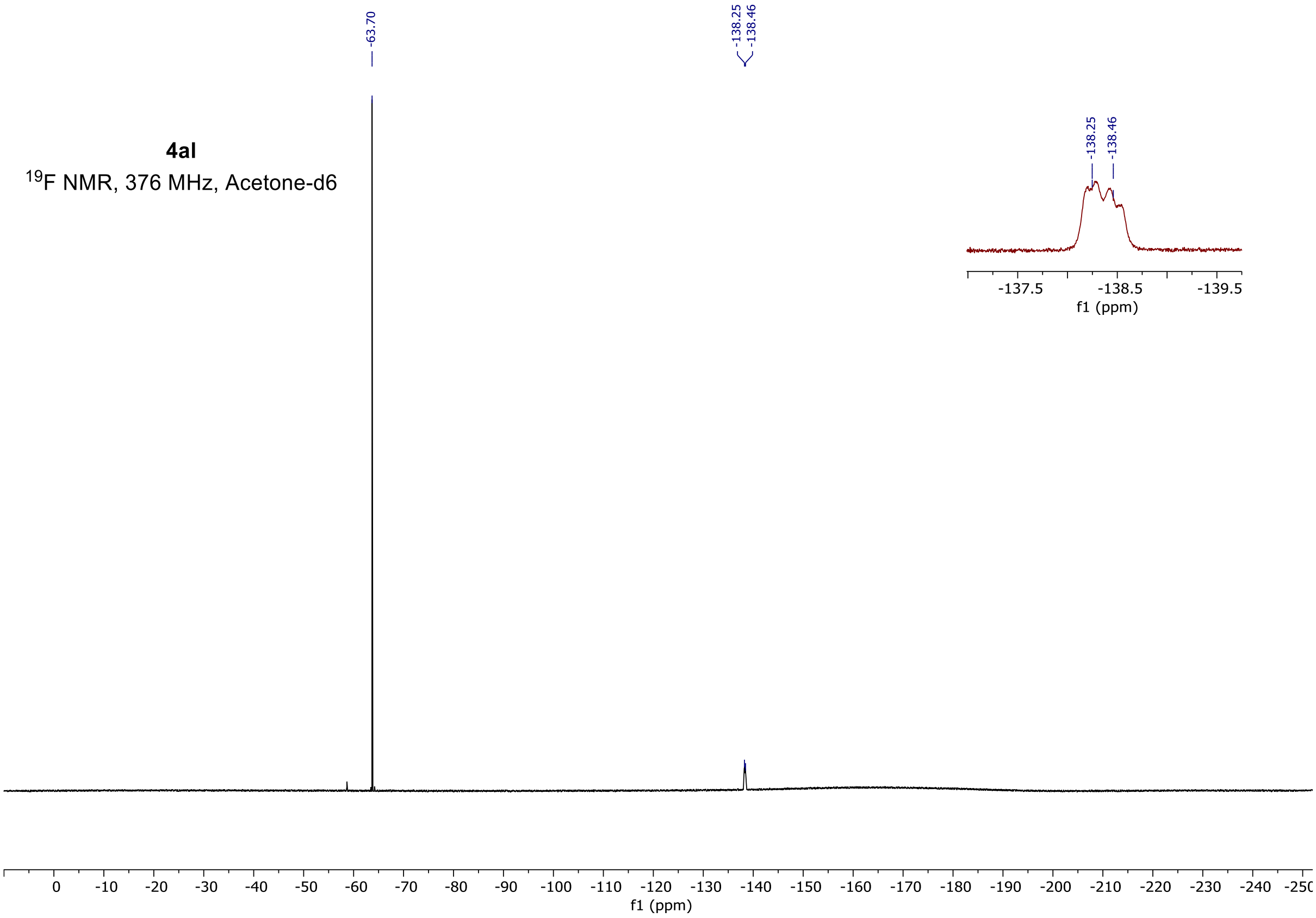
-63.70
-138.25
-138.46
4al
19F NMR, 376 MHz, Acetone-d6
-137.5
-138.5
-139.5
f1 (ppm)
0 -10 -20 -30 -40 -50 -60 -70 -80 -90 -100 -110 -120 -130 -140 -150 -160 -170 -180 -190 -200 -210 -220 -230 -240 -250
f1 (ppm)

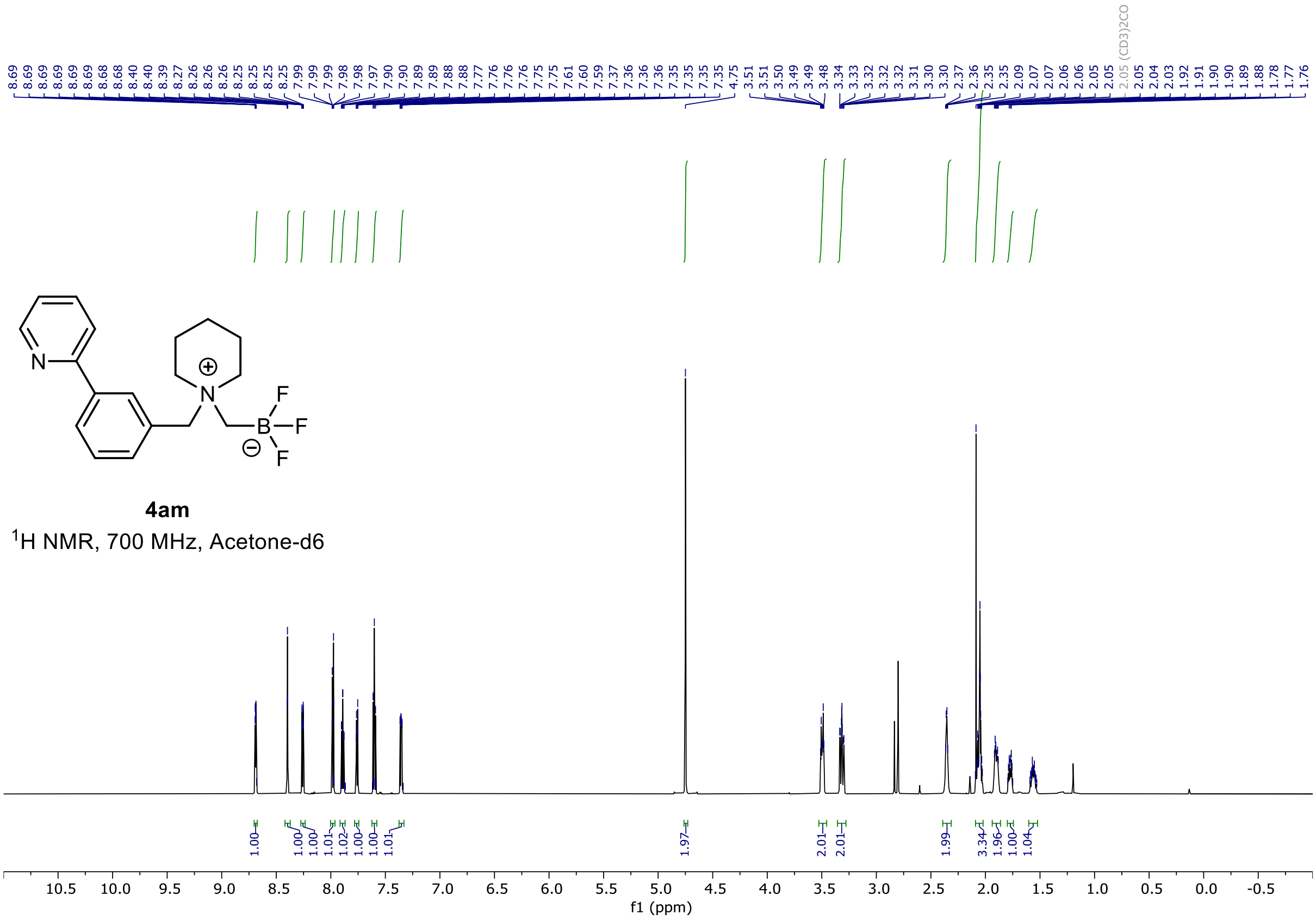
4am
1H NMR, 700 MHz, Acetone-d6
f1 (ppm)

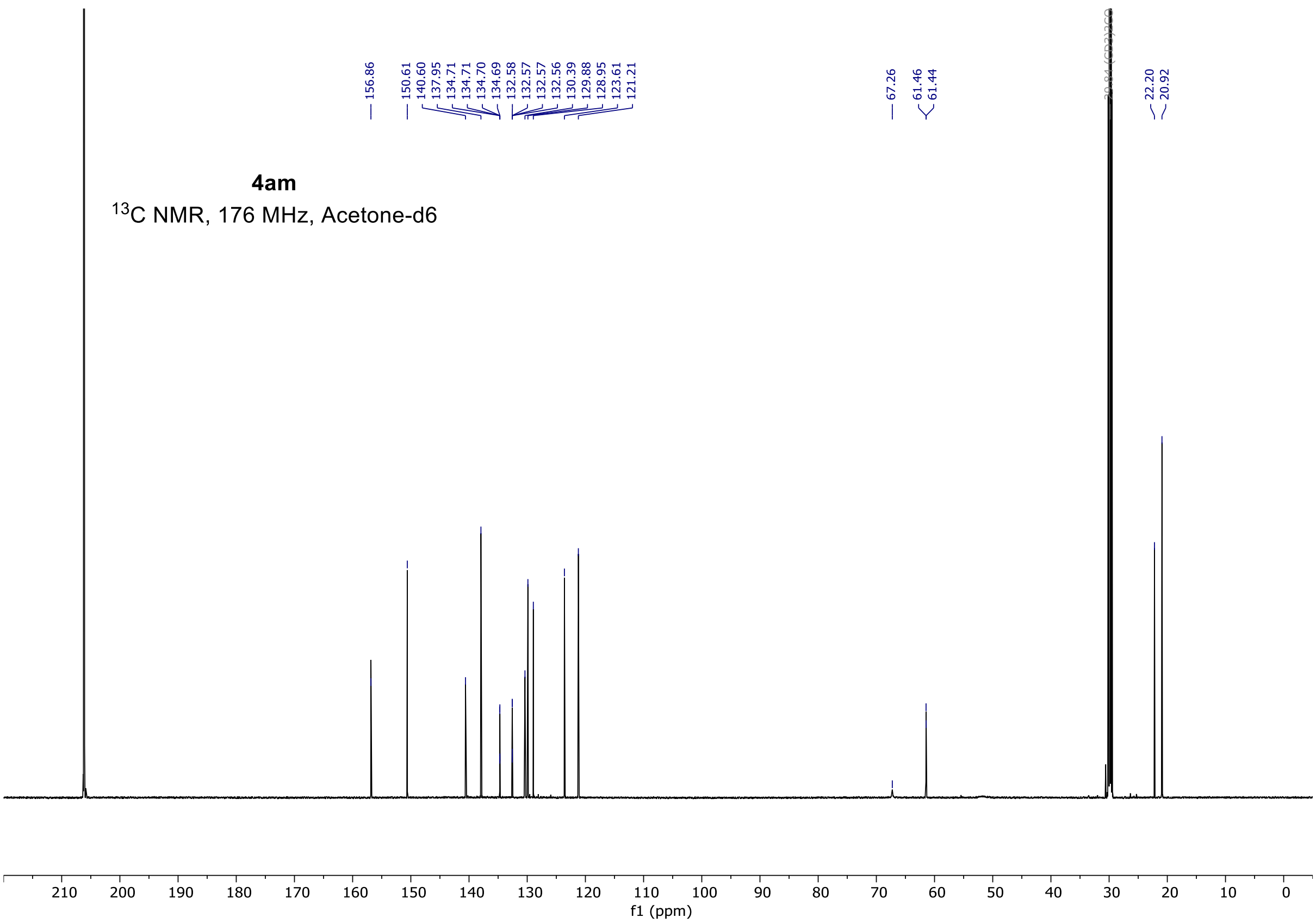
4am
13C NMR, 176 MHz, Acetone-d6
156.86
150.61
140.60
137.95
134.71
134.71
134.70
134.69
132.58
132.57
132.57
132.56
130.39
129.88
128.95
123.61
121.21
67.26
61.46
61.44
22.20
20.92
f1 (ppm)

4am

^{19}F NMR, 471 MHz, Acetone-d6

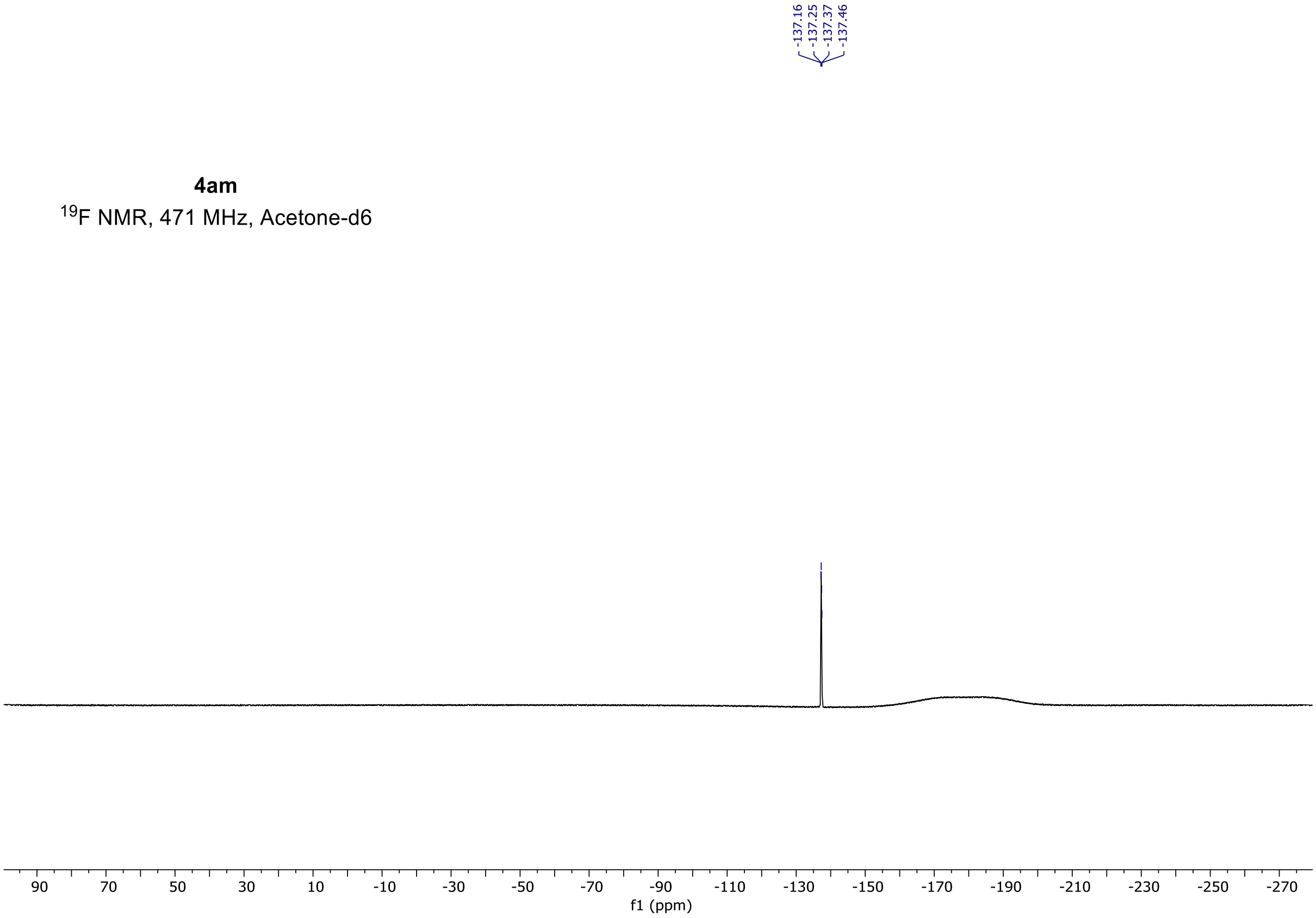

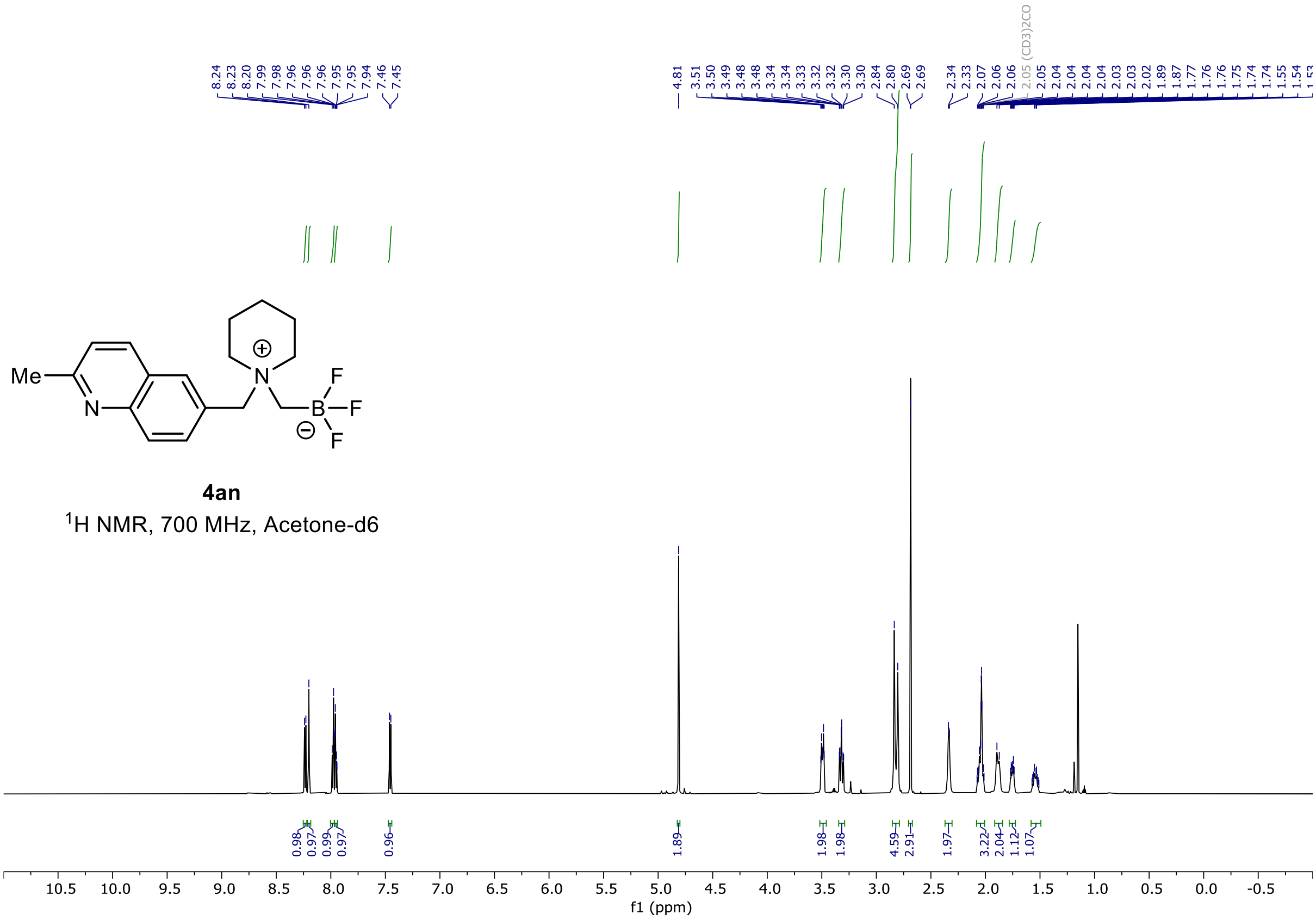

4an

^{1}H NMR, 700 MHz, Acetone-d6

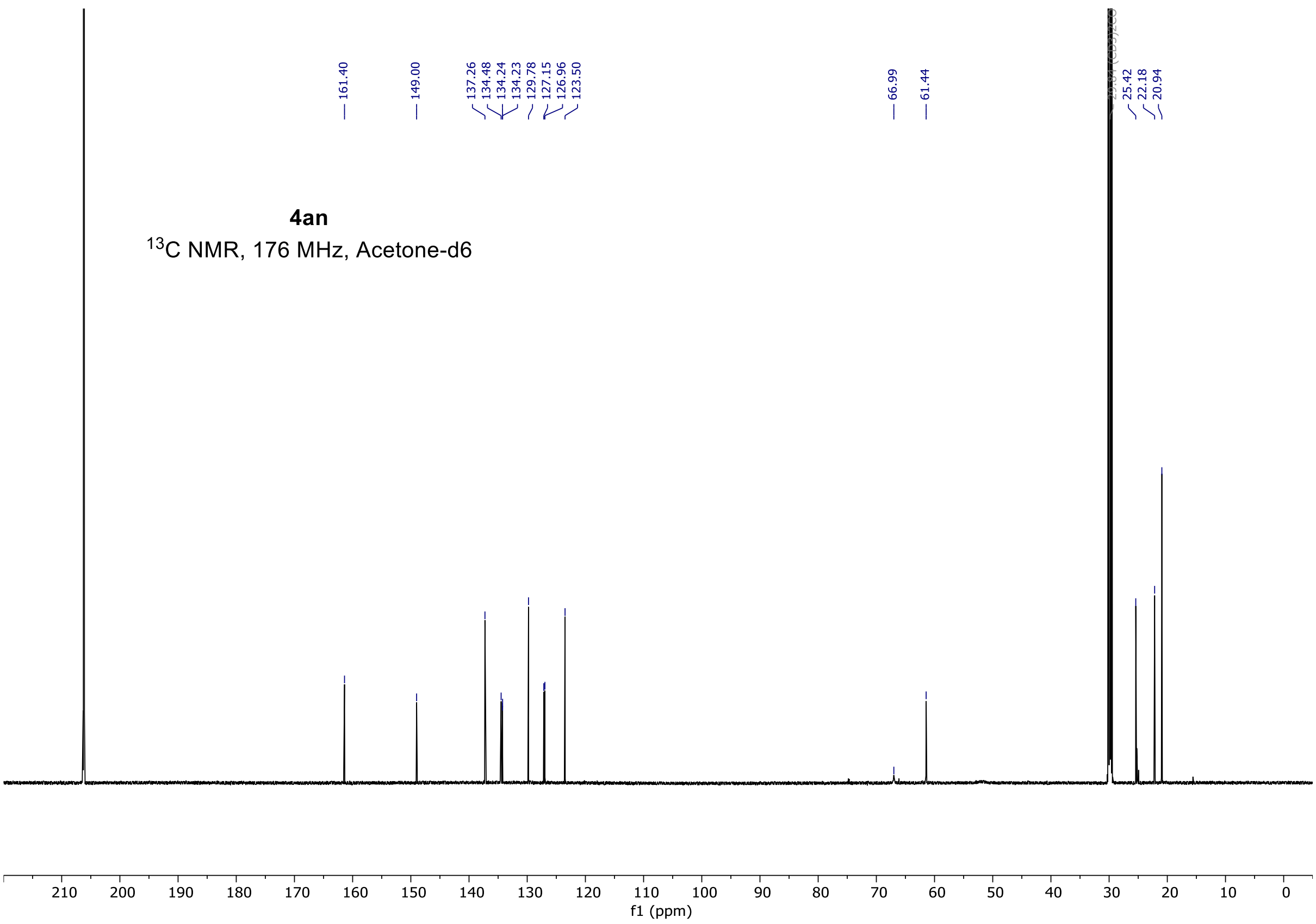

4an

^{13}C NMR, 176 MHz, Acetone-d6

4an

^{19}F NMR, 376 MHz, Acetone-d6

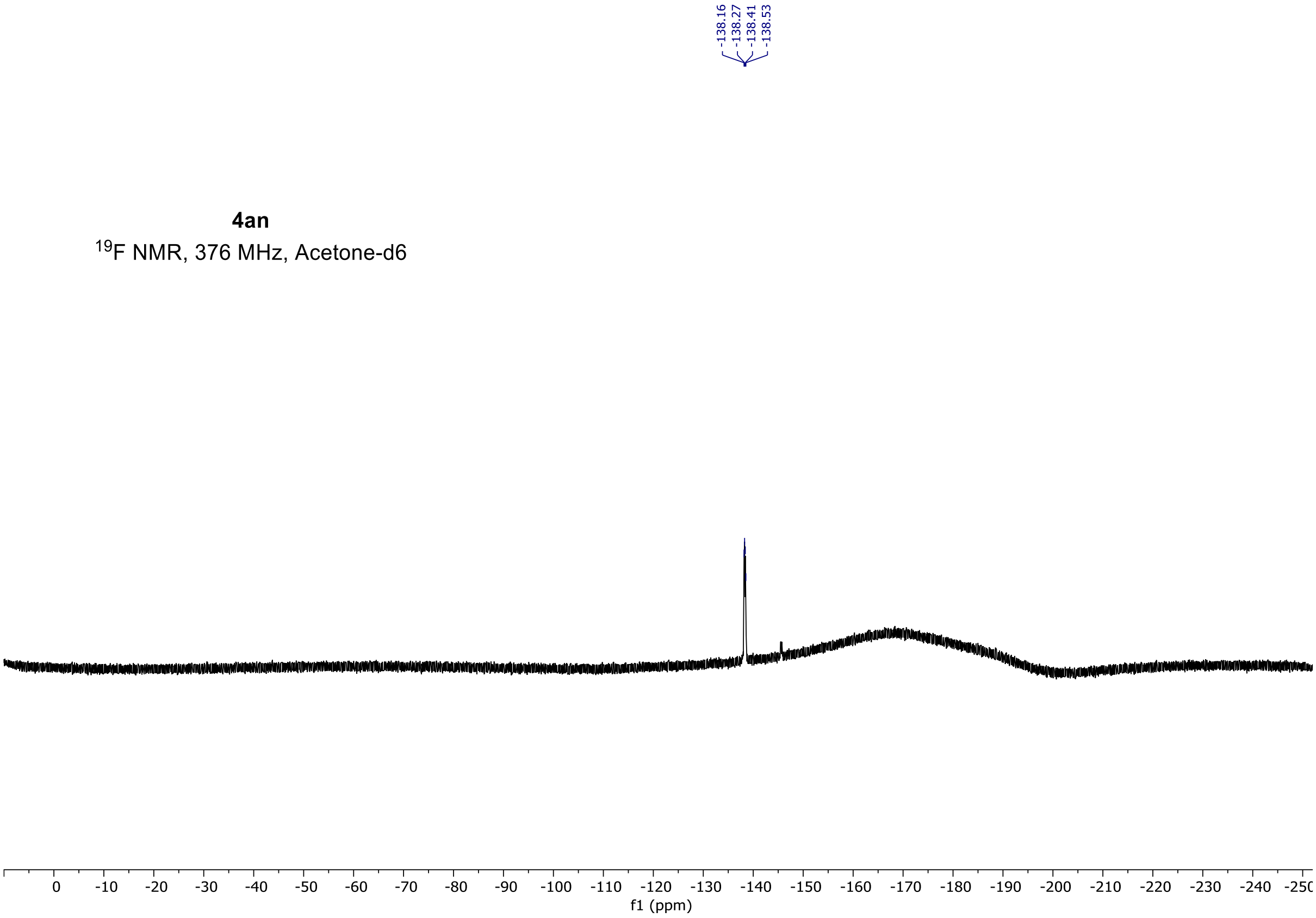

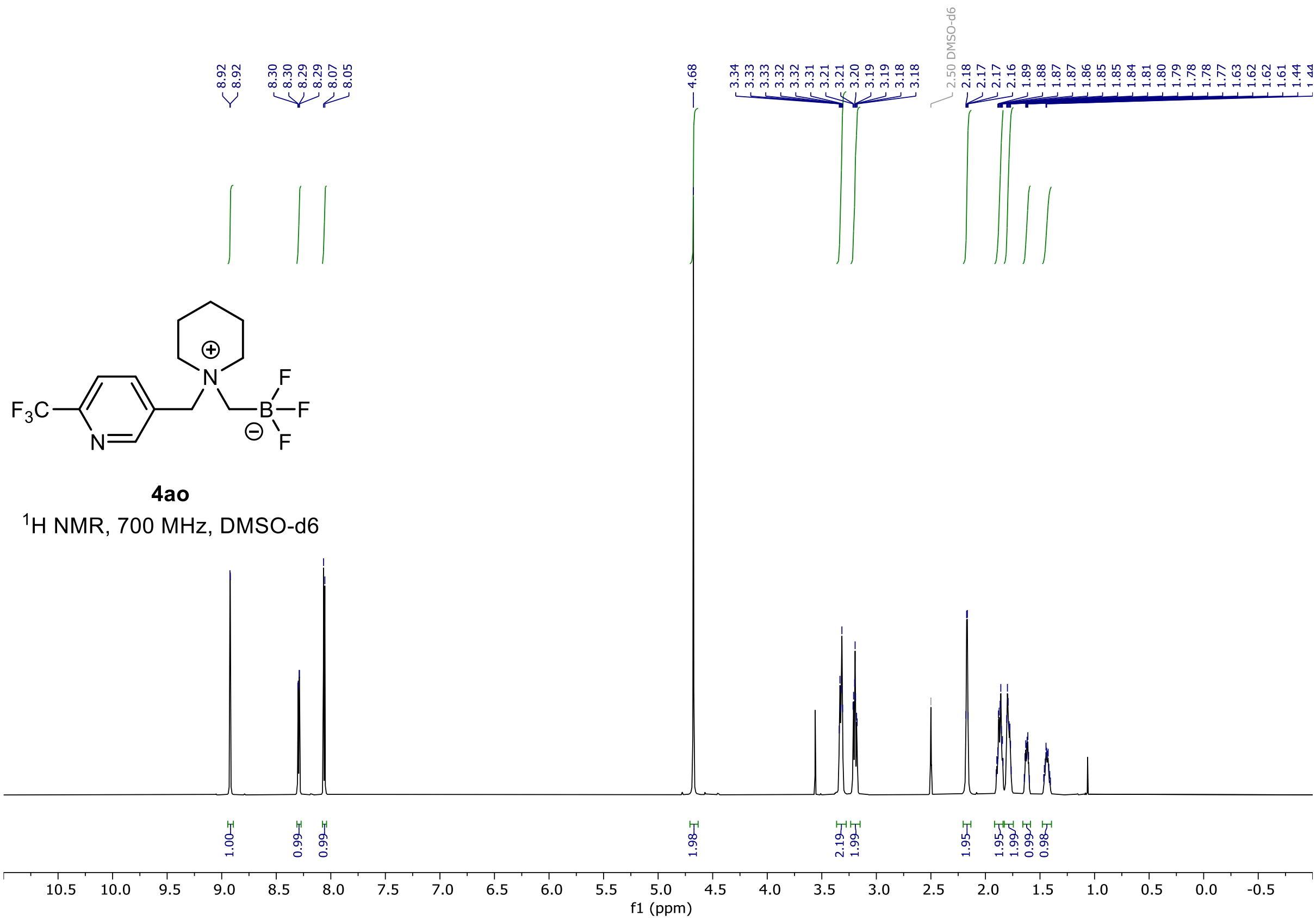

4ao

^{1}H NMR, 700 MHz, DMSO-d6

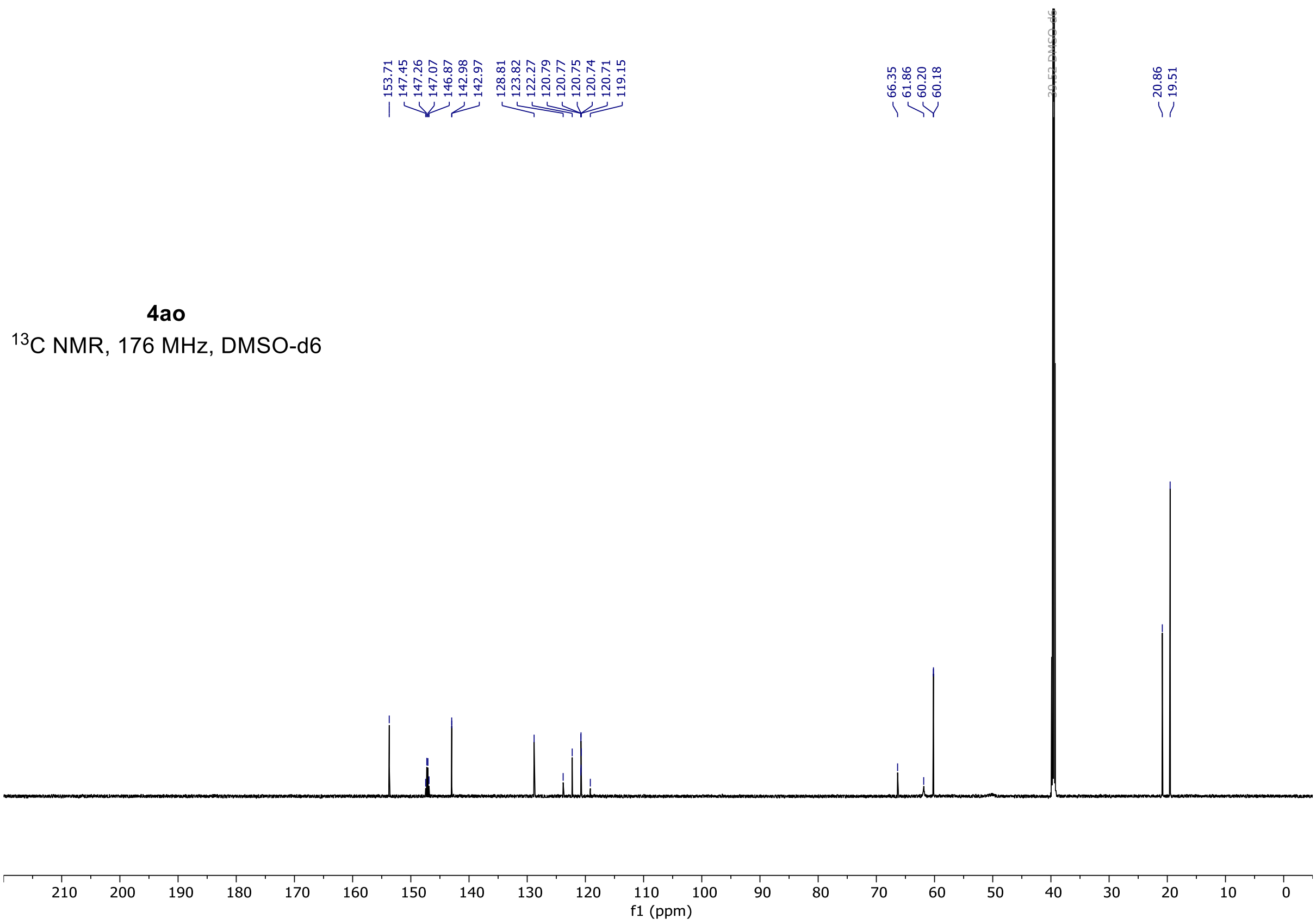

4ao

^{13}C NMR, 176 MHz, DMSO-d6

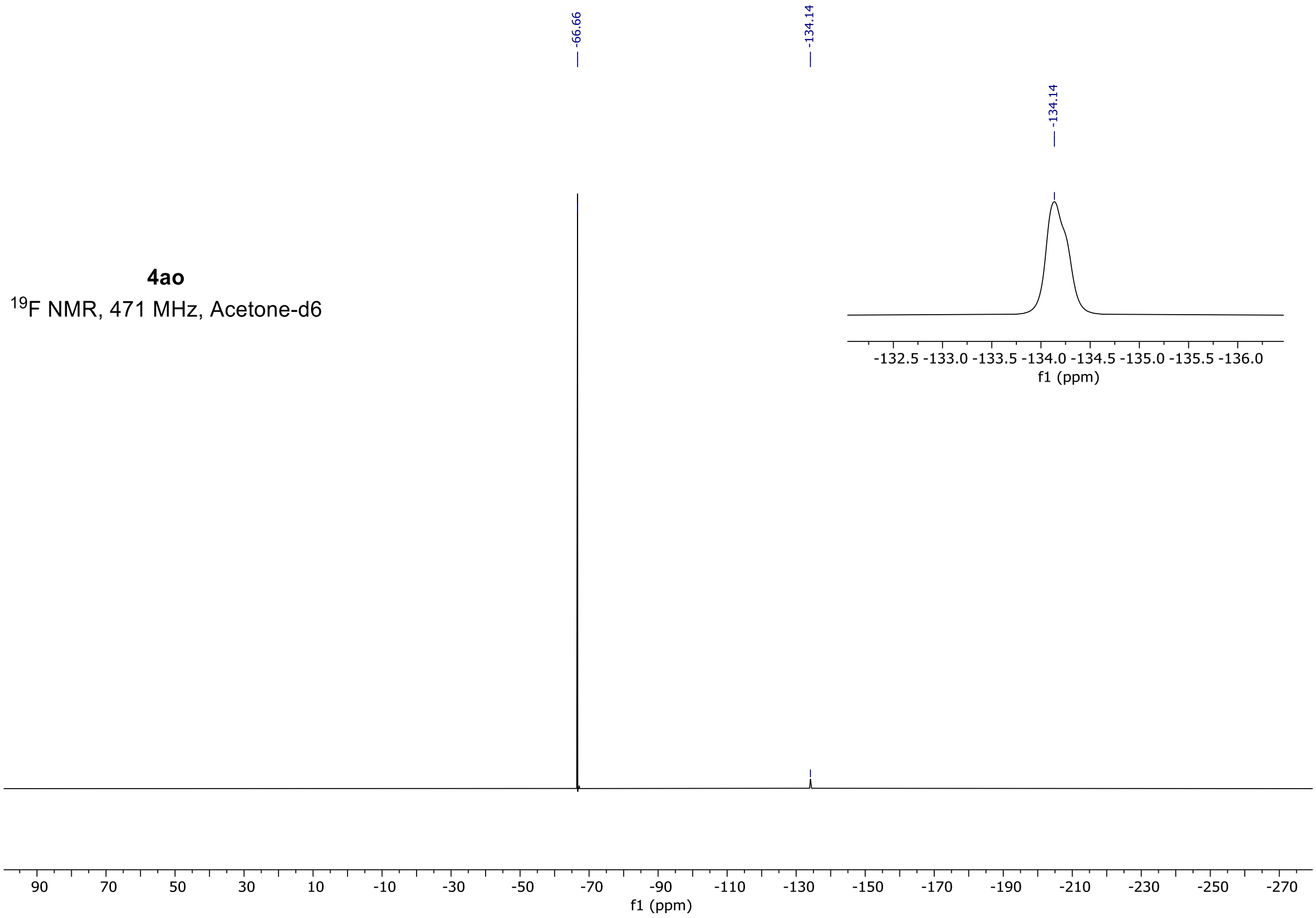
4ao
19F NMR, 471 MHz, Acetone-d6
-66.66
-134.14
-134.14
-132.5 -133.0 -133.5 -134.0 -134.5 -135.0 -135.5 -136.0
f1 (ppm)
90 70 50 30 10 -10 -30 -50 -70 -90 -110 -130 -150 -170 -190 -210 -230 -250 -270
f1 (ppm)

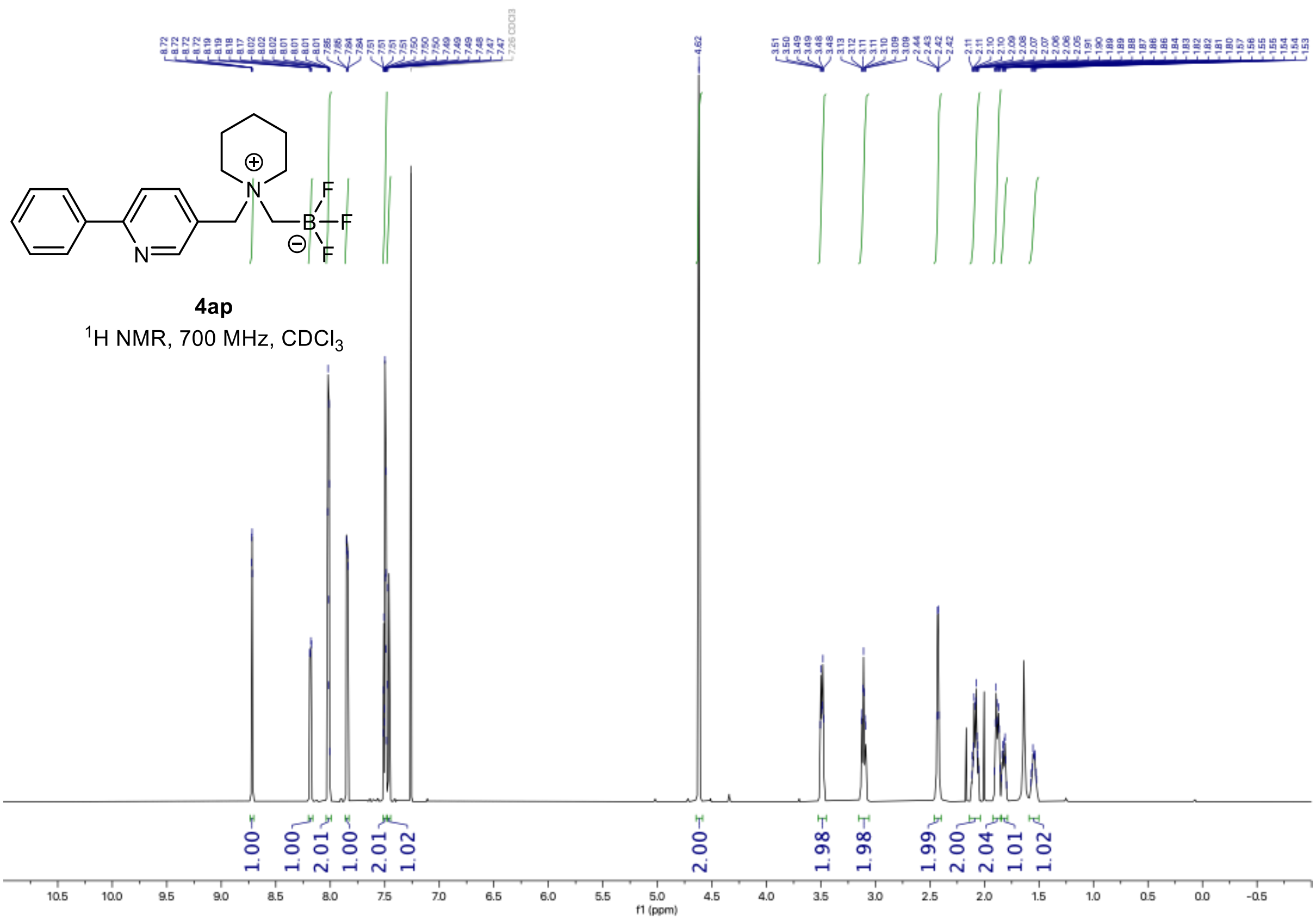
4ap
^{1}H NMR, 700 MHz, $CDCl_3$
f1 (ppm)

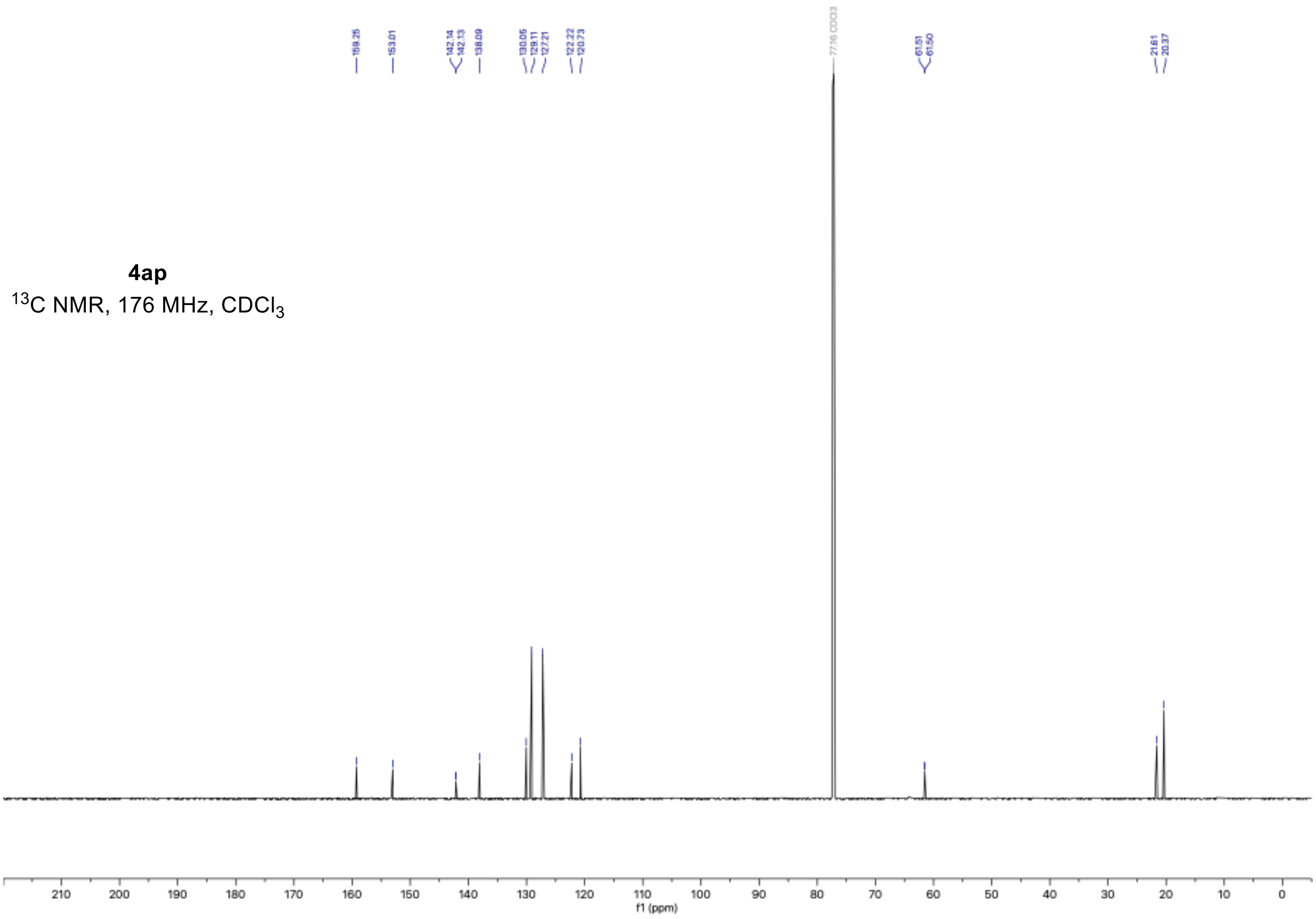
4ap
^{13}C NMR, 176 MHz, $CDCl_3$
f1 (ppm)

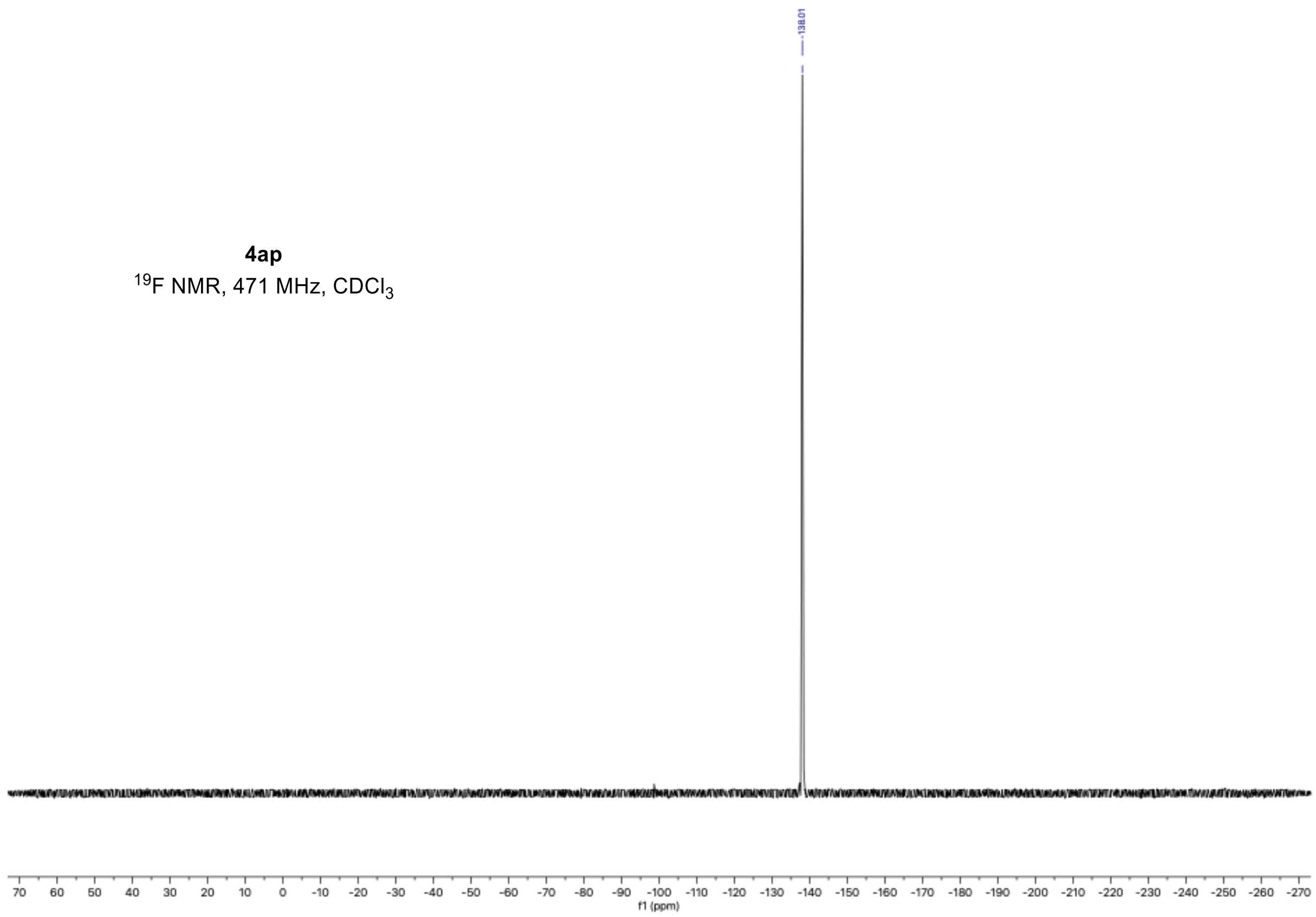
-138.01
4ap
19F NMR, 471 MHz, CDCl3
70 60 50 40 30 20 10 0 -10 -20 -30 -40 -50 -60 -70 -80 -90 -100 -110 -120 -130 -140 -150 -160 -170 -180 -190 -200 -210 -220 -230 -240 -250 -260 -270
f1 (ppm)

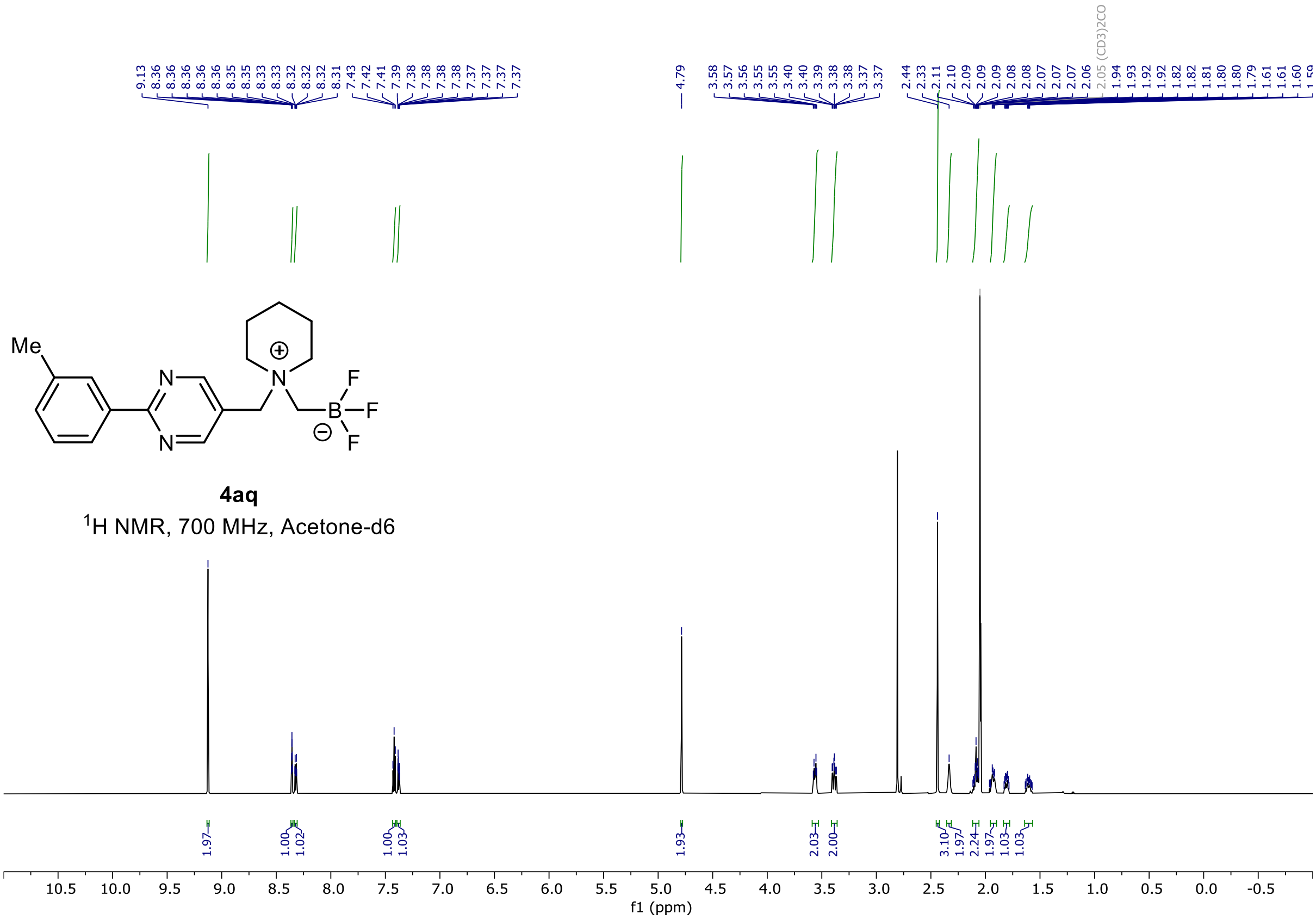

Me
N
N
N
B
F
F
F
4aq
^{1}H NMR, 700 MHz, Acetone-d6
f1 (ppm)

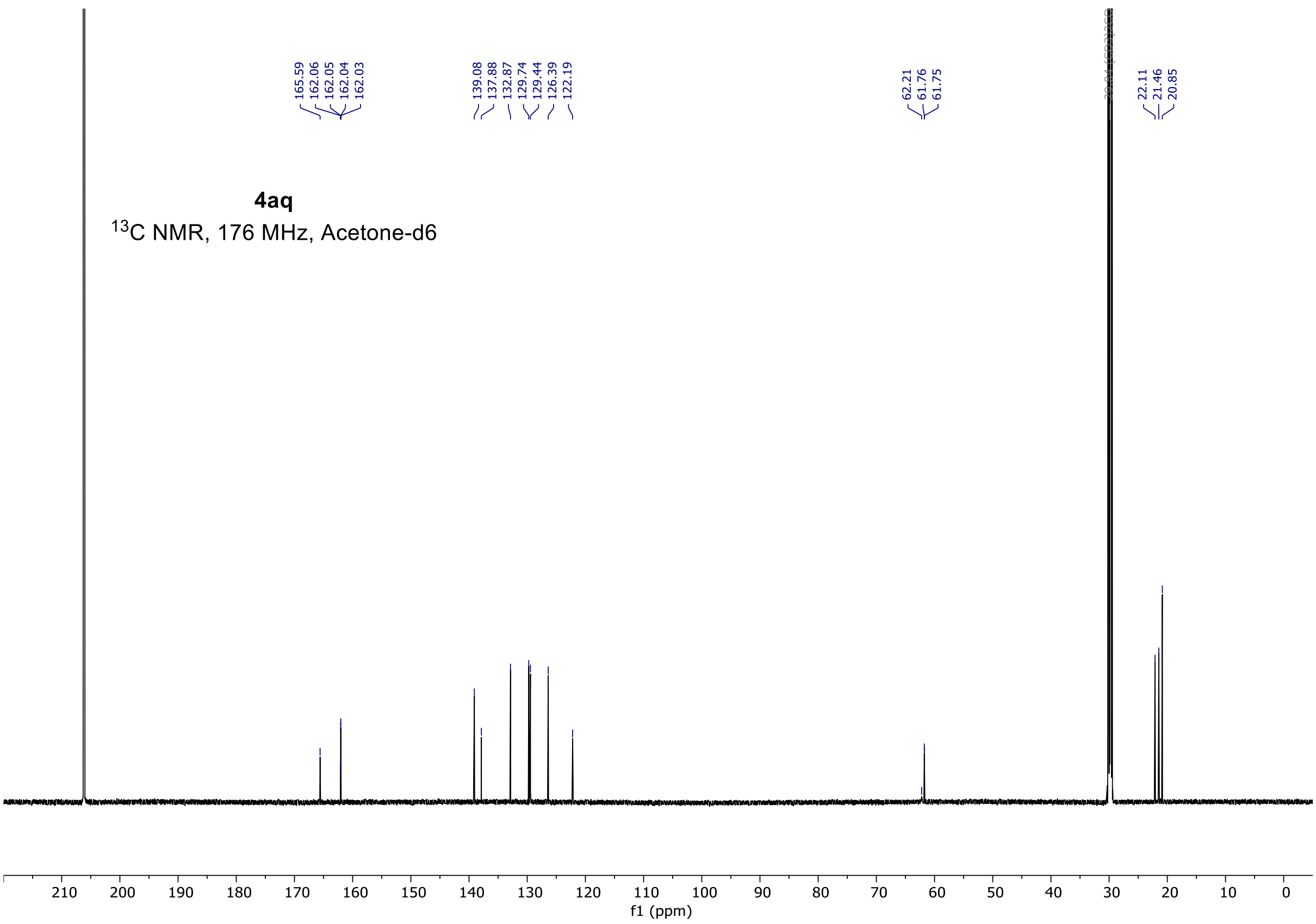

4aq
^{13}C NMR, 176 MHz, Acetone-d6
f1 (ppm)

4aq
^{19}F NMR, 471 MHz, Acetone-d6

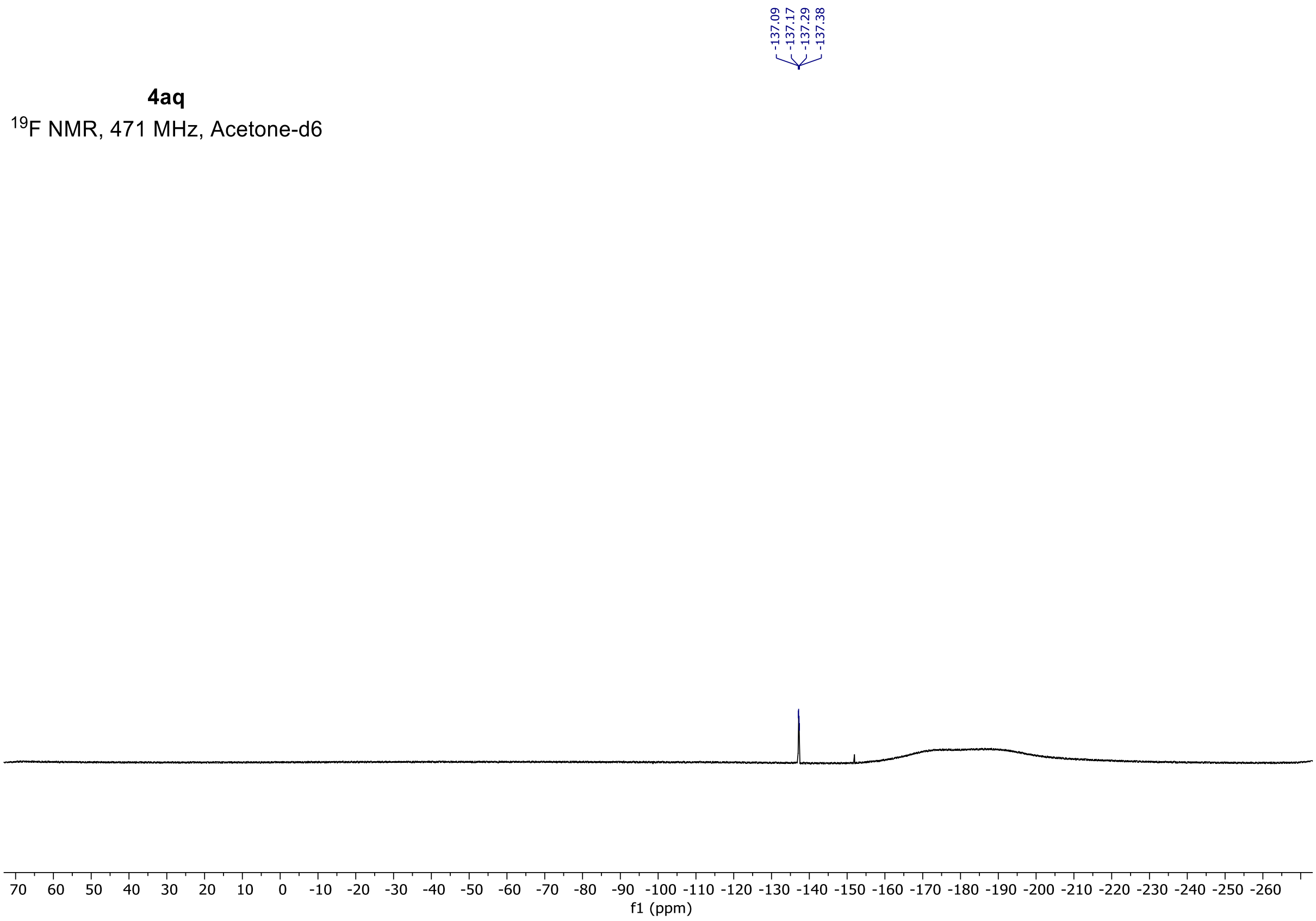

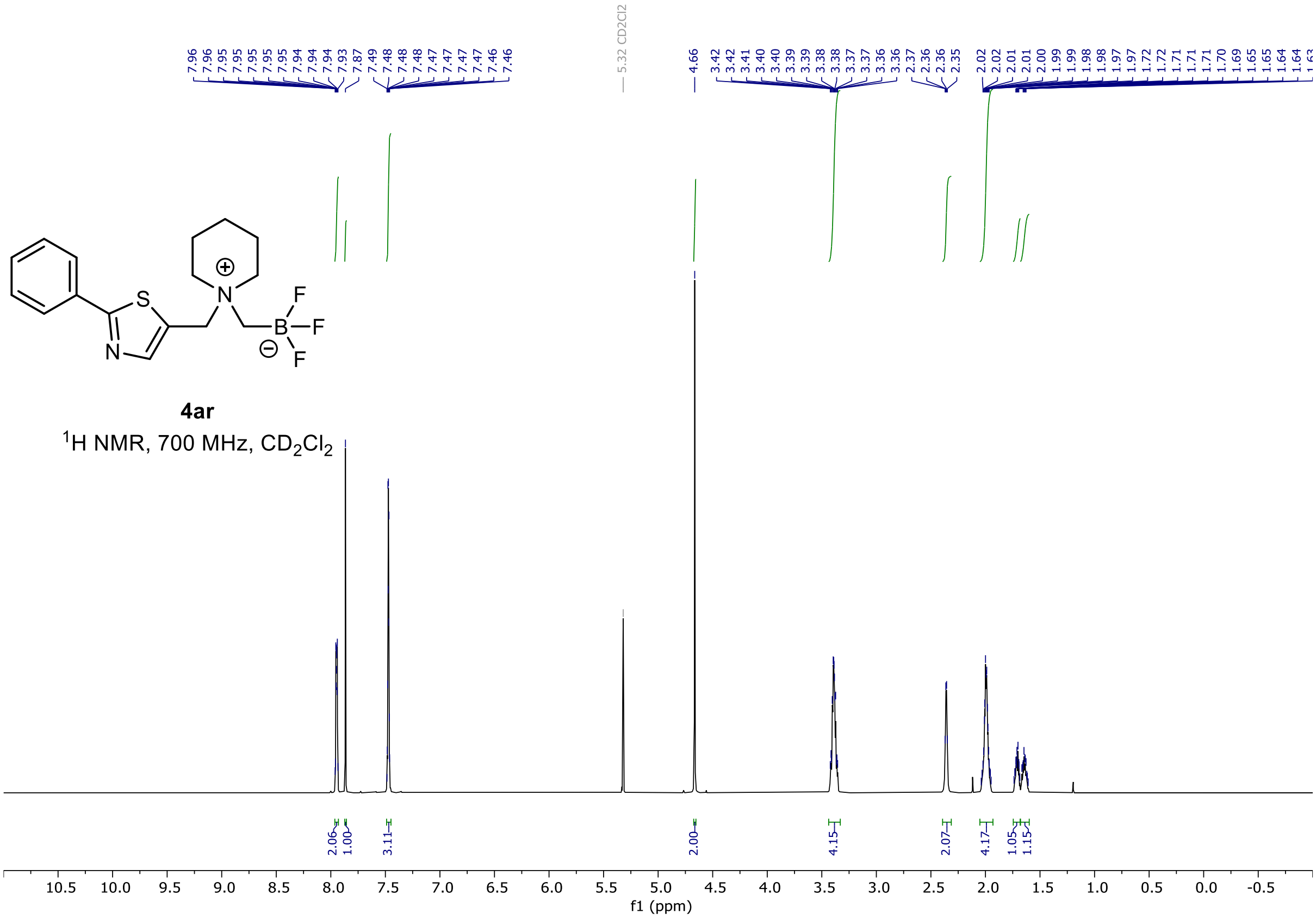

4ar

^{1}H NMR, 700 MHz, CD_2Cl_2

4ar

^{13}C NMR, 176 MHz, CD_2Cl_2

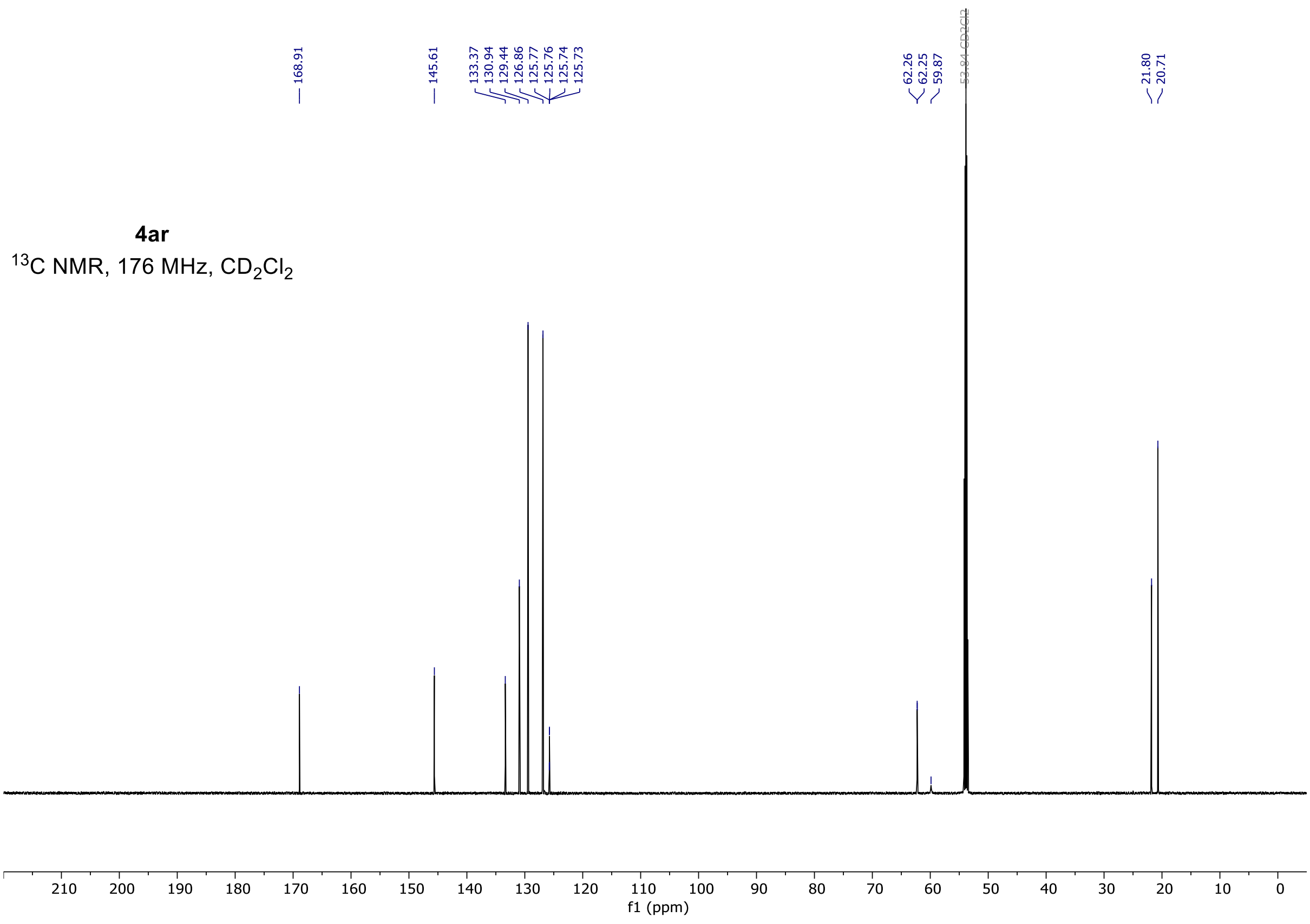

4ar

^{19}F NMR, 471 MHz, CD_2Cl_2

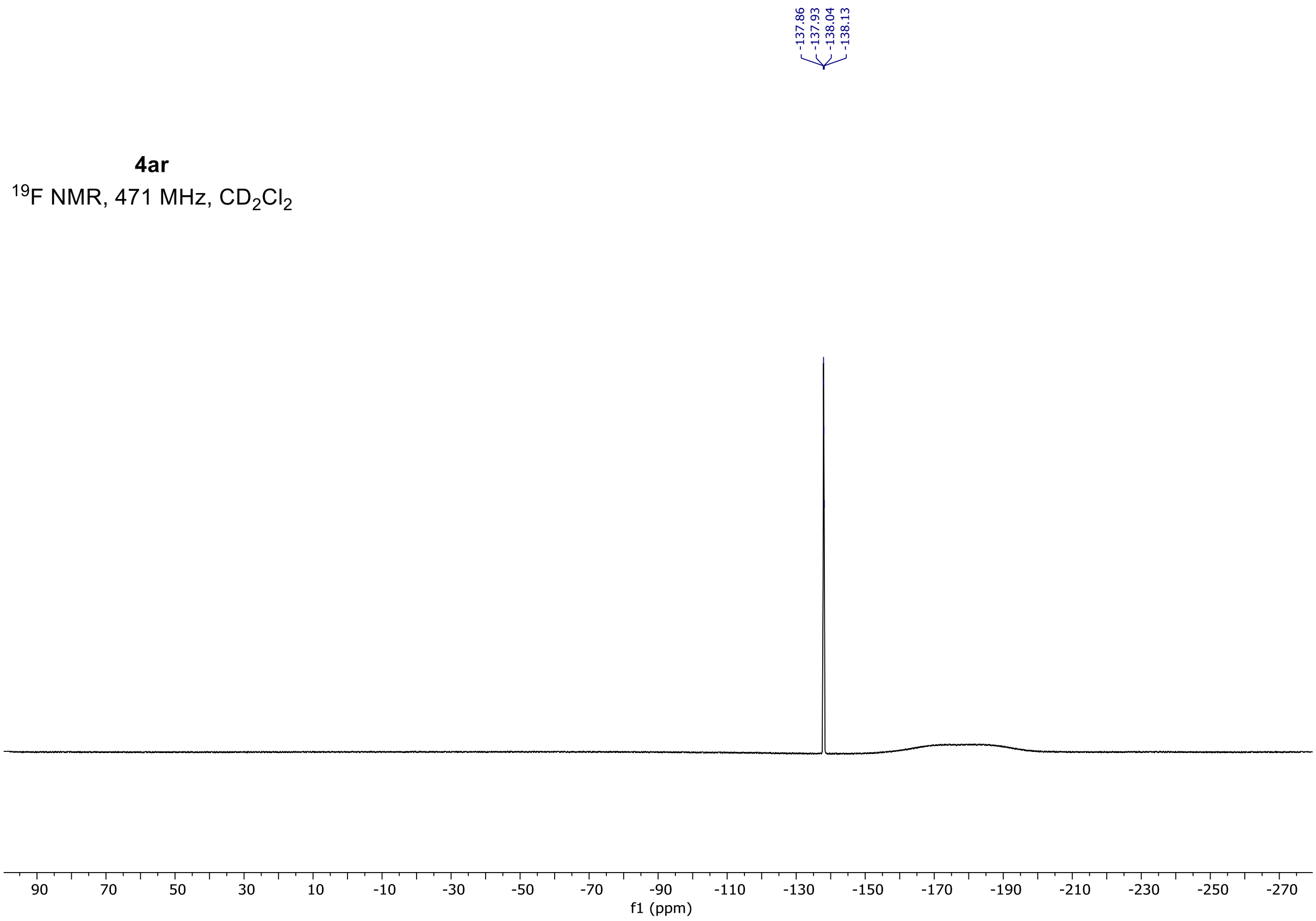

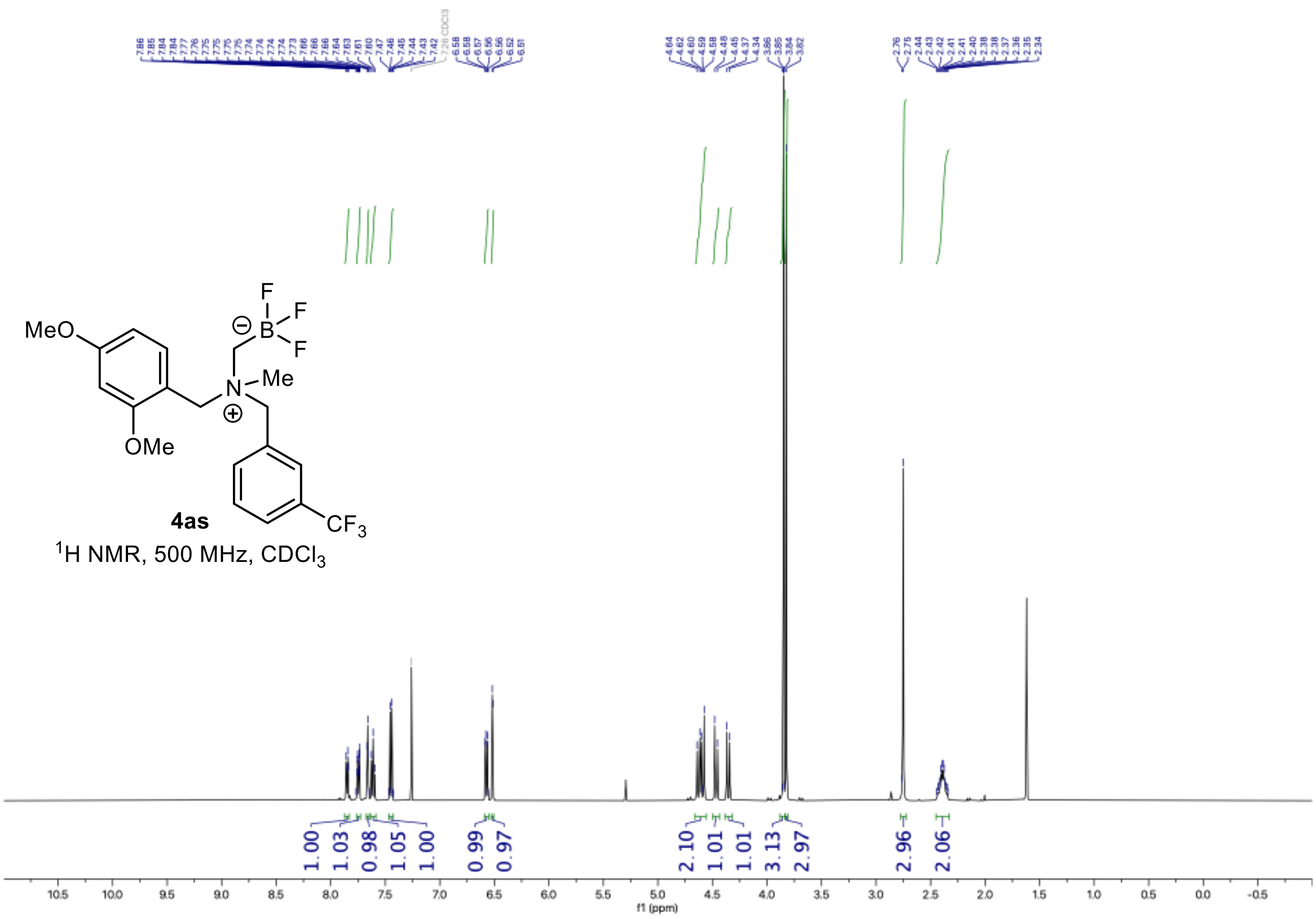
4as
^{1}H NMR, 500 MHz, CDCl$_3$
f1 (ppm)

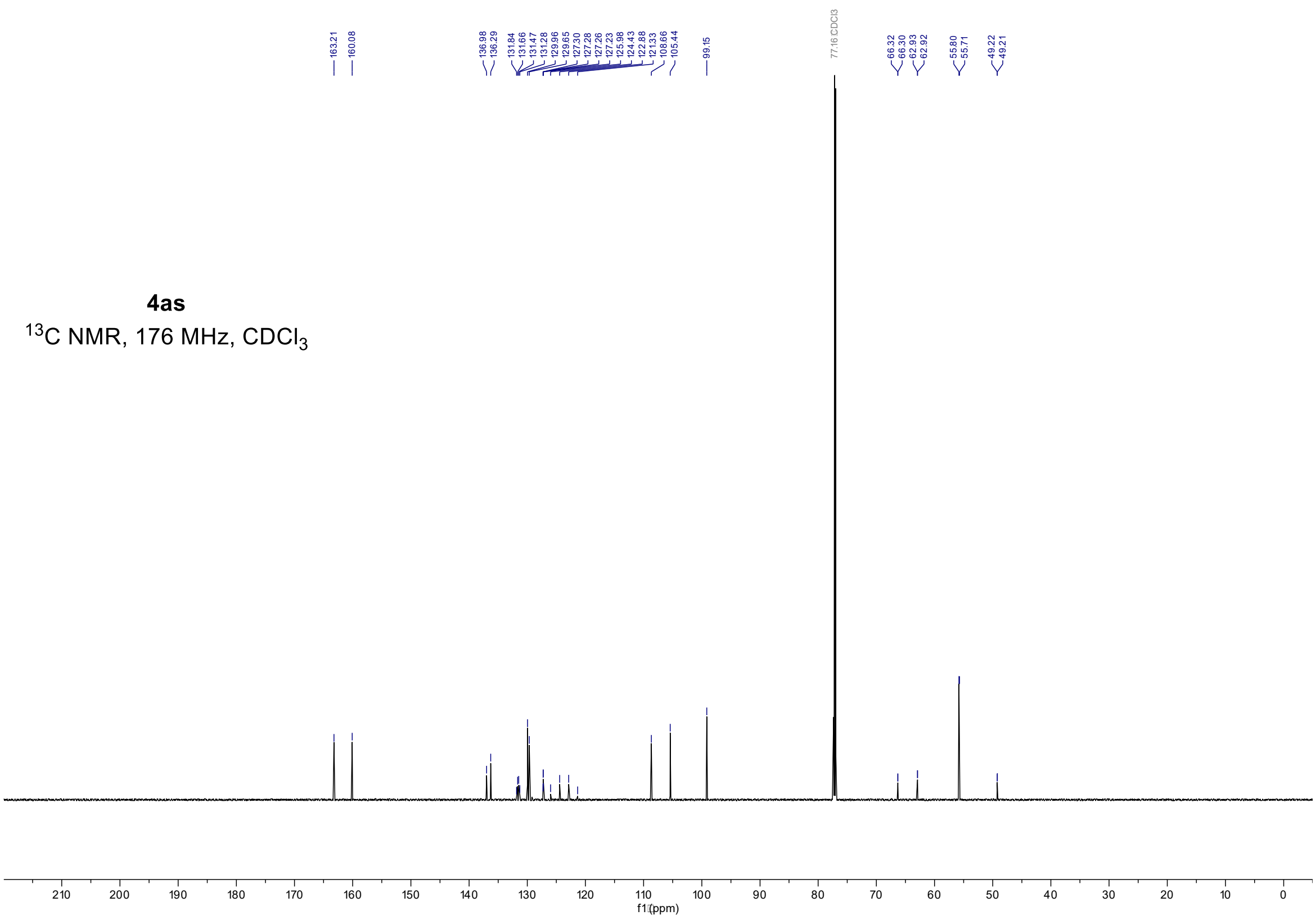
4as
^{13}C NMR, 176 MHz, CDCl$_3$
f1 (ppm)

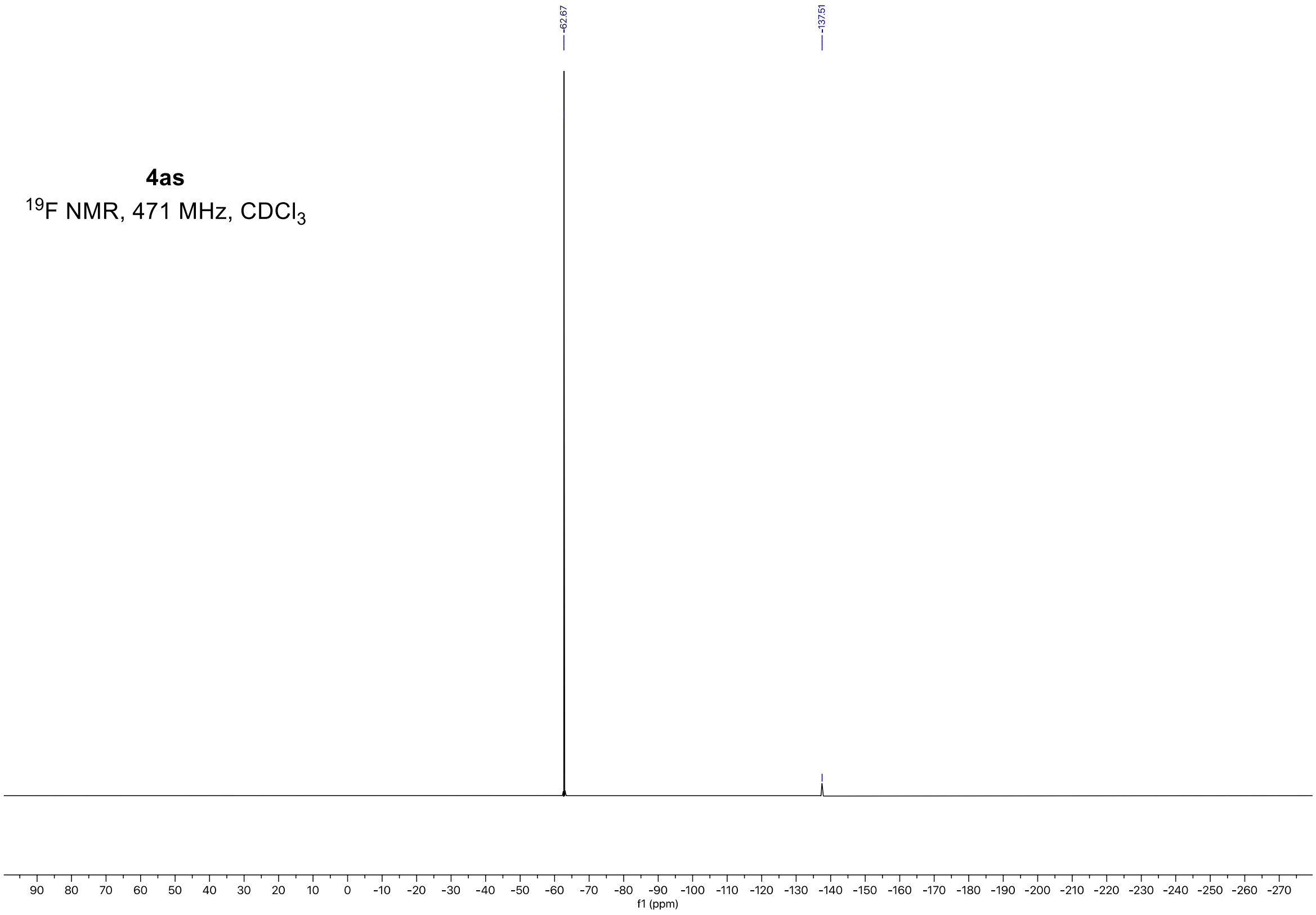
-62.67
-137.51
4as
^{19}F NMR, 471 MHz, CDCl$_3$
90 80 70 60 50 40 30 20 10 0 -10 -20 -30 -40 -50 -60 -70 -80 -90 -100 -110 -120 -130 -140 -150 -160 -170 -180 -190 -200 -210 -220 -230 -240 -250 -260 -270
f1 (ppm)

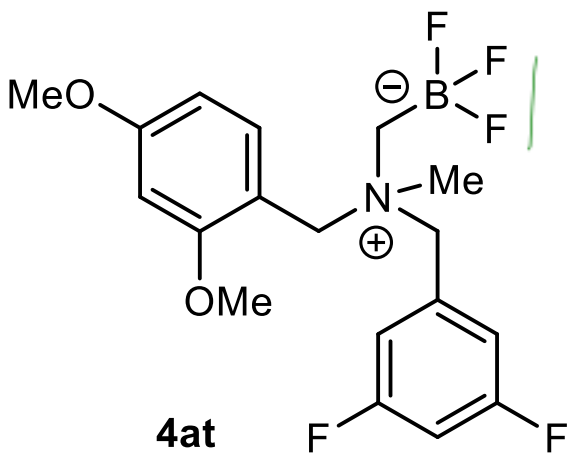

4at

^{1}H NMR, 500 MHz, $CDCl_3$

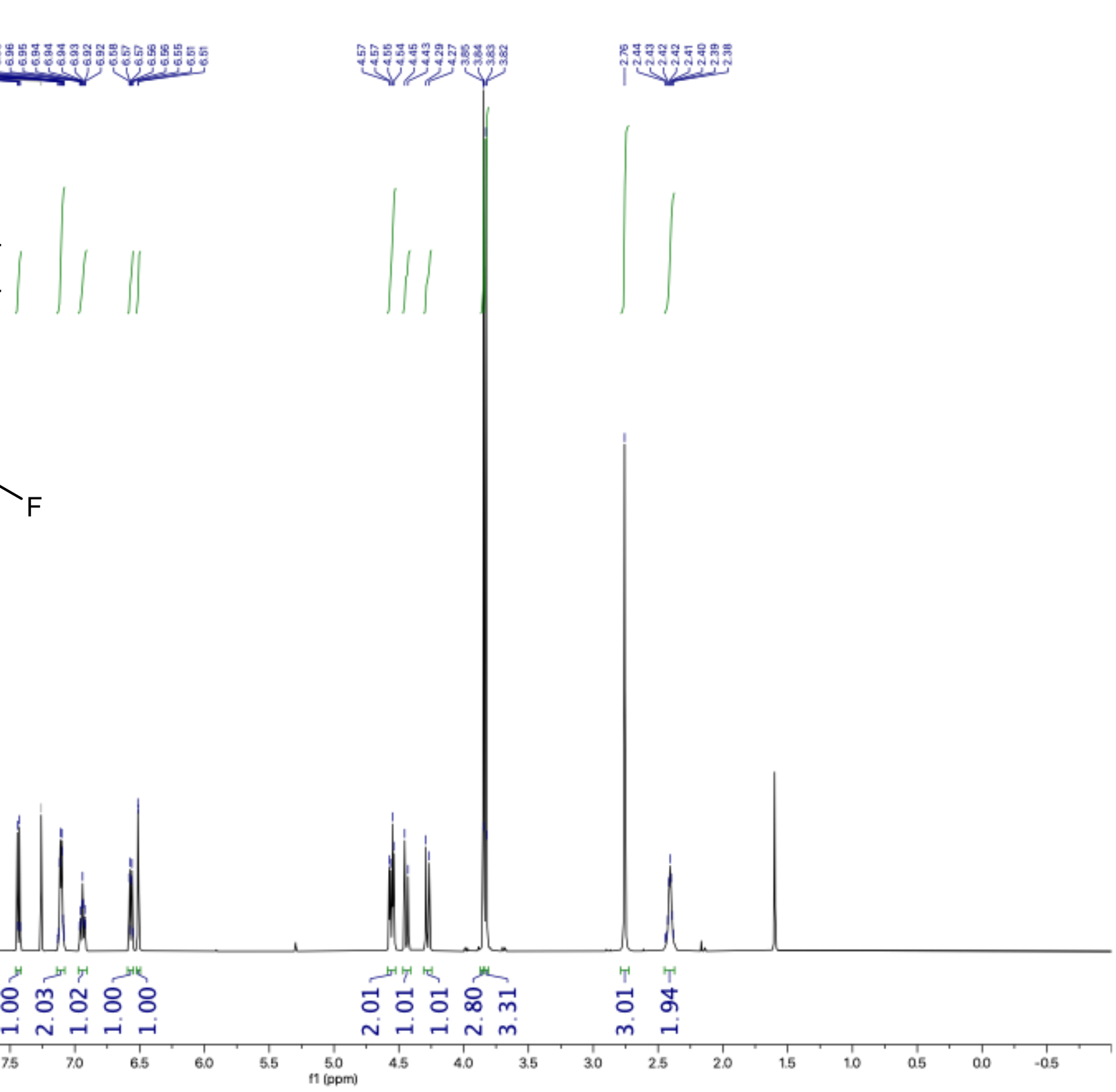

4at

^{13}C NMR, 126 MHz, $CDCl_3$

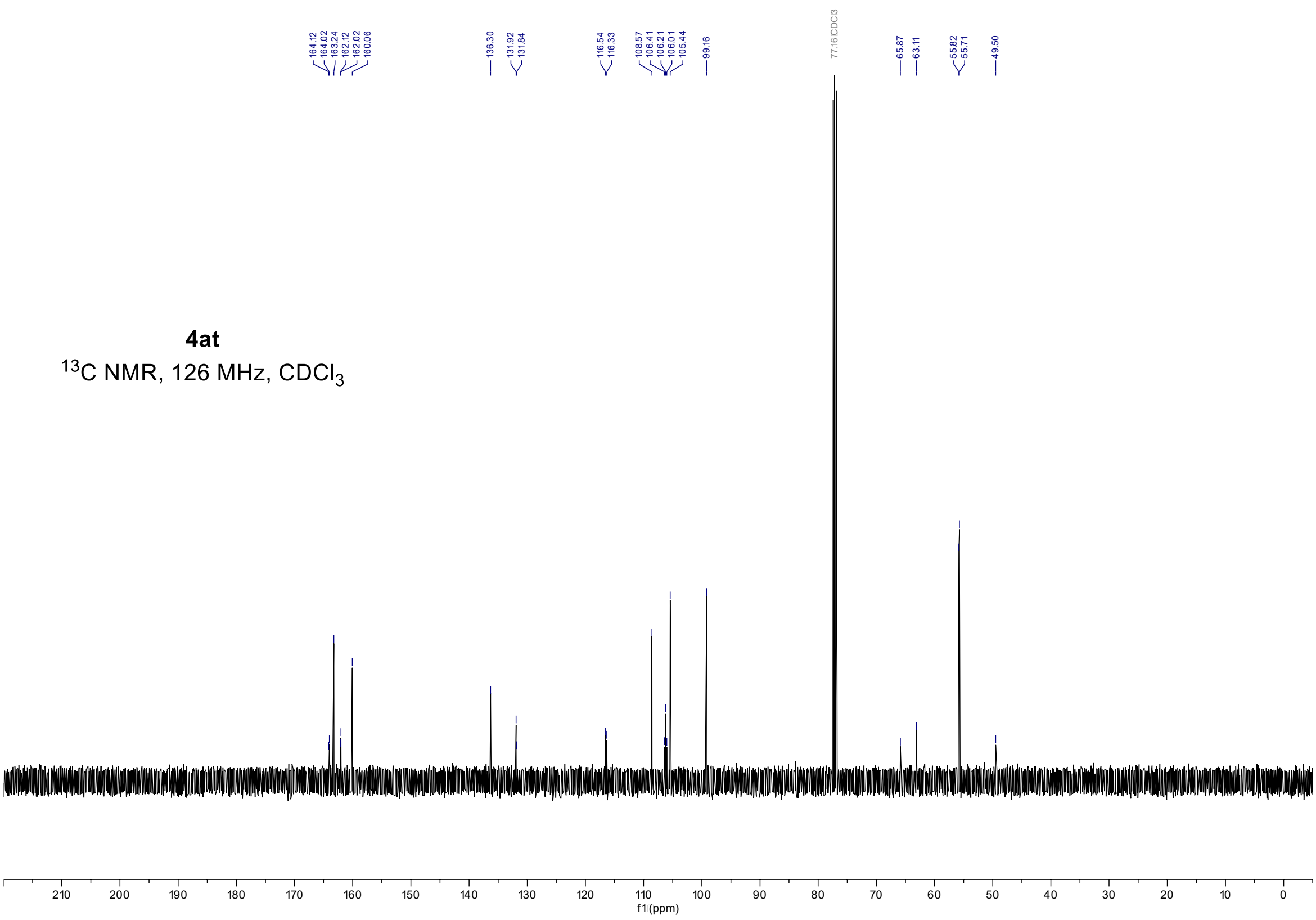

4at

^{19}F NMR, 471 MHz, $CDCl_3$

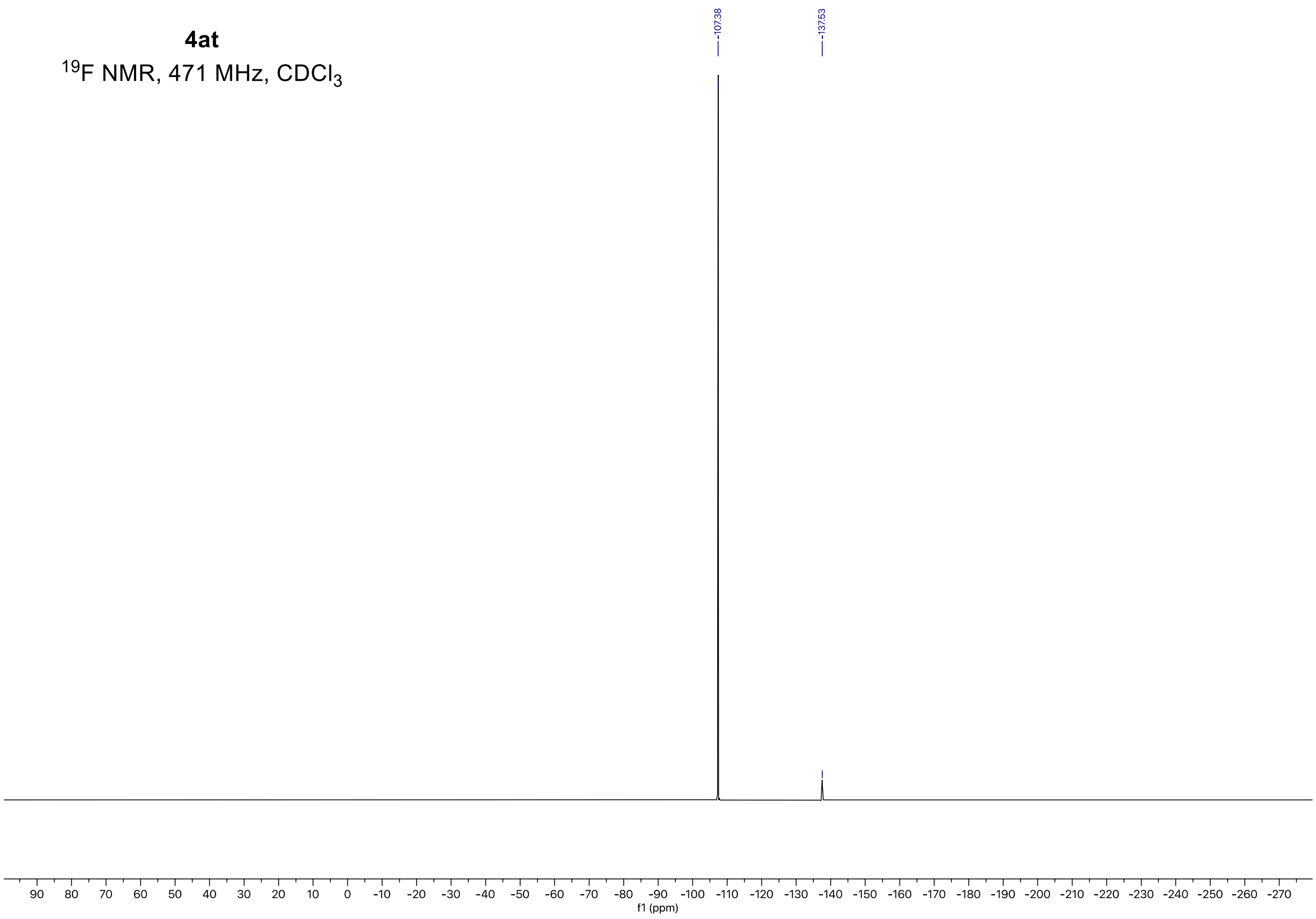

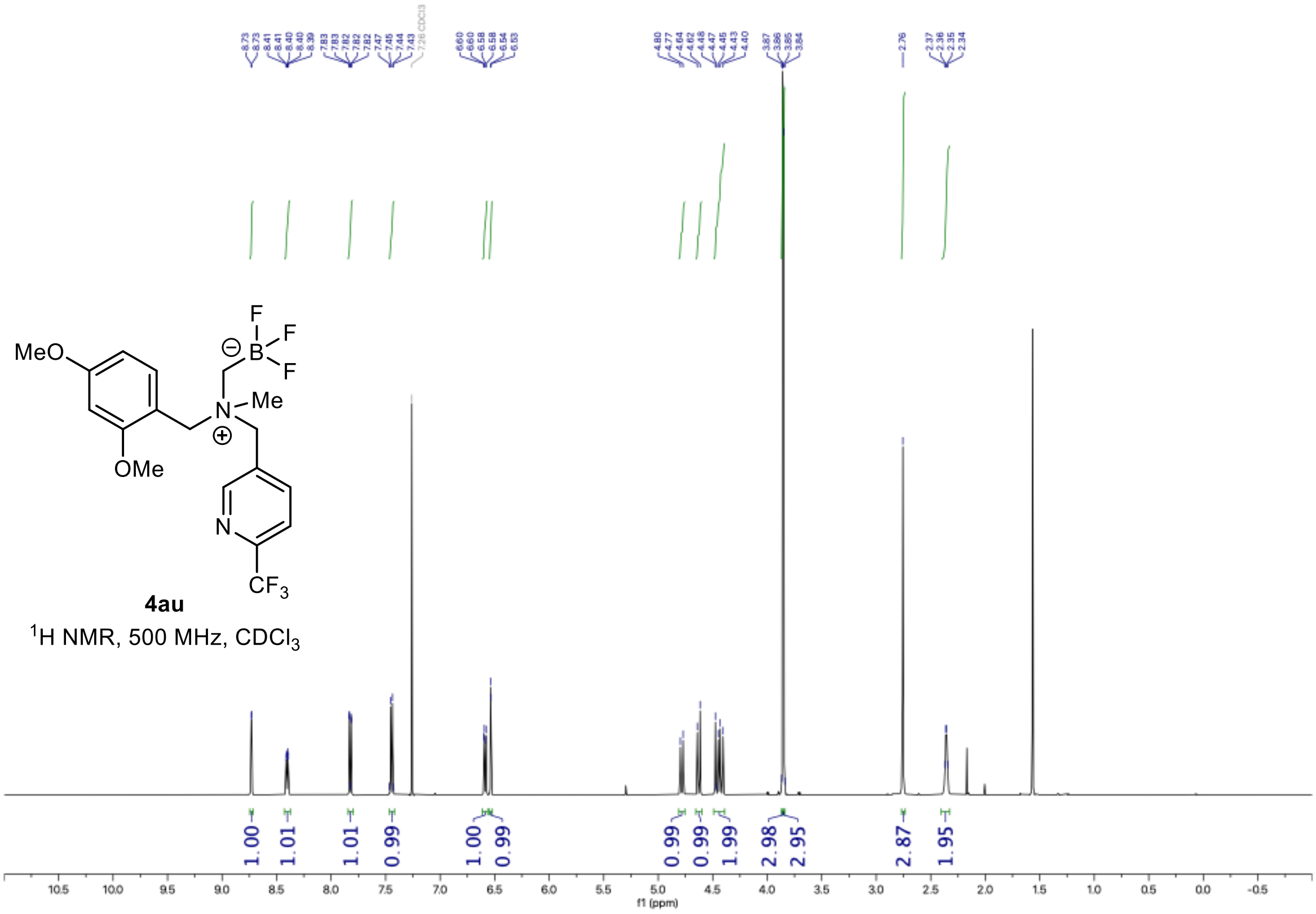

4au

^{1}H NMR, 500 MHz, $CDCl_3$

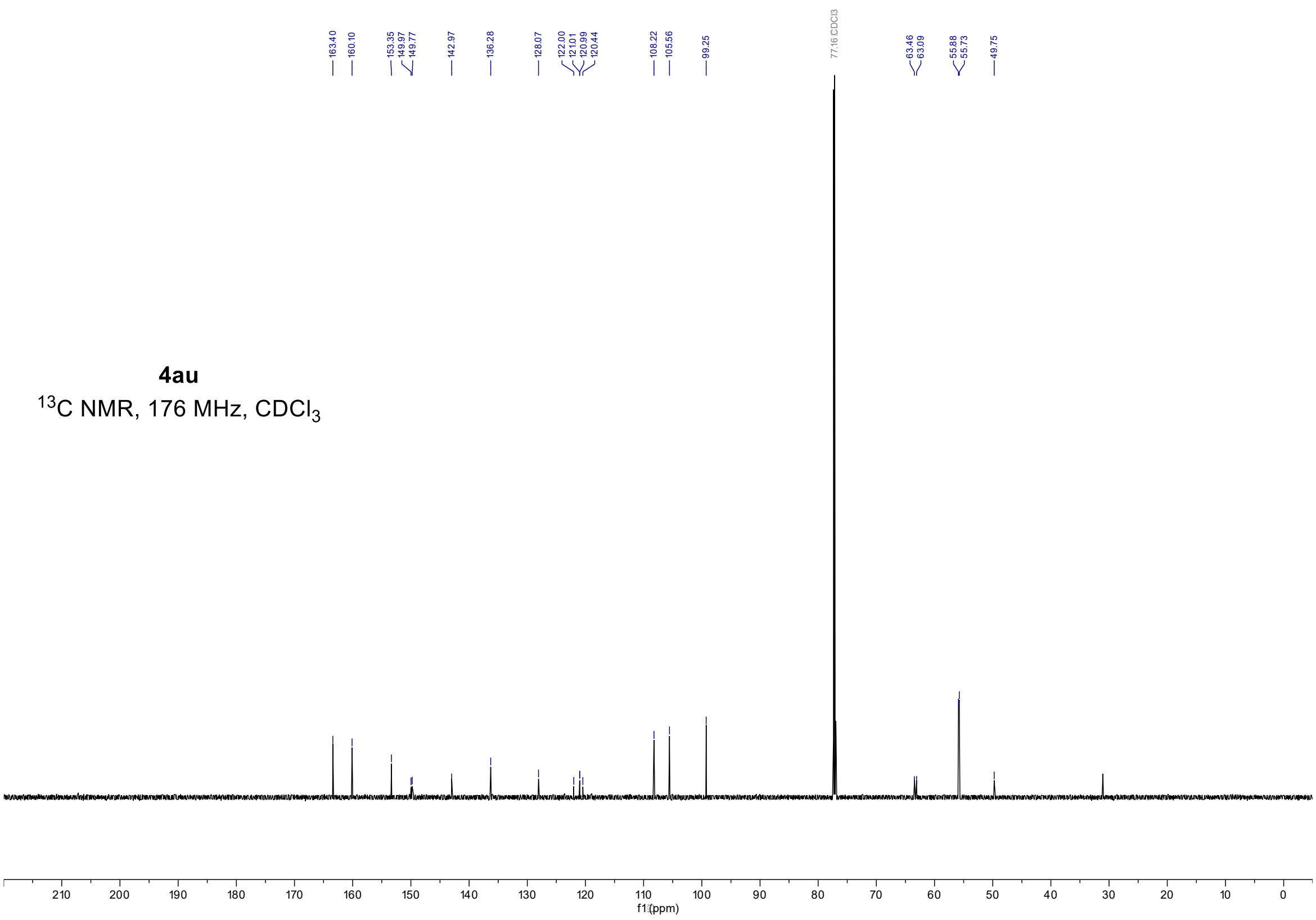

4au

^{13}C NMR, 176 MHz, $CDCl_3$

4au
^{19}F NMR, 471 MHz, $CDCl_3$

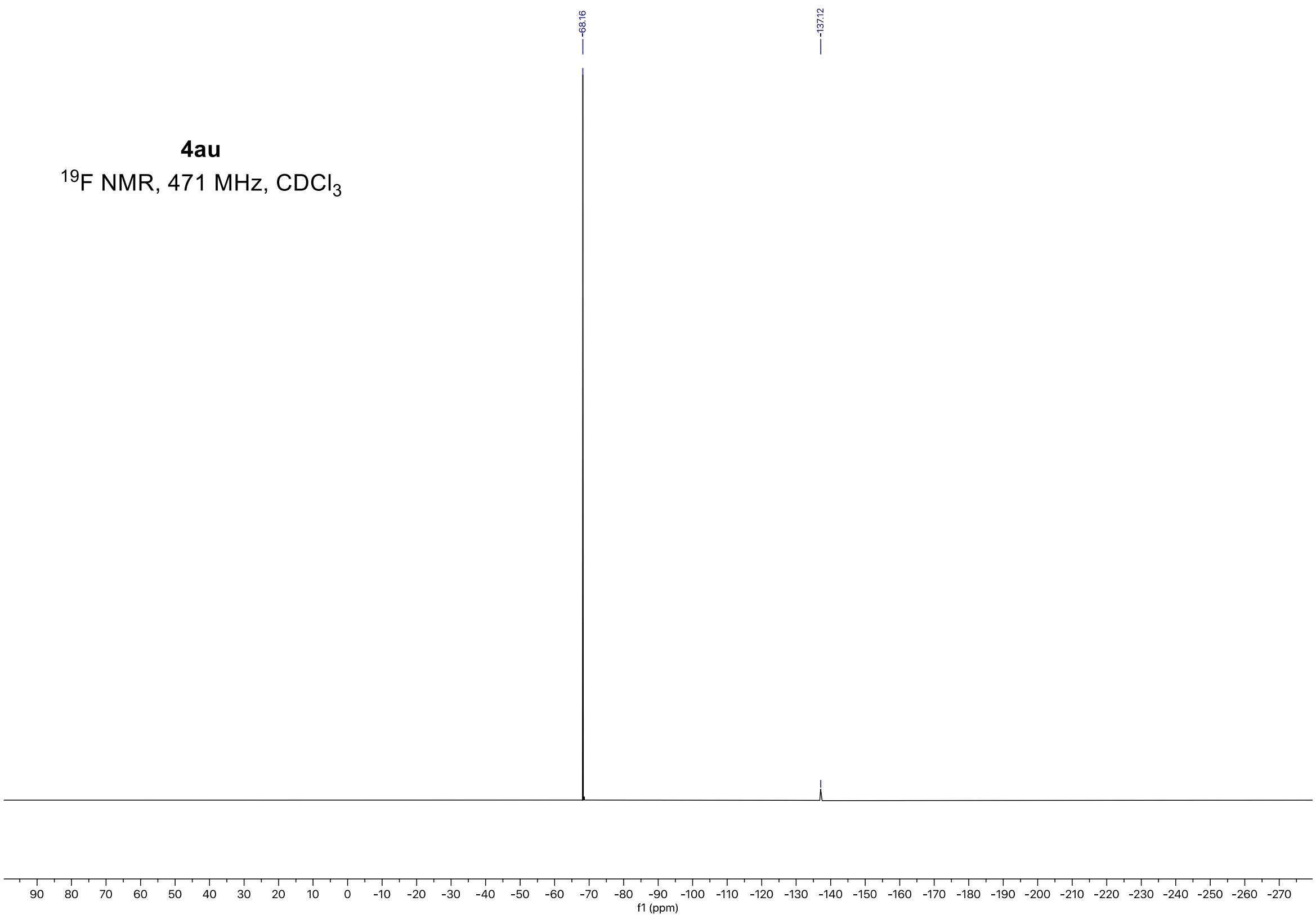

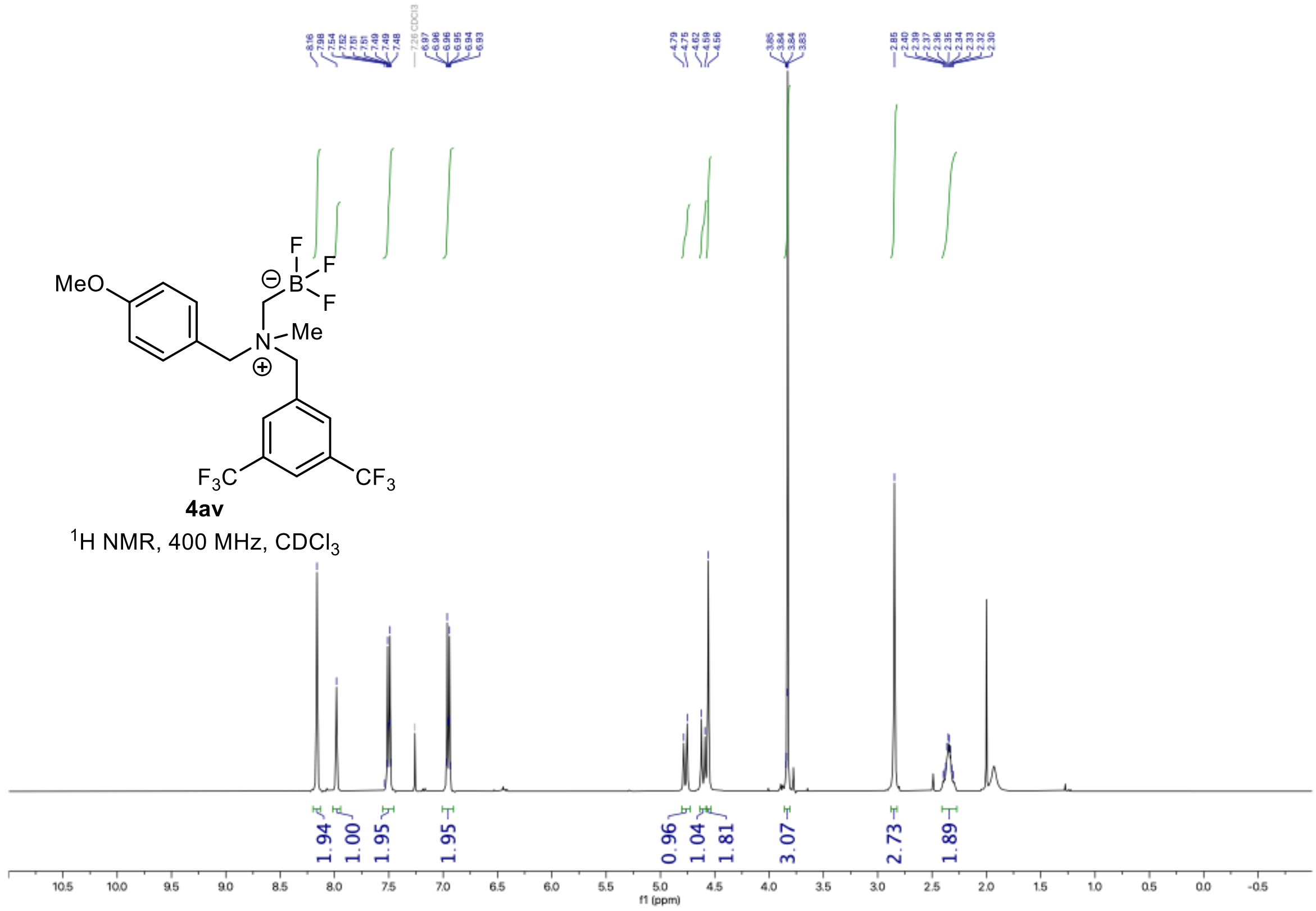
MeO
F
B
F
F
N
Me
F3C
CF3
4av
1H NMR, 400 MHz, CDCl3
f1 (ppm)

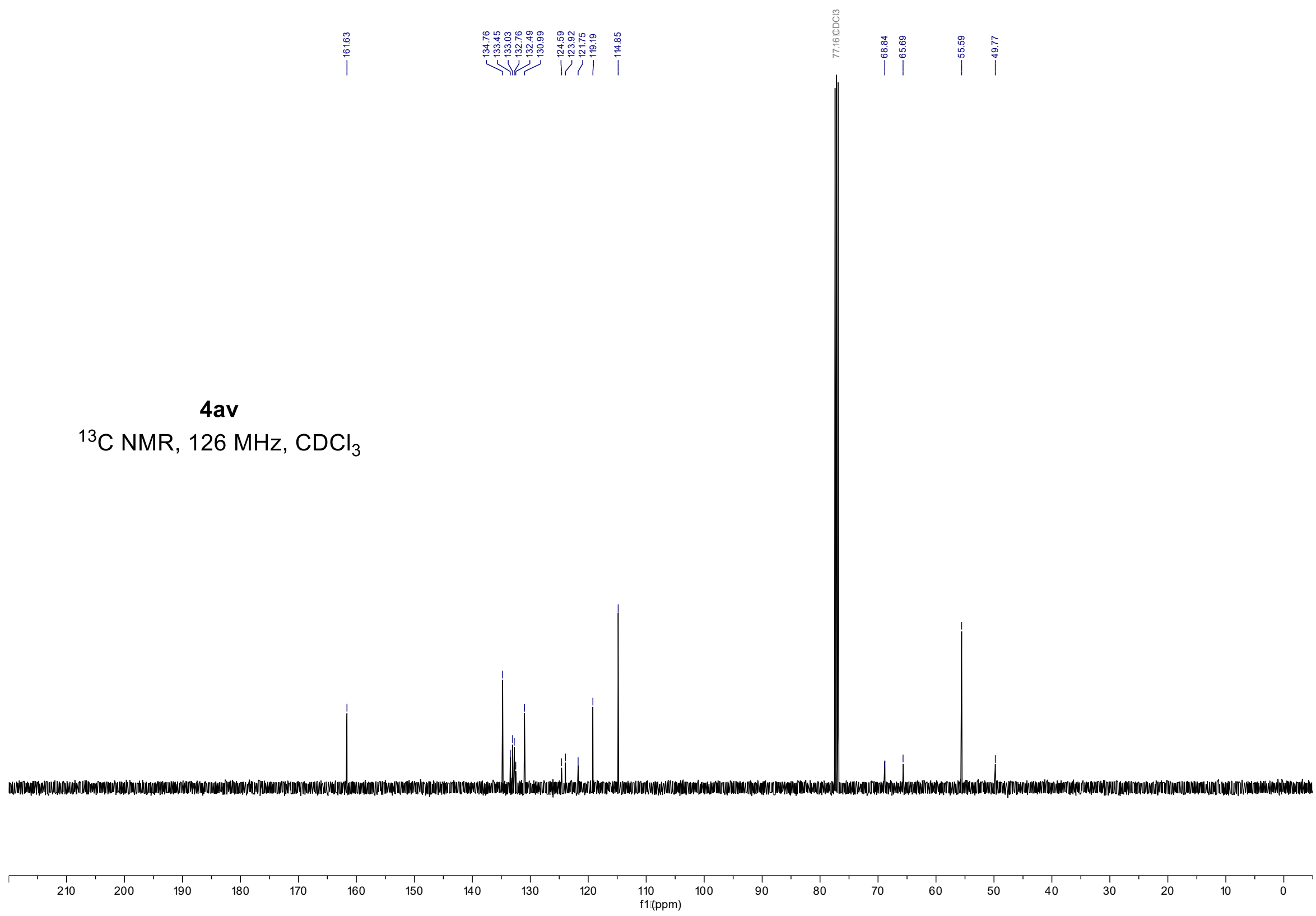
4av
13C NMR, 126 MHz, CDCl3
f1 (ppm)

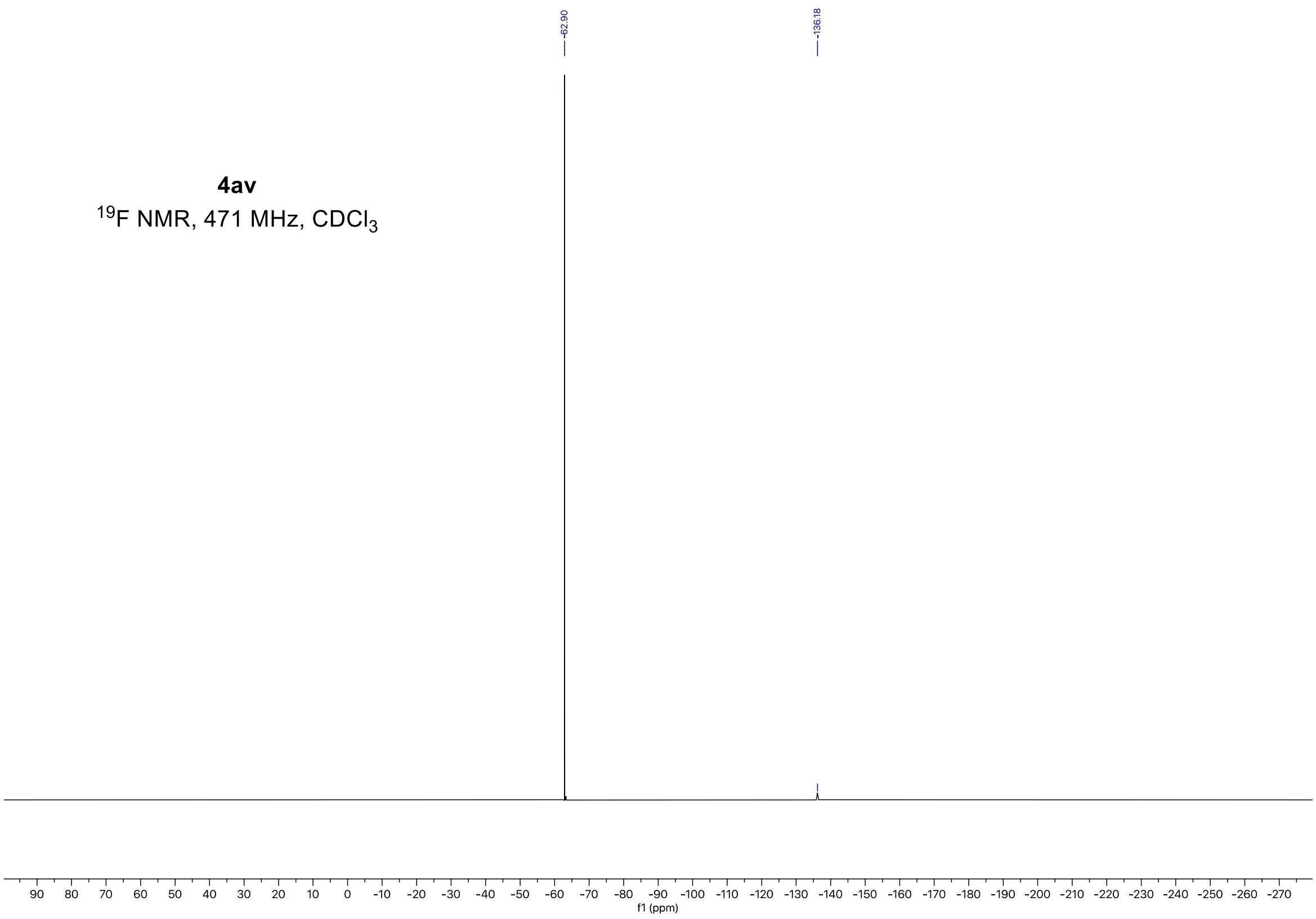
-62.90
-136.18
4av
^{19}F NMR, 471 MHz, $CDCl_3$
90 80 70 60 50 40 30 20 10 0 -10 -20 -30 -40 -50 -60 -70 -80 -90 -100 -110 -120 -130 -140 -150 -160 -170 -180 -190 -200 -210 -220 -230 -240 -250 -260 -270
f1 (ppm)

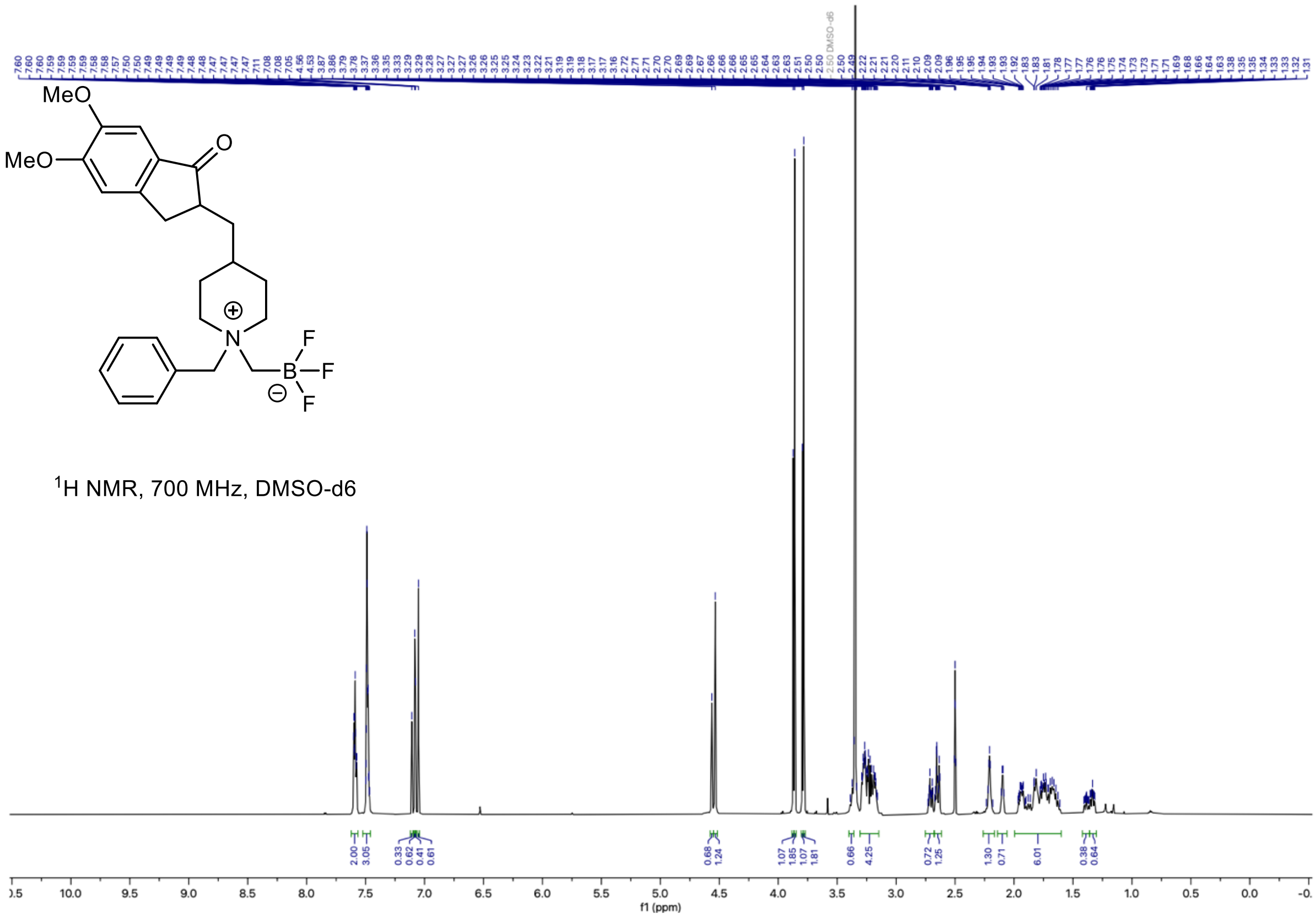
MeO
MeO
O
N
B
F
F
F
1H NMR, 700 MHz, DMSO-d6
f1 (ppm)

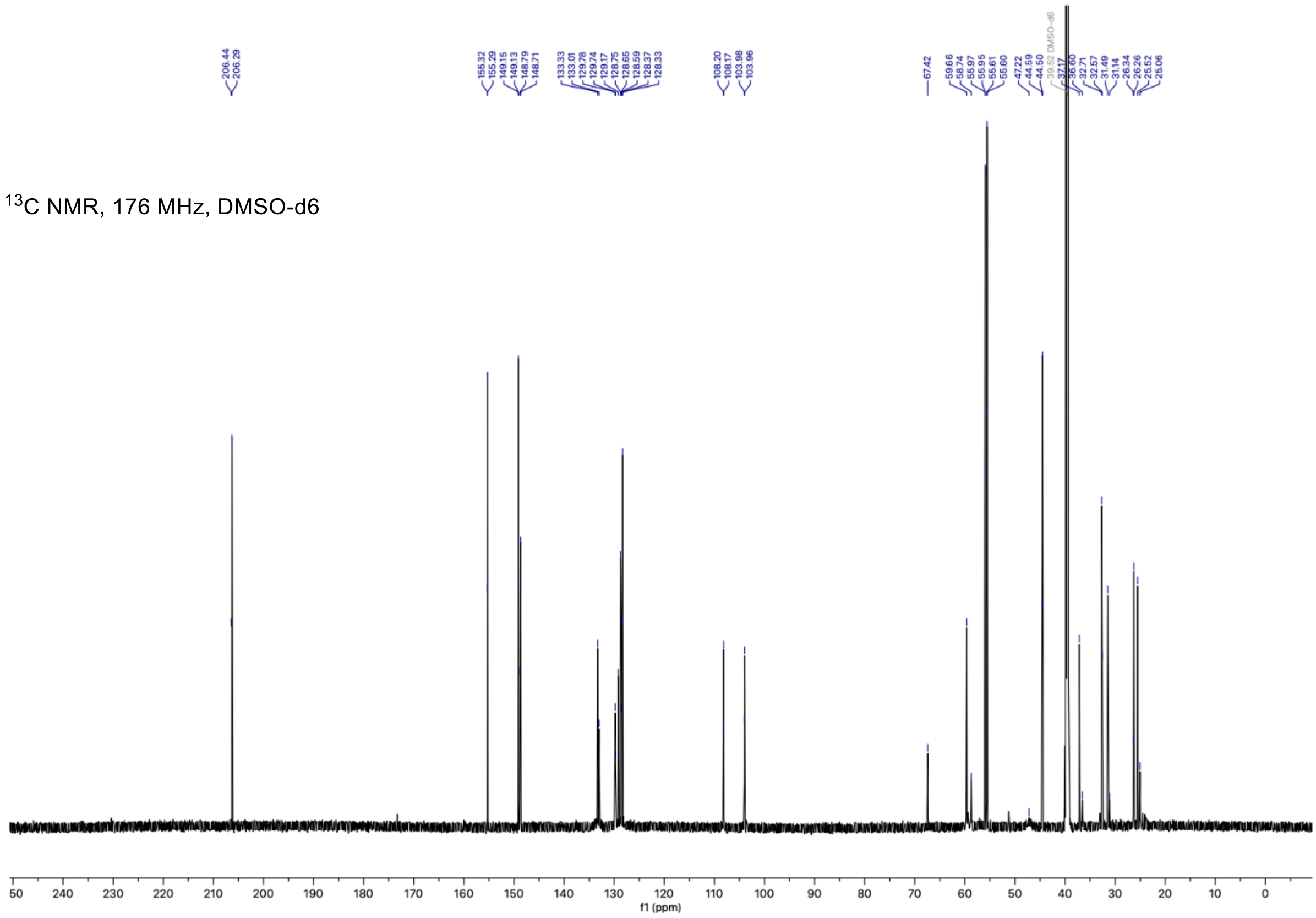
13C NMR, 176 MHz, DMSO-d6
f1 (ppm)

^{19}F NMR, 376 MHz, DMSO-d6

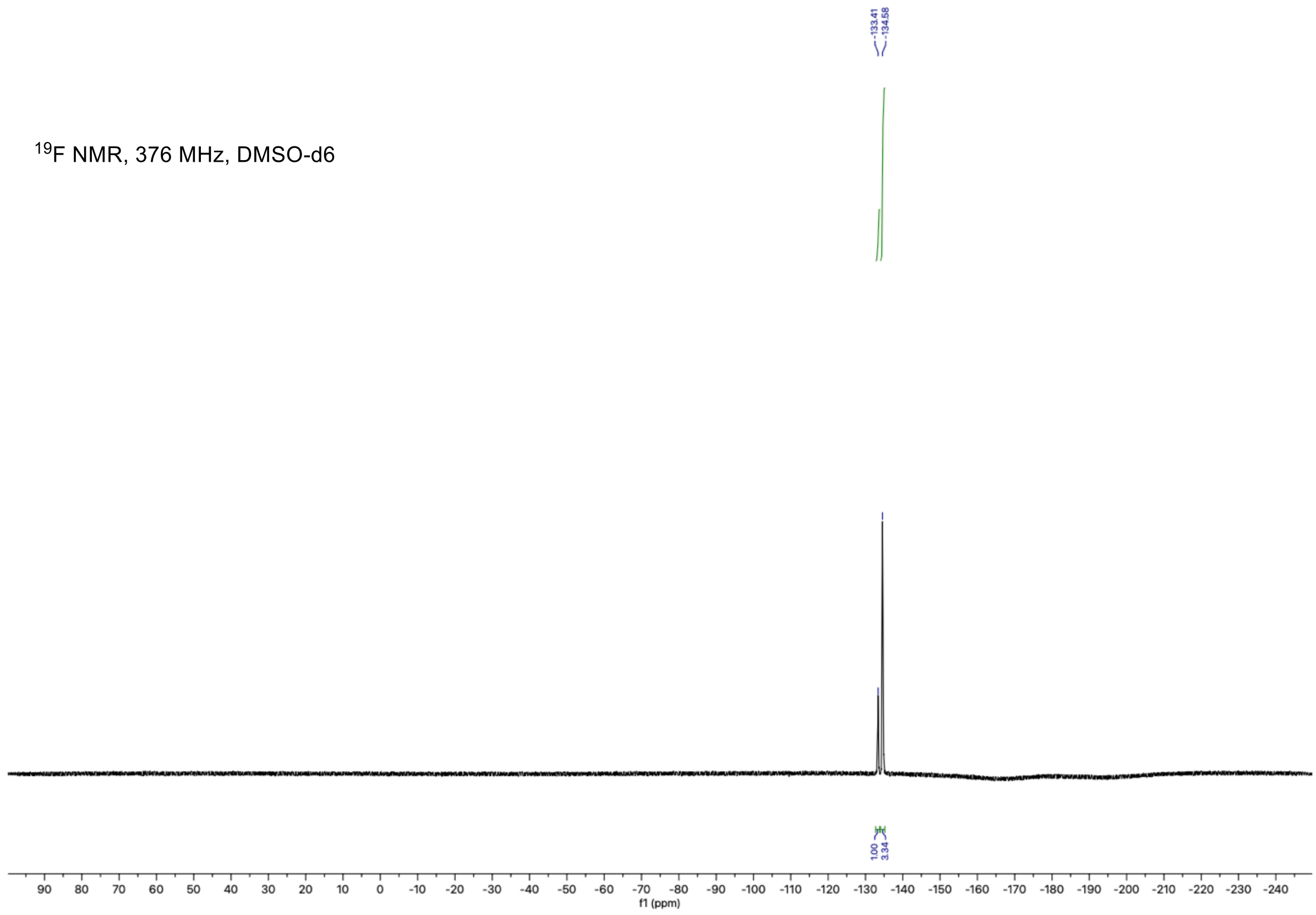

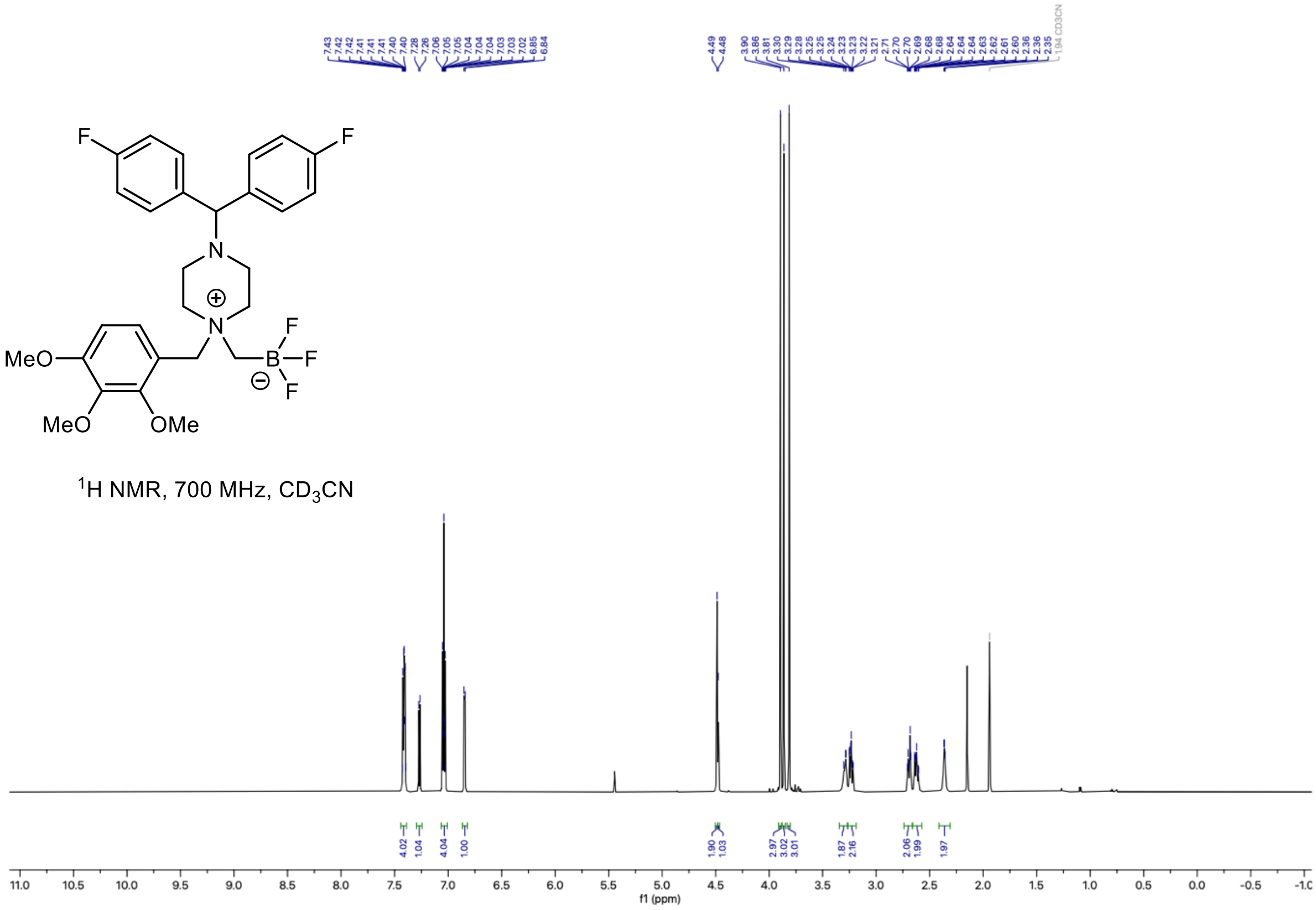

F
F
N
N
MeO
MeO
OMe
B
F
F
F
1H NMR, 700 MHz, CD3CN
7.43 7.42 7.41 7.41 7.41 7.40 7.40 7.28 7.26 7.06 7.05 7.05 7.04 7.04 7.04 7.03 7.03 7.03 7.02 6.85 6.84
4.49 4.48
3.90 3.86 3.81 3.30 3.29 3.28 3.25 3.25 3.24 3.23 3.23 3.22 3.21 2.71 2.70 2.69 2.68 2.68 2.64 2.64 2.64 2.63 2.62 2.61 2.60 2.36 2.35
1.94 CD3CN
4.02 1.04 4.04 1.00
1.90 1.03
2.97 3.02 3.01
1.87 2.16
2.06 1.99
1.97
11.0 10.5 10.0 9.5 9.0 8.5 8.0 7.5 7.0 6.5 6.0 5.5 5.0 4.5 4.0 3.5 3.0 2.5 2.0 1.5 1.0 0.5 0.0 -0.5 -1.0
f1 (ppm)

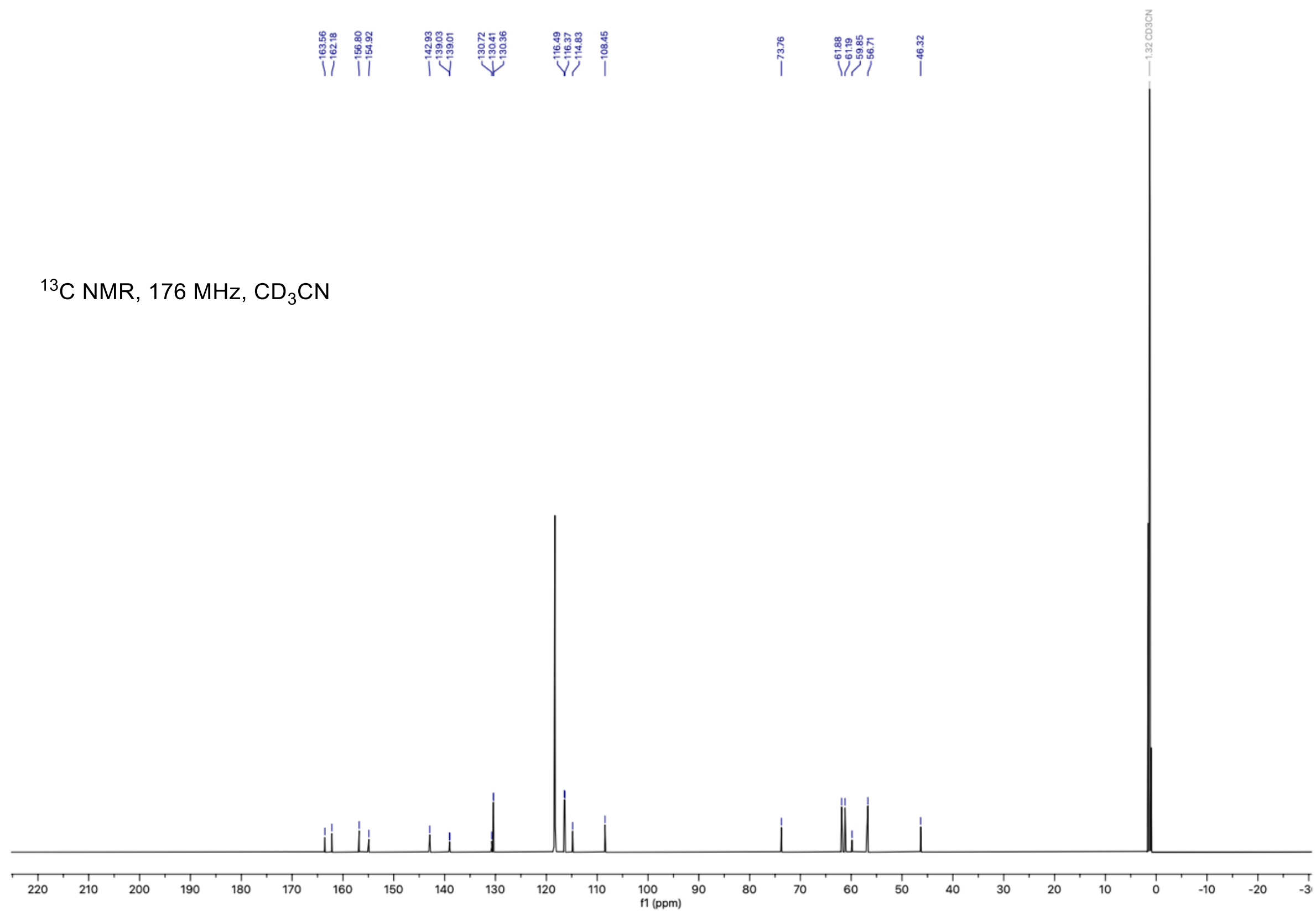

163.56 162.18 156.80 154.92
142.93 139.03 139.01
130.72 130.41 130.36
116.49 116.37 114.83
108.45
73.76
61.88 61.19 59.85 56.71
46.32
1.32 CD3CN
13C NMR, 176 MHz, CD3CN
220 210 200 190 180 170 160 150 140 130 120 110 100 90 80 70 60 50 40 30 20 10 0 -10 -20
f1 (ppm)

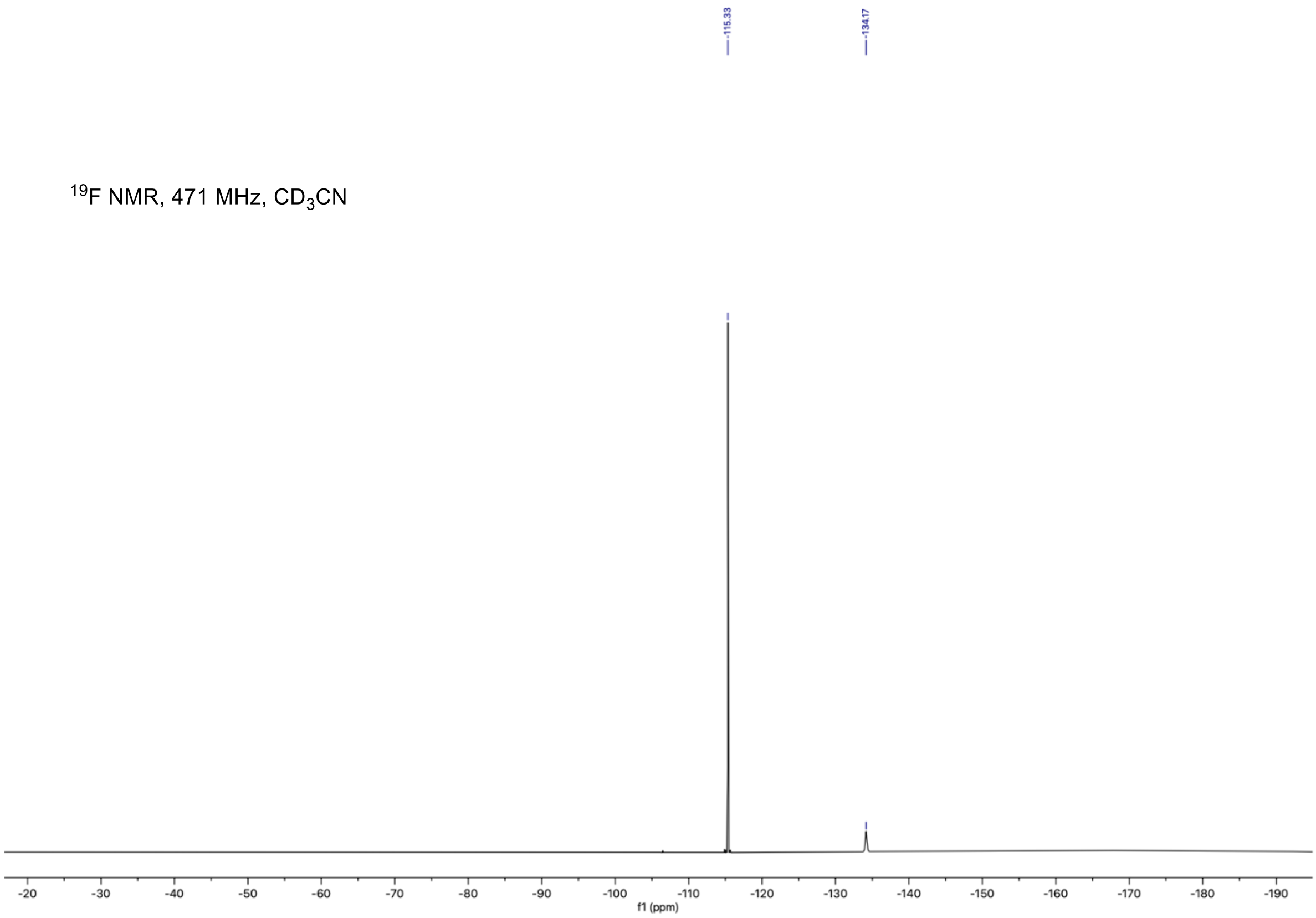

-115.33
-134.17
^{19}F NMR, 471 MHz, CD_3CN
-20
-30
-40
-50
-60
-70
-80
-90
-100
-110
-120
-130
-140
-150
-160
-170
-180
-190
f1 (ppm)

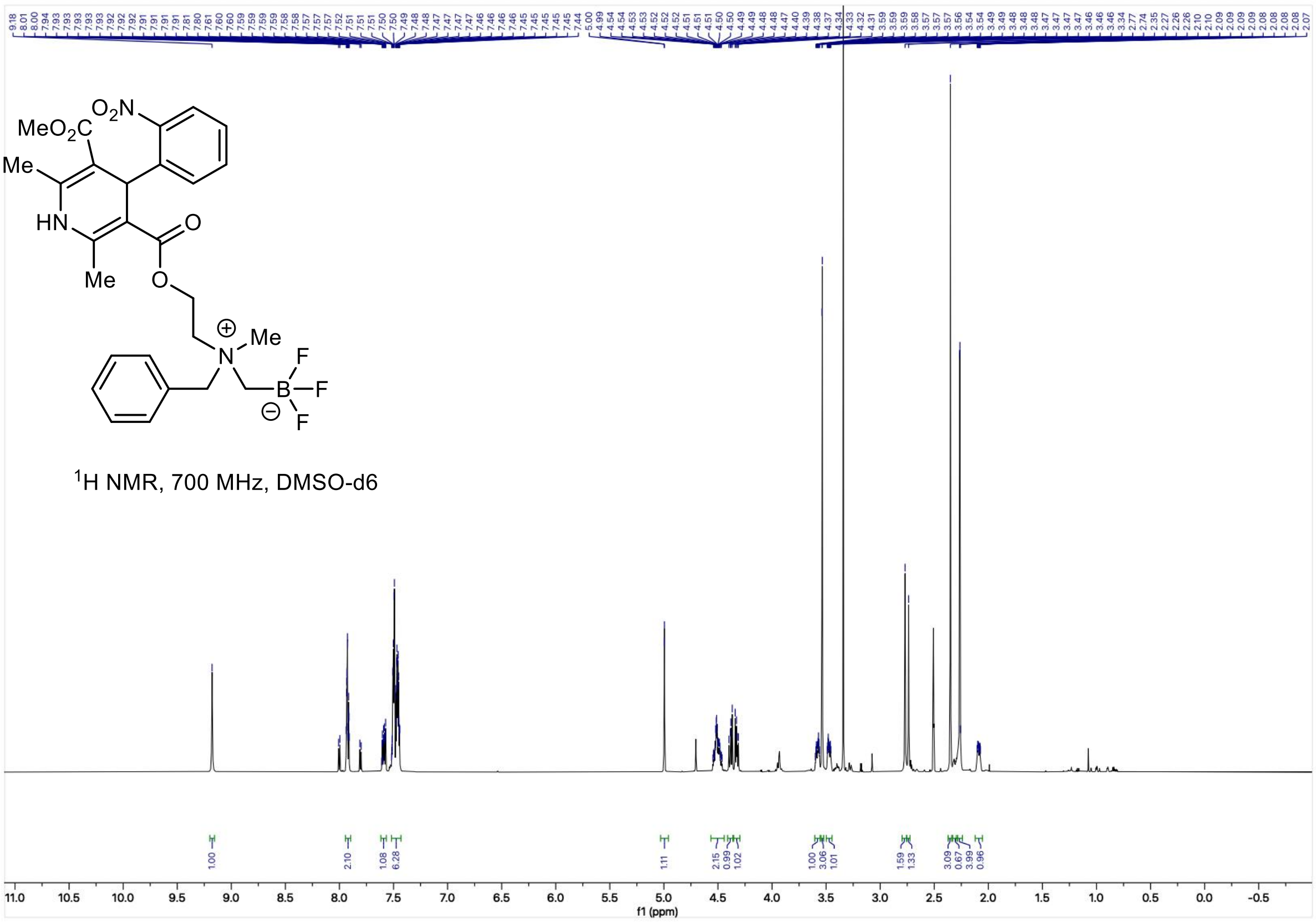

^{1}H NMR, 700 MHz, DMSO-d6
f1 (ppm)

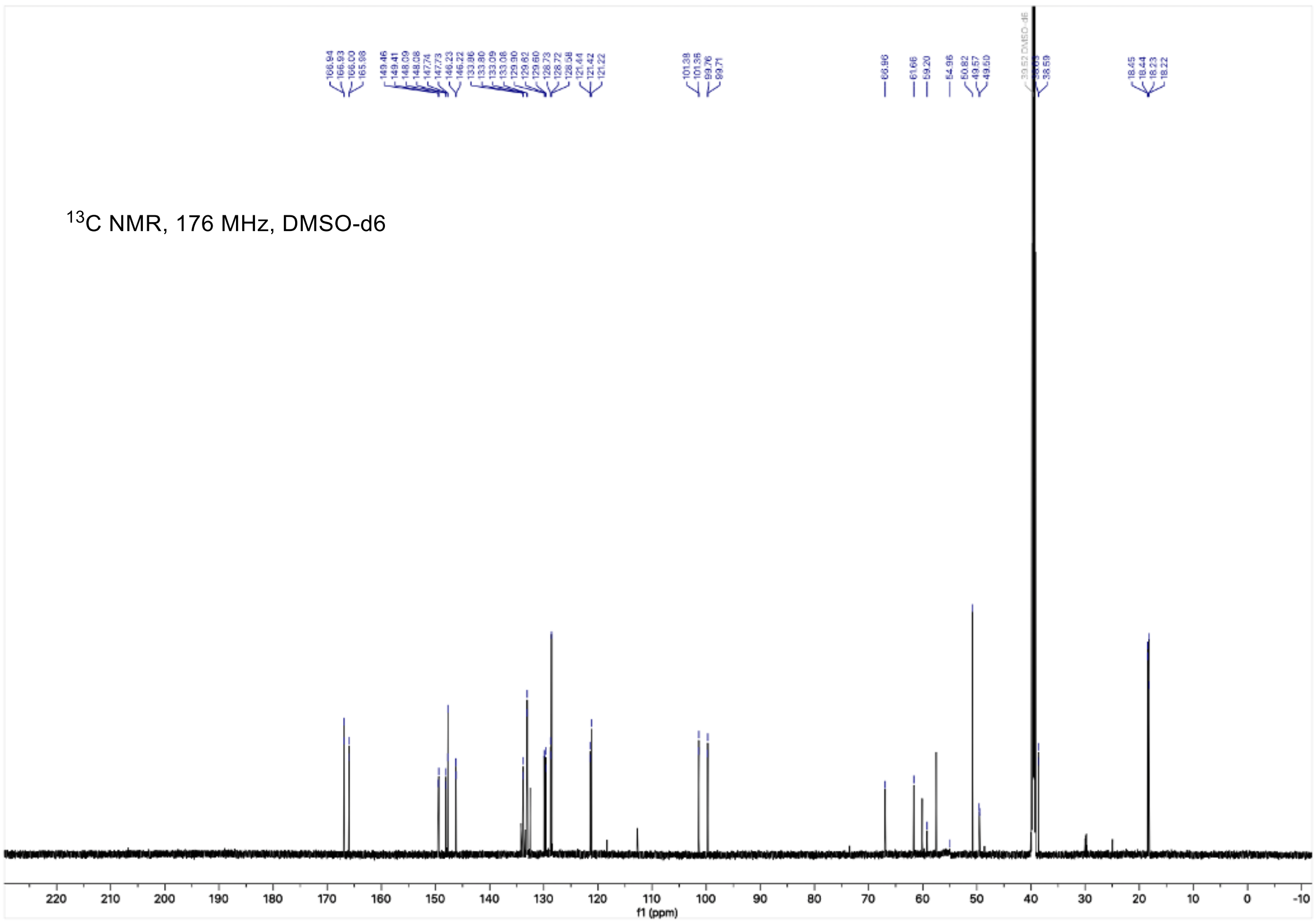

^{13}C NMR, 176 MHz, DMSO-d6
f1 (ppm)

^{19}F NMR, 471 MHz, DMSO-d6

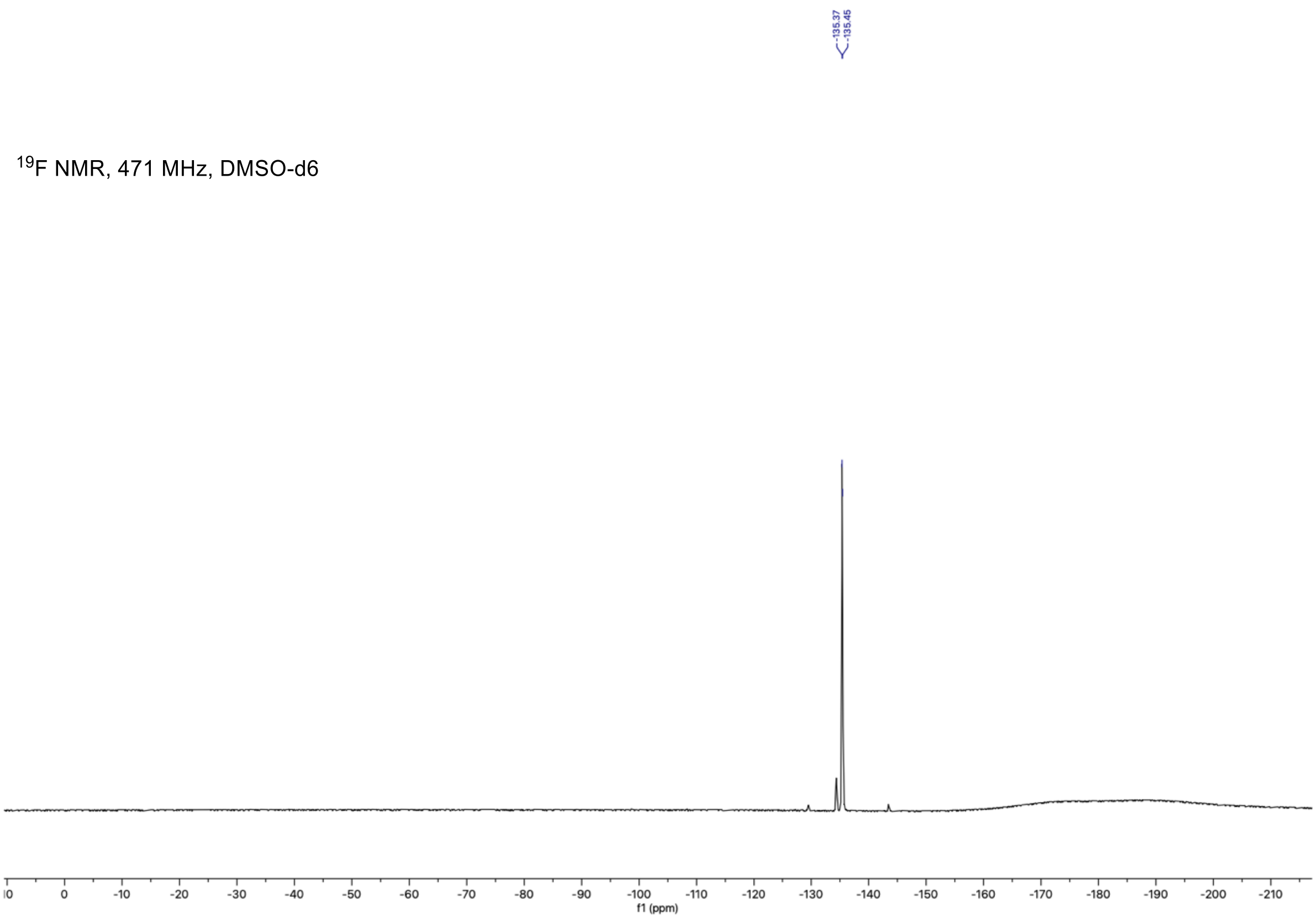

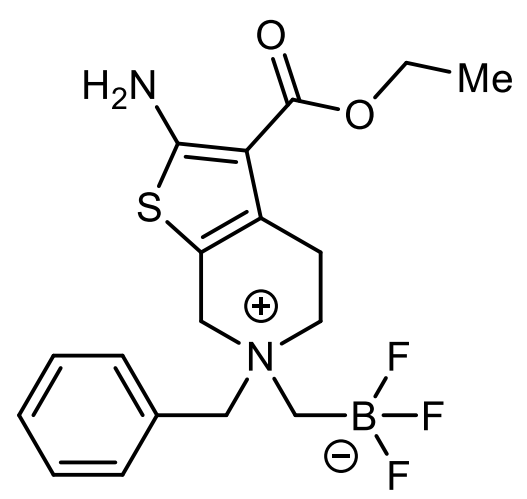

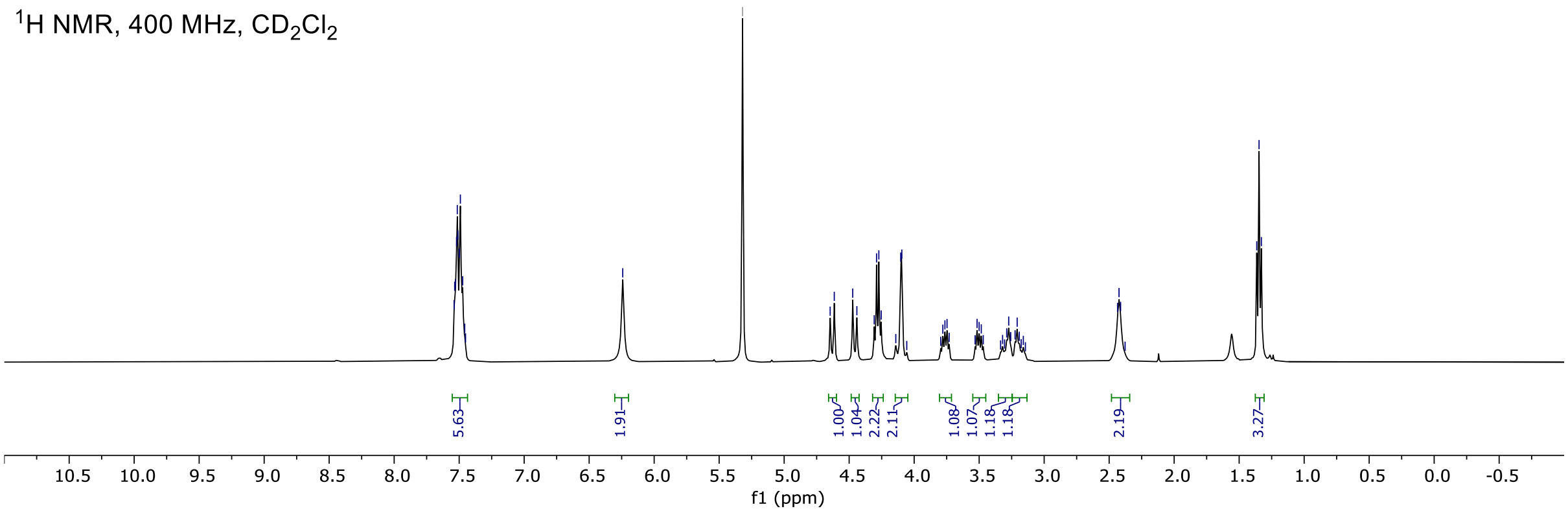

^{1}H NMR, 400 MHz, CD_2Cl_2

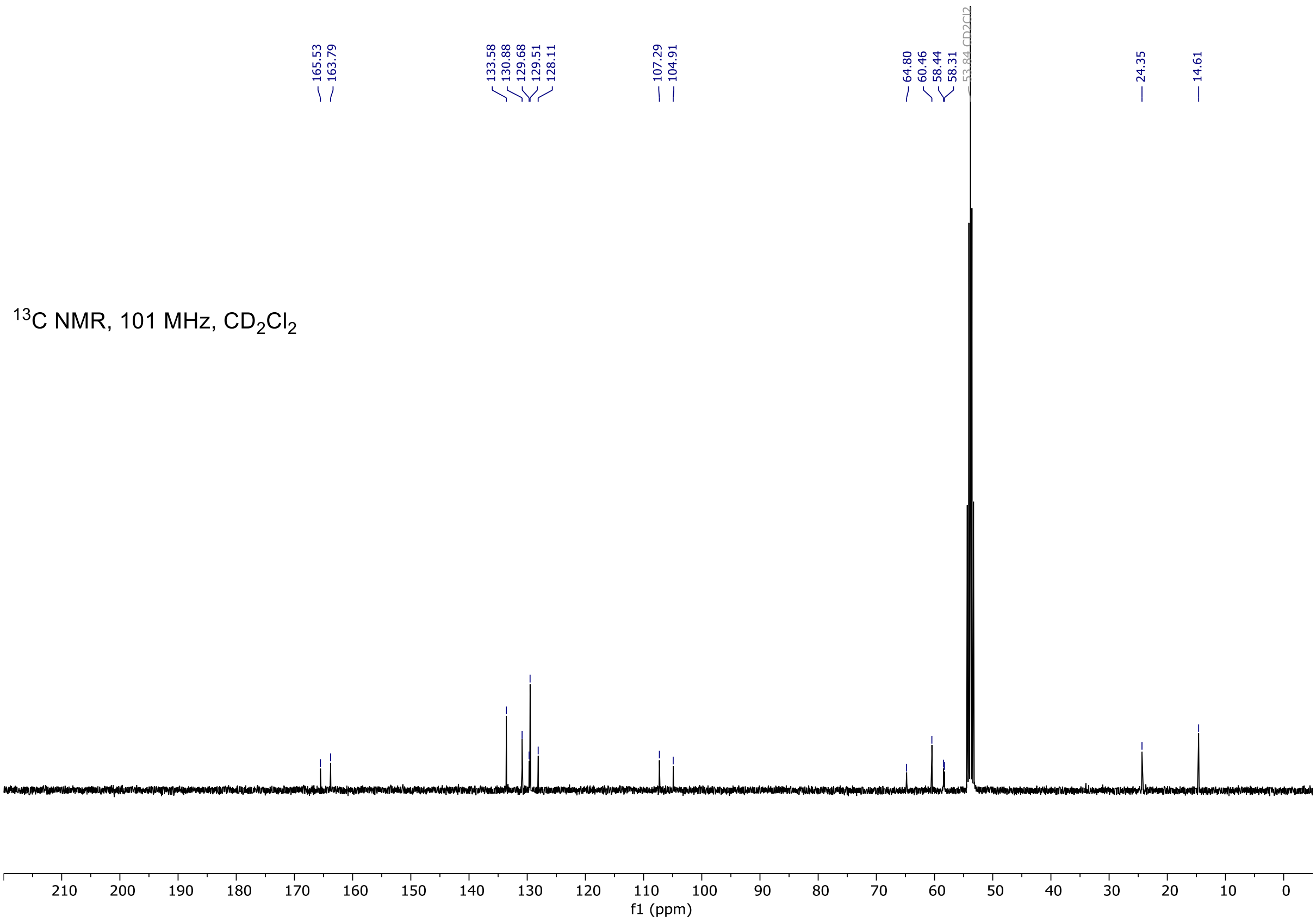

^{13}C NMR, 101 MHz, CD_2Cl_2

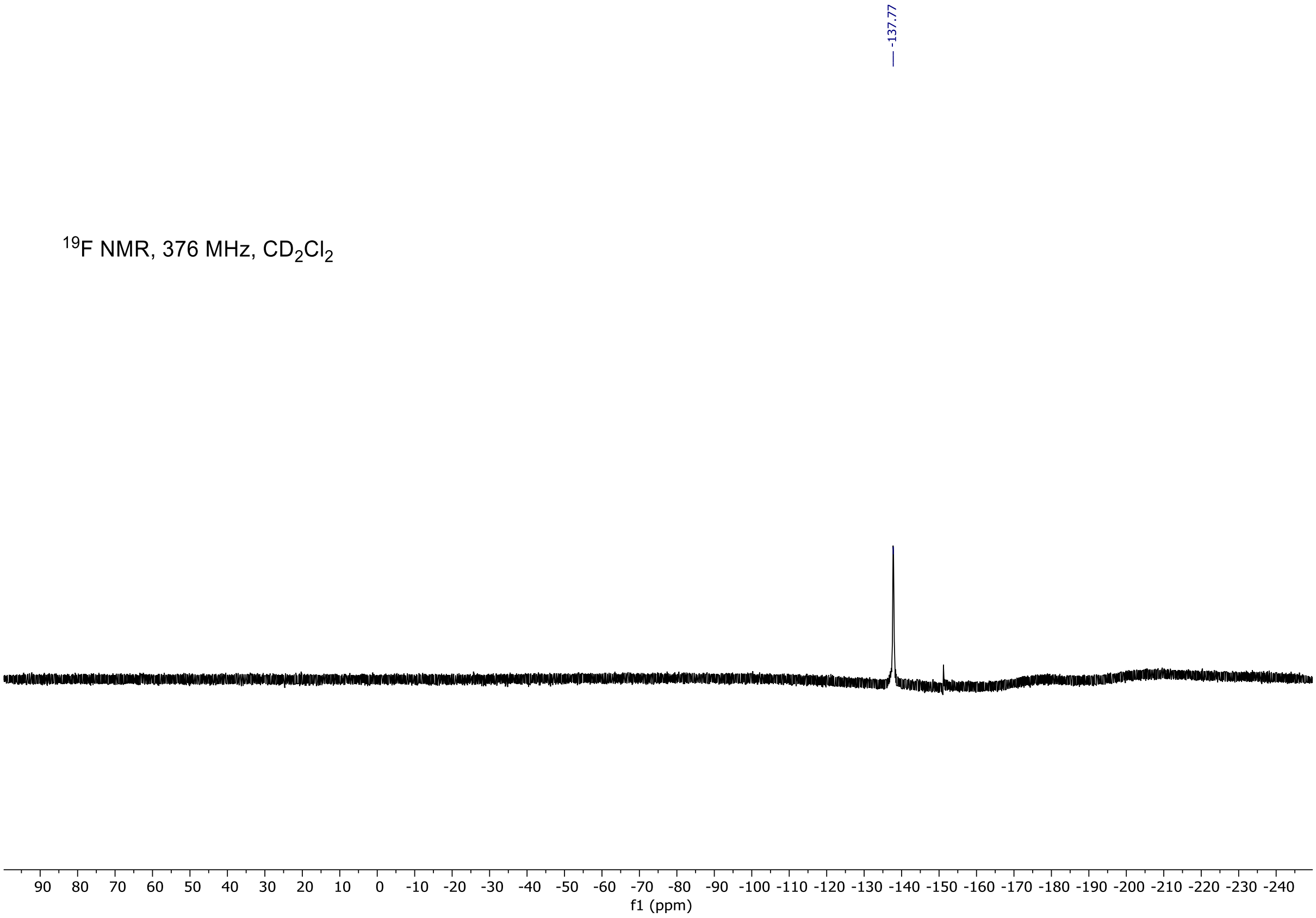

-137.77
^{19}F NMR, 376 MHz, CD_2Cl_2
90 80 70 60 50 40 30 20 10 0 -10 -20 -30 -40 -50 -60 -70 -80 -90 -100 -110 -120 -130 -140 -150 -160 -170 -180 -190 -200 -210 -220 -230 -240
f1 (ppm)

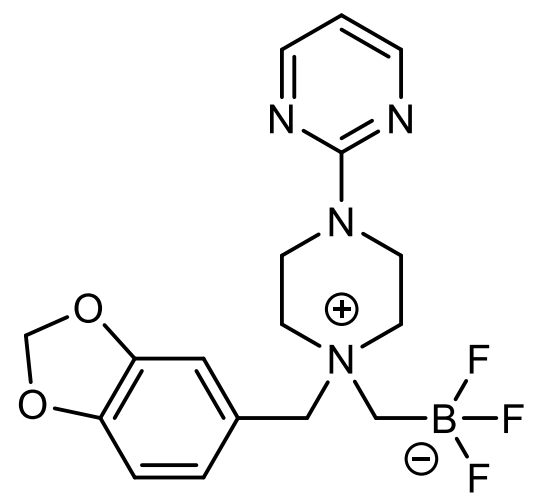

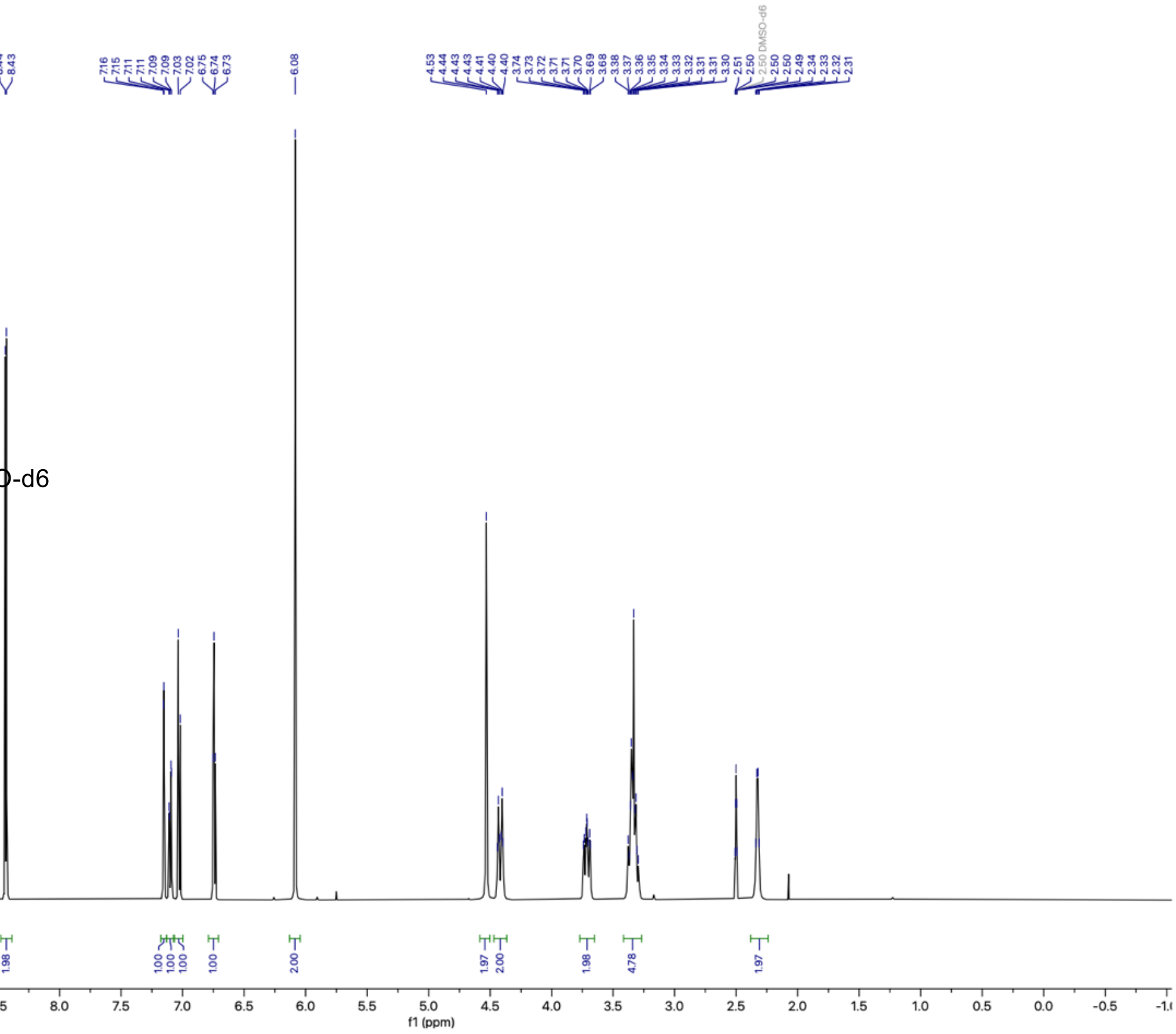

^{1}H NMR, 500 MHz, DMSO-d6

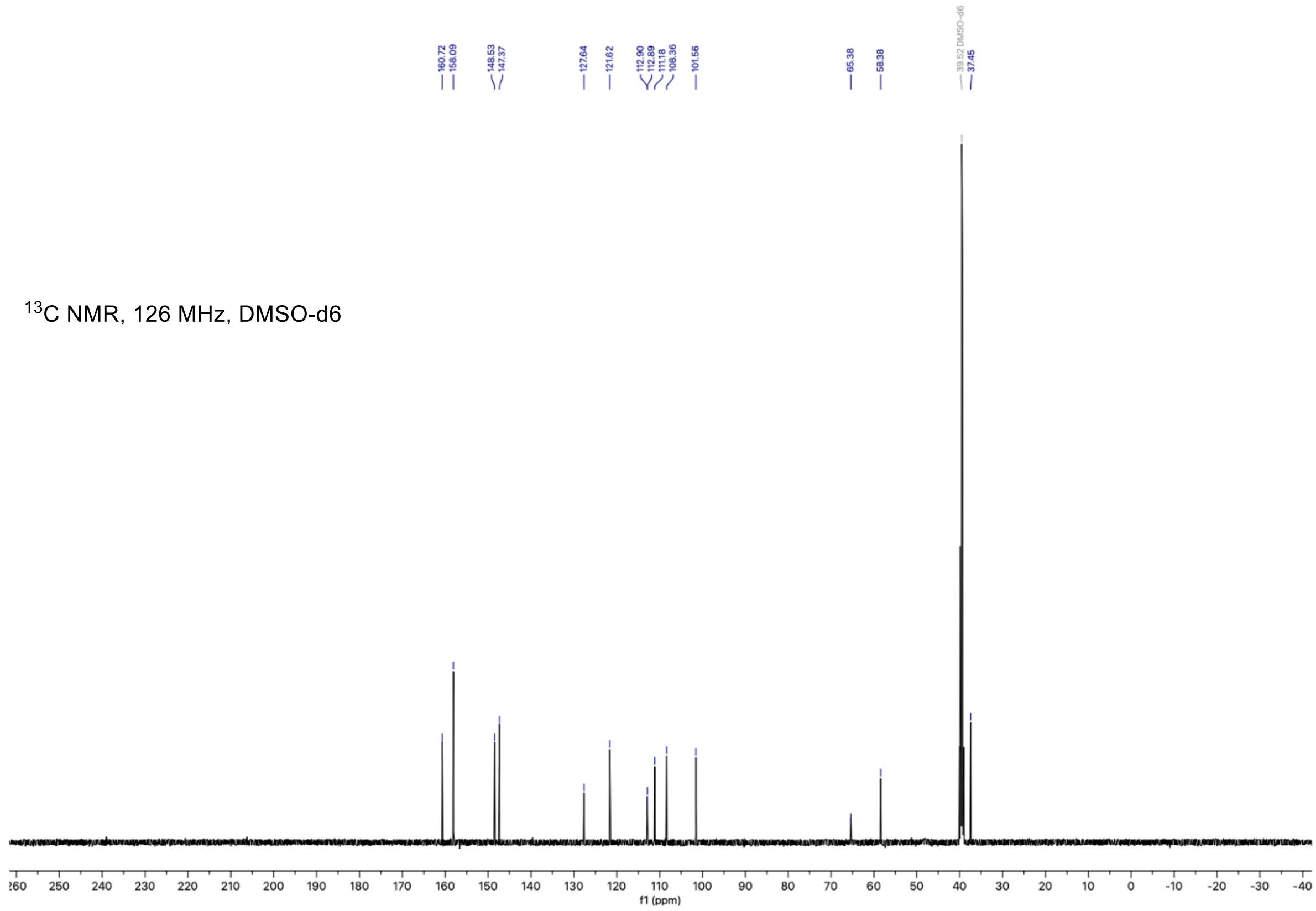

^{13}C NMR, 126 MHz, DMSO-d6

^{19}F NMR, 471 MHz, DMSO-d6

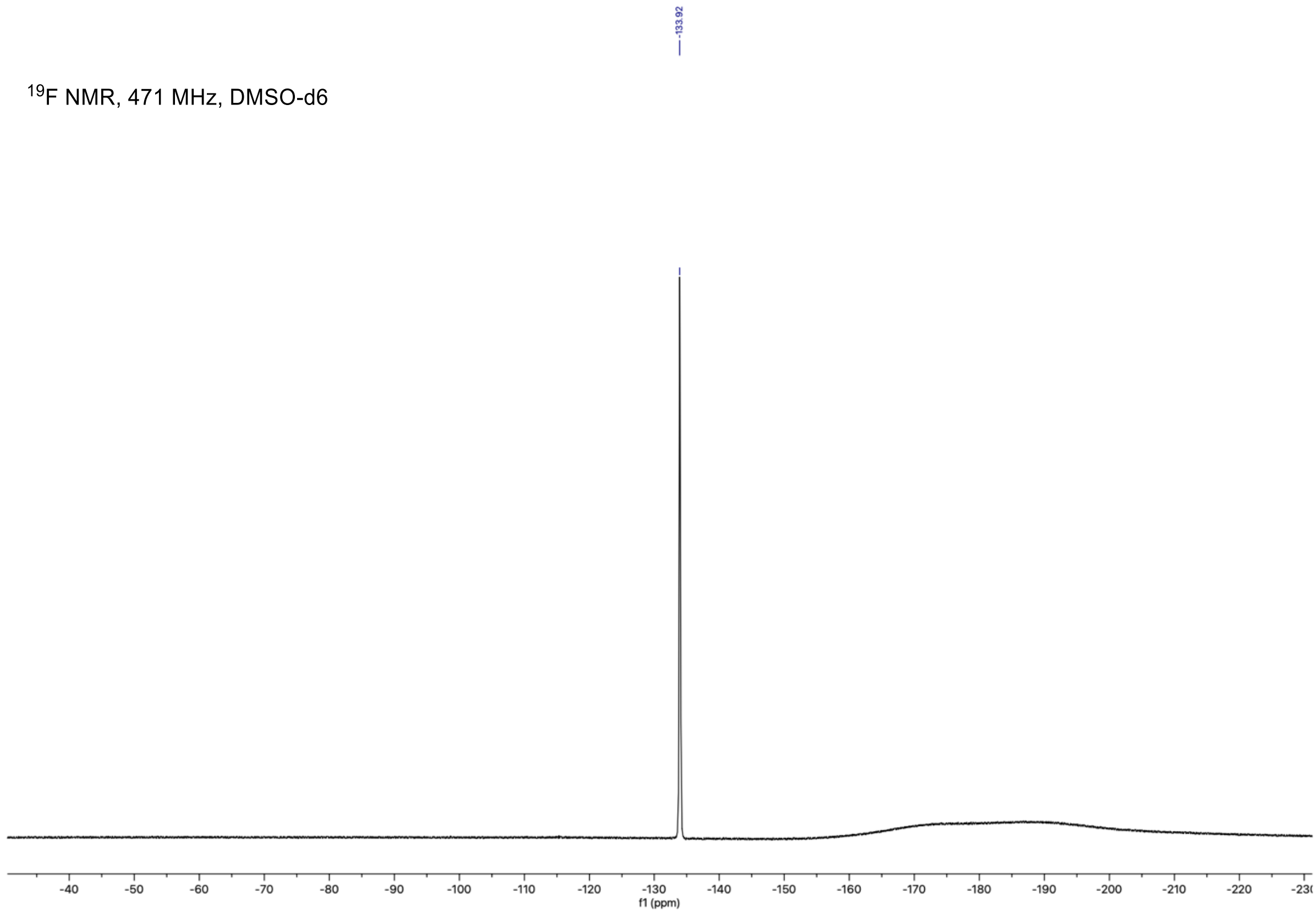

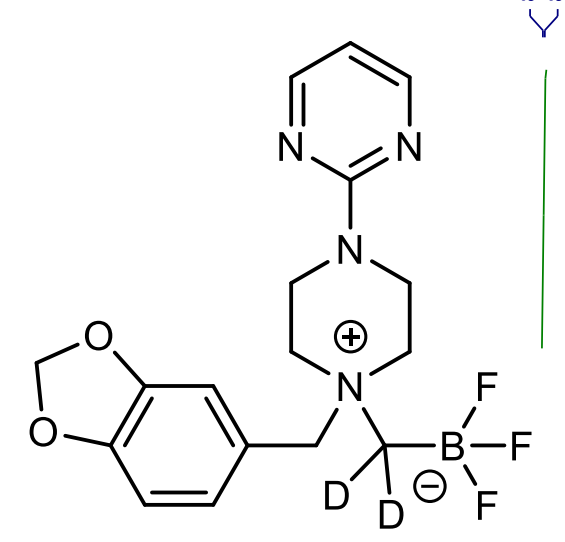

^{1}H NMR, 500 MHz, CD_2Cl_2

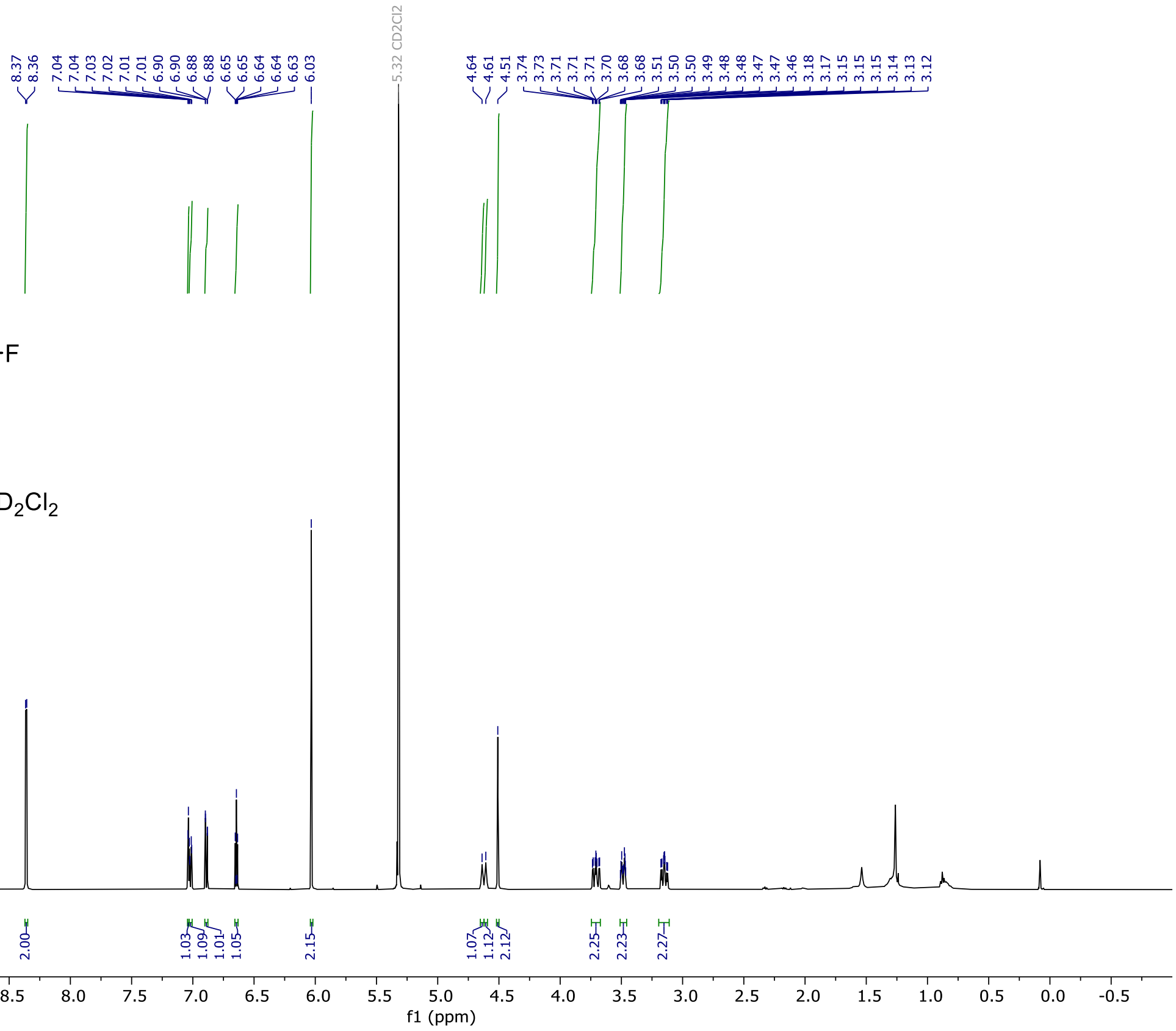

^{13}C NMR, 176 MHz, CD_2Cl_2

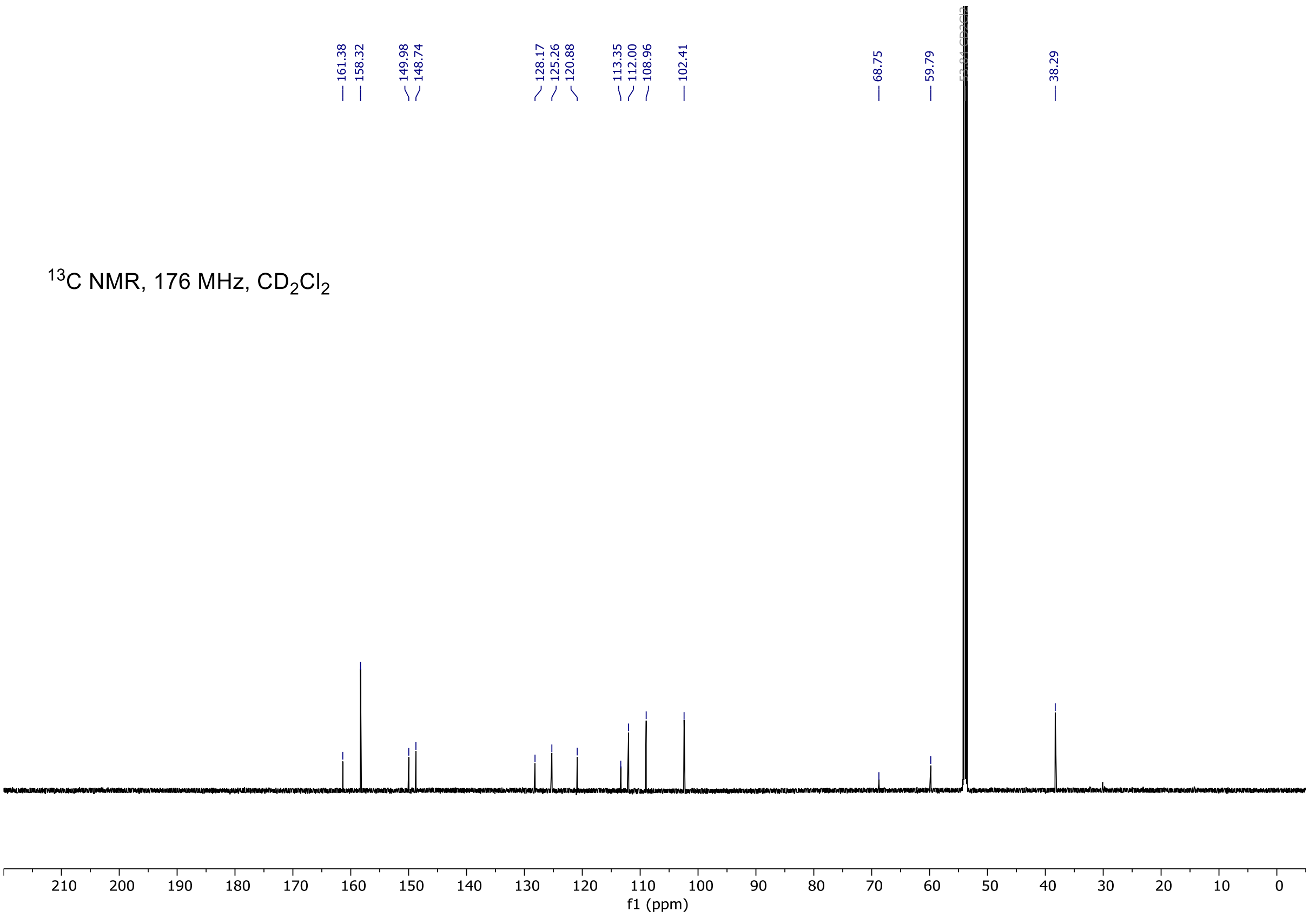

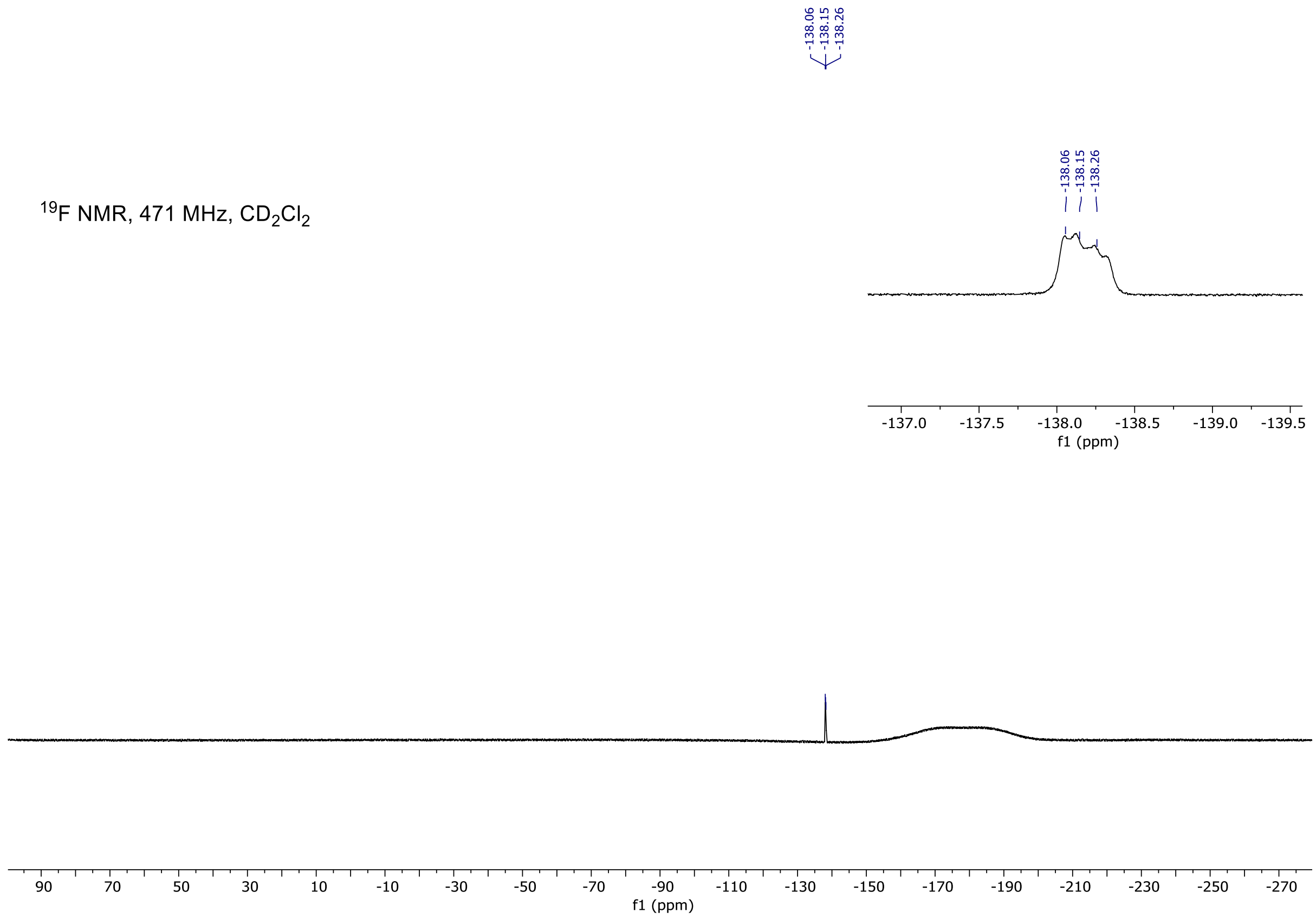
-138.06
-138.15
-138.26
^{19}F NMR, 471 MHz, CD_2Cl_2
-137.0
-137.5
-138.0
-138.5
-139.0
-139.5
f1 (ppm)
90
70
50
30
10
-10
-30
-50
-70
-90
-110
-130
-150
-170
-190
-210
-230
-250
-270

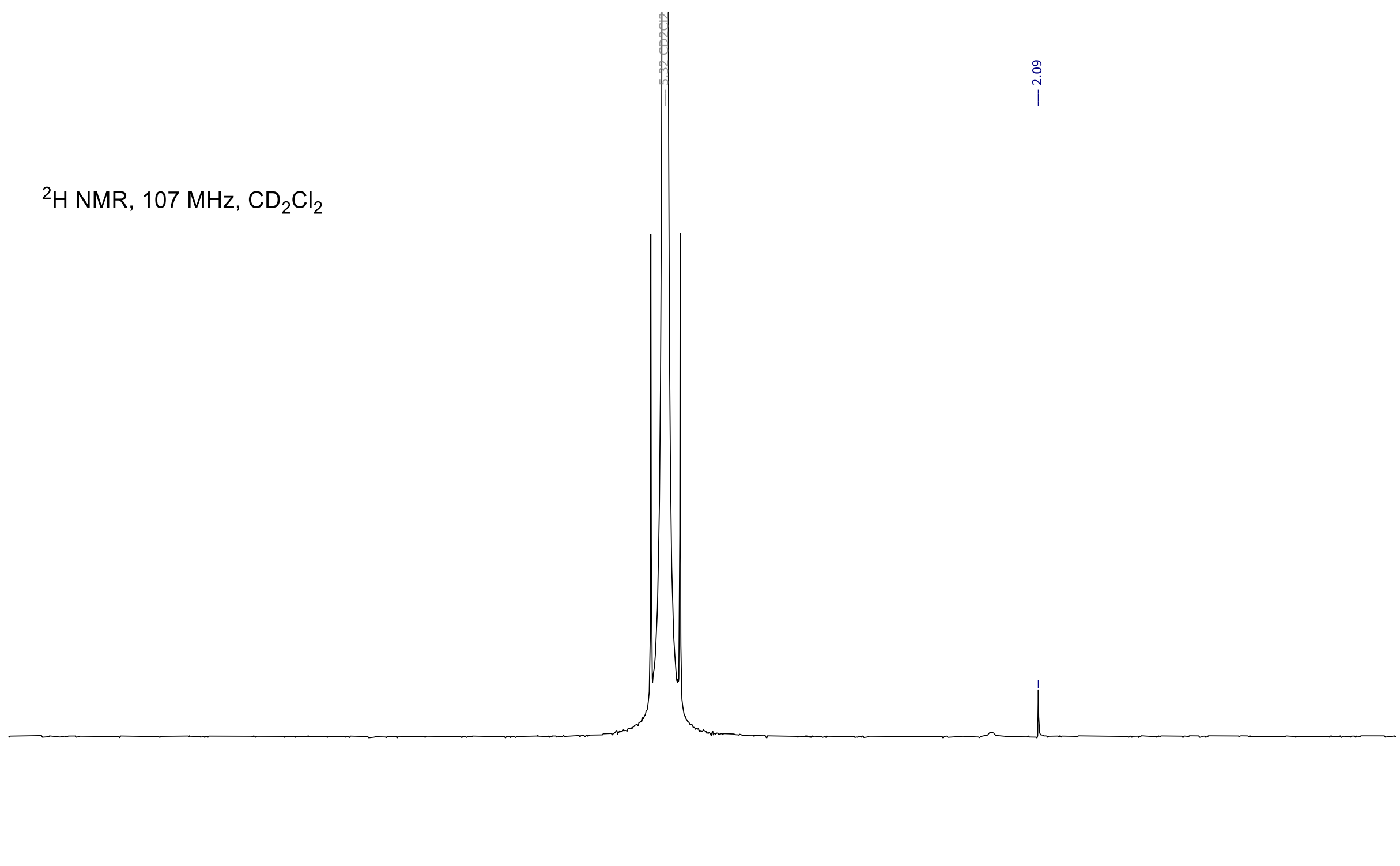
2.09
^{2}H NMR, 107 MHz, CD_2Cl_2
10.5
10.0
9.5
9.0
8.5
8.0
7.5
7.0
6.5
6.0
5.5
5.0
4.5
4.0
3.5
3.0
2.5
2.0
1.5
1.0
0.5
0.0
-0.5
f1 (ppm)

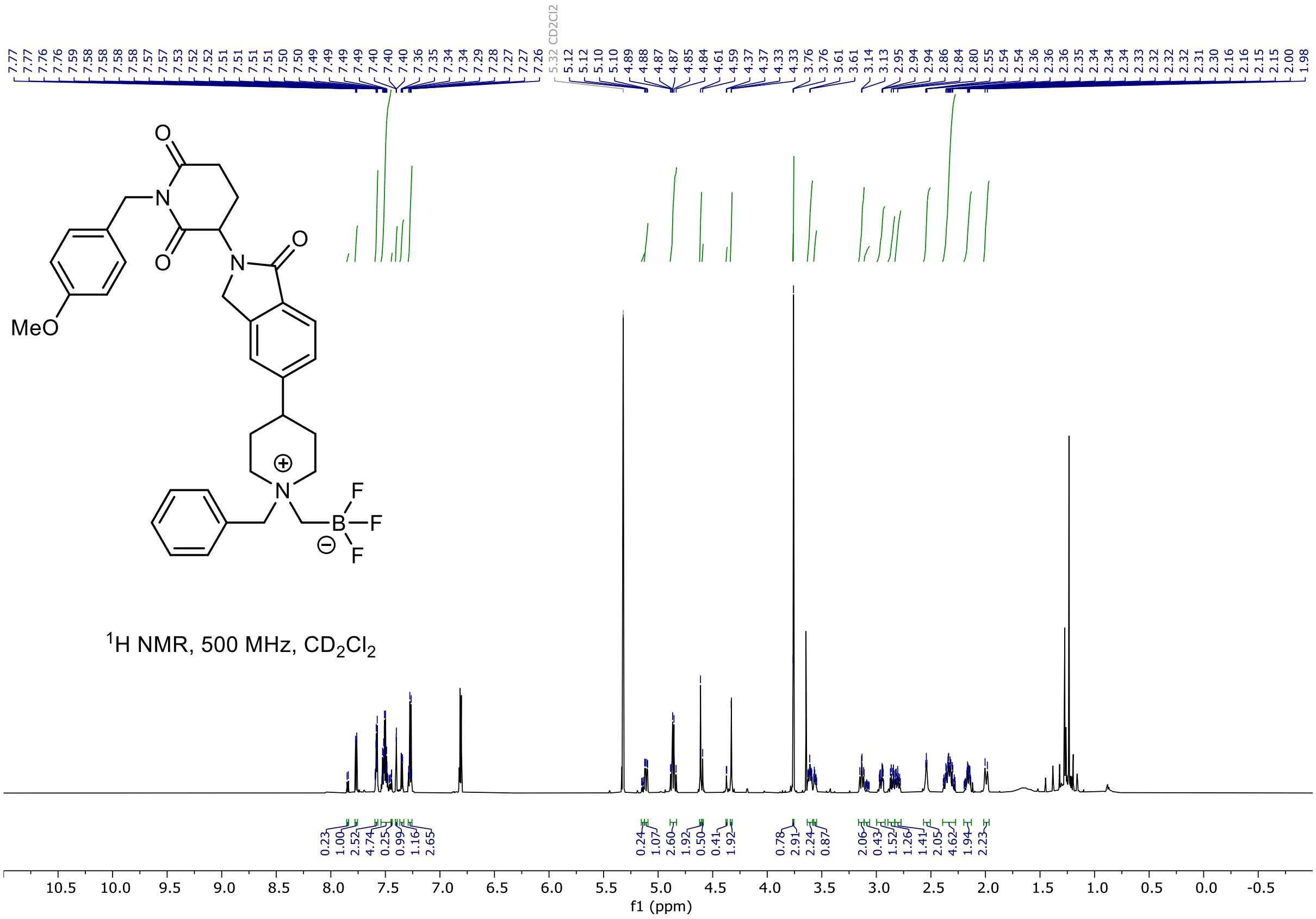
^{1}H NMR, 500 MHz, CD_2Cl_2

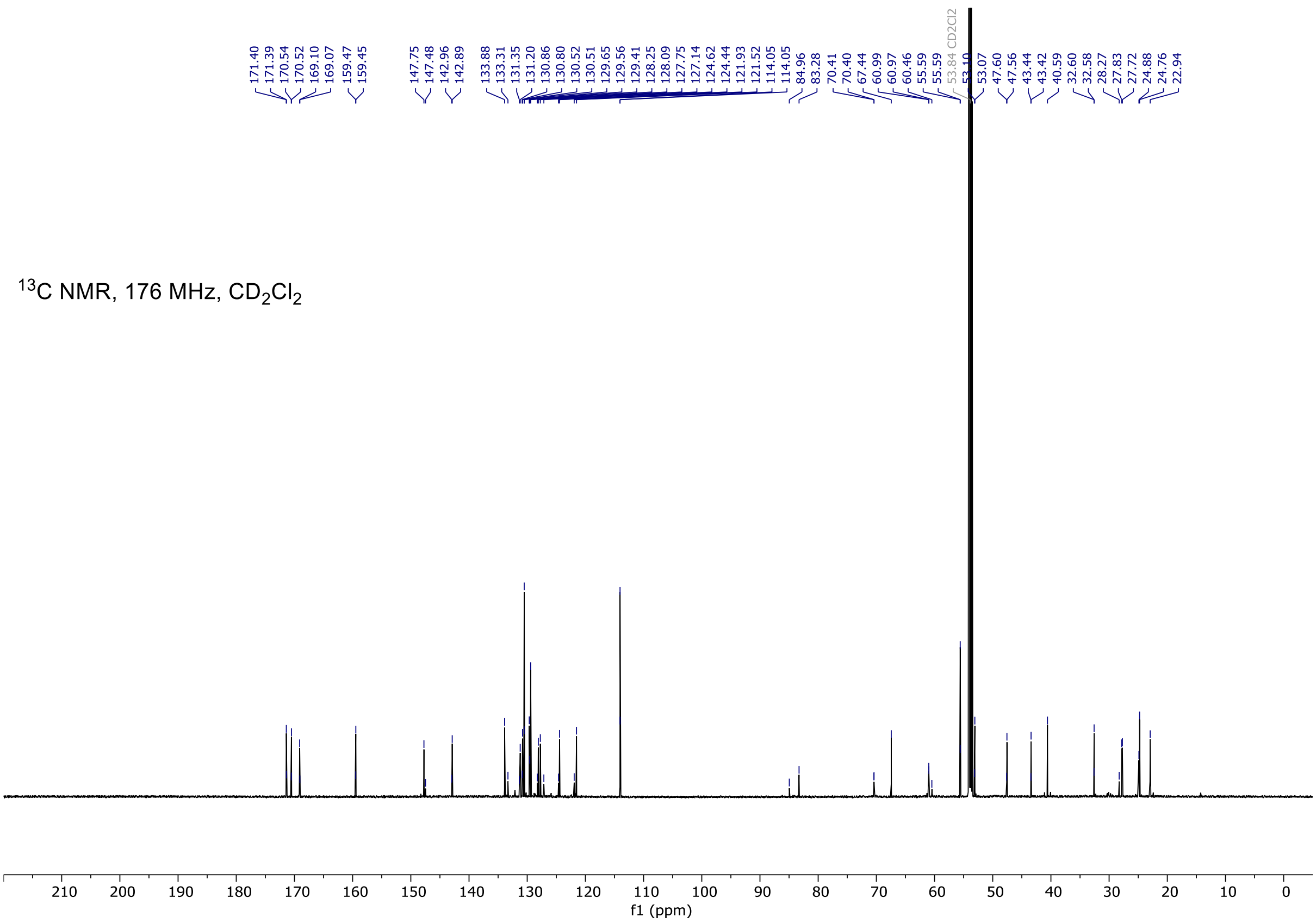
^{13}C NMR, 176 MHz, CD_2Cl_2

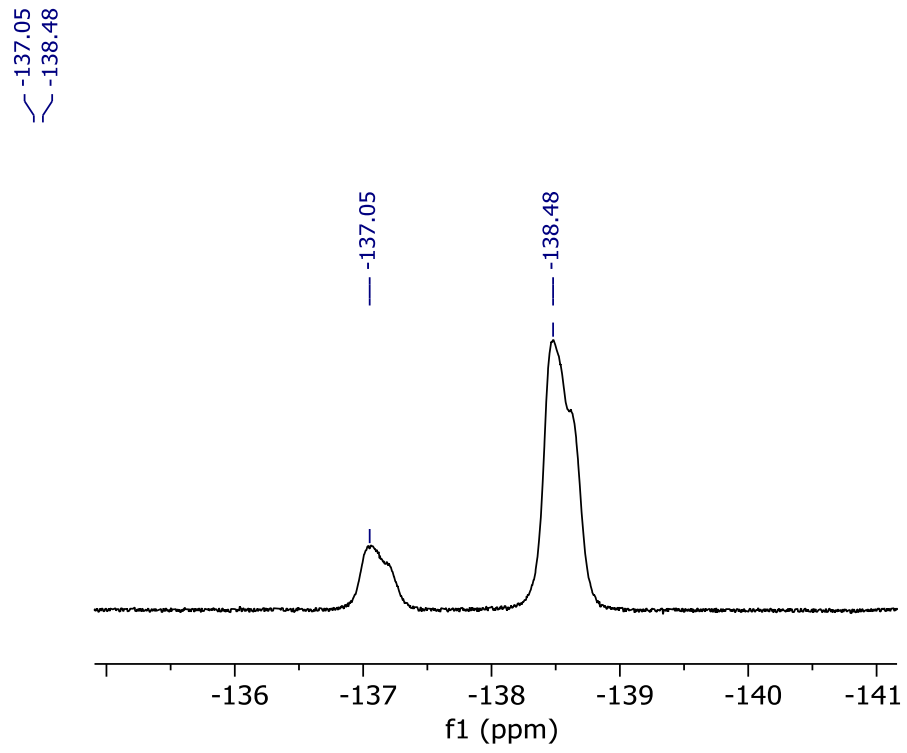

^{19}F NMR, 471 MHz, CD_2Cl_2

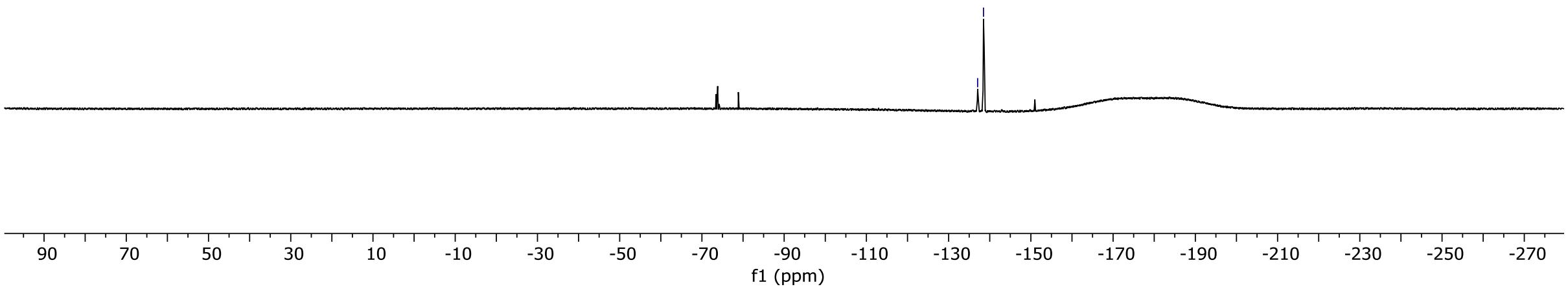

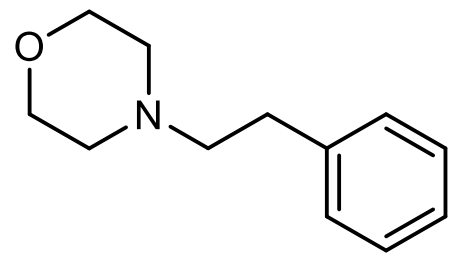

2a

^{1}H NMR, 700 MHz, $CDCl_3$

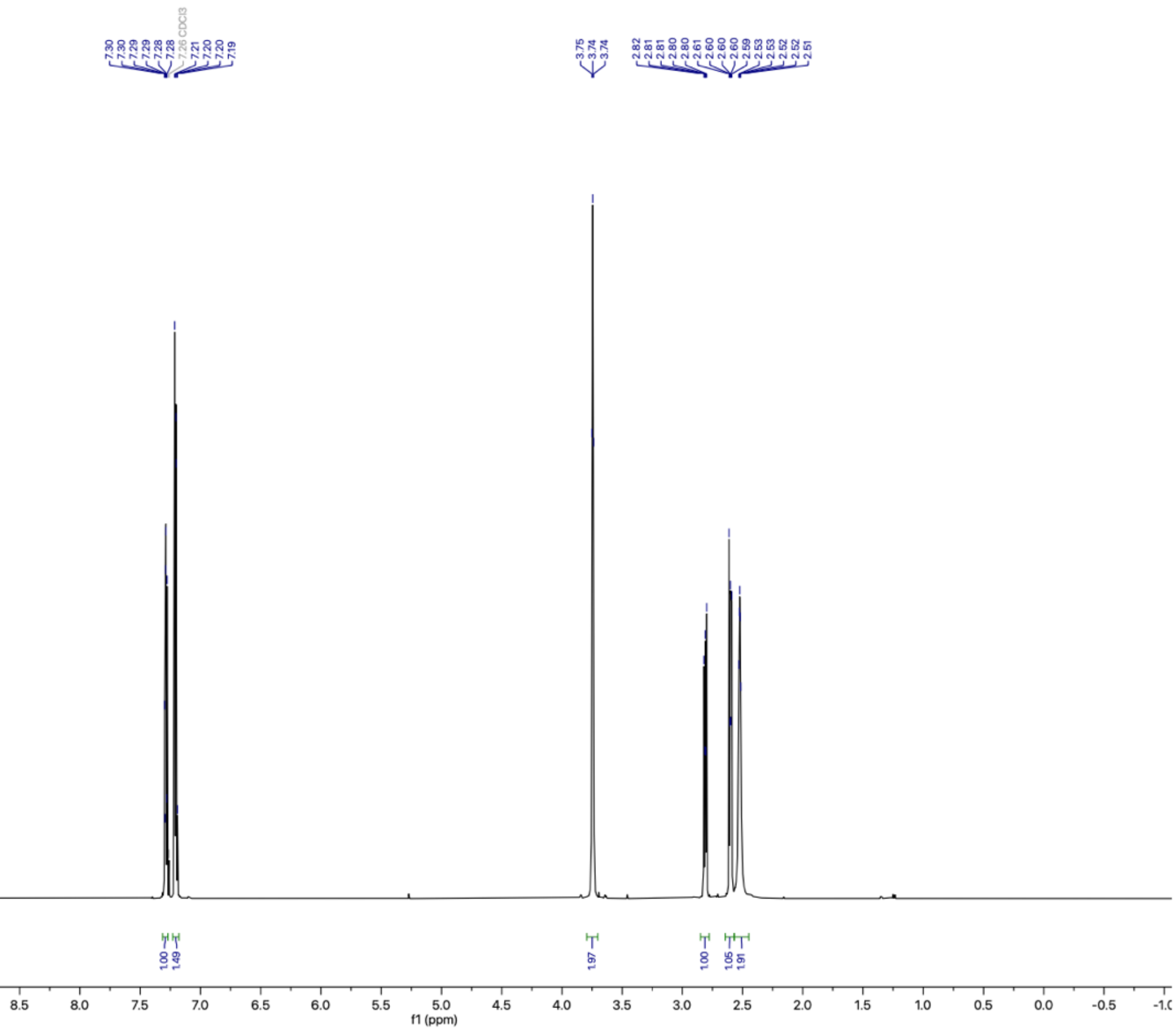

2a

^{13}C NMR, 176 MHz, $CDCl_3$

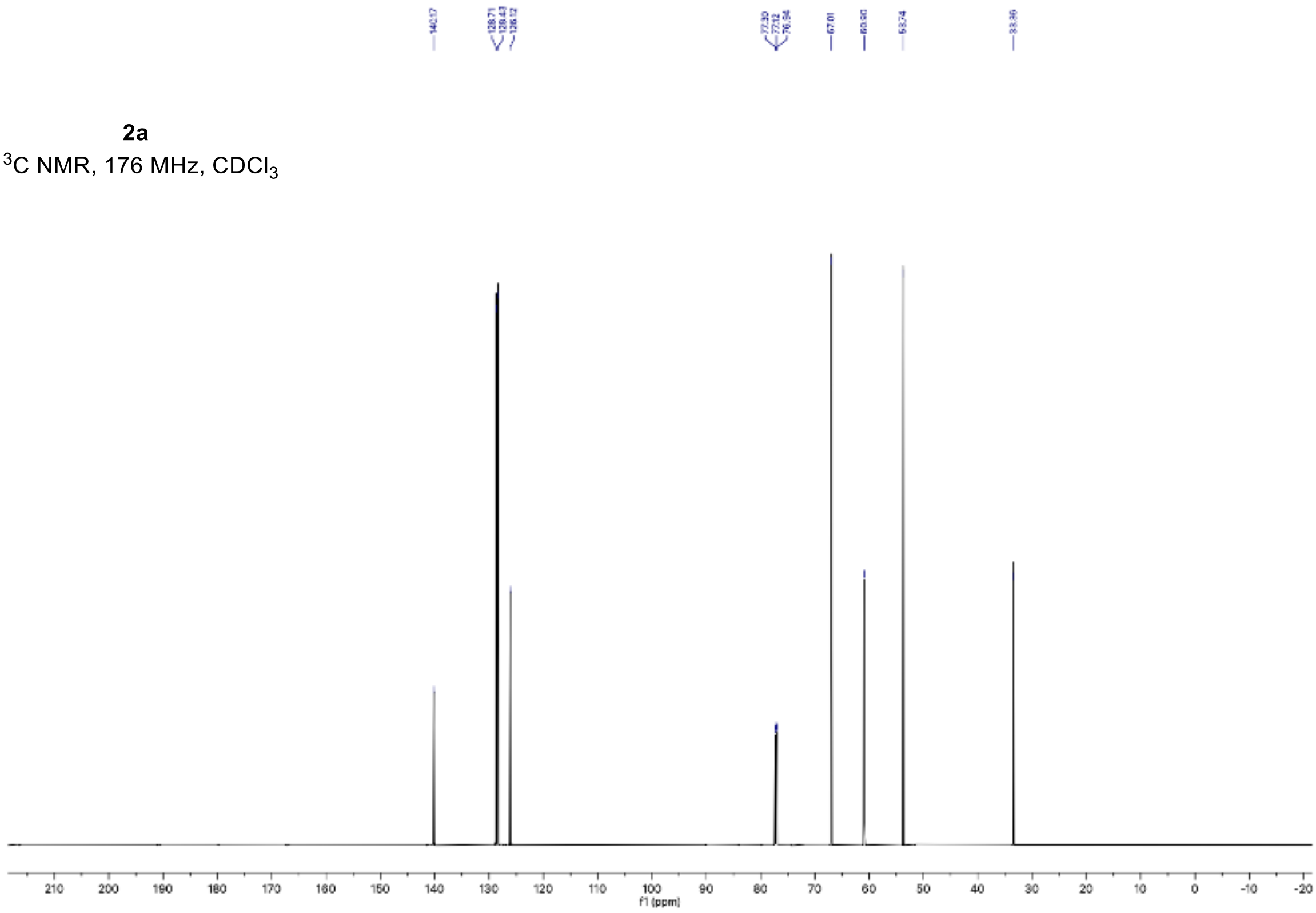

2b

^1H NMR, 700 MHz, $CDCl_3$

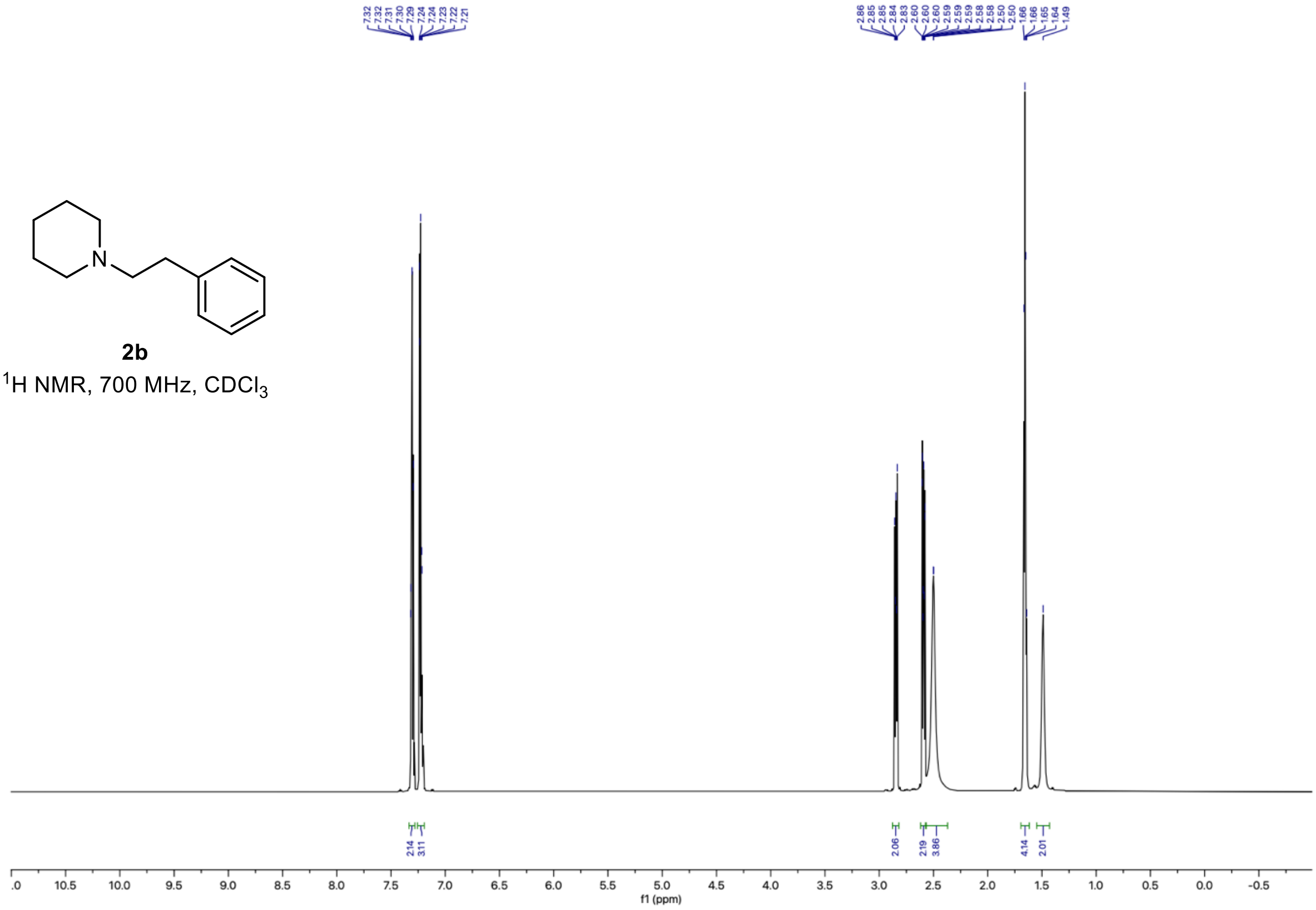

2b

^{13}C NMR, 101 MHz, $CDCl_3$

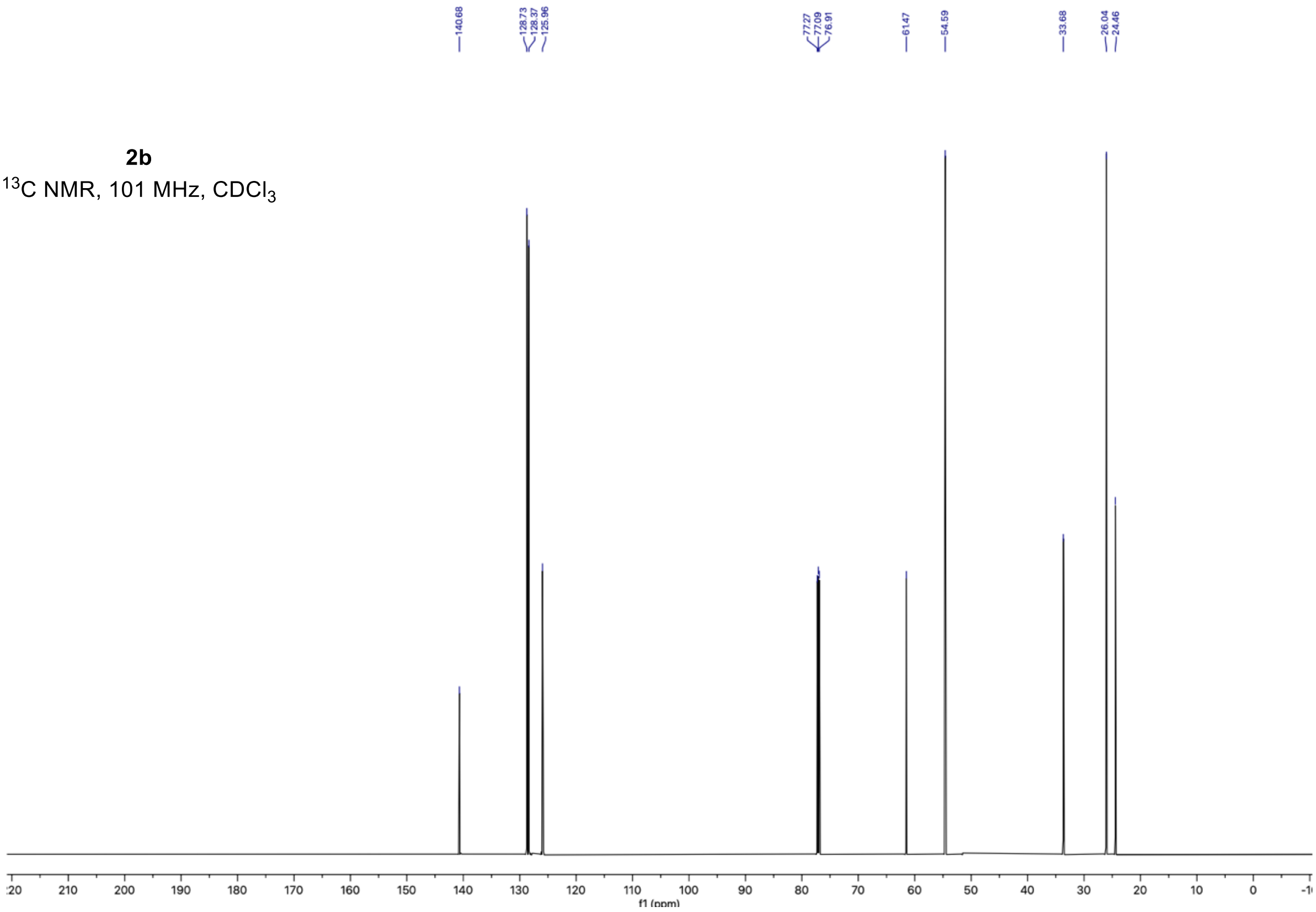

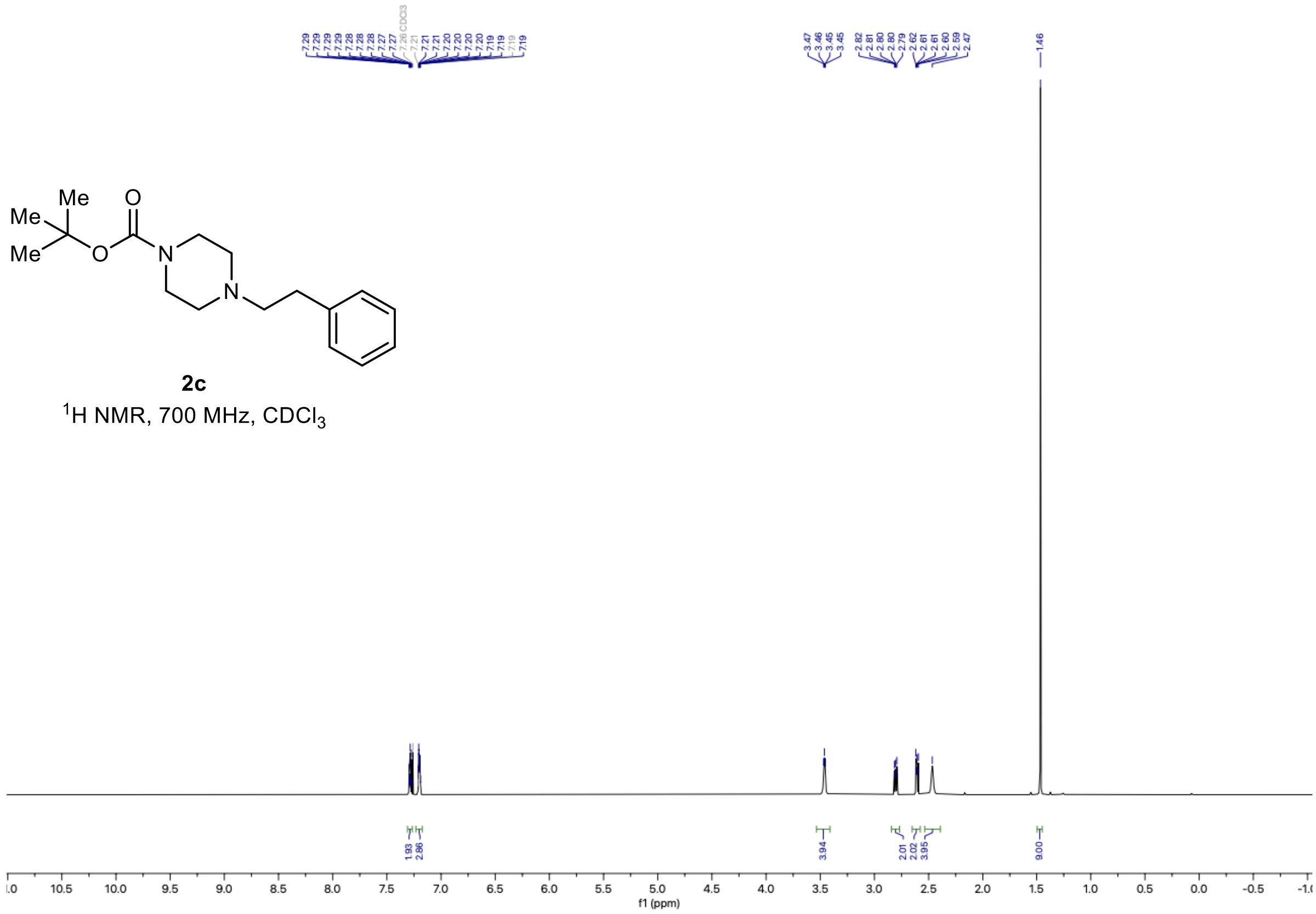

2c

^{1}H NMR, 700 MHz, $CDCl_3$

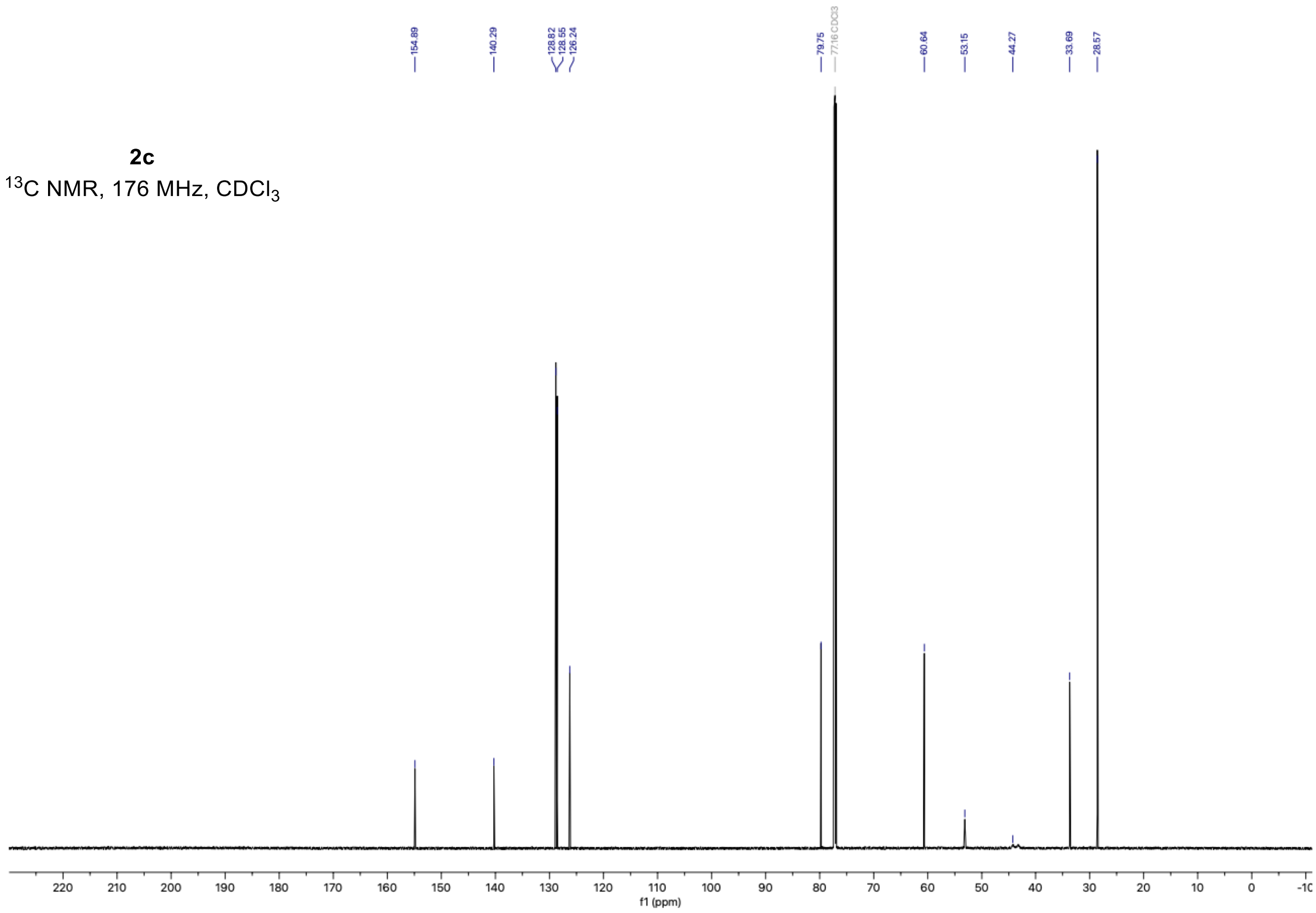

2c

^{13}C NMR, 176 MHz, $CDCl_3$

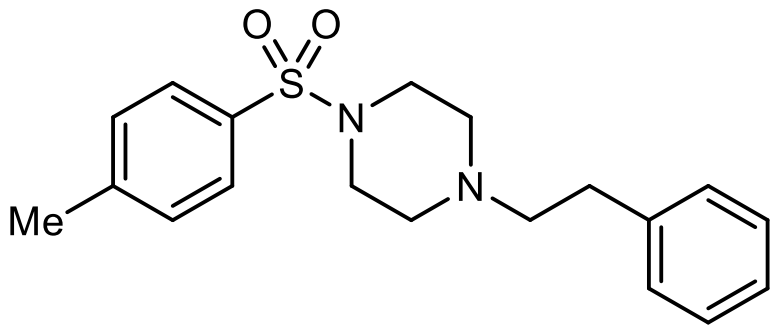

2d

^{1}H NMR, 700 MHz, $CDCl_3$

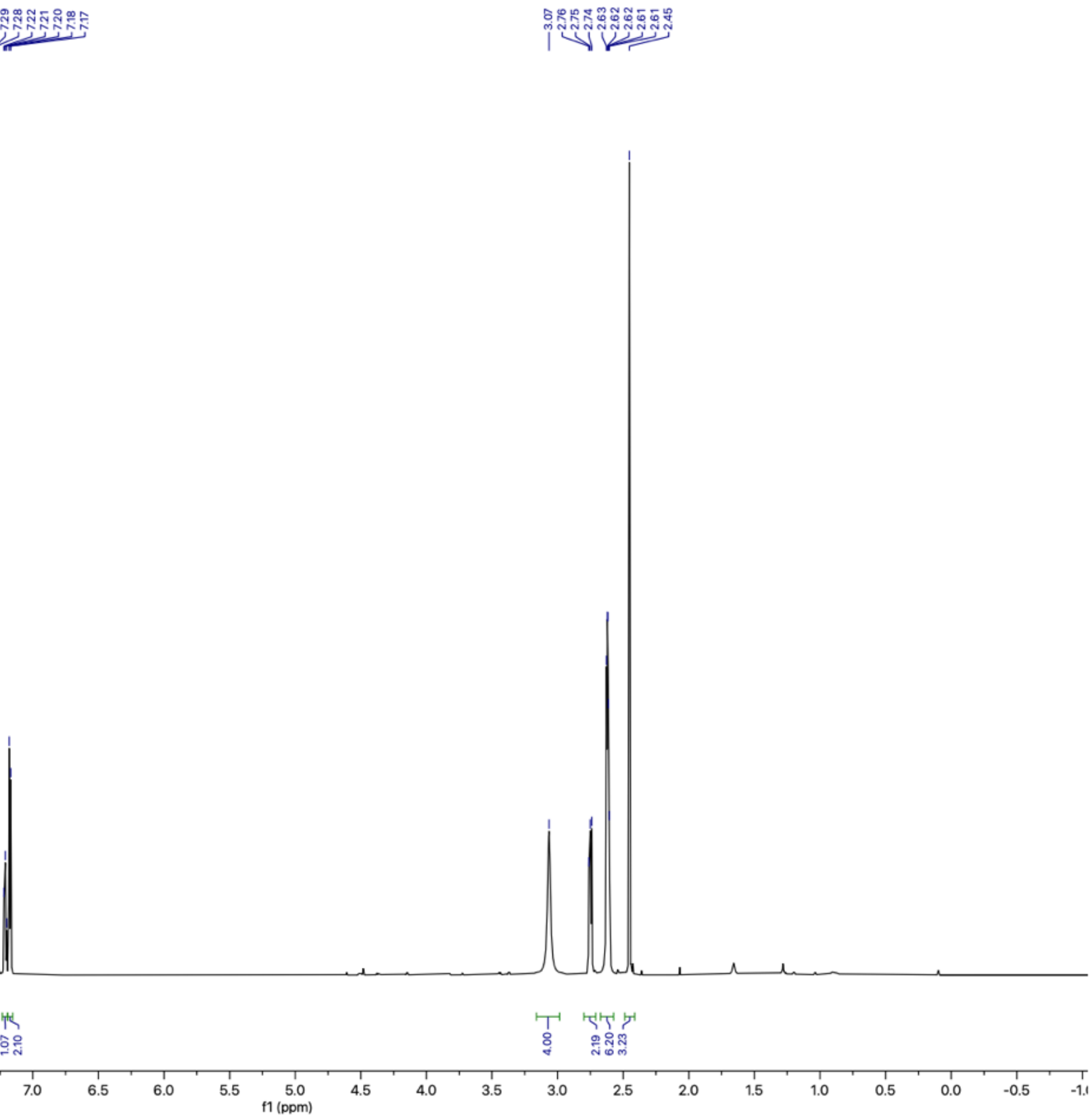

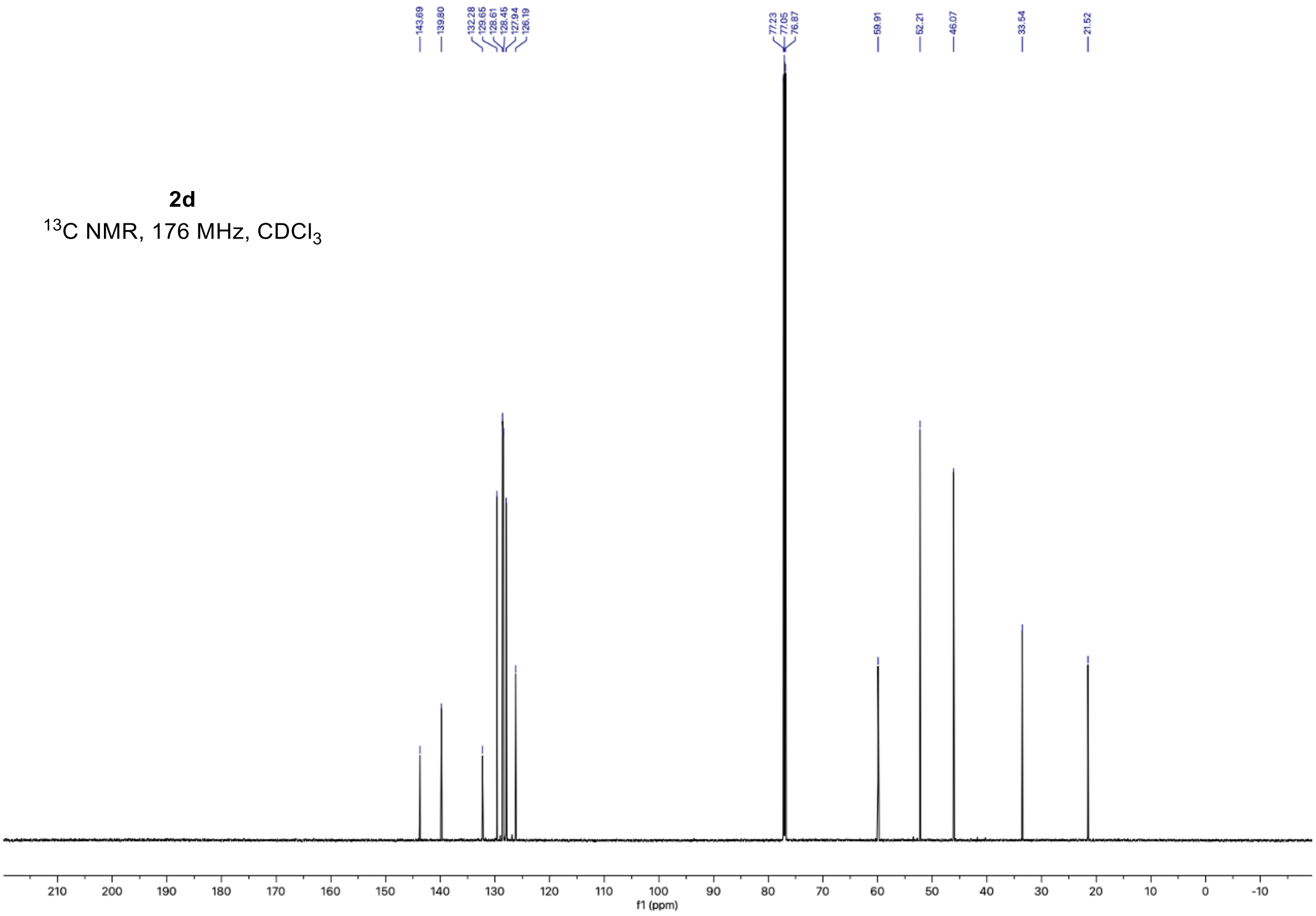

2d

^{13}C NMR, 176 MHz, $CDCl_3$

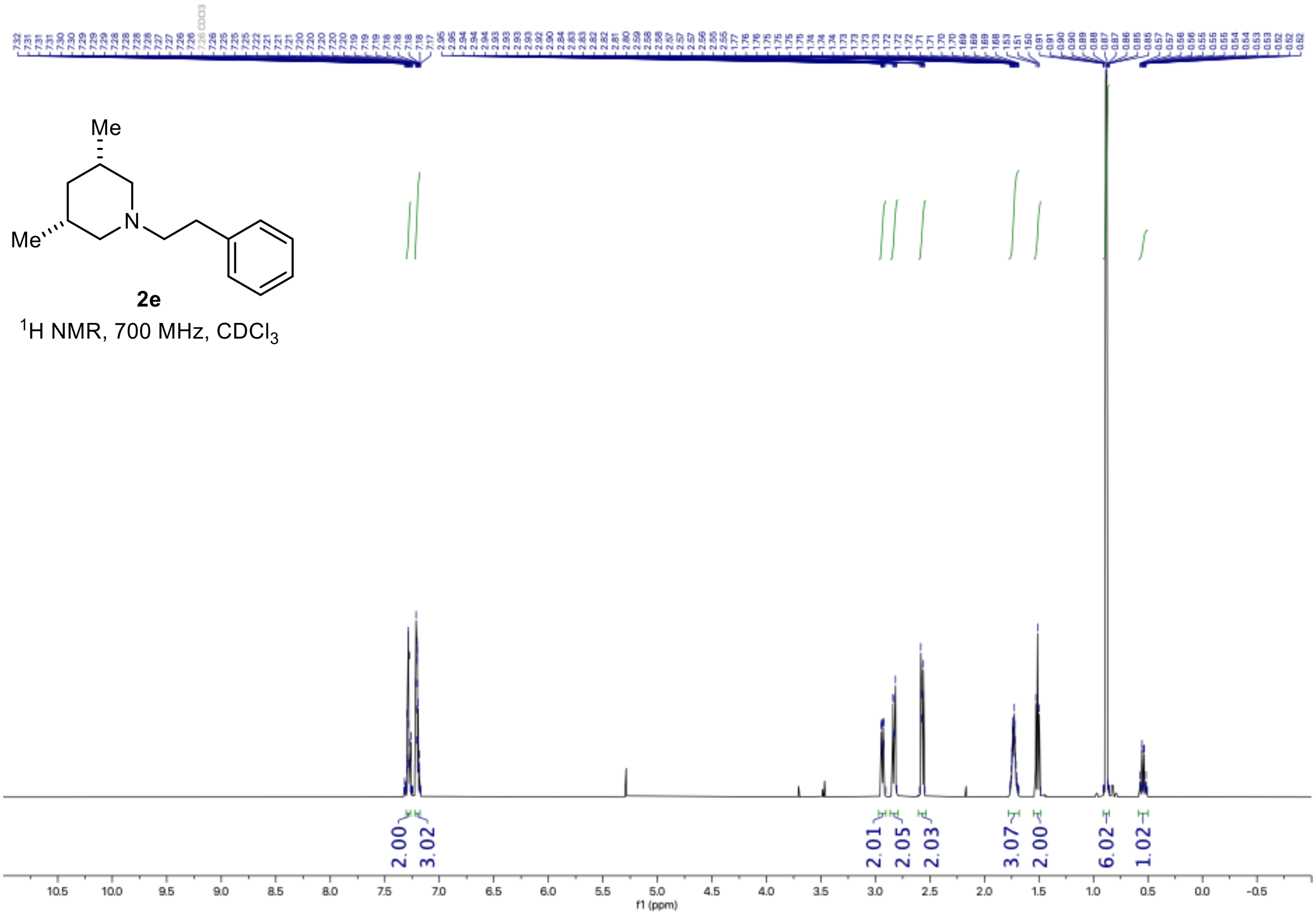
Me
Me
N
2e
^{1}H NMR, 700 MHz, CDCl$_3$
2.00
3.02
2.01
2.05
2.03
3.07
2.00
6.02
1.02
f1 (ppm)

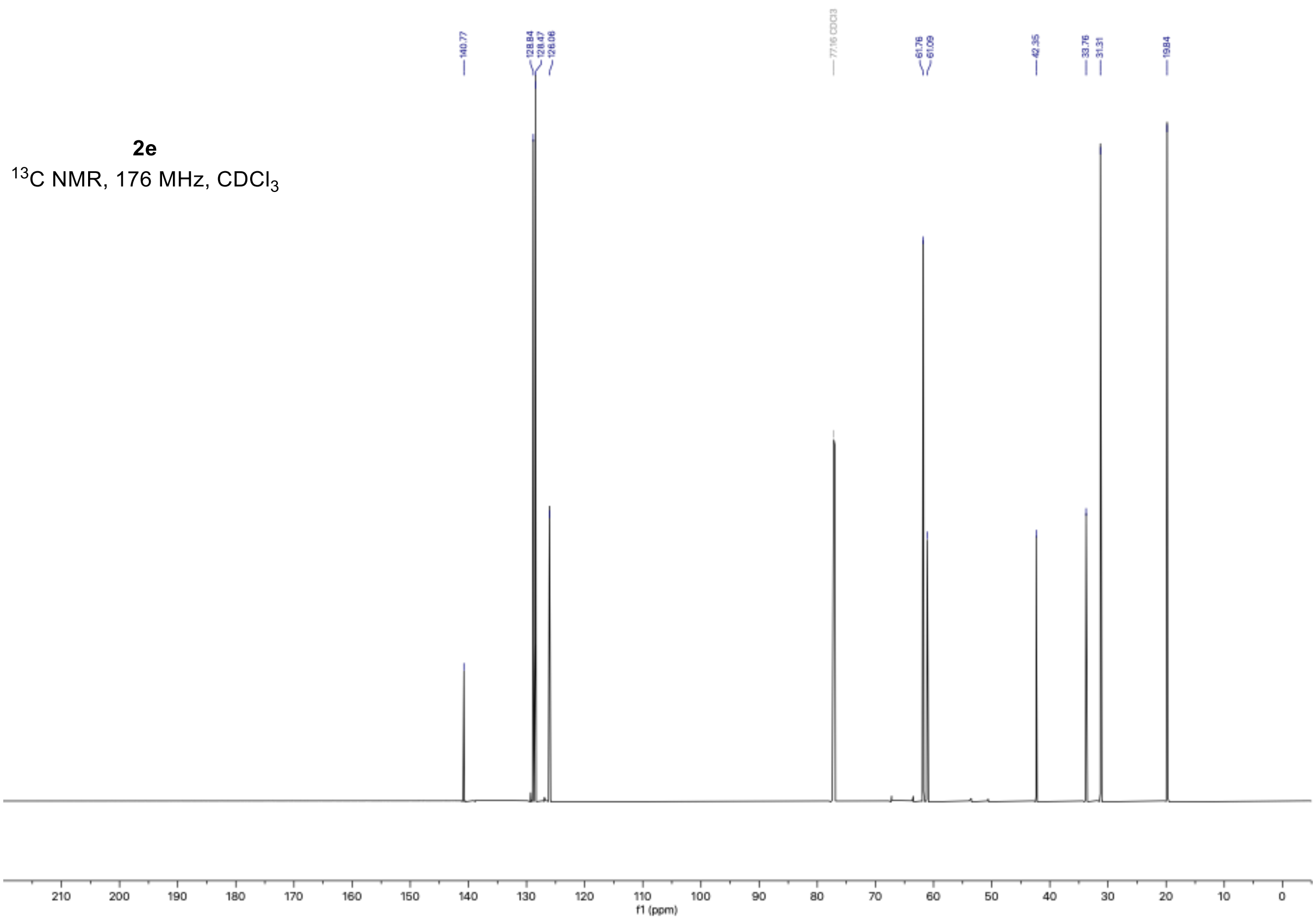
2e
^{13}C NMR, 176 MHz, CDCl$_3$
140.77
128.84
128.47
126.06
61.76
61.09
42.35
33.76
31.31
19.84
f1 (ppm)

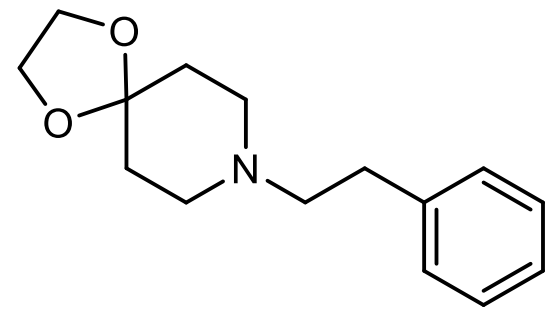

2f

^{1}H NMR, 500 MHz, $CDCl_3$

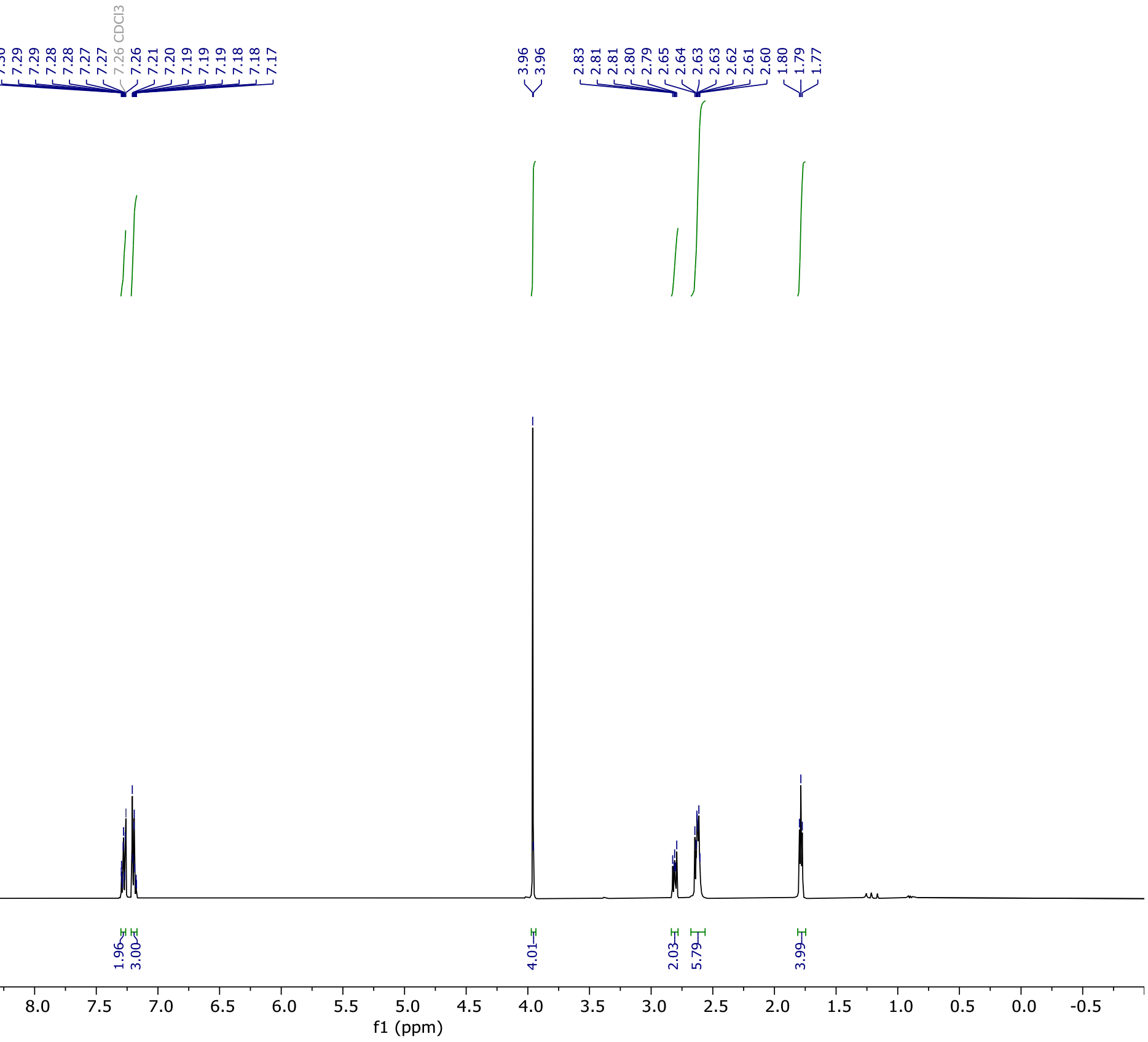

2f

^{13}C NMR, 126 MHz, $CDCl_3$

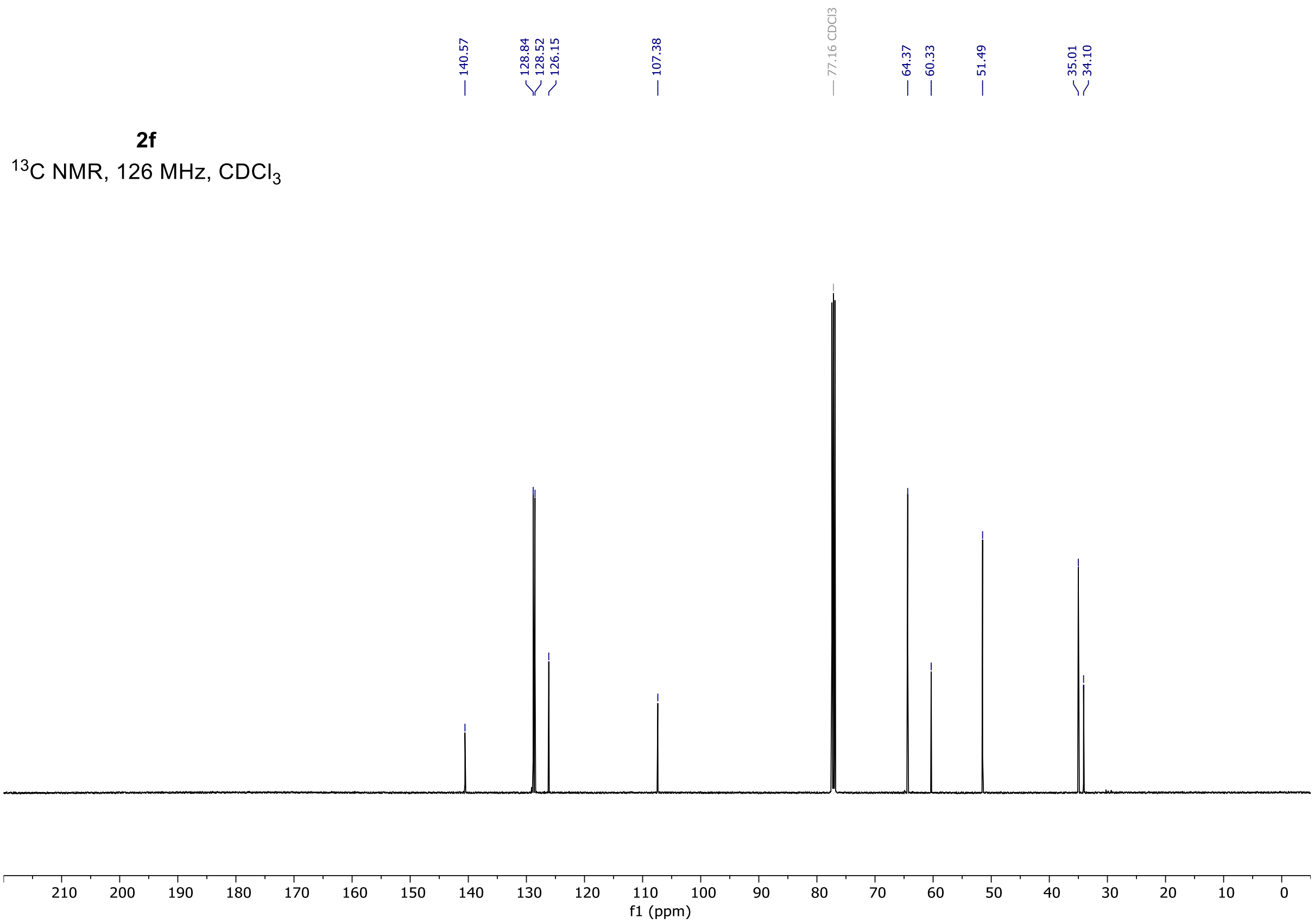

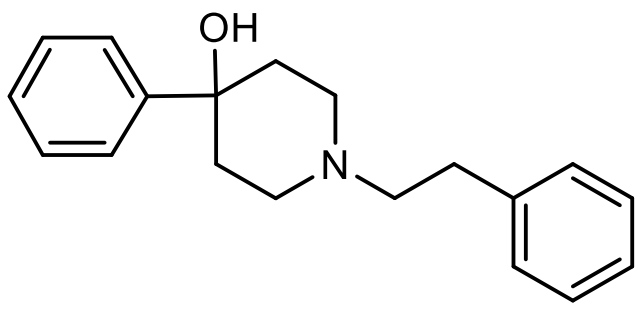

2g

^{1}H NMR, 500 MHz, $CDCl_3$

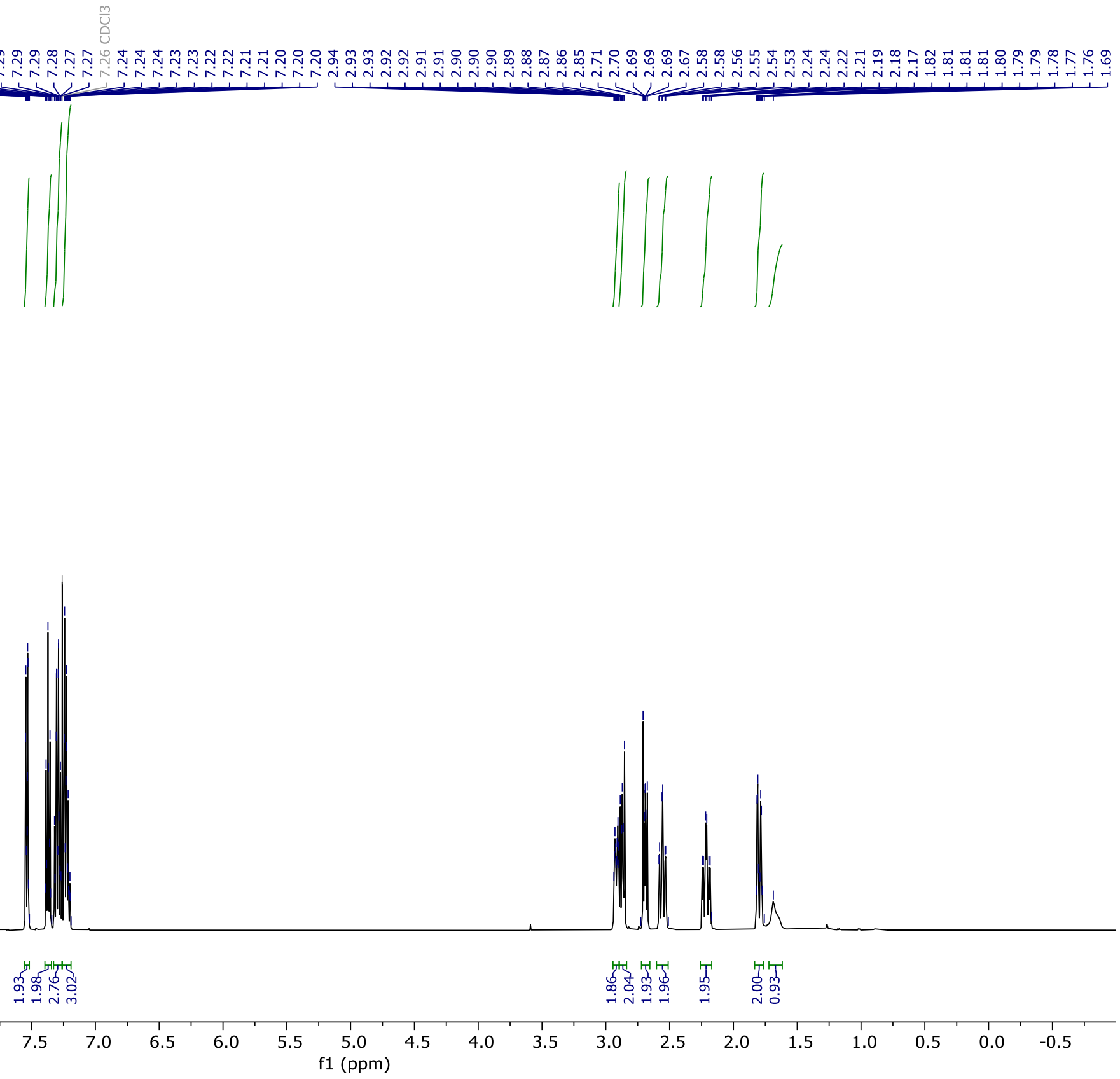

2g

^{13}C NMR, 126 MHz, $CDCl_3$

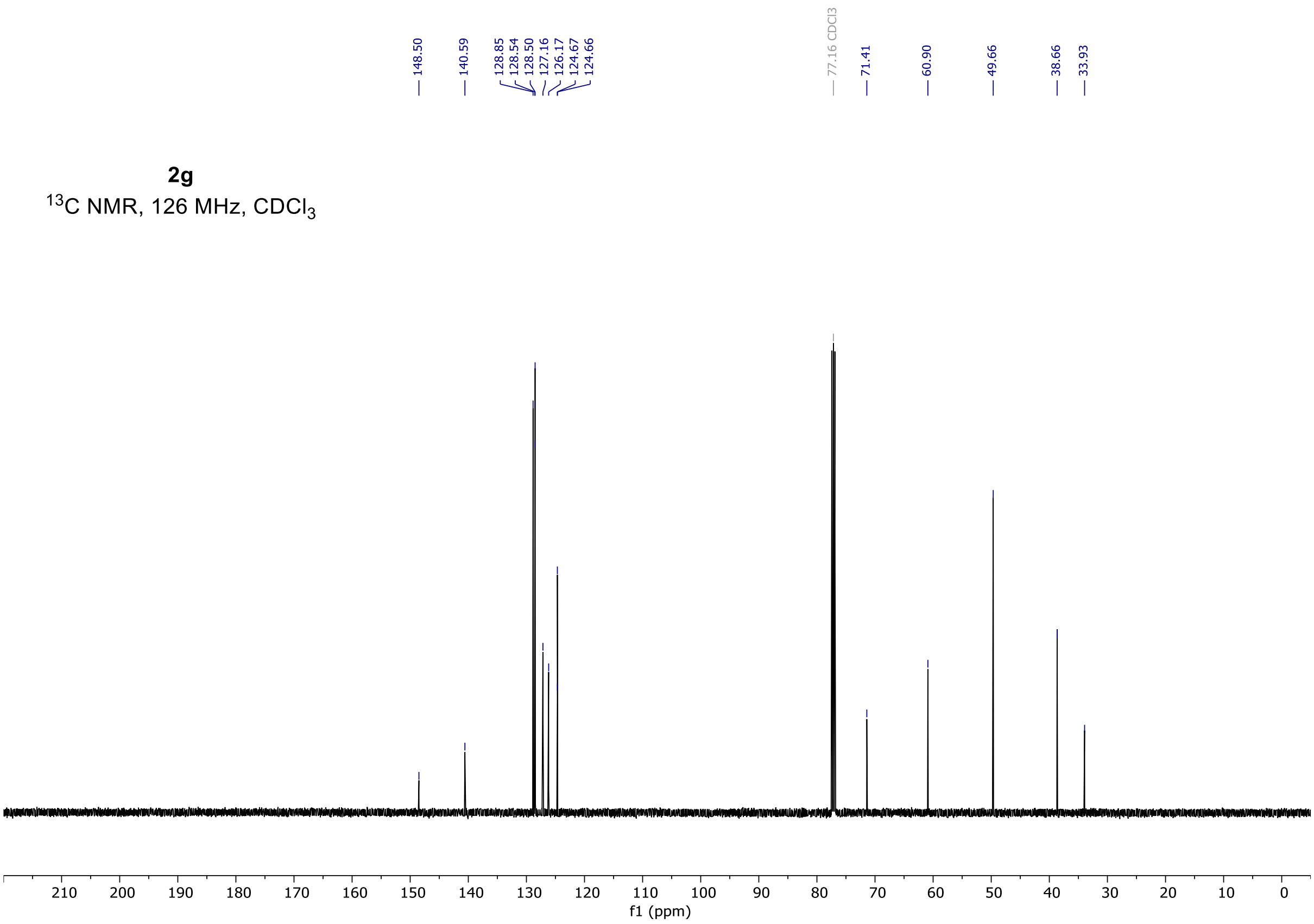

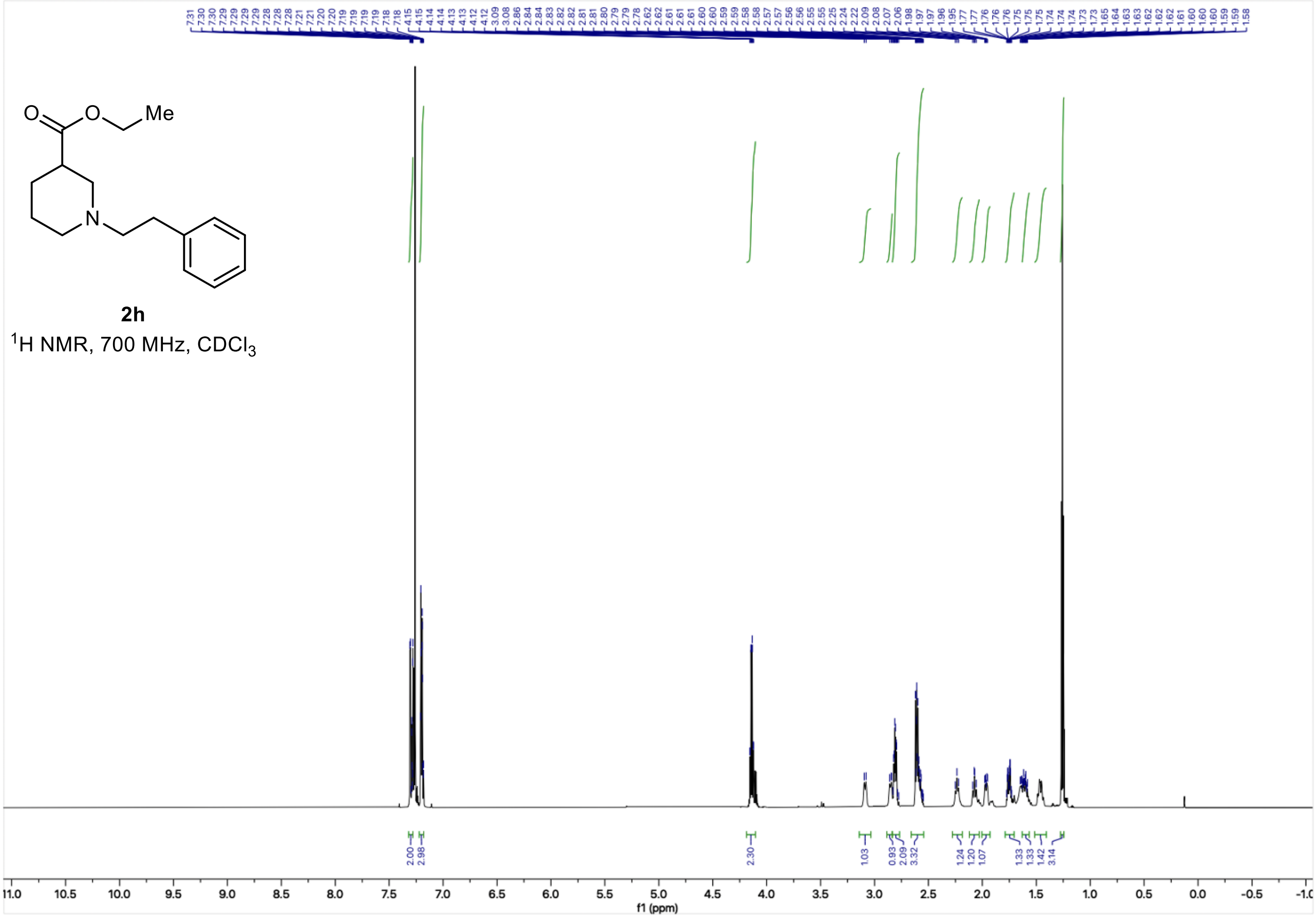

2h

^{1}H NMR, 700 MHz, $CDCl_3$

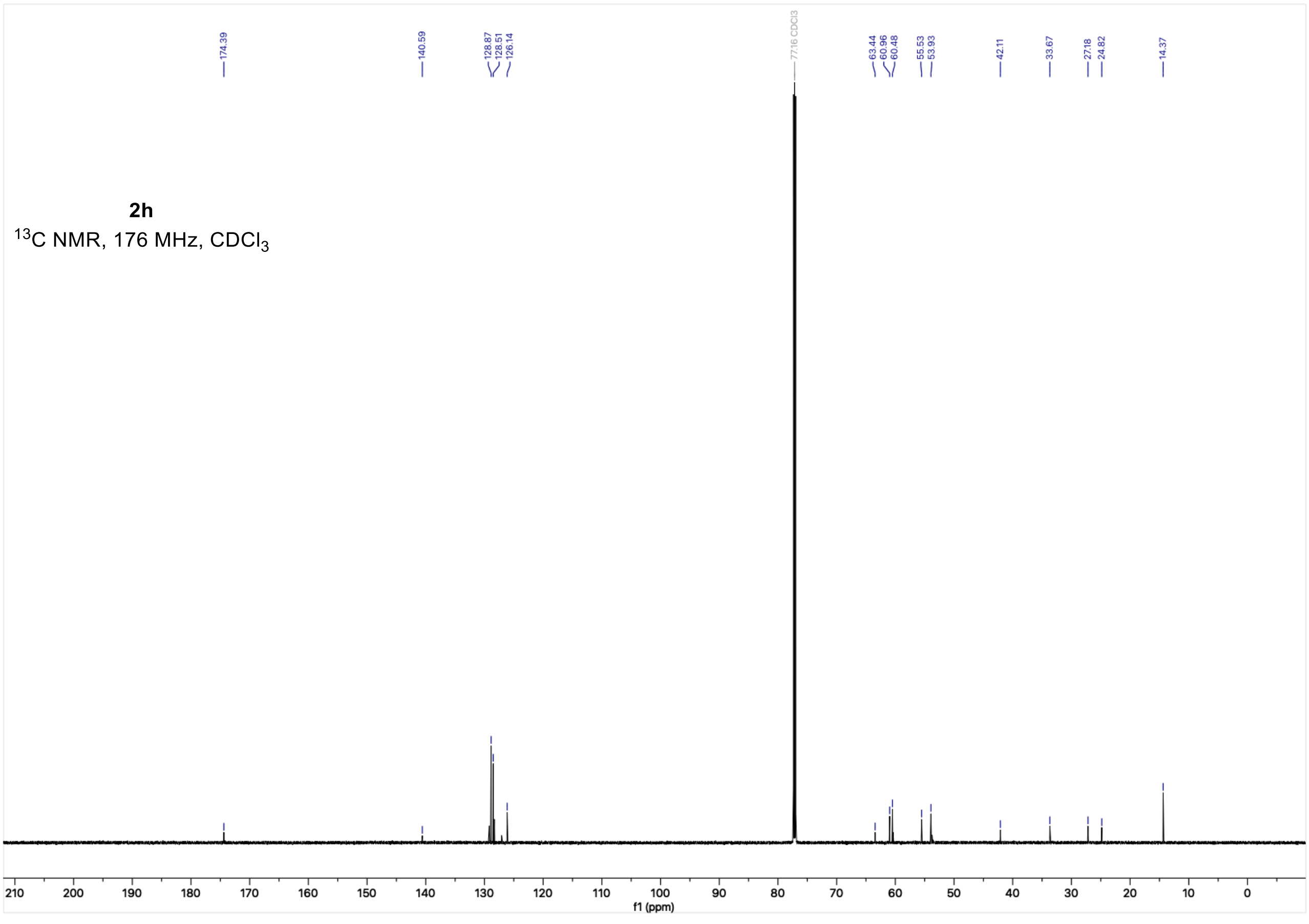

2h

^{13}C NMR, 176 MHz, $CDCl_3$

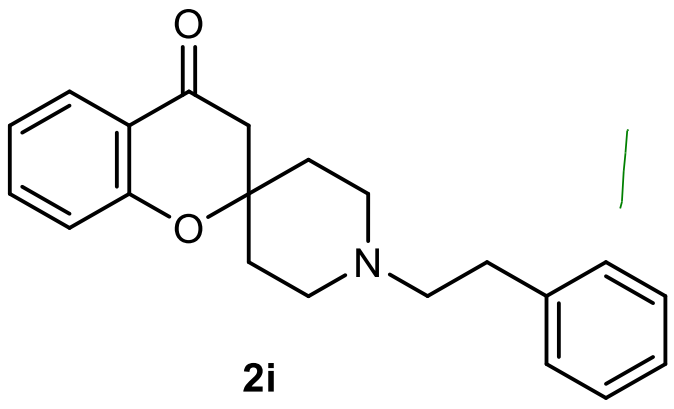

2i

^{1}H NMR, 500 MHz, $CDCl_3$

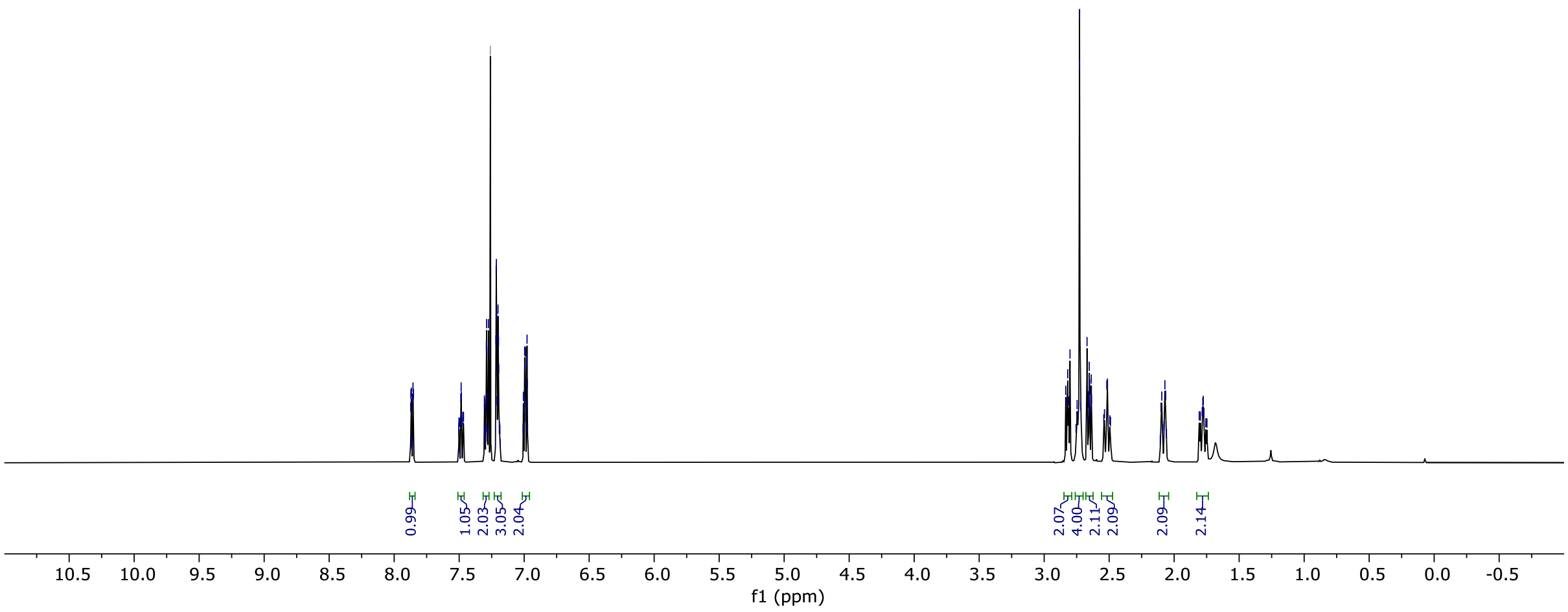

2i

^{13}C NMR, 126 MHz, $CDCl_3$

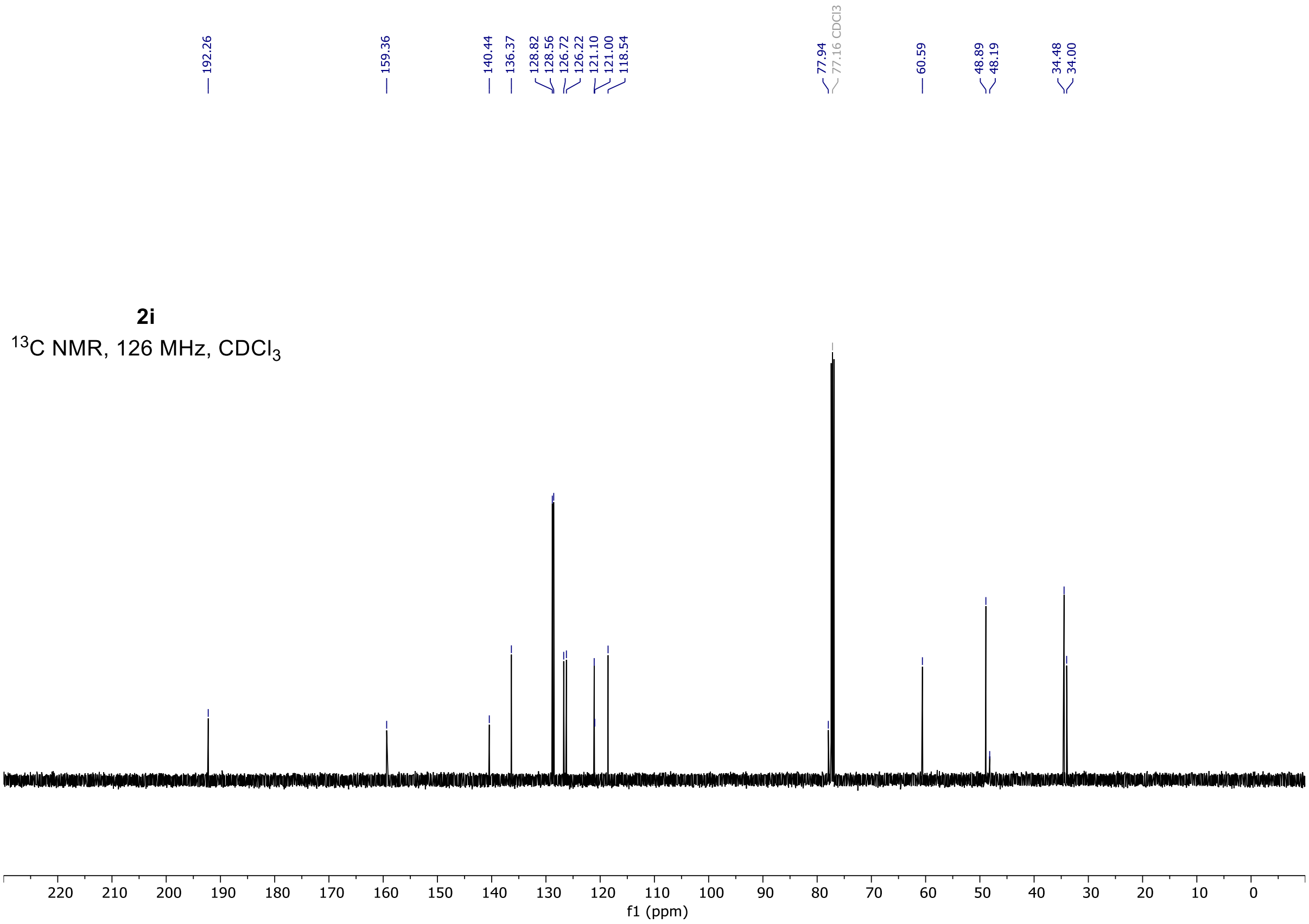

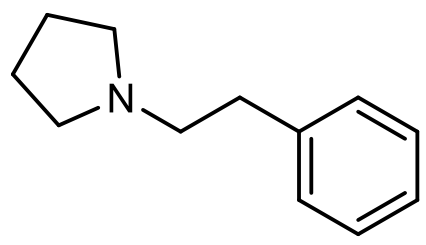

2j

^{1}H NMR, 700 MHz, $CDCl_3$

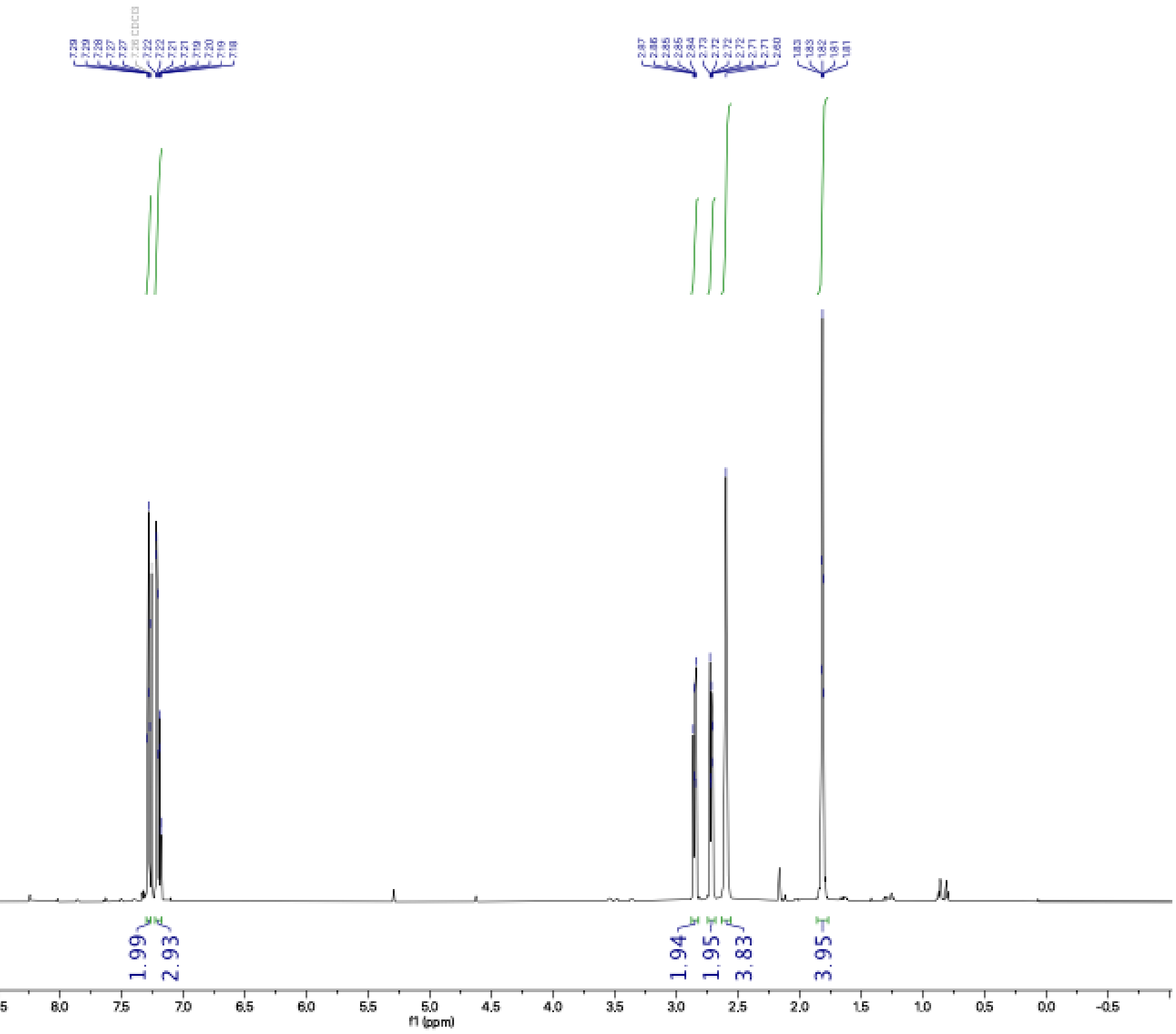

2j

^{13}C NMR, 176 MHz, $CDCl_3$

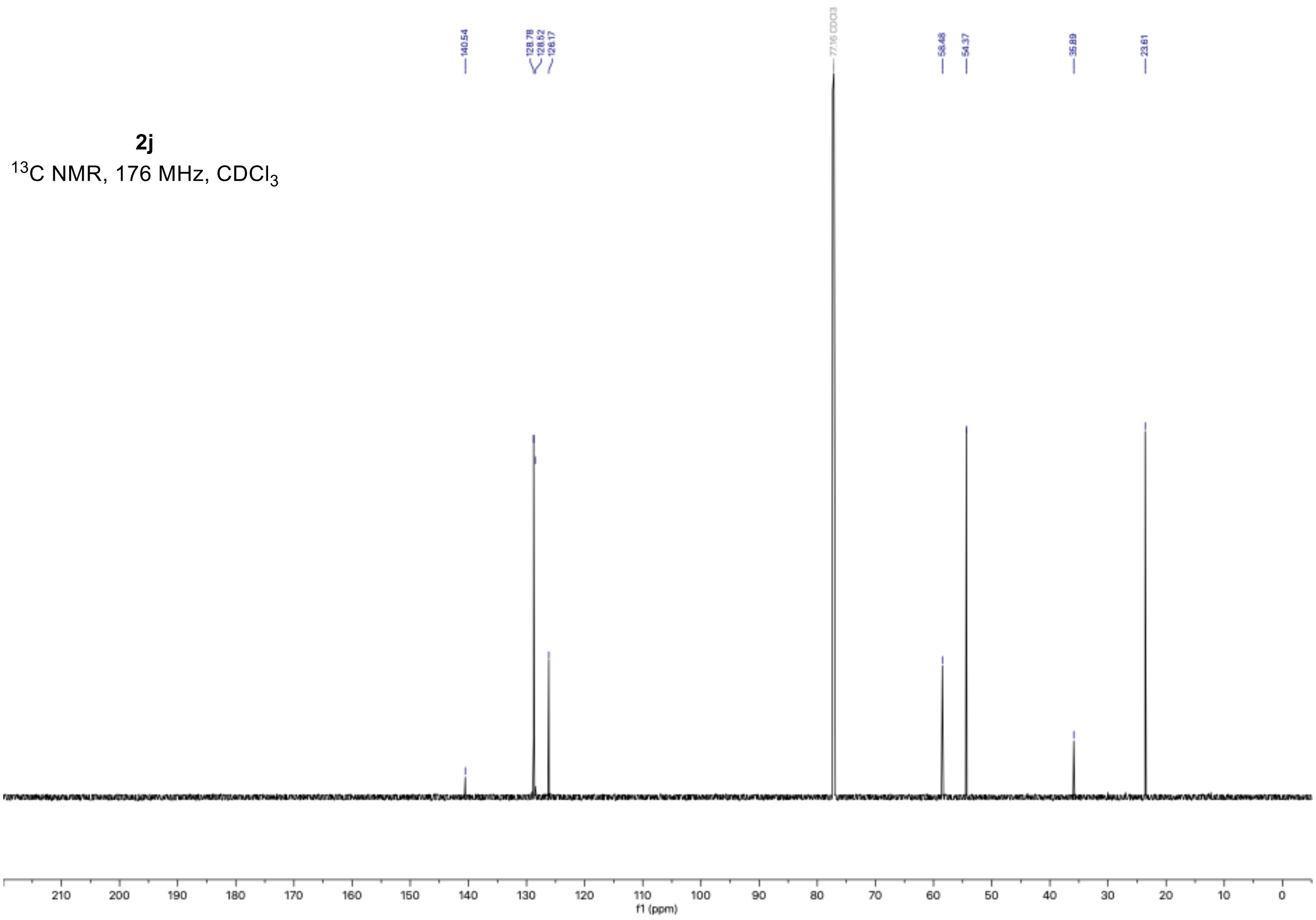

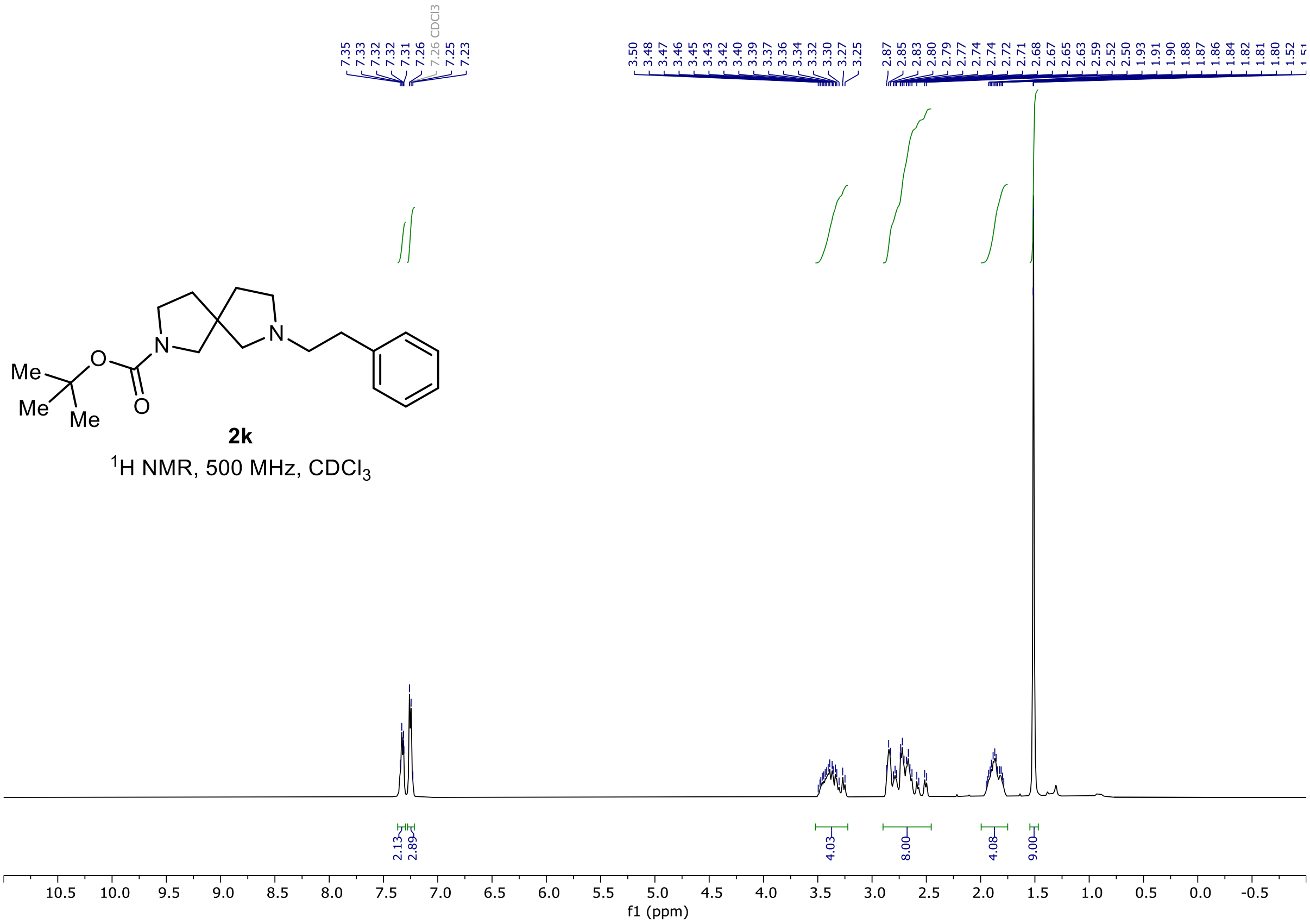

2k

^{1}H NMR, 500 MHz, $CDCl_3$

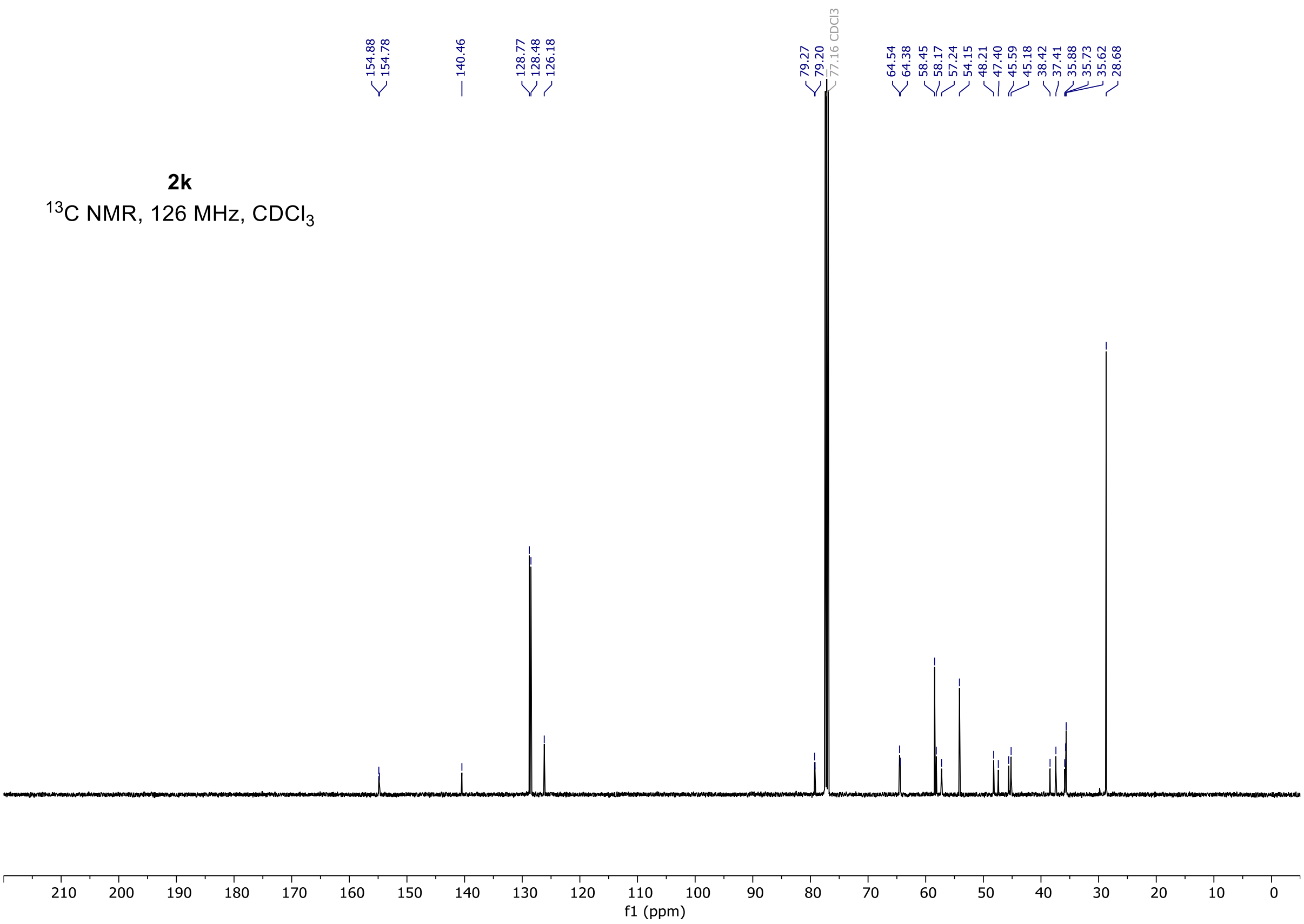

2k

^{13}C NMR, 126 MHz, $CDCl_3$

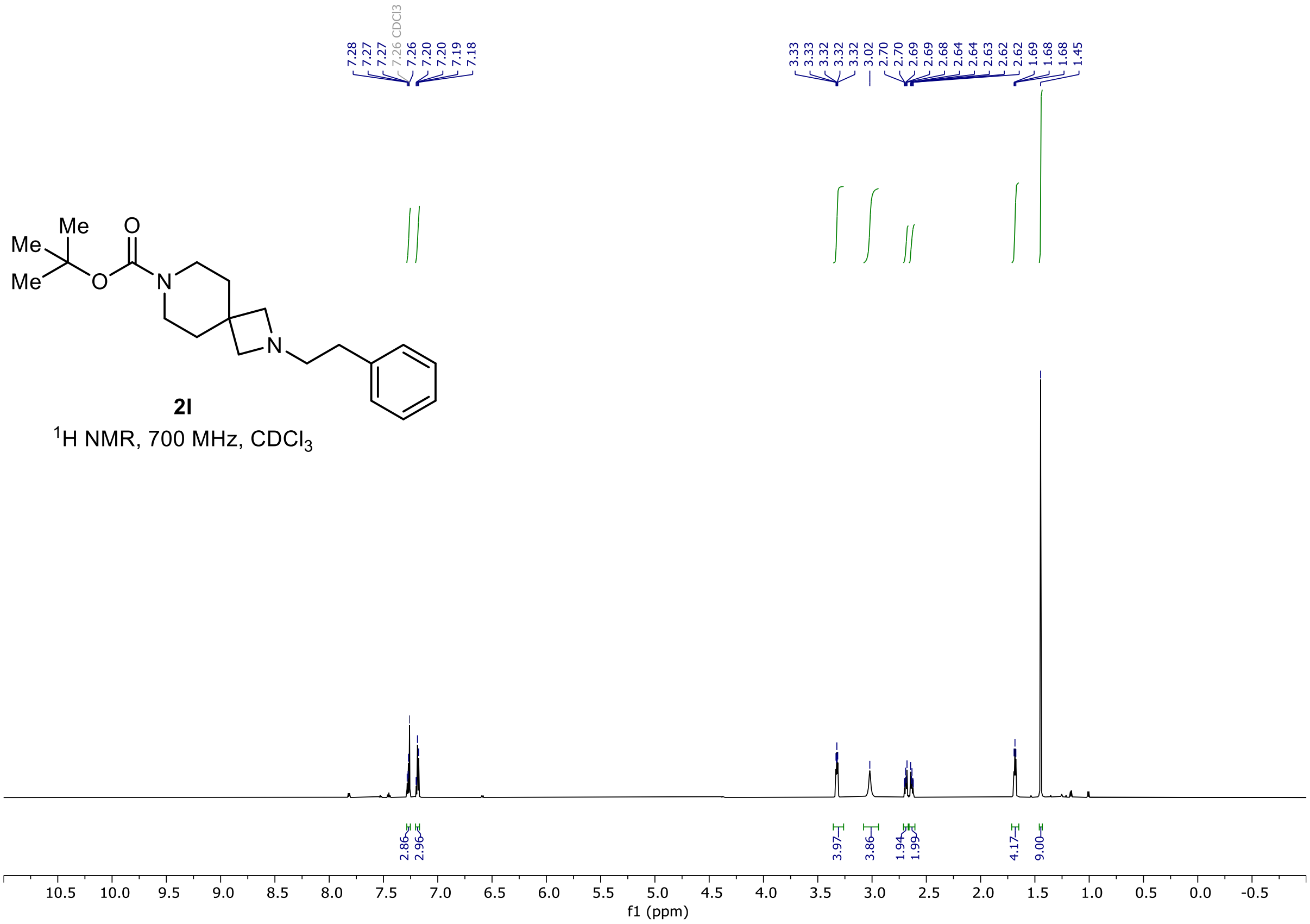

2l

^{1}H NMR, 700 MHz, $CDCl_3$

2l

^{13}C NMR, 176 MHz, $CDCl_3$

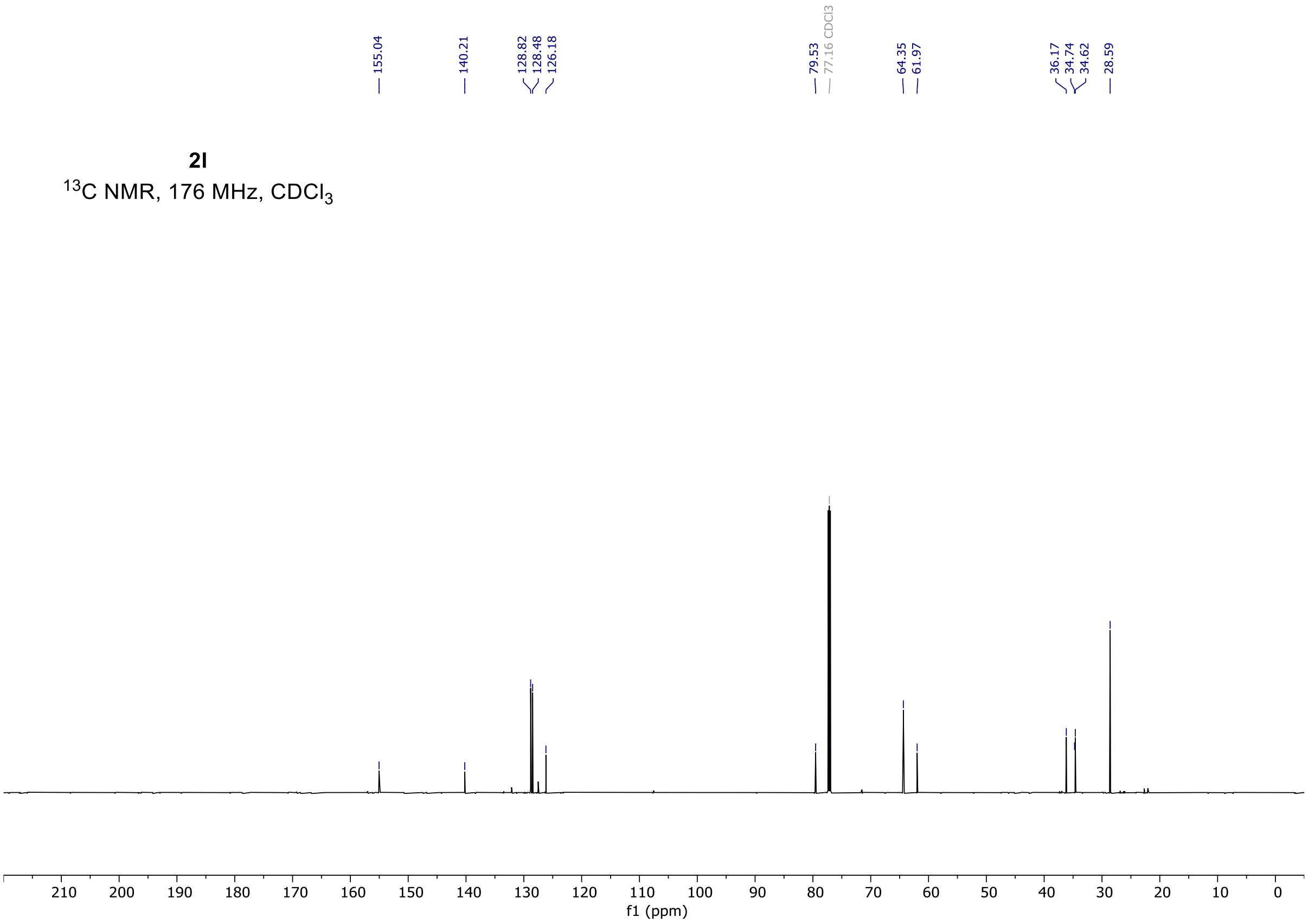

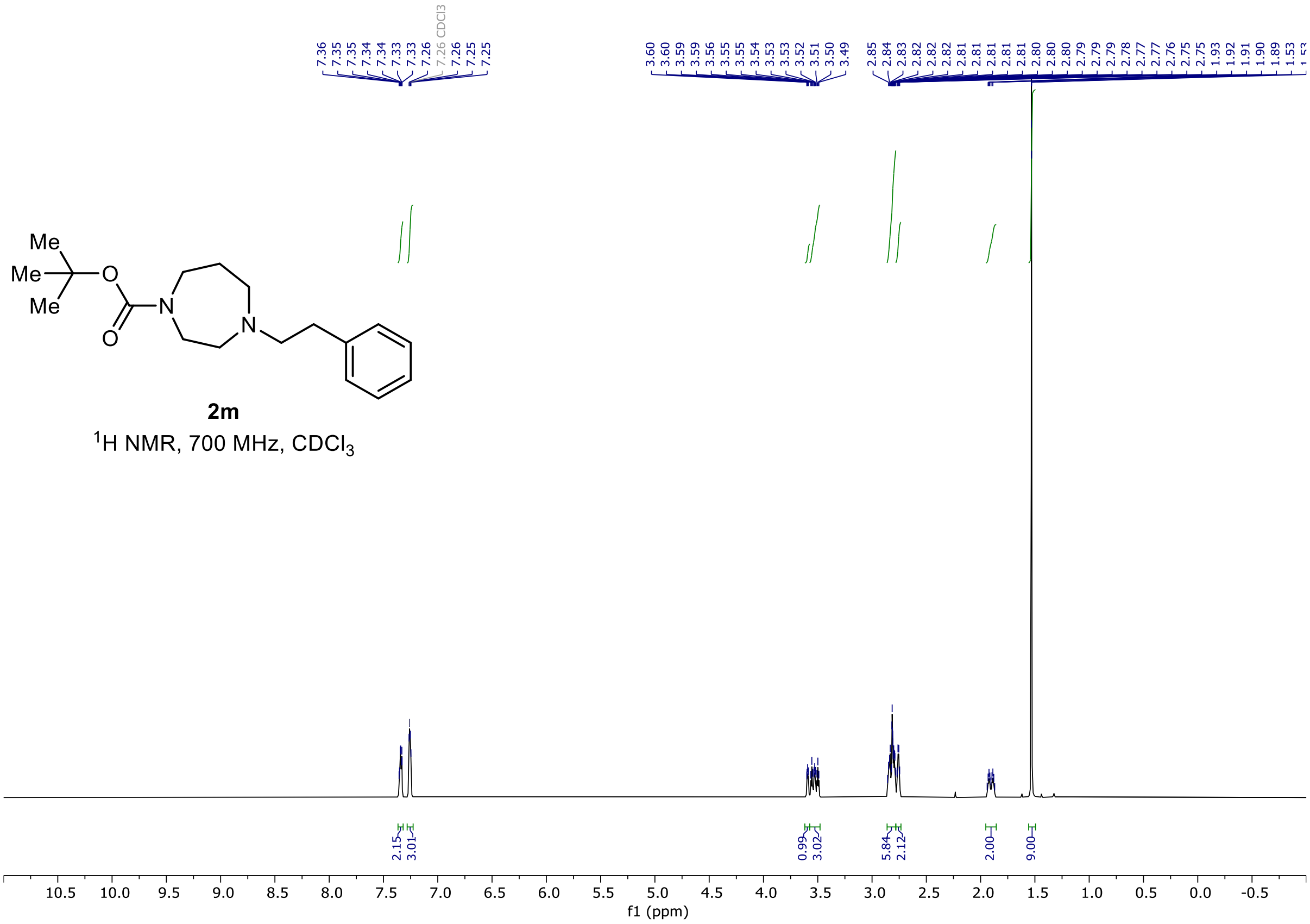

2m

^{1}H NMR, 700 MHz, $CDCl_3$

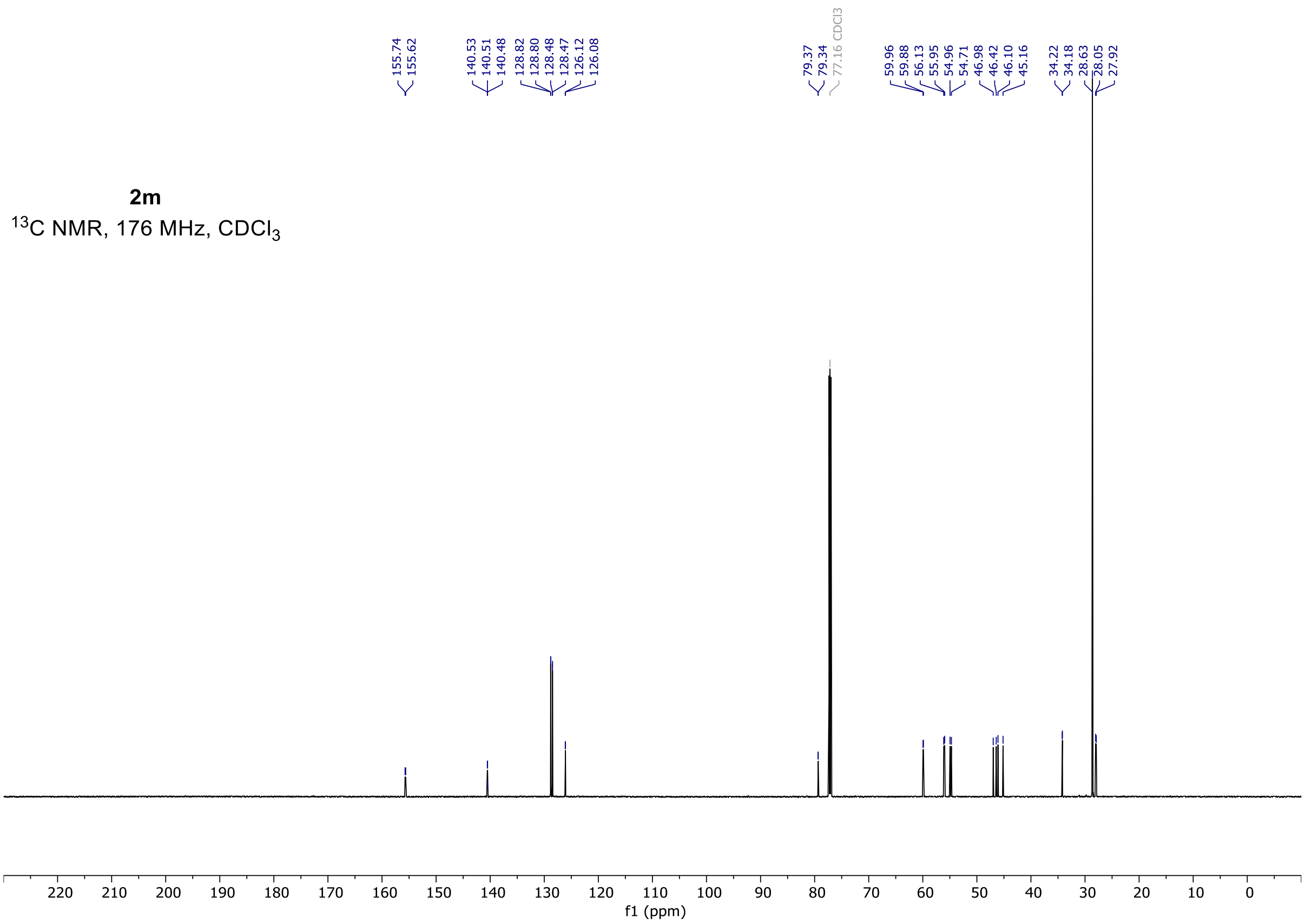

2m

^{13}C NMR, 176 MHz, $CDCl_3$

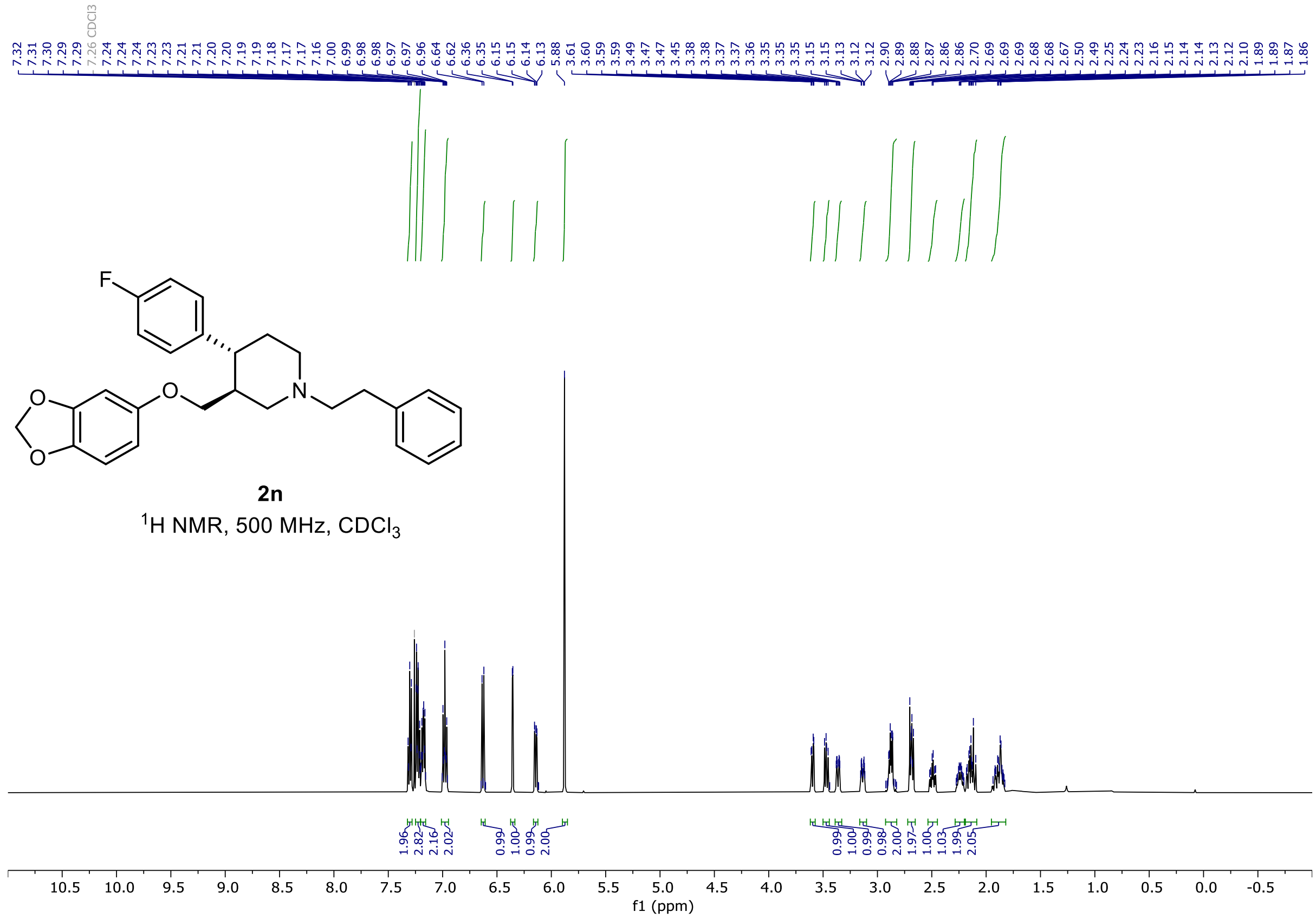

2n

^{1}H NMR, 500 MHz, $CDCl_3$

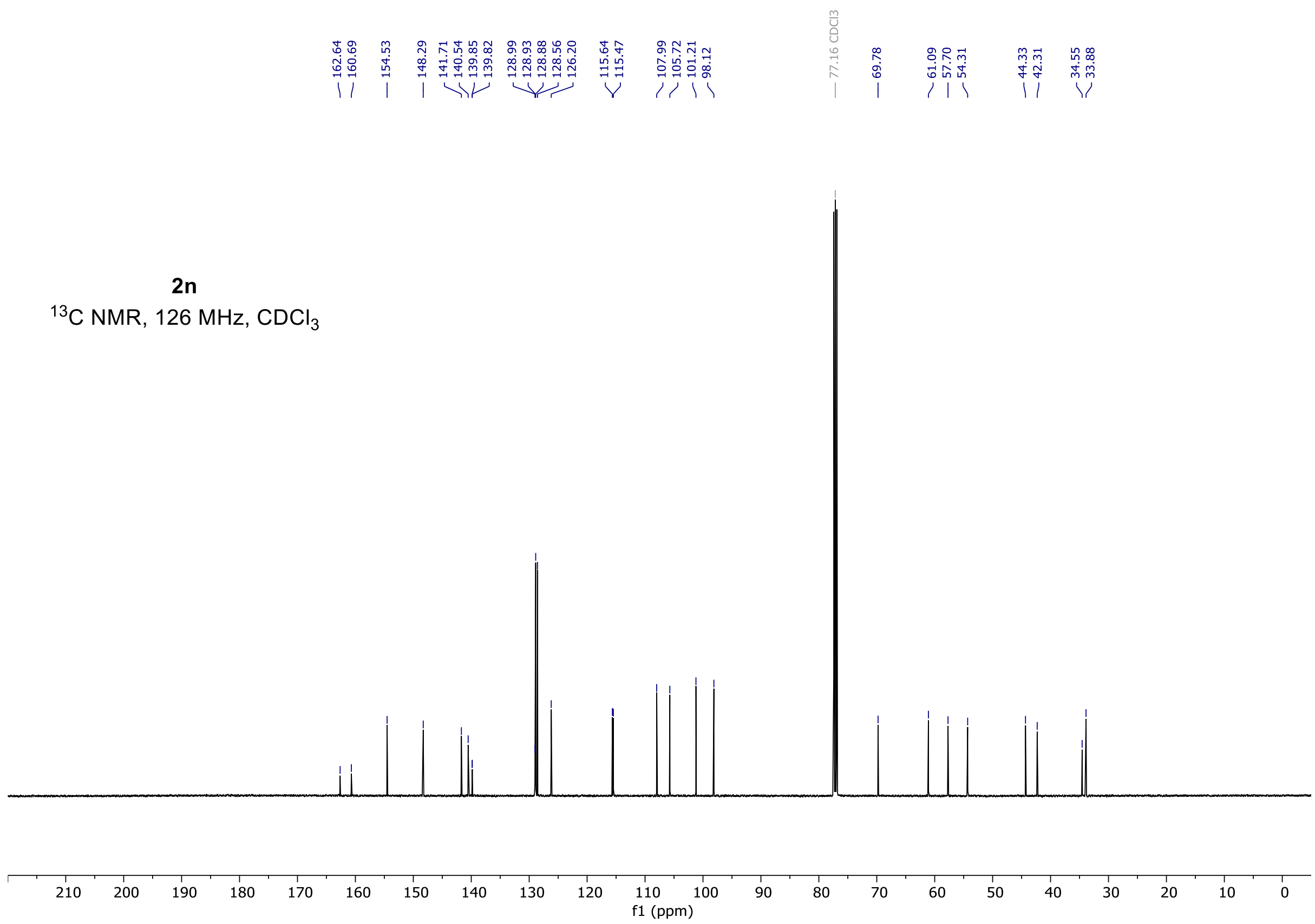

2n

^{13}C NMR, 126 MHz, $CDCl_3$

2n

^{19}F NMR, 471 MHz, $CDCl_3$

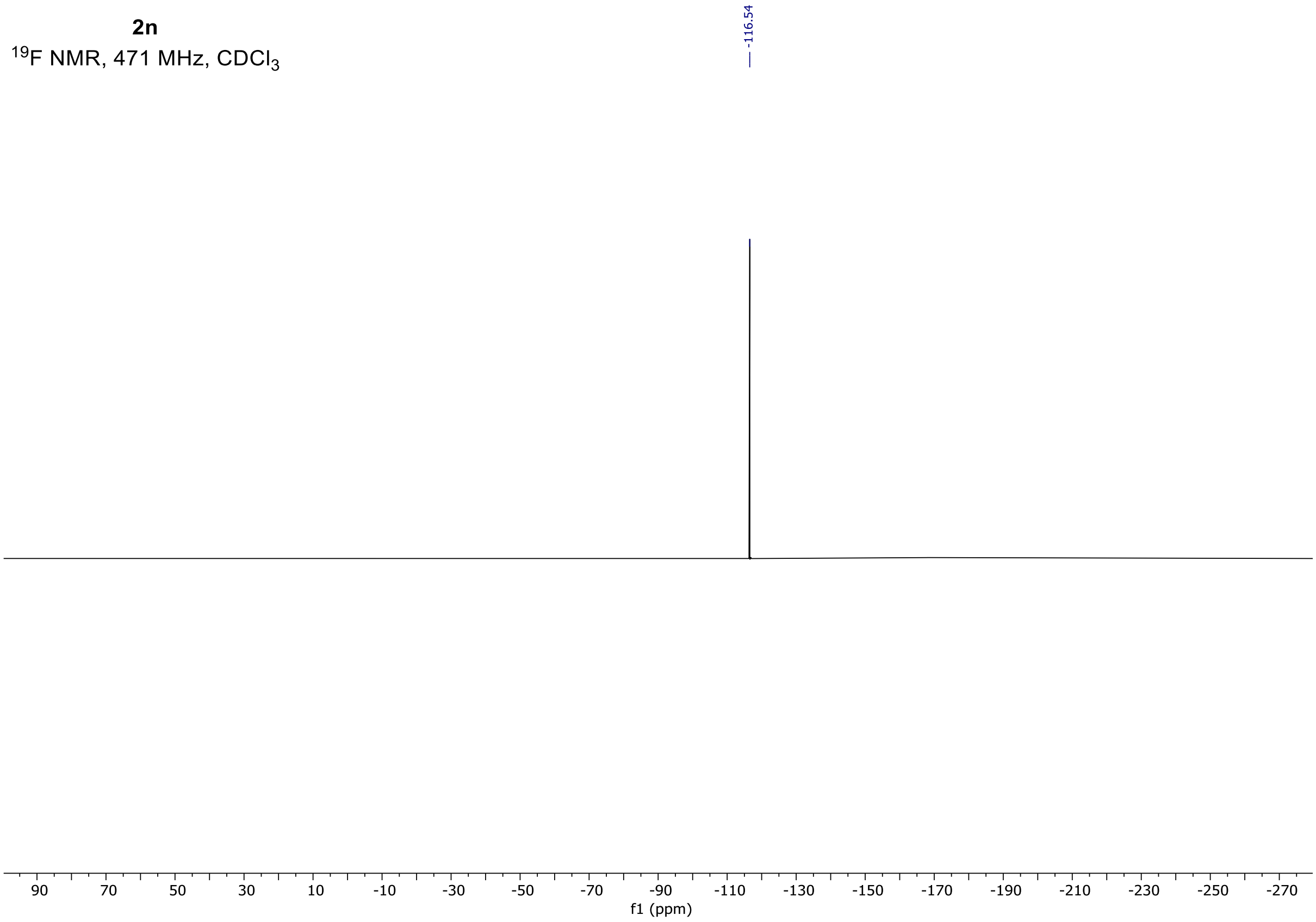

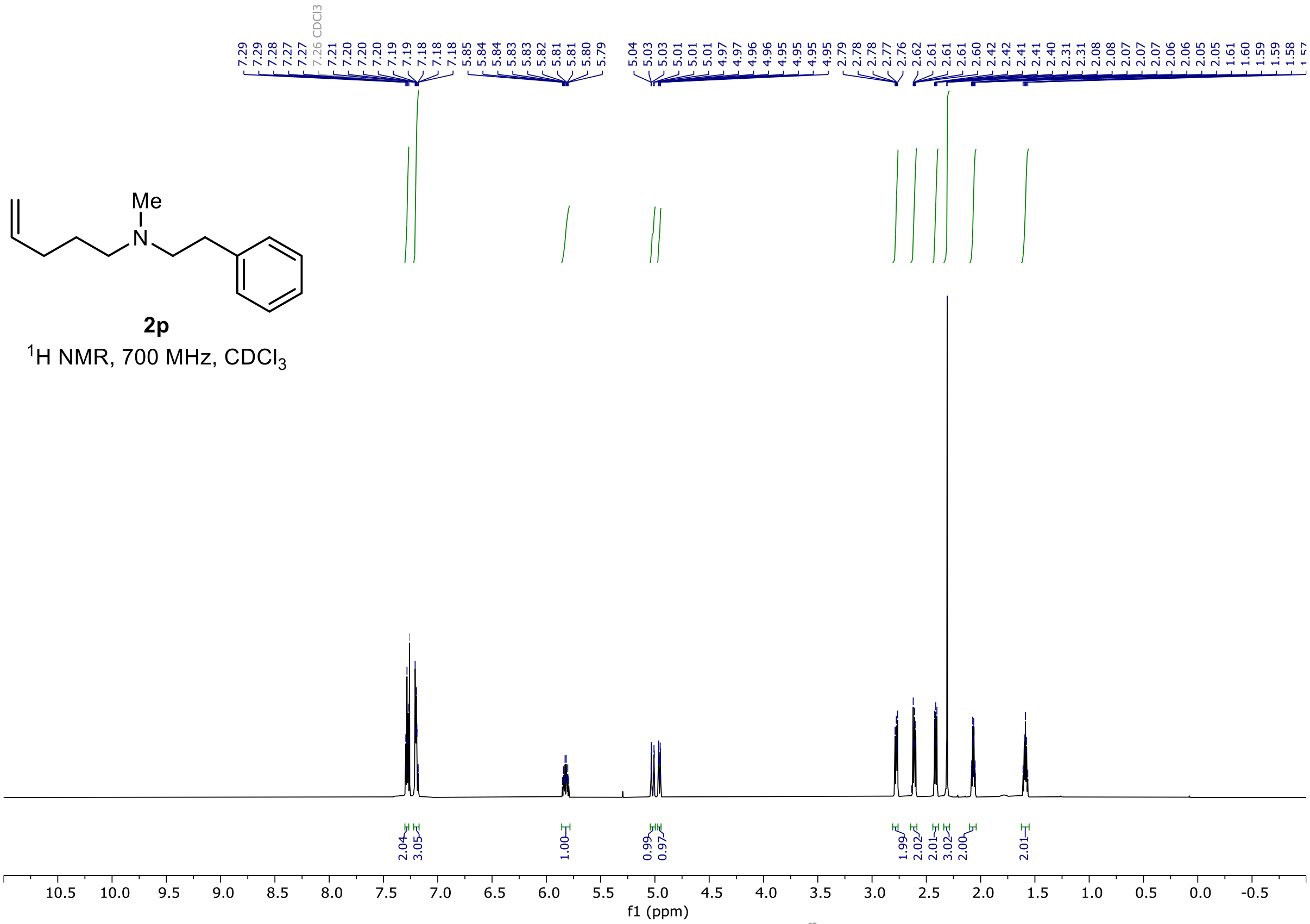
2p
^{1}H NMR, 700 MHz, $CDCl_3$
f1 (ppm)

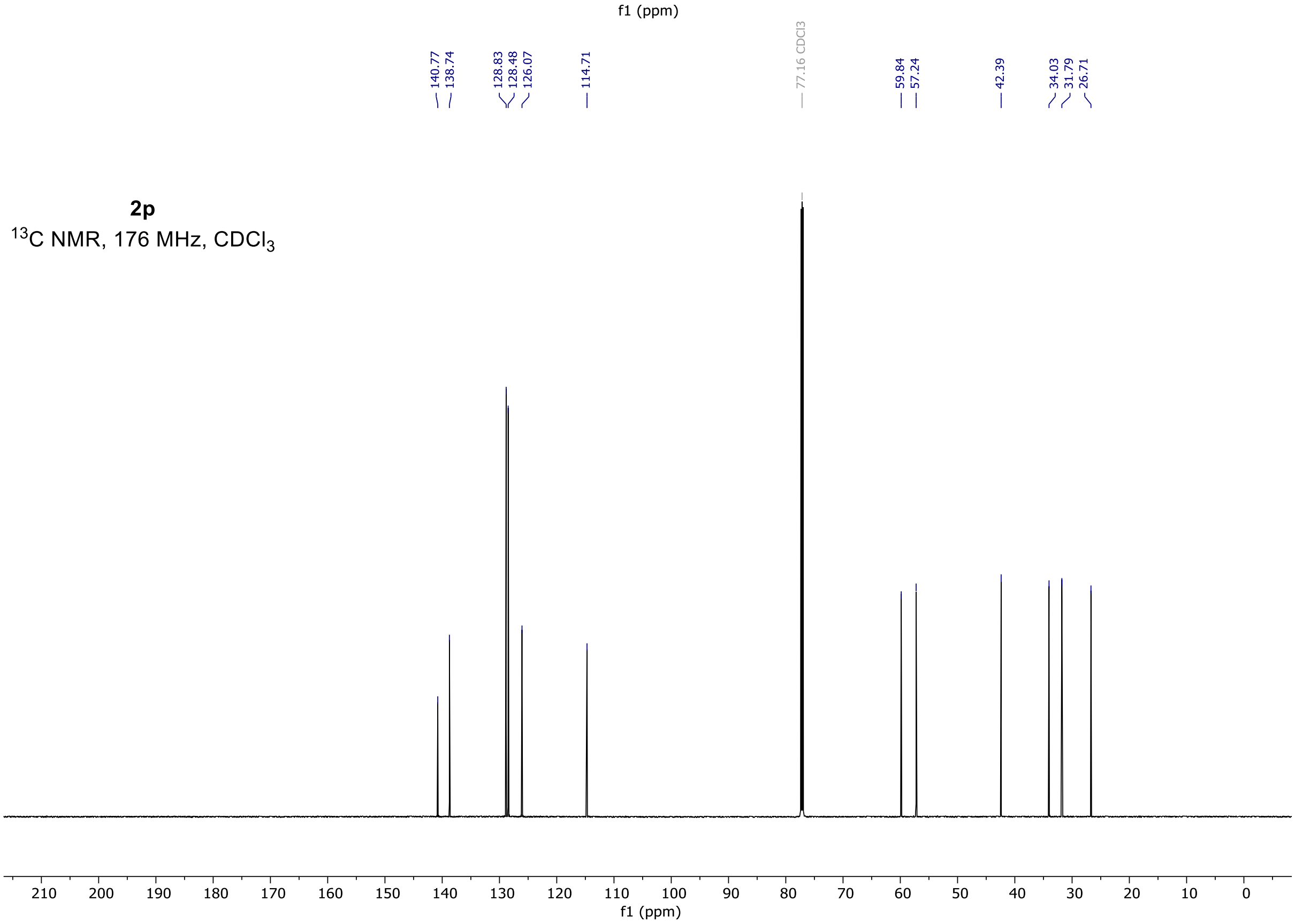
2p
^{13}C NMR, 176 MHz, $CDCl_3$
140.77
138.74
128.83
128.48
126.07
114.71
77.16 CDCl3
59.84
57.24
42.39
34.03
31.79
26.71
f1 (ppm)

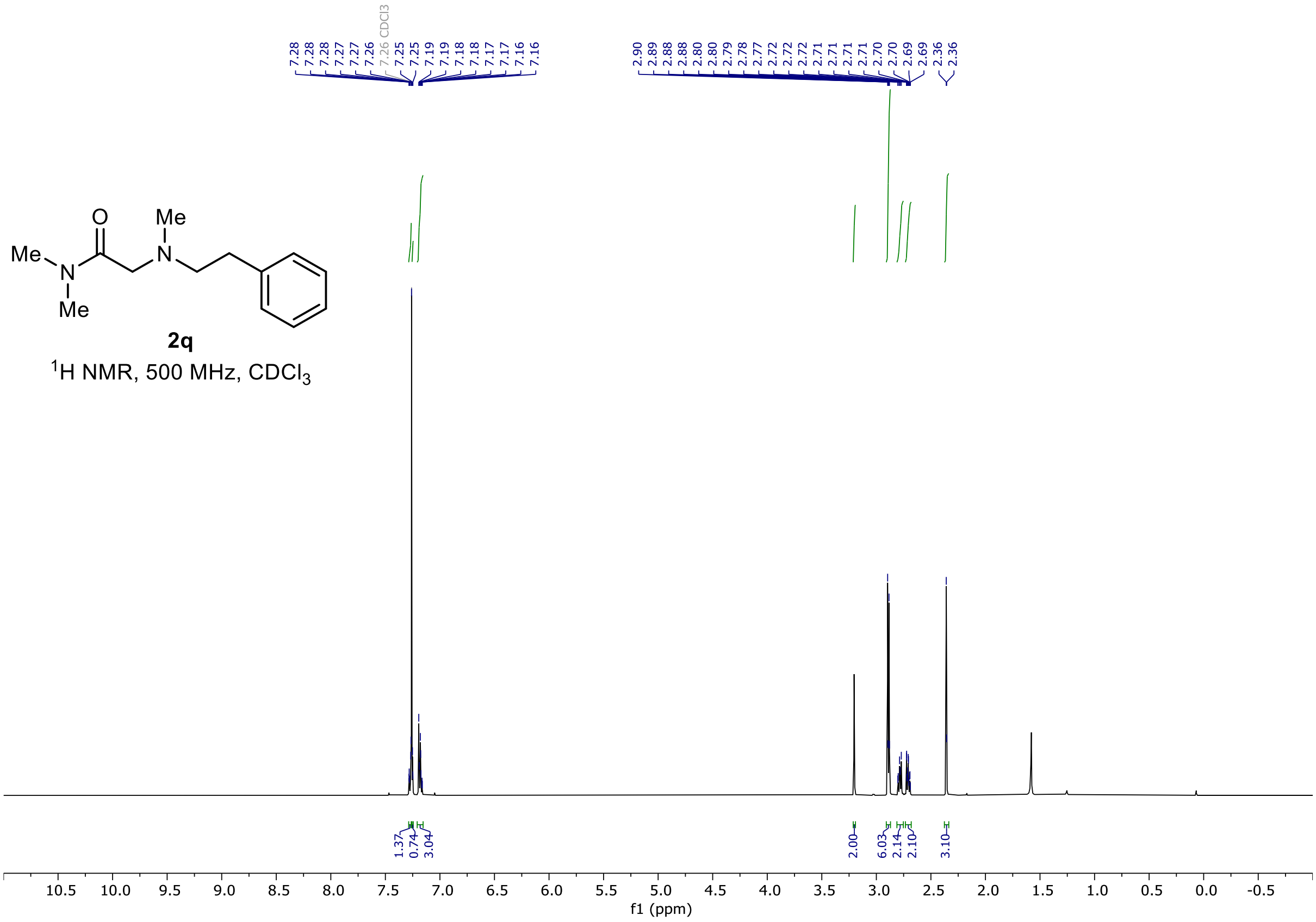

2q

^{1}H NMR, 500 MHz, $CDCl_3$

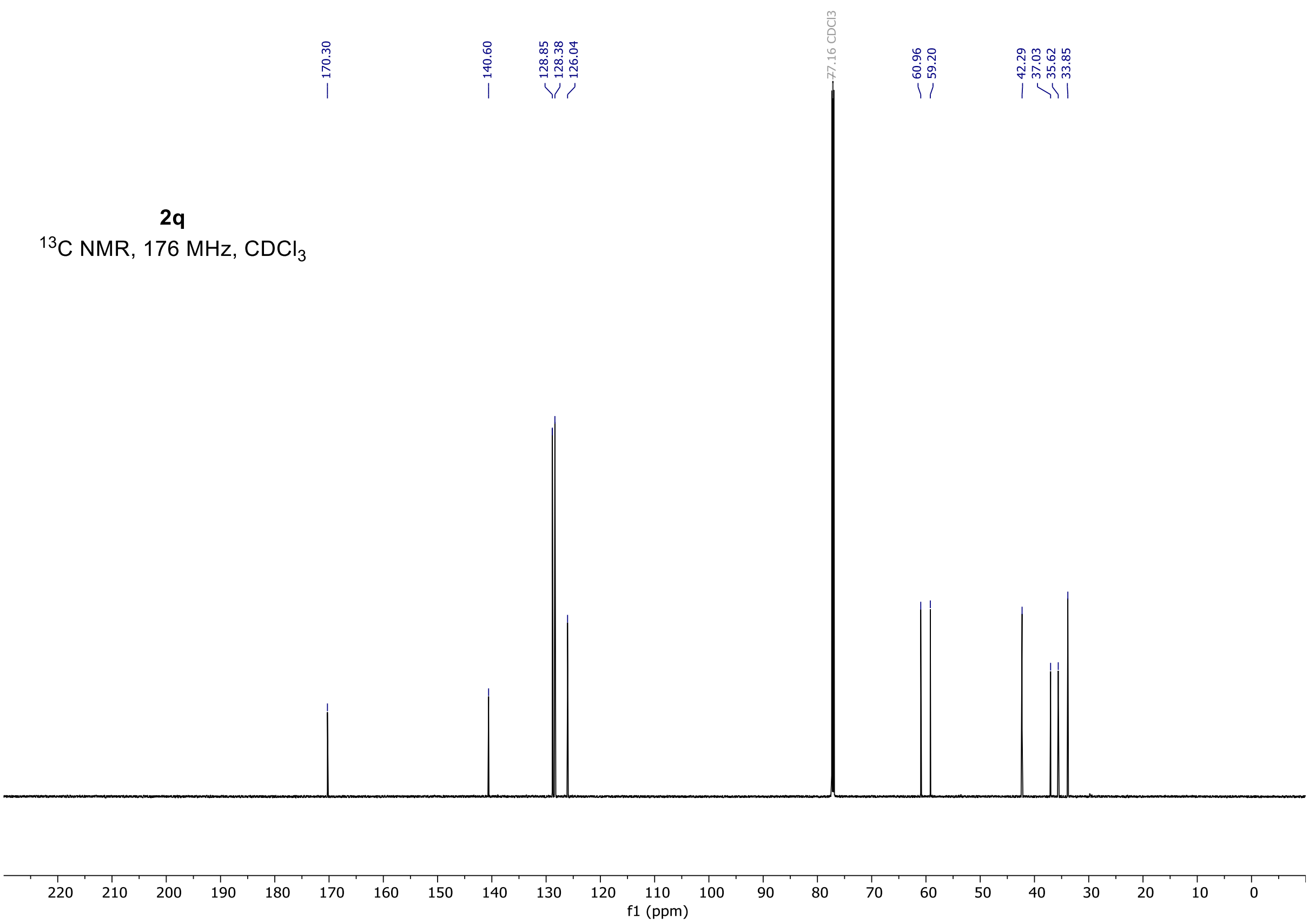

2q

^{13}C NMR, 176 MHz, $CDCl_3$

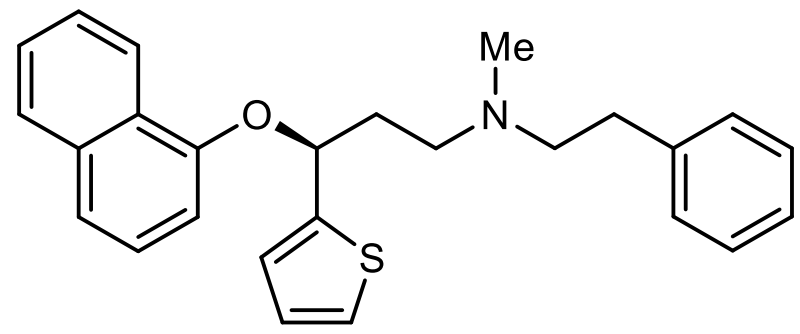

2r

^{1}H NMR, 700 MHz, $CDCl_3$

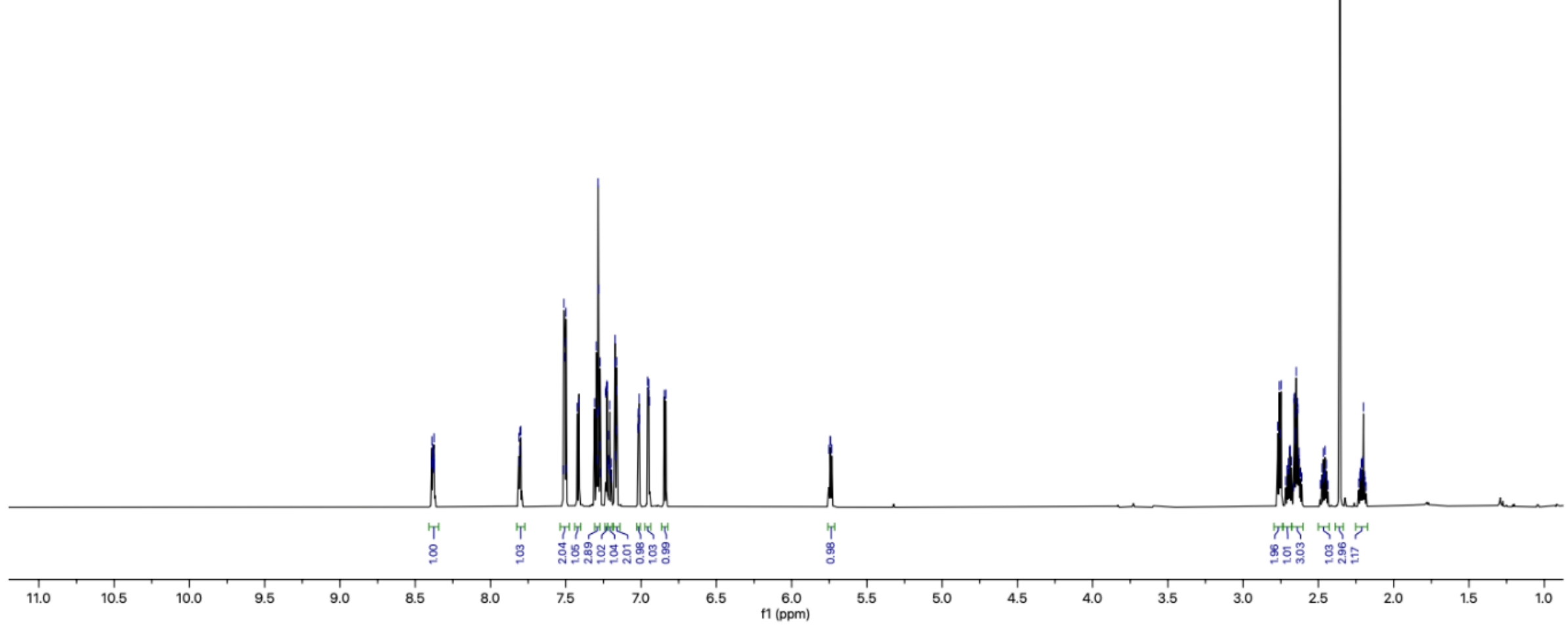

2r

^{13}C NMR, 176 MHz, $CDCl_3$

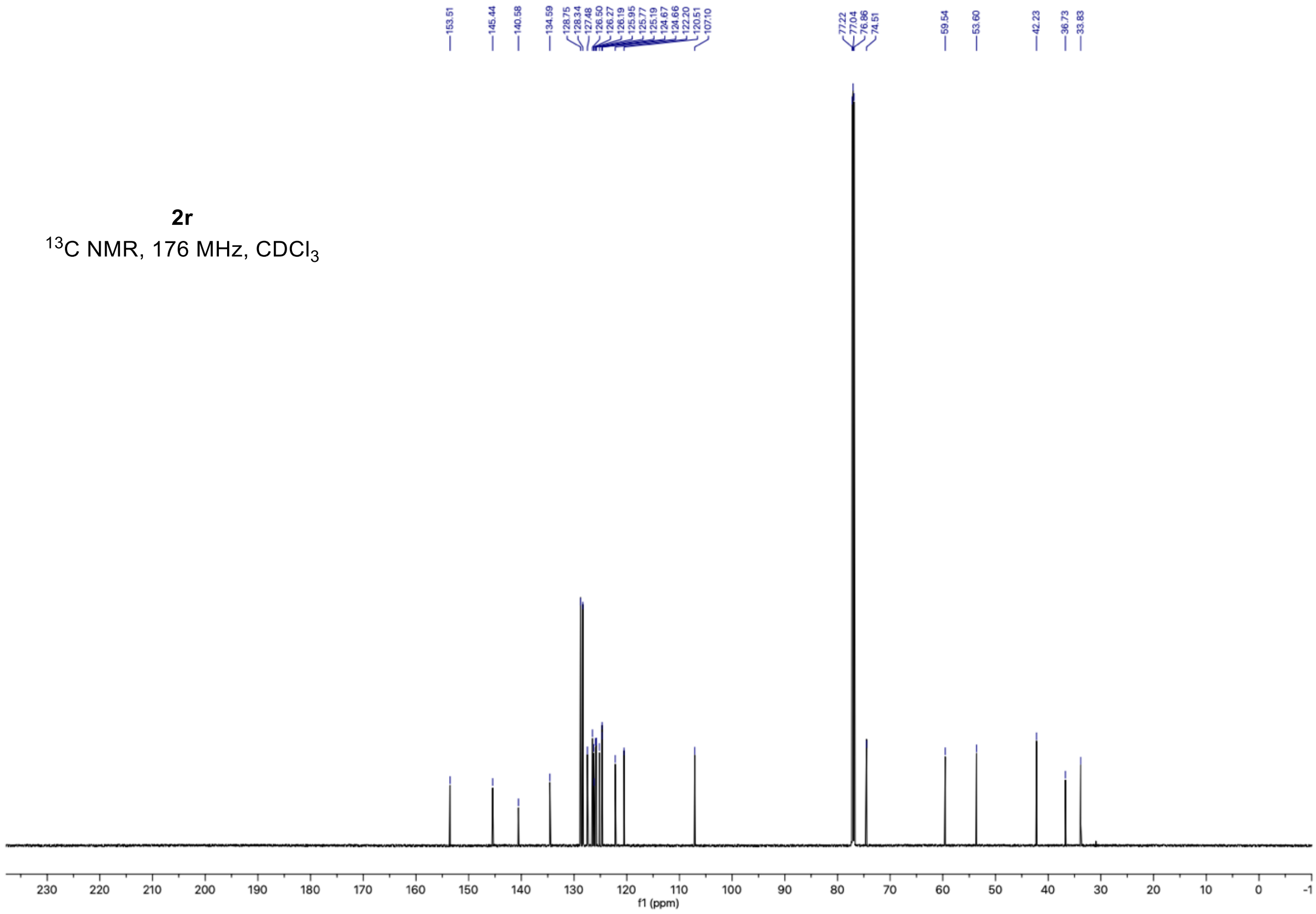

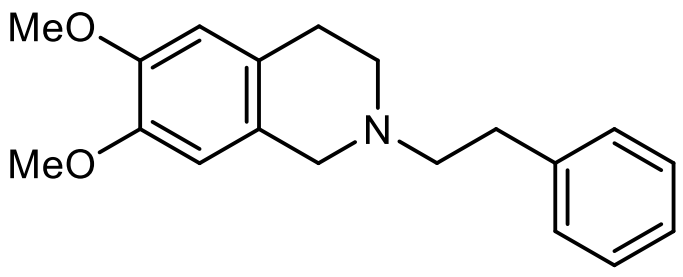

2s

^{1}H NMR, 700 MHz, $CDCl_3$

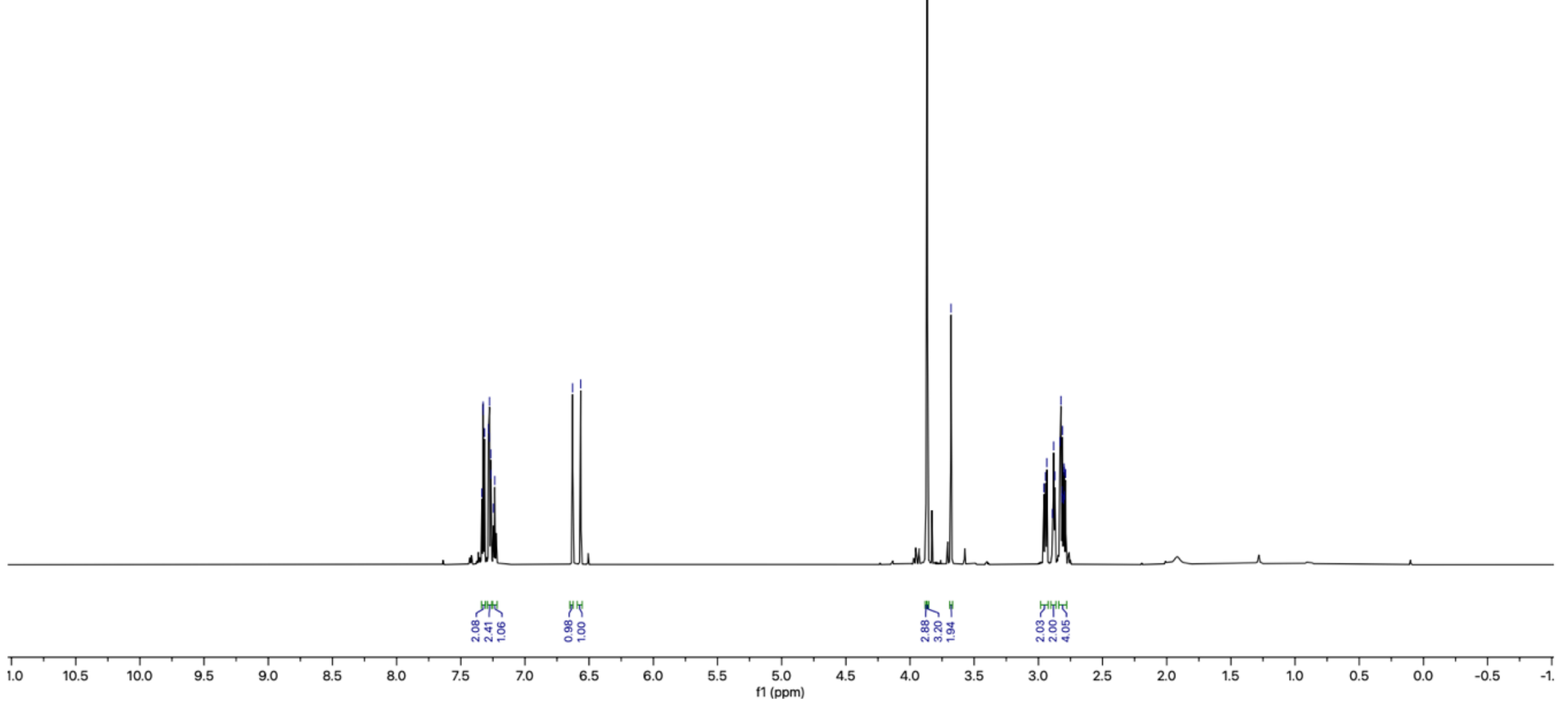

2s

^{13}C NMR, 176 MHz, $CDCl_3$

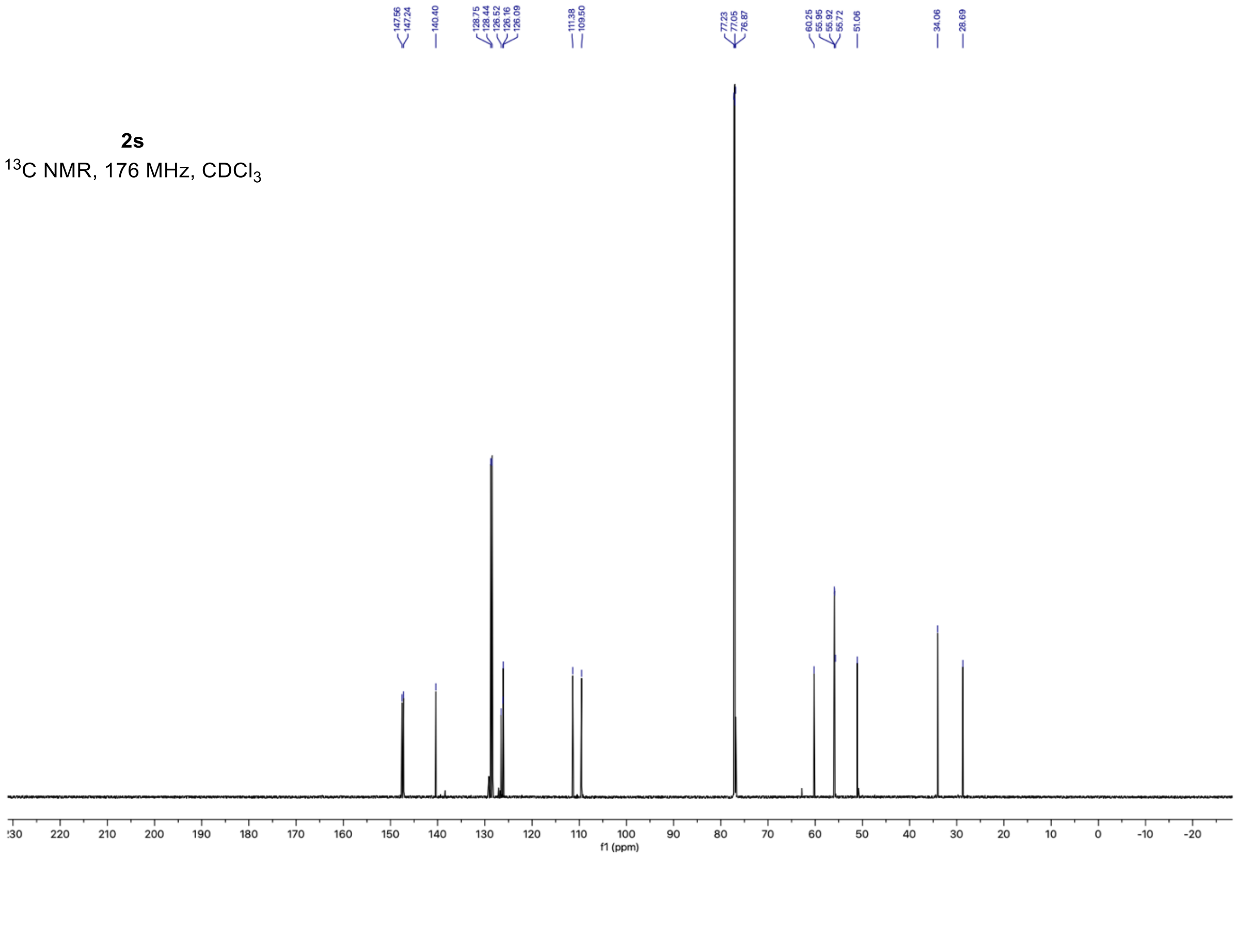

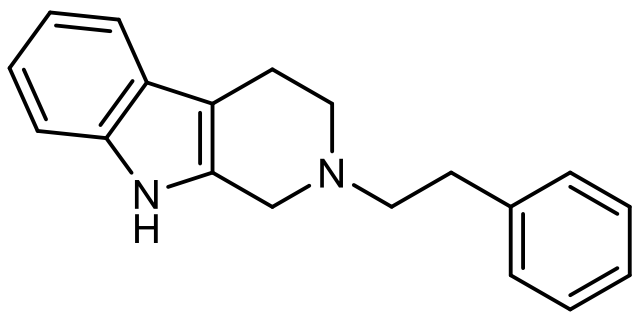

2t

^{1}H NMR, 500 MHz, $CDCl_3$

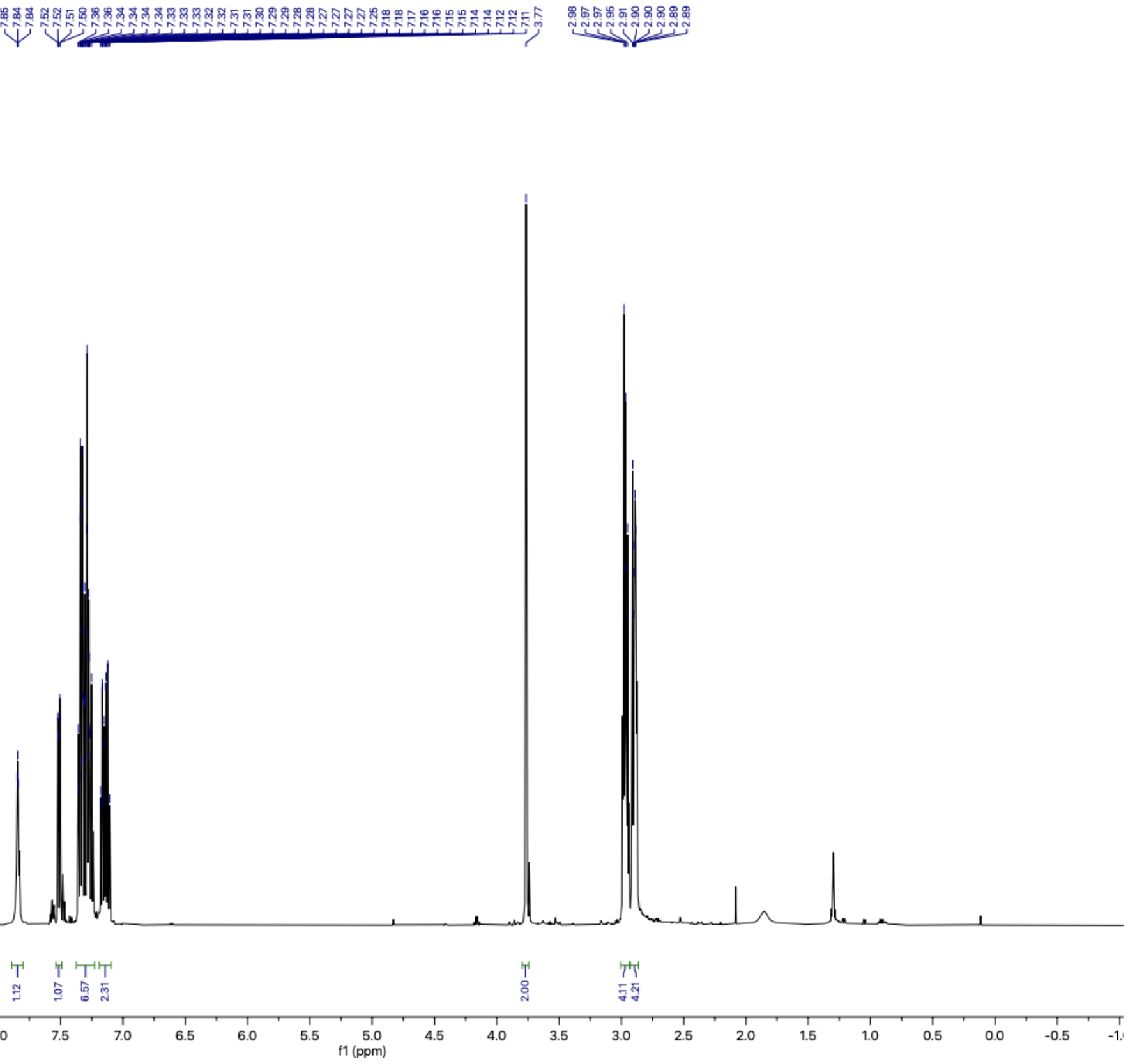

2t

^{13}C NMR, 126 MHz, $CDCl_3$

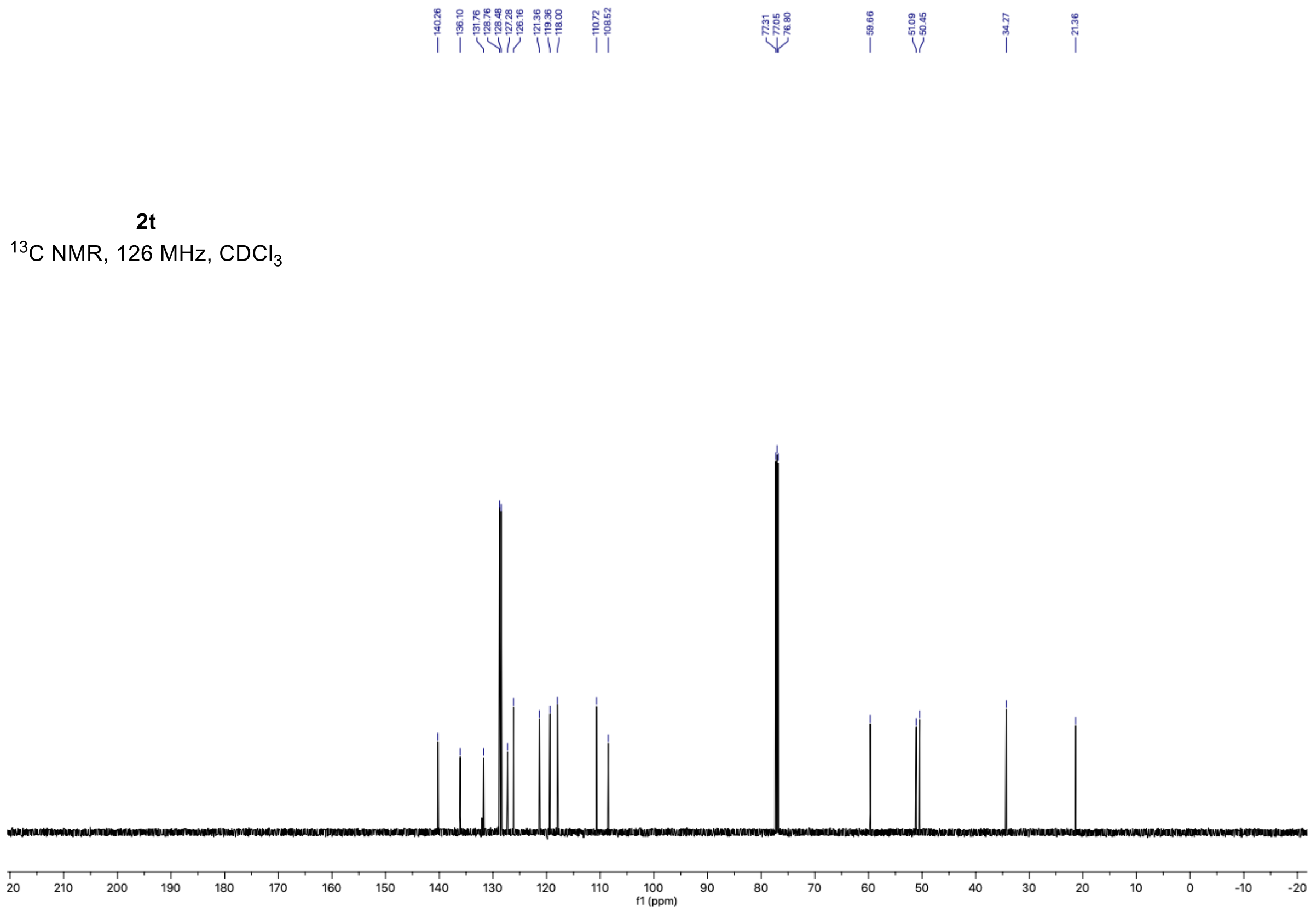

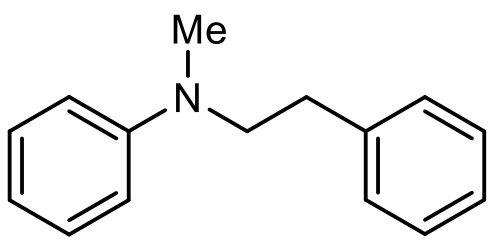

2u

^{1}H NMR, 500 MHz, $CDCl_3$

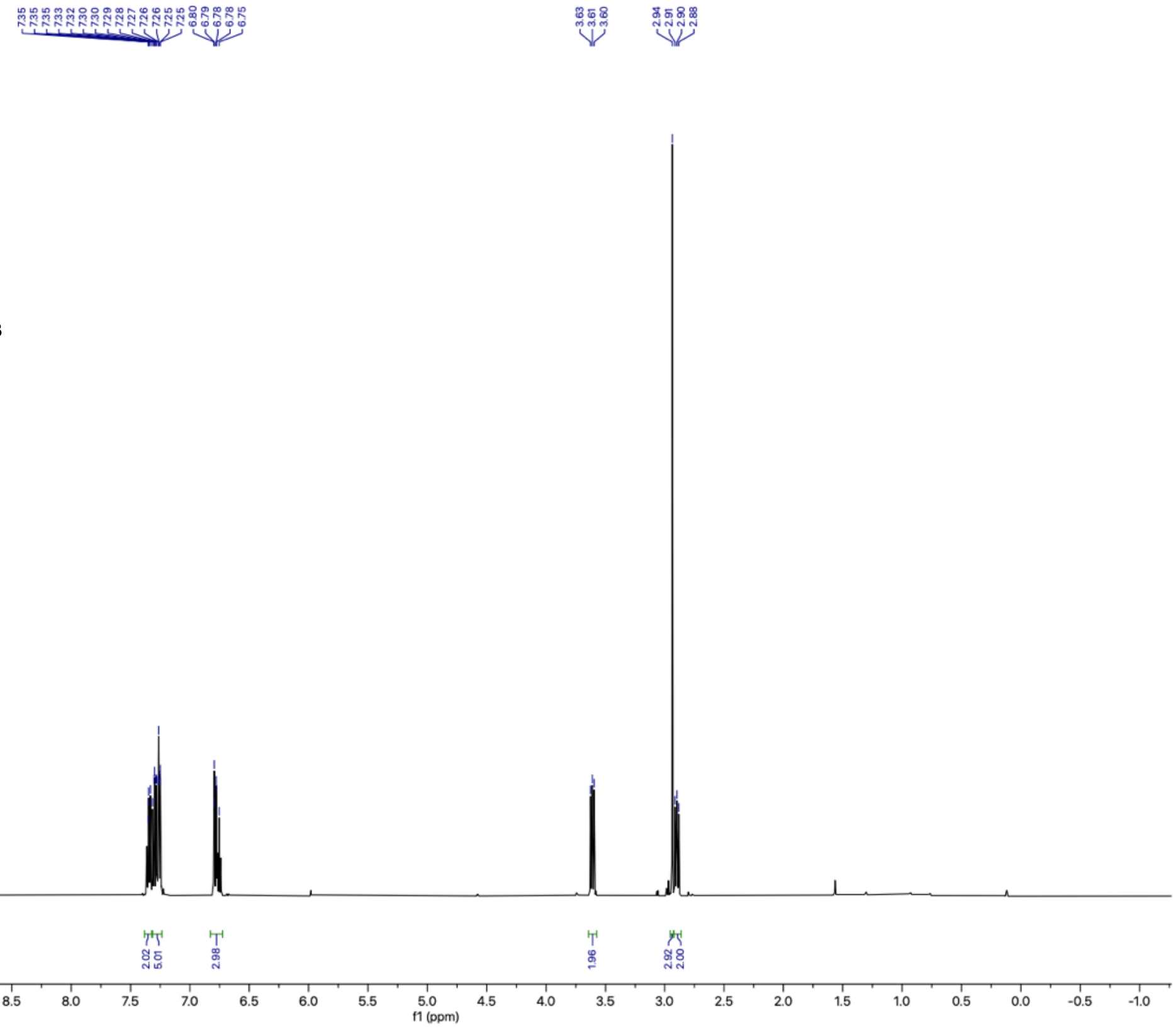

2u

^{13}C NMR, 126 MHz, $CDCl_3$

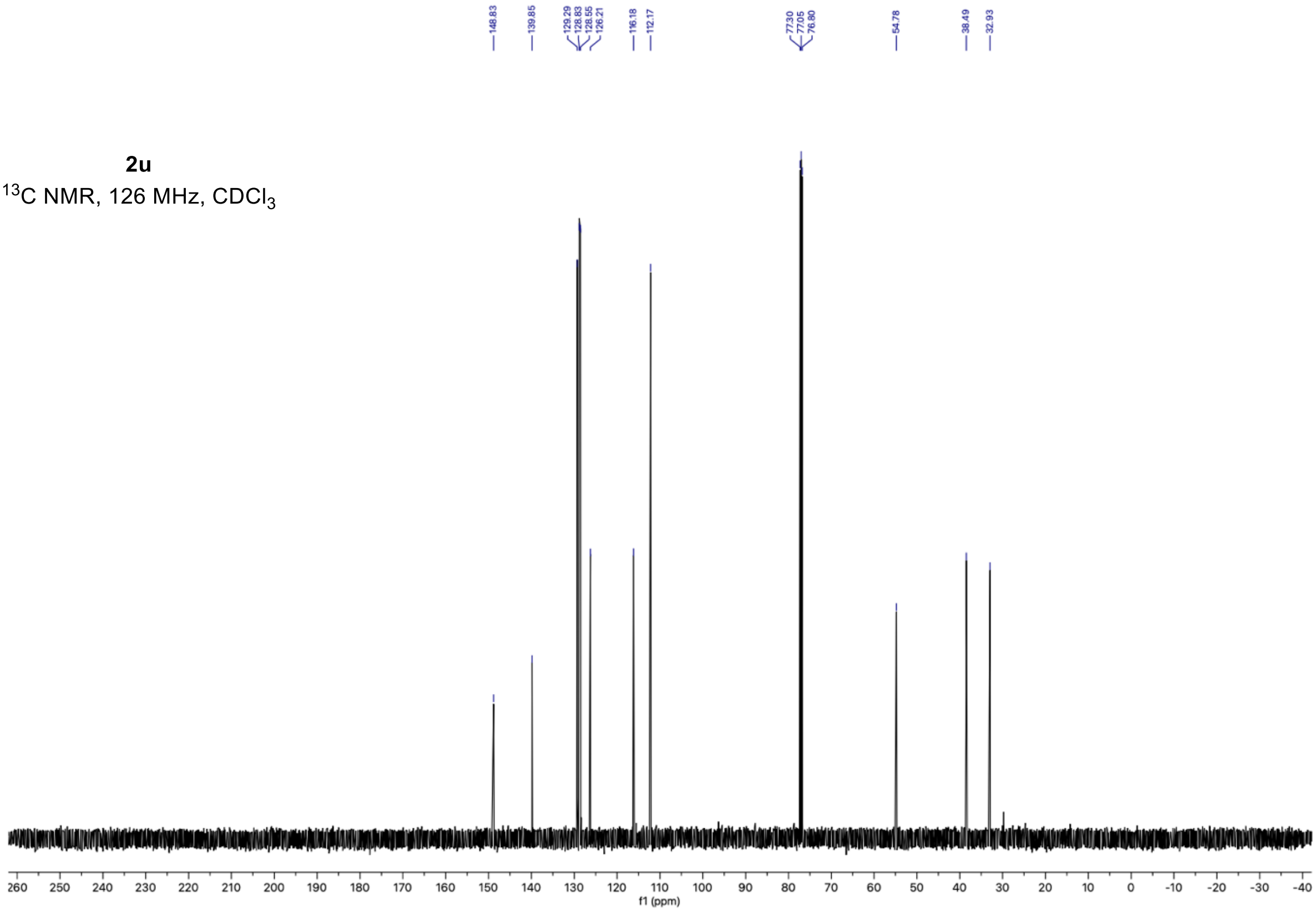

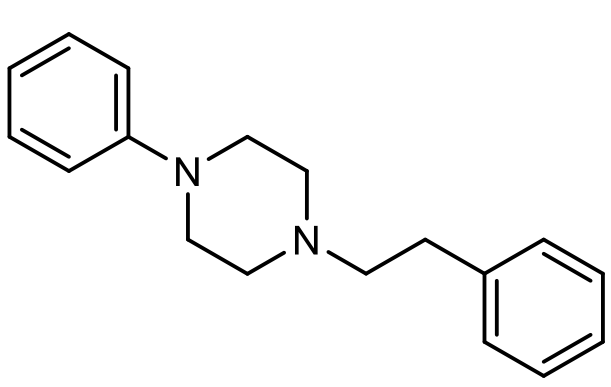

2v

^{1}H NMR, 700 MHz, $CDCl_3$

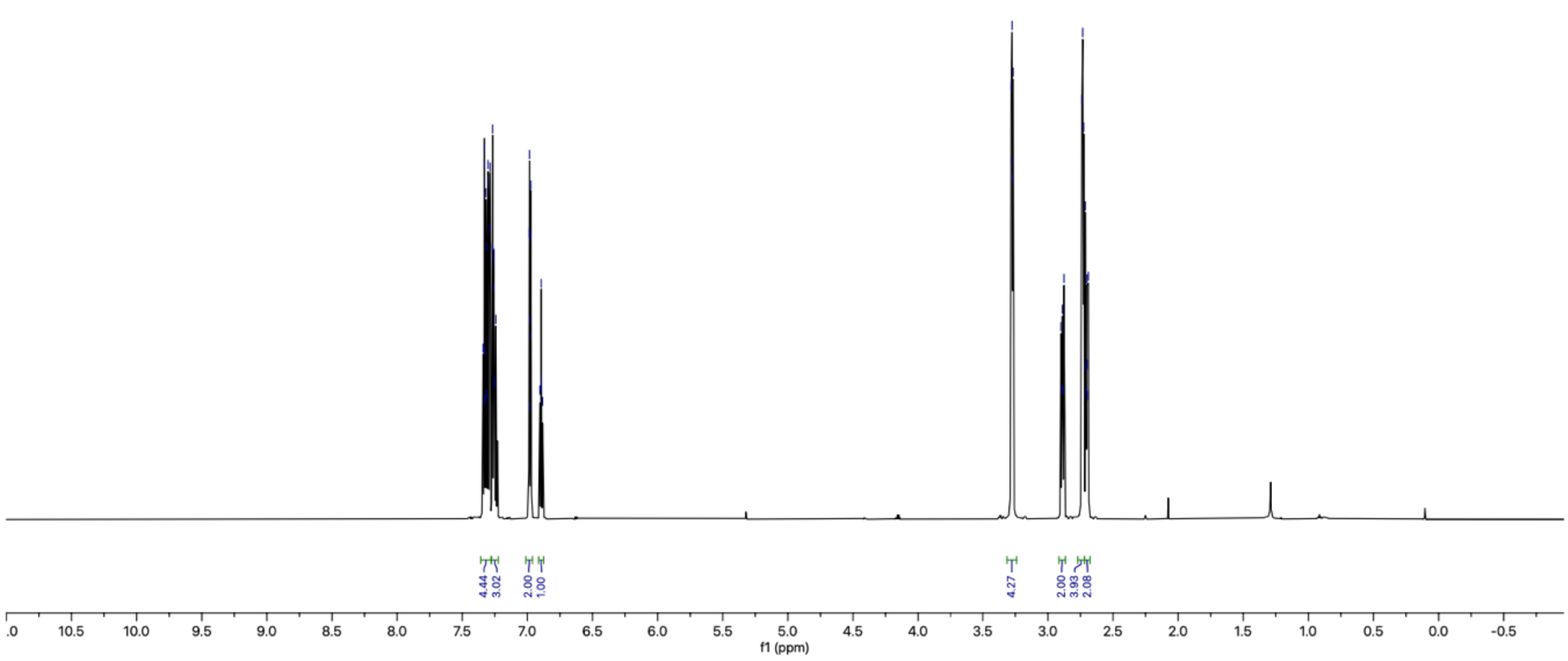

2v

^{13}C NMR, 176 MHz, $CDCl_3$

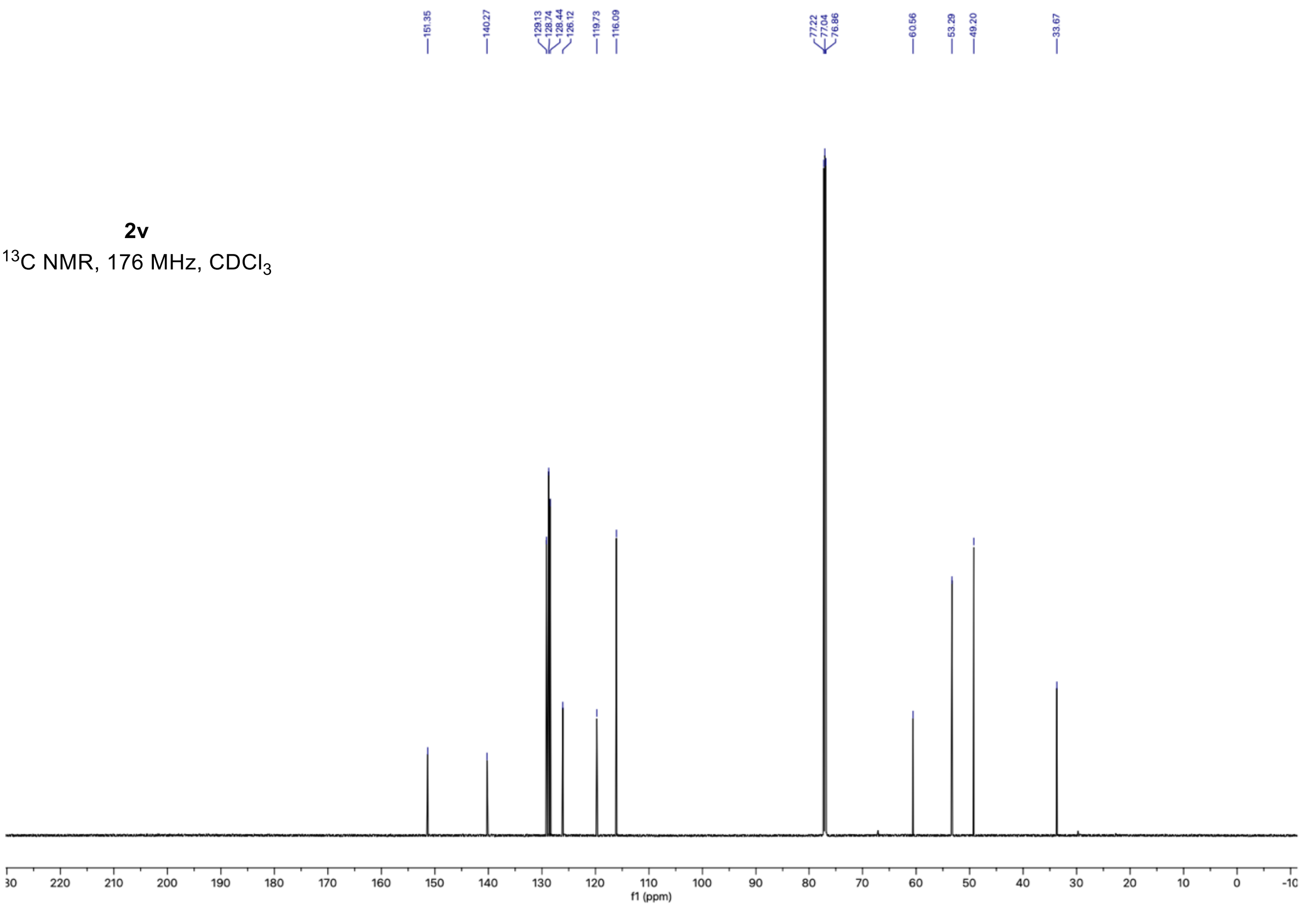

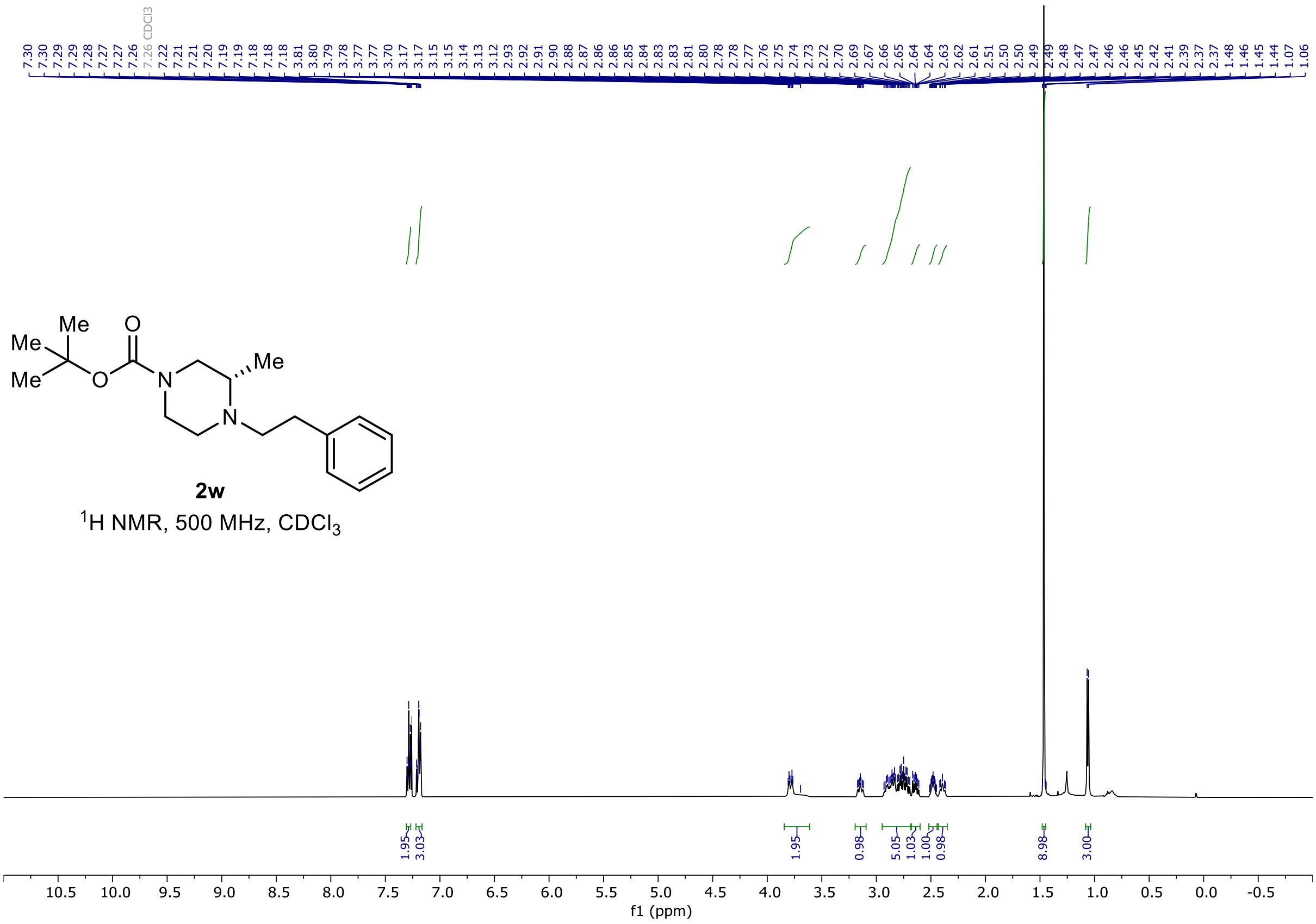

2w
^{1}H NMR, 500 MHz, CDCl$_3$

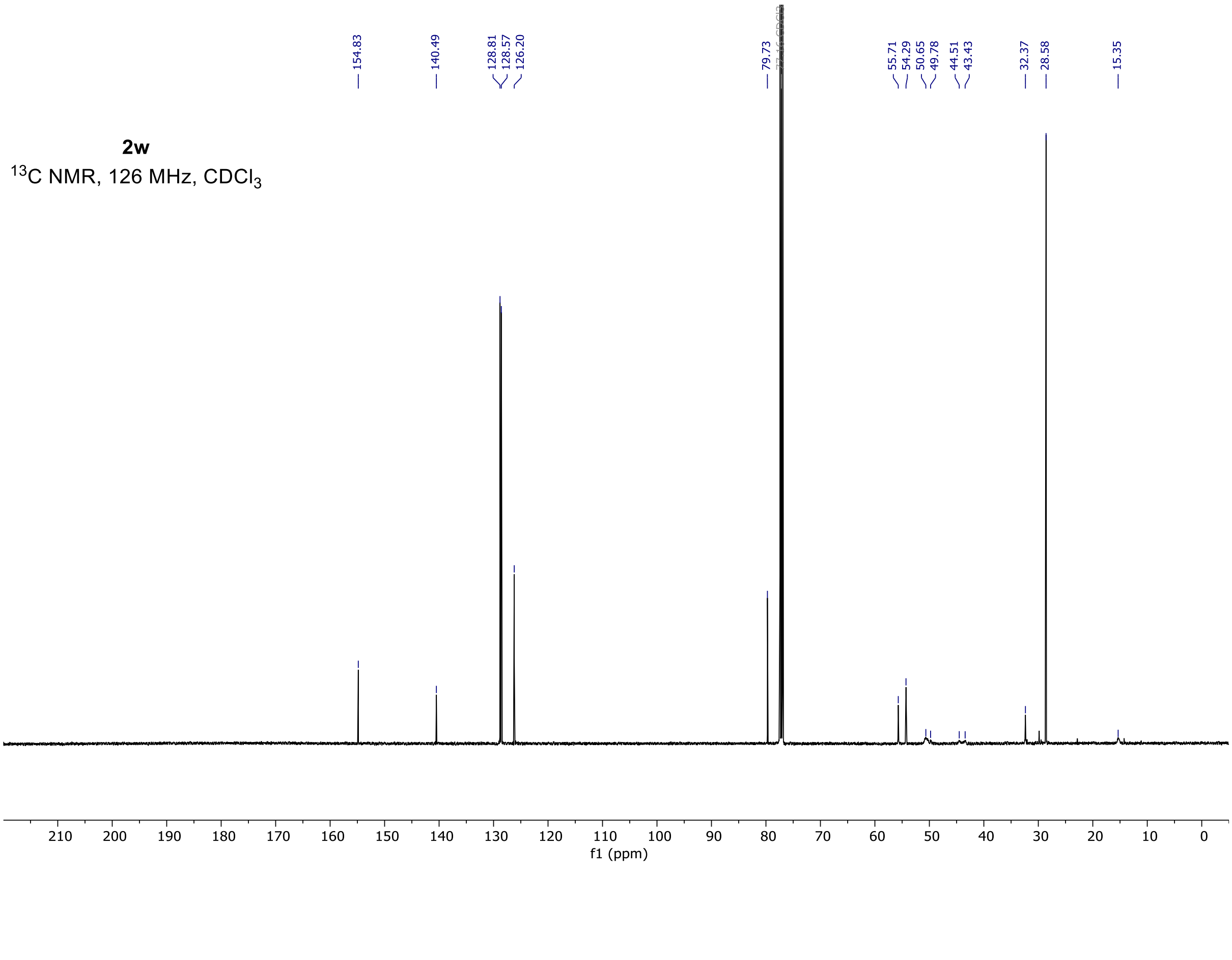

2w
^{13}C NMR, 126 MHz, CDCl$_3$

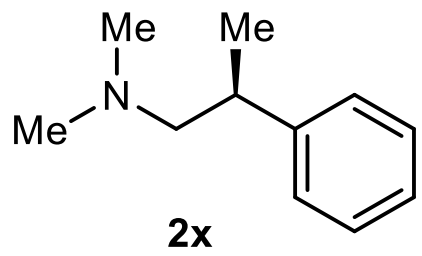

2x

^{1}H NMR, 500 MHz, $CDCl_3$

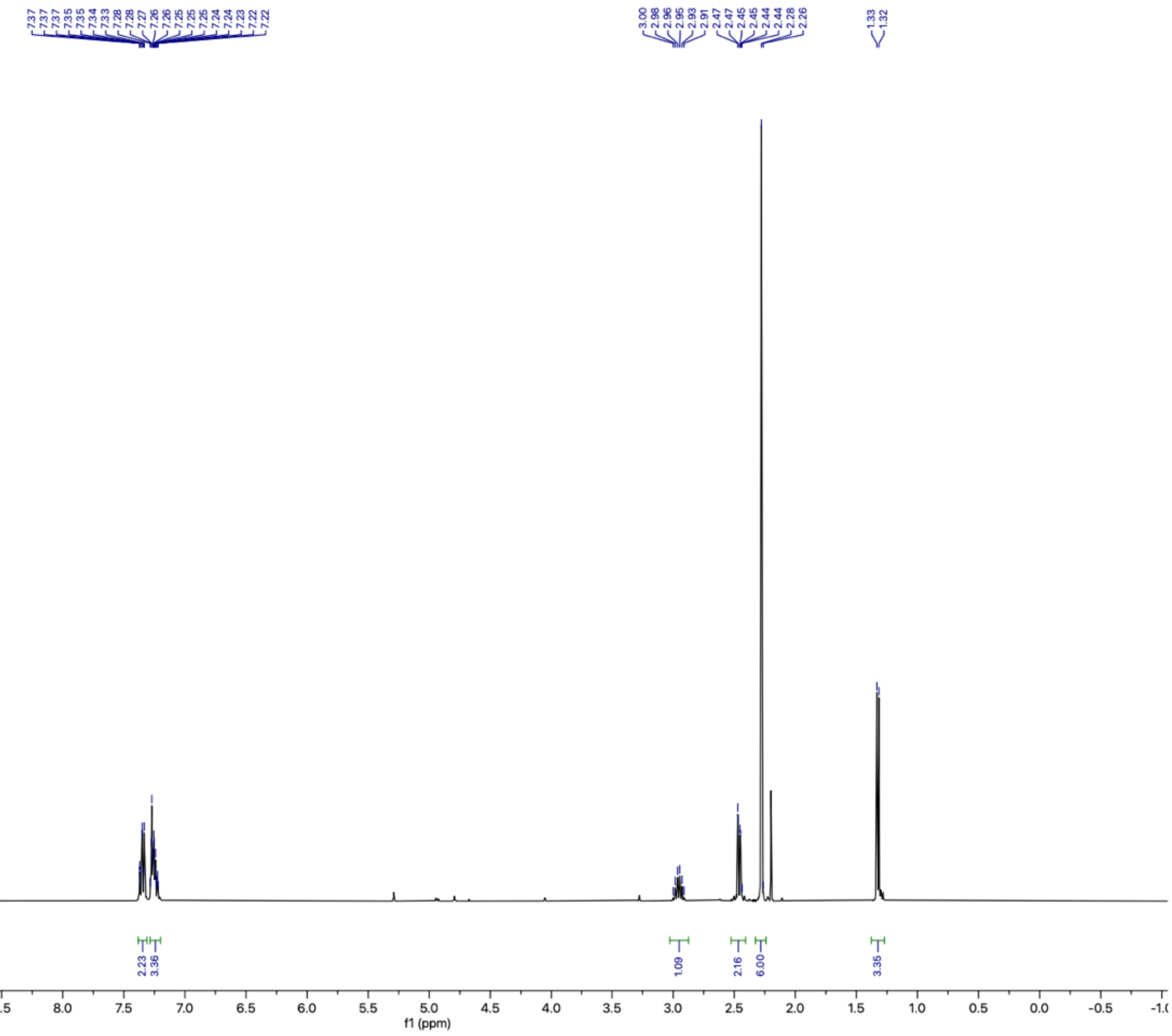

2x

^{13}C NMR, 176 MHz, $CDCl_3$

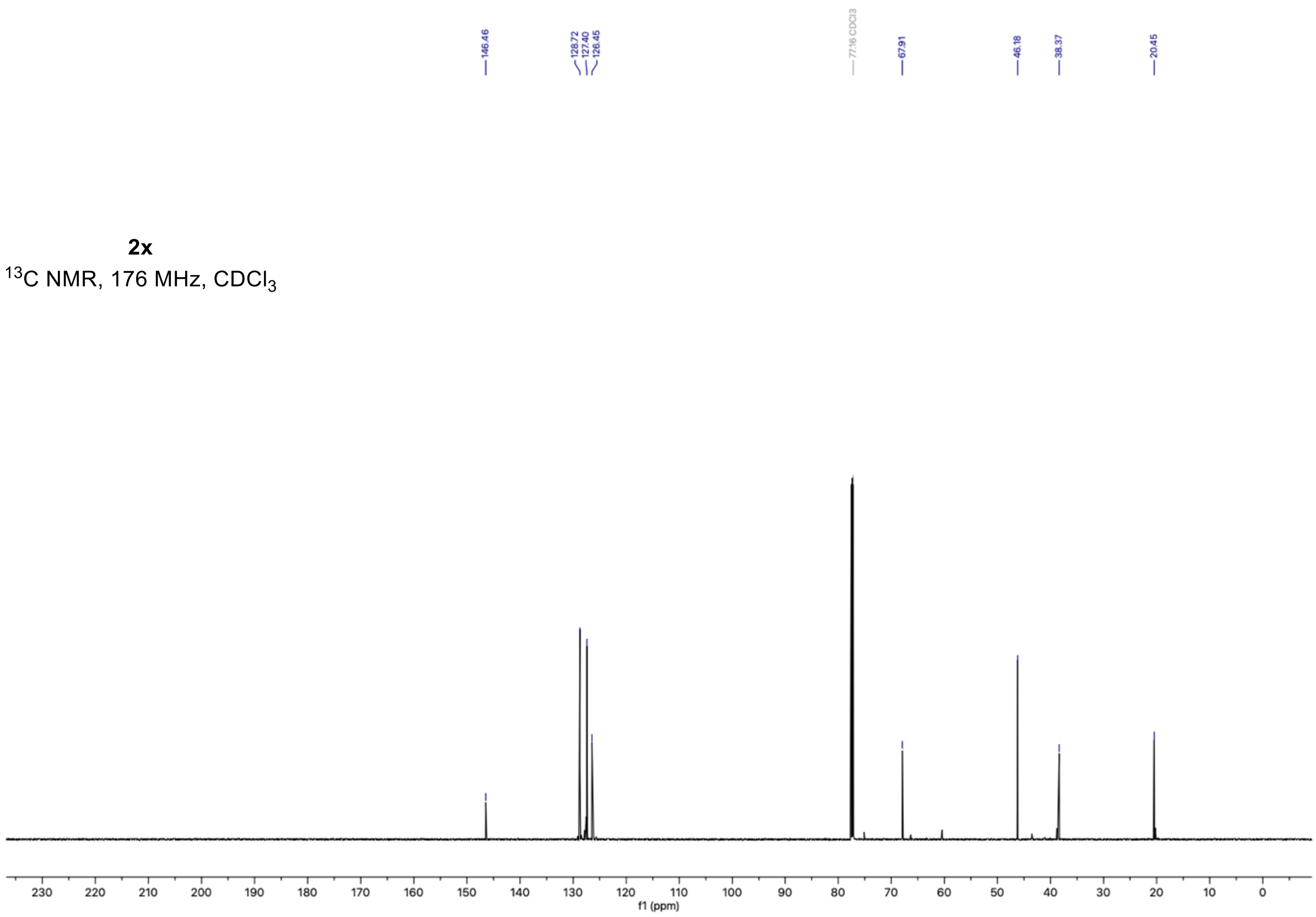

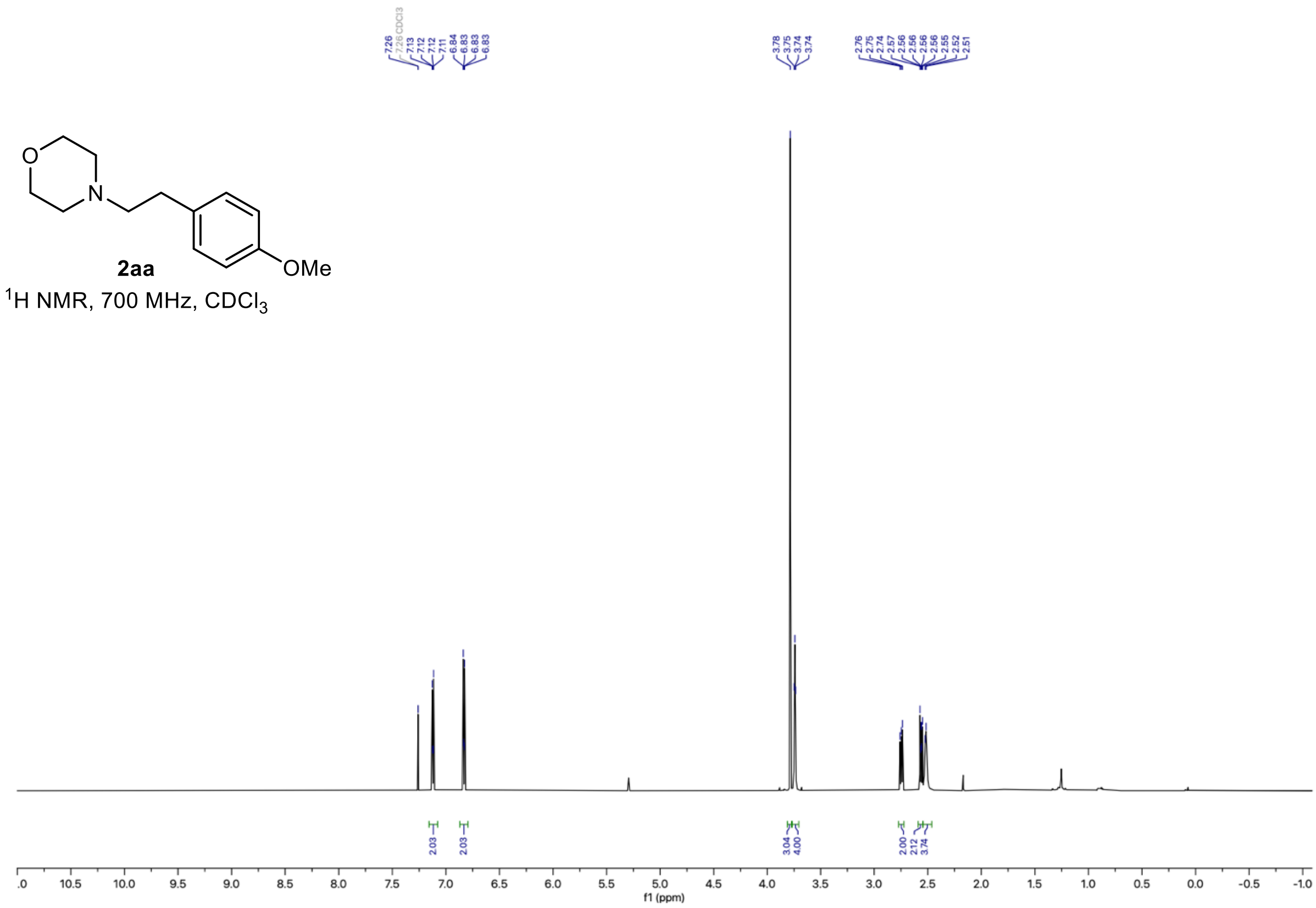

2aa
^{1}H NMR, 700 MHz, $CDCl_3$
OMe
f1 (ppm)

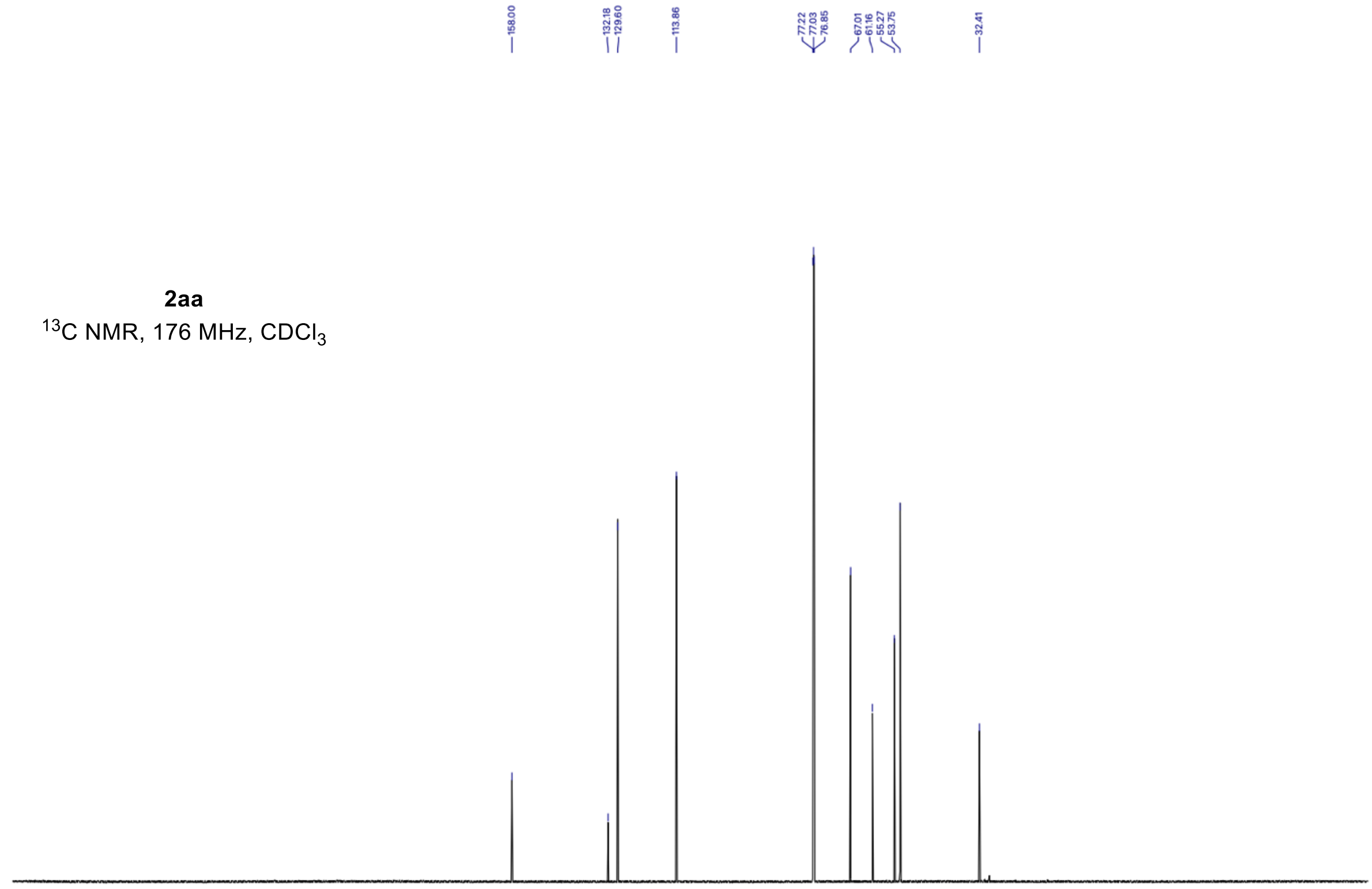

2aa
^{13}C NMR, 176 MHz, $CDCl_3$
f1 (ppm)

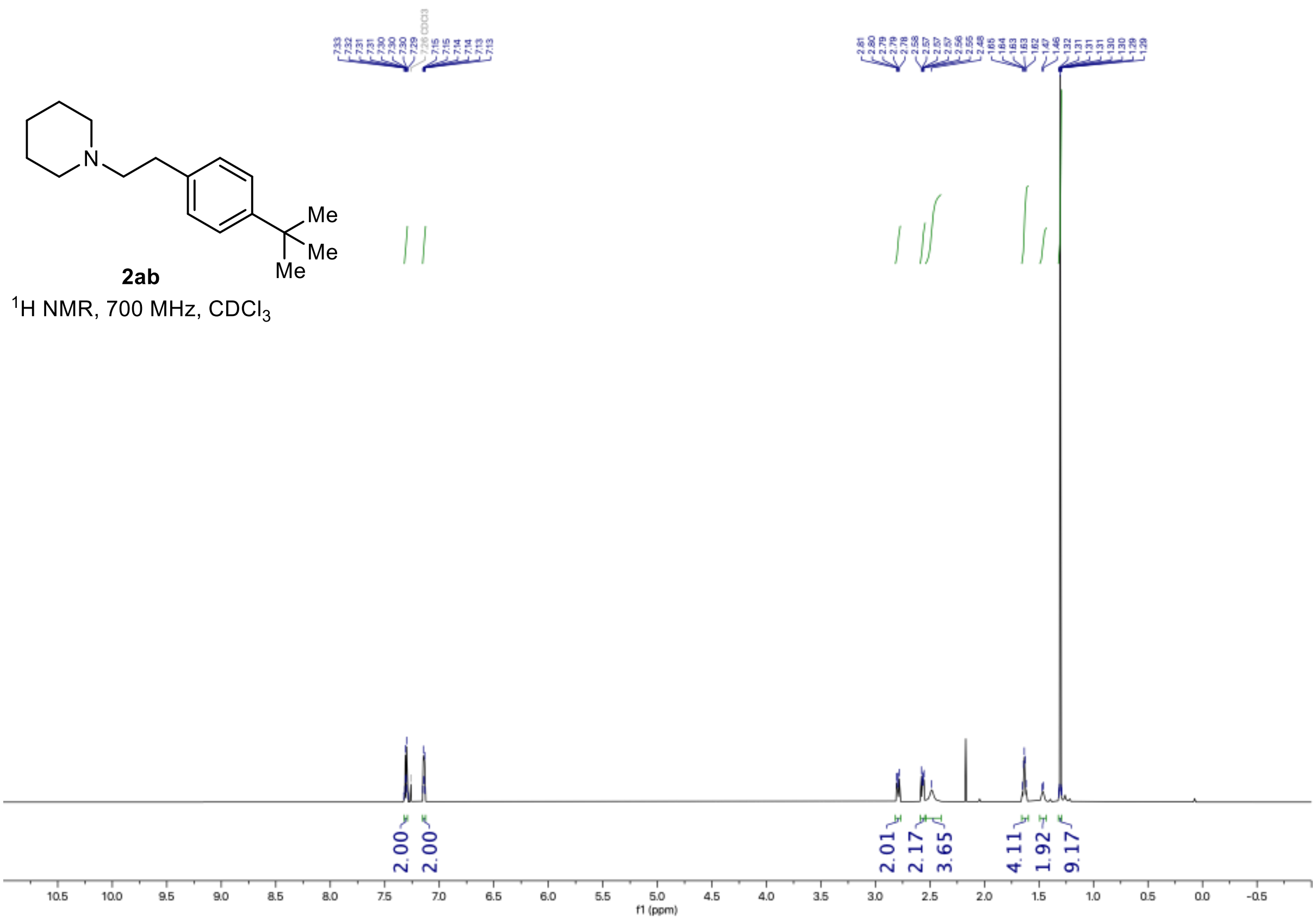

2ab

^1H NMR, 700 MHz, $CDCl_3$

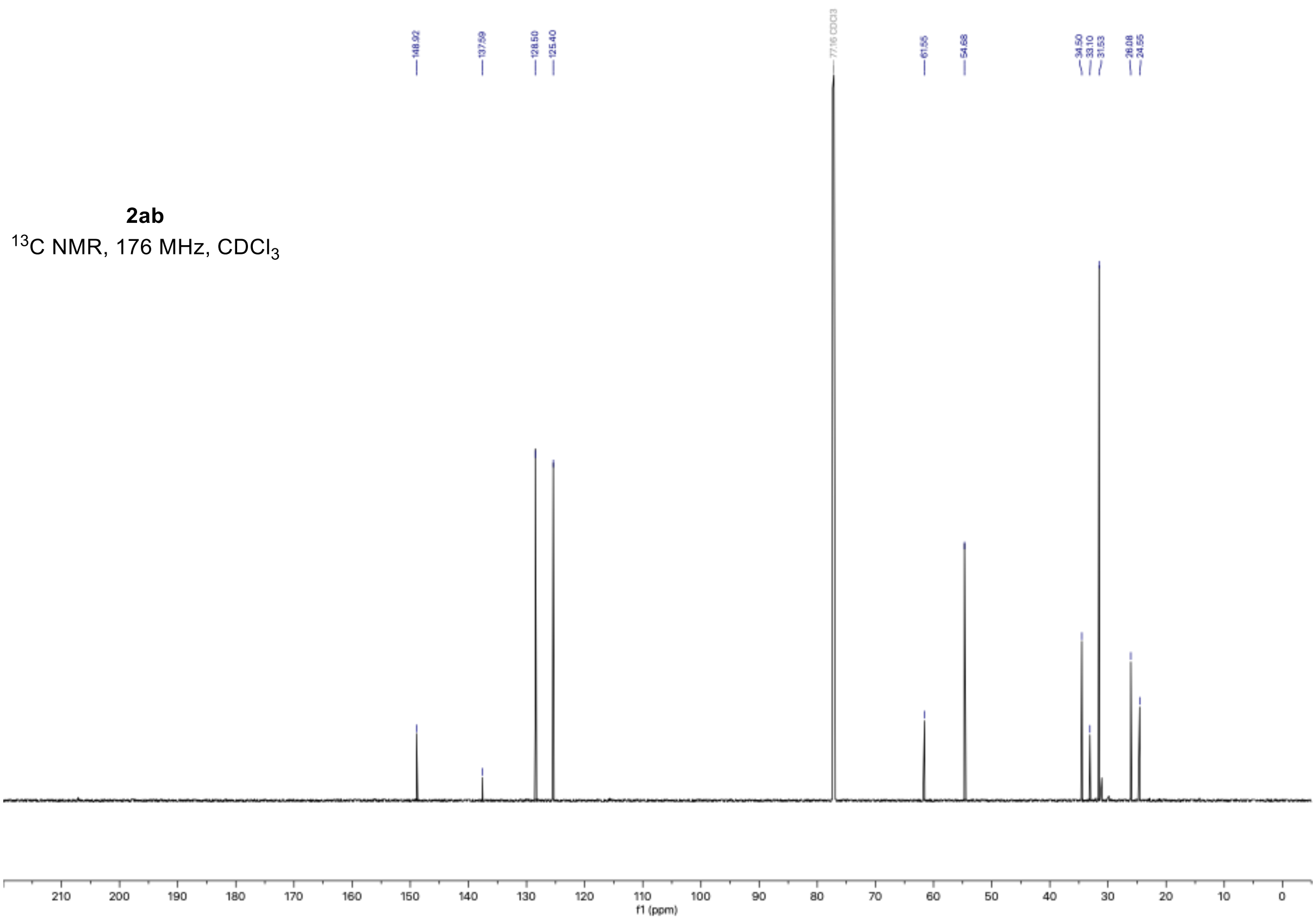

2ab

^{13}C NMR, 176 MHz, $CDCl_3$

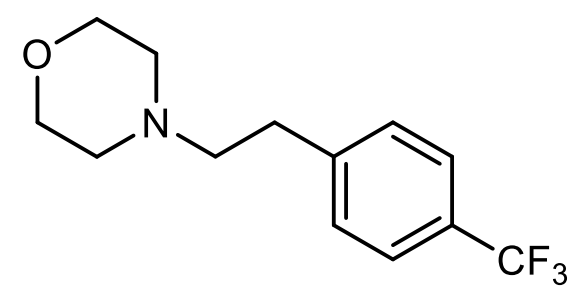

2ac

^1H NMR, 500 MHz, $CDCl_3$

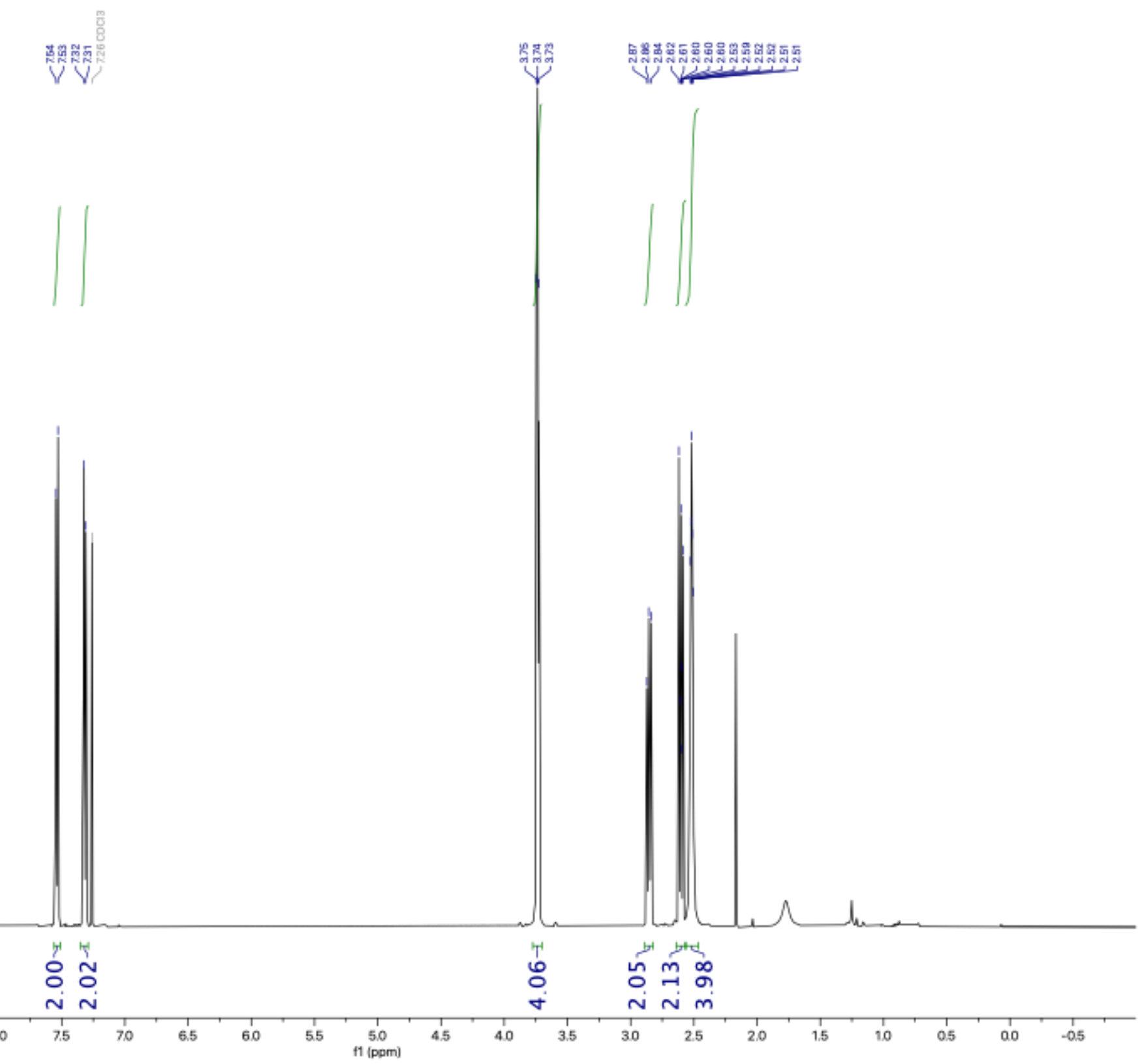

2ac

^{13}C NMR, 101 MHz, $CDCl_3$

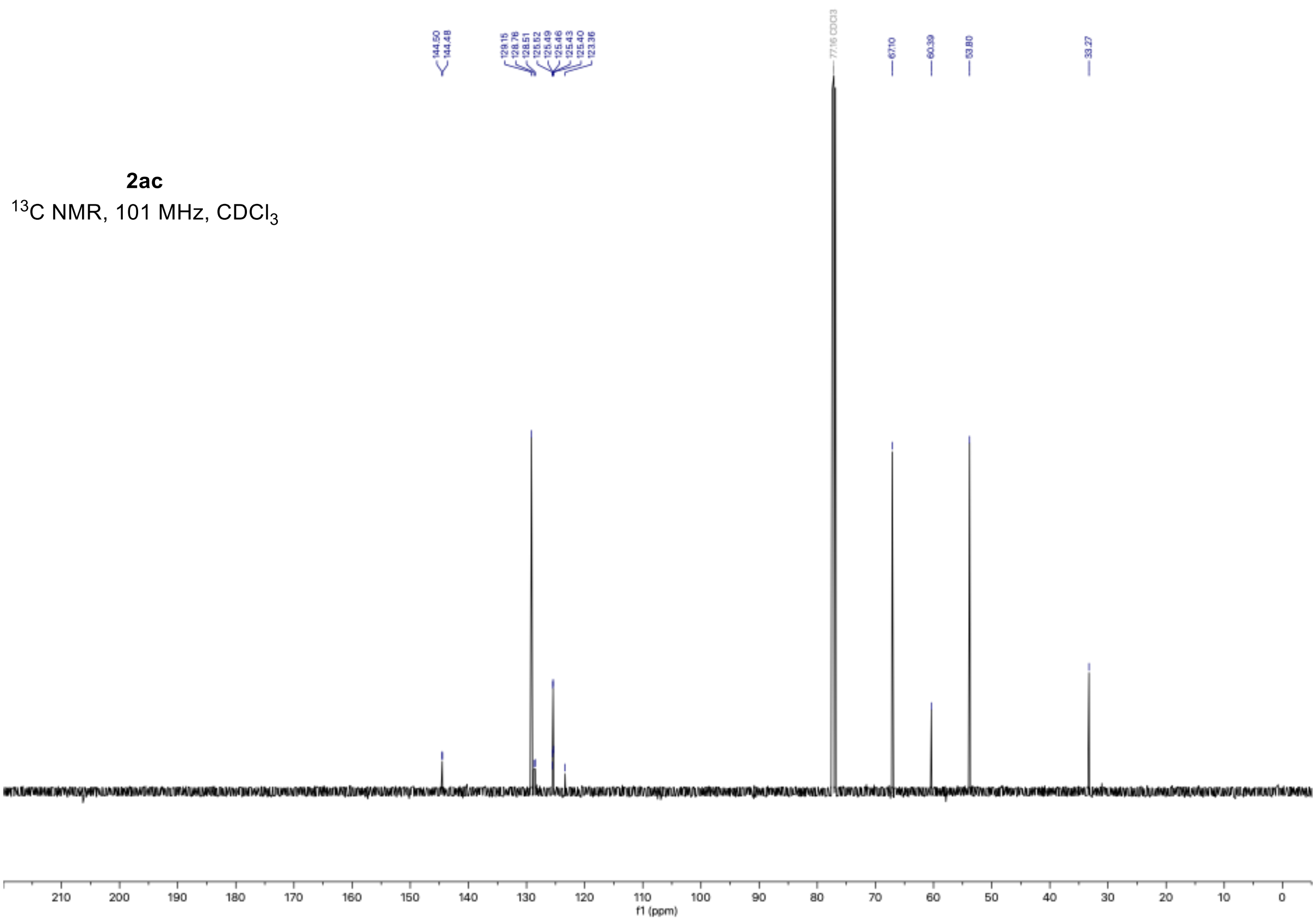

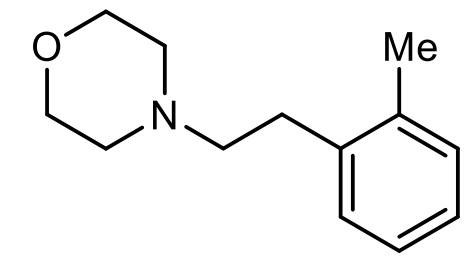

2ad

^{1}H NMR, 400 MHz, $CDCl_3$

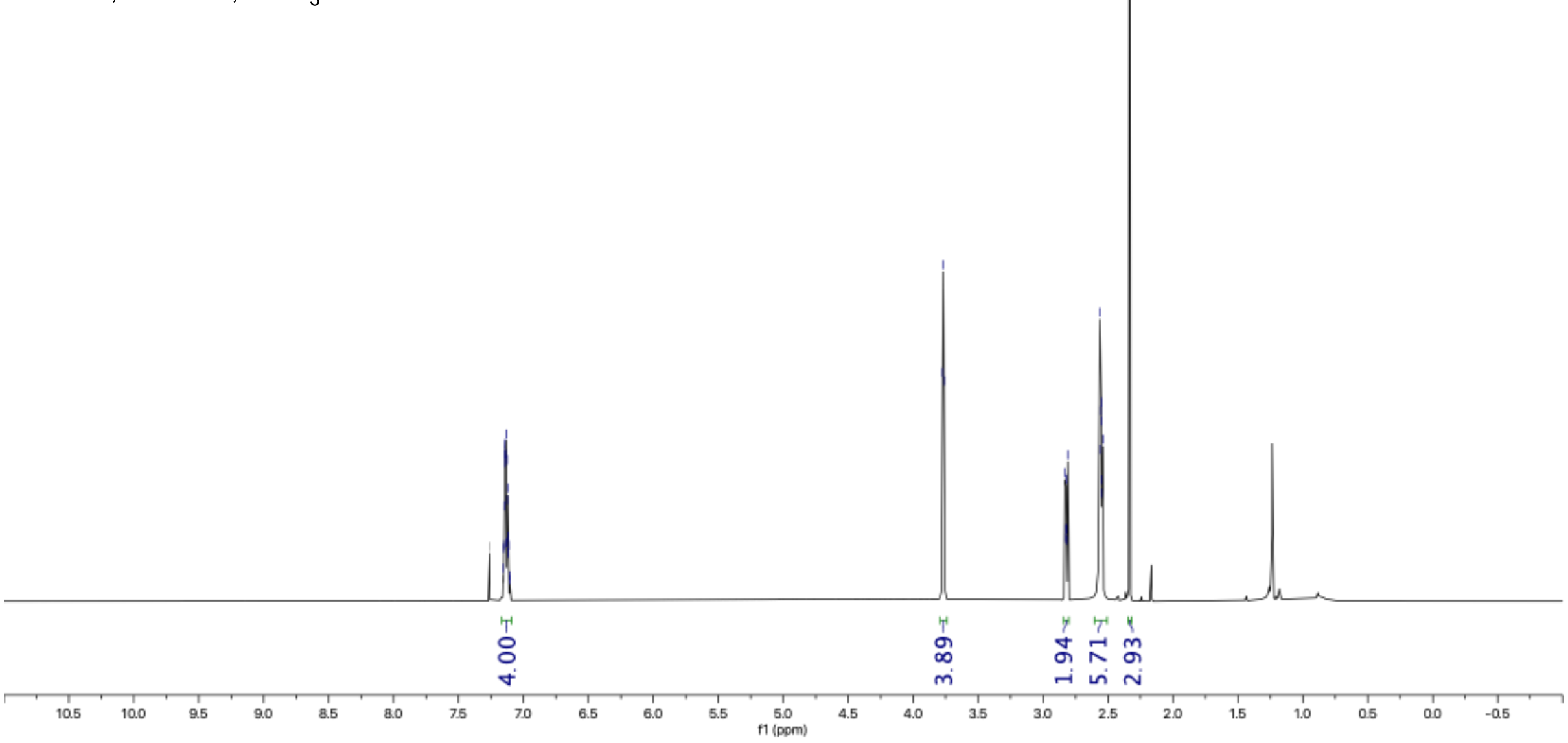

2ad

^{13}C NMR, 176 MHz, $CDCl_3$

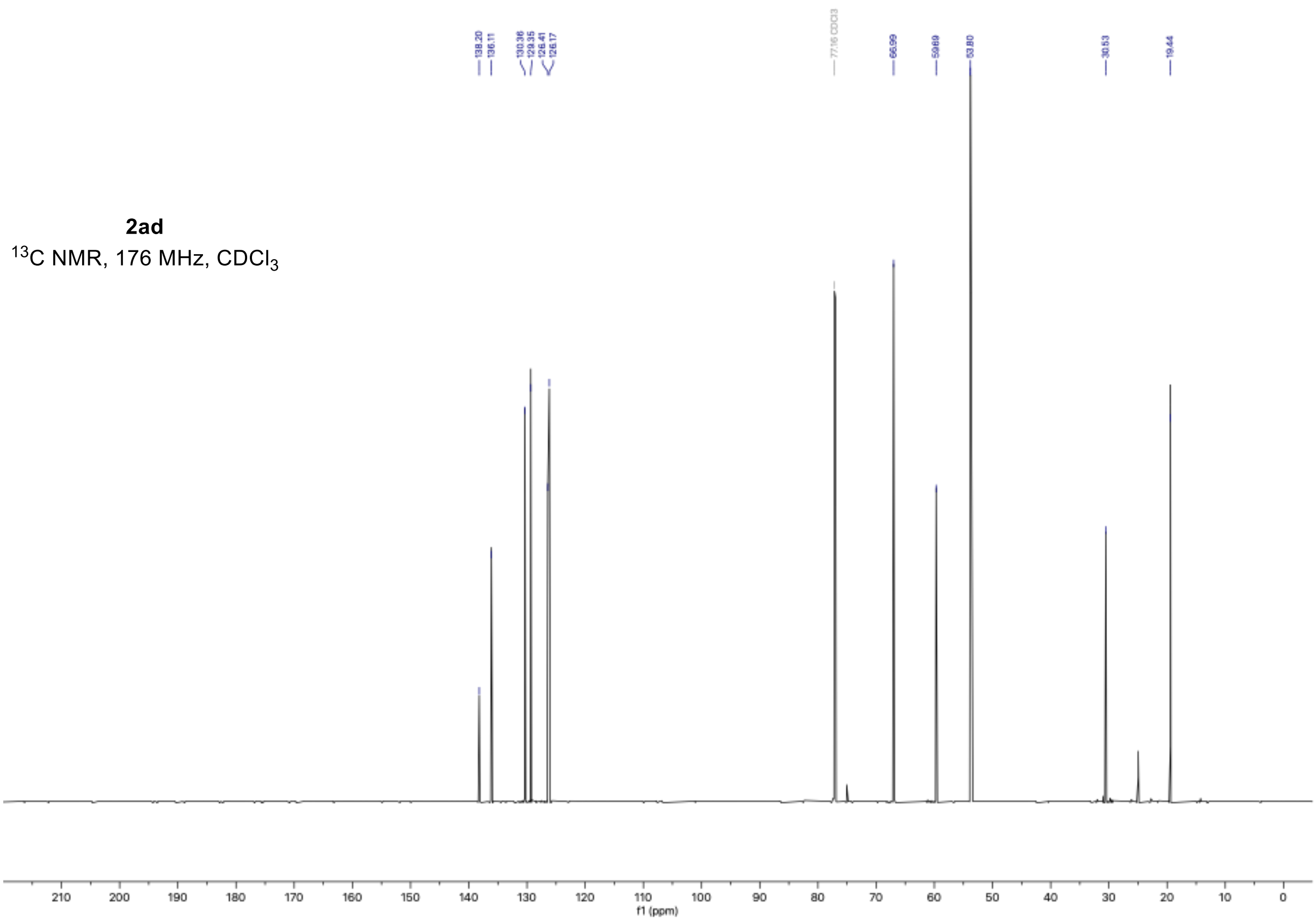

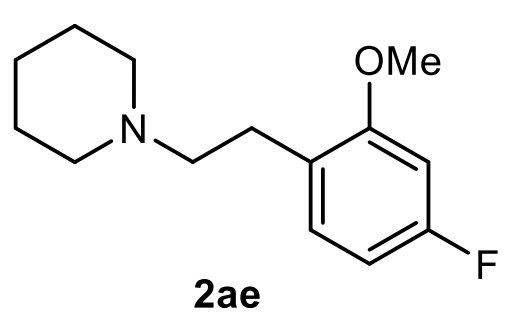

2ae

^{1}H NMR, 400 MHz, $CDCl_3$

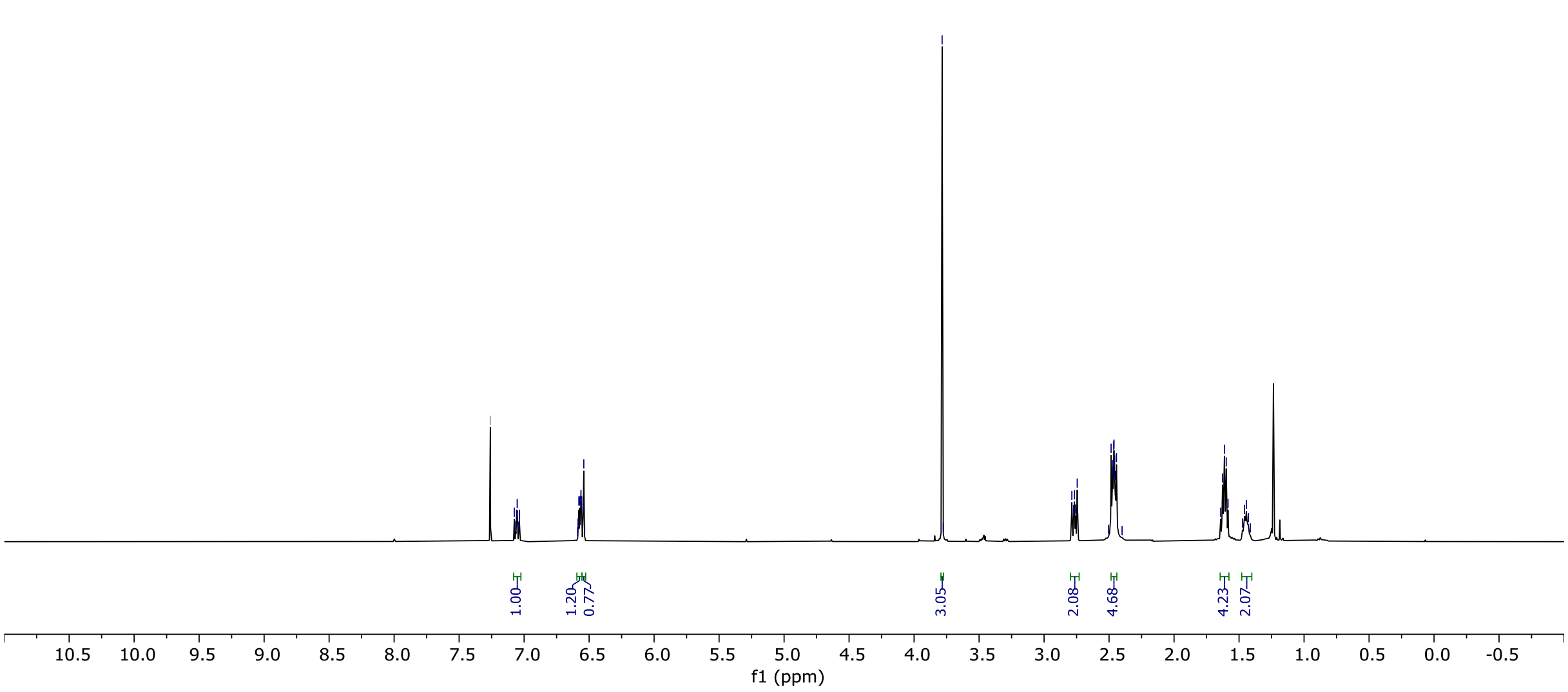

2ae

^{13}C NMR, 126 MHz, $CDCl_3$

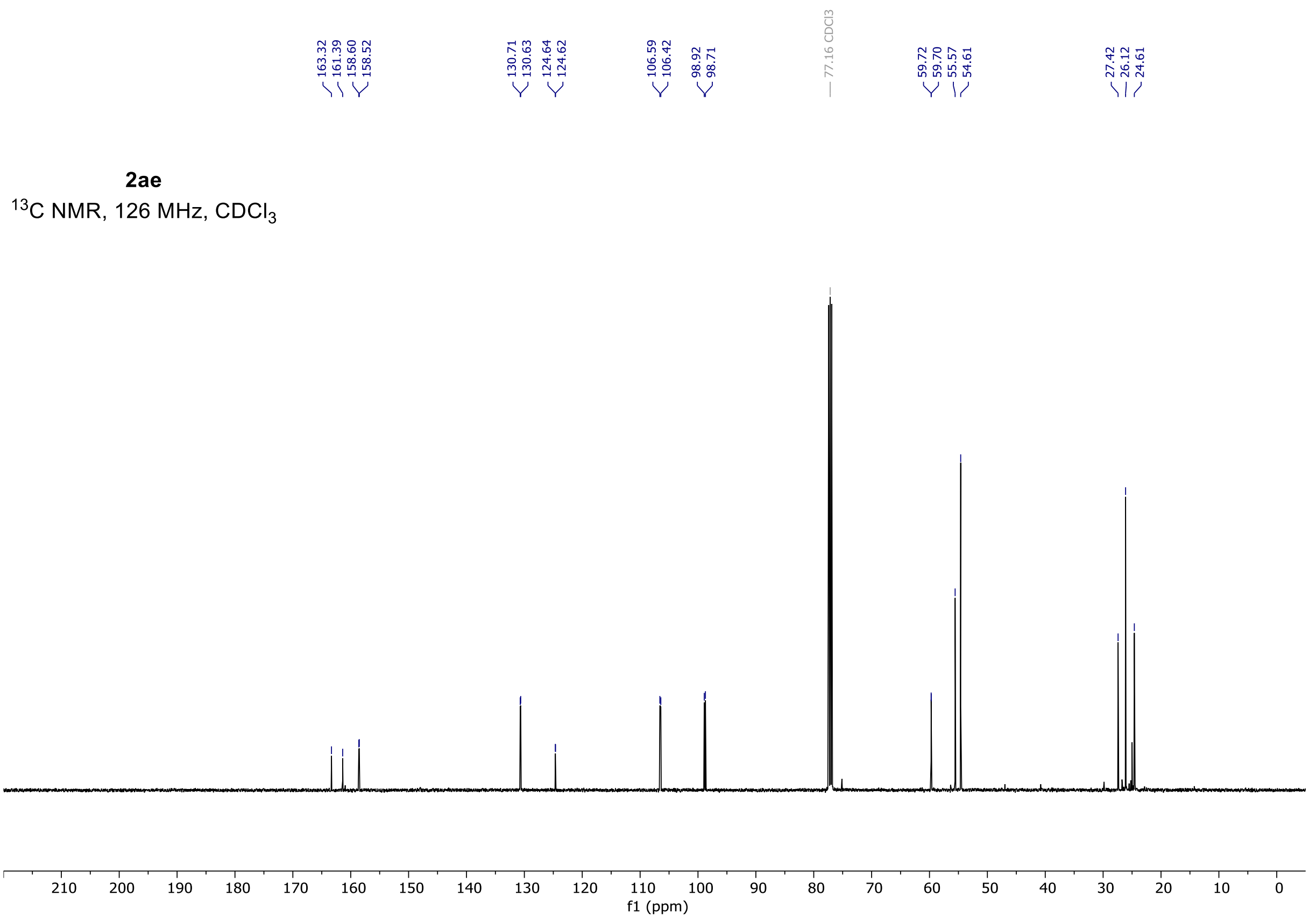

2ae

^{19}F NMR, 376 MHz, $CDCl_3$

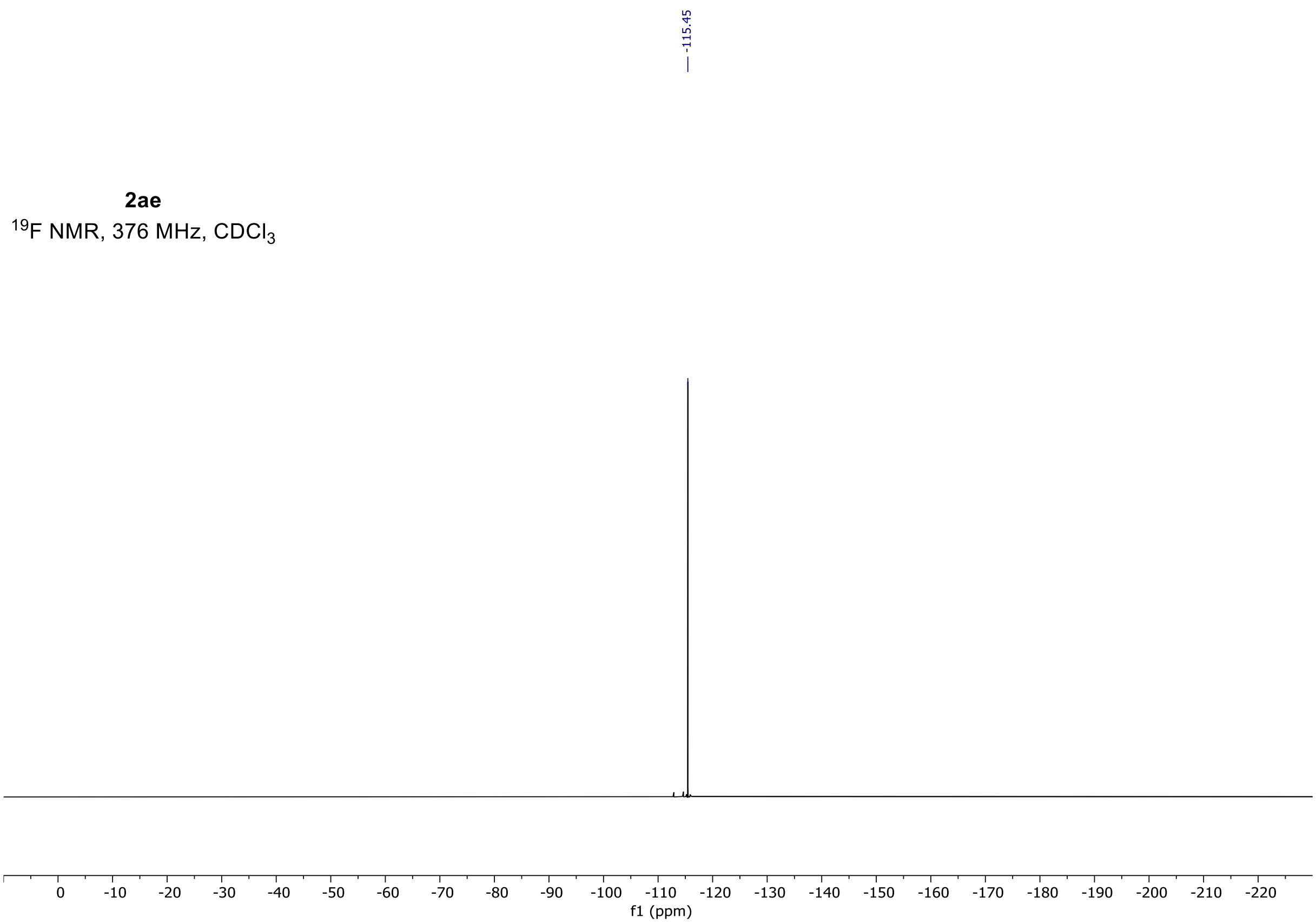

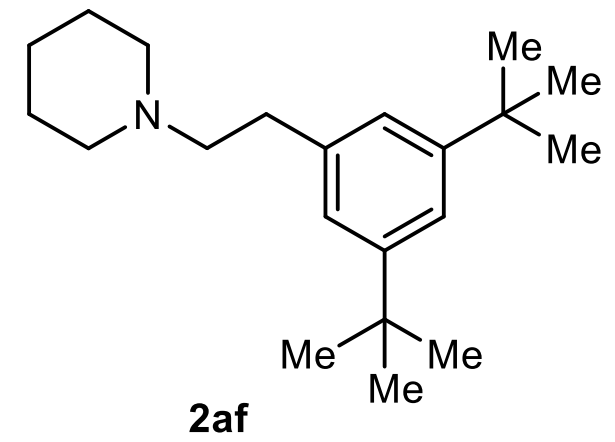

2af

^{1}H NMR, 700 MHz, $CDCl_3$

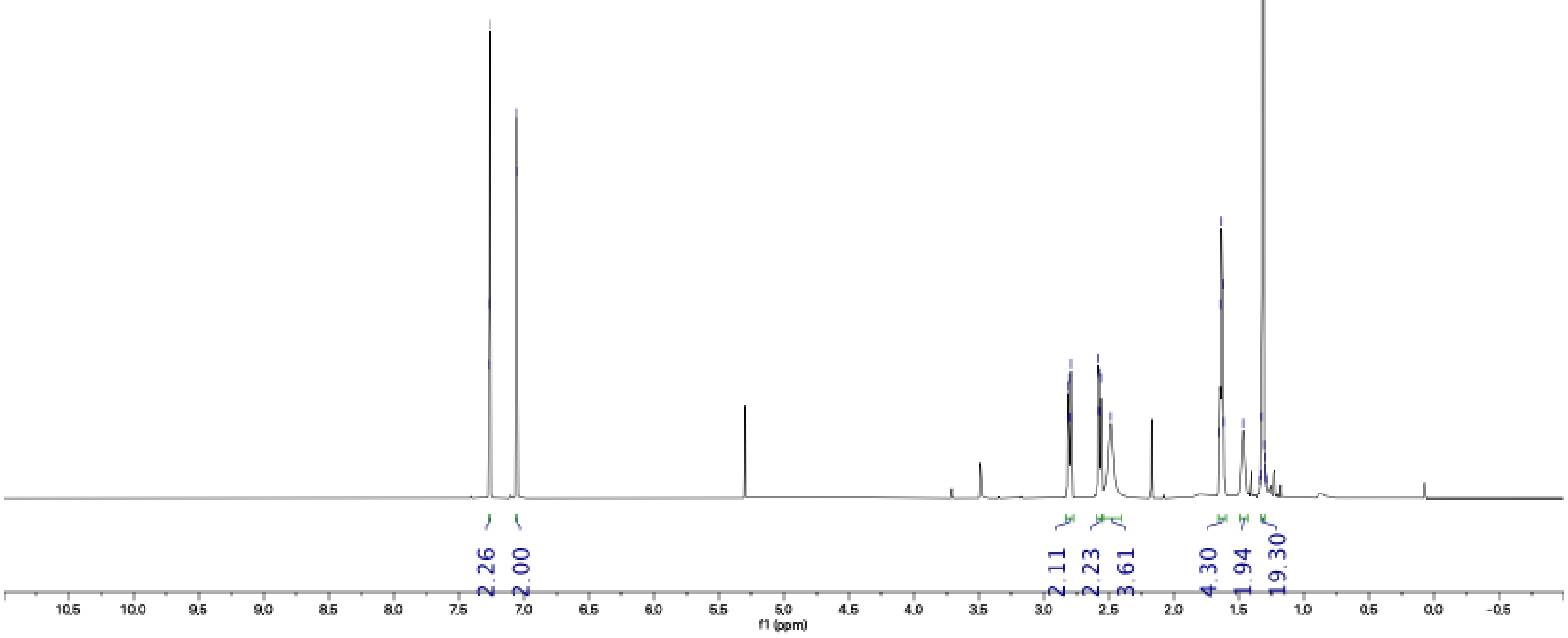

2af

^{13}C NMR, 176 MHz, $CDCl_3$

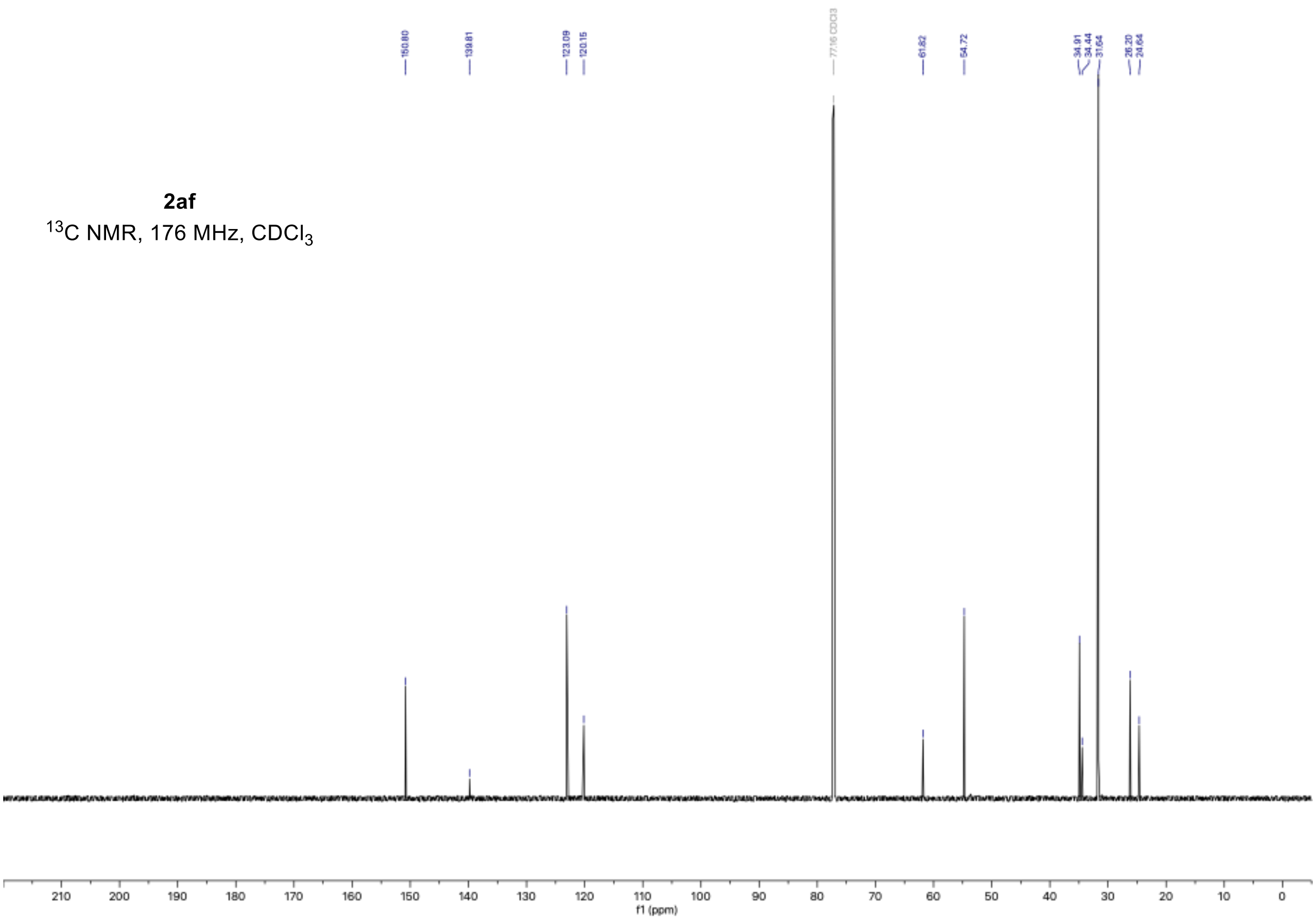

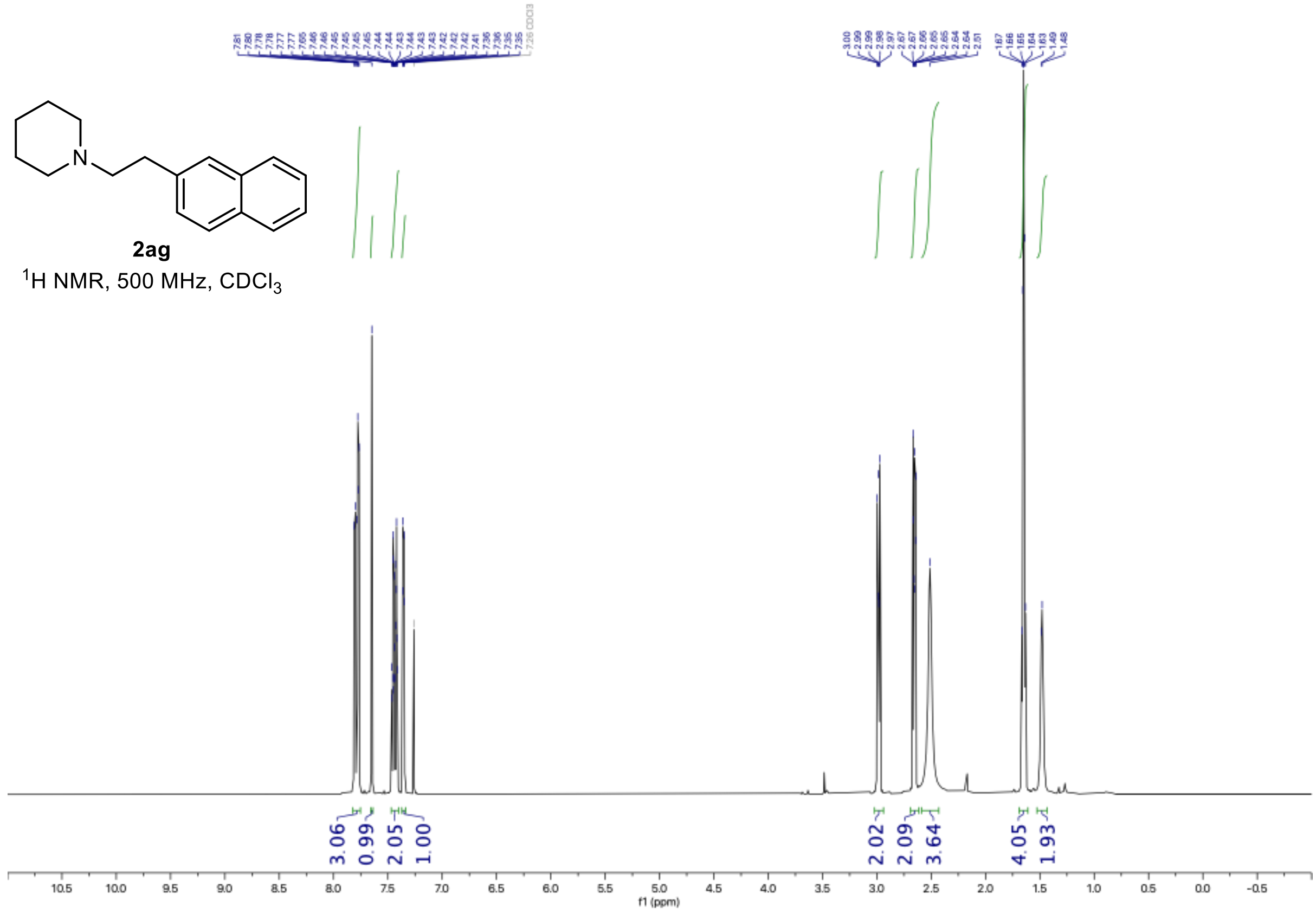
2ag
^{1}H NMR, 500 MHz, $CDCl_3$

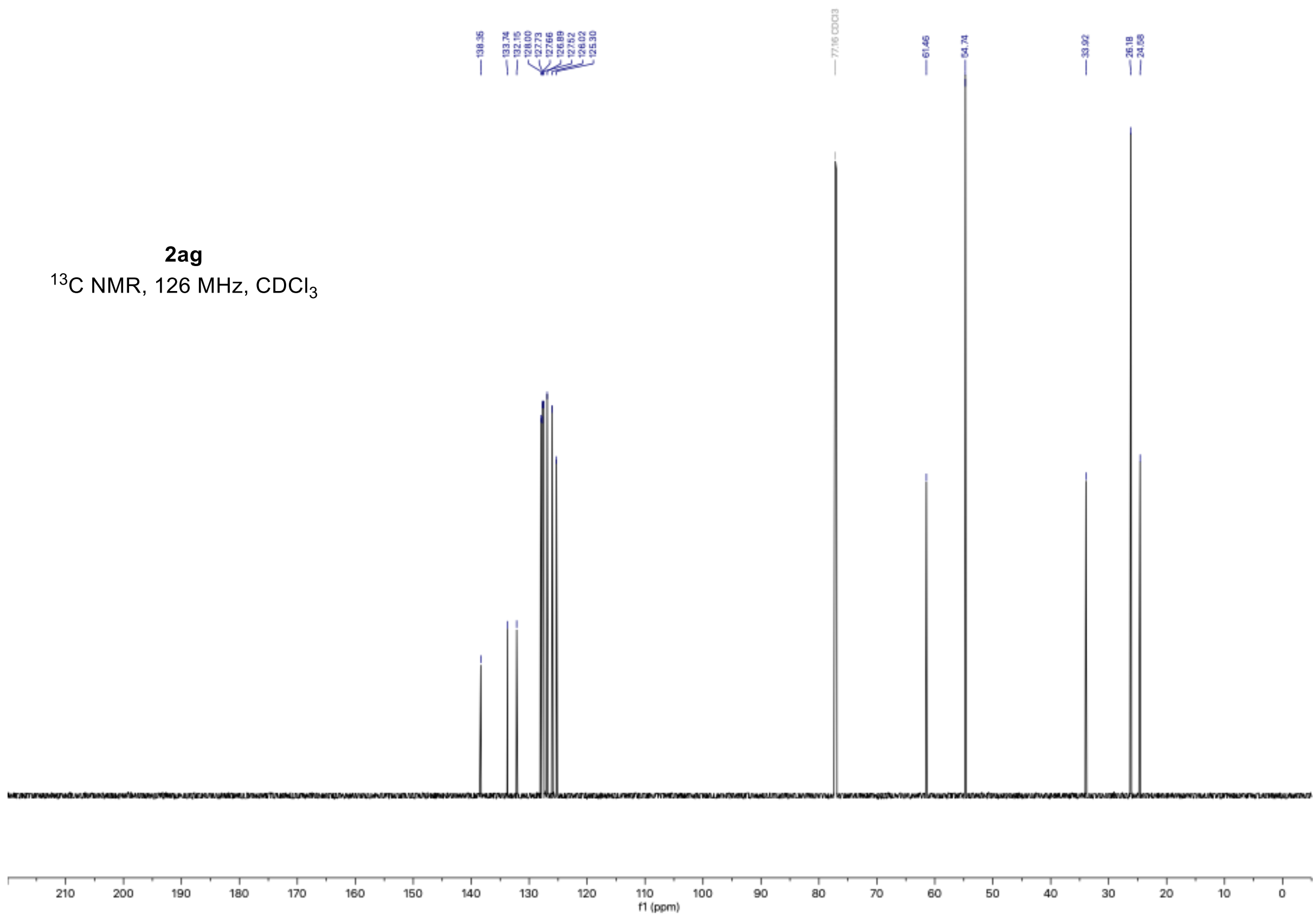
2ag
^{13}C NMR, 126 MHz, $CDCl_3$

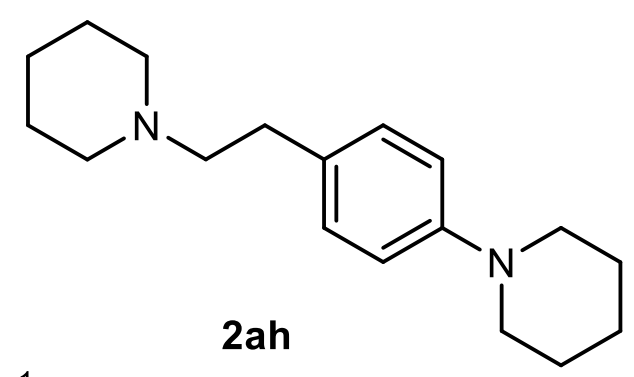

2ah

^{1}H NMR, 500 MHz, $CDCl_3$

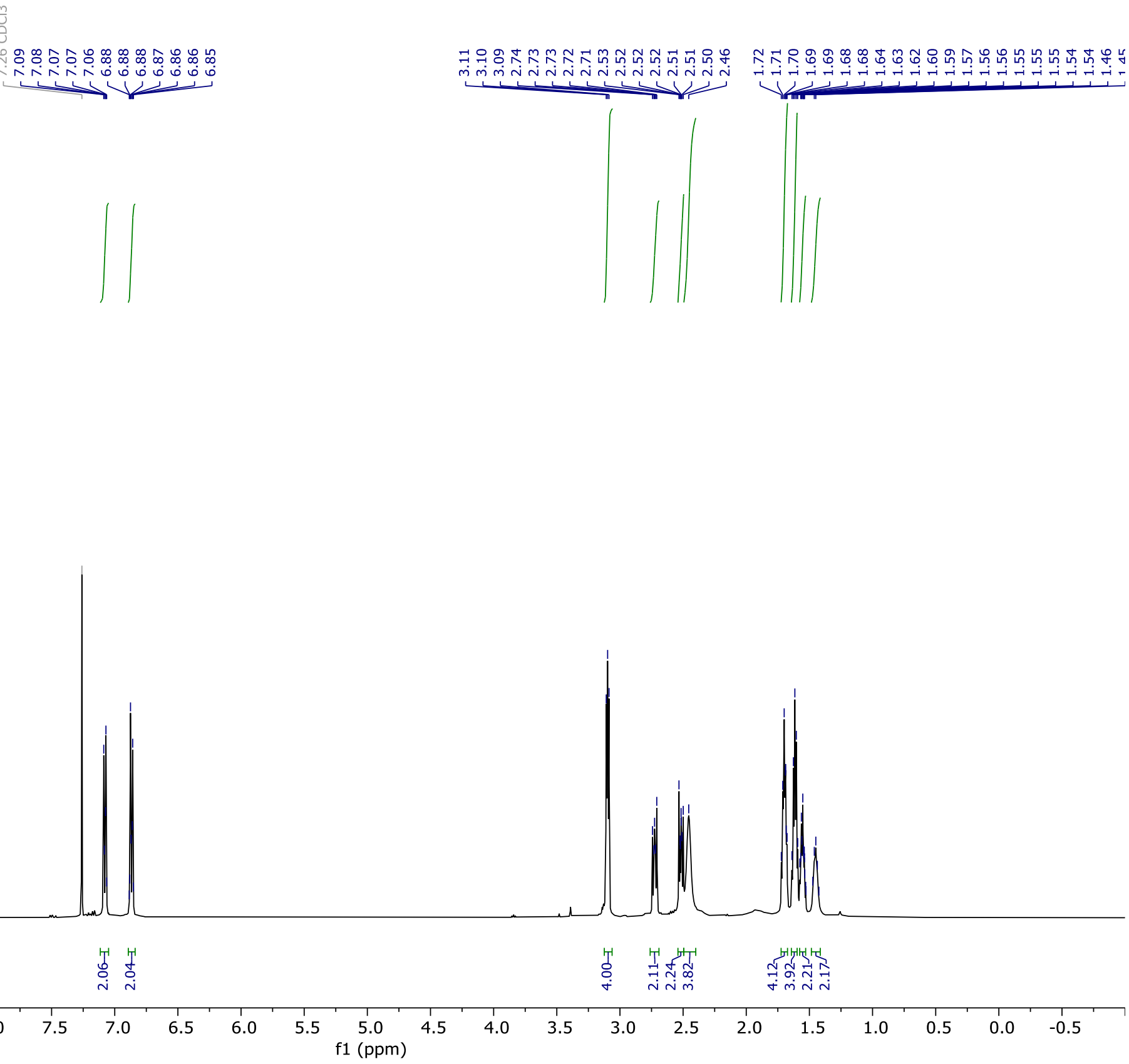

2ah

^{13}C NMR, 126 MHz, $CDCl_3$

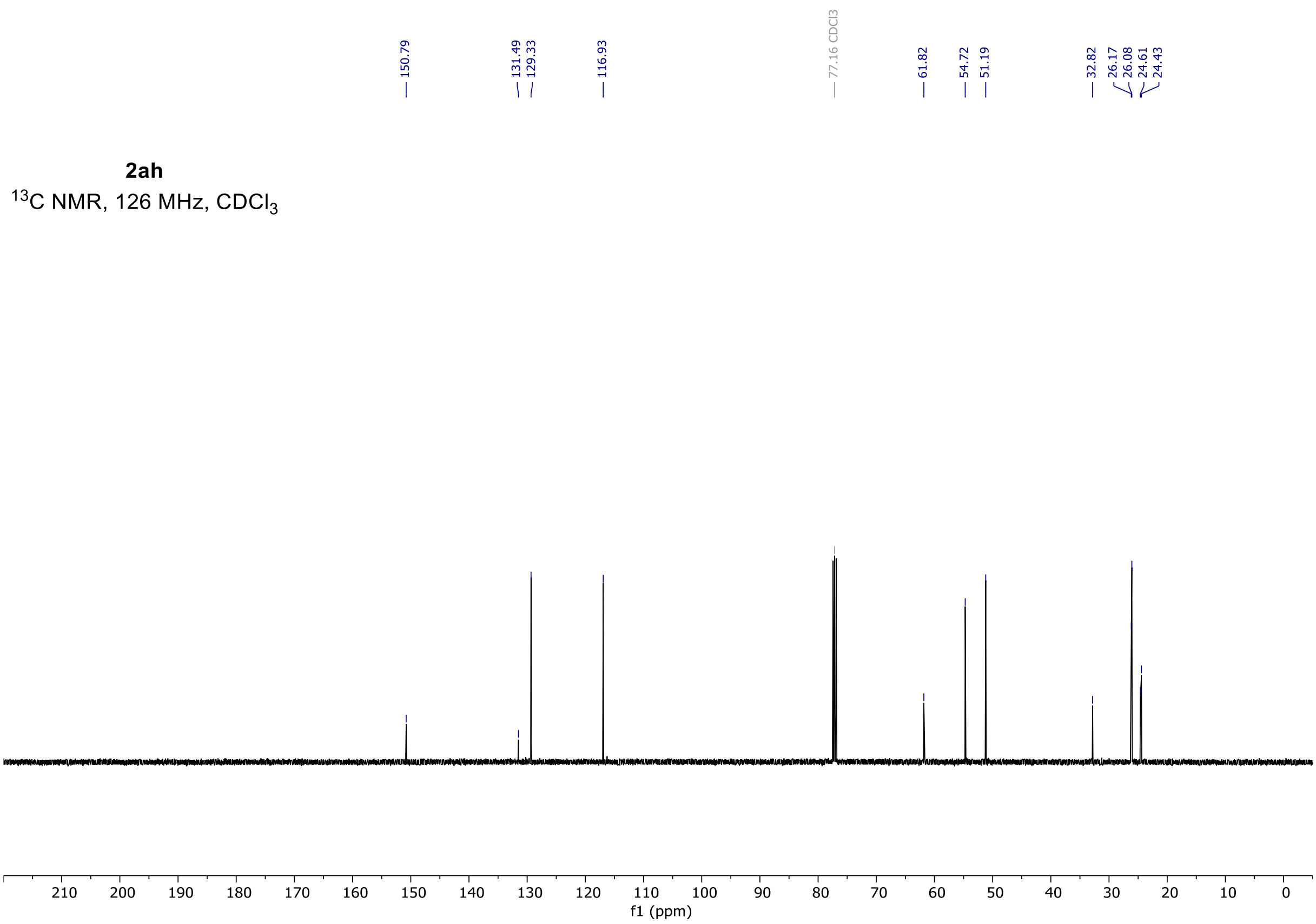

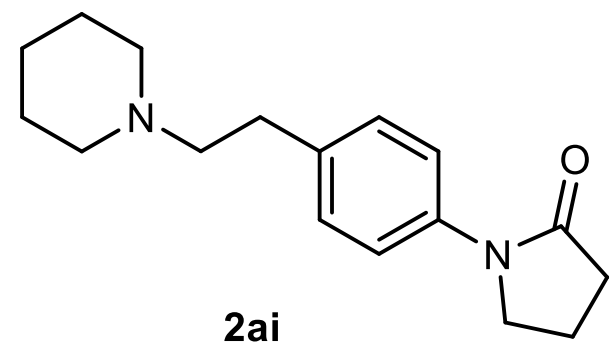

2ai

^{1}H NMR, 500 MHz, $CDCl_3$

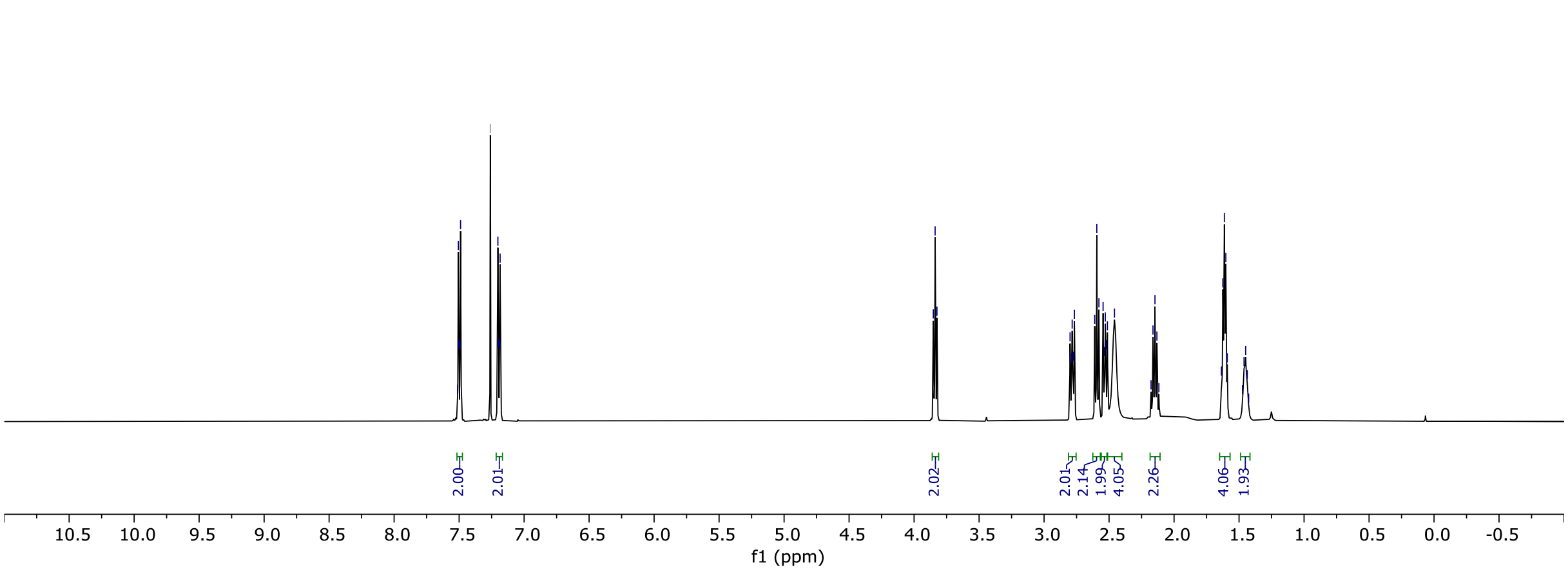

2ai

^{13}C NMR, 126 MHz, $CDCl_3$

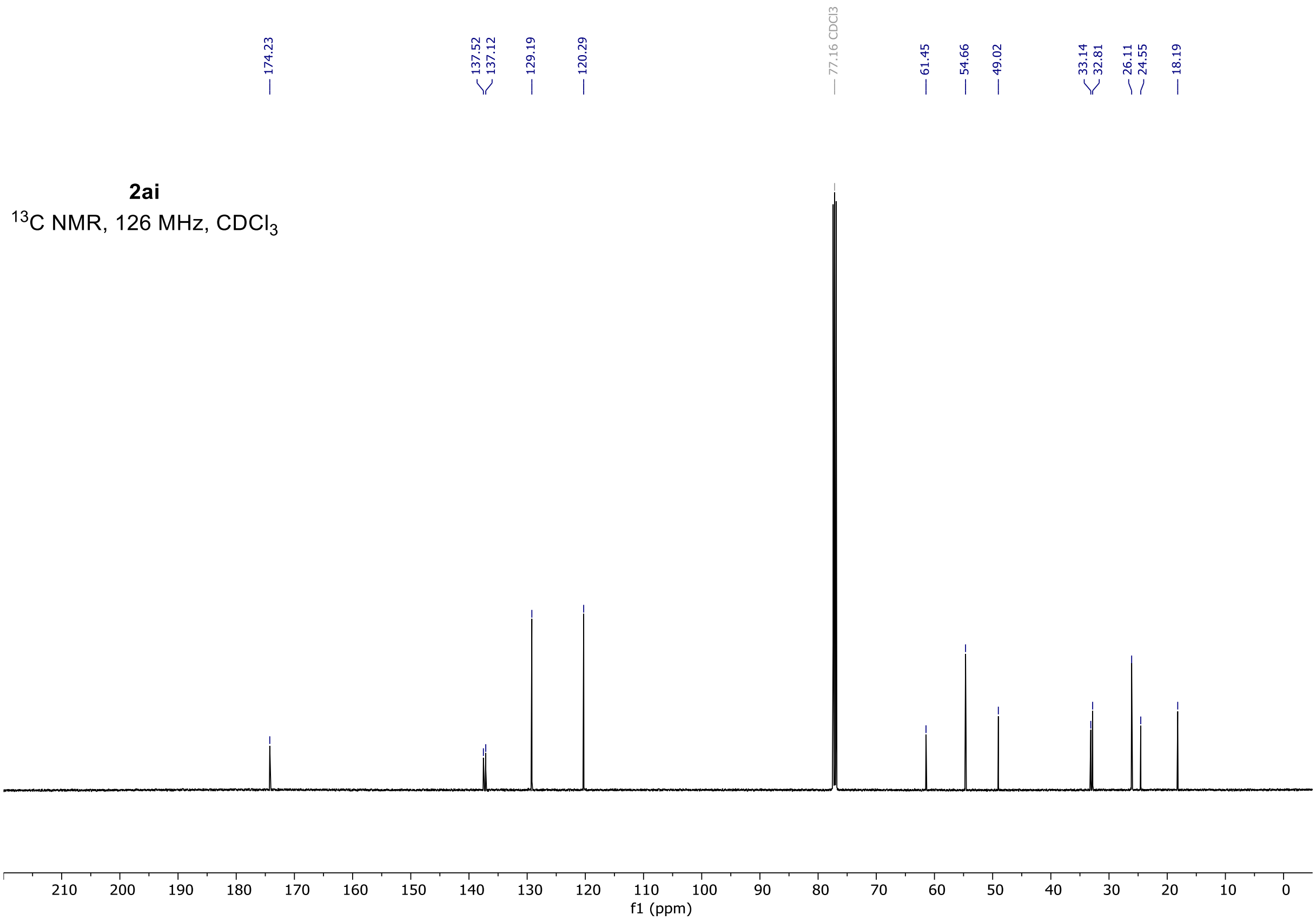

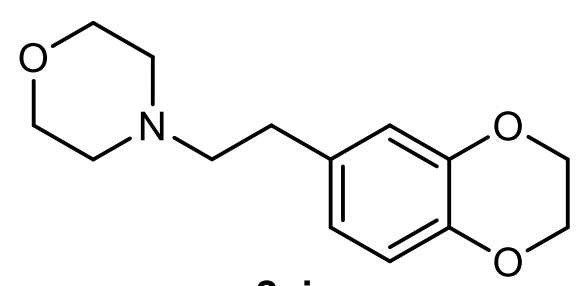

2aj

^{1}H NMR, 700 MHz, $CDCl_3$

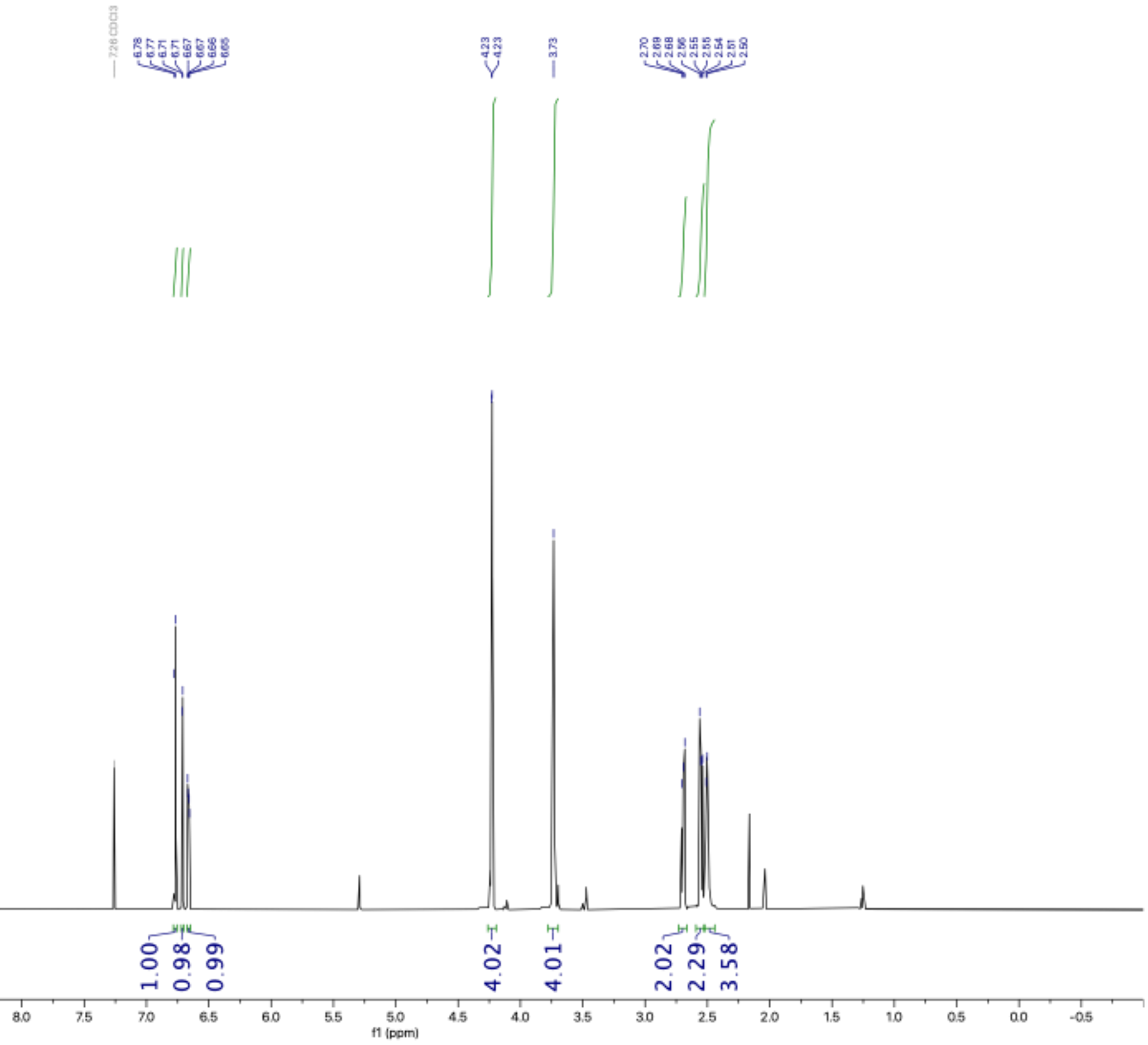

2aj

^{13}C NMR, 176 MHz, $CDCl_3$

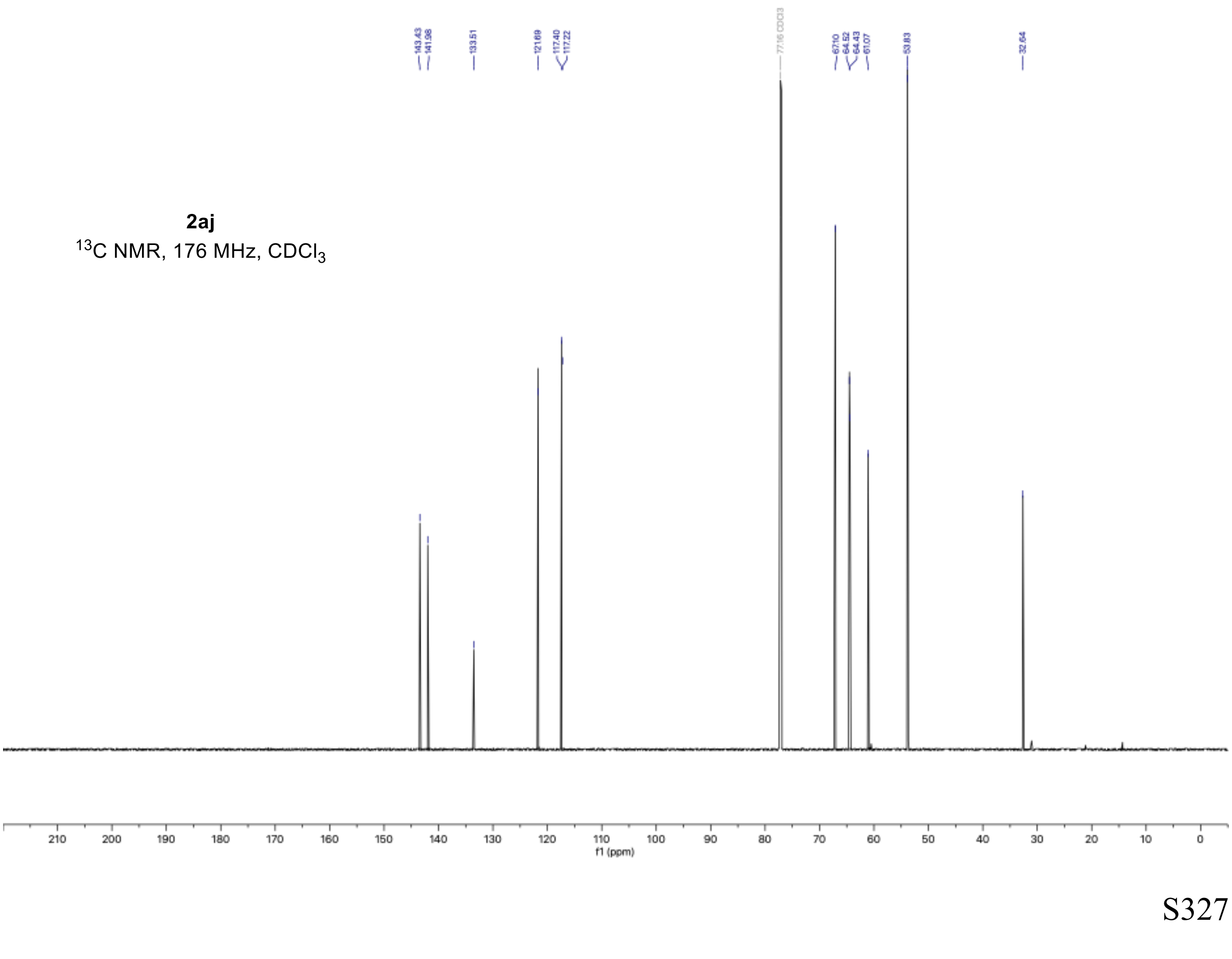

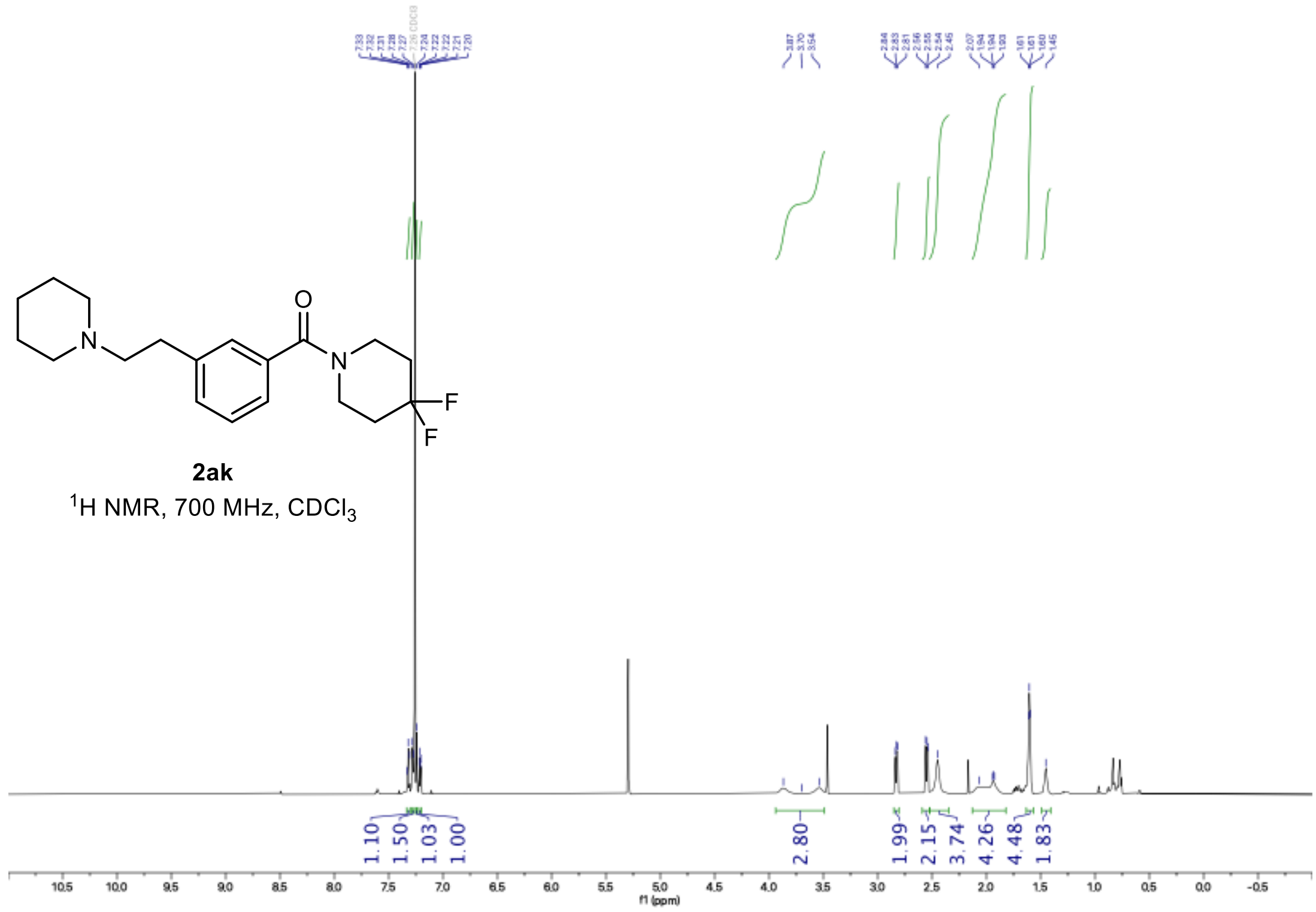

2ak
^{1}H NMR, 700 MHz, $CDCl_3$

2ak
^{13}C NMR, 176 MHz, $CDCl_3$

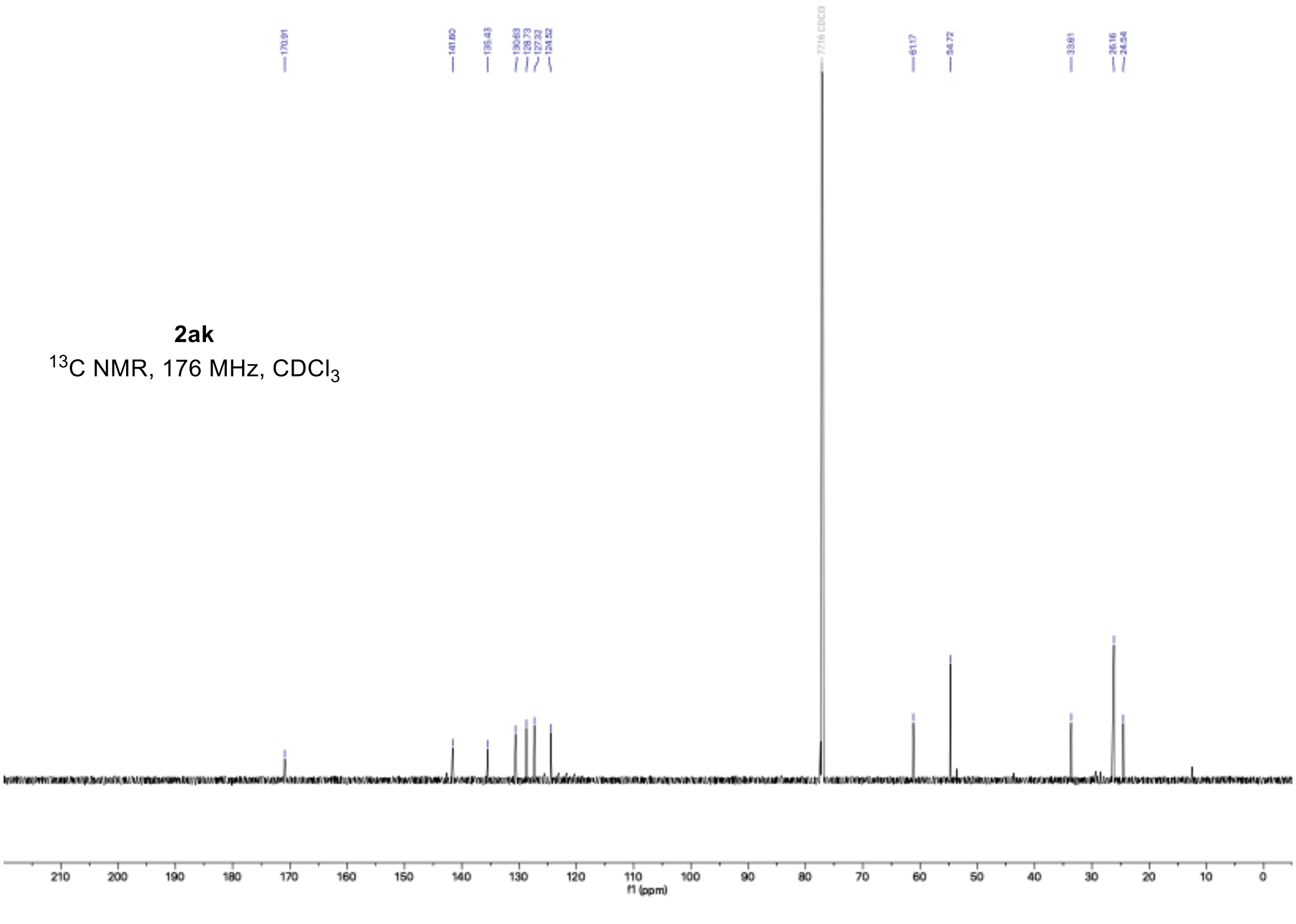

2ak
^{19}F NMR, 376 MHz, $CDCl_3$

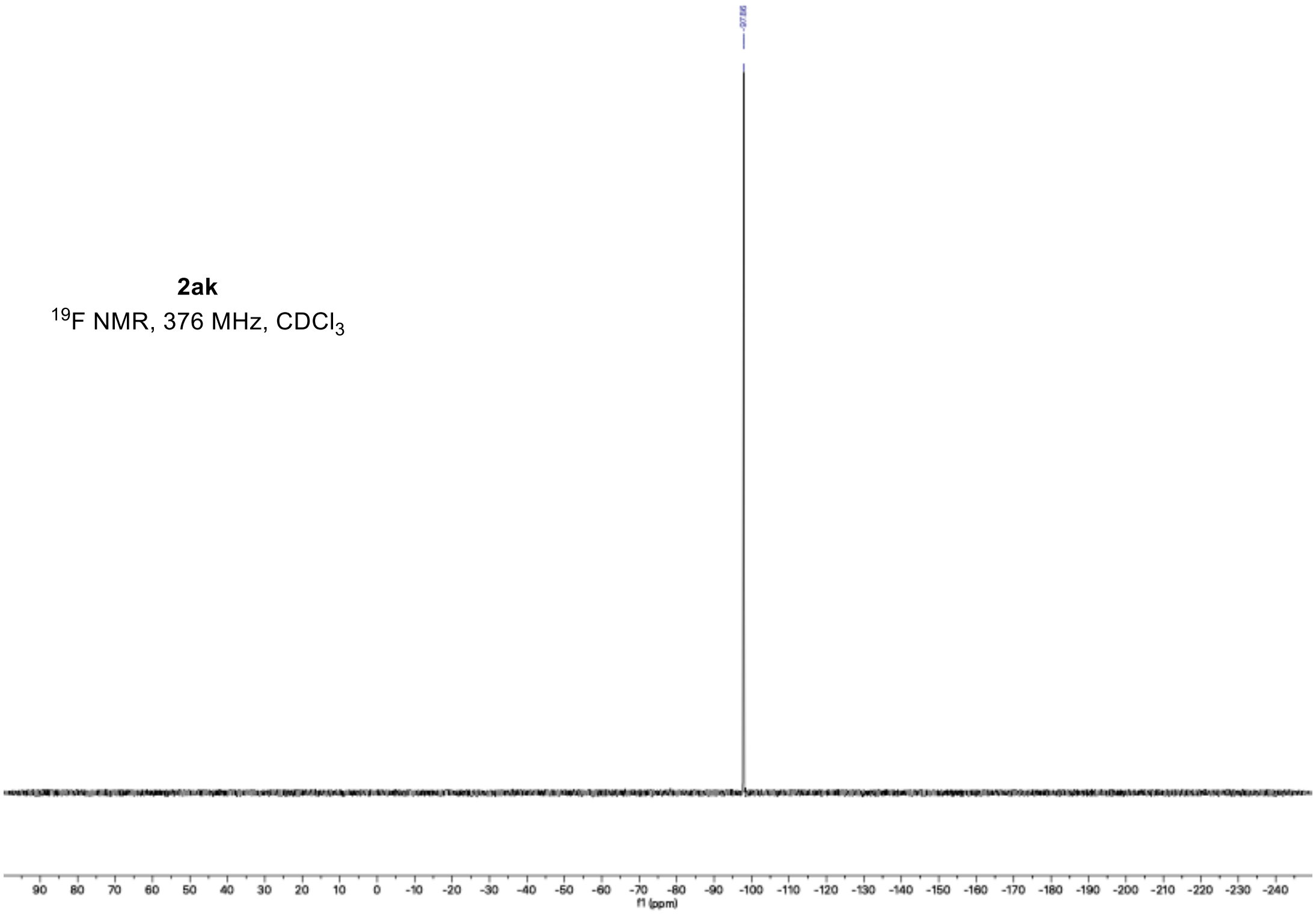

2al

^{1}H NMR, 700 MHz, CDCl$_3$

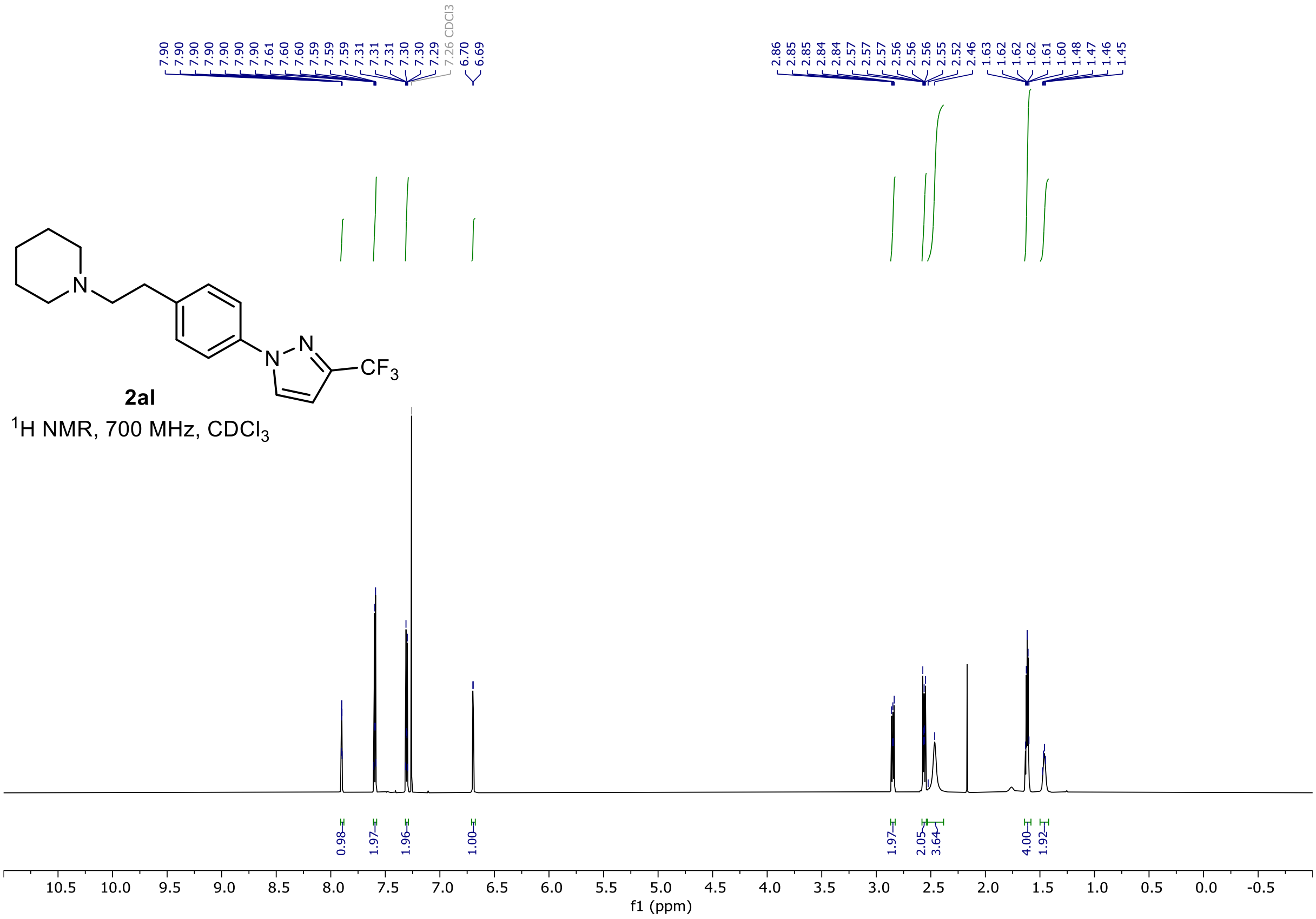

2al

^{13}C NMR, 176 MHz, CDCl$_3$

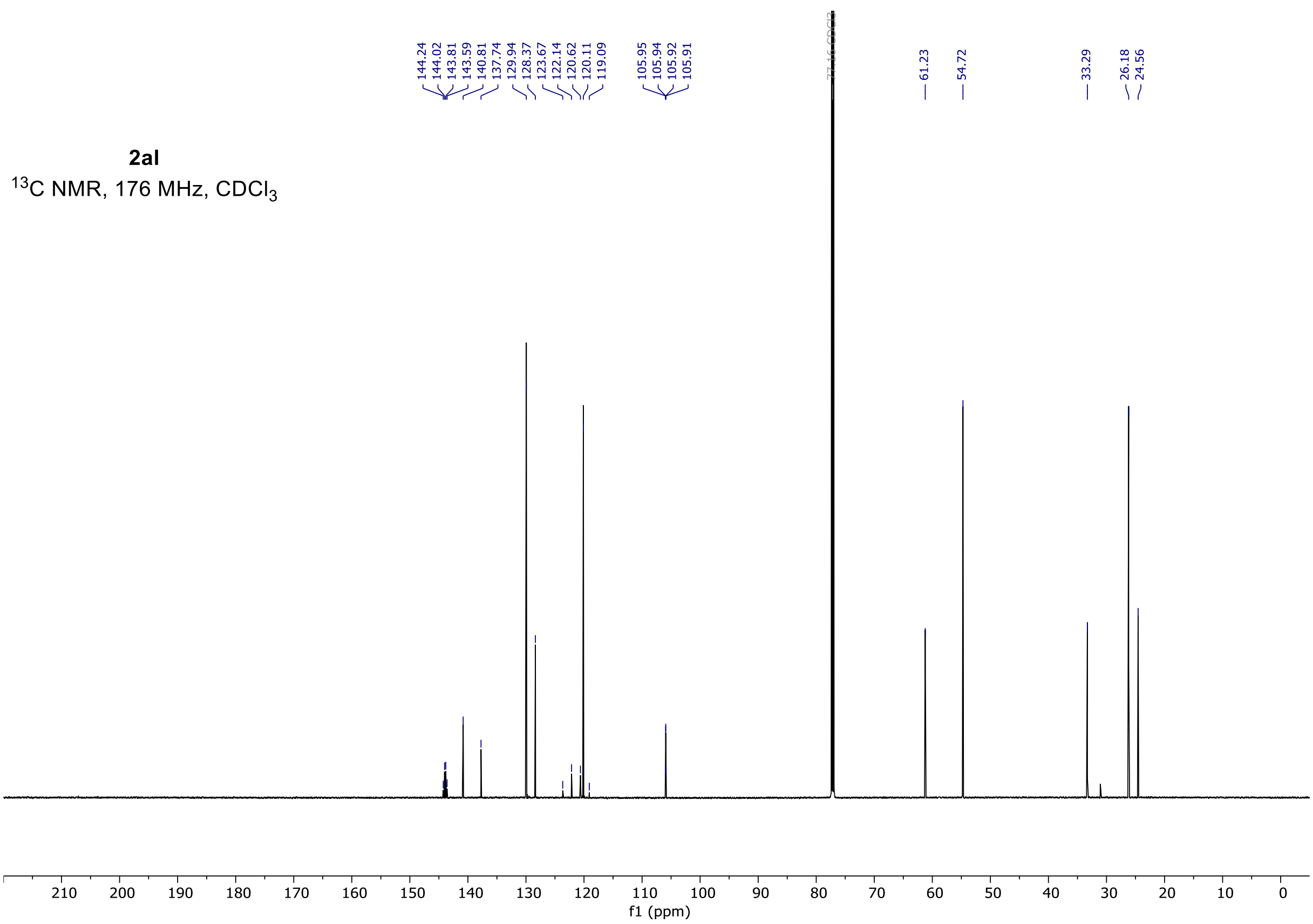

2al

^{19}F NMR, 376 MHz, $CDCl_3$

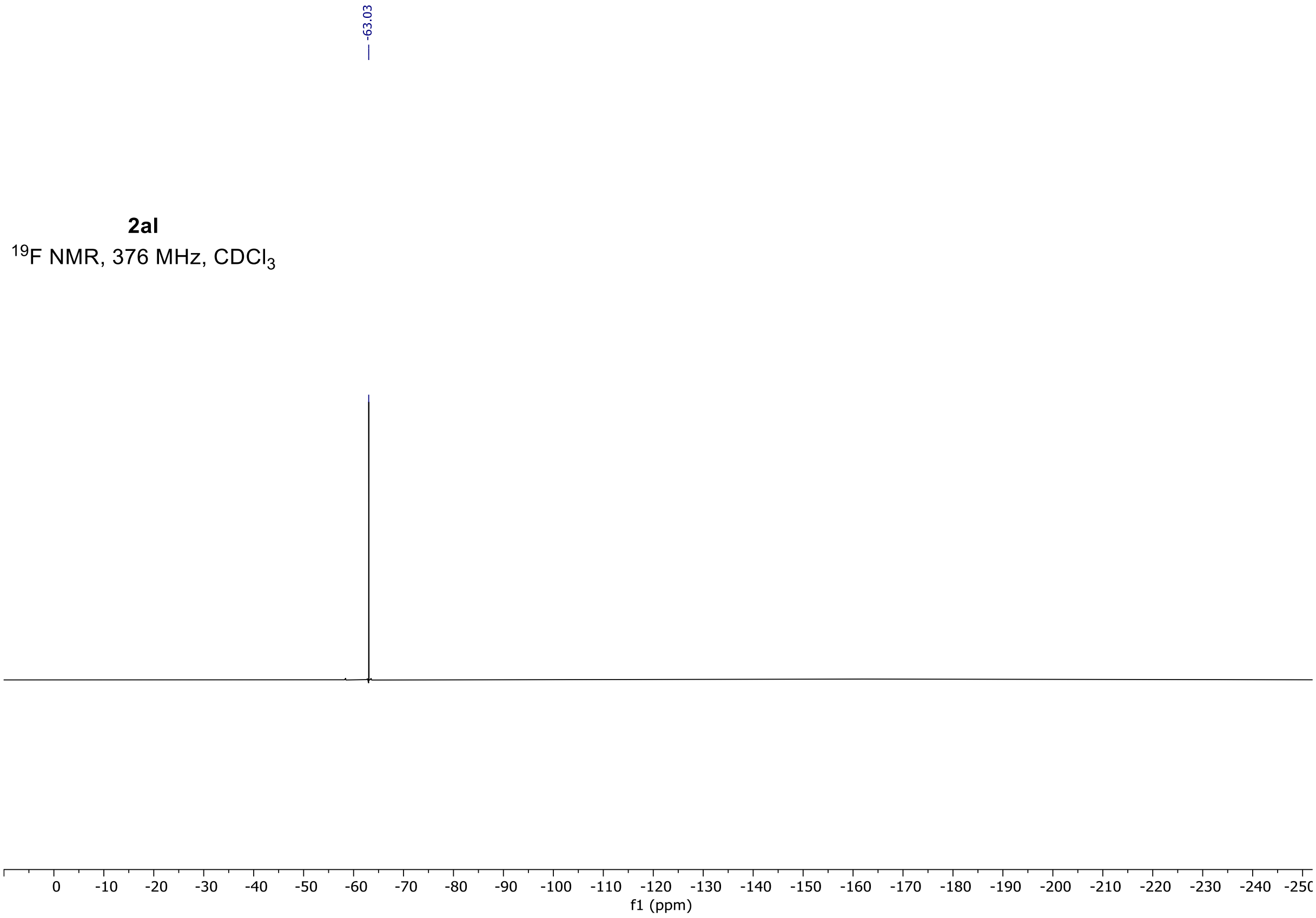

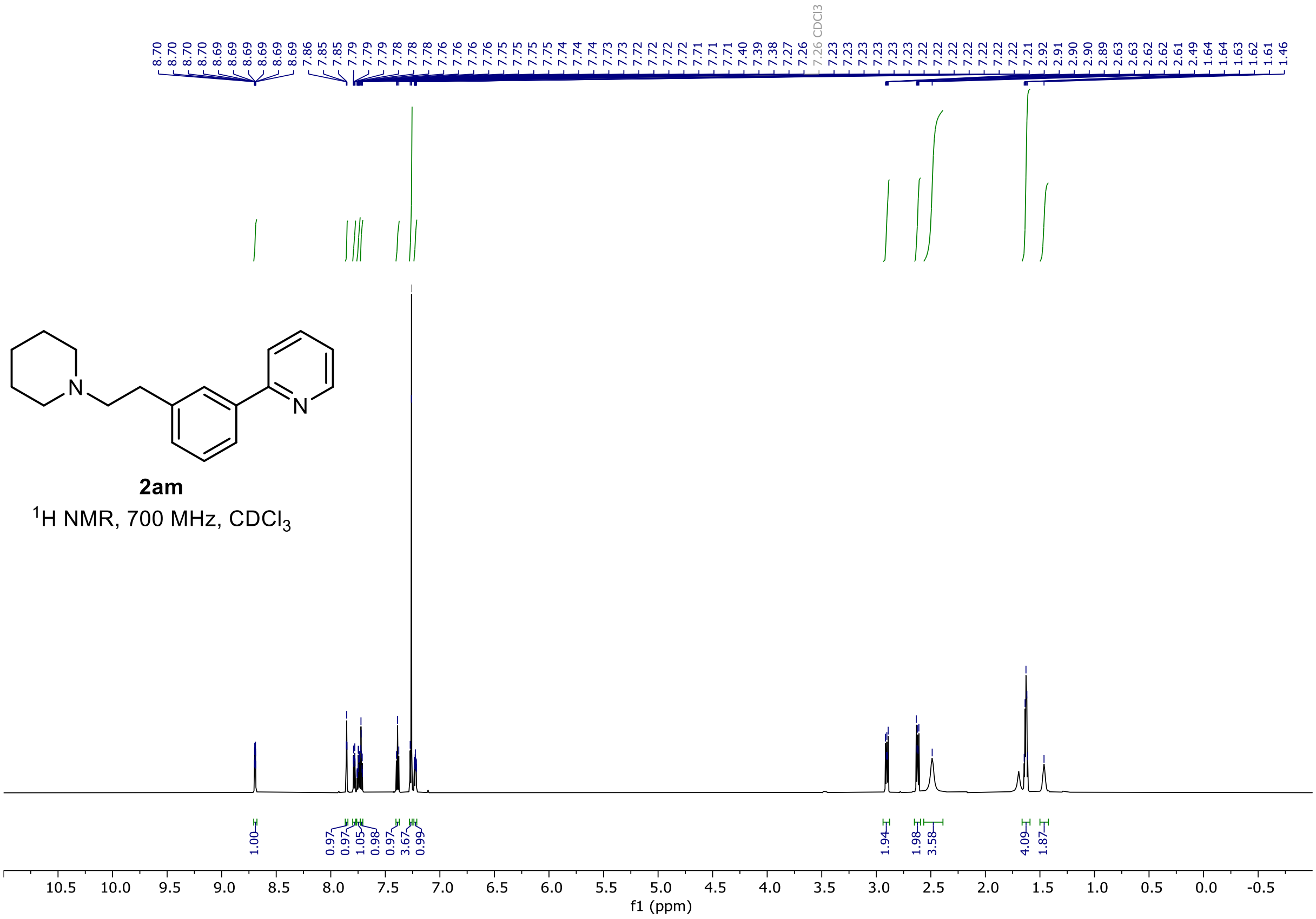

2am

^{1}H NMR, 700 MHz, $CDCl_3$

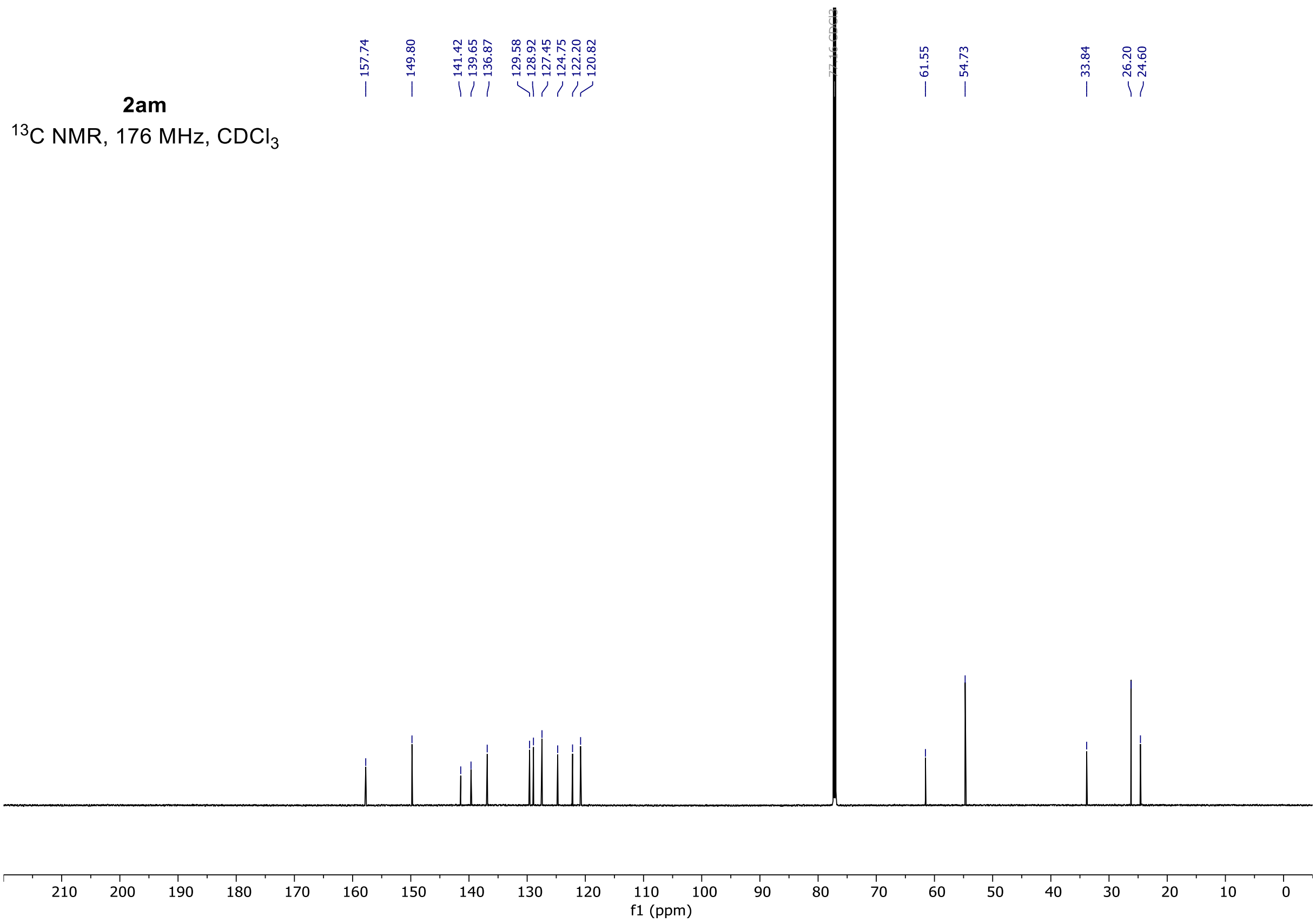

2am

^{13}C NMR, 176 MHz, $CDCl_3$

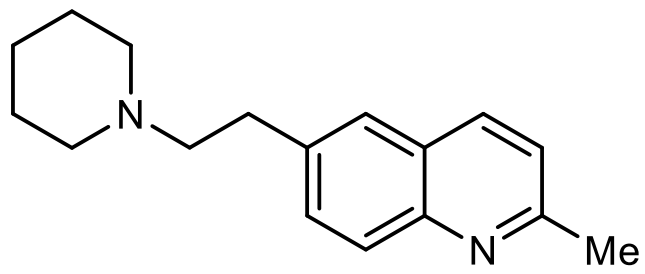

2an

^{1}H NMR, 700 MHz, $CDCl_3$

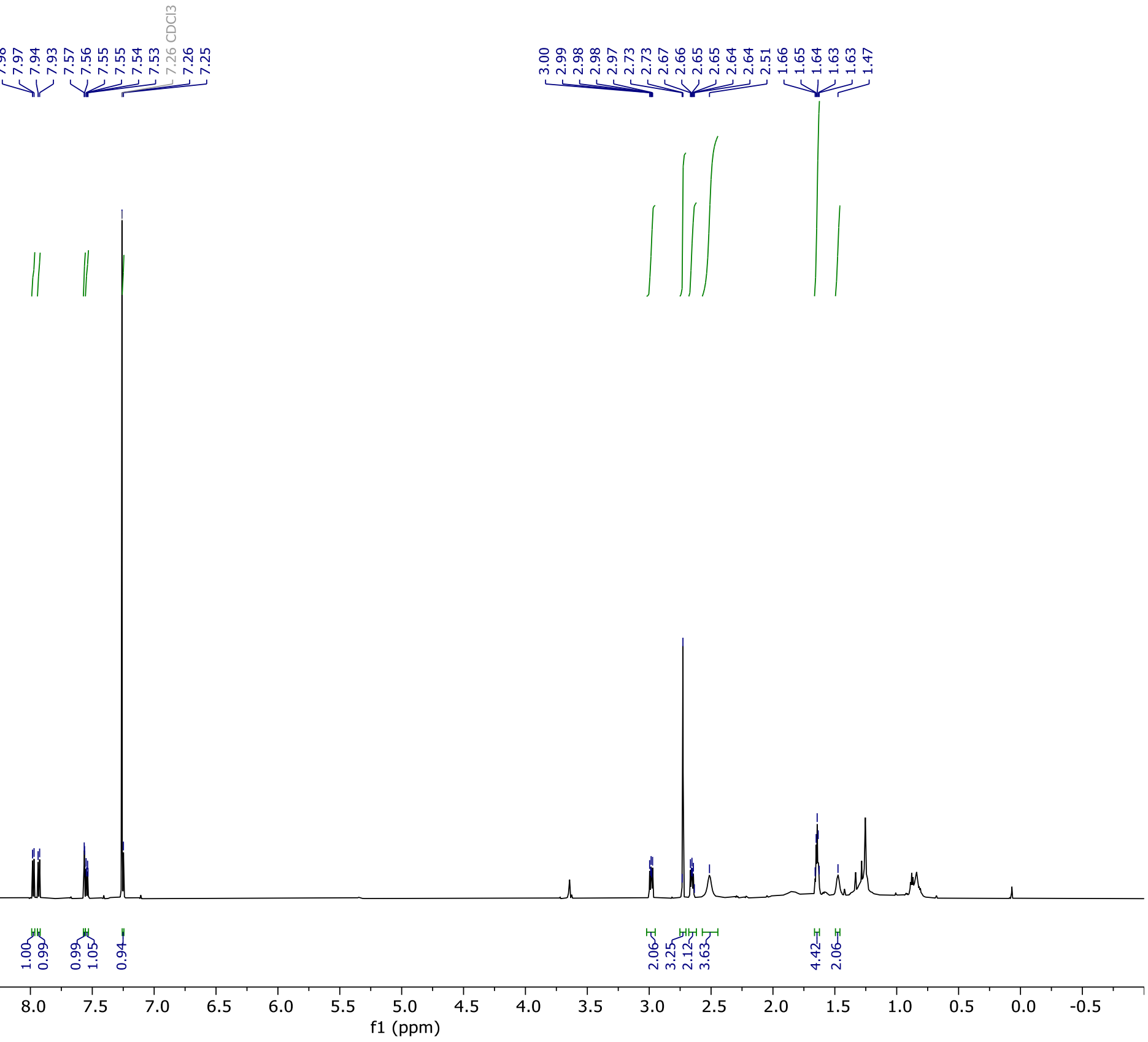

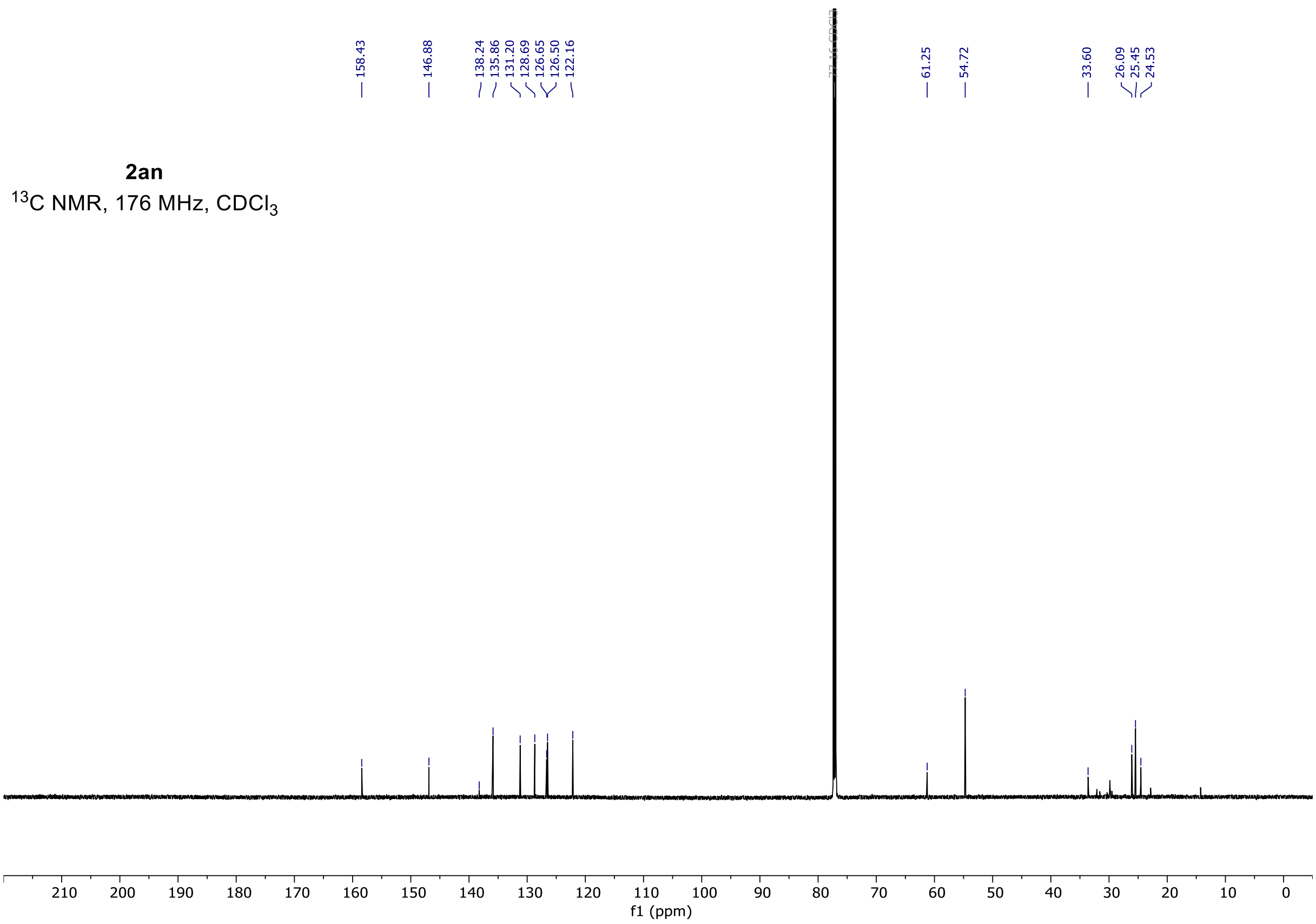

2an

^{13}C NMR, 176 MHz, $CDCl_3$

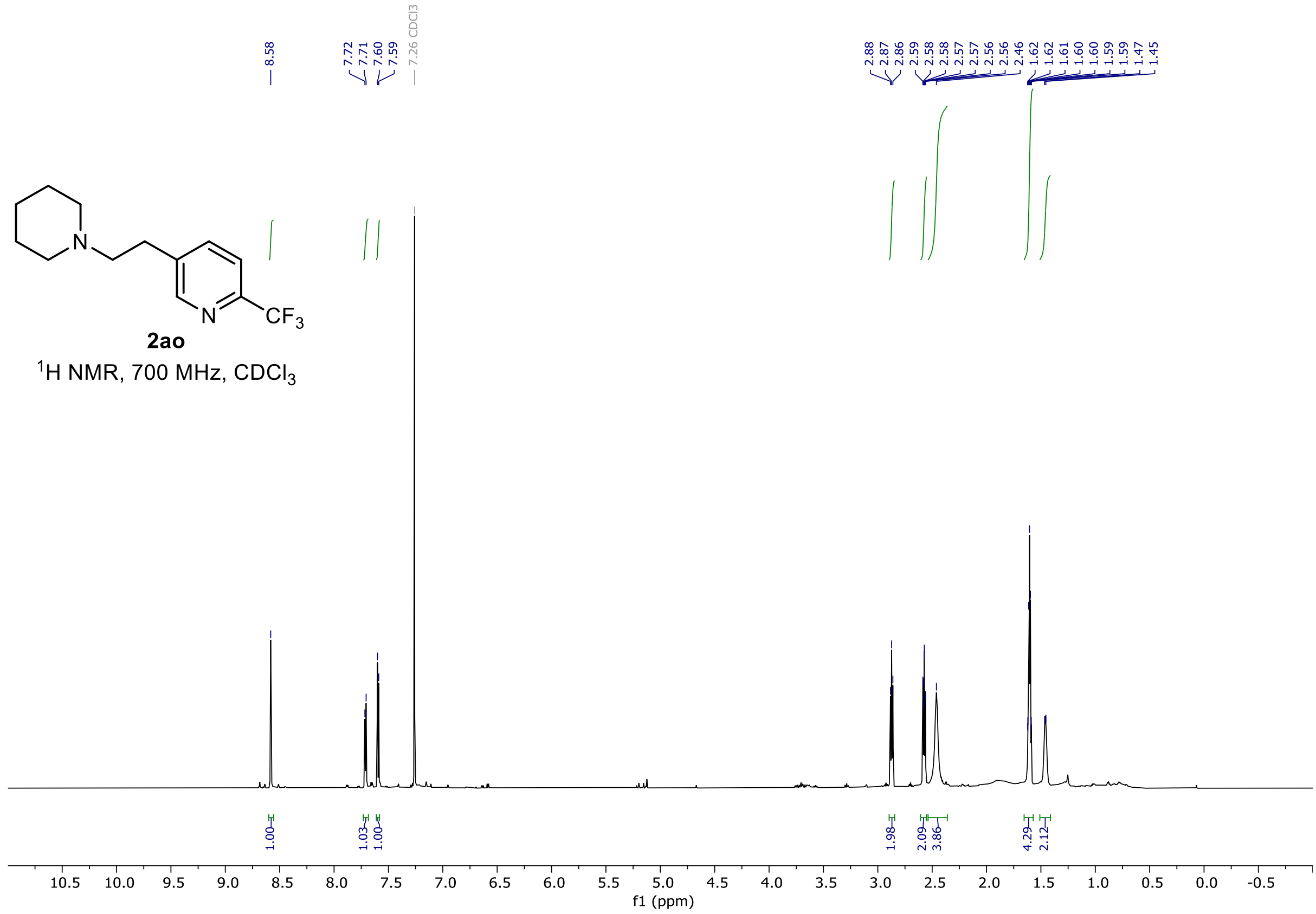

2ao
^{1}H NMR, 700 MHz, $CDCl_3$

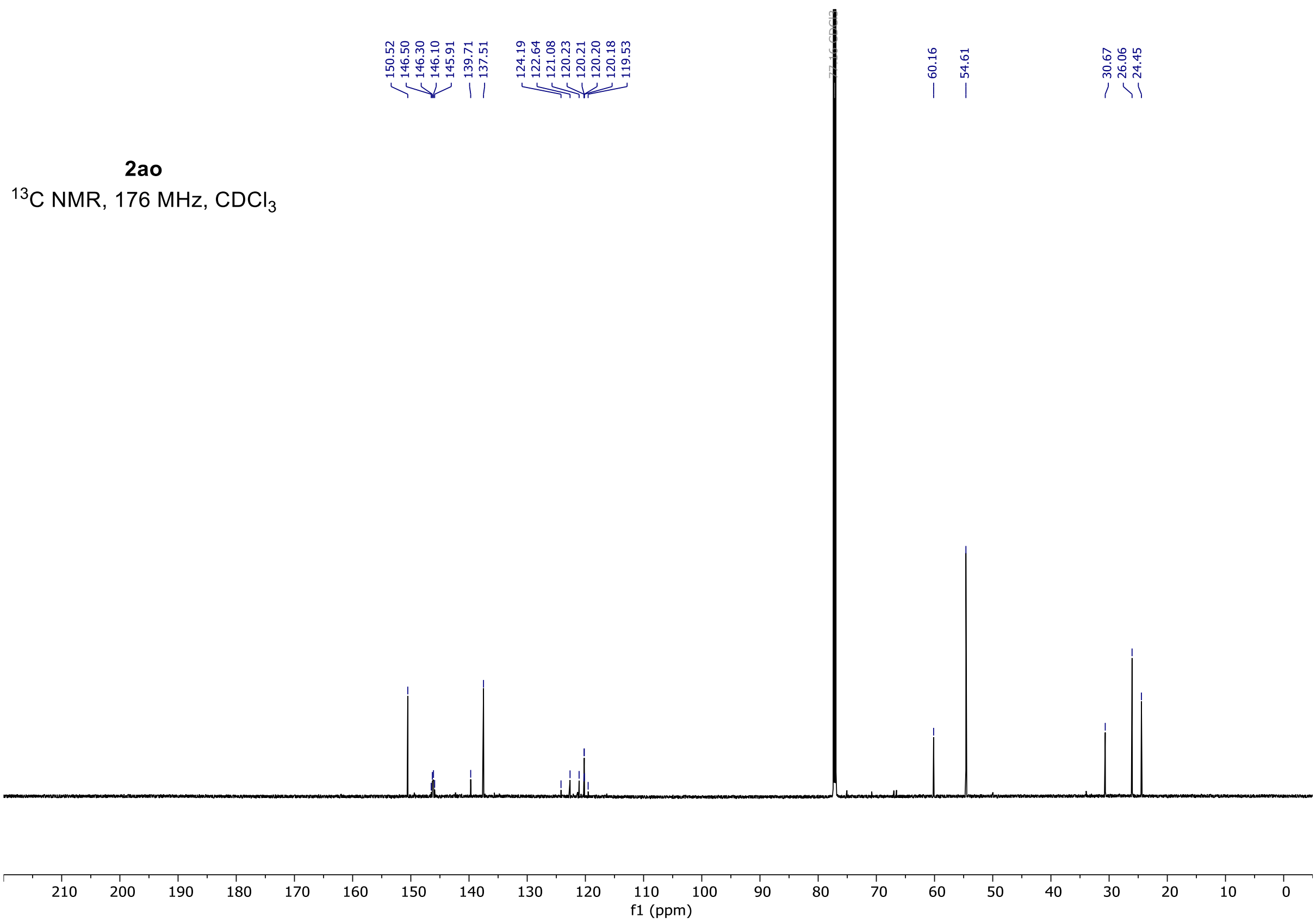

2ao
^{13}C NMR, 176 MHz, $CDCl_3$

2ao

^{19}F NMR, 471 MHz, $CDCl_3$

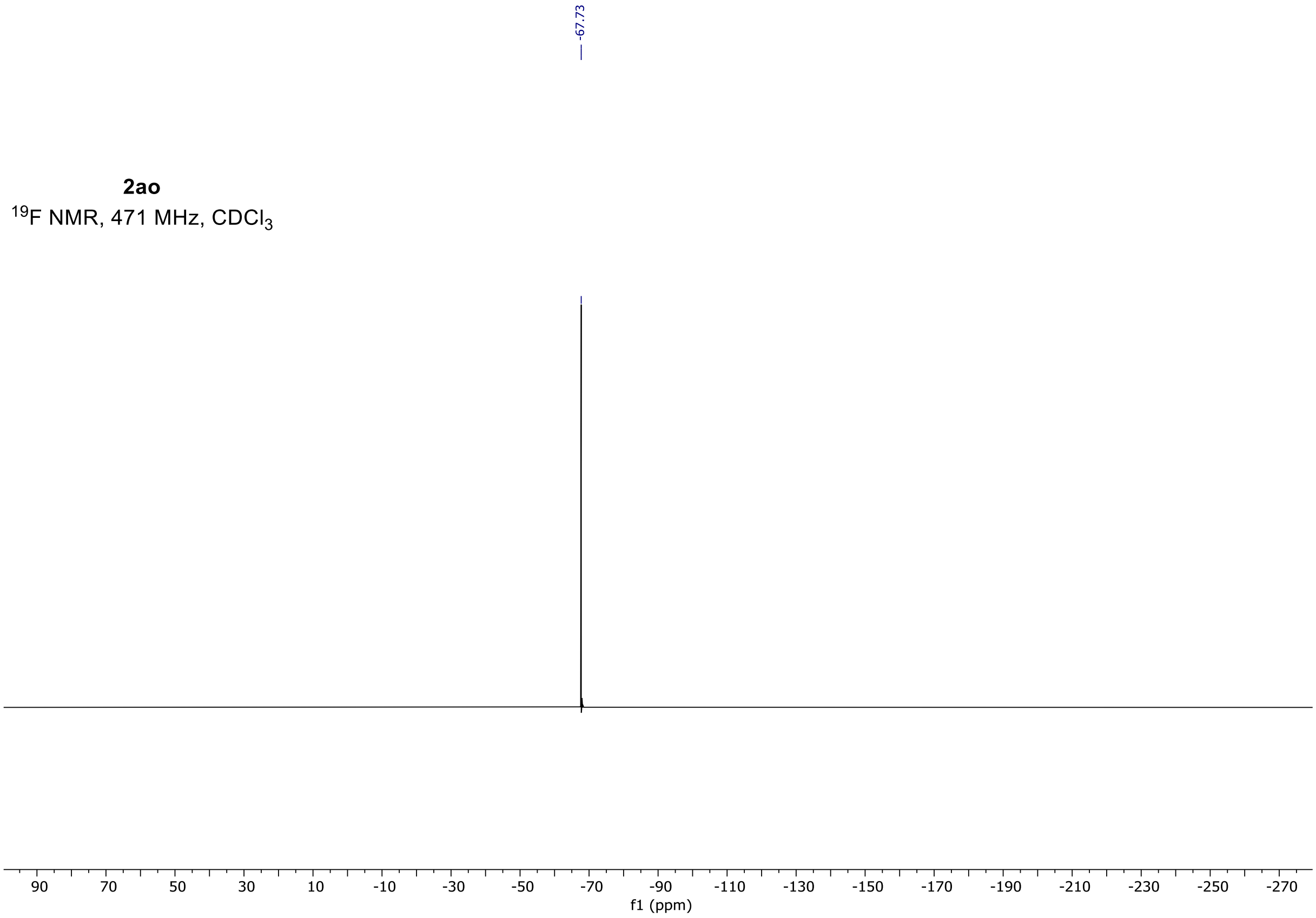

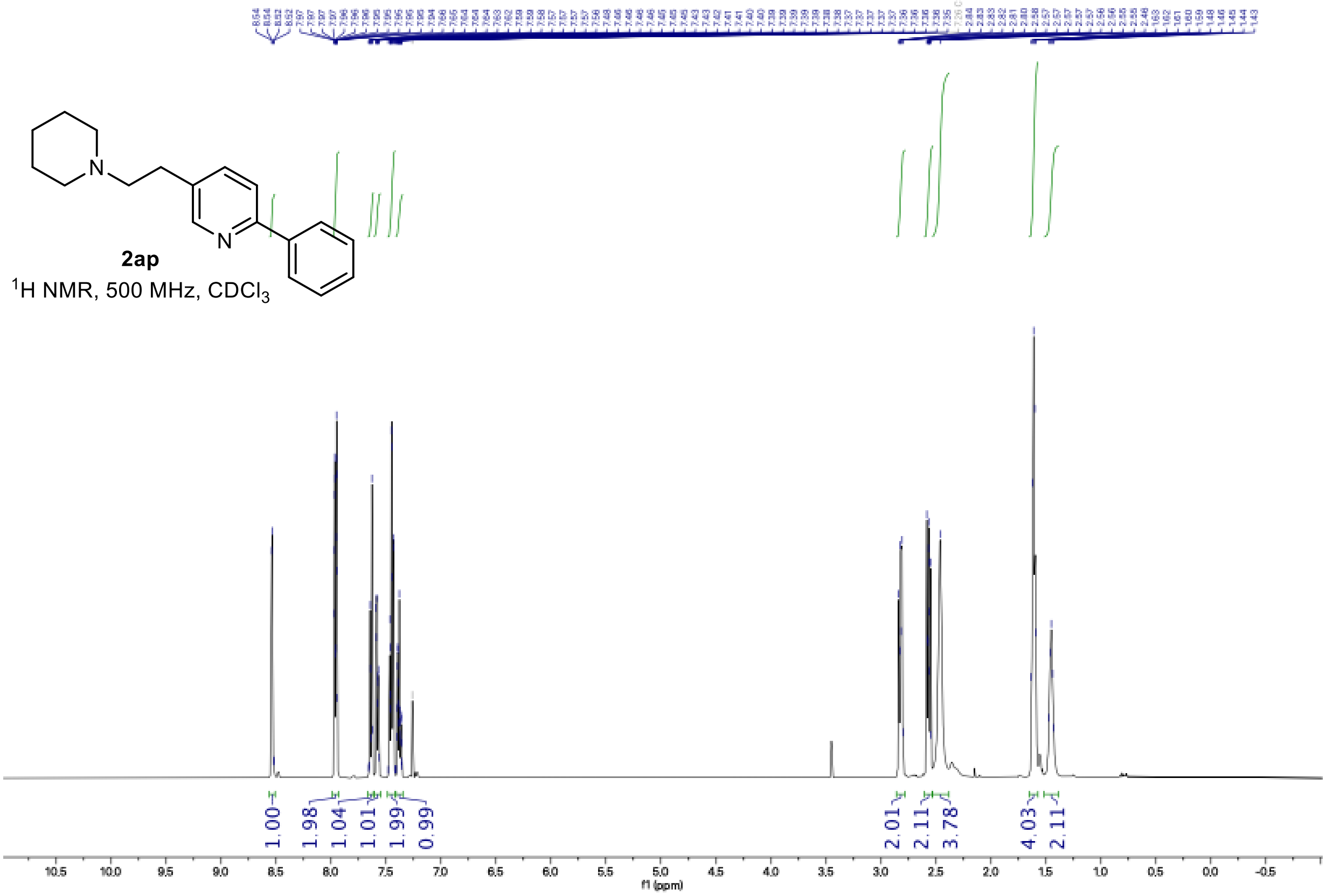
2ap
1H NMR, 500 MHz, CDCl3
1.00
1.98
1.04
1.01
1.99
0.99
2.01
2.11
3.78
4.03
2.11
f1 (ppm)

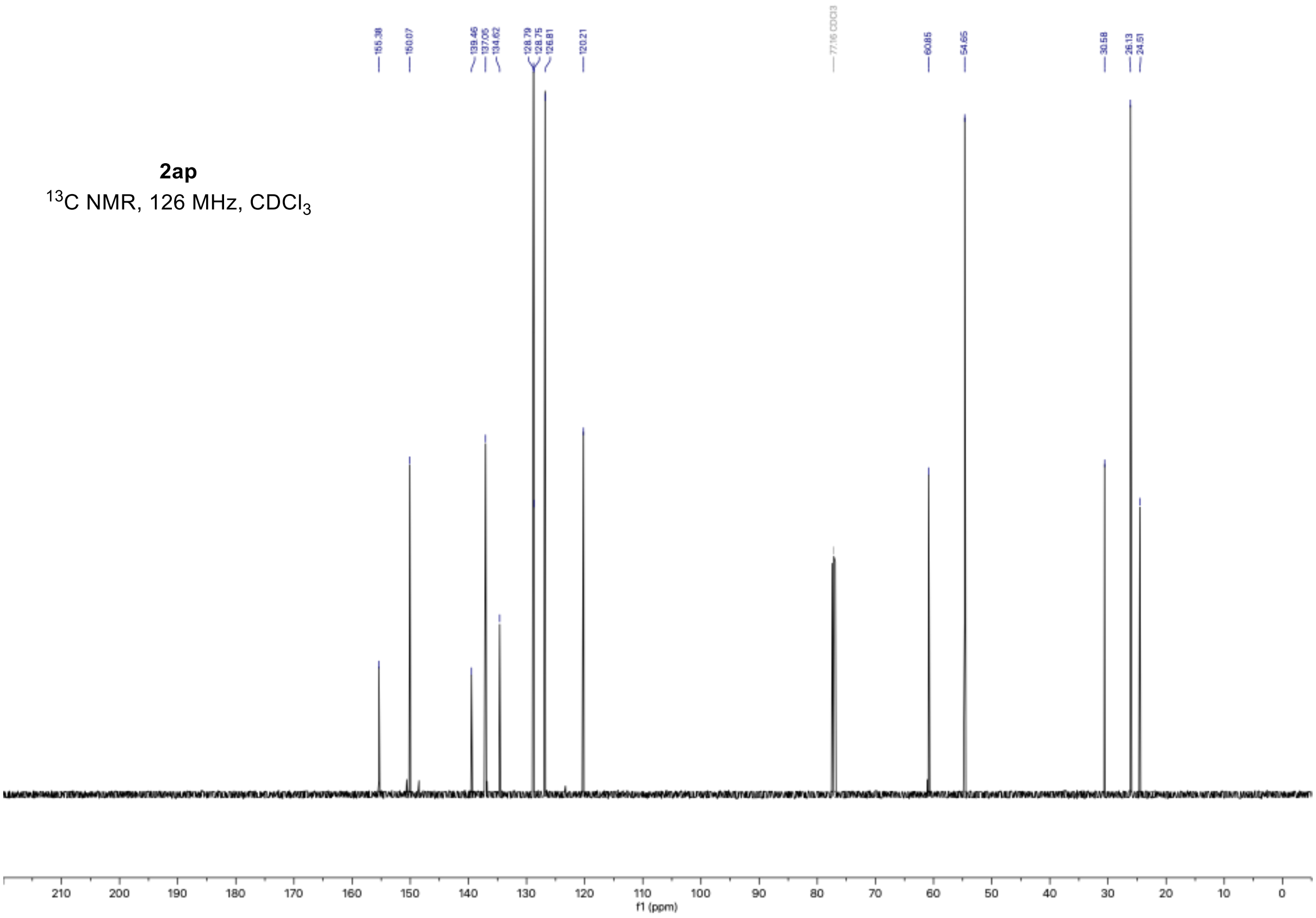
2ap
13C NMR, 126 MHz, CDCl3
155.38
150.07
139.46
137.06
134.62
128.79
128.75
126.81
120.21
77.16 CDCl3
60.86
54.65
30.58
26.13
24.51
f1 (ppm)

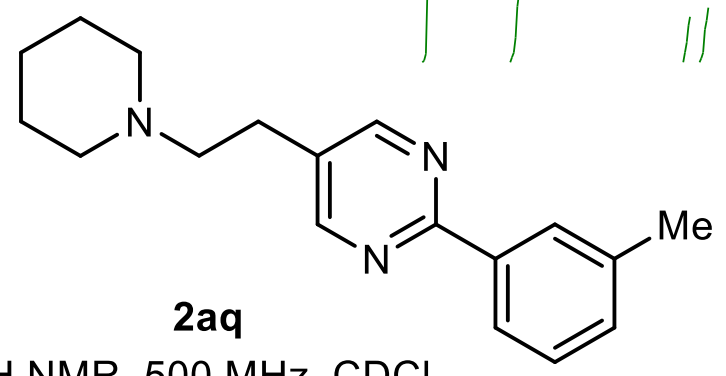

2aq

^{1}H NMR, 500 MHz, $CDCl_3$

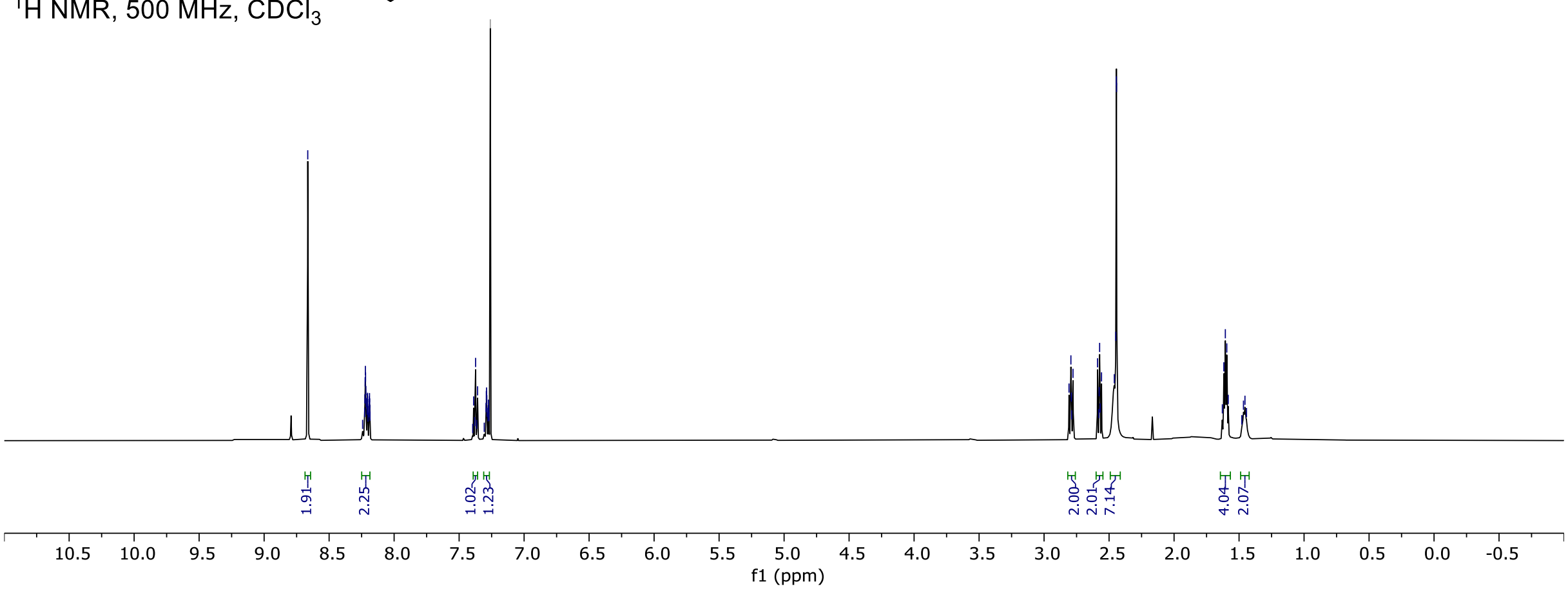

2aq

^{13}C NMR, 126 MHz, $CDCl_3$

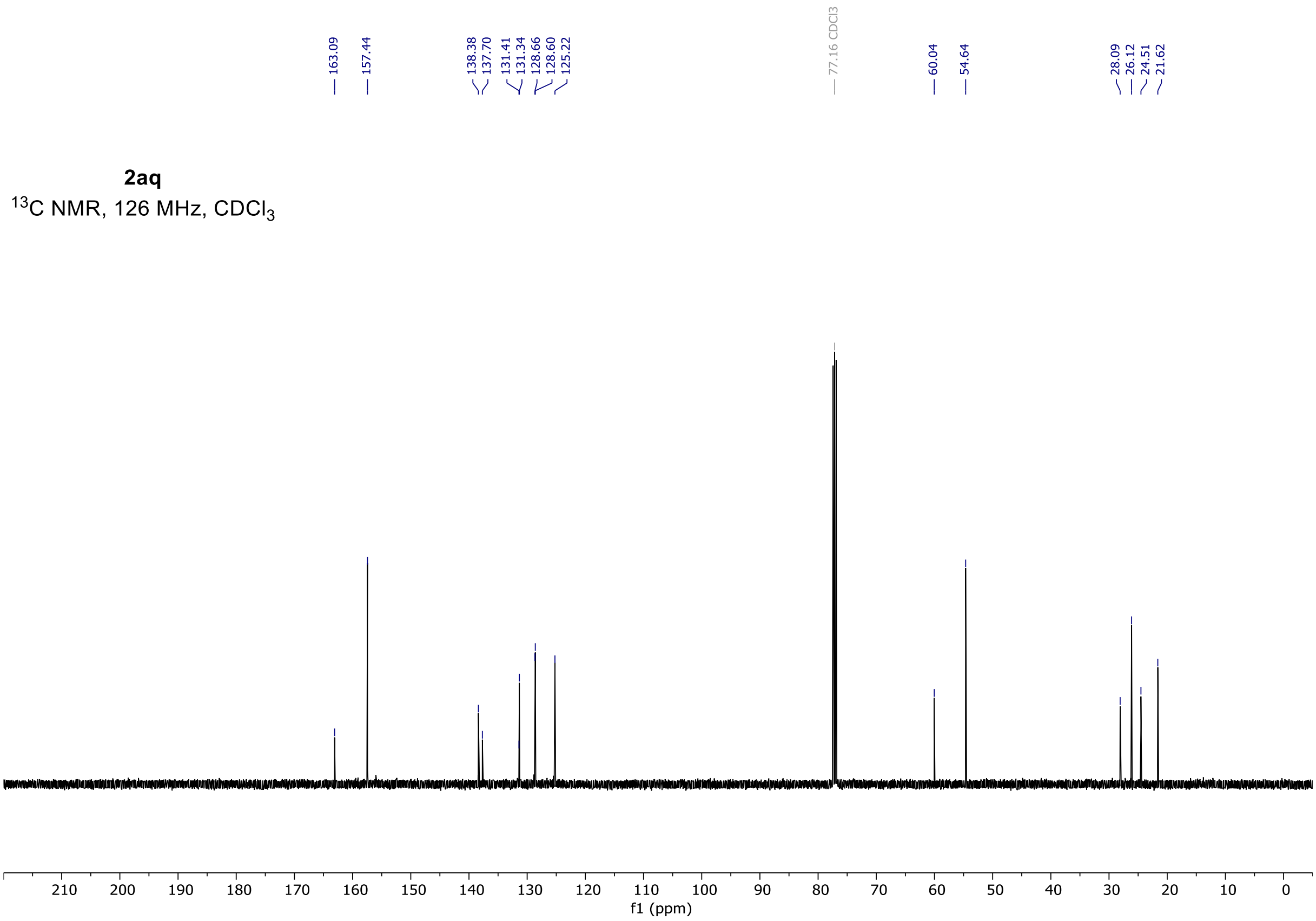

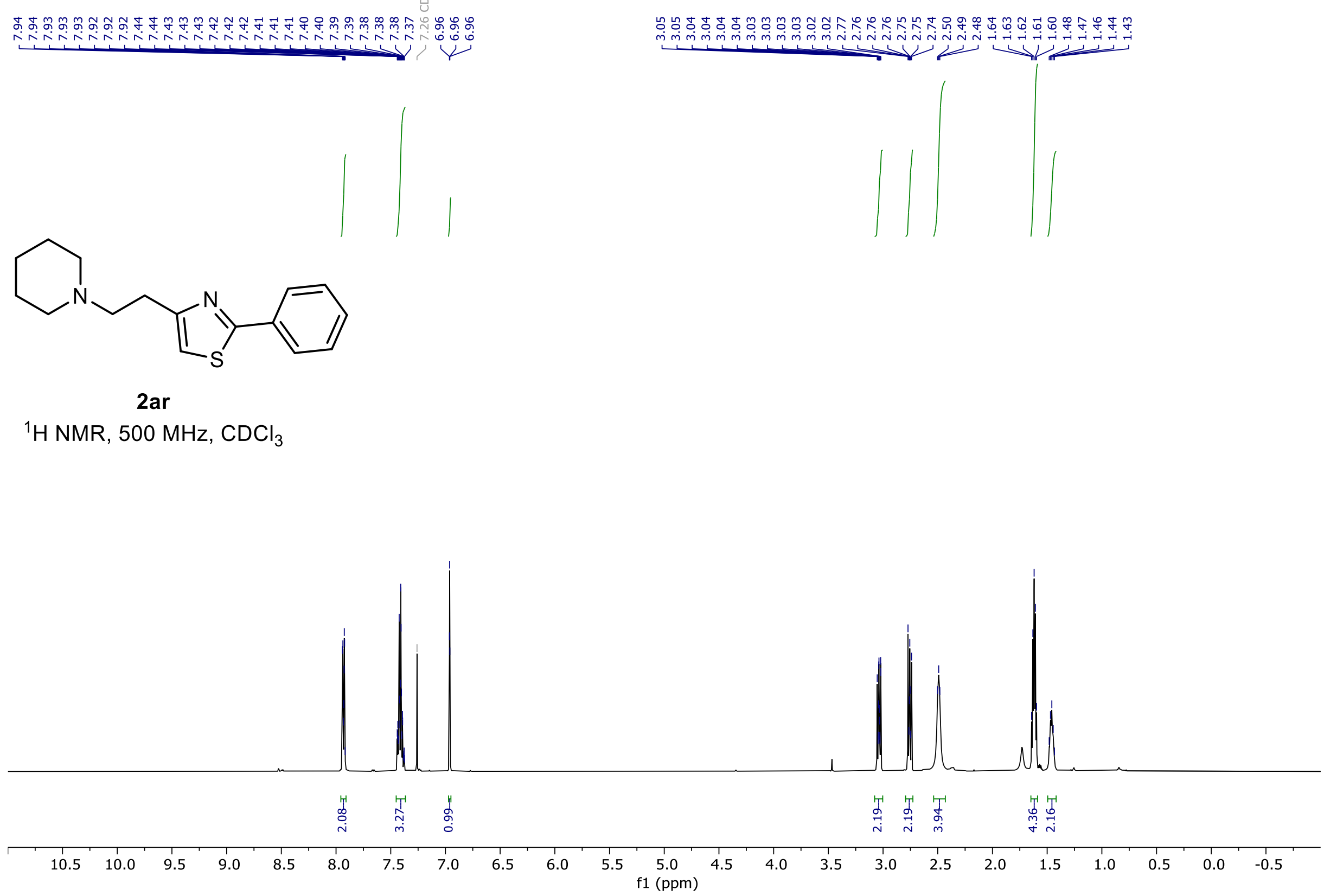

2ar
^{1}H NMR, 500 MHz, $CDCl_3$

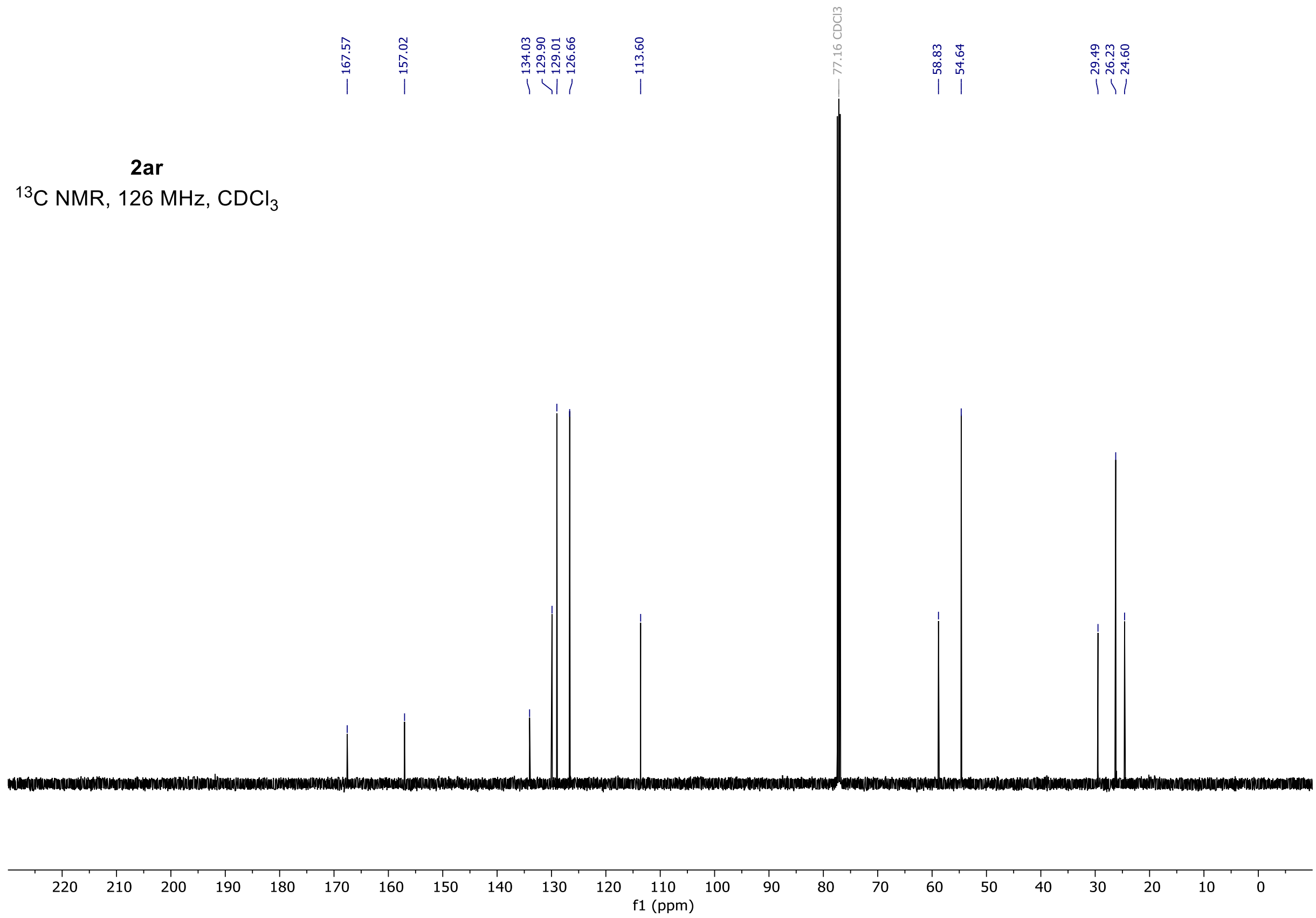

2ar
^{13}C NMR, 126 MHz, $CDCl_3$

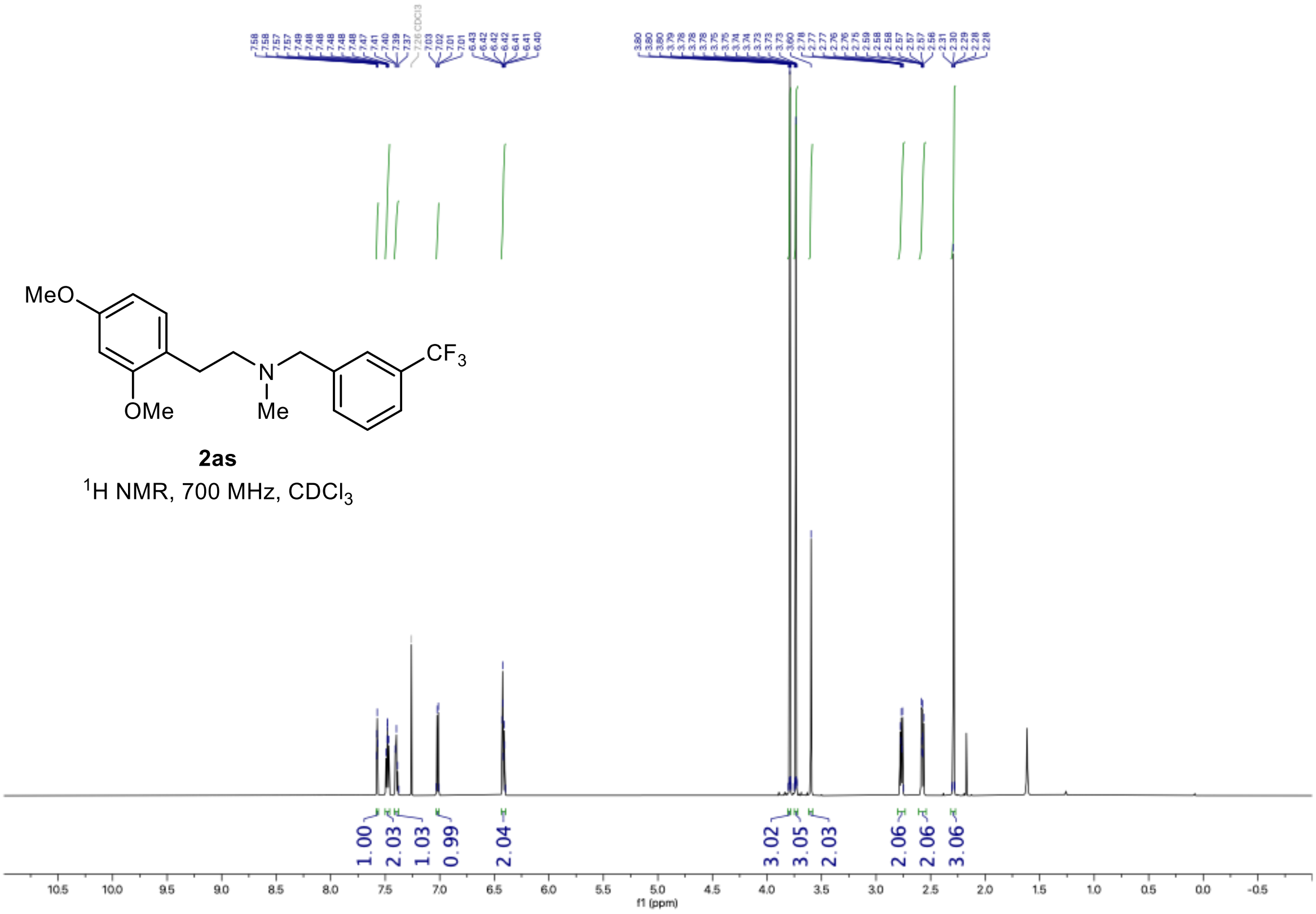

2as

^{1}H NMR, 700 MHz, $CDCl_3$

2as

^{13}C NMR, 176 MHz, $CDCl_3$

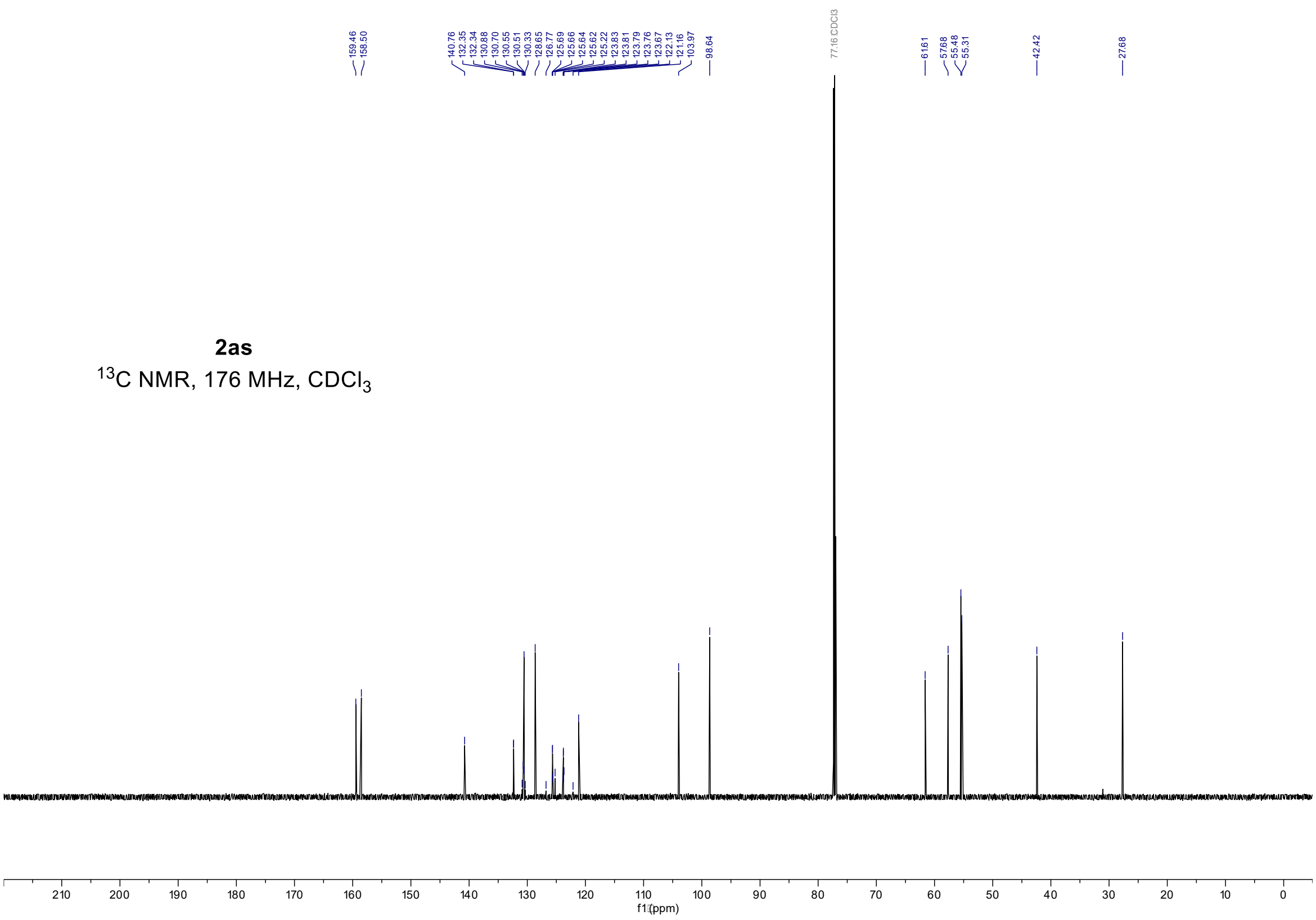

2as

^{19}F NMR, 376 MHz, $CDCl_3$

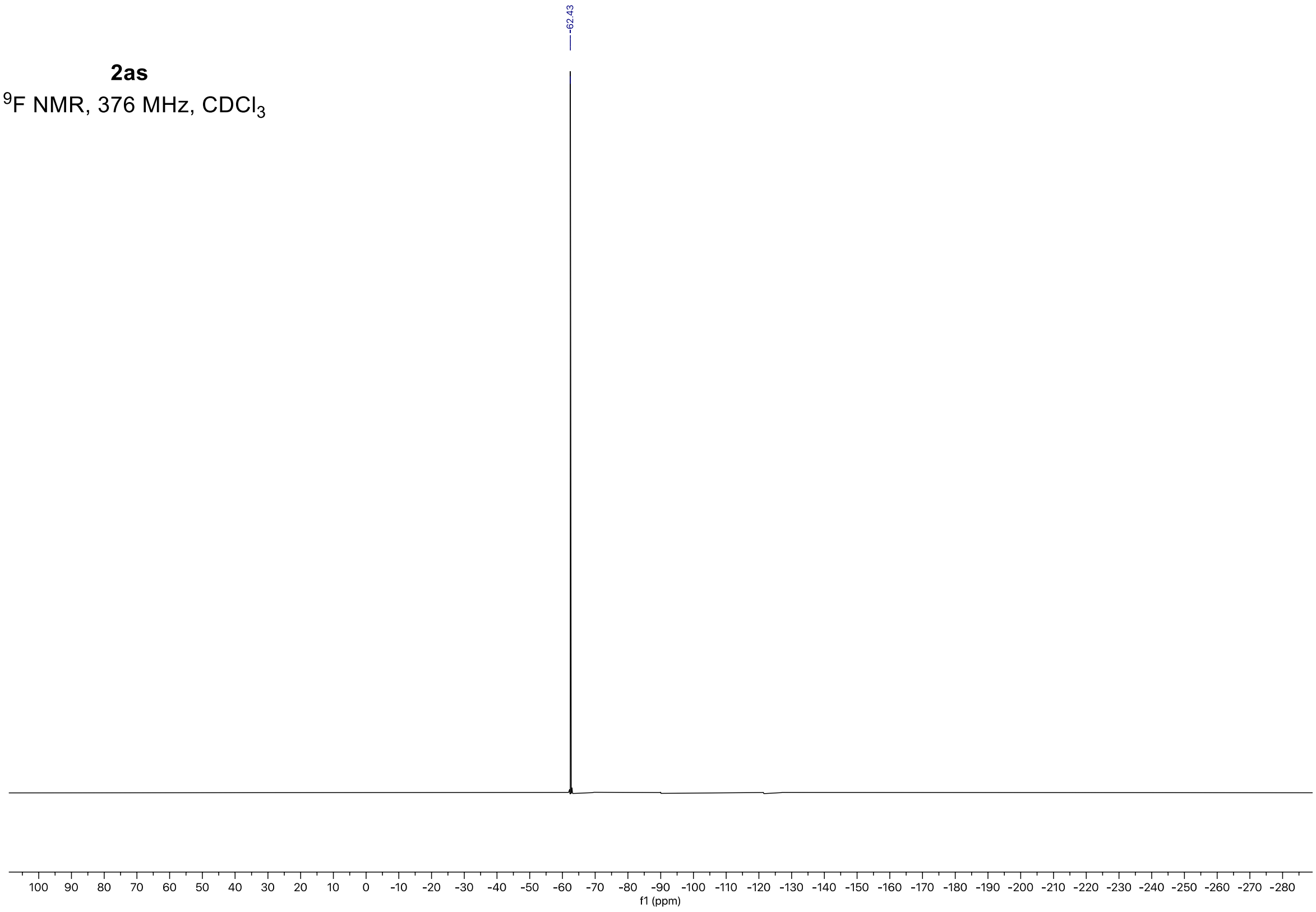

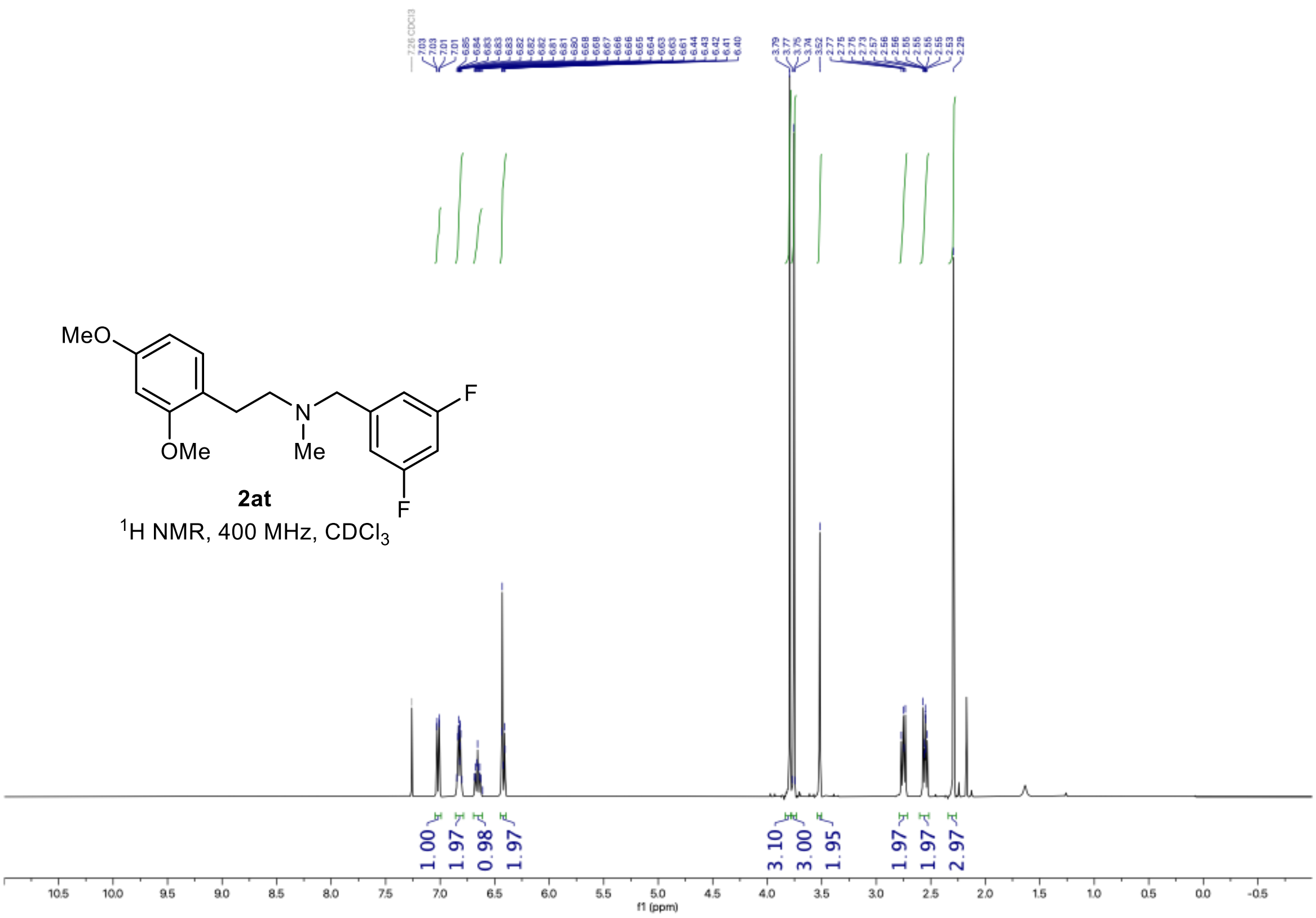

2at

^{1}H NMR, 400 MHz, $CDCl_3$

2at

^{13}C NMR, 176 MHz, $CDCl_3$

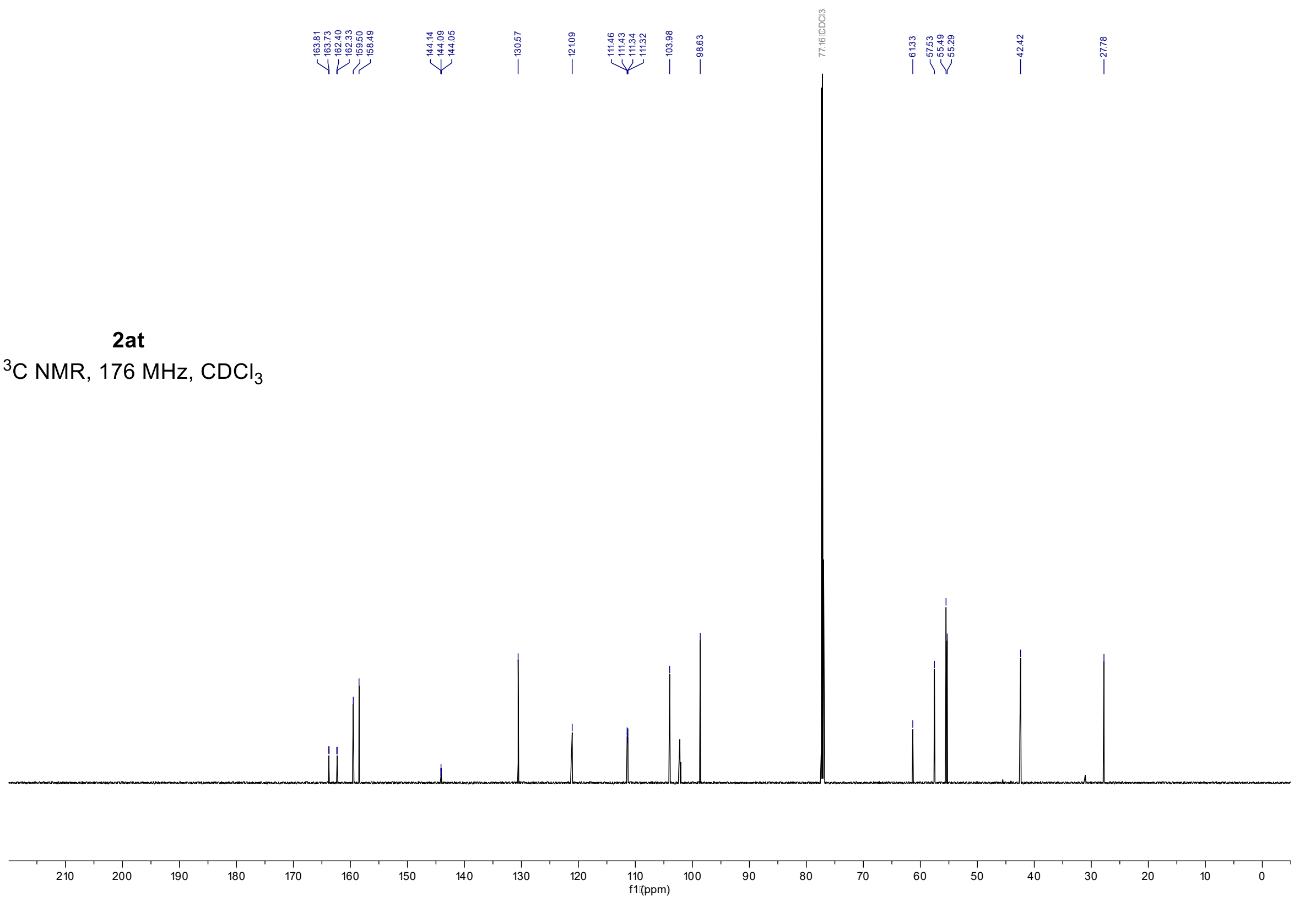

2at
^{19}F NMR, 376 MHz, $CDCl_3$

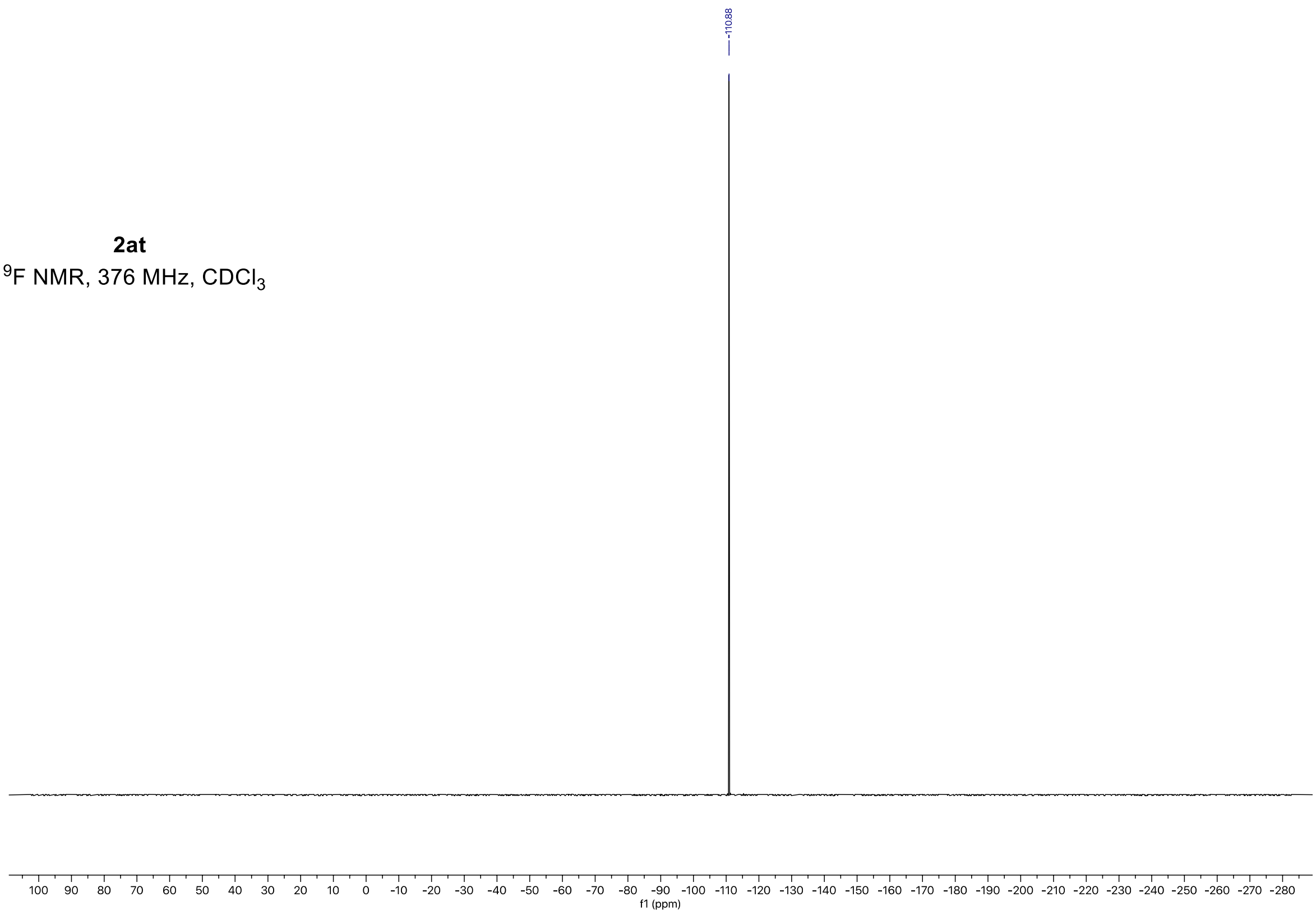

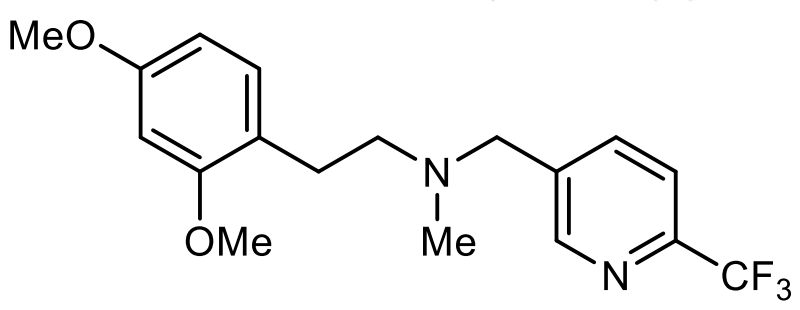

2au

^{1}H NMR, 500 MHz, $CDCl_3$

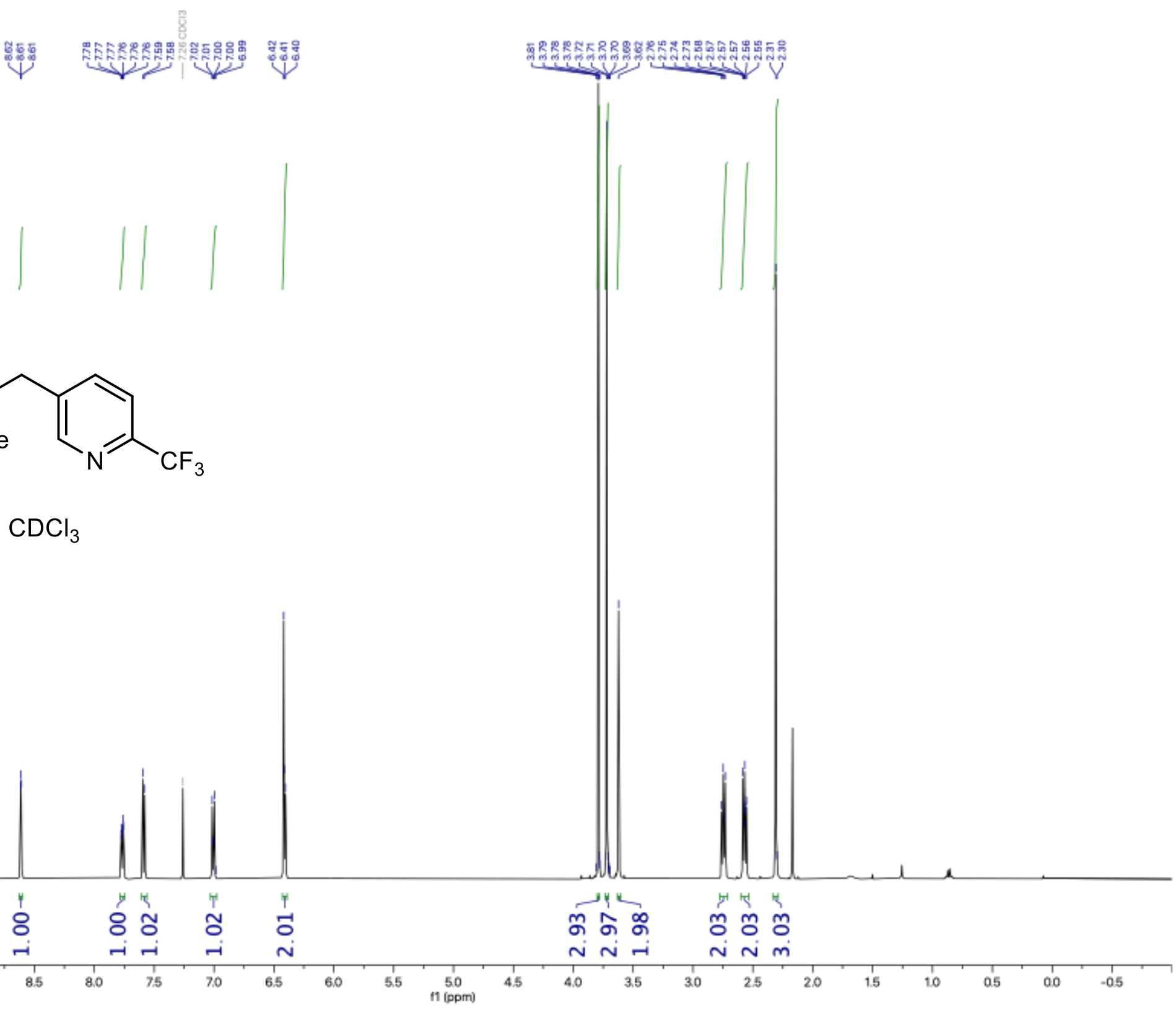

2au

^{13}C NMR, 176 MHz, $CDCl_3$

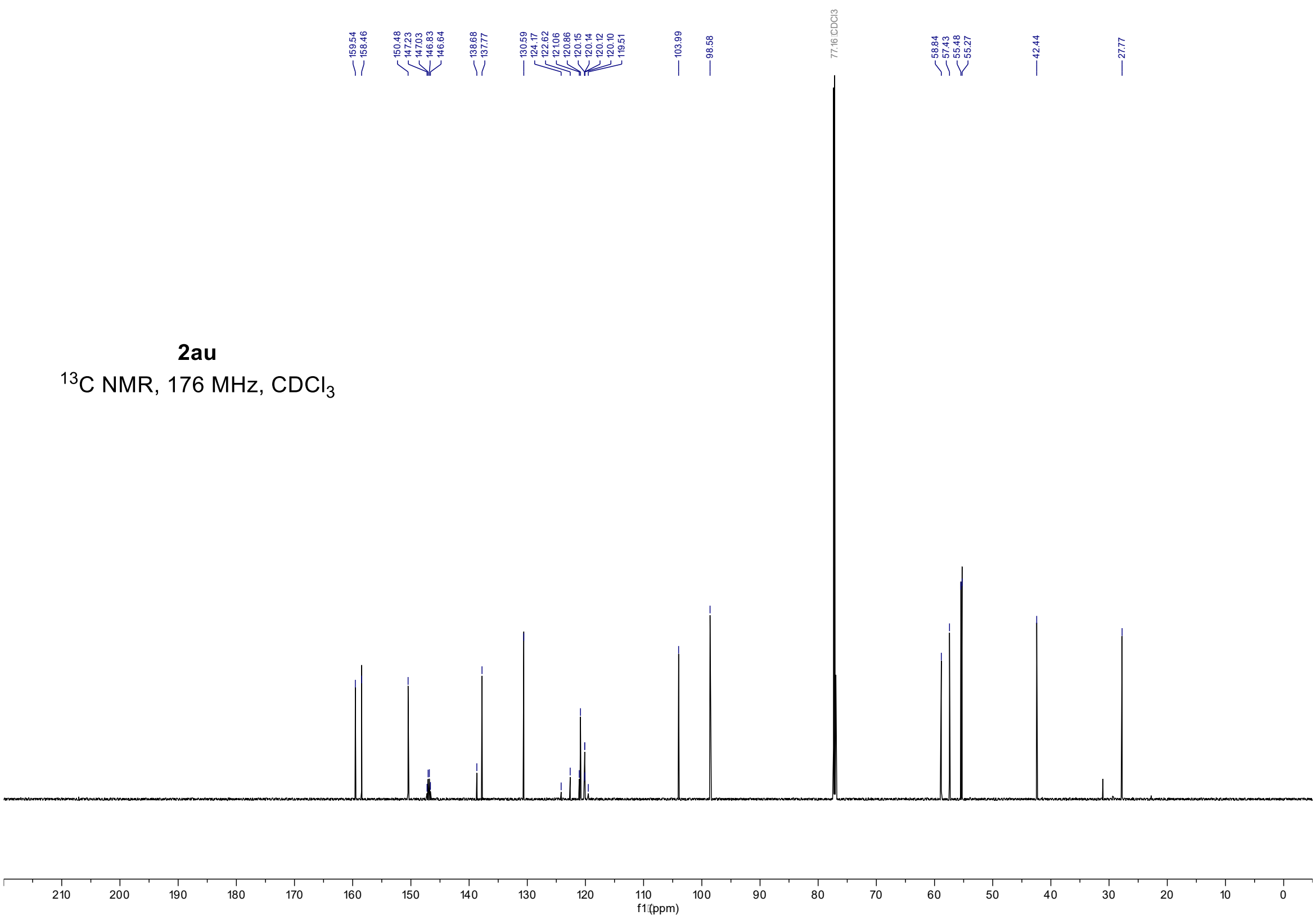

2au

^{19}F NMR, 376 MHz, $CDCl_3$

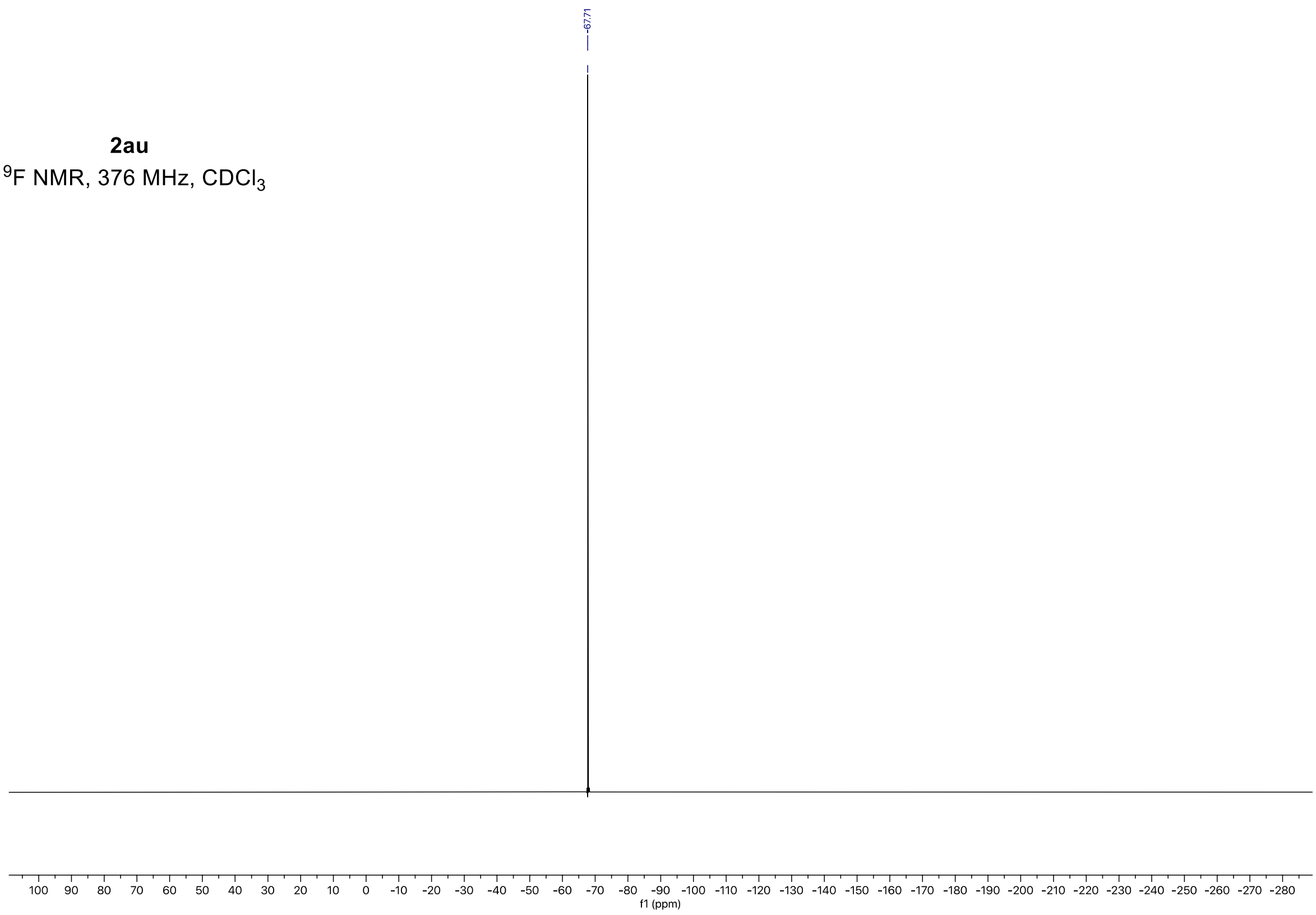

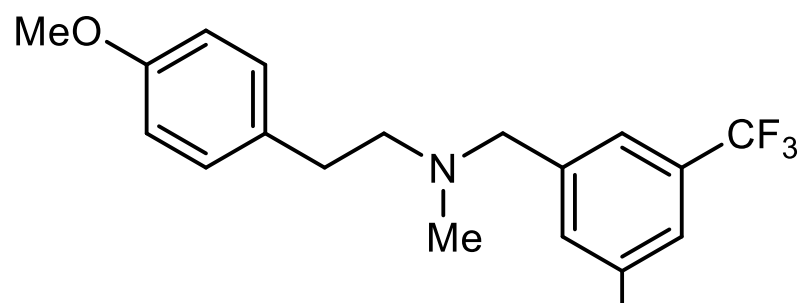

2av

^{1}H NMR, 500 MHz, $CDCl_3$

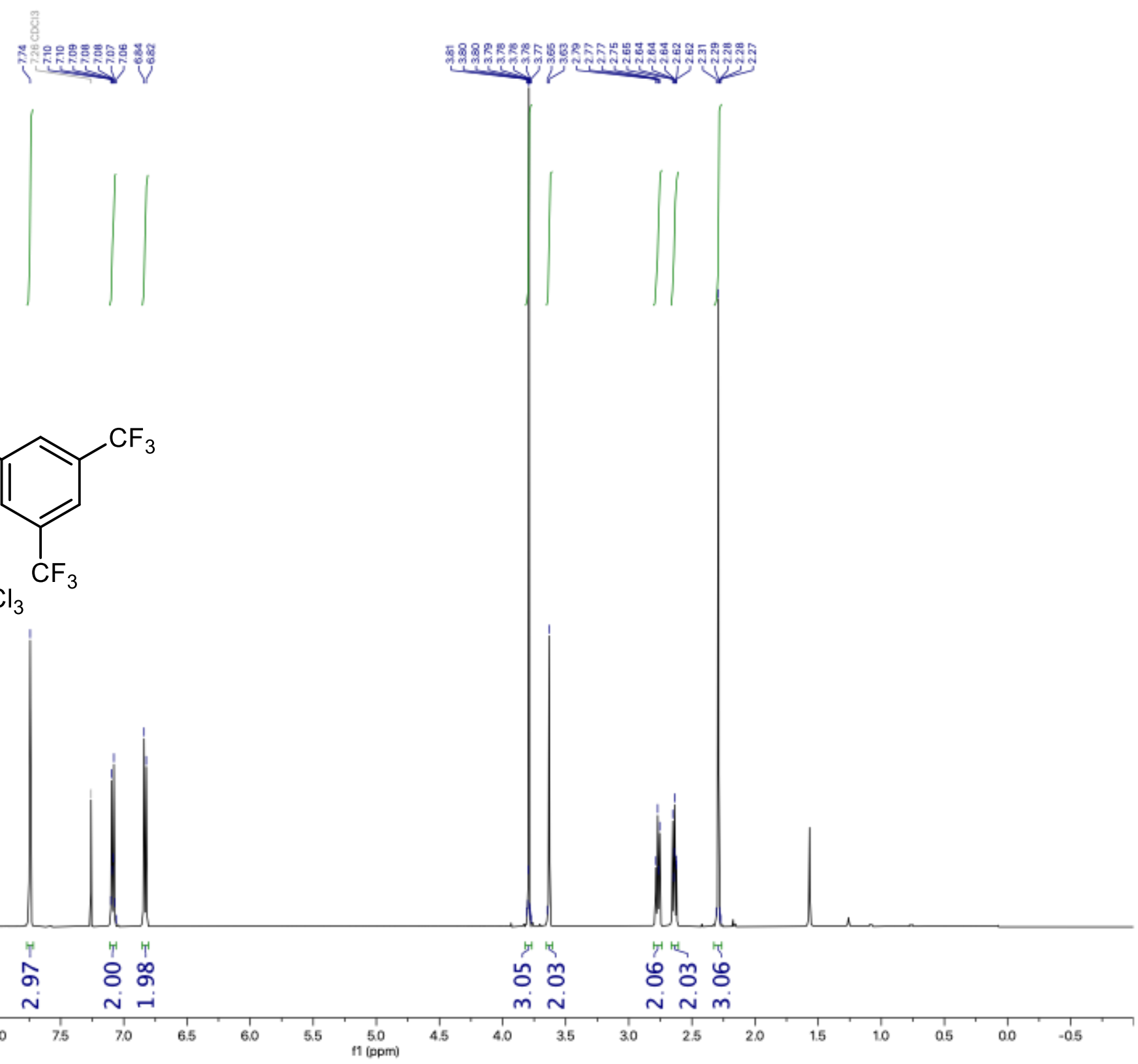

2av

^{13}C NMR, 126 MHz, $CDCl_3$

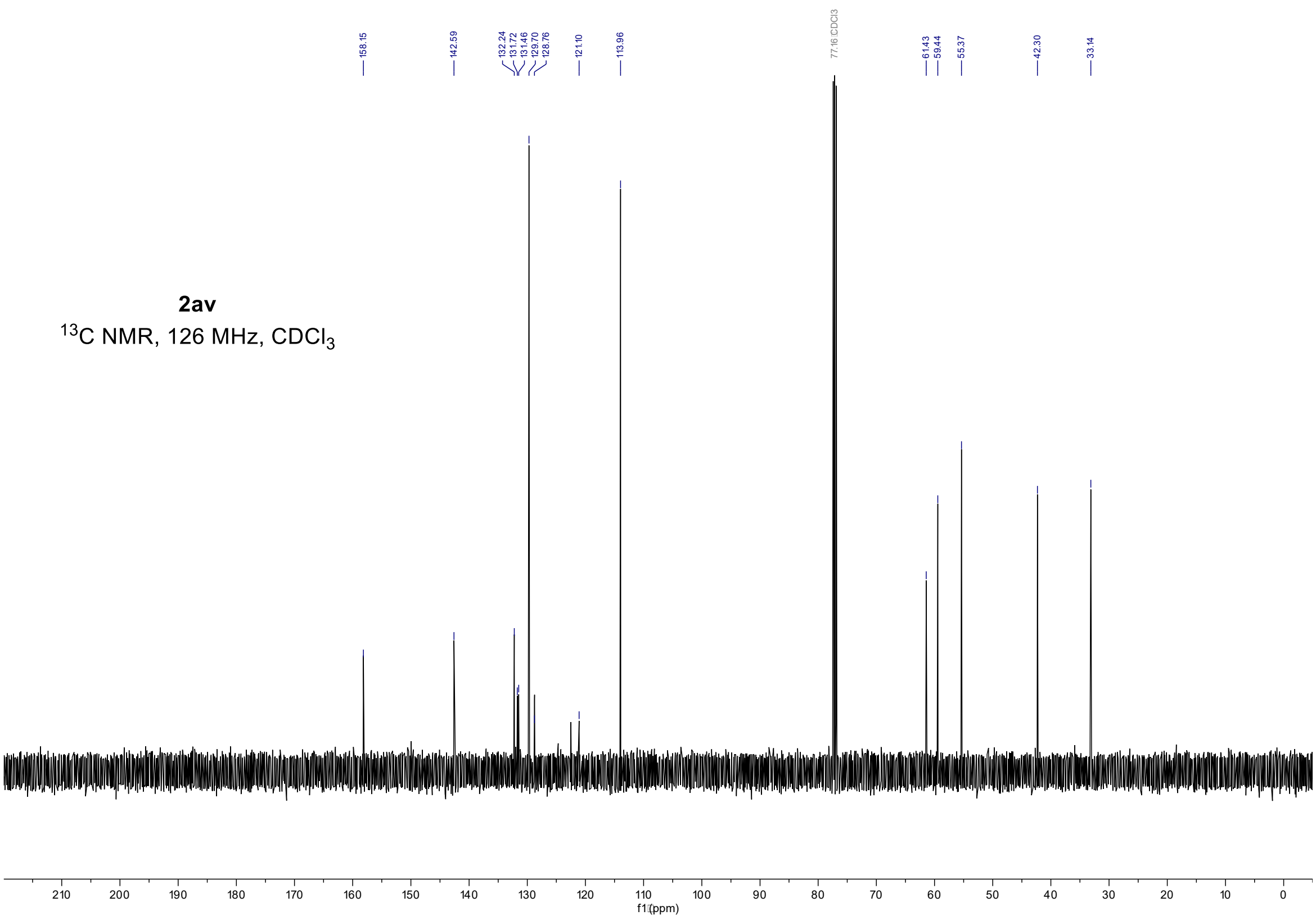

2av
^{19}F NMR, 376 MHz, $CDCl_3$

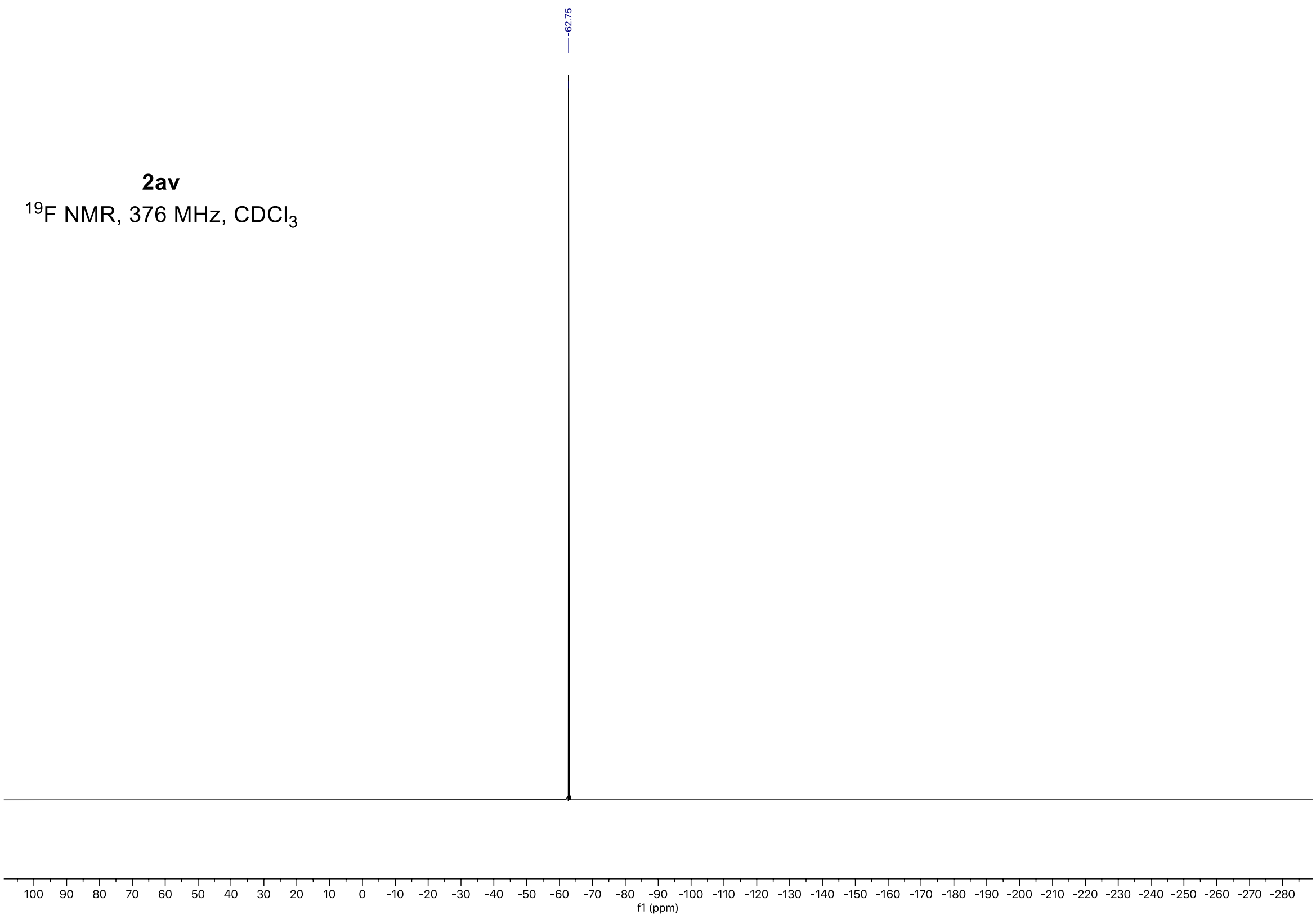

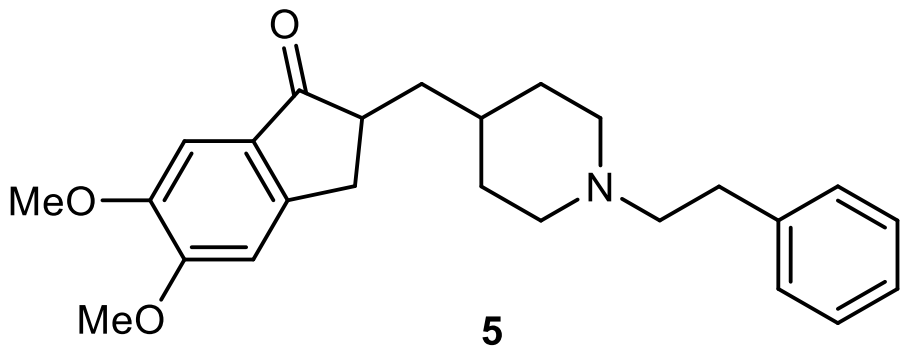

5

^{1}H NMR, 500 MHz, $CDCl_3$

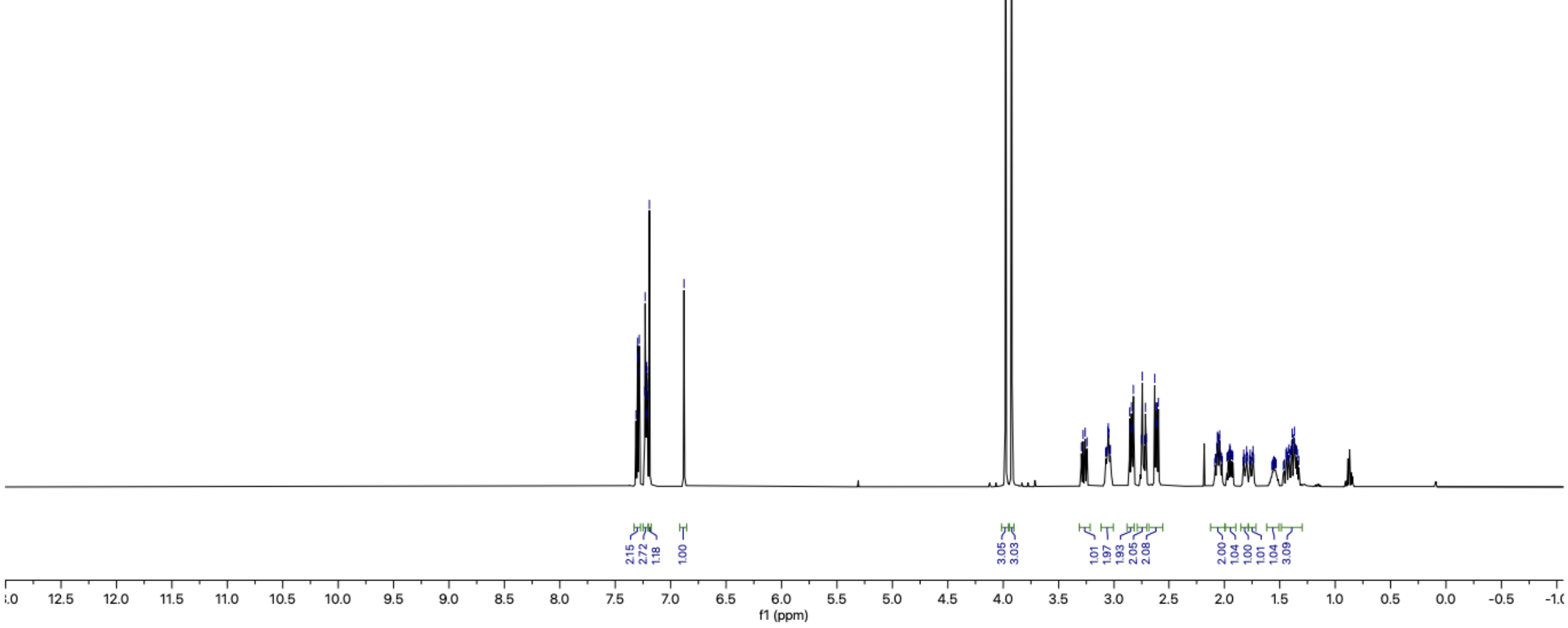

5

^{13}C NMR, 126 MHz, $CDCl_3$

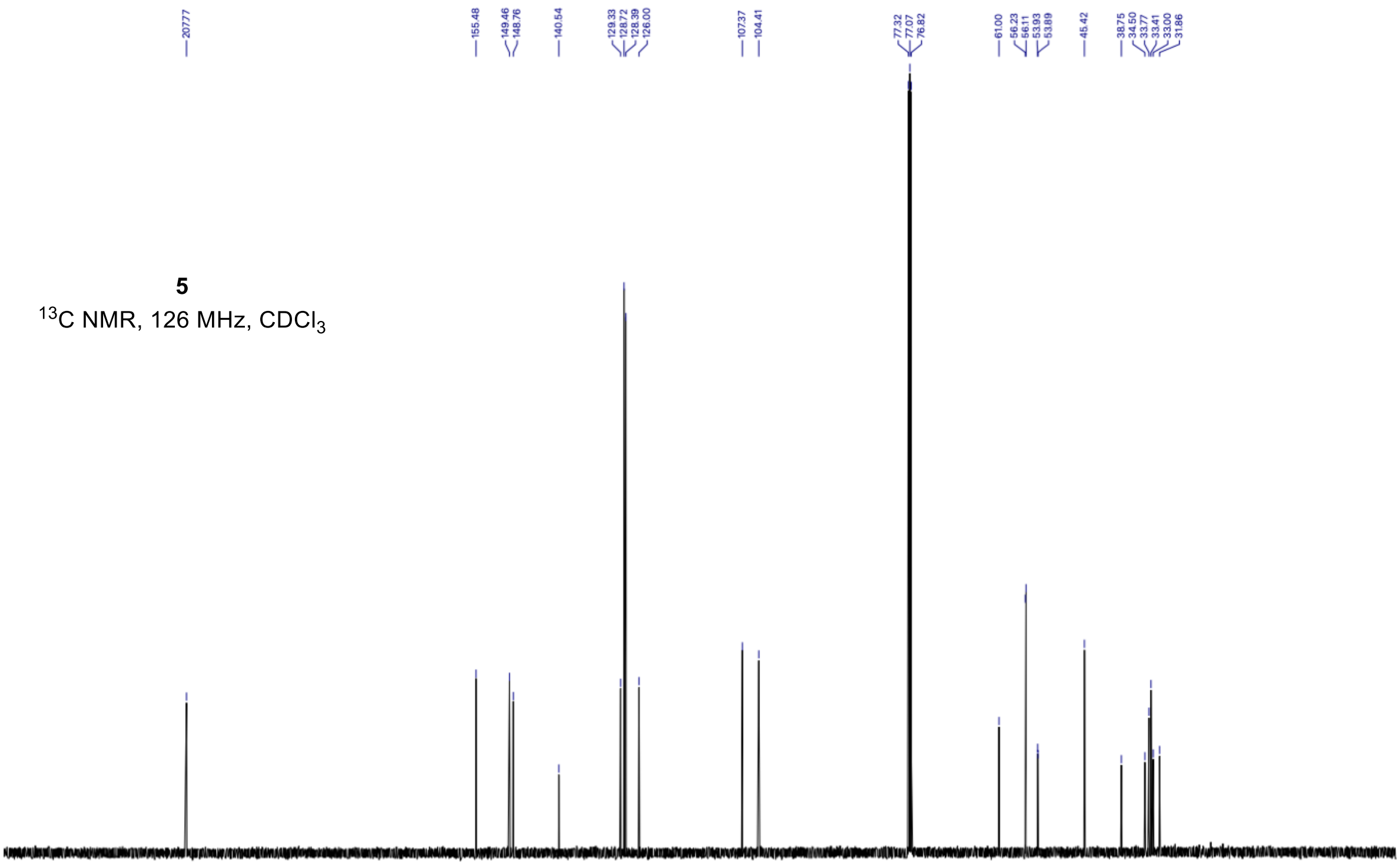

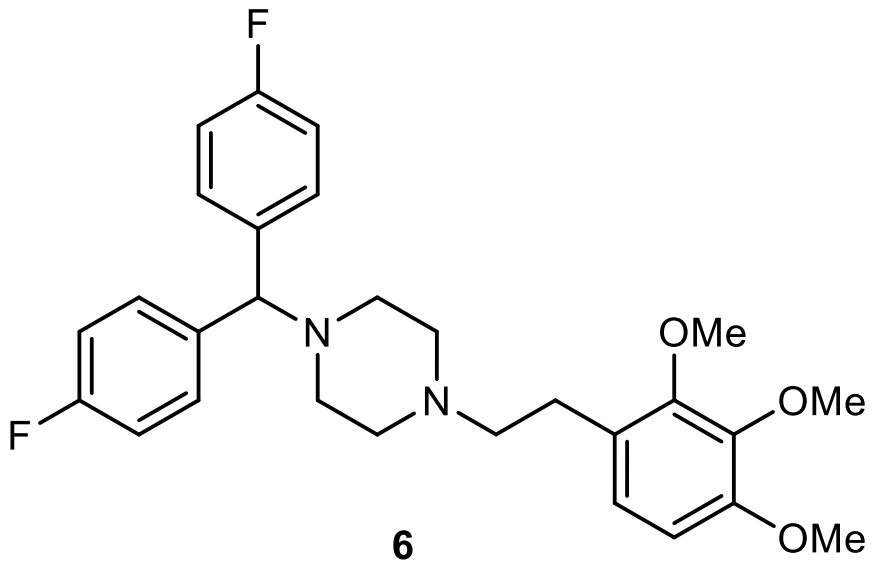

6

^{1}H NMR, 700 MHz, $CDCl_3$

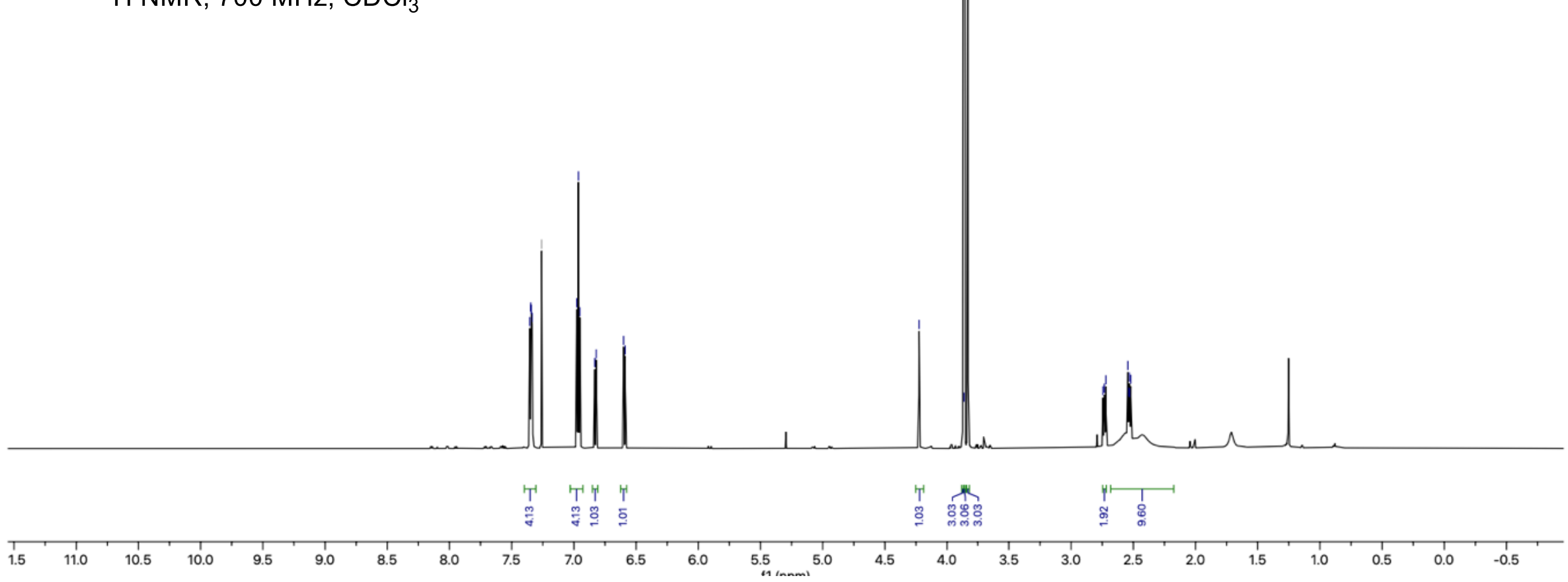

6

^{13}C NMR, 176 MHz, $CDCl_3$

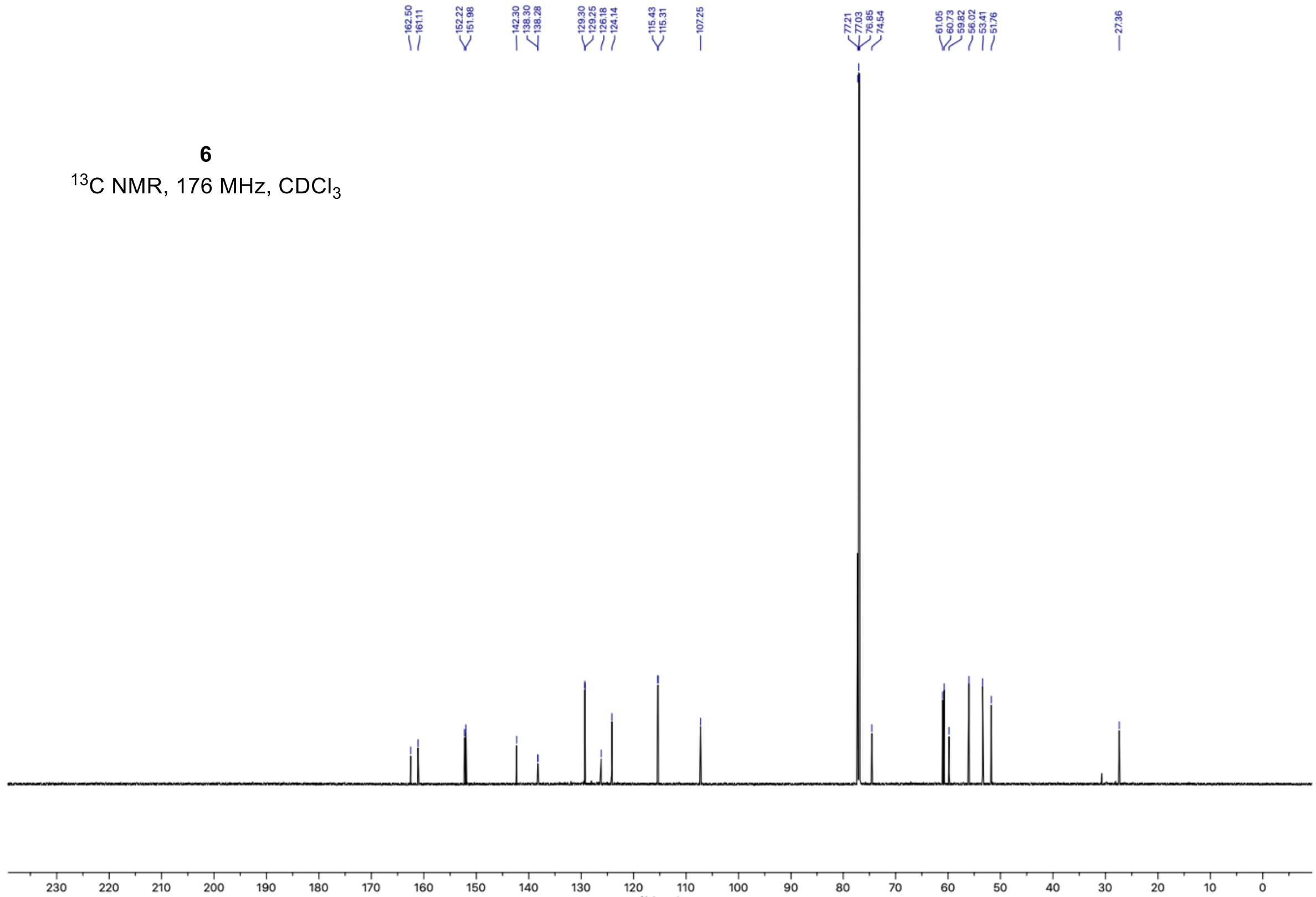

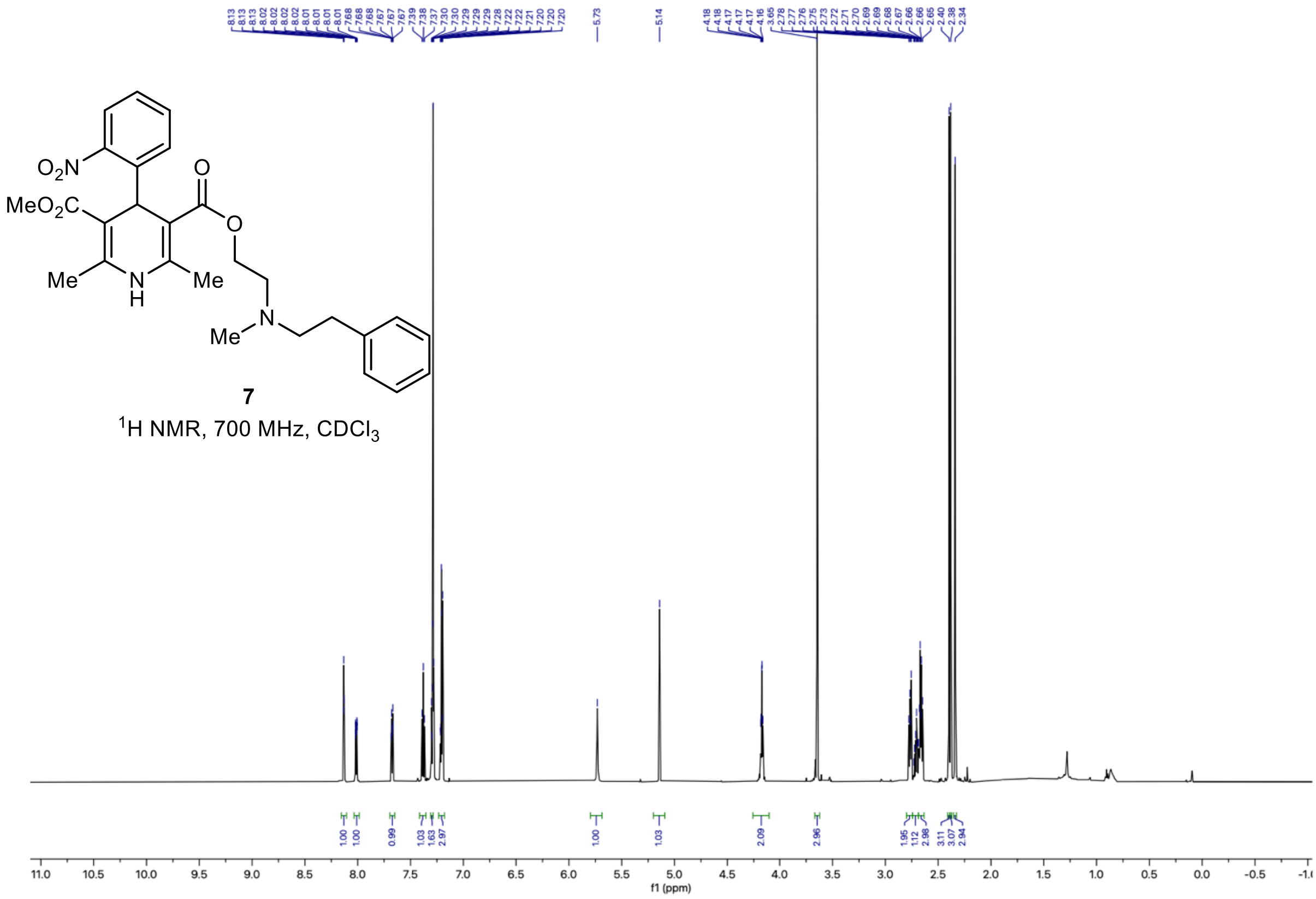

7

^{1}H NMR, 700 MHz, $CDCl_3$

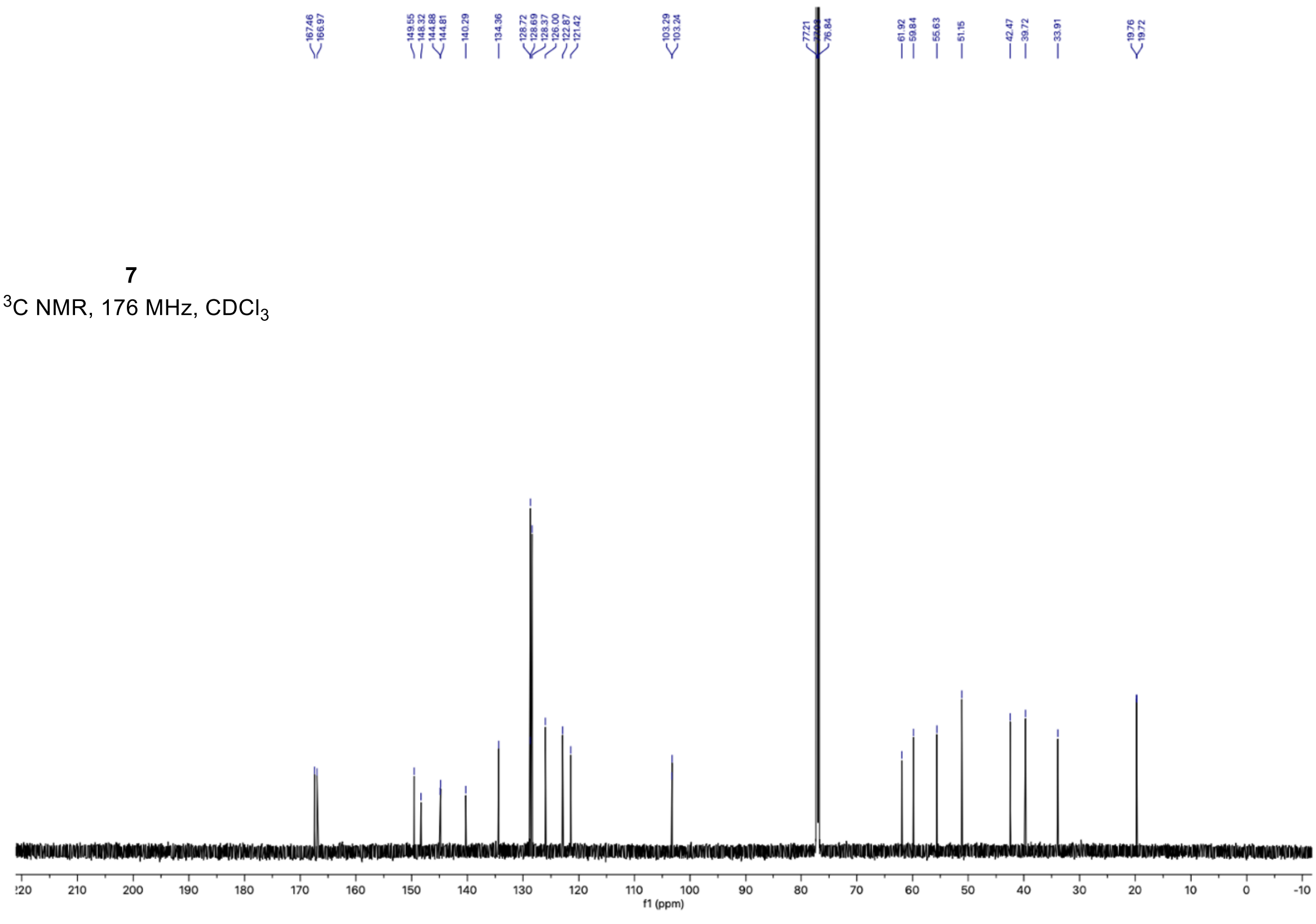

7

^{13}C NMR, 176 MHz, $CDCl_3$

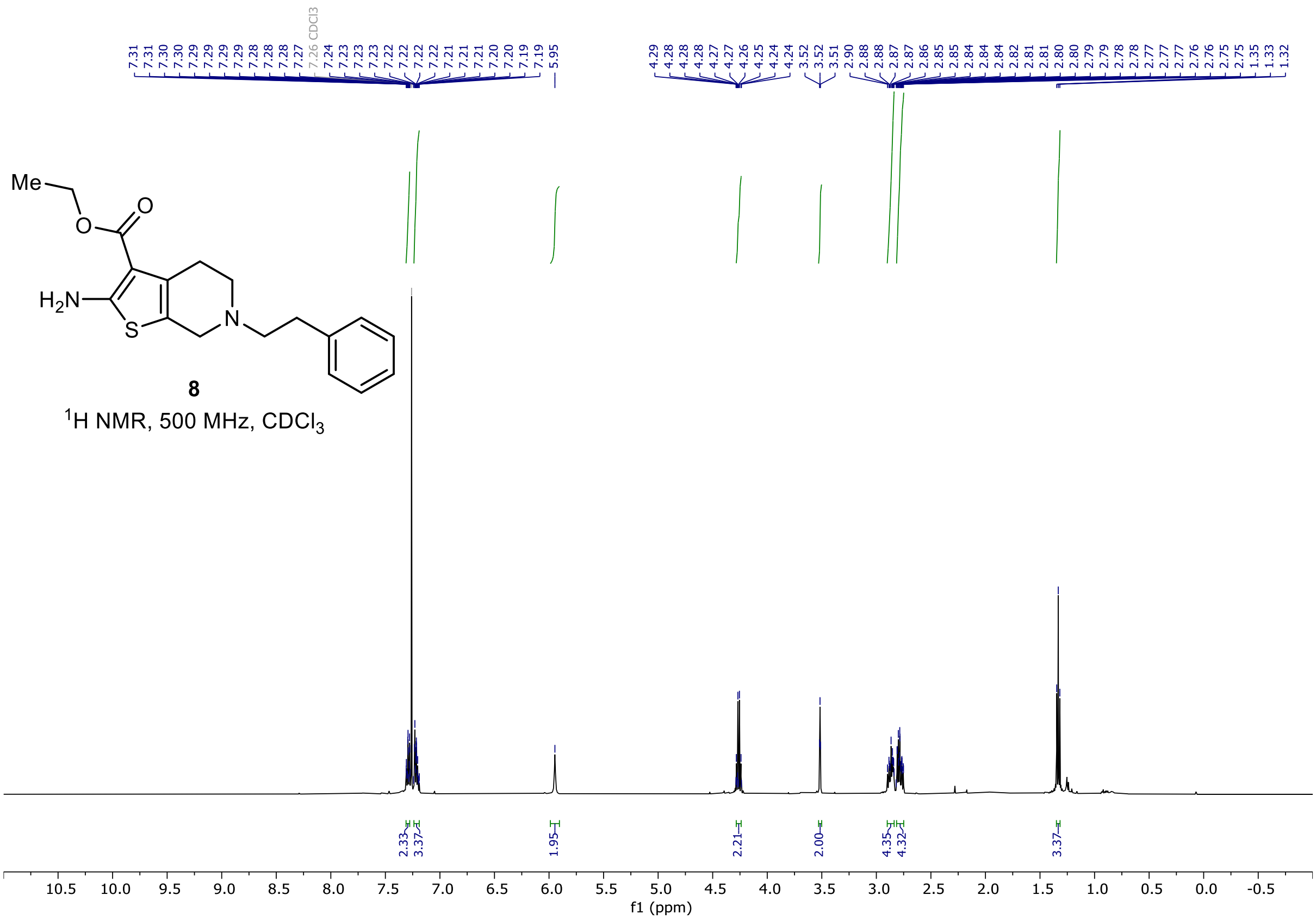
7.31
7.31
7.30
7.29
7.29
7.29
7.28
7.28
7.28
7.27
7.26 CDCl3
7.24
7.23
7.23
7.23
7.22
7.22
7.22
7.22
7.21
7.21
7.21
7.20
7.19
7.19
5.95
4.29
4.28
4.28
4.27
4.27
4.26
4.25
4.24
4.24
3.52
3.52
3.51
2.90
2.88
2.88
2.87
2.87
2.86
2.85
2.84
2.84
2.82
2.81
2.81
2.80
2.80
2.80
2.79
2.79
2.78
2.78
2.77
2.77
2.77
2.76
2.76
2.75
2.75
1.35
1.33
1.32
Me
O
O
H2N
S
N
8
1H NMR, 500 MHz, CDCl3
2.33
3.37
1.95
2.21
2.00
4.35
4.32
3.37
10.5 10.0 9.5 9.0 8.5 8.0 7.5 7.0 6.5 6.0 5.5 5.0 4.5 4.0 3.5 3.0 2.5 2.0 1.5 1.0 0.5 0.0 -0.5
f1 (ppm)

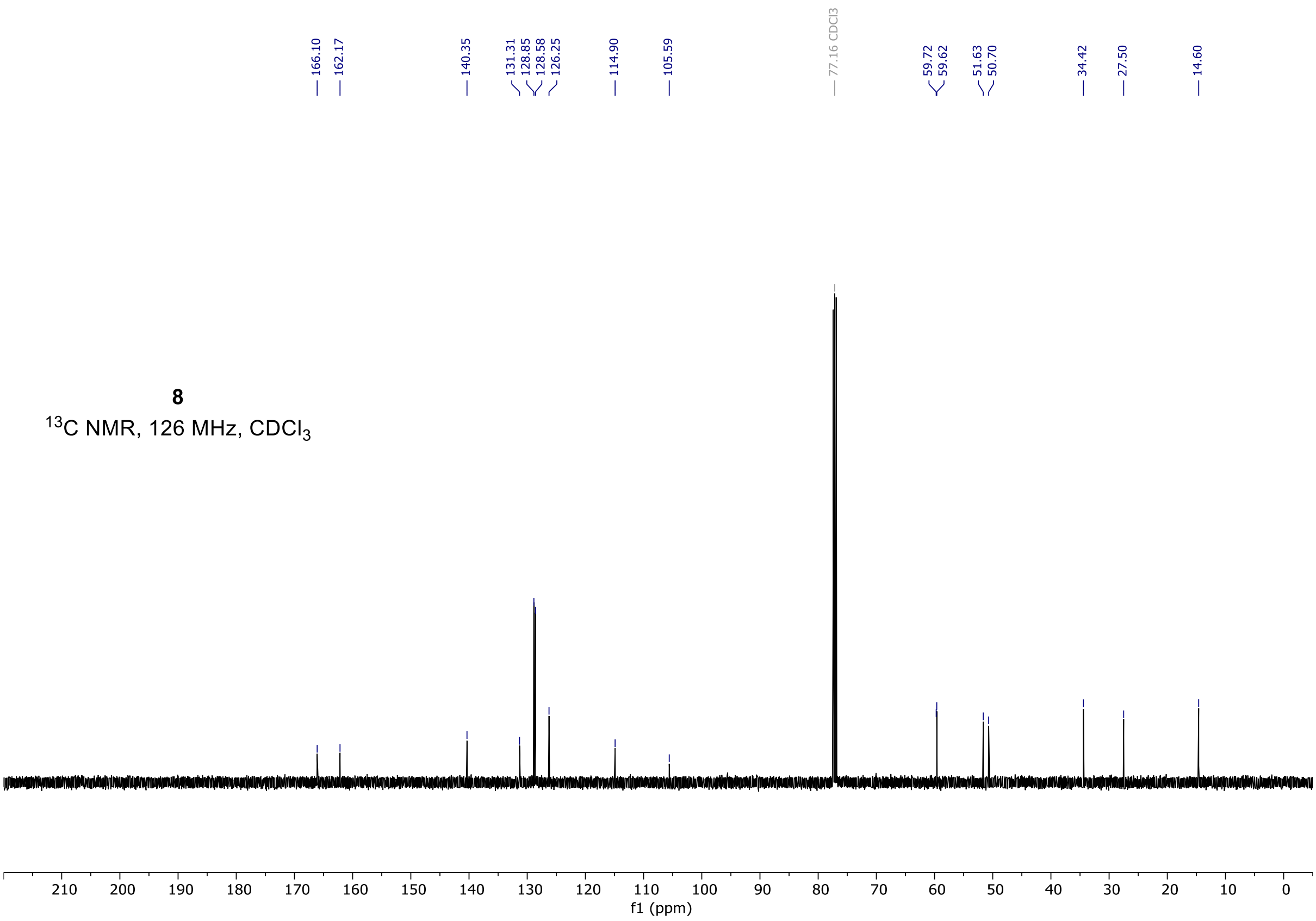
166.10
162.17
140.35
131.31
128.85
128.58
126.25
114.90
105.59
77.16 CDCl3
59.72
59.62
51.63
50.70
34.42
27.50
14.60
8
13C NMR, 126 MHz, CDCl3
210 200 190 180 170 160 150 140 130 120 110 100 90 80 70 60 50 40 30 20 10 0
f1 (ppm)

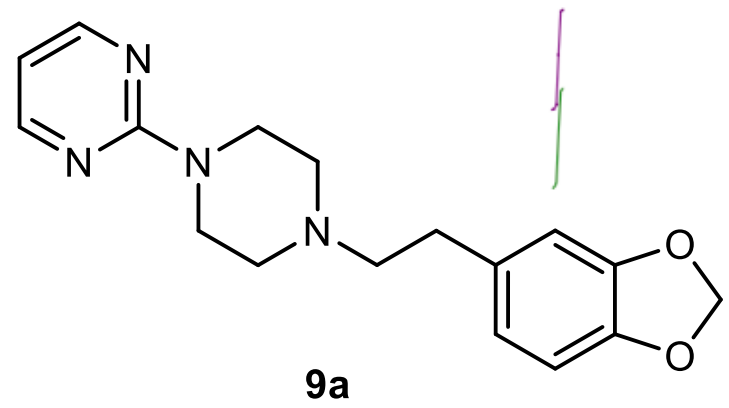

9a

^{1}H NMR, 500 MHz, $CDCl_3$

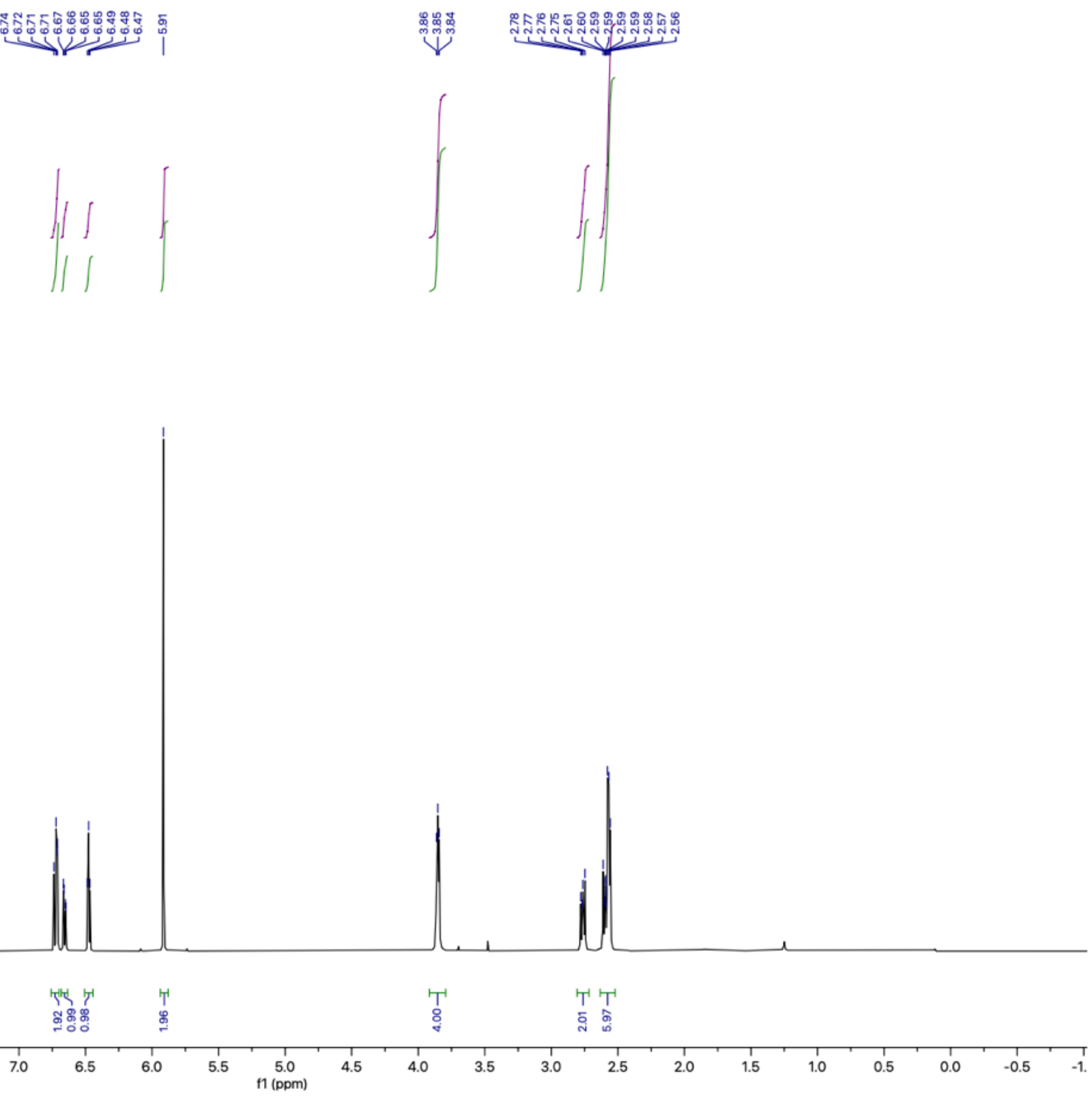

9a

^{13}C NMR, 126 MHz, $CDCl_3$

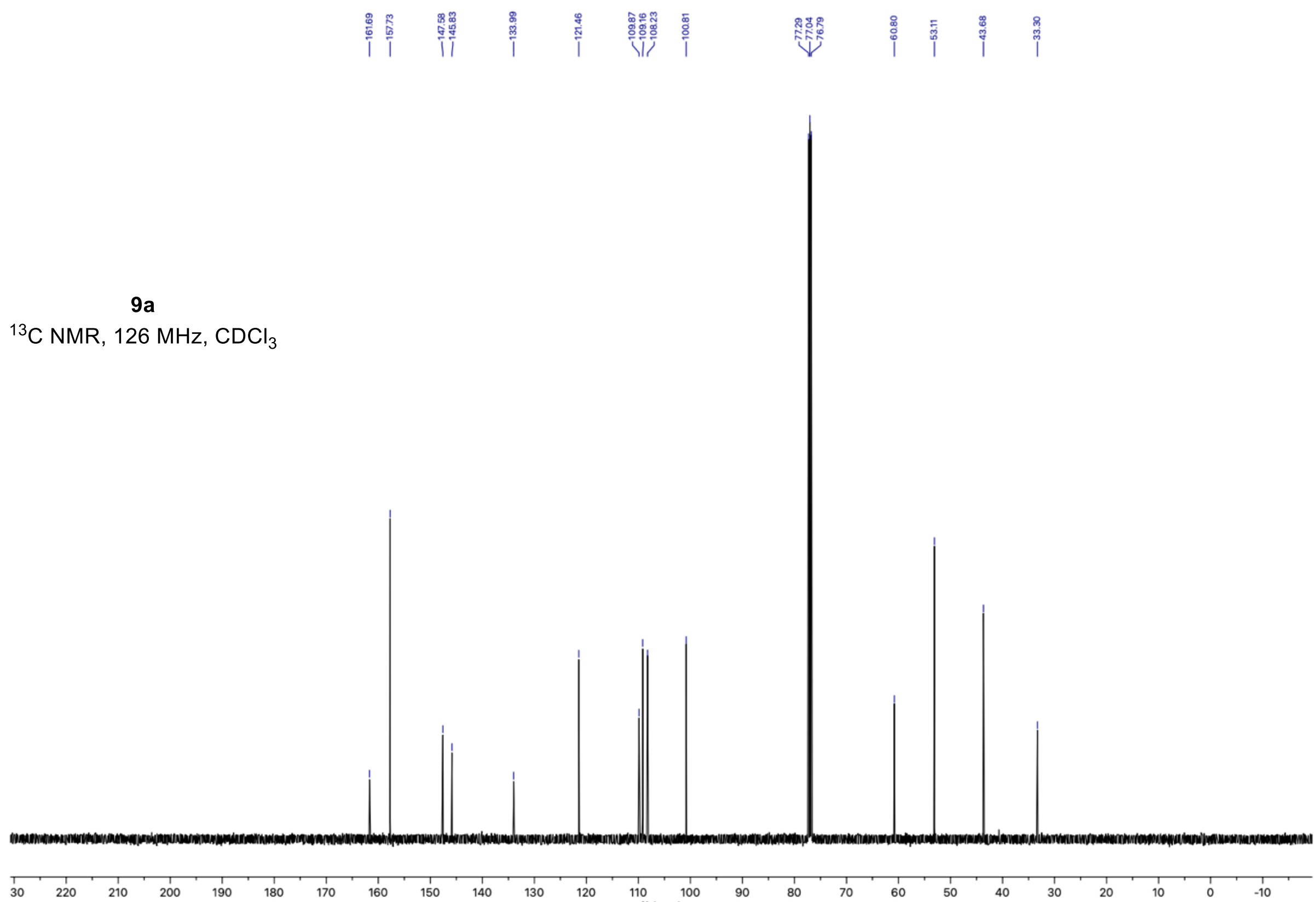

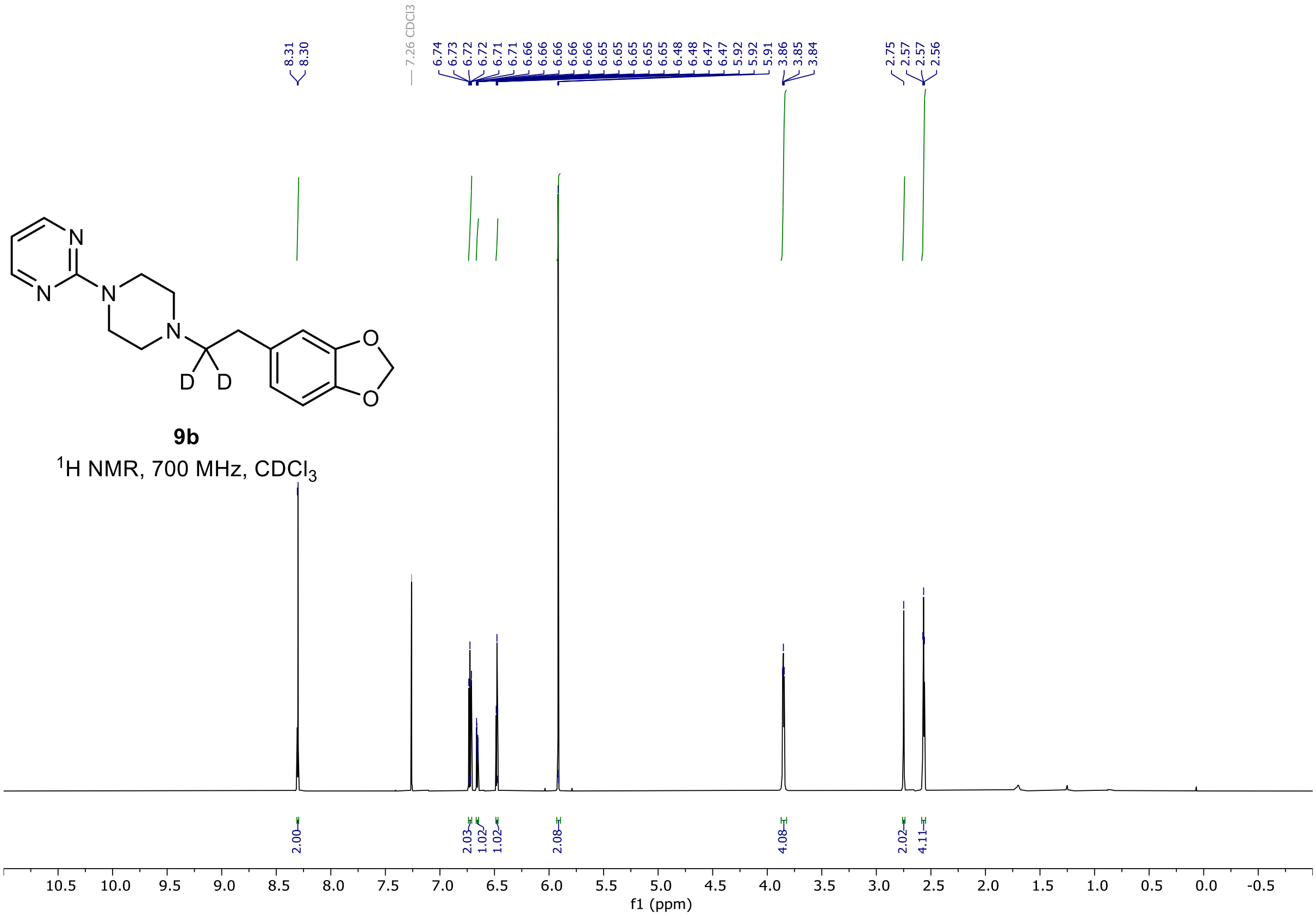
9b
¹H NMR, 700 MHz, CDCl₃
f1 (ppm)

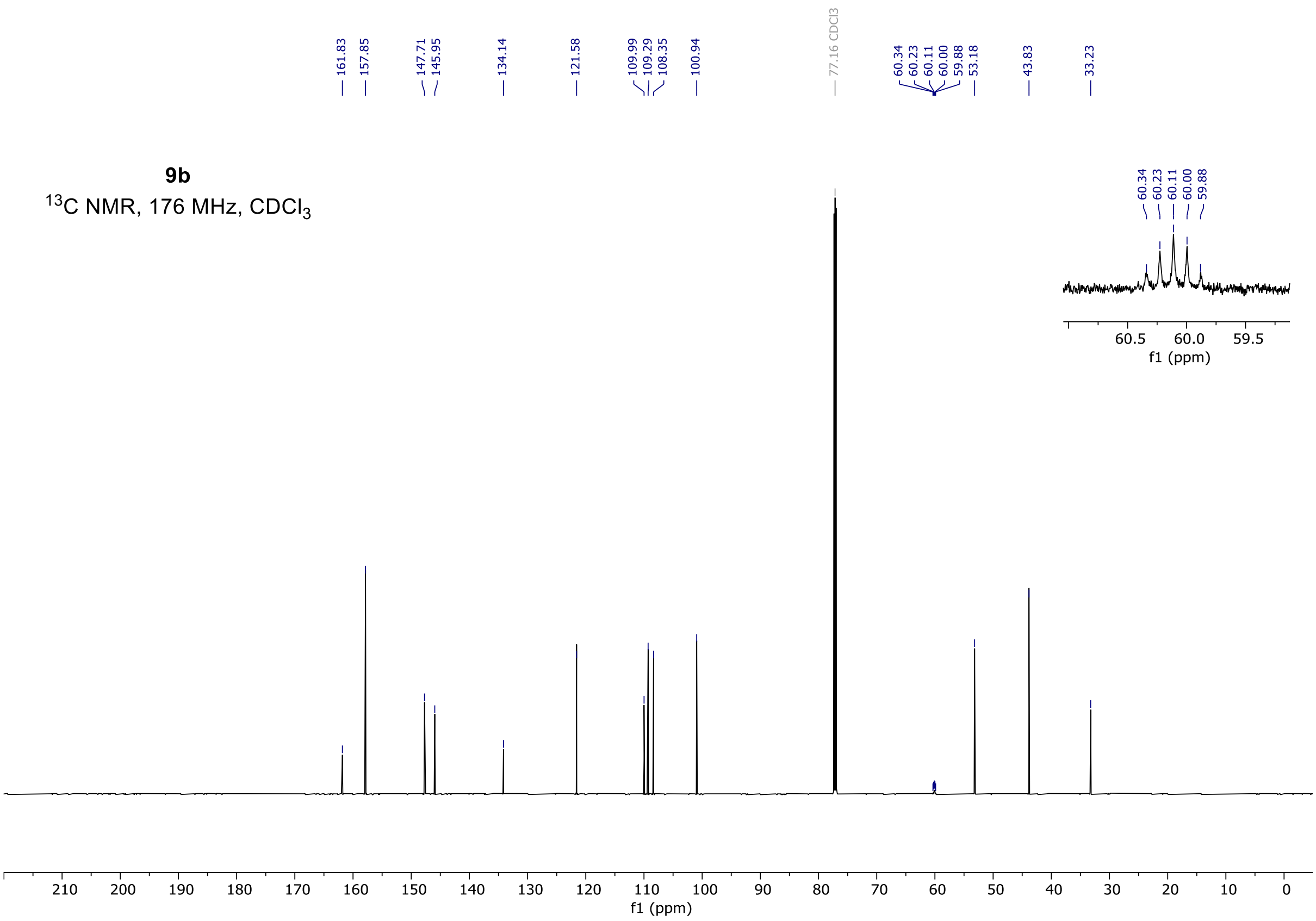
9b
¹³C NMR, 176 MHz, CDCl₃
f1 (ppm)

9b
^{2}H NMR, 107 MHz, $CDCl_3$

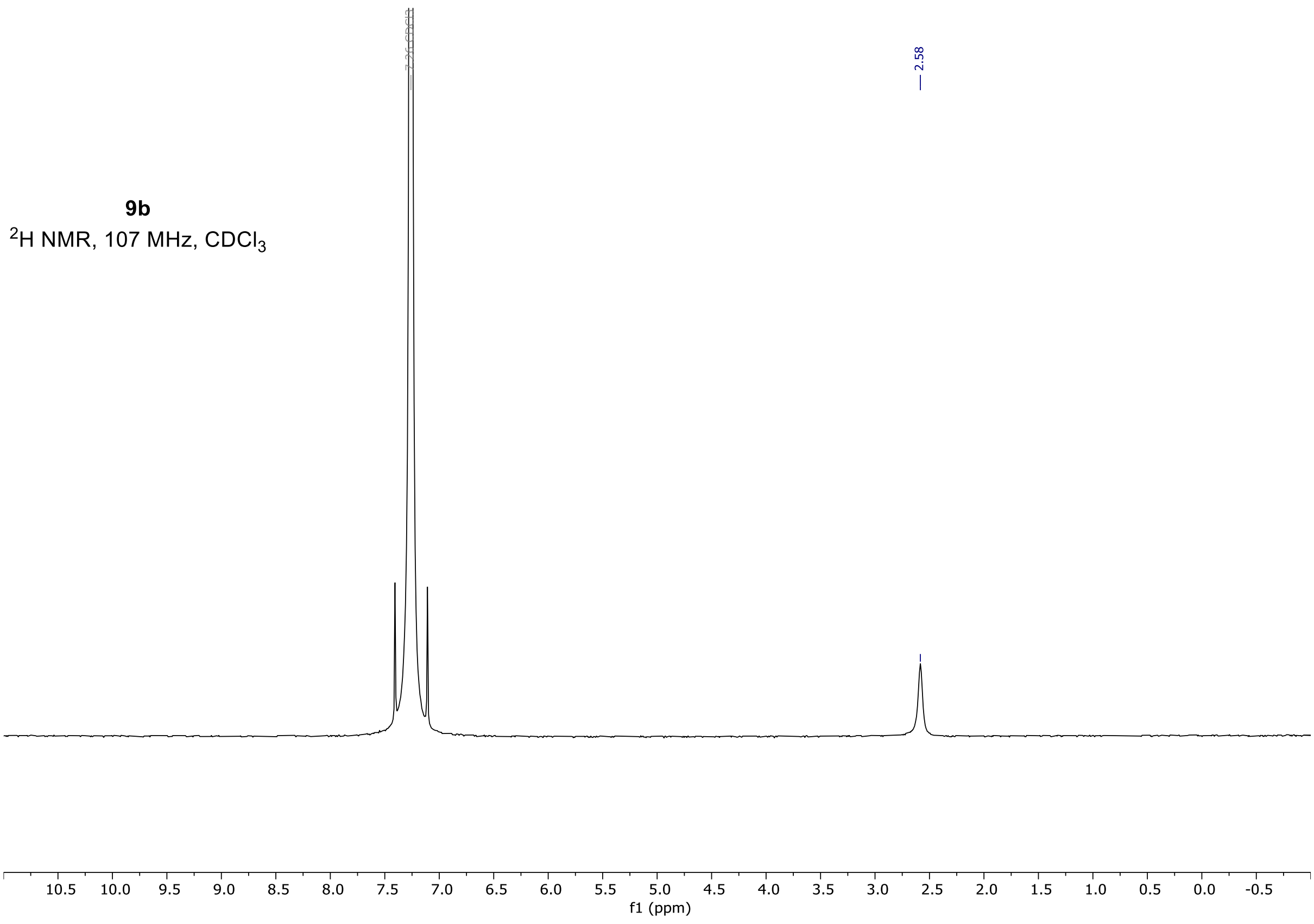

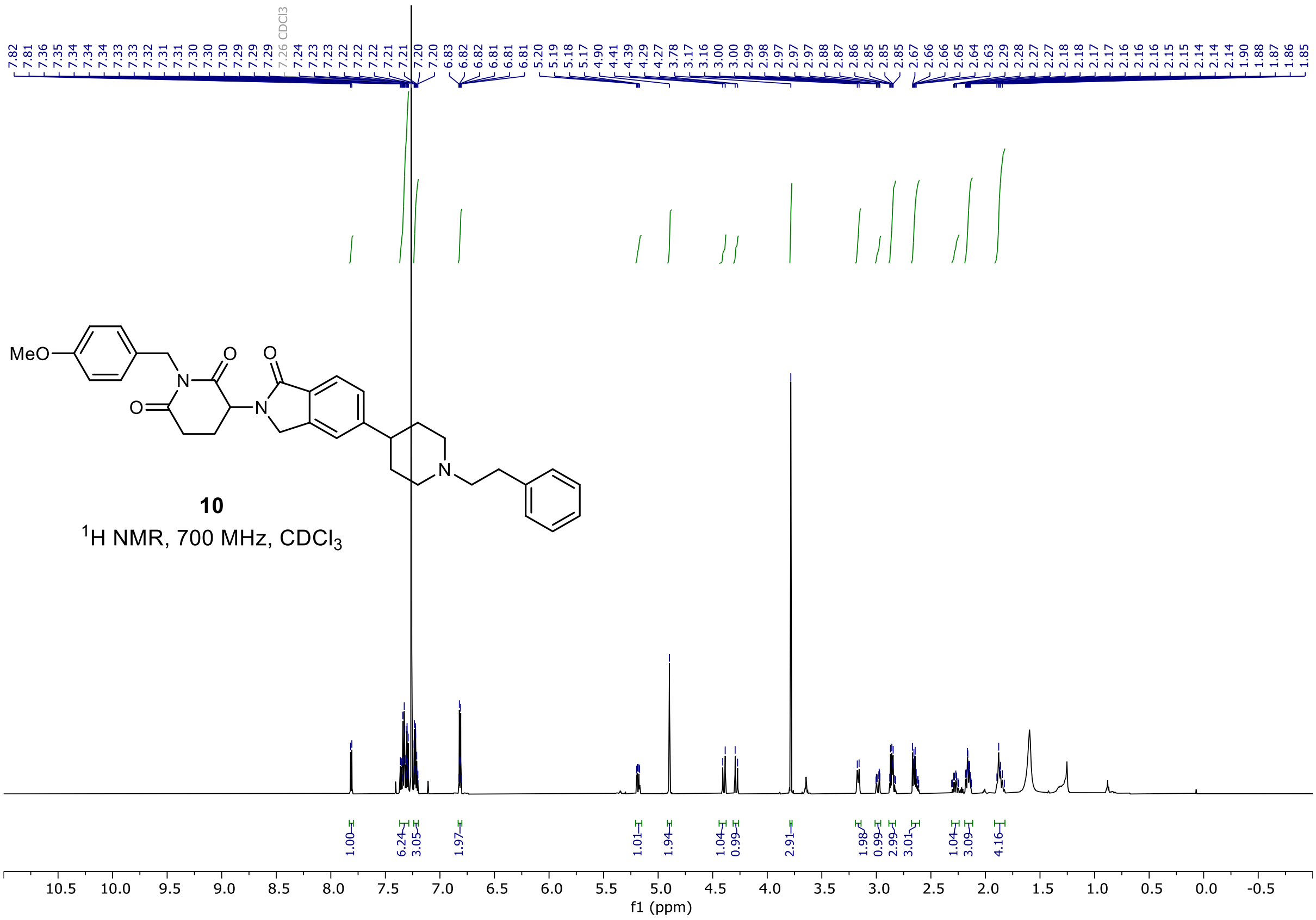

10

^{1}H NMR, 700 MHz, $CDCl_3$

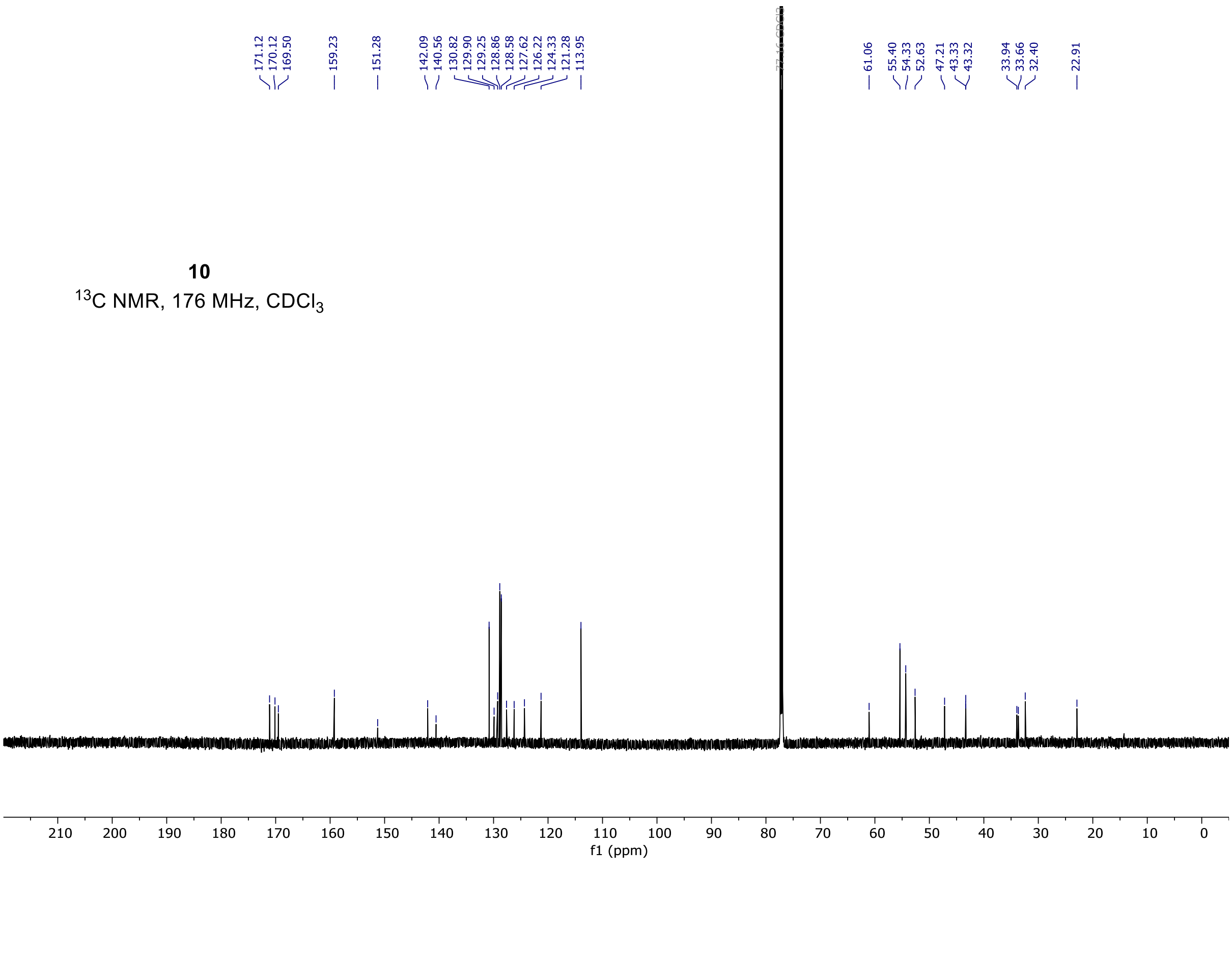

10

^{13}C NMR, 176 MHz, $CDCl_3$

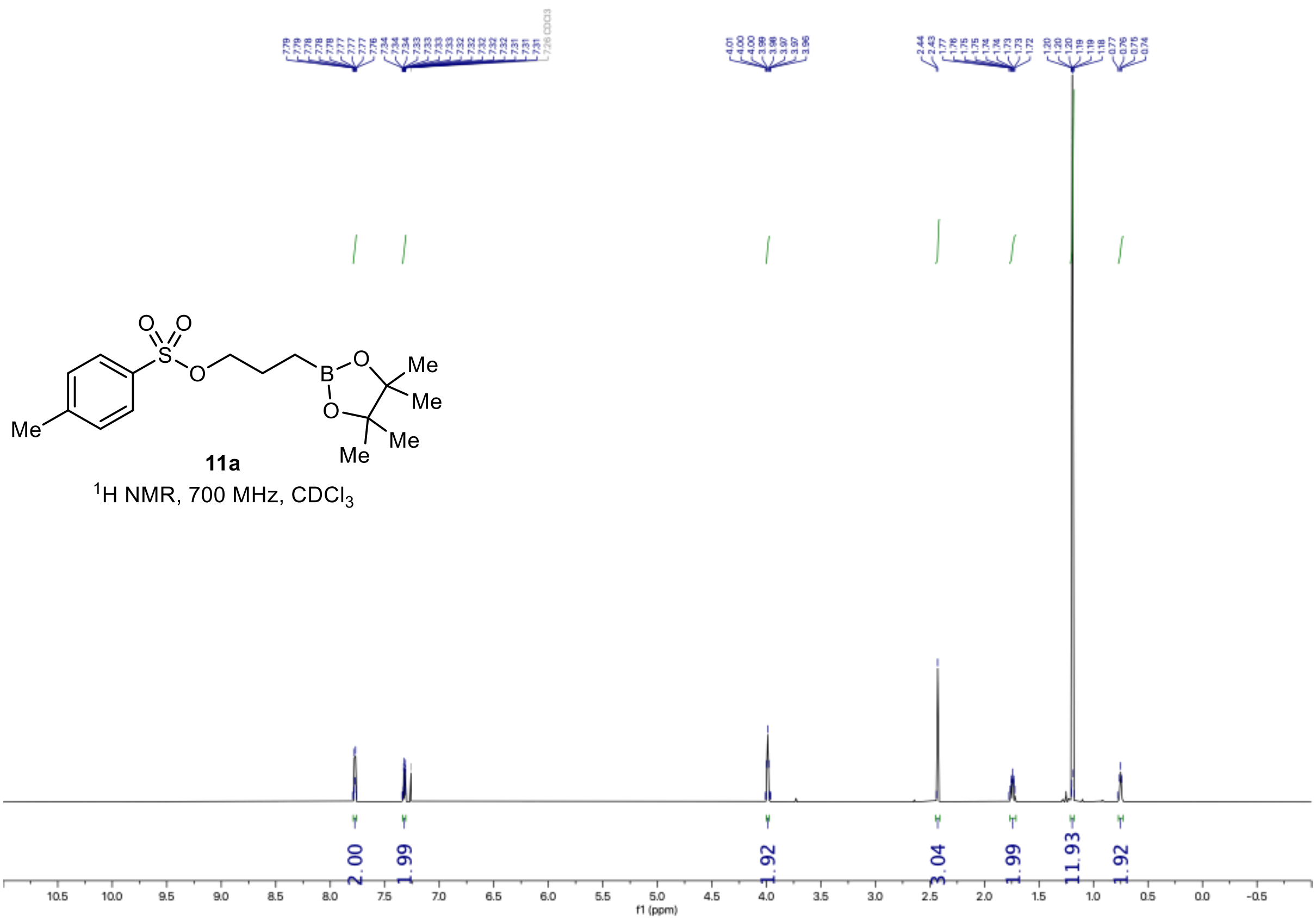

11a
^{1}H NMR, 700 MHz, $CDCl_3$

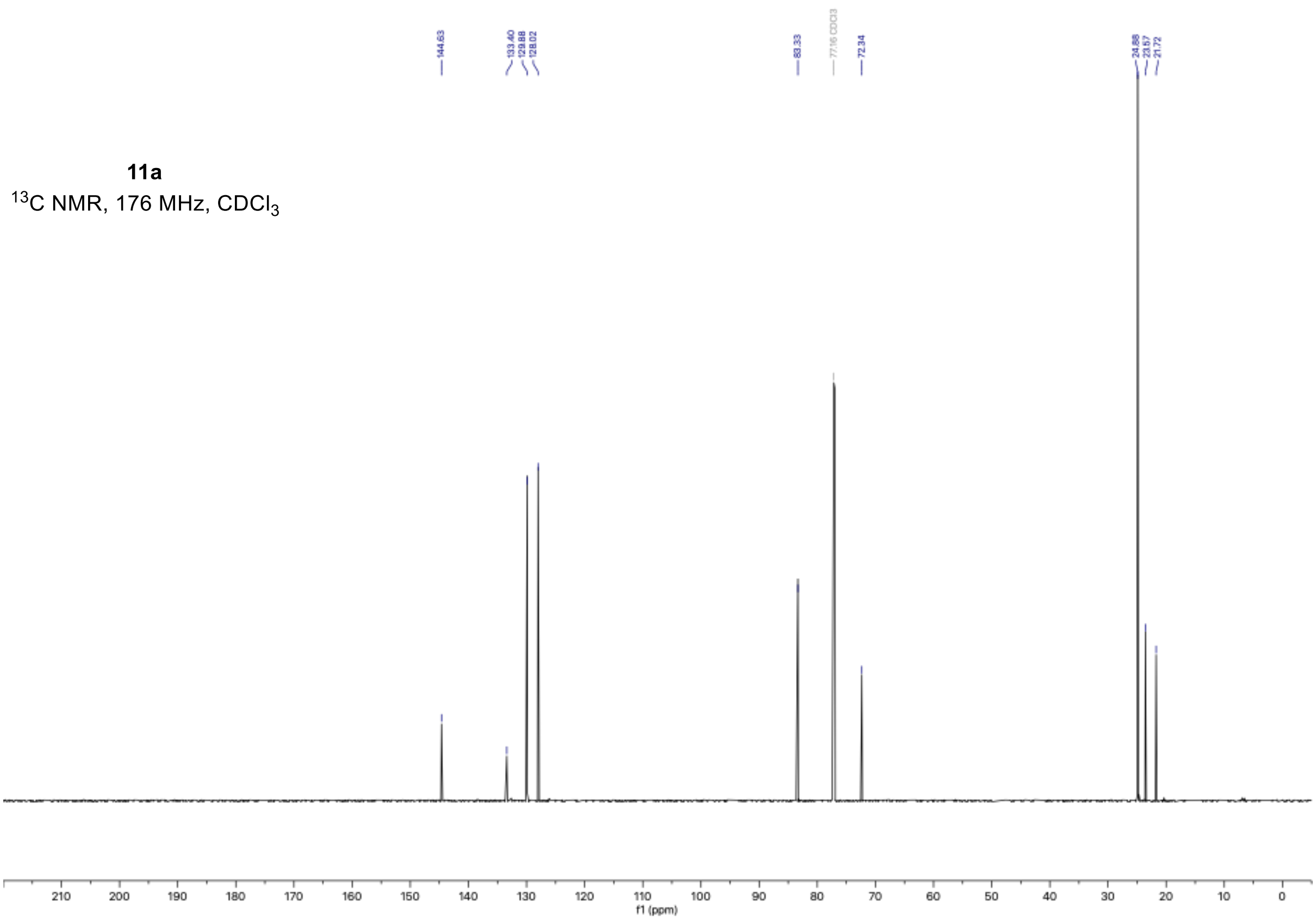

11a
^{13}C NMR, 176 MHz, $CDCl_3$

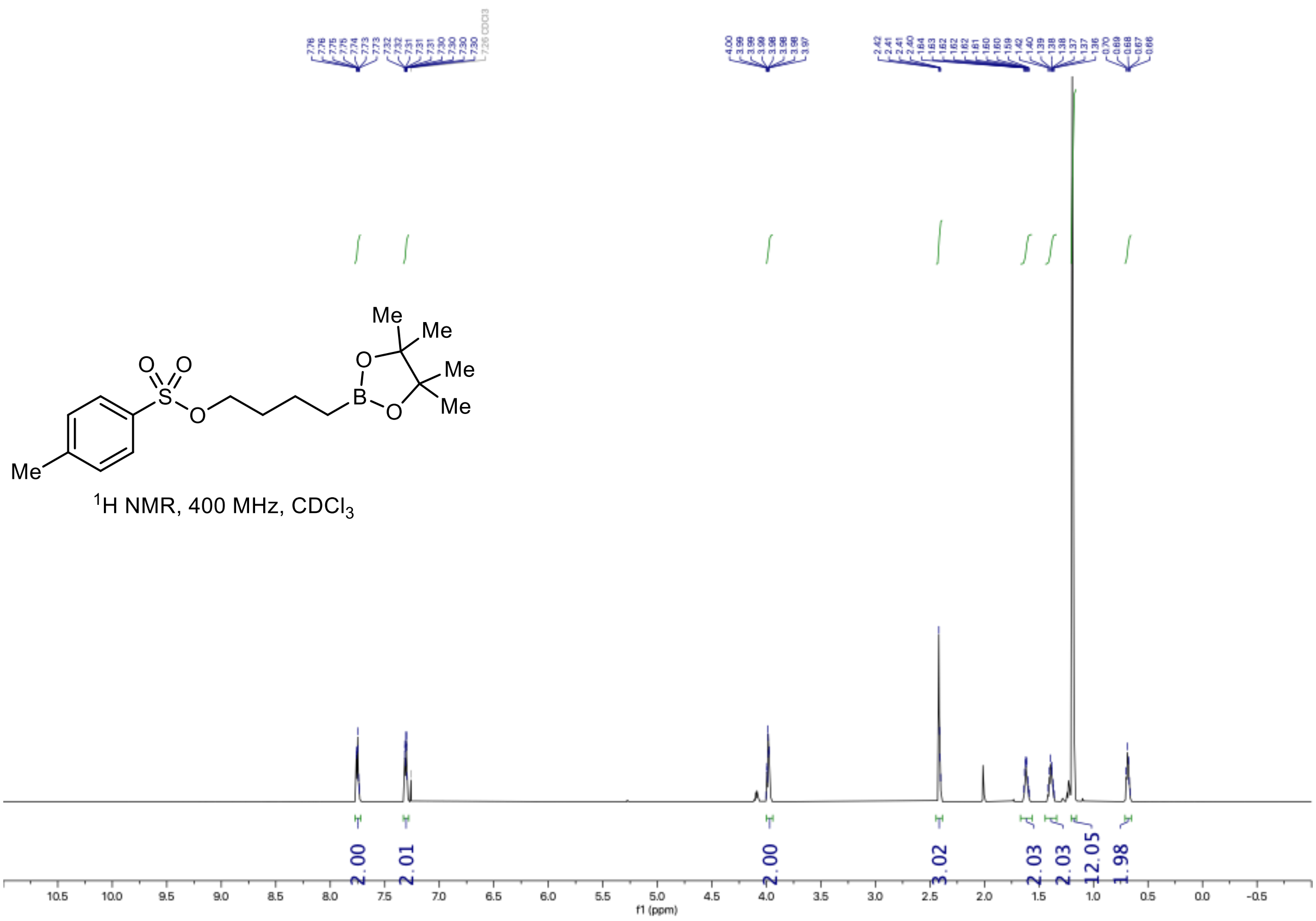
Me
Me
Me
Me
O
O
B
S
O
O
O
Me
^{1}H NMR, 400 MHz, CDCl$_3$
2.00
2.01
2.00
3.02
2.03
2.03
12.05
1.98
f1 (ppm)

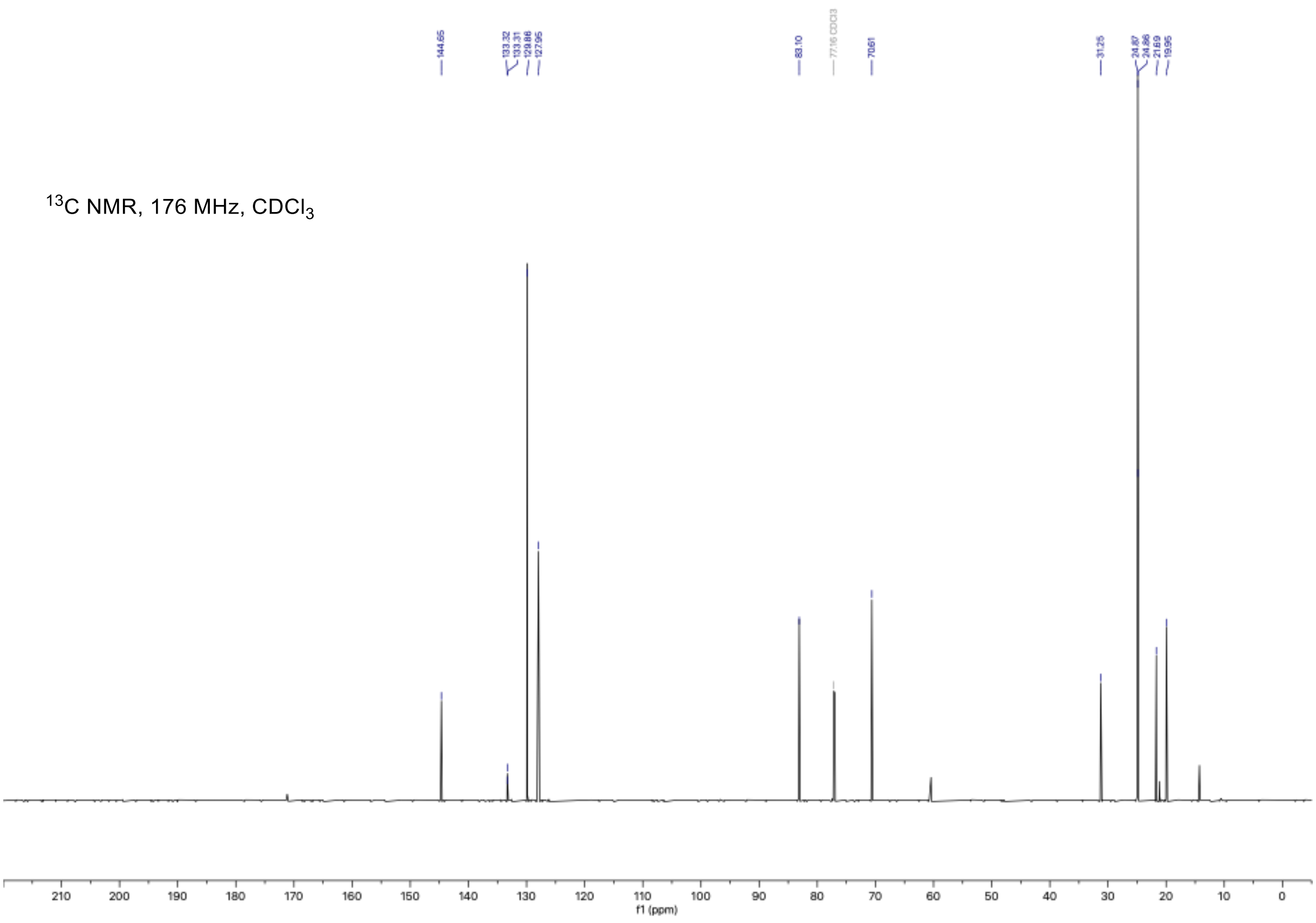
^{13}C NMR, 176 MHz, CDCl$_3$
f1 (ppm)

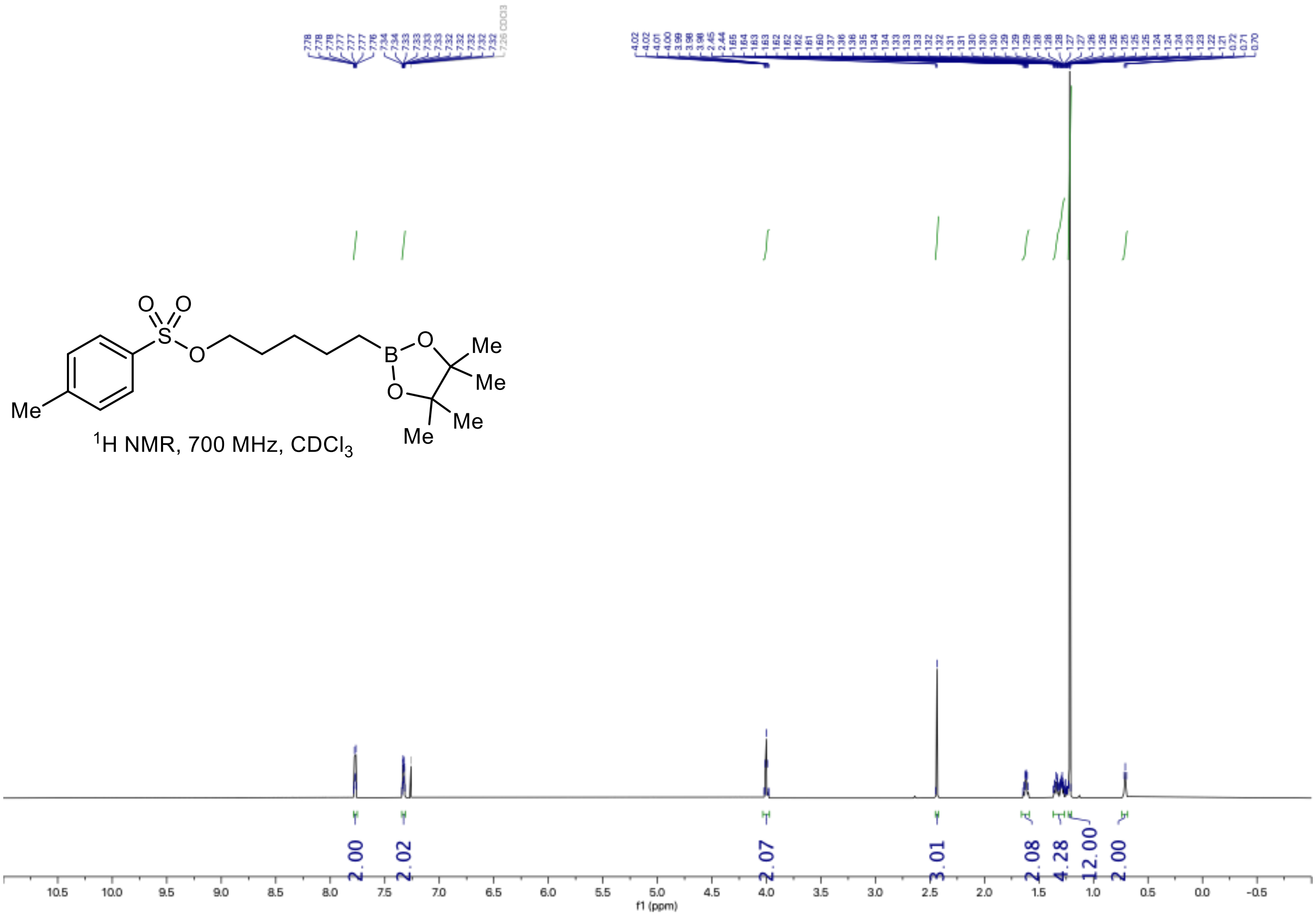

^{1}H NMR, 700 MHz, $CDCl_3$

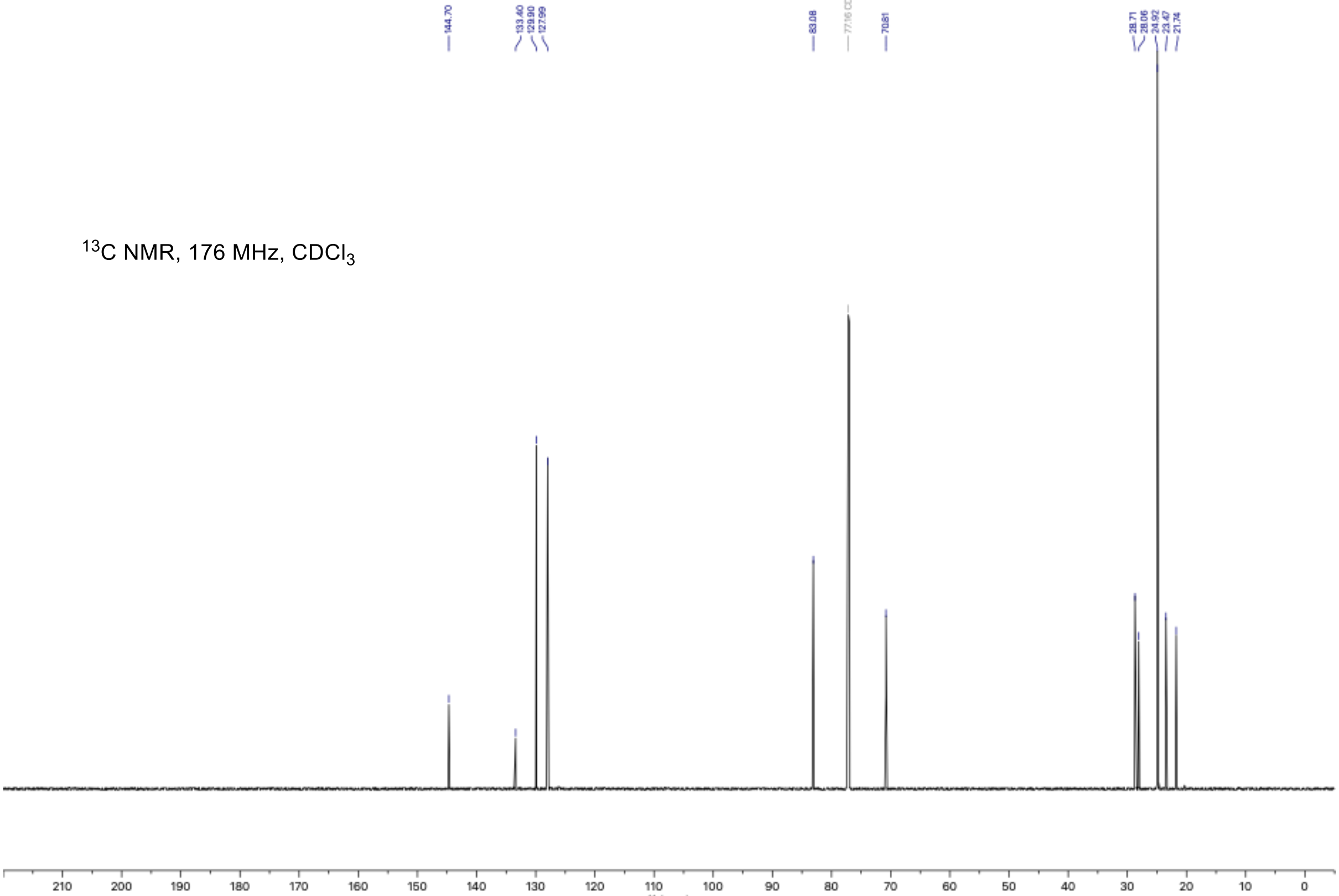

^{13}C NMR, 176 MHz, $CDCl_3$

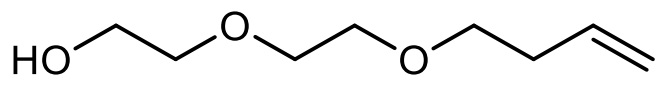

^{1}H NMR, 500 MHz, $CDCl_3$

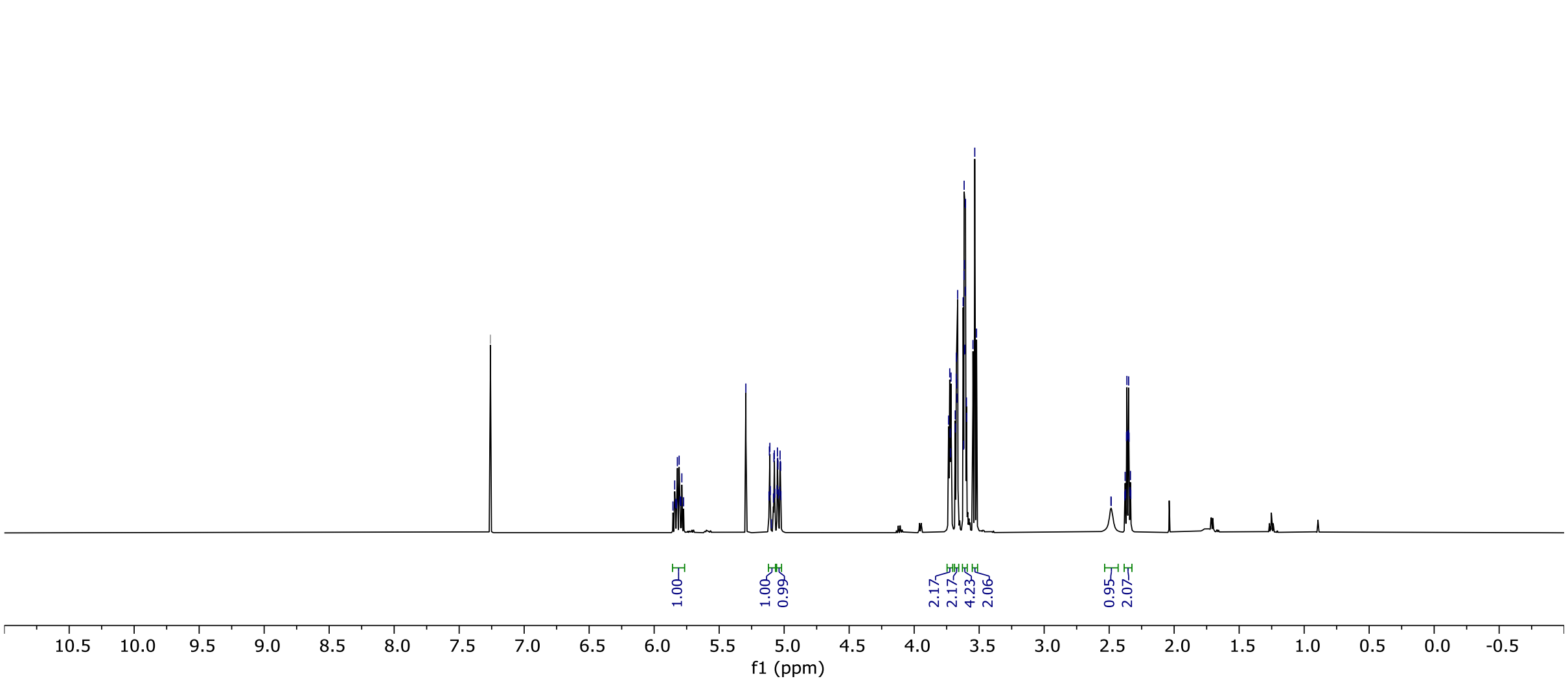

^{13}C NMR, 176 MHz, $CDCl_3$

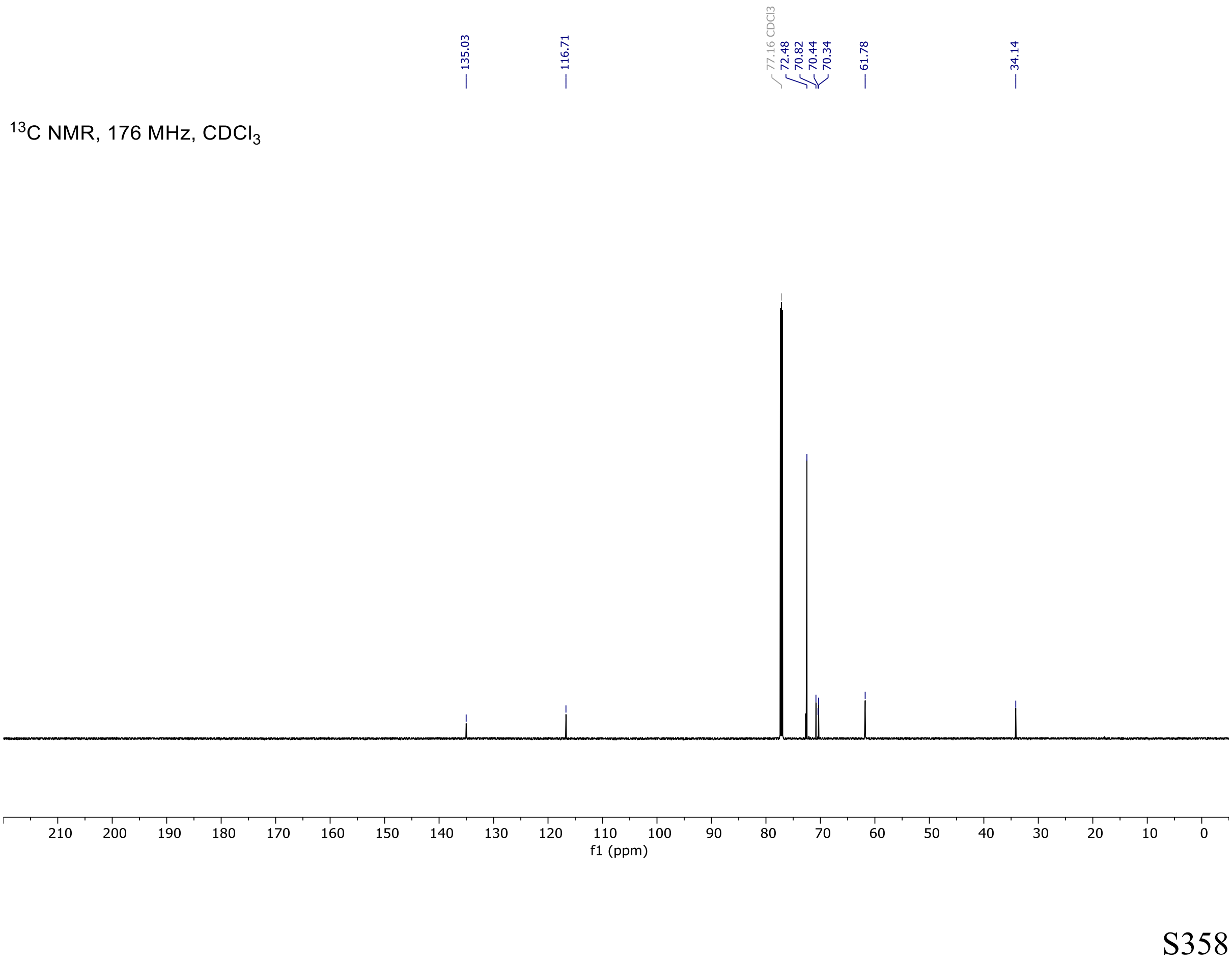

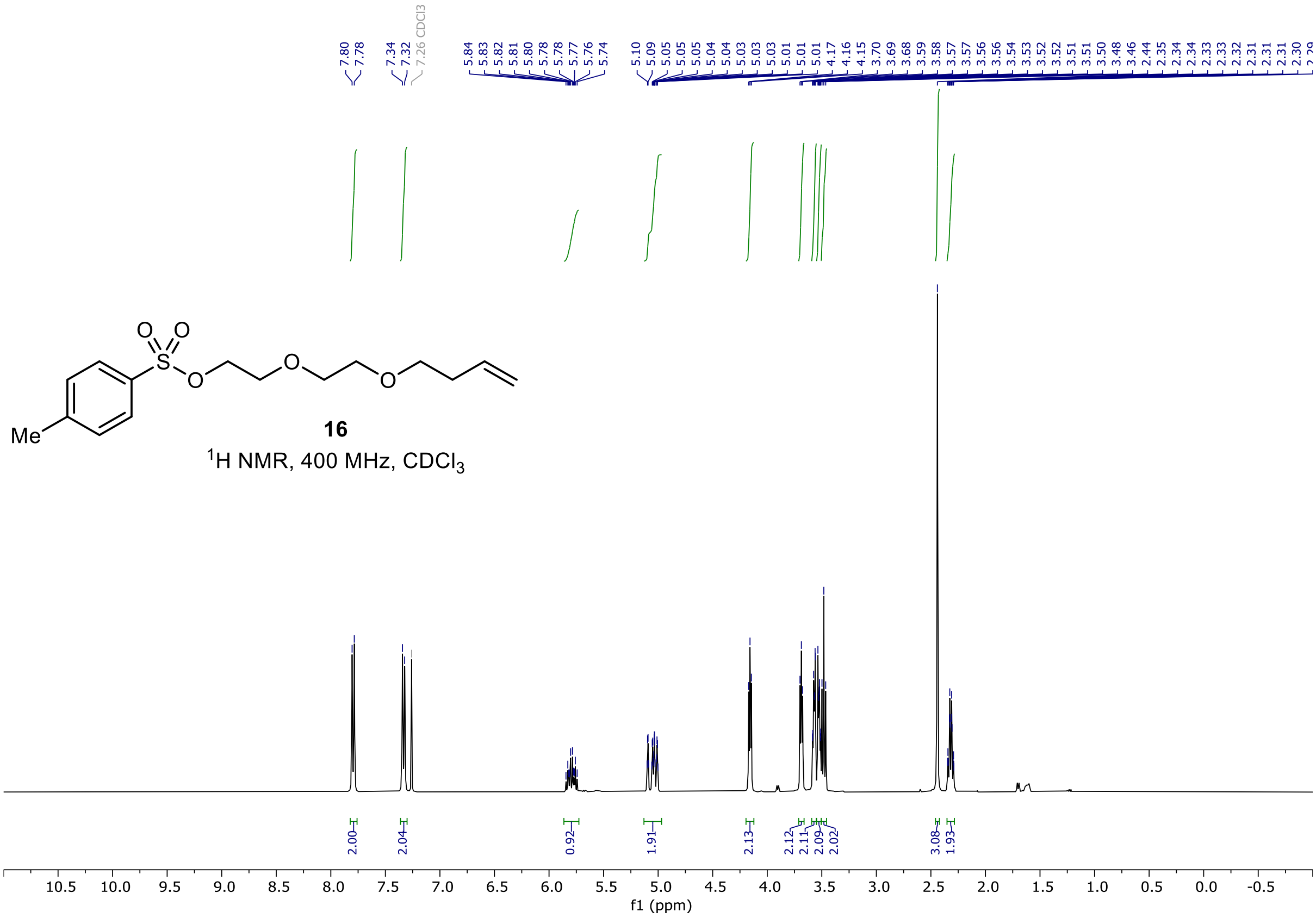

16
^{1}H NMR, 400 MHz, $CDCl_3$

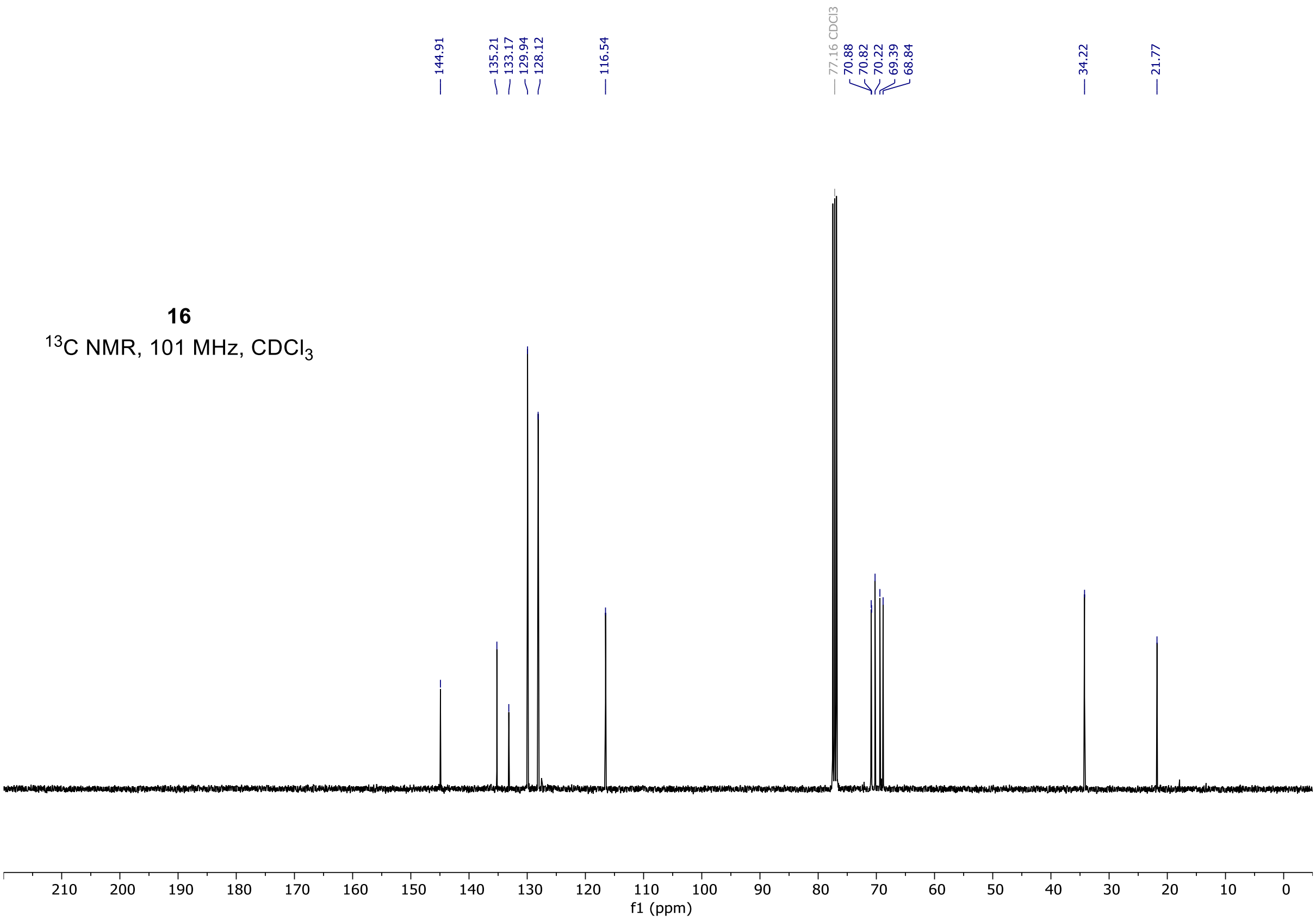

16
^{13}C NMR, 101 MHz, $CDCl_3$

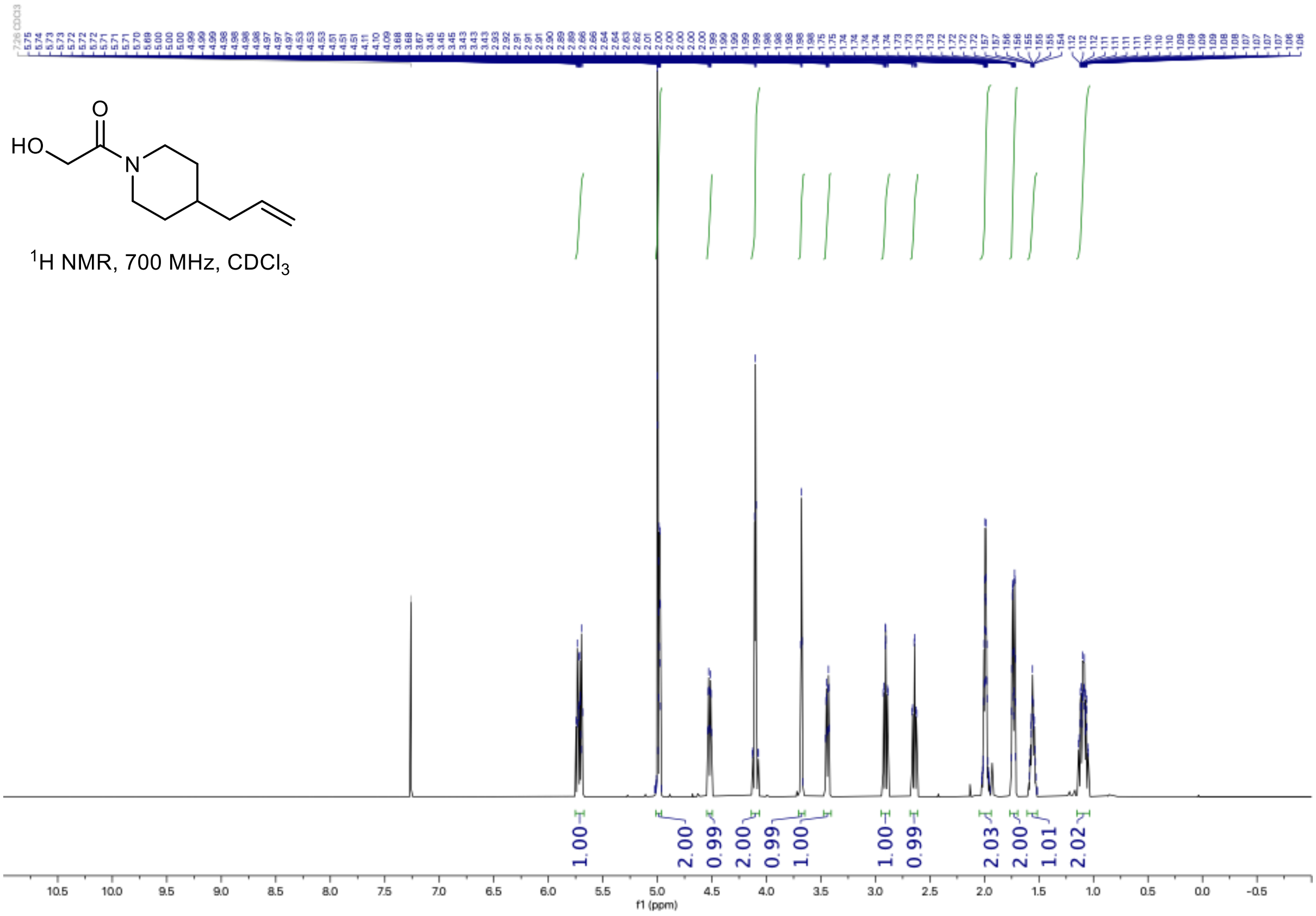

^{1}H NMR, 700 MHz, $CDCl_3$

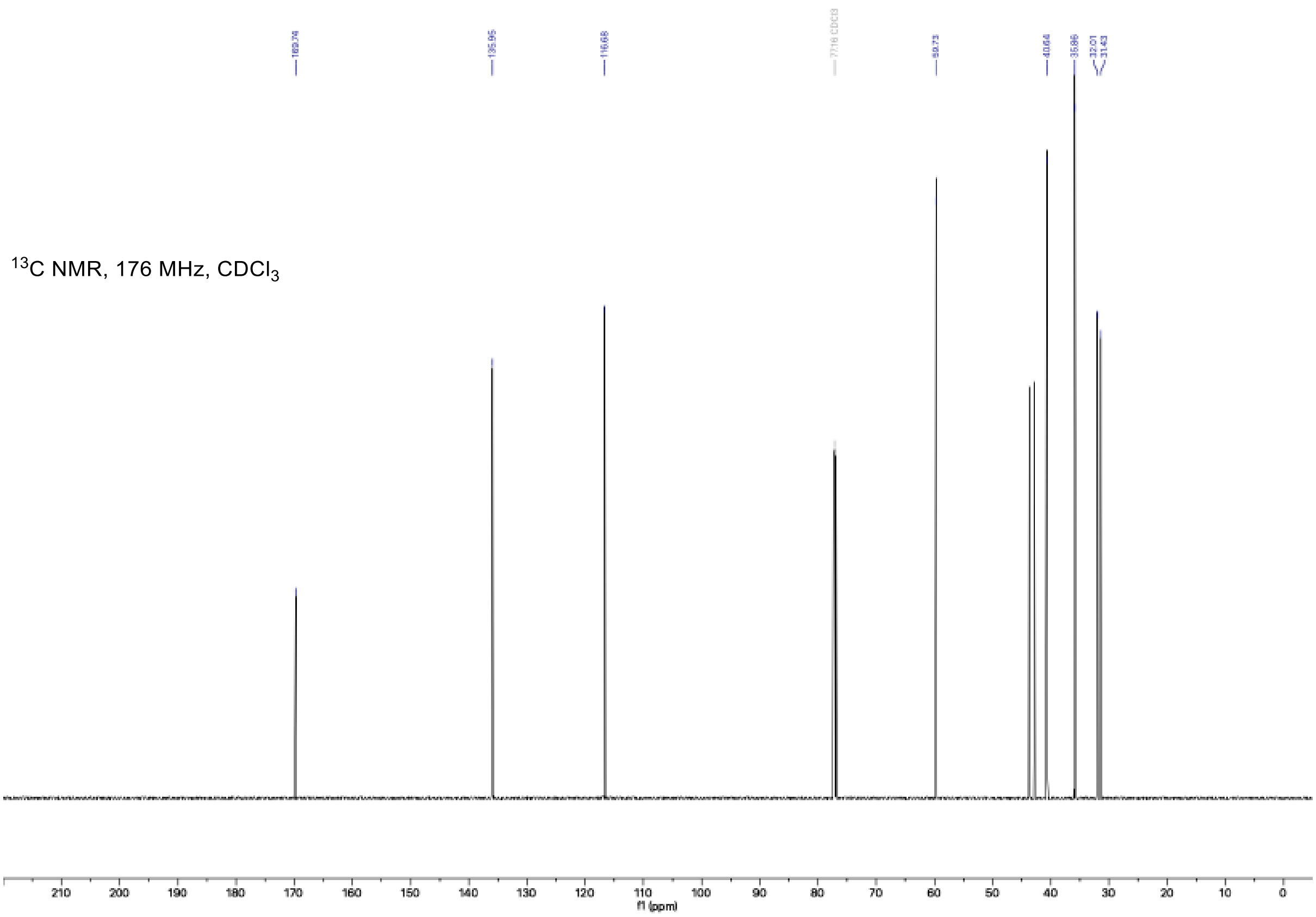

^{13}C NMR, 176 MHz, $CDCl_3$

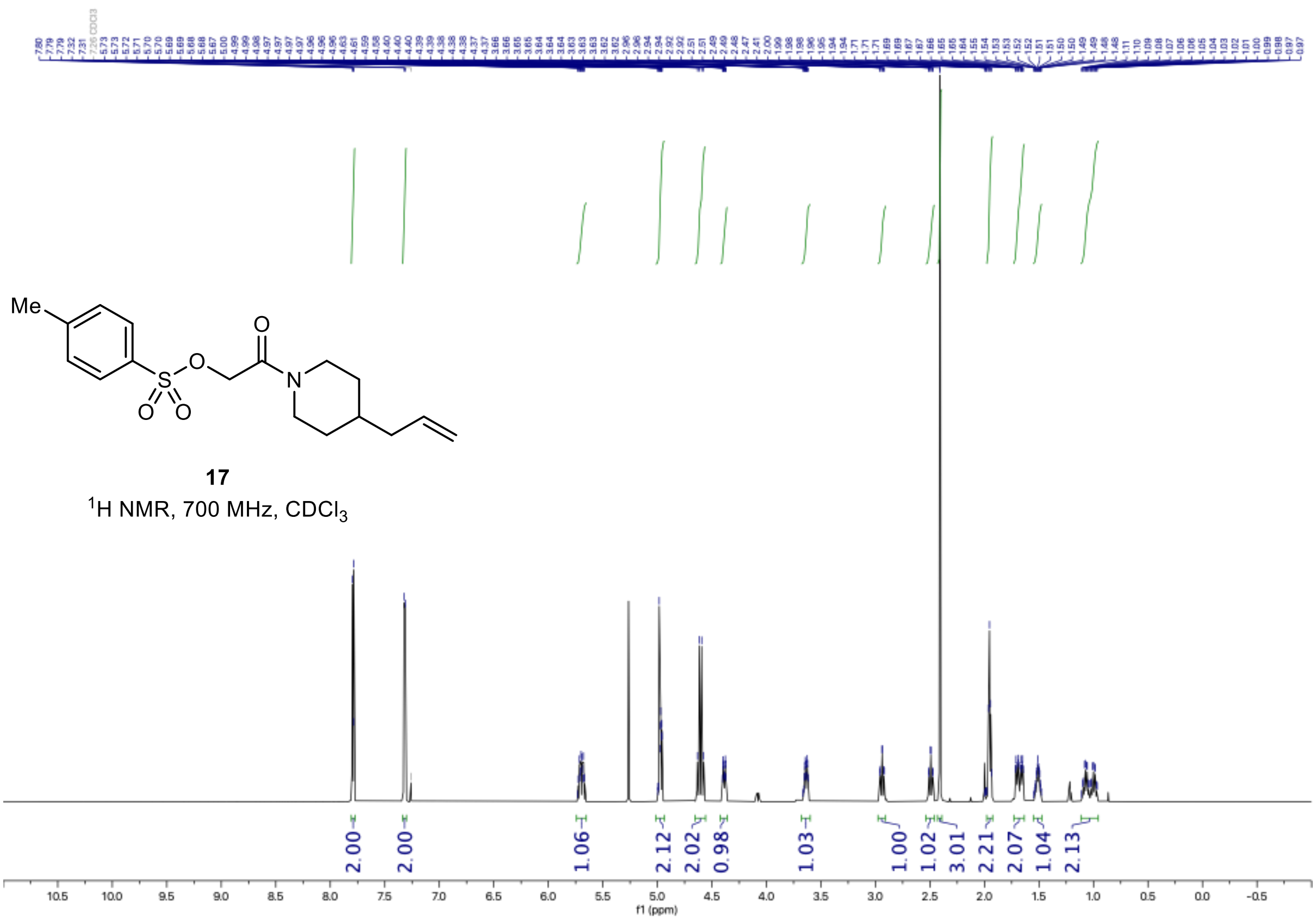

17

^1H NMR, 700 MHz, $CDCl_3$

17

^{13}C NMR, 176 MHz, $CDCl_3$

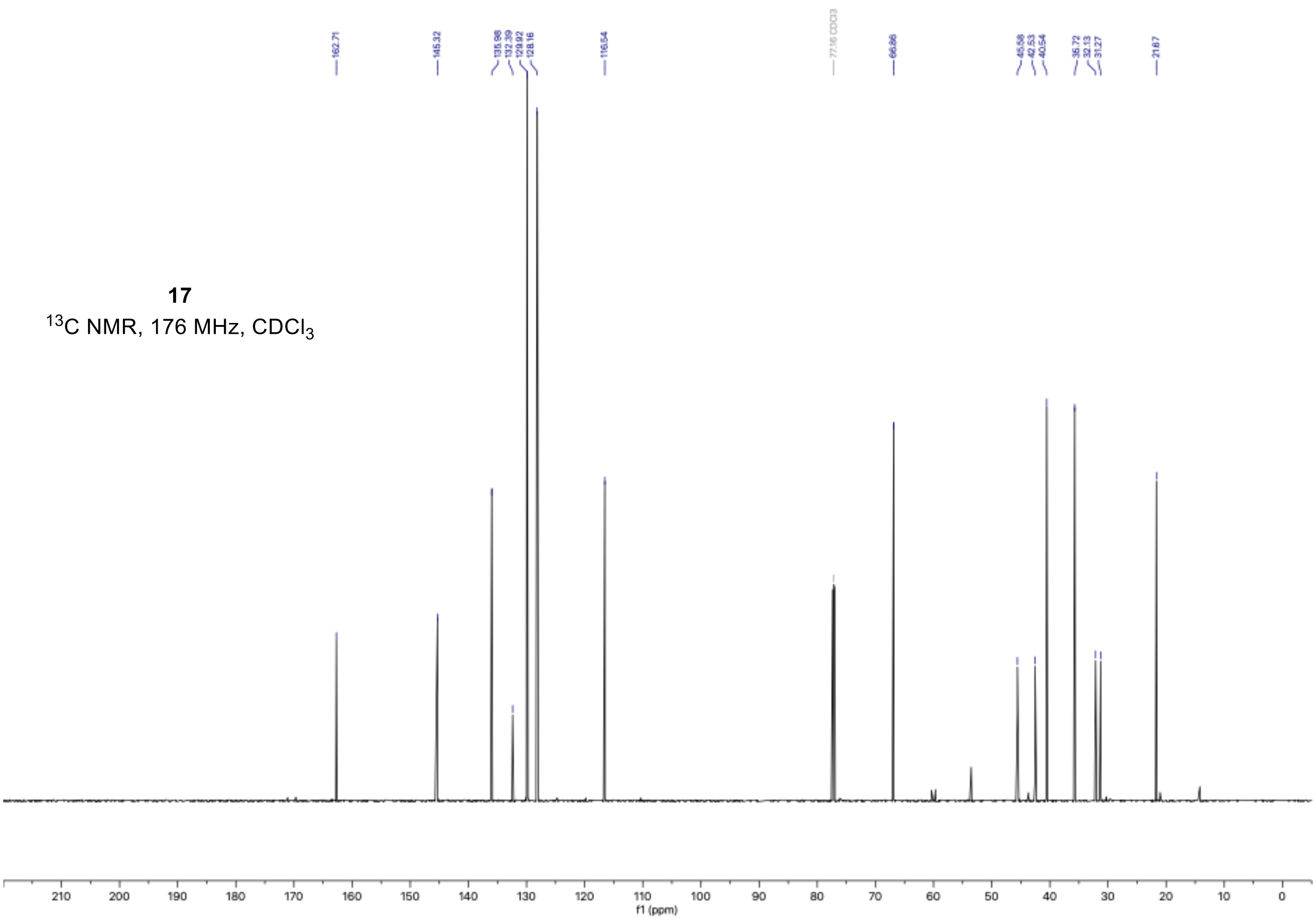

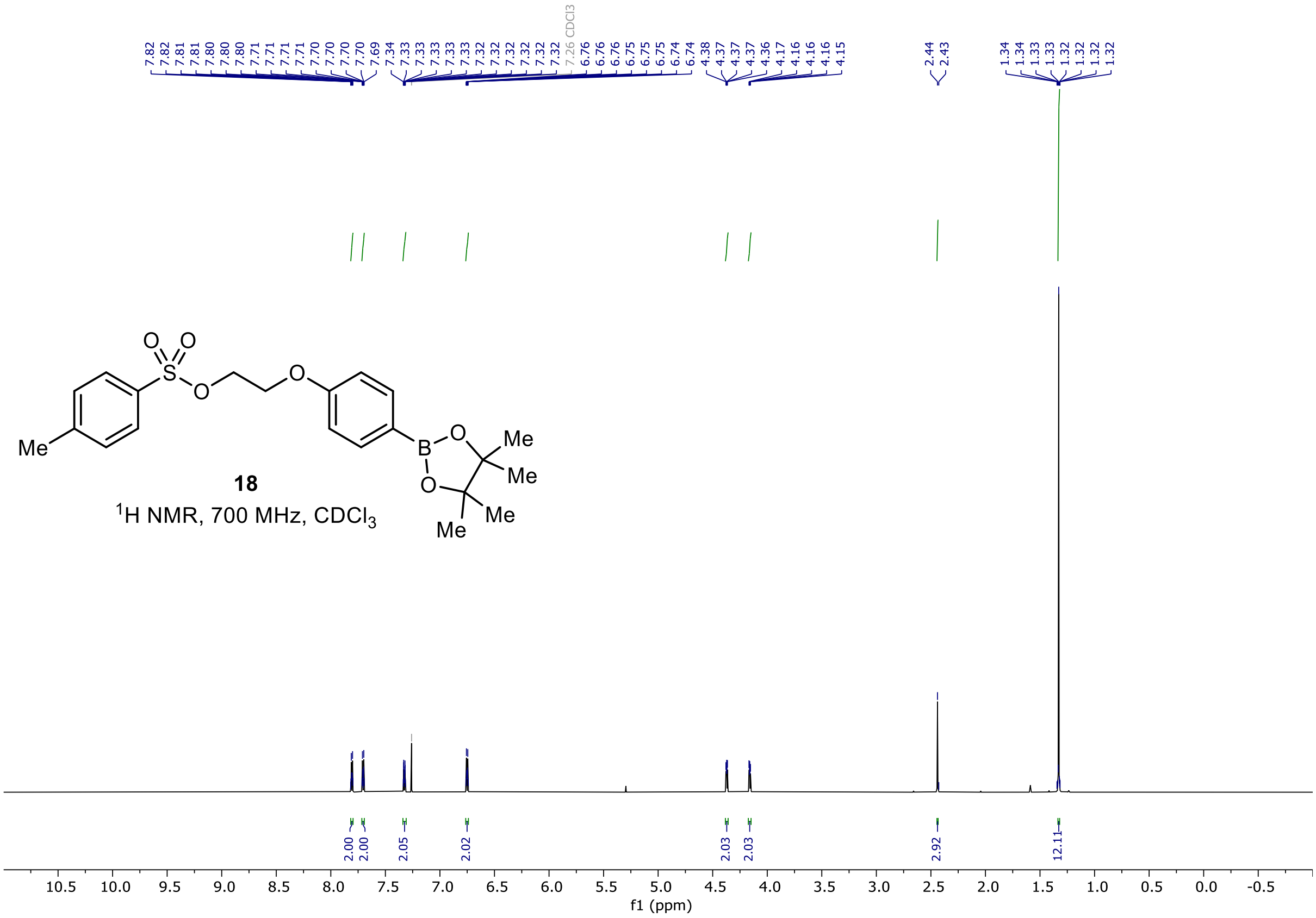

18
^{1}H NMR, 700 MHz, $CDCl_3$

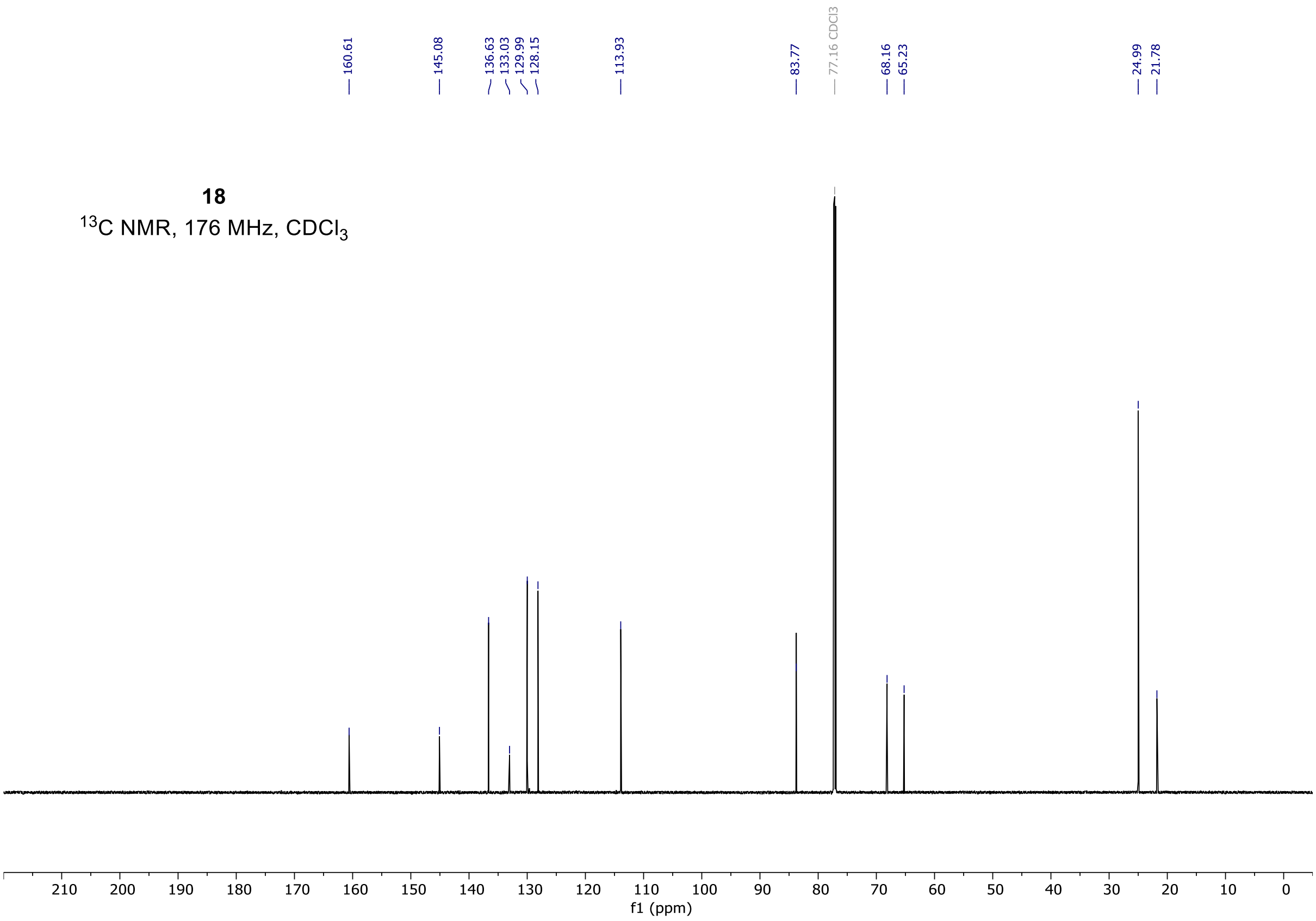

18
^{13}C NMR, 176 MHz, $CDCl_3$

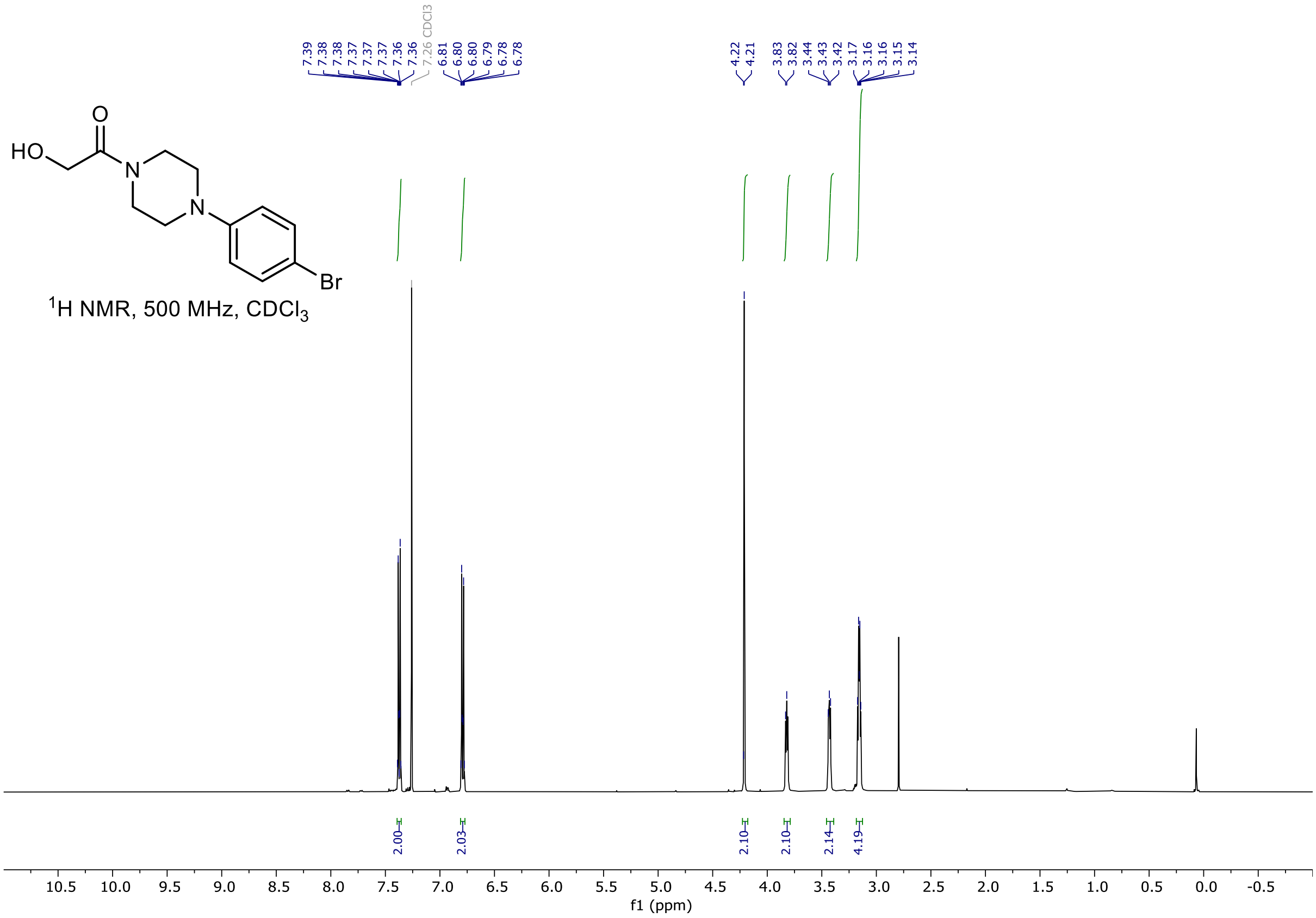

^{1}H NMR, 500 MHz, $CDCl_3$
f1 (ppm)

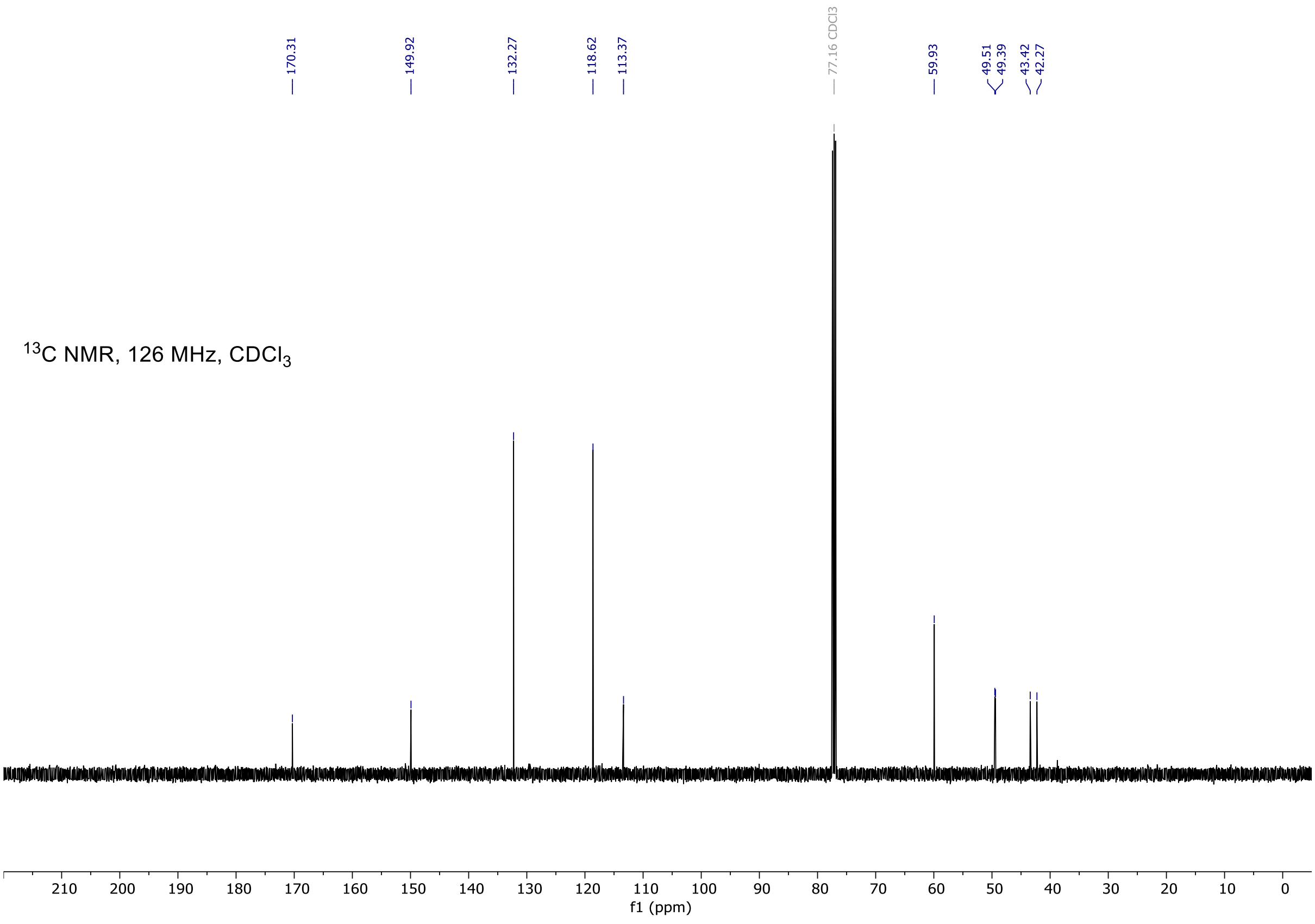

^{13}C NMR, 126 MHz, $CDCl_3$
f1 (ppm)

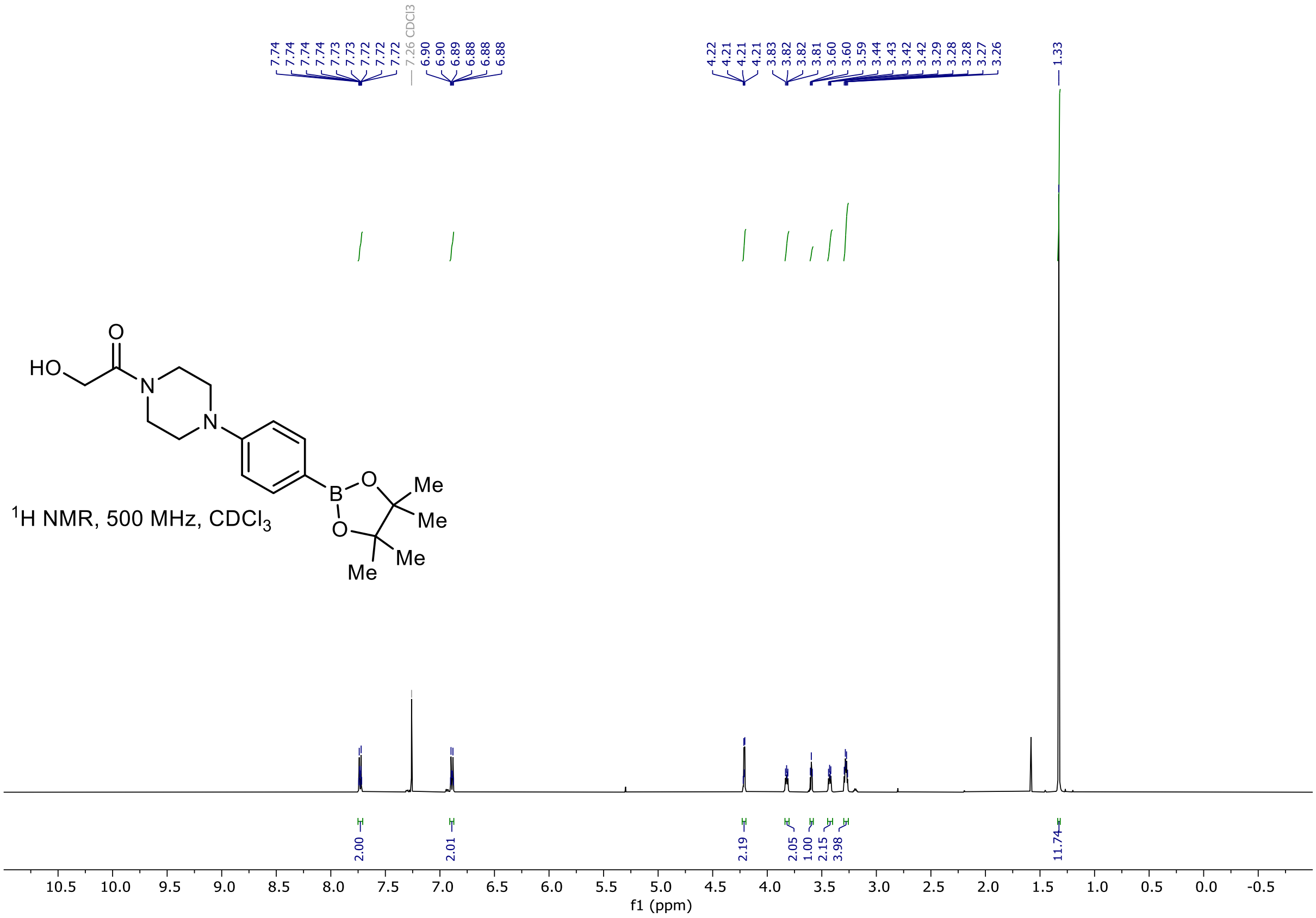

HO
O
N
N
B
O
O
Me
Me
Me
Me
^{1}H NMR, 500 MHz, $CDCl_3$
7.74 7.74 7.74 7.74 7.73 7.73 7.72 7.72 7.72
7.26 CDCl3
6.90 6.90 6.89 6.88 6.88 6.88
4.22 4.21 4.21 4.21 3.83 3.82 3.82 3.81 3.60 3.60 3.59 3.44 3.43 3.42 3.42 3.29 3.28 3.28 3.27 3.26
1.33
2.00
2.01
2.19
2.05
1.00
2.15
3.98
11.74
10.5 10.0 9.5 9.0 8.5 8.0 7.5 7.0 6.5 6.0 5.5 5.0 4.5 4.0 3.5 3.0 2.5 2.0 1.5 1.0 0.5 0.0 -0.5
f1 (ppm)

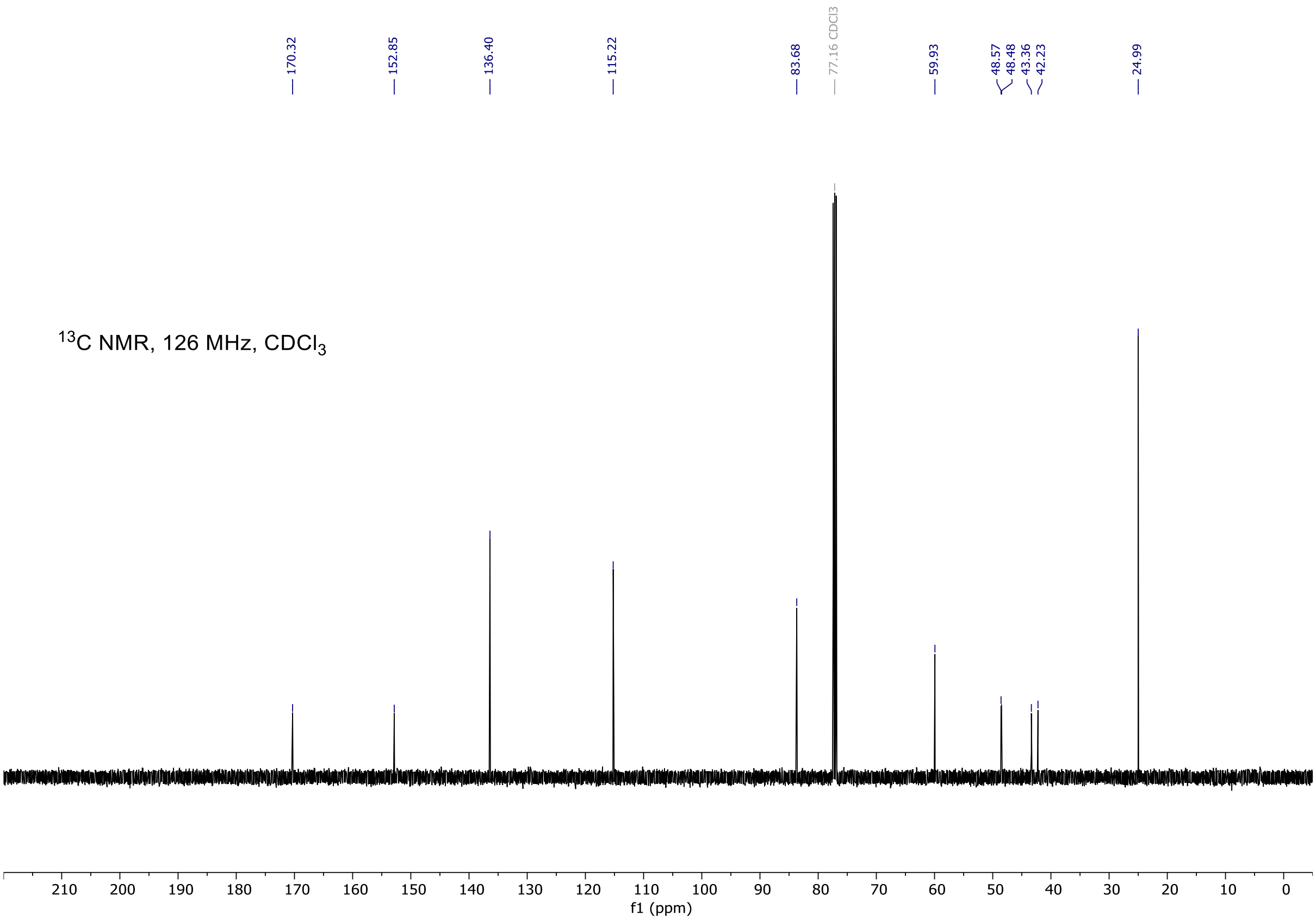

^{13}C NMR, 126 MHz, $CDCl_3$
170.32
152.85
136.40
115.22
83.68
77.16 CDCl3
59.93
48.57 48.48 43.36 42.23
24.99
210 200 190 180 170 160 150 140 130 120 110 100 90 80 70 60 50 40 30 20 10 0
f1 (ppm)

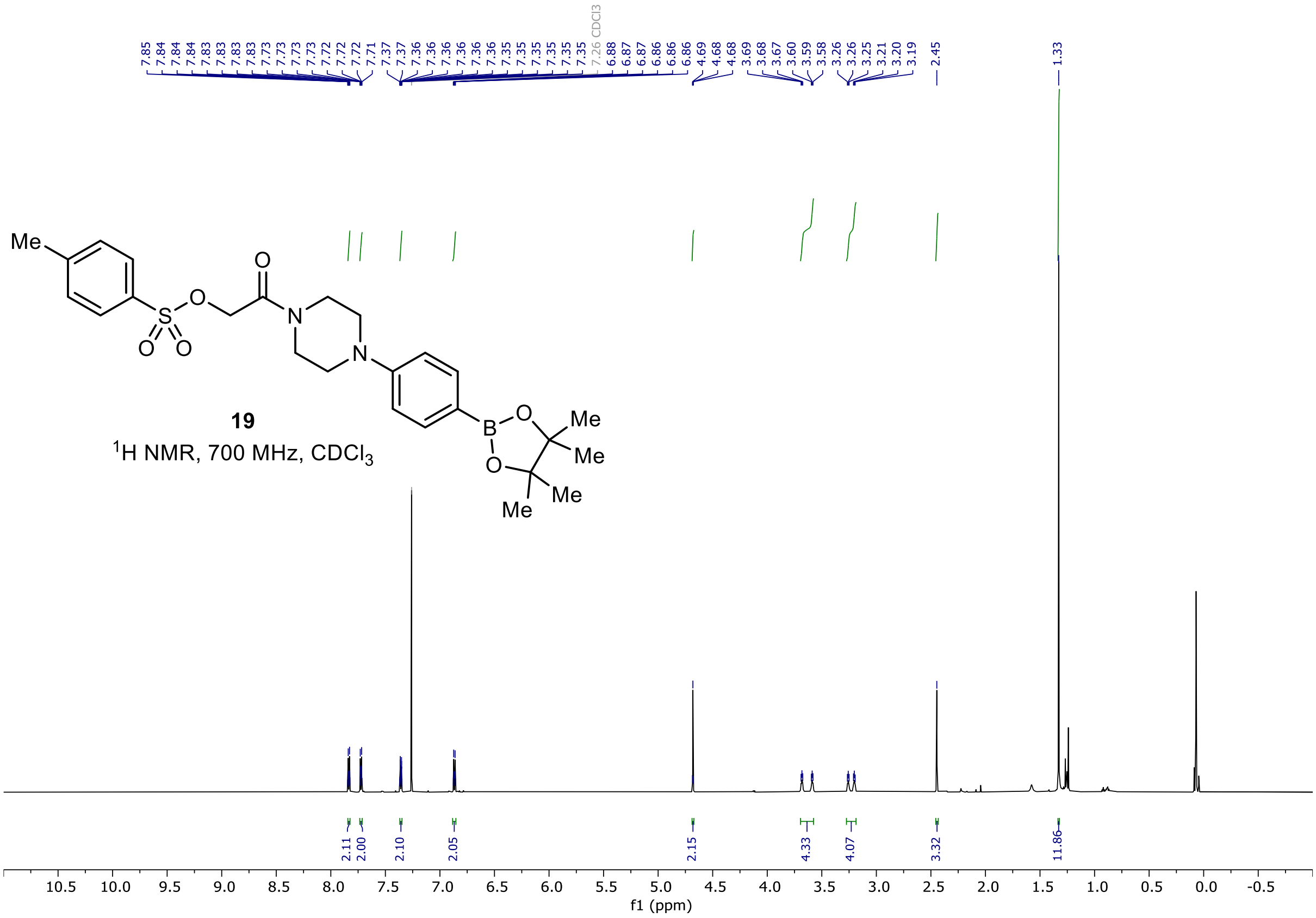

19

^{1}H NMR, 700 MHz, $CDCl_3$

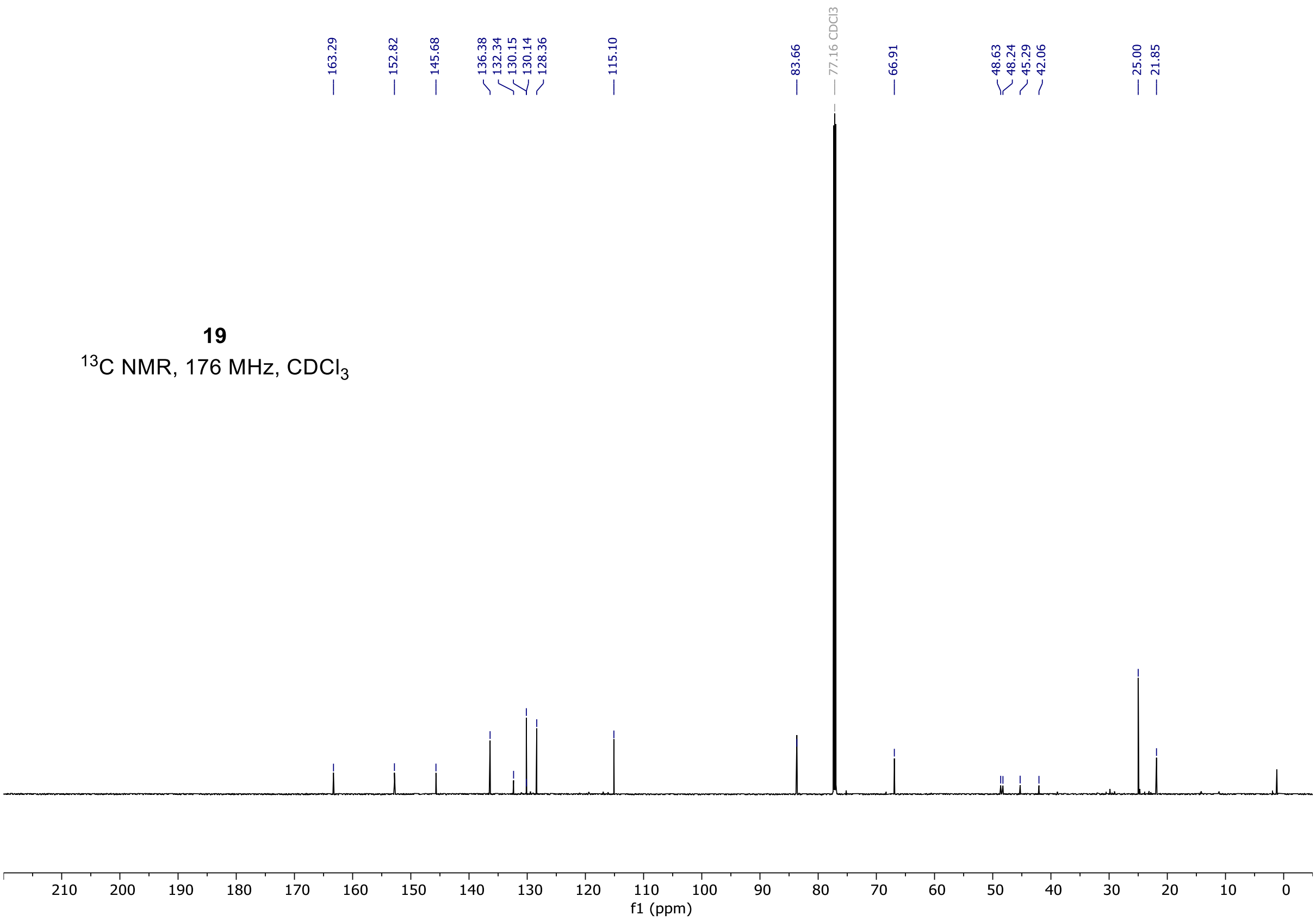

19

^{13}C NMR, 176 MHz, $CDCl_3$

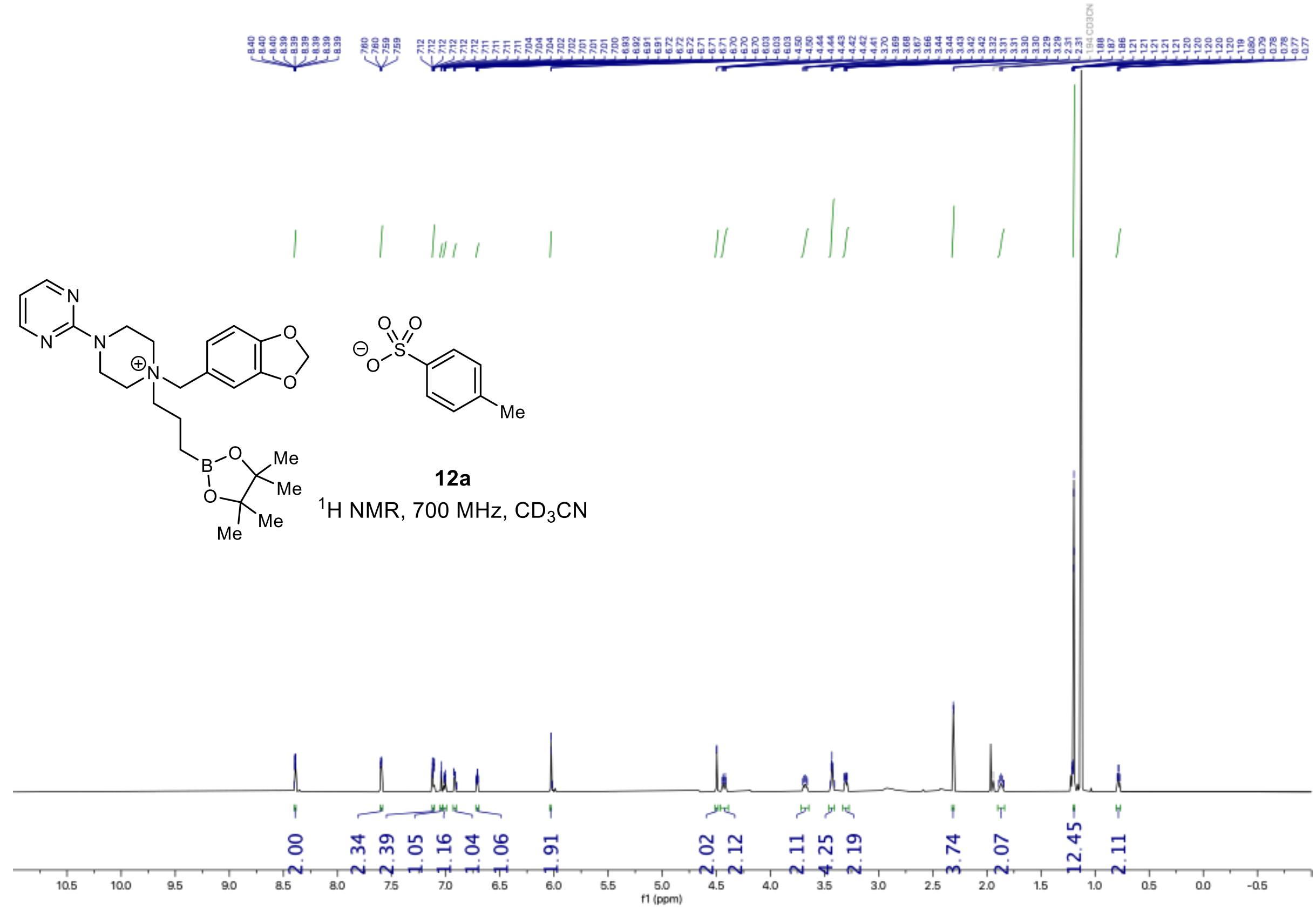

12a

^{1}H NMR, 700 MHz, CD_3CN

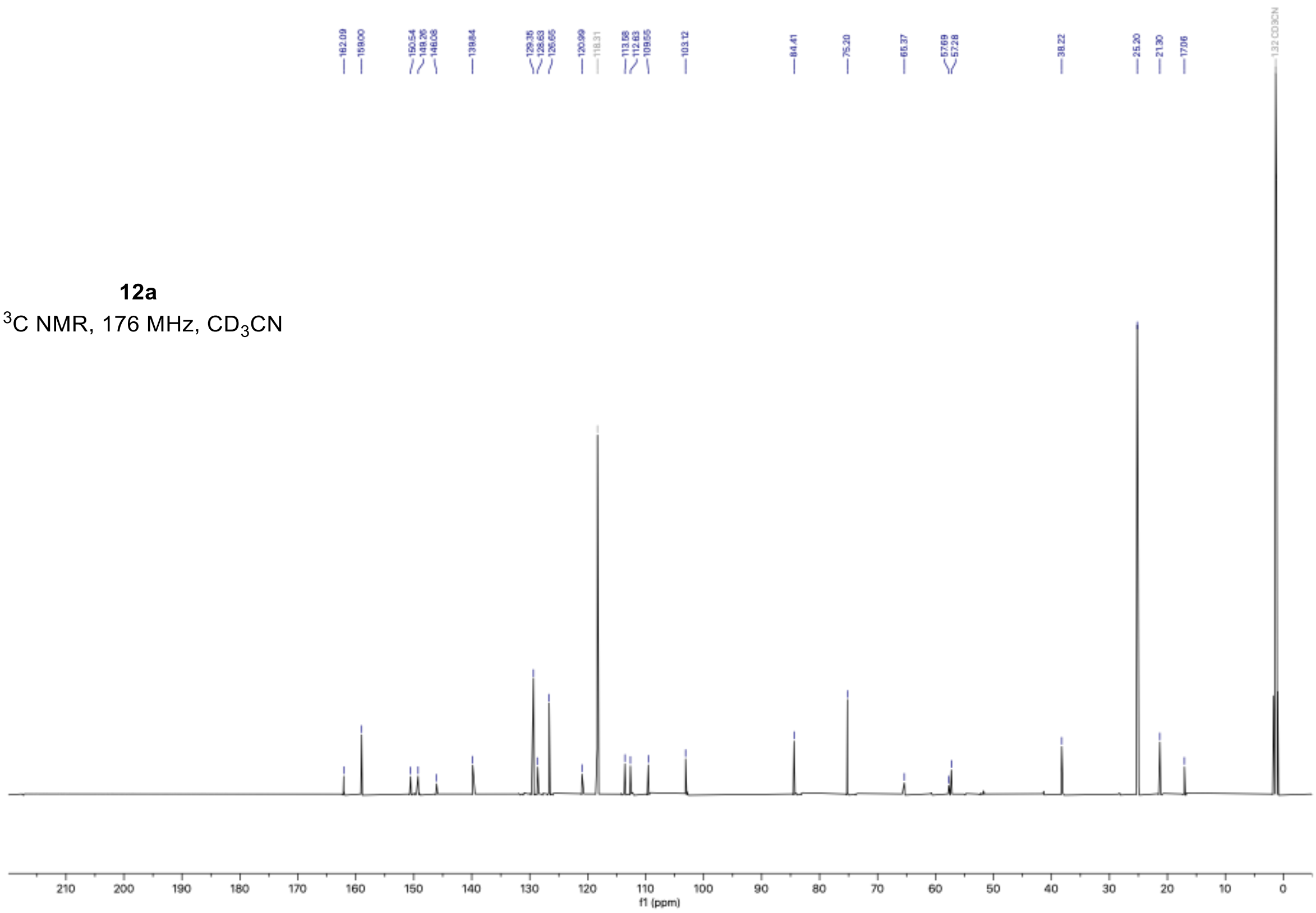

12a

^{13}C NMR, 176 MHz, CD_3CN

^{1}H NMR, 700 MHz, CD_3CN

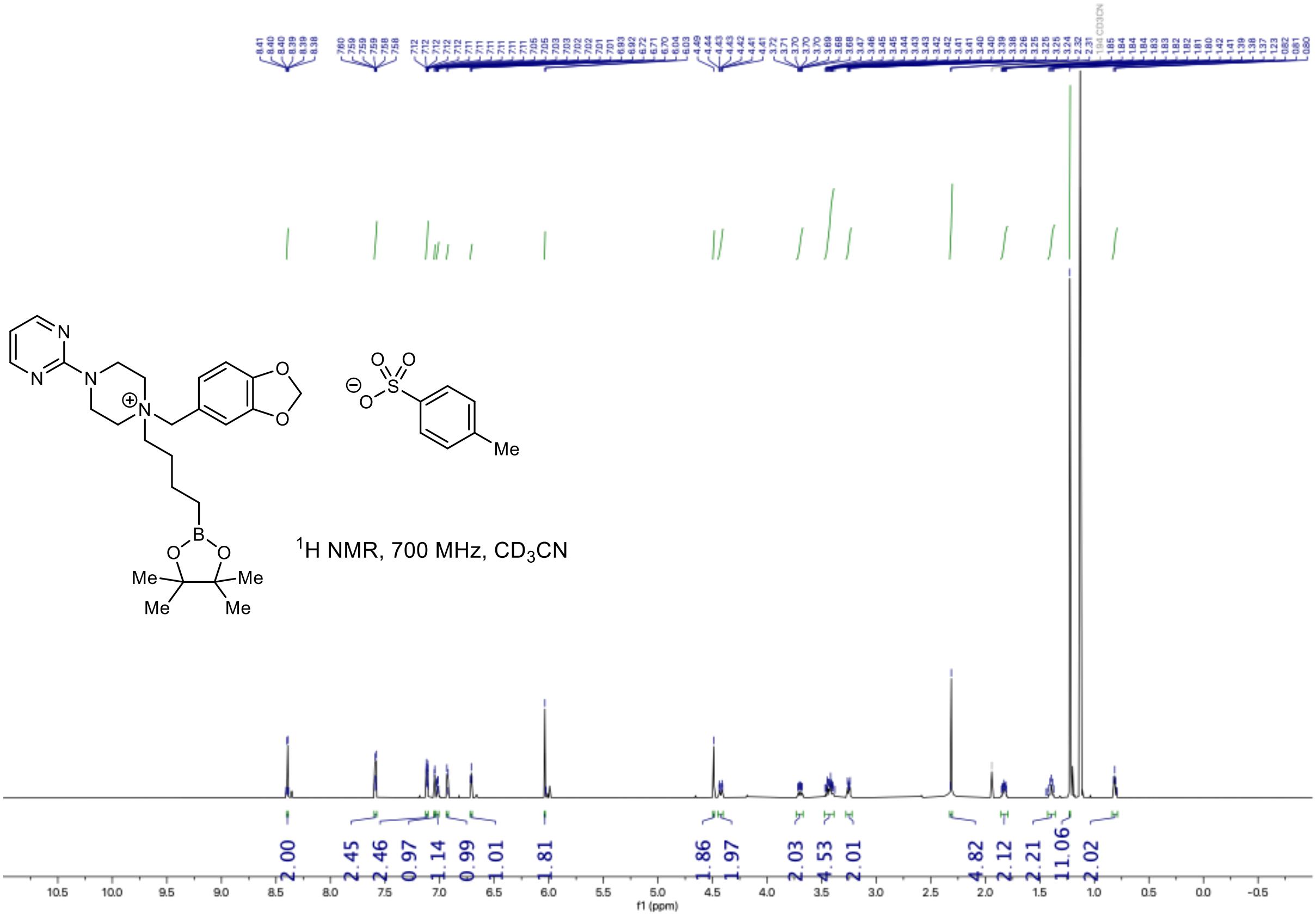

^{13}C NMR, 176 MHz, CD_3CN

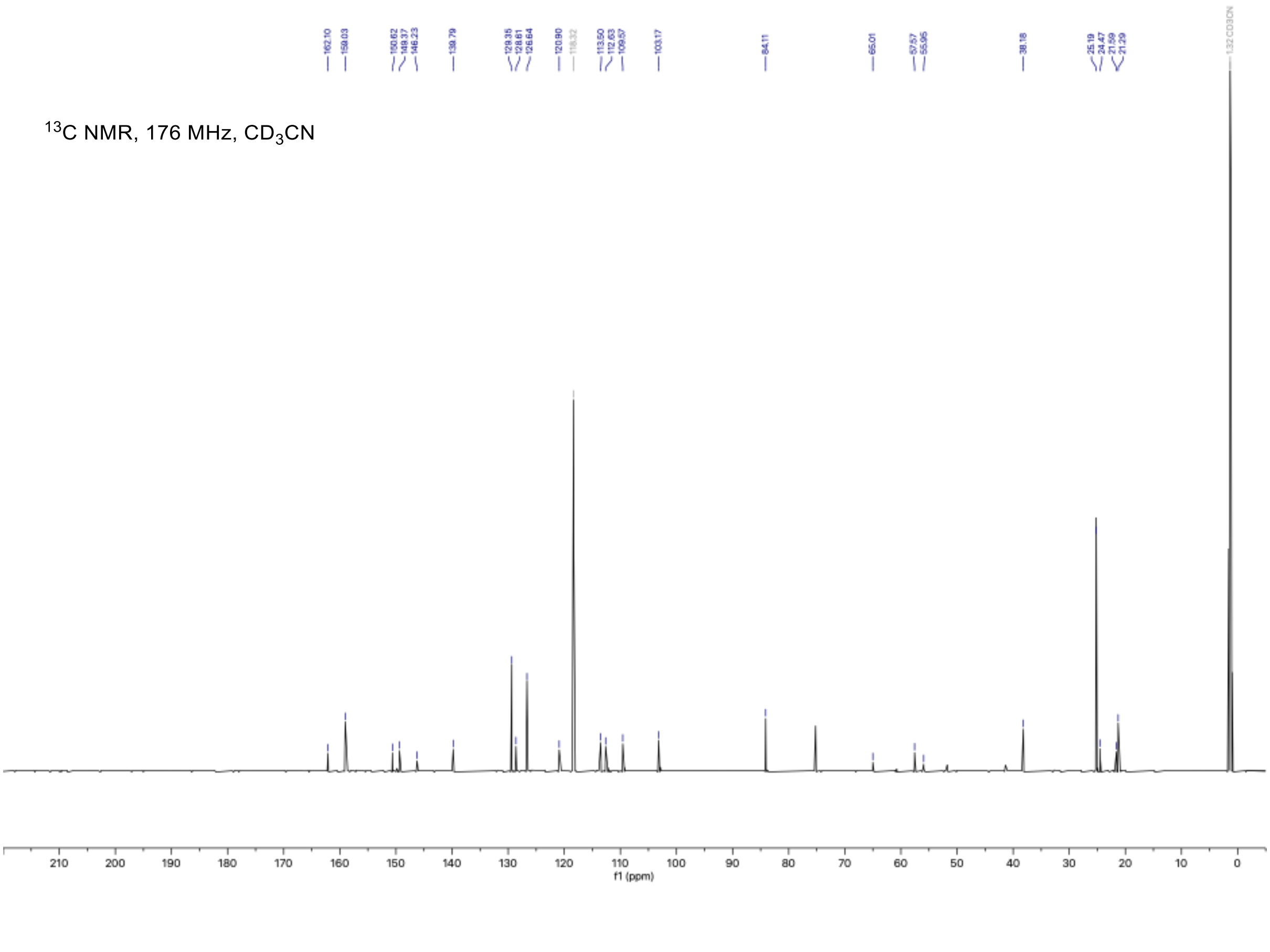

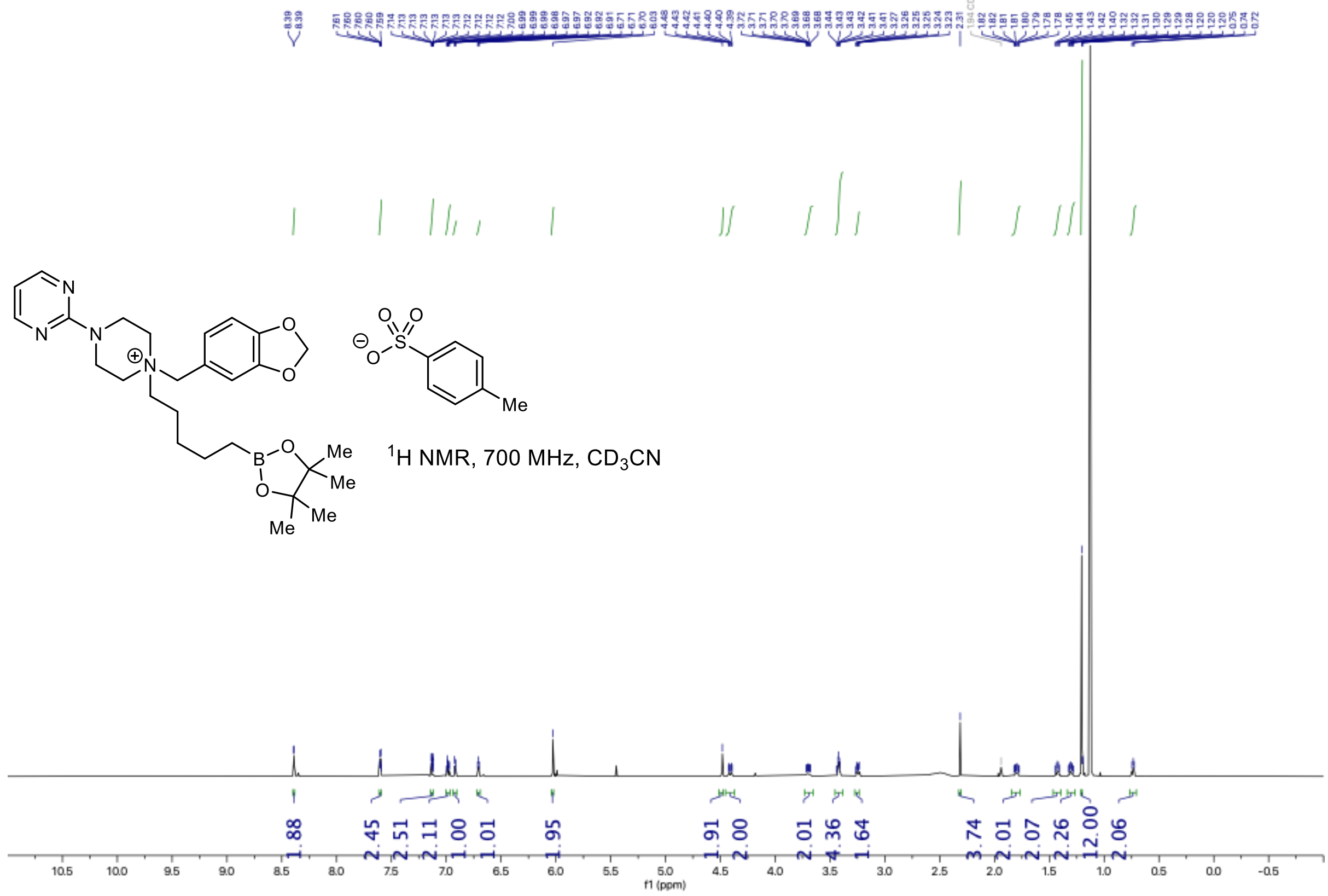

^{1}H NMR, 700 MHz, CD_3CN

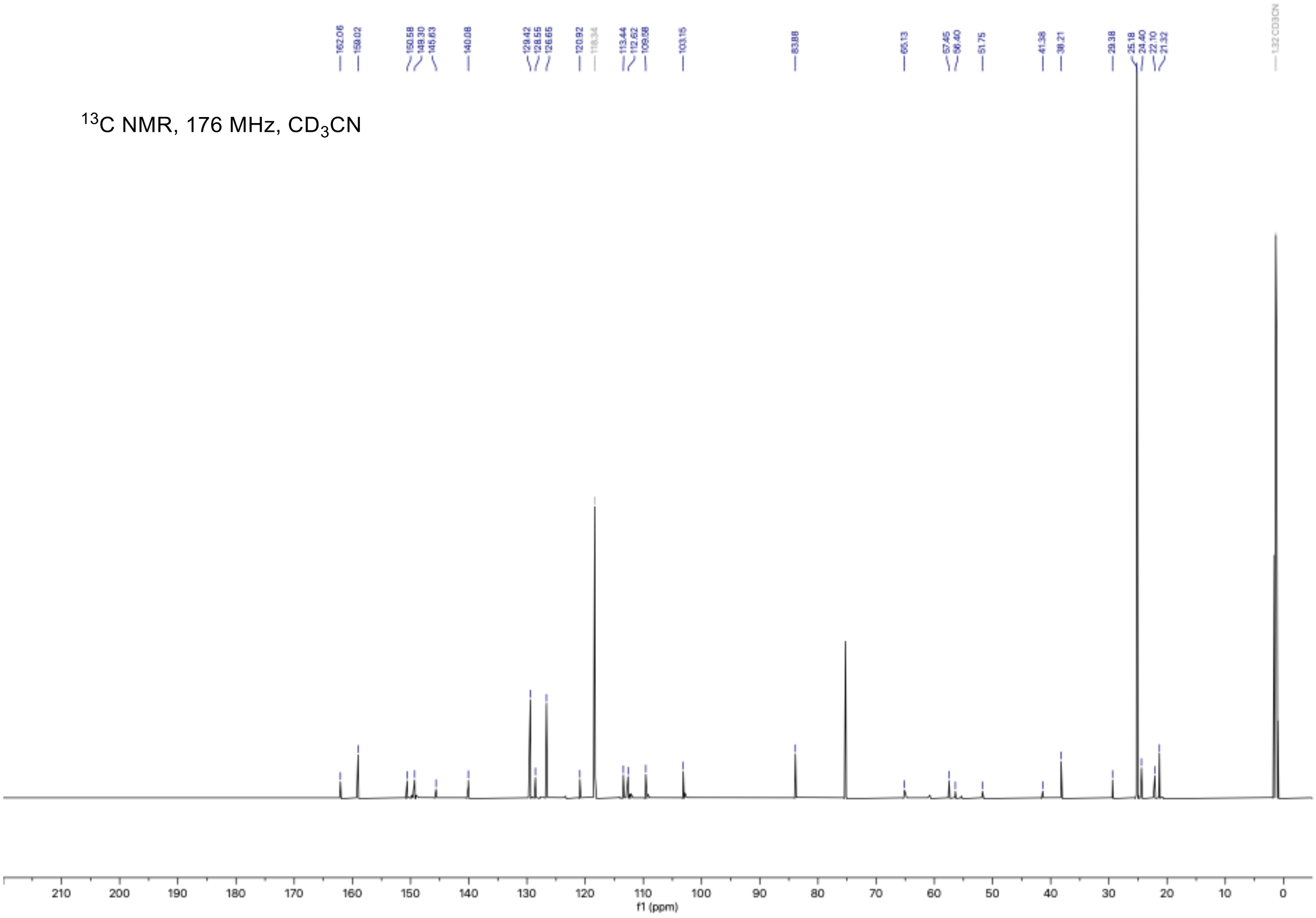

^{13}C NMR, 176 MHz, CD_3CN

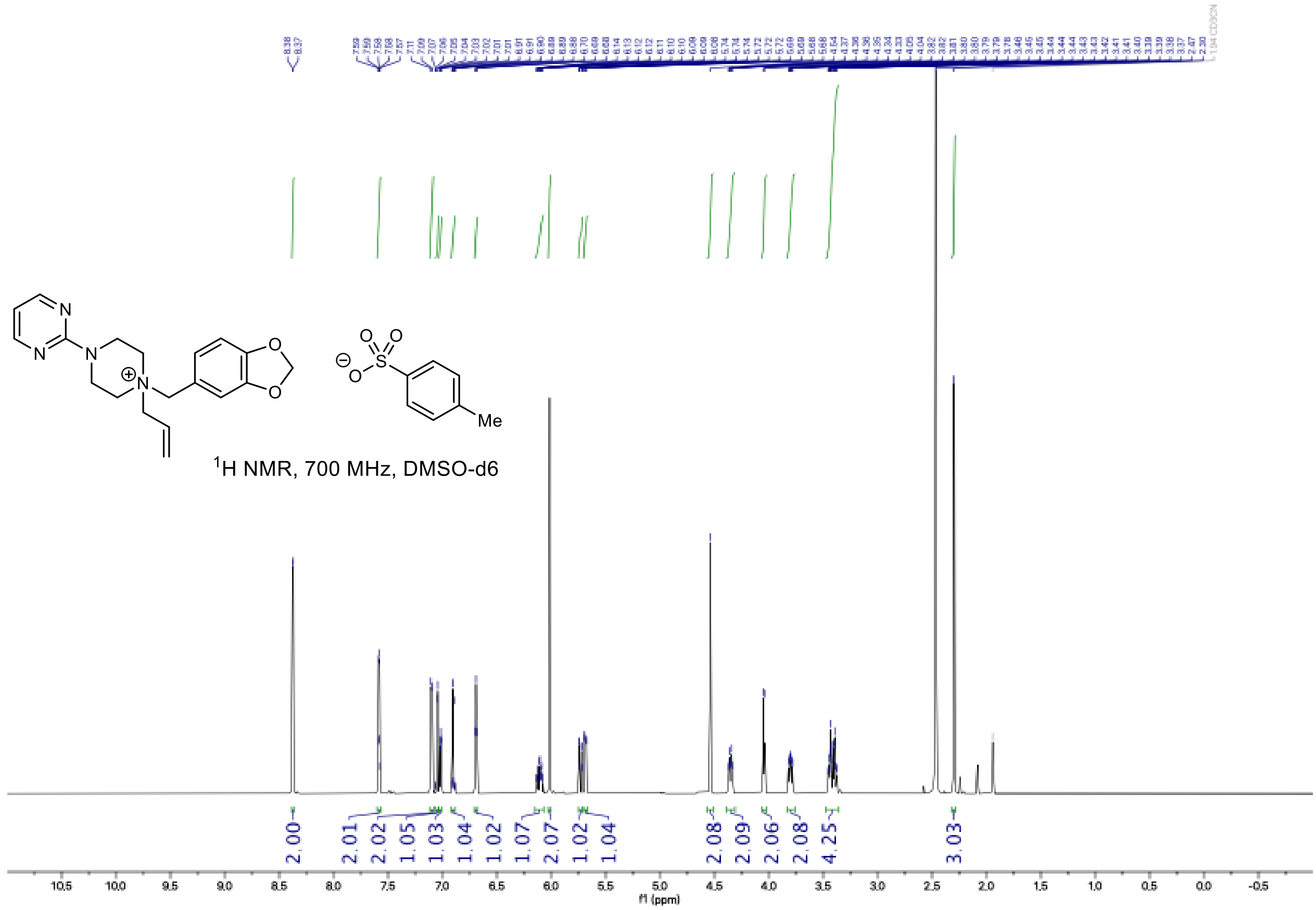

^{1}H NMR, 700 MHz, DMSO-d6

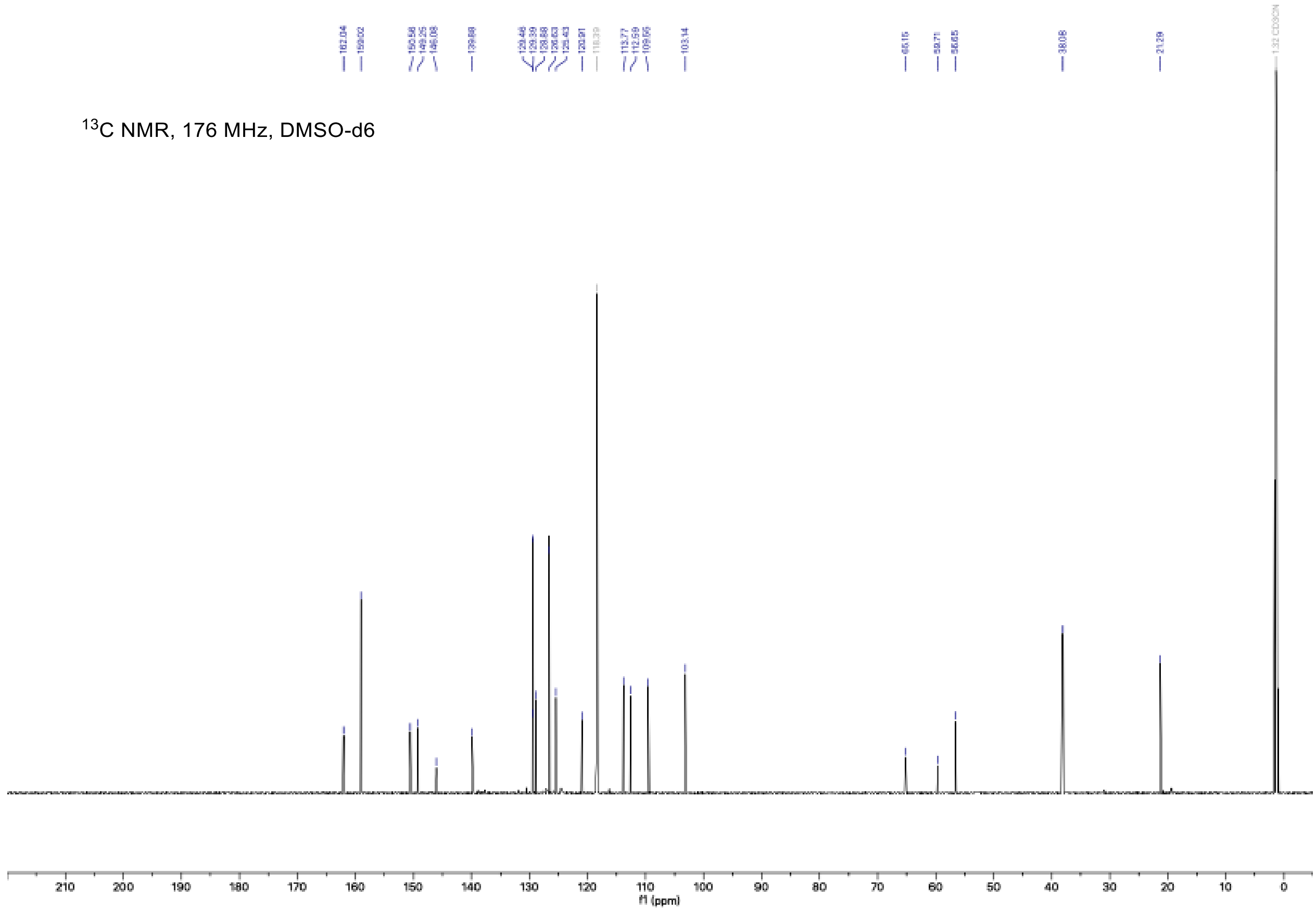

^{13}C NMR, 176 MHz, DMSO-d6

12b

^{1}H NMR, 500 MHz, DMSO-d6

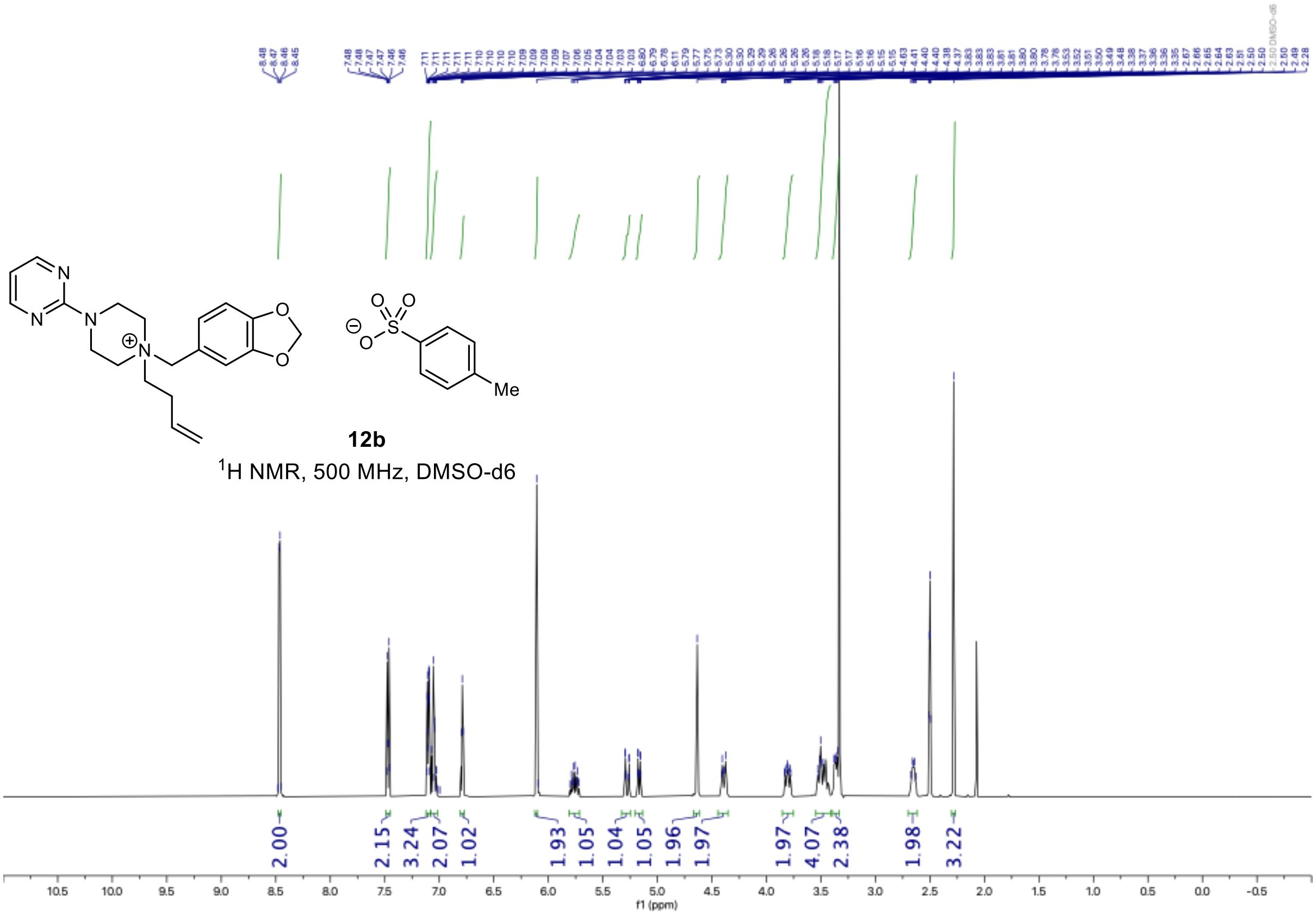

12b

^{13}C NMR, 126 MHz, DMSO-d6

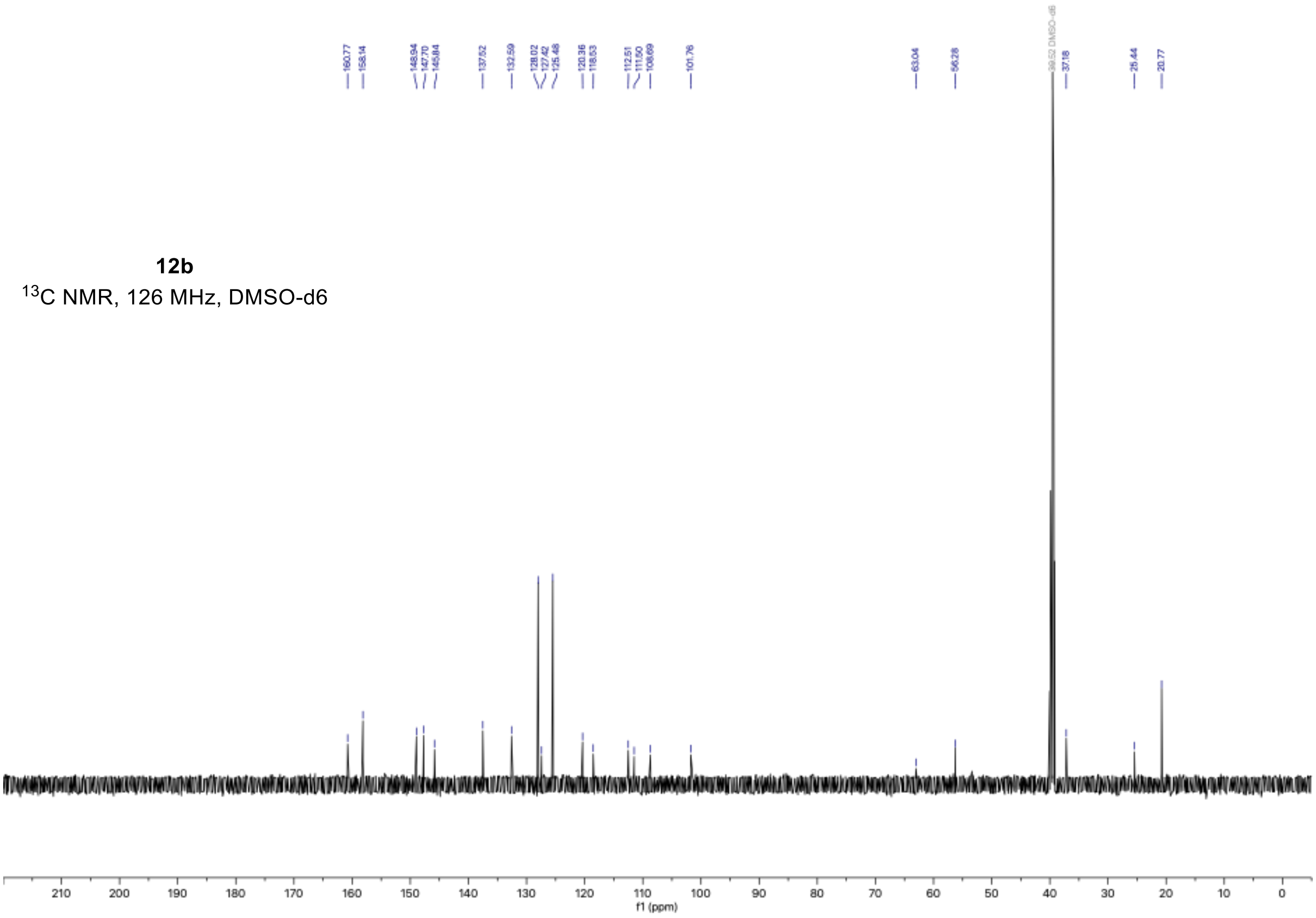

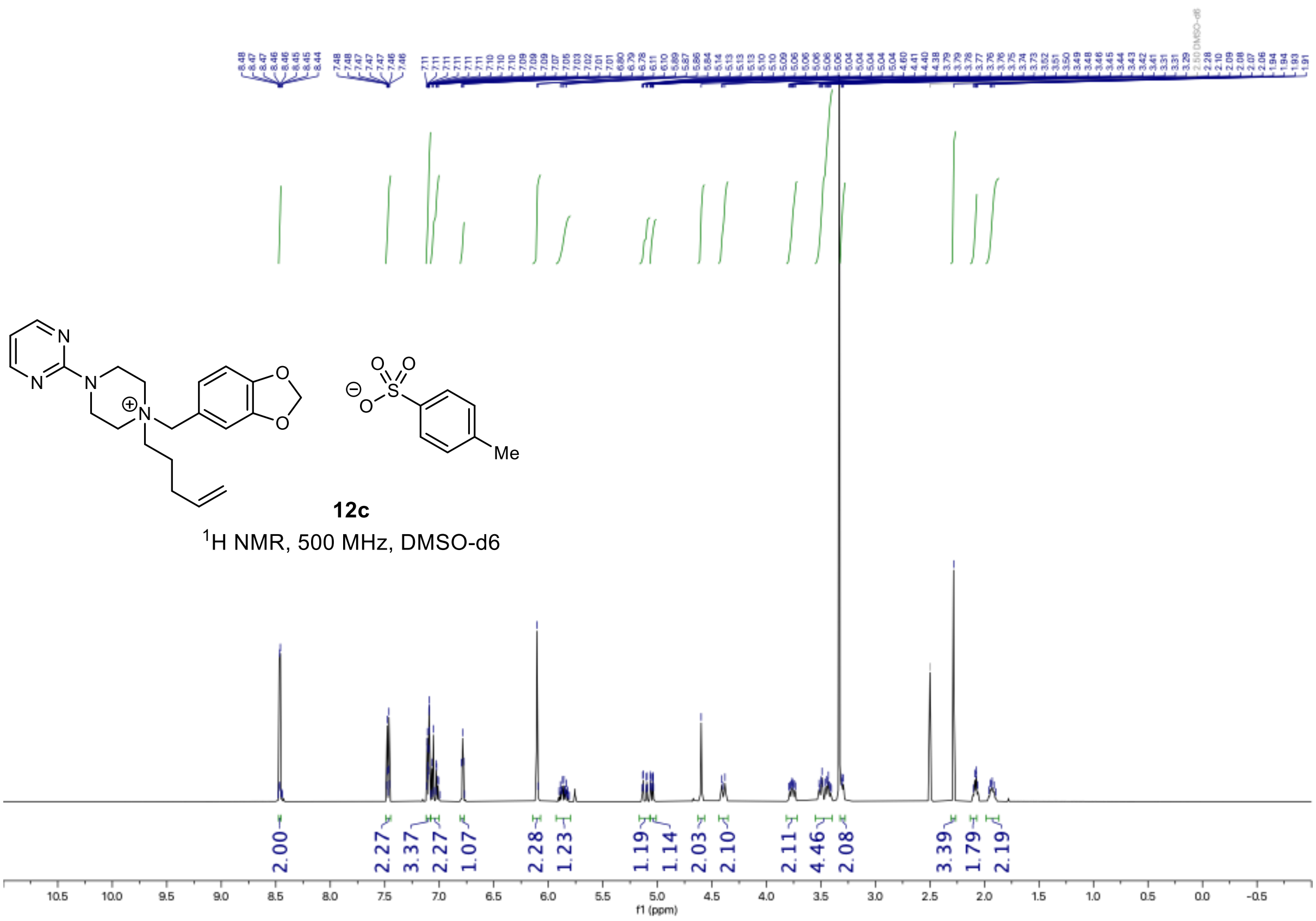

12c

^{1}H NMR, 500 MHz, DMSO-d6

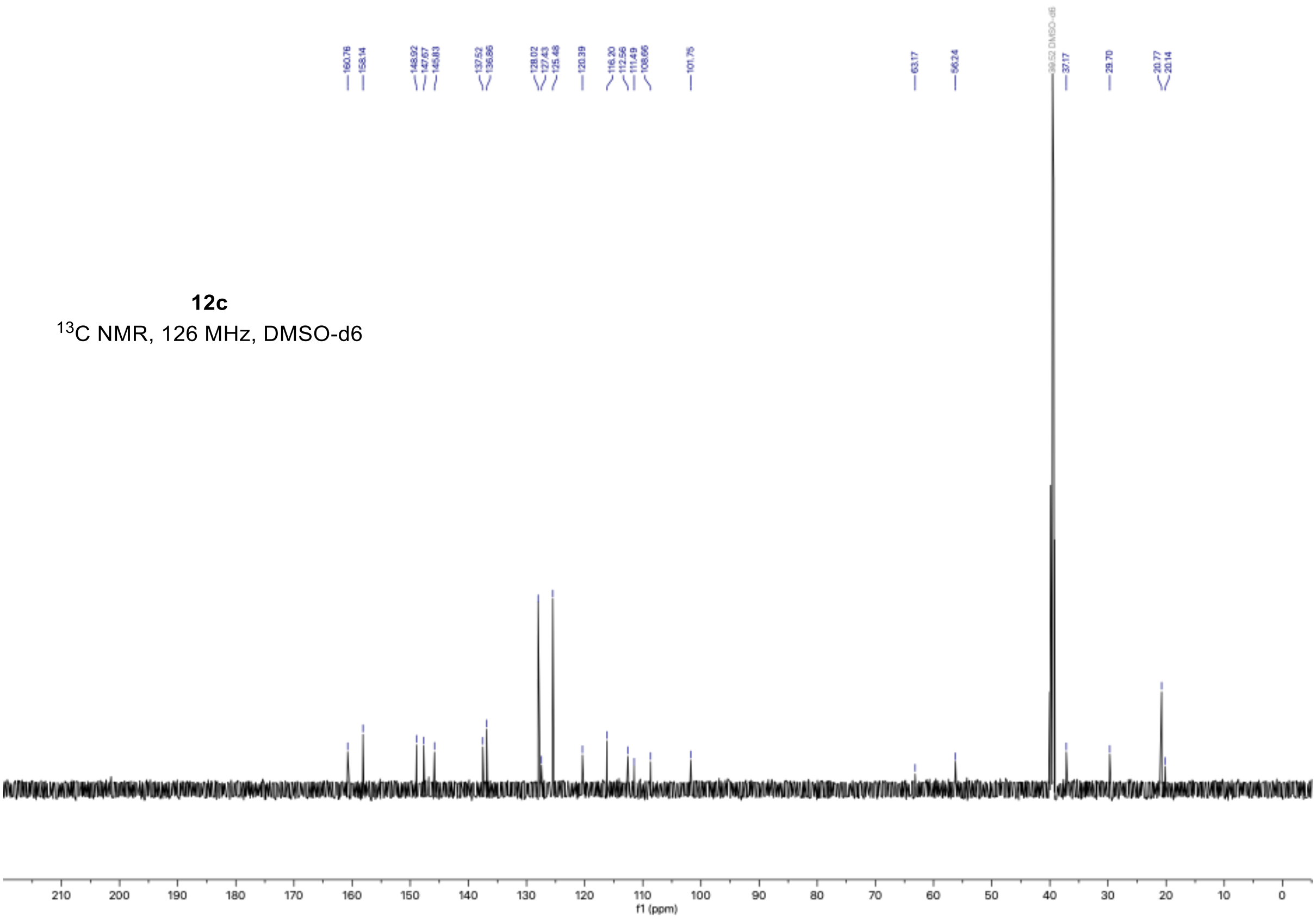

12c

^{13}C NMR, 126 MHz, DMSO-d6

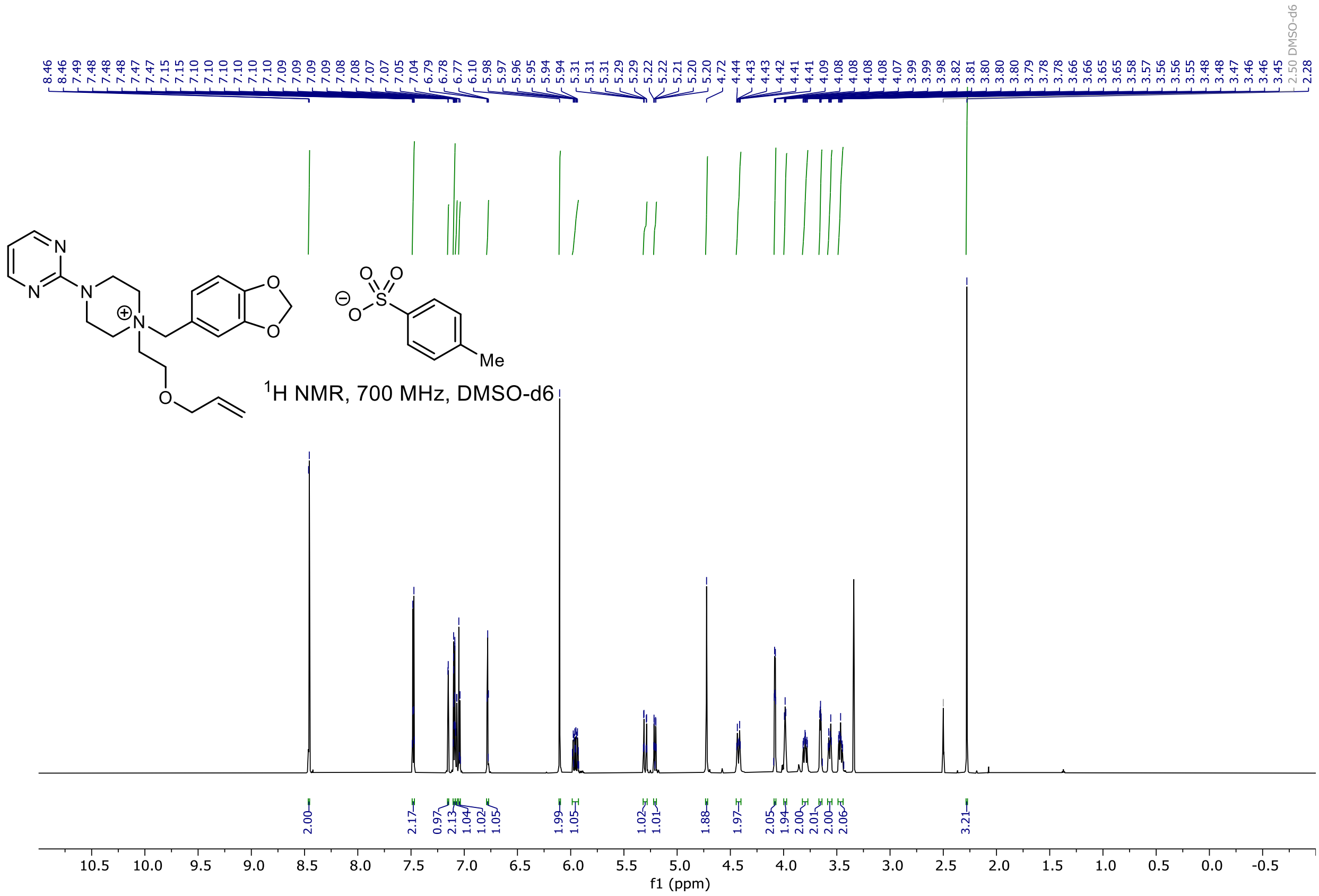

^{1}H NMR, 700 MHz, DMSO-d6

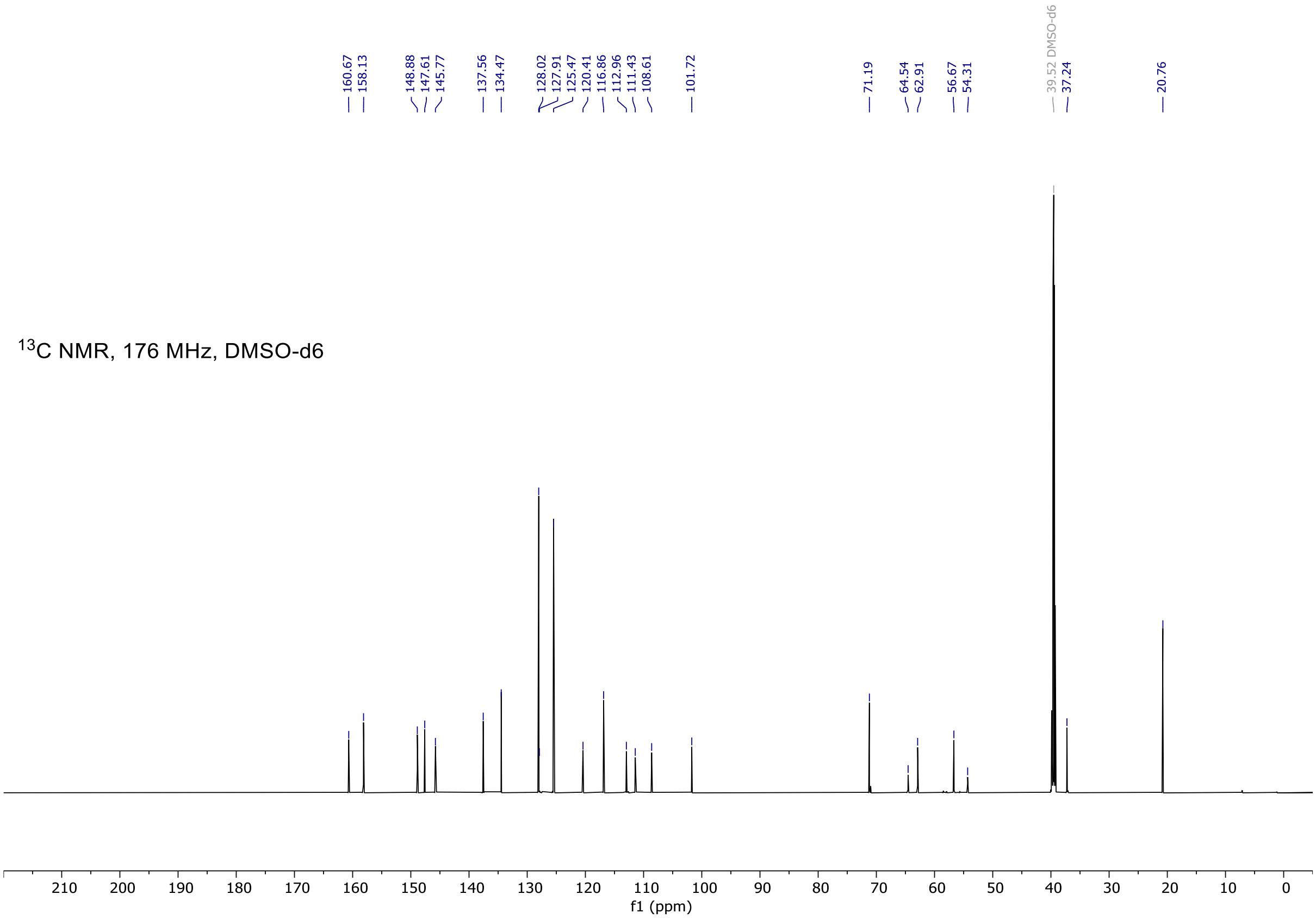

^{13}C NMR, 176 MHz, DMSO-d6

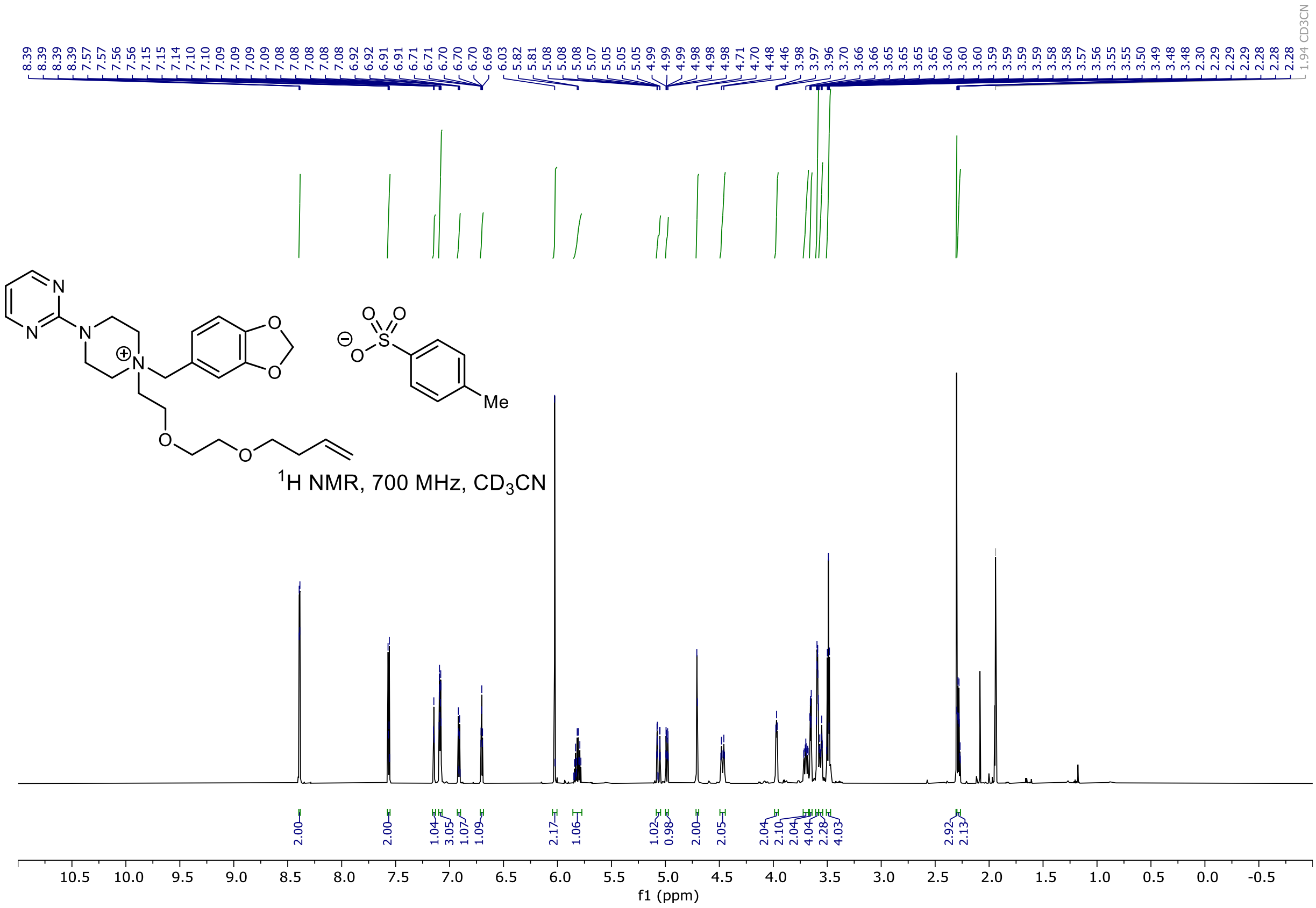

^{1}H NMR, 700 MHz, CD_3CN

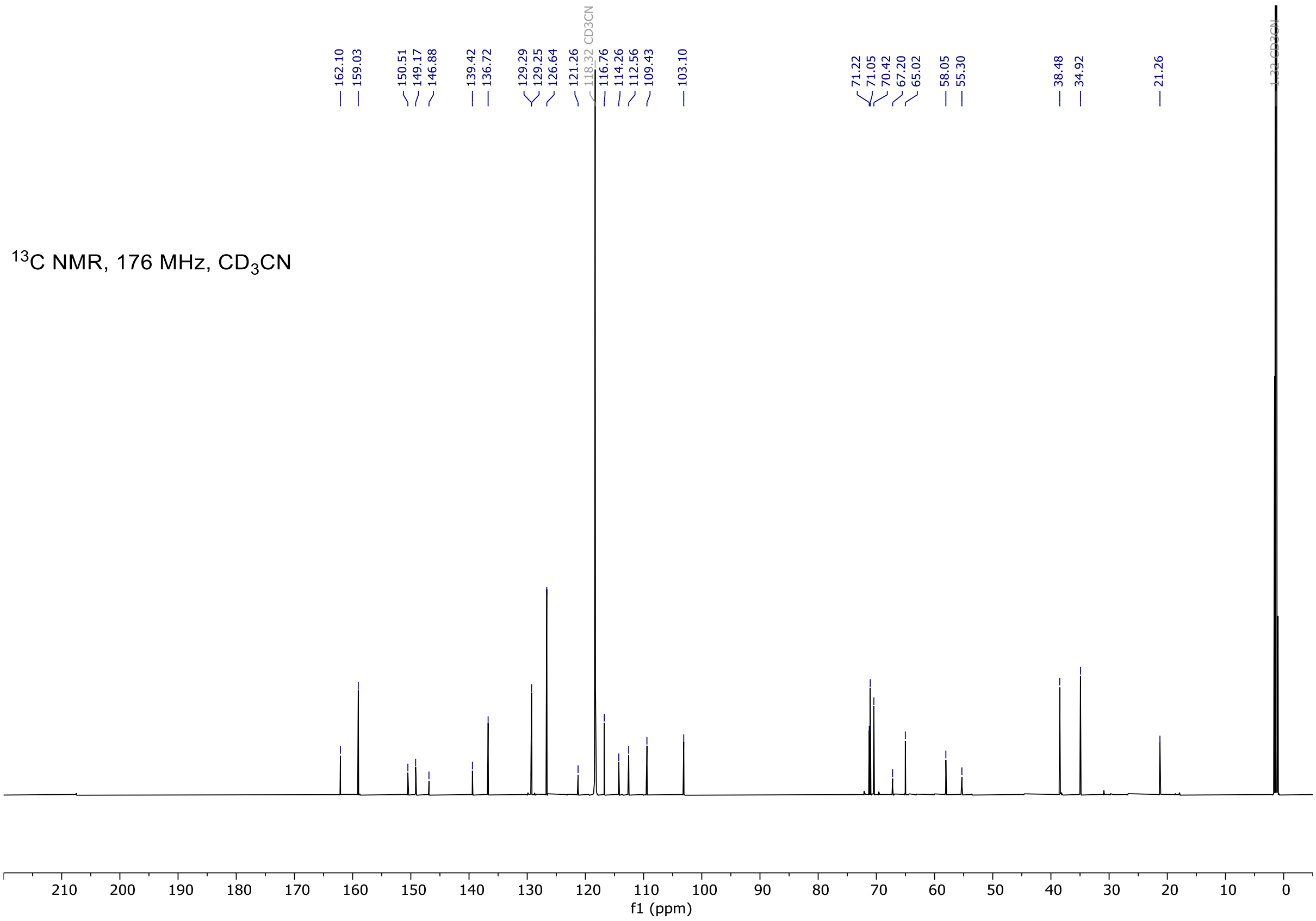

^{13}C NMR, 176 MHz, CD_3CN

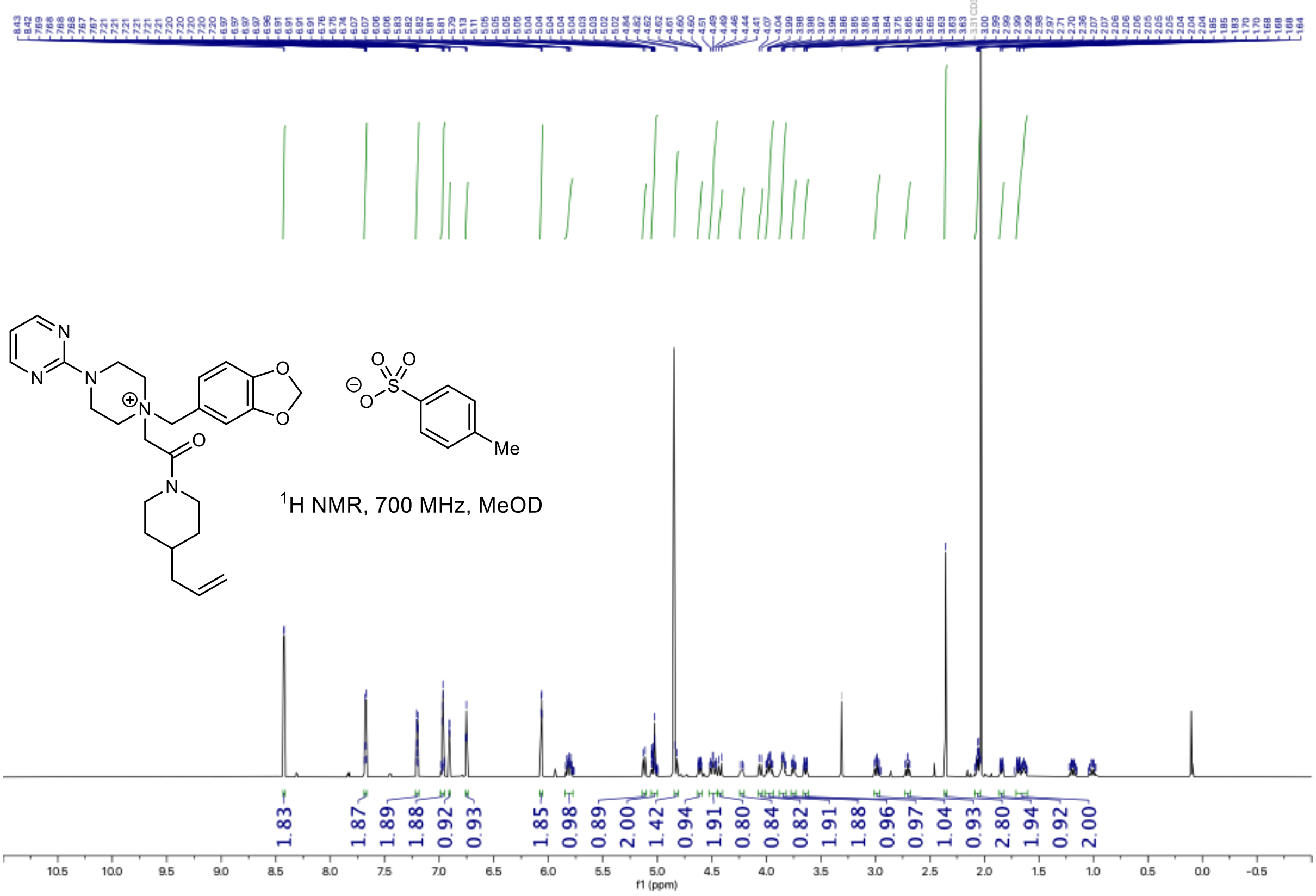

^{1}H NMR, 700 MHz, MeOD
Me
f1 (ppm)

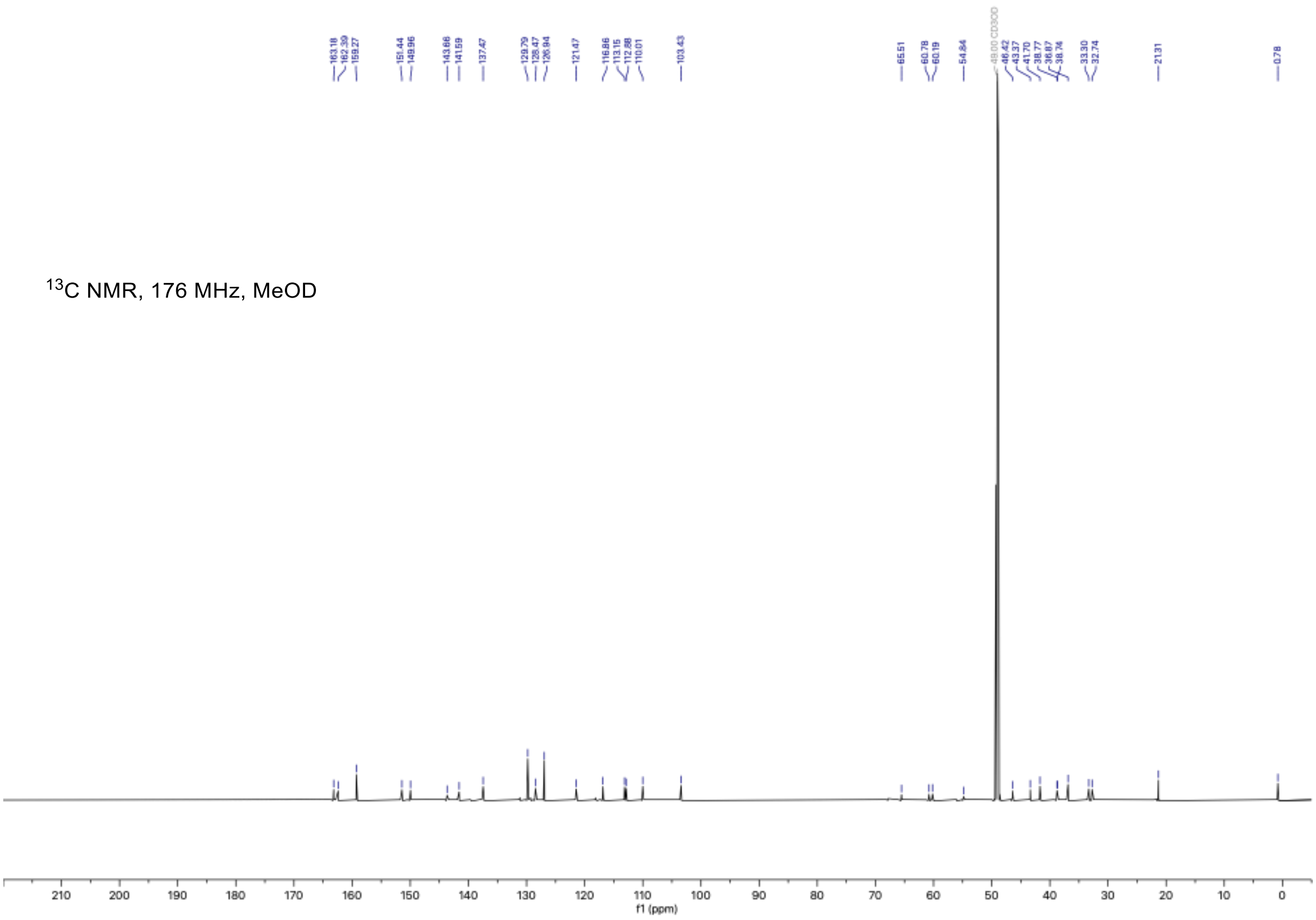

^{13}C NMR, 176 MHz, MeOD
f1 (ppm)

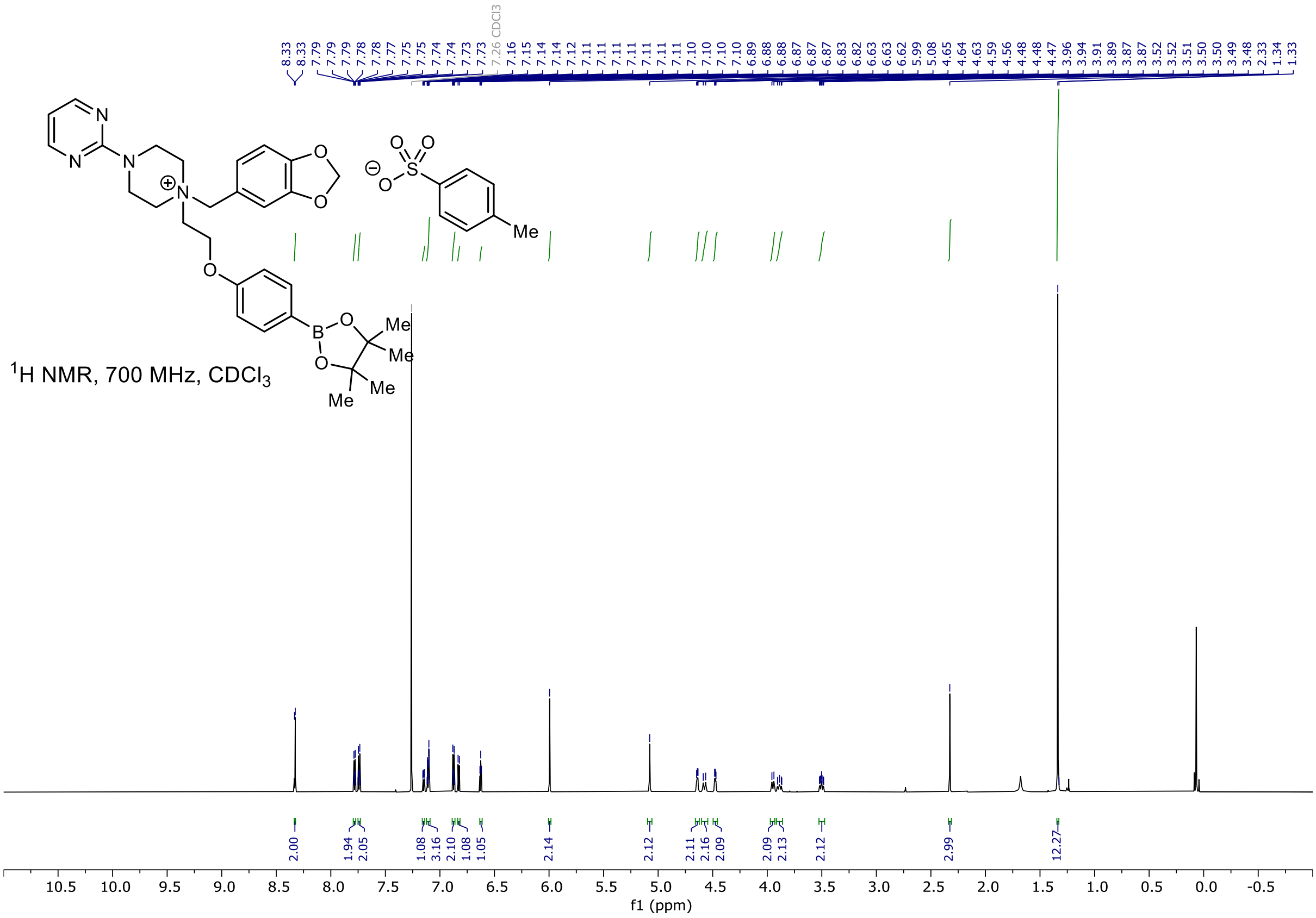

^{1}H NMR, 700 MHz, $CDCl_3$

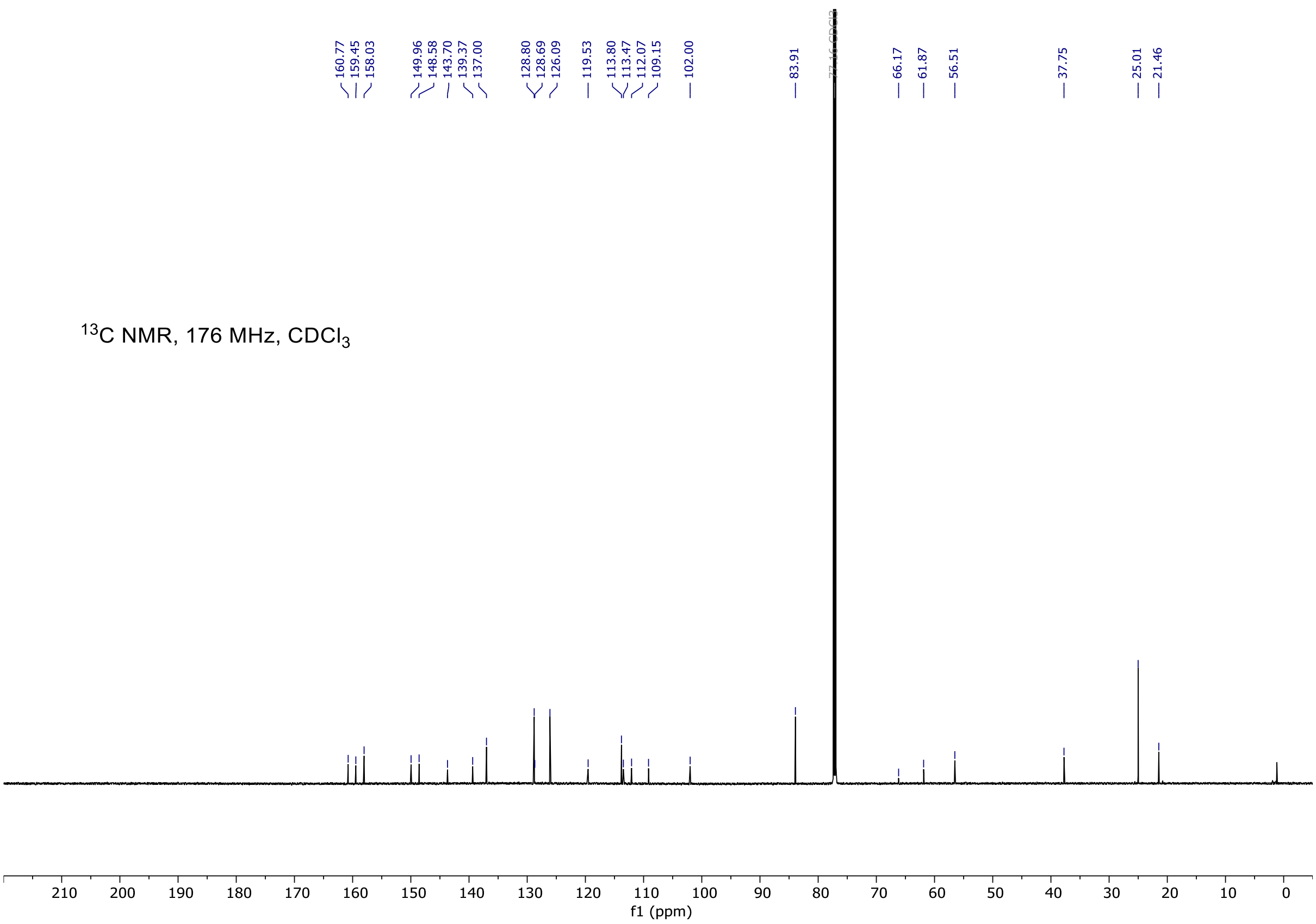

^{13}C NMR, 176 MHz, $CDCl_3$

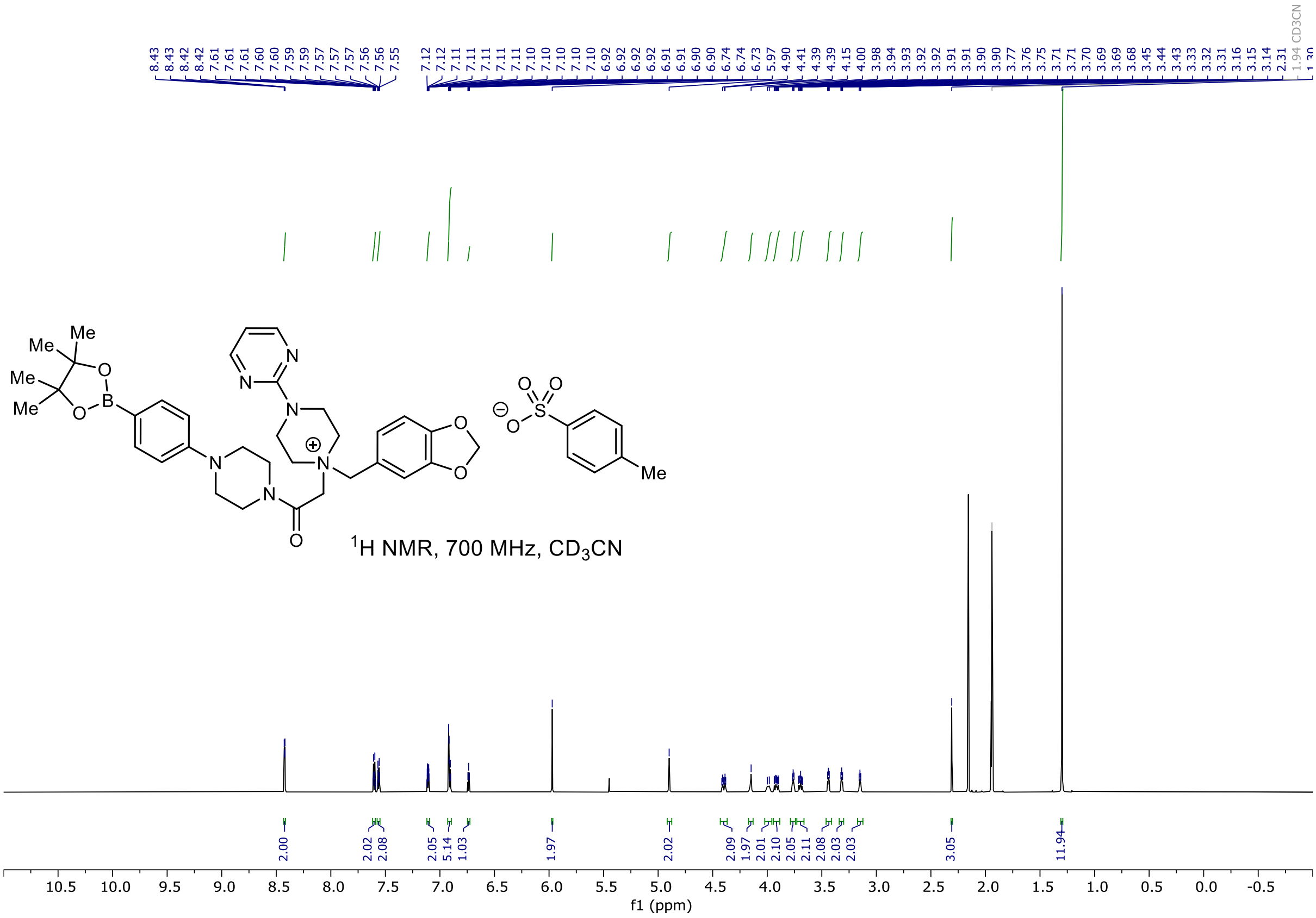
Me
Me
Me
Me
O
B
O
N
N
N
N
N
O
O
O
O
S
O
O
Me
^{1}H NMR, 700 MHz, CD_3CN
2.00
2.02
2.08
2.05
5.14
1.03
1.97
2.02
2.09
1.97
2.01
2.10
2.05
2.11
2.08
2.03
2.03
3.05
11.94
f1 (ppm)

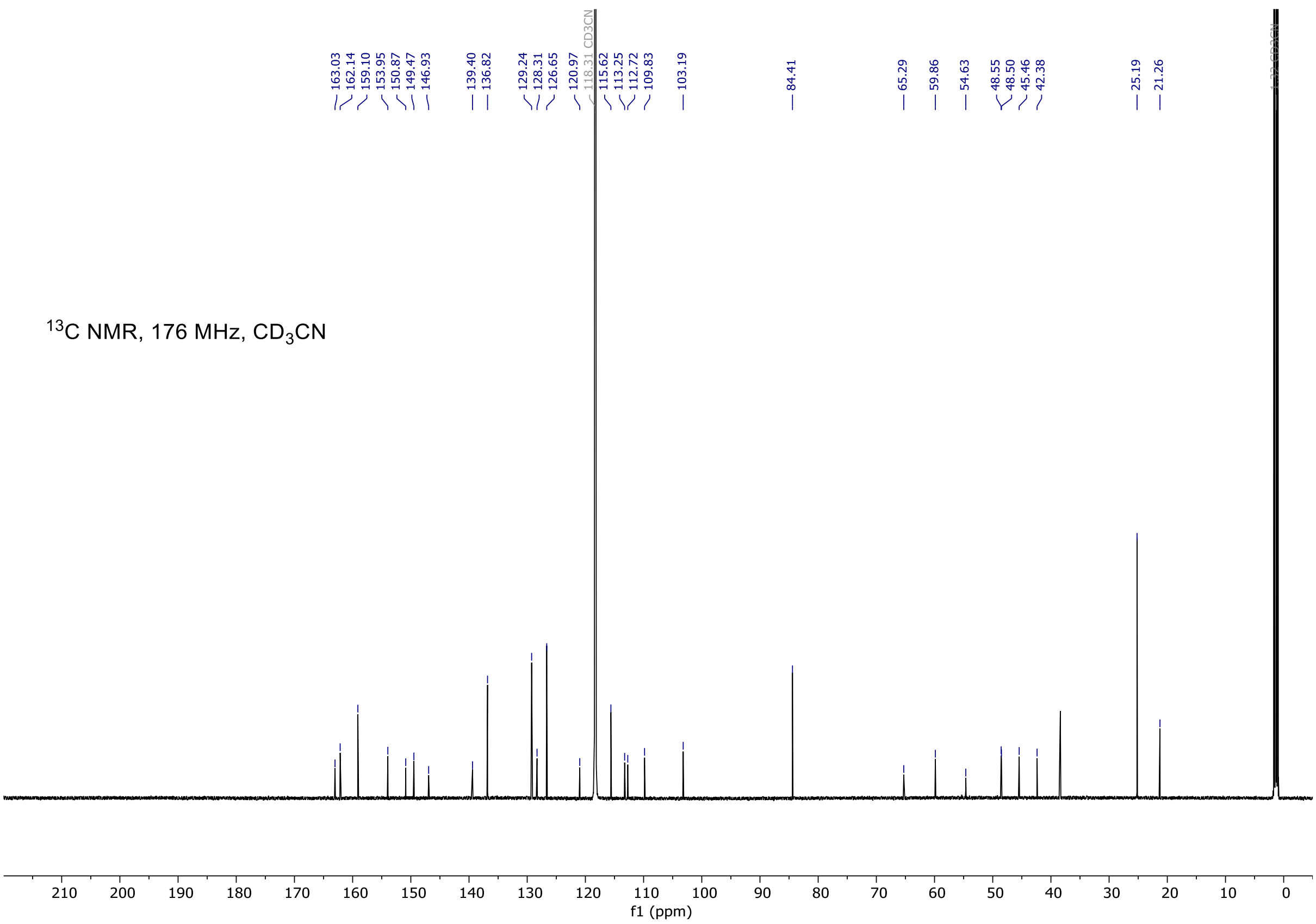
^{13}C NMR, 176 MHz, CD_3CN
163.03
162.14
159.10
153.95
150.87
149.47
146.93
139.40
136.82
129.24
128.31
126.65
120.97
118.31 CD3CN
115.62
113.25
112.72
109.83
103.19
84.41
65.29
59.86
54.63
48.55
48.50
45.46
42.38
25.19
21.26
f1 (ppm)

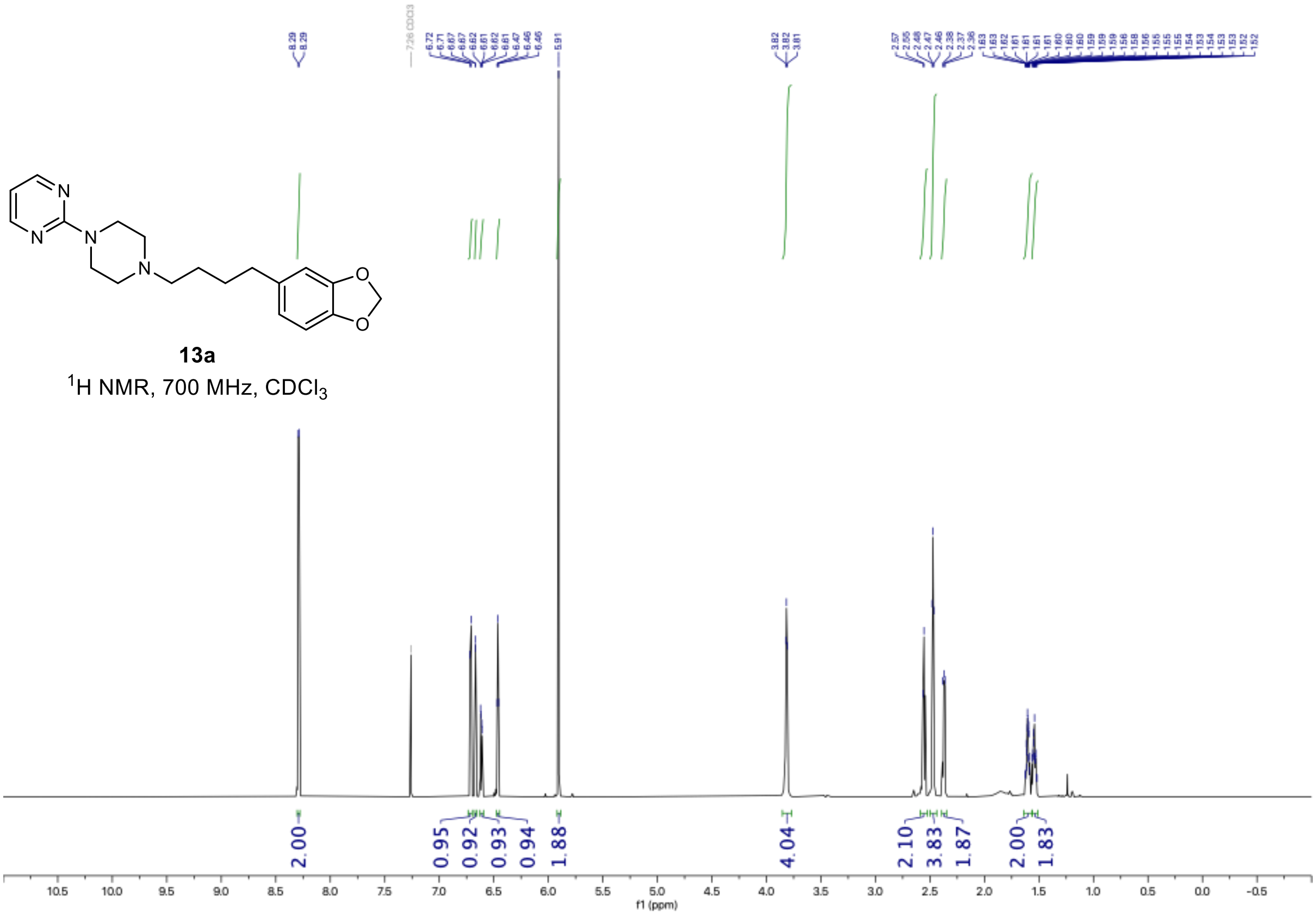

13a

^1H NMR, 700 MHz, $CDCl_3$

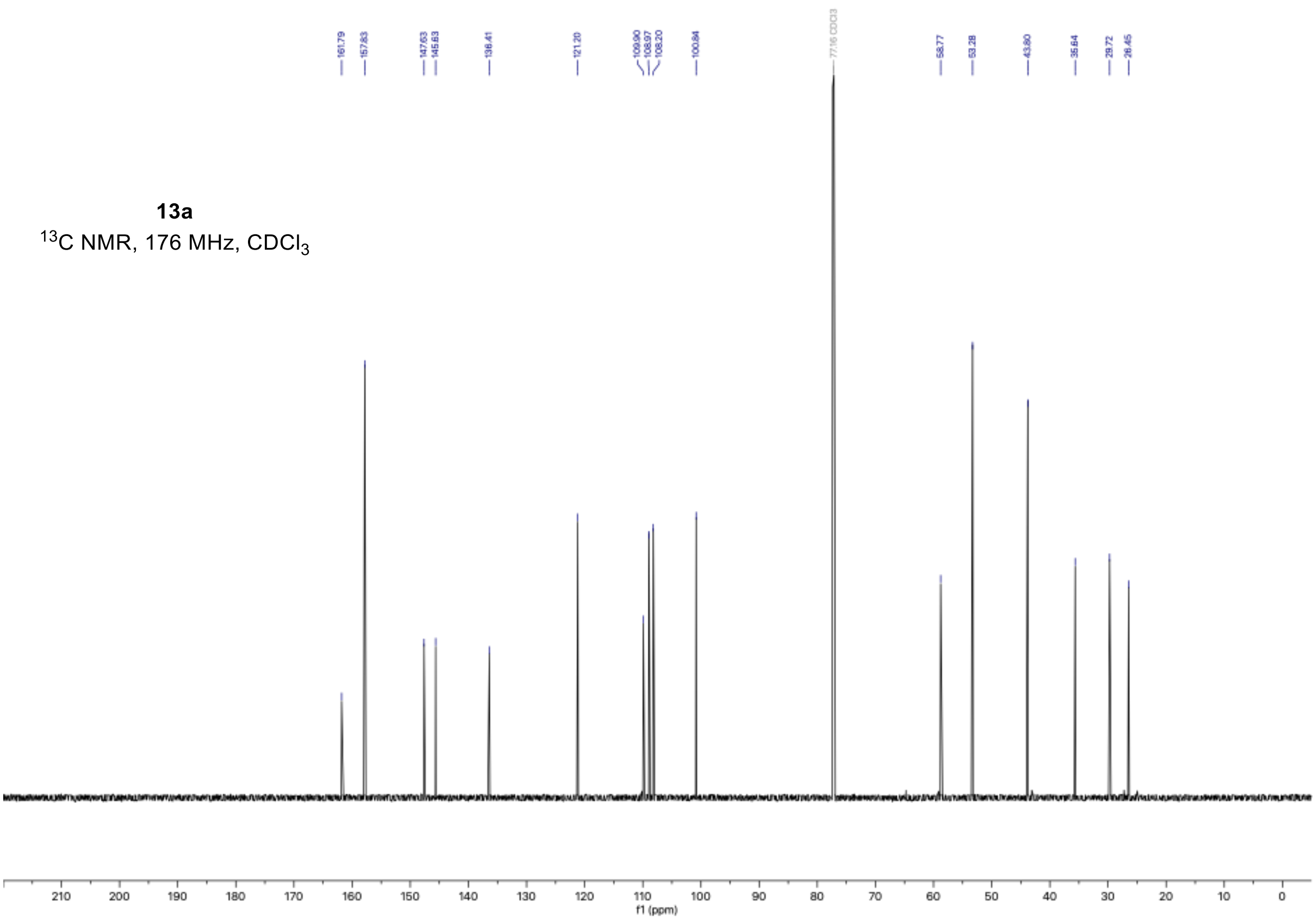

13a

^{13}C NMR, 176 MHz, $CDCl_3$

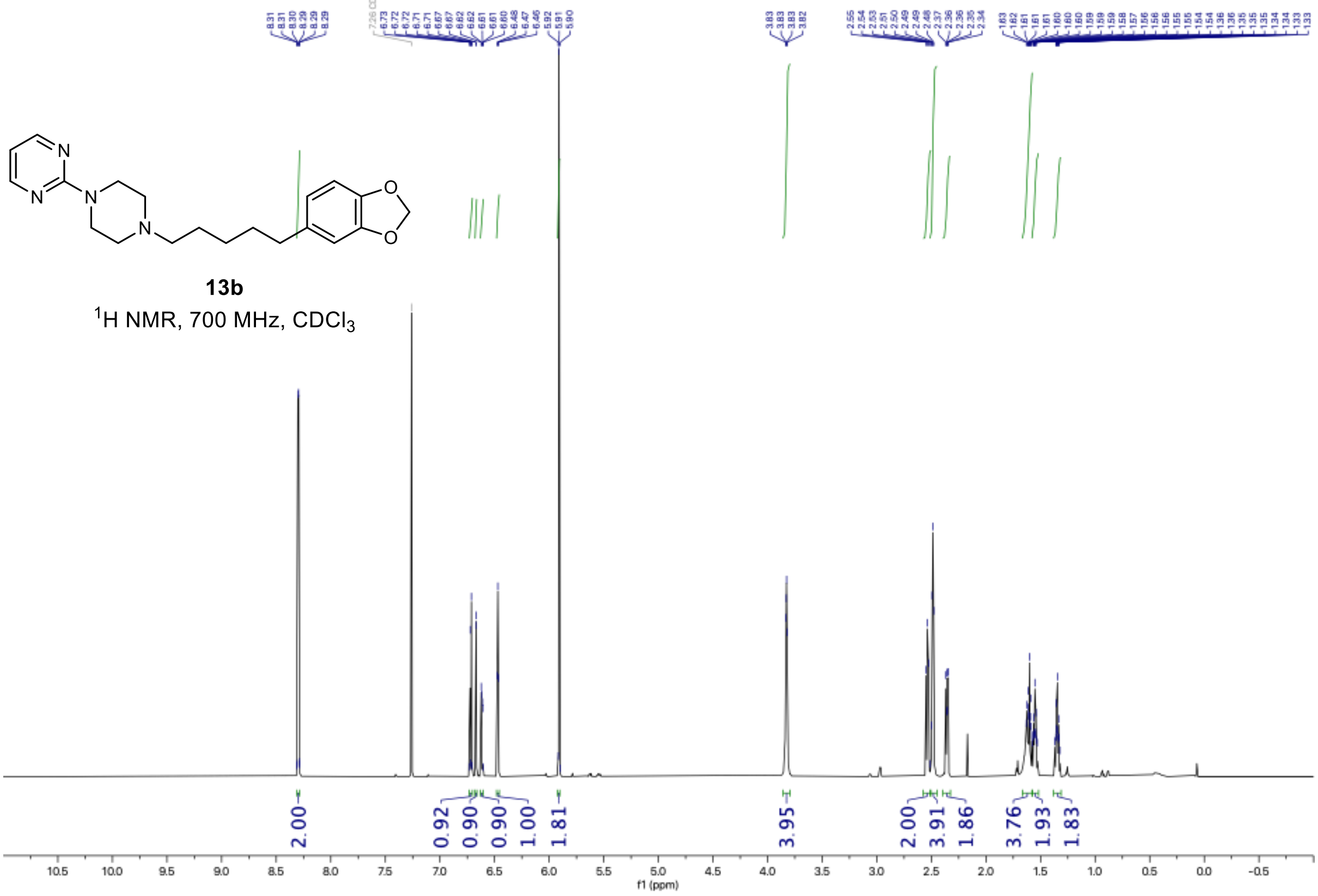

13b

^{1}H NMR, 700 MHz, $CDCl_3$

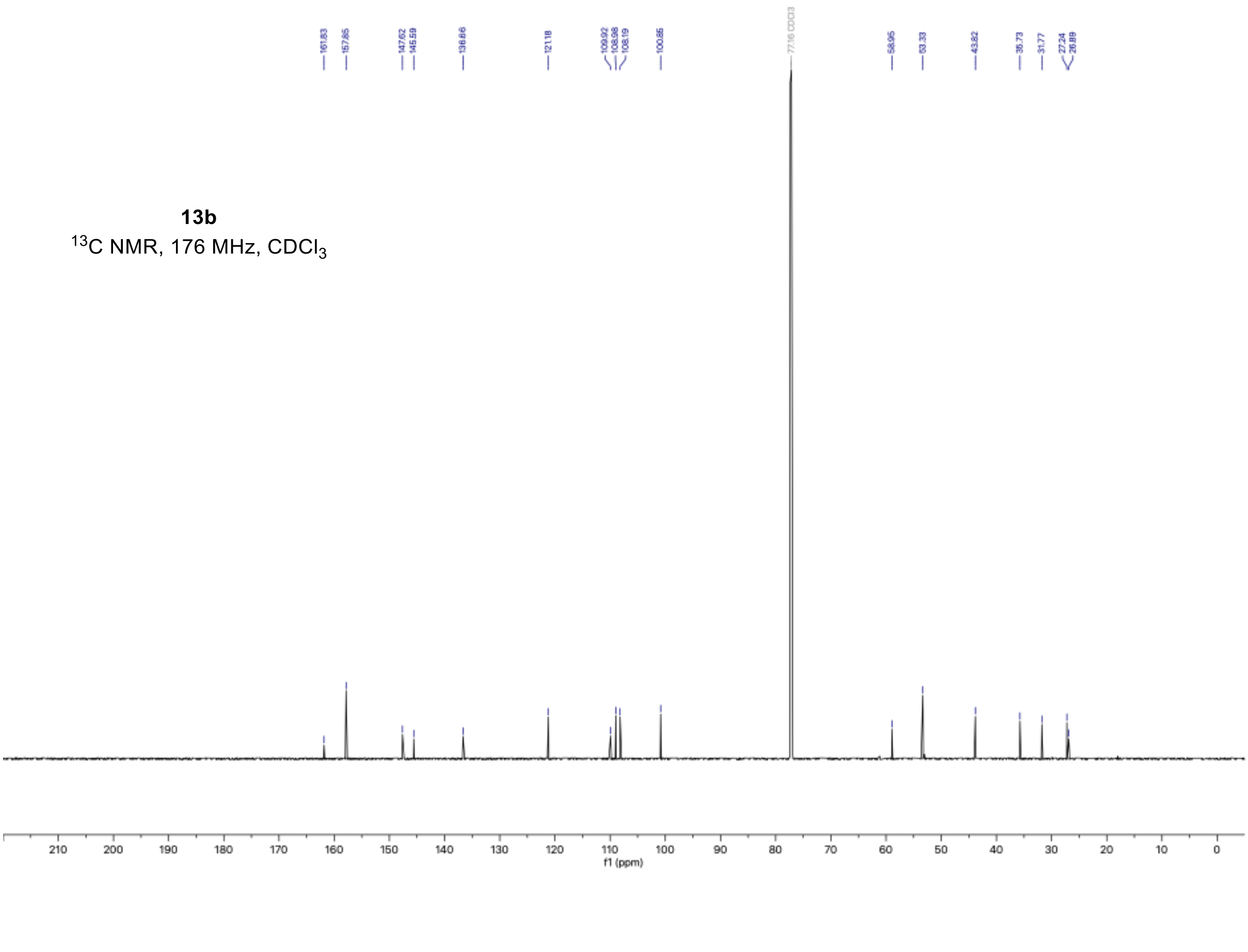

13b

^{13}C NMR, 176 MHz, $CDCl_3$

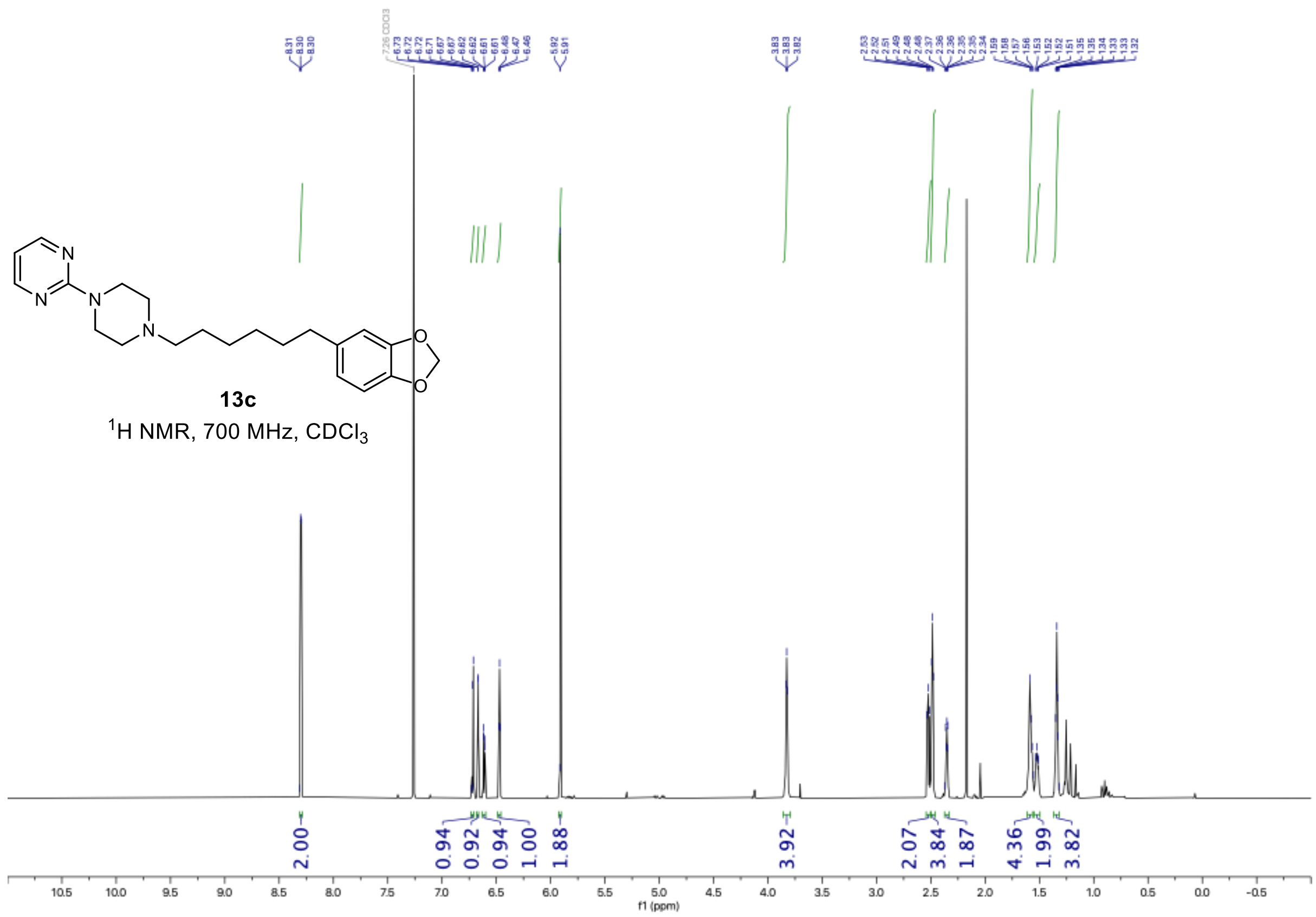
13c
1H NMR, 700 MHz, CDCl3
f1 (ppm)

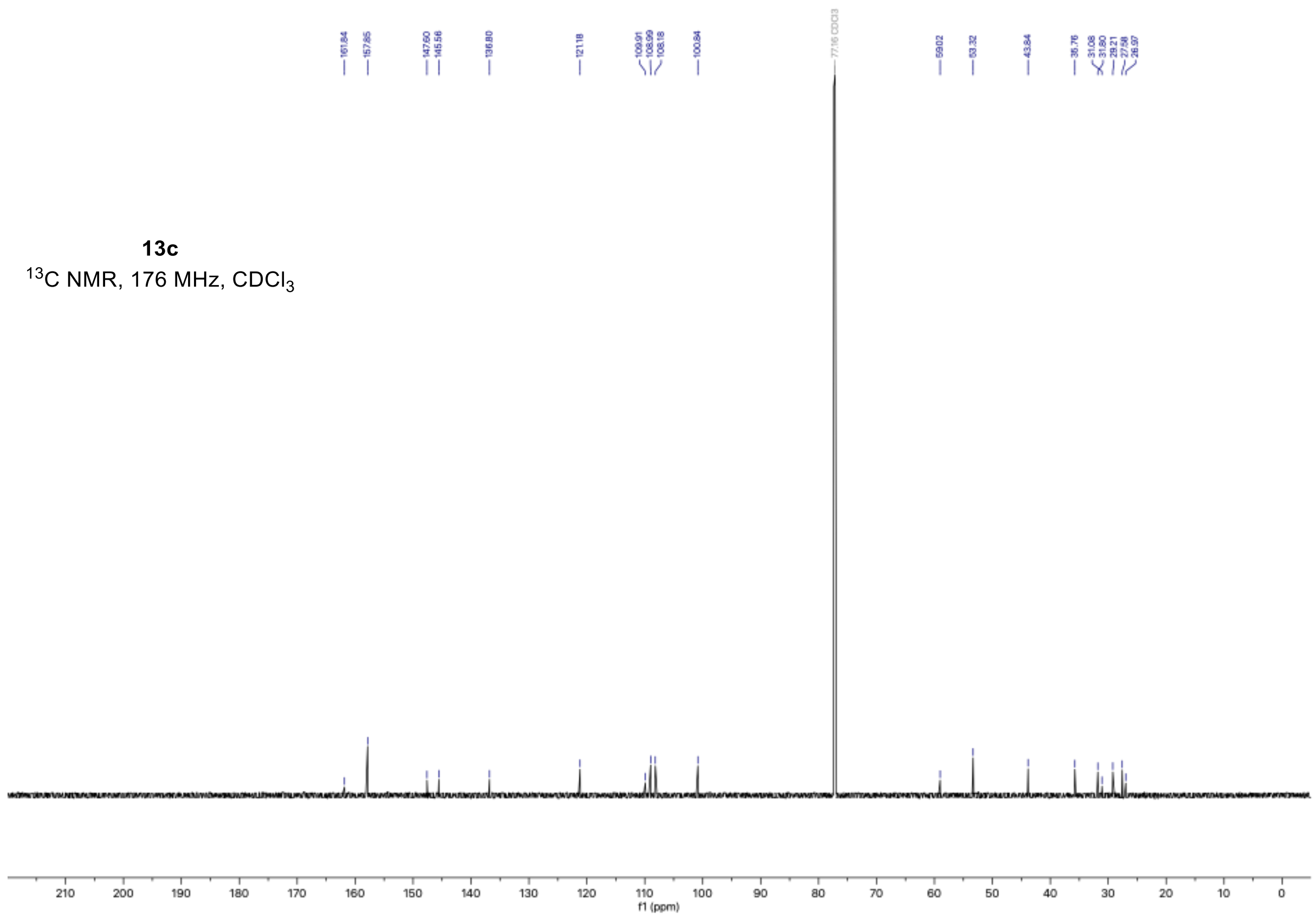
13c
13C NMR, 176 MHz, CDCl3
f1 (ppm)

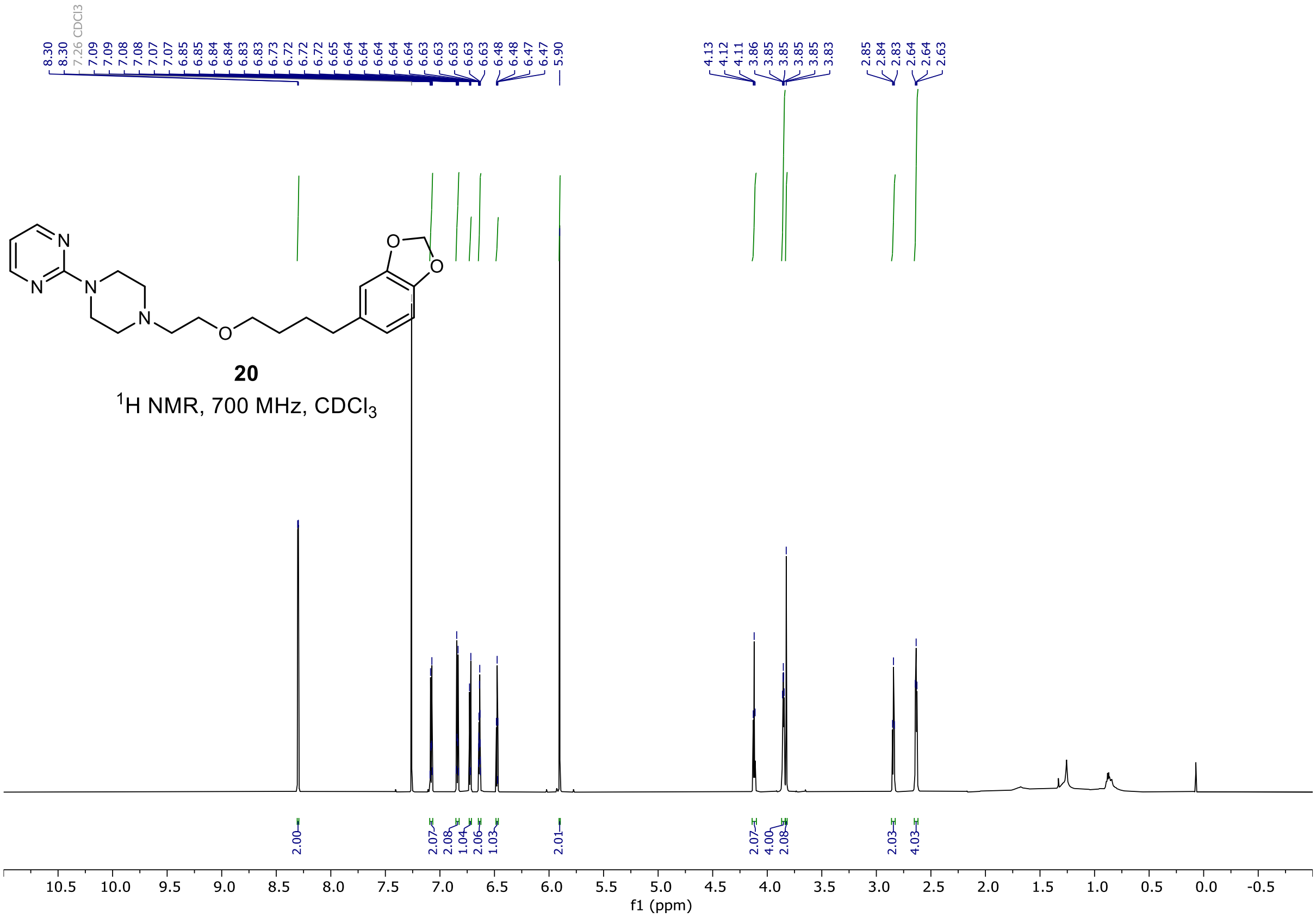

20

^{1}H NMR, 700 MHz, $CDCl_3$

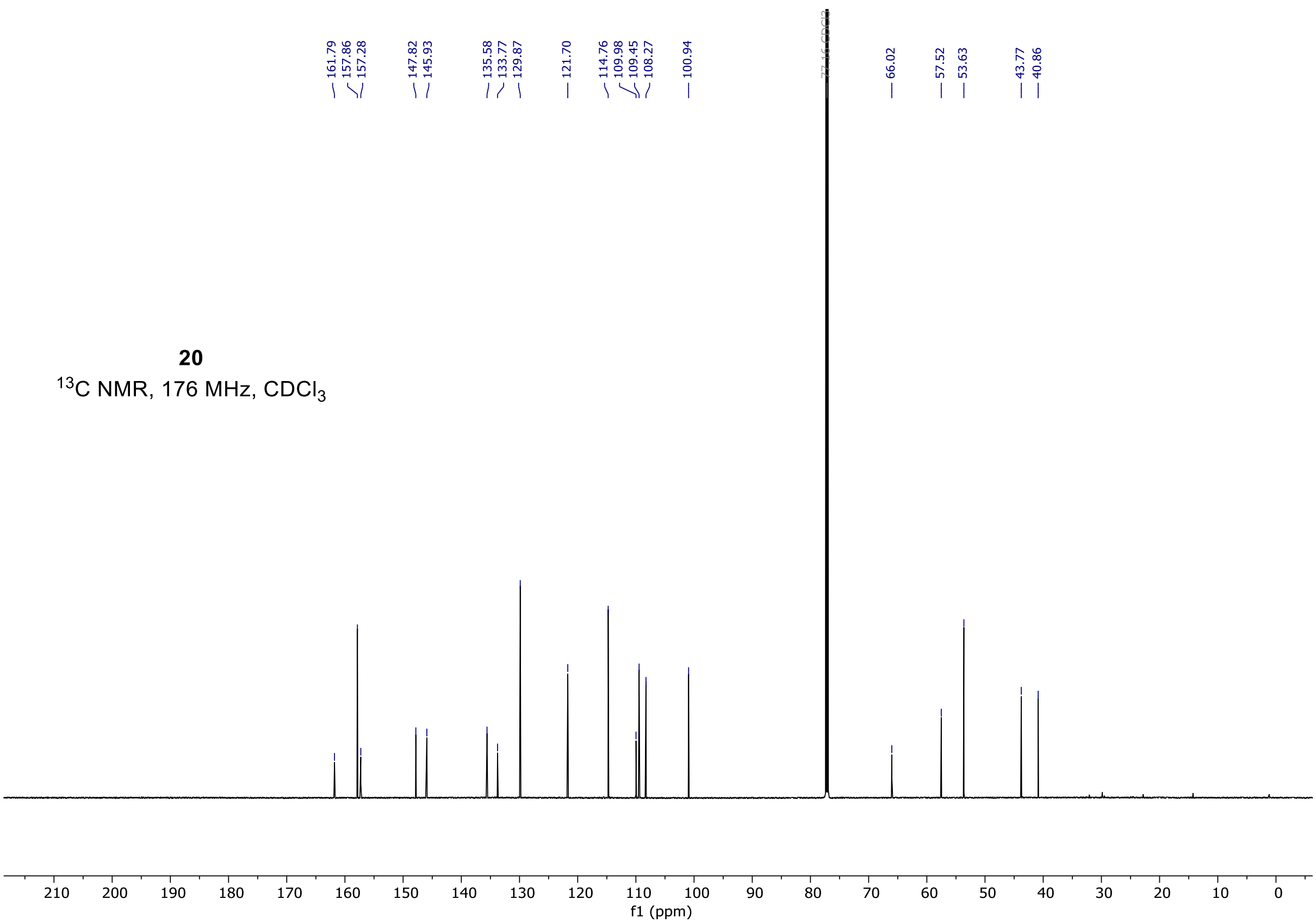

20

^{13}C NMR, 176 MHz, $CDCl_3$

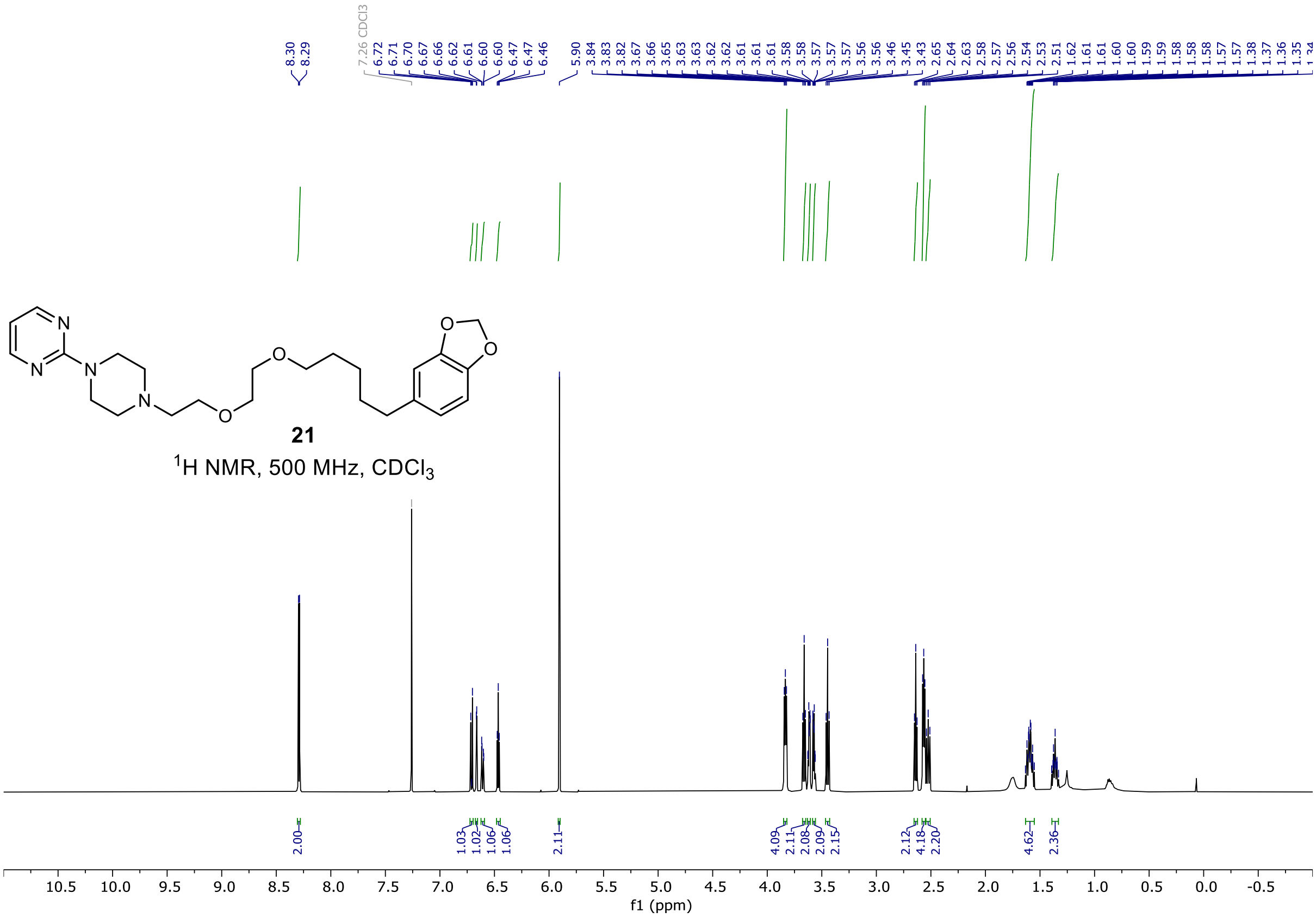

21

^{1}H NMR, 500 MHz, $CDCl_3$

21

^{13}C NMR, 126 MHz, $CDCl_3$

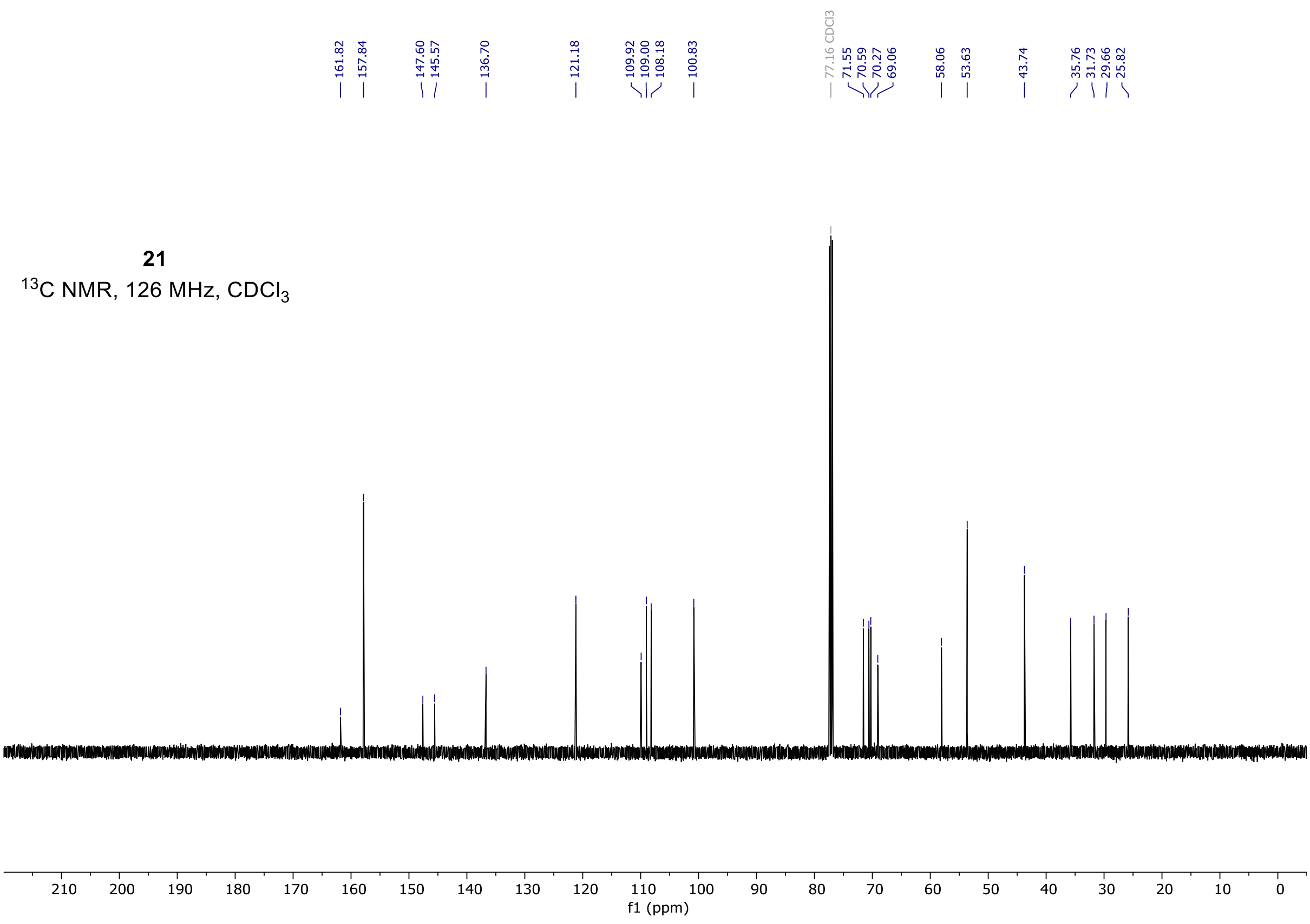

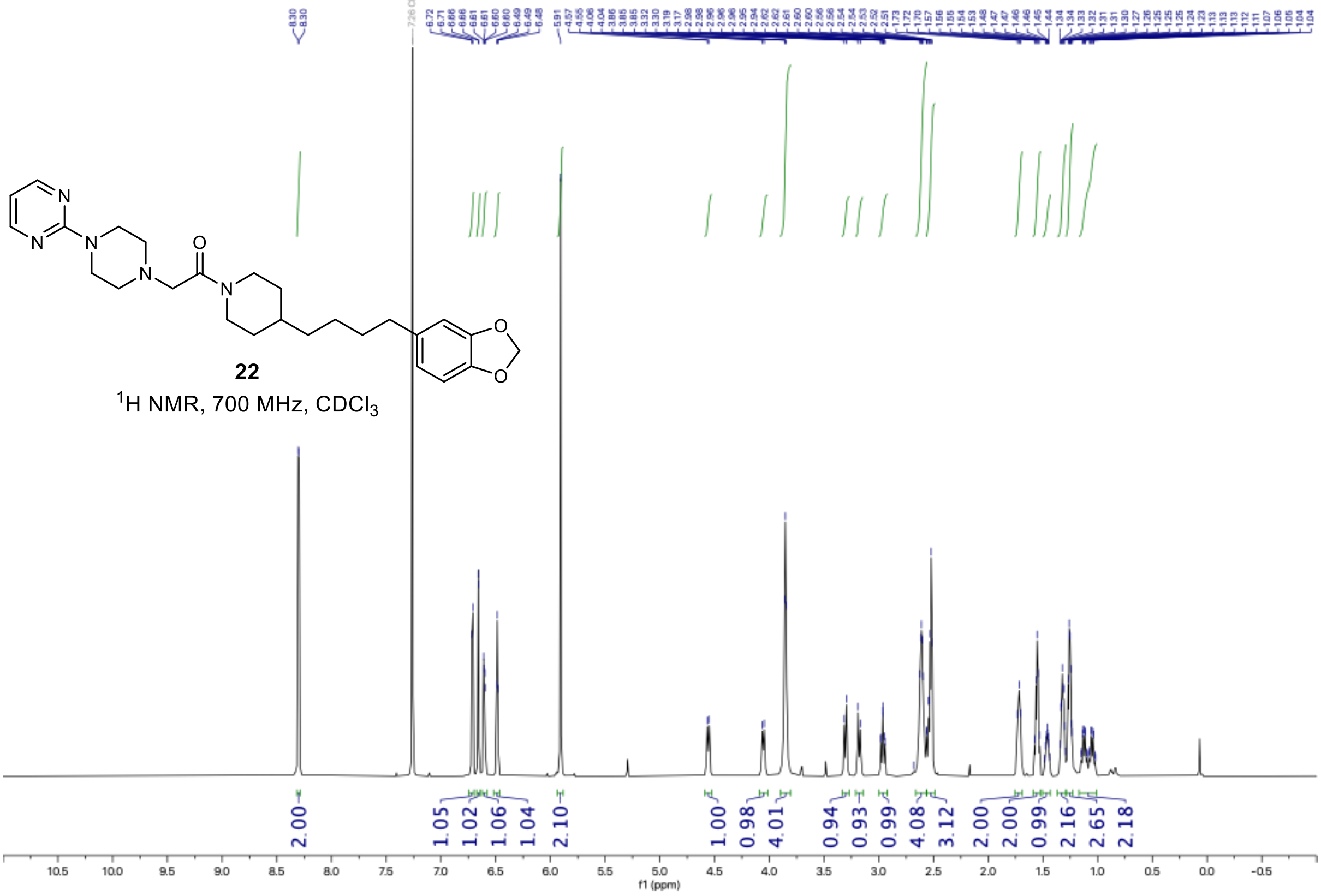

22

^{1}H NMR, 700 MHz, $CDCl_3$

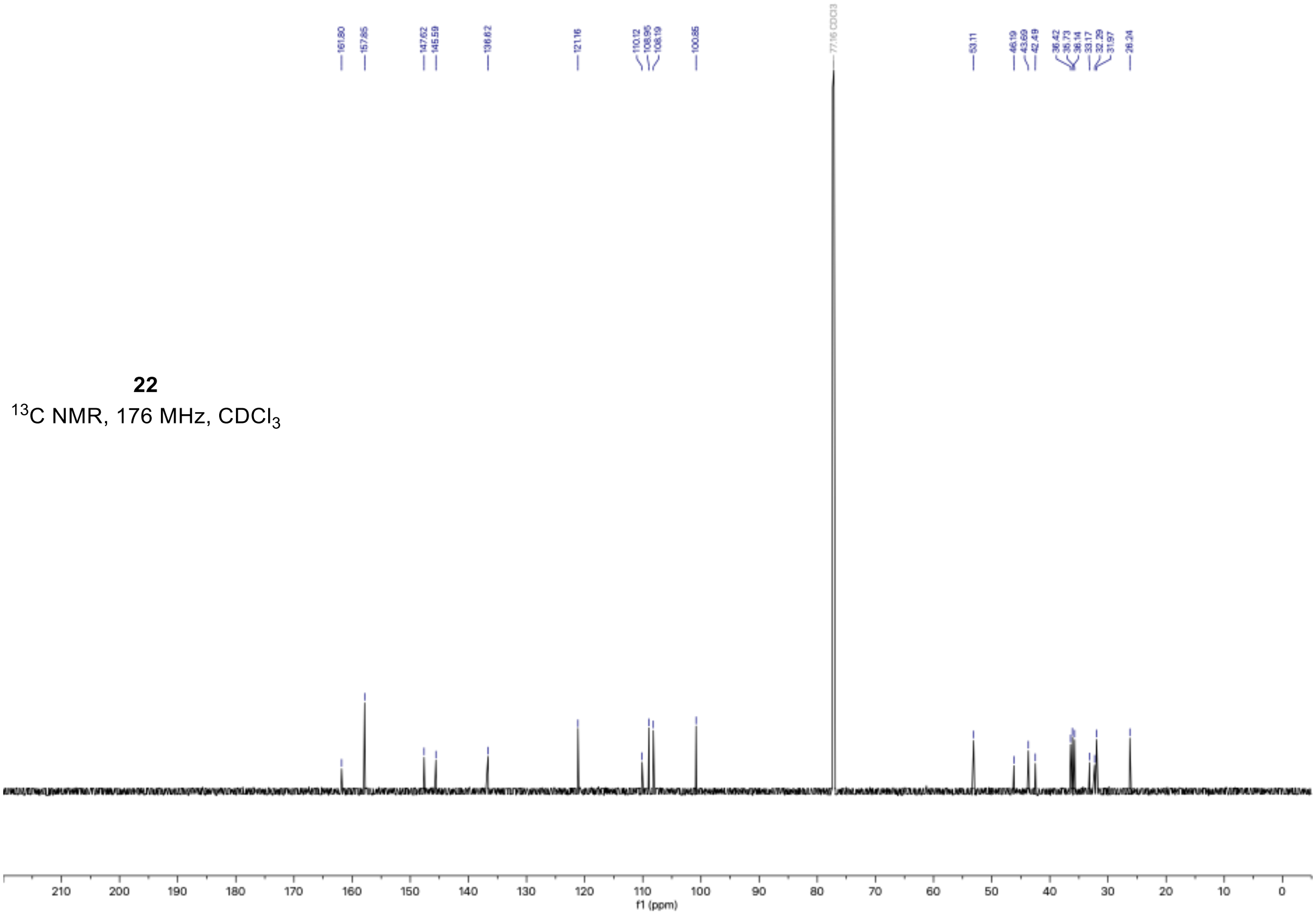

22

^{13}C NMR, 176 MHz, $CDCl_3$

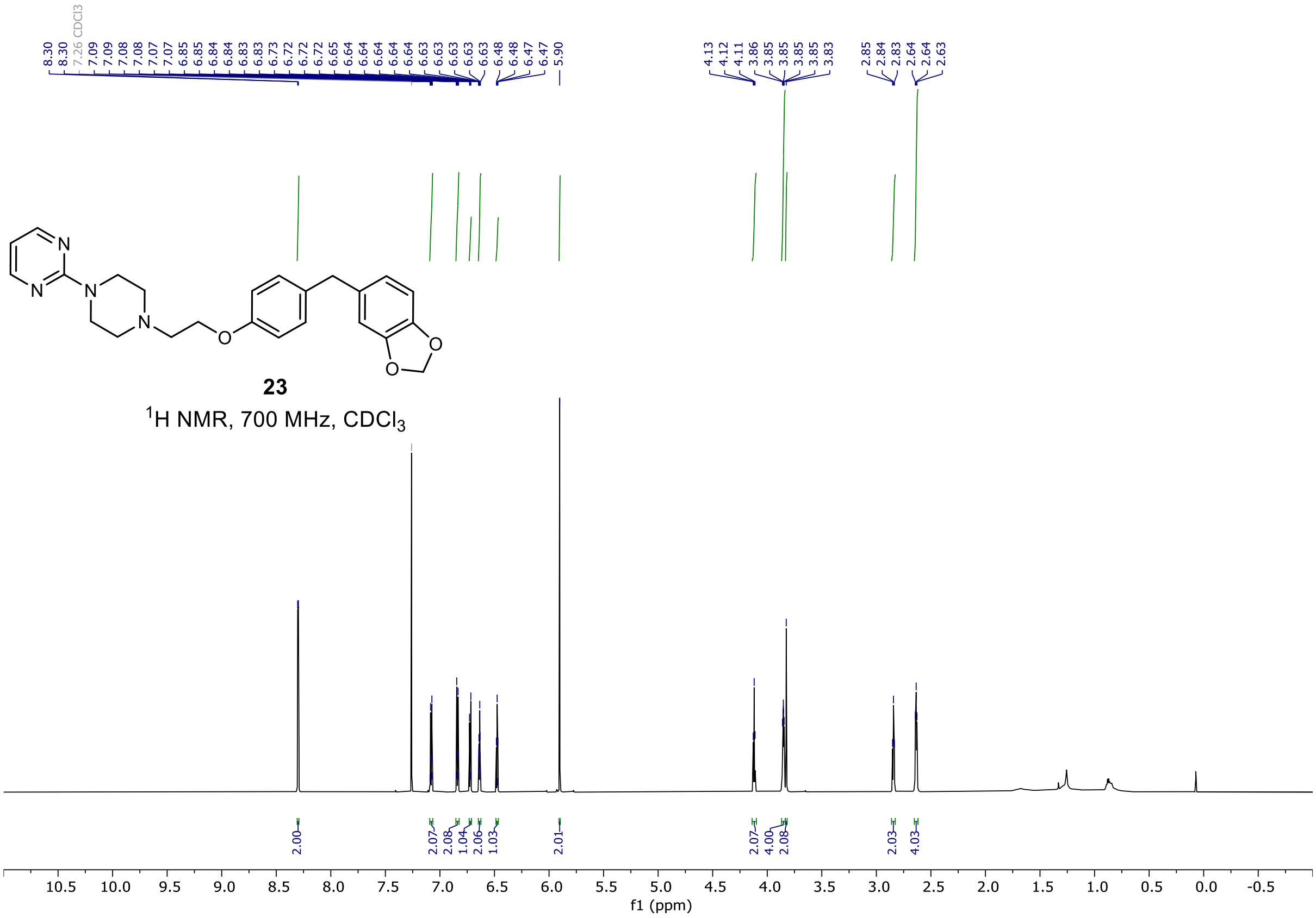

23

^{1}H NMR, 700 MHz, $CDCl_3$

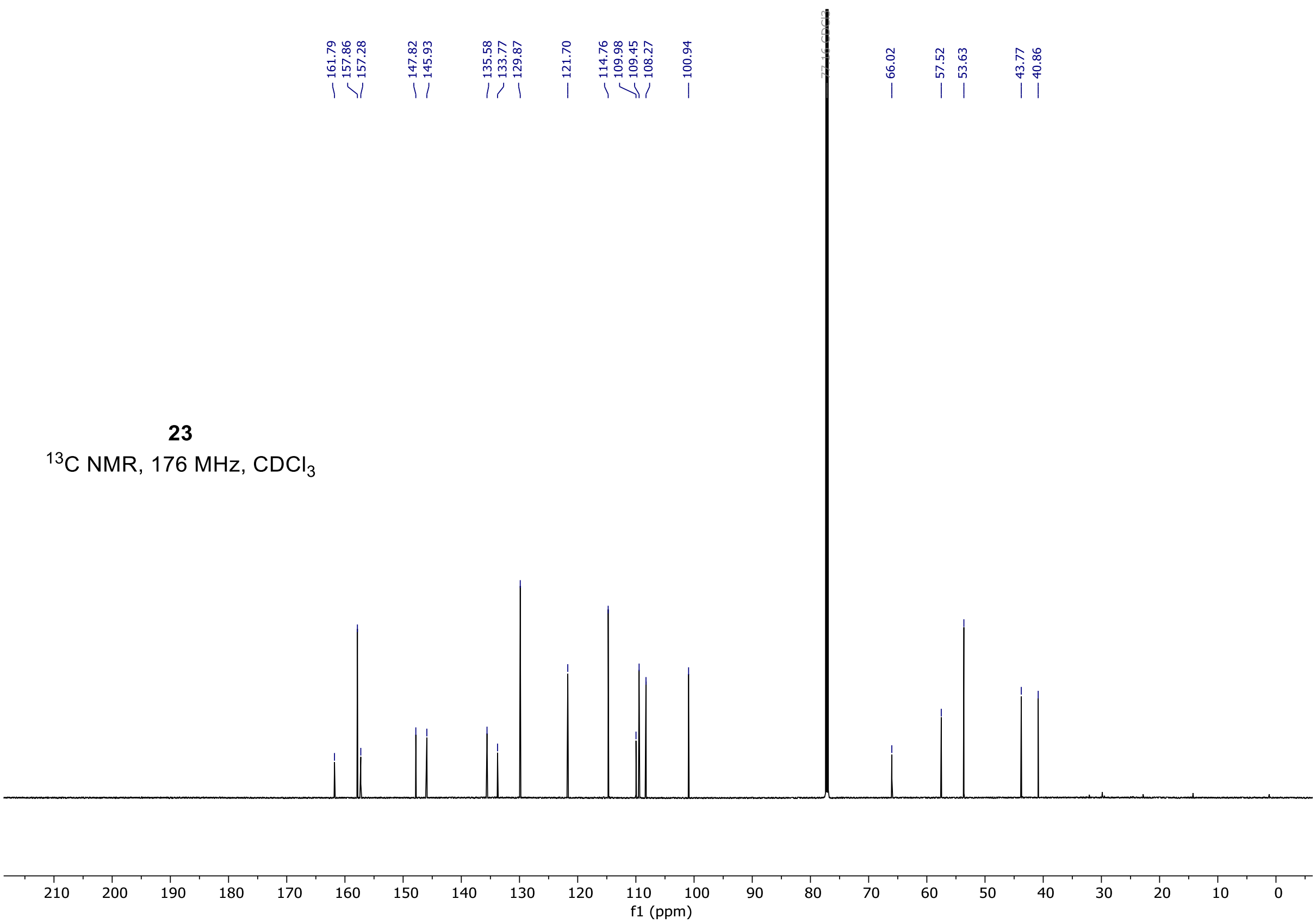

23

^{13}C NMR, 176 MHz, $CDCl_3$

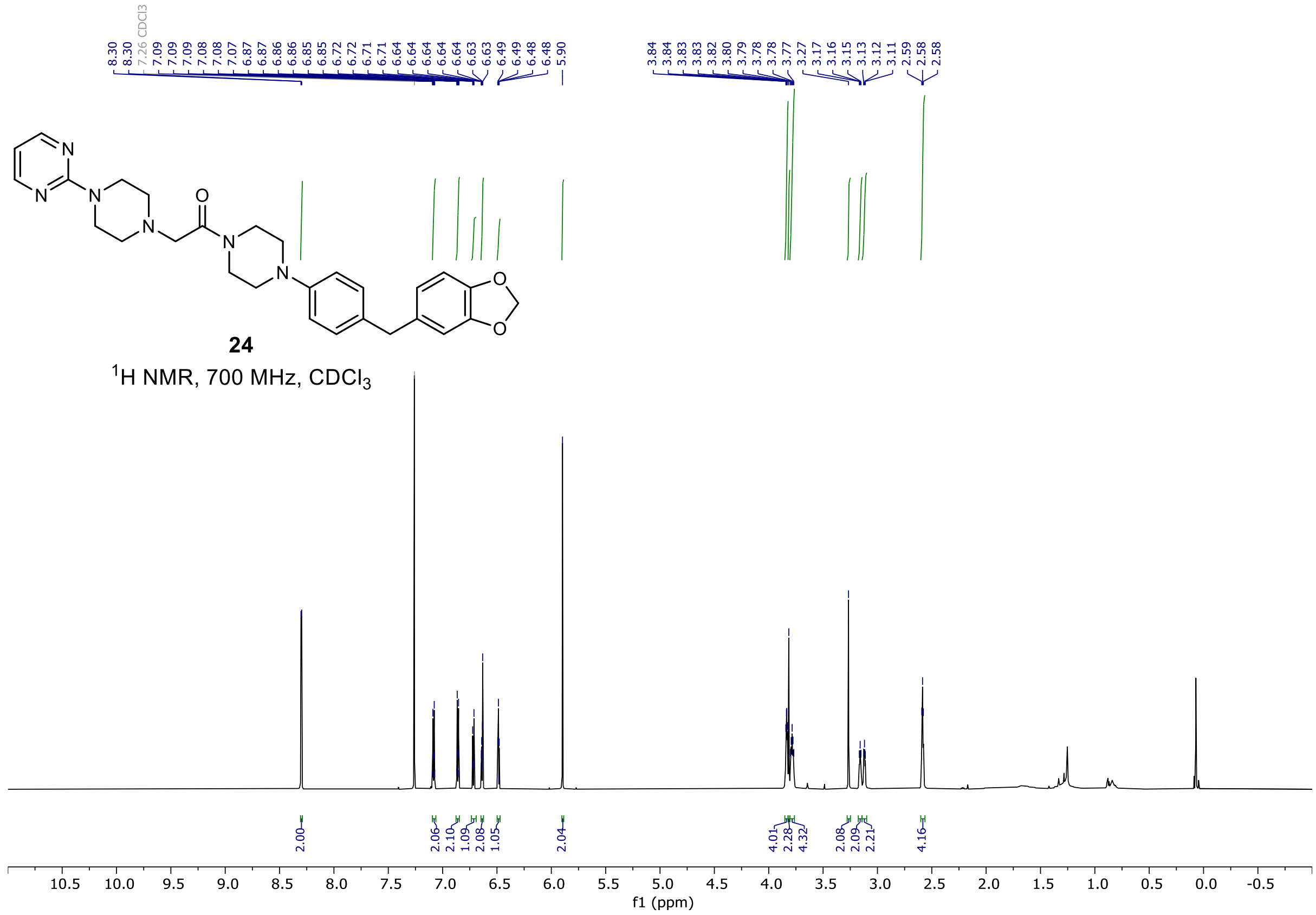

24

^{1}H NMR, 700 MHz, $CDCl_3$

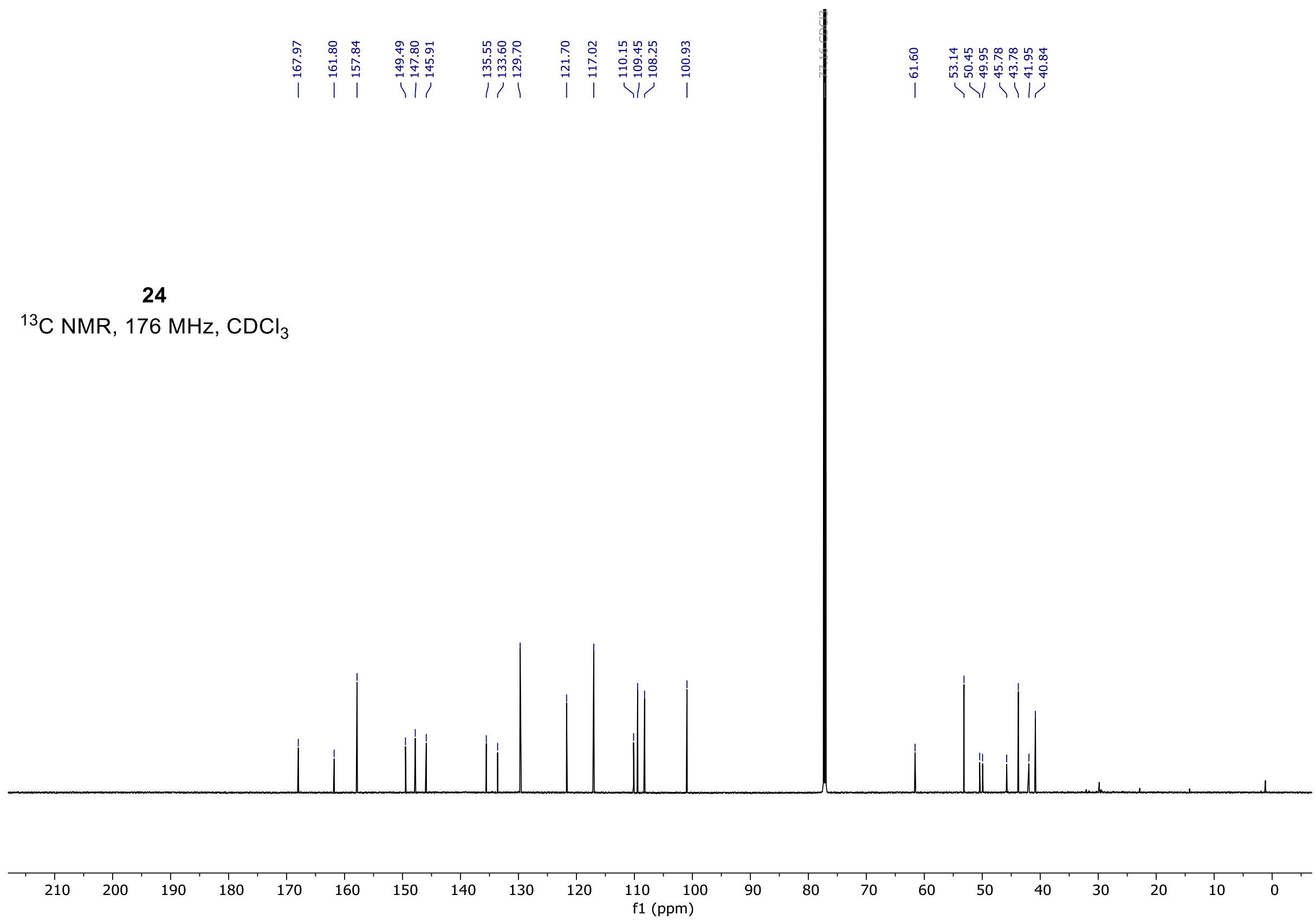

24

^{13}C NMR, 176 MHz, $CDCl_3$

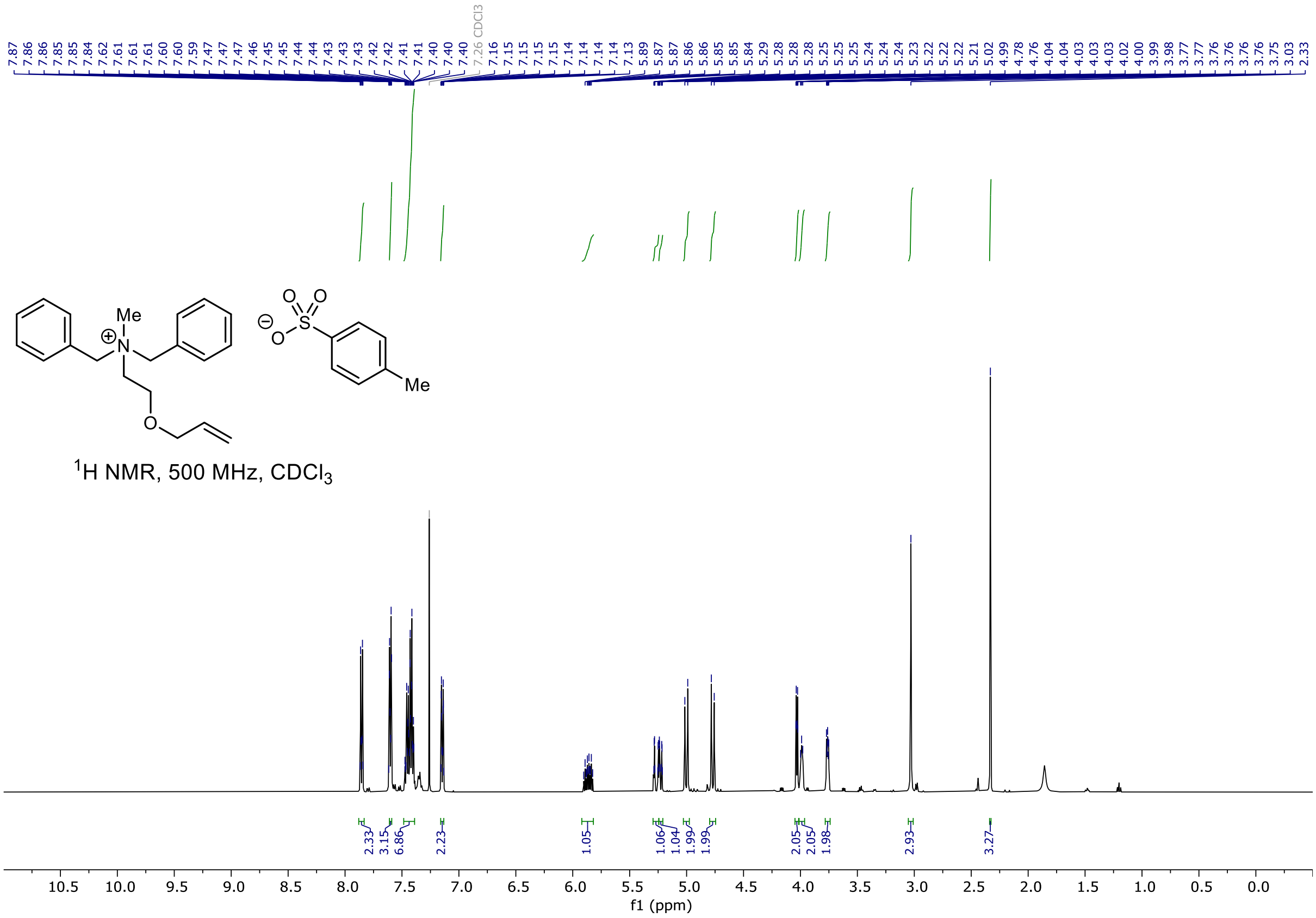

^{1}H NMR, 500 MHz, $CDCl_3$

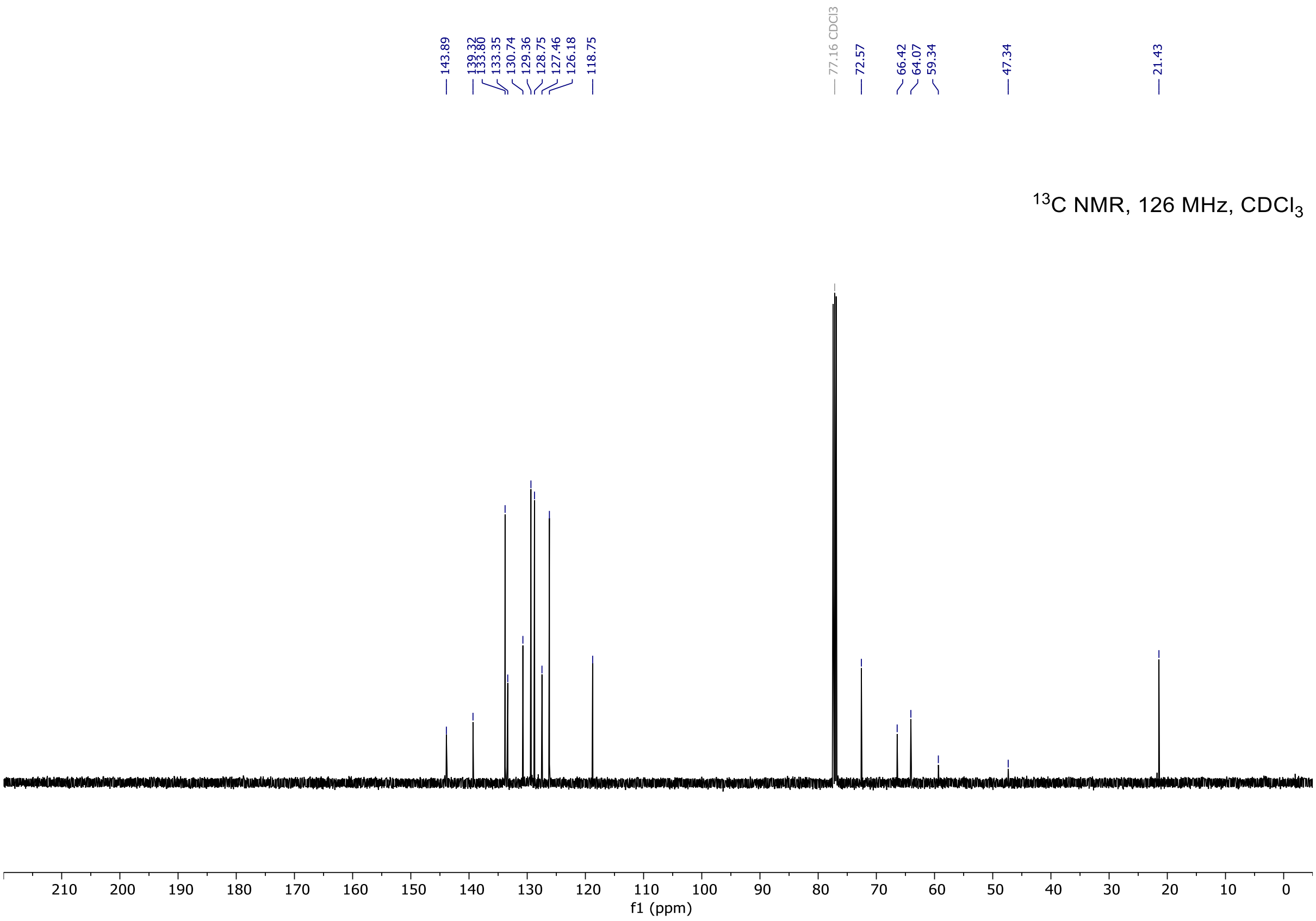

^{13}C NMR, 126 MHz, $CDCl_3$

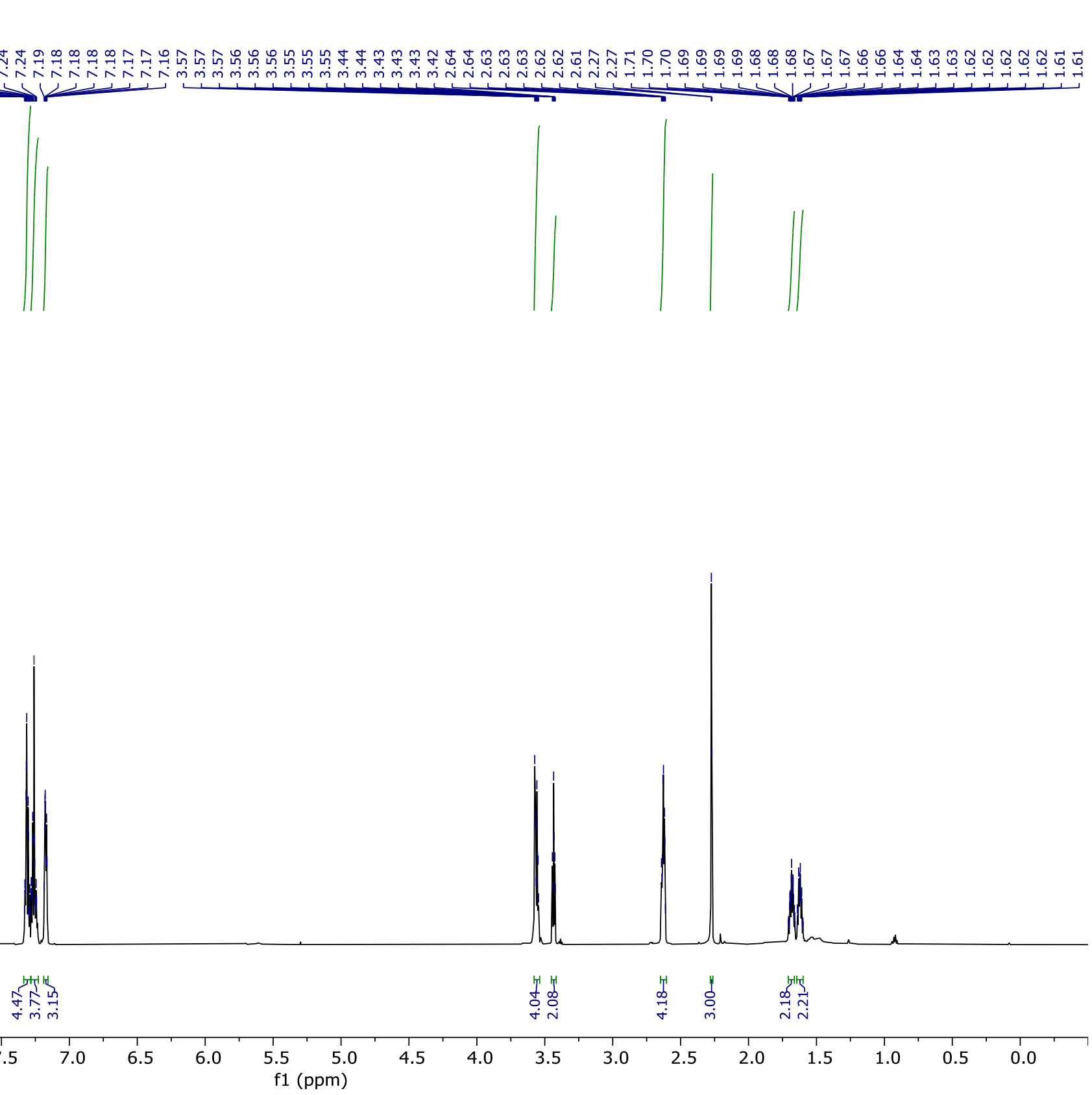

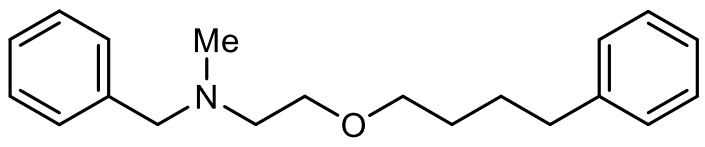

25

^{1}H NMR, 700 MHz, $CDCl_3$

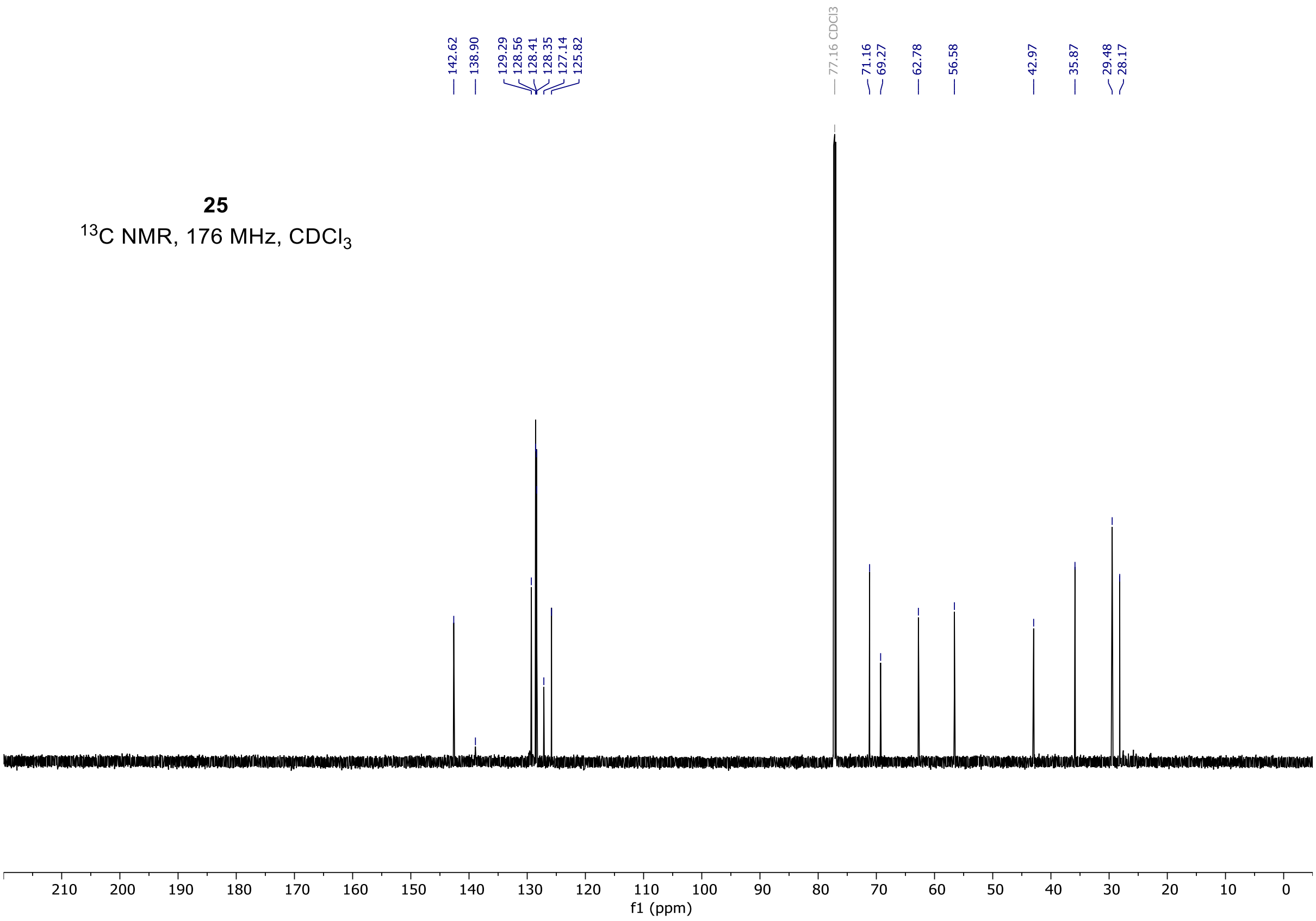

25

^{13}C NMR, 176 MHz, $CDCl_3$

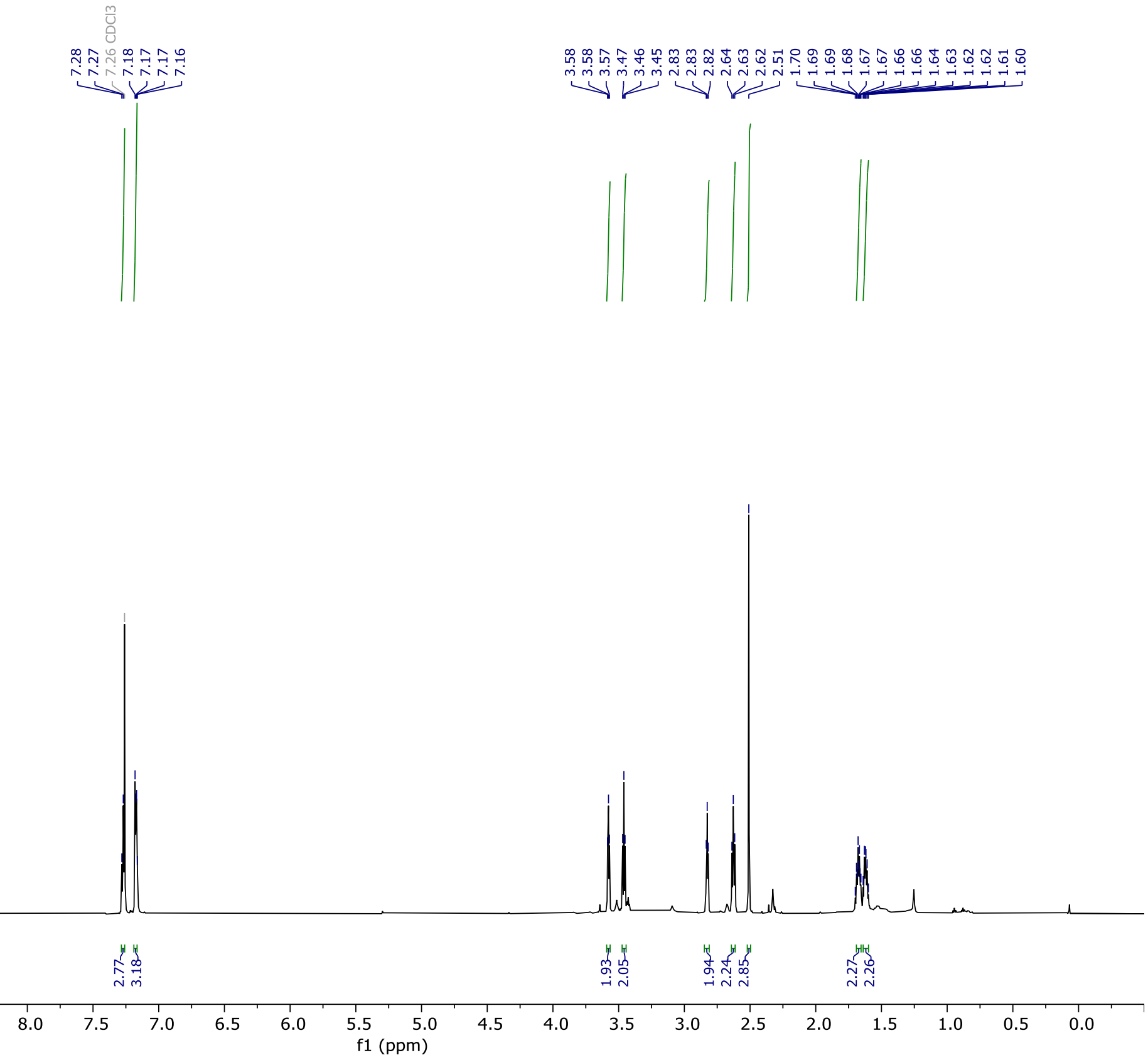

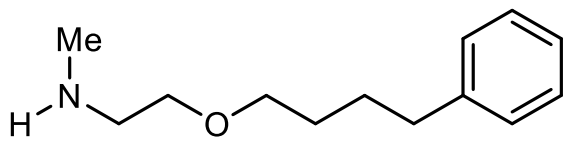

26

^{1}H NMR, 700 MHz, $CDCl_3$

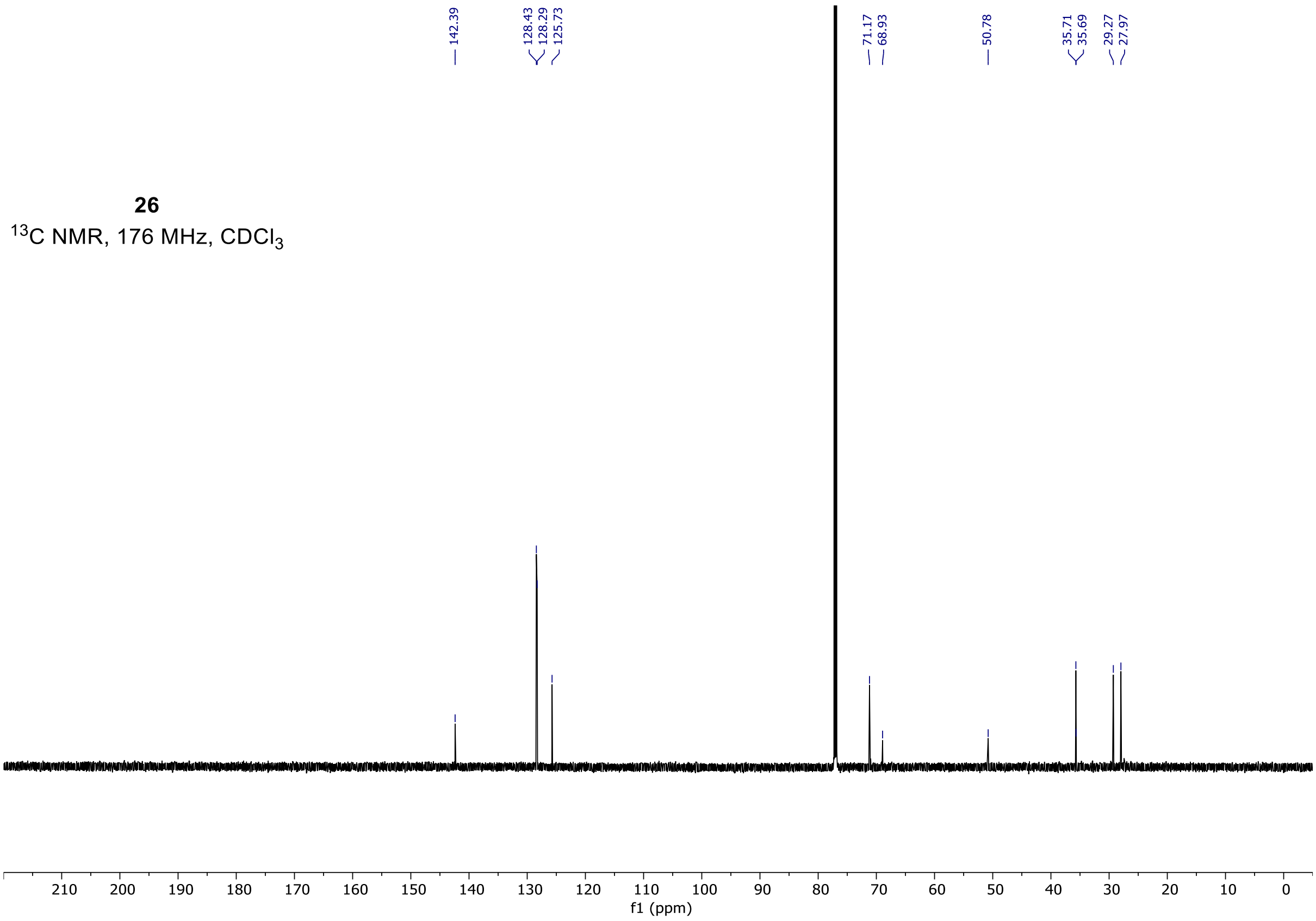

26

^{13}C NMR, 176 MHz, $CDCl_3$

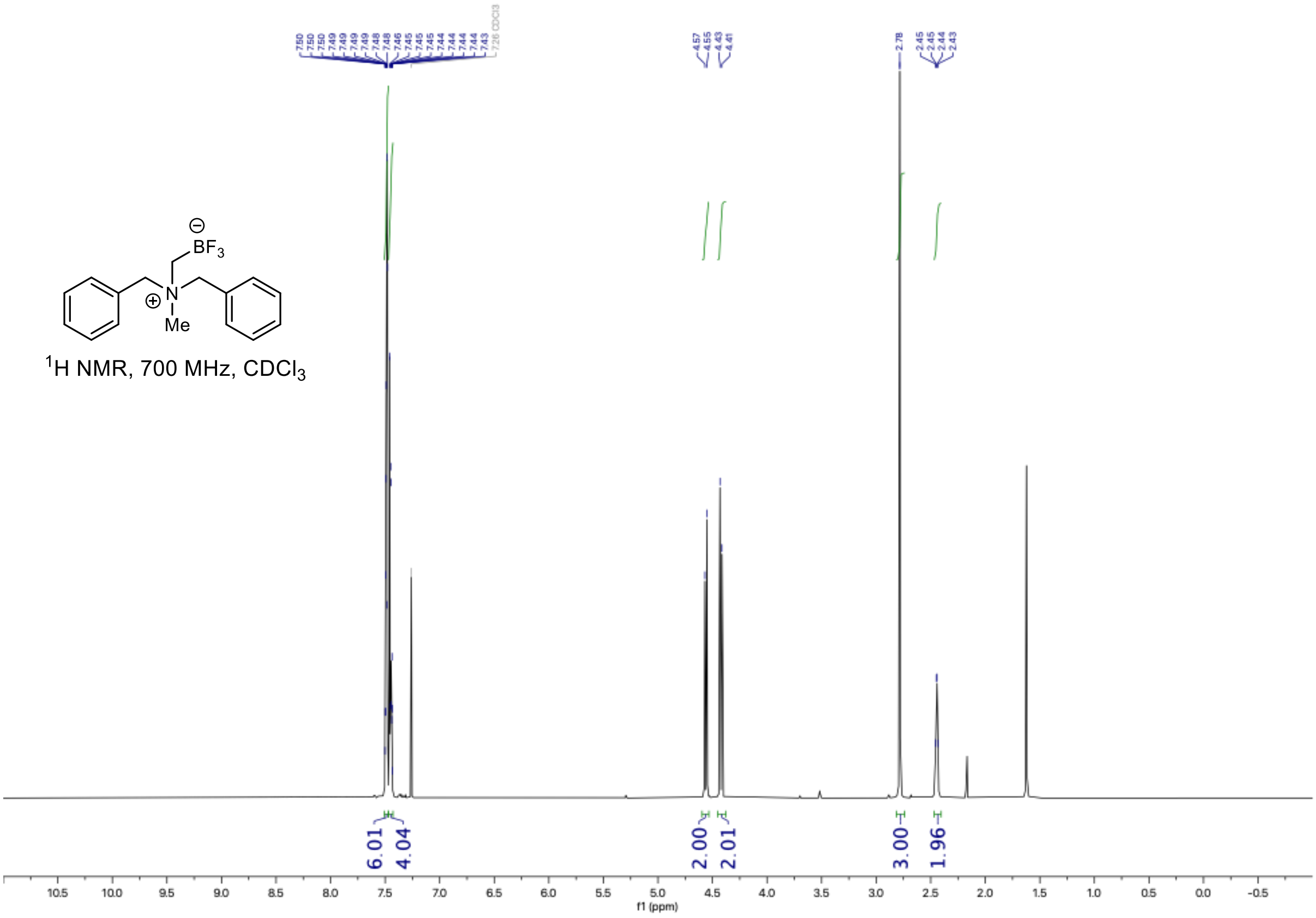

^{1}H NMR, 700 MHz, $CDCl_3$

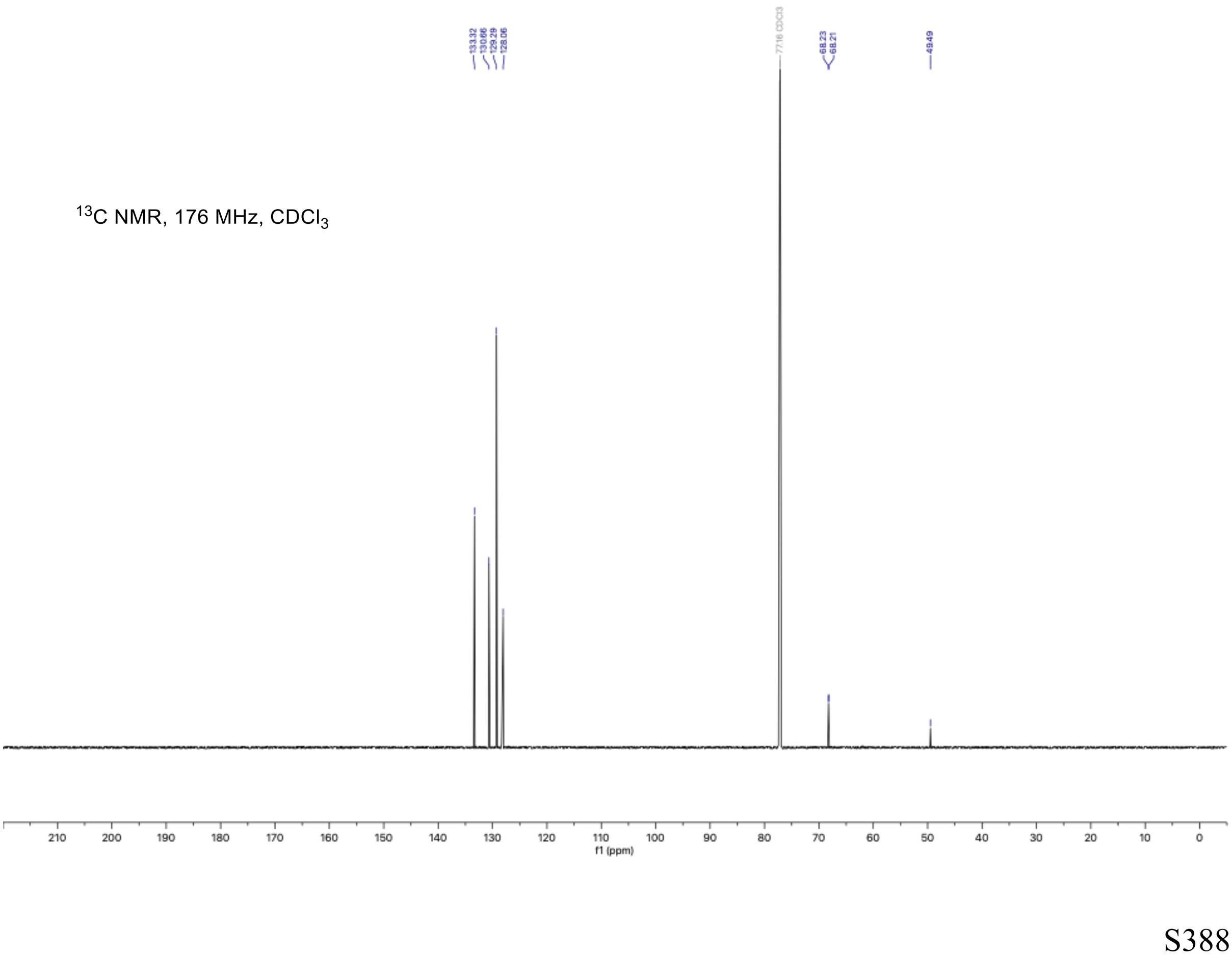

^{13}C NMR, 176 MHz, $CDCl_3$

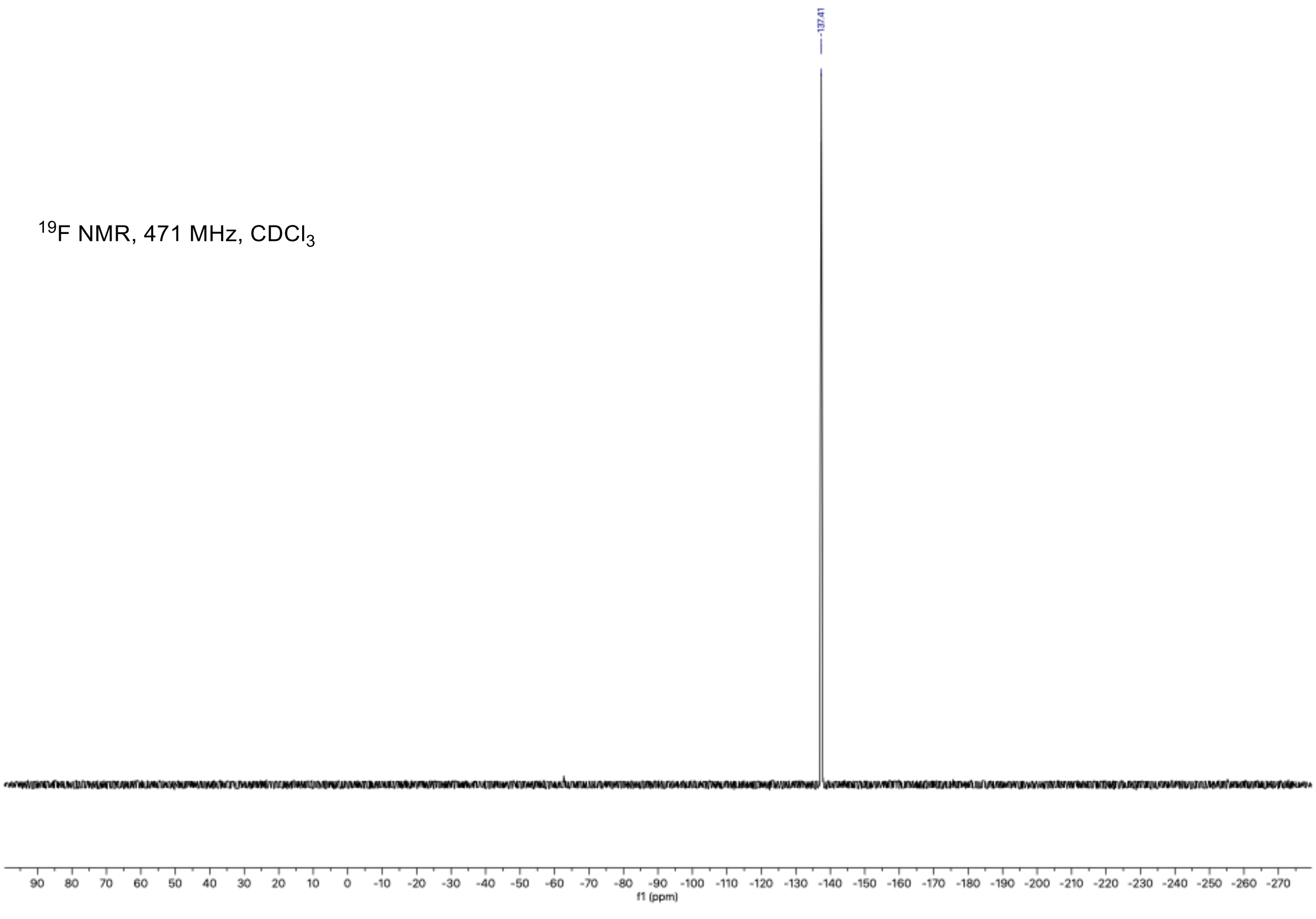
-137.41
^{19}F NMR, 471 MHz, CDCl$_3$
90 80 70 60 50 40 30 20 10 0 -10 -20 -30 -40 -50 -60 -70 -80 -90 -100 -110 -120 -130 -140 -150 -160 -170 -180 -190 -200 -210 -220 -230 -240 -250 -260 -270
f1 (ppm)

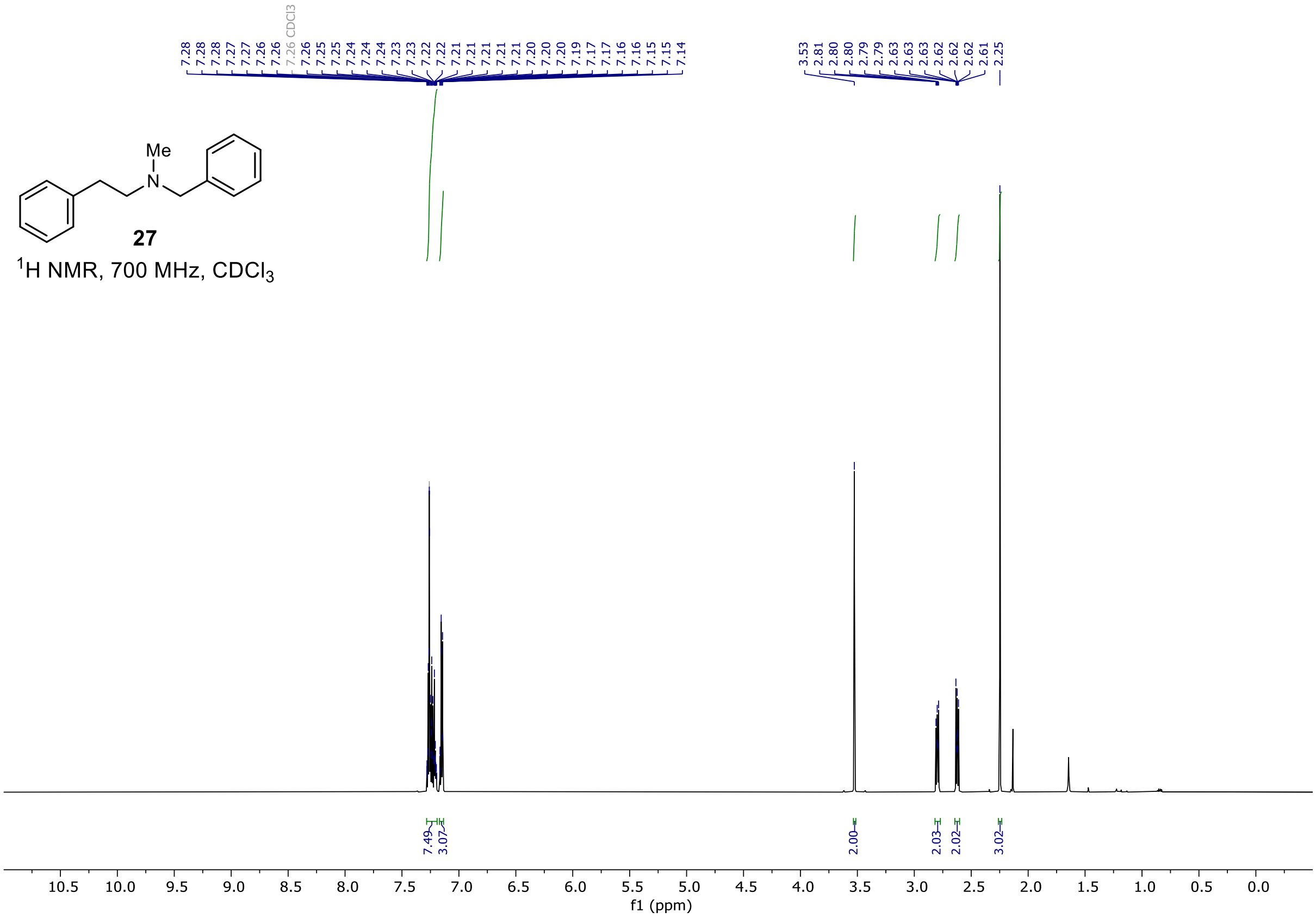

27

^1H NMR, 700 MHz, $CDCl_3$

27

^{13}C NMR, 176 MHz, $CDCl_3$

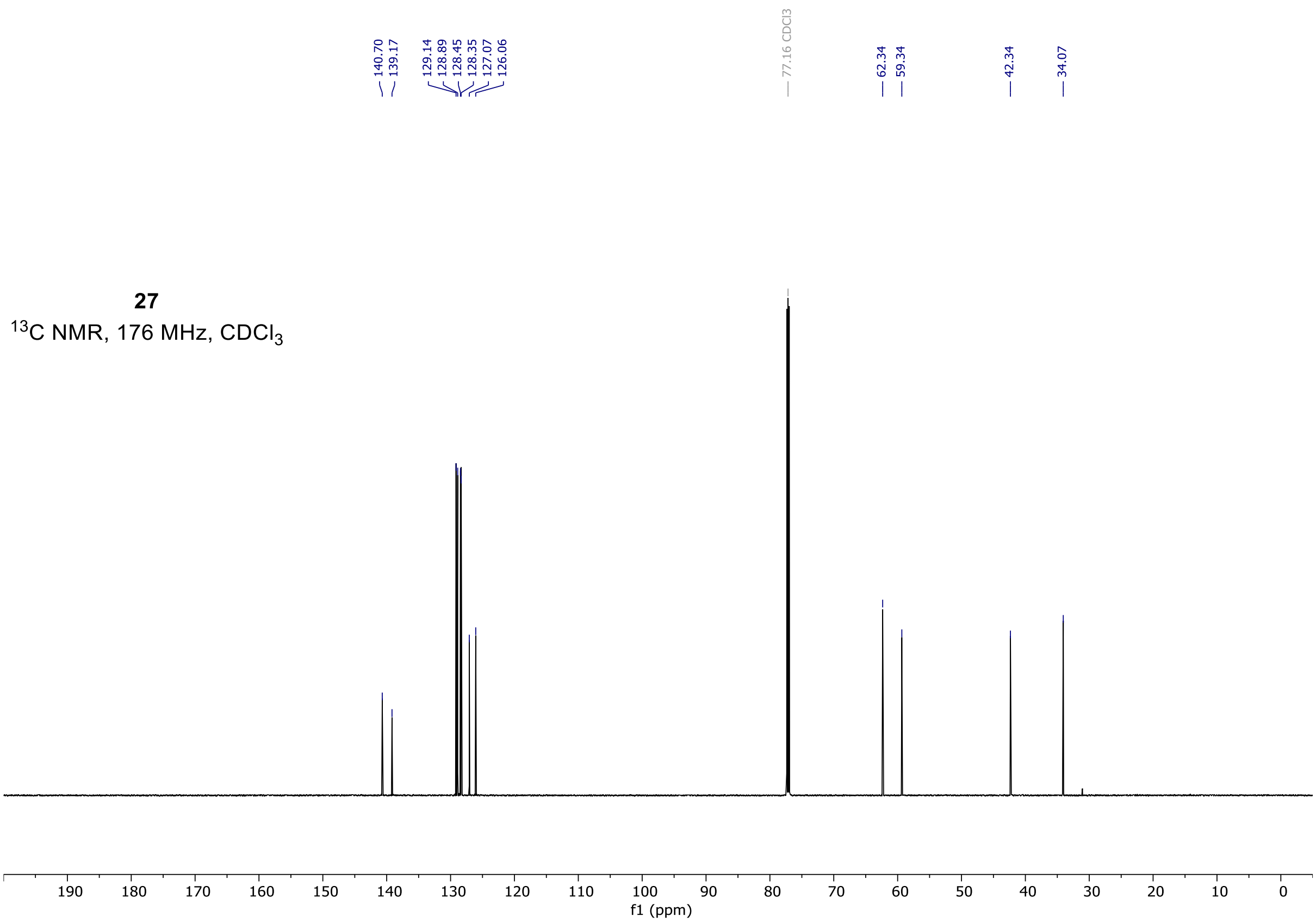

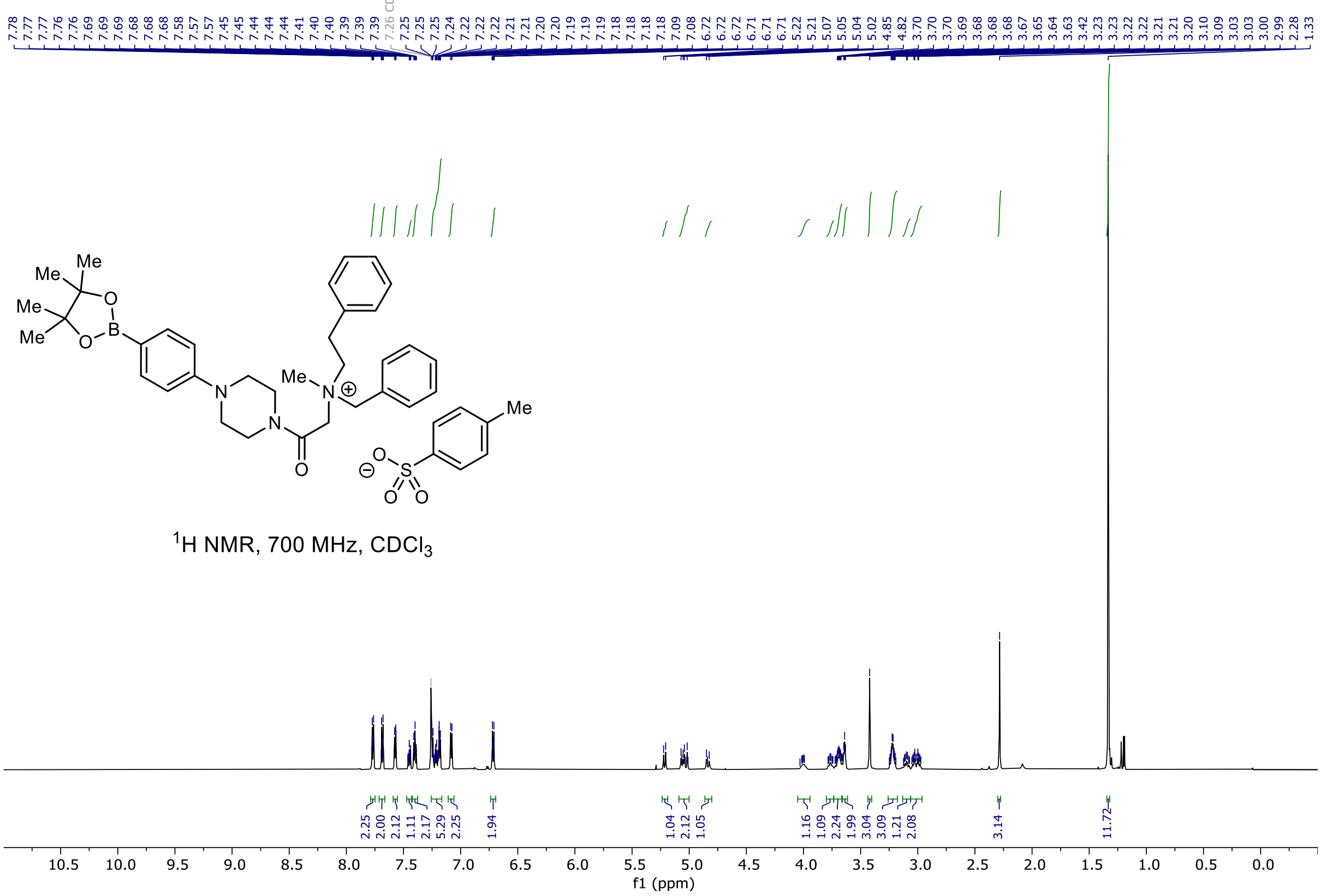
7.78 7.77 7.77 7.76 7.69 7.69 7.68 7.68 7.58 7.57 7.57 7.45 7.44 7.44 7.41 7.40 7.39 7.39 7.39 7.26 CDCl3 7.25 7.25 7.24 7.22 7.22 7.22 7.21 7.20 7.19 7.19 7.18 7.18 7.18 7.09 7.08 6.72 6.72 6.72 6.71 6.71 5.22 5.21 5.07 5.05 5.04 5.02 4.85 4.82 3.70 3.70 3.69 3.68 3.68 3.67 3.65 3.64 3.63 3.42 3.23 3.23 3.22 3.22 3.21 3.21 3.20 3.10 3.09 3.03 3.03 3.00 2.99 2.28 1.33
Me
Me
Me
Me
O
B
O
N
N
O
Me
N
Me
O
S
O
O
1H NMR, 700 MHz, CDCl3
2.25 2.00 2.12 1.11 2.17 5.29 2.25 1.94 1.04 2.12 1.05 1.16 1.09 2.24 1.99 3.04 3.09 1.21 2.08 3.14 11.72
10.5 10.0 9.5 9.0 8.5 8.0 7.5 7.0 6.5 6.0 5.5 5.0 4.5 4.0 3.5 3.0 2.5 2.0 1.5 1.0 0.5 0.0
f1 (ppm)

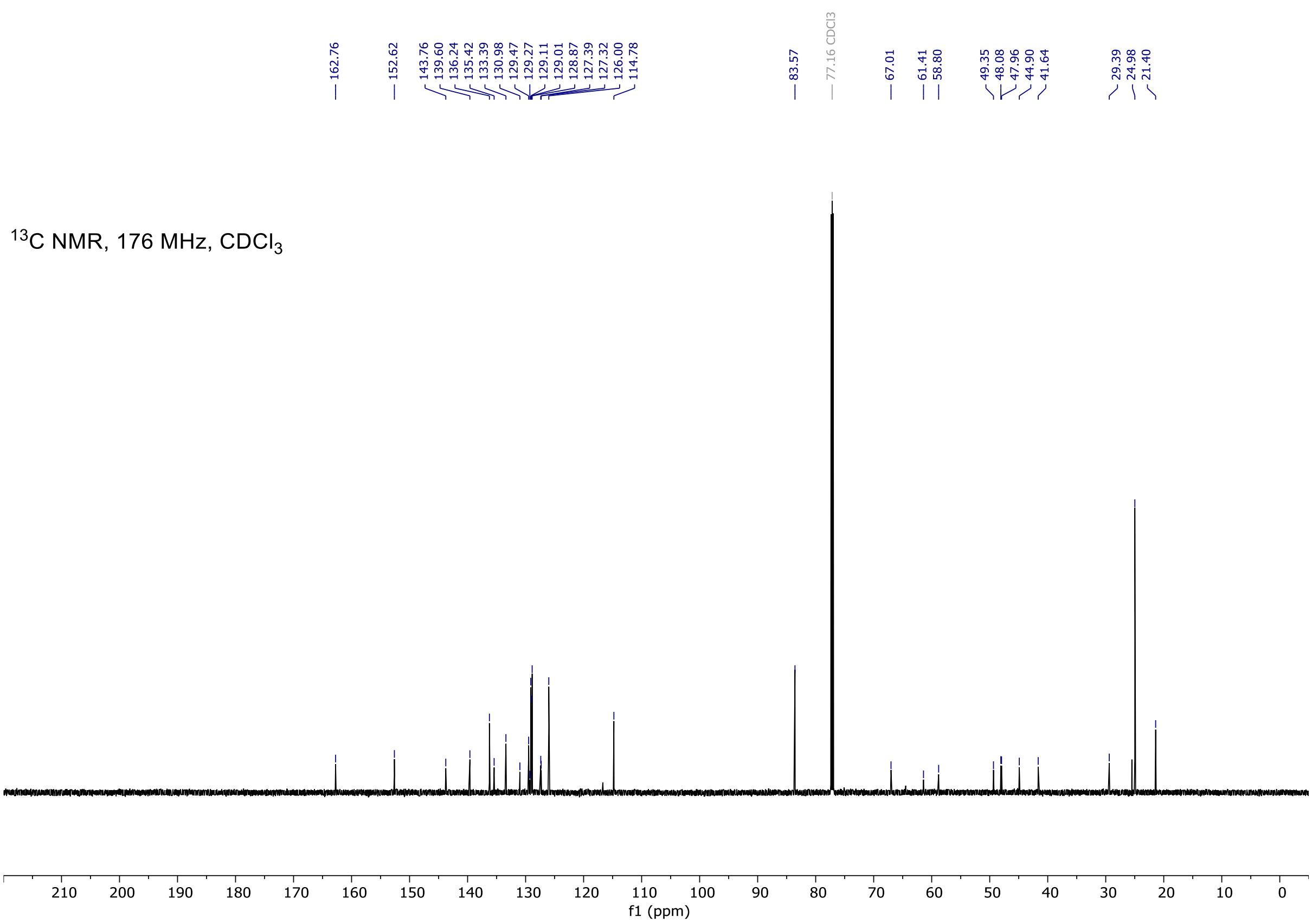
162.76 152.62 143.76 139.60 136.24 135.42 133.39 130.98 129.47 129.27 129.11 129.01 128.87 127.39 127.32 126.00 114.78 83.57 77.16 CDCl3 67.01 61.41 58.80 49.35 48.08 47.96 44.90 41.64 29.39 24.98 21.40
13C NMR, 176 MHz, CDCl3
210 200 190 180 170 160 150 140 130 120 110 100 90 80 70 60 50 40 30 20 10 0
f1 (ppm)

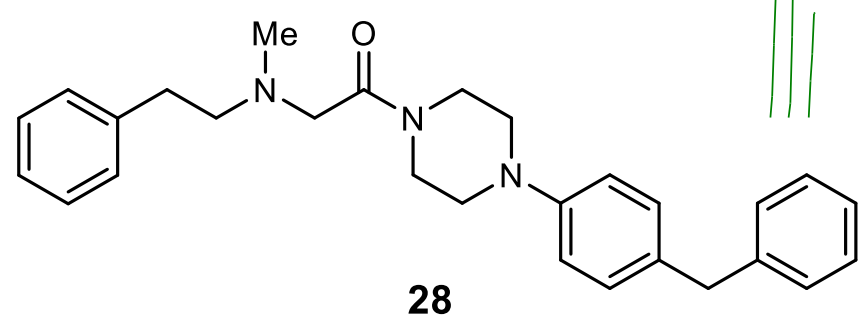

28

^{1}H NMR, 700 MHz, $CDCl_3$

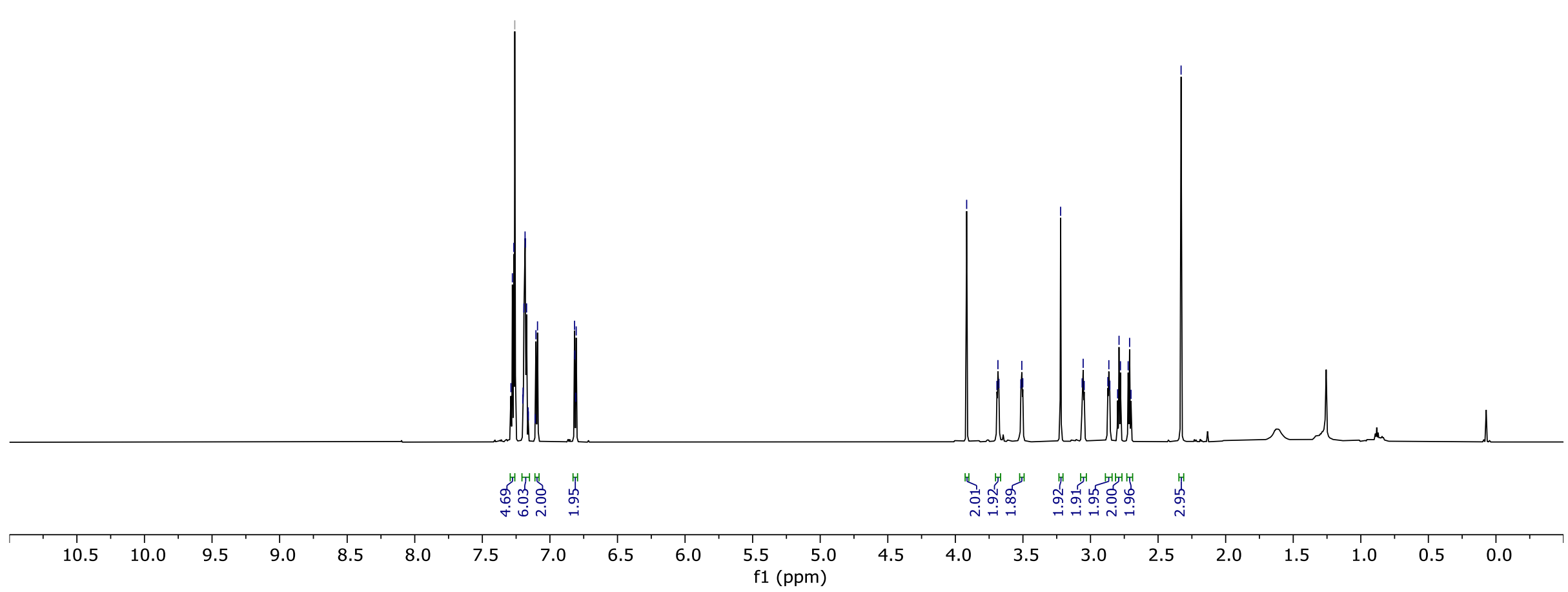

28

^{13}C NMR, 176 MHz, $CDCl_3$

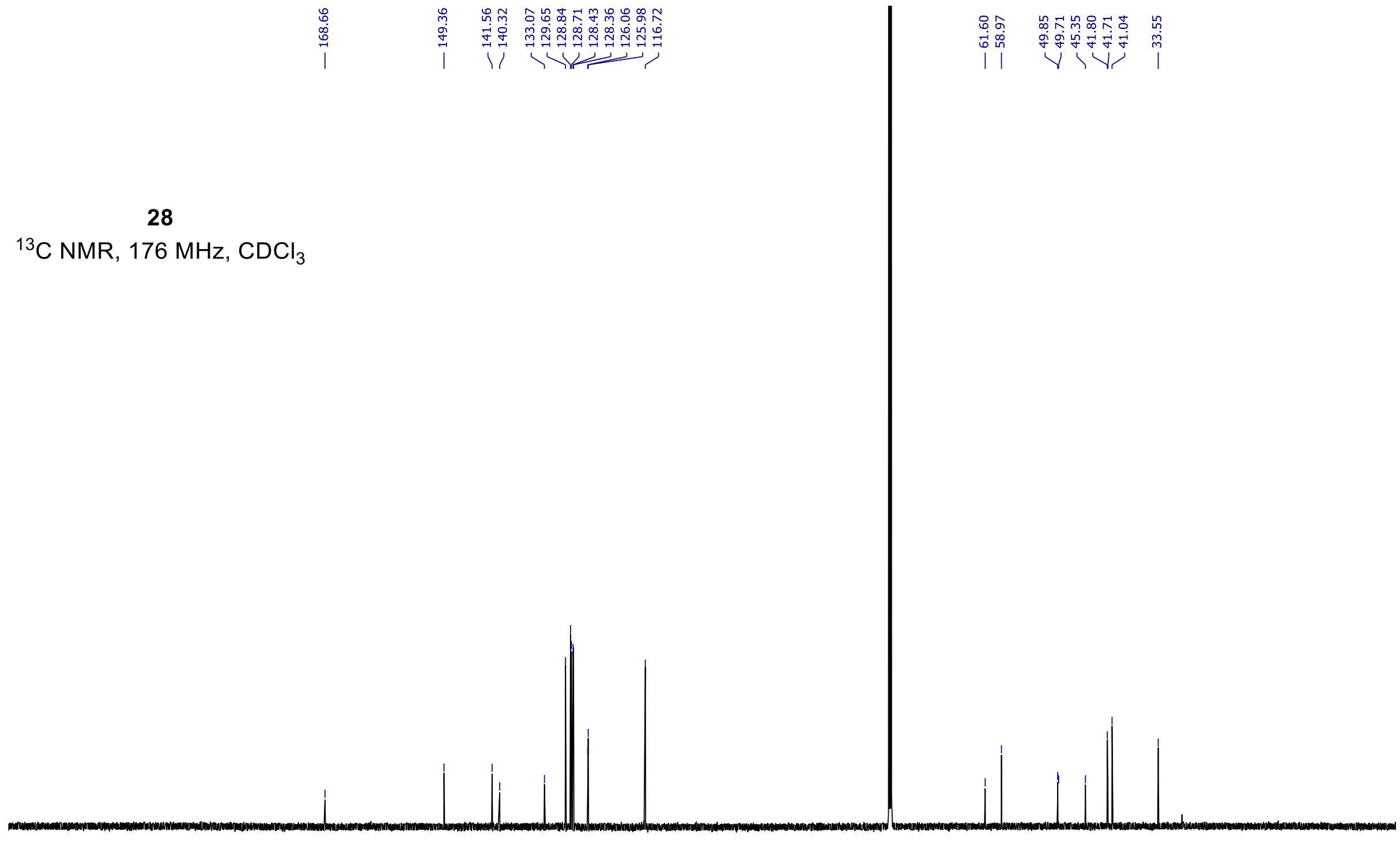

5. References

51. Vinayagam, V., Sadhukhan, S.D., Karre, S.K., Srinath, R., Maroju, R.K., Karra, P.R., Bathula, H.S.N.B., Kundrapu, S., Surukonti, S.R. TBAT-Catalyzed Deoxygenative Reduction of Tertiary Amides to Amines. *Org. Lett.* **25**, 4610-4614 (2023).

52. Kovalenko, O.O., Volkov, A., Adolfsson, H. Mild and Selective Et_2Zn-Catalyzed Reduction of Tertiary Amides under Hydrosilylation Conditions. *Org. Lett.* **17**, 446-449 (2015).

53. Huang, J., Xu, W., Xie, H., Li, S. One-Step Cyclization: Synthesis of *N*-Heteroalkyl-*N'*-tosylpiperazines. *J. Org. Chem.* **77**, 7506-7511 (2012).

54. Wang, T., He, F., Jiang, W., Liu, J. Electrohydrogenation of Nitriles with Amines by Cobalt Catalysis. *Angew. Chem. Int. Ed.* **63**, e202316140 (2024).

55. Lutz, M.D.R., Gasser, V.C.M., Morandi, B. Shuttle arylation by Rh(I) catalyzed reversible carbon-carbon bond activation of unstrained alcohols. *Chem.* **7**, 1108-1119 (2021).

56. van der Waals, D., Pettman, A., Williams, J.M.J. Copper-catalysed reductive amination of nitriles and organic-group reductions using dimethylamine borane. *RSC Adv.* **4**, 51845- 51849 (2014).

57. Weinhold, T.D., Law, J.A., Frederich, J.H. Access to 3-Azetidines via Halogenation of Titanacyclobutanes. *Adv. Synth. Catal.* **366**, 2214-2219 (2024).

58. Palkowitz, M.D., Tan, B., Hu, H., Roth, K., Bauer, R.A. Synthesis of Diverse *N*-Acryloyl Azetidines and Evaluation of Their Enhanced Thiol Reactivities. *Org. Lett.* **19**, 2270- 2273 (2017).

59. Chang, Y., Yesilcimen, A., Cao, M., Zhang, Y., Zhang, B., Chan, J.Z., Wasa, M. Catalytic Deuterium Incorporation within Metabolically Stable β-Amino C–H Bonds of Drug Molecules. *J. Am. Chem. Soc.* **141**, 14570-14575 (2019).

60. Itoh, T., Matsueda, T., Shimizu, Y., Kanai, M. Copper-Catalyzed Oxyboration of Unactivated Alkenes. *Chem. Eur. J.* **21**, 15955-15959 (2015).

61. Zhao, Y., Jin, J., Chan, P.W.H. Gold Catalyzed Photoredox C1-Alkynylation of *N*-Alkyl-1,2,3,4-tetrahydroisoquinolines by 1-Bromoalkynes with UVA LED Light. *Adv. Synth. Catal.* **361**, 1313-1321 (2019).

62. Pumphrey, A.L., Dong, H., Driver, T.G. Rh^{II}_2-Catalyzed Synthesis of α-, β-, or δ-Carbolines from Aryl Azides. *Angew. Chem. Int. Ed.* **51**, 5920-5923 (2012).

63. Dang, N., Martin, J.S., Shaikh, M., Yan, Y. Ni-Doped Perovskite for Photocatalytic Benzylic C–H Amination. *J. Am. Chem. Soc.* **147**, 17273-17280 (2025).
64. Li, Q., Liskey, C.W., Hartwig, J.F. Regioselective Borylation of the C–H Bonds in Alkylamines and Alkyl Ethers. Observation and Origin of High Reactivity of Primary C– H Bonds Beta to Nitrogen and Oxygen. *J. Am. Chem. Soc.* **136**, 8755-8765 (2014).
65. Seo, H., Katcher, M.H., Jamison, T.F. Photoredox activation of carbon dioxide for amino acid synthesis in continuous flow. *Nat. Chem.* **9**, 453-456 (2017).
66. Chen, W., Zhang, C., Shao, L. Base-promoted N-alkylation using formamides as the *N*-sources in neat water. *Tetrahedron* **70**, 880-885 (2014).
67. Das, S., Addis, D., Zhou, S., Junge, K., Beller, M. Zinc-Catalyzed Reduction of Amides: Unprecedented Selectivity and Functional Group Tolerance. *J. Am. Chem. Soc.* **132**, 1770- 1771 (2010).
68. Evers, B., Brennan, P.E. *N*-substituted-3,4-(fused 5-ring)-5-phenyl-pyrrolidine-2-one compounds as inhibitors of isoQC and/or QC enzyme. World Intellectual Property Organization, WO 2022123039 A1, June 16, 2022.
69. Luo, Y., Yao, J., He, Y., Xu, C., Liu, D. Nickel-catalysed reductive C–N bond cross-coupling between aryl halides and *N*-chloroamides. *Org. Biomol. Chem.* **22**, 6955-6959 (2024).
70. Fabitha, K., Kallingal, A., Maciejewska, N., Haneefa P.T., H., Kundil, V.T., Alharbi, M., Banothu, J. Improved anti-breast cancer activity through structural modification of fused pyran derivatives and mechanistic investigations. *RSC Med. Chem.* **16**, 3671-3696 (2025).
71. Lou, S., Zhang, L., Luo, Y., Nishiura, M., Luo, G., Luo, Y., Hou, Z. Regiodivergent C–H Alkylation of Quinolines with Alkenes by Half-Sandwich Rare-Earth Catalysts. *J. Am. Chem. Soc.* **142**, 18128-18137 (2020).
72. Alam, R., Molander, G.A. Photoredox-catalyzed Direct Reductive Amination of Aldehydes without an External Hydrogen/Hydride Source. *Org. Lett.* **20**, 2680-2684 (2018).
73. Hayhow, T.G., Borrows, R.E.A., Diène, C.R., Fairley, G., Fallan, C., Fillery, S.M., Scott, J.S., Watson, D.W. A Buchwald-Hartwig Protocol to Enable Rapid Linker Exploration of Cereblon E3-Ligase PROTACs. *Chem. Eur. J.* **26**, 16818-16823 (2020).
74. Steiner, A., Krieger, J., Jones, R., Böse, D., Wang, Y., Eggenweiler, H., Williams, J.D., Kappe, C.O. Photoredox C_{sp}^3-C_{sp}^2 Reductive Cross-Couplings of Cereblon Ligands for PROTAC Linker Exploration in Batch and Flow. *ChemCatChem* **14**, e202201184 (2022).

75. Bonazzi, S., d'Hennezel, E., Beckwith, R.E.J., Xu, L., Fazal, A., Magracheva, A., Ramesh, R., Cernijenko, A., Antonakos, B., Bhang, H.C., Caro, R.G., Cobb, J.S., Orneleas, E., Ma, X., Wartchow, C.A., Clifton, M.C., Forseth, R.R., Fortnam, B.H., Lu, H., Csibi, A., Tullai, J., Carbonneau, S., Thomsen, N.M., Larrow, J., Chie-Leon, B., Hainzl, D., Gu, Y., Lu, D., Meyer, M.J., Alexander, D., Kinyamu-Akunda, J., Sabatos-Peyton, C.A., Dales, N.A., Zécri, F.J., Jain, R.K., Shulok, J., Wang, Y.K., Briner, K., Porter, J.A., Tallarico, J.A., Engelman, J.A., Dranoff, G., Bradner, J.E., Visser, M., Solomon, J.M. Discovery and characterization of a selective IKZF2 glue degrader for cancer immunotherapy. *Cell Chem. Biol.* **30**, 235-247.E12 (2023).

76. Liu, Y., Chen, Z., Li, Y., Qian, J., Li, Q., Wang, H. Boryl-Dictated Site-Selective Intermolecular Allylic and Propargylic C–H Amination. *J. Am. Chem. Soc.* **31**, 14380- 14387 (2022).

77. Zhang, G., Favela, D., Chow, W.L., Iyer, R.N., Pell, A.J., Olson, D.E. Synthesis of Tertiary Amines through Extrusive Alkylation of Carbamates. *Org. Lett.* **24**, 6208-6212 (2022).

78. Paudel, S., Acharya, S., Yoon, G., Kim, K., Cheon, S.H. Design, synthesis and in vitro activity of 1,4-disubstituted piperazines and piperidines as triple reuptake inhibitors. *Bioorg. Med. Chem.* **25**, 2266-2276 (2017).

79. Utsunomiya, M., Hartwig, J.F. Ruthenium-Catalyzed Anti-Markovnikov Hydroamination of Vinylarenes. *J. Am. Chem. Soc.* **126**, 2702-2703 (2004).

80. Imberdis, T., Heeres, J.T., Yueh, H., Fang, C., Zhen, J., Rich, C.B., Glicksman, M., Beeler, A.B., Harris, D.A. Identification of Anti-prion Compounds using a Novel Cellular Assay. *J. Biol. Chem.* **291**, 26164-26167 (2016).

81. Barrett, A.G.M., Brinkmann, C., Crimmin, M.R., Hill, M.S., Hunt, P., Procopiou, P.A. Heavier Group 2 Metals and Intermolecular Hydroamination: A Computational and Synthetic Assessment. *J. Am. Chem. Soc.* **131**, 12906-12907 (2009).

82. Abe, T., Morimoto, Y., Tano, T., Mieda, K., Sugimoto, H., Fujieda, N., Ogura, T., Itoh, S., Geometric Control of Nuclearity in Copper(I)/Dioxygen Chemistry. *Inorg. Chem.* **53**, 8786-8794 (2014).

83. Peng, Y., Wang, G., Klare, H.F.T., Oestreich, M. Ring Contraction of Saturated Cyclic Amines and Rearrangement of Acyclic Amines Through Their Corresponding Hydroxylamines. *Angew. Chem. Int. Ed.* **63**, e202410483 (2024).

84. We, X., Zhang, M., Guo, Y., Chang, Q., Qiao, W. Design, synthesis, and biological evaluation of THIQ as antidepressive agents. *Bioorg. Med. Chem. Lett.* **95**, 129470 (2023).

85. Li, S., Rajeshkumar, T., Liu, J., Maron, L., Zhou, X. La-Catalyzed Decarbonylation of Formamides and Its Applications. *Org. Lett.* **25**, 163-168 (2023).

86. Wolfe, J.P., Wagaw, S., Buchwald, S.L. An Improved Catalyst System for Aromatic Carbon–Nitrogen Bond Formation: The Possible Involvement of Bis(Phosphine) Palladium Complexes as Key Intermediates. *J. Am. Chem. Soc.* **118**, 7215-7216 (1996).

87. Larouche-Gauthier, R., Elford, T.G., Aggarwal, V.K. Ate Complexes of Secondary Boronic Esters as Chiral Organometallic-Type Nucleophiles for Asymmetric Synthesis. *J. Am. Chem. Soc.* **133**, 16794-16797 (2011).

88. Zhang, X., Zhai, D., Liu, Y., Guan, B. A potassium magnesiate complex: Synthesis, structure and catalytic intermolecular hydroamination of styrenes. *J. Organomet. Chem.* **961**, 122254 (2022).

89. Beller, M., Trauthwein, H., Eichberger, M., Breindl, C., Herwig, J., Müller, T.E., Thiel, O.R. The First Rhodium-Catalyzed Anti-Markovnikov Hydroamination: Studies on Hydroamination and Oxidative Amination of Aromatic Olefins. *Chem. Eur. J.* **5**, 1306- 1319 (1999).

90. Romeiro, L.S., Fraga, C.M., Barreiro, E.D.J.D.L., da Silva, C.M., Miranda, A.P., Tibirica, E., de Brito, F.F., de Almeida, H.C.C., Noel, F. Use of Adrenergic *N*-Phenylpiperazine Antagonists, Pharmaceutical Compositions Containning Them, and Methods of Preparing Them. United States, US20070219213A1, September 20, 2007.

91. Spiske, M., Meyners, C., Bauder, M., Repity, M., Brudy, C., Sugiarto, W.O., Achaq, H., Geiger, T.M., Hausch, F. Conformationally Restricted Macrocycles as Improved FKBP51 Inhibitors Enabled by Systematic Linker Derivitization. *Angew. Chem. Int. Ed.* **64**, e202418511 (2025).

92. Clark, P.G., Day, M.W., Grubbs, R.H. Switching and Extension of a [c2]Daisy-Chain Polymer. *J. Am. Chem. Soc.* **131**, 13631-13633 (2009).

93. Germain, S., Schulz, E. and Hannedouche, J. Anti-Markovnikov Hydroamination of Aromatic Alkenes with Secondary Amines Catalyzed by Easily Accessible Yttrium Complexes. *ChemCatChem.* **6**, 2065-2073 (2014).
