# Supplementary material for: Programmable remodelling of carbon–nitrogen connectivity in amines
Source: Nature. 2026 Aug 17;658(8134):94–102. doi: 10.1038/s41586-026-11009-1 (PMC13626948; doi:10.1038/s41586-026-11009-1)
Supplement: Supplementary file 2 [file 41586_2026_11009_MOESM2_ESM.pdf]

nature portfolio

Peer Review File

Programmable remodelling of carbon–nitrogen connectivity in amines

Corresponding Author: Professor Matthew Gaunt

Any redactions in this file are there to maintain patient confidentiality, the confidentiality of unpublished data, or to remove third-party material.

This file contains all reviewer reports in order by version, followed by all author rebuttals in order by version.

Version 0:

Reviewer comments:

Referee #1

(Remarks to the Author)
"Programmable remodeling of carbon–nitrogen connectivity in amines" is reported by Gaunt and co-workers. The Cambridge team provided a novel and highly interesting strategy for the one-carbon homologation (CH2-moiety) of tertiary benzylic amines (major part of the paper). In the final part the strategy is further extended toward the introduction of larger fragments into the benzylic C-N bond in tertiary benzylic amines. Structural reorganization (editing) is currently a very hot field in modern synthetic chemistry. The current contribution is located in this area and should attract a large readership. The transformation is unprecedented (the advantage over the classical 1,2-Stevens rearrangement is well addressed in the manuscript) and the method is applicable at late stage in more complex systems. Importantly, the homologation works stereospecifically on phenethyl amines that are valuable and abundant structures (see product 2x). The homologative C-N remodeling with different linkers is also highly interesting to this referee. Yields are mostly good for these transformations and the potential of the method is convincingly demonstrated in Figure 5c. Taken together, this is a very nice piece of work that deserves to be published in Nature. Prior to publication the following issues should be addressed.
1) The authors do not present any examples demonstrating the homologation of bisbenzylalkyl amines. It is anticipated that substrates bearing two benzyl groups with different electronic properties could enable good chemoselectivity in the C–N bond activation step. The authors are encouraged to provide representative examples along these lines.
2) Unfortunately, longer-chain bromoalkyl boronates derivatives did not engage in the homologation sequence, as beta-hydride elimination turned out to be problematic. I am wondering whether the reagent BrCH(OMe)Bpin is accessible and would work. Please comment.
[redacted]

Referee #2

(Remarks to the Author)
In this manuscript, Gaunt and co-workers report on a deceptively simple, but highly effective method for the ‘programmable remodelling’ of tertiary benzylamines. Most of the reactions report are 1-carbon homologations. As the authors rightly note, the starting material scaffolds are highly prominent in biologically active molecules and are ubiquitous in the literature – driven in part by the ease of their synthesis. The products (β-aryl-ethylamines) are also very common motifs in bioactive but are less easily to synthesise, and hence I am confident the transformation will be a much value and interest. The scope and efficacy of the reaction has been very well demonstrated, including in several pharmaceuticals. The concept is then further extended in Figure 5 to enable much longer linkers to be inserted into the same class of starting material. This is my favourite part of the manuscript – this transformation looks to be very powerful and extends concept significantly. It is easy to imagine many applications of this method; for example [redacted]

[redacted] I did not read every page of the 351 page SI, but certainly it is extensive, and looks to have been properly and carefully written. In summary, this is a fascinating paper. It is underpinned by a very clever idea and has been very well executed. I support publication, subject to the following revision:

1) relating to β-aryl–ethylamine, the authors write that 'access to such architectures typically relies on synthesis from less readily available building blocks19-21, limiting the efficiency and modularity of analog diversification.' Can the authors be a more explicit here in how/why the building blocks are less readily available and how this limits their use? I'm assuming that the difference in reactivity/stability/availability of benzyl halides and aldehydes vs their homologues is main point here? If so, I fully agree, but I think this should be noted more directly (in addition to any other key points).

2) Very trivially, we have 2 x Figure 3's, so this will need fixing.

3) I am glad that the authors frame their new method in the context of the classical 1,2-Stevens rearrangement (and the Sommelet-Hauser rearrangement), citing references 31 and 32. However, while I concede that this point is subjective, and I do not insist on this change, I'd have liked to have this comparison made and discussed earlier in the manuscript. The advantages to the new method are clear. But to give my impression as a reader seeing this work for the first time, I was initially a bit underwhelmed reading the first few pages, and I was left thinking that surely the 1,2 rearrangement looked like it would be known. And of course it is. But the 1,2-Stevens rearrangements I am aware of almost always involved N-benzylation followed by migration of the same group, meaning the ubiquitous substrates in this manuscript could not be transformed, in addition to the limitations noted in the manuscript, and the strengths of the new catalytic approach. So, there is no problem – justification for the development of this new method is strong – but I think that discussion of the above sooner (e.g. during the Figure 1C discussion) would strengthen the manuscript.

4) In relation to the 1,2-Stevens rearrangement comparison, the following phrase is used: 'representing the first general, catalytic 1,2- Stevens-type rearrangement in non-activated benzylammonium substrates'. What feature of the benzylammonium substrates in this study mean that they can be described as 'non-activated', compared to substrates typically used in classical 1,2-Stevens rearrangements? Apologies if I am missing something, but to me they both similarly activated. Whatever the case, I recommend considering rewording around this section to make this point clearer.

5) As far as I can tell, in all examples reported the migrating group in benzylic (or a functionalised aryl equivalent). Where any non-benzylic, simple alkyl example tried? And if so, what was the outcome? I suspect this change would make the reaction much more difficult and/or shut it down. But nonetheless, I would like to see note of a few such examples included in the manuscript – even if the reactions are unsuccessful.

Version 1:

Reviewer comments:

Referee #1

(Remarks to the Author)
I reviewed the first version of the Gaunt-manuscript entitled "Programmable remodeling of carbon–nitrogen connectivity in amines". Authors did a very good job in revising their manuscript. My requests were mostly addressed and the paper further improved in quality. I am now very happy to strongly support this manuscript to be published in Nature as is.

Referee #2

(Remarks to the Author)
I reviewed the original submission favorably and recommended publication subject to revision. The authors have duly taken all of my suggestions, and those of another reviewer, fully on board and improved the manuscript significantly. I agree with the author's decision to add the new results of the non-benzylic alkyl ammonium substrates to the SI. [redacted]

In summary, I am very happy to recommend publication of this excellent work.

Open Access This Peer Review File is licensed under a Creative Commons Attribution 4.0 International License, which permits use, sharing, adaptation, distribution and reproduction in any medium or format, as long as you give appropriate credit to the original author(s) and the source, provide a link to the Creative Commons license, and indicate if changes were made.
In cases where reviewers are anonymous, credit should be given to 'Anonymous Referee' and the source.
The images or other third party material in this Peer Review File are included in the article's Creative Commons license, unless indicated otherwise in a credit line to the material. If material is not included in the article's Creative Commons license and your intended use is not permitted by statutory regulation or exceeds the permitted use, you will need to obtain permission directly from the copyright holder.
To view a copy of this license, visit https://creativecommons.org/licenses/by/4.0/

Response to the Reviewers

We sincerely thank the Editor and both Reviewers for their careful, thoughtful and constructive assessment of our manuscript. We were particularly encouraged by their positive evaluation of the work and their recognition of both the conceptual novelty and the broader synthetic significance of the work. Importantly, both reviewers recognised the central advance of establishing programmable C–N bond remodeling as a new strategy for molecular editing, with the comments focusing primarily on opportunities to further strengthen the scope, mechanistic understanding and presentation of the catalytic platform. We are grateful for the insightful suggestions, which we believe have substantially improved the manuscript.

In preparing the revised manuscript, we have carefully considered every comment and have undertaken significant additional experimental and mechanistic studies, alongside revisions to the text. Collectively, these additions further define the capabilities, design principles and operating boundaries of the C–N remodeling platform, while clarifying its conceptual relationship to existing chemistry.

The principal additions to the revised manuscript are summarised below:

1. **Electronically differentiated bisbenzylamine substrates.** We have expanded the scope of the methodology through a new study of electronically differentiated bisbenzylamine substrates, establishing highly chemoselective benzylic C–N remodeling and defining the electronic design principles governing selective activation.
2. **Mechanistic operating boundaries.** We have undertaken studies establishing the requirement for benzylic activation through evaluation of non-benzylic alkyl ammonium substrates, thereby defining an important operating boundary of the catalytic manifold. [redacted]
3. **Reagent-design strategy for programmable multi-carbon remodeling.** We have clarified the rationale underpinning the evolution of the multi-carbon remodeling platform, including additional discussion of the behaviour of longer-chain alkyl boronate reagents and the advantages of the latent alkenyl tosylate/in situ hydroboration strategy.
4. **Sequential programmable scaffold diversification.** We have added new sequential editing studies illustrating how selective benzylic C–N remodeling can be translated into practical scaffold diversification through successive transformations of bisbenzylamine substrates.

In addition to these new experimental studies, we have implemented several further revisions throughout the manuscript in response to the reviewers' suggestions. These include strengthening the discussion of feedstock accessibility and the synthetic motivation for remodeling, repositioning the comparison with the classical Stevens rearrangement earlier in the manuscript to improve the conceptual narrative, refining the associated mechanistic discussion and terminology, and addressing all remaining textual and editorial comments.

Our detailed responses to each reviewer comment are provided below.

Reviewer 1 – Comment

"The authors do not present any examples demonstrating the homologation of bisbenzylalkyl amines. It is anticipated that substrates bearing two benzyl groups with different electronic properties could enable good chemoselectivity in the C–N bond activation step. The authors are encouraged to provide representative examples along these lines."

Response

We thank the referee for this excellent suggestion. We agree that electronically differentiated bisbenzylamine substrates provide an important test of whether individual benzylic C–N bonds can be selectively addressed within a more complex amine framework. We have therefore undertaken this study and added a new series of examples to the revised manuscript (Figure X).

Our previous mechanistic studies, which are retained in the Supplementary Information (Figure S6b), showed that substrates bearing electronically similar benzyl substituents (for example, benzyl versus *p*-methoxybenzyl) exhibit little intrinsic selectivity and undergo significant crossover under standard reaction conditions. By contrast, introducing a clearer electronic bias through pairing an electron-rich benzyl group with an electron-deficient benzyl group enables highly chemoselective and synthetically efficient remodeling at the electron-rich benzylic position. This behaviour is observed across several electronically differentiated substrate pairs, including 2,4-dimethoxybenzyl/4-trifluoromethylbenzyl, 2,4-dimethoxybenzyl/3,5-difluorobenzyl, 2,4-dimethoxybenzyl/heteroaryl and 4-methoxy/3,5-bis(trifluoromethyl)benzyl systems, providing the desired products in generally high yields.

We believe these new experiments directly address the referee's suggestion and significantly strengthen the manuscript by defining the electronic design principles governing selective C–N remodeling within multiply benzylated amines. More broadly, they establish that selective benzylic C–N remodeling can be achieved through predictable electronic differentiation of closely related benzyl substituents, providing a practical foundation for programmable molecular editing within multiply benzylated amine scaffolds.

New substrates have been added as part of Figure 4c and the following text has been added to the manuscript.

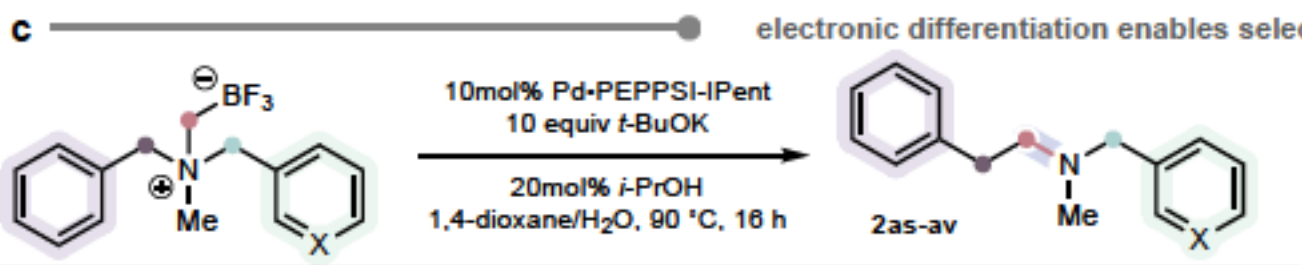

	electron rich arene	electron poor arene	yield, (assay yield)
2as	2,4-(OMe)$_2$	3-CF$_3$, X=CH	65% (66%)
2at	2,4-(OMe)$_2$	3,5-(F)$_2$, X=CH	70% (78%)
2au	2,4-(OMe)$_2$	4-(CF$_3$), X=N	72% (85%)
2av	4-OMe	3,5-(CF$_3$)$_2$, X=CH	83% (91%)

An important extension of this strategy is the ability to selectively address individual benzylic C–N bonds within multiply benzylated amines. Whereas electronically similar benzyl substituents exhibit little intrinsic differentiation (see Supplementary Information), introducing a pronounced electronic bias between an electron-rich and an electron-deficient benzylic substituent enables highly chemoselective remodeling at the electron-rich benzylic position across a range of substrate classes, providing desired products **2as-av** in generally high assay yields (Figure 4c). Rather than representing an isolated selectivity effect, these studies establish predictable electronic differentiation as a general design principle for programmable C–N remodeling, enabling individual benzylic C–N bonds to be selectively addressed within complex amine frameworks.

2. Defining the operating boundaries of the catalytic manifold

(a) Non-benzylic alkyl ammonium substrates

Reviewer 1 – Comment

"As far as I can tell, in all examples reported the migrating group is benzylic (or a functionalised aryl equivalent). Were any non-benzylic, simple alkyl examples investigated? If so, what was the outcome? I suspect this change would make the reaction much more difficult and/or shut it down. Nonetheless, I would like to see note of a few such examples included in the manuscript—even if the reactions are unsuccessful."

Response

We thank the referee for this insightful suggestion. We agree that evaluation of a simple non-benzylic alkyl ammonium substrate provides an important test of the operating boundaries of the catalytic manifold. We have therefore prepared the corresponding alkyl ammonium salt and evaluated it under the standard reaction conditions. In contrast to the benzylic ammonium substrates described throughout the manuscript, no productive C–N remodeling was observed (supplementary information, Figure S5b). We believe this result is mechanistically informative rather than simply negative, as it defines an important operating boundary of the transformation and demonstrates that productive C–N remodeling is intrinsically favored with benzylic ammonium systems. This experiment strengthens the mechanistic framework of the reaction by establishing that benzylic activation is an inherent design feature of the current remodeling strategy rather than a general feature of alkyl ammonium salts. While we have incorporated this experiment to the supplementary information, we have chosen not to comment further on it in the manuscript. We are, of course, happy to include a sentence in the text if the reviewer feels it is important to do so.

[redacted]

[redacted]

[redacted]

[redacted]

[redacted]

[redacted]

[redacted]

[redacted]

3. Clarification of the reagent-design strategy
(a) β-Hydride elimination and alkyl-Bpin reagents
Reviewer 1 – Comment
"Longer-chain alkylboron reagents are expected to suffer from β-hydride elimination. Have the authors considered alternative reagents, such as bromomethoxy-substituted boronates, to address this issue?"

Response
We thank the referee for this helpful suggestion. We agree that β-hydride elimination represents an important consideration in extending the remodeling strategy beyond one-carbon insertion and have revised the manuscript to better clarify the rationale underpinning the development of the multi-carbon remodeling platform.

While longer-chain bromoalkyl boronate reagents operate with reduced efficiency, they are not incompatible with the catalytic manifold. Indeed, the corresponding alkyl-Bpin reagent undergoes productive C–N remodeling, albeit in lower yield, indicating that β-hydride elimination is a competing rather than prohibitive pathway. This experiment was present in the original supplementary information (retained on S17-18) but we have revised the manuscript to make this point explicit.

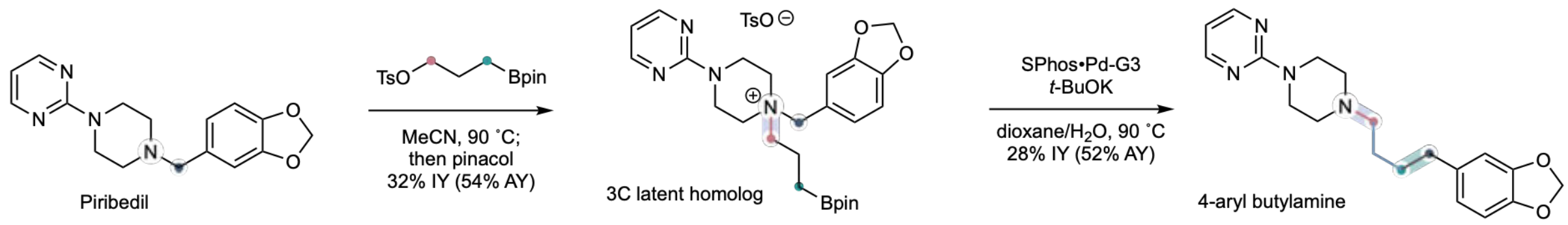

The following text has been added:

Initial attempts to extend the transformation using longer-chain bromoalkyl boronate derivatives—effectively demanding $C(sp^3)–C(sp^3)$ bond formation under conditions susceptible to β-hydride elimination—demonstrated that although the corresponding three-carbon homolog could be obtained in modest yield, inefficient transmetalation and competing β-hydride elimination limited the efficiency of this approach (see Supplementary Information), necessitating a more sophisticated reactivity-matched design.

However, rather than simply overcoming β-hydride elimination, the latent alkenyl tosylate strategy was conceived as a more general reagent platform for programmable multi-carbon remodeling. This approach employs stable alkylation reagents that are readily prepared from abundant alkenol feedstocks, while *in situ* hydroboration generates a highly reactive organoborane immediately prior to transmetalation. Together, these features accelerate transmetalation, suppress competing decomposition pathways, and substantially broaden both the efficiency and synthetic versatility of programmable multi-carbon remodeling. We have revised the manuscript to make this design rationale more explicit.

With respect to the suggested bromomethoxy-substituted boronate reagent, we have not investigated this substrate. Its preparation would require a multistep synthesis f and would introduce a hemiaminal- into the product, representing a transformation of benzyl amine to protected ketone. While an interesting transformation, this differs substantially from that of the systems investigated here in both concept and outcome. Given that the latent alkenyl tosylate strategy already provides a general and experimentally validated solution to programmable multi-carbon remodeling, we therefore chose to focus our efforts on developing and defining this more broadly applicable reagent platform rather than pursuing an alternative substrate class with distinct mechanistic characteristics.

4. Sequential programmable scaffold diversification

Reviewer 1 – Comment

The referee suggested that selective remodeling of multiply benzylated amines would significantly enhance the synthetic utility of the methodology.

Response

We thank the referee for highlighting the broader synthetic opportunities that arise from selective benzylic C–N remodeling. While establishing chemoselective activation was the principal objective of the new bisbenzylamine studies, we recognised that this selectivity also creates opportunities for the sequential differentiation of initially equivalent benzyl substituents. We have therefore expanded the manuscript to include two additional studies that illustrate the synthetic potential of this concept.

Following chemoselective one-carbon homologation of one benzylic C–N bond, the remaining benzyl substituent can be manipulated independently in two distinct ways. In one example, selective hydrogenolytic debenzylation furnishes a structurally differentiated secondary amine, demonstrating that selective C–N remodeling can be integrated with established synthetic transformations to rapidly access new amine architectures. Perhaps most importantly, the remaining benzyl substituent can

undergo a second, orthogonal C–N remodeling event further using programmable linker insertion manifold, providing a substantially more elaborated aryl–alkylamine product from the same precursor. We believe these studies move beyond simply demonstrating selective bond activation. Instead, they show how selective benzylic C–N remodeling can be translated into sequential programmable scaffold diversification, enabling differentiated molecular editing from common bisbenzylamine precursors.

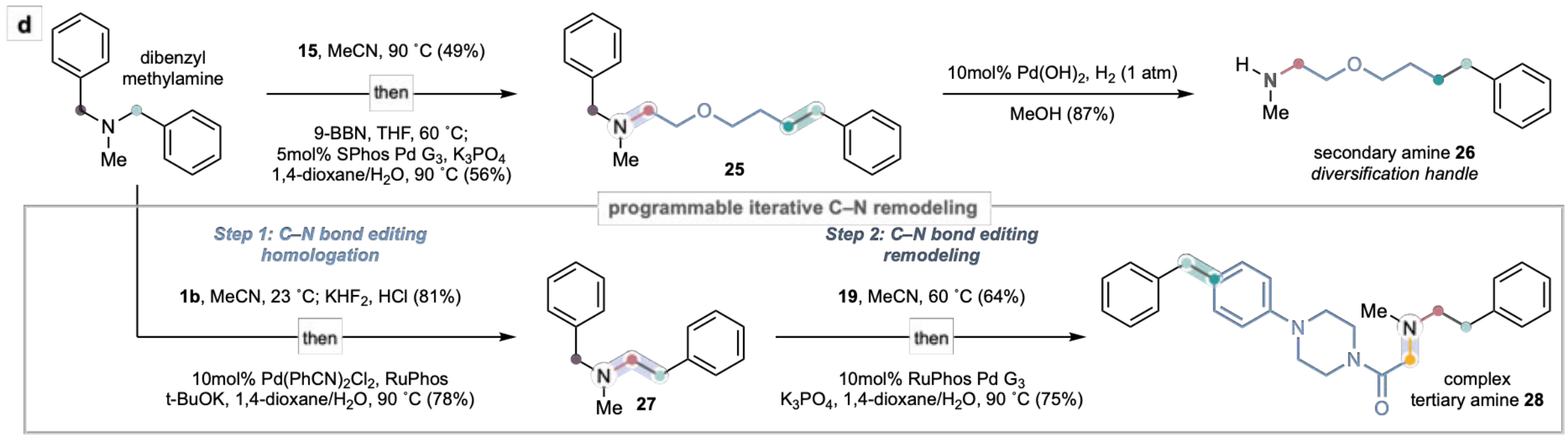

This scheme forms part of Figure 5. The following text has been added to support these observations Beyond expanding the diversity accessible through individual remodeling reactions, the C–N activation strategy enables sequential editing of multiply benzylated amine frameworks (Figure 5d). Following remodeling of one benzylic C–N bond in dibenzyl methylamine with bifunctional linker **15** (to **25**), the remaining benzylic C–N bond can be exploited independently. Thus, selective debenzylation after linker insertion provides direct access to a structurally differentiated secondary amine **26**, illustrating how one benzylic substituent can serve as a programmable handle while the second remains available for subsequent diversification. More significantly, the residual benzylic C–N bond can itself undergo a subsequent remodeling event. For example, sequential one-carbon homologation of dibenzyl methylamine with **1b** (to **27**) followed by complex linker insertion (with **19**) and remodeling furnishes a highly elaborated tertiary amine **28** wherein two distinct C–N remodeling operations are leveraged from a readily accessible dibenzylamine precursor. Rather than representing isolated synthetic transformations, these studies illustrate programmable iterative C–N remodeling as a practical strategy for the stepwise evolution of tertiary benzylamines into architecturally and functionally distinct amine scaffolds.

Collectively, these new studies directly address the referee's suggestion and further strengthen the central concept of the manuscript by demonstrating that programmable C–N remodeling is not only selective, but can also be exploited to access structurally distinct molecular architectures through sequential, programmable editing of common amine scaffolds.

5. Additional conceptual refinements and editorial revisions

In addition to the new experimental studies described above, we have implemented several further revisions throughout the manuscript in response to the reviewers' suggestions. These revisions strengthen the conceptual framing of the work, clarify aspects of the mechanistic discussion, and improve the overall presentation of the manuscript.

(a) Feedstock accessibility and motivation for C–N remodeling
Reviewer comment
"Can the authors be more explicit in explaining why β-aryl–alkylamine architectures generally require less readily available building blocks and how this limits their use?"

Response

We thank the referee for this helpful suggestion and agree that this point was insufficiently developed in the original manuscript. We have revised the text to explain more explicitly why higher-order aryl–alkylamine architectures are generally less accessible than tertiary benzylamines. In particular, we now note that benzyl-derived feedstocks (including benzyl halides, aldehydes, alcohols and related derivatives) are widely available and readily prepared directly from simple aromatic starting materials. By contrast, homologated building blocks are substantially less accessible. Arylethyl halides are more prone to elimination, phenethyl aldehydes are susceptible to enolisation and oxidative degradation, and higher homologues generally require multistep synthesis from the corresponding aromatic feedstocks. Together, these factors reduce the efficiency and modularity of analogue synthesis and provide a key motivation for developing a direct remodeling strategy from readily available tertiary benzylamines.

The following text has been added:
However, access to such architectures typically relies on homologated aryl–alkyl building blocks, whose availability declines sharply beyond the benzylic position. This disparity reflects a fundamental bias in synthetic methodology: while direct one-carbon functionalisation of arenes provides ready access to a diverse array of benzyl-derived feedstocks, comparatively few general strategies enable analogous access to higher homologues[19-21]. Consequently, homologated ω-aryl–alkyl building blocks typically require multistep synthesis and, in the case of phenethyl derivatives, often exhibit less favourable synthetic properties, limiting the efficiency and modularity of analogue diversification.

(b) Positioning of the Stevens rearrangement discussion

Reviewer comment

"I would have liked the comparison with the classical 1,2-Stevens rearrangement to be introduced earlier in the manuscript."

Response

We thank the referee for this thoughtful suggestion and agree that the conceptual relationship to the classical Stevens rearrangement is better introduced at an earlier stage of the manuscript. In the revised version, we have therefore added discussion to the reaction design section, where it now provides context and conceptual motivation for the catalytic remodeling strategy. This revised positioning allows the reader to appreciate the inspiration for the present work at the outset while immediately highlighting the fundamental distinctions between the present catalytic approach and classical substrate-controlled Stevens rearrangements. We believe this results in a clearer conceptual narrative and thank the referee for the suggestion.

The following text has been added:
Conceptually, this design draws inspiration from the classical Stevens rearrangement, a transformation that formally effects carbon migration across a quaternary ammonium centre[22]. Although this reaction demonstrates that carbon–nitrogen connectivity can, in principle, be reorganised through a quaternary ammonium intermediate, its reliance on substrate-controlled reactivity has confined its utility to a narrow and often unpredictable reaction space. Notably, the simple benzylammonium salts most directly relevant to the present study do not undergo productive 1,2-Stevens rearrangement, but instead preferentially react through the competing 2,3-Sommelet–Hauser pathway[23]. Rather than attempting to overcome these intrinsic limitations, we instead abstract its underlying conceptual logic—the reorganisation of C–N connectivity—and reimplement it within a catalytic framework based on programmable bond activation and fragment recombination. Accordingly, we implemented this strategy through a Pd-catalyzed process for the controlled

reconfiguration of tertiary benzylamines. This catalytic manifold is first realised through one-carbon homologation[24–29], providing the simplest demonstration of the underlying design principle (Figure 1c). Building on this foundation, extension of the activation mode enables progressively more complex molecular units to be encoded within the bifunctional reagent and inserted across the C–N bond, establishing a programmable platform for the direct diversification of tertiary benzylamines into structurally distinct aryl–alkylamine architectures.

(c) Clarification of the Stevens terminology

Reviewer comment

"Please clarify the description of 'non-activated benzylammonium substrates'."

Response

We thank the referee for highlighting this point. We agree that the term *"non-activated"* could be interpreted ambiguously in this context. We have therefore revised the wording where appropriate to more clearly distinguish the present catalytic remodeling strategy from classical Stevens rearrangements without relying on this terminology.

(d) Minor editorial corrections

Reviewer comment

"Figure 3 appears twice."

Response

We thank the referee for spotting this oversight. The duplicated figure numbering has been corrected in the revised manuscript.

We again sincerely thank both reviewers for their thoughtful, constructive and insightful assessment of our work. We believe their suggestions have substantially strengthened both the manuscript and the scientific presentation, and we are grateful for the opportunity to further develop the work through this revision.

We respectfully submit the revised manuscript for your consideration.
